# The Novel Gallium Aminobisphenolate Initiator of the Ring-Opening Copolymerization of L-Lactide and ε-Caprolactone: A Computational Study

**DOI:** 10.3390/ijms232415523

**Published:** 2022-12-08

**Authors:** Maxim V. Zabalov, Badma N. Mankaev, Mikhail P. Egorov, Sergey S. Karlov

**Affiliations:** 1 N.D. Zelinsky Institute of Organic Chemistry, Russian Academy of Science, 119991 Moscow, Russia; 2 N.N. Semenov Federal Research Center for Chemical Physics, Russian Academy of Sciences, 119991 Moscow, Russia; 3 Chemistry Department, Moscow State University, 119991 Moscow, Russia

**Keywords:** ring-opening polymerization, copolymerization, gallium complexes, L-lactide, biodegradable polymers, ε-caprolactone, DFT calculations, initiators

## Abstract

Density functional theory (DFT) simulations of ring-opening copolymerization of ε-caprolactone (CL) and L-lactide (LA) in presence of novel gallium complex on aminobis (phenolate) ligand are conducted. The initial steps of polymerization of CL and LA as well as the first steps of propagation which led to LGa-LA-LA-OMe, LGa-LA-CL-OMe, LGa-CL-LA-OMe, or LGa-CL-CL-OMe derivatives have been analyzed in detail. According to these data, the studied catalyst is a rare example of a catalyst in which, during copolymerization, the polymerization of CL should proceed faster than LA. Thus, we predict the formation of a mainly block copolymer poly(CL-block-LA) using this catalyst.

## 1. Introduction

To date, biodegradable polymers have become one of the most rapidly developing classes of technologically important compounds due to the environmental concerns associated with «classical» plastics pollution and waste. Biodegradable polymers are characterized by an acceptable decomposition time in the natural environment (usually several years) as well as non-toxic decomposition products. The comparative rapid decomposition in various natural environments allows the use of such polymers for several applications as packaging, as well as for various biomedical applications (suture material, tissue engineering) [1]. Among natural and synthetic biodegradable polymers, one of the most used at the moment is polylactide (PLA), which is a polymer of the cyclic dimer of lactic acid, lactide (LA). The advantages of PLA are the production from renewable resources and reduction in energy costs and greenhouse gas emission, both better than that of oil-based plastics preparation [2,3]. In addition to PLA, polycaprolactone (PCL) is another of the most important biodegradable polyesters due to its biocompatibility, nontoxicity, and relatively cost efficiency [4]. In general, there are two approaches for the preparation of synthetic polyesters: polycondensation of corresponding hydroxy acids and catalytic ring-opening polymerization of cyclic lactones. Although the first approach also allows the production of appropriate polymers, such as PLA, with high molecular weight on an industrial scale, the second is used much more often, because the required synthesis conditions in the condensation approach were harsh, which resulted in many byproducts. The ring-opening polymerization (ROP) of lactones requires an appropriate catalyst to proceed in reasonable conditions and to afford polymers with controlled properties. Therefore, metal-based catalytic systems remain and are likely to remain for a long time to come as the most suitable ROP initiators, although alternative strategies based on nucleophilic (organocatalysts), cationic (strongly organic acids) promoters or enzymes have also been extensively studied [5,6,7].

**Scheme 1:**
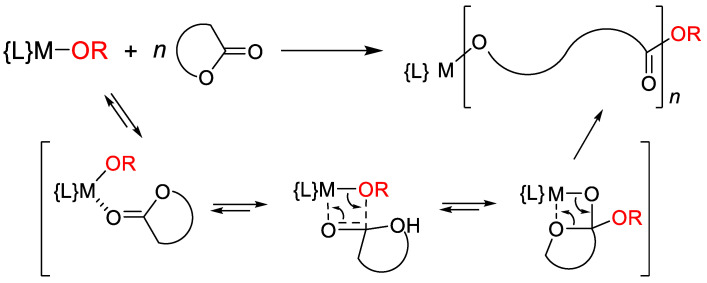
Coordination insertion mechanism of ROP [8].

Two different ROP mechanisms are possible in catalysis by metal compounds: the ‘‘coordination insertion mechanism’’, where the catalyst is complex M–Nu, in which Nu is a nucleophilic group *capable of ring-opening the heterocyclic monomer* (such a mechanism is depicted in Scheme 1), or the ‘‘activated monomer mechanism’’, where the catalytic system is Lewis acidic metal salts/ROH [8].

It should be also noted that PLA displays physical and mechanical properties that make it comparable to traditional petroleum-based polymers like polystyrene and poly(ethylene terephthalate) [9]. However, a number of drawbacks of PLA are also well-known, including brittleness, poor elasticity, low thermal stability, poor gas/water permeability, etc. [10]. There are two different approaches for improving some of these properties: (i) modification of PLA by plasticization or blending and (ii) ring-opening copolymerization (ROCOP) of LA with another comonomer [10,11].

One of the most important copolymers of LA is poly(lactide-co-caprolactone), poly(LA-CL). These two homopolymers have contrasting physical and thermal properties: PCL exhibits good elasticity and permeability but poor mechanical characteristics (toughness), which is the opposite to PLA [12]. The preparation of a statistical copolymer poly(LA-stat-CL) may lead to biodegradable materials with improved properties. At the same time, the copolymerization of LA and ε-CL, in most cases, results in the formation of block poly(LA-block-CL) or gradient poly(LA-grad-CL), copolymers due to the different rate of propagation of these monomers on most of the studied initiators. Of interest, ε-CL is typically faster than LA in their respective homopolymerizations, while copolymerization of both monomers often leads to the preferential consumption of LA over ε-CL [11]. The literature describes a number of aluminum [13,14,15,16], zinc [17], molybdenum [18], barium [19], and lanthanum [20] complexes that catalyze polymerization successfully enough, leading to the production of a statistical copolymer. Most aluminum complexes are derivatives of salen-type ligands or iminophenols [13,14,15]. At the same time the activity of these catalysts is poor. Thus, the preparation of novel, more effective catalysts of statistical copolymerization based on non-toxic metals is one of the remaining challenges in this extensively researched area of ROP.

Very recently, Chandanabodhi and Nanok have studied the mechanisms of the copolymerization of LA and ε-CL initiated by aluminum alkoxide complexes supported by salen-type ligands [21]. Despite the fact that ROP has been studied by quantum chemistry methods rather intensively [22], to our knowledge only one paper published to date deals with DFT calculations of copolymeryzation of LA and CL with metal-based catalysts. The authors showed a noticeable effect of the structure of the initiator on the rate of both the initiation stage and the first propagation stage. The computational results are correlated with experimental data. Another recently published computational paper is devoted to the study of benzoic acid as a catalyst for copolymerization [23].

It can be assumed that for a detailed study of the copolymerization mechanism, it is necessary to carry out quantum chemical calculations of this reaction, which will allow someone to establish the regularities of the “initiator structure-copolymer composition” and to offer the structure of initiators which are more active in copolymerization.

In this work, the mechanisms of the initiation and first propagation steps of the ring-opening polymerization of ε-caprolactone and L-lactide are studied in the case of catalysis by a methoxygallium complex based on an amino(bis)phenolate ligand. A different alkoxy-substituent on the metal atom could be used for ROP, and the choice of this group does not affect the reaction mechanism and rate. Closely related titanium and aluminum complexes have been prepared and studied in ROP [24,25,26,27,28,29]. We used the methoxy-gallium complex as a simple model of the initiation of polymerization. For the calculation, the general Scheme 1, as appropriate for both the initiation step and first propagation steps with both monomers, was used.

## 2. Results and Discussion

**Scheme 2:**
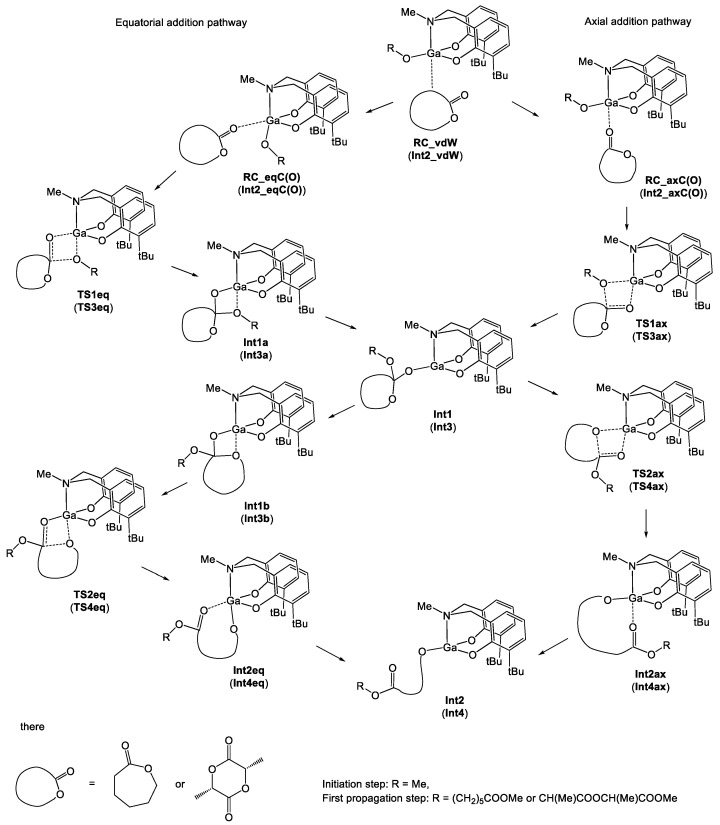
General scheme of initiation and first propagation steps. Labels for transition states and intermediates of first propagation step are in parentheses.

According to the coordination insertion mechanism, the initiator forms the Van der Waals complex, first with the monomer and then the coordination complex, with formation of a bond between the monomer carbonyl O atom and Ga atom (Scheme 2). The monomer can approach the metal center through either an equatorial or axial position. Both variants are considered by us. We investigate the potential energy surface (PES) for finding any transition states of monomer addition. In most cases, this region of PES looks like a curve inflection without the formation of a maximum or with the formation of a weakly pronounced maximum with energy of less than 1 kcal mol^−1^ higher than complex **RC_C(O)**.

The first transition states for both paths are four-member cyclic structures which correspond to the addition of an alkoxy group to the carbonyl C atom, promoted by the gallium complex. In the case of the equatorial monomer addition, the bonding of the Ga atom with the axial alkoxy O atom is strong enough to form the four-member cyclic intermediate **Int1a**. In the case of the axial monomer addition, the **Int1** with penta-coordinated Ga immediately forms. We also investigated this region of the PES and found no transition states of the transition between **Int1a**, **Int1,** and **Int1b**; their transformations into each other occur without barriers.

The second transformation involves rotation around the Ga-O single bond in **Int1** to coordinate the cyclic alkoxy O atom to the Ga atom. In the case of equatorial addition, this rotation also gives an additional stable four-member cyclic **Int1b**. In the literature, two intermediates of type **Int1** are often distinguished. They are different in the orientation of the O atoms from O-R or from the cycle in terms of which of them is closer to the metal atom [21,22,30,31]. These intermediates are the result of IRC calculations from **TS1** and **TS2**. However, rotation around the single bond in **Int1** (and **Int3**) occurs without a barrier, and there is no reason to split **Int1** into two structures on PES, because both structures are conformers of one compound. Thus, the global minimum of **Int1** (**Int3**) is the proper energy level for the calculation of barriers of reaction proceeding via **TS2** (**TS4**).

At the second stage, the monomer cycle is opening through a four-member transition state to form a product complex with coordination of the carbonyl O atom to the metal atom. The latter, without a barrier, goes to **Int2** (**Int4**), which becomes the new initiator for the next propagation step. The Ga-O bond, in complexes with coordination of the carbonyl oxygen atom, is generally weak. In some cases, such complexes do not form a minimum on PES due to steric hindrances or insufficient electrophilicity of the metal center.

**Figure 1:**
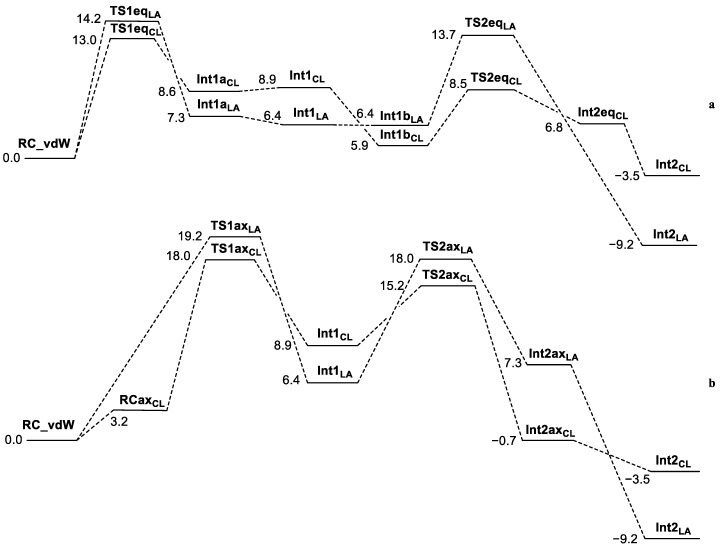
The Gibbs free energy profiles of initiation steps of CL and LA polymerization proceeding via paths with equatorial (**a**) and axial (**b**) addition of monomer.

**Figure 2:**
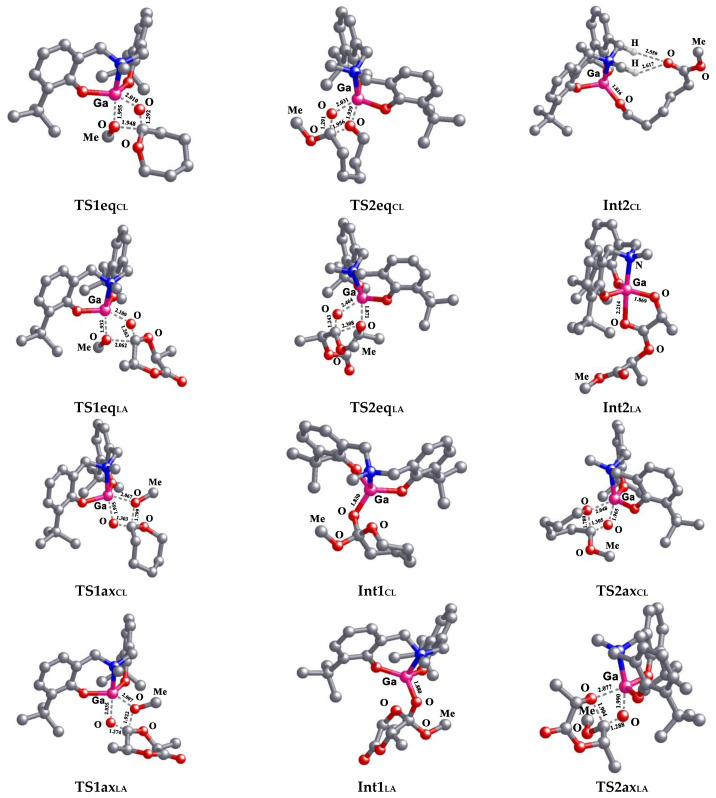
The representative structures of transition states and intermediates of initiation step. Hydrogen atoms are omitted for clarity where they do not participate in the formation of hydrogen bonds.

**Initiation step of polymerization of CL and LA.** We have considered both equatorial and axial pathways of monomer addition (Figure 1). The key structures are shown in Figure 2. In all cases considered, the limiting stage is the first stage. For the equatorial path of the reaction, the barriers of the limiting step are very close and are 13.0 and 14.2 kcal mol^−1^ for CL and LA, respectively. Furthermore, the series of intermediates **Int1a**, **Int1**, and **Int1b** is somewhat more stable for LA, but the difference is not essential. The biggest difference in energies is observed in **TS2eq**, which is associated with the need to open a stronger six-member cycle in LA compared to the seven-member one in CL. This difference has a small effect on the decrease in the rate of LA polymerization, but not fundamentally, since the stage is not limiting.

The barriers of the reaction path with the axial addition of monomers are significantly higher than for the path with equatorial addition. This is not due to the presence of steric hindrance created by the ligand but is due to the formation of stronger bonds in **TS1eq** (Figure 2). The rate-limiting stage for the axial pathway is also the first stage, and the reaction for CL proceeds with a slightly lower barrier. The probability of the reaction proceeding through axial paths with such a high barrier is low, and the initiation step takes place almost entirely along the equatorial path.

An important difference was found between the reactions of the two monomers, which is that the formation of the product is much more favorable for LA than for CL. This is due to the formation of an additional coordination bond of the Ga atom with the nearest carbonyl O atom of the LA residue. As a result, the product of LA addition is a stable axial chelate complex with a five-member ring and a stabilization energy of 8.8 kcal mol^−1^ relative to the **Int2_LA_** conformer without the cycle or other specific interactions. Other, but weaker, specific interactions are also possible in **Int2_LA_**, such as the equatorial interaction of the nearest carbonyl O atom of the LA tail with the Ga atom to form a weaker chelate (a gain in energy of 4.3 kcal mol^−1^), the interaction of the nearest carbonyl O atom of the LA tail with two α-protons at the quaternary N atom (an energy gain of 2.4 kcal mol^−1^), and the interaction of the remote from the metal carbonyl O atom of the LA residue with two α-protons at the quaternary N atom (an energy gain of 2.0 kcal mol^−1^).

**Int2_CL_** has only one stabilization factor, the interaction of a carbonyl O atom of the CL tail with two α-protons at the quaternary N atom, which gives an energy gain of 3.3 kcal mol^−1^. Thus, the energy barriers are somewhat larger for the initiation step of LA polymerization, and stabilization factors of the product will play an important role for the propagation step (see below).

In our study, we obtained the most complete picture of the mechanism of polymerization of cyclic ethers by main group metal complexes due to the finding of both reaction pathways with the axial and equatorial addition of monomers and due to the stability of many intermediates. In the literature, most often only one reaction path is found with the formation of four-member cyclic intermediates (analogous to the equatorial path) [32,33,34,35,36,37] or without their formation (analogous to the axial path) [21,30,31,38,39,40,41]. The stability of four-member cyclic intermediates is directly related to the structures of the initiators, namely, the choice of the central metal atom, the saturation of its coordination sphere, the structure of the ligand, its tension, and the presence of electron donor or acceptor substituents. The rate-limiting stage in most cases is also the first [22]. The activation barriers of the equatorial reaction path calculated in this article lie in the middle range of values for different initiators studied earlier in the literature. In general, for different initiators, the activation barriers of the limiting stage fall within the range of 2.1–36.4 kcal/mol [22].

**First propagation step for CL and LA homo- and copolymerization.** The mechanism of addition of any second monomer to the product of the initiation step has no fundamental differences with the initiation step. The structures of transition states and intermediates are very similar. The differences arise from stabilization effects in structures due to the presence of the monomer tail from the initiation step.

**Figure 3:**
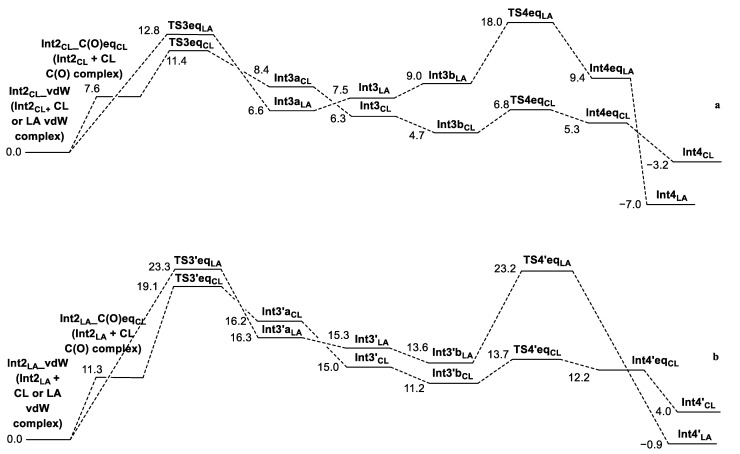
The Gibbs free energy profiles of propagation steps with equatorial CL or LA addition to **Int2_CL_** (**a**) and **Int2_LA_** (**b**).

**Figure 4:**
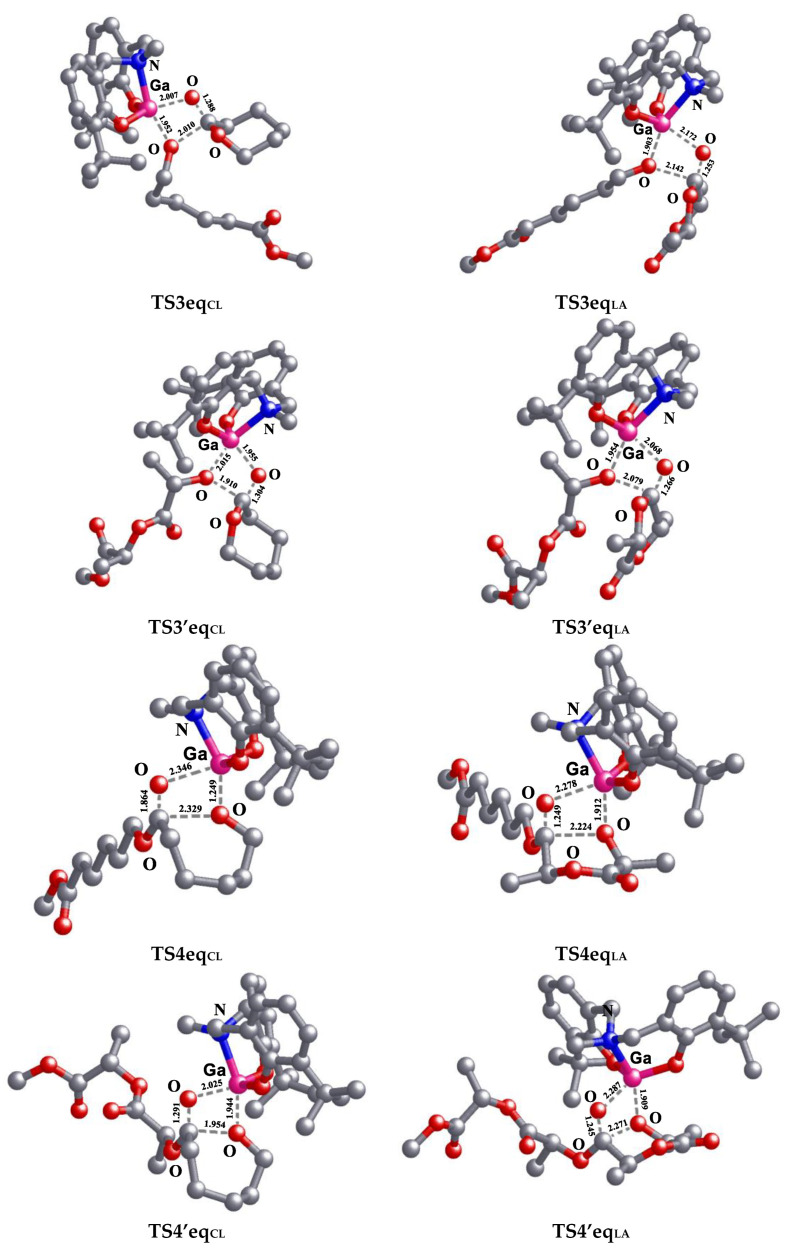
Structures of transition states of first propagation step proceeding via equatorial monomer addition path. Hydrogen atoms are omitted for clarity.

Thus, the second step of CL homo-polymerization differs from the initiation step only by a slightly decreased barrier (by 1.6 kcal/mol) of the rate-limiting stage and by small differences in the energies of other structures (Figure 3a). The addition of LA to Int2_CL_ occurs with a slightly higher barrier (by 1.4 kcal/mol) due to a more flat LA structure and therefore a greater steric repulsion between LA and the CL residue in the transition state (Figure 4). For the paths of the axial addition of monomers, even higher barriers were found, and they are 17.7 and 18.5 kcal/mol for the addition of CL and LA, respectively (see Figure S1a in Supplementary Materials). Along the path with the axial attachment of the monomer, the reaction cannot proceed.

A fundamental difference in polymerization reactions is observed at the stage of propagation after the addition of LA at the previous stage (Figure 3b). As already noted, the formation of a stable chelate is possible for **Int2_LA_** with the coordination of the nearest carbonyl O atom of the LA residue along the chain to the metal atom to form a five-member cycle. The formation of such a complex leads to the need to overcome the higher barrier on the first stage of the propagation step both in the case of the reaction with CL (19.1 kcal mol^−1^) and especially with LA (23.3 kcal mol^−1^). Similarly, for the axial addition of monomers, the barriers are very high and rich, 23.9 and 30.5 kcal mol^−1^ for the reaction with CL and LA, respectively (see Figure S1b in Supplementary Materials). For the same reasons, the formation of the product **Int4′_CL_** is thermodynamically unfavorable, since it has no factors of additional stabilization of the structure. However, such factors exist in structure **Int4′_LA_**, and the process of its formation is thermodynamically favorable. Thus, the propagation step after the addition of LA in the previous step appears to be very slow with both monomers.

The formation of chelate intermediates was noted earlier by many researchers, but their key role in the kinetics of lactide polymerization processes compared to CL polymerization was not emphasized [21,40,41,42]. Of particular note are the quantum chemical studies of Chandanabodhi and Tanok of the ROP mechanism of LA and CL using aluminum-bridged salen-type initiators [21]. Stabilization of the intermediate formed after the initiation step with the participation of LA due to the formation of a chelate complex led to an increase in the activation barrier of the propagation step, but not for all initiators. The introduction of electronegative substituents into the phenyl rings of the ligand led to a decrease in the barrier of this stage. Thus, the effect of the formation of an intramolecular complex plays a key role for such reactions and should be taken into account when choosing catalysts for the random copolymerization of LA and CL.

## 3. Materials and Methods

The quantum chemical calculations were carried out within the framework of the DFT method using the non-empirical generalized gradient approximation and the PBE functional [43,44] in the TZ2P basis set using the PRIRODA program [45,46] with the following parameters: numerical integration accuracy = 1 × 10^−8^; acceptable gradient value at which optimization can be completed = 1 × 10^−5^; accuracy of solving the self-consistent field equations = 1 × 10^−6^. Geometry optimization was performed for all stable compounds, and a saddle point search was performed for transition states. The characters of the stationary points found (minima or saddle point on the PES) were determined by calculating the eigenvalues of the matrix of the second energy derivatives with respect to the nuclear coordinates. Correspondence between a particular TS and a transformation under study was verified by calculating the intrinsic reaction coordinate (IRC). All the reported Gibbs free energy values were the sum of the electronic energy, the thermal corrections obtained by the frequency calculations (298K), and the dispersion corrections. Grimme’s PBE-D3 dispersion corrections were calculated with BJ damping [47,48]. Relative energies were calculated in kcal mol^−1^. Solvent effects were not taken into account, since polymerization reactions are usually carried out in bulk.

When calculating relatively big molecules, the differences in the energies of conformers can exceed the activation energies of individual stages, and it is necessary to take into account as many variations as possible for each structure in order to separate the conformational energies from the energies of chemical transformations. Only then can the calculated activation barriers of reactions be considered reliable [49,50]. Thus, for all stable compounds, the conformational analysis was performed with the aim to find the global minimum structure or close to it. A search for all possible conformers and isomers was also carried out for TS. Because the energy difference between the two LA conformers is less than 1.7 kcal mol^−1^ [51], both conformations were used to construct the initial structures. For CL, only the most favorable chair conformation was used [52].

## 4. Conclusions

For the polymerization reaction of CL and LA catalyzed by a gallium complex, two pathways are possible with the axial and equatorial addition of monomers. The equatorial path is beneficial at all stages. The rate-limiting stages of the initiation step of CL and LA polymerization along the equatorial paths have close energy barriers of 13.0 and 14.2 kcal mol^−1^, respectively. The first propagation stage after addition of CL on the previous step differs little from the initiation step, proceeding via slightly lower energy barriers of 11.4 and 12.8 kcal mol^−1^, respectively. The main difference was found at the propagation step after the addition of LA on the previous step. Due to the high degree of stabilization of the intermediate, the barrier of the rate-limiting stage is higher (19.1 and 23.3 kcal mol^−1^ for CL and LA addition, respectively), and this slows down the further reaction with both monomers. The stability of the intermediate (**Int2_LA_**) plays a key role in the kinetics of the homo- and copolymerization of LA. Thus, the initialization steps are similar for the two monomers. The propagation step after the addition of CL is most advantageous for the addition of a second CL. The addition of LA is slower due to the somewhat larger barrier of the limiting stage. The propagation step after LA addition is extremely slow for the addition of both monomers, due to very high reaction barriers. We predict the formation of a mainly block copolymer using this catalyst. Moreover, at first CL will mainly be spent, then LA will start to polymerize. The investigated catalyst is a rare example of a catalyst in which, during copolymerization, the reaction of CL should proceed faster than LA. However, modification of the initiator is required in order to equalize the rates of reactions with the two monomers, which can be done, for example, by introducing electronegative substituents into the ligand.

## Supplementary Materials

The following supporting information can be downloaded at: https://www.mdpi.com/article/10.3390/ijms232415523/s1.
