# Supplementary material for: The Novel Gallium Aminobisphenolate Initiator of the Ring-Opening Copolymerization of L-Lactide and ε-Caprolactone: A Computational Study
Source: Int J Mol Sci. 2022 Dec 8;23(24):15523. doi: 10.3390/ijms232415523 (PMC9779187; doi:10.3390/ijms232415523)
Supplement: Supplementary file 1 [file ijms-23-15523-s001.zip › ijms-2034927-supplementary.pdf]

Supporting information for

*Article*

# The novel gallium aminobisphenolate initiator of the ring-opening copolymerization of L-lactide and ε-caprolactone: a computational study

**Maxim V. Zabalov, Badma N. Mankaev, Mikhail P. Egorov, and Sergey S. Karlov**

# Content

Fig S1. The Gibbs free energy profiles of propagation step with axial CL or LA addition to **Int2$_{CL}$** (a) and **Int2$_{LA}$** (b)........ S3
Calculated Cartesian coordinates and energies of transition states and minima ........ S4
Initiation step of CL addition........ S4
Initiation step with axial CL addition........ S33
Initiation step with equatorial CL addition........ S101
Initiation step of LA addition ........ S196
Initiation step with axial LA addition........ S211
Initiation step with equatorial LA addition ........ S281
Propagation step of CL addition to the product of CL addition on initiation step ........ S439
Propagation step of axial CL addition to the product of CL addition on initiation step ........ S465
Propagation step of equatorial CL addition to the product of CL addition on initiation step .. S543
Propagation step of LA addition to the product of CL addition on initiation step........ S690
Propagation step of axial LA addition to the product of CL addition on initiation step ........ S726
Propagation step of equatorial LA addition to the product of CL addition on initiation step .. S765
Propagation step of CL addition to the product of LA addition on initiation step........ S832
Propagation step of axial CL addition to the product of LA addition on initiation step ........ S845
Propagation step of equatorial CL addition to the product of LA addition on initiation step .. S888
Propagation step of LA addition to the product of LA addition on initiation step........ S981
Propagation step of axial LA addition to the product of LA addition on initiation step........ S1010
Propagation step of equatorial LA addition to the product of LA addition on initiation step S1051

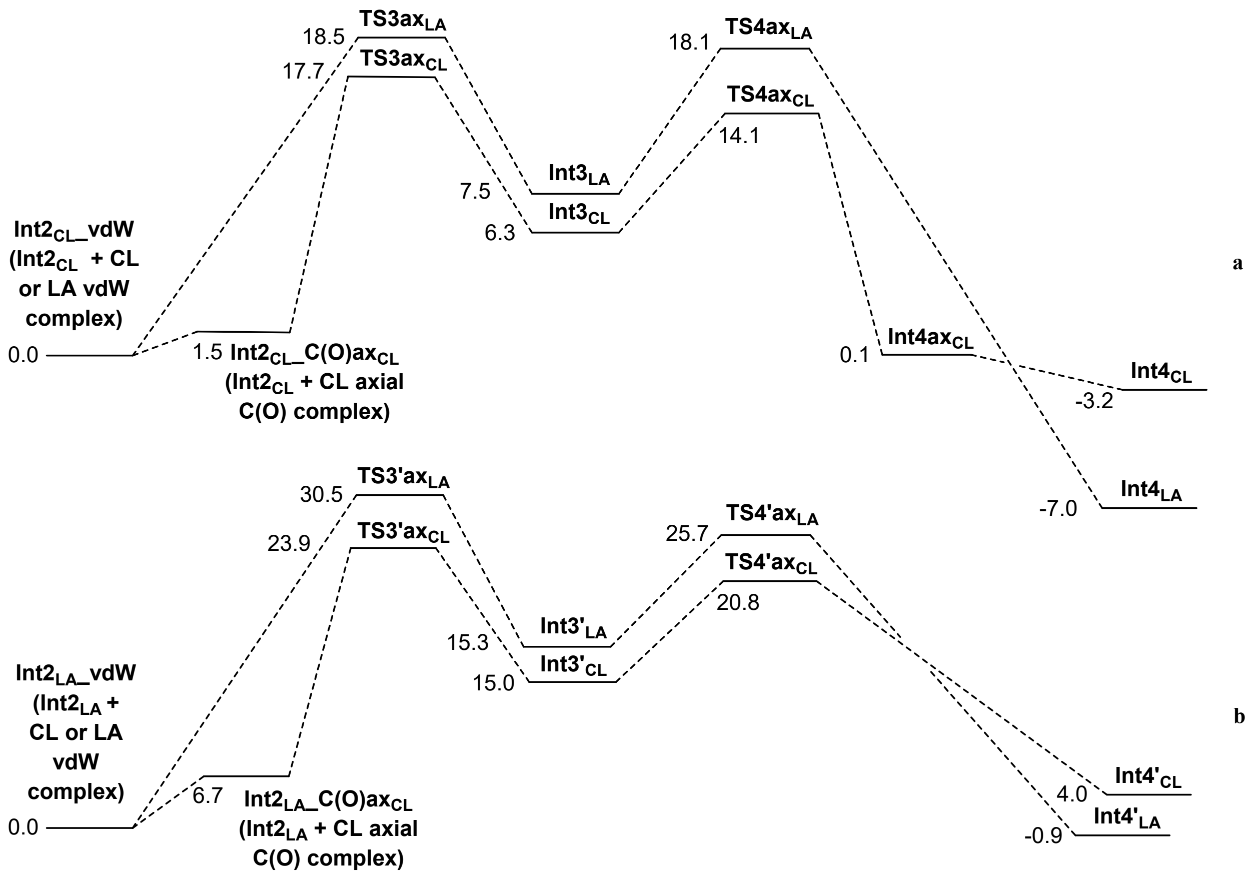


Figure S1. The Gibbs free energy profiles of propagation step with axial CL or LA addition to **Int2**$_{CL}$ (a) and **Int2**$_{LA}$ (b).

## Calculated Cartesian coordinates and energies of transition states and minima

Isomers/conformers are sorted in order of increasing Gibbs free energy.

## Initiation step of CL addition

**RC vdW CL isomer/conformer 1**

| Atomic N. | X | Y | Z |
|---|---|---|---|
| 7 | 0.81288246 | 1.45372885 | 1.40677276 |
| 8 | -0.47802149 | 0.33326154 | -1.04443704 |
| 8 | 2.58617868 | 0.71817255 | -0.92928587 |
| 6 | 0.56852516 | 0.85408446 | 2.74649469 |
| 6 | -1.62630430 | 0.59229514 | -0.36245256 |
| 6 | -2.84343472 | -0.04729520 | -0.73365805 |
| 6 | -3.98537554 | 0.26456943 | 0.01955412 |
| 6 | -3.96499423 | 1.16017153 | 1.09264294 |
| 6 | -2.77426117 | 1.79660448 | 1.42851701 |
| 6 | -1.60975106 | 1.53452341 | 0.69952636 |
| 6 | -0.35139327 | 2.31396159 | 0.98059889 |
| 1 | -4.87665755 | 1.36004381 | 1.65553848 |
| 6 | -2.90829777 | -1.03721409 | -1.91343725 |
| 6 | 2.76754526 | 2.06319734 | -1.00707581 |
| 6 | 3.26248432 | 2.64975892 | -2.20726267 |
| 6 | 3.43478178 | 4.04144695 | -2.21663315 |
| 6 | 3.14357091 | 4.84967549 | -1.11401091 |
| 6 | 2.67565987 | 4.26241844 | 0.05599992 |
| 6 | 2.49678682 | 2.87564526 | 0.12502920 |
| 6 | 2.09870990 | 2.24897410 | 1.43686158 |
| 1 | 3.28695574 | 5.92843496 | -1.17380319 |
| 6 | 3.59574873 | 1.79616622 | -3.44683305 |
| 6 | -4.33868436 | -1.55719806 | -2.15046847 |
| 6 | -2.01366590 | -2.26649617 | -1.62828776 |
| 6 | -2.44954888 | -0.34110021 | -3.21693960 |
| 6 | 4.14595479 | 2.65209551 | -4.60339317 |
| 6 | 2.32175255 | 1.08826058 | -3.96530195 |
| 6 | 4.67871202 | 0.74857699 | -3.09470460 |
| 1 | 0.54055752 | 1.64323994 | 3.51310129 |
| 1 | -0.39327519 | 0.33473236 | 2.74611460 |
| 1 | -0.53298602 | 3.06114602 | 1.77041179 |
| 1 | -0.01838849 | 2.84466716 | 0.07666391 |
| 1 | -4.93068001 | -0.21256022 | -0.23330447 |
| 1 | -2.74093425 | 2.51046889 | 2.25396347 |
| 1 | 2.87033449 | 1.54010616 | 1.77754481 |
| 1 | 1.99531816 | 3.03003065 | 2.20775515 |
| 1 | 3.80462278 | 4.52080097 | -3.12112886 |
| 1 | 2.45721225 | 4.87391582 | 0.93429638 |
| 1 | -4.33122359 | -2.24454377 | -3.00837583 |
| 1 | -4.72752773 | -2.11370983 | -1.28516420 |
| 1 | -5.04029037 | -0.74355741 | -2.38432217 |
| 1 | -0.96656067 | -1.98048991 | -1.48223228 |
| 1 | -2.35746118 | -2.79398287 | -0.72621599 |

| | | | |
|---|---|---|---|
| 1 | -2.06660798 | -2.97023425 | -2.47292493 |
| 1 | -3.10605108 | 0.50927278 | -3.45206403 |
| 1 | -1.42052886 | 0.02555668 | -3.13754412 |
| 1 | -2.50414808 | -1.05122845 | -4.05617496 |
| 1 | 4.37879506 | 1.99518661 | -5.45346133 |
| 1 | 5.07253355 | 3.17551567 | -4.32651183 |
| 1 | 3.41581031 | 3.39640814 | -4.95267531 |
| 1 | 1.89700404 | 0.41442652 | -3.21388919 |
| 1 | 1.55343791 | 1.82423993 | -4.24251154 |
| 1 | 2.56402938 | 0.49707367 | -4.86148739 |
| 1 | 5.60473995 | 1.24397863 | -2.76803545 |
| 1 | 4.34406488 | 0.07626546 | -2.29734087 |
| 1 | 4.91484745 | 0.14537171 | -3.98453719 |
| 1 | 1.36200540 | 0.13508469 | 2.97686085 |
| 8 | 1.31597297 | -1.58175318 | 0.68002046 |
| 6 | 2.23306106 | -2.49441534 | 0.07386288 |
| 1 | 1.82416417 | -2.93493541 | -0.85300441 |
| 8 | -1.64483743 | -0.26153200 | 5.04050640 |
| 1 | 2.41803105 | -3.31462080 | 0.78523830 |
| 1 | 3.20313594 | -2.02570919 | -0.16590513 |
| 31 | 1.08001887 | 0.06538029 | -0.05739856 |
| 6 | -2.84130465 | -2.29439609 | 4.54447648 |
| 6 | -3.04564702 | -2.72333218 | 3.07459789 |
| 6 | -2.19919838 | -3.92973733 | 2.65550709 |
| 6 | -1.60114135 | -1.44479406 | 4.75871022 |
| 6 | -0.68655681 | -3.73160561 | 2.80235697 |
| 6 | -0.22721073 | -3.41738833 | 4.22230447 |
| 1 | -2.80897737 | -3.18665464 | 5.19163939 |
| 1 | -4.10897684 | -2.97269236 | 2.94069169 |
| 1 | -2.42933917 | -4.18452506 | 1.60934710 |
| 1 | -0.31750090 | -2.93860536 | 2.13075385 |
| 1 | -3.67919819 | -1.67202663 | 4.87893130 |
| 1 | -2.84529128 | -1.86575967 | 2.41289894 |
| 1 | -2.50480029 | -4.80268760 | 3.25925875 |
| 1 | -0.17557240 | -4.65881436 | 2.49422042 |
| 1 | 0.85268839 | -3.58395465 | 4.31917637 |
| 1 | -0.73565925 | -4.05200566 | 4.96694175 |
| 8 | -0.37036878 | -2.01738034 | 4.59850623 |

Electronic energy = -3523.865360 a.u.
DFT-D3(BJ) dispersion correction = -0.113389 a.u.
Thermal free energy = 0.592423 a.u.
Gibbs free energy = -3523.386325 a.u.
Number of imaginary frequencies = 0.

**RC vdW CL isomer/conformer 2**

| Atomic N. | X | Y | Z |
|---|---|---|---|
| 8 | -0.07195404 | -1.81773353 | 0.97876041 |
| 6 | -0.63662723 | -2.24159487 | 2.22076587 |
| 1 | -1.45269540 | -1.58878763 | 2.57392693 |
| 1 | -1.05599145 | -3.25198948 | 2.08853011 |
| 1 | 0.13441761 | -2.29080878 | 3.00994079 |
| 7 | 1.22366036 | 1.15624614 | 1.49474643 |

| | | | |
|---|---|---|---|
| 8 | -1.67298240 | 0.87895484 | 0.76217383 |
| 8 | 0.54735138 | 0.06225553 | -1.23398248 |
| 6 | 1.68118049 | 0.60732268 | 2.79950756 |
| 6 | -1.86623587 | 1.62436717 | 1.88103521 |
| 6 | -3.16036900 | 1.69524890 | 2.47481889 |
| 6 | -3.28478111 | 2.45007626 | 3.65053808 |
| 6 | -2.21310998 | 3.13496937 | 4.23031203 |
| 6 | -0.96963657 | 3.10403086 | 3.60804363 |
| 6 | -0.79004299 | 2.37087083 | 2.42992831 |
| 6 | 0.51229489 | 2.47083679 | 1.67978726 |
| 1 | -2.35946595 | 3.70149562 | 5.14986483 |
| 6 | -4.38351485 | 1.00407360 | 1.83822887 |
| 6 | 1.18715346 | 1.18220506 | -1.65807582 |
| 6 | 0.97369623 | 1.65906289 | -2.98441445 |
| 6 | 1.67626346 | 2.80792949 | -3.37539692 |
| 6 | 2.55261234 | 3.48604614 | -2.52378113 |
| 6 | 2.75433221 | 3.00765174 | -1.23408156 |
| 6 | 2.09138537 | 1.85526148 | -0.79481062 |
| 6 | 2.41340242 | 1.30109997 | 0.57053440 |
| 1 | 3.07053828 | 4.38006159 | -2.87079856 |
| 6 | 0.00484957 | 0.95081318 | -3.95276872 |
| 6 | -5.67363217 | 1.27088053 | 2.63656500 |
| 6 | -4.18574083 | -0.52830655 | 1.78481920 |
| 6 | -4.60370303 | 1.55258732 | 0.40781531 |
| 6 | -0.03080091 | 1.63440240 | -5.33284072 |
| 6 | -1.43408698 | 0.98527444 | -3.38549793 |
| 6 | 0.44805343 | -0.51445141 | -4.17865002 |
| 1 | 2.39083672 | 1.31085447 | 3.26440524 |
| 1 | 0.81970376 | 0.48289945 | 3.46522694 |
| 1 | 1.19766922 | 3.16123606 | 2.19947285 |
| 1 | 0.34063858 | 2.86575370 | 0.66732223 |
| 1 | -4.25598662 | 2.50981431 | 4.13828908 |
| 1 | -0.12903564 | 3.66692912 | 4.02012074 |
| 1 | 2.85180767 | 0.29325148 | 0.49724724 |
| 1 | 3.14898426 | 1.95370130 | 1.06894910 |
| 1 | 1.53157433 | 3.19883982 | -4.38080605 |
| 1 | 3.44277422 | 3.51723798 | -0.55626982 |
| 1 | -6.51458452 | 0.77515964 | 2.13114269 |
| 1 | -5.62016153 | 0.86935625 | 3.65906852 |
| 1 | -5.90917227 | 2.34319366 | 2.69555753 |
| 1 | -3.32452058 | -0.80137506 | 1.16576982 |
| 1 | -4.03986393 | -0.93769780 | 2.79539697 |
| 1 | -5.08108750 | -1.00324825 | 1.35570924 |
| 1 | -4.79330749 | 2.63547624 | 0.43385955 |
| 1 | -3.73472376 | 1.36625399 | -0.23294361 |
| 1 | -5.48104885 | 1.06670933 | -0.04568978 |
| 1 | -0.72600524 | 1.08617622 | -5.98441030 |
| 1 | 0.95395878 | 1.63135947 | -5.82231904 |
| 1 | -0.38637445 | 2.67302946 | -5.27202939 |
| 1 | -1.50339081 | 0.47131542 | -2.42103815 |
| 1 | -1.77274730 | 2.02250721 | -3.25024222 |
| 1 | -2.12282283 | 0.49303087 | -4.08894394 |
| 1 | 1.45834775 | -0.54979756 | -4.61286425 |

| | | | |
|---|---|---|---|
| 1 | 0.44762411 | -1.08036869 | -3.24082684 |
| 1 | -0.23976090 | -1.00612073 | -4.88332769 |
| 1 | 2.17902307 | -0.35758097 | 2.64005647 |
| 31 | -0.08371115 | -0.06469398 | 0.50287608 |
| 6 | 2.33718363 | -4.26581576 | 1.04273566 |
| 6 | 3.22504059 | -5.29428984 | 0.31125006 |
| 6 | 3.14149808 | -5.21745295 | -1.21670067 |
| 6 | 2.93390240 | -2.87495900 | 1.14056918 |
| 6 | 3.55496957 | -3.86607462 | -1.80823064 |
| 6 | 2.74442519 | -2.67724939 | -1.30359045 |
| 1 | 1.35231449 | -4.19575369 | 0.55336145 |
| 1 | 4.26980631 | -5.18043332 | 0.64327768 |
| 1 | 2.10374222 | -5.43476425 | -1.52345017 |
| 1 | 4.62250043 | -3.66581296 | -1.61961418 |
| 1 | 2.16758348 | -4.57968079 | 2.07931356 |
| 1 | 2.90900986 | -6.29877585 | 0.63078124 |
| 1 | 3.76569481 | -6.01209582 | -1.65447943 |
| 1 | 3.43496681 | -3.90090547 | -2.90335413 |
| 1 | 2.88546341 | -1.81047528 | -1.95958327 |
| 1 | 1.66611775 | -2.89211792 | -1.26170455 |
| 8 | 3.23339768 | -2.34983602 | 2.19996979 |
| 8 | 3.18192546 | -2.17706209 | -0.00485436 |

Electronic energy = -3523.868770 a.u.
DFT-D3(BJ) dispersion correction = -0.115346 a.u.
Thermal free energy = 0.598268 a.u.
Gibbs free energy = -3523.385848 a.u.
Number of imaginary frequencies = 0.

**RC vdW CL isomer/conformer 3**

| Atomic N. | X | Y | Z |
|---|---|---|---|
| 7 | -1.90420040 | 1.07408012 | 0.50685013 |
| 8 | 0.47541455 | 1.50632717 | -1.27309124 |
| 8 | 0.63072608 | -0.42918578 | 1.13979928 |
| 6 | -3.21502228 | 0.76470577 | -0.12466297 |
| 6 | -0.27254760 | 2.53223178 | -1.75836443 |
| 6 | 0.06997657 | 3.13851969 | -3.00235127 |
| 6 | -0.76643210 | 4.16469649 | -3.46614654 |
| 6 | -1.88175554 | 4.61564694 | -2.75469088 |
| 6 | -2.16876155 | 4.05600548 | -1.51430115 |
| 6 | -1.36446402 | 3.03229750 | -1.00078747 |
| 6 | -1.58857641 | 2.54305146 | 0.40610242 |
| 1 | -2.50562348 | 5.41089149 | -3.16252099 |
| 6 | 1.32593194 | 2.71735597 | -3.79217322 |
| 6 | 0.58114393 | 0.30238692 | 2.28354509 |
| 6 | 1.75876481 | 0.46808116 | 3.06922274 |
| 6 | 1.64305066 | 1.21350745 | 4.25140774 |
| 6 | 0.44026799 | 1.79001135 | 4.66822438 |
| 6 | -0.70104759 | 1.61864371 | 3.89297759 |
| 6 | -0.64775748 | 0.87037427 | 2.71088913 |
| 6 | -1.92363877 | 0.61365544 | 1.94865970 |
| 1 | 0.40149940 | 2.36737575 | 5.59185890 |
| 6 | 3.11080669 | -0.13639518 | 2.63936767 |

| | | | |
|---|---|---|---|
| 6 | 1.50302121 | 3.54963490 | -5.07622917 |
| 6 | 1.24010494 | 1.23314624 | -4.21524381 |
| 6 | 2.58458439 | 2.93935130 | -2.91931711 |
| 6 | 4.22236710 | 0.15241630 | 3.66614915 |
| 6 | 3.56103907 | 0.47412317 | 1.29105177 |
| 6 | 2.99495926 | -1.67392056 | 2.50998103 |
| 1 | -4.01294674 | 1.31582459 | 0.39879814 |
| 1 | -3.19749161 | 1.07874165 | -1.17429047 |
| 1 | -2.41326779 | 3.10454048 | 0.87618649 |
| 1 | -0.68512878 | 2.70018061 | 1.01400500 |
| 1 | -0.54269595 | 4.63559936 | -4.42154134 |
| 1 | -3.00922120 | 4.42306412 | -0.92095316 |
| 1 | -2.15514088 | -0.46246549 | 1.91083611 |
| 1 | -2.76284028 | 1.12079682 | 2.45241796 |
| 1 | 2.52522737 | 1.36040226 | 4.87168762 |
| 1 | -1.65306720 | 2.05294393 | 4.20609416 |
| 1 | 2.41985129 | 3.22449021 | -5.58817434 |
| 1 | 0.66705875 | 3.41341959 | -5.77777768 |
| 1 | 1.60715845 | 4.62308058 | -4.86264652 |
| 1 | 1.20170209 | 0.57128487 | -3.34401284 |
| 1 | 0.34866739 | 1.05404057 | -4.83424169 |
| 1 | 2.12522061 | 0.96577712 | -4.81232378 |
| 1 | 2.68728326 | 4.00089470 | -2.65137454 |
| 1 | 2.54342991 | 2.34962914 | -1.99670649 |
| 1 | 3.48394116 | 2.64417495 | -3.48105658 |
| 1 | 5.15831193 | -0.30643310 | 3.31703349 |
| 1 | 3.99389523 | -0.27226036 | 4.65446858 |
| 1 | 4.40783087 | 1.22966824 | 3.78439949 |
| 1 | 2.84220821 | 0.26768693 | 0.49109568 |
| 1 | 3.67763922 | 1.56389892 | 1.37866050 |
| 1 | 4.53426826 | 0.05173276 | 0.99799736 |
| 1 | 2.70477981 | -2.12187335 | 3.47202570 |
| 1 | 2.25513086 | -1.95582842 | 1.75299382 |
| 1 | 3.96909281 | -2.09680357 | 2.22066292 |
| 1 | -3.41348697 | -0.31141244 | -0.05448297 |
| 8 | -0.81217505 | -1.38702222 | -1.29009315 |
| 6 | -1.15239896 | -1.30593282 | -2.67436261 |
| 1 | -1.56480268 | -0.32582462 | -2.97155101 |
| 6 | -2.57013355 | -4.28959849 | -0.51640973 |
| 6 | -2.52071323 | -5.71516611 | 0.07221095 |
| 6 | -1.19157977 | -6.06621893 | 0.74896808 |
| 6 | -2.86658487 | -3.20254319 | 0.49891822 |
| 6 | -0.81057848 | -5.15109802 | 1.91722857 |
| 6 | -0.69712977 | -3.67378784 | 1.55630677 |
| 1 | -1.61995020 | -4.05382032 | -1.02251262 |
| 1 | -3.35625173 | -5.84911517 | 0.77824771 |
| 1 | -0.38975348 | -6.02365954 | -0.00837654 |
| 1 | -1.53012037 | -5.25325812 | 2.74612241 |
| 1 | -3.37042063 | -4.21410438 | -1.26174058 |
| 1 | -2.69595950 | -6.42573860 | -0.74959221 |
| 1 | -1.22861489 | -7.10900222 | 1.10105468 |
| 1 | 0.16765494 | -5.46583191 | 2.31580540 |
| 1 | -0.15324317 | -3.12834371 | 2.33598654 |

| | | | |
|---|---|---|---|
| 1 | -0.16850736 | -3.51261895 | 0.60455471 |
| 8 | -3.87679233 | -2.51962938 | 0.47024283 |
| 8 | -1.98062072 | -2.98195535 | 1.51156526 |
| 1 | -0.27480435 | -1.50621898 | -3.31211323 |
| 1 | -1.91786919 | -2.06852968 | -2.89240328 |
| 31 | -0.32989212 | 0.09520664 | -0.35404429 |

Electronic energy = -3523.868549 a.u.
DFT-D3(BJ) dispersion correction = -0.115000 a.u.
Thermal free energy = 0.598171 a.u.
Gibbs free energy = -3523.385377 a.u.
Number of imaginary frequencies = 0.

**RC vdW CL isomer/conformer 4**

| Atomic N. | X | Y | Z |
|---|---|---|---|
| 7 | -0.62836909 | 1.60175534 | 1.46677415 |
| 8 | -1.50112939 | 0.49161124 | -1.17915526 |
| 8 | 1.37808129 | 0.02748696 | -0.14850205 |
| 6 | -1.47625869 | 1.35070562 | 2.66280615 |
| 6 | -2.58305854 | 1.28808392 | -0.98298349 |
| 6 | -3.76332814 | 1.09114377 | -1.75814917 |
| 6 | -4.85956236 | 1.91996937 | -1.47743070 |
| 6 | -4.82434075 | 2.92196381 | -0.50345242 |
| 6 | -3.64672444 | 3.14247231 | 0.20307765 |
| 6 | -2.52075553 | 2.34827641 | -0.04000015 |
| 6 | -1.20701820 | 2.69600007 | 0.60963067 |
| 1 | -5.70632233 | 3.53463430 | -0.31709737 |
| 6 | -3.82722431 | 0.03867300 | -2.88384285 |
| 6 | 2.03924492 | 1.21303590 | -0.17261846 |
| 6 | 3.04564472 | 1.44110553 | -1.15635752 |
| 6 | 3.70883206 | 2.67625515 | -1.12442412 |
| 6 | 3.41649900 | 3.66802359 | -0.18426714 |
| 6 | 2.43638982 | 3.43030660 | 0.77271861 |
| 6 | 1.75274421 | 2.20873607 | 0.79836205 |
| 6 | 0.77614237 | 1.94089450 | 1.91627303 |
| 1 | 3.95360740 | 4.61618207 | -0.20411926 |
| 6 | 3.39189306 | 0.37958035 | -2.21985610 |
| 6 | -5.18607851 | 0.05566480 | -3.60918005 |
| 6 | -3.62610059 | -1.38647786 | -2.31948455 |
| 6 | -2.73643480 | 0.34110356 | -3.93941762 |
| 6 | 4.53065547 | 0.84411476 | -3.14743294 |
| 6 | 2.15968228 | 0.09676918 | -3.11155105 |
| 6 | 3.86105351 | -0.92769841 | -1.53770212 |
| 1 | -1.52562287 | 2.26508981 | 3.27613410 |
| 1 | -2.48966043 | 1.08402946 | 2.34236575 |
| 1 | -1.31952461 | 3.59759696 | 1.23496737 |
| 1 | -0.44744890 | 2.91054414 | -0.15698707 |
| 1 | -5.78166438 | 1.78284413 | -2.03929347 |
| 1 | -3.58296534 | 3.94903939 | 0.93704422 |
| 1 | 1.10051714 | 1.08682779 | 2.53073846 |
| 1 | 0.71500114 | 2.82504284 | 2.57203804 |
| 1 | 4.47929180 | 2.88195824 | -1.86527682 |
| 1 | 2.20069148 | 4.18813120 | 1.52322068 |

| | | | |
|---|---|---|---|
| 1 | -5.17109050 | -0.69510739 | -4.41201437 |
| 1 | -6.01922574 | -0.19658773 | -2.93698759 |
| 1 | -5.39542532 | 1.03064485 | -4.07223418 |
| 1 | -2.64175752 | -1.49886687 | -1.85255259 |
| 1 | -4.40070381 | -1.62344273 | -1.57515306 |
| 1 | -3.70574486 | -2.12275873 | -3.13371052 |
| 1 | -2.89437649 | 1.33395450 | -4.38506654 |
| 1 | -1.73282423 | 0.31159294 | -3.50084618 |
| 1 | -2.78494319 | -0.40396014 | -4.74814657 |
| 1 | 4.74393609 | 0.04897961 | -3.87595701 |
| 1 | 5.45988275 | 1.04476546 | -2.59459698 |
| 1 | 4.26129586 | 1.74662087 | -3.71471604 |
| 1 | 1.31673589 | -0.29002499 | -2.52932971 |
| 1 | 1.83436483 | 1.01313525 | -3.62469232 |
| 1 | 2.41947620 | -0.64760937 | -3.87960848 |
| 1 | 4.76008001 | -0.74464124 | -0.93032534 |
| 1 | 3.07882551 | -1.34417849 | -0.89384787 |
| 1 | 4.11778568 | -1.67546097 | -2.30354484 |
| 1 | -1.03591852 | 0.54122964 | 3.25823343 |
| 8 | -0.78894824 | -1.64674921 | 1.01036759 |
| 6 | -2.12932830 | -2.04175314 | 1.29911298 |
| 1 | -2.86729668 | -1.63642120 | 0.58617731 |
| 8 | 0.29733888 | -0.82127874 | 4.61025125 |
| 1 | -2.42717203 | -1.73388704 | 2.31757604 |
| 1 | -2.18738039 | -3.14087962 | 1.24687726 |
| 31 | -0.42823491 | -0.03383351 | 0.25724489 |
| 6 | 1.27846757 | -3.02006313 | 4.70179129 |
| 6 | 0.87801068 | -4.08826082 | 3.66153179 |
| 6 | 1.96375813 | -4.37813203 | 2.62076396 |
| 6 | 1.14655538 | -1.59425130 | 4.19821368 |
| 6 | 2.40037750 | -3.15709818 | 1.80462789 |
| 6 | 2.99649790 | -2.02207088 | 2.62957722 |
| 1 | 2.31096398 | -3.20314339 | 5.04335399 |
| 1 | 0.64717226 | -5.01703077 | 4.20483704 |
| 1 | 1.60290249 | -5.15963501 | 1.93432153 |
| 1 | 1.55798750 | -2.75649903 | 1.21917914 |
| 1 | 0.62270239 | -3.07878144 | 5.57824981 |
| 1 | -0.05220160 | -3.77611728 | 3.16229094 |
| 1 | 2.84507899 | -4.80131135 | 3.13484156 |
| 1 | 3.17156052 | -3.46696938 | 1.08068780 |
| 1 | 3.55626662 | -1.33603303 | 1.98325796 |
| 1 | 3.68205970 | -2.39573473 | 3.40831716 |
| 8 | 2.00610954 | -1.14382617 | 3.24407751 |

Electronic energy = -3523.868177 a.u.
DFT-D3(BJ) dispersion correction = -0.115250 a.u.
Thermal free energy = 0.598183 a.u.
Gibbs free energy = -3523.385244 a.u.
Number of imaginary frequencies = 0.

**RC_vdW_CL isomer/conformer 5**

| Atomic N. | X | Y | Z |
|---|---|---|---|
| 8 | -0.41230696 | -1.82219213 | 0.53856922 |

| | | | |
|---|---|---|---|
| 6 | -0.33434883 | -2.84002997 | 1.54342406 |
| 1 | -1.31454611 | -2.98669029 | 2.02962984 |
| 1 | -0.04762076 | -3.78848307 | 1.06155351 |
| 1 | 0.41502888 | -2.62014976 | 2.32235823 |
| 7 | -1.23160631 | 1.08787672 | 1.79659827 |
| 8 | 0.59867496 | 0.85521438 | -0.56403282 |
| 8 | 1.47657582 | -0.14311311 | 2.25623034 |
| 6 | -2.61803425 | 0.55906049 | 1.70856017 |
| 6 | -0.36611374 | 1.58750274 | -1.18160816 |
| 6 | -0.41907723 | 1.64257481 | -2.60406672 |
| 6 | -1.45093616 | 2.40115334 | -3.17676117 |
| 6 | -2.39520218 | 3.09524214 | -2.41484420 |
| 6 | -2.30645509 | 3.06504343 | -1.02696125 |
| 6 | -1.29110891 | 2.33185106 | -0.40363295 |
| 6 | -1.11395154 | 2.41427405 | 1.08995785 |
| 1 | -3.18407409 | 3.66360569 | -2.90734773 |
| 6 | 0.62191856 | 0.91766396 | -3.48053999 |
| 6 | 1.68213342 | 0.97313087 | 3.00332667 |
| 6 | 3.00742421 | 1.38152826 | 3.32950033 |
| 6 | 3.14651251 | 2.53614164 | 4.11317644 |
| 6 | 2.05459498 | 3.27900420 | 4.57144970 |
| 6 | 0.76653982 | 2.86135824 | 4.25673177 |
| 6 | 0.56948687 | 1.70856974 | 3.48719169 |
| 6 | -0.83203593 | 1.21007717 | 3.24930363 |
| 1 | 2.21581504 | 4.17392763 | 5.17223553 |
| 6 | 4.23982965 | 0.59312395 | 2.84394870 |
| 6 | 0.39268575 | 1.18074584 | -4.98097472 |
| 6 | 0.53992072 | -0.61158906 | -3.26473893 |
| 6 | 2.04356040 | 1.42405092 | -3.13765712 |
| 6 | 5.55514084 | 1.21291490 | 3.35267516 |
| 6 | 4.29904174 | 0.59452348 | 1.29808529 |
| 6 | 4.18250847 | -0.86064517 | 3.37076524 |
| 1 | -3.30735625 | 1.23017251 | 2.24556436 |
| 1 | -2.92766726 | 0.48927604 | 0.66206121 |
| 1 | -1.85844802 | 3.10242872 | 1.52403309 |
| 1 | -0.11304751 | 2.79817000 | 1.33671927 |
| 1 | -1.52639801 | 2.45518356 | -4.26131998 |
| 1 | -3.01528209 | 3.62580020 | -0.41371295 |
| 1 | -0.96264357 | 0.20194320 | 3.67412772 |
| 1 | -1.55496979 | 1.87908759 | 3.74475641 |
| 1 | 4.14608768 | 2.87846934 | 4.37448063 |
| 1 | -0.10081768 | 3.41886640 | 4.61716253 |
| 1 | 1.16815872 | 0.65673259 | -5.55759888 |
| 1 | -0.58284607 | 0.80647257 | -5.32424746 |
| 1 | 0.46192861 | 2.24981282 | -5.22833758 |
| 1 | 0.71030884 | -0.88728193 | -2.21833194 |
| 1 | -0.44665687 | -0.99045860 | -3.57104974 |
| 1 | 1.29867660 | -1.11529468 | -3.88286972 |
| 1 | 2.12353605 | 2.50475145 | -3.32501116 |
| 1 | 2.29901091 | 1.23273465 | -2.08994158 |
| 1 | 2.78222180 | 0.91532874 | -3.77556961 |
| 1 | 6.39790955 | 0.60768489 | 2.98976925 |
| 1 | 5.60792927 | 1.22818179 | 4.45092889 |

| | | | |
|---|---|---|---|
| 1 | 5.70193171 | 2.23768292 | 2.98195018 |
| 1 | 3.42031808 | 0.11256679 | 0.85682463 |
| 1 | 4.36077951 | 1.62221082 | 0.91212445 |
| 1 | 5.19483227 | 0.05141924 | 0.96072376 |
| 1 | 4.18254158 | -0.87406317 | 4.47053632 |
| 1 | 3.28725830 | -1.38222609 | 3.01568680 |
| 1 | 5.06886440 | -1.41494578 | 3.02662633 |
| 1 | -2.66556239 | -0.44214052 | 2.14991615 |
| 31 | 0.16643578 | -0.14709864 | 0.93761043 |
| 8 | -4.43362099 | -1.76677044 | 0.31513789 |
| 6 | -3.18881981 | -1.80229977 | -1.75027351 |
| 6 | -3.75241537 | -2.44738392 | -3.03409273 |
| 6 | -3.08181714 | -3.76973937 | -3.42022348 |
| 6 | -3.73633975 | -2.39228758 | -0.46309778 |
| 6 | -3.17656513 | -4.86531686 | -2.35326488 |
| 6 | -2.57074534 | -4.48338154 | -1.00542632 |
| 1 | -2.08914659 | -1.86858077 | -1.73847706 |
| 1 | -4.84031541 | -2.58787069 | -2.92639543 |
| 1 | -2.01615472 | -3.57541425 | -3.63167049 |
| 1 | -4.22428646 | -5.16658140 | -2.19018151 |
| 1 | -3.45354447 | -0.73878846 | -1.72026096 |
| 1 | -3.61574289 | -1.73059569 | -3.85783630 |
| 1 | -3.52148171 | -4.13828335 | -4.36027821 |
| 1 | -2.64616160 | -5.76277528 | -2.71153185 |
| 1 | -2.39251037 | -5.38173638 | -0.40095621 |
| 1 | -1.61134737 | -3.95565169 | -1.12446599 |
| 8 | -3.45599358 | -3.69527708 | -0.16242284 |

Electronic energy = -3523.865723 a.u.
DFT-D3(BJ) dispersion correction = -0.114063 a.u.
Thermal free energy = 0.594742 a.u.
Gibbs free energy = -3523.385043 a.u.
Number of imaginary frequencies = 0.

**RC_vdW_CL isomer/conformer 6**

| Atomic N. | X | Y | Z |
|---|---|---|---|
| 7 | -0.83797972 | 2.08072921 | 0.28183780 |
| 8 | 1.01996779 | 0.62439609 | -1.57346842 |
| 8 | 0.34544777 | -0.53231349 | 1.21671099 |
| 6 | -2.16081699 | 2.52572978 | -0.23217063 |
| 6 | 0.96349448 | 1.77971995 | -2.28664623 |
| 6 | 1.40731545 | 1.80525411 | -3.64129302 |
| 6 | 1.28256228 | 3.01904917 | -4.33332172 |
| 6 | 0.77347729 | 4.18008882 | -3.74489909 |
| 6 | 0.40312343 | 4.15661325 | -2.40450473 |
| 6 | 0.51081552 | 2.97386895 | -1.66481636 |
| 6 | 0.26246625 | 2.99831934 | -0.17922059 |
| 1 | 0.68874804 | 5.09692183 | -4.32818992 |
| 6 | 2.03622448 | 0.56747282 | -4.31286524 |
| 6 | 0.92836673 | 0.25806647 | 2.15525475 |
| 6 | 2.06932485 | -0.21455070 | 2.86720594 |
| 6 | 2.62046076 | 0.63556664 | 3.83671359 |
| 6 | 2.10154205 | 1.90280162 | 4.11501584 |

| | | | |
|---|---|---|---|
| 6 | 0.98518657 | 2.34887745 | 3.41654522 |
| 6 | 0.38338808 | 1.53597362 | 2.44844845 |
| 6 | -0.89175567 | 2.00300813 | 1.79226198 |
| 1 | 2.56946247 | 2.53146886 | 4.87254419 |
| 6 | 2.68314869 | -1.60045232 | 2.58342324 |
| 6 | 2.47688525 | 0.86039255 | -5.75953305 |
| 6 | 1.02877740 | -0.60340825 | -4.36774541 |
| 6 | 3.29546883 | 0.13524468 | -3.52360654 |
| 6 | 3.88568566 | -1.89620049 | 3.49973107 |
| 6 | 3.19035503 | -1.66962161 | 1.12353528 |
| 6 | 1.63456513 | -2.71099553 | 2.83105740 |
| 1 | -2.38580097 | 3.53227397 | 0.15591056 |
| 1 | -2.13354129 | 2.56422966 | -1.32690346 |
| 1 | 0.01513155 | 4.02242507 | 0.14716890 |
| 1 | 1.16555002 | 2.68108990 | 0.36348592 |
| 1 | 1.59484639 | 3.06643340 | -5.37488186 |
| 1 | 0.04622059 | 5.06374064 | -1.91155025 |
| 1 | -1.72928199 | 1.32663702 | 2.02211330 |
| 1 | -1.15592993 | 3.00421996 | 2.17072495 |
| 1 | 3.49470698 | 0.30450778 | 4.39414921 |
| 1 | 0.55693725 | 3.33136911 | 3.62720555 |
| 1 | 2.93608284 | -0.04442895 | -6.18243479 |
| 1 | 1.62878111 | 1.13268756 | -6.40465752 |
| 1 | 3.22487687 | 1.66459937 | -5.80963034 |
| 1 | 0.73937080 | -0.92774053 | -3.36293369 |
| 1 | 0.12256887 | -0.31411707 | -4.91996304 |
| 1 | 1.48326623 | -1.45973508 | -4.88891221 |
| 1 | 4.04419380 | 0.94061343 | -3.51832648 |
| 1 | 3.05172285 | -0.12063837 | -2.48637400 |
| 1 | 3.75026430 | -0.74579011 | -4.00161247 |
| 1 | 4.27604937 | -2.89654123 | 3.26423138 |
| 1 | 3.60701060 | -1.89137823 | 4.56352699 |
| 1 | 4.70601332 | -1.17928989 | 3.35147597 |
| 1 | 2.37853070 | -1.52027225 | 0.40398221 |
| 1 | 3.95770328 | -0.90310729 | 0.94328722 |
| 1 | 3.64393282 | -2.65434701 | 0.93343451 |
| 1 | 1.29755864 | -2.69670583 | 3.87836367 |
| 1 | 0.76340917 | -2.58896313 | 2.17830966 |
| 1 | 2.08314556 | -3.69679408 | 2.63453933 |
| 1 | -2.93523303 | 1.82769588 | 0.10629474 |
| 8 | -1.74133640 | -0.77963876 | -0.90993600 |
| 6 | -2.14548771 | -0.78251403 | -2.27824405 |
| 1 | -1.93813243 | 0.16710935 | -2.80258977 |
| 6 | -5.20922978 | -1.58893831 | 1.74822622 |
| 6 | -4.76551163 | -2.63244460 | 0.70018591 |
| 6 | -3.79031384 | -3.68136648 | 1.24321359 |
| 6 | -4.19282857 | -0.48533483 | 1.97675660 |
| 6 | -2.49039449 | -3.10253255 | 1.81152000 |
| 6 | -2.67997790 | -2.14679066 | 2.98402851 |
| 1 | -5.43800390 | -2.09343387 | 2.70180200 |
| 1 | -5.66764452 | -3.14160598 | 0.32877103 |
| 1 | -4.29864991 | -4.26599056 | 2.03057297 |
| 1 | -1.85604531 | -3.92958496 | 2.17005689 |

| | | | |
|---|---|---|---|
| 1 | -6.12054058 | -1.07891710 | 1.41504080 |
| 1 | -4.31887844 | -2.11151629 | -0.16071937 |
| 1 | -3.54597950 | -4.39357527 | 0.43996342 |
| 1 | -1.91940943 | -2.58233395 | 1.02650138 |
| 1 | -1.73531317 | -2.01556158 | 3.52458316 |
| 1 | -3.43414869 | -2.51727873 | 3.69813589 |
| 8 | -4.37437866 | 0.66848847 | 1.62458390 |
| 8 | -3.01704509 | -0.78188143 | 2.59495593 |
| 1 | -1.64792162 | -1.59005601 | -2.84182128 |
| 1 | -3.23340549 | -0.95533586 | -2.31934027 |
| 31 | -0.32321760 | 0.20633934 | -0.34427622 |

Electronic energy = -3523.868085 a.u.
DFT-D3(BJ) dispersion correction = -0.115117 a.u.
Thermal free energy = 0.598194 a.u.
Gibbs free energy = -3523.385008 a.u.
Number of imaginary frequencies = 0.

**RC vdW CL isomer/conformer 7**

| Atomic N. | X | Y | Z |
|---|---|---|---|
| 7 | -1.03979615 | 2.10823093 | -0.02862843 |
| 8 | 1.14999742 | 0.67253968 | -1.48789204 |
| 8 | 0.35550597 | -0.15488822 | 1.39897420 |
| 6 | -2.37398751 | 2.27760111 | -0.66417958 |
| 6 | 0.98132281 | 1.66157788 | -2.40359319 |
| 6 | 1.51206487 | 1.51814714 | -3.71861560 |
| 6 | 1.26958451 | 2.56247468 | -4.62337022 |
| 6 | 0.56120088 | 3.71718698 | -4.27860626 |
| 6 | 0.09762117 | 3.86796675 | -2.97573367 |
| 6 | 0.31646443 | 2.85967582 | -2.03091051 |
| 6 | -0.04430282 | 3.09129072 | -0.58738885 |
| 6 | 2.34519244 | 0.28596250 | -4.12601386 |
| 6 | 0.79340286 | 0.84280756 | 2.21220146 |
| 6 | 1.92497125 | 0.62848255 | 3.05073874 |
| 6 | 2.32363349 | 1.69093389 | 3.87471432 |
| 6 | 1.66220477 | 2.92183416 | 3.89595149 |
| 6 | 0.55119933 | 3.11196606 | 3.08184108 |
| 6 | 0.09982691 | 2.08063393 | 2.24943032 |
| 6 | -1.17781846 | 2.26976359 | 1.47093229 |
| 6 | 2.68440042 | -0.71330831 | 3.05663142 |
| 6 | 2.85040502 | 0.38921790 | -5.57751692 |
| 6 | 1.50259140 | -1.00637050 | -4.02355355 |
| 6 | 3.58829486 | 0.17324986 | -3.21092424 |
| 6 | 3.84413636 | -0.71285040 | 4.07060651 |
| 6 | 3.29409141 | -0.98860491 | 1.66176057 |
| 6 | 1.72884724 | -1.86423879 | 3.45315506 |
| 1 | -2.75678817 | 3.28888823 | -0.45134286 |
| 1 | -2.28014174 | 2.15445268 | -1.74894580 |
| 1 | -0.45522882 | 4.10627082 | -0.45542300 |
| 1 | 0.85026167 | 3.00379040 | 0.04710614 |
| 1 | 1.64690338 | 2.47672034 | -5.64075962 |
| 1 | -0.42058464 | 4.78081463 | -2.67322623 |
| 1 | -1.94302195 | 1.53808520 | 1.77491825 |

| | | | |
|---|---|---|---|
| 1 | -1.58051026 | 3.27831557 | 1.66210463 |
| 1 | 3.18825635 | 1.56093341 | 4.52292047 |
| 1 | 0.01026407 | 4.06079423 | 3.09616867 |
| 1 | 3.45284836 | -0.50051905 | -5.80955998 |
| 1 | 2.02494287 | 0.42722394 | -6.30331658 |
| 1 | 3.48973004 | 1.27044822 | -5.73100515 |
| 1 | 1.16501433 | -1.18747910 | -2.99747860 |
| 1 | 0.62224255 | -0.95059205 | -4.68081741 |
| 1 | 2.10680175 | -1.86897845 | -4.34336014 |
| 1 | 4.22254287 | 1.06631177 | -3.30840607 |
| 1 | 3.30460228 | 0.06027070 | -2.15871005 |
| 1 | 4.18851050 | -0.70107427 | -3.50546562 |
| 1 | 4.34145656 | -1.69286297 | 4.04400288 |
| 1 | 3.49408880 | -0.54708678 | 5.09979523 |
| 1 | 4.60206436 | 0.04764037 | 3.83350469 |
| 1 | 2.52261870 | -1.06206900 | 0.88813303 |
| 1 | 3.99298216 | -0.18834314 | 1.37881180 |
| 1 | 3.85385935 | -1.93611494 | 1.68140831 |
| 1 | 1.32119985 | -1.69765093 | 4.46116162 |
| 1 | 0.89479584 | -1.95297150 | 2.74850790 |
| 1 | 2.28007581 | -2.81691735 | 3.46457583 |
| 1 | -3.06836365 | 1.53382456 | -0.25495303 |
| 31 | -0.23381630 | 0.24613505 | -0.30815062 |
| 8 | -1.37239956 | -1.06615430 | -0.84207694 |
| 6 | -1.93034274 | -1.05691386 | -2.15621284 |
| 1 | -1.97876543 | -2.09298402 | -2.52987407 |
| 1 | -2.95633249 | -0.64869160 | -2.14193111 |
| 1 | -1.33642176 | -0.47552163 | -2.88206850 |
| 1 | 0.39259012 | 4.49702858 | -5.02117004 |
| 1 | 2.01465119 | 3.72081050 | 4.54819333 |
| 8 | -3.78198483 | 0.08213910 | 1.50421694 |
| 8 | -4.43951677 | -1.69430093 | 0.36636044 |
| 6 | -3.66383289 | -1.12095361 | 1.32735074 |
| 6 | -2.73279689 | -1.99263520 | 2.14702983 |
| 1 | -2.00661366 | -2.47294489 | 1.47201735 |
| 1 | -2.16770212 | -1.30151384 | 2.78220746 |
| 6 | -3.44969093 | -3.05095350 | 3.01146628 |
| 1 | -4.32765519 | -2.59634295 | 3.49869526 |
| 1 | -2.76464044 | -3.34046964 | 3.82227618 |
| 6 | -3.86166939 | -4.31240145 | 2.24561404 |
| 1 | -2.95127958 | -4.80193684 | 1.85871780 |
| 1 | -4.32376694 | -5.02738534 | 2.94419226 |
| 6 | -4.81746714 | -4.06048079 | 1.07493300 |
| 1 | -5.78601134 | -3.67588882 | 1.43418909 |
| 1 | -5.03010626 | -5.01757453 | 0.57097750 |
| 6 | -4.28096397 | -3.09845812 | 0.01906225 |
| 1 | -3.22423229 | -3.29652314 | -0.21879257 |
| 1 | -4.86171057 | -3.18849234 | -0.90758292 |

Electronic energy = -3523.868312 a.u.
DFT-D3(BJ) dispersion correction = -0.114872 a.u.
Thermal free energy = 0.598911 a.u.
Gibbs free energy = -3523.384273 a.u.
Number of imaginary frequencies = 0.

**RC vdW CL isomer/conformer 8**

| Atomic N. | X | Y | Z |
|---|---|---|---|
| 7 | -0.94923156 | 1.09640876 | 1.44808298 |
| 8 | -0.52181852 | 1.55536739 | -1.48731412 |
| 8 | 0.98505188 | -0.71220565 | 0.01341653 |
| 6 | -2.32131987 | 0.90464435 | 1.98852136 |
| 6 | -1.29003538 | 2.65674858 | -1.27278755 |
| 6 | -1.86755261 | 3.35335375 | -2.37398142 |
| 6 | -2.68366413 | 4.45501043 | -2.07653192 |
| 6 | -2.92038975 | 4.89466732 | -0.77105572 |
| 6 | -2.29708747 | 4.24497396 | 0.28924661 |
| 6 | -1.46939631 | 3.14255098 | 0.04960637 |
| 6 | -0.66727185 | 2.55032798 | 1.17818646 |
| 1 | -3.56987554 | 5.75150509 | -0.59221232 |
| 6 | -1.57823869 | 2.94706918 | -3.83331668 |
| 6 | 1.90479628 | -0.07819000 | 0.78949849 |
| 6 | 3.28383420 | -0.11940728 | 0.43471169 |
| 6 | 4.18430296 | 0.54197431 | 1.28263597 |
| 6 | 3.77950000 | 1.22326743 | 2.43370149 |
| 6 | 2.43147849 | 1.24426477 | 2.77426549 |
| 6 | 1.48995812 | 0.58946381 | 1.97140037 |
| 6 | 0.05378325 | 0.52268420 | 2.42516469 |
| 1 | 4.51659523 | 1.72895144 | 3.05740175 |
| 6 | 3.77316573 | -0.85825148 | -0.82698520 |
| 6 | -2.27462538 | 3.88330356 | -4.83893643 |
| 6 | -2.07848370 | 1.51277099 | -4.11863990 |
| 6 | -0.05586742 | 3.03067021 | -4.09993335 |
| 6 | 5.30507250 | -0.79039364 | -0.97490070 |
| 6 | 3.16070926 | -0.21605523 | -2.09406471 |
| 6 | 3.38496451 | -2.35439159 | -0.75211518 |
| 1 | -2.40865867 | 1.40616446 | 2.96512187 |
| 1 | -3.05015610 | 1.33687010 | 1.29363769 |
| 1 | -0.84648954 | 3.11361766 | 2.10928002 |
| 1 | 0.40750786 | 2.60833513 | 0.94983092 |
| 1 | -3.15558984 | 4.99714999 | -2.89386014 |
| 1 | -2.42907396 | 4.60132267 | 1.31336686 |
| 1 | -0.26366396 | -0.51705861 | 2.59136722 |
| 1 | -0.06196990 | 1.05882545 | 3.38090398 |
| 1 | 5.24445827 | 0.53437018 | 1.03623007 |
| 1 | 2.09639888 | 1.75623519 | 3.67900493 |
| 1 | -2.01886977 | 3.56423459 | -5.85930415 |
| 1 | -3.37013855 | 3.84981819 | -4.74742152 |
| 1 | -1.94768148 | 4.92706544 | -4.72751290 |
| 1 | -1.56024311 | 0.77728806 | -3.49471731 |
| 1 | -3.15991007 | 1.43219056 | -3.93467444 |
| 1 | -1.89550100 | 1.25913124 | -5.17389020 |
| 1 | 0.30782578 | 4.05733765 | -3.94859126 |
| 1 | 0.50542197 | 2.36093769 | -3.43873188 |
| 1 | 0.15535798 | 2.74752117 | -5.14237629 |
| 1 | 5.60078524 | -1.34117657 | -1.87912730 |
| 1 | 5.82415505 | -1.25117991 | -0.12196618 |

| | | | |
|---|---|---|---|
| 1 | 5.66571411 | 0.24241082 | -1.08556319 |
| 1 | 2.06748300 | -0.27830578 | -2.09312931 |
| 1 | 3.44705472 | 0.84279307 | -2.16985681 |
| 1 | 3.53447465 | -0.73362977 | -2.99065362 |
| 1 | 3.84776214 | -2.82960035 | 0.12552980 |
| 1 | 2.29900847 | -2.48415947 | -0.68914890 |
| 1 | 3.74782011 | -2.87751680 | -1.65000944 |
| 1 | -2.51251864 | -0.16660992 | 2.11501064 |
| 8 | -1.89184174 | -1.17431433 | -0.64243039 |
| 6 | -3.09266967 | -0.88969702 | -1.35856521 |
| 1 | -2.96209233 | -1.05452793 | -2.44128651 |
| 1 | -3.87984523 | -1.57232755 | -0.99964877 |
| 1 | -3.45511883 | 0.14412034 | -1.21835874 |
| 31 | -0.64318820 | 0.10512921 | -0.31636371 |
| 6 | -1.28063821 | -4.33278149 | 4.30196055 |
| 6 | 0.11697792 | -4.88115781 | 3.94003847 |
| 6 | 0.23926698 | -5.37635454 | 2.49518712 |
| 6 | -1.51398850 | -2.90908716 | 3.82982708 |
| 6 | -0.06496508 | -4.32028502 | 1.42730788 |
| 6 | -1.46589515 | -3.72311129 | 1.49401921 |
| 1 | -2.05755187 | -4.99911600 | 3.89206346 |
| 1 | 0.87380244 | -4.10693346 | 4.14459564 |
| 1 | -0.44782368 | -6.22948478 | 2.35601995 |
| 1 | 0.66415695 | -3.49499557 | 1.46372762 |
| 1 | -1.40762372 | -4.30255994 | 5.39034276 |
| 1 | 0.33884644 | -5.71631628 | 4.62129850 |
| 1 | 1.25472411 | -5.77091373 | 2.33562709 |
| 1 | 0.04363366 | -4.77607868 | 0.42985537 |
| 1 | -1.70438468 | -3.19743205 | 0.56051820 |
| 1 | -2.23189566 | -4.49483773 | 1.67504592 |
| 8 | -1.60335750 | -1.95910793 | 4.58691824 |
| 8 | -1.60764632 | -2.66329498 | 2.49048605 |

Electronic energy = -3523.867182 a.u.
DFT-D3(BJ) dispersion correction = -0.113729 a.u.
Thermal free energy = 0.596955 a.u.
Gibbs free energy = -3523.383957 a.u.
Number of imaginary frequencies = 0.

**RC vdW CL isomer/conformer 9**

| Atomic N. | X | Y | Z |
|---|---|---|---|
| 8 | -0.36979240 | -1.76680205 | -0.71430184 |
| 6 | -0.95050592 | -2.31032849 | -1.89807897 |
| 1 | -0.51426245 | -3.30631977 | -2.07426414 |
| 8 | -2.84936094 | -1.88542118 | 2.32201887 |
| 1 | -2.04362843 | -2.43202389 | -1.79007626 |
| 1 | -0.75999307 | -1.69968219 | -2.79666372 |
| 7 | -1.92337823 | 1.03678318 | -0.05165144 |
| 8 | 0.41960938 | 1.02782618 | -1.92485004 |
| 8 | 0.77469139 | 0.30543931 | 1.07733980 |
| 6 | -3.14535798 | 0.25776957 | -0.38412136 |
| 6 | -0.46788678 | 1.57260788 | -2.79728912 |
| 6 | -0.17129178 | 1.60683197 | -4.19111862 |

| | | | |
|---|---|---|---|
| 6 | -1.14969506 | 2.14506604 | -5.04021767 |
| 6 | -2.36345270 | 2.65676205 | -4.57234494 |
| 6 | -2.61251014 | 2.67346743 | -3.20390144 |
| 6 | -1.66897198 | 2.15382888 | -2.31100659 |
| 6 | -1.86728969 | 2.32545063 | -0.82762761 |
| 1 | -3.09490510 | 3.05690844 | -5.27438670 |
| 6 | 1.17978116 | 1.10988187 | -4.74485062 |
| 6 | 0.62527227 | 1.47066191 | 1.76068129 |
| 6 | 1.76244516 | 2.10562493 | 2.33810149 |
| 6 | 1.54358691 | 3.30079707 | 3.03829399 |
| 6 | 0.27659471 | 3.87291161 | 3.18242787 |
| 6 | -0.82618987 | 3.23454674 | 2.62590819 |
| 6 | -0.66802558 | 2.03118371 | 1.92802107 |
| 6 | -1.89079736 | 1.29700975 | 1.43940115 |
| 1 | 0.15801943 | 4.80837222 | 3.72914281 |
| 6 | 3.17705761 | 1.50779917 | 2.20255676 |
| 6 | 1.28236575 | 1.30634474 | -6.26921515 |
| 6 | 1.37131059 | -0.39781986 | -4.46225274 |
| 6 | 2.33315510 | 1.91308368 | -4.09669282 |
| 6 | 4.23207526 | 2.35537828 | 2.93840057 |
| 6 | 3.59271389 | 1.44808022 | 0.71364849 |
| 6 | 3.21505305 | 0.08897942 | 2.81929556 |
| 1 | -4.04029095 | 0.83510827 | -0.10103421 |
| 1 | -3.17261811 | 0.06367447 | -1.46229732 |
| 1 | -2.79685963 | 2.88555426 | -0.63062216 |
| 1 | -1.03128626 | 2.89646101 | -0.39667680 |
| 1 | -0.96181286 | 2.16942469 | -6.11208007 |
| 1 | -3.53369485 | 3.11145465 | -2.81283276 |
| 1 | -1.97819354 | 0.30523835 | 1.90908700 |
| 1 | -2.79615963 | 1.87039392 | 1.69880526 |
| 1 | 2.39297499 | 3.81392830 | 3.48546852 |
| 1 | -1.82717137 | 3.65606018 | 2.74209433 |
| 1 | 2.26818067 | 0.95745495 | -6.60812481 |
| 1 | 0.52234290 | 0.72829502 | -6.81496917 |
| 1 | 1.18881358 | 2.36334473 | -6.55703891 |
| 1 | 1.38252089 | -0.60806270 | -3.38738243 |
| 1 | 0.56874356 | -0.98680252 | -4.93041691 |
| 1 | 2.32825210 | -0.73650154 | -4.88759221 |
| 1 | 2.23691618 | 2.98369331 | -4.32876485 |
| 1 | 2.34543403 | 1.79211038 | -3.00782541 |
| 1 | 3.29816710 | 1.56610960 | -4.49642818 |
| 1 | 5.21623083 | 1.87969423 | 2.82158021 |
| 1 | 4.02577170 | 2.42882141 | 4.01604456 |
| 1 | 4.30701900 | 3.37268438 | 2.52777875 |
| 1 | 2.92252736 | 0.80780371 | 0.13070428 |
| 1 | 3.58676306 | 2.45321197 | 0.26789327 |
| 1 | 4.61371937 | 1.04573951 | 0.62910364 |
| 1 | 2.96464442 | 0.12680576 | 3.88976739 |
| 1 | 2.51140975 | -0.58482506 | 2.31841227 |
| 1 | 4.22850844 | -0.33002734 | 2.72431596 |
| 1 | -3.13198297 | -0.68563493 | 0.17449024 |
| 31 | -0.19407690 | 0.02517562 | -0.47357001 |
| 6 | -0.93755527 | -3.35569232 | 2.26030662 |

| | | | |
|---|---|---|---|
| 6 | -1.08576029 | -4.83253365 | 1.83465092 |
| 6 | -0.95775653 | -5.83822900 | 2.98307497 |
| 6 | -2.19784821 | -2.76596302 | 2.86038828 |
| 6 | -1.97835771 | -5.65009870 | 4.11048244 |
| 6 | -1.93712155 | -4.27860264 | 4.77844286 |
| 1 | -0.10205229 | -3.25717813 | 2.97352967 |
| 1 | -2.04867274 | -4.96687918 | 1.31546940 |
| 1 | 0.05725159 | -5.75854178 | 3.40980274 |
| 1 | -3.00388986 | -5.82617359 | 3.74668464 |
| 1 | -0.70033991 | -2.73906003 | 1.38371797 |
| 1 | -0.30440317 | -5.04270456 | 1.08947202 |
| 1 | -1.04382053 | -6.86059052 | 2.58300598 |
| 1 | -1.79304195 | -6.40304786 | 4.89394797 |
| 1 | -2.45870484 | -4.30802916 | 5.74309690 |
| 1 | -0.90151211 | -3.95008665 | 4.96218447 |
| 8 | -2.65764370 | -3.24397727 | 4.05352658 |

Electronic energy = -3523.866455 a.u.
DFT-D3(BJ) dispersion correction = -0.111950 a.u.
Thermal free energy = 0.594903 a.u.
Gibbs free energy = -3523.383501 a.u.
Number of imaginary frequencies = 0.

**RC_vdW_CL isomer/conformer 10**

| Atomic N. | X | Y | Z |
|---|---|---|---|
| 7 | 1.52938654 | 1.06439633 | 1.42193937 |
| 8 | -1.15637748 | 1.99168865 | 0.48023230 |
| 8 | 0.28604884 | -0.33311667 | -0.94836946 |
| 6 | 1.76082538 | 0.65203677 | 2.83295249 |
| 6 | -0.99519563 | 2.94047601 | 1.44219971 |
| 6 | -2.11357796 | 3.67851790 | 1.92534430 |
| 6 | -1.86813027 | 4.63296880 | 2.92383499 |
| 6 | -0.59320570 | 4.88237973 | 3.43940635 |
| 6 | 0.49533400 | 4.17912137 | 2.93386125 |
| 6 | 0.30797899 | 3.22295506 | 1.93033205 |
| 6 | 1.49848139 | 2.56554900 | 1.28463916 |
| 1 | -0.45646122 | 5.62926692 | 4.22139340 |
| 6 | -3.53352062 | 3.45234337 | 1.37026471 |
| 6 | 1.29527606 | 0.27052882 | -1.63310792 |
| 6 | 1.21406966 | 0.40610687 | -3.04880933 |
| 6 | 2.29126843 | 1.02729643 | -3.69761859 |
| 6 | 3.41141152 | 1.50675383 | -3.01360272 |
| 6 | 3.48263775 | 1.35613801 | -1.63293994 |
| 6 | 2.44333926 | 0.72967980 | -0.93478332 |
| 6 | 2.60545909 | 0.46470322 | 0.54181329 |
| 1 | 4.22024788 | 1.99108294 | -3.56064944 |
| 6 | -0.00661718 | -0.10350729 | -3.84099385 |
| 6 | -4.56434682 | 4.39260267 | 2.02264499 |
| 6 | -3.99308746 | 2.00229826 | 1.64726114 |
| 6 | -3.55303159 | 3.72846265 | -0.15210916 |
| 6 | 0.15693215 | 0.11860824 | -5.35665201 |
| 6 | -1.28101248 | 0.65504595 | -3.40126892 |
| 6 | -0.19063416 | -1.62397358 | -3.61925037 |

| | | | |
|---|---|---|---|
| 1 | 2.74381629 | 1.01940157 | 3.16820494 |
| 1 | 0.97579249 | 1.07479885 | 3.46902369 |
| 1 | 2.43469389 | 2.96648817 | 1.70720953 |
| 1 | 1.50162199 | 2.76393684 | 0.20251277 |
| 1 | -2.70374102 | 5.20531113 | 3.32265494 |
| 1 | 1.50478035 | 4.38032629 | 3.29972137 |
| 1 | 2.59297414 | -0.61517599 | 0.75816177 |
| 1 | 3.57537495 | 0.86229859 | 0.88351288 |
| 1 | 2.25669813 | 1.15156482 | -4.77841322 |
| 1 | 4.35721275 | 1.70939540 | -1.08209693 |
| 1 | -5.55219476 | 4.20121934 | 1.57984259 |
| 1 | -4.65040635 | 4.22613149 | 3.10629190 |
| 1 | -4.32453346 | 5.45181223 | 1.85084773 |
| 1 | -3.34217624 | 1.27735603 | 1.14741238 |
| 1 | -3.98773557 | 1.78989772 | 2.72616355 |
| 1 | -5.01953846 | 1.85898950 | 1.27653055 |
| 1 | -3.27982286 | 4.77339215 | -0.35925262 |
| 1 | -2.85706118 | 3.07391699 | -0.68831859 |
| 1 | -4.56632117 | 3.55957540 | -0.54766345 |
| 1 | -0.73148047 | -0.27246278 | -5.87272752 |
| 1 | 1.03360466 | -0.40857400 | -5.76014632 |
| 1 | 0.24347546 | 1.18444451 | -5.61227253 |
| 1 | -1.49153379 | 0.51388901 | -2.33598467 |
| 1 | -1.17383877 | 1.73256243 | -3.59217985 |
| 1 | -2.14703800 | 0.29331812 | -3.97643389 |
| 1 | 0.69499269 | -2.17497873 | -3.96902394 |
| 1 | -0.35299146 | -1.85766744 | -2.56132736 |
| 1 | -1.05888153 | -1.98076510 | -4.19410070 |
| 1 | 1.73644740 | -0.44142556 | 2.89527117 |
| 8 | -0.90689826 | -0.85793964 | 1.83848606 |
| 6 | -2.04008519 | -1.64098650 | 1.47443542 |
| 1 | -1.93098732 | -2.11625819 | 0.48201751 |
| 6 | 1.49821008 | -3.82264748 | -0.00205148 |
| 6 | 0.29953528 | -4.69543907 | 0.42875713 |
| 6 | 0.52850050 | -6.20197128 | 0.27212808 |
| 6 | 2.58918269 | -3.72368219 | 1.04835440 |
| 6 | 1.71951534 | -6.75191920 | 1.06381079 |
| 6 | 3.06002698 | -6.11284467 | 0.71306696 |
| 1 | 1.91845549 | -4.20941229 | -0.94548402 |
| 1 | -0.56390330 | -4.40456987 | -0.18763692 |
| 1 | 0.68236636 | -6.42676346 | -0.79767525 |
| 1 | 1.55686026 | -6.64037776 | 2.14820435 |
| 1 | 1.16526350 | -2.79432219 | -0.18692948 |
| 1 | 0.03019103 | -4.45340859 | 1.46912947 |
| 1 | -0.38481289 | -6.73949754 | 0.57092571 |
| 1 | 1.81069859 | -7.83359094 | 0.87239396 |
| 1 | 3.88683681 | -6.73643782 | 1.07488388 |
| 1 | 3.17745879 | -5.99303448 | -0.37622428 |
| 8 | 2.85819387 | -2.69213930 | 1.63949543 |
| 8 | 3.30166413 | -4.84099175 | 1.37508740 |
| 1 | -2.15381489 | -2.44150075 | 2.22221788 |
| 1 | -2.97324832 | -1.04946233 | 1.46980744 |
| 31 | -0.23489802 | 0.37981027 | 0.69141490 |

Electronic energy = -3523.865494 a.u.
DFT-D3(BJ) dispersion correction = -0.112190 a.u.
Thermal free energy = 0.594516 a.u.
Gibbs free energy = -3523.383168 a.u.
Number of imaginary frequencies = 0.

**RC vdW CL isomer/conformer 11**

| Atomic N. | X | Y | Z |
|---|---|---|---|
| 7 | 0.13801619 | 0.84647861 | 2.08285449 |
| 8 | -1.78993823 | 1.29150149 | -0.17462571 |
| 8 | 0.94245779 | -0.10468999 | -0.65997679 |
| 6 | -0.29868485 | 0.14524494 | 3.31935223 |
| 6 | -2.48219000 | 2.00042621 | 0.75632597 |
| 6 | -3.84414850 | 2.35060208 | 0.52399457 |
| 6 | -4.50949392 | 3.04405184 | 1.54607262 |
| 6 | -3.88815061 | 3.41476785 | 2.74191753 |
| 6 | -2.54051720 | 3.12400935 | 2.92694832 |
| 6 | -1.82703354 | 2.43794551 | 1.93817666 |
| 6 | -0.33513199 | 2.27522940 | 2.06782313 |
| 1 | -4.45181841 | 3.94535000 | 3.50922308 |
| 6 | -4.55073471 | 2.02258132 | -0.80700422 |
| 6 | 1.87054819 | 0.88386568 | -0.55432335 |
| 6 | 2.51281645 | 1.38375129 | -1.72347182 |
| 6 | 3.46917765 | 2.39445386 | -1.54771329 |
| 6 | 3.80069137 | 2.91318212 | -0.29312040 |
| 6 | 3.17457770 | 2.40669304 | 0.84047354 |
| 6 | 2.22226046 | 1.38706657 | 0.72538073 |
| 6 | 1.64606873 | 0.77219170 | 1.97565325 |
| 1 | 4.54530044 | 3.70461958 | -0.20810813 |
| 6 | 2.17512955 | 0.84132805 | -3.12671402 |
| 6 | -5.99797256 | 2.54939750 | -0.83395971 |
| 6 | -4.61310361 | 0.49573447 | -1.03860017 |
| 6 | -3.79043400 | 2.69646911 | -1.97463606 |
| 6 | 3.02429388 | 1.51322710 | -4.22239304 |
| 6 | 0.69047818 | 1.11620152 | -3.46338625 |
| 6 | 2.45926856 | -0.67845341 | -3.19236835 |
| 1 | 0.15006825 | 0.63456693 | 4.19876539 |
| 1 | -1.39036457 | 0.19440439 | 3.40093939 |
| 1 | 0.02216044 | 2.76527831 | 2.98907796 |
| 1 | 0.17281474 | 2.74865170 | 1.21432855 |
| 1 | -5.55595322 | 3.30960556 | 1.40803172 |
| 1 | -2.02227796 | 3.44730720 | 3.83259695 |
| 1 | 1.89133713 | -0.29852675 | 2.04703043 |
| 1 | 2.06972998 | 1.27048092 | 2.86330020 |
| 1 | 3.97366380 | 2.80201375 | -2.42182211 |
| 1 | 3.43154327 | 2.78684438 | 1.83169941 |
| 1 | -6.44697313 | 2.30890923 | -1.80808894 |
| 1 | -6.62364052 | 2.08431914 | -0.05815405 |
| 1 | -6.04331036 | 3.64085787 | -0.70969092 |
| 1 | -3.61076401 | 0.06181815 | -1.11492012 |
| 1 | -5.15162949 | -0.00214277 | -0.21891588 |
| 1 | -5.15218641 | 0.28415985 | -1.97456062 |

| | | | |
|---|---|---|---|
| 1 | -3.77917981 | 3.78896349 | -1.84983510 |
| 1 | -2.75545044 | 2.34217275 | -2.03804460 |
| 1 | -4.29439117 | 2.47022958 | -2.92664869 |
| 1 | 2.75427572 | 1.08253477 | -5.19710835 |
| 1 | 4.10077475 | 1.34550297 | -4.07306986 |
| 1 | 2.84519945 | 2.59657981 | -4.27992563 |
| 1 | 0.01566985 | 0.61707071 | -2.76018143 |
| 1 | 0.47982538 | 2.19504945 | -3.43892663 |
| 1 | 0.46519842 | 0.74945664 | -4.47642999 |
| 1 | 3.52194590 | -0.88137328 | -2.99307741 |
| 1 | 1.85464939 | -1.23054196 | -2.46460768 |
| 1 | 2.22620809 | -1.05653356 | -4.19954099 |
| 1 | 0.03270529 | -0.89862242 | 3.27589908 |
| 8 | -1.20718525 | -1.71055684 | 0.65919350 |
| 6 | -2.56691638 | -1.96660201 | 1.00706149 |
| 1 | -3.03034479 | -1.15858680 | 1.60060496 |
| 8 | 1.96190582 | -2.48315399 | 2.96251878 |
| 1 | -2.60137687 | -2.88820984 | 1.61032450 |
| 1 | -3.18844596 | -2.12023976 | 0.10895638 |
| 31 | -0.58696067 | -0.02575993 | 0.37933209 |
| 6 | 1.27295957 | -3.97370819 | 1.19550715 |
| 6 | 1.94915056 | -3.89722680 | -0.19107726 |
| 6 | 2.88847416 | -5.06691261 | -0.49897434 |
| 6 | 2.16462489 | -3.50586697 | 2.32796565 |
| 6 | 4.03965267 | -5.24011185 | 0.49791831 |
| 6 | 3.59916704 | -5.46192098 | 1.94221759 |
| 1 | 0.93343183 | -5.00577293 | 1.38650044 |
| 1 | 2.48388458 | -2.93863535 | -0.28097452 |
| 1 | 2.29840692 | -6.00000858 | -0.52019210 |
| 1 | 4.71544023 | -4.36972774 | 0.47304491 |
| 1 | 0.39266446 | -3.31769085 | 1.21202057 |
| 1 | 1.15206113 | -3.86820240 | -0.94801500 |
| 1 | 3.30247613 | -4.94213353 | -1.51158552 |
| 1 | 4.64525789 | -6.11232821 | 0.20185030 |
| 1 | 4.42546334 | -5.87334865 | 2.53513298 |
| 1 | 2.75427942 | -6.16708660 | 2.00268667 |
| 8 | 3.26854753 | -4.23847222 | 2.65606762 |

Electronic energy = -3523.867046 a.u.
DFT-D3(BJ) dispersion correction = -0.113070 a.u.
Thermal free energy = 0.597217 a.u.
Gibbs free energy = -3523.382900 a.u.
Number of imaginary frequencies = 0.

**RC vdW CL isomer/conformer 12**

| Atomic N. | X | Y | Z |
|---|---|---|---|
| 8 | -0.29734453 | -1.67417303 | -0.74227809 |
| 6 | 0.85147818 | -2.50705027 | -0.89595705 |
| 1 | 1.44401328 | -2.24744649 | -1.79118891 |
| 8 | -2.92166105 | -1.85181099 | 2.54780618 |
| 1 | 1.52101765 | -2.46922210 | -0.01727162 |
| 1 | 0.50594290 | -3.54592925 | -1.01837237 |
| 7 | -1.82371747 | 0.98472518 | 0.02923728 |

| | | | |
|---|---|---|---|
| 8 | 0.45338029 | 1.15522567 | -1.91224275 |
| 8 | 0.96829141 | 0.46742775 | 1.05307438 |
| 6 | -3.00719233 | 0.14033979 | -0.28929504 |
| 6 | -0.50754426 | 1.62385610 | -2.75271749 |
| 6 | -0.29045510 | 1.63397194 | -4.16093756 |
| 6 | -1.32772094 | 2.12746422 | -4.96613928 |
| 6 | -2.53354730 | 2.60382598 | -4.44426282 |
| 6 | -2.72043049 | 2.61685190 | -3.06604774 |
| 6 | -1.71400066 | 2.14612904 | -2.21614151 |
| 6 | -1.86473904 | 2.28993150 | -0.72518041 |
| 1 | -3.31210465 | 2.97022285 | -5.11331092 |
| 6 | 1.02947907 | 1.13414019 | -4.78035054 |
| 6 | 0.74333644 | 1.60188563 | 1.76645576 |
| 6 | 1.84139210 | 2.32249995 | 2.31936576 |
| 6 | 1.54605652 | 3.47909844 | 3.05504406 |
| 6 | 0.24058073 | 3.93577113 | 3.25585801 |
| 6 | -0.82203309 | 3.21651055 | 2.72049855 |
| 6 | -0.58695858 | 2.04580852 | 1.98933126 |
| 6 | -1.76414274 | 1.22537314 | 1.52423982 |
| 1 | 0.06135798 | 4.84555001 | 3.82872193 |
| 6 | 3.29695329 | 1.85540863 | 2.11893018 |
| 6 | 1.04066631 | 1.28424264 | -6.31324547 |
| 6 | 1.23260603 | -0.36546065 | -4.46341828 |
| 6 | 2.21742733 | 1.95678686 | -4.22691995 |
| 6 | 4.30104169 | 2.77620328 | 2.83801423 |
| 6 | 3.65889739 | 1.86814841 | 0.61495460 |
| 6 | 3.48399960 | 0.43252901 | 2.69767128 |
| 1 | -3.92710777 | 0.67723632 | -0.00787039 |
| 1 | -3.02468949 | -0.07290527 | -1.36345923 |
| 1 | -2.81600968 | 2.79237576 | -0.48389196 |
| 1 | -1.04586929 | 2.89854974 | -0.31369414 |
| 1 | -1.19616600 | 2.13890705 | -6.04659550 |
| 1 | -3.64305976 | 3.01178038 | -2.63468282 |
| 1 | -1.76603182 | 0.22434679 | 1.98164946 |
| 1 | -2.70196841 | 1.72351815 | 1.81939340 |
| 1 | 2.36372959 | 4.05445864 | 3.48529477 |
| 1 | -1.85062331 | 3.54798517 | 2.87922653 |
| 1 | 2.00647378 | 0.92836440 | -6.69942434 |
| 1 | 0.25248997 | 0.68729547 | -6.79468175 |
| 1 | 0.92491799 | 2.33178238 | -6.62670432 |
| 1 | 1.28846601 | -0.53899736 | -3.38366023 |
| 1 | 0.40543664 | -0.96531959 | -4.86977758 |
| 1 | 2.16841200 | -0.72085140 | -4.92113469 |
| 1 | 2.10479961 | 3.01970137 | -4.48569295 |
| 1 | 2.29417337 | 1.87068960 | -3.13755121 |
| 1 | 3.15775346 | 1.59872173 | -4.67325989 |
| 1 | 5.31794592 | 2.39216072 | 2.67376564 |
| 1 | 4.13018035 | 2.80684040 | 3.92386589 |
| 1 | 4.27094848 | 3.80506024 | 2.45123329 |
| 1 | 3.01700297 | 1.19493859 | 0.03713802 |
| 1 | 3.55790953 | 2.88139295 | 0.20018566 |
| 1 | 4.70401941 | 1.54970917 | 0.48114267 |
| 1 | 3.27426027 | 0.42406571 | 3.77736130 |

| | | | |
|---|---|---|---|
| 1 | 2.82169254 | -0.28995492 | 2.20852539 |
| 1 | 4.52564666 | 0.10704646 | 2.55365568 |
| 1 | -2.94587425 | -0.79306440 | 0.28127767 |
| 31 | -0.06034445 | 0.11030876 | -0.45282014 |
| 6 | -1.33865932 | -3.59539082 | 2.03260143 |
| 6 | -1.81818960 | -4.95268592 | 1.47431238 |
| 6 | -1.72903807 | -6.11129489 | 2.47279796 |
| 6 | -2.37565959 | -2.88899975 | 2.88479822 |
| 6 | -2.53276182 | -5.90671977 | 3.76141762 |
| 6 | -2.14140737 | -4.66796248 | 4.56219132 |
| 1 | -0.41192072 | -3.73935851 | 2.61307377 |
| 1 | -2.84948098 | -4.84830669 | 1.10028293 |
| 1 | -0.66903246 | -6.26590455 | 2.73965406 |
| 1 | -3.61270943 | -5.85266608 | 3.54758693 |
| 1 | -1.11016619 | -2.90575775 | 1.20930944 |
| 1 | -1.19883845 | -5.19312937 | 0.59742855 |
| 1 | -2.06090965 | -7.03958770 | 1.98233632 |
| 1 | -2.38944719 | -6.78105198 | 4.41712161 |
| 1 | -2.52331891 | -4.73992875 | 5.58798341 |
| 1 | -1.04696588 | -4.55022248 | 4.61526358 |
| 8 | -2.74433464 | -3.43601105 | 4.07910079 |

Electronic energy = -3523.865850 a.u.
DFT-D3(BJ) dispersion correction = -0.110994 a.u.
Thermal free energy = 0.594212 a.u.
Gibbs free energy = -3523.382632 a.u.
Number of imaginary frequencies = 0.

**RC vdW CL isomer/conformer 13**

| Atomic N. | X | Y | Z |
|---|---|---|---|
| 7 | 0.43001352 | 2.09800752 | 0.95600460 |
| 8 | -2.10729196 | 0.51121774 | 0.82964656 |
| 8 | 0.26327630 | -0.17757285 | -1.02120554 |
| 6 | 1.15966631 | 2.28747248 | 2.23945290 |
| 6 | -2.54997648 | 1.43765658 | 1.72235884 |
| 6 | -3.70774328 | 1.18388163 | 2.51262369 |
| 6 | -4.09771609 | 2.18264352 | 3.41732413 |
| 6 | -3.40952236 | 3.39090619 | 3.55787182 |
| 6 | -2.30098813 | 3.64013477 | 2.75547034 |
| 6 | -1.87518549 | 2.68242930 | 1.82911474 |
| 6 | -0.76625290 | 3.01149533 | 0.86541028 |
| 1 | -3.74819430 | 4.13186516 | 4.28196760 |
| 6 | -4.51052564 | -0.12473639 | 2.37565165 |
| 6 | 0.28365704 | 0.82827005 | -1.93743641 |
| 6 | -0.15814938 | 0.58699588 | -3.26996482 |
| 6 | -0.10250568 | 1.66148600 | -4.16967835 |
| 6 | 0.35980842 | 2.92901339 | -3.80616639 |
| 6 | 0.79977859 | 3.14643974 | -2.50498530 |
| 6 | 0.78071454 | 2.10612273 | -1.56831880 |
| 6 | 1.37766804 | 2.33577895 | -0.20161708 |
| 1 | 0.37610887 | 3.73589077 | -4.53877821 |
| 6 | -0.67825419 | -0.79573778 | -3.71157287 |
| 6 | -5.73824484 | -0.14815159 | 3.30546365 |

| | | | |
|---|---|---|---|
| 6 | -3.62690824 | -1.33712149 | 2.74927213 |
| 6 | -5.02604187 | -0.27613655 | 0.92457003 |
| 6 | -1.06243971 | -0.81651997 | -5.20325349 |
| 6 | -1.94393761 | -1.17311630 | -2.90642867 |
| 6 | 0.41907612 | -1.86760330 | -3.50649085 |
| 1 | 1.53516419 | 3.32145005 | 2.29978993 |
| 1 | 0.47890783 | 2.09703013 | 3.07608902 |
| 1 | -0.41739018 | 4.04558498 | 1.02241681 |
| 1 | -1.12574007 | 2.92753841 | -0.17092873 |
| 1 | -4.97164513 | 2.01412996 | 4.04396876 |
| 1 | -1.76675197 | 4.59033239 | 2.82634605 |
| 1 | 2.23446731 | 1.66778687 | -0.02038399 |
| 1 | 1.74012856 | 3.37436050 | -0.12741561 |
| 1 | -0.43887374 | 1.51126728 | -5.19388491 |
| 1 | 1.17960949 | 4.12585575 | -2.20570574 |
| 1 | -6.28088748 | -1.09248413 | 3.15609990 |
| 1 | -5.45609659 | -0.09372117 | 4.36696903 |
| 1 | -6.43690361 | 0.67261418 | 3.08820964 |
| 1 | -2.76554826 | -1.42162438 | 2.07841574 |
| 1 | -3.25783191 | -1.24832065 | 3.78128888 |
| 1 | -4.21512535 | -2.26458005 | 2.67598919 |
| 1 | -5.69247376 | 0.55841417 | 0.66223927 |
| 1 | -4.20026667 | -0.30105078 | 0.20499273 |
| 1 | -5.60097063 | -1.20999480 | 0.82945294 |
| 1 | -1.41284051 | -1.82441034 | -5.46766105 |
| 1 | -0.20798437 | -0.58137359 | -5.85428674 |
| 1 | -1.87732453 | -0.11436274 | -5.43084053 |
| 1 | -1.73982324 | -1.23190365 | -1.83222076 |
| 1 | -2.73987668 | -0.43156368 | -3.06525552 |
| 1 | -2.32018705 | -2.15176812 | -3.24148728 |
| 1 | 1.30838837 | -1.62969652 | -4.10883054 |
| 1 | 0.71662430 | -1.93826966 | -2.45440876 |
| 1 | 0.04433440 | -2.85041680 | -3.83095379 |
| 1 | 2.00193830 | 1.58823371 | 2.28058967 |
| 8 | 0.50648081 | -0.87254647 | 1.96238816 |
| 6 | 0.31902648 | -2.28332566 | 1.91012172 |
| 1 | -0.71765994 | -2.57970398 | 2.15129561 |
| 6 | 3.61662789 | -1.57713355 | -0.07269141 |
| 6 | 4.46455173 | -1.99598456 | -1.29317155 |
| 6 | 5.49034222 | -3.09372537 | -0.99249579 |
| 6 | 4.32455401 | -0.60393338 | 0.85015713 |
| 6 | 6.50283213 | -2.74041022 | 0.10262947 |
| 6 | 5.88373679 | -2.40218714 | 1.45620868 |
| 1 | 3.31051473 | -2.47334887 | 0.49160701 |
| 1 | 4.96541375 | -1.10831415 | -1.71182288 |
| 1 | 4.94976898 | -4.00770630 | -0.69048294 |
| 1 | 7.17941046 | -3.59707507 | 0.25554913 |
| 1 | 2.70030906 | -1.07111455 | -0.39891466 |
| 1 | 3.77736440 | -2.35667520 | -2.07273456 |
| 1 | 6.03150807 | -3.34819542 | -1.91697880 |
| 1 | 7.13824286 | -1.89445504 | -0.20656854 |
| 1 | 6.64200008 | -2.45769207 | 2.24692184 |
| 1 | 5.07139075 | -3.10021057 | 1.71586685 |

| | | | |
|---|---|---|---|
| 8 | 3.99292314 | 0.56234414 | 0.97773869 |
| 8 | 5.40644104 | -1.03275752 | 1.56256420 |
| 1 | 0.57589931 | -2.71219688 | 0.92341026 |
| 1 | 0.98043480 | -2.74192413 | 2.66226541 |
| 31 | -0.26702416 | 0.20820311 | 0.72223715 |

Electronic energy = -3523.865950 a.u.
DFT-D3(BJ) dispersion correction = -0.112573 a.u.
Thermal free energy = 0.596959 a.u.
Gibbs free energy = -3523.381564 a.u.
Number of imaginary frequencies = 0.

**RC vdW CL isomer/conformer 14**

| Atomic N. | X | Y | Z |
|---|---|---|---|
| 7 | 3.05748895 | 1.21539214 | 1.16916912 |
| 8 | 0.75321041 | 1.20878839 | -0.73427790 |
| 8 | 1.24167145 | -1.17611594 | 1.25499647 |
| 6 | 3.24165872 | 2.41594082 | 2.02154551 |
| 6 | 1.37820064 | 2.30886219 | -1.23260607 |
| 6 | 0.65898882 | 3.22982245 | -2.04748406 |
| 6 | 1.36462929 | 4.33892471 | -2.53670676 |
| 6 | 2.71615427 | 4.56226195 | -2.25921124 |
| 6 | 3.41116693 | 3.64482523 | -1.47875701 |
| 6 | 2.75872481 | 2.51403371 | -0.97498364 |
| 6 | 3.54559983 | 1.46565940 | -0.23552000 |
| 6 | -0.83169239 | 3.02296710 | -2.37994394 |
| 6 | 2.34991062 | -1.83298564 | 0.81875030 |
| 6 | 2.22681511 | -3.10717594 | 0.19371221 |
| 6 | 3.41264079 | -3.73127870 | -0.21986297 |
| 6 | 4.67576315 | -3.15853113 | -0.04230961 |
| 6 | 4.78149293 | -1.92076201 | 0.58132062 |
| 6 | 3.63028594 | -1.25784534 | 1.02432234 |
| 6 | 3.76834516 | 0.03296233 | 1.78810608 |
| 6 | 0.85502232 | -3.77602924 | -0.01703812 |
| 6 | -1.37071936 | 4.12322800 | -3.31368327 |
| 6 | -1.67370106 | 3.06620331 | -1.08307940 |
| 6 | -1.03466373 | 1.66906022 | -3.10112586 |
| 6 | 0.98247365 | -5.15472787 | -0.69229284 |
| 6 | -0.03584676 | -2.90237170 | -0.93224276 |
| 6 | 0.15906972 | -3.99921010 | 1.34731735 |
| 1 | 4.31451202 | 2.64926335 | 2.11724004 |
| 1 | 2.72448420 | 3.26892547 | 1.56937130 |
| 1 | 4.60961210 | 1.75198893 | -0.18523137 |
| 1 | 3.48107396 | 0.49565884 | -0.75009054 |
| 1 | 0.84130660 | 5.06457171 | -3.15656589 |
| 1 | 4.47385890 | 3.78685615 | -1.26928639 |
| 1 | 3.34434167 | -0.06730335 | 2.80031360 |
| 1 | 4.83495859 | 0.29287064 | 1.89265337 |
| 1 | 3.35227523 | -4.70268632 | -0.70713623 |
| 1 | 5.75966102 | -1.46200708 | 0.74228200 |
| 1 | -2.42958907 | 3.92072145 | -3.52872590 |
| 1 | -1.31263033 | 5.12193779 | -2.85706705 |
| 1 | -0.83725523 | 4.14761630 | -4.27490365 |

| | | | |
|---|---|---|---|
| 1 | -1.37568463 | 2.28517886 | -0.37553184 |
| 1 | -1.56959161 | 4.04068169 | -0.58424639 |
| 1 | -2.73762275 | 2.92533231 | -1.32920972 |
| 1 | -0.46978141 | 1.64504041 | -4.04442344 |
| 1 | -0.71027978 | 0.82678523 | -2.48093628 |
| 1 | -2.09983337 | 1.53474232 | -3.34390416 |
| 1 | -0.02169119 | -5.58606980 | -0.80540222 |
| 1 | 1.57920802 | -5.85655855 | -0.09155231 |
| 1 | 1.42834145 | -5.08636723 | -1.69537503 |
| 1 | -0.21033916 | -1.90998738 | -0.50293207 |
| 1 | 0.43130989 | -2.77306186 | -1.91946320 |
| 1 | -1.01093554 | -3.39074890 | -1.07170885 |
| 1 | 0.74756889 | -4.68713297 | 1.97232300 |
| 1 | 0.03520580 | -3.05685735 | 1.89258539 |
| 1 | -0.83456395 | -4.44200761 | 1.18677733 |
| 1 | 2.81850113 | 2.22662204 | 3.01501174 |
| 31 | 1.08487274 | 0.65568196 | 1.00391672 |
| 8 | -0.03092985 | 1.42970674 | 2.21018292 |
| 6 | -0.21258851 | 0.83276723 | 3.49423664 |
| 1 | -0.24217211 | -0.26981394 | 3.45726172 |
| 1 | 0.58549923 | 1.12754491 | 4.20149525 |
| 1 | -1.16967712 | 1.18992010 | 3.90547810 |
| 1 | 3.21808441 | 5.44327985 | -2.65894498 |
| 1 | 5.56677221 | -3.68231760 | -0.38827347 |
| 8 | -3.59970807 | -3.73889783 | 0.33743419 |
| 8 | -5.49833892 | -2.60453086 | 0.47315119 |
| 6 | -4.16491489 | -2.74751220 | 0.75439587 |
| 6 | -3.46287681 | -1.65684048 | 1.54720095 |
| 1 | -3.97702387 | -1.50018944 | 2.51038743 |
| 1 | -2.46598229 | -2.05552927 | 1.76701778 |
| 6 | -3.34507769 | -0.31314893 | 0.79604434 |
| 1 | -3.04592073 | -0.50563547 | -0.24684241 |
| 1 | -2.52303638 | 0.26180251 | 1.24855399 |
| 6 | -4.61764200 | 0.53823493 | 0.83668340 |
| 1 | -4.82585539 | 0.81161927 | 1.88611551 |
| 1 | -4.43524083 | 1.48491875 | 0.30523408 |
| 6 | -5.86117679 | -0.14004179 | 0.25244419 |
| 1 | -5.73205615 | -0.33778380 | -0.82421727 |
| 1 | -6.72027568 | 0.54496769 | 0.34236239 |
| 6 | -6.24791816 | -1.44967844 | 0.93492040 |
| 1 | -6.17235354 | -1.36863808 | 2.03198341 |
| 1 | -7.28357446 | -1.71578917 | 0.68879785 |

Electronic energy = -3523.861333 a.u.
DFT-D3(BJ) dispersion correction = -0.112580 a.u.
Thermal free energy = 0.594869 a.u.
Gibbs free energy = -3523.379044 a.u.
Number of imaginary frequencies = 0.

**RC vdW CL isomer/conformer 15**

| Atomic N. | X | Y | Z |
|---|---|---|---|
| 7 | 0.27841974 | 3.26815424 | 1.05966601 |
| 8 | -0.46506964 | 1.20436903 | -0.96955883 |

| | | | |
|---|---|---|---|
| 8 | 1.22979008 | 0.45823272 | 1.55750819 |
| 6 | -0.80864766 | 4.12040454 | 1.60260974 |
| 6 | -0.89731754 | 2.28961588 | -1.66748443 |
| 6 | -1.82470065 | 2.12789172 | -2.73654690 |
| 6 | -2.22061704 | 3.28608630 | -3.42168283 |
| 6 | -1.74605251 | 4.56012070 | -3.09784612 |
| 6 | -0.82807320 | 4.70154040 | -2.06298879 |
| 6 | -0.38950381 | 3.57732533 | -1.35447105 |
| 6 | 0.69122517 | 3.72408159 | -0.31741908 |
| 6 | -2.37735519 | 0.74303932 | -3.12693370 |
| 6 | 2.45771142 | 1.02001781 | 1.40266836 |
| 6 | 3.58583916 | 0.20212536 | 1.10532768 |
| 6 | 4.82420043 | 0.84511564 | 0.96617571 |
| 6 | 4.98307835 | 2.22653097 | 1.10893554 |
| 6 | 3.87725854 | 3.01074315 | 1.41565689 |
| 6 | 2.61836344 | 2.41927236 | 1.57579358 |
| 6 | 1.45398536 | 3.27123092 | 2.00903261 |
| 6 | 3.45980564 | -1.32640352 | 0.95104205 |
| 6 | -3.32234766 | 0.82196918 | -4.34088816 |
| 6 | -3.18597289 | 0.14725143 | -1.95076795 |
| 6 | -1.21696131 | -0.20598816 | -3.51076011 |
| 6 | 4.81784053 | -1.98885529 | 0.65137383 |
| 6 | 2.51258653 | -1.67301564 | -0.22189919 |
| 6 | 2.92677717 | -1.94440063 | 2.26547966 |
| 1 | -0.44880713 | 5.15422329 | 1.72968566 |
| 1 | -1.65803881 | 4.11484175 | 0.91130014 |
| 1 | 1.01514502 | 4.77624458 | -0.25008192 |
| 1 | 1.56934552 | 3.11673563 | -0.58153513 |
| 1 | -2.93322277 | 3.19653013 | -4.23944785 |
| 1 | -0.42720087 | 5.68500295 | -1.80726706 |
| 1 | 1.05412701 | 2.92061261 | 2.97423034 |
| 1 | 1.78469514 | 4.31492683 | 2.14046495 |
| 1 | 5.70397667 | 0.24901411 | 0.73150312 |
| 1 | 3.98223259 | 4.08997237 | 1.54695427 |
| 1 | -3.67557783 | -0.19030860 | -4.58371944 |
| 1 | -4.20912026 | 1.43982269 | -4.13856214 |
| 1 | -2.81696512 | 1.21847391 | -5.23335699 |
| 1 | -2.57205782 | 0.02551513 | -1.05220489 |
| 1 | -4.03944692 | 0.79338681 | -1.69943607 |
| 1 | -3.58328547 | -0.83900711 | -2.23660422 |
| 1 | -0.66092223 | 0.19169964 | -4.37226793 |
| 1 | -0.51687298 | -0.34553322 | -2.68015012 |
| 1 | -1.62210688 | -1.18863759 | -3.79710401 |
| 1 | 4.67304346 | -3.07488764 | 0.56346905 |
| 1 | 5.54820426 | -1.81794804 | 1.45520251 |
| 1 | 5.25169075 | -1.63625584 | -0.29546284 |
| 1 | 1.50184648 | -1.28495833 | -0.05726273 |
| 1 | 2.89247483 | -1.25719697 | -1.16612208 |
| 1 | 2.45017234 | -2.76613368 | -0.33498676 |
| 1 | 3.62286117 | -1.75096773 | 3.09448096 |
| 1 | 1.94670921 | -1.53490396 | 2.53370919 |
| 1 | 2.83306167 | -3.03530327 | 2.15356539 |
| 1 | -1.13382390 | 3.72396484 | 2.57153726 |

| | | | |
|---|---|---|---|
| 31 | -0.28154205 | 1.29878967 | 0.87285894 |
| 8 | -1.79808279 | 0.86112771 | 1.76568327 |
| 6 | -1.73479674 | 0.47249012 | 3.13870643 |
| 1 | -2.60087679 | -0.17227250 | 3.35402235 |
| 1 | -0.81844441 | -0.08945284 | 3.38798166 |
| 1 | -1.79239647 | 1.34790824 | 3.81225852 |
| 1 | -2.08947045 | 5.43003117 | -3.65745237 |
| 1 | 5.96645735 | 2.67943031 | 0.98440357 |
| 8 | -1.09837929 | -6.78200439 | 2.40530435 |
| 8 | -2.20483866 | -6.13112732 | 0.59726887 |
| 6 | -1.22787962 | -5.94091692 | 1.53938730 |
| 6 | -0.37727585 | -4.68249664 | 1.46769086 |
| 1 | 0.11215450 | -4.60900661 | 0.48224788 |
| 1 | 0.40958201 | -4.82545437 | 2.21745672 |
| 6 | -1.16094080 | -3.38437474 | 1.75561601 |
| 1 | -0.43846584 | -2.61429192 | 2.06376477 |
| 1 | -1.82596705 | -3.54817942 | 2.61922065 |
| 6 | -1.95897139 | -2.85542332 | 0.56016163 |
| 1 | -2.45544026 | -1.91465440 | 0.84281430 |
| 1 | -1.25239360 | -2.59552985 | -0.24743157 |
| 6 | -2.99725969 | -3.83444908 | 0.00351723 |
| 1 | -3.78304315 | -4.04157031 | 0.74837267 |
| 1 | -3.50184504 | -3.36870308 | -0.85885818 |
| 6 | -2.42343814 | -5.16808555 | -0.46809710 |
| 1 | -1.49177701 | -5.02452967 | -1.04000243 |
| 1 | -3.14106837 | -5.68263496 | -1.11944964 |

Electronic energy = -3523.860705 a.u.
DFT-D3(BJ) dispersion correction = -0.112711 a.u.
Thermal free energy = 0.595782 a.u.
Gibbs free energy = -3523.377634 a.u.
Number of imaginary frequencies = 0.

**RC vdW CL isomer/conformer 16**

| Atomic N. | X | Y | Z |
|---|---|---|---|
| 7 | -0.67817635 | 2.88686064 | 1.23642686 |
| 8 | -0.38078728 | 1.06703779 | -1.11660403 |
| 8 | 1.09516753 | 0.48197489 | 1.57067136 |
| 6 | -2.05418405 | 3.25117888 | 1.65669768 |
| 6 | -1.05172465 | 2.06057958 | -1.75762835 |
| 6 | -1.70561315 | 1.79835353 | -2.99607201 |
| 6 | -2.37122289 | 2.86996648 | -3.60919656 |
| 6 | -2.41145490 | 4.15413584 | -3.05893782 |
| 6 | -1.75329161 | 4.40098398 | -1.85905356 |
| 6 | -1.06305438 | 3.37055974 | -1.21115525 |
| 6 | -0.25468586 | 3.67342198 | 0.02176063 |
| 6 | -1.68929298 | 0.39754788 | -3.63860508 |
| 6 | 2.06483921 | 1.41843530 | 1.72629383 |
| 6 | 3.43481578 | 1.05953279 | 1.56733572 |
| 6 | 4.38823343 | 2.07105897 | 1.75054596 |
| 6 | 4.04640823 | 3.38653863 | 2.07775691 |
| 6 | 2.70754768 | 3.71966188 | 2.24650501 |
| 6 | 1.71442838 | 2.74580208 | 2.08642632 |

| | | | |
|---|---|---|---|
| 6 | 0.28099355 | 3.10069914 | 2.38403850 |
| 6 | 3.85668884 | -0.38031984 | 1.21364727 |
| 6 | -2.41765614 | 0.37819072 | -4.99584072 |
| 6 | -2.40748882 | -0.61467018 | -2.71544005 |
| 6 | -0.23210534 | -0.05621495 | -3.89395808 |
| 6 | 5.38774774 | -0.52415663 | 1.12250238 |
| 6 | 3.27601507 | -0.78563846 | -0.16188599 |
| 6 | 3.36657825 | -1.35909847 | 2.30733970 |
| 1 | -2.08814193 | 4.31143156 | 1.95555540 |
| 1 | -2.74690588 | 3.08660598 | 0.82465504 |
| 1 | -0.31928471 | 4.74715425 | 0.26600460 |
| 1 | 0.80523259 | 3.42681084 | -0.13810020 |
| 1 | -2.88634715 | 2.69973309 | -4.55278253 |
| 1 | -1.75237722 | 5.40236062 | -1.42246198 |
| 1 | -0.10673106 | 2.48427421 | 3.21115045 |
| 1 | 0.21563663 | 4.15760610 | 2.69200537 |
| 1 | 5.44264030 | 1.82994528 | 1.62933874 |
| 1 | 2.41975368 | 4.73758553 | 2.51877297 |
| 1 | -2.36450025 | -0.63694088 | -5.41444270 |
| 1 | -3.48153337 | 0.63954726 | -4.90093295 |
| 1 | -1.95456644 | 1.06057553 | -5.72321107 |
| 1 | -1.92908196 | -0.68220519 | -1.73261207 |
| 1 | -3.45831139 | -0.32686426 | -2.56686814 |
| 1 | -2.39316762 | -1.61306968 | -3.17962008 |
| 1 | 0.27401223 | 0.63759122 | -4.58104659 |
| 1 | 0.34445837 | -0.10627107 | -2.96417929 |
| 1 | -0.23129811 | -1.05279636 | -4.36146568 |
| 1 | 5.63399446 | -1.56749544 | 0.87921413 |
| 1 | 5.88313252 | -0.28169192 | 2.07367082 |
| 1 | 5.81831225 | 0.10898231 | 0.33323923 |
| 1 | 2.18102792 | -0.76161285 | -0.16429084 |
| 1 | 3.63730435 | -0.11137223 | -0.95169962 |
| 1 | 3.60162782 | -1.80638628 | -0.41498871 |
| 1 | 3.80629067 | -1.10212798 | 3.28193019 |
| 1 | 2.27578954 | -1.34586101 | 2.40653526 |
| 1 | 3.68333231 | -2.38343891 | 2.05755053 |
| 1 | -2.35614119 | 2.62312629 | 2.50293293 |
| 31 | -0.50860340 | 0.90073192 | 0.72842649 |
| 8 | -1.90429689 | -0.13439583 | 1.24219544 |
| 6 | -1.95765153 | -0.63325466 | 2.58570800 |
| 1 | -1.01229108 | -1.09730440 | 2.90793663 |
| 1 | -2.22167381 | 0.15984222 | 3.31037457 |
| 1 | -2.74357886 | -1.40065417 | 2.63219617 |
| 1 | -2.94800395 | 4.95226067 | -3.57178562 |
| 1 | 4.82496679 | 4.13877340 | 2.20314610 |
| 8 | -1.85890756 | -4.23179451 | 3.69619000 |
| 8 | -0.27459276 | -3.88296689 | 2.18541233 |
| 6 | -1.43691443 | -4.48382934 | 2.58487006 |
| 6 | -2.15107584 | -5.41533598 | 1.61791268 |
| 1 | -1.46356313 | -6.20466628 | 1.27111353 |
| 1 | -2.93667051 | -5.89714414 | 2.21148363 |
| 6 | -2.76799512 | -4.69192584 | 0.40166462 |
| 1 | -3.26643760 | -3.77020202 | 0.74174200 |

| | | | |
|---|---|---|---|
| 1 | -3.55822992 | -5.33640395 | -0.01222921 |
| 6 | -1.76245614 | -4.36979845 | -0.70825080 |
| 1 | -1.36684332 | -5.31795356 | -1.11353618 |
| 1 | -2.28938756 | -3.87508425 | -1.53865937 |
| 6 | -0.58654857 | -3.49314597 | -0.26546805 |
| 1 | -0.94091118 | -2.50140925 | 0.06030944 |
| 1 | 0.07605459 | -3.31849941 | -1.12880199 |
| 6 | 0.26491651 | -4.09140559 | 0.85088891 |
| 1 | 0.45070100 | -5.16638651 | 0.68950795 |
| 1 | 1.23711157 | -3.58566541 | 0.90022885 |

Electronic energy = -3523.861800 a.u.
DFT-D3(BJ) dispersion correction = -0.113059 a.u.
Thermal free energy = 0.597575 a.u.
Gibbs free energy = -3523.377284 a.u.
Number of imaginary frequencies = 0.

**RC vdW CL isomer/conformer 17**

| Atomic N. | X | Y | Z |
|---|---|---|---|
| 7 | -0.17185780 | 2.58678044 | 0.90953524 |
| 8 | -1.51445535 | 0.86317642 | -1.14980703 |
| 8 | 1.18107632 | 0.01121049 | 0.14035302 |
| 6 | -0.94121931 | 3.03405277 | 2.09721465 |
| 6 | -2.34631361 | 1.93376539 | -1.27297638 |
| 6 | -3.60238278 | 1.79041357 | -1.93036014 |
| 6 | -4.42604197 | 2.92465160 | -1.98838290 |
| 6 | -4.05361404 | 4.16293953 | -1.45807178 |
| 6 | -2.79931320 | 4.30334614 | -0.87429137 |
| 6 | -1.93492192 | 3.20565283 | -0.79303367 |
| 6 | -0.52315106 | 3.40358786 | -0.30666557 |
| 6 | -4.02959118 | 0.46246405 | -2.58807719 |
| 6 | 2.11955005 | 0.84825693 | -0.37685486 |
| 6 | 3.04150218 | 0.37403000 | -1.35372409 |
| 6 | 3.99248174 | 1.28681554 | -1.83253144 |
| 6 | 4.06181478 | 2.61208568 | -1.39357145 |
| 6 | 3.16407375 | 3.05840779 | -0.43071477 |
| 6 | 2.20164952 | 2.18552471 | 0.09071575 |
| 6 | 1.30823886 | 2.65483857 | 1.20910411 |
| 6 | 3.00021461 | -1.07803646 | -1.86967924 |
| 6 | -5.40455131 | 0.57770644 | -3.27297148 |
| 6 | -4.13349844 | -0.66632421 | -1.53741177 |
| 6 | -3.00114473 | 0.07065289 | -3.67634293 |
| 6 | 4.11599927 | -1.35632787 | -2.89455353 |
| 6 | 1.65066784 | -1.35694118 | -2.57255113 |
| 6 | 3.19956695 | -2.06445034 | -0.69438577 |
| 1 | -0.63844027 | 4.05544561 | 2.37887282 |
| 1 | -2.01111711 | 3.03013841 | 1.86055932 |
| 1 | -0.34609554 | 4.46710757 | -0.07413920 |
| 1 | 0.19517852 | 3.10387845 | -1.08386090 |
| 1 | -5.39997151 | 2.84389197 | -2.46724248 |
| 1 | -2.46863635 | 5.27349045 | -0.49644100 |
| 1 | 1.45325013 | 2.03425001 | 2.10789961 |
| 1 | 1.55582763 | 3.69642090 | 1.47309357 |

```
   1     4.70703683   0.95732123  -2.58440962
   1     3.21026187   4.08598947  -0.06328971
   1    -5.65063336  -0.38532784  -3.74202084
   1    -6.20675757   0.81050862  -2.55758347
   1    -5.41042417   1.34101982  -4.06416431
   1    -3.16422800  -0.86847592  -1.07000799
   1    -4.85715347  -0.40439404  -0.75165332
   1    -4.48059924  -1.59193128  -2.02069212
   1    -2.95151830   0.84247659  -4.45797255
   1    -1.99879204  -0.06316181  -3.25437595
   1    -3.30603320  -0.87328664  -4.15285271
   1     4.04928846  -2.40466806  -3.21791831
   1     5.11787204  -1.20540480  -2.46731088
   1     4.02099133  -0.72846617  -3.79218346
   1     0.80310370  -1.24146338  -1.88880719
   1     1.50592879  -0.67325394  -3.42133449
   1     1.64072003  -2.38631596  -2.96133098
   1     4.17711082  -1.90471006  -0.21619075
   1     2.41677011  -1.94601653   0.06279504
   1     3.17191005  -3.09894100  -1.06890292
   1    -0.75430399   2.34668506   2.93024849
  31    -0.54285811   0.62569236   0.42059384
   8    -1.26382006  -0.24560389   1.84172051
   6    -2.67436532  -0.41317192   1.99141579
   1    -3.04576006  -1.27186922   1.40752176
   1    -2.88297337  -0.60710129   3.05528975
   1    -3.25564396   0.47620194   1.68909874
   1    -4.73382275   5.01204064  -1.52246325
   1     4.81561283   3.28453226  -1.80262194
   8     1.54663795  -6.47909769   3.78705259
   8     0.29350908  -4.70397165   3.34332077
   6     1.51218193  -5.31867718   3.42873674
   6     2.76157031  -4.51767816   3.09368067
   1     2.68309894  -4.09513515   2.07846640
   1     3.57760594  -5.24955923   3.08588743
   6     3.06642133  -3.38988559   4.10251651
   1     4.13465710  -3.13845889   4.01818681
   1     2.91932911  -3.77137284   5.12603631
   6     2.24302169  -2.11607346   3.88400215
   1     2.54168583  -1.36264102   4.63040898
   1     2.50098190  -1.69294040   2.89799836
   6     0.72461061  -2.31340974   3.94486599
   1     0.40910339  -2.63341386   4.95176753
   1     0.22760196  -1.35182268   3.74047898
   6     0.17622375  -3.31272801   2.93126272
   1     0.63591594  -3.17535434   1.93974393
   1    -0.90585133  -3.17958733   2.81628398
Electronic energy = -3523.861368 a.u.
DFT-D3(BJ) dispersion correction = -0.111775 a.u.
Thermal free energy = 0.595954 a.u.
Gibbs free energy = -3523.377189 a.u.
Number of imaginary frequencies = 0.
```

## Initiation step with axial CL addition

**RC ax CL isomer/conformer 1**

| Atomic N. | X | Y | Z |
|---|---|---|---|
| 7 | 0.71385127 | 2.71587329 | 1.16555394 |
| 8 | -0.87762531 | 1.19297209 | -0.80651902 |
| 8 | 1.52732814 | -0.11492932 | 0.84924279 |
| 6 | -0.16885197 | 3.59713225 | 1.96544405 |
| 6 | -1.12470993 | 2.42314020 | -1.30218007 |
| 6 | -2.23522970 | 2.61186073 | -2.18216560 |
| 6 | -2.45819453 | 3.90454051 | -2.67616760 |
| 6 | -1.64230397 | 4.99166941 | -2.34986046 |
| 6 | -0.54936930 | 4.79057295 | -1.51378017 |
| 6 | -0.27457151 | 3.51878074 | -0.99951846 |
| 6 | 0.97376073 | 3.30439789 | -0.18700882 |
| 6 | -3.14889784 | 1.43805201 | -2.58965146 |
| 6 | 2.75171012 | 0.42311927 | 0.68854485 |
| 6 | 3.78087716 | -0.33499409 | 0.04750434 |
| 6 | 5.04347391 | 0.25984664 | -0.07886484 |
| 6 | 5.32428890 | 1.54702203 | 0.38827062 |
| 6 | 4.31695411 | 2.27496669 | 1.01180733 |
| 6 | 3.04159581 | 1.72342850 | 1.17547129 |
| 6 | 1.99286152 | 2.50179295 | 1.92200535 |
| 6 | 3.51700005 | -1.75576424 | -0.48864429 |
| 6 | -4.25345681 | 1.87983404 | -3.56818462 |
| 6 | -3.84750202 | 0.84260315 | -1.34517253 |
| 6 | -2.31733733 | 0.34119067 | -3.29699717 |
| 6 | 4.77243486 | -2.38072482 | -1.12535959 |
| 6 | 2.42069844 | -1.71744444 | -1.57945242 |
| 6 | 3.08468342 | -2.68190578 | 0.67336145 |
| 1 | 0.31891110 | 4.57438704 | 2.12493989 |
| 1 | -1.11310189 | 3.75409056 | 1.43309094 |
| 1 | 1.50915924 | 4.26272513 | -0.06450291 |
| 1 | 1.65569519 | 2.61017123 | -0.70040309 |
| 1 | -3.30201338 | 4.07777247 | -3.34173509 |
| 1 | 0.11511660 | 5.62038975 | -1.26152847 |
| 1 | 1.70395347 | 1.98227245 | 2.85031265 |
| 1 | 2.40214993 | 3.48836738 | 2.20291720 |
| 1 | 5.84340026 | -0.29485783 | -0.56645275 |
| 1 | 4.51337983 | 3.28153766 | 1.38842216 |
| 1 | -4.86566227 | 1.00589458 | -3.83373425 |
| 1 | -4.92395282 | 2.63107147 | -3.12584161 |
| 1 | -3.83971995 | 2.28967383 | -4.50090234 |
| 1 | -3.11758370 | 0.44172872 | -0.63466152 |
| 1 | -4.45431338 | 1.60664056 | -0.83695206 |
| 1 | -4.51967558 | 0.02546468 | -1.65049763 |
| 1 | -1.84442129 | 0.74128452 | -4.20585907 |
| 1 | -1.53513211 | -0.05255486 | -2.63893739 |
| 1 | -2.97632554 | -0.48860117 | -3.59719084 |
| 1 | 4.52765890 | -3.39254742 | -1.48013414 |
| 1 | 5.59970532 | -2.47271586 | -0.40682815 |

| | | | |
|---|---|---|---|
| 1 | 5.12713069 | -1.80460156 | -1.99196580 |
| 1 | 1.47962577 | -1.30834635 | -1.19774309 |
| 1 | 2.74498882 | -1.09809464 | -2.42821792 |
| 1 | 2.23547125 | -2.73485876 | -1.95921392 |
| 1 | 3.89723478 | -2.78191092 | 1.40811106 |
| 1 | 2.20269882 | -2.28361430 | 1.18670021 |
| 1 | 2.85782100 | -3.68792484 | 0.28432180 |
| 1 | -0.37836980 | 3.11206259 | 2.92412657 |
| 8 | -0.90348244 | -1.29816275 | 0.25438579 |
| 8 | -0.42527286 | -2.37729398 | 2.12400951 |
| 6 | -0.80698461 | -2.37781765 | 0.83926345 |
| 6 | -1.13639168 | -3.67597367 | 0.13658198 |
| 1 | -0.25885152 | -4.34285891 | 0.17668572 |
| 1 | -1.29973795 | -3.40979772 | -0.91365094 |
| 6 | -2.37361666 | -4.40072970 | 0.70958376 |
| 1 | -2.73832922 | -5.09568272 | -0.06102690 |
| 1 | -3.18122418 | -3.66901190 | 0.87130506 |
| 6 | -2.09990609 | -5.18753366 | 1.99536035 |
| 1 | -3.02369533 | -5.69711153 | 2.30949410 |
| 1 | -1.36857082 | -5.98416698 | 1.77424084 |
| 6 | -1.56990700 | -4.34311355 | 3.15920255 |
| 1 | -2.31631070 | -3.59795091 | 3.47870504 |
| 1 | -1.39447742 | -4.99770923 | 4.02812274 |
| 6 | -0.25969469 | -3.61931118 | 2.87199147 |
| 1 | 0.46260786 | -4.26388687 | 2.34727872 |
| 1 | 0.20548320 | -3.27347645 | 3.80212227 |
| 31 | -0.12945679 | 0.77954156 | 0.86481449 |
| 8 | -1.06818734 | 0.76232267 | 2.44341484 |
| 6 | -2.45479179 | 0.48681121 | 2.49964521 |
| 1 | -2.71713948 | -0.50029935 | 2.07324516 |
| 1 | -2.76491448 | 0.48428690 | 3.55810564 |
| 1 | -3.06550752 | 1.24481311 | 1.97279076 |
| 1 | -1.85900928 | 5.98069838 | -2.75400155 |
| 1 | 6.32007130 | 1.97147549 | 0.26101064 |

Electronic energy = -3523.864906 a.u.
DFT-D3(BJ) dispersion correction = -0.116096 a.u.
Thermal free energy = 0.599758 a.u.
Gibbs free energy = -3523.381245 a.u.
Number of imaginary frequencies = 0.

**RC ax CL isomer/conformer 2**

| Atomic N. | X | Y | Z |
|---|---|---|---|
| 7 | -1.71469702 | 1.70605787 | 1.77166578 |
| 8 | -0.69421508 | 1.21104669 | -0.96653440 |
| 8 | 0.45438258 | -0.31749295 | 1.61279666 |
| 6 | -3.19277388 | 1.79973851 | 1.78755863 |
| 6 | -1.23862680 | 2.42490029 | -1.20380945 |
| 6 | -1.49248991 | 2.82408609 | -2.55202750 |
| 6 | -2.06668452 | 4.08689740 | -2.75438198 |
| 6 | -2.37941790 | 4.95498637 | -1.70442042 |
| 6 | -2.09344970 | 4.56983089 | -0.39927272 |
| 6 | -1.51307772 | 3.32326580 | -0.13979272 |

| | | | |
|---|---|---|---|
| 6 | -1.10372500 | 2.97736932 | 1.26652652 |
| 1 | -2.83106468 | 5.92524934 | -1.91146592 |
| 6 | -1.12935025 | 1.91707093 | -3.74544262 |
| 6 | 1.04229685 | 0.49638836 | 2.51867015 |
| 6 | 2.45933195 | 0.46185458 | 2.69816099 |
| 6 | 3.00640798 | 1.29871216 | 3.68109603 |
| 6 | 2.23097761 | 2.15586899 | 4.46668808 |
| 6 | 0.85481182 | 2.19133010 | 4.27312824 |
| 6 | 0.25557332 | 1.36559846 | 3.31572788 |
| 6 | -1.23959431 | 1.38033701 | 3.15942119 |
| 1 | 2.70425318 | 2.79223813 | 5.21442685 |
| 6 | 3.36437618 | -0.44319809 | 1.83953056 |
| 6 | -1.46365872 | 2.57995379 | -5.09506794 |
| 6 | -1.91818779 | 0.58905360 | -3.67655510 |
| 6 | 0.38964209 | 1.62075735 | -3.73859480 |
| 6 | 4.84959156 | -0.31528824 | 2.22730780 |
| 6 | 3.24152226 | -0.05163115 | 0.34808388 |
| 6 | 2.96655223 | -1.92452185 | 2.03292238 |
| 1 | -3.51381839 | 2.59470830 | 2.48242213 |
| 1 | -3.55813544 | 2.04099357 | 0.78340544 |
| 1 | -1.37472024 | 3.80227072 | 1.94918797 |
| 1 | -0.01373809 | 2.84255264 | 1.33001725 |
| 1 | -2.28130322 | 4.41250710 | -3.77064412 |
| 1 | -2.30297561 | 5.24269065 | 0.43561912 |
| 1 | -1.67224444 | 0.39455502 | 3.39622812 |
| 1 | -1.67449689 | 2.11550146 | 3.85915283 |
| 1 | 4.08336898 | 1.29250532 | 3.83888857 |
| 1 | 0.22745760 | 2.85685640 | 4.87055458 |
| 1 | -1.17284973 | 1.89917071 | -5.90791977 |
| 1 | -2.53912006 | 2.78380835 | -5.20315480 |
| 1 | -0.91490876 | 3.52165581 | -5.24045654 |
| 1 | -1.66297218 | 0.02530039 | -2.77371626 |
| 1 | -3.00212535 | 0.77762714 | -3.68453708 |
| 1 | -1.67628680 | -0.03280196 | -4.55228805 |
| 1 | 0.96563985 | 2.55409666 | -3.82122089 |
| 1 | 0.69225370 | 1.10081732 | -2.82338001 |
| 1 | 0.64775894 | 0.98704086 | -4.60106002 |
| 1 | 5.44287229 | -0.98686428 | 1.59011449 |
| 1 | 5.03228015 | -0.60297573 | 3.27295301 |
| 1 | 5.23089346 | 0.70466532 | 2.07500410 |
| 1 | 2.21857666 | -0.16882868 | -0.02352448 |
| 1 | 3.54894880 | 0.99340033 | 0.19758940 |
| 1 | 3.90303423 | -0.68792696 | -0.25984448 |
| 1 | 3.11066304 | -2.23292704 | 3.07902931 |
| 1 | 1.91670256 | -2.08133354 | 1.76432561 |
| 1 | 3.59781374 | -2.56691714 | 1.39905107 |
| 1 | -3.60630126 | 0.83491669 | 2.09953391 |
| 8 | -2.48897513 | -0.95951798 | 0.70437155 |
| 6 | -3.40560224 | -1.08005625 | -0.37474700 |
| 1 | -2.95609715 | -1.56067222 | -1.26466715 |
| 6 | -0.86750320 | -3.59145984 | -0.09256525 |
| 6 | -0.09322562 | -4.50184864 | 0.88471875 |
| 6 | 0.54894816 | -5.73325690 | 0.23968488 |

| | | | |
|---|---|---|---|
| 6 | -0.00079022 | -2.67927795 | -0.93086781 |
| 6 | 1.54977762 | -5.41504546 | -0.87536282 |
| 6 | 0.95834027 | -4.63892329 | -2.04651865 |
| 1 | -1.48310402 | -4.21165185 | -0.76708686 |
| 1 | 0.66600129 | -3.90601563 | 1.41354645 |
| 1 | -0.24758450 | -6.37603006 | -0.17445631 |
| 1 | 2.41212598 | -4.85158346 | -0.48395516 |
| 1 | -1.54281602 | -2.92315865 | 0.45994423 |
| 1 | -0.80654912 | -4.83548680 | 1.65282404 |
| 1 | 1.05062988 | -6.32855658 | 1.01807914 |
| 1 | 1.95227756 | -6.35776604 | -1.28041753 |
| 1 | 1.61459554 | -4.70177854 | -2.92261699 |
| 1 | -0.03231094 | -5.02690739 | -2.33261480 |
| 8 | -0.02485542 | -1.45130315 | -0.86436518 |
| 8 | 0.86722940 | -3.20097183 | -1.82454604 |
| 1 | -4.25175412 | -1.70513566 | -0.04485778 |
| 1 | -3.81577345 | -0.10735869 | -0.70462782 |
| 31 | -1.00395846 | 0.12623768 | 0.52342762 |

Electronic energy = -3523.865353 a.u.
DFT-D3(BJ) dispersion correction = -0.116864 a.u.
Thermal free energy = 0.601177 a.u.
Gibbs free energy = -3523.381040 a.u.
Number of imaginary frequencies = 0.

**RC ax CL isomer/conformer 3**

| Atomic N. | X | Y | Z |
|---|---|---|---|
| 7 | -1.61084623 | 1.83064983 | 1.74278205 |
| 8 | -0.73973483 | 0.88197737 | -0.88061165 |
| 8 | 0.71446769 | 0.01546129 | 1.94355807 |
| 6 | -3.08848090 | 1.85938289 | 1.64049193 |
| 6 | -1.17189471 | 2.09652415 | -1.28437464 |
| 6 | -1.42991936 | 2.30750493 | -2.67573679 |
| 6 | -1.87117605 | 3.57664829 | -3.07276740 |
| 6 | -2.06874064 | 4.62517517 | -2.17086514 |
| 6 | -1.81023713 | 4.41139610 | -0.82219157 |
| 6 | -1.35281295 | 3.16783839 | -0.37110311 |
| 6 | -0.99021032 | 3.02082393 | 1.08252106 |
| 1 | -2.41951809 | 5.59498260 | -2.52362506 |
| 6 | -1.23664113 | 1.18146194 | -3.71095553 |
| 6 | 1.15456652 | 0.93086039 | 2.82889402 |
| 6 | 2.54563693 | 0.97303172 | 3.15380322 |
| 6 | 2.96154317 | 1.92913704 | 4.08993883 |
| 6 | 2.08058702 | 2.82843933 | 4.69861339 |
| 6 | 0.72990631 | 2.77774967 | 4.37418930 |
| 6 | 0.25710393 | 1.83195555 | 3.45664198 |
| 6 | -1.22239666 | 1.75027933 | 3.19077842 |
| 1 | 2.45278583 | 3.55993598 | 5.41601563 |
| 6 | 3.55444287 | 0.00841520 | 2.49910485 |
| 6 | -1.53492020 | 1.65340546 | -5.14693466 |
| 6 | -2.20879058 | 0.01989890 | -3.39932648 |
| 6 | 0.22692169 | 0.67928801 | -3.69375187 |
| 6 | 4.98565305 | 0.23067139 | 3.02368561 |

| | | | |
|---|---|---|---|
| 6 | 3.58371671 | 0.22819766 | 0.96789584 |
| 6 | 3.17262318 | -1.45810341 | 2.81245968 |
| 1 | -3.48992912 | 2.73995560 | 2.17027431 |
| 1 | -3.38440010 | 1.91134537 | 0.58723982 |
| 1 | -1.28657281 | 3.93242814 | 1.63093019 |
| 1 | 0.09815174 | 2.91019179 | 1.20679133 |
| 1 | -2.07443346 | 3.75966367 | -4.12628534 |
| 1 | -1.94857678 | 5.21791285 | -0.09842201 |
| 1 | -1.63953656 | 0.79735874 | 3.55656341 |
| 1 | -1.73323946 | 2.56464556 | 3.73410530 |
| 1 | 4.01589131 | 1.98447667 | 4.35483193 |
| 1 | 0.02230711 | 3.46799858 | 4.83955662 |
| 1 | -1.37659417 | 0.81349062 | -5.83890045 |
| 1 | -2.57453578 | 1.98998477 | -5.26803136 |
| 1 | -0.86544841 | 2.46822723 | -5.45774563 |
| 1 | -2.06094808 | -0.35978534 | -2.38295254 |
| 1 | -3.25237935 | 0.35407850 | -3.49396558 |
| 1 | -2.05315787 | -0.80324211 | -4.11514402 |
| 1 | 0.91367044 | 1.49217515 | -3.97132546 |
| 1 | 0.51849699 | 0.30714311 | -2.70623951 |
| 1 | 0.35185958 | -0.12914781 | -4.43164211 |
| 1 | 5.66010299 | -0.48449008 | 2.53140078 |
| 1 | 5.06048528 | 0.06403204 | 4.10826721 |
| 1 | 5.35698797 | 1.24147458 | 2.80042184 |
| 1 | 2.61071435 | 0.02136488 | 0.51079297 |
| 1 | 3.87376647 | 1.26255038 | 0.73105347 |
| 1 | 4.32692578 | -0.44343817 | 0.51131420 |
| 1 | 3.18153729 | -1.63719972 | 3.89791716 |
| 1 | 2.17859164 | -1.70201463 | 2.42311220 |
| 1 | 3.90568567 | -2.13789996 | 2.35150608 |
| 1 | -3.49005273 | 0.93801897 | 2.07623158 |
| 8 | -2.14588960 | -1.08087503 | 1.19259225 |
| 6 | -1.90422174 | -2.09542502 | 2.15983053 |
| 1 | -1.90596296 | -1.70225399 | 3.19408618 |
| 6 | -1.21721180 | -3.01785210 | -1.25861374 |
| 6 | -1.31377092 | -4.49429310 | -0.81133603 |
| 6 | -0.78260373 | -5.50083094 | -1.83672030 |
| 6 | 0.15811877 | -2.42178729 | -1.07225423 |
| 6 | 0.69209389 | -5.31063583 | -2.20581349 |
| 6 | 1.02537536 | -3.94081451 | -2.78563247 |
| 1 | -1.51383262 | -2.92895225 | -2.31637446 |
| 1 | -0.79461629 | -4.62098299 | 0.15200334 |
| 1 | -1.38999815 | -5.42449239 | -2.75519675 |
| 1 | 1.34284833 | -5.48594824 | -1.33377393 |
| 1 | -1.89479972 | -2.39219304 | -0.66163710 |
| 1 | -2.37453175 | -4.71107212 | -0.61729377 |
| 1 | -0.93118504 | -6.52199177 | -1.45304924 |
| 1 | 0.97613466 | -6.06175972 | -2.96065500 |
| 1 | 2.00182713 | -3.95946298 | -3.28383654 |
| 1 | 0.27279377 | -3.61284722 | -3.51977172 |
| 8 | 0.41727760 | -1.52293800 | -0.27430962 |
| 8 | 1.20327788 | -2.89992918 | -1.78026805 |
| 1 | -2.70893014 | -2.84642663 | 2.08893793 |

| | | | |
|---|---|---|---|
| 1 | -0.93784996 | -2.61219771 | 2.01165550 |
| 31 | -0.78334802 | 0.11167353 | 0.82664435 |

Electronic energy = -3523.864777 a.u.
DFT-D3(BJ) dispersion correction = -0.116309 a.u.
Thermal free energy = 0.600146 a.u.
Gibbs free energy = -3523.380939 a.u.
Number of imaginary frequencies = 0.

**RC ax CL isomer/conformer 4**

| Atomic N. | X | Y | Z |
|---|---|---|---|
| 7 | 0.95888735 | 2.89526436 | -0.00425829 |
| 8 | 0.90004413 | 0.31437237 | -1.44417903 |
| 8 | -0.18470426 | 0.64180807 | 1.54609073 |
| 6 | 0.56979891 | 4.00157438 | -0.90915962 |
| 6 | 1.88303804 | 0.89002529 | -2.16710015 |
| 6 | 2.21421973 | 0.35225928 | -3.44957334 |
| 6 | 3.23965289 | 0.98029461 | -4.16997526 |
| 6 | 3.94076919 | 2.08673809 | -3.68209100 |
| 6 | 3.62865775 | 2.58359317 | -2.42124749 |
| 6 | 2.62012670 | 1.98960016 | -1.65486530 |
| 6 | 2.37448643 | 2.47478075 | -0.25179336 |
| 6 | 1.48727885 | -0.88335097 | -4.01877684 |
| 6 | 0.71081885 | 1.04127065 | 2.47049549 |
| 6 | 1.11726158 | 0.13693498 | 3.50030636 |
| 6 | 2.03178445 | 0.60604469 | 4.45311372 |
| 6 | 2.55434100 | 1.90224371 | 4.42775544 |
| 6 | 2.15361306 | 2.77326579 | 3.42073435 |
| 6 | 1.23280314 | 2.35993843 | 2.45178483 |
| 6 | 0.76225425 | 3.34288447 | 1.41511627 |
| 6 | 0.57530690 | -1.30426077 | 3.56466813 |
| 6 | 2.03900320 | -1.29271310 | -5.39747874 |
| 6 | -0.02049679 | -0.58893347 | -4.19523372 |
| 6 | 1.67423619 | -2.09109091 | -3.06930994 |
| 6 | 1.14272503 | -2.08548924 | 4.76466302 |
| 6 | 0.96890105 | -2.07820006 | 2.28428840 |
| 6 | -0.96385532 | -1.27976735 | 3.71846280 |
| 1 | 1.20561120 | 4.88419126 | -0.72173703 |
| 1 | 0.69750977 | 3.68806191 | -1.95099471 |
| 1 | 3.04611011 | 3.32227449 | -0.02608485 |
| 1 | 2.59242531 | 1.67947803 | 0.47642806 |
| 1 | 3.50640464 | 0.59681775 | -5.15323732 |
| 1 | 4.17791228 | 3.43357161 | -2.00940773 |
| 1 | -0.31817268 | 3.53629349 | 1.51732783 |
| 1 | 1.29010277 | 4.30279253 | 1.55582200 |
| 1 | 2.35945243 | -0.06397147 | 5.24608055 |
| 1 | 2.54686648 | 3.79177223 | 3.38056899 |
| 1 | 1.49661222 | -2.18215807 | -5.74932727 |
| 1 | 1.90252335 | -0.50286597 | -6.15056796 |
| 1 | 3.10677120 | -1.55181544 | -5.35525018 |
| 1 | -0.49535349 | -0.36731803 | -3.23414023 |
| 1 | -0.17449461 | 0.26419804 | -4.87248947 |
| 1 | -0.52141197 | -1.46487562 | -4.63657265 |

| | | | |
|---|---|---|---|
| 1 | 2.74055677 | -2.33881871 | -2.96400701 |
| 1 | 1.26384132 | -1.88312177 | -2.07506120 |
| 1 | 1.16185312 | -2.97237584 | -3.48606061 |
| 1 | 0.71732621 | -3.09963592 | 4.76480511 |
| 1 | 0.88264180 | -1.61685768 | 5.72486131 |
| 1 | 2.23626628 | -2.18689922 | 4.71228935 |
| 1 | 0.56429980 | -1.60543951 | 1.38338805 |
| 1 | 2.06290820 | -2.12941071 | 2.18592148 |
| 1 | 0.58841623 | -3.11047145 | 2.34044972 |
| 1 | -1.25024450 | -0.78874288 | 4.66039956 |
| 1 | -1.43381334 | -0.74051599 | 2.88859123 |
| 1 | -1.35091471 | -2.31130836 | 3.74561962 |
| 1 | -0.48340438 | 4.24569053 | -0.73688816 |
| 8 | -1.38052536 | -0.81861042 | -0.52626381 |
| 8 | -3.21349426 | -0.33483711 | 0.61398679 |
| 6 | -2.51109098 | -1.15918351 | -0.17395548 |
| 6 | -3.08921448 | -2.49079093 | -0.60192705 |
| 1 | -4.02747966 | -2.32054595 | -1.15648087 |
| 1 | -2.36373527 | -2.91456488 | -1.30530760 |
| 6 | -3.34439913 | -3.46691736 | 0.56686867 |
| 1 | -2.47609158 | -3.45885579 | 1.24422507 |
| 1 | -3.39716211 | -4.48160737 | 0.14552420 |
| 6 | -4.63450809 | -3.18834128 | 1.34509241 |
| 1 | -5.49317834 | -3.30883313 | 0.66168781 |
| 1 | -4.75503392 | -3.95362436 | 2.12707608 |
| 6 | -4.70709345 | -1.79897246 | 1.98770859 |
| 1 | -3.93446970 | -1.68063315 | 2.76352822 |
| 1 | -5.67813541 | -1.68920560 | 2.49731516 |
| 6 | -4.58624108 | -0.63922034 | 1.00680183 |
| 1 | -5.19343054 | -0.80067975 | 0.10204774 |
| 1 | -4.91030825 | 0.29846292 | 1.47252921 |
| 31 | -0.27572998 | 1.16762877 | -0.25462844 |
| 8 | -1.77319336 | 2.10200030 | -0.77628202 |
| 6 | -2.35912875 | 1.92302460 | -2.05124461 |
| 1 | -2.72951740 | 0.89198420 | -2.21714956 |
| 1 | -3.22404489 | 2.60242266 | -2.13503707 |
| 1 | -1.66812445 | 2.15614932 | -2.88431237 |
| 1 | 4.72689738 | 2.54565979 | -4.28183125 |
| 1 | 3.26887891 | 2.22046421 | 5.18686444 |

Electronic energy = -3523.864582 a.u.
DFT-D3(BJ) dispersion correction = -0.116366 a.u.
Thermal free energy = 0.600507 a.u.
Gibbs free energy = -3523.380441 a.u.
Number of imaginary frequencies = 0.

**RC ax CL isomer/conformer 5**

| Atomic N. | X | Y | Z |
|---|---|---|---|
| 7 | 0.17102027 | 2.81166754 | 1.00705494 |
| 8 | -0.72399791 | 1.01202990 | -1.13239726 |
| 8 | 1.39254646 | 0.10677764 | 1.16850225 |
| 6 | -0.99129663 | 3.56629713 | 1.53088563 |
| 6 | -1.05525606 | 2.17342150 | -1.73273870 |

| | | | |
|---|---|---|---|
| 6 | -2.01493680 | 2.16446228 | -2.79068466 |
| 6 | -2.30732125 | 3.38841198 | -3.40824651 |
| 6 | -1.70495957 | 4.59150819 | -3.02821907 |
| 6 | -0.76995075 | 4.58657572 | -1.99849079 |
| 6 | -0.43276661 | 3.39053512 | -1.35616770 |
| 6 | 0.63505269 | 3.38638569 | -0.29733190 |
| 6 | -2.70596147 | 0.86057411 | -3.23783037 |
| 6 | 2.54554714 | 0.80505585 | 1.21869714 |
| 6 | 3.78055046 | 0.15227557 | 0.91737341 |
| 6 | 4.95328186 | 0.91555828 | 0.99831883 |
| 6 | 4.95482168 | 2.26736129 | 1.35424703 |
| 6 | 3.74817545 | 2.88968641 | 1.65387576 |
| 6 | 2.54879667 | 2.17059111 | 1.60193264 |
| 6 | 1.27041970 | 2.84063928 | 2.02698496 |
| 6 | 3.82800457 | -1.33446340 | 0.51291363 |
| 6 | -3.67931448 | 1.09673065 | -4.40804189 |
| 6 | -3.52652518 | 0.26815357 | -2.06748210 |
| 6 | -1.65194224 | -0.16697590 | -3.71537574 |
| 6 | 5.26933522 | -1.82458375 | 0.27895731 |
| 6 | 3.04903685 | -1.54678106 | -0.80644494 |
| 6 | 3.22415318 | -2.21236509 | 1.63581190 |
| 1 | -0.71126135 | 4.61927808 | 1.70736290 |
| 1 | -1.81291952 | 3.52950913 | 0.80877444 |
| 1 | 0.99757381 | 4.41572636 | -0.12577073 |
| 1 | 1.49807724 | 2.78078627 | -0.61334664 |
| 1 | -3.03683249 | 3.41222700 | -4.21594976 |
| 1 | -0.28092045 | 5.51338640 | -1.68924528 |
| 1 | 0.85883464 | 2.35322400 | 2.92601921 |
| 1 | 1.47929816 | 3.89374883 | 2.28691693 |
| 1 | 5.90741723 | 0.44408420 | 0.76953249 |
| 1 | 3.72529730 | 3.94275100 | 1.94394672 |
| 1 | -4.13897048 | 0.13819215 | -4.68925261 |
| 1 | -4.49244999 | 1.78657971 | -4.13904350 |
| 1 | -3.16791276 | 1.49222470 | -5.29766942 |
| 1 | -2.89042309 | 0.03816671 | -1.20636086 |
| 1 | -4.30716028 | 0.97303751 | -1.74575279 |
| 1 | -4.02301020 | -0.65939364 | -2.39385091 |
| 1 | -1.08200777 | 0.23199191 | -4.56740635 |
| 1 | -0.95111835 | -0.42236980 | -2.91340297 |
| 1 | -2.15722419 | -1.08674173 | -4.05003503 |
| 1 | 5.24422371 | -2.88973149 | 0.00690435 |
| 1 | 5.89132903 | -1.72740593 | 1.18065940 |
| 1 | 5.76232614 | -1.28640721 | -0.54339001 |
| 1 | 2.00021408 | -1.24732555 | -0.71109247 |
| 1 | 3.50269398 | -0.96257646 | -1.62018086 |
| 1 | 3.08610379 | -2.60919443 | -1.09539931 |
| 1 | 3.80771762 | -2.10737288 | 2.56243636 |
| 1 | 2.18577045 | -1.93318222 | 1.84468825 |
| 1 | 3.25779987 | -3.27206115 | 1.33625655 |
| 1 | -1.32668892 | 3.10986026 | 2.46804177 |
| 8 | -0.60840215 | -1.42923328 | -0.07803586 |
| 8 | -0.60243080 | -2.54280131 | 1.83503484 |
| 6 | -0.76590707 | -2.50067383 | 0.50081258 |

| | | | |
|---|---|---|---|
| 6 | -1.15795143 | -3.74927535 | -0.25858237 |
| 1 | -0.43560418 | -4.55607540 | -0.05154949 |
| 1 | -1.07116172 | -3.48833926 | -1.31918582 |
| 6 | -2.59030230 | -4.23138725 | 0.06070390 |
| 1 | -2.91891663 | -4.87572471 | -0.76794176 |
| 1 | -3.27032842 | -3.36489649 | 0.06718458 |
| 6 | -2.70700684 | -5.01411165 | 1.37255840 |
| 1 | -3.75013444 | -5.33905840 | 1.50650228 |
| 1 | -2.10537603 | -5.93603821 | 1.29064649 |
| 6 | -2.25695539 | -4.24299662 | 2.61842540 |
| 1 | -2.89937390 | -3.36457272 | 2.79145714 |
| 1 | -2.37041345 | -4.89085660 | 3.50258762 |
| 6 | -0.80557618 | -3.77686155 | 2.58577804 |
| 1 | -0.13098652 | -4.55560168 | 2.19638845 |
| 1 | -0.46358980 | -3.50298775 | 3.59073970 |
| 31 | -0.30249069 | 0.74757260 | 0.66742189 |
| 8 | -1.70012387 | 0.53837485 | 1.84170429 |
| 6 | -1.44917314 | 0.28060695 | 3.21242775 |
| 1 | -2.32560324 | -0.23370305 | 3.64357205 |
| 1 | -0.56436781 | -0.35826041 | 3.38489205 |
| 1 | -1.30430776 | 1.21417498 | 3.79581015 |
| 1 | -1.96784697 | 5.51987766 | -3.53549754 |
| 1 | 5.89204032 | 2.82230712 | 1.39565153 |

Electronic energy = -3523.862911 a.u.
DFT-D3(BJ) dispersion correction = -0.115769 a.u.
Thermal free energy = 0.599020 a.u.
Gibbs free energy = -3523.379660 a.u.
Number of imaginary frequencies = 0.

**RC ax CL isomer/conformer 6**

| Atomic N. | X | Y | Z |
|---|---|---|---|
| 7 | 1.07206536 | 2.81965707 | -0.11874757 |
| 8 | 0.59532661 | 0.18568562 | -1.29367070 |
| 8 | 0.15065544 | 0.87037933 | 1.90062153 |
| 6 | 0.59538805 | 3.76258495 | -1.15714607 |
| 6 | 1.59583989 | 0.55825259 | -2.12141238 |
| 6 | 1.74720825 | -0.12727365 | -3.36746792 |
| 6 | 2.80395283 | 0.26103693 | -4.20149413 |
| 6 | 3.69413017 | 1.28502882 | -3.86804882 |
| 6 | 3.53392844 | 1.94966076 | -2.65817421 |
| 6 | 2.50573293 | 1.59194270 | -1.77829039 |
| 6 | 2.43070295 | 2.28188171 | -0.44279542 |
| 6 | 0.77657719 | -1.24625314 | -3.79434106 |
| 6 | 1.12440101 | 1.42274405 | 2.65132746 |
| 6 | 1.62527990 | 0.70624477 | 3.78191535 |
| 6 | 2.62528384 | 1.32281039 | 4.54582235 |
| 6 | 3.14356198 | 2.58513823 | 4.24090809 |
| 6 | 2.64918257 | 3.27160174 | 3.13806528 |
| 6 | 1.63845545 | 2.70948307 | 2.34920952 |
| 6 | 1.06877577 | 3.51431194 | 1.21200399 |
| 6 | 1.09386315 | -0.69374112 | 4.14850754 |
| 6 | 1.15727660 | -1.85500969 | -5.15742959 |

| | | | |
|---|---|---|---|
| 6 | -0.65017111 | -0.66590393 | -3.93544532 |
| 6 | 0.78705308 | -2.39800263 | -2.76104943 |
| 6 | 1.76974715 | -1.25165158 | 5.41536959 |
| 6 | 1.37719929 | -1.68764097 | 2.99720035 |
| 6 | -0.42675409 | -0.63254545 | 4.42871891 |
| 1 | 1.25863312 | 4.64283411 | -1.20648378 |
| 1 | 0.58990129 | 3.26482131 | -2.13266773 |
| 1 | 3.16204201 | 3.10877481 | -0.41229971 |
| 1 | 2.69171314 | 1.59121085 | 0.37393192 |
| 1 | 2.93982098 | -0.24801315 | -5.15387107 |
| 1 | 4.21804888 | 2.75244998 | -2.37362338 |
| 1 | 0.01675867 | 3.78253371 | 1.40464203 |
| 1 | 1.63626358 | 4.45654623 | 1.11433439 |
| 1 | 3.02603101 | 0.79903157 | 5.41180594 |
| 1 | 3.03668231 | 4.26049472 | 2.88188457 |
| 1 | 0.43805373 | -2.64843249 | -5.40733242 |
| 1 | 1.12506689 | -1.11313426 | -5.96840910 |
| 1 | 2.15829240 | -2.30957304 | -5.14114685 |
| 1 | -1.00501076 | -0.24348092 | -2.98889771 |
| 1 | -0.67109707 | 0.12600069 | -4.69848618 |
| 1 | -1.34654032 | -1.45743653 | -4.25421249 |
| 1 | 1.79032680 | -2.84322712 | -2.69216790 |
| 1 | 0.49478782 | -2.05023337 | -1.76471239 |
| 1 | 0.09102222 | -3.18984962 | -3.08045577 |
| 1 | 1.35005612 | -2.24384377 | 5.63474280 |
| 1 | 1.59284481 | -0.61520533 | 6.29476664 |
| 1 | 2.85507325 | -1.37391458 | 5.28679202 |
| 1 | 0.86927423 | -1.39259844 | 2.07349731 |
| 1 | 2.45764716 | -1.75533830 | 2.80201202 |
| 1 | 1.02152127 | -2.69089794 | 3.27800682 |
| 1 | -0.63833341 | 0.04140095 | 5.27220395 |
| 1 | -0.97884683 | -0.28206062 | 3.55040824 |
| 1 | -0.79436401 | -1.63522381 | 4.69593223 |
| 1 | -0.42662899 | 4.07157147 | -0.91178468 |
| 8 | -1.27629488 | -0.89777966 | 0.42340431 |
| 8 | -2.59170857 | -2.58265213 | 0.88999263 |
| 6 | -2.37928366 | -1.40970948 | 0.25193717 |
| 6 | -3.45730720 | -0.82266510 | -0.63014900 |
| 1 | -4.36474012 | -0.64893343 | -0.02691693 |
| 1 | -3.07559763 | 0.15843867 | -0.94321487 |
| 6 | -3.80628067 | -1.69677832 | -1.85508057 |
| 1 | -2.87712887 | -2.06590387 | -2.31521677 |
| 1 | -4.27775355 | -1.04241875 | -2.60300401 |
| 6 | -4.74849151 | -2.86583410 | -1.55423486 |
| 1 | -5.70794075 | -2.46557598 | -1.18268638 |
| 1 | -4.97826343 | -3.39520454 | -2.49180534 |
| 6 | -4.20312868 | -3.86660337 | -0.53102208 |
| 1 | -3.29750801 | -4.36567615 | -0.91213761 |
| 1 | -4.94949285 | -4.66047848 | -0.36531500 |
| 6 | -3.88111630 | -3.25995654 | 0.82940968 |
| 1 | -4.67152379 | -2.56963315 | 1.16448950 |
| 1 | -3.77234296 | -4.04571084 | 1.58640775 |
| 31 | -0.21361421 | 1.15050532 | 0.08570558 |

| | | | |
|---|---|---|---|
| 8 | -1.83100746 | 1.96137749 | -0.28202192 |
| 6 | -2.68735473 | 2.29108925 | 0.80399907 |
| 1 | -2.78107970 | 1.47734521 | 1.54724626 |
| 1 | -2.34826721 | 3.19102601 | 1.35204505 |
| 1 | -3.69276349 | 2.50811185 | 0.40545958 |
| 1 | 4.49891001 | 1.55766494 | -4.55071786 |
| 1 | 3.92737724 | 3.02032552 | 4.86085994 |

Electronic energy = -3523.863442 a.u.
DFT-D3(BJ) dispersion correction = -0.115982 a.u.
Thermal free energy = 0.601753 a.u.
Gibbs free energy = -3523.377670 a.u.
Number of imaginary frequencies = 0.

**TS1 ax CL isomer/conformer 1**

| Atomic N. | X | Y | Z |
|---|---|---|---|
| 7 | 0.54889922 | 2.55311100 | -0.06713645 |
| 8 | 1.03995841 | -0.10201124 | -1.18472009 |
| 8 | 0.13476536 | 0.33296012 | 1.87655333 |
| 6 | -0.19721745 | 3.43280073 | -0.99767946 |
| 6 | 1.71448868 | 0.57786125 | -2.13247389 |
| 6 | 1.99779796 | -0.03579867 | -3.39055190 |
| 6 | 2.69906155 | 0.72929983 | -4.33359086 |
| 6 | 3.12666142 | 2.03788462 | -4.08663705 |
| 6 | 2.86634997 | 2.61477957 | -2.84786721 |
| 6 | 2.17750482 | 1.89044316 | -1.86980462 |
| 6 | 1.98895672 | 2.45726200 | -0.49482717 |
| 6 | 1.56573304 | -1.48251541 | -3.69862601 |
| 6 | 1.04997907 | 1.00744162 | 2.60342154 |
| 6 | 1.75303853 | 0.33788197 | 3.64978912 |
| 6 | 2.67896785 | 1.08933114 | 4.38629675 |
| 6 | 2.93256295 | 2.44103845 | 4.13289710 |
| 6 | 2.23679913 | 3.08316560 | 3.11499710 |
| 6 | 1.29050901 | 2.38317869 | 2.35701999 |
| 6 | 0.48099106 | 3.12360910 | 1.32561500 |
| 6 | 1.50702515 | -1.15230122 | 3.95828693 |
| 6 | 2.00850069 | -1.92874535 | -5.10461969 |
| 6 | 0.02511398 | -1.60780368 | -3.63402224 |
| 6 | 2.20984636 | -2.45263195 | -2.67953776 |
| 6 | 2.36280608 | -1.64151080 | 5.14196838 |
| 6 | 1.87796092 | -2.01903725 | 2.73139299 |
| 6 | 0.02360102 | -1.38201646 | 4.33492861 |
| 1 | 0.23721910 | 4.44686925 | -0.98967927 |
| 1 | -0.13307214 | 3.02916560 | -2.01391142 |
| 1 | 2.43275181 | 3.46640473 | -0.43311809 |
| 1 | 2.48794294 | 1.82825569 | 0.25658100 |
| 1 | 2.92323558 | 0.29199075 | -5.30501472 |
| 1 | 3.21134156 | 3.62658045 | -2.62175012 |
| 1 | -0.58328401 | 3.13373483 | 1.60263586 |
| 1 | 0.82167987 | 4.17286529 | 1.27994352 |
| 1 | 3.23362973 | 0.60462268 | 5.18774729 |
| 1 | 2.41407042 | 4.14046770 | 2.90422441 |
| 1 | 1.68642336 | -2.96733292 | -5.26683453 |

| | | | |
|---|---|---|---|
| 1 | 1.55596226 | -1.31456047 | -5.89701488 |
| 1 | 3.10133213 | -1.89925588 | -5.22334209 |
| 1 | -0.35059672 | -1.38747820 | -2.62880542 |
| 1 | -0.44888498 | -0.92435239 | -4.35509118 |
| 1 | -0.27355658 | -2.63469458 | -3.89642424 |
| 1 | 3.30701238 | -2.40212414 | -2.73787941 |
| 1 | 1.90158132 | -2.21782247 | -1.65499012 |
| 1 | 1.90605193 | -3.48604576 | -2.90670033 |
| 1 | 2.14408499 | -2.70318821 | 5.32485058 |
| 1 | 2.13986348 | -1.09319917 | 6.06895491 |
| 1 | 3.44016160 | -1.55718669 | 4.93793893 |
| 1 | 1.25971055 | -1.77549700 | 1.86121867 |
| 1 | 2.93473557 | -1.87787613 | 2.46163816 |
| 1 | 1.72901793 | -3.08292740 | 2.97095726 |
| 1 | -0.24450671 | -0.79568216 | 5.22645001 |
| 1 | -0.64614608 | -1.10324145 | 3.51438556 |
| 1 | -0.13749142 | -2.44535055 | 4.56961352 |
| 1 | -1.24894156 | 3.48349540 | -0.69902290 |
| 8 | -1.34804713 | -1.00618021 | -0.27845292 |
| 8 | -3.18944669 | -0.93342660 | 0.98213619 |
| 6 | -2.57880777 | -0.58484469 | -0.19690528 |
| 6 | -3.41463320 | -0.59014863 | -1.47065776 |
| 1 | -4.25961470 | 0.11035716 | -1.38836341 |
| 1 | -2.75218061 | -0.20707558 | -2.25595625 |
| 6 | -3.92060705 | -1.99884756 | -1.83765811 |
| 1 | -3.11973015 | -2.73124992 | -1.65050933 |
| 1 | -4.11454989 | -2.02325612 | -2.92047984 |
| 6 | -5.20565997 | -2.39899271 | -1.10184995 |
| 1 | -6.00656942 | -1.69331634 | -1.38490396 |
| 1 | -5.53279276 | -3.38994882 | -1.45353475 |
| 6 | -5.09192965 | -2.42395815 | 0.42703188 |
| 1 | -4.40138655 | -3.21723685 | 0.75497693 |
| 1 | -6.07735035 | -2.66737776 | 0.85765115 |
| 6 | -4.62820911 | -1.11014977 | 1.05221252 |
| 1 | -5.15299715 | -0.24771382 | 0.61043272 |
| 1 | -4.82898083 | -1.10923654 | 2.13145158 |
| 31 | -0.21740276 | 0.55343850 | 0.05698148 |
| 8 | -2.18856501 | 1.16591082 | -0.05413136 |
| 6 | -2.90194375 | 1.87049305 | 0.96076779 |
| 1 | -2.71239286 | 1.45696880 | 1.96309109 |
| 1 | -2.61093113 | 2.93217271 | 0.94858533 |
| 1 | -3.98163032 | 1.82679174 | 0.74655012 |
| 1 | 3.66568333 | 2.59152846 | -4.85548351 |
| 1 | 3.66868571 | 2.98098519 | 4.72838429 |

Electronic energy = -3523.844562 a.u.
DFT-D3(BJ) dispersion correction = -0.116911 a.u.
Thermal free energy = 0.603882 a.u.
Gibbs free energy = -3523.357591 a.u.
Number of imaginary frequencies = 1.

**TS1 ax CL isomer/conformer 2**

Atomic N. X Y Z

| | | | |
|---|---|---|---|
| 8 | 1.79209905 | 0.39202416 | 1.52633449 |
| 6 | 1.49657302 | 0.22490930 | 2.91496080 |
| 1 | 0.48018034 | 0.57482775 | 3.14337472 |
| 6 | 3.87724382 | -0.48517957 | 0.32086305 |
| 6 | 4.74776206 | -1.52219015 | -0.41488599 |
| 6 | 5.44603627 | -2.50818885 | 0.53021396 |
| 6 | 2.47938690 | -1.01176389 | 0.63443721 |
| 6 | 4.50008185 | -3.35765344 | 1.38720808 |
| 6 | 3.51012376 | -2.55852694 | 2.23071488 |
| 1 | 4.38357234 | -0.14063477 | 1.23465427 |
| 1 | 4.13017124 | -2.05807553 | -1.15271532 |
| 1 | 6.11446847 | -1.93848512 | 1.19951432 |
| 1 | 3.92192599 | -4.05254796 | 0.75744923 |
| 1 | 3.72034956 | 0.39571997 | -0.31392677 |
| 1 | 5.51666839 | -0.98337350 | -0.98876422 |
| 1 | 6.09613246 | -3.17698198 | -0.05518205 |
| 1 | 5.09649012 | -3.97982726 | 2.07486561 |
| 1 | 3.05493357 | -3.20176889 | 2.99520354 |
| 1 | 3.99835098 | -1.71489238 | 2.74451732 |
| 8 | 1.60502572 | -1.01790062 | -0.33359192 |
| 8 | 2.36812904 | -2.09757468 | 1.45961279 |
| 1 | 1.53715292 | -0.84252186 | 3.17978688 |
| 1 | 2.22008866 | 0.78769060 | 3.52781836 |
| 7 | 0.00107956 | 2.57557558 | 0.42112254 |
| 8 | -1.20120179 | -0.19654067 | 0.61844758 |
| 8 | 0.41305387 | 0.78614633 | -1.86977988 |
| 6 | 0.67586272 | 3.16089762 | 1.60274028 |
| 6 | -1.91829954 | 0.36392264 | 1.61730631 |
| 6 | -2.61122548 | -0.46462161 | 2.55112094 |
| 6 | -3.29794511 | 0.17710985 | 3.59192187 |
| 6 | -3.34695426 | 1.56805268 | 3.72695100 |
| 6 | -2.72167021 | 2.36480905 | 2.77340562 |
| 6 | -2.02511442 | 1.77720967 | 1.71129047 |
| 6 | -1.49296621 | 2.63580226 | 0.59687343 |
| 1 | -3.88982195 | 2.01839337 | 4.55799016 |
| 6 | -2.63153033 | -1.99986318 | 2.40990030 |
| 6 | -0.25609676 | 1.72899202 | -2.55694208 |
| 6 | -0.86993305 | 1.41379630 | -3.80675641 |
| 6 | -1.52714027 | 2.45503029 | -4.47667957 |
| 6 | -1.59958179 | 3.75870639 | -3.97346424 |
| 6 | -0.99087430 | 4.05379244 | -2.75844143 |
| 6 | -0.31266270 | 3.05146862 | -2.05612241 |
| 6 | 0.41816987 | 3.38054029 | -0.78820527 |
| 1 | -2.12872038 | 4.53121733 | -4.53146331 |
| 6 | -0.81435379 | -0.00943580 | -4.39406425 |
| 6 | -3.50031787 | -2.66466621 | 3.49440650 |
| 6 | -1.20493329 | -2.58114335 | 2.53995622 |
| 6 | -3.23011829 | -2.38898533 | 1.03662803 |
| 6 | -1.51770917 | -0.09431998 | -5.76160499 |
| 6 | -1.52496755 | -1.00363399 | -3.44528567 |
| 6 | 0.65664953 | -0.44318106 | -4.60089853 |
| 1 | 0.43575054 | 4.23554443 | 1.67165026 |
| 1 | 0.32954328 | 2.66619898 | 2.51427822 |

| | | | |
|---|---|---|---|
| 1 | -1.78150625 | 3.68882799 | 0.76028705 |
| 1 | -1.92284376 | 2.31950996 | -0.36428408 |
| 1 | -3.82090693 | -0.42837743 | 4.33011661 |
| 1 | -2.78924900 | 3.45388358 | 2.82977244 |
| 1 | 1.50049541 | 3.20379815 | -0.90176233 |
| 1 | 0.27508369 | 4.44816180 | -0.54812551 |
| 1 | -2.01016146 | 2.24639700 | -5.42978940 |
| 1 | -1.02767256 | 5.06618269 | -2.34909105 |
| 1 | -3.49950581 | -3.75224057 | 3.33400168 |
| 1 | -3.11372609 | -2.48055481 | 4.50764128 |
| 1 | -4.54529458 | -2.32485741 | 3.45364688 |
| 1 | -0.53908317 | -2.20548548 | 1.75642196 |
| 1 | -0.77442151 | -2.33356230 | 3.52212005 |
| 1 | -1.24365954 | -3.67822657 | 2.45745054 |
| 1 | -4.26189325 | -2.01921195 | 0.94546471 |
| 1 | -2.63747432 | -1.98158632 | 0.21037695 |
| 1 | -3.25400745 | -3.48512708 | 0.93983686 |
| 1 | -1.44060300 | -1.12395873 | -6.13904286 |
| 1 | -1.05357577 | 0.56754100 | -6.50737560 |
| 1 | -2.58720948 | 0.15299645 | -5.69422600 |
| 1 | -1.03840572 | -1.04623104 | -2.46531233 |
| 1 | -2.57738271 | -0.71882710 | -3.30112334 |
| 1 | -1.50394062 | -2.01342333 | -3.88285014 |
| 1 | 1.16664406 | 0.23760243 | -5.29862736 |
| 1 | 1.20604659 | -0.45414774 | -3.65334742 |
| 1 | 0.68644690 | -1.45482819 | -5.03391559 |
| 1 | 1.75974410 | 3.03182525 | 1.51020729 |
| 31 | 0.41914483 | 0.50002041 | -0.00721623 |

Electronic energy = -3523.843557 a.u.
DFT-D3(BJ) dispersion correction = -0.117757 a.u.
Thermal free energy = 0.604694 a.u.
Gibbs free energy = -3523.356619 a.u.
Number of imaginary frequencies = 1.

**TS1 ax CL isomer/conformer 3**

| Atomic N. | X | Y | Z |
|---|---|---|---|
| 7 | -1.62491198 | 1.33654094 | 1.41806861 |
| 8 | -0.40532773 | 1.09243665 | -1.21972990 |
| 8 | 0.91220105 | -0.12077479 | 1.52397215 |
| 6 | -3.08853627 | 1.34043314 | 1.19030670 |
| 6 | -0.99196547 | 2.28747692 | -1.42786655 |
| 6 | -1.22027084 | 2.72707491 | -2.76761755 |
| 6 | -1.82588553 | 3.97937343 | -2.93922253 |
| 6 | -2.20827561 | 4.79142852 | -1.86668000 |
| 6 | -1.97978764 | 4.35166138 | -0.56748198 |
| 6 | -1.36719056 | 3.11430152 | -0.34024476 |
| 6 | -1.05301570 | 2.68417441 | 1.06451891 |
| 1 | -2.67879593 | 5.75713780 | -2.05149769 |
| 6 | -0.82047745 | 1.85905629 | -3.97918059 |
| 6 | 1.13599050 | 0.62923033 | 2.61894812 |
| 6 | 2.47289531 | 0.80514819 | 3.09152499 |
| 6 | 2.65133729 | 1.57837222 | 4.24651565 |

| | | | |
|---|---|---|---|
| 6 | 1.58942833 | 2.17880396 | 4.93041369 |
| 6 | 0.29419703 | 2.00743271 | 4.45573097 |
| 6 | 0.05823305 | 1.23113848 | 3.31516414 |
| 6 | -1.35785190 | 1.00201776 | 2.86361140 |
| 1 | 1.78008035 | 2.77780217 | 5.82080529 |
| 6 | 3.67518597 | 0.17803631 | 2.35810311 |
| 6 | -1.14891312 | 2.55514614 | -5.31455557 |
| 6 | -1.59518556 | 0.52129109 | -3.95151693 |
| 6 | 0.70254847 | 1.58610981 | -3.96242520 |
| 6 | 5.00721612 | 0.49313889 | 3.06467692 |
| 6 | 3.77144320 | 0.74014312 | 0.91969013 |
| 6 | 3.53229926 | -1.36197122 | 2.31068660 |
| 1 | -3.54734160 | 2.20045931 | 1.70577492 |
| 1 | -3.30719051 | 1.41605509 | 0.11976103 |
| 1 | -1.44373814 | 3.42548146 | 1.78221874 |
| 1 | 0.03276964 | 2.61450165 | 1.22510223 |
| 1 | -2.01325660 | 4.34089549 | -3.94875658 |
| 1 | -2.26229676 | 4.97222688 | 0.28626016 |
| 1 | -1.64458491 | -0.05647866 | 2.98036749 |
| 1 | -2.03845024 | 1.60710500 | 3.48700339 |
| 1 | 3.65885782 | 1.72918445 | 4.62995530 |
| 1 | -0.55084370 | 2.46887106 | 4.97215047 |
| 1 | -0.83959997 | 1.90124104 | -6.14239032 |
| 1 | -2.22581402 | 2.74676363 | -5.42974480 |
| 1 | -0.61204960 | 3.50819875 | -5.42764175 |
| 1 | -1.34793697 | -0.06950581 | -3.06284015 |
| 1 | -2.68070789 | 0.70161295 | -3.97011956 |
| 1 | -1.33756508 | -0.07491413 | -4.84039079 |
| 1 | 1.26541545 | 2.53010853 | -4.00349471 |
| 1 | 1.00107455 | 1.03730698 | -3.06318973 |
| 1 | 0.98073187 | 0.98817500 | -4.84390729 |
| 1 | 5.82770996 | 0.02126133 | 2.50557488 |
| 1 | 5.03553587 | 0.09833791 | 4.09077281 |
| 1 | 5.21170131 | 1.57299967 | 3.10140211 |
| 1 | 2.87908720 | 0.50084760 | 0.33155181 |
| 1 | 3.89755677 | 1.83255738 | 0.93857849 |
| 1 | 4.64553862 | 0.30784360 | 0.40929447 |
| 1 | 3.48404501 | -1.77868636 | 3.32763106 |
| 1 | 2.63161856 | -1.65969190 | 1.76332756 |
| 1 | 4.40727324 | -1.79991092 | 1.80643925 |
| 1 | -3.52005486 | 0.41886977 | 1.59186529 |
| 8 | -1.70478477 | -1.69759318 | 0.63342563 |
| 6 | -3.09066768 | -2.01242089 | 0.50748448 |
| 1 | -3.52868266 | -1.61992527 | -0.42294086 |
| 6 | -0.03505089 | -3.59878395 | 0.56557051 |
| 6 | 1.02413280 | -4.49574350 | -0.10151334 |
| 6 | 0.43127970 | -5.68170674 | -0.87209237 |
| 6 | -0.62178361 | -2.57130354 | -0.39842368 |
| 6 | -0.52215863 | -5.29742144 | -2.00949504 |
| 6 | -1.71023599 | -4.43853167 | -1.57985733 |
| 1 | -0.82717367 | -4.21251444 | 1.02259104 |
| 1 | 1.65179205 | -3.87680761 | -0.76156566 |
| 1 | -0.11174882 | -6.32858404 | -0.16045444 |

| | | | |
|---|---|---|---|
| 1 | 0.01517713 | -4.75863990 | -2.80616199 |
| 1 | 0.42045642 | -3.00736317 | 1.36946220 |
| 1 | 1.69011100 | -4.88477856 | 0.68335716 |
| 1 | 1.24911824 | -6.29587166 | -1.28056603 |
| 1 | -0.92511806 | -6.21617872 | -2.46726541 |
| 1 | -2.48499879 | -4.44634934 | -2.35788944 |
| 1 | -2.16299746 | -4.82332341 | -0.65079368 |
| 8 | 0.15170100 | -1.57027170 | -0.74520690 |
| 8 | -1.38140258 | -3.03245019 | -1.45653158 |
| 1 | -3.21895654 | -3.10493305 | 0.52434406 |
| 1 | -3.63388199 | -1.60711612 | 1.37298643 |
| 31 | -0.47827586 | -0.04526574 | 0.26426235 |

Electronic energy = -3523.842014 a.u.
DFT-D3(BJ) dispersion correction = -0.116285 a.u.
Thermal free energy = 0.602339 a.u.
Gibbs free energy = -3523.355959 a.u.
Number of imaginary frequencies = 1.

**TS1 ax CL isomer/conformer 4**

| Atomic N. | X | Y | Z |
|---|---|---|---|
| 7 | -0.15687719 | 2.12741446 | 1.15753663 |
| 8 | -0.35481289 | 0.66958928 | -1.37374771 |
| 8 | 1.70989442 | -0.18940265 | 0.92556518 |
| 6 | -1.47301269 | 2.49098897 | 1.73376584 |
| 6 | -0.96120328 | 1.79035032 | -1.81310999 |
| 6 | -1.74781680 | 1.76000214 | -3.00481749 |
| 6 | -2.36992860 | 2.95546174 | -3.39287925 |
| 6 | -2.23659825 | 4.15049934 | -2.67871261 |
| 6 | -1.43405366 | 4.17603350 | -1.54278934 |
| 6 | -0.78663415 | 3.01182580 | -1.11696494 |
| 6 | 0.18356387 | 3.05688691 | 0.02527613 |
| 6 | -1.89041972 | 0.47734463 | -3.84707117 |
| 6 | 2.65267324 | 0.75352514 | 1.13851762 |
| 6 | 4.00775242 | 0.49140391 | 0.77536148 |
| 6 | 4.95004606 | 1.49519992 | 1.03846169 |
| 6 | 4.61211061 | 2.71778131 | 1.62700117 |
| 6 | 3.28913856 | 2.95934749 | 1.97905278 |
| 6 | 2.30931854 | 1.98560119 | 1.75135565 |
| 6 | 0.90131861 | 2.23547792 | 2.22457194 |
| 6 | 4.42154646 | -0.84029120 | 0.11820932 |
| 6 | -2.75929523 | 0.70365149 | -5.09845018 |
| 6 | -2.55419111 | -0.64532322 | -3.01629186 |
| 6 | -0.49582046 | 0.00985697 | -4.32825327 |
| 6 | 5.93547344 | -0.89574355 | -0.16085383 |
| 6 | 3.69601393 | -1.01424801 | -1.23734879 |
| 6 | 4.08089066 | -2.02589822 | 1.05271149 |
| 1 | -1.44102693 | 3.52086067 | 2.12822366 |
| 1 | -2.24153972 | 2.43529153 | 0.95495909 |
| 1 | 0.24912021 | 4.08206224 | 0.43037668 |
| 1 | 1.18962169 | 2.76817846 | -0.31216300 |
| 1 | -2.98615646 | 2.96121502 | -4.29028463 |
| 1 | -1.28621256 | 5.10493326 | -0.98674762 |

```
   1     0.62013739   1.51869178   3.00952625
   1     0.83943212   3.24717474   2.66235805
   1     5.99100971   1.32550645   0.76928252
   1     3.00383449   3.90559387   2.44478798
   1    -2.81451821  -0.23486228  -5.66839123
   1    -3.78829885   0.99583930  -4.84200096
   1    -2.33589149   1.46989524  -5.76382542
   1    -1.93359661  -0.92423409  -2.15795970
   1    -3.54564452  -0.32788971  -2.65827990
   1    -2.69414234  -1.53804994  -3.64549112
   1    -0.02456668   0.77963370  -4.95663548
   1     0.16691230  -0.20543870  -3.48272228
   1    -0.59922391  -0.90467781  -4.93208790
   1     6.17761898  -1.86463141  -0.62024009
   1     6.53010225  -0.80836707   0.76023310
   1     6.25774384  -0.10989711  -0.85944527
   1     2.60908961  -1.05684483  -1.11311328
   1     3.93910575  -0.18476026  -1.91727090
   1     4.02333260  -1.95031643  -1.71487737
   1     4.61763430  -1.93200743   2.00847745
   1     3.00603414  -2.08018088   1.25558257
   1     4.39398670  -2.96997213   0.58123344
   1    -1.72867024   1.79607844   2.53998208
   8    -0.60226357  -1.70636415  -0.04856406
   8    -0.80743396  -3.10332465   1.70370519
   6    -1.36162066  -2.11525059   0.92779338
   6    -2.86554480  -2.13198495   0.66964298
   1    -3.04755116  -2.94220448  -0.05913924
   1    -3.06860280  -1.20038307   0.12626237
   6    -3.81954718  -2.25691533   1.86460865
   1    -4.81382132  -1.91983235   1.53293848
   1    -3.50587940  -1.55263877   2.65051746
   6    -3.95552611  -3.67102671   2.43663931
   1    -4.71968317  -3.67155433   3.22972202
   1    -4.33300114  -4.34172726   1.64502323
   6    -2.65220189  -4.25182009   2.98941445
   1    -2.30238676  -3.67432547   3.86068916
   1    -2.83626127  -5.27652550   3.35346413
   6    -1.52098310  -4.33910894   1.96967232
   1    -1.88204968  -4.76009035   1.01667547
   1    -0.72647667  -4.99678946   2.34454846
  31    -0.05433582   0.11989984   0.40507606
   8    -1.20800138  -0.61401194   1.94637704
   6    -0.66513091  -0.79776525   3.25582099
   1    -1.15060472  -1.66082120   3.73269510
   1     0.42088482  -0.97727227   3.23458123
   1    -0.88098210   0.09307174   3.86829925
   1    -2.74528003   5.05190229  -3.02061057
   1     5.37998056   3.47070241   1.80456460
Electronic energy = -3523.840431 a.u.
DFT-D3(BJ) dispersion correction = -0.117369 a.u.
Thermal free energy = 0.603734 a.u.
Gibbs free energy = -3523.354066 a.u.
```

Number of imaginary frequencies = 1.

**TS1_ax_CL isomer/conformer 5**

| Atomic N. | X | Y | Z |
|---|---|---|---|
| 7 | 0.17226977 | 2.44800635 | 0.19995727 |
| 8 | 1.02412750 | -0.11179285 | -1.14627039 |
| 8 | 0.07405927 | -0.00286870 | 1.83775946 |
| 6 | -0.64835083 | 3.39519850 | -0.58930085 |
| 6 | 1.62781477 | 0.73132683 | -2.01117780 |
| 6 | 2.08701440 | 0.24449636 | -3.27230285 |
| 6 | 2.64742708 | 1.17890122 | -4.15521628 |
| 6 | 2.80037658 | 2.53109076 | -3.83363169 |
| 6 | 2.42273792 | 2.97514023 | -2.57055989 |
| 6 | 1.85671075 | 2.08545894 | -1.65052248 |
| 6 | 1.61020641 | 2.52844938 | -0.23380926 |
| 6 | 2.02716286 | -1.25481975 | -3.63070149 |
| 6 | 0.90221949 | 0.67609175 | 2.65209539 |
| 6 | 1.70467668 | -0.02535627 | 3.60238395 |
| 6 | 2.53300891 | 0.74249355 | 4.43215792 |
| 6 | 2.59833576 | 2.13835140 | 4.36110434 |
| 6 | 1.80551403 | 2.80941092 | 3.43680062 |
| 6 | 0.95205862 | 2.08972023 | 2.59277380 |
| 6 | 0.03328863 | 2.82017057 | 1.65750359 |
| 6 | 1.66227269 | -1.56169186 | 3.71188695 |
| 6 | 2.63184711 | -1.53726664 | -5.01897592 |
| 6 | 0.57115654 | -1.77373113 | -3.64901389 |
| 6 | 2.84793223 | -2.06051269 | -2.59448130 |
| 6 | 2.59122597 | -2.08394504 | 4.82385407 |
| 6 | 2.12615838 | -2.20357636 | 2.38267755 |
| 6 | 0.22640587 | -2.03120780 | 4.04782858 |
| 1 | -0.33192932 | 4.42946010 | -0.37145266 |
| 1 | -0.50637760 | 3.20690892 | -1.65775699 |
| 1 | 1.95844893 | 3.56713051 | -0.09717043 |
| 1 | 2.17479927 | 1.89587796 | 0.46641857 |
| 1 | 2.98636749 | 0.84297614 | -5.13361294 |
| 1 | 2.58618956 | 4.01449449 | -2.27585177 |
| 1 | -1.02194676 | 2.62546820 | 1.91059257 |
| 1 | 0.20714050 | 3.90625092 | 1.74736404 |
| 1 | 3.16095921 | 0.23660203 | 5.16356437 |
| 1 | 1.83329216 | 3.89952255 | 3.36845426 |
| 1 | 2.58503118 | -2.61788256 | -5.21535370 |
| 1 | 2.07911214 | -1.03057892 | -5.82383105 |
| 1 | 3.68746441 | -1.23598443 | -5.07935609 |
| 1 | 0.10326053 | -1.68686829 | -2.66212475 |
| 1 | -0.03034551 | -1.21907264 | -4.38514314 |
| 1 | 0.56101980 | -2.83507772 | -3.94087936 |
| 1 | 3.89914860 | -1.73778958 | -2.59863600 |
| 1 | 2.44727857 | -1.93607653 | -1.58218731 |
| 1 | 2.82101652 | -3.13099518 | -2.84922932 |
| 1 | 2.51606314 | -3.17991913 | 4.86575206 |
| 1 | 2.31126196 | -1.69479137 | 5.81369622 |
| 1 | 3.64535762 | -1.83249074 | 4.63627254 |

| | | | |
|---|---|---|---|
| 1 | 1.46832931 | -1.93091947 | 1.55090244 |
| 1 | 3.15119406 | -1.89013457 | 2.13621378 |
| 1 | 2.12134013 | -3.30003640 | 2.47933765 |
| 1 | -0.10163218 | -1.61302696 | 5.01106849 |
| 1 | -0.48507888 | -1.72864469 | 3.27201210 |
| 1 | 0.20632081 | -3.12866798 | 4.13184922 |
| 1 | -1.70544733 | 3.26819295 | -0.33435500 |
| 8 | -1.47920682 | -1.24832317 | -0.07557772 |
| 8 | -3.12467614 | -0.17560129 | 0.98174443 |
| 6 | -2.64833264 | -0.69594844 | -0.20412259 |
| 6 | -3.65642629 | -1.36089374 | -1.13441821 |
| 1 | -4.26967769 | -0.60635648 | -1.64880593 |
| 1 | -3.06686542 | -1.88110322 | -1.89879172 |
| 6 | -4.58521463 | -2.35229772 | -0.40361146 |
| 1 | -4.01356785 | -2.91202029 | 0.35267783 |
| 1 | -4.93460576 | -3.09258193 | -1.13898392 |
| 6 | -5.81181117 | -1.68021240 | 0.22968208 |
| 1 | -6.36493141 | -1.14867145 | -0.56453062 |
| 1 | -6.49409258 | -2.45603229 | 0.61089298 |
| 6 | -5.49757236 | -0.69714865 | 1.36394376 |
| 1 | -5.12798129 | -1.24039814 | 2.24822521 |
| 1 | -6.42548727 | -0.18772495 | 1.67280575 |
| 6 | -4.46557721 | 0.37169296 | 1.01667890 |
| 1 | -4.68975347 | 0.88369952 | 0.06722488 |
| 1 | -4.42813294 | 1.13258701 | 1.80791003 |
| 31 | -0.33841948 | 0.36435478 | 0.04454424 |
| 8 | -2.12171493 | 0.84488115 | -0.96964836 |
| 6 | -2.05585913 | 0.82312784 | -2.40087683 |
| 1 | -1.60724165 | -0.10853427 | -2.77885848 |
| 1 | -3.06741292 | 0.93961889 | -2.82031861 |
| 1 | -1.44222873 | 1.66355975 | -2.75512870 |
| 1 | 3.23695242 | 3.21965091 | -4.55708030 |
| 1 | 3.26454162 | 2.68921008 | 5.02504616 |

Electronic energy = -3523.841447 a.u.
DFT-D3(BJ) dispersion correction = -0.117546 a.u.
Thermal free energy = 0.605019 a.u.
Gibbs free energy = -3523.353973 a.u.
Number of imaginary frequencies = 1.

**TS1 ax CL isomer/conformer 6**

| Atomic N. | X | Y | Z |
|---|---|---|---|
| 7 | 0.41411718 | 1.97208594 | 1.44723660 |
| 8 | -0.68681056 | 0.89529034 | -1.13414136 |
| 8 | 1.61054250 | -0.54200678 | 0.45645326 |
| 6 | -0.57010452 | 2.52255510 | 2.40772544 |
| 6 | -1.12023470 | 2.16520218 | -1.29058123 |
| 6 | -2.11289198 | 2.45721594 | -2.27453160 |
| 6 | -2.56745736 | 3.78140295 | -2.35475154 |
| 6 | -2.06747438 | 4.80631410 | -1.54580500 |
| 6 | -1.04671920 | 4.52244589 | -0.64453907 |
| 6 | -0.55571338 | 3.21767014 | -0.52273384 |
| 6 | 0.65341493 | 2.95088925 | 0.33248467 |

| | | | |
|---|---|---|---|
| 6 | -2.62698140 | 1.37861302 | -3.25042865 |
| 6 | 2.76275014 | 0.15157310 | 0.52064882 |
| 6 | 3.89999842 | -0.28969231 | -0.22160252 |
| 6 | 5.07612697 | 0.46304145 | -0.10137547 |
| 6 | 5.16894416 | 1.60530363 | 0.70088194 |
| 6 | 4.05614340 | 2.01933286 | 1.42450946 |
| 6 | 2.86084949 | 1.29529174 | 1.34937429 |
| 6 | 1.69101485 | 1.69454134 | 2.20185677 |
| 6 | 3.83837593 | -1.54115657 | -1.11877504 |
| 6 | -3.65403403 | 1.94915247 | -4.24666440 |
| 6 | -3.31357725 | 0.21744445 | -2.49558436 |
| 6 | -1.43863197 | 0.82613347 | -4.07447183 |
| 6 | 5.19237876 | -1.83300189 | -1.79227571 |
| 6 | 2.79574224 | -1.33919703 | -2.24397145 |
| 6 | 3.46441701 | -2.78309371 | -0.27424250 |
| 1 | -0.14986164 | 3.41623462 | 2.89974938 |
| 1 | -1.48074078 | 2.81571831 | 1.87586738 |
| 1 | 1.02335828 | 3.89580433 | 0.76757633 |
| 1 | 1.46548975 | 2.52723507 | -0.27628218 |
| 1 | -3.33955530 | 4.03119313 | -3.08027323 |
| 1 | -0.60309126 | 5.31750297 | -0.04049832 |
| 1 | 1.43931616 | 0.89901174 | 2.92227794 |
| 1 | 1.94970009 | 2.60050613 | 2.77673181 |
| 1 | 5.95894487 | 0.15483481 | -0.65886100 |
| 1 | 4.10728447 | 2.90345285 | 2.06434121 |
| 1 | -3.97066844 | 1.14847885 | -4.93014837 |
| 1 | -4.55482965 | 2.32930703 | -3.74258588 |
| 1 | -3.23103285 | 2.75821754 | -4.85926009 |
| 1 | -2.60846741 | -0.30368732 | -1.83889470 |
| 1 | -4.15921928 | 0.58843096 | -1.89680960 |
| 1 | -3.70984507 | -0.51093558 | -3.21963429 |
| 1 | -0.96440090 | 1.63001182 | -4.65599561 |
| 1 | -0.68206848 | 0.36803248 | -3.42756273 |
| 1 | -1.79870751 | 0.06334026 | -4.78168566 |
| 1 | 5.09609201 | -2.73724816 | -2.41009421 |
| 1 | 5.98945298 | -2.01715041 | -1.05717848 |
| 1 | 5.51131837 | -1.01428394 | -2.45359852 |
| 1 | 1.78869962 | -1.19136113 | -1.84038840 |
| 1 | 3.05681449 | -0.46820188 | -2.86258156 |
| 1 | 2.77838319 | -2.22508912 | -2.89708672 |
| 1 | 4.21773090 | -2.96021186 | 0.50780416 |
| 1 | 2.48471958 | -2.65904827 | 0.19987691 |
| 1 | 3.43143202 | -3.67401631 | -0.92005372 |
| 1 | -0.81262310 | 1.76310255 | 3.15854561 |
| 8 | -0.87046109 | -1.73951654 | 0.09669550 |
| 8 | -0.32738581 | -2.17995881 | 2.27545659 |
| 6 | -1.27833006 | -2.03899054 | 1.27187744 |
| 6 | -2.56482901 | -2.82639042 | 1.41835772 |
| 1 | -2.41390634 | -3.75677657 | 0.84275965 |
| 1 | -3.33685460 | -2.27649620 | 0.86351852 |
| 6 | -3.02513390 | -3.14987163 | 2.84557555 |
| 1 | -4.09620978 | -3.40144752 | 2.80602773 |
| 1 | -2.93690530 | -2.25029122 | 3.47510887 |

| | | | |
|---|---|---|---|
| 6 | -2.27667158 | -4.32257334 | 3.49707493 |
| 1 | -2.79887885 | -4.61334299 | 4.42197967 |
| 1 | -2.33562895 | -5.19578691 | 2.82395944 |
| 6 | -0.80761993 | -4.03841401 | 3.82861073 |
| 1 | -0.73906454 | -3.28764543 | 4.63229091 |
| 1 | -0.33183378 | -4.95673565 | 4.21201452 |
| 6 | 0.01990492 | -3.53832322 | 2.65087979 |
| 1 | -0.06273961 | -4.20409835 | 1.77568216 |
| 1 | 1.08270395 | -3.48018221 | 2.92121043 |
| 31 | -0.13705275 | 0.13266908 | 0.48301612 |
| 8 | -1.62919748 | -0.22659405 | 1.80641977 |
| 6 | -2.92171221 | 0.23877319 | 1.41279812 |
| 1 | -3.18975474 | -0.08882088 | 0.39444449 |
| 1 | -3.67436377 | -0.13103528 | 2.12661948 |
| 1 | -2.95293795 | 1.33879119 | 1.42656147 |
| 1 | -2.45795347 | 5.81942230 | -1.64213097 |
| 1 | 6.10500622 | 2.16110350 | 0.75519824 |

Electronic energy = -3523.834634 a.u.
DFT-D3(BJ) dispersion correction = -0.117804 a.u.
Thermal free energy = 0.603061 a.u.
Gibbs free energy = -3523.349376 a.u.
Number of imaginary frequencies = 1.

**Int1 CL isomer/conformer 1**

| Atomic N. | X | Y | Z |
|---|---|---|---|
| 7 | 0.12169451 | 0.88944251 | 2.05976972 |
| 8 | -1.18608969 | 1.13331065 | -0.61171517 |
| 8 | 1.48644420 | -0.34402034 | -0.32483511 |
| 6 | -0.68350180 | 0.46538682 | 3.23218333 |
| 6 | -2.00217584 | 1.99480968 | 0.04430492 |
| 6 | -3.27759087 | 2.33492032 | -0.49320449 |
| 6 | -4.06238326 | 3.23455537 | 0.24332686 |
| 6 | -3.64306137 | 3.79827747 | 1.45226785 |
| 6 | -2.38585449 | 3.47708604 | 1.95324435 |
| 6 | -1.55868596 | 2.59229213 | 1.25313833 |
| 6 | -0.15367538 | 2.33696346 | 1.72335170 |
| 6 | -3.77704314 | 1.74315308 | -1.82560434 |
| 6 | 2.44356142 | 0.57570374 | -0.03790425 |
| 6 | 3.38462968 | 0.96360946 | -1.03694834 |
| 6 | 4.36449304 | 1.89622263 | -0.66727791 |
| 6 | 4.44267270 | 2.45052969 | 0.61325705 |
| 6 | 3.51711993 | 2.06677793 | 1.57630094 |
| 6 | 2.52585762 | 1.12773586 | 1.26695452 |
| 6 | 1.58913169 | 0.66929263 | 2.35394443 |
| 6 | 3.33418052 | 0.39361944 | -2.46867235 |
| 6 | -5.15592352 | 2.30411249 | -2.22183997 |
| 6 | -3.91993601 | 0.20751451 | -1.70579472 |
| 6 | -2.79261050 | 2.09676081 | -2.96599114 |
| 6 | 4.45481718 | 0.96753955 | -3.35667875 |
| 6 | 1.98841159 | 0.75495039 | -3.14069110 |
| 6 | 3.51675998 | -1.14178890 | -2.43624667 |
| 1 | -0.39760393 | 1.06294481 | 4.11298364 |

| | | | |
|---|---|---|---|
| 1 | -1.74649965 | 0.62447287 | 3.02088447 |
| 1 | 0.06942865 | 2.94164709 | 2.61807134 |
| 1 | 0.57087686 | 2.60737503 | 0.94201102 |
| 1 | -5.04558282 | 3.50759731 | -0.13577359 |
| 1 | -2.02637773 | 3.92623238 | 2.88206204 |
| 1 | 1.68818851 | -0.41238988 | 2.53244153 |
| 1 | 1.82772517 | 1.19580177 | 3.29271931 |
| 1 | 5.09532405 | 2.21354854 | -1.40858406 |
| 1 | 3.56053563 | 2.48493765 | 2.58448714 |
| 1 | -5.45655749 | 1.86371007 | -3.18312530 |
| 1 | -5.93413564 | 2.05185966 | -1.48683863 |
| 1 | -5.13755073 | 3.39630327 | -2.34927237 |
| 1 | -2.96803198 | -0.27751499 | -1.46557106 |
| 1 | -4.64750096 | -0.05306307 | -0.92302264 |
| 1 | -4.28731995 | -0.20547639 | -2.65774061 |
| 1 | -2.71504565 | 3.18737030 | -3.08606183 |
| 1 | -1.79171722 | 1.69546994 | -2.77441189 |
| 1 | -3.16053921 | 1.67812343 | -3.91509894 |
| 1 | 4.37403883 | 0.52606052 | -4.36008218 |
| 1 | 5.45560150 | 0.72667363 | -2.96975819 |
| 1 | 4.37716693 | 2.05855730 | -3.46938091 |
| 1 | 1.13460197 | 0.33516791 | -2.59903595 |
| 1 | 1.86150194 | 1.84586893 | -3.19270030 |
| 1 | 1.97103068 | 0.36277300 | -4.16881428 |
| 1 | 4.49031288 | -1.40736867 | -1.99864028 |
| 1 | 2.72817573 | -1.62686474 | -1.85181619 |
| 1 | 3.48565525 | -1.54205991 | -3.46096082 |
| 1 | -0.50287343 | -0.59782358 | 3.42230668 |
| 8 | -1.30760263 | -1.65317425 | 0.29189742 |
| 8 | 0.03144442 | -2.44454532 | 1.91606166 |
| 6 | -0.97297252 | -2.83125060 | 0.93752953 |
| 6 | -0.50261258 | -3.92872155 | -0.03980571 |
| 1 | -0.73649874 | -4.90799248 | 0.40451526 |
| 1 | -1.15307972 | -3.82124205 | -0.91831347 |
| 6 | 0.97374489 | -3.88019016 | -0.45216436 |
| 1 | 1.28848679 | -2.83886299 | -0.62141426 |
| 1 | 1.07387828 | -4.39352263 | -1.42099530 |
| 6 | 1.91660219 | -4.56261739 | 0.55117244 |
| 1 | 1.55665812 | -5.59188095 | 0.72532546 |
| 1 | 2.91762126 | -4.65909973 | 0.10177838 |
| 6 | 2.05445199 | -3.84579094 | 1.89972850 |
| 1 | 2.60850468 | -2.90224348 | 1.76631986 |
| 1 | 2.65228026 | -4.46736954 | 2.58790466 |
| 6 | 0.72967753 | -3.51799042 | 2.58434003 |
| 1 | 0.06989389 | -4.39774805 | 2.65661367 |
| 1 | 0.91263103 | -3.16076145 | 3.61023048 |
| 31 | -0.24197548 | -0.16604606 | 0.33480798 |
| 8 | -2.10356370 | -3.40402373 | 1.57971186 |
| 6 | -2.87430492 | -2.51823913 | 2.39823622 |
| 1 | -2.30951464 | -2.19674756 | 3.28874103 |
| 1 | -3.20659887 | -1.63779654 | 1.83040580 |
| 1 | -3.74699095 | -3.09758434 | 2.72095139 |
| 1 | -4.29400698 | 4.48919723 | 1.98794597 |

```
   1     5.21977603   3.17755256   0.84855864
Electronic energy = -3523.859547 a.u.
DFT-D3(BJ) dispersion correction = -0.118218 a.u.
Thermal free energy = 0.605551 a.u.
Gibbs free energy = -3523.372215 a.u.
Number of imaginary frequencies = 0.
```

**Int1 CL isomer/conformer 2**

| Atomic N. | X | Y | Z |
|---|---|---|---|
| 7 | 0.03867938 | 2.33514050 | 0.00044847 |
| 8 | 1.33987041 | -0.11177063 | -1.11794427 |
| 8 | -0.08098907 | -0.23723826 | 1.58192790 |
| 6 | -0.86110310 | 3.14162013 | -0.86475529 |
| 6 | 1.83957312 | 0.76380999 | -2.02471883 |
| 6 | 2.35010369 | 0.29941463 | -3.27188797 |
| 6 | 2.85303195 | 1.26396903 | -4.15741069 |
| 6 | 2.87348784 | 2.63044445 | -3.86228227 |
| 6 | 2.39376506 | 3.06825984 | -2.63227124 |
| 6 | 1.89080266 | 2.14690943 | -1.70746665 |
| 6 | 1.48746971 | 2.60310233 | -0.33190134 |
| 6 | 2.35352821 | -1.19835035 | -3.63538065 |
| 6 | 0.62915465 | 0.47988027 | 2.48595251 |
| 6 | 1.36247731 | -0.19703067 | 3.50505714 |
| 6 | 2.06884591 | 0.59301805 | 4.42257818 |
| 6 | 2.08010338 | 1.98986903 | 4.37025684 |
| 6 | 1.35329068 | 2.63785630 | 3.37838513 |
| 6 | 0.61657430 | 1.89958333 | 2.44390594 |
| 6 | -0.24886271 | 2.63750799 | 1.45342069 |
| 6 | 1.38253296 | -1.73583929 | 3.59760830 |
| 6 | 3.00209836 | -1.45501452 | -5.00883515 |
| 6 | 0.90366959 | -1.73364071 | -3.70242603 |
| 6 | 3.16526490 | -1.99754187 | -2.58797662 |
| 6 | 2.21579124 | -2.22854301 | 4.79598827 |
| 6 | 2.01377479 | -2.33938799 | 2.32058608 |
| 6 | -0.05486559 | -2.27758800 | 3.78601671 |
| 1 | -0.70656078 | 4.21334329 | -0.65924295 |
| 1 | -0.62883942 | 2.93865129 | -1.91567027 |
| 1 | 1.66856159 | 3.68478145 | -0.21737402 |
| 1 | 2.07338915 | 2.07829368 | 0.43661584 |
| 1 | 3.24286304 | 0.94169942 | -5.12135507 |
| 1 | 2.42328003 | 4.12846061 | -2.37018463 |
| 1 | -1.31074289 | 2.38519237 | 1.59401391 |
| 1 | -0.13220281 | 3.72384656 | 1.60123742 |
| 1 | 2.64187151 | 0.10411825 | 5.20824841 |
| 1 | 1.33888262 | 3.72895464 | 3.32656921 |
| 1 | 2.99230718 | -2.53552700 | -5.21116746 |
| 1 | 2.45515015 | -0.96201769 | -5.82571693 |
| 1 | 4.04963484 | -1.12185263 | -5.04022613 |
| 1 | 0.38112613 | -1.62385194 | -2.74635128 |
| 1 | 0.33022423 | -1.19860752 | -4.47393269 |
| 1 | 0.91490577 | -2.80138886 | -3.97019849 |
| 1 | 4.20899808 | -1.65155928 | -2.56201294 |

| | | | |
|---|---|---|---|
| 1 | 2.73978835 | -1.89564935 | -1.58393738 |
| 1 | 3.17010380 | -3.06442575 | -2.85876813 |
| 1 | 2.18770424 | -3.32715059 | 4.82164497 |
| 1 | 1.81814486 | -1.86497455 | 5.75466784 |
| 1 | 3.27067136 | -1.92779026 | 4.71937993 |
| 1 | 1.44757922 | -2.07502645 | 1.42144841 |
| 1 | 3.04697444 | -1.98508422 | 2.19418505 |
| 1 | 2.03986218 | -3.43666527 | 2.40277592 |
| 1 | -0.49665081 | -1.88682718 | 4.71453595 |
| 1 | -0.70199166 | -1.99942409 | 2.94717612 |
| 1 | -0.03040463 | -3.37548586 | 3.85984833 |
| 1 | -1.90239622 | 2.86233457 | -0.67010848 |
| 8 | -1.60180229 | -0.37518715 | -1.17064860 |
| 8 | -2.84964632 | 0.67353741 | 0.38028118 |
| 6 | -2.90290568 | -0.01977545 | -0.86492833 |
| 6 | -3.80042086 | -1.27410433 | -0.85415671 |
| 1 | -4.84835553 | -0.96982735 | -1.00434386 |
| 1 | -3.50833423 | -1.86105177 | -1.73529227 |
| 6 | -3.68172823 | -2.13792305 | 0.41143995 |
| 1 | -2.63073939 | -2.17304289 | 0.73842367 |
| 1 | -3.95962517 | -3.17159799 | 0.15402323 |
| 6 | -4.58974839 | -1.66942057 | 1.55921304 |
| 1 | -5.62921699 | -1.63607193 | 1.18799231 |
| 1 | -4.57588030 | -2.42232937 | 2.36301855 |
| 6 | -4.22844743 | -0.30519294 | 2.15957375 |
| 1 | -3.28300874 | -0.37712115 | 2.72047791 |
| 1 | -5.00532061 | -0.00645759 | 2.88364074 |
| 6 | -4.07217273 | 0.81771017 | 1.13766091 |
| 1 | -4.93165276 | 0.88855589 | 0.45256716 |
| 1 | -3.98230287 | 1.78663321 | 1.65140616 |
| 31 | -0.21711424 | 0.31498540 | -0.18378604 |
| 8 | -3.40589132 | 0.97095982 | -1.78645196 |
| 6 | -3.42210500 | 0.56464033 | -3.15947725 |
| 1 | -2.44094282 | 0.17556512 | -3.47086099 |
| 1 | -4.19808255 | -0.19430970 | -3.35637437 |
| 1 | -3.65776932 | 1.46485830 | -3.73997931 |
| 1 | 3.27045926 | 3.34052366 | -4.58773858 |
| 1 | 2.65245134 | 2.56063630 | 5.10134405 |

Electronic energy = -3523.859111 a.u.
DFT-D3(BJ) dispersion correction = -0.116822 a.u.
Thermal free energy = 0.604671 a.u.
Gibbs free energy = -3523.371262 a.u.
Number of imaginary frequencies = 0.

**Int1 CL isomer/conformer 3**

| Atomic N. | X | Y | Z |
|---|---|---|---|
| 7 | 0.25028685 | 1.75353062 | 1.41300965 |
| 8 | -0.45405925 | 0.99189152 | -1.39253027 |
| 8 | 1.48030005 | -0.73426048 | 0.23635463 |
| 6 | -0.82998388 | 2.13997186 | 2.35811035 |
| 6 | -1.00771528 | 2.22930115 | -1.41730830 |
| 6 | -1.98169585 | 2.56419230 | -2.40267106 |

| | | | |
|---|---|---|---|
| 6 | -2.50838355 | 3.86374383 | -2.36650645 |
| 6 | -2.11543510 | 4.81782295 | -1.42298969 |
| 6 | -1.14677140 | 4.48553735 | -0.48169329 |
| 6 | -0.58099991 | 3.20602896 | -0.47961947 |
| 6 | 0.55186863 | 2.88297585 | 0.45548055 |
| 6 | -2.43809052 | 1.54583742 | -3.46571351 |
| 6 | 2.63878287 | -0.06000401 | 0.43675905 |
| 6 | 3.81649348 | -0.45049971 | -0.26652612 |
| 6 | 4.98981133 | 0.27111119 | -0.00581225 |
| 6 | 5.03965658 | 1.33855916 | 0.89529661 |
| 6 | 3.88585003 | 1.70579104 | 1.57784625 |
| 6 | 2.68886789 | 1.00978357 | 1.36851385 |
| 6 | 1.48338252 | 1.36766376 | 2.20040140 |
| 6 | 3.80550404 | -1.61433356 | -1.27757846 |
| 6 | -3.45440068 | 2.15742871 | -4.44850105 |
| 6 | -3.12370924 | 0.33697949 | -2.78606476 |
| 6 | -1.22489365 | 1.06675657 | -4.29830436 |
| 6 | 5.19999511 | -1.86287631 | -1.88315690 |
| 6 | 2.84657495 | -1.29258419 | -2.44826766 |
| 6 | 3.36872111 | -2.92459325 | -0.57990593 |
| 1 | -0.48789065 | 2.98428113 | 2.97840369 |
| 1 | -1.71425286 | 2.44806212 | 1.78978949 |
| 1 | 0.82269513 | 3.76988358 | 1.05198068 |
| 1 | 1.44233135 | 2.57003178 | -0.10882474 |
| 1 | -3.26007310 | 4.14797727 | -3.10078871 |
| 1 | -0.80611191 | 5.22308294 | 0.24871929 |
| 1 | 1.16664368 | 0.52333538 | 2.83159626 |
| 1 | 1.73399434 | 2.21222279 | 2.86318687 |
| 1 | 5.90395006 | -0.00126875 | -0.52998560 |
| 1 | 3.90480223 | 2.53149809 | 2.29275911 |
| 1 | -3.73378089 | 1.39685966 | -5.19142981 |
| 1 | -4.37722045 | 2.48047262 | -3.94494552 |
| 1 | -3.03675550 | 3.01580404 | -4.99444643 |
| 1 | -2.44985341 | -0.18348442 | -2.09724917 |
| 1 | -4.01207068 | 0.66299030 | -2.22478584 |
| 1 | -3.45499059 | -0.38053974 | -3.55234946 |
| 1 | -0.75447851 | 1.91397080 | -4.81855967 |
| 1 | -0.47036039 | 0.58183966 | -3.66984628 |
| 1 | -1.55990289 | 0.34669470 | -5.06038716 |
| 1 | 5.13793414 | -2.70542219 | -2.58644025 |
| 1 | 5.94373592 | -2.12569370 | -1.11690021 |
| 1 | 5.56974701 | -0.99245679 | -2.44413168 |
| 1 | 1.81724415 | -1.14803405 | -2.10391346 |
| 1 | 3.16718411 | -0.38061464 | -2.97219566 |
| 1 | 2.85526630 | -2.12036437 | -3.17356918 |
| 1 | 4.06630164 | -3.18280734 | 0.23047099 |
| 1 | 2.36081858 | -2.83778030 | -0.16027154 |
| 1 | 3.37437286 | -3.75135687 | -1.30638854 |
| 1 | -1.09000300 | 1.28286290 | 2.98835282 |
| 8 | -1.58499746 | -1.00835418 | 0.49148464 |
| 8 | -0.64809809 | -1.35482768 | 2.58763025 |
| 6 | -1.83218643 | -1.45439271 | 1.78355721 |
| 6 | -2.45353535 | -2.85885623 | 1.70574285 |

| | | | |
|---|---|---|---|
| 1 | -1.84044379 | -3.46998018 | 1.02720952 |
| 1 | -3.41537861 | -2.73114046 | 1.18938261 |
| 6 | -2.65135104 | -3.56892725 | 3.05224986 |
| 1 | -3.48192397 | -4.28509802 | 2.95377555 |
| 1 | -2.96142033 | -2.83476840 | 3.81267958 |
| 6 | -1.40582739 | -4.33366595 | 3.52098089 |
| 1 | -1.65074079 | -4.91584645 | 4.42355701 |
| 1 | -1.13574460 | -5.07003291 | 2.74401189 |
| 6 | -0.18708483 | -3.45132050 | 3.81333036 |
| 1 | -0.36661247 | -2.83602450 | 4.70988790 |
| 1 | 0.68018362 | -4.09417989 | 4.04032123 |
| 6 | 0.21474189 | -2.51139333 | 2.67835547 |
| 1 | 0.27304102 | -3.03091398 | 1.70849931 |
| 1 | 1.21135395 | -2.09223729 | 2.87428822 |
| 31 | -0.16457045 | 0.12880412 | 0.23444365 |
| 8 | -2.68497658 | -0.54423787 | 2.50399738 |
| 6 | -3.90070110 | -0.19241930 | 1.83707219 |
| 1 | -3.71802358 | 0.10880203 | 0.79518488 |
| 1 | -4.63244718 | -1.01790868 | 1.85530041 |
| 1 | -4.31779544 | 0.65340129 | 2.39737538 |
| 1 | -2.55934765 | 5.81324689 | -1.43468474 |
| 1 | 5.97465283 | 1.87446899 | 1.05789344 |

Electronic energy = -3523.858507 a.u.
DFT-D3(BJ) dispersion correction = -0.117212 a.u.
Thermal free energy = 0.605181 a.u.
Gibbs free energy = -3523.370538 a.u.
Number of imaginary frequencies = 0.

**Int1 CL isomer/conformer 4**

| Atomic N. | X | Y | Z |
|---|---|---|---|
| 7 | -1.80434031 | 0.61583779 | 0.84639761 |
| 8 | 0.39360696 | 1.26016607 | -1.06483549 |
| 8 | 0.98147535 | -0.32980645 | 1.47939820 |
| 6 | -3.07442263 | 0.04675061 | 0.33395463 |
| 6 | -0.55182336 | 2.04381539 | -1.64122891 |
| 6 | -0.40062337 | 2.48997088 | -2.98612549 |
| 6 | -1.41936376 | 3.29990065 | -3.50936811 |
| 6 | -2.54559024 | 3.67828636 | -2.77219580 |
| 6 | -2.66863572 | 3.25675960 | -1.45238969 |
| 6 | -1.67578412 | 2.45570330 | -0.87810546 |
| 6 | -1.74054142 | 2.10195902 | 0.58214195 |
| 1 | -3.31177788 | 4.30531418 | -3.22811032 |
| 6 | 0.82770381 | 2.10329460 | -3.83266297 |
| 6 | 0.84559442 | 0.52093779 | 2.52887386 |
| 6 | 2.00206517 | 1.01160192 | 3.20393863 |
| 6 | 1.79313894 | 1.86495117 | 4.29687955 |
| 6 | 0.52068806 | 2.24585822 | 4.73184826 |
| 6 | -0.59802722 | 1.76058210 | 4.06502595 |
| 6 | -0.44857200 | 0.89388732 | 2.97580927 |
| 6 | -1.67725290 | 0.31669381 | 2.32371209 |
| 1 | 0.41158305 | 2.91766417 | 5.58307924 |
| 6 | 3.42757698 | 0.62896436 | 2.75821848 |

| | | | |
|---|---|---|---|
| 6 | 0.78906235 | 2.75042055 | -5.23006479 |
| 6 | 0.87393145 | 0.57040410 | -4.03437849 |
| 6 | 2.12380844 | 2.58647868 | -3.13884188 |
| 6 | 4.50370266 | 1.28249789 | 3.64613547 |
| 6 | 3.68087753 | 1.10476644 | 1.30823995 |
| 6 | 3.62020131 | -0.90256457 | 2.85645588 |
| 1 | -3.92792388 | 0.50886510 | 0.85631902 |
| 1 | -3.15775572 | 0.24643666 | -0.73986784 |
| 1 | -2.62172680 | 2.56898318 | 1.05281439 |
| 1 | -0.84423103 | 2.46200827 | 1.10707325 |
| 1 | -1.33803578 | 3.64978424 | -4.53694833 |
| 1 | -3.52646827 | 3.56280657 | -0.84897440 |
| 1 | -1.68478062 | -0.78200875 | 2.39750243 |
| 1 | -2.57935274 | 0.70223862 | 2.82675420 |
| 1 | 2.65636024 | 2.25835563 | 4.83025379 |
| 1 | -1.60232993 | 2.04102819 | 4.39042660 |
| 1 | 1.69186003 | 2.45596727 | -5.78386632 |
| 1 | -0.07976334 | 2.41947005 | -5.81746650 |
| 1 | 0.77639101 | 3.84884723 | -5.17742064 |
| 1 | 0.94121729 | 0.03229565 | -3.08316250 |
| 1 | -0.02595081 | 0.22269901 | -4.56250722 |
| 1 | 1.74926089 | 0.30357758 | -4.64624198 |
| 1 | 2.11857679 | 3.68079427 | -3.02788518 |
| 1 | 2.24264081 | 2.13667006 | -2.14729205 |
| 1 | 2.99570961 | 2.31379541 | -3.75277247 |
| 1 | 5.49582359 | 0.97242216 | 3.28856134 |
| 1 | 4.41962526 | 0.97118637 | 4.69743293 |
| 1 | 4.46616451 | 2.38058300 | 3.60298599 |
| 1 | 2.98808908 | 0.64055594 | 0.59891282 |
| 1 | 3.57033400 | 2.19606296 | 1.23294137 |
| 1 | 4.70773726 | 0.84686677 | 1.00783486 |
| 1 | 3.49119071 | -1.24422034 | 3.89390342 |
| 1 | 2.90418153 | -1.43705186 | 2.22347963 |
| 1 | 4.63832644 | -1.17124125 | 2.53623076 |
| 1 | -3.07314557 | -1.03464461 | 0.50617072 |
| 8 | 0.61811827 | -3.69546908 | -0.73240997 |
| 6 | 1.73910781 | -3.13794869 | -0.03569299 |
| 1 | 2.50068111 | -3.92620228 | -0.02132588 |
| 6 | -1.47372381 | -3.62136791 | -1.77519077 |
| 6 | -2.98235118 | -3.55054645 | -1.51110373 |
| 6 | -3.47768331 | -4.53815747 | -0.44068505 |
| 6 | -0.55554105 | -2.89089708 | -0.76711519 |
| 6 | -3.05291128 | -4.22202882 | 0.99948012 |
| 6 | -1.55523191 | -3.99285086 | 1.18726804 |
| 1 | -1.15950678 | -4.67482566 | -1.81273628 |
| 1 | -3.28041349 | -2.52105154 | -1.25771226 |
| 1 | -3.11432432 | -5.54560205 | -0.70793882 |
| 1 | -3.58458104 | -3.32539212 | 1.36054265 |
| 1 | -1.23620204 | -3.18618954 | -2.75506217 |
| 1 | -3.49777085 | -3.78751480 | -2.45519695 |
| 1 | -4.57770031 | -4.59109605 | -0.47726904 |
| 1 | -3.36032826 | -5.04901220 | 1.66198057 |
| 1 | -1.31678490 | -3.88692281 | 2.25666297 |

| | | | |
|---|---|---|---|
| 1 | -0.95860315 | -4.83221871 | 0.79606257 |
| 8 | -0.31762196 | -1.59034343 | -1.17451224 |
| 8 | -1.12621523 | -2.76463902 | 0.56216571 |
| 1 | 2.14179494 | -2.26016919 | -0.56376930 |
| 1 | 1.47972072 | -2.85703529 | 0.99577991 |
| 31 | -0.12221411 | -0.18674827 | -0.00921562 |

Electronic energy = -3523.858081 a.u.
DFT-D3(BJ) dispersion correction = -0.117461 a.u.
Thermal free energy = 0.605326 a.u.
Gibbs free energy = -3523.370216 a.u.
Number of imaginary frequencies = 0.

**Int1 CL isomer/conformer 5**

| Atomic N. | X | Y | Z |
|---|---|---|---|
| 8 | -3.16936157 | 0.76653660 | 0.11609534 |
| 6 | -4.25152513 | 0.92663411 | 1.04079714 |
| 1 | -4.31125472 | 2.00096484 | 1.25536541 |
| 6 | -3.91245260 | -1.15313687 | -1.22829251 |
| 6 | -3.51072800 | -2.41903837 | -1.99947022 |
| 6 | -3.55866577 | -3.71172767 | -1.17900083 |
| 6 | -2.84414791 | -0.60272286 | -0.26378071 |
| 6 | -2.72408426 | -3.67904280 | 0.10590517 |
| 6 | -3.19762327 | -2.64147243 | 1.12730737 |
| 1 | -4.85727340 | -1.31287790 | -0.68474484 |
| 1 | -2.50089173 | -2.26786671 | -2.40771073 |
| 1 | -4.60682181 | -3.92922587 | -0.90630507 |
| 1 | -1.66055529 | -3.49896578 | -0.11692596 |
| 1 | -4.08859756 | -0.33744221 | -1.94347414 |
| 1 | -4.18768406 | -2.52421408 | -2.86193344 |
| 1 | -3.22656694 | -4.55054669 | -1.81109920 |
| 1 | -2.78258727 | -4.66683190 | 0.59241305 |
| 1 | -2.84525519 | -2.91696642 | 2.13100975 |
| 1 | -4.30370685 | -2.62388913 | 1.16126090 |
| 8 | -1.63968734 | -0.51197121 | -0.93046089 |
| 8 | -2.68381913 | -1.30944959 | 0.96513674 |
| 1 | -4.06247057 | 0.37740522 | 1.97303288 |
| 1 | -5.21175076 | 0.60313444 | 0.60516384 |
| 7 | -0.30916365 | 2.44589425 | -0.77014731 |
| 8 | 1.31224901 | -0.05747183 | -0.87244444 |
| 8 | -0.19803155 | 0.69750359 | 1.68752397 |
| 6 | -1.28227649 | 2.71396672 | -1.85953228 |
| 6 | 1.72797720 | 0.45200649 | -2.05896530 |
| 6 | 2.32971049 | -0.39428105 | -3.03525690 |
| 6 | 2.73949123 | 0.20292502 | -4.23639012 |
| 6 | 2.58472496 | 1.56744751 | -4.49831909 |
| 6 | 2.01686176 | 2.38654248 | -3.52805610 |
| 6 | 1.60042781 | 1.84420353 | -2.30758685 |
| 6 | 1.10021404 | 2.74878707 | -1.21374963 |
| 1 | 2.91485327 | 1.98216133 | -5.45074837 |
| 6 | 2.52706082 | -1.90225564 | -2.78473399 |
| 6 | 0.39798686 | 1.78738501 | 2.23003692 |
| 6 | 1.16581310 | 1.64734719 | 3.42336143 |

```
   6     1.74981602   2.80712868   3.95154277
   6     1.60863509   4.06526595   3.35852067
   6     0.84728959   4.18983937   2.20215532
   6     0.22884852   3.06700944   1.63868832
   6    -0.68300123   3.25103778   0.45243625
   1     2.08982068   4.93668106   3.80239589
   6     1.34910612   0.27809331   4.10752765
   6     3.23961878  -2.59257851  -3.96310639
   6     1.15623071  -2.59626265  -2.60472876
   6     3.40115885  -2.12176146  -1.52683512
   6     2.18271128   0.38888202   5.39822350
   6     2.09386799  -0.69408552   3.16211354
   6    -0.02731905  -0.31237606   4.49785894
   1    -1.26706601   3.78591195  -2.11566713
   1    -1.00907947   2.12704363  -2.74305051
   1     1.13905532   3.80173887  -1.53969339
   1     1.72512783   2.64924383  -0.31417579
   1     3.19522938  -0.41840834  -5.00530312
   1     1.90888946   3.45986657  -3.70102303
   1    -1.70994439   2.93847188   0.69711213
   1    -0.70655293   4.31554196   0.16536603
   1     2.34628652   2.73119386   4.85884732
   1     0.71248505   5.16596227   1.73053824
   1     3.36752071  -3.65867641  -3.72716239
   1     2.65942594  -2.52843618  -4.89514724
   1     4.23914754  -2.17190923  -4.14540219
   1     0.60209854  -2.18831266  -1.75276652
   1     0.53986978  -2.47493988  -3.50762894
   1     1.30572056  -3.67431930  -2.43876297
   1     4.39396186  -1.66834099  -1.66256428
   1     2.93942970  -1.68689209  -0.63392816
   1     3.54303290  -3.20010248  -1.35735393
   1     2.27251547  -0.60884974   5.85088017
   1     1.71010386   1.04795535   6.14097678
   1     3.20127649   0.75543007   5.20466848
   1     1.53644693  -0.86752820   2.23574960
   1     3.08561115  -0.29782682   2.90029948
   1     2.23803534  -1.66246199   3.66509229
   1    -0.54665676   0.35261440   5.20368021
   1    -0.66727639  -0.45952109   3.62114198
   1     0.11583132  -1.28426271   4.99464871
   1    -2.28315092   2.41983119  -1.52396096
  31    -0.31454590   0.49366047  -0.14748444
Electronic energy = -3523.856028 a.u.
DFT-D3(BJ) dispersion correction = -0.115934 a.u.
Thermal free energy = 0.602178 a.u.
Gibbs free energy = -3523.369784 a.u.
Number of imaginary frequencies = 0.
```

**Int1_CL isomer/conformer 6**

```
Atomic N.     X            Y            Z
   7     0.24747755   1.19007650   1.86958445
```

| | | | |
|---|---|---|---|
| 8 | -1.43441913 | 0.84540809 | -0.56569519 |
| 8 | 1.52499455 | 0.13725074 | -0.66250533 |
| 6 | -0.17961064 | 0.63630088 | 3.17926791 |
| 6 | -2.38061063 | 1.46232074 | 0.18470062 |
| 6 | -3.75935228 | 1.37437068 | -0.16475253 |
| 6 | -4.67296180 | 2.04014660 | 0.66581786 |
| 6 | -4.28497652 | 2.77424449 | 1.79086927 |
| 6 | -2.93346453 | 2.87759727 | 2.10366103 |
| 6 | -1.98003673 | 2.24068062 | 1.30243408 |
| 6 | -0.51298488 | 2.45612272 | 1.55131868 |
| 1 | -5.03649391 | 3.26821468 | 2.40674090 |
| 6 | -4.23094101 | 0.58663295 | -1.40245881 |
| 6 | 2.19010788 | 1.32002923 | -0.61335014 |
| 6 | 2.81226873 | 1.83612628 | -1.78834787 |
| 6 | 3.49943836 | 3.05259960 | -1.67085396 |
| 6 | 3.58955344 | 3.76099049 | -0.46951822 |
| 6 | 2.98460827 | 3.24412906 | 0.66998790 |
| 6 | 2.29647507 | 2.02597773 | 0.61427032 |
| 6 | 1.73707325 | 1.44635692 | 1.88866463 |
| 1 | 4.12866980 | 4.70740405 | -0.43105818 |
| 6 | 2.73956040 | 1.09334221 | -3.13745656 |
| 6 | -5.75445121 | 0.69120275 | -1.60425522 |
| 6 | -3.88842145 | -0.91305976 | -1.24180519 |
| 6 | -3.55888588 | 1.15055757 | -2.67720868 |
| 6 | 3.49368646 | 1.84523430 | -4.25075961 |
| 6 | 1.26855355 | 0.95439203 | -3.59546837 |
| 6 | 3.39122140 | -0.30354937 | -3.00657063 |
| 1 | 0.04149905 | 1.36149775 | 3.97916932 |
| 1 | -1.25758026 | 0.44183831 | 3.15720118 |
| 1 | -0.36241993 | 3.16125330 | 2.38572740 |
| 1 | -0.03033154 | 2.87683971 | 0.65754346 |
| 1 | -5.73466715 | 1.98363512 | 0.43209732 |
| 1 | -2.60483659 | 3.47134680 | 2.95994119 |
| 1 | 2.19446774 | 0.47076162 | 2.11161010 |
| 1 | 1.95325809 | 2.12653011 | 2.72912604 |
| 1 | 3.97991513 | 3.47362909 | -2.55195657 |
| 1 | 3.05004104 | 3.77671713 | 1.62148604 |
| 1 | -6.03363948 | 0.12899995 | -2.50676207 |
| 1 | -6.31556197 | 0.26098382 | -0.76195869 |
| 1 | -6.08270921 | 1.73106700 | -1.74703013 |
| 1 | -2.81204933 | -1.07803528 | -1.12790262 |
| 1 | -4.39380550 | -1.33328063 | -0.36015901 |
| 1 | -4.23390953 | -1.46878737 | -2.12712667 |
| 1 | -3.83075535 | 2.20631082 | -2.82334233 |
| 1 | -2.46734781 | 1.07650612 | -2.62417900 |
| 1 | -3.90514113 | 0.58815982 | -3.55778792 |
| 1 | 3.41641164 | 1.26962096 | -5.18407240 |
| 1 | 4.56300298 | 1.96169186 | -4.02227592 |
| 1 | 3.06686680 | 2.84051563 | -4.44114910 |
| 1 | 0.67072833 | 0.37979682 | -2.88072144 |
| 1 | 0.80430304 | 1.94389409 | -3.71435038 |
| 1 | 1.23053058 | 0.44326180 | -4.56943006 |
| 1 | 4.45369207 | -0.21185756 | -2.73740652 |

| | | | |
|---|---|---|---|
| 1 | 2.89105419 | -0.90847913 | -2.24266849 |
| 1 | 3.33080737 | -0.83507159 | -3.96844237 |
| 1 | 0.35932589 | -0.29961004 | 3.36348597 |
| 8 | 0.46955179 | -3.70535206 | 0.27834920 |
| 6 | 0.76580599 | -3.26354722 | -1.05596931 |
| 1 | 1.53327005 | -2.47395753 | -1.07026697 |
| 6 | -0.65258000 | -3.39064589 | 2.35455377 |
| 6 | 0.11076420 | -4.51756378 | 3.05664750 |
| 6 | 1.50319772 | -4.14598461 | 3.59077794 |
| 6 | 0.06483650 | -2.70002897 | 1.18504845 |
| 6 | 2.54045974 | -3.94630827 | 2.47530054 |
| 6 | 2.50858073 | -2.57374053 | 1.79007671 |
| 1 | -0.89802019 | -2.59393808 | 3.07260233 |
| 1 | -0.51373175 | -4.86875891 | 3.89358964 |
| 1 | 1.84979759 | -4.96055351 | 4.24571485 |
| 1 | 3.55820988 | -4.06976527 | 2.88030986 |
| 1 | -1.60489967 | -3.77319970 | 1.95997553 |
| 1 | 0.21480665 | -5.37055472 | 2.36777471 |
| 1 | 1.44095357 | -3.24459969 | 4.22464509 |
| 1 | 2.40097464 | -4.73699487 | 1.72465750 |
| 1 | 2.97026583 | -2.63900141 | 0.79172509 |
| 1 | 3.09663673 | -1.85310655 | 2.37916258 |
| 8 | -0.76253206 | -1.75004704 | 0.62129034 |
| 8 | 1.21683791 | -1.93357116 | 1.68809026 |
| 1 | 1.14594724 | -4.14903039 | -1.57833586 |
| 1 | -0.13955476 | -2.89860526 | -1.56095420 |
| 31 | -0.05680567 | -0.08198068 | 0.29131492 |

Electronic energy = -3523.857228 a.u.
DFT-D3(BJ) dispersion correction = -0.117874 a.u.
Thermal free energy = 0.605667 a.u.
Gibbs free energy = -3523.369435 a.u.
Number of imaginary frequencies = 0.

**Int1_CL isomer/conformer 7**

| Atomic N. | X | Y | Z |
|---|---|---|---|
| 8 | -2.19355057 | -0.29728717 | -1.05183677 |
| 6 | -3.15639137 | -0.22278755 | -2.10947224 |
| 1 | -2.66467783 | 0.31751995 | -2.92742048 |
| 6 | -3.11858674 | -2.31368679 | 0.09225174 |
| 6 | -4.62926398 | -2.55413654 | -0.05844994 |
| 6 | -5.41718174 | -2.42653572 | 1.25451087 |
| 6 | -2.61263838 | -0.85129906 | 0.20379623 |
| 6 | -5.53789398 | -0.99545616 | 1.78877081 |
| 6 | -4.20553181 | -0.26910525 | 1.95052822 |
| 1 | -2.76011164 | -2.83423221 | 0.99235061 |
| 1 | -5.06422733 | -1.88403505 | -0.81577318 |
| 1 | -6.42930028 | -2.83706770 | 1.10969638 |
| 1 | -6.17199600 | -0.39542027 | 1.11526571 |
| 1 | -2.57015283 | -2.76295695 | -0.74883625 |
| 1 | -4.76273537 | -3.57822720 | -0.44271418 |
| 1 | -4.93356093 | -3.06076570 | 2.01778749 |
| 1 | -6.04413528 | -1.00885802 | 2.76902960 |

| | | | |
|---|---|---|---|
| 1 | -4.36497167 | 0.69221563 | 2.46265243 |
| 1 | -3.49054913 | -0.84872767 | 2.55694200 |
| 8 | -1.48621803 | -0.82795244 | 0.99944225 |
| 8 | -3.61353517 | 0.06533478 | 0.67758128 |
| 1 | -4.05921799 | 0.31978106 | -1.79260947 |
| 1 | -3.43206609 | -1.22754955 | -2.46711994 |
| 7 | -0.34584246 | 2.29873000 | 0.23808944 |
| 8 | 0.97430626 | -0.11122971 | -0.97807648 |
| 8 | 0.95405100 | 0.26611992 | 2.03223960 |
| 6 | -1.71181419 | 2.73251139 | -0.15080981 |
| 6 | 0.76701601 | 0.55202089 | -2.14195374 |
| 6 | 0.84310471 | -0.13534340 | -3.38769509 |
| 6 | 0.58701006 | 0.61146720 | -4.54750254 |
| 6 | 0.28595604 | 1.97662237 | -4.52140469 |
| 6 | 0.26789105 | 2.64665291 | -3.30184564 |
| 6 | 0.52289594 | 1.95066626 | -2.11558367 |
| 6 | 0.65834959 | 2.69856142 | -0.81594634 |
| 1 | 0.08785078 | 2.51059172 | -5.45080899 |
| 6 | 1.21783566 | -1.62881111 | -3.46540852 |
| 6 | 1.79591090 | 1.28206511 | 2.32181852 |
| 6 | 3.06947883 | 1.01673256 | 2.90697858 |
| 6 | 3.88725336 | 2.11898100 | 3.19164421 |
| 6 | 3.50481083 | 3.43811420 | 2.92741939 |
| 6 | 2.25551099 | 3.68510362 | 2.37012287 |
| 6 | 1.39356579 | 2.62110888 | 2.07764861 |
| 6 | 0.00405831 | 2.91345106 | 1.57823044 |
| 1 | 4.18075400 | 4.26071328 | 3.16055473 |
| 6 | 3.53014290 | -0.42156094 | 3.21347341 |
| 6 | 1.29916666 | -2.12648686 | -4.92087527 |
| 6 | 0.16144156 | -2.49393259 | -2.74027900 |
| 6 | 2.60844366 | -1.85123469 | -2.82344098 |
| 6 | 4.92202711 | -0.45124320 | 3.87258500 |
| 6 | 3.62295101 | -1.24081127 | 1.90439582 |
| 6 | 2.54150125 | -1.09888255 | 4.19249942 |
| 1 | -1.75988965 | 3.83364552 | -0.14921139 |
| 1 | -1.93671914 | 2.35597429 | -1.15254898 |
| 1 | 0.56034851 | 3.78375321 | -0.98597158 |
| 1 | 1.64737835 | 2.51357685 | -0.37223017 |
| 1 | 0.62264328 | 0.11260590 | -5.51439527 |
| 1 | 0.07883058 | 3.72197692 | -3.26224728 |
| 1 | -0.75385271 | 2.52943111 | 2.28000733 |
| 1 | -0.13553152 | 4.00372613 | 1.49234455 |
| 1 | 4.86782192 | 1.94785834 | 3.63228946 |
| 1 | 1.93048978 | 4.70853199 | 2.16892771 |
| 1 | 1.58729827 | -3.18735928 | -4.91962040 |
| 1 | 0.33347238 | -2.04778939 | -5.44145762 |
| 1 | 2.05427966 | -1.57930052 | -5.50352502 |
| 1 | 0.06914601 | -2.21676156 | -1.68515814 |
| 1 | -0.82308858 | -2.38302197 | -3.21828090 |
| 1 | 0.44794289 | -3.55520556 | -2.79835917 |
| 1 | 3.37714445 | -1.27152928 | -3.35504044 |
| 1 | 2.61679477 | -1.55601617 | -1.76856319 |
| 1 | 2.88194934 | -2.91528945 | -2.88976309 |

| | | | |
|---|---|---|---|
| 1 | 5.19600112 | -1.49547125 | 4.07987044 |
| 1 | 4.93863391 | 0.09122722 | 4.82907616 |
| 1 | 5.69986592 | -0.02995791 | 3.21923523 |
| 1 | 2.65368969 | -1.31032686 | 1.39979100 |
| 1 | 4.34271111 | -0.78332121 | 1.21021473 |
| 1 | 3.96965904 | -2.26101209 | 2.12905091 |
| 1 | 2.50081074 | -0.54752934 | 5.14346134 |
| 1 | 1.53064811 | -1.14824397 | 3.77370133 |
| 1 | 2.87996226 | -2.12325563 | 4.41091710 |
| 1 | -2.44618343 | 2.31805782 | 0.54925423 |
| 31 | -0.09939053 | 0.27158425 | 0.49578870 |

Electronic energy = -3523.856375 a.u.
DFT-D3(BJ) dispersion correction = -0.117569 a.u.
Thermal free energy = 0.605506 a.u.
Gibbs free energy = -3523.368439 a.u.
Number of imaginary frequencies = 0.

**Int1 CL isomer/conformer 8**

| Atomic N. | X | Y | Z |
|---|---|---|---|
| 7 | -0.22074715 | 2.33732154 | 1.05991260 |
| 8 | -0.20130689 | 0.80333100 | -1.49979961 |
| 8 | 1.48482261 | -0.14728519 | 0.87815715 |
| 6 | -1.52833283 | 2.71483955 | 1.65465540 |
| 6 | -0.88590718 | 1.87948013 | -1.96204136 |
| 6 | -1.65521025 | 1.78429094 | -3.15805195 |
| 6 | -2.33174127 | 2.93867645 | -3.57864492 |
| 6 | -2.27421110 | 4.14965632 | -2.88246142 |
| 6 | -1.50205220 | 4.23535226 | -1.72872089 |
| 6 | -0.79758080 | 3.11670456 | -1.27094884 |
| 6 | 0.13187359 | 3.24282479 | -0.09422997 |
| 6 | -1.74223634 | 0.47062721 | -3.95942667 |
| 6 | 2.49473890 | 0.73518780 | 1.07453677 |
| 6 | 3.82956712 | 0.36246060 | 0.73922026 |
| 6 | 4.83871713 | 1.30659462 | 0.97415853 |
| 6 | 4.58382970 | 2.57157919 | 1.51209041 |
| 6 | 3.27977165 | 2.91728471 | 1.84702486 |
| 6 | 2.23388417 | 2.00783736 | 1.64743635 |
| 6 | 0.85016780 | 2.36712181 | 2.12585127 |
| 6 | 4.15371413 | -1.02174081 | 0.14305523 |
| 6 | -2.59080535 | 0.63038633 | -5.23510978 |
| 6 | -2.40426900 | -0.63314974 | -3.10122276 |
| 6 | -0.32856599 | 0.02280058 | -4.40154064 |
| 6 | 5.66411661 | -1.20280998 | -0.09959231 |
| 6 | 3.44645837 | -1.19269618 | -1.22231299 |
| 6 | 3.70676938 | -2.13928790 | 1.11609676 |
| 1 | -1.46570569 | 3.73240535 | 2.07345013 |
| 1 | -2.30083185 | 2.69012664 | 0.87851098 |
| 1 | 0.14266614 | 4.28167772 | 0.27591034 |
| 1 | 1.15911335 | 2.97511341 | -0.38186941 |
| 1 | -2.93432573 | 2.89584333 | -4.48419143 |
| 1 | -1.42296328 | 5.17814590 | -1.18239309 |
| 1 | 0.50709449 | 1.65867381 | 2.89574162 |

| | | | |
|---|---|---|---|
| 1 | 0.86259708 | 3.37713303 | 2.56823888 |
| 1 | 5.86704567 | 1.05296131 | 0.72322167 |
| 1 | 3.06065367 | 3.89509060 | 2.28207969 |
| 1 | -2.60774093 | -0.32759051 | -5.77416314 |
| 1 | -3.63290227 | 0.90030043 | -5.01014279 |
| 1 | -2.17449446 | 1.38632745 | -5.91666819 |
| 1 | -1.83597468 | -0.83551582 | -2.18721286 |
| 1 | -3.42463170 | -0.33988892 | -2.81415449 |
| 1 | -2.47062910 | -1.56524022 | -3.68334551 |
| 1 | 0.13305461 | 0.78355648 | -5.04798604 |
| 1 | 0.32824970 | -0.14756335 | -3.54183242 |
| 1 | -0.39948798 | -0.91196344 | -4.97848027 |
| 1 | 5.84089135 | -2.20659715 | -0.51140762 |
| 1 | 6.24562003 | -1.11954779 | 0.83006205 |
| 1 | 6.05846059 | -0.47548454 | -0.82401670 |
| 1 | 2.35756866 | -1.12627073 | -1.12790463 |
| 1 | 3.77951244 | -0.42088136 | -1.93116402 |
| 1 | 3.69766801 | -2.17558923 | -1.64932974 |
| 1 | 4.24120522 | -2.05407158 | 2.07373584 |
| 1 | 2.63022025 | -2.09721557 | 1.31383598 |
| 1 | 3.94638023 | -3.12290822 | 0.68377306 |
| 1 | -1.78254364 | 1.99684666 | 2.44236599 |
| 8 | -1.69755966 | -0.55540048 | 0.69192928 |
| 8 | -0.79102011 | -2.35420405 | 1.86809066 |
| 6 | -1.72598024 | -1.27742856 | 1.87113425 |
| 6 | -3.19422847 | -1.71517595 | 2.12888915 |
| 1 | -3.69407174 | -1.73144733 | 1.14929250 |
| 1 | -3.66497620 | -0.89748300 | 2.69455684 |
| 6 | -3.43217247 | -3.05951825 | 2.83541277 |
| 1 | -4.47140175 | -3.05769542 | 3.20220029 |
| 1 | -2.79263632 | -3.15639236 | 3.72610050 |
| 6 | -3.24249117 | -4.28801990 | 1.93192601 |
| 1 | -3.66387576 | -5.17296244 | 2.43521999 |
| 1 | -3.83652092 | -4.14420952 | 1.01251119 |
| 6 | -1.78699143 | -4.58282700 | 1.55425057 |
| 1 | -1.22233487 | -4.89271129 | 2.44899574 |
| 1 | -1.75253948 | -5.43206038 | 0.85079184 |
| 6 | -1.05201012 | -3.40755995 | 0.91720471 |
| 1 | -1.59904757 | -3.00401363 | 0.04898381 |
| 1 | -0.06137442 | -3.72842306 | 0.56267170 |
| 31 | -0.18513575 | 0.42675929 | 0.32577736 |
| 8 | -1.23155615 | -0.37910740 | 2.88720945 |
| 6 | -1.06784320 | -0.91808692 | 4.20542187 |
| 1 | -2.04174975 | -1.13905087 | 4.67229115 |
| 1 | -0.44492879 | -1.82232419 | 4.19403850 |
| 1 | -0.56681070 | -0.13788594 | 4.79212029 |
| 1 | -2.82365937 | 5.01711292 | -3.24811244 |
| 1 | 5.40168146 | 3.27485403 | 1.66833668 |

Electronic energy = -3523.857146 a.u.
DFT-D3(BJ) dispersion correction = -0.115550 a.u.
Thermal free energy = 0.604391 a.u.
Gibbs free energy = -3523.368305 a.u.
Number of imaginary frequencies = 0.

**Int1 CL isomer/conformer 9**

| Atomic N. | X | Y | Z |
|---|---|---|---|
| 7 | 0.43240051 | 2.54252149 | -0.18538807 |
| 8 | 1.28558722 | -0.18884496 | -1.02609770 |
| 8 | 0.10500132 | 0.26600632 | 1.77124403 |
| 6 | -0.46351397 | 3.32699987 | -1.07274051 |
| 6 | 1.78053499 | 0.47216847 | -2.10268590 |
| 6 | 2.08164745 | -0.23072543 | -3.30528029 |
| 6 | 2.59491233 | 0.51884754 | -4.37399254 |
| 6 | 2.81912967 | 1.89670334 | -4.29784979 |
| 6 | 2.54263811 | 2.56686614 | -3.11068596 |
| 6 | 2.03921566 | 1.86520271 | -2.01015063 |
| 6 | 1.85340542 | 2.56457256 | -0.69077539 |
| 6 | 1.85625008 | -1.75026308 | -3.43036362 |
| 6 | 1.01023789 | 0.97555443 | 2.48936970 |
| 6 | 1.74064800 | 0.33328943 | 3.53194653 |
| 6 | 2.65274719 | 1.11578855 | 4.25376131 |
| 6 | 2.86522614 | 2.47184536 | 3.98848472 |
| 6 | 2.13747596 | 3.08874484 | 2.97742697 |
| 6 | 1.20187942 | 2.35896497 | 2.23390278 |
| 6 | 0.34586183 | 3.07986190 | 1.22375335 |
| 6 | 1.54028339 | -1.16100359 | 3.85389199 |
| 6 | 2.29994225 | -2.28582785 | -4.80481175 |
| 6 | 0.35375079 | -2.08143227 | -3.26923720 |
| 6 | 2.68172497 | -2.49935742 | -2.35704139 |
| 6 | 2.40877561 | -1.61284422 | 5.04329889 |
| 6 | 1.94316263 | -2.02598640 | 2.63603936 |
| 6 | 0.06444707 | -1.43352601 | 4.23213639 |
| 1 | -0.15509267 | 4.38520661 | -1.07076170 |
| 1 | -0.40005021 | 2.93330638 | -2.09286212 |
| 1 | 2.17482985 | 3.61696585 | -0.76605340 |
| 1 | 2.45656976 | 2.08036199 | 0.09110077 |
| 1 | 2.82704441 | 0.01245840 | -5.30923349 |
| 1 | 2.73359084 | 3.63880443 | -3.02063146 |
| 1 | -0.71936215 | 3.00757794 | 1.49185882 |
| 1 | 0.62220710 | 4.14721142 | 1.19911349 |
| 1 | 3.22913881 | 0.65231650 | 5.05233171 |
| 1 | 2.27793859 | 4.15069508 | 2.76308553 |
| 1 | 2.13249840 | -3.37192893 | -4.83497310 |
| 1 | 1.72502415 | -1.84070527 | -5.62994073 |
| 1 | 3.36945792 | -2.11059149 | -4.99130196 |
| 1 | -0.03027449 | -1.77284817 | -2.29127378 |
| 1 | -0.23904717 | -1.57783070 | -4.04686655 |
| 1 | 0.20043994 | -3.16645236 | -3.37550962 |
| 1 | 3.75594511 | -2.30385950 | -2.48970635 |
| 1 | 2.39526184 | -2.19652137 | -1.34421784 |
| 1 | 2.52136512 | -3.58383386 | -2.45619948 |
| 1 | 2.21912830 | -2.67792474 | 5.23831490 |
| 1 | 2.17022805 | -1.06023074 | 5.96371749 |
| 1 | 3.48349945 | -1.50200320 | 4.83854358 |
| 1 | 1.32934845 | -1.80233096 | 1.75737908 |

| | | | |
|---|---|---|---|
| 1 | 2.99786989 | -1.85917457 | 2.37372324 |
| 1 | 1.81951696 | -3.09181658 | 2.88137045 |
| 1 | -0.21983438 | -0.85263449 | 5.12206921 |
| 1 | -0.61578307 | -1.17586733 | 3.41314456 |
| 1 | -0.06404928 | -2.50030175 | 4.47131637 |
| 1 | -1.49374559 | 3.23389843 | -0.71094108 |
| 8 | -1.67767355 | 0.18387647 | -0.91290007 |
| 8 | -2.84073894 | -0.84019844 | 0.75075893 |
| 6 | -2.83595818 | 0.24429168 | -0.17259245 |
| 6 | -4.03907608 | 0.32921711 | -1.12474702 |
| 1 | -4.96040530 | 0.51649709 | -0.54999268 |
| 1 | -3.85081455 | 1.23198614 | -1.72128337 |
| 6 | -4.22410682 | -0.88178103 | -2.05238178 |
| 1 | -3.23694343 | -1.20340672 | -2.41773277 |
| 1 | -4.79139073 | -0.55411147 | -2.93744212 |
| 6 | -4.96776007 | -2.05722827 | -1.40785942 |
| 1 | -5.97874250 | -1.72112253 | -1.11559598 |
| 1 | -5.11468178 | -2.84917157 | -2.15943522 |
| 6 | -4.27437005 | -2.65242891 | -0.17845081 |
| 1 | -3.31118290 | -3.10892941 | -0.45717527 |
| 1 | -4.90164628 | -3.46131524 | 0.23281418 |
| 6 | -4.01284803 | -1.65031529 | 0.94697612 |
| 1 | -4.90575450 | -1.02562414 | 1.12874029 |
| 1 | -3.79567907 | -2.18834288 | 1.88080518 |
| 31 | -0.10448137 | 0.56889475 | -0.04263799 |
| 8 | -2.73350764 | 1.49419405 | 0.59044946 |
| 6 | -3.57311332 | 1.60202448 | 1.74288070 |
| 1 | -3.33978396 | 0.82292664 | 2.48194954 |
| 1 | -3.36962698 | 2.58826926 | 2.17896979 |
| 1 | -4.64376161 | 1.55491052 | 1.47853286 |
| 1 | 3.21462050 | 2.43455297 | -5.15949372 |
| 1 | 3.59239121 | 3.03626104 | 4.57209177 |

Electronic energy = -3523.856158 a.u.
DFT-D3(BJ) dispersion correction = -0.115706 a.u.
Thermal free energy = 0.603792 a.u.
Gibbs free energy = -3523.368072 a.u.
Number of imaginary frequencies = 0.

**Int1 CL isomer/conformer 10**

| Atomic N. | X | Y | Z |
|---|---|---|---|
| 8 | -0.86596809 | -3.50405731 | 1.35582553 |
| 6 | -0.63436799 | -4.45619620 | 2.39311092 |
| 1 | -1.61828540 | -4.67742289 | 2.82446522 |
| 1 | 0.01820672 | -4.05002305 | 3.18398637 |
| 1 | -0.20303997 | -5.39791198 | 2.01339242 |
| 6 | 0.30332778 | -2.95606541 | 0.71570026 |
| 8 | -0.17638069 | -2.01658586 | -0.16352726 |
| 7 | -0.92207256 | 0.44081133 | 2.09096500 |
| 8 | -1.42469504 | 0.57959384 | -0.85235550 |
| 8 | 1.43196337 | 0.61969811 | 0.21596777 |
| 6 | -1.61708391 | -0.58311101 | 2.91320419 |
| 6 | -2.71517448 | 0.73415838 | -0.46286450 |

| | | | |
|---|---|---|---|
| 6 | -3.78146112 | 0.48973876 | -1.37583420 |
| 6 | -5.08722145 | 0.67594383 | -0.89837049 |
| 6 | -5.36892387 | 1.08669748 | 0.40806706 |
| 6 | -4.31817387 | 1.34373724 | 1.28235558 |
| 6 | -2.99619064 | 1.18412215 | 0.85373421 |
| 6 | -1.85732214 | 1.57899729 | 1.75279297 |
| 1 | -6.40256603 | 1.21057284 | 0.73109633 |
| 6 | -3.51697959 | 0.03883635 | -2.82555158 |
| 6 | 1.63129619 | 1.80364867 | 0.84642344 |
| 6 | 2.43183120 | 2.81163475 | 0.23199451 |
| 6 | 2.62762821 | 4.00168931 | 0.94717045 |
| 6 | 2.06785781 | 4.23133478 | 2.20710327 |
| 6 | 1.28447083 | 3.24346667 | 2.79223601 |
| 6 | 1.06958333 | 2.02806199 | 2.13116200 |
| 6 | 0.29551325 | 0.94248888 | 2.83497452 |
| 1 | 2.24296593 | 5.17701656 | 2.71982608 |
| 6 | 3.04522747 | 2.61809542 | -1.16962275 |
| 6 | -4.82347016 | -0.10800053 | -3.62839381 |
| 6 | -2.81253721 | -1.33830204 | -2.83417101 |
| 6 | -2.64408781 | 1.08646684 | -3.55701161 |
| 6 | 3.88567654 | 3.83477888 | -1.60217449 |
| 6 | 1.92100051 | 2.44065847 | -2.21758950 |
| 6 | 3.98229810 | 1.38748543 | -1.18590000 |
| 1 | -1.95482802 | -0.12720427 | 3.85811031 |
| 1 | -2.48227918 | -0.96821084 | 2.36378239 |
| 1 | -2.24057939 | 1.99453364 | 2.69952074 |
| 1 | -1.23402843 | 2.34683187 | 1.27222593 |
| 1 | -5.92437725 | 0.48861700 | -1.56847522 |
| 1 | -4.51387250 | 1.68947540 | 2.30009038 |
| 1 | 0.91996446 | 0.05058294 | 2.99633620 |
| 1 | -0.03888595 | 1.30881117 | 3.81961748 |
| 1 | 3.23308077 | 4.78983175 | 0.50362720 |
| 1 | 0.83934349 | 3.40103932 | 3.77730483 |
| 1 | -4.57780956 | -0.41386555 | -4.65534530 |
| 1 | -5.48642016 | -0.87690335 | -3.20568031 |
| 1 | -5.38081253 | 0.83816167 | -3.68890215 |
| 1 | -1.84855287 | -1.31099489 | -2.31573671 |
| 1 | -3.44169471 | -2.09790908 | -2.34797175 |
| 1 | -2.63801852 | -1.65774850 | -3.87318751 |
| 1 | -3.15553300 | 2.05966361 | -3.59255576 |
| 1 | -1.67558474 | 1.22013108 | -3.06332702 |
| 1 | -2.46582159 | 0.76095867 | -4.59328683 |
| 1 | 4.30251891 | 3.64222963 | -2.60091003 |
| 1 | 4.72939821 | 4.01899425 | -0.92124436 |
| 1 | 3.28389785 | 4.75259729 | -1.66832904 |
| 1 | 1.29807924 | 1.56560198 | -2.00447214 |
| 1 | 1.27177361 | 3.32758924 | -2.24304194 |
| 1 | 2.36229674 | 2.31502470 | -3.21802892 |
| 1 | 4.79927455 | 1.51132426 | -0.45982363 |
| 1 | 3.43868787 | 0.46741007 | -0.94710985 |
| 1 | 4.43148861 | 1.27721210 | -2.18456587 |
| 1 | -0.92736776 | -1.40857145 | 3.11633281 |
| 31 | -0.21241015 | -0.24454315 | 0.29747537 |

| | | | |
|---|---|---|---|
| 6 | 1.06563577 | -4.04339396 | -0.06208213 |
| 6 | 2.24535469 | -3.54528277 | -0.90978313 |
| 6 | 3.54833141 | -3.35171910 | -0.12663156 |
| 6 | 3.46068677 | -2.33258149 | 1.01362098 |
| 6 | 2.41255732 | -2.66251163 | 2.07566098 |
| 1 | 1.39797782 | -4.84269272 | 0.61807013 |
| 1 | 1.95303993 | -2.60825897 | -1.40715139 |
| 1 | 3.86167406 | -4.32516024 | 0.29155903 |
| 1 | 3.24836718 | -1.32389322 | 0.62607837 |
| 1 | 0.29938569 | -4.48271121 | -0.71593068 |
| 1 | 2.42553817 | -4.27888900 | -1.71079224 |
| 1 | 4.34658759 | -3.04683016 | -0.82171460 |
| 1 | 4.43825248 | -2.27894686 | 1.52182194 |
| 1 | 2.62403442 | -2.09566285 | 2.99452834 |
| 1 | 2.44547238 | -3.73429794 | 2.34023160 |
| 8 | 1.06333109 | -2.26989638 | 1.74789764 |

Electronic energy = -3523.854509 a.u.
DFT-D3(BJ) dispersion correction = -0.117667 a.u.
Thermal free energy = 0.604349 a.u.
Gibbs free energy = -3523.367828 a.u.
Number of imaginary frequencies = 0.

**Int1_CL isomer/conformer 11**

| Atomic N. | X | Y | Z |
|---|---|---|---|
| 7 | 0.34017697 | 1.28487633 | 1.86562875 |
| 8 | -1.27302091 | 0.84238979 | -0.61031298 |
| 8 | 1.77386785 | 0.62045147 | -0.70705207 |
| 6 | -0.05683744 | 0.59505812 | 3.12002764 |
| 6 | -2.28491800 | 1.31468870 | 0.15829512 |
| 6 | -3.64160492 | 1.06986272 | -0.20422279 |
| 6 | -4.63435345 | 1.59318113 | 0.63716669 |
| 6 | -4.34410183 | 2.33181678 | 1.78824367 |
| 6 | -3.01654413 | 2.58289870 | 2.11878993 |
| 6 | -1.98708054 | 2.09335358 | 1.30776220 |
| 6 | -0.55883094 | 2.46795454 | 1.59526835 |
| 1 | -5.15286729 | 2.71253522 | 2.41198317 |
| 6 | -4.00684839 | 0.26531259 | -1.46698201 |
| 6 | 2.31283043 | 1.85022166 | -0.53079610 |
| 6 | 2.91546010 | 2.52196866 | -1.63545349 |
| 6 | 3.46988845 | 3.78659562 | -1.39459809 |
| 6 | 3.44884640 | 4.39763867 | -0.13730810 |
| 6 | 2.86736568 | 3.72845763 | 0.93323783 |
| 6 | 2.31117065 | 2.45581166 | 0.75387044 |
| 6 | 1.78754390 | 1.71302647 | 1.95697911 |
| 1 | 3.88575303 | 5.38691883 | -0.00199660 |
| 6 | 2.95646817 | 1.88708310 | -3.03961188 |
| 6 | -5.53101457 | 0.17948829 | -1.67299751 |
| 6 | -3.47533228 | -1.18268422 | -1.34760515 |
| 6 | -3.41129923 | 0.94610165 | -2.72249663 |
| 6 | 3.68538424 | 2.78667738 | -4.05576222 |
| 6 | 1.51895234 | 1.66906438 | -3.56859582 |
| 6 | 3.71524818 | 0.53916847 | -2.99295420 |

| | | | |
|---|---|---|---|
| 1 | 0.09062722 | 1.27284897 | 3.97651274 |
| 1 | -1.11399015 | 0.31516984 | 3.05908662 |
| 1 | -0.50661809 | 3.14129566 | 2.46702706 |
| 1 | -0.11523117 | 2.99139489 | 0.73583532 |
| 1 | -5.68001204 | 1.41656408 | 0.39134591 |
| 1 | -2.76684368 | 3.17896944 | 2.99968755 |
| 1 | 2.35161080 | 0.78222777 | 2.12061378 |
| 1 | 1.89687265 | 2.34328306 | 2.85500689 |
| 1 | 3.93210162 | 4.32548974 | -2.21964684 |
| 1 | 2.85054770 | 4.18279301 | 1.92659089 |
| 1 | -5.73494736 | -0.38889495 | -2.59162314 |
| 1 | -6.03380731 | -0.34117435 | -0.84495590 |
| 1 | -5.98923666 | 1.17236346 | -1.78941572 |
| 1 | -2.38566297 | -1.21203727 | -1.24271422 |
| 1 | -3.92042326 | -1.68644109 | -0.47675768 |
| 1 | -3.75251541 | -1.75424786 | -2.24674070 |
| 1 | -3.81575291 | 1.96221569 | -2.83958645 |
| 1 | -2.31939415 | 1.01048006 | -2.66718104 |
| 1 | -3.68230567 | 0.36962233 | -3.62017128 |
| 1 | 3.69564264 | 2.28386321 | -5.03325281 |
| 1 | 4.73049326 | 2.97325997 | -3.76882703 |
| 1 | 3.18191145 | 3.75518841 | -4.18895335 |
| 1 | 0.94486336 | 0.99562282 | -2.92377194 |
| 1 | 0.98095262 | 2.62574160 | -3.63530901 |
| 1 | 1.55789871 | 1.23142558 | -4.57776292 |
| 1 | 4.75345235 | 0.69191242 | -2.66307170 |
| 1 | 3.23369513 | -0.17107614 | -2.31241806 |
| 1 | 3.74337139 | 0.09512293 | -3.99973795 |
| 1 | 0.54431843 | -0.31210114 | 3.24422619 |
| 8 | 1.87402088 | -1.70794850 | 1.43271732 |
| 6 | 2.91759691 | -2.34729767 | 2.17613969 |
| 1 | 3.64245753 | -1.56119657 | 2.42113735 |
| 8 | -0.08024211 | -1.69519650 | 0.31917119 |
| 1 | 3.42559595 | -3.11626001 | 1.57307702 |
| 1 | 2.53328283 | -2.79559439 | 3.10415424 |
| 31 | 0.23934865 | 0.10662942 | 0.19611989 |
| 6 | 1.07379357 | -3.78172002 | 0.29611269 |
| 6 | 1.25367975 | -5.10117037 | 1.06362628 |
| 6 | -0.06775902 | -5.76437124 | 1.48294955 |
| 6 | 0.72279391 | -2.50200238 | 1.10210135 |
| 6 | -0.81878851 | -5.03836243 | 2.60444803 |
| 6 | -1.09080501 | -3.56204450 | 2.32944238 |
| 1 | 0.27218904 | -3.90654068 | -0.44623822 |
| 1 | 1.89291421 | -4.96235728 | 1.94897827 |
| 1 | -0.72079842 | -5.83839812 | 0.59597407 |
| 1 | -0.24831498 | -5.10980215 | 3.54534548 |
| 1 | 1.97840193 | -3.54199298 | -0.28254095 |
| 1 | 1.79398719 | -5.79658603 | 0.40117887 |
| 1 | 0.13382678 | -6.79888314 | 1.80397053 |
| 1 | -1.78567274 | -5.53846057 | 2.78450175 |
| 1 | -1.73931934 | -3.14792687 | 3.11613744 |
| 1 | -1.60530613 | -3.40973042 | 1.36680360 |
| 8 | 0.11719616 | -2.77358665 | 2.37214967 |

Electronic energy = -3523.856329 a.u.
DFT-D3(BJ) dispersion correction = -0.115456 a.u.
Thermal free energy = 0.604581 a.u.
Gibbs free energy = -3523.367205 a.u.
Number of imaginary frequencies = 0.

**Int1 CL isomer/conformer 12**

| Atomic N. | X | Y | Z |
|---|---|---|---|
| 8 | -0.67416286 | -1.27117923 | 2.67596036 |
| 6 | -0.97656123 | -1.65959625 | 4.02067080 |
| 1 | -0.82967977 | -0.76560741 | 4.63900344 |
| 8 | -0.58850699 | -1.64141377 | 0.46171715 |
| 1 | -2.01891525 | -2.00091093 | 4.11005428 |
| 1 | -0.29349331 | -2.44673430 | 4.37674950 |
| 7 | -1.49948599 | 1.55451335 | 1.00479250 |
| 8 | -0.01457433 | 0.57477200 | -1.39555976 |
| 8 | 1.37574771 | 0.67858054 | 1.31752249 |
| 6 | -2.85458027 | 1.01471865 | 1.28649638 |
| 6 | -1.10044499 | 1.04103498 | -2.05952117 |
| 6 | -1.38060697 | 0.60013940 | -3.38589263 |
| 6 | -2.52043207 | 1.12811339 | -4.00987222 |
| 6 | -3.36673565 | 2.05308312 | -3.39113044 |
| 6 | -3.06902453 | 2.49378058 | -2.10589705 |
| 6 | -1.93788899 | 2.00733492 | -1.44189529 |
| 6 | -1.54666161 | 2.57659822 | -0.10562338 |
| 1 | -4.24406535 | 2.42898970 | -3.91750887 |
| 6 | -0.47087735 | -0.41225217 | -4.10895975 |
| 6 | 1.55690370 | 1.96980000 | 1.68245528 |
| 6 | 2.86940125 | 2.52881645 | 1.66677927 |
| 6 | 3.00463672 | 3.86613354 | 2.06397310 |
| 6 | 1.91996560 | 4.65270431 | 2.46304542 |
| 6 | 0.64788317 | 4.09316169 | 2.48540832 |
| 6 | 0.45557520 | 2.75667892 | 2.11376752 |
| 6 | -0.91693875 | 2.15073336 | 2.26568393 |
| 1 | 2.07450170 | 5.69151411 | 2.75434071 |
| 6 | 4.09250982 | 1.70024743 | 1.22638827 |
| 6 | -0.95013523 | -0.69229935 | -5.54590780 |
| 6 | -0.46916990 | -1.76158746 | -3.35233434 |
| 6 | 0.97037088 | 0.14264311 | -4.20591281 |
| 6 | 5.40027969 | 2.50826040 | 1.32646084 |
| 6 | 3.93520528 | 1.26324815 | -0.24951911 |
| 6 | 4.25068817 | 0.45750414 | 2.13489758 |
| 1 | -3.50579090 | 1.82484139 | 1.65309976 |
| 1 | -3.27723901 | 0.59792352 | 0.36621012 |
| 1 | -2.24979373 | 3.37175289 | 0.19342917 |
| 1 | -0.53911902 | 3.01512000 | -0.15189529 |
| 1 | -2.76570259 | 0.80438329 | -5.01985668 |
| 1 | -3.70173273 | 3.23548660 | -1.61276259 |
| 1 | -0.90212377 | 1.32444750 | 2.99255415 |
| 1 | -1.61898228 | 2.91781996 | 2.63231837 |
| 1 | 3.99350028 | 4.32092366 | 2.05665266 |
| 1 | -0.21163528 | 4.68595815 | 2.80683174 |

| | | | |
|---|---|---|---|
| 1 | -0.26038356 | -1.40583451 | -6.01888099 |
| 1 | -1.95446091 | -1.13980246 | -5.56970003 |
| 1 | -0.95970600 | 0.21766316 | -6.16329827 |
| 1 | -0.10185811 | -1.65419341 | -2.32630252 |
| 1 | -1.48458466 | -2.18317175 | -3.31381645 |
| 1 | 0.17619089 | -2.48108752 | -3.87946410 |
| 1 | 0.98517661 | 1.08058236 | -4.78019881 |
| 1 | 1.39652096 | 0.33458201 | -3.21547064 |
| 1 | 1.61243873 | -0.58304226 | -4.72818777 |
| 1 | 6.23838204 | 1.86942195 | 1.01357223 |
| 1 | 5.60387888 | 2.84096065 | 2.35470164 |
| 1 | 5.39503689 | 3.38981950 | 0.66916455 |
| 1 | 3.04634565 | 0.64123354 | -0.39789476 |
| 1 | 3.85614771 | 2.14113672 | -0.90692207 |
| 1 | 4.81829981 | 0.68499726 | -0.56142912 |
| 1 | 4.40938729 | 0.76120211 | 3.18015981 |
| 1 | 3.36824158 | -0.18949838 | 2.08978147 |
| 1 | 5.12870901 | -0.12476755 | 1.81607677 |
| 1 | -2.78105607 | 0.21851588 | 2.03493508 |
| 31 | -0.14946104 | 0.13789031 | 0.41370588 |
| 6 | -0.16655753 | -3.54376342 | 1.85846236 |
| 6 | -0.02505727 | -4.42722884 | 0.61082485 |
| 6 | -1.29461104 | -5.18829827 | 0.21529894 |
| 6 | -0.96832963 | -2.24224842 | 1.64467658 |
| 6 | -2.53906551 | -4.30789553 | 0.04972541 |
| 6 | -3.00397999 | -3.64775230 | 1.35082229 |
| 1 | -0.59775122 | -4.11890573 | 2.69332019 |
| 1 | 0.30658612 | -3.79162885 | -0.22257036 |
| 1 | -1.51297605 | -5.95420756 | 0.98076560 |
| 1 | -2.37578314 | -3.52893025 | -0.71098052 |
| 1 | 0.83387862 | -3.21330083 | 2.17388276 |
| 1 | 0.78065338 | -5.15499228 | 0.79539836 |
| 1 | -1.10567424 | -5.73658312 | -0.72117698 |
| 1 | -3.37047002 | -4.93600475 | -0.31048297 |
| 1 | -4.07057853 | -3.39200807 | 1.27997422 |
| 1 | -2.89311218 | -4.36167015 | 2.18971326 |
| 8 | -2.39252591 | -2.39136978 | 1.69460015 |

Electronic energy = -3523.855060 a.u.
DFT-D3(BJ) dispersion correction = -0.115936 a.u.
Thermal free energy = 0.603956 a.u.
Gibbs free energy = -3523.367041 a.u.
Number of imaginary frequencies = 0.

**Int1 CL isomer/conformer 13**

| Atomic N. | X | Y | Z |
|---|---|---|---|
| 8 | 1.60512647 | 0.13223366 | 2.39094495 |
| 6 | 1.58251949 | -0.39674882 | 3.72235089 |
| 1 | 0.73044647 | 0.07688707 | 4.22435551 |
| 6 | 3.95489878 | -0.34611098 | 1.82007899 |
| 6 | 4.95413045 | -0.58865346 | 0.67968789 |
| 6 | 5.16896162 | -2.06161214 | 0.31688604 |
| 6 | 2.47086133 | -0.53321660 | 1.44164433 |

| | | | |
|---|---|---|---|
| 6 | 3.88211598 | -2.83125066 | -0.00265414 |
| 6 | 2.93239817 | -2.96804921 | 1.19075203 |
| 1 | 4.19605537 | -0.97655848 | 2.69066769 |
| 1 | 4.60712633 | -0.03321230 | -0.20358305 |
| 1 | 5.67807594 | -2.57119220 | 1.15427722 |
| 1 | 3.34063190 | -2.36769660 | -0.84194136 |
| 1 | 4.03716843 | 0.69952759 | 2.15064747 |
| 1 | 5.92243398 | -0.15179959 | 0.97131899 |
| 1 | 5.85634165 | -2.12193258 | -0.54182447 |
| 1 | 4.15101100 | -3.85161445 | -0.32202155 |
| 1 | 2.26602885 | -3.82912506 | 1.04220654 |
| 1 | 3.51646234 | -3.16067844 | 2.11135517 |
| 8 | 2.22115748 | 0.09516146 | 0.23833930 |
| 8 | 2.01260723 | -1.88599183 | 1.41229634 |
| 1 | 1.43828607 | -1.48592076 | 3.71283762 |
| 1 | 2.50680417 | -0.14554141 | 4.26919653 |
| 7 | 0.22208213 | 2.58701067 | 0.25348363 |
| 8 | -1.12107811 | -0.09533246 | 0.50000357 |
| 8 | 0.45230717 | 0.60730244 | -1.99699289 |
| 6 | 1.10461914 | 3.11065846 | 1.32476854 |
| 6 | -1.72380824 | 0.46600809 | 1.57995603 |
| 6 | -2.43424863 | -0.34907462 | 2.50862880 |
| 6 | -2.97723267 | 0.28939624 | 3.63403872 |
| 6 | -2.87772081 | 1.66677459 | 3.84973652 |
| 6 | -2.26053216 | 2.46104051 | 2.88875136 |
| 6 | -1.70492012 | 1.87672876 | 1.74554912 |
| 6 | -1.22855257 | 2.75586648 | 0.61872198 |
| 1 | -3.30797234 | 2.11288528 | 4.74639962 |
| 6 | -2.64952697 | -1.85771510 | 2.27078558 |
| 6 | -0.26886007 | 1.52776731 | -2.67597790 |
| 6 | -0.96539186 | 1.16128888 | -3.86547472 |
| 6 | -1.68345792 | 2.16816570 | -4.52553631 |
| 6 | -1.73696764 | 3.48853297 | -4.06789822 |
| 6 | -1.04055322 | 3.83766006 | -2.91655504 |
| 6 | -0.29730817 | 2.87373925 | -2.22470274 |
| 6 | 0.54078564 | 3.29371509 | -1.04637638 |
| 1 | -2.31601988 | 4.23352048 | -4.61345581 |
| 6 | -0.93085995 | -0.28130848 | -4.40675462 |
| 6 | -3.53961780 | -2.48773048 | 3.35902333 |
| 6 | -1.30475668 | -2.61638694 | 2.27788951 |
| 6 | -3.36444196 | -2.06883229 | 0.91413896 |
| 6 | -1.72463394 | -0.42234269 | -5.71927387 |
| 6 | -1.56266772 | -1.25106949 | -3.38035308 |
| 6 | 0.52860148 | -0.70160390 | -4.70373605 |
| 1 | 0.95313801 | 4.19717251 | 1.43133637 |
| 1 | 0.87217658 | 2.60643154 | 2.26727810 |
| 1 | -1.40132871 | 3.81685990 | 0.86602479 |
| 1 | -1.79275053 | 2.52810850 | -0.29785216 |
| 1 | -3.50558519 | -0.30868858 | 4.37417430 |
| 1 | -2.22884286 | 3.54708975 | 3.00320573 |
| 1 | 1.60689476 | 3.08816668 | -1.23424278 |
| 1 | 0.42837973 | 4.37784862 | -0.87843476 |
| 1 | -2.23109712 | 1.91709168 | -5.43216053 |

| | | | |
|---|---|---|---|
| 1 | -1.05508712 | 4.86678243 | -2.55023084 |
| 1 | -3.68415536 | -3.55262278 | 3.12717343 |
| 1 | -3.08087780 | -2.42817733 | 4.35681866 |
| 1 | -4.53404817 | -2.02051083 | 3.40374393 |
| 1 | -0.60934810 | -2.23070274 | 1.52618337 |
| 1 | -0.82401774 | -2.53801161 | 3.26393905 |
| 1 | -1.48222336 | -3.68408121 | 2.07507744 |
| 1 | -4.34322270 | -1.56718929 | 0.90952882 |
| 1 | -2.76975100 | -1.67951930 | 0.08061256 |
| 1 | -3.53400249 | -3.14364429 | 0.74874945 |
| 1 | -1.65647293 | -1.46301766 | -6.06688156 |
| 1 | -1.32324649 | 0.21977094 | -6.51674281 |
| 1 | -2.79097128 | -0.18848834 | -5.58678937 |
| 1 | -1.01953818 | -1.24617322 | -2.42961864 |
| 1 | -2.60952654 | -0.97905618 | -3.18222661 |
| 1 | -1.54894379 | -2.27606839 | -3.78127017 |
| 1 | 0.97212654 | -0.04478555 | -5.46648983 |
| 1 | 1.15030507 | -0.65951521 | -3.80293259 |
| 1 | 0.54568501 | -1.73080375 | -5.09376567 |
| 1 | 2.14926972 | 2.90518125 | 1.06413816 |
| 31 | 0.48814641 | 0.58698349 | -0.13495243 |

Electronic energy = -3523.853985 a.u.
DFT-D3(BJ) dispersion correction = -0.117125 a.u.
Thermal free energy = 0.604329 a.u.
Gibbs free energy = -3523.366781 a.u.
Number of imaginary frequencies = 0.

**TS2 ax CL isomer/conformer 1**

| Atomic N. | X | Y | Z |
|---|---|---|---|
| 7 | 0.43888342 | 1.26283753 | 1.76420379 |
| 8 | -1.46183133 | 0.45424384 | -0.29534975 |
| 8 | 1.69115794 | 0.19097669 | -0.71427482 |
| 6 | -0.06540416 | 0.91635734 | 3.11389852 |
| 6 | -2.28599238 | 1.31777287 | 0.32951695 |
| 6 | -3.69692588 | 1.23109591 | 0.12470882 |
| 6 | -4.50253105 | 2.15249181 | 0.80880499 |
| 6 | -3.98320389 | 3.13458657 | 1.65849996 |
| 6 | -2.60589957 | 3.22350168 | 1.83075511 |
| 6 | -1.75763464 | 2.33339930 | 1.16406488 |
| 6 | -0.26929909 | 2.49210739 | 1.25883472 |
| 1 | -4.65324068 | 3.82515478 | 2.17065120 |
| 6 | -4.31205463 | 0.17974553 | -0.81929135 |
| 6 | 2.36470079 | 1.36244631 | -0.67262620 |
| 6 | 2.94810915 | 1.89142597 | -1.86468244 |
| 6 | 3.67330456 | 3.08594942 | -1.75292444 |
| 6 | 3.82906914 | 3.77060390 | -0.54447168 |
| 6 | 3.23766327 | 3.25868011 | 0.60425508 |
| 6 | 2.51517940 | 2.06091595 | 0.55325538 |
| 6 | 1.92415071 | 1.51206601 | 1.82491183 |
| 1 | 4.39799404 | 4.69959784 | -0.51013607 |
| 6 | 2.78303218 | 1.19255674 | -3.22922492 |
| 6 | -5.84590960 | 0.29844248 | -0.89177084 |

| | | | |
|---|---|---|---|
| 6 | -3.97905946 | -1.24762046 | -0.32481655 |
| 6 | -3.76319838 | 0.37569508 | -2.25284886 |
| 6 | 3.48476195 | 1.96502936 | -4.36244059 |
| 6 | 1.28393519 | 1.09599066 | -3.59979892 |
| 6 | 3.40712237 | -0.22130205 | -3.18287182 |
| 1 | 0.11209720 | 1.75460565 | 3.80864763 |
| 1 | -1.14201450 | 0.72156972 | 3.06468606 |
| 1 | -0.01666437 | 3.33372641 | 1.92686057 |
| 1 | 0.16213734 | 2.71180630 | 0.27200067 |
| 1 | -5.58238554 | 2.10625410 | 0.67896819 |
| 1 | -2.17422271 | 3.99630308 | 2.47129822 |
| 1 | 2.38542295 | 0.55010837 | 2.09501672 |
| 1 | 2.11949730 | 2.21842980 | 2.65010142 |
| 1 | 4.13044262 | 3.51020122 | -2.64484215 |
| 1 | 3.33510613 | 3.78261280 | 1.55790043 |
| 1 | -6.23000860 | -0.46398550 | -1.58443320 |
| 1 | -6.32195330 | 0.12749393 | 0.08496714 |
| 1 | -6.16821384 | 1.27990162 | -1.26873863 |
| 1 | -2.89925098 | -1.43080807 | -0.33291173 |
| 1 | -4.36336899 | -1.40409350 | 0.69460487 |
| 1 | -4.45910358 | -1.98870432 | -0.98247629 |
| 1 | -4.02526903 | 1.37342262 | -2.63439870 |
| 1 | -2.67393899 | 0.26362780 | -2.28183222 |
| 1 | -4.20836639 | -0.37215513 | -2.92686057 |
| 1 | 3.33326674 | 1.42487442 | -5.30782318 |
| 1 | 4.56919670 | 2.04752040 | -4.19941187 |
| 1 | 3.07343125 | 2.97661495 | -4.49083948 |
| 1 | 0.72052020 | 0.50774211 | -2.86803031 |
| 1 | 0.83509147 | 2.09800792 | -3.66059852 |
| 1 | 1.17507207 | 0.61706197 | -4.58474112 |
| 1 | 4.48223209 | -0.16516154 | -2.95726585 |
| 1 | 2.92018247 | -0.83949167 | -2.42213988 |
| 1 | 3.29111052 | -0.71295786 | -4.16085911 |
| 1 | 0.44615713 | 0.02372686 | 3.48547864 |
| 8 | 1.11449075 | -3.74006104 | -0.02571276 |
| 6 | 1.99867415 | -3.24248528 | -1.05655169 |
| 1 | 2.52586293 | -2.34029818 | -0.71799719 |
| 6 | -0.95419955 | -3.66033792 | 1.14965761 |
| 6 | -0.44079009 | -4.61874628 | 2.22974873 |
| 6 | 0.38207892 | -3.99107885 | 3.36932635 |
| 6 | 0.09357273 | -2.90861034 | 0.34155837 |
| 6 | 1.78817165 | -3.52087426 | 2.95390439 |
| 6 | 1.84775066 | -2.09262443 | 2.39415979 |
| 1 | -1.62191319 | -2.90303731 | 1.58222032 |
| 1 | -1.32081115 | -5.11577034 | 2.66785073 |
| 1 | 0.49239376 | -4.75349426 | 4.15607691 |
| 1 | 2.46394086 | -3.54248452 | 3.82409692 |
| 1 | -1.55006421 | -4.22339249 | 0.41217768 |
| 1 | 0.15754130 | -5.41317654 | 1.75681806 |
| 1 | -0.17937352 | -3.15794563 | 3.82475376 |
| 1 | 2.19875026 | -4.22909927 | 2.22127080 |
| 1 | 2.79081583 | -1.94056439 | 1.83909571 |
| 1 | 1.84825337 | -1.38521719 | 3.24182034 |

| | | | |
|---|---|---|---|
| 8 | -0.29905826 | -1.99128151 | -0.49972481 |
| 8 | 0.71825927 | -1.77011716 | 1.57163429 |
| 1 | 2.70970917 | -4.05484819 | -1.23851454 |
| 1 | 1.43523479 | -3.00724840 | -1.96686935 |
| 31 | 0.17762545 | -0.26020846 | 0.29803994 |

Electronic energy = -3523.848507 a.u.
DFT-D3(BJ) dispersion correction = -0.119163 a.u.
Thermal free energy = 0.605594 a.u.
Gibbs free energy = -3523.362077 a.u.
Number of imaginary frequencies = 1.

**TS2 ax CL isomer/conformer 2**

| Atomic N. | X | Y | Z |
|---|---|---|---|
| 7 | -0.19306231 | 0.66206223 | 2.25319314 |
| 8 | -1.27142894 | 0.95960766 | -0.44842264 |
| 8 | 1.63899982 | -0.12667958 | 0.00001808 |
| 6 | -1.10327005 | 0.05937781 | 3.25588870 |
| 6 | -2.19264603 | 1.74760520 | 0.13934644 |
| 6 | -3.40093136 | 2.08969617 | -0.54173428 |
| 6 | -4.32476902 | 2.88504195 | 0.15226233 |
| 6 | -4.10042381 | 3.36260319 | 1.44750226 |
| 6 | -2.89481258 | 3.07128334 | 2.07751298 |
| 6 | -1.93876696 | 2.28605914 | 1.42583334 |
| 6 | -0.57485563 | 2.09847713 | 2.01683640 |
| 1 | -4.85485888 | 3.97275305 | 1.94416678 |
| 6 | -3.67253637 | 1.63328528 | -1.98822749 |
| 6 | 2.41521621 | 0.85275859 | 0.51456285 |
| 6 | 3.43541694 | 1.44601476 | -0.28927636 |
| 6 | 4.22528505 | 2.44201875 | 0.30118167 |
| 6 | 4.04784250 | 2.86848402 | 1.62051094 |
| 6 | 3.05200171 | 2.28349566 | 2.39369941 |
| 6 | 2.24228477 | 1.27376425 | 1.85947037 |
| 6 | 1.22871006 | 0.60512620 | 2.75338364 |
| 1 | 4.68250418 | 3.65267920 | 2.03300261 |
| 6 | 3.66549349 | 1.01283360 | -1.75110590 |
| 6 | -5.00635862 | 2.18378305 | -2.52668095 |
| 6 | -3.74082470 | 0.09107007 | -2.06266570 |
| 6 | -2.54912162 | 2.15081096 | -2.91825223 |
| 6 | 4.82206154 | 1.79062676 | -2.40734076 |
| 6 | 2.39646649 | 1.27806759 | -2.59540319 |
| 6 | 4.03438377 | -0.48862898 | -1.80497813 |
| 1 | -1.00118065 | 0.58264238 | 4.22157907 |
| 1 | -2.13828921 | 0.15726823 | 2.90934730 |
| 1 | -0.49475679 | 2.63440919 | 2.97888112 |
| 1 | 0.19107862 | 2.50628304 | 1.34187663 |
| 1 | -5.26197386 | 3.14855933 | -0.33496296 |
| 1 | -2.67460608 | 3.47050524 | 3.07058907 |
| 1 | 1.46619213 | -0.45884880 | 2.88913465 |
| 1 | 1.25490355 | 1.08060122 | 3.74921560 |
| 1 | 5.00809336 | 2.91402268 | -0.28968275 |
| 1 | 2.89737964 | 2.59974265 | 3.42793655 |
| 1 | -5.13810635 | 1.84579146 | -3.56452560 |

| | | | |
|---|---|---|---|
| 1 | -5.86913586 | 1.82025075 | -1.94935524 |
| 1 | -5.02900934 | 3.28318167 | -2.53091884 |
| 1 | -2.79365253 | -0.36496788 | -1.75563526 |
| 1 | -4.54858398 | -0.28961650 | -1.41879761 |
| 1 | -3.95829415 | -0.22209144 | -3.09570742 |
| 1 | -2.51228833 | 3.24999261 | -2.90529180 |
| 1 | -1.56988943 | 1.76396549 | -2.61550570 |
| 1 | -2.74599814 | 1.83086050 | -3.95292425 |
| 1 | 4.94561386 | 1.43949211 | -3.44171786 |
| 1 | 5.77782297 | 1.63030803 | -1.88750660 |
| 1 | 4.62393093 | 2.87146473 | -2.44739223 |
| 1 | 1.53604937 | 0.71007431 | -2.22716761 |
| 1 | 2.13853598 | 2.34686923 | -2.58106327 |
| 1 | 2.57985640 | 0.98861802 | -3.64137793 |
| 1 | 4.96072102 | -0.68018287 | -1.24348044 |
| 1 | 3.23562098 | -1.10811627 | -1.38334346 |
| 1 | 4.20149803 | -0.79790789 | -2.84735203 |
| 1 | -0.86577094 | -1.00098562 | 3.38674784 |
| 8 | -1.51033103 | -3.75612617 | -0.29457134 |
| 6 | -2.83105993 | -3.22727060 | -0.04389991 |
| 1 | -2.83486795 | -2.64193344 | 0.88572431 |
| 6 | 0.75360042 | -3.49833941 | -0.97000414 |
| 6 | 1.06376708 | -4.91076803 | -0.46417820 |
| 6 | 1.66313505 | -4.98545933 | 0.94479817 |
| 6 | -0.52044916 | -2.83999395 | -0.46245295 |
| 6 | 0.82930523 | -4.35477638 | 2.06995797 |
| 6 | 0.82182276 | -2.82235551 | 2.09293032 |
| 1 | 1.58424807 | -2.80211568 | -0.78581434 |
| 1 | 1.77915335 | -5.37051630 | -1.16386139 |
| 1 | 1.83715487 | -6.04603958 | 1.18771577 |
| 1 | 1.25631166 | -4.67869282 | 3.03333330 |
| 1 | 0.61368281 | -3.52241468 | -2.06360221 |
| 1 | 0.14937736 | -5.51990175 | -0.51385379 |
| 1 | 2.65813494 | -4.50709248 | 0.94130969 |
| 1 | -0.20791107 | -4.72475100 | 2.04813123 |
| 1 | 0.58665544 | -2.49552584 | 3.12186623 |
| 1 | 1.83373189 | -2.44753003 | 1.84767294 |
| 8 | -0.83643705 | -1.65939415 | -0.91901124 |
| 8 | -0.15227421 | -2.22609425 | 1.22611403 |
| 1 | -3.47439599 | -4.10631418 | 0.06389315 |
| 1 | -3.16563439 | -2.60192299 | -0.87900847 |
| 31 | -0.18048100 | -0.35093361 | 0.38092729 |

Electronic energy = -3523.848602 a.u.
DFT-D3(BJ) dispersion correction = -0.118652 a.u.
Thermal free energy = 0.605425 a.u.
Gibbs free energy = -3523.361829 a.u.
Number of imaginary frequencies = 1.

**TS2 ax CL isomer/conformer 3**

| Atomic N. | X | Y | Z |
|---|---|---|---|
| 8 | -3.81689620 | 0.80182368 | -1.66348100 |
| 6 | -5.16599655 | 0.71307135 | -1.17679691 |

| | | | |
|---|---|---|---|
| 1 | -5.58607101 | 1.71864080 | -1.29123533 |
| 6 | -3.57335448 | -1.65132987 | -1.64074731 |
| 6 | -2.68285680 | -2.83177805 | -1.22882164 |
| 6 | -2.83787203 | -3.29861832 | 0.22824214 |
| 6 | -2.96332693 | -0.26790750 | -1.48270369 |
| 6 | -2.25812006 | -2.38484812 | 1.31627595 |
| 6 | -2.88375902 | -0.99735165 | 1.42589498 |
| 1 | -4.53845406 | -1.70873523 | -1.11813557 |
| 1 | -1.63398814 | -2.58629251 | -1.44803739 |
| 1 | -3.90910196 | -3.47103763 | 0.43723238 |
| 1 | -1.16977692 | -2.26669574 | 1.17540884 |
| 1 | -3.80250311 | -1.71593451 | -2.71903491 |
| 1 | -2.93646359 | -3.68258476 | -1.87919188 |
| 1 | -2.35336566 | -4.28371572 | 0.32026637 |
| 1 | -2.38242149 | -2.88020325 | 2.29428577 |
| 1 | -2.53562784 | -0.50151247 | 2.34524298 |
| 1 | -3.98454571 | -1.07500434 | 1.48207486 |
| 8 | -1.76658916 | -0.02503387 | -1.87372386 |
| 8 | -2.55941677 | -0.12441390 | 0.33464357 |
| 1 | -5.18065357 | 0.43481115 | -0.11340380 |
| 1 | -5.76836109 | 0.00575995 | -1.76450598 |
| 7 | -0.53911960 | 2.50052881 | -0.50195009 |
| 8 | 0.89671123 | -0.14782663 | -0.84145343 |
| 8 | -0.27906168 | 0.51950020 | 1.70745504 |
| 6 | -1.45868456 | 3.05380893 | -1.52587795 |
| 6 | 1.38642740 | 0.42991188 | -1.96509337 |
| 6 | 2.03736377 | -0.34745669 | -2.96859813 |
| 6 | 2.47312832 | 0.32343966 | -4.12100792 |
| 6 | 2.32445288 | 1.70106888 | -4.30219603 |
| 6 | 1.76200676 | 2.46119404 | -3.28190517 |
| 6 | 1.31339109 | 1.84237838 | -2.11106157 |
| 6 | 0.89458853 | 2.68352222 | -0.93560821 |
| 1 | 2.67124319 | 2.17304373 | -5.22152185 |
| 6 | 2.29959512 | -1.85513985 | -2.78549981 |
| 6 | 0.39240178 | 1.51125634 | 2.31570458 |
| 6 | 1.28760433 | 1.22894835 | 3.39542079 |
| 6 | 1.93269551 | 2.31421208 | 4.00337839 |
| 6 | 1.74325228 | 3.63843989 | 3.59593678 |
| 6 | 0.87516254 | 3.90382099 | 2.54329038 |
| 6 | 0.19303158 | 2.85878420 | 1.90936542 |
| 6 | -0.79505581 | 3.18662620 | 0.82261533 |
| 1 | 2.27449322 | 4.44775438 | 4.09663010 |
| 6 | 1.54375625 | -0.21364117 | 3.87384987 |
| 6 | 3.06147647 | -2.46127772 | -3.97890186 |
| 6 | 0.97004592 | -2.62667942 | -2.64599347 |
| 6 | 3.16469455 | -2.07622266 | -1.52143478 |
| 6 | 2.53335500 | -0.26686648 | 5.05312729 |
| 6 | 2.15208507 | -1.05150926 | 2.72484350 |
| 6 | 0.22259711 | -0.85669661 | 4.35703802 |
| 1 | -1.26143742 | 4.13120651 | -1.65014374 |
| 1 | -1.30275166 | 2.53287888 | -2.47510004 |
| 1 | 1.04827213 | 3.75276637 | -1.15812147 |
| 1 | 1.50668526 | 2.42992640 | -0.05880419 |

| | | | |
|---|---|---|---|
| 1 | 2.95133758 | -0.24861972 | -4.91414213 |
| 1 | 1.69003332 | 3.54758978 | -3.37394428 |
| 1 | -1.81764352 | 2.89189935 | 1.11031723 |
| 1 | -0.79476905 | 4.27491665 | 0.64502698 |
| 1 | 2.62054157 | 2.12525392 | 4.82572699 |
| 1 | 0.71078956 | 4.93018484 | 2.20651865 |
| 1 | 3.23686910 | -3.52917814 | -3.78451443 |
| 1 | 2.49126291 | -2.38513160 | -4.91626740 |
| 1 | 4.04205608 | -1.98694921 | -4.12852144 |
| 1 | 0.41544339 | -2.28119469 | -1.76835108 |
| 1 | 0.33820623 | -2.48281193 | -3.53427505 |
| 1 | 1.17098463 | -3.70366359 | -2.53616905 |
| 1 | 4.13590527 | -1.57057858 | -1.62373316 |
| 1 | 2.66647744 | -1.69297242 | -0.62378496 |
| 1 | 3.35613370 | -3.15150857 | -1.38269198 |
| 1 | 2.67520046 | -1.31526732 | 5.35255051 |
| 1 | 2.16215324 | 0.28118700 | 5.93138170 |
| 1 | 3.52049255 | 0.13524549 | 4.78328657 |
| 1 | 1.49465728 | -1.07642663 | 1.84953821 |
| 1 | 3.11999035 | -0.63289458 | 2.41324377 |
| 1 | 2.32443380 | -2.08399391 | 3.06608701 |
| 1 | -0.19841567 | -0.29122943 | 5.20141745 |
| 1 | -0.51979136 | -0.88600481 | 3.55264330 |
| 1 | 0.41061896 | -1.88597238 | 4.69930935 |
| 1 | -2.49532604 | 2.89628839 | -1.21037757 |
| 31 | -0.74252647 | 0.47902134 | -0.14840582 |

Electronic energy = -3523.842065 a.u.
DFT-D3(BJ) dispersion correction = -0.120424 a.u.
Thermal free energy = 0.604484 a.u.
Gibbs free energy = -3523.358005 a.u.
Number of imaginary frequencies = 1.

**TS2 ax CL isomer/conformer 4**

| Atomic N. | X | Y | Z |
|---|---|---|---|
| 8 | -0.78488290 | -3.44144368 | -0.46767151 |
| 6 | -1.02759898 | -4.65366268 | 0.26138756 |
| 1 | -2.10420609 | -4.83605766 | 0.17540273 |
| 1 | -0.76387805 | -4.52880049 | 1.32194090 |
| 1 | -0.48314977 | -5.50711060 | -0.16874465 |
| 6 | 0.46833187 | -2.87007666 | -0.40466392 |
| 8 | 0.53389561 | -1.74113917 | -1.02934277 |
| 7 | -0.72491705 | 0.52767533 | 2.11042380 |
| 8 | -1.51782358 | 0.09037153 | -0.67467529 |
| 8 | 1.52673376 | 0.64652127 | 0.18845563 |
| 6 | -1.50338674 | -0.36638743 | 2.99938059 |
| 6 | -2.71274376 | 0.56282330 | -0.26670673 |
| 6 | -3.87369990 | 0.35227853 | -1.07364750 |
| 6 | -5.09379816 | 0.83776611 | -0.58231449 |
| 6 | -5.21024799 | 1.51901388 | 0.63335502 |
| 6 | -4.06639862 | 1.75761592 | 1.38674831 |
| 6 | -2.82233667 | 1.30144525 | 0.93771082 |
| 6 | -1.57638335 | 1.68938029 | 1.67645800 |

| | | | |
|---|---|---|---|
| 1 | -6.18430185 | 1.87085032 | 0.97335923 |
| 6 | -3.79605174 | -0.36024284 | -2.43813682 |
| 6 | 1.77790844 | 1.82400715 | 0.79837620 |
| 6 | 2.58118415 | 2.80168247 | 0.13613027 |
| 6 | 2.83768439 | 3.99692297 | 0.82156771 |
| 6 | 2.33782601 | 4.26074314 | 2.10034752 |
| 6 | 1.55326891 | 3.30326581 | 2.73242068 |
| 6 | 1.27764165 | 2.08518767 | 2.09965873 |
| 6 | 0.48609000 | 1.04052079 | 2.84236813 |
| 1 | 2.55845547 | 5.20918226 | 2.58994055 |
| 6 | 3.14326715 | 2.55922437 | -1.27896345 |
| 6 | -5.16232014 | -0.39434004 | -3.14891481 |
| 6 | -3.33138776 | -1.82226193 | -2.25150061 |
| 6 | -2.81197691 | 0.38721859 | -3.36978126 |
| 6 | 3.98332906 | 3.75130844 | -1.77540874 |
| 6 | 1.98563457 | 2.36075497 | -2.28626728 |
| 6 | 4.06455564 | 1.31596982 | -1.28246224 |
| 1 | -1.84269905 | 0.18918064 | 3.89000559 |
| 1 | -2.38016725 | -0.74340373 | 2.46205091 |
| 1 | -1.83577287 | 2.27899814 | 2.57317567 |
| 1 | -0.93325561 | 2.31926322 | 1.04464352 |
| 1 | -5.99658632 | 0.68118501 | -1.16997063 |
| 1 | -4.12347317 | 2.31955361 | 2.32210469 |
| 1 | 1.10856056 | 0.16089402 | 3.06543541 |
| 1 | 0.15055931 | 1.45971847 | 3.80697536 |
| 1 | 3.44558001 | 4.76095343 | 0.34050122 |
| 1 | 1.15051222 | 3.48890686 | 3.73086381 |
| 1 | -5.04427528 | -0.89390665 | -4.12112951 |
| 1 | -5.91410303 | -0.95888764 | -2.57801557 |
| 1 | -5.55581284 | 0.61465573 | -3.34028316 |
| 1 | -2.33752823 | -1.87546456 | -1.79745936 |
| 1 | -4.04002142 | -2.37371516 | -1.61486065 |
| 1 | -3.29274821 | -2.32707429 | -3.22937036 |
| 1 | -3.14713883 | 1.42140293 | -3.53766894 |
| 1 | -1.80105698 | 0.40935680 | -2.94929361 |
| 1 | -2.77148557 | -0.11806363 | -4.34685373 |
| 1 | 4.36146355 | 3.52554059 | -2.78260517 |
| 1 | 4.85348845 | 3.94267845 | -1.13062239 |
| 1 | 3.39120293 | 4.67527676 | -1.84496880 |
| 1 | 1.37363577 | 1.48802698 | -2.03605461 |
| 1 | 1.33647346 | 3.24809957 | -2.30961752 |
| 1 | 2.39486575 | 2.21484852 | -3.29745984 |
| 1 | 4.91043711 | 1.45910347 | -0.59379995 |
| 1 | 3.51730776 | 0.41467884 | -0.98647052 |
| 1 | 4.47310114 | 1.15973353 | -2.29247403 |
| 1 | -0.88303101 | -1.21266806 | 3.31046152 |
| 31 | -0.00992724 | -0.41285449 | 0.33157569 |
| 6 | 1.65060782 | -3.82534122 | -0.48848584 |
| 6 | 3.03228259 | -3.16028571 | -0.43161479 |
| 6 | 3.61194968 | -3.00604630 | 0.98033988 |
| 6 | 2.85717297 | -2.05875802 | 1.92155433 |
| 6 | 1.42592919 | -2.47800088 | 2.27113223 |
| 1 | 1.56787956 | -4.61828375 | 0.26886022 |

| | | | |
|---|---|---|---|
| 1 | 2.97514176 | -2.18328547 | -0.93357933 |
| 1 | 3.67568779 | -4.00296164 | 1.45278883 |
| 1 | 2.83845139 | -1.04087639 | 1.50123358 |
| 1 | 1.51612186 | -4.31888199 | -1.46637213 |
| 1 | 3.73030853 | -3.77553654 | -1.01928985 |
| 1 | 4.64954042 | -2.64732718 | 0.89074087 |
| 1 | 3.41631699 | -1.99936163 | 2.87101507 |
| 1 | 1.11871290 | -1.97816181 | 3.20656204 |
| 1 | 1.39067805 | -3.56299400 | 2.48142457 |
| 8 | 0.42079821 | -2.19012213 | 1.28864515 |

Electronic energy = -3523.841276 a.u.
DFT-D3(BJ) dispersion correction = -0.119541 a.u.
Thermal free energy = 0.605044 a.u.
Gibbs free energy = -3523.355772 a.u.
Number of imaginary frequencies = 1.

**TS2 ax CL isomer/conformer 5**

| Atomic N. | X | Y | Z |
|---|---|---|---|
| 8 | -2.36827254 | -0.99985397 | -1.47837210 |
| 6 | -3.66346097 | -0.60144657 | -1.95686305 |
| 1 | -3.48508263 | -0.15871508 | -2.94260907 |
| 6 | -3.33638072 | -2.58643031 | 0.19028452 |
| 6 | -4.66933775 | -2.09172344 | 0.78494984 |
| 6 | -4.62393618 | -1.66212428 | 2.25944805 |
| 6 | -2.24932361 | -1.61506104 | -0.25071940 |
| 6 | -4.02794647 | -0.27930218 | 2.54832697 |
| 6 | -2.55977702 | -0.10733827 | 2.17034101 |
| 1 | -2.84858418 | -3.25987768 | 0.90826505 |
| 1 | -5.10056019 | -1.28235054 | 0.17993665 |
| 1 | -5.65541172 | -1.67846358 | 2.64712214 |
| 1 | -4.61565781 | 0.50213754 | 2.03748417 |
| 1 | -3.54903841 | -3.19437313 | -0.70606124 |
| 1 | -5.37050629 | -2.93757105 | 0.70292920 |
| 1 | -4.07097816 | -2.42235231 | 2.83775735 |
| 1 | -4.11977243 | -0.08294398 | 3.62968969 |
| 1 | -2.14985919 | 0.77383620 | 2.69208431 |
| 1 | -1.96398485 | -0.97278947 | 2.51830506 |
| 8 | -1.04051995 | -1.82880497 | 0.09359593 |
| 8 | -2.39364982 | 0.07711431 | 0.75617510 |
| 1 | -4.10714340 | 0.14458673 | -1.28345048 |
| 1 | -4.33434534 | -1.46552002 | -2.06517696 |
| 7 | -0.51736397 | 2.23400593 | -0.10821656 |
| 8 | 0.76344740 | -0.14105824 | -1.26730537 |
| 8 | 0.50209576 | 0.11660328 | 1.79324806 |
| 6 | -1.78789806 | 2.70417404 | -0.70915622 |
| 6 | 0.81603736 | 0.59738606 | -2.39413118 |
| 6 | 1.08183622 | -0.01752992 | -3.65557981 |
| 6 | 1.07068861 | 0.81233370 | -4.78667974 |
| 6 | 0.84676892 | 2.19125962 | -4.71983147 |
| 6 | 0.66579020 | 2.78922701 | -3.47649384 |
| 6 | 0.66942686 | 2.00637841 | -2.31794786 |
| 6 | 0.64578885 | 2.64975095 | -0.96225417 |

| | | | |
|---|---|---|---|
| 1 | 0.84298033 | 2.78973150 | -5.63095999 |
| 6 | 1.41945434 | -1.51720738 | -3.77314186 |
| 6 | 1.27353323 | 1.13223636 | 2.23540878 |
| 6 | 2.45294571 | 0.84389168 | 2.98711061 |
| 6 | 3.20976710 | 1.93122613 | 3.44393921 |
| 6 | 2.85919189 | 3.26025915 | 3.18806386 |
| 6 | 1.70853400 | 3.52890038 | 2.45635867 |
| 6 | 0.90574926 | 2.48143649 | 1.98744524 |
| 6 | -0.37956873 | 2.81731081 | 1.27409458 |
| 1 | 3.48461413 | 4.07287931 | 3.55753994 |
| 6 | 2.88433290 | -0.60661387 | 3.28255916 |
| 6 | 1.74239576 | -1.92316818 | -5.22354221 |
| 6 | 0.23622534 | -2.39008498 | -3.29839563 |
| 6 | 2.66985798 | -1.83070517 | -2.91607428 |
| 6 | 4.17417383 | -0.66255754 | 4.12303209 |
| 6 | 3.16433167 | -1.35899925 | 1.95980084 |
| 6 | 1.78257787 | -1.33986831 | 4.08452177 |
| 1 | -1.79433703 | 3.80623221 | -0.76075965 |
| 1 | -1.87821209 | 2.30197287 | -1.72474504 |
| 1 | 0.62924361 | 3.74972343 | -1.05799794 |
| 1 | 1.54884529 | 2.37530875 | -0.39803156 |
| 1 | 1.24919903 | 0.36991298 | -5.76526928 |
| 1 | 0.54544091 | 3.87204123 | -3.39170837 |
| 1 | -1.25417209 | 2.45756698 | 1.83721721 |
| 1 | -0.47014010 | 3.91442060 | 1.19083154 |
| 1 | 4.11534166 | 1.74049234 | 4.01696205 |
| 1 | 1.41383219 | 4.56012106 | 2.24812293 |
| 1 | 2.00008392 | -2.99171686 | -5.24495125 |
| 1 | 0.88406122 | -1.77742040 | -5.89600039 |
| 1 | 2.60022449 | -1.36746311 | -5.62920904 |
| 1 | 0.00532185 | -2.20989227 | -2.24425650 |
| 1 | -0.66360265 | -2.18441081 | -3.89718747 |
| 1 | 0.48978442 | -3.45435023 | -3.42353344 |
| 1 | 3.53565454 | -1.24932384 | -3.26561236 |
| 1 | 2.49674320 | -1.60057521 | -1.85889494 |
| 1 | 2.92171979 | -2.89879918 | -3.00261879 |
| 1 | 4.43195057 | -1.71476078 | 4.30998278 |
| 1 | 4.05391264 | -0.17258634 | 5.10030699 |
| 1 | 5.02724981 | -0.20139466 | 3.60453224 |
| 1 | 2.27343440 | -1.41268778 | 1.32581949 |
| 1 | 3.96465898 | -0.86125606 | 1.39322960 |
| 1 | 3.49392128 | -2.38583231 | 2.18018985 |
| 1 | 1.60275614 | -0.83519709 | 5.04538584 |
| 1 | 0.84113997 | -1.37900674 | 3.52636957 |
| 1 | 2.10428786 | -2.37043500 | 4.29928398 |
| 1 | -2.62581038 | 2.34948874 | -0.09995395 |
| 31 | -0.42672044 | 0.12799791 | 0.17564006 |

Electronic energy = -3523.836780 a.u.
DFT-D3(BJ) dispersion correction = -0.119372 a.u.
Thermal free energy = 0.605645 a.u.
Gibbs free energy = -3523.350507 a.u.
Number of imaginary frequencies = 1.

**Int2_ax_CL isomer/conformer 1**

| Atomic N. | X | Y | Z |
|---|---|---|---|
| 7 | 0.31238204 | 1.83578725 | 1.93099137 |
| 8 | -1.50453761 | 0.28829786 | 0.25588936 |
| 8 | 1.75463209 | 0.36212419 | -0.18769643 |
| 6 | -0.27532473 | 1.71361429 | 3.28525722 |
| 6 | -2.34959826 | 1.31602944 | 0.49232415 |
| 6 | -3.74647872 | 1.12947101 | 0.24455501 |
| 6 | -4.60326860 | 2.20892935 | 0.49723910 |
| 6 | -4.14874182 | 3.44000306 | 0.97651169 |
| 6 | -2.78878997 | 3.61412803 | 1.20315112 |
| 6 | -1.88480234 | 2.57493825 | 0.95353193 |
| 6 | -0.41355139 | 2.85222100 | 1.10875044 |
| 1 | -4.85295915 | 4.25005417 | 1.16598408 |
| 6 | -4.30243393 | -0.21011545 | -0.27884111 |
| 6 | 2.44843906 | 1.51237711 | -0.29755753 |
| 6 | 3.19069458 | 1.76589250 | -1.49264977 |
| 6 | 3.91074467 | 2.96557590 | -1.56700611 |
| 6 | 3.92148482 | 3.90756718 | -0.53428513 |
| 6 | 3.19558494 | 3.65076691 | 0.62293948 |
| 6 | 2.47071201 | 2.46073695 | 0.75728121 |
| 6 | 1.76571799 | 2.18428793 | 2.05933801 |
| 1 | 4.49043413 | 4.83114986 | -0.64013891 |
| 6 | 3.20130171 | 0.76007828 | -2.66118079 |
| 6 | -5.82330641 | -0.15633142 | -0.51803837 |
| 6 | -4.04212290 | -1.32196527 | 0.76338679 |
| 6 | -3.64347777 | -0.57593714 | -1.63057064 |
| 6 | 4.08340755 | 1.24084871 | -3.82884112 |
| 6 | 1.76801125 | 0.57305415 | -3.21295308 |
| 6 | 3.76791586 | -0.59983124 | -2.18787268 |
| 1 | -0.18090801 | 2.66897878 | 3.82904919 |
| 1 | -1.33612733 | 1.45294858 | 3.20477120 |
| 1 | -0.27198342 | 3.84953873 | 1.56173689 |
| 1 | 0.08693333 | 2.87093926 | 0.12826542 |
| 1 | -5.67016050 | 2.08910859 | 0.31830226 |
| 1 | -2.40694988 | 4.57115559 | 1.56615040 |
| 1 | 2.22914023 | 1.33612768 | 2.58955732 |
| 1 | 1.85592475 | 3.06945233 | 2.71348353 |
| 1 | 4.48364111 | 3.18328476 | -2.46658120 |
| 1 | 3.19143710 | 4.37113579 | 1.44428252 |
| 1 | -6.15945953 | -1.13234302 | -0.89696071 |
| 1 | -6.38277569 | 0.04873687 | 0.40598216 |
| 1 | -6.09715163 | 0.60149246 | -1.26615517 |
| 1 | -2.97468541 | -1.40963651 | 0.98908872 |
| 1 | -4.57720594 | -1.10532798 | 1.69954790 |
| 1 | -4.40449833 | -2.28972058 | 0.38259957 |
| 1 | -3.87375812 | 0.18699510 | -2.38859261 |
| 1 | -2.55483832 | -0.65312940 | -1.53924106 |
| 1 | -4.04156419 | -1.53767978 | -1.99023349 |
| 1 | 4.05966144 | 0.48671063 | -4.62866421 |
| 1 | 5.13310595 | 1.37127814 | -3.52837514 |
| 1 | 3.72391591 | 2.18750908 | -4.25738597 |

| | | | |
|---|---|---|---|
| 1 | 1.09059827 | 0.17966759 | -2.44766047 |
| 1 | 1.36655471 | 1.52928038 | -3.57894282 |
| 1 | 1.78529549 | -0.12937271 | -4.06131513 |
| 1 | 4.80248463 | -0.48523820 | -1.83250320 |
| 1 | 3.16470100 | -1.02133571 | -1.37638426 |
| 1 | 3.77945297 | -1.31123431 | -3.02876270 |
| 1 | 0.24596616 | 0.91608075 | 3.82573083 |
| 8 | -0.30855416 | -3.75908295 | -1.49699710 |
| 6 | 0.24058783 | -3.22743205 | -2.73311945 |
| 1 | 1.28184695 | -2.91853163 | -2.58329513 |
| 6 | -0.91201533 | -3.55249411 | 0.77820905 |
| 6 | -0.00466767 | -4.64282191 | 1.40784950 |
| 6 | 0.99328840 | -4.12341940 | 2.45447257 |
| 6 | -0.33447291 | -2.90639190 | -0.45446578 |
| 6 | 2.03399275 | -3.09742737 | 1.95072392 |
| 6 | 1.83498406 | -1.68342884 | 2.51366116 |
| 1 | -1.11516915 | -2.76616389 | 1.51355461 |
| 1 | -0.66296146 | -5.37814470 | 1.89471383 |
| 1 | 1.51015703 | -5.00017375 | 2.87471867 |
| 1 | 3.04875847 | -3.41541530 | 2.24273840 |
| 1 | -1.86154200 | -4.01268203 | 0.46807733 |
| 1 | 0.52452688 | -5.18828291 | 0.61131144 |
| 1 | 0.42333617 | -3.67499377 | 3.28410891 |
| 1 | 2.04350270 | -3.05373170 | 0.85033519 |
| 1 | 2.63199137 | -1.03345050 | 2.10448997 |
| 1 | 1.98229071 | -1.72393581 | 3.61039049 |
| 8 | 0.07679564 | -1.75053289 | -0.57603516 |
| 8 | 0.53501648 | -1.14236276 | 2.28556098 |
| 1 | 0.17450269 | -4.05192959 | -3.44855761 |
| 1 | -0.34921132 | -2.36660972 | -3.06926613 |
| 31 | 0.24420000 | 0.00028376 | 0.86653129 |

Electronic energy = -3523.868816 a.u.
DFT-D3(BJ) dispersion correction = -0.118380 a.u.
Thermal free energy = 0.601915 a.u.
Gibbs free energy = -3523.385282 a.u.
Number of imaginary frequencies = 0.

**Int2 ax CL isomer/conformer 2**

| Atomic N. | X | Y | Z |
|---|---|---|---|
| 7 | 0.33956849 | 2.16393834 | 2.01544672 |
| 8 | -1.51744620 | 0.68053871 | 0.26356499 |
| 8 | 1.69288031 | 0.46188764 | -0.00901369 |
| 6 | -0.14667120 | 2.11807505 | 3.41394508 |
| 6 | -2.37448594 | 1.65722671 | 0.63343725 |
| 6 | -3.77769071 | 1.45402154 | 0.45566405 |
| 6 | -4.63584162 | 2.48875872 | 0.85299670 |
| 6 | -4.17358214 | 3.69052449 | 1.39697956 |
| 6 | -2.80448209 | 3.88728761 | 1.53869823 |
| 6 | -1.90148791 | 2.89155060 | 1.14967312 |
| 6 | -0.42415997 | 3.17136989 | 1.21227588 |
| 1 | -4.88045694 | 4.46415552 | 1.69712990 |
| 6 | -4.33336364 | 0.15703502 | -0.16687055 |

| | | | |
|---|---|---|---|
| 6 | 2.34279222 | 1.61109025 | -0.29556139 |
| 6 | 2.98284163 | 1.77003014 | -1.56323845 |
| 6 | 3.66577934 | 2.97167178 | -1.79734561 |
| 6 | 3.73487719 | 4.00113335 | -0.85458499 |
| 6 | 3.10567334 | 3.83659179 | 0.37413427 |
| 6 | 2.42123625 | 2.65133519 | 0.66478469 |
| 6 | 1.80986598 | 2.46867386 | 2.02679668 |
| 1 | 4.27252374 | 4.92073372 | -1.08545731 |
| 6 | 2.92438379 | 0.67071896 | -2.64201373 |
| 6 | -5.86915450 | 0.18622352 | -0.28254044 |
| 6 | -3.96054100 | -1.06357800 | 0.70627110 |
| 6 | -3.76548044 | -0.02997246 | -1.59431463 |
| 6 | 3.69423585 | 1.06517904 | -3.91654874 |
| 6 | 1.45686726 | 0.40926865 | -3.05502143 |
| 6 | 3.56940651 | -0.62834467 | -2.10391669 |
| 1 | 0.01534522 | 3.09340117 | 3.90406481 |
| 1 | -1.21859369 | 1.89205061 | 3.42471496 |
| 1 | -0.25140965 | 4.17615143 | 1.63702936 |
| 1 | 0.01692827 | 3.16090479 | 0.20424768 |
| 1 | -5.71016770 | 2.35758184 | 0.73693679 |
| 1 | -2.41848356 | 4.82708045 | 1.94052569 |
| 1 | 2.28107864 | 1.62735577 | 2.56091869 |
| 1 | 1.97312217 | 3.38135489 | 2.62660480 |
| 1 | 4.15982313 | 3.11975905 | -2.75591820 |
| 1 | 3.14660815 | 4.62626927 | 1.12791096 |
| 1 | -6.20956440 | -0.75310553 | -0.74138696 |
| 1 | -6.35856374 | 0.27145570 | 0.69879527 |
| 1 | -6.22036516 | 1.01116817 | -0.91914825 |
| 1 | -2.87494807 | -1.19135854 | 0.76353172 |
| 1 | -4.36077608 | -0.95144507 | 1.72496659 |
| 1 | -4.39297603 | -1.97779393 | 0.27122940 |
| 1 | -4.05127664 | 0.81541863 | -2.23731406 |
| 1 | -2.67331167 | -0.11033961 | -1.58398678 |
| 1 | -4.17833983 | -0.94801778 | -2.04018730 |
| 1 | 3.62558121 | 0.24286345 | -4.64336001 |
| 1 | 4.76101449 | 1.24461261 | -3.71875822 |
| 1 | 3.27277774 | 1.96181699 | -4.39320101 |
| 1 | 0.84398667 | 0.08231598 | -2.20908901 |
| 1 | 1.00817216 | 1.32132647 | -3.47459068 |
| 1 | 1.42052178 | -0.37074165 | -3.83117692 |
| 1 | 4.63687071 | -0.47082202 | -1.89009867 |
| 1 | 3.07827184 | -0.95720308 | -1.18202974 |
| 1 | 3.48972261 | -1.42624851 | -2.85916930 |
| 1 | 0.39415973 | 1.33067029 | 3.94969763 |
| 8 | 0.39999923 | -0.76077434 | 2.53153578 |
| 6 | -0.67971315 | -1.49094945 | 3.09336141 |
| 1 | -0.29075981 | -2.10939807 | 3.91946108 |
| 8 | -0.13600212 | -1.61293710 | -0.23795260 |
| 1 | -1.17124138 | -2.16585804 | 2.36729631 |
| 1 | -1.46753458 | -0.83483752 | 3.51089584 |
| 31 | 0.13904856 | 0.28484747 | 1.03176095 |
| 6 | 1.65987503 | -3.02309022 | 0.58289888 |
| 6 | 1.36591476 | -4.36165247 | 1.29775439 |

| | | | |
|---|---|---|---|
| 6 | 1.68542838 | -5.60825347 | 0.46722696 |
| 6 | 0.57770549 | -2.60510221 | -0.38449213 |
| 6 | 0.90952131 | -5.70605842 | -0.85003132 |
| 6 | 1.12483414 | -4.53012643 | -1.79553809 |
| 1 | 2.62183690 | -3.08638788 | 0.04837135 |
| 1 | 0.31376384 | -4.37947587 | 1.62379427 |
| 1 | 2.76613851 | -5.61767107 | 0.24289909 |
| 1 | -0.17196208 | -5.80899457 | -0.66424032 |
| 1 | 1.73047444 | -2.21445275 | 1.32091750 |
| 1 | 1.96967524 | -4.38164989 | 2.21684986 |
| 1 | 1.48843817 | -6.50717592 | 1.07156441 |
| 1 | 1.22009649 | -6.61694672 | -1.38713250 |
| 1 | 0.78598678 | -4.78111264 | -2.80759696 |
| 1 | 2.18529617 | -4.23866645 | -1.85189065 |
| 8 | 0.31988377 | -3.35688273 | -1.47484330 |

Electronic energy = -3523.866483 a.u.
DFT-D3(BJ) dispersion correction = -0.116879 a.u.
Thermal free energy = 0.600519 a.u.
Gibbs free energy = -3523.382844 a.u.
Number of imaginary frequencies = 0.

**Int2 ax CL isomer/conformer 3**

| Atomic N. | X | Y | Z |
|---|---|---|---|
| 8 | 1.74588453 | 1.37943949 | 1.62843079 |
| 6 | 1.78282904 | 0.92728238 | 2.97498339 |
| 1 | 0.90146303 | 1.24526177 | 3.56262718 |
| 6 | 3.50282824 | -1.04951689 | 0.53090577 |
| 6 | 4.42015573 | -1.02058006 | -0.71052660 |
| 6 | 5.19217767 | -2.31807460 | -0.96598617 |
| 6 | 2.18951693 | -1.77155523 | 0.33082660 |
| 6 | 4.30748035 | -3.55322211 | -1.16102038 |
| 6 | 3.40895366 | -3.87533862 | 0.02755269 |
| 1 | 4.03777744 | -1.51733116 | 1.37563027 |
| 1 | 3.82625559 | -0.74747437 | -1.59587948 |
| 1 | 5.87307093 | -2.50233909 | -0.11654600 |
| 1 | 3.67516099 | -3.44859074 | -2.05744738 |
| 1 | 3.22990327 | -0.02789569 | 0.83036209 |
| 1 | 5.14061200 | -0.20231220 | -0.56388716 |
| 1 | 5.83367952 | -2.18829891 | -1.85122084 |
| 1 | 4.94764653 | -4.43379283 | -1.33286524 |
| 1 | 3.03636383 | -4.90448576 | -0.03742668 |
| 1 | 3.94459826 | -3.76509385 | 0.98395591 |
| 8 | 1.08753165 | -1.22847562 | 0.38902062 |
| 8 | 2.17692375 | -3.09729754 | 0.07242797 |
| 1 | 1.84397311 | -0.17502560 | 3.05373974 |
| 1 | 2.67812744 | 1.35027437 | 3.45980095 |
| 7 | -0.59884245 | 2.88337409 | 0.58401366 |
| 8 | -1.21225795 | 0.04593299 | 1.18276607 |
| 8 | 0.64273396 | 0.93344580 | -1.28275560 |
| 6 | -0.28673140 | 3.64199960 | 1.81723253 |
| 6 | -2.25137296 | 0.59052558 | 1.85343297 |
| 6 | -2.94294244 | -0.19521884 | 2.82600363 |

| | | | |
|---|---|---|---|
| 6 | -4.00838960 | 0.41065968 | 3.50659245 |
| 6 | -4.41626951 | 1.72472974 | 3.26018255 |
| 6 | -3.75926831 | 2.46637583 | 2.28456013 |
| 6 | -2.69386258 | 1.90875805 | 1.56905135 |
| 6 | -2.07662209 | 2.68554675 | 0.43746687 |
| 1 | -5.24697209 | 2.15469630 | 3.81984764 |
| 6 | -2.55467075 | -1.66169025 | 3.10413116 |
| 6 | 0.00083425 | 1.67808662 | -2.21133091 |
| 6 | -0.28929991 | 1.12497165 | -3.49627589 |
| 6 | -0.92018953 | 1.95832211 | -4.43081960 |
| 6 | -1.27728789 | 3.28013045 | -4.15060516 |
| 6 | -1.00190446 | 3.80324007 | -2.89238690 |
| 6 | -0.36048252 | 3.01919014 | -1.92733222 |
| 6 | -0.02294093 | 3.62094161 | -0.59160308 |
| 1 | -1.77220855 | 3.88672933 | -4.90899444 |
| 6 | 0.05788053 | -0.33477688 | -3.84853813 |
| 6 | -3.46811289 | -2.30821612 | 4.16257380 |
| 6 | -1.10429092 | -1.74767602 | 3.63293849 |
| 6 | -2.68600388 | -2.49635073 | 1.80743754 |
| 6 | -0.34003134 | -0.69433455 | -5.29248391 |
| 6 | -0.69969287 | -1.30057739 | -2.90721728 |
| 6 | 1.58227685 | -0.55997728 | -3.72386529 |
| 1 | -0.72938085 | 4.65142446 | 1.76242508 |
| 1 | -0.70354566 | 3.12141320 | 2.68628712 |
| 1 | -2.56461745 | 3.67259927 | 0.35069944 |
| 1 | -2.22450174 | 2.16041696 | -0.51785943 |
| 1 | -4.54665240 | -0.16048162 | 4.26098011 |
| 1 | -4.07858662 | 3.48556838 | 2.05441346 |
| 1 | 1.06695359 | 3.63382861 | -0.42736467 |
| 1 | -0.38072942 | 4.66502510 | -0.55839935 |
| 1 | -1.15339464 | 1.56234797 | -5.41751512 |
| 1 | -1.27562991 | 4.83202459 | -2.64724275 |
| 1 | -3.15737090 | -3.35229735 | 4.31122715 |
| 1 | -3.40026648 | -1.80113712 | 5.13627727 |
| 1 | -4.52189891 | -2.31856906 | 3.84873971 |
| 1 | -0.39135976 | -1.37576166 | 2.89032786 |
| 1 | -0.99089426 | -1.16475088 | 4.55914760 |
| 1 | -0.85249839 | -2.79528366 | 3.85911299 |
| 1 | -3.72104785 | -2.47300551 | 1.43617613 |
| 1 | -2.02193914 | -2.12202156 | 1.02103676 |
| 1 | -2.42475710 | -3.54572067 | 2.01388320 |
| 1 | -0.06162505 | -1.73982488 | -5.48855551 |
| 1 | 0.17732175 | -0.06855224 | -6.03426533 |
| 1 | -1.42350668 | -0.60606494 | -5.45737603 |
| 1 | -0.42831706 | -1.14706448 | -1.85793944 |
| 1 | -1.78617400 | -1.16480105 | -3.00908631 |
| 1 | -0.46417746 | -2.34232663 | -3.17433043 |
| 1 | 2.12903252 | 0.07590121 | -4.43585203 |
| 1 | 1.92643401 | -0.32566946 | -2.71112625 |
| 1 | 1.82480824 | -1.61015279 | -3.95027108 |
| 1 | 0.80076631 | 3.71124284 | 1.92580631 |
| 31 | 0.30156404 | 0.94729749 | 0.55916305 |

Electronic energy = -3523.865358 a.u.

DFT-D3(BJ) dispersion correction = -0.116837 a.u.
Thermal free energy = 0.601189 a.u.
Gibbs free energy = -3523.381006 a.u.
Number of imaginary frequencies = 0.

**Int2 ax CL isomer/conformer 4**

| Atomic N. | X | Y | Z |
|---|---|---|---|
| 7 | -1.59621966 | 1.83580004 | 1.74380227 |
| 8 | -0.70350783 | 0.91286711 | -0.88203372 |
| 8 | 0.69418782 | -0.02276547 | 1.94774969 |
| 6 | -3.07161367 | 1.89301726 | 1.62243020 |
| 6 | -1.11612741 | 2.13869318 | -1.27218671 |
| 6 | -1.36090012 | 2.37260442 | -2.66224372 |
| 6 | -1.77993183 | 3.65371647 | -3.04470765 |
| 6 | -1.96827660 | 4.69258934 | -2.12974514 |
| 6 | -1.72423662 | 4.45600860 | -0.78216380 |
| 6 | -1.28939950 | 3.19941023 | -0.34519064 |
| 6 | -0.94535938 | 3.02425068 | 1.10979888 |
| 1 | -2.30141031 | 5.67247799 | -2.47158221 |
| 6 | -1.17792766 | 1.25790409 | -3.71148045 |
| 6 | 1.13719539 | 0.86984223 | 2.85467277 |
| 6 | 2.52371105 | 0.88094227 | 3.20099583 |
| 6 | 2.94272787 | 1.81437285 | 4.15838596 |
| 6 | 2.06934741 | 2.72036927 | 4.76806392 |
| 6 | 0.72301764 | 2.69970904 | 4.42299894 |
| 6 | 0.24698567 | 1.77720028 | 3.48370799 |
| 6 | -1.22953727 | 1.72601679 | 3.19546133 |
| 1 | 2.44406825 | 3.43369692 | 5.50225193 |
| 6 | 3.52444805 | -0.09212024 | 2.54641609 |
| 6 | -1.46600792 | 1.75169273 | -5.14197891 |
| 6 | -2.16598083 | 0.10473312 | -3.41912914 |
| 6 | 0.27884624 | 0.73651704 | -3.69488465 |
| 6 | 4.95129153 | 0.09531710 | 3.09591682 |
| 6 | 3.58137545 | 0.15049423 | 1.01944022 |
| 6 | 3.11091907 | -1.55592811 | 2.83096064 |
| 1 | -3.46404415 | 2.77329495 | 2.15942279 |
| 1 | -3.35256708 | 1.96507021 | 0.56630848 |
| 1 | -1.23105112 | 3.93277155 | 1.66885782 |
| 1 | 0.13919609 | 2.89097125 | 1.24461854 |
| 1 | -1.97272006 | 3.85428460 | -4.09700049 |
| 1 | -1.85677467 | 5.25412375 | -0.04806022 |
| 1 | -1.66790291 | 0.77467816 | 3.54002067 |
| 1 | -1.73480085 | 2.53975371 | 3.74485557 |
| 1 | 3.99374529 | 1.84589889 | 4.43990880 |
| 1 | 0.02113026 | 3.39548177 | 4.88878487 |
| 1 | -1.31523720 | 0.91896538 | -5.84432865 |
| 1 | -2.50183292 | 2.10072095 | -5.26123217 |
| 1 | -0.78842447 | 2.56377667 | -5.44233763 |
| 1 | -2.02455847 | -0.29259367 | -2.40869876 |
| 1 | -3.20485947 | 0.45435761 | -3.50966685 |
| 1 | -2.02037516 | -0.70897829 | -4.14769763 |
| 1 | 0.97755502 | 1.54369877 | -3.95883769 |

| | | | |
|---|---|---|---|
| 1 | 0.56091803 | 0.34742480 | -2.71105764 |
| 1 | 0.39635781 | -0.06408105 | -4.44249595 |
| 1 | 5.61988698 | -0.62480946 | 2.60287880 |
| 1 | 5.00636391 | -0.08915692 | 4.17879157 |
| 1 | 5.34465009 | 1.10225515 | 2.89392317 |
| 1 | 2.61210847 | -0.03198473 | 0.54428907 |
| 1 | 3.89335870 | 1.18292747 | 0.80304983 |
| 1 | 4.31961427 | -0.52737976 | 0.56399881 |
| 1 | 3.10077494 | -1.75212722 | 3.91344268 |
| 1 | 2.11826934 | -1.77496328 | 2.42367285 |
| 1 | 3.83776944 | -2.24208502 | 2.36959470 |
| 1 | -3.49561713 | 0.97321493 | 2.03976837 |
| 8 | -2.17582490 | -1.05573417 | 1.13850341 |
| 6 | -1.97317814 | -2.08248708 | 2.10153547 |
| 1 | -1.97328447 | -1.69623777 | 3.13847292 |
| 6 | -1.24552821 | -2.97656178 | -1.32131612 |
| 6 | -1.37981843 | -4.45545981 | -0.89224820 |
| 6 | -0.85113886 | -5.46157937 | -1.91931335 |
| 6 | 0.13788510 | -2.40976034 | -1.10604835 |
| 6 | 0.63332828 | -5.29729849 | -2.26052465 |
| 6 | 1.00381377 | -3.92864967 | -2.82025408 |
| 1 | -1.52250394 | -2.87027605 | -2.38281727 |
| 1 | -0.88041834 | -4.60315259 | 0.07849018 |
| 1 | -1.44065861 | -5.36306718 | -2.84729121 |
| 1 | 1.26485880 | -5.49419172 | -1.37900059 |
| 1 | -1.92046847 | -2.34397588 | -0.72880325 |
| 1 | -2.44809235 | -4.65243940 | -0.71947975 |
| 1 | -1.02697457 | -6.48355191 | -1.54957206 |
| 1 | 0.91584876 | -6.04644581 | -3.01789379 |
| 1 | 1.98802924 | -3.96195776 | -3.30213544 |
| 1 | 0.27045736 | -3.57819691 | -3.56340873 |
| 8 | 0.40113021 | -1.52340767 | -0.29555610 |
| 8 | 1.18531357 | -2.90177534 | -1.80120666 |
| 1 | -2.79867066 | -2.80914867 | 2.01822139 |
| 1 | -1.02068565 | -2.62622561 | 1.95940323 |
| 31 | -0.78603730 | 0.11664030 | 0.81174254 |

Electronic energy = -3523.864780 a.u.
DFT-D3(BJ) dispersion correction = -0.116291 a.u.
Thermal free energy = 0.600119 a.u.
Gibbs free energy = -3523.380952 a.u.
Number of imaginary frequencies = 0.

**Int2 ax CL isomer/conformer 5**

| Atomic N. | X | Y | Z |
|---|---|---|---|
| 7 | 0.65694933 | 2.03254011 | 2.13231102 |
| 8 | -1.24077381 | 0.45925353 | 0.56095144 |
| 8 | 1.96807736 | 0.70388995 | -0.15765235 |
| 6 | 0.22333547 | 1.82031435 | 3.53287545 |
| 6 | -2.10907290 | 1.41083737 | 0.96790010 |
| 6 | -3.51078142 | 1.13366898 | 0.90668749 |
| 6 | -4.39358336 | 2.14303386 | 1.31293102 |
| 6 | -3.95870631 | 3.38684570 | 1.77769219 |

| | | | |
|---|---|---|---|
| 6 | -2.59432679 | 3.64359206 | 1.83966173 |
| 6 | -1.66754470 | 2.67779214 | 1.43041794 |
| 6 | -0.20596306 | 3.03656925 | 1.43396934 |
| 1 | -4.68176866 | 4.14068799 | 2.08905140 |
| 6 | -4.04182592 | -0.23172481 | 0.42706477 |
| 6 | 2.57350589 | 1.89908810 | -0.30545323 |
| 6 | 3.17221809 | 2.22774290 | -1.56083157 |
| 6 | 3.80505634 | 3.47345220 | -1.66788070 |
| 6 | 3.86289010 | 4.38871743 | -0.61240517 |
| 6 | 3.27694956 | 4.05753799 | 0.60370423 |
| 6 | 2.64514525 | 2.81954309 | 0.77103441 |
| 6 | 2.09634450 | 2.45812808 | 2.12513061 |
| 1 | 4.35978782 | 5.34954138 | -0.74657762 |
| 6 | 3.12221338 | 1.25566576 | -2.75636655 |
| 6 | -5.58151701 | -0.28424867 | 0.41771515 |
| 6 | -3.55237805 | -1.34106561 | 1.38727341 |
| 6 | -3.56647410 | -0.52356018 | -1.01627033 |
| 6 | 3.85535520 | 1.82057101 | -3.98777915 |
| 6 | 1.65385283 | 0.99785953 | -3.16991265 |
| 6 | 3.80759213 | -0.08188065 | -2.38779618 |
| 1 | 0.32591193 | 2.75516861 | 4.10969136 |
| 1 | -0.82471770 | 1.50312160 | 3.55094184 |
| 1 | -0.06849167 | 4.02305920 | 1.91087999 |
| 1 | 0.18399387 | 3.11776425 | 0.40754327 |
| 1 | -5.46493911 | 1.95529208 | 1.27324200 |
| 1 | -2.22907049 | 4.60901327 | 2.19745035 |
| 1 | 2.65688847 | 1.61806583 | 2.56768631 |
| 1 | 2.20745246 | 3.32194426 | 2.80399604 |
| 1 | 4.26804792 | 3.75050758 | -2.61326746 |
| 1 | 3.31262029 | 4.75493706 | 1.44397642 |
| 1 | -5.90270159 | -1.27422001 | 0.06267717 |
| 1 | -6.00930513 | -0.13863811 | 1.42016212 |
| 1 | -6.01582657 | 0.46624351 | -0.25835697 |
| 1 | -2.45823292 | -1.38730176 | 1.41950422 |
| 1 | -3.92228077 | -1.15976579 | 2.40711117 |
| 1 | -3.93939546 | -2.31852979 | 1.05884932 |
| 1 | -3.95154929 | 0.23887809 | -1.70904230 |
| 1 | -2.47387877 | -0.53466624 | -1.08808778 |
| 1 | -3.95506228 | -1.50059550 | -1.34475401 |
| 1 | 3.79596247 | 1.08697200 | -4.80449469 |
| 1 | 4.92046864 | 2.00754956 | -3.78707673 |
| 1 | 3.40014085 | 2.75403138 | -4.34947559 |
| 1 | 1.08176260 | 0.53076425 | -2.36192571 |
| 1 | 1.16154232 | 1.93932869 | -3.45504919 |
| 1 | 1.62695361 | 0.32564178 | -4.04129556 |
| 1 | 4.86371685 | 0.08280094 | -2.12721992 |
| 1 | 3.30687034 | -0.56464809 | -1.54204531 |
| 1 | 3.77410412 | -0.76478813 | -3.25058794 |
| 1 | 0.83929557 | 1.03127204 | 3.97805633 |
| 8 | 0.98371198 | -0.97794047 | 2.30785067 |
| 6 | 2.30545312 | -1.50007071 | 2.35147690 |
| 1 | 3.02966138 | -0.78229891 | 2.78231235 |
| 6 | 0.08507701 | -3.56094445 | 0.56585495 |

| | | | |
|---|---|---|---|
| 6 | -1.23256668 | -4.36265281 | 0.47918164 |
| 6 | -1.15185040 | -5.62738135 | -0.38043475 |
| 6 | 0.34348917 | -2.67894458 | -0.63416718 |
| 6 | -0.77880029 | -5.37131873 | -1.84371717 |
| 6 | 0.56464352 | -4.67811864 | -2.03639564 |
| 1 | 0.93336690 | -4.25443111 | 0.69615723 |
| 1 | -2.03542378 | -3.70482956 | 0.11311019 |
| 1 | -0.40753106 | -6.31299358 | 0.06085270 |
| 1 | -1.55415657 | -4.77303922 | -2.34905229 |
| 1 | 0.07618882 | -2.88257312 | 1.42957854 |
| 1 | -1.51388277 | -4.64570422 | 1.50417059 |
| 1 | -2.11769998 | -6.15421618 | -0.33969481 |
| 1 | -0.72911389 | -6.33296034 | -2.37993109 |
| 1 | 0.90470576 | -4.77697858 | -3.07410234 |
| 1 | 1.34030648 | -5.10448203 | -1.38055385 |
| 8 | 0.38432941 | -1.45238541 | -0.59002696 |
| 8 | 0.52090996 | -3.23239640 | -1.85491028 |
| 1 | 2.69202190 | -1.79047966 | 1.35672657 |
| 1 | 2.30845263 | -2.39609588 | 2.99485609 |
| 31 | 0.56386966 | 0.24964052 | 0.99432301 |

Electronic energy = -3523.863440 a.u.
DFT-D3(BJ) dispersion correction = -0.115983 a.u.
Thermal free energy = 0.601457 a.u.
Gibbs free energy = -3523.377965 a.u.
Number of imaginary frequencies = 0.

**Int2 ax CL isomer/conformer 6**

| Atomic N. | X | Y | Z |
|---|---|---|---|
| 8 | -1.34095374 | 1.18104795 | -0.10284609 |
| 6 | -2.17403149 | 1.29478940 | 1.04233418 |
| 1 | -1.78335985 | 1.99602008 | 1.80259169 |
| 6 | -2.04025398 | -1.97159069 | -0.80222410 |
| 6 | -3.00864893 | -2.62941279 | 0.20550662 |
| 6 | -3.32026499 | -4.10080043 | -0.08553949 |
| 6 | -0.58252057 | -2.29795579 | -0.56757362 |
| 6 | -2.09260639 | -5.01695455 | -0.10153463 |
| 6 | -1.03723410 | -4.63720831 | -1.13440855 |
| 1 | -2.31429842 | -2.27101095 | -1.82725594 |
| 1 | -2.61053703 | -2.51833783 | 1.22576776 |
| 1 | -4.04050144 | -4.47269310 | 0.65951712 |
| 1 | -1.61205669 | -5.05085580 | 0.88968443 |
| 1 | -2.10642434 | -0.87627522 | -0.74943925 |
| 1 | -3.94775761 | -2.05714815 | 0.17927385 |
| 1 | -3.82532206 | -4.17041938 | -1.06469079 |
| 1 | -2.41310573 | -6.04718362 | -0.32671938 |
| 1 | -0.34639365 | -5.47144948 | -1.30476288 |
| 1 | -1.49176236 | -4.36663110 | -2.10064788 |
| 8 | 0.26322038 | -1.47188092 | -0.23421920 |
| 8 | -0.14350511 | -3.56924906 | -0.70541273 |
| 1 | -2.33328583 | 0.32508600 | 1.54964463 |
| 1 | -3.15824437 | 1.67074104 | 0.71690403 |
| 7 | 1.27752700 | 2.83529778 | 0.27571120 |

| | | | |
|---|---|---|---|
| 8 | 1.20270399 | 0.61266257 | -1.68193895 |
| 8 | 1.14404087 | 0.24212852 | 1.69161568 |
| 6 | 0.22040702 | 3.87202530 | 0.21758598 |
| 6 | 1.28431794 | 1.60889989 | -2.59640540 |
| 6 | 0.96405237 | 1.36979745 | -3.96714103 |
| 6 | 1.07645218 | 2.45138544 | -4.85316456 |
| 6 | 1.49177064 | 3.72470118 | -4.45376436 |
| 6 | 1.84309248 | 3.93486584 | -3.12474089 |
| 6 | 1.75805627 | 2.88549627 | -2.20520421 |
| 6 | 2.28698752 | 3.05040477 | -0.81389373 |
| 6 | 0.50935148 | -0.01411206 | -4.46681014 |
| 6 | 0.96316894 | 0.99090633 | 2.80759780 |
| 6 | 0.54870572 | 0.39360056 | 4.03593876 |
| 6 | 0.31957242 | 1.25384811 | 5.12068977 |
| 6 | 0.51552313 | 2.63640842 | 5.05321337 |
| 6 | 1.01572022 | 3.19284561 | 3.88007818 |
| 6 | 1.26107256 | 2.37739363 | 2.77165299 |
| 6 | 2.00899948 | 2.90299375 | 1.58276451 |
| 6 | 0.39970421 | -1.13245639 | 4.19062148 |
| 1 | 0.67491196 | 4.87736632 | 0.25616713 |
| 1 | -0.35379049 | 3.76197476 | -0.70755905 |
| 1 | 2.72768508 | 4.05543070 | -0.68875163 |
| 1 | 3.08071455 | 2.30753227 | -0.63537854 |
| 1 | 0.82841702 | 2.29956101 | -5.90216445 |
| 1 | 2.20808711 | 4.90942478 | -2.79212788 |
| 1 | 2.91792205 | 2.29624936 | 1.44653452 |
| 1 | 2.32300735 | 3.94746903 | 1.75573964 |
| 1 | -0.02218303 | 0.83104607 | 6.06386428 |
| 1 | 1.24708886 | 4.25910955 | 3.82295478 |
| 1 | -0.45420103 | 3.74931805 | 1.07175490 |
| 31 | 0.46432733 | 0.84025436 | 0.03088020 |
| 6 | 1.56171049 | -1.09032984 | -4.10999910 |
| 6 | 0.32089413 | -0.05031331 | -5.99507894 |
| 6 | -0.84974231 | -0.36278758 | -3.82190017 |
| 1 | 1.23848892 | -2.06759126 | -4.50181251 |
| 1 | 1.70666666 | -1.17780827 | -3.02805931 |
| 1 | 2.53041490 | -0.85029701 | -4.57233383 |
| 1 | -1.62643347 | 0.34425042 | -4.14762445 |
| 1 | -0.78689524 | -0.31869926 | -2.73041131 |
| 1 | -1.16226126 | -1.37538761 | -4.12448543 |
| 1 | 0.01152440 | -1.06289268 | -6.29230556 |
| 1 | 1.25240971 | 0.18227831 | -6.53113150 |
| 1 | -0.45959427 | 0.64516366 | -6.33578573 |
| 6 | 0.05880328 | -1.53890493 | 5.63630029 |
| 6 | 1.73093468 | -1.82558252 | 3.81387996 |
| 6 | -0.73116239 | -1.65674602 | 3.27977696 |
| 1 | 2.54386664 | -1.48402165 | 4.47119926 |
| 1 | 2.00876426 | -1.61867508 | 2.77452564 |
| 1 | 1.63136537 | -2.91477878 | 3.93866758 |
| 1 | -0.81963496 | -2.74962739 | 3.38640916 |
| 1 | -0.51883011 | -1.42399636 | 2.23157788 |
| 1 | -1.69581360 | -1.20776785 | 3.55974318 |
| 1 | 0.83201035 | -1.21676668 | 6.34846042 |

| | | | |
|---|---|---|---|
| 1 | -0.00735766 | -2.63503863 | 5.69253689 |
| 1 | -0.90854519 | -1.13277623 | 5.96628577 |
| 1 | 0.31080709 | 3.26088614 | 5.92282151 |
| 1 | 1.55424473 | 4.53375394 | -5.18152679 |

Electronic energy = -3523.859105 a.u.
DFT-D3(BJ) dispersion correction = -0.118312 a.u.
Thermal free energy = 0.601774 a.u.
Gibbs free energy = -3523.375643 a.u.
Number of imaginary frequencies = 0.

**Int2 ax CL isomer/conformer 7**

| Atomic N. | X | Y | Z |
|---|---|---|---|
| 8 | -0.57037508 | 1.38756971 | 0.85408622 |
| 6 | -0.05699846 | 1.80836399 | 2.11024344 |
| 1 | -0.36736956 | 2.84939063 | 2.30942642 |
| 6 | -1.68942341 | -1.65067845 | 1.66440853 |
| 6 | -3.15963242 | -2.11942031 | 1.59814205 |
| 6 | -3.41386558 | -3.51319110 | 2.17867547 |
| 6 | -0.80458325 | -2.21554364 | 0.57557292 |
| 6 | -2.65547517 | -4.63587049 | 1.46518338 |
| 6 | -1.14093387 | -4.47019653 | 1.47602093 |
| 1 | -1.26001263 | -1.91010420 | 2.64672896 |
| 1 | -3.51085523 | -2.07355071 | 0.55627247 |
| 1 | -3.13074814 | -3.51498250 | 3.24572640 |
| 1 | -2.98816119 | -4.73268278 | 0.41900514 |
| 1 | -1.62604094 | -0.55958389 | 1.55069341 |
| 1 | -3.76187035 | -1.38605082 | 2.15439811 |
| 1 | -4.49344977 | -3.72663195 | 2.14572861 |
| 1 | -2.88148340 | -5.59750449 | 1.95413776 |
| 1 | -0.64760585 | -5.41290998 | 1.21073013 |
| 1 | -0.77343147 | -4.15934150 | 2.46685434 |
| 8 | -0.22939588 | -1.52805089 | -0.26389782 |
| 8 | -0.63062004 | -3.55150969 | 0.46603173 |
| 1 | 1.04363709 | 1.76447850 | 2.18502853 |
| 1 | -0.46056862 | 1.17962772 | 2.92628883 |
| 7 | 1.58042610 | 2.35237732 | -1.29779032 |
| 8 | -0.59087010 | 0.47783538 | -2.01321231 |
| 8 | 2.05228090 | -0.19507270 | 0.11917602 |
| 6 | 1.25200118 | 3.61680013 | -0.59880528 |
| 6 | -1.08613163 | 1.54213930 | -2.68954720 |
| 6 | -2.45144766 | 1.57769069 | -3.10400411 |
| 6 | -2.87821222 | 2.70947465 | -3.81462213 |
| 6 | -2.03299732 | 3.77742027 | -4.12914852 |
| 6 | -0.69699042 | 3.71775899 | -3.74760990 |
| 6 | -0.22150896 | 2.60518097 | -3.04785860 |
| 6 | 1.23879041 | 2.45291700 | -2.75660479 |
| 1 | -2.41815150 | 4.63706551 | -4.67764501 |
| 6 | -3.43092550 | 0.43492300 | -2.77942046 |
| 6 | 3.06312714 | 0.47277957 | 0.72333769 |
| 6 | 3.69845545 | -0.05752829 | 1.88771195 |
| 6 | 4.71233484 | 0.71464773 | 2.47392310 |
| 6 | 5.13009621 | 1.94439766 | 1.95659564 |

| | | | |
|---|---|---|---|
| 6 | 4.55338078 | 2.41568813 | 0.78146720 |
| 6 | 3.54313643 | 1.67989555 | 0.15551489 |
| 6 | 3.04861495 | 2.06328022 | -1.20631580 |
| 1 | 5.91607615 | 2.50984680 | 2.45711804 |
| 6 | 3.30875884 | -1.42856469 | 2.47335217 |
| 1 | 1.75954629 | 4.46522155 | -1.09001557 |
| 1 | 0.16898691 | 3.77600494 | -0.61899231 |
| 1 | 1.80722749 | 3.29497533 | -3.18981656 |
| 1 | 1.60868923 | 1.52173533 | -3.21445468 |
| 1 | -3.91660047 | 2.76750004 | -4.13610767 |
| 1 | -0.00599804 | 4.52324358 | -4.00722530 |
| 1 | 3.23085836 | 1.22732237 | -1.90010087 |
| 1 | 3.60271954 | 2.94138763 | -1.58277707 |
| 1 | 5.20257780 | 0.34442029 | 3.37266931 |
| 1 | 4.89950515 | 3.34600161 | 0.32494541 |
| 1 | 1.58721163 | 3.55322031 | 0.44209312 |
| 31 | 0.47474107 | 0.66956644 | -0.47729674 |
| 6 | -2.89162869 | -0.91133050 | -3.31788392 |
| 6 | -4.81710222 | 0.65878707 | -3.41292046 |
| 6 | -3.62771028 | 0.35518408 | -1.24924672 |
| 1 | -3.60447439 | -1.71697166 | -3.08179599 |
| 1 | -1.91978232 | -1.16017380 | -2.87878212 |
| 1 | -2.78098994 | -0.87298018 | -4.41162802 |
| 1 | -4.05047709 | 1.29367829 | -0.86265860 |
| 1 | -2.67858384 | 0.17294500 | -0.73430741 |
| 1 | -4.32943938 | -0.45817566 | -1.00595856 |
| 1 | -5.46424476 | -0.19525701 | -3.16574446 |
| 1 | -4.76342497 | 0.72746731 | -4.50912229 |
| 1 | -5.30885101 | 1.56509054 | -3.03095525 |
| 6 | 4.20407690 | -1.83138143 | 3.65978057 |
| 6 | 3.45125389 | -2.52592247 | 1.39204127 |
| 6 | 1.85241828 | -1.38247297 | 2.98530822 |
| 1 | 4.49128211 | -2.58660788 | 1.03958036 |
| 1 | 2.80193697 | -2.33086190 | 0.53177754 |
| 1 | 3.18476400 | -3.50518825 | 1.81920748 |
| 1 | 1.55527285 | -2.37354024 | 3.36445660 |
| 1 | 1.17042104 | -1.09082669 | 2.18062967 |
| 1 | 1.74984187 | -0.65968425 | 3.80783363 |
| 1 | 5.26287373 | -1.90049991 | 3.37172203 |
| 1 | 3.89384529 | -2.82271308 | 4.02081484 |
| 1 | 4.11827611 | -1.13174367 | 4.50383227 |

Electronic energy = -3523.858241 a.u.
DFT-D3(BJ) dispersion correction = -0.118504 a.u.
Thermal free energy = 0.601255 a.u.
Gibbs free energy = -3523.375490 a.u.
Number of imaginary frequencies = 0.

**Int2_ax_CL isomer/conformer 8**

| Atomic N. | X | Y | Z |
|---|---|---|---|
| 8 | 1.01104563 | -2.49222803 | -2.74743028 |
| 6 | 0.77984795 | -3.76025524 | -3.40104353 |
| 1 | 1.17524210 | -3.63119909 | -4.41324622 |

| | | | |
|---|---|---|---|
| 1 | -0.29550395 | -3.97745369 | -3.45418748 |
| 1 | 1.30964679 | -4.58040982 | -2.90084252 |
| 6 | 0.70275884 | -2.32408189 | -1.43863548 |
| 8 | 0.83532481 | -1.18001704 | -1.00875723 |
| 7 | -0.69054215 | 1.16808997 | 2.36827851 |
| 8 | -1.47917136 | -0.08772913 | -0.14440365 |
| 8 | 1.61330661 | 0.91913039 | 0.53823461 |
| 6 | -1.48166339 | 0.43251796 | 3.38165789 |
| 6 | -2.65236173 | 0.53773654 | 0.09390096 |
| 6 | -3.82241992 | 0.09776724 | -0.60260436 |
| 6 | -5.02681265 | 0.76401496 | -0.34052751 |
| 6 | -5.12391579 | 1.82427821 | 0.56402226 |
| 6 | -3.98006855 | 2.24888940 | 1.22932861 |
| 6 | -2.74700424 | 1.62922517 | 0.99564065 |
| 6 | -1.52195032 | 2.19295762 | 1.66343491 |
| 1 | -6.08370058 | 2.30941540 | 0.74135381 |
| 6 | -3.77261980 | -1.07077309 | -1.60735926 |
| 6 | 1.87233444 | 2.17925930 | 0.94222194 |
| 6 | 2.73014642 | 3.00379823 | 0.15078351 |
| 6 | 2.98650322 | 4.30140501 | 0.61426851 |
| 6 | 2.43731025 | 4.80716468 | 1.79632293 |
| 6 | 1.60456212 | 3.99532977 | 2.55757405 |
| 6 | 1.32739598 | 2.68516722 | 2.14973885 |
| 6 | 0.48987404 | 1.80626670 | 3.03989259 |
| 1 | 2.65971727 | 5.82648296 | 2.11196066 |
| 6 | 3.34930077 | 2.49322706 | -1.16566946 |
| 6 | -5.14232816 | -1.33674257 | -2.26034350 |
| 6 | -3.35774088 | -2.36736402 | -0.87401752 |
| 6 | -2.77807930 | -0.75535767 | -2.74997573 |
| 6 | 4.25361994 | 3.55213529 | -1.82446973 |
| 6 | 2.23252495 | 2.14459297 | -2.17799934 |
| 6 | 4.22472557 | 1.24588360 | -0.89678961 |
| 1 | -1.84540050 | 1.12672331 | 4.15833076 |
| 1 | -2.34110055 | -0.04818278 | 2.90243231 |
| 1 | -1.82177747 | 2.97188666 | 2.38676555 |
| 1 | -0.85971791 | 2.67542927 | 0.92790097 |
| 1 | -5.92999915 | 0.44528618 | -0.85756856 |
| 1 | -4.02783322 | 3.08220587 | 1.93412354 |
| 1 | 1.08601500 | 0.97404585 | 3.44881972 |
| 1 | 0.12424931 | 2.40073731 | 3.89580820 |
| 1 | 3.63527710 | 4.95171037 | 0.03032091 |
| 1 | 1.16673998 | 4.36756601 | 3.48677446 |
| 1 | -5.04391256 | -2.17256307 | -2.96830853 |
| 1 | -5.90772075 | -1.61771878 | -1.52266931 |
| 1 | -5.50618389 | -0.46645994 | -2.82512991 |
| 1 | -2.39201469 | -2.24741705 | -0.37264141 |
| 1 | -4.10771453 | -2.63909520 | -0.11683951 |
| 1 | -3.28690908 | -3.20061122 | -1.59136955 |
| 1 | -3.10687286 | 0.13212621 | -3.31003076 |
| 1 | -1.76866218 | -0.56562866 | -2.37071717 |
| 1 | -2.74206746 | -1.60024724 | -3.45614619 |
| 1 | 4.67329516 | 3.13472822 | -2.75091447 |
| 1 | 5.09649569 | 3.83786741 | -1.17828820 |

| | | | |
|---|---|---|---|
| 1 | 3.69809696 | 4.46193156 | -2.09479597 |
| 1 | 1.58330961 | 1.34546072 | -1.80612938 |
| 1 | 1.61626078 | 3.02980040 | -2.39402114 |
| 1 | 2.68233583 | 1.80778814 | -3.12464212 |
| 1 | 5.04541280 | 1.49023683 | -0.20608326 |
| 1 | 3.63363045 | 0.43047867 | -0.46686018 |
| 1 | 4.67000550 | 0.89544844 | -1.84057275 |
| 1 | -0.84963774 | -0.34074354 | 3.83155713 |
| 31 | 0.09539765 | -0.12460100 | 0.87449150 |
| 6 | 0.27957069 | -3.51746699 | -0.61997447 |
| 6 | 1.48138371 | -4.41107247 | -0.18251059 |
| 6 | 1.95653269 | -4.17123814 | 1.25953860 |
| 6 | 2.55824464 | -2.77688680 | 1.54996191 |
| 6 | 1.68231798 | -1.88287845 | 2.43667906 |
| 1 | -0.22342320 | -3.13150127 | 0.27509198 |
| 1 | 2.33431844 | -4.27676207 | -0.86702471 |
| 1 | 1.10975067 | -4.35164353 | 1.94148593 |
| 1 | 2.78548236 | -2.24450605 | 0.61341256 |
| 1 | -0.44110412 | -4.11219450 | -1.19707559 |
| 1 | 1.17501010 | -5.46389394 | -0.27782612 |
| 1 | 2.70322851 | -4.94858506 | 1.48517907 |
| 1 | 3.52284138 | -2.89124976 | 2.07207175 |
| 1 | 2.24083141 | -0.94726862 | 2.63226207 |
| 1 | 1.54469561 | -2.38614574 | 3.41323134 |
| 8 | 0.38201691 | -1.62601995 | 1.91158773 |

Electronic energy = -3523.856529 a.u.
DFT-D3(BJ) dispersion correction = -0.118293 a.u.
Thermal free energy = 0.602555 a.u.
Gibbs free energy = -3523.372266 a.u.
Number of imaginary frequencies = 0.

**Int2_ax_CL isomer/conformer 9**

| Atomic N. | X | Y | Z |
|---|---|---|---|
| 7 | 0.02678674 | 2.77931581 | 1.35516882 |
| 8 | -1.26509634 | 0.56667140 | -0.11209790 |
| 8 | 1.90990773 | 0.63927078 | 0.51232617 |
| 6 | -0.85561846 | 3.15654678 | 2.48470268 |
| 6 | -2.17956216 | 1.52422821 | -0.39468387 |
| 6 | -3.48159523 | 1.13074000 | -0.83181059 |
| 6 | -4.39987873 | 2.14530020 | -1.13470068 |
| 6 | -4.09251529 | 3.50358870 | -1.02204571 |
| 6 | -2.82128489 | 3.87525565 | -0.60082948 |
| 6 | -1.86011575 | 2.90360132 | -0.29911162 |
| 6 | -0.46786123 | 3.34712977 | 0.05899990 |
| 6 | -3.87432685 | -0.35387711 | -0.96314690 |
| 6 | 2.63289146 | 1.68057147 | 0.04526385 |
| 6 | 3.64518362 | 1.45030389 | -0.93588341 |
| 6 | 4.38036872 | 2.55930704 | -1.37657100 |
| 6 | 4.15820657 | 3.85542851 | -0.90212376 |
| 6 | 3.17414289 | 4.06606382 | 0.05635476 |
| 6 | 2.41988754 | 2.99168871 | 0.54297931 |
| 6 | 1.42793963 | 3.23897429 | 1.64749047 |

| | | | |
|---|---|---|---|
| 6 | 3.92235656 | 0.04138575 | -1.49659708 |
| 6 | -5.31527451 | -0.52769263 | -1.47960493 |
| 6 | -3.79730595 | -1.03872651 | 0.42128781 |
| 6 | -2.93888096 | -1.07022533 | -1.96616290 |
| 6 | 5.07960688 | 0.04381799 | -2.51340864 |
| 6 | 2.66715565 | -0.49279376 | -2.22619617 |
| 6 | 4.31885896 | -0.92170742 | -0.35240345 |
| 1 | -0.84849144 | 4.25078137 | 2.62278805 |
| 1 | -1.87960120 | 2.82801303 | 2.27805116 |
| 1 | -0.42983732 | 4.44908372 | 0.11436526 |
| 1 | 0.25615528 | 3.03389780 | -0.70863096 |
| 1 | -5.39902281 | 1.87082013 | -1.46759310 |
| 1 | -2.55342721 | 4.93079564 | -0.51434501 |
| 1 | 1.72764618 | 2.71405826 | 2.56950943 |
| 1 | 1.39777614 | 4.31845588 | 1.87610112 |
| 1 | 5.15613434 | 2.41336735 | -2.12594557 |
| 1 | 2.98760514 | 5.06871532 | 0.44802059 |
| 1 | -5.53826130 | -1.60130445 | -1.56077815 |
| 1 | -6.05737731 | -0.08741002 | -0.79805417 |
| 1 | -5.45282044 | -0.08547547 | -2.47686812 |
| 1 | -2.78405831 | -0.99342320 | 0.83515618 |
| 1 | -4.48500245 | -0.55580093 | 1.13084183 |
| 1 | -4.09579065 | -2.09498263 | 0.33077344 |
| 1 | -3.01464552 | -0.60775965 | -2.96113945 |
| 1 | -1.89347488 | -1.02842649 | -1.64207026 |
| 1 | -3.23910946 | -2.12546377 | -2.06394030 |
| 1 | 5.24084185 | -0.98323596 | -2.87092706 |
| 1 | 6.02308207 | 0.39030848 | -2.06701957 |
| 1 | 4.85976183 | 0.66797580 | -3.39175071 |
| 1 | 1.81349272 | -0.58516489 | -1.54707752 |
| 1 | 2.38922372 | 0.17258596 | -3.05680363 |
| 1 | 2.88151548 | -1.48704796 | -2.64864032 |
| 1 | 5.23327961 | -0.57117872 | 0.14873341 |
| 1 | 3.52106567 | -1.00738431 | 0.39237619 |
| 1 | 4.52152892 | -1.92268675 | -0.76289842 |
| 1 | -0.50310656 | 2.66041917 | 3.39559084 |
| 8 | -0.13761285 | 0.07254435 | 2.80561909 |
| 6 | 0.99391135 | -0.16793636 | 3.63530406 |
| 1 | 1.83804645 | -0.63450459 | 3.09633687 |
| 6 | -0.24196800 | -3.22556030 | 2.22116893 |
| 6 | -1.42514424 | -4.11707830 | 1.74045849 |
| 6 | -1.10787317 | -5.22254104 | 0.70878430 |
| 6 | 0.50038162 | -2.79312877 | 0.99066639 |
| 6 | -0.70768938 | -4.70940491 | -0.69493060 |
| 6 | 0.77225821 | -4.31405989 | -0.80258026 |
| 1 | 0.43300424 | -3.81928740 | 2.85366175 |
| 1 | -2.19949860 | -3.45869511 | 1.31831072 |
| 1 | -0.32897828 | -5.89628084 | 1.09999159 |
| 1 | -1.34384269 | -3.84961359 | -0.95758127 |
| 1 | -0.61440289 | -2.35083741 | 2.76773938 |
| 1 | -1.86355547 | -4.58275509 | 2.63570828 |
| 1 | -2.01986119 | -5.82981717 | 0.60255493 |
| 1 | -0.90965486 | -5.48472862 | -1.44971640 |

| | | | |
|---|---|---|---|
| 1 | 0.94567650 | -3.54666871 | -1.57040833 |
| 1 | 1.41113311 | -5.18171926 | -1.00709892 |
| 8 | 0.40331819 | -1.72929649 | 0.40150990 |
| 8 | 1.23712183 | -3.84268052 | 0.49605685 |
| 1 | 0.69286054 | -0.84546087 | 4.45151900 |
| 1 | 1.37491809 | 0.76068700 | 4.10382833 |
| 31 | 0.13841135 | 0.69703889 | 1.10272833 |
| 1 | -4.84154122 | 4.25782857 | -1.26303432 |
| 1 | 4.75152266 | 4.68725639 | -1.28161795 |

Electronic energy = -3523.850780 a.u.
DFT-D3(BJ) dispersion correction = -0.117063 a.u.
Thermal free energy = 0.596265 a.u.
Gibbs free energy = -3523.371579 a.u.
Number of imaginary frequencies = 0.

**Int2 ax CL isomer/conformer 10**

| Atomic N. | X | Y | Z |
|---|---|---|---|
| 7 | -1.49324542 | 1.92273670 | 0.82699110 |
| 8 | 0.00410177 | 0.67387908 | -1.35465141 |
| 8 | 0.55471241 | 0.05120697 | 1.77521032 |
| 6 | -2.87238268 | 2.15440757 | 0.33331319 |
| 6 | -0.22714546 | 1.84837169 | -1.97505890 |
| 6 | -0.13516910 | 1.92600127 | -3.39955928 |
| 6 | -0.38233766 | 3.16881763 | -3.99856898 |
| 6 | -0.71004931 | 4.31267288 | -3.26496840 |
| 6 | -0.79177707 | 4.22672893 | -1.87940952 |
| 6 | -0.54445904 | 3.01262226 | -1.23021471 |
| 6 | -0.55786900 | 2.95960425 | 0.27144694 |
| 1 | -0.89678803 | 5.25723104 | -3.77588859 |
| 6 | 0.22812003 | 0.69344034 | -4.24983076 |
| 6 | 0.81264925 | 1.00461654 | 2.68733135 |
| 6 | 2.06528676 | 1.00192384 | 3.37565172 |
| 6 | 2.28537704 | 2.01104192 | 4.32239122 |
| 6 | 1.34151157 | 3.00324116 | 4.60581749 |
| 6 | 0.12614859 | 2.99492831 | 3.93027265 |
| 6 | -0.15148219 | 2.00064820 | 2.98524848 |
| 6 | -1.50789655 | 1.96714404 | 2.33379688 |
| 1 | 1.56079204 | 3.77317087 | 5.34551878 |
| 6 | 3.13495668 | -0.06947258 | 3.08697063 |
| 6 | 0.27021805 | 1.01753904 | -5.75507002 |
| 6 | -0.82840026 | -0.41884445 | -4.05026900 |
| 6 | 1.63073462 | 0.17550167 | -3.85239924 |
| 6 | 4.38695934 | 0.11314955 | 3.96513589 |
| 6 | 3.59098069 | 0.02105332 | 1.61126331 |
| 6 | 2.56933613 | -1.47988038 | 3.38044176 |
| 1 | -3.19050472 | 3.17899579 | 0.58851130 |
| 1 | -2.90550716 | 2.03301553 | -0.75434844 |
| 1 | -0.84556106 | 3.94368825 | 0.67917250 |
| 1 | 0.44076241 | 2.72172135 | 0.66640966 |
| 1 | -0.32116240 | 3.25384314 | -5.08215483 |
| 1 | -1.03724125 | 5.10858277 | -1.28293004 |
| 1 | -2.07983133 | 1.07898097 | 2.64672666 |

```
   1    -2.07721168   2.86084145   2.64187552
   1     3.23165220   2.03359310   4.86023748
   1    -0.62753137   3.75829264   4.13785517
   1     0.53737384   0.10631714  -6.30955397
   1    -0.70371195   1.36139610  -6.13272048
   1     1.02460145   1.78140331  -5.99228623
   1    -0.89274230  -0.72281984  -2.99963001
   1    -1.82020590  -0.07088010  -4.37560421
   1    -0.56400057  -1.29954008  -4.65600262
   1     2.39401596   0.94061704  -4.05596853
   1     1.67611397  -0.08236056  -2.78865009
   1     1.88240498  -0.71878743  -4.44336300
   1     5.11092674  -0.67868778   3.72474125
   1     4.15477028   0.03555377   5.03726056
   1     4.88196768   1.07851753   3.78550681
   1     2.75536383  -0.13733614   0.92109675
   1     4.03161505   1.00698165   1.40289146
   1     4.35881977  -0.74176834   1.40994441
   1     2.28998150  -1.56971607   4.44064875
   1     1.68617171  -1.69074866   2.76746582
   1     3.33715300  -2.23992172   3.16805965
   1    -3.54611440   1.43044155   0.80023531
   8    -2.33357171  -2.42096869   2.04129194
   6    -2.34587151  -3.82596639   2.35698900
   1    -2.32868997  -3.87027873   3.45046741
   6    -3.14312125  -2.82463539  -0.30260168
   6    -2.51916886  -4.13845973  -0.82503029
   6    -1.22422888  -4.06798830  -1.65902861
   6    -2.53940757  -1.96465982   0.79523267
   6     0.10174507  -3.96050673  -0.87889056
   6     0.54388352  -2.52111626  -0.62064869
   1    -3.26851705  -2.13433792  -1.14653014
   1    -2.38324750  -4.86928255  -0.01627122
   1    -1.19697020  -4.99327308  -2.25637809
   1     0.01389140  -4.49919953   0.08063852
   1    -4.16445968  -3.05837285   0.05704719
   1    -3.30111489  -4.56965624  -1.47176210
   1    -1.29415288  -3.24103701  -2.38412246
   1     0.90428318  -4.46567208  -1.44047004
   1     1.42866513  -2.51115593   0.04412470
   1     0.84184676  -2.05989591  -1.58149765
   8    -2.56726475  -0.70568714   0.70510692
   8    -0.53534413  -1.80424878  -0.05422827
   1    -1.44933823  -4.31428202   1.95476657
   1    -3.25398825  -4.31607588   1.98237150
  31    -0.63110929   0.05305890   0.31308385
Electronic energy = -3523.842638 a.u.
DFT-D3(BJ) dispersion correction = -0.117855 a.u.
Thermal free energy = 0.602494 a.u.
Gibbs free energy = -3523.357998 a.u.
Number of imaginary frequencies = 0.
```

## Initiation step with equatorial CL addition

**TS1 eq CL isomer/conformer 1**

| Atomic N. | X | Y | Z |
|---|---|---|---|
| 7 | 0.54781044 | 2.37697315 | 0.41169080 |
| 8 | -1.39348805 | 0.28091687 | 1.05647969 |
| 8 | 0.38704935 | 0.17688140 | -1.59136200 |
| 6 | 1.23774099 | 2.84754491 | 1.63797188 |
| 6 | -1.97493732 | 1.26035917 | 1.77886653 |
| 6 | -2.88380671 | 0.92277777 | 2.82731819 |
| 6 | -3.46398687 | 1.97962415 | 3.54270768 |
| 6 | -3.18799806 | 3.32279611 | 3.26915479 |
| 6 | -2.30760264 | 3.63867521 | 2.23959112 |
| 6 | -1.70938325 | 2.62218714 | 1.48775494 |
| 6 | -0.82952404 | 2.97004676 | 0.32051423 |
| 1 | -3.66125154 | 4.10965157 | 3.85659838 |
| 6 | -3.21759605 | -0.54467493 | 3.15949488 |
| 6 | 0.27478430 | 1.19695568 | -2.46755052 |
| 6 | -0.27593619 | 0.96114427 | -3.76496530 |
| 6 | -0.35541844 | 2.05054164 | -4.64315414 |
| 6 | 0.07146183 | 3.33571267 | -4.29706430 |
| 6 | 0.60445380 | 3.55380464 | -3.03172755 |
| 6 | 0.72044313 | 2.49843335 | -2.11962342 |
| 6 | 1.37581468 | 2.75505781 | -0.78797030 |
| 1 | -0.01702812 | 4.15400362 | -5.01158810 |
| 6 | -0.76801699 | -0.43664542 | -4.18958902 |
| 6 | -4.22896242 | -0.65883571 | 4.31567049 |
| 6 | -1.93758667 | -1.30025434 | 3.58980942 |
| 6 | -3.84988999 | -1.23412502 | 1.92693353 |
| 6 | -1.30580628 | -0.44865796 | -5.63300562 |
| 6 | -1.92180312 | -0.89578372 | -3.26703668 |
| 6 | 0.39947098 | -1.44995761 | -4.12919521 |
| 1 | 1.30819201 | 3.94823122 | 1.62709332 |
| 1 | 0.67266786 | 2.53621912 | 2.52287722 |
| 1 | -0.73376334 | 4.06607962 | 0.23164199 |
| 1 | -1.26366544 | 2.59953284 | -0.61975157 |
| 1 | -4.15718079 | 1.75235558 | 4.35080910 |
| 1 | -2.08565879 | 4.68120193 | 1.99945724 |
| 1 | 2.31272078 | 2.18442988 | -0.68853098 |
| 1 | 1.62407422 | 3.82742214 | -0.70826423 |
| 1 | -0.77269238 | 1.89828968 | -5.63693523 |
| 1 | 0.94638973 | 4.54946899 | -2.73972154 |
| 1 | -4.43678141 | -1.72189009 | 4.50460577 |
| 1 | -3.84097099 | -0.22674926 | 5.24959087 |
| 1 | -5.18588209 | -0.17181620 | 4.07823992 |
| 1 | -1.18365908 | -1.30896831 | 2.79502439 |
| 1 | -1.49689102 | -0.83445883 | 4.48374128 |
| 1 | -2.18965030 | -2.34195018 | 3.84272051 |
| 1 | -4.79105520 | -0.73828191 | 1.64749062 |
| 1 | -3.17508316 | -1.20720351 | 1.06432343 |
| 1 | -4.07850838 | -2.28488302 | 2.16338372 |
| 1 | -1.63697076 | -1.46706510 | -5.88216019 |
| 1 | -0.53589928 | -0.16274796 | -6.36427784 |

| | | | |
|---|---|---|---|
| 1 | -2.17017913 | 0.21920480 | -5.75840425 |
| 1 | -1.60925615 | -0.93601573 | -2.21843410 |
| 1 | -2.77560616 | -0.20732351 | -3.34594893 |
| 1 | -2.26626253 | -1.89705825 | -3.56813216 |
| 1 | 1.19975471 | -1.15814352 | -4.82513618 |
| 1 | 0.82050669 | -1.50993836 | -3.11973619 |
| 1 | 0.04364865 | -2.44950080 | -4.42294550 |
| 1 | 2.23908257 | 2.40879631 | 1.67759109 |
| 8 | 0.62761503 | -1.61839330 | 0.67259759 |
| 6 | 0.03570993 | -2.72612715 | 0.01313291 |
| 1 | -1.06055999 | -2.61149383 | 0.00717922 |
| 6 | 3.13402033 | -1.73277473 | -0.26580873 |
| 6 | 4.67606020 | -1.70073211 | -0.20443720 |
| 6 | 5.27503681 | -2.88554168 | 0.56552368 |
| 6 | 2.49097037 | -1.13462484 | 0.97216862 |
| 6 | 4.86665821 | -2.97059083 | 2.04153681 |
| 6 | 3.36154366 | -2.97530413 | 2.29242897 |
| 1 | 2.79630637 | -2.76817918 | -0.41952559 |
| 1 | 5.01344156 | -0.74565256 | 0.22907583 |
| 1 | 4.97124434 | -3.81785131 | 0.05848410 |
| 1 | 5.28021955 | -3.89433503 | 2.47851181 |
| 1 | 2.76006055 | -1.14320397 | -1.11183059 |
| 1 | 5.05863714 | -1.72366452 | -1.23574960 |
| 1 | 6.37352085 | -2.84550405 | 0.50057143 |
| 1 | 5.30053759 | -2.13415670 | 2.61291456 |
| 1 | 3.14527965 | -3.26389146 | 3.32842135 |
| 1 | 2.82999229 | -3.67261791 | 1.62723005 |
| 8 | 2.19725323 | 0.12291160 | 1.00859380 |
| 8 | 2.78251123 | -1.64623356 | 2.18513870 |
| 1 | 0.37585816 | -2.82481909 | -1.03292572 |
| 1 | 0.27167991 | -3.65442944 | 0.55987376 |
| 31 | 0.32597509 | 0.27481827 | 0.28995904 |

Electronic energy = -3523.852826 a.u.
DFT-D3(BJ) dispersion correction = -0.116858 a.u.
Thermal free energy = 0.604092 a.u.
Gibbs free energy = -3523.365592 a.u.
Number of imaginary frequencies = 1.

**TS1 eq CL isomer/conformer 2**

| Atomic N. | X | Y | Z |
|---|---|---|---|
| 7 | -0.49450606 | 2.40910292 | 0.76652074 |
| 8 | 0.39645150 | 0.59744430 | -1.35590911 |
| 8 | 0.66521001 | -0.12050531 | 1.76498139 |
| 6 | -1.71122146 | 3.12667847 | 0.31612962 |
| 6 | 0.55785680 | 1.74370122 | -2.04514885 |
| 6 | 0.61479956 | 1.70257998 | -3.47189140 |
| 6 | 0.78566879 | 2.91935897 | -4.14651775 |
| 6 | 0.90143114 | 4.14441156 | -3.48227334 |
| 6 | 0.85405099 | 4.16983747 | -2.09225225 |
| 6 | 0.69423854 | 2.98281074 | -1.36978114 |
| 6 | 0.72783339 | 3.00946164 | 0.13279545 |
| 1 | 1.03004539 | 5.06556368 | -4.05074072 |

| | | | |
|---|---|---|---|
| 6 | 0.50310344 | 0.37123966 | -4.24096632 |
| 6 | 1.34869504 | 0.69823545 | 2.59067535 |
| 6 | 2.55245090 | 0.24147804 | 3.21286440 |
| 6 | 3.21123767 | 1.12586784 | 4.07770538 |
| 6 | 2.74620485 | 2.41712523 | 4.34184933 |
| 6 | 1.57904780 | 2.85349154 | 3.72584200 |
| 6 | 0.87336570 | 2.00614262 | 2.86410761 |
| 6 | -0.42138374 | 2.48761511 | 2.26859856 |
| 1 | 3.29703379 | 3.07133818 | 5.01755381 |
| 6 | 3.11464381 | -1.16808569 | 2.94577003 |
| 6 | 0.59002930 | 0.57743126 | -5.76485348 |
| 6 | -0.85373986 | -0.31168592 | -3.94363070 |
| 6 | 1.66092193 | -0.57114202 | -3.83404684 |
| 6 | 4.41279936 | -1.43597603 | 3.73070526 |
| 6 | 3.45120072 | -1.32436144 | 1.44407392 |
| 6 | 2.08691907 | -2.23750925 | 3.38457394 |
| 1 | -1.61794472 | 4.20024300 | 0.55201268 |
| 1 | -1.83466387 | 3.01059723 | -0.76557928 |
| 1 | 0.83512145 | 4.04846764 | 0.48952413 |
| 1 | 1.58809268 | 2.44076800 | 0.51520187 |
| 1 | 0.82829404 | 2.91931319 | -5.23436499 |
| 1 | 0.95409912 | 5.11383486 | -1.55126607 |
| 1 | -1.27514958 | 1.89561951 | 2.63593030 |
| 1 | -0.58660787 | 3.53751183 | 2.56613946 |
| 1 | 4.12976217 | 0.80162048 | 4.56359100 |
| 1 | 1.19616091 | 3.85919237 | 3.91441393 |
| 1 | 0.51187491 | -0.39967507 | -6.26290703 |
| 1 | -0.22584905 | 1.20960987 | -6.14490604 |
| 1 | 1.54700649 | 1.02657700 | -6.06733561 |
| 1 | -0.94605547 | -0.56718969 | -2.88196182 |
| 1 | -1.68842363 | 0.34668472 | -4.22850561 |
| 1 | -0.93863380 | -1.23824418 | -4.53253126 |
| 1 | 2.63223863 | -0.11817520 | -4.08075428 |
| 1 | 1.63860381 | -0.78780341 | -2.76045132 |
| 1 | 1.58060718 | -1.52079153 | -4.38545561 |
| 1 | 4.76329565 | -2.45307946 | 3.50331306 |
| 1 | 4.25935268 | -1.37449479 | 4.81784630 |
| 1 | 5.21704340 | -0.73972988 | 3.45234299 |
| 1 | 2.57314754 | -1.15411234 | 0.81208307 |
| 1 | 4.23062515 | -0.60708630 | 1.14831972 |
| 1 | 3.83294559 | -2.33804035 | 1.24772131 |
| 1 | 1.90648925 | -2.17354441 | 4.46775913 |
| 1 | 1.13094294 | -2.10957646 | 2.86649513 |
| 1 | 2.47617793 | -3.24395299 | 3.16582370 |
| 1 | -2.58392143 | 2.70968056 | 0.82683438 |
| 8 | -1.04120517 | -1.46797311 | -0.14184900 |
| 6 | -0.35884735 | -2.64174700 | 0.25871512 |
| 1 | 0.73025054 | -2.52500510 | 0.14069615 |
| 6 | -3.73034239 | -1.15406859 | -0.70097929 |
| 6 | -3.67335606 | -2.56716466 | -1.29458320 |
| 6 | -4.37983608 | -3.65178752 | -0.47932953 |
| 6 | -2.90876341 | -0.82999617 | 0.53340447 |
| 6 | -3.85216022 | -3.80804563 | 0.94915956 |

| | | | |
|---|---|---|---|
| 6 | -4.05197573 | -2.57795477 | 1.82535470 |
| 1 | -4.77555847 | -0.88693815 | -0.44999889 |
| 1 | -4.13741779 | -2.51958394 | -2.29163933 |
| 1 | -5.46025181 | -3.42770457 | -0.43192023 |
| 1 | -4.39131737 | -4.63230467 | 1.44486189 |
| 1 | -3.40025520 | -0.42880088 | -1.45423806 |
| 1 | -2.61828208 | -2.82750082 | -1.45567000 |
| 1 | -4.28922558 | -4.61349440 | -1.00766194 |
| 1 | -2.78755260 | -4.08771563 | 0.95454121 |
| 1 | -3.95102835 | -2.84174109 | 2.88486028 |
| 1 | -5.05581141 | -2.14714527 | 1.67457926 |
| 8 | -2.43523431 | 0.35268146 | 0.66652161 |
| 8 | -3.07269573 | -1.50604022 | 1.68398297 |
| 1 | -0.55307281 | -2.88976407 | 1.31860411 |
| 1 | -0.67447388 | -3.49271202 | -0.37056729 |
| 31 | -0.44060728 | 0.32663241 | 0.30708814 |

Electronic energy = -3523.849159 a.u.
DFT-D3(BJ) dispersion correction = -0.117743 a.u.
Thermal free energy = 0.603410 a.u.
Gibbs free energy = -3523.363492 a.u.
Number of imaginary frequencies = 1.

**TS1_eq_CL isomer/conformer 3**

| Atomic N. | X | Y | Z |
|---|---|---|---|
| 7 | 2.01693439 | 1.26847649 | 0.85781872 |
| 8 | -0.89464396 | 1.07909214 | 0.97654676 |
| 8 | 0.93278462 | -0.16822986 | -1.45256793 |
| 6 | 2.45306301 | 1.21802843 | 2.27401233 |
| 6 | -0.79239106 | 2.29484153 | 1.56018591 |
| 6 | -1.87974966 | 2.79728961 | 2.34315825 |
| 6 | -1.73103702 | 4.06213808 | 2.92834306 |
| 6 | -0.57800460 | 4.83672094 | 2.77983928 |
| 6 | 0.47097230 | 4.34162045 | 2.01439619 |
| 6 | 0.37098929 | 3.08980918 | 1.39727592 |
| 6 | 1.49256253 | 2.62915897 | 0.50924838 |
| 1 | -0.50646549 | 5.81352425 | 3.25813031 |
| 6 | -3.17592788 | 1.98884356 | 2.54345274 |
| 6 | 1.84095812 | 0.54294825 | -2.14488292 |
| 6 | 1.68208098 | 0.69691944 | -3.55686569 |
| 6 | 2.65932035 | 1.43195534 | -4.24106073 |
| 6 | 3.75701547 | 2.01410532 | -3.59942484 |
| 6 | 3.89870286 | 1.86015391 | -2.22478414 |
| 6 | 2.95997858 | 1.12201357 | -1.49477124 |
| 6 | 3.18294096 | 0.90675569 | -0.02133677 |
| 1 | 4.48793077 | 2.58427477 | -4.17277527 |
| 6 | 0.48059395 | 0.08021996 | -4.30048704 |
| 6 | -4.20983648 | 2.74218416 | 3.40201521 |
| 6 | -2.85764337 | 0.66437024 | 3.27583838 |
| 6 | -3.84076238 | 1.71473825 | 1.17389107 |
| 6 | 0.53624272 | 0.35835567 | -5.81445122 |
| 6 | -0.84043163 | 0.68520975 | -3.76859307 |
| 6 | 0.46624279 | -1.45625591 | -4.11460543 |

| | | | |
|---|---|---|---|
| 1 | 3.22416019 | 1.98525298 | 2.45689607 |
| 1 | 1.59984493 | 1.40768456 | 2.93363118 |
| 1 | 2.32535410 | 3.35184097 | 0.55764741 |
| 1 | 1.16891456 | 2.57884932 | -0.54113644 |
| 1 | -2.54514217 | 4.46295929 | 3.52937651 |
| 1 | 1.38121533 | 4.92958307 | 1.87578619 |
| 1 | 3.40168667 | -0.15007114 | 0.19997144 |
| 1 | 4.05332041 | 1.50366485 | 0.30211678 |
| 1 | 2.56345463 | 1.56534386 | -5.31720304 |
| 1 | 4.74859238 | 2.30614400 | -1.70267069 |
| 1 | -5.11048651 | 2.11926174 | 3.50239229 |
| 1 | -3.83681202 | 2.94844198 | 4.41562462 |
| 1 | -4.51560545 | 3.69319248 | 2.94267988 |
| 1 | -2.11423302 | 0.07231750 | 2.73327613 |
| 1 | -2.46778512 | 0.87056005 | 4.28352928 |
| 1 | -3.77375579 | 0.06324391 | 3.38415956 |
| 1 | -4.14648056 | 2.66028047 | 0.70263636 |
| 1 | -3.15859032 | 1.19865072 | 0.49006328 |
| 1 | -4.74234056 | 1.09746790 | 1.30869472 |
| 1 | -0.33689845 | -0.10554530 | -6.29524851 |
| 1 | 1.43658781 | -0.06775618 | -6.28060722 |
| 1 | 0.50411284 | 1.43422472 | -6.03967285 |
| 1 | -0.97401762 | 0.48045981 | -2.70096064 |
| 1 | -0.85662526 | 1.77418864 | -3.92179346 |
| 1 | -1.69402504 | 0.25427246 | -4.31404972 |
| 1 | 1.38869393 | -1.90246201 | -4.51510191 |
| 1 | 0.37176585 | -1.72748840 | -3.05744863 |
| 1 | -0.38403767 | -1.88838828 | -4.66454840 |
| 1 | 2.85862660 | 0.22442988 | 2.48713541 |
| 8 | -0.61789894 | -1.69972038 | 0.22174492 |
| 6 | -1.98379004 | -1.85081589 | 0.55272073 |
| 1 | -2.53971577 | -0.91904366 | 0.37338209 |
| 6 | 1.41399574 | -3.52339387 | 0.21237303 |
| 6 | 0.42985976 | -4.47270155 | -0.48251733 |
| 6 | -0.05777027 | -5.65183544 | 0.36080578 |
| 6 | 0.91307622 | -2.59501576 | 1.30458641 |
| 6 | -0.77200204 | -5.24407911 | 1.65161192 |
| 6 | 0.11096148 | -4.49347830 | 2.64033151 |
| 1 | 2.25148511 | -4.09451771 | 0.65868473 |
| 1 | 0.93557048 | -4.86594868 | -1.37768555 |
| 1 | 0.80042416 | -6.29600143 | 0.62232894 |
| 1 | -1.66983140 | -4.64355040 | 1.43879938 |
| 1 | 1.86003077 | -2.85618711 | -0.53571349 |
| 1 | -0.41929004 | -3.87498403 | -0.84202534 |
| 1 | -0.73316646 | -6.27293968 | -0.24772182 |
| 1 | -1.12158585 | -6.15006304 | 2.17408037 |
| 1 | -0.33947632 | -4.50216866 | 3.64010715 |
| 1 | 1.10722446 | -4.96062708 | 2.71015167 |
| 8 | 1.47935796 | -1.45455968 | 1.46190500 |
| 8 | 0.29544646 | -3.06567526 | 2.40230894 |
| 1 | -2.43505430 | -2.64564610 | -0.06795637 |
| 1 | -2.11687946 | -2.11720896 | 1.61853683 |
| 31 | 0.45711342 | -0.08970320 | 0.36643809 |

Electronic energy = -3523.848634 a.u.
DFT-D3(BJ) dispersion correction = -0.117778 a.u.
Thermal free energy = 0.603657 a.u.
Gibbs free energy = -3523.362755 a.u.
Number of imaginary frequencies = 1.

**Int1a CL isomer/conformer 1**

| Atomic N. | X | Y | Z |
|---|---|---|---|
| 8 | 0.39972872 | -1.87844105 | 0.32644548 |
| 6 | 1.63482270 | -2.45820679 | 0.76822383 |
| 1 | 2.42908138 | -1.96162776 | 0.19975282 |
| 8 | -1.66344294 | -1.20114962 | 0.80188879 |
| 1 | 1.78897651 | -2.29077597 | 1.84211546 |
| 1 | 1.65417288 | -3.53608781 | 0.54183152 |
| 7 | -1.83614450 | 1.74324651 | 0.49886223 |
| 8 | -0.01772884 | 0.47506059 | -1.47612614 |
| 8 | 0.79646145 | 0.79388797 | 1.53215440 |
| 6 | -3.24961658 | 1.29366945 | 0.45641937 |
| 6 | -0.86900645 | 1.03121062 | -2.37100225 |
| 6 | -0.89928359 | 0.55604000 | -3.71592383 |
| 6 | -1.80206908 | 1.17123580 | -4.59543219 |
| 6 | -2.64622932 | 2.21600574 | -4.20887942 |
| 6 | -2.58578329 | 2.68968786 | -2.90251864 |
| 6 | -1.69719760 | 2.11719725 | -1.98645431 |
| 6 | -1.55063987 | 2.71259678 | -0.61299242 |
| 1 | -3.33507448 | 2.65797116 | -4.92881143 |
| 6 | 0.03054938 | -0.57641744 | -4.19499688 |
| 6 | 0.95619599 | 2.09731039 | 1.85602645 |
| 6 | 2.26328984 | 2.62020546 | 2.09161936 |
| 6 | 2.36238529 | 3.97321470 | 2.44432593 |
| 6 | 1.24803492 | 4.80830432 | 2.56799499 |
| 6 | -0.02055425 | 4.28559789 | 2.34249300 |
| 6 | -0.17676759 | 2.93784004 | 1.99936712 |
| 6 | -1.56062794 | 2.36743119 | 1.84320842 |
| 1 | 1.37685266 | 5.85634990 | 2.83814744 |
| 6 | 3.51943003 | 1.73633560 | 1.96451612 |
| 6 | -0.17024370 | -0.89493440 | -5.68857799 |
| 6 | -0.25078124 | -1.87513360 | -3.40394636 |
| 6 | 1.50860162 | -0.15845132 | -4.00705179 |
| 6 | 4.81093426 | 2.51304056 | 2.28233057 |
| 6 | 3.64962115 | 1.20693043 | 0.51672357 |
| 6 | 3.43579679 | 0.55658128 | 2.96200879 |
| 1 | -3.92069162 | 2.15771892 | 0.59584387 |
| 1 | -3.45794493 | 0.82804624 | -0.51244877 |
| 1 | -2.22217100 | 3.58163725 | -0.50243220 |
| 1 | -0.52063984 | 3.06404244 | -0.45360211 |
| 1 | -1.85513255 | 0.82339105 | -5.62552390 |
| 1 | -3.21684558 | 3.52287727 | -2.58448924 |
| 1 | -1.74002982 | 1.56879845 | 2.58101976 |
| 1 | -2.30839245 | 3.16074210 | 2.01389734 |
| 1 | 3.34717455 | 4.40045006 | 2.62526549 |
| 1 | -0.90603866 | 4.91765846 | 2.44128166 |

| | | | |
|---|---|---|---|
| 1 | 0.52268506 | -1.69789652 | -5.97815668 |
| 1 | -1.19031540 | -1.24452982 | -5.90489192 |
| 1 | 0.04328875 | -0.02716587 | -6.32919364 |
| 1 | -0.05404862 | -1.74166849 | -2.33487268 |
| 1 | -1.29686844 | -2.18993876 | -3.53515944 |
| 1 | 0.39521693 | -2.68489371 | -3.77722902 |
| 1 | 1.73518327 | 0.73780498 | -4.60274722 |
| 1 | 1.73349553 | 0.05555954 | -2.95636017 |
| 1 | 2.17087691 | -0.96836979 | -4.34988670 |
| 1 | 5.66948907 | 1.83320392 | 2.18401722 |
| 1 | 4.81577862 | 2.90503762 | 3.30966921 |
| 1 | 4.97244634 | 3.35082129 | 1.58872842 |
| 1 | 2.77234638 | 0.62384218 | 0.21797709 |
| 1 | 3.76331658 | 2.04052410 | -0.19119466 |
| 1 | 4.54192345 | 0.56764307 | 0.43148980 |
| 1 | 3.41580830 | 0.92877806 | 3.99677992 |
| 1 | 2.53681594 | -0.04621459 | 2.79496126 |
| 1 | 4.32155970 | -0.08834364 | 2.85311444 |
| 1 | -3.41611256 | 0.55570518 | 1.24779045 |
| 31 | -0.46970337 | 0.16953247 | 0.31070038 |
| 6 | -1.42049528 | -3.54353653 | 0.29245477 |
| 6 | -2.75658226 | -4.06284655 | 0.84867056 |
| 6 | -2.60765299 | -4.99078334 | 2.06065067 |
| 6 | -0.88449042 | -2.30176876 | 1.01151432 |
| 6 | -1.94583298 | -4.34553604 | 3.28267874 |
| 6 | -0.57022897 | -3.73639025 | 3.01135143 |
| 1 | -0.66596075 | -4.34682840 | 0.29925742 |
| 1 | -3.39901108 | -3.20316947 | 1.09579757 |
| 1 | -2.01273990 | -5.87096287 | 1.75881284 |
| 1 | -2.58813910 | -3.55333911 | 3.69982125 |
| 1 | -1.54034135 | -3.23031576 | -0.75256392 |
| 1 | -3.27265956 | -4.61510086 | 0.04845518 |
| 1 | -3.59870751 | -5.37628790 | 2.34826488 |
| 1 | -1.82774026 | -5.10561475 | 4.07325152 |
| 1 | -0.05310062 | -3.54397471 | 3.96195795 |
| 1 | 0.06074984 | -4.42915832 | 2.42807463 |
| 8 | -0.63328169 | -2.43490556 | 2.39346023 |

Electronic energy = -3523.860859 a.u.
DFT-D3(BJ) dispersion correction = -0.116852 a.u.
Thermal free energy = 0.605097 a.u.
Gibbs free energy = -3523.372613 a.u.
Number of imaginary frequencies = 0.

**Int1a CL isomer/conformer 2**

| Atomic N. | X | Y | Z |
|---|---|---|---|
| 7 | -1.97993021 | 1.25845425 | 1.08551315 |
| 8 | 0.11661927 | 1.15135602 | -1.02527748 |
| 8 | 0.58660587 | -0.12261698 | 1.77771460 |
| 6 | -3.35585610 | 0.94071449 | 0.63066167 |
| 6 | -0.63105716 | 2.13209519 | -1.58453833 |
| 6 | -0.46976398 | 2.44598418 | -2.96715570 |
| 6 | -1.27642786 | 3.46430501 | -3.49511060 |

| | | | |
|---|---|---|---|
| 6 | -2.20466348 | 4.17234355 | -2.72652083 |
| 6 | -2.32900403 | 3.87921728 | -1.37266818 |
| 6 | -1.54227933 | 2.87846615 | -0.79231904 |
| 6 | -1.59599577 | 2.65759786 | 0.69528567 |
| 1 | -2.81393924 | 4.95155281 | -3.18449511 |
| 6 | 0.56041963 | 1.71045678 | -3.84718817 |
| 6 | 0.61791375 | 0.79392969 | 2.77113331 |
| 6 | 1.85218624 | 1.09273379 | 3.42419085 |
| 6 | 1.82385251 | 2.03819070 | 4.45842883 |
| 6 | 0.65298050 | 2.68664066 | 4.86172864 |
| 6 | -0.54371694 | 2.38352274 | 4.22274164 |
| 6 | -0.57481910 | 1.43738662 | 3.19155323 |
| 6 | -1.89803839 | 1.06465966 | 2.57823890 |
| 1 | 0.68241056 | 3.41999935 | 5.66747544 |
| 6 | 3.16818981 | 0.40427459 | 3.01289727 |
| 6 | 0.56972021 | 2.24342810 | -5.29230524 |
| 6 | 0.23254915 | 0.20047756 | -3.91355379 |
| 6 | 1.98264471 | 1.91337685 | -3.27185229 |
| 6 | 4.36031826 | 0.87357733 | 3.86797440 |
| 6 | 3.50336710 | 0.73801185 | 1.53996451 |
| 6 | 3.04291806 | -1.12616547 | 3.20223201 |
| 1 | -4.07570865 | 1.62090229 | 1.11595206 |
| 1 | -3.42086692 | 1.06269064 | -0.45555832 |
| 1 | -2.31092922 | 3.36040951 | 1.15690375 |
| 1 | -0.61018754 | 2.83979633 | 1.14803306 |
| 1 | -1.18302646 | 3.71833729 | -4.54946665 |
| 1 | -3.02772542 | 4.43850213 | -0.74608865 |
| 1 | -2.12398747 | -0.00060735 | 2.74823130 |
| 1 | -2.70046370 | 1.65923536 | 3.04731389 |
| 1 | 2.75145451 | 2.28773789 | 4.97050380 |
| 1 | -1.47268368 | 2.87170879 | 4.52621550 |
| 1 | 1.32821524 | 1.69552665 | -5.86959068 |
| 1 | -0.39756674 | 2.09727851 | -5.79482945 |
| 1 | 0.82752993 | 3.31136620 | -5.33778459 |
| 1 | 0.27851958 | -0.26140006 | -2.92153227 |
| 1 | -0.77203318 | 0.04018027 | -4.33268746 |
| 1 | 0.95713991 | -0.30882305 | -4.56765079 |
| 1 | 2.24686231 | 2.98088835 | -3.26306624 |
| 1 | 2.05971654 | 1.52709168 | -2.24954404 |
| 1 | 2.71748704 | 1.38868547 | -3.90176178 |
| 1 | 5.26662276 | 0.34673339 | 3.53637284 |
| 1 | 4.21857784 | 0.64799296 | 4.93480897 |
| 1 | 4.54792804 | 1.95179271 | 3.76163363 |
| 1 | 2.70596610 | 0.41907538 | 0.86068416 |
| 1 | 3.64850245 | 1.82051064 | 1.41262117 |
| 1 | 4.43785332 | 0.23545215 | 1.24625270 |
| 1 | 2.87471172 | -1.36991802 | 4.26147754 |
| 1 | 2.21167079 | -1.53501143 | 2.61809617 |
| 1 | 3.97547891 | -1.61941579 | 2.88698196 |
| 1 | -3.58818522 | -0.09821251 | 0.88598407 |
| 8 | 0.41716926 | -1.77325027 | -0.65174528 |
| 6 | 1.63260488 | -2.50264528 | -0.43451927 |
| 1 | 2.44993224 | -1.78300497 | -0.55592807 |

| | | | |
|---|---|---|---|
| 6 | -1.29890128 | -3.14824413 | -1.76482328 |
| 6 | -2.64461069 | -3.89046238 | -1.72332203 |
| 6 | -2.54613437 | -5.32389462 | -1.18668070 |
| 6 | -0.90854212 | -2.48244470 | -0.44151434 |
| 6 | -2.04606632 | -5.43279232 | 0.25756882 |
| 6 | -0.69773099 | -4.76027112 | 0.51476107 |
| 1 | -0.49920944 | -3.82778839 | -2.10176711 |
| 1 | -3.36050245 | -3.30222485 | -1.12801553 |
| 1 | -1.86714923 | -5.89834284 | -1.84148152 |
| 1 | -2.77824486 | -4.99678834 | 0.95609709 |
| 1 | -1.33998591 | -2.32492032 | -2.48943068 |
| 1 | -3.04978262 | -3.92845495 | -2.74608278 |
| 1 | -3.53084383 | -5.81137679 | -1.26534991 |
| 1 | -1.94773197 | -6.49792620 | 0.52690667 |
| 1 | -0.28707295 | -5.10190393 | 1.47534217 |
| 1 | 0.03266261 | -5.02660834 | -0.26855079 |
| 8 | -1.71473572 | -1.42843427 | -0.12529479 |
| 8 | -0.79553607 | -3.33038525 | 0.67909181 |
| 1 | 1.66310226 | -2.92837387 | 0.57684563 |
| 1 | 1.74175422 | -3.29779552 | -1.18895985 |
| 31 | -0.53252152 | -0.01414335 | 0.28359767 |

Electronic energy = -3523.860949 a.u.
DFT-D3(BJ) dispersion correction = -0.116749 a.u.
Thermal free energy = 0.605210 a.u.
Gibbs free energy = -3523.372488 a.u.
Number of imaginary frequencies = 0.

**Int1a CL isomer/conformer 3**

| Atomic N. | X | Y | Z |
|---|---|---|---|
| 7 | -0.61109115 | 2.43536031 | 0.82058996 |
| 8 | 0.64338991 | 0.71122392 | -1.25789539 |
| 8 | 0.58341246 | -0.15783805 | 1.72818863 |
| 6 | -1.93257395 | 3.00706658 | 0.46359791 |
| 6 | 0.59536005 | 1.84800530 | -1.99282966 |
| 6 | 0.71645234 | 1.78150767 | -3.41290197 |
| 6 | 0.65374008 | 2.98966286 | -4.12204177 |
| 6 | 0.48945793 | 4.22900687 | -3.49701547 |
| 6 | 0.40329499 | 4.28335631 | -2.11001406 |
| 6 | 0.46947505 | 3.10879266 | -1.35280997 |
| 6 | 0.50074041 | 3.19189317 | 0.14928873 |
| 1 | 0.44015811 | 5.14040888 | -4.09284749 |
| 6 | 0.92099450 | 0.43986020 | -4.14407903 |
| 6 | 1.32496736 | 0.65099531 | 2.51759963 |
| 6 | 2.54649440 | 0.16868055 | 3.07747186 |
| 6 | 3.26677663 | 1.04180197 | 3.90395383 |
| 6 | 2.83951920 | 2.34205679 | 4.18932281 |
| 6 | 1.64539259 | 2.79853647 | 3.64346732 |
| 6 | 0.87927543 | 1.96338682 | 2.82157707 |
| 6 | -0.45396262 | 2.45060461 | 2.31984083 |
| 1 | 3.43832176 | 2.98663692 | 4.83265536 |
| 6 | 3.05774075 | -1.25580248 | 2.78616661 |
| 6 | 1.05626926 | 0.62763834 | -5.66687192 |

| | | | |
|---|---|---|---|
| 6 | -0.28679052 | -0.49453474 | -3.89897825 |
| 6 | 2.21984747 | -0.23842935 | -3.64647337 |
| 6 | 4.37949680 | -1.55670980 | 3.51756997 |
| 6 | 3.32461945 | -1.42260017 | 1.27140459 |
| 6 | 2.02062830 | -2.29977851 | 3.26447431 |
| 1 | -1.99839164 | 4.04854795 | 0.82022386 |
| 1 | -2.05819406 | 2.98964842 | -0.62403266 |
| 1 | 0.45253890 | 4.24633656 | 0.47160566 |
| 1 | 1.43637505 | 2.76586235 | 0.54058015 |
| 1 | 0.73270599 | 2.96892612 | -5.20750029 |
| 1 | 0.30173228 | 5.24300453 | -1.59780303 |
| 1 | -1.27220794 | 1.82129329 | 2.70657557 |
| 1 | -0.62552735 | 3.48151048 | 2.67371273 |
| 1 | 4.20371578 | 0.70155916 | 4.34135448 |
| 1 | 1.28776381 | 3.80790642 | 3.85954461 |
| 1 | 1.21259123 | -0.35488966 | -6.13478486 |
| 1 | 0.15171006 | 1.06595579 | -6.11311471 |
| 1 | 1.91613658 | 1.26006203 | -5.93096116 |
| 1 | -0.40326449 | -0.72558594 | -2.83474240 |
| 1 | -1.21592724 | -0.03083522 | -4.26223716 |
| 1 | -0.14309545 | -1.43871982 | -4.44698517 |
| 1 | 3.09227182 | 0.39759510 | -3.85586554 |
| 1 | 2.18244995 | -0.43398359 | -2.56920217 |
| 1 | 2.36528774 | -1.19469555 | -4.17225339 |
| 1 | 4.69248380 | -2.58371329 | 3.28076677 |
| 1 | 4.27288907 | -1.48713516 | 4.60981208 |
| 1 | 5.19045678 | -0.88405865 | 3.20291985 |
| 1 | 2.42144584 | -1.24205438 | 0.67906393 |
| 1 | 4.10109832 | -0.72023827 | 0.93543109 |
| 1 | 3.68105215 | -2.44370504 | 1.06560555 |
| 1 | 1.87190572 | -2.22465741 | 4.35167158 |
| 1 | 1.05224630 | -2.15951218 | 2.77254864 |
| 1 | 2.38601165 | -3.31481546 | 3.04451640 |
| 1 | -2.72216733 | 2.40240557 | 0.92174674 |
| 8 | -0.90725981 | -1.66557897 | -0.34544580 |
| 6 | -0.39528600 | -2.99758252 | -0.15100907 |
| 1 | 0.67023949 | -2.94310356 | -0.40002135 |
| 6 | -3.30612594 | -1.77134363 | -1.04837296 |
| 6 | -3.95933981 | -3.15506014 | -0.90599902 |
| 6 | -5.10104076 | -3.19964198 | 0.12186376 |
| 6 | -2.29326895 | -1.33959361 | 0.03550664 |
| 6 | -4.65708989 | -3.06489595 | 1.58293351 |
| 6 | -3.78380493 | -1.84656273 | 1.87081469 |
| 1 | -4.09189769 | -1.00087212 | -1.06605509 |
| 1 | -4.37014135 | -3.43190259 | -1.88992941 |
| 1 | -5.81638082 | -2.39308331 | -0.11683769 |
| 1 | -5.54559019 | -3.01377773 | 2.23507480 |
| 1 | -2.77901704 | -1.68553714 | -2.00907659 |
| 1 | -3.20862580 | -3.92241551 | -0.66141057 |
| 1 | -5.65516496 | -4.14480453 | 0.00651969 |
| 1 | -4.09395938 | -3.96171073 | 1.88903466 |
| 1 | -3.61437172 | -1.75183691 | 2.95334198 |
| 1 | -4.25601461 | -0.91083367 | 1.52753764 |

| | | | |
|---|---|---|---|
| 8 | -2.24141409 | 0.03108217 | 0.12722157 |
| 8 | -2.47092364 | -1.98024850 | 1.28990338 |
| 1 | -0.52326304 | -3.32123857 | 0.88820866 |
| 1 | -0.89116606 | -3.69947474 | -0.83682772 |
| 31 | -0.40659337 | 0.43721341 | 0.26241610 |

Electronic energy = -3523.860347 a.u.
DFT-D3(BJ) dispersion correction = -0.116515 a.u.
Thermal free energy = 0.604901 a.u.
Gibbs free energy = -3523.371962 a.u.
Number of imaginary frequencies = 0.

**Int1a CL isomer/conformer 4**

| Atomic N. | X | Y | Z |
|---|---|---|---|
| 7 | 2.29340735 | 1.14190520 | 0.68192689 |
| 8 | -0.68579017 | 1.01276276 | 0.59667401 |
| 8 | 1.07885254 | -0.08166360 | -1.74384471 |
| 6 | 2.83476375 | 0.79585307 | 2.01852643 |
| 6 | -0.59015631 | 2.03169517 | 1.48445945 |
| 6 | -1.72398325 | 2.37969969 | 2.28030150 |
| 6 | -1.57562209 | 3.43557464 | 3.19054927 |
| 6 | -0.38233993 | 4.14720532 | 3.34015999 |
| 6 | 0.70603917 | 3.81750625 | 2.54085905 |
| 6 | 0.61012382 | 2.78111599 | 1.60495999 |
| 6 | 1.75780615 | 2.54494922 | 0.66003900 |
| 1 | -0.31341308 | 4.95601866 | 4.06756907 |
| 6 | -3.06469858 | 1.62904684 | 2.15372474 |
| 6 | 1.85834903 | 0.80989073 | -2.38985146 |
| 6 | 1.55771775 | 1.15786518 | -3.74173674 |
| 6 | 2.40863601 | 2.07182940 | -4.37749525 |
| 6 | 3.51458111 | 2.64609424 | -3.74307193 |
| 6 | 3.79789465 | 2.29898959 | -2.42712652 |
| 6 | 2.98909486 | 1.37916383 | -1.74908682 |
| 6 | 3.37139743 | 0.95413226 | -0.35593781 |
| 1 | 4.14360522 | 3.35820797 | -4.27706349 |
| 6 | 0.34189346 | 0.55616384 | -4.47385822 |
| 6 | -4.15570408 | 2.23742854 | 3.05558034 |
| 6 | -2.88137105 | 0.15520576 | 2.58264116 |
| 6 | -3.58703612 | 1.70158356 | 0.69890825 |
| 6 | 0.23468365 | 1.06158723 | -5.92498098 |
| 6 | -0.96449513 | 0.95976766 | -3.74956096 |
| 6 | 0.46123734 | -0.98564199 | -4.53178212 |
| 1 | 3.67983019 | 1.46030160 | 2.26438943 |
| 1 | 2.05119289 | 0.91690511 | 2.77411023 |
| 1 | 2.58326448 | 3.23962695 | 0.89224235 |
| 1 | 1.44990767 | 2.73531619 | -0.37899578 |
| 1 | -2.42234160 | 3.71640872 | 3.81411931 |
| 1 | 1.64107923 | 4.37692944 | 2.62014416 |
| 1 | 3.62468774 | -0.11831198 | -0.32550259 |
| 1 | 4.26190070 | 1.51950213 | -0.03233730 |
| 1 | 2.20242549 | 2.35677643 | -5.40766789 |
| 1 | 4.65979457 | 2.73150101 | -1.91373616 |
| 1 | -5.08962006 | 1.67504191 | 2.91281218 |

| | | | |
|---|---|---|---|
| 1 | -3.89568682 | 2.17695437 | 4.12229431 |
| 1 | -4.36039374 | 3.28868205 | 2.80600079 |
| 1 | -2.12215064 | -0.35524229 | 1.98176322 |
| 1 | -2.57854984 | 0.09773154 | 3.63880054 |
| 1 | -3.83420386 | -0.38668766 | 2.47607412 |
| 1 | -3.75921588 | 2.74661860 | 0.40224728 |
| 1 | -2.87926468 | 1.25418745 | -0.00669379 |
| 1 | -4.54678008 | 1.16765810 | 0.62298902 |
| 1 | -0.64209052 | 0.59900893 | -6.40033470 |
| 1 | 1.11673558 | 0.79079719 | -6.52327369 |
| 1 | 0.10075197 | 2.15183414 | -5.97575675 |
| 1 | -0.97750918 | 0.60517719 | -2.71335975 |
| 1 | -1.08141095 | 2.05316623 | -3.74175639 |
| 1 | -1.83057085 | 0.53122285 | -4.27707601 |
| 1 | 1.36578211 | -1.28234251 | -5.08282834 |
| 1 | 0.50590673 | -1.42275541 | -3.52840505 |
| 1 | -0.40811775 | -1.40527885 | -5.06116597 |
| 1 | 3.16693925 | -0.24768163 | 2.01254986 |
| 8 | -0.52017558 | -1.90413581 | -0.24415821 |
| 6 | -1.85739469 | -2.33597386 | -0.55281784 |
| 1 | -2.25720018 | -1.59098670 | -1.24954358 |
| 6 | 0.82046706 | -3.91416835 | 0.40042150 |
| 6 | -0.00865683 | -5.16903634 | 0.71600593 |
| 6 | 0.09571625 | -5.63076715 | 2.17834156 |
| 6 | 0.24775749 | -2.54572666 | 0.83437864 |
| 6 | -0.59044767 | -4.71066054 | 3.19458933 |
| 6 | -0.16528842 | -3.24721223 | 3.11249416 |
| 1 | 1.80838056 | -4.00463434 | 0.87706438 |
| 1 | 0.35924599 | -5.98236798 | 0.07049347 |
| 1 | 1.16458488 | -5.72617651 | 2.43856042 |
| 1 | -1.68383696 | -4.75119409 | 3.06045398 |
| 1 | 1.01292585 | -3.83281149 | -0.67906263 |
| 1 | -1.06615122 | -5.02006314 | 0.44788534 |
| 1 | -0.33356132 | -6.64119614 | 2.26996223 |
| 1 | -0.38548761 | -5.07254695 | 4.21650520 |
| 1 | -0.60169680 | -2.67937297 | 3.94713892 |
| 1 | 0.92977514 | -3.13178303 | 3.17187436 |
| 8 | 1.26020055 | -1.63712822 | 1.03235139 |
| 8 | -0.66548458 | -2.61299468 | 1.91801511 |
| 1 | -1.83584169 | -3.31860193 | -1.04650470 |
| 1 | -2.47612742 | -2.37439788 | 0.35154360 |
| 31 | 0.73053153 | -0.09304778 | 0.09087868 |

Electronic energy = -3523.859697 a.u.
DFT-D3(BJ) dispersion correction = -0.116374 a.u.
Thermal free energy = 0.604399 a.u.
Gibbs free energy = -3523.371672 a.u.
Number of imaginary frequencies = 0.

**Int1a CL isomer/conformer 5**

| Atomic N. | X | Y | Z |
|---|---|---|---|
| 7 | 0.68144204 | 2.47825725 | 0.08871399 |
| 8 | -1.11484891 | 0.22813436 | 0.84239424 |

| | | | |
|---|---|---|---|
| 8 | 0.35390479 | 0.34318352 | -1.94586263 |
| 6 | 1.51066449 | 2.89337622 | 1.24624083 |
| 6 | -1.67128129 | 1.16221286 | 1.64998698 |
| 6 | -2.50551624 | 0.74672707 | 2.73300081 |
| 6 | -3.06071217 | 1.74602660 | 3.54414716 |
| 6 | -2.83035971 | 3.10818374 | 3.33516386 |
| 6 | -2.03122314 | 3.50181846 | 2.26830799 |
| 6 | -1.46359631 | 2.54742908 | 1.41620819 |
| 6 | -0.71544333 | 3.01792163 | 0.19786353 |
| 1 | -3.27814267 | 3.84870031 | 3.99783831 |
| 6 | -2.78361841 | -0.74296706 | 3.01605200 |
| 6 | 0.10364857 | 1.38164215 | -2.76862096 |
| 6 | -0.58488700 | 1.15252984 | -3.99874184 |
| 6 | -0.80622820 | 2.25813901 | -4.83064272 |
| 6 | -0.38926812 | 3.55122710 | -4.49984915 |
| 6 | 0.28046462 | 3.76197027 | -3.30002891 |
| 6 | 0.54138362 | 2.69017925 | -2.43799798 |
| 6 | 1.34224485 | 2.93551519 | -1.18649677 |
| 1 | -0.59073114 | 4.38161647 | -5.17645108 |
| 6 | -1.06982914 | -0.25575963 | -4.39625956 |
| 6 | -3.77057350 | -0.93755368 | 4.18300418 |
| 6 | -1.46504265 | -1.44996690 | 3.40651066 |
| 6 | -3.41215277 | -1.42130763 | 1.77542767 |
| 6 | -1.76185392 | -0.25975177 | -5.77248547 |
| 6 | -2.09923339 | -0.77246894 | -3.36311373 |
| 6 | 0.12912272 | -1.23045985 | -4.48181946 |
| 1 | 1.59281444 | 3.99275634 | 1.27389047 |
| 1 | 1.04677884 | 2.54396529 | 2.17467881 |
| 1 | -0.66823265 | 4.12049648 | 0.19123996 |
| 1 | -1.23407271 | 2.70687382 | -0.72140339 |
| 1 | -3.69619108 | 1.45486112 | 4.37843554 |
| 1 | -1.85272437 | 4.56156132 | 2.07194613 |
| 1 | 2.30787969 | 2.40489090 | -1.22357679 |
| 1 | 1.55549680 | 4.01427282 | -1.09535449 |
| 1 | -1.33017748 | 2.11302542 | -5.77372558 |
| 1 | 0.61867142 | 4.76327637 | -3.02336895 |
| 1 | -3.94114431 | -2.01392437 | 4.32919657 |
| 1 | -3.38135878 | -0.53513730 | 5.12933145 |
| 1 | -4.74611911 | -0.47188895 | 3.98130834 |
| 1 | -0.70094089 | -1.34695618 | 2.62920811 |
| 1 | -1.06231078 | -1.02447642 | 4.33739490 |
| 1 | -1.65160163 | -2.52193221 | 3.57862120 |
| 1 | -4.37269405 | -0.94845563 | 1.52345546 |
| 1 | -2.75548892 | -1.35375463 | 0.90199687 |
| 1 | -3.60678390 | -2.48313614 | 1.99177466 |
| 1 | -2.08162715 | -1.28482215 | -6.00844857 |
| 1 | -1.08663851 | 0.06942707 | -6.57560882 |
| 1 | -2.65803309 | 0.37725738 | -5.78814705 |
| 1 | -1.66735350 | -0.82956367 | -2.35813705 |
| 1 | -2.97649166 | -0.11037020 | -3.32557438 |
| 1 | -2.44586488 | -1.77651088 | -3.65254714 |
| 1 | 0.84798342 | -0.89242668 | -5.24266265 |
| 1 | 0.64983157 | -1.31102036 | -3.52153224 |

| | | | |
|---|---|---|---|
| 1 | -0.22623766 | -2.23037972 | -4.77514748 |
| 1 | 2.50497749 | 2.44382933 | 1.15469706 |
| 8 | 0.96224241 | -1.72198798 | -0.11438487 |
| 6 | 0.28091065 | -2.96525298 | 0.09551775 |
| 1 | -0.62241866 | -2.92953987 | -0.52385089 |
| 6 | 3.40462946 | -2.00318049 | 0.00441687 |
| 6 | 4.74640353 | -1.72443154 | 0.70069661 |
| 6 | 5.07493746 | -2.71018134 | 1.82893849 |
| 6 | 2.19058852 | -1.39383050 | 0.71352500 |
| 6 | 4.07220721 | -2.71408588 | 2.98766842 |
| 6 | 2.62378757 | -2.97428506 | 2.57422107 |
| 1 | 3.26444082 | -3.08817095 | -0.13009053 |
| 1 | 4.74622260 | -0.68979863 | 1.07818446 |
| 1 | 5.12771021 | -3.72752965 | 1.40231184 |
| 1 | 4.36054481 | -3.49447509 | 3.71191402 |
| 1 | 3.39262412 | -1.55595173 | -0.99799582 |
| 1 | 5.54600821 | -1.77946089 | -0.05382879 |
| 1 | 6.08031231 | -2.49189846 | 2.22269081 |
| 1 | 4.10019538 | -1.75499824 | 3.52929782 |
| 1 | 2.02207874 | -3.22644736 | 3.45873499 |
| 1 | 2.55818695 | -3.82761589 | 1.87722457 |
| 8 | 2.17697404 | -0.03055927 | 0.66034945 |
| 8 | 1.96356611 | -1.80784869 | 2.04079527 |
| 1 | 0.90902220 | -3.80694406 | -0.23653668 |
| 1 | -0.00027339 | -3.09148416 | 1.14930936 |
| 31 | 0.49841849 | 0.41366567 | -0.08354033 |

Electronic energy = -3523.860039 a.u.
DFT-D3(BJ) dispersion correction = -0.116576 a.u.
Thermal free energy = 0.605635 a.u.
Gibbs free energy = -3523.370979 a.u.
Number of imaginary frequencies = 0.

**Int1b CL isomer/conformer 1**

| Atomic N. | X | Y | Z |
|---|---|---|---|
| 8 | -1.57017963 | -1.87020267 | 3.16364476 |
| 6 | -0.42935339 | -1.35617178 | 3.87417149 |
| 1 | 0.46705658 | -1.30990620 | 3.23787240 |
| 6 | -3.34522207 | -1.58235121 | 1.60396305 |
| 6 | -3.65912412 | -3.08005510 | 1.53030465 |
| 6 | -2.79117955 | -3.89725144 | 0.55938807 |
| 6 | -1.91240908 | -1.19855833 | 1.98173084 |
| 6 | -1.34098123 | -4.07335992 | 1.03807657 |
| 6 | -0.39729378 | -2.91046336 | 0.72584540 |
| 1 | -3.54385524 | -1.10067617 | 0.63599203 |
| 1 | -4.71530577 | -3.18088023 | 1.23404515 |
| 1 | -3.24374594 | -4.89575831 | 0.45635734 |
| 1 | -0.89308112 | -4.96045372 | 0.56119016 |
| 1 | -3.99909808 | -1.10177546 | 2.34698600 |
| 1 | -3.57542525 | -3.52011662 | 2.53661174 |
| 1 | -2.81092640 | -3.44324388 | -0.44527610 |
| 1 | -1.34681982 | -4.26755217 | 2.12010810 |
| 1 | 0.49950562 | -2.96358029 | 1.36212803 |

| | | | |
|---|---|---|---|
| 1 | -0.07395070 | -2.95237318 | -0.32427841 |
| 8 | -1.71399121 | 0.15438288 | 2.00470594 |
| 8 | -0.98766040 | -1.58982798 | 0.84023981 |
| 1 | -0.26167439 | -2.05824550 | 4.69877364 |
| 1 | -0.63807501 | -0.35471213 | 4.27525236 |
| 7 | -0.20821341 | 2.52331871 | 1.11367671 |
| 8 | -0.56640465 | 0.57251731 | -1.10271279 |
| 8 | 1.42521714 | 0.02143129 | 1.24086040 |
| 6 | -1.47672434 | 3.10421024 | 1.61856059 |
| 6 | -1.06530634 | 1.65083189 | -1.75292409 |
| 6 | -1.85521815 | 1.46374324 | -2.92688092 |
| 6 | -2.36207954 | 2.61130574 | -3.55315954 |
| 6 | -2.11424171 | 3.90406135 | -3.08359004 |
| 6 | -1.31128839 | 4.07492981 | -1.96125648 |
| 6 | -0.77250061 | 2.96443866 | -1.30228490 |
| 6 | 0.20259068 | 3.17428162 | -0.17579470 |
| 1 | -2.53673730 | 4.76529991 | -3.60106973 |
| 6 | -2.12798928 | 0.05975698 | -3.50230506 |
| 6 | 2.45383344 | 0.89792362 | 1.30825781 |
| 6 | 3.77448514 | 0.47321123 | 0.96960689 |
| 6 | 4.80552692 | 1.41594917 | 1.08476702 |
| 6 | 4.58794643 | 2.73149791 | 1.50351860 |
| 6 | 3.29751779 | 3.13502645 | 1.82554489 |
| 6 | 2.23225108 | 2.23074116 | 1.74187090 |
| 6 | 0.85914220 | 2.68007698 | 2.16586031 |
| 1 | 5.42180730 | 3.43002594 | 1.57122156 |
| 6 | 4.06510267 | -0.96244224 | 0.48905269 |
| 6 | -2.97762662 | 0.11654422 | -4.78577672 |
| 6 | -2.89847109 | -0.80250894 | -2.47626734 |
| 6 | -0.78964228 | -0.62735363 | -3.86533232 |
| 6 | 5.55932018 | -1.17890542 | 0.18304081 |
| 6 | 3.28629148 | -1.25234037 | -0.81593334 |
| 6 | 3.67151223 | -1.97933516 | 1.58573840 |
| 1 | -1.34000876 | 4.17947110 | 1.82215162 |
| 1 | -2.26324062 | 2.97801809 | 0.86723212 |
| 1 | 0.34414568 | 4.25395508 | 0.00455528 |
| 1 | 1.18446351 | 2.74978658 | -0.43228556 |
| 1 | -2.97807001 | 2.49790815 | -4.44335558 |
| 1 | -1.07786862 | 5.07701073 | -1.59401735 |
| 1 | 0.50243566 | 2.10324430 | 3.03461898 |
| 1 | 0.89793295 | 3.74220775 | 2.46227259 |
| 1 | 5.82172185 | 1.11973289 | 0.83138477 |
| 1 | 3.10334523 | 4.15820115 | 2.15520231 |
| 1 | -3.12913055 | -0.90647492 | -5.15886582 |
| 1 | -3.97082615 | 0.55362094 | -4.60700773 |
| 1 | -2.48381651 | 0.68938769 | -5.58383168 |
| 1 | -2.31517966 | -0.94474237 | -1.56028066 |
| 1 | -3.85746878 | -0.33100128 | -2.21461055 |
| 1 | -3.11526601 | -1.79142235 | -2.90919760 |
| 1 | -0.25377986 | -0.04827235 | -4.63140315 |
| 1 | -0.14040185 | -0.72885197 | -2.98862646 |
| 1 | -0.98491596 | -1.63018797 | -4.27556139 |
| 1 | 5.70840554 | -2.21604862 | -0.14974621 |

| | | | |
|---|---|---|---|
| 1 | 6.19112700 | -1.02238111 | 1.06926741 |
| 1 | 5.91685184 | -0.51974591 | -0.62108730 |
| 1 | 2.20403660 | -1.16733259 | -0.67272403 |
| 1 | 3.58311064 | -0.54701771 | -1.60541893 |
| 1 | 3.51377511 | -2.26984657 | -1.16919266 |
| 1 | 4.26805411 | -1.81732570 | 2.49539752 |
| 1 | 2.61126151 | -1.89262081 | 1.84636935 |
| 1 | 3.86456490 | -3.00417635 | 1.23329059 |
| 1 | -1.77346478 | 2.58205621 | 2.53374247 |
| 31 | -0.32910502 | 0.46774300 | 0.74760499 |

Electronic energy = -3523.863686 a.u.
DFT-D3(BJ) dispersion correction = -0.119433 a.u.
Thermal free energy = 0.606198 a.u.
Gibbs free energy = -3523.376920 a.u.
Number of imaginary frequencies = 0.

**Int1b CL isomer/conformer 2**

| Atomic N. | X | Y | Z |
|---|---|---|---|
| 7 | -1.33703046 | 1.63300705 | 1.77810534 |
| 8 | -0.79521640 | 0.74644337 | -1.00390737 |
| 8 | 1.13919502 | -0.00233594 | 1.32530099 |
| 6 | -2.75632634 | 1.49509846 | 2.18676288 |
| 6 | -1.61000397 | 1.77863683 | -1.32572806 |
| 6 | -2.16262673 | 1.85516232 | -2.63981460 |
| 6 | -3.02947973 | 2.92254170 | -2.91405852 |
| 6 | -3.34748525 | 3.90309584 | -1.97034287 |
| 6 | -2.76060053 | 3.84670533 | -0.71097739 |
| 6 | -1.88248318 | 2.80686989 | -0.38575196 |
| 6 | -1.14079213 | 2.85000423 | 0.92308128 |
| 1 | -4.03353449 | 4.70982851 | -2.22825979 |
| 6 | -1.79971671 | 0.82735338 | -3.73058579 |
| 6 | 1.73897454 | 1.05270452 | 1.92195551 |
| 6 | 3.14082000 | 1.26637206 | 1.74899580 |
| 6 | 3.71419136 | 2.36578955 | 2.40261981 |
| 6 | 2.97527317 | 3.24454611 | 3.19948624 |
| 6 | 1.61272206 | 3.02543460 | 3.36409092 |
| 6 | 0.99037519 | 1.93525684 | 2.74427995 |
| 6 | -0.47056976 | 1.68411688 | 3.01064590 |
| 1 | 3.46472531 | 4.08987381 | 3.68291012 |
| 6 | 3.99973050 | 0.32619541 | 0.88059671 |
| 6 | -2.48793126 | 1.14173251 | -5.07225104 |
| 6 | -2.23971964 | -0.59301511 | -3.30886233 |
| 6 | -0.27239285 | 0.84998484 | -3.97990283 |
| 6 | 5.47879651 | 0.75547200 | 0.84727549 |
| 6 | 3.48986043 | 0.33521528 | -0.58001732 |
| 6 | 3.95483783 | -1.10787457 | 1.45914714 |
| 1 | -3.05625202 | 2.36397970 | 2.79615854 |
| 1 | -3.39142680 | 1.44815690 | 1.29541132 |
| 1 | -1.44645420 | 3.74001131 | 1.50002031 |
| 1 | -0.05750955 | 2.92676480 | 0.74705422 |
| 1 | -3.47793180 | 2.99721134 | -3.90303662 |
| 1 | -2.96253548 | 4.62389723 | 0.02979911 |

| | | | |
|---|---|---|---|
| 1 | -0.62176130 | 0.71580766 | 3.51455697 |
| 1 | -0.86156283 | 2.47452591 | 3.67381445 |
| 1 | 4.77988603 | 2.55277627 | 2.28408481 |
| 1 | 1.01571313 | 3.69577890 | 3.98660922 |
| 1 | -2.18257979 | 0.39037765 | -5.81449615 |
| 1 | -3.58430957 | 1.10354504 | -4.99510663 |
| 1 | -2.20071688 | 2.12830716 | -5.46335360 |
| 1 | -1.73412933 | -0.90759513 | -2.38949767 |
| 1 | -3.32695158 | -0.62959988 | -3.14439710 |
| 1 | -1.99510952 | -1.31198909 | -4.10595414 |
| 1 | 0.04709031 | 1.84252158 | -4.32924385 |
| 1 | 0.28645698 | 0.60583293 | -3.06953923 |
| 1 | -0.01100858 | 0.11750698 | -4.75912054 |
| 1 | 6.04107716 | 0.04926796 | 0.21961696 |
| 1 | 5.93618600 | 0.74501426 | 1.84713129 |
| 1 | 5.60934245 | 1.75773689 | 0.41442711 |
| 1 | 2.44367646 | 0.01964010 | -0.64757021 |
| 1 | 3.57199515 | 1.34384323 | -1.01018497 |
| 1 | 4.10143102 | -0.34391683 | -1.19368764 |
| 1 | 4.38853102 | -1.12903950 | 2.46960084 |
| 1 | 2.92730868 | -1.48316998 | 1.51794294 |
| 1 | 4.54381459 | -1.78786436 | 0.82455250 |
| 1 | -2.87652286 | 0.57179074 | 2.76268510 |
| 8 | -2.33260045 | -3.31946926 | 0.69375867 |
| 6 | -3.38539268 | -2.69307226 | -0.05860835 |
| 1 | -3.00466274 | -2.26396671 | -0.99651868 |
| 6 | -0.45214847 | -3.25443436 | 2.13079794 |
| 6 | -0.16914227 | -4.72505492 | 1.80145630 |
| 6 | 0.81973836 | -4.95725954 | 0.65333536 |
| 6 | -1.30396828 | -2.47526729 | 1.11913586 |
| 6 | 0.45779883 | -4.26877507 | -0.67038649 |
| 6 | 0.61833855 | -2.75126892 | -0.65298667 |
| 1 | 0.47814459 | -2.68700146 | 2.28902854 |
| 1 | 0.23071060 | -5.20651495 | 2.70788369 |
| 1 | 0.90957754 | -6.04157685 | 0.48050223 |
| 1 | 1.13921856 | -4.63797655 | -1.45432626 |
| 1 | -1.01934847 | -3.19526359 | 3.07123575 |
| 1 | -1.12262977 | -5.22465053 | 1.57725762 |
| 1 | 1.82255031 | -4.61141892 | 0.95945463 |
| 1 | -0.56139402 | -4.53463568 | -0.98999562 |
| 1 | 0.69504727 | -2.36248862 | -1.67829687 |
| 1 | 1.53716163 | -2.47206509 | -0.11224118 |
| 8 | -1.73635878 | -1.26296689 | 1.59011329 |
| 8 | -0.49505347 | -2.00597826 | -0.08982408 |
| 1 | -4.09613517 | -3.49562796 | -0.28497842 |
| 1 | -3.87874065 | -1.90982387 | 0.53170302 |
| 31 | -0.63005039 | -0.00877797 | 0.69942816 |

Electronic energy = -3523.863090 a.u.
DFT-D3(BJ) dispersion correction = -0.118799 a.u.
Thermal free energy = 0.605941 a.u.
Gibbs free energy = -3523.375948 a.u.
Number of imaginary frequencies = 0.

**Int1b_CL isomer/conformer 3**

| Atomic N. | X | Y | Z |
|---|---|---|---|
| 8 | -4.16804177 | -0.06686938 | 0.59736204 |
| 6 | -3.82505833 | 0.95204175 | 1.54983542 |
| 1 | -4.64535752 | 0.95985430 | 2.27655845 |
| 6 | -3.93321340 | -1.21787599 | -1.42203346 |
| 6 | -3.09859697 | -2.27040448 | -2.15859627 |
| 6 | -2.89330801 | -3.57751268 | -1.37050040 |
| 6 | -3.18973085 | -0.36898072 | -0.37544364 |
| 6 | -1.94675845 | -3.50035801 | -0.16357213 |
| 6 | -2.26597910 | -2.39377522 | 0.83300346 |
| 1 | -4.78666947 | -1.70046736 | -0.92301002 |
| 1 | -2.13224081 | -1.84001366 | -2.46037360 |
| 1 | -3.88199321 | -3.93455521 | -1.03221694 |
| 1 | -0.90995735 | -3.35728220 | -0.50469429 |
| 1 | -4.35281831 | -0.49307525 | -2.13379266 |
| 1 | -3.62378137 | -2.52081499 | -3.09360625 |
| 1 | -2.50977482 | -4.34935162 | -2.05655797 |
| 1 | -1.96810061 | -4.46148651 | 0.37759901 |
| 1 | -1.60150659 | -2.44798552 | 1.70709500 |
| 1 | -3.30629530 | -2.43550214 | 1.19317550 |
| 8 | -2.53375498 | 0.72370714 | -0.88980636 |
| 8 | -2.04765137 | -1.08887902 | 0.24936471 |
| 1 | -3.75899760 | 1.93618317 | 1.06304471 |
| 1 | -2.87784712 | 0.73085865 | 2.06250325 |
| 7 | -0.23635294 | 2.55669991 | -0.86245779 |
| 8 | 0.62056178 | -0.29521136 | -1.11106649 |
| 8 | -0.42003993 | 0.87189986 | 1.57067128 |
| 6 | -0.92765508 | 2.98557217 | -2.10259321 |
| 6 | 1.50511767 | 0.21175312 | -2.00655669 |
| 6 | 2.22196321 | -0.68201399 | -2.86293301 |
| 6 | 3.07857452 | -0.11988469 | -3.81955549 |
| 6 | 3.27299434 | 1.25673834 | -3.95058670 |
| 6 | 2.62452736 | 2.11240710 | -3.06922236 |
| 6 | 1.76481708 | 1.60693608 | -2.08631098 |
| 6 | 1.25101567 | 2.56050421 | -1.04069679 |
| 1 | 3.94068549 | 1.64776576 | -4.71822959 |
| 6 | 2.08909845 | -2.21347806 | -2.74148456 |
| 6 | 0.16812974 | 1.93604301 | 2.15579861 |
| 6 | 0.84962767 | 1.75954455 | 3.39922609 |
| 6 | 1.41938927 | 2.89448213 | 3.99113096 |
| 6 | 1.35670606 | 4.16456098 | 3.41017294 |
| 6 | 0.70506802 | 4.32126595 | 2.19275726 |
| 6 | 0.10203731 | 3.22474108 | 1.56419630 |
| 6 | -0.64839979 | 3.45368878 | 0.27697333 |
| 1 | 1.82123676 | 5.01727151 | 3.90504236 |
| 6 | 0.96714724 | 0.37279831 | 4.06369614 |
| 6 | 3.03161248 | -2.95871334 | -3.70574385 |
| 6 | 0.64693166 | -2.64454727 | -3.08234970 |
| 6 | 2.45523611 | -2.66551755 | -1.30746926 |
| 6 | 1.73879245 | 0.43584132 | 5.39556804 |
| 6 | 1.73644624 | -0.59547316 | 3.13329066 |

| | | | |
|---|---|---|---|
| 6 | -0.43684168 | -0.19715270 | 4.37581505 |
| 1 | -0.63635107 | 4.01838279 | -2.35605182 |
| 1 | -0.64579116 | 2.32175175 | -2.92722118 |
| 1 | 1.57462422 | 3.58721553 | -1.28411006 |
| 1 | 1.67530640 | 2.31223755 | -0.05618217 |
| 1 | 3.62007750 | -0.78100076 | -4.49316695 |
| 1 | 2.79680821 | 3.19008141 | -3.11689929 |
| 1 | -1.73039766 | 3.28676787 | 0.40467875 |
| 1 | -0.50867812 | 4.50076739 | -0.04141109 |
| 1 | 1.94200678 | 2.78918987 | 4.94005007 |
| 1 | 0.65008989 | 5.30344331 | 1.71772408 |
| 1 | 2.90929814 | -4.04100340 | -3.55558600 |
| 1 | 2.80284010 | -2.74558587 | -4.75991117 |
| 1 | 4.08813005 | -2.71595117 | -3.52221018 |
| 1 | -0.06859064 | -2.17070141 | -2.40403801 |
| 1 | 0.38996539 | -2.36323034 | -4.11413319 |
| 1 | 0.54627180 | -3.73733508 | -2.99221685 |
| 1 | 3.49775176 | -2.40354999 | -1.07544826 |
| 1 | 1.80660801 | -2.19855062 | -0.55847701 |
| 1 | 2.35653888 | -3.75887918 | -1.22548606 |
| 1 | 1.79251483 | -0.57480000 | 5.82484608 |
| 1 | 1.24059274 | 1.08210326 | 6.13271708 |
| 1 | 2.77022744 | 0.79201965 | 5.26056169 |
| 1 | 1.22820159 | -0.72273489 | 2.17132658 |
| 1 | 2.75269434 | -0.22162058 | 2.94160360 |
| 1 | 1.82512617 | -1.58189798 | 3.61373750 |
| 1 | -0.98053705 | 0.46577969 | 5.06473552 |
| 1 | -1.03170292 | -0.31552007 | 3.46388929 |
| 1 | -0.33927413 | -1.18037218 | 4.86116376 |
| 1 | -2.01021956 | 2.92591123 | -1.94927902 |
| 31 | -0.75512579 | 0.64110516 | -0.25762582 |

Electronic energy = -3523.861947 a.u.
DFT-D3(BJ) dispersion correction = -0.119617 a.u.
Thermal free energy = 0.605874 a.u.
Gibbs free energy = -3523.375690 a.u.
Number of imaginary frequencies = 0.

**Int1b CL isomer/conformer 4**

| Atomic N. | X | Y | Z |
|---|---|---|---|
| 8 | -3.98968733 | -0.36999370 | 0.10737973 |
| 6 | -3.90448591 | 0.66345528 | 1.10524358 |
| 1 | -4.79164115 | 0.52987816 | 1.73494939 |
| 6 | -3.36920921 | -1.30448595 | -1.97970213 |
| 6 | -3.21684572 | -2.83616186 | -1.93358116 |
| 6 | -3.52940978 | -3.53704505 | -0.59939918 |
| 6 | -2.88917383 | -0.48362328 | -0.76516815 |
| 6 | -2.34427804 | -3.52668438 | 0.37866898 |
| 6 | -2.00839670 | -2.16036575 | 0.95860855 |
| 1 | -4.42640211 | -1.03823497 | -2.11908536 |
| 1 | -2.19278317 | -3.10805579 | -2.23231253 |
| 1 | -4.41749983 | -3.08899044 | -0.12526566 |
| 1 | -1.45484657 | -3.93603649 | -0.12742989 |

| | | | |
|---|---|---|---|
| 1 | -2.82217068 | -0.91843829 | -2.85000127 |
| 1 | -3.87516301 | -3.24307876 | -2.71707440 |
| 1 | -3.78368272 | -4.58747869 | -0.81038599 |
| 1 | -2.55766698 | -4.19176272 | 1.23286507 |
| 1 | -1.09411301 | -2.20185547 | 1.56484052 |
| 1 | -2.83031602 | -1.79550491 | 1.59085680 |
| 8 | -2.30816218 | 0.70840494 | -1.10329698 |
| 8 | -1.73054228 | -1.16965327 | -0.06429787 |
| 1 | -3.92851280 | 1.65687224 | 0.63652036 |
| 1 | -2.99635717 | 0.57603845 | 1.72065188 |
| 7 | -0.11129266 | 2.65200162 | -0.79467370 |
| 8 | 0.89467267 | -0.14225182 | -1.01198514 |
| 8 | -0.48623059 | 0.88837574 | 1.58620863 |
| 6 | -0.81716616 | 3.13260611 | -2.00805922 |
| 6 | 1.64653893 | 0.38178975 | -2.00994770 |
| 6 | 2.25746744 | -0.48708325 | -2.96329414 |
| 6 | 3.01324400 | 0.10499727 | -3.98514921 |
| 6 | 3.19442961 | 1.48659738 | -4.09172103 |
| 6 | 2.62521355 | 2.32007397 | -3.13537191 |
| 6 | 1.86794831 | 1.78154949 | -2.08917225 |
| 6 | 1.37731766 | 2.68623672 | -0.99178066 |
| 1 | 3.78531854 | 1.90039817 | -4.90881124 |
| 6 | 2.11328212 | -2.01870834 | -2.86914187 |
| 6 | 0.17473988 | 1.88508688 | 2.21903341 |
| 6 | 0.82066277 | 1.62891292 | 3.46658098 |
| 6 | 1.46367118 | 2.70493419 | 4.09390675 |
| 6 | 1.50121017 | 3.99022134 | 3.54606014 |
| 6 | 0.87546042 | 4.22579748 | 2.32755696 |
| 6 | 0.20534055 | 3.19072791 | 1.66471510 |
| 6 | -0.52288911 | 3.49741213 | 0.38279323 |
| 1 | 2.01993701 | 4.79375559 | 4.06865297 |
| 6 | 0.82355419 | 0.22465495 | 4.10259847 |
| 6 | 2.88414851 | -2.74182553 | -3.98947338 |
| 6 | 0.62728992 | -2.42631704 | -2.99274478 |
| 6 | 2.68438220 | -2.51781025 | -1.52011228 |
| 6 | 1.57488854 | 0.19913343 | 5.44725639 |
| 6 | 1.53423437 | -0.77844272 | 3.16322963 |
| 6 | -0.62611675 | -0.23737975 | 4.38175772 |
| 1 | -0.53875363 | 4.18004385 | -2.21199480 |
| 1 | -0.53643093 | 2.51265203 | -2.86586096 |
| 1 | 1.68124029 | 3.72718974 | -1.19730710 |
| 1 | 1.81914768 | 2.39677701 | -0.02685623 |
| 1 | 3.48063278 | -0.53324664 | -4.73275983 |
| 1 | 2.77843695 | 3.40082553 | -3.18012361 |
| 1 | -1.60802881 | 3.33914209 | 0.49355013 |
| 1 | -0.36513952 | 4.55696256 | 0.11837206 |
| 1 | 1.96470006 | 2.53781808 | 5.04558420 |
| 1 | 0.89288744 | 5.22200210 | 1.87977960 |
| 1 | 2.75689424 | -3.82695114 | -3.86691710 |
| 1 | 2.51044860 | -2.47603322 | -4.98902813 |
| 1 | 3.96242218 | -2.52970165 | -3.95320951 |
| 1 | 0.02911380 | -1.99478926 | -2.18330181 |
| 1 | 0.21089412 | -2.09356591 | -3.95516509 |

| | | | |
|---|---|---|---|
| 1 | 0.53783899 | -3.52282216 | -2.94659747 |
| 1 | 3.75344680 | -2.27293403 | -1.43935290 |
| 1 | 2.15870364 | -2.06733454 | -0.67103190 |
| 1 | 2.58293568 | -3.61209865 | -1.45479477 |
| 1 | 1.54332837 | -0.82177474 | 5.85398217 |
| 1 | 1.11520194 | 0.86563092 | 6.19130106 |
| 1 | 2.63278874 | 0.47727778 | 5.33631706 |
| 1 | 1.04917184 | -0.83155852 | 2.18267068 |
| 1 | 2.58339747 | -0.48716074 | 3.01019201 |
| 1 | 1.52538271 | -1.78306053 | 3.61317870 |
| 1 | -1.11972120 | 0.44454088 | 5.08958913 |
| 1 | -1.21943483 | -0.27114709 | 3.46204161 |
| 1 | -0.61792712 | -1.24186611 | 4.83210882 |
| 1 | -1.89779074 | 3.05344549 | -1.85185841 |
| 31 | -0.60569859 | 0.68909327 | -0.27500380 |

Electronic energy = -3523.861347 a.u.
DFT-D3(BJ) dispersion correction = -0.119489 a.u.
Thermal free energy = 0.606384 a.u.
Gibbs free energy = -3523.374452 a.u.
Number of imaginary frequencies = 0.

**Int1b CL isomer/conformer 5**

| Atomic N. | X | Y | Z |
|---|---|---|---|
| 7 | -1.59043278 | 2.04768447 | 0.99708697 |
| 8 | 0.10025552 | 0.83952311 | -1.13255650 |
| 8 | 0.41648434 | -0.00742098 | 1.85564628 |
| 6 | -3.03346642 | 2.15559403 | 0.67052528 |
| 6 | -0.32387128 | 1.90961149 | -1.84700091 |
| 6 | -0.21079363 | 1.90242231 | -3.26949962 |
| 6 | -0.67431482 | 3.03233061 | -3.95856384 |
| 6 | -1.22151906 | 4.14414562 | -3.31215136 |
| 6 | -1.29530143 | 4.15283246 | -1.92351708 |
| 6 | -0.83864083 | 3.05519813 | -1.18583501 |
| 6 | -0.80431676 | 3.13226027 | 0.31630513 |
| 1 | -1.57449311 | 4.99642022 | -3.89275801 |
| 6 | 0.40919652 | 0.71027752 | -4.02501360 |
| 6 | 0.86666720 | 1.00294621 | 2.63464783 |
| 6 | 2.19297474 | 0.95867386 | 3.16182629 |
| 6 | 2.60100099 | 2.02539600 | 3.97429638 |
| 6 | 1.77106283 | 3.10800932 | 4.27959575 |
| 6 | 0.47873343 | 3.13584741 | 3.76880702 |
| 6 | 0.01564442 | 2.09075145 | 2.96061061 |
| 6 | -1.41510715 | 2.10594984 | 2.49303585 |
| 1 | 2.13645811 | 3.91756655 | 4.91124709 |
| 6 | 3.14190146 | -0.21747666 | 2.86010806 |
| 6 | 0.45268374 | 0.95032081 | -5.54587067 |
| 6 | -0.42329204 | -0.57009165 | -3.78254424 |
| 6 | 1.86584167 | 0.48774322 | -3.55216509 |
| 6 | 4.51197570 | -0.04688328 | 3.54318009 |
| 6 | 3.40061739 | -0.31903748 | 1.33829516 |
| 6 | 2.52629354 | -1.53400402 | 3.39061875 |
| 1 | -3.42977074 | 3.11590219 | 1.04063464 |

| | | | |
|---|---|---|---|
| 1 | -3.16891839 | 2.10419405 | -0.41484584 |
| 1 | -1.18931580 | 4.10990862 | 0.65406676 |
| 1 | 0.22779234 | 3.03545197 | 0.68417812 |
| 1 | -0.61083819 | 3.05029153 | -5.04505692 |
| 1 | -1.69213958 | 5.02254584 | -1.39464775 |
| 1 | -1.96698248 | 1.23717533 | 2.88751539 |
| 1 | -1.91315121 | 3.01825304 | 2.86322549 |
| 1 | 3.60909055 | 2.01961499 | 4.38484081 |
| 1 | -0.19104463 | 3.96696117 | 4.00122636 |
| 1 | 0.91539496 | 0.07915239 | -6.03135974 |
| 1 | -0.55208433 | 1.07323639 | -5.97575855 |
| 1 | 1.05384323 | 1.83278264 | -5.80829765 |
| 1 | -0.44021473 | -0.83977538 | -2.72124529 |
| 1 | -1.45865418 | -0.43064283 | -4.12758398 |
| 1 | 0.01139376 | -1.40889207 | -4.34822226 |
| 1 | 2.48007541 | 1.37502245 | -3.76408445 |
| 1 | 1.91155072 | 0.28167188 | -2.47713585 |
| 1 | 2.30650443 | -0.36435572 | -4.09234987 |
| 1 | 5.14203214 | -0.91443687 | 3.29996366 |
| 1 | 4.42570015 | 0.00181112 | 4.63827814 |
| 1 | 5.03987388 | 0.85253221 | 3.19453882 |
| 1 | 2.47277084 | -0.47196291 | 0.77785869 |
| 1 | 3.87963847 | 0.59803223 | 0.96587263 |
| 1 | 4.07796501 | -1.16168285 | 1.13100157 |
| 1 | 2.41482899 | -1.49295539 | 4.48404413 |
| 1 | 1.54080944 | -1.72239884 | 2.95101806 |
| 1 | 3.18818677 | -2.38047073 | 3.15095876 |
| 1 | -3.57161117 | 1.32283012 | 1.13490752 |
| 8 | -1.90691563 | -2.81343952 | 1.39669922 |
| 6 | -3.14106316 | -2.85188336 | 2.11794151 |
| 1 | -2.91755728 | -3.33905426 | 3.07394121 |
| 6 | -2.76994770 | -2.72631509 | -0.92912826 |
| 6 | -2.41575556 | -4.18354307 | -1.24335355 |
| 6 | -0.94065192 | -4.45392881 | -1.58526147 |
| 6 | -1.95697635 | -2.05576749 | 0.18758670 |
| 6 | -0.00828756 | -4.36395360 | -0.36622843 |
| 6 | 0.42550538 | -2.95083234 | 0.03016176 |
| 1 | -2.62821758 | -2.09577969 | -1.81885677 |
| 1 | -2.69963337 | -4.82602615 | -0.39348997 |
| 1 | -0.86791049 | -5.47131826 | -1.99992134 |
| 1 | -0.49827011 | -4.85077610 | 0.48902412 |
| 1 | -3.83428292 | -2.64736788 | -0.65979044 |
| 1 | -3.04302621 | -4.49995642 | -2.09179941 |
| 1 | -0.60261010 | -3.76936107 | -2.38069398 |
| 1 | 0.91809576 | -4.92777816 | -0.56223236 |
| 1 | 0.70948373 | -2.91238377 | 1.09007717 |
| 1 | 1.28132266 | -2.62359968 | -0.57616552 |
| 8 | -2.35737643 | -0.75548368 | 0.37713988 |
| 8 | -0.55965683 | -1.90985693 | -0.23276432 |
| 1 | -3.90895703 | -3.44066887 | 1.58754690 |
| 1 | -3.52215609 | -1.83664872 | 2.30288914 |
| 31 | -0.75012314 | 0.22933548 | 0.41478019 |

Electronic energy = -3523.860678 a.u.

DFT-D3(BJ) dispersion correction = -0.118065 a.u.
Thermal free energy = 0.605612 a.u.
Gibbs free energy = -3523.373131 a.u.
Number of imaginary frequencies = 0.

**Int1b CL isomer/conformer 6**

| Atomic N. | X | Y | Z |
|---|---|---|---|
| 7 | -0.53500705 | 2.20629122 | 1.53470856 |
| 8 | -1.32226382 | 0.14308296 | -0.46884201 |
| 8 | 1.69335627 | 0.85194049 | 0.12225003 |
| 6 | -1.38995839 | 2.12355543 | 2.74348155 |
| 6 | -2.47097379 | 0.86108740 | -0.50376248 |
| 6 | -3.65263338 | 0.27085442 | -1.04853535 |
| 6 | -4.81722944 | 1.05091032 | -1.06724156 |
| 6 | -4.86284614 | 2.35948353 | -0.57965409 |
| 6 | -3.70386836 | 2.92905997 | -0.06600631 |
| 6 | -2.50818243 | 2.20181576 | -0.03666855 |
| 6 | -1.24924467 | 2.89648519 | 0.40796015 |
| 1 | -5.79451293 | 2.92437403 | -0.60957822 |
| 6 | -3.66330086 | -1.17302562 | -1.58843140 |
| 6 | 2.11399892 | 2.11406168 | -0.10054272 |
| 6 | 3.04757577 | 2.36804868 | -1.15081859 |
| 6 | 3.47129826 | 3.69145406 | -1.33145986 |
| 6 | 3.01504954 | 4.74866273 | -0.53808094 |
| 6 | 2.10698848 | 4.48880796 | 0.48188499 |
| 6 | 1.66189919 | 3.18228854 | 0.71594247 |
| 6 | 0.74762614 | 2.91788184 | 1.88258879 |
| 1 | 3.36846178 | 5.76323285 | -0.72150998 |
| 6 | 3.56753828 | 1.23275367 | -2.05481481 |
| 6 | -5.02800083 | -1.55637560 | -2.19224867 |
| 6 | -3.37931620 | -2.15993039 | -0.43243528 |
| 6 | -2.61070392 | -1.33919443 | -2.71037220 |
| 6 | 4.58296222 | 1.74340688 | -3.09445839 |
| 6 | 2.39177532 | 0.59737436 | -2.83453498 |
| 6 | 4.28334187 | 0.15600036 | -1.20433046 |
| 1 | -1.63785450 | 3.13769430 | 3.09863279 |
| 1 | -2.31647859 | 1.59172671 | 2.50247521 |
| 1 | -1.48191735 | 3.92941256 | 0.71894040 |
| 1 | -0.52497153 | 2.95808212 | -0.41811490 |
| 1 | -5.73135196 | 0.62254920 | -1.47401424 |
| 1 | -3.70877918 | 3.95727570 | 0.30305763 |
| 1 | 1.24118132 | 2.28226116 | 2.63612382 |
| 1 | 0.48872289 | 3.87430410 | 2.36800356 |
| 1 | 4.18154271 | 3.91474937 | -2.12557612 |
| 1 | 1.74110580 | 5.29881408 | 1.11711412 |
| 1 | -4.97194841 | -2.58651585 | -2.57242804 |
| 1 | -5.83691638 | -1.52450742 | -1.44814774 |
| 1 | -5.30422153 | -0.90665497 | -3.03534623 |
| 1 | -2.42104061 | -1.95524113 | 0.05638404 |
| 1 | -4.17208018 | -2.09699118 | 0.32784759 |
| 1 | -3.36144234 | -3.19233058 | -0.81516262 |
| 1 | -2.83799290 | -0.67029778 | -3.55337786 |

| | | | |
|---|---|---|---|
| 1 | -1.60024757 | -1.11337031 | -2.35436497 |
| 1 | -2.63028429 | -2.37344409 | -3.08709751 |
| 1 | 4.92469963 | 0.89612250 | -3.70602446 |
| 1 | 5.47000764 | 2.18919725 | -2.62150918 |
| 1 | 4.14171677 | 2.48509929 | -3.77586101 |
| 1 | 1.63677288 | 0.18102182 | -2.15896462 |
| 1 | 1.90505613 | 1.34467420 | -3.47791946 |
| 1 | 2.76649974 | -0.21148182 | -3.48053128 |
| 1 | 5.14633319 | 0.59012264 | -0.67835458 |
| 1 | 3.60825635 | -0.28335045 | -0.46193989 |
| 1 | 4.65733644 | -0.64588944 | -1.85949186 |
| 1 | -0.85408533 | 1.57349104 | 3.52445733 |
| 8 | -0.75419274 | -2.77830256 | 2.08308669 |
| 6 | -1.50255808 | -2.82942431 | 3.30191463 |
| 1 | -2.38336604 | -3.44589262 | 3.08900822 |
| 6 | 1.52855528 | -2.36036780 | 2.96389650 |
| 6 | 2.03298754 | -3.77945959 | 2.68226706 |
| 6 | 2.43765064 | -4.06716029 | 1.22660027 |
| 6 | 0.34830692 | -1.87409924 | 2.10971015 |
| 6 | 1.23730847 | -4.17362054 | 0.27191283 |
| 6 | 0.68462641 | -2.84135700 | -0.23688096 |
| 1 | 2.33254247 | -1.62821054 | 2.79803397 |
| 1 | 1.26591357 | -4.51229394 | 2.98256381 |
| 1 | 2.98075933 | -5.02468177 | 1.20639566 |
| 1 | 0.43664555 | -4.73747575 | 0.77156467 |
| 1 | 1.23538367 | -2.26751886 | 4.02081916 |
| 1 | 2.90151536 | -3.95701766 | 3.33617565 |
| 1 | 3.14562765 | -3.30049767 | 0.87023267 |
| 1 | 1.52004496 | -4.75148621 | -0.62284317 |
| 1 | -0.35864366 | -2.95044867 | -0.56270550 |
| 1 | 1.28133589 | -2.47293446 | -1.08272341 |
| 8 | -0.00753679 | -0.58975421 | 2.44410780 |
| 8 | 0.77363668 | -1.74041075 | 0.71213159 |
| 1 | -0.92529782 | -3.29823029 | 4.11712920 |
| 1 | -1.82283818 | -1.82350127 | 3.60974663 |
| 31 | 0.03014427 | 0.34323472 | 0.80455627 |

Electronic energy = -3523.860157 a.u.
DFT-D3(BJ) dispersion correction = -0.117807 a.u.
Thermal free energy = 0.605956 a.u.
Gibbs free energy = -3523.372008 a.u.
Number of imaginary frequencies = 0.

**Int1b CL isomer/conformer 7**

| Atomic N. | X | Y | Z |
|---|---|---|---|
| 7 | -0.78235838 | 1.93688397 | 1.86755521 |
| 8 | -1.30096725 | -0.50439371 | 0.23325489 |
| 8 | 0.99709806 | 1.43316456 | -0.50737614 |
| 6 | -0.74379199 | 1.76784062 | 3.34174995 |
| 6 | -2.48870993 | -0.50338158 | 0.89099146 |
| 6 | -3.30939200 | -1.67000021 | 0.87461850 |
| 6 | -4.51128375 | -1.62128847 | 1.59574400 |
| 6 | -4.93079555 | -0.48801701 | 2.29741393 |

| | | | |
|---|---|---|---|
| 6 | -4.14870544 | 0.66101002 | 2.25817620 |
| 6 | -2.94414180 | 0.67015668 | 1.54652745 |
| 6 | -2.19932993 | 1.96543217 | 1.37000847 |
| 1 | -5.86920873 | -0.50308569 | 2.85168340 |
| 6 | -2.91387886 | -2.92891223 | 0.07786770 |
| 6 | 0.46130421 | 2.60653329 | -0.92809871 |
| 6 | 0.48350620 | 2.95461012 | -2.31282357 |
| 6 | -0.07725309 | 4.18634639 | -2.67892418 |
| 6 | -0.63866776 | 5.07086019 | -1.75426168 |
| 6 | -0.64058598 | 4.72975858 | -0.40705237 |
| 6 | -0.08928185 | 3.51421160 | 0.01464640 |
| 6 | -0.05049655 | 3.19939696 | 1.48645487 |
| 1 | -1.06701694 | 6.01534711 | -2.08941076 |
| 6 | 1.12803486 | 2.03713578 | -3.36961898 |
| 6 | -3.98840085 | -4.02906378 | 0.16520018 |
| 6 | -1.59804814 | -3.52137682 | 0.63001701 |
| 6 | -2.74356144 | -2.57060421 | -1.41810078 |
| 6 | 0.98718038 | 2.60218714 | -4.79603029 |
| 6 | 0.46031691 | 0.64195109 | -3.37078329 |
| 6 | 2.64164301 | 1.91872027 | -3.07124299 |
| 1 | -1.23812347 | 2.62602639 | 3.82652384 |
| 1 | -1.26584995 | 0.84557941 | 3.61793965 |
| 1 | -2.73421370 | 2.78250311 | 1.88402073 |
| 1 | -2.13973083 | 2.22517055 | 0.30295805 |
| 1 | -5.15005773 | -2.50236245 | 1.61498062 |
| 1 | -4.47685404 | 1.57305990 | 2.76214737 |
| 1 | 0.98512838 | 3.05784522 | 1.83460070 |
| 1 | -0.48789763 | 4.03738634 | 2.05517847 |
| 1 | -0.08215118 | 4.47098000 | -3.72943213 |
| 1 | -1.06543428 | 5.40841766 | 0.33599949 |
| 1 | -3.66365812 | -4.89209781 | -0.43359757 |
| 1 | -4.13917861 | -4.38087529 | 1.19615000 |
| 1 | -4.95689552 | -3.69499933 | -0.23420393 |
| 1 | -0.77506713 | -2.80290935 | 0.55663327 |
| 1 | -1.71556057 | -3.80779617 | 1.68546100 |
| 1 | -1.33089743 | -4.42547707 | 0.06032151 |
| 1 | -3.68654719 | -2.18599323 | -1.83341687 |
| 1 | -1.96475598 | -1.81404842 | -1.56409546 |
| 1 | -2.46764013 | -3.47203137 | -1.98675837 |
| 1 | 1.46588239 | 1.90877066 | -5.50231259 |
| 1 | 1.48084292 | 3.57807575 | -4.90819100 |
| 1 | -0.06540523 | 2.70657891 | -5.09675059 |
| 1 | 0.55457997 | 0.14237722 | -2.40186897 |
| 1 | -0.60977455 | 0.72649715 | -3.60930101 |
| 1 | 0.92802403 | 0.00827228 | -4.13975971 |
| 1 | 3.12888584 | 2.89759030 | -3.18887544 |
| 1 | 2.82248042 | 1.56576204 | -2.04990117 |
| 1 | 3.11432317 | 1.21721388 | -3.77575605 |
| 1 | 0.29748334 | 1.69440482 | 3.67094477 |
| 8 | 1.31618331 | -2.15669124 | 2.85856527 |
| 6 | 1.56243104 | -3.55015899 | 2.67105181 |
| 1 | 1.00474776 | -4.06008903 | 3.46542561 |
| 6 | 3.47524015 | -1.21565214 | 2.17288104 |

| | | | |
|---|---|---|---|
| 6 | 4.25671494 | -0.32236373 | 1.19772984 |
| 6 | 4.63072253 | -1.01496542 | -0.12002854 |
| 6 | 1.95412037 | -1.24308787 | 1.96416783 |
| 6 | 3.44125530 | -1.39061806 | -1.00996827 |
| 6 | 2.38770547 | -2.26431503 | -0.33927004 |
| 1 | 3.88527302 | -2.23735425 | 2.15336881 |
| 1 | 3.67716726 | 0.59276925 | 0.99941608 |
| 1 | 5.21097754 | -1.92606054 | 0.11133151 |
| 1 | 2.94009535 | -0.48475722 | -1.38098363 |
| 1 | 3.59363339 | -0.85188975 | 3.20329312 |
| 1 | 5.18508909 | -0.00045787 | 1.69434132 |
| 1 | 5.30638638 | -0.36022596 | -0.69288645 |
| 1 | 3.80788364 | -1.93492925 | -1.89682480 |
| 1 | 1.68891350 | -2.66336342 | -1.08851404 |
| 1 | 2.84740143 | -3.12238437 | 0.17665052 |
| 8 | 1.39181650 | -0.02035220 | 2.21164730 |
| 8 | 1.53640155 | -1.54142498 | 0.58238515 |
| 1 | 1.18654783 | -3.90244570 | 1.69787522 |
| 1 | 2.62986800 | -3.81187560 | 2.77231205 |
| 31 | 0.19828003 | 0.41734941 | 0.84820879 |

Electronic energy = -3523.855805 a.u.
DFT-D3(BJ) dispersion correction = -0.119272 a.u.
Thermal free energy = 0.605684 a.u.
Gibbs free energy = -3523.369393 a.u.
Number of imaginary frequencies = 0.

**Int1b CL isomer/conformer 8**

| Atomic N. | X | Y | Z |
|---|---|---|---|
| 7 | 0.02990956 | 2.57219693 | 1.09577221 |
| 8 | -1.38619053 | 0.26957381 | -0.16176409 |
| 8 | 1.69294063 | 0.52862731 | -0.36115212 |
| 6 | -0.36094824 | 2.91114754 | 2.48712820 |
| 6 | -2.48091769 | 1.05529066 | 0.00260254 |
| 6 | -3.78446884 | 0.48562663 | -0.10425034 |
| 6 | -4.87851099 | 1.33852437 | 0.10176128 |
| 6 | -4.73776283 | 2.69981934 | 0.38401595 |
| 6 | -3.46313965 | 3.25332461 | 0.43200189 |
| 6 | -2.33676458 | 2.44961964 | 0.22495845 |
| 6 | -0.98083059 | 3.09220482 | 0.11404315 |
| 1 | -5.61941432 | 3.31982999 | 0.54620780 |
| 6 | -3.99400570 | -1.00051192 | -0.45583277 |
| 6 | 2.01619299 | 1.60181293 | -1.12518580 |
| 6 | 2.54076627 | 1.41739816 | -2.44066384 |
| 6 | 2.86382241 | 2.56682562 | -3.17543544 |
| 6 | 2.70073333 | 3.86063714 | -2.67352090 |
| 6 | 2.20526521 | 4.02880907 | -1.38613430 |
| 6 | 1.87215858 | 2.91544932 | -0.60574933 |
| 6 | 1.40113783 | 3.12989993 | 0.80819931 |
| 1 | 2.96147353 | 4.72341851 | -3.28628411 |
| 6 | 2.77680809 | 0.01358468 | -3.03015041 |
| 6 | -5.48681428 | -1.36157539 | -0.57322678 |
| 6 | -3.38437521 | -1.90105815 | 0.64181864 |

| | | | |
|---|---|---|---|
| 6 | -3.33684830 | -1.31491786 | -1.82130600 |
| 6 | 3.31392338 | 0.06996353 | -4.47295571 |
| 6 | 1.45780089 | -0.79344690 | -3.07114158 |
| 6 | 3.83752805 | -0.71918405 | -2.17500105 |
| 1 | -0.39181821 | 4.00676845 | 2.60747717 |
| 1 | -1.35149698 | 2.49541690 | 2.70013466 |
| 1 | -1.06789476 | 4.18437152 | 0.24622017 |
| 1 | -0.55526057 | 2.91015744 | -0.88370404 |
| 1 | -5.88486111 | 0.92819992 | 0.04208687 |
| 1 | -3.32912355 | 4.32292471 | 0.60945682 |
| 1 | 2.07613166 | 2.64190686 | 1.52940291 |
| 1 | 1.38897709 | 4.20985910 | 1.03299818 |
| 1 | 3.25620240 | 2.45365217 | -4.18430076 |
| 1 | 2.07800094 | 5.02977723 | -0.96770362 |
| 1 | -5.57596486 | -2.42387200 | -0.84232830 |
| 1 | -6.02490206 | -1.21520867 | 0.37461567 |
| 1 | -5.99388501 | -0.77844675 | -1.35548952 |
| 1 | -2.30872773 | -1.72677845 | 0.74917287 |
| 1 | -3.86564552 | -1.70840462 | 1.61192917 |
| 1 | -3.54792680 | -2.95986362 | 0.38588289 |
| 1 | -3.79200742 | -0.70871256 | -2.61813163 |
| 1 | -2.25976046 | -1.11539477 | -1.80456280 |
| 1 | -3.49325609 | -2.37512188 | -2.07343500 |
| 1 | 3.46732645 | -0.95648452 | -4.83586962 |
| 1 | 4.28083156 | 0.58924990 | -4.53708187 |
| 1 | 2.60768857 | 0.56090765 | -5.15810641 |
| 1 | 1.02562635 | -0.92144965 | -2.07391201 |
| 1 | 0.71487916 | -0.28618122 | -3.70352200 |
| 1 | 1.64513215 | -1.78844334 | -3.50300751 |
| 1 | 4.80664979 | -0.20366056 | -2.24229558 |
| 1 | 3.54370619 | -0.75836771 | -1.12033891 |
| 1 | 3.97513240 | -1.74823606 | -2.54122804 |
| 1 | 0.36161503 | 2.47321678 | 3.18294045 |
| 8 | -0.71260668 | -1.40393167 | 3.48990280 |
| 6 | -1.25149187 | -2.71513724 | 3.65669387 |
| 1 | -2.13379951 | -2.59470637 | 4.29632247 |
| 6 | 1.68976201 | -1.89807348 | 3.42633112 |
| 6 | 3.00266377 | -1.87898773 | 2.62890808 |
| 6 | 3.17126278 | -3.06917761 | 1.67405911 |
| 6 | 0.48166593 | -1.26861069 | 2.71747594 |
| 6 | 2.16768937 | -3.11501504 | 0.51686949 |
| 6 | 0.70296459 | -3.08949050 | 0.93678130 |
| 1 | 1.45882865 | -2.92776553 | 3.74073123 |
| 1 | 3.07832822 | -0.92949196 | 2.07627066 |
| 1 | 3.09311436 | -4.00419770 | 2.25708332 |
| 1 | 2.33559561 | -2.27211776 | -0.16919071 |
| 1 | 1.79294490 | -1.30680636 | 4.34713425 |
| 1 | 3.83953447 | -1.88942210 | 3.34418281 |
| 1 | 4.19030193 | -3.05464791 | 1.25605621 |
| 1 | 2.32928290 | -4.03602328 | -0.06865532 |
| 1 | 0.05578592 | -3.33719146 | 0.08312778 |
| 1 | 0.50046129 | -3.82479022 | 1.73166344 |
| 8 | 0.65542219 | 0.07512241 | 2.52657720 |

| | | | |
|---|---|---|---|
| 8 | 0.24463779 | -1.78128032 | 1.35586089 |
| 1 | -1.57165664 | -3.14852000 | 2.69639364 |
| 1 | -0.54982520 | -3.40402025 | 4.15798800 |
| 31 | 0.20429564 | 0.52961018 | 0.77537738 |

Electronic energy = -3523.855821 a.u.
DFT-D3(BJ) dispersion correction = -0.119258 a.u.
Thermal free energy = 0.605711 a.u.
Gibbs free energy = -3523.369368 a.u.
Number of imaginary frequencies = 0.

**TS2 eq CL isomer/conformer 1**

| Atomic N. | X | Y | Z |
|---|---|---|---|
| 7 | -1.30396855 | 1.53332722 | 1.63256419 |
| 8 | -0.83378220 | 0.67592758 | -1.13100028 |
| 8 | 1.18870902 | -0.03597856 | 1.24969697 |
| 6 | -2.75180936 | 1.47143734 | 1.94598317 |
| 6 | -1.57847500 | 1.75939405 | -1.43084490 |
| 6 | -2.18996811 | 1.86092603 | -2.71778727 |
| 6 | -2.96029210 | 3.00276566 | -2.97829270 |
| 6 | -3.14141011 | 4.02514601 | -2.04188395 |
| 6 | -2.52577877 | 3.92312932 | -0.79878986 |
| 6 | -1.73861778 | 2.80859041 | -0.49066228 |
| 6 | -0.99652416 | 2.75543308 | 0.81518871 |
| 1 | -3.75320458 | 4.89259863 | -2.28952289 |
| 6 | -2.00107098 | 0.76563799 | -3.78579998 |
| 6 | 1.74529076 | 0.97362924 | 1.95206666 |
| 6 | 3.15461040 | 1.19869912 | 1.87863159 |
| 6 | 3.68404126 | 2.24571967 | 2.64537168 |
| 6 | 2.89495540 | 3.06652761 | 3.45598507 |
| 6 | 1.52427578 | 2.84284449 | 3.51429892 |
| 6 | 0.94482094 | 1.80110300 | 2.78068423 |
| 6 | -0.53101635 | 1.54229283 | 2.92555332 |
| 1 | 3.35157514 | 3.87307215 | 4.02947378 |
| 6 | 4.06553507 | 0.33141893 | 0.98774254 |
| 6 | -2.74432826 | 1.09876919 | -5.09301043 |
| 6 | -2.55314040 | -0.58605236 | -3.27389741 |
| 6 | -0.49976081 | 0.62189269 | -4.13210249 |
| 6 | 5.54410362 | 0.75233334 | 1.08584726 |
| 6 | 3.64608693 | 0.47574112 | -0.49433157 |
| 6 | 3.98345542 | -1.14984930 | 1.42580247 |
| 1 | -3.05499220 | 2.38124347 | 2.49082112 |
| 1 | -3.33007479 | 1.40621018 | 1.01806879 |
| 1 | -1.23022306 | 3.64922833 | 1.41914105 |
| 1 | 0.09029352 | 2.74870801 | 0.64593875 |
| 1 | -3.44327641 | 3.10399246 | -3.94866323 |
| 1 | -2.63808727 | 4.71787739 | -0.05751796 |
| 1 | -0.72215253 | 0.56077933 | 3.38767385 |
| 1 | -0.97003764 | 2.31425118 | 3.58059621 |
| 1 | 4.75468111 | 2.43825817 | 2.60758257 |
| 1 | 0.88706201 | 3.47193050 | 4.14013338 |
| 1 | -2.56943107 | 0.29216155 | -5.81938457 |
| 1 | -3.83087850 | 1.17975163 | -4.94323730 |

| | | | |
|---|---|---|---|
| 1 | -2.38526940 | 2.03428984 | -5.54549122 |
| 1 | -2.02013230 | -0.91843110 | -2.37587667 |
| 1 | -3.62534881 | -0.50181472 | -3.04070354 |
| 1 | -2.43751192 | -1.35526776 | -4.05300808 |
| 1 | -0.10807125 | 1.56213188 | -4.54668045 |
| 1 | 0.09138908 | 0.35952565 | -3.24778247 |
| 1 | -0.36485842 | -0.16420805 | -4.89106035 |
| 1 | 6.14457321 | 0.10010167 | 0.43558925 |
| 1 | 5.93622303 | 0.65174890 | 2.10827065 |
| 1 | 5.70332718 | 1.78772247 | 0.75188136 |
| 1 | 2.60454488 | 0.17620400 | -0.65223384 |
| 1 | 3.75863481 | 1.51793718 | -0.82663119 |
| 1 | 4.28959036 | -0.15254490 | -1.12906015 |
| 1 | 4.32956123 | -1.26533270 | 2.46346593 |
| 1 | 2.95828342 | -1.52902639 | 1.35707080 |
| 1 | 4.63043404 | -1.76661253 | 0.78323102 |
| 1 | -2.94859815 | 0.58783239 | 2.56026745 |
| 8 | -2.38447475 | -3.35509491 | 0.98640645 |
| 6 | -3.52514553 | -2.74756527 | 0.33538064 |
| 1 | -3.19240856 | -2.17226887 | -0.53795880 |
| 6 | -0.36904061 | -3.22051930 | 2.24129105 |
| 6 | 0.09442509 | -4.58800936 | 1.72299027 |
| 6 | 1.08358634 | -4.57741213 | 0.54597700 |
| 6 | -1.44369030 | -2.50976610 | 1.44750345 |
| 6 | 0.56288403 | -4.06093073 | -0.80741847 |
| 6 | 0.53750902 | -2.54137921 | -0.96041477 |
| 1 | 0.47154331 | -2.52074194 | 2.34795213 |
| 1 | 0.58428395 | -5.10442924 | 2.56362319 |
| 1 | 1.42772496 | -5.61519146 | 0.40764055 |
| 1 | 1.22319460 | -4.44232082 | -1.60399413 |
| 1 | -0.80603510 | -3.34266996 | 3.24894738 |
| 1 | -0.78514779 | -5.19621515 | 1.46280468 |
| 1 | 1.97986841 | -3.99836826 | 0.82832575 |
| 1 | -0.44032183 | -4.46890783 | -1.01421356 |
| 1 | 0.33246624 | -2.28312135 | -2.01496720 |
| 1 | 1.53186429 | -2.13453817 | -0.70108831 |
| 8 | -1.76048040 | -1.28515899 | 1.70687604 |
| 8 | -0.46984684 | -1.93761861 | -0.14936627 |
| 1 | -4.15490341 | -3.58617783 | 0.02335042 |
| 1 | -4.06566048 | -2.09595513 | 1.03200710 |
| 31 | -0.55616093 | -0.13076264 | 0.54897928 |

Electronic energy = -3523.859222 a.u.
DFT-D3(BJ) dispersion correction = -0.118859 a.u.
Thermal free energy = 0.605300 a.u.
Gibbs free energy = -3523.372782 a.u.
Number of imaginary frequencies = 1.

**TS2_eq_CL isomer/conformer 2**

| Atomic N. | X | Y | Z |
|---|---|---|---|
| 8 | -1.47519457 | -1.84862149 | 3.40839958 |
| 6 | -0.23146258 | -1.42060268 | 4.01633978 |
| 1 | 0.55878568 | -1.36633325 | 3.25624990 |

| | | | |
|---|---|---|---|
| 6 | -3.29540443 | -1.53358388 | 1.91353416 |
| 6 | -3.53985882 | -3.04405880 | 1.79613125 |
| 6 | -2.88678527 | -3.76957464 | 0.60522902 |
| 6 | -1.93285489 | -1.08951998 | 2.39648032 |
| 6 | -1.35672176 | -3.94724107 | 0.65091646 |
| 6 | -0.55756164 | -2.76634264 | 0.10263950 |
| 1 | -3.48018956 | -1.02286851 | 0.95945096 |
| 1 | -4.62956142 | -3.18433094 | 1.71500695 |
| 1 | -3.34595466 | -4.76995420 | 0.55589825 |
| 1 | -1.07705808 | -4.82526493 | 0.04571688 |
| 1 | -4.00856638 | -1.10979903 | 2.64389515 |
| 1 | -3.23971224 | -3.53462219 | 2.73491001 |
| 1 | -3.16705942 | -3.26280284 | -0.33404842 |
| 1 | -1.03537643 | -4.16530085 | 1.68244171 |
| 1 | 0.52288520 | -2.99474955 | 0.15533195 |
| 1 | -0.81920886 | -2.61151075 | -0.96073556 |
| 8 | -1.54844499 | 0.13323542 | 2.28049111 |
| 8 | -0.83730853 | -1.58227968 | 0.83685422 |
| 1 | -0.00089613 | -2.18755913 | 4.76176786 |
| 1 | -0.35777044 | -0.44242093 | 4.49621296 |
| 7 | -0.22945879 | 2.34547186 | 1.02563679 |
| 8 | -0.72891235 | 0.44495448 | -1.15164769 |
| 8 | 1.50969744 | -0.03195771 | 1.13013136 |
| 6 | -1.52676141 | 2.95170784 | 1.41143370 |
| 6 | -1.08840406 | 1.55548608 | -1.82923603 |
| 6 | -1.89580643 | 1.43425548 | -3.00209522 |
| 6 | -2.25032353 | 2.61471343 | -3.66941667 |
| 6 | -1.84606314 | 3.88091087 | -3.23672247 |
| 6 | -1.04813409 | 3.98550057 | -2.10276604 |
| 6 | -0.65714097 | 2.83784771 | -1.40459061 |
| 6 | 0.28589109 | 2.96600580 | -0.24136204 |
| 1 | -2.15059876 | 4.77141476 | -3.78652763 |
| 6 | -2.35522747 | 0.05904571 | -3.52455425 |
| 6 | 2.48565769 | 0.87172139 | 1.34600461 |
| 6 | 3.84750581 | 0.51353729 | 1.10392201 |
| 6 | 4.82650805 | 1.48116744 | 1.36700392 |
| 6 | 4.51992035 | 2.76128006 | 1.83831203 |
| 6 | 3.19039178 | 3.10090137 | 2.06168890 |
| 6 | 2.17341685 | 2.16725850 | 1.83089340 |
| 6 | 0.75086987 | 2.54126382 | 2.15248895 |
| 1 | 5.31589460 | 3.48294473 | 2.02151752 |
| 6 | 4.22976971 | -0.87939942 | 0.56494033 |
| 6 | -3.20271850 | 0.17818968 | -4.80511379 |
| 6 | -3.22670221 | -0.65056789 | -2.46182489 |
| 6 | -1.12323666 | -0.81187409 | -3.86792326 |
| 6 | 5.75343800 | -1.03696537 | 0.40220791 |
| 6 | 3.59322000 | -1.09938562 | -0.82804281 |
| 6 | 3.75731397 | -1.98252523 | 1.54157925 |
| 1 | -1.40785742 | 4.04140472 | 1.53316987 |
| 1 | -2.27231717 | 2.76144624 | 0.63224542 |
| 1 | 0.50056320 | 4.03113937 | -0.04779306 |
| 1 | 1.24542689 | 2.47411537 | -0.45927843 |
| 1 | -2.86933446 | 2.55062485 | -4.56253815 |

| | | | |
|---|---|---|---|
| 1 | -0.70664942 | 4.96281052 | -1.75367486 |
| 1 | 0.35847515 | 1.94299936 | 2.99041414 |
| 1 | 0.71707463 | 3.60228729 | 2.45432997 |
| 1 | 5.87244177 | 1.23543751 | 1.19223750 |
| 1 | 2.92740774 | 4.09626341 | 2.42734218 |
| 1 | -3.49473166 | -0.82907087 | -5.13538456 |
| 1 | -4.12566662 | 0.75291198 | -4.64003277 |
| 1 | -2.64398193 | 0.64638549 | -5.62818003 |
| 1 | -2.66692233 | -0.81156462 | -1.53400934 |
| 1 | -4.12192965 | -0.05297185 | -2.23385024 |
| 1 | -3.56097054 | -1.62762153 | -2.84372926 |
| 1 | -0.53065306 | -0.34179351 | -4.66617966 |
| 1 | -0.47647819 | -0.95365304 | -2.99509501 |
| 1 | -1.45102012 | -1.79947150 | -4.22763205 |
| 1 | 5.96942234 | -2.04467607 | 0.01940562 |
| 1 | 6.28543282 | -0.92489713 | 1.35814536 |
| 1 | 6.17052984 | -0.31536889 | -0.31493735 |
| 1 | 2.50041103 | -1.03716075 | -0.78717715 |
| 1 | 3.95692658 | -0.34664217 | -1.54248190 |
| 1 | 3.87068629 | -2.09259176 | -1.21310198 |
| 1 | 4.22708416 | -1.85366607 | 2.52788401 |
| 1 | 2.66924214 | -1.96408749 | 1.66634786 |
| 1 | 4.04911423 | -2.97126341 | 1.15560102 |
| 1 | -1.86555052 | 2.50659156 | 2.35158491 |
| 31 | -0.29490122 | 0.25528556 | 0.67131567 |

Electronic energy = -3523.858439 a.u.
DFT-D3(BJ) dispersion correction = -0.118850 a.u.
Thermal free energy = 0.605079 a.u.
Gibbs free energy = -3523.372209 a.u.
Number of imaginary frequencies = 1.

**TS2_eq_CL isomer/conformer 3**

| Atomic N. | X | Y | Z |
|---|---|---|---|
| 7 | -0.62871790 | 1.70066679 | 1.74767888 |
| 8 | -1.19072723 | -0.74270433 | 0.22123486 |
| 8 | 0.98705405 | 1.30643773 | -0.71982688 |
| 6 | -0.73314244 | 1.47805309 | 3.21025133 |
| 6 | -2.41236353 | -0.63682020 | 0.77972060 |
| 6 | -3.28705788 | -1.76622093 | 0.79120791 |
| 6 | -4.53559732 | -1.60912144 | 1.40916848 |
| 6 | -4.94970465 | -0.40588647 | 1.98874044 |
| 6 | -4.10508442 | 0.69815630 | 1.93514621 |
| 6 | -2.85130382 | 0.59688479 | 1.32380021 |
| 6 | -2.00060773 | 1.82244313 | 1.14783549 |
| 1 | -5.92853975 | -0.33463368 | 2.46306133 |
| 6 | -2.88770843 | -3.09880400 | 0.12755661 |
| 6 | 0.54705679 | 2.55760145 | -0.98755467 |
| 6 | 0.48042393 | 3.02319860 | -2.33797669 |
| 6 | 0.07415805 | 4.34908533 | -2.54570770 |
| 6 | -0.28380689 | 5.20761585 | -1.50343931 |
| 6 | -0.25672048 | 4.73111153 | -0.19828746 |
| 6 | 0.15777484 | 3.42158937 | 0.06901552 |

| | | | |
|---|---|---|---|
| 6 | 0.18262221 | 2.94763350 | 1.49771357 |
| 1 | -0.59681332 | 6.22955322 | -1.71701360 |
| 6 | 0.82165569 | 2.11342502 | -3.53538442 |
| 6 | -4.00093508 | -4.15701580 | 0.24005884 |
| 6 | -1.62410426 | -3.68095851 | 0.80237323 |
| 6 | -2.61593914 | -2.87179446 | -1.37911415 |
| 6 | 0.61643410 | 2.82747030 | -4.88528728 |
| 6 | -0.09815581 | 0.86954319 | -3.53624249 |
| 6 | 2.30319381 | 1.68080199 | -3.46793962 |
| 1 | -1.30496967 | 2.30239129 | 3.66841888 |
| 1 | -1.25152397 | 0.53315705 | 3.40507078 |
| 1 | -2.50029087 | 2.69907880 | 1.59507513 |
| 1 | -1.85479128 | 2.03647304 | 0.07950568 |
| 1 | -5.21824408 | -2.45654726 | 1.44306326 |
| 1 | -4.41916704 | 1.65786767 | 2.35248828 |
| 1 | 1.20414948 | 2.71108770 | 1.83329773 |
| 1 | -0.20920824 | 3.74711466 | 2.14926481 |
| 1 | 0.02258600 | 4.73028183 | -3.56373096 |
| 1 | -0.55587423 | 5.37414789 | 0.63259381 |
| 1 | -3.66936135 | -5.07920122 | -0.25856492 |
| 1 | -4.22857809 | -4.40961409 | 1.28609228 |
| 1 | -4.93082333 | -3.83280778 | -0.24897031 |
| 1 | -0.77199805 | -2.99923468 | 0.70351154 |
| 1 | -1.81203067 | -3.86669040 | 1.87096083 |
| 1 | -1.36205196 | -4.64237547 | 0.33322623 |
| 1 | -3.51955986 | -2.49827862 | -1.88244593 |
| 1 | -1.80656481 | -2.14987063 | -1.53388262 |
| 1 | -2.33265734 | -3.82345581 | -1.85486841 |
| 1 | 0.86205226 | 2.12827945 | -5.69745684 |
| 1 | 1.27166295 | 3.70360804 | -4.99581242 |
| 1 | -0.42473885 | 3.14994717 | -5.02982378 |
| 1 | 0.00797990 | 0.28378940 | -2.61717892 |
| 1 | -1.15204883 | 1.16832602 | -3.63296366 |
| 1 | 0.14940920 | 0.22348873 | -4.39231873 |
| 1 | 2.96590018 | 2.55713367 | -3.51965117 |
| 1 | 2.51542807 | 1.14035785 | -2.54102588 |
| 1 | 2.54235435 | 1.02440846 | -4.31889343 |
| 1 | 0.27071419 | 1.43388140 | 3.64104199 |
| 8 | 1.16295516 | -1.74719739 | 2.99876881 |
| 6 | 1.35036075 | -3.17679524 | 3.03824782 |
| 1 | 0.41842693 | -3.57983255 | 3.44620323 |
| 6 | 3.54296494 | -1.41009939 | 2.44806957 |
| 6 | 4.52565193 | -0.60227817 | 1.59210002 |
| 6 | 4.72103739 | -1.11591220 | 0.15646566 |
| 6 | 2.09747124 | -0.97415930 | 2.38086033 |
| 6 | 3.57993960 | -0.85188884 | -0.83640581 |
| 6 | 2.31222653 | -1.69513810 | -0.67566961 |
| 1 | 3.62538266 | -2.48052454 | 2.21659112 |
| 1 | 4.21281385 | 0.45260918 | 1.57860422 |
| 1 | 4.94377518 | -2.19804978 | 0.18458001 |
| 1 | 3.30190659 | 0.21305905 | -0.80747223 |
| 1 | 3.80673170 | -1.30749321 | 3.51742220 |
| 1 | 5.50299978 | -0.63212007 | 2.09786057 |

| | | | |
|---|---|---|---|
| 1 | 5.62862778 | -0.63490653 | -0.24249646 |
| 1 | 3.95990372 | -1.04712665 | -1.85399377 |
| 1 | 1.68315434 | -1.56705439 | -1.57447517 |
| 1 | 2.58425760 | -2.76745605 | -0.62947577 |
| 8 | 1.76559341 | 0.24643877 | 2.26393056 |
| 8 | 1.52143872 | -1.39834118 | 0.47285169 |
| 1 | 1.50153601 | -3.56129694 | 2.02077937 |
| 1 | 2.18846989 | -3.45290399 | 3.69280839 |
| 31 | 0.39127728 | 0.18441173 | 0.66703302 |

Electronic energy = -3523.846538 a.u.
DFT-D3(BJ) dispersion correction = -0.119984 a.u.
Thermal free energy = 0.604138 a.u.
Gibbs free energy = -3523.362384 a.u.
Number of imaginary frequencies = 1.

**TS2 eq CL isomer/conformer 4**

| Atomic N. | X | Y | Z |
|---|---|---|---|
| 7 | -0.07560727 | 2.33331847 | 1.09360421 |
| 8 | -1.45345509 | 0.10306274 | -0.20510463 |
| 8 | 1.64983356 | 0.42363319 | -0.44966152 |
| 6 | -0.61966491 | 2.65826154 | 2.43532586 |
| 6 | -2.53125548 | 0.90733075 | -0.11604185 |
| 6 | -3.84931183 | 0.36302966 | -0.20891254 |
| 6 | -4.92527723 | 1.25718760 | -0.11597459 |
| 6 | -4.75706768 | 2.63366675 | 0.06276248 |
| 6 | -3.46931338 | 3.15382481 | 0.14219205 |
| 6 | -2.36174703 | 2.30625796 | 0.04216211 |
| 6 | -0.97427613 | 2.87677264 | 0.01673878 |
| 1 | -5.62651873 | 3.28724217 | 0.13356186 |
| 6 | -4.08481264 | -1.14798665 | -0.39465547 |
| 6 | 2.06305289 | 1.56131196 | -1.05411601 |
| 6 | 2.68980622 | 1.49924016 | -2.33783221 |
| 6 | 3.10596728 | 2.70606375 | -2.91661835 |
| 6 | 2.93470263 | 3.94539356 | -2.29493785 |
| 6 | 2.33009100 | 3.99419737 | -1.04474449 |
| 6 | 1.90338910 | 2.81910729 | -0.41505271 |
| 6 | 1.30939829 | 2.91761684 | 0.96642900 |
| 1 | 3.26953983 | 4.85809755 | -2.78760481 |
| 6 | 2.92773414 | 0.15896262 | -3.06050587 |
| 6 | -5.58092546 | -1.49267757 | -0.51769149 |
| 6 | -3.53324723 | -1.91414154 | 0.83133990 |
| 6 | -3.39522958 | -1.64161909 | -1.68865466 |
| 6 | 3.58154273 | 0.34918666 | -4.44262791 |
| 6 | 1.58900511 | -0.57999688 | -3.29445791 |
| 6 | 3.89124322 | -0.70936060 | -2.21730852 |
| 1 | -0.70376003 | 3.75218248 | 2.54558158 |
| 1 | -1.61396646 | 2.21223092 | 2.54691458 |
| 1 | -1.01017511 | 3.97487593 | 0.11943627 |
| 1 | -0.48048314 | 2.64919925 | -0.93875289 |
| 1 | -5.94035959 | 0.86887479 | -0.18063237 |
| 1 | -3.31066608 | 4.22784710 | 0.26549184 |
| 1 | 1.92537498 | 2.39008355 | 1.70939827 |

| | | | |
|---|---|---|---|
| 1 | 1.25631583 | 3.97934842 | 1.26120126 |
| 1 | 3.58010888 | 2.68438005 | -3.89604807 |
| 1 | 2.18743014 | 4.95051670 | -0.53629547 |
| 1 | -5.68720627 | -2.57817698 | -0.65774834 |
| 1 | -6.14619541 | -1.21955860 | 0.38519779 |
| 1 | -6.04810143 | -0.99974364 | -1.38251495 |
| 1 | -2.46639562 | -1.71596920 | 0.98330116 |
| 1 | -4.07573462 | -1.61787820 | 1.74181867 |
| 1 | -3.67509961 | -2.99737096 | 0.69137317 |
| 1 | -3.82276392 | -1.13803530 | -2.56805229 |
| 1 | -2.31777954 | -1.44558895 | -1.66988134 |
| 1 | -3.55666161 | -2.72414207 | -1.80839729 |
| 1 | 3.72705317 | -0.63728875 | -4.90598392 |
| 1 | 4.56831789 | 0.82910329 | -4.37380409 |
| 1 | 2.95099998 | 0.94306695 | -5.12004185 |
| 1 | 1.06647217 | -0.78618050 | -2.35526896 |
| 1 | 0.92416275 | 0.02230484 | -3.93055224 |
| 1 | 1.77554393 | -1.53456342 | -3.80979657 |
| 1 | 4.87947178 | -0.23104562 | -2.15240788 |
| 1 | 3.51382160 | -0.85362107 | -1.19928288 |
| 1 | 4.02216196 | -1.69627774 | -2.68730545 |
| 1 | 0.04924205 | 2.25281286 | 3.19988513 |
| 8 | -0.22456709 | -0.84390080 | 3.86375785 |
| 6 | -0.78725141 | -2.08004165 | 4.34432459 |
| 1 | -1.75395310 | -1.80748975 | 4.77992058 |
| 6 | 1.95830679 | -1.91474187 | 3.39420509 |
| 6 | 3.00252581 | -2.14225554 | 2.28823733 |
| 6 | 2.69804573 | -3.30413461 | 1.32124615 |
| 6 | 0.90206146 | -0.87293661 | 3.09761429 |
| 6 | 1.69203234 | -3.03438926 | 0.19259267 |
| 6 | 0.25887692 | -2.74298263 | 0.62584317 |
| 1 | 1.49068725 | -2.86803770 | 3.67465138 |
| 1 | 3.15181994 | -1.20568955 | 1.73040068 |
| 1 | 2.36975861 | -4.18252087 | 1.90623033 |
| 1 | 2.04588485 | -2.19363856 | -0.42220056 |
| 1 | 2.45562363 | -1.53715825 | 4.30664825 |
| 1 | 3.96022820 | -2.36441207 | 2.78292131 |
| 1 | 3.64900923 | -3.60025692 | 0.85022658 |
| 1 | 1.66411150 | -3.91721630 | -0.46954361 |
| 1 | -0.39314988 | -2.67209363 | -0.26280993 |
| 1 | -0.13150910 | -3.57726121 | 1.23918164 |
| 8 | 1.20078874 | 0.24902008 | 2.58226991 |
| 8 | 0.12262572 | -1.54177308 | 1.38415265 |
| 1 | -0.94690812 | -2.77367926 | 3.50826240 |
| 1 | -0.15598279 | -2.53958321 | 5.11744452 |
| 31 | 0.19220918 | 0.27880272 | 0.72597671 |

Electronic energy = -3523.847127 a.u.
DFT-D3(BJ) dispersion correction = -0.120234 a.u.
Thermal free energy = 0.605598 a.u.
Gibbs free energy = -3523.361763 a.u.
Number of imaginary frequencies = 1.

**TS2 eq CL isomer/conformer 5**

| Atomic N. | X | Y | Z |
|---|---|---|---|
| 8 | -4.34746075 | 0.00582708 | -1.41470873 |
| 6 | -4.49265957 | 1.43501723 | -1.34548545 |
| 1 | -5.57212734 | 1.61706066 | -1.32958782 |
| 6 | -3.12228155 | -1.96654618 | -1.76677132 |
| 6 | -2.17098093 | -2.92536974 | -1.03230596 |
| 6 | -2.63063502 | -3.40321517 | 0.36023292 |
| 6 | -3.06697297 | -0.47372246 | -1.45342994 |
| 6 | -2.26517105 | -2.49374771 | 1.54065621 |
| 6 | -2.89212561 | -1.10786867 | 1.49330235 |
| 1 | -4.16009665 | -2.30108285 | -1.62851453 |
| 1 | -1.16137290 | -2.49391007 | -0.96919698 |
| 1 | -3.72175288 | -3.57194853 | 0.34670568 |
| 1 | -1.16947067 | -2.38006306 | 1.59387267 |
| 1 | -2.91557097 | -2.01671267 | -2.84872627 |
| 1 | -2.07218599 | -3.81248236 | -1.67698073 |
| 1 | -2.17613769 | -4.38911295 | 0.54645854 |
| 1 | -2.57352805 | -2.97676420 | 2.48337913 |
| 1 | -2.62106991 | -0.52613503 | 2.38861609 |
| 1 | -3.99458814 | -1.17762411 | 1.45733488 |
| 8 | -2.10448194 | 0.28583050 | -1.83793998 |
| 8 | -2.48962140 | -0.36695406 | 0.33971184 |
| 1 | -4.03858614 | 1.91529286 | -2.22151208 |
| 1 | -4.03672028 | 1.82046497 | -0.42211896 |
| 7 | -0.39413863 | 2.45767665 | -0.54459500 |
| 8 | 0.64137739 | -0.35306275 | -1.01293838 |
| 8 | -0.28950313 | 0.57301384 | 1.66566610 |
| 6 | -1.04574418 | 3.06444812 | -1.72935116 |
| 6 | 1.41468763 | 0.27021325 | -1.93366289 |
| 6 | 2.07479548 | -0.48668838 | -2.95260882 |
| 6 | 2.87006164 | 0.21297105 | -3.87102938 |
| 6 | 3.03704596 | 1.59915698 | -3.83517122 |
| 6 | 2.40829253 | 2.32489920 | -2.83108759 |
| 6 | 1.61895418 | 1.67624187 | -1.87447107 |
| 6 | 1.10550368 | 2.47539735 | -0.70903146 |
| 1 | 3.65958476 | 2.09766412 | -4.57802439 |
| 6 | 1.90075421 | -2.01253605 | -3.08148026 |
| 6 | 0.23550312 | 1.60066819 | 2.34862471 |
| 6 | 1.02587688 | 1.36262929 | 3.51913261 |
| 6 | 1.51985359 | 2.47876239 | 4.20664835 |
| 6 | 1.29195058 | 3.79391170 | 3.78929496 |
| 6 | 0.55081350 | 4.01571703 | 2.63498211 |
| 6 | 0.01729410 | 2.93741632 | 1.91956055 |
| 6 | -0.80781674 | 3.22173715 | 0.69470012 |
| 1 | 1.70425713 | 4.62893724 | 4.35537672 |
| 6 | 1.33903551 | -0.06645552 | 4.00371599 |
| 6 | 2.82086372 | -2.61962581 | -4.15836191 |
| 6 | 0.44204736 | -2.30198669 | -3.50333166 |
| 6 | 2.24007130 | -2.72771168 | -1.75240672 |
| 6 | 2.22688556 | -0.07129417 | 5.26247358 |
| 6 | 2.10010862 | -0.84301215 | 2.90261865 |
| 6 | 0.02906763 | -0.80379730 | 4.36453152 |

| | | | |
|---|---|---|---|
| 1 | -0.66754353 | 4.09008217 | -1.87126708 |
| 1 | -0.83136749 | 2.46639609 | -2.61949992 |
| 1 | 1.42522097 | 3.52631187 | -0.80436552 |
| 1 | 1.52003944 | 2.08996701 | 0.23215805 |
| 1 | 3.37939143 | -0.34394652 | -4.65519285 |
| 1 | 2.54504251 | 3.40659046 | -2.76081538 |
| 1 | -1.87149358 | 2.97475457 | 0.85487008 |
| 1 | -0.74951279 | 4.29819345 | 0.46342707 |
| 1 | 2.11924362 | 2.32267594 | 5.10181713 |
| 1 | 0.37465766 | 5.03298807 | 2.27713943 |
| 1 | 2.66265154 | -3.70741200 | -4.18797731 |
| 1 | 2.60368371 | -2.23066735 | -5.16331911 |
| 1 | 3.88395023 | -2.44421124 | -3.93820262 |
| 1 | -0.26824111 | -1.82217503 | -2.82305336 |
| 1 | 0.25529021 | -1.91659570 | -4.51632023 |
| 1 | 0.25221595 | -3.38669276 | -3.51024246 |
| 1 | 3.28817654 | -2.54273987 | -1.47446859 |
| 1 | 1.60360372 | -2.38651371 | -0.93037456 |
| 1 | 2.11181593 | -3.81417823 | -1.87422073 |
| 1 | 2.42118287 | -1.11254168 | 5.55724859 |
| 1 | 1.74248636 | 0.42812189 | 6.11412811 |
| 1 | 3.19997096 | 0.40874645 | 5.08448887 |
| 1 | 1.52076197 | -0.90457571 | 1.97511017 |
| 1 | 3.05891466 | -0.35270077 | 2.67914867 |
| 1 | 2.31714082 | -1.86508071 | 3.24904823 |
| 1 | -0.49499467 | -0.28664887 | 5.18170881 |
| 1 | -0.64127594 | -0.86061299 | 3.50111127 |
| 1 | 0.25461352 | -1.82734489 | 4.70117712 |
| 1 | -2.12859130 | 3.09276342 | -1.57260048 |
| 31 | -0.82891864 | 0.46552292 | -0.17342614 |

Electronic energy = -3523.844803 a.u.
DFT-D3(BJ) dispersion correction = -0.121023 a.u.
Thermal free energy = 0.605940 a.u.
Gibbs free energy = -3523.359885 a.u.
Number of imaginary frequencies = 1.

**TS2 eq CL isomer/conformer 6**

| Atomic N. | X | Y | Z |
|---|---|---|---|
| 7 | 0.90344552 | 1.62534592 | 1.61803933 |
| 8 | -1.36183483 | 0.03704816 | 0.72026516 |
| 8 | 1.33267326 | 0.60391691 | -1.04802806 |
| 6 | 0.80293401 | 1.45461677 | 3.08653709 |
| 6 | -2.09037529 | 1.05514339 | 1.23693380 |
| 6 | -3.47434037 | 0.84191416 | 1.53689311 |
| 6 | -4.20555494 | 1.91888605 | 2.05542594 |
| 6 | -3.63835390 | 3.17012050 | 2.30569125 |
| 6 | -2.29034573 | 3.36213891 | 2.03094638 |
| 6 | -1.51605042 | 2.32789864 | 1.49196519 |
| 6 | -0.08842007 | 2.63554703 | 1.12725153 |
| 1 | -4.24466577 | 3.97779881 | 2.71554129 |
| 6 | -4.14783641 | -0.52880375 | 1.33254909 |
| 6 | 2.03461603 | 1.73202915 | -1.24201379 |

| | | | |
|---|---|---|---|
| 6 | 2.30839444 | 2.15803583 | -2.57977740 |
| 6 | 3.04351879 | 3.33836463 | -2.74736947 |
| 6 | 3.50135177 | 4.10396490 | -1.67039425 |
| 6 | 3.22518772 | 3.68254198 | -0.37464034 |
| 6 | 2.50937642 | 2.50090814 | -0.14885962 |
| 6 | 2.30727847 | 2.04015191 | 1.27151639 |
| 1 | 4.06198473 | 5.02153684 | -1.84854913 |
| 6 | 1.81285377 | 1.34699390 | -3.79359974 |
| 6 | -5.65100621 | -0.50112099 | 1.67018064 |
| 6 | -3.48844846 | -1.55484652 | 2.28415208 |
| 6 | -4.02839473 | -0.98297336 | -0.14112433 |
| 6 | 2.24798416 | 1.98168602 | -5.12784935 |
| 6 | 0.26672596 | 1.28463733 | -3.79621653 |
| 6 | 2.39654704 | -0.08618205 | -3.75372695 |
| 1 | 0.95284080 | 2.42526560 | 3.58790893 |
| 1 | -0.18691550 | 1.06719638 | 3.35002747 |
| 1 | 0.18738445 | 3.62479888 | 1.53095156 |
| 1 | 0.03545213 | 2.68921843 | 0.03576320 |
| 1 | -5.26071037 | 1.77838682 | 2.28188675 |
| 1 | -1.82015116 | 4.32986181 | 2.22026779 |
| 1 | 2.93748994 | 1.16623405 | 1.50312569 |
| 1 | 2.60248148 | 2.85429511 | 1.95588454 |
| 1 | 3.26628618 | 3.68415164 | -3.75531160 |
| 1 | 3.57173013 | 4.26623128 | 0.48160003 |
| 1 | -6.07194324 | -1.50269603 | 1.50028591 |
| 1 | -5.83780828 | -0.23983220 | 2.72159188 |
| 1 | -6.20430795 | 0.20302726 | 1.03215246 |
| 1 | -2.40418302 | -1.60002318 | 2.13286302 |
| 1 | -3.67865216 | -1.27803739 | 3.33159451 |
| 1 | -3.91019261 | -2.55800282 | 2.11649506 |
| 1 | -4.55265657 | -0.27621289 | -0.80089833 |
| 1 | -2.98393999 | -1.04159789 | -0.46240041 |
| 1 | -4.49511731 | -1.97193426 | -0.26765179 |
| 1 | 1.87510838 | 1.36291501 | -5.95667246 |
| 1 | 3.34229355 | 2.03730774 | -5.22194086 |
| 1 | 1.83610576 | 2.99271678 | -5.25947797 |
| 1 | -0.11637158 | 0.80204519 | -2.89058097 |
| 1 | -0.16048231 | 2.29603763 | -3.86106271 |
| 1 | -0.08265660 | 0.71361346 | -4.67018162 |
| 1 | 3.49575192 | -0.05491087 | -3.79407693 |
| 1 | 2.09080394 | -0.61355607 | -2.84339338 |
| 1 | 2.04317237 | -0.65552175 | -4.62738182 |
| 1 | 1.56881635 | 0.74568884 | 3.41569320 |
| 8 | 3.22772170 | -1.76481395 | 0.53109112 |
| 6 | 3.73190307 | -2.69031597 | -0.45474632 |
| 1 | 4.43667843 | -2.10992937 | -1.05812829 |
| 6 | 1.97650057 | -3.53727542 | 1.71883696 |
| 6 | 0.54614999 | -3.97064723 | 2.08088440 |
| 6 | -0.27763220 | -4.59489959 | 0.93230735 |
| 6 | 2.15872371 | -2.10294939 | 1.28656412 |
| 6 | -1.07471438 | -3.63562098 | 0.03457922 |
| 6 | -0.24812485 | -2.73805073 | -0.88255935 |
| 1 | 2.39799446 | -4.22011366 | 0.97014321 |

| | | | |
|---|---|---|---|
| 1 | 0.00895792 | -3.11817429 | 2.52214055 |
| 1 | 0.38402993 | -5.22498327 | 0.31073520 |
| 1 | -1.71686392 | -3.00175405 | 0.66272300 |
| 1 | 2.62473864 | -3.62445707 | 2.61111848 |
| 1 | 0.63413570 | -4.72459463 | 2.87798011 |
| 1 | -1.00113571 | -5.28907963 | 1.38880451 |
| 1 | -1.75104374 | -4.23163028 | -0.60285985 |
| 1 | -0.92605005 | -2.11306658 | -1.49205973 |
| 1 | 0.32733506 | -3.36280940 | -1.59469971 |
| 8 | 1.64209332 | -1.13751212 | 1.92411406 |
| 8 | 0.66262404 | -1.91220310 | -0.17731250 |
| 1 | 2.90828139 | -3.04186033 | -1.08903174 |
| 1 | 4.25666709 | -3.53265600 | 0.01610994 |
| 31 | 0.50215611 | -0.11456872 | 0.48446480 |

Electronic energy = -3523.843836 a.u.
DFT-D3(BJ) dispersion correction = -0.119269 a.u.
Thermal free energy = 0.603613 a.u.
Gibbs free energy = -3523.359492 a.u.
Number of imaginary frequencies = 1.

**TS2 eq CL isomer/conformer 7**

| Atomic N. | X | Y | Z |
|---|---|---|---|
| 7 | 0.13488743 | 1.93640530 | 1.53562391 |
| 8 | -0.51572627 | 0.87273335 | -1.12614322 |
| 8 | 1.08645988 | -0.77076632 | 1.02472341 |
| 6 | -0.95587343 | 2.90252280 | 1.80337214 |
| 6 | -0.03517117 | 2.05741453 | -1.57742560 |
| 6 | -0.22556067 | 2.41463208 | -2.95197606 |
| 6 | 0.19545145 | 3.68728375 | -3.36172843 |
| 6 | 0.80766094 | 4.59936857 | -2.50053716 |
| 6 | 1.05656266 | 4.21557713 | -1.18927848 |
| 6 | 0.66923088 | 2.95197320 | -0.72664416 |
| 6 | 1.16455472 | 2.53454709 | 0.63264346 |
| 1 | 1.10609305 | 5.58372307 | -2.86091566 |
| 6 | -0.84685045 | 1.44248414 | -3.97540331 |
| 6 | 2.10183764 | -0.42659903 | 1.83515751 |
| 6 | 3.30565929 | -1.19964397 | 1.79131365 |
| 6 | 4.34951401 | -0.82992715 | 2.64831066 |
| 6 | 4.26419878 | 0.26287365 | 3.51599050 |
| 6 | 3.10016513 | 1.02159202 | 3.53331184 |
| 6 | 2.01502109 | 0.68654412 | 2.71358538 |
| 6 | 0.75667548 | 1.51184821 | 2.83924103 |
| 1 | 5.10523510 | 0.52056593 | 4.15959787 |
| 6 | 3.45726204 | -2.39172316 | 0.82439226 |
| 6 | -0.81376815 | 2.00180554 | -5.41095400 |
| 6 | -2.32529163 | 1.18380475 | -3.61821675 |
| 6 | -0.05484385 | 0.11350633 | -3.99394321 |
| 6 | 4.82738924 | -3.08078170 | 0.96875280 |
| 6 | 3.34092999 | -1.90211821 | -0.63927680 |
| 6 | 2.37453485 | -3.46008778 | 1.10756552 |
| 1 | -0.54504311 | 3.81164336 | 2.27339244 |
| 1 | -1.44031000 | 3.18010902 | 0.86018312 |

| | | | |
|---|---|---|---|
| 1 | 1.61327922 | 3.40514040 | 1.14200389 |
| 1 | 1.95902324 | 1.78073025 | 0.53081864 |
| 1 | 0.04363264 | 3.98237133 | -4.39802170 |
| 1 | 1.58150411 | 4.88808584 | -0.50690728 |
| 1 | -0.03237847 | 0.96373862 | 3.37606359 |
| 1 | 0.98748946 | 2.42274523 | 3.41822386 |
| 1 | 5.27247381 | -1.40687478 | 2.63746452 |
| 1 | 3.01676655 | 1.88743591 | 4.19456863 |
| 1 | -1.23920941 | 1.25155795 | -6.09309578 |
| 1 | -1.41334450 | 2.91737270 | -5.51636267 |
| 1 | 0.21058017 | 2.21472669 | -5.74877167 |
| 1 | -2.41490793 | 0.78686714 | -2.60408926 |
| 1 | -2.90639853 | 2.11579299 | -3.67948651 |
| 1 | -2.76742911 | 0.46073329 | -4.32137775 |
| 1 | 0.98470098 | 0.29065335 | -4.30598545 |
| 1 | -0.03928780 | -0.36336055 | -3.00814033 |
| 1 | -0.50948328 | -0.58212411 | -4.71590948 |
| 1 | 4.88061476 | -3.92101073 | 0.26162887 |
| 1 | 4.98202658 | -3.48736477 | 1.97888339 |
| 1 | 5.65986538 | -2.40103483 | 0.73642218 |
| 1 | 2.36894417 | -1.43444788 | -0.82996100 |
| 1 | 4.13172960 | -1.17199898 | -0.86555433 |
| 1 | 3.45920873 | -2.75270987 | -1.32807171 |
| 1 | 2.46924472 | -3.84055638 | 2.13587666 |
| 1 | 1.36875248 | -3.04887176 | 0.97217733 |
| 1 | 2.50303531 | -4.31046247 | 0.42011219 |
| 1 | -1.68915343 | 2.43923044 | 2.47080946 |
| 8 | -1.23589575 | -1.89643705 | 2.78614855 |
| 6 | -1.29643857 | -3.33409429 | 2.72995043 |
| 1 | -0.35811549 | -3.68179965 | 3.17312050 |
| 6 | -3.60779977 | -1.69126821 | 2.19594383 |
| 6 | -4.66284895 | -0.86375993 | 1.45729601 |
| 6 | -4.83851910 | -1.22346926 | -0.02263699 |
| 6 | -2.18547273 | -1.17143953 | 2.12989759 |
| 6 | -3.66711497 | -0.87421697 | -0.94936579 |
| 6 | -2.40109372 | -1.72978389 | -0.82860661 |
| 1 | -3.63999629 | -2.73881030 | 1.86522794 |
| 1 | -4.42288208 | 0.20517506 | 1.56282592 |
| 1 | -5.06221962 | -2.30193090 | -0.10991785 |
| 1 | -3.39682341 | 0.18670550 | -0.82241344 |
| 1 | -3.83519769 | -1.70501864 | 3.27732682 |
| 1 | -5.62683344 | -1.01997030 | 1.96547961 |
| 1 | -5.73428774 | -0.69965690 | -0.39337870 |
| 1 | -4.01440001 | -0.97770727 | -1.99083614 |
| 1 | -1.78214824 | -1.57605255 | -1.72792208 |
| 1 | -2.68043470 | -2.80026484 | -0.80824894 |
| 8 | -1.93545032 | 0.08859869 | 2.10235310 |
| 8 | -1.57044327 | -1.47423410 | 0.30448464 |
| 1 | -1.35246062 | -3.66570234 | 1.68405318 |
| 1 | -2.14409089 | -3.72558880 | 3.31032062 |
| 31 | -0.49119675 | 0.15305053 | 0.62086511 |

Electronic energy = -3523.843262 a.u.
DFT-D3(BJ) dispersion correction = -0.119372 a.u.

Thermal free energy = 0.605347 a.u.
Gibbs free energy = -3523.357287 a.u.
Number of imaginary frequencies = 1.

**TS2_eq_CL isomer/conformer 8**

| Atomic N. | X | Y | Z |
|---|---|---|---|
| 7 | -0.61901093 | 2.16596150 | 1.45205033 |
| 8 | -1.45021105 | 0.13630933 | -0.50437188 |
| 8 | 1.61712027 | 0.75047910 | 0.12168191 |
| 6 | -1.55419886 | 2.16702580 | 2.60329652 |
| 6 | -2.55313015 | 0.90788454 | -0.59270179 |
| 6 | -3.75985527 | 0.35423288 | -1.11967146 |
| 6 | -4.87692261 | 1.19713748 | -1.19979215 |
| 6 | -4.84965992 | 2.53280401 | -0.78762591 |
| 6 | -3.66737437 | 3.06186628 | -0.28096798 |
| 6 | -2.51965880 | 2.26728177 | -0.18988404 |
| 6 | -1.22668564 | 2.87663531 | 0.27647495 |
| 1 | -5.74567223 | 3.14864969 | -0.86546934 |
| 6 | -3.83410501 | -1.11389554 | -1.58333302 |
| 6 | 2.09539795 | 1.99756062 | -0.05995677 |
| 6 | 3.07620430 | 2.24165535 | -1.07070768 |
| 6 | 3.55826998 | 3.55075407 | -1.20358002 |
| 6 | 3.11459756 | 4.60701704 | -0.40261272 |
| 6 | 2.15558791 | 4.35959959 | 0.57297009 |
| 6 | 1.65100634 | 3.06743193 | 0.75895315 |
| 6 | 0.66967887 | 2.82105541 | 1.87358308 |
| 1 | 3.51405215 | 5.61067820 | -0.54770398 |
| 6 | 3.58212018 | 1.11286223 | -1.99003160 |
| 6 | -5.22816992 | -1.47839522 | -2.12762904 |
| 6 | -3.54204893 | -2.06158137 | -0.39521983 |
| 6 | -2.81739473 | -1.36263728 | -2.72295547 |
| 6 | 4.65062189 | 1.60783935 | -2.98299932 |
| 6 | 2.40770197 | 0.54916310 | -2.82451129 |
| 6 | 4.22824430 | -0.01229922 | -1.14749551 |
| 1 | -1.80420077 | 3.20533252 | 2.87930989 |
| 1 | -2.47415113 | 1.63762522 | 2.33465505 |
| 1 | -1.38523948 | 3.93457937 | 0.54840428 |
| 1 | -0.47207662 | 2.85254908 | -0.52347696 |
| 1 | -5.81077576 | 0.80057853 | -1.59456193 |
| 1 | -3.61922097 | 4.10568476 | 0.03816005 |
| 1 | 1.09822834 | 2.15831447 | 2.64280939 |
| 1 | 0.42439252 | 3.78186417 | 2.35807657 |
| 1 | 4.30573463 | 3.76323915 | -1.96590984 |
| 1 | 1.79294527 | 5.16945982 | 1.21030879 |
| 1 | -5.22272015 | -2.52999163 | -2.44889331 |
| 1 | -6.01255751 | -1.36976528 | -1.36452842 |
| 1 | -5.50415897 | -0.86770606 | -2.99940515 |
| 1 | -2.54676938 | -1.88540637 | 0.02730511 |
| 1 | -4.28709173 | -1.92131877 | 0.40184745 |
| 1 | -3.60055447 | -3.10852385 | -0.73203093 |
| 1 | -3.05120325 | -0.73138416 | -3.59269929 |
| 1 | -1.79361510 | -1.14308584 | -2.40106559 |

| | | | |
|---|---|---|---|
| 1 | -2.86839342 | -2.41416025 | -3.04580855 |
| 1 | 4.97753620 | 0.76309359 | -3.60630083 |
| 1 | 5.53900862 | 2.00471473 | -2.47065473 |
| 1 | 4.26177502 | 2.38424182 | -3.65746832 |
| 1 | 1.60584509 | 0.16300564 | -2.18597984 |
| 1 | 1.98468995 | 1.33139431 | -3.47136760 |
| 1 | 2.76437831 | -0.26747209 | -3.47099757 |
| 1 | 5.09838915 | 0.37132105 | -0.59461641 |
| 1 | 3.51533508 | -0.42854840 | -0.42757115 |
| 1 | 4.57785034 | -0.82041556 | -1.80851626 |
| 1 | -1.08291757 | 1.65797865 | 3.44921184 |
| 8 | -1.01770186 | -2.34541845 | 2.92546058 |
| 6 | -1.00871944 | -3.75230026 | 3.20798540 |
| 1 | -2.04408765 | -3.99623537 | 3.46770072 |
| 6 | 1.48499525 | -2.14909697 | 3.13803220 |
| 6 | 2.14755678 | -3.46023417 | 2.68017340 |
| 6 | 2.54497218 | -3.57362843 | 1.19926929 |
| 6 | 0.12354219 | -1.66431022 | 2.64385438 |
| 6 | 1.38319337 | -3.83004308 | 0.22508892 |
| 6 | 0.69185972 | -2.56732440 | -0.27689213 |
| 1 | 2.16185617 | -1.31609619 | 2.90532422 |
| 1 | 1.54415882 | -4.33696747 | 2.95491195 |
| 1 | 3.25809956 | -4.40921974 | 1.12113488 |
| 1 | 0.64794981 | -4.50778675 | 0.69133466 |
| 1 | 1.39123714 | -2.16698670 | 4.23993063 |
| 1 | 3.06729436 | -3.54093289 | 3.28233910 |
| 1 | 3.09551740 | -2.66989470 | 0.88985336 |
| 1 | 1.75496781 | -4.36074734 | -0.66648668 |
| 1 | -0.19207792 | -2.83673334 | -0.88166416 |
| 1 | 1.39443350 | -2.01746583 | -0.92780358 |
| 8 | -0.07819577 | -0.38110298 | 2.65598536 |
| 8 | 0.30027407 | -1.68953049 | 0.77967536 |
| 1 | -0.71032411 | -4.33546782 | 2.32612753 |
| 1 | -0.35507131 | -3.98969746 | 4.05879736 |
| 31 | -0.07269715 | 0.23694752 | 0.77184266 |

Electronic energy = -3523.842754 a.u.
DFT-D3(BJ) dispersion correction = -0.118193 a.u.
Thermal free energy = 0.603991 a.u.
Gibbs free energy = -3523.356956 a.u.
Number of imaginary frequencies = 1.

**TS2 eq CL isomer/conformer 9**

| Atomic N. | X | Y | Z |
|---|---|---|---|
| 7 | 0.90782589 | 1.63203526 | 1.79488826 |
| 8 | -1.07890904 | -0.08283364 | 0.43289262 |
| 8 | 0.92367142 | 1.25113928 | -1.23847139 |
| 6 | 1.61419845 | 1.20608330 | 3.02835989 |
| 6 | -1.76832771 | -0.00873150 | 1.58659458 |
| 6 | -2.88209176 | -0.86946166 | 1.83278096 |
| 6 | -3.48057270 | -0.79269826 | 3.09953332 |
| 6 | -3.06750441 | 0.10987335 | 4.08513212 |
| 6 | -2.05546618 | 1.01941967 | 3.79108739 |

| | | | |
|---|---|---|---|
| 6 | -1.42206788 | 0.98167509 | 2.54522943 |
| 6 | -0.48902944 | 2.08199358 | 2.12520552 |
| 1 | -3.56219959 | 0.12297510 | 5.05629158 |
| 6 | -3.45807695 | -1.78356242 | 0.73096365 |
| 6 | 0.71545458 | 2.58250999 | -1.22420788 |
| 6 | 0.19991192 | 3.21936250 | -2.39513516 |
| 6 | 0.00411288 | 4.60550165 | -2.34725714 |
| 6 | 0.28699937 | 5.37233782 | -1.21278512 |
| 6 | 0.79727823 | 4.74514008 | -0.08264086 |
| 6 | 1.02867723 | 3.36337185 | -0.07769516 |
| 6 | 1.68824100 | 2.75042629 | 1.13306165 |
| 1 | 0.11039672 | 6.44784164 | -1.21937358 |
| 6 | -0.12826467 | 2.41217899 | -3.66706347 |
| 6 | -4.70885229 | -2.54338384 | 1.21106279 |
| 6 | -2.43036866 | -2.83857632 | 0.26331714 |
| 6 | -3.87867832 | -0.91031152 | -0.47613648 |
| 6 | -0.63792890 | 3.31442738 | -4.80677700 |
| 6 | -1.23896027 | 1.37711418 | -3.36906528 |
| 6 | 1.14124441 | 1.69309688 | -4.18395615 |
| 1 | 1.70288432 | 2.06214476 | 3.71740818 |
| 1 | 1.03717351 | 0.41489515 | 3.52151990 |
| 1 | -0.42325538 | 2.85342169 | 2.91141510 |
| 1 | -0.87059420 | 2.56314063 | 1.21311545 |
| 1 | -4.31226015 | -1.45649457 | 3.33011341 |
| 1 | -1.76890945 | 1.78562760 | 4.51568604 |
| 1 | 2.66268992 | 2.30999494 | 0.87503231 |
| 1 | 1.85439885 | 3.53462720 | 1.89041770 |
| 1 | -0.39114577 | 5.11363411 | -3.22506738 |
| 1 | 1.03694081 | 5.32681894 | 0.81065071 |
| 1 | -5.09588146 | -3.15355396 | 0.38240844 |
| 1 | -4.48607302 | -3.22374415 | 2.04626608 |
| 1 | -5.51251411 | -1.86135840 | 1.52340424 |
| 1 | -1.53587663 | -2.36779547 | -0.15908571 |
| 1 | -2.13285279 | -3.49077177 | 1.09849656 |
| 1 | -2.88333225 | -3.47615290 | -0.51152390 |
| 1 | -4.64902401 | -0.18392177 | -0.17932525 |
| 1 | -3.02376699 | -0.36361569 | -0.89020878 |
| 1 | -4.30094385 | -1.54731238 | -1.26844966 |
| 1 | -0.84637076 | 2.69148898 | -5.68845034 |
| 1 | 0.10605204 | 4.06846571 | -5.10238266 |
| 1 | -1.57127261 | 3.83144689 | -4.54058313 |
| 1 | -0.93562013 | 0.67167836 | -2.58861899 |
| 1 | -2.15919328 | 1.88175499 | -3.04045987 |
| 1 | -1.47263777 | 0.80962741 | -4.28315306 |
| 1 | 1.91909468 | 2.42464828 | -4.44803238 |
| 1 | 1.54806149 | 1.00967717 | -3.43082714 |
| 1 | 0.89744401 | 1.11797154 | -5.09076262 |
| 1 | 2.60831141 | 0.83233976 | 2.76476312 |
| 8 | 2.90818977 | -1.01869309 | -1.40334368 |
| 6 | 2.89647460 | -2.14629269 | -2.29145598 |
| 1 | 2.78105497 | -1.72133362 | -3.29445577 |
| 6 | 3.92187953 | -2.28880572 | 0.48869509 |
| 6 | 3.57683301 | -3.79010677 | 0.42480505 |

| | | | |
|---|---|---|---|
| 6 | 2.65923238 | -4.30735540 | 1.54157519 |
| 6 | 2.97422481 | -1.20900846 | -0.04242024 |
| 6 | 1.18664050 | -3.89679670 | 1.44000256 |
| 6 | 0.93343729 | -2.39502192 | 1.48403275 |
| 1 | 4.13302374 | -2.01170492 | 1.53118753 |
| 1 | 3.15205836 | -4.06252241 | -0.55021572 |
| 1 | 2.71450973 | -5.40783882 | 1.54830766 |
| 1 | 0.73619133 | -4.29460144 | 0.51609159 |
| 1 | 4.85971785 | -2.11323643 | -0.06563270 |
| 1 | 4.53344917 | -4.33165598 | 0.49919331 |
| 1 | 3.06025791 | -3.97698212 | 2.51589298 |
| 1 | 0.63457781 | -4.35413218 | 2.27771115 |
| 1 | -0.12201903 | -2.20166945 | 1.72316861 |
| 1 | 1.54787481 | -1.92776930 | 2.28043056 |
| 8 | 2.87812972 | -0.10446891 | 0.61296874 |
| 8 | 1.20968330 | -1.76328218 | 0.21566048 |
| 1 | 2.04365444 | -2.80311561 | -2.07062769 |
| 1 | 3.83984995 | -2.71030855 | -2.24382067 |
| 31 | 0.80883497 | 0.19907390 | 0.29264620 |

Electronic energy = -3523.833621 a.u.
DFT-D3(BJ) dispersion correction = -0.119850 a.u.
Thermal free energy = 0.606411 a.u.
Gibbs free energy = -3523.347061 a.u.
Number of imaginary frequencies = 1.

**Int2 eq CL isomer/conformer 1**

| Atomic N. | X | Y | Z |
|---|---|---|---|
| 7 | -1.30119536 | 1.46525298 | 1.56984126 |
| 8 | -0.79293102 | 0.67955210 | -1.19691330 |
| 8 | 1.21187305 | -0.05734355 | 1.20553862 |
| 6 | -2.75924067 | 1.42178260 | 1.84245735 |
| 6 | -1.53688986 | 1.76499410 | -1.48273959 |
| 6 | -2.16120684 | 1.88362382 | -2.76214353 |
| 6 | -2.91762509 | 3.03841403 | -3.00572810 |
| 6 | -3.07986125 | 4.05381491 | -2.05777784 |
| 6 | -2.46531183 | 3.92756360 | -0.81615913 |
| 6 | -1.69045673 | 2.79981646 | -0.52670802 |
| 6 | -0.95951488 | 2.70170999 | 0.78089946 |
| 1 | -3.68012702 | 4.93245183 | -2.29394550 |
| 6 | -2.01167061 | 0.78523696 | -3.83245123 |
| 6 | 1.74213720 | 0.93645604 | 1.94946595 |
| 6 | 3.14991541 | 1.17884847 | 1.91178157 |
| 6 | 3.65159069 | 2.20639413 | 2.72236978 |
| 6 | 2.83659774 | 2.99340595 | 3.54080053 |
| 6 | 1.46713160 | 2.75572334 | 3.56080015 |
| 6 | 0.91479085 | 1.73110246 | 2.78353829 |
| 6 | -0.56223485 | 1.45702149 | 2.88485999 |
| 1 | 3.27216873 | 3.78619926 | 4.14879967 |
| 6 | 4.08800063 | 0.35304456 | 1.00961183 |
| 6 | -2.76174671 | 1.13702350 | -5.13074183 |
| 6 | -2.59722313 | -0.54812564 | -3.30819813 |
| 6 | -0.51964012 | 0.59947593 | -4.19744064 |

| | | | |
|---|---|---|---|
| 6 | 5.55940022 | 0.78587920 | 1.15311260 |
| 6 | 3.69632136 | 0.54618993 | -0.47446318 |
| 6 | 4.01337991 | -1.14412675 | 1.39041897 |
| 1 | -3.06553246 | 2.34187460 | 2.36728367 |
| 1 | -3.31151066 | 1.35406669 | 0.89905686 |
| 1 | -1.18142730 | 3.58223409 | 1.40755667 |
| 1 | 0.12821449 | 2.67681085 | 0.62158865 |
| 1 | -3.40639212 | 3.15529217 | -3.97153107 |
| 1 | -2.56980576 | 4.71195041 | -0.06272248 |
| 1 | -0.75845504 | 0.46781026 | 3.32601558 |
| 1 | -1.02530192 | 2.21676295 | 3.53720896 |
| 1 | 4.72059826 | 2.41104353 | 2.71352013 |
| 1 | 0.80972582 | 3.36043435 | 4.18984639 |
| 1 | -2.61755375 | 0.32540058 | -5.85830878 |
| 1 | -3.84384340 | 1.24705636 | -4.96790473 |
| 1 | -2.38333366 | 2.06241476 | -5.58845528 |
| 1 | -2.07736924 | -0.88646407 | -2.40452756 |
| 1 | -3.66771621 | -0.43449627 | -3.07957288 |
| 1 | -2.49891250 | -1.32823948 | -4.07888591 |
| 1 | -0.10660499 | 1.52896678 | -4.61576039 |
| 1 | 0.07478878 | 0.31925385 | -3.32106745 |
| 1 | -0.41662483 | -0.18896159 | -4.95892583 |
| 1 | 6.18009957 | 0.16334364 | 0.49286186 |
| 1 | 5.93178461 | 0.65330754 | 2.17928702 |
| 1 | 5.71391303 | 1.83400250 | 0.85898973 |
| 1 | 2.66329033 | 0.23621780 | -0.66530993 |
| 1 | 3.79942990 | 1.60144557 | -0.76618322 |
| 1 | 4.36206898 | -0.04887716 | -1.11810159 |
| 1 | 4.33521275 | -1.29489733 | 2.43141259 |
| 1 | 2.99487662 | -1.53119642 | 1.28057601 |
| 1 | 4.68323579 | -1.72882509 | 0.74147097 |
| 1 | -2.98259264 | 0.54933529 | 2.46252411 |
| 8 | -2.43509969 | -3.41128084 | 1.28574266 |
| 6 | -3.62155298 | -2.81389757 | 0.70571752 |
| 1 | -3.33754468 | -2.22024186 | -0.17205089 |
| 6 | -0.29166365 | -3.24603372 | 2.30449582 |
| 6 | 0.18034393 | -4.55680230 | 1.65186607 |
| 6 | 1.12644205 | -4.44295448 | 0.44049850 |
| 6 | -1.49367154 | -2.55931869 | 1.70644542 |
| 6 | 0.53612506 | -3.98316947 | -0.90750060 |
| 6 | 0.43522244 | -2.46975893 | -1.09449199 |
| 1 | 0.51397027 | -2.50082032 | 2.34790466 |
| 1 | 0.71811895 | -5.12149511 | 2.42980136 |
| 1 | 1.55765142 | -5.44655264 | 0.29326550 |
| 1 | 1.17730748 | -4.36533479 | -1.71972881 |
| 1 | -0.58398533 | -3.45253194 | 3.35139355 |
| 1 | -0.69393140 | -5.16951191 | 1.38500691 |
| 1 | 1.97619863 | -3.78979569 | 0.70337704 |
| 1 | -0.45934892 | -4.43316202 | -1.05961711 |
| 1 | 0.13430784 | -2.24646344 | -2.13537858 |
| 1 | 1.43447815 | -2.02122316 | -0.93517594 |
| 8 | -1.74335974 | -1.33350835 | 1.85670403 |
| 8 | -0.51722098 | -1.93451393 | -0.18639899 |

| | | | |
|---|---|---|---|
| 1 | -4.25199021 | -3.65889579 | 0.41429644 |
| 1 | -4.13372048 | -2.18235329 | 1.44108491 |
| 31 | -0.51777045 | -0.16882859 | 0.47191441 |

Electronic energy = -3523.859584 a.u.
DFT-D3(BJ) dispersion correction = -0.118859 a.u.
Thermal free energy = 0.602971 a.u.
Gibbs free energy = -3523.375472 a.u.
Number of imaginary frequencies = 0.

**Int2 eq CL isomer/conformer 2**

| Atomic N. | X | Y | Z |
|---|---|---|---|
| 8 | -1.39524773 | -1.85027881 | 3.55073789 |
| 6 | -0.11128784 | -1.44233759 | 4.09220418 |
| 1 | 0.62254447 | -1.36142293 | 3.28049604 |
| 6 | -3.26656940 | -1.52071513 | 2.12284734 |
| 6 | -3.47683843 | -3.03315430 | 1.93651010 |
| 6 | -2.95723477 | -3.67226270 | 0.63234058 |
| 6 | -1.91707220 | -1.05476716 | 2.61181163 |
| 6 | -1.43808119 | -3.90554971 | 0.49874029 |
| 6 | -0.65507032 | -2.71609873 | -0.05652584 |
| 1 | -3.47905752 | -0.96994912 | 1.19771315 |
| 1 | -4.56468381 | -3.20360181 | 1.97174994 |
| 1 | -3.45675701 | -4.65127370 | 0.55192751 |
| 1 | -1.26400146 | -4.75670763 | -0.18044420 |
| 1 | -3.98348861 | -1.15367461 | 2.88120972 |
| 1 | -3.06007273 | -3.56866662 | 2.80309830 |
| 1 | -3.31299316 | -3.08390836 | -0.23075464 |
| 1 | -1.01705216 | -4.19473691 | 1.47601643 |
| 1 | 0.41614123 | -2.98386765 | -0.13660088 |
| 1 | -1.01747078 | -2.48302334 | -1.07550483 |
| 8 | -1.47190525 | 0.11028432 | 2.41527212 |
| 8 | -0.83184313 | -1.60564432 | 0.80317728 |
| 1 | 0.16478249 | -2.23547970 | 4.79289033 |
| 1 | -0.20634055 | -0.48147116 | 4.61213102 |
| 7 | -0.25481875 | 2.26602652 | 0.99219856 |
| 8 | -0.74128649 | 0.40712604 | -1.20688454 |
| 8 | 1.53039658 | -0.06340419 | 1.08351415 |
| 6 | -1.56623575 | 2.86351139 | 1.34432353 |
| 6 | -1.08173343 | 1.52665568 | -1.87795652 |
| 6 | -1.88918573 | 1.43013044 | -3.05358705 |
| 6 | -2.21553440 | 2.62218405 | -3.71467623 |
| 6 | -1.78829215 | 3.87736827 | -3.27180845 |
| 6 | -0.99893199 | 3.95877100 | -2.13001416 |
| 6 | -0.63516538 | 2.79822935 | -1.43877358 |
| 6 | 0.28935295 | 2.89805497 | -0.25951986 |
| 1 | -2.07097684 | 4.77718329 | -3.81810360 |
| 6 | -2.38348335 | 0.06965276 | -3.58139285 |
| 6 | 2.48298982 | 0.85124095 | 1.34848808 |
| 6 | 3.85711171 | 0.52034469 | 1.13897766 |
| 6 | 4.81155422 | 1.49830972 | 1.44961702 |
| 6 | 4.46950516 | 2.76352666 | 1.93666545 |
| 6 | 3.12842430 | 3.07649340 | 2.12807160 |

| | | | |
|---|---|---|---|
| 6 | 2.13451965 | 2.13089189 | 1.85017343 |
| 6 | 0.69758437 | 2.47466857 | 2.14184516 |
| 1 | 5.24731070 | 3.49470369 | 2.15654703 |
| 6 | 4.27742934 | -0.85580526 | 0.58523869 |
| 6 | -3.22325311 | 0.21395282 | -4.86444303 |
| 6 | -3.28001174 | -0.61282865 | -2.52144065 |
| 6 | -1.17555192 | -0.83477337 | -3.92288431 |
| 6 | 5.80763069 | -0.98578772 | 0.46535368 |
| 6 | 3.68577360 | -1.05938791 | -0.82984840 |
| 6 | 3.79370675 | -1.98537604 | 1.52569436 |
| 1 | -1.45923981 | 3.95519333 | 1.45643442 |
| 1 | -2.29292378 | 2.65946660 | 0.55085102 |
| 1 | 0.51106516 | 3.95725134 | -0.04442997 |
| 1 | 1.24796276 | 2.40022038 | -0.46731779 |
| 1 | -2.83120927 | 2.57637737 | -4.61125757 |
| 1 | -0.64426745 | 4.92697286 | -1.76883279 |
| 1 | 0.29782778 | 1.86692347 | 2.96822495 |
| 1 | 0.63628565 | 3.53433330 | 2.44357939 |
| 1 | 5.86616420 | 1.27337798 | 1.30088361 |
| 1 | 2.83818409 | 4.05996583 | 2.50524753 |
| 1 | -3.54247761 | -0.78439807 | -5.19664369 |
| 1 | -4.13026454 | 0.81412267 | -4.70173013 |
| 1 | -2.64879841 | 0.66705426 | -5.68517826 |
| 1 | -2.73578562 | -0.77437066 | -1.58444373 |
| 1 | -4.16614187 | 0.00446201 | -2.31108228 |
| 1 | -3.62863343 | -1.58761831 | -2.89604423 |
| 1 | -0.56987115 | -0.38064990 | -4.72061502 |
| 1 | -0.53328542 | -0.99411728 | -3.04994578 |
| 1 | -1.52959036 | -1.81310862 | -4.28286814 |
| 1 | 6.05162469 | -1.98240809 | 0.07061292 |
| 1 | 6.30913372 | -0.88327217 | 1.43870730 |
| 1 | 6.23382628 | -0.24402562 | -0.22543077 |
| 1 | 2.59110941 | -1.02175875 | -0.81858182 |
| 1 | 4.05352494 | -0.28445791 | -1.51806387 |
| 1 | 3.99512473 | -2.03839834 | -1.22666563 |
| 1 | 4.22929876 | -1.86675722 | 2.52889953 |
| 1 | 2.70189580 | -1.98988231 | 1.61391940 |
| 1 | 4.11647446 | -2.96105265 | 1.13127211 |
| 1 | -1.91896671 | 2.42571909 | 2.28243292 |
| 31 | -0.27727594 | 0.18148003 | 0.61164776 |

Electronic energy = -3523.858657 a.u.
DFT-D3(BJ) dispersion correction = -0.118675 a.u.
Thermal free energy = 0.602686 a.u.
Gibbs free energy = -3523.374646 a.u.
Number of imaginary frequencies = 0.

**Int2 eq CL isomer/conformer 3**

| Atomic N. | X | Y | Z |
|---|---|---|---|
| 7 | -0.67121056 | 2.01924350 | 1.44258674 |
| 8 | -1.48550372 | 0.10170326 | -0.60476306 |
| 8 | 1.58016211 | 0.67117289 | 0.07057458 |
| 6 | -1.64674964 | 2.00194197 | 2.56136214 |

| | | | |
|---|---|---|---|
| 6 | -2.57743068 | 0.88391907 | -0.69144308 |
| 6 | -3.78378646 | 0.36798022 | -1.25741236 |
| 6 | -4.88495375 | 1.23240237 | -1.32781737 |
| 6 | -4.84441786 | 2.55315902 | -0.86958608 |
| 6 | -3.66398621 | 3.04483714 | -0.32189345 |
| 6 | -2.53299898 | 2.22639212 | -0.23910004 |
| 6 | -1.23606869 | 2.78168144 | 0.27268322 |
| 1 | -5.72888134 | 3.18593665 | -0.94354472 |
| 6 | -3.87376714 | -1.08359480 | -1.76560454 |
| 6 | 2.07095581 | 1.92251975 | -0.02959066 |
| 6 | 3.07459611 | 2.21297480 | -1.00552080 |
| 6 | 3.56815151 | 3.52340416 | -1.05882757 |
| 6 | 3.11377989 | 4.53943361 | -0.21337817 |
| 6 | 2.13145390 | 4.24801600 | 0.72605320 |
| 6 | 1.61471136 | 2.95166912 | 0.83467966 |
| 6 | 0.60738293 | 2.66302240 | 1.91738483 |
| 1 | 3.52203661 | 5.54655643 | -0.29693626 |
| 6 | 3.59400322 | 1.13089711 | -1.97259152 |
| 6 | -5.26186132 | -1.40819343 | -2.34871744 |
| 6 | -3.62355981 | -2.06715686 | -0.59710932 |
| 6 | -2.83722192 | -1.31749011 | -2.89020067 |
| 6 | 4.68541127 | 1.67131880 | -2.91577161 |
| 6 | 2.43563562 | 0.61571274 | -2.85925225 |
| 6 | 4.21779423 | -0.04017475 | -1.17725416 |
| 1 | -1.89848053 | 3.03631896 | 2.84849061 |
| 1 | -2.56156848 | 1.48932961 | 2.24553537 |
| 1 | -1.36756792 | 3.83306392 | 0.58018422 |
| 1 | -0.46481909 | 2.76164489 | -0.51082465 |
| 1 | -5.81769602 | 0.86519051 | -1.75276631 |
| 1 | -3.60467999 | 4.07585521 | 0.03505420 |
| 1 | 1.01841997 | 1.98090749 | 2.67635329 |
| 1 | 0.34109832 | 3.60809507 | 2.42055897 |
| 1 | 4.33327559 | 3.76987088 | -1.79282268 |
| 1 | 1.75886870 | 5.02661606 | 1.39590309 |
| 1 | -5.26736455 | -2.45010559 | -2.70016261 |
| 1 | -6.06040185 | -1.30772803 | -1.59926484 |
| 1 | -5.50891344 | -0.76797129 | -3.20797122 |
| 1 | -2.63854551 | -1.91325510 | -0.14227350 |
| 1 | -4.39051284 | -1.94119070 | 0.18160804 |
| 1 | -3.68212316 | -3.10398403 | -0.96349885 |
| 1 | -3.04386618 | -0.65941075 | -3.74693180 |
| 1 | -1.81719086 | -1.12246277 | -2.54194980 |
| 1 | -2.89640642 | -2.35906625 | -3.24229979 |
| 1 | 5.02050828 | 0.85864894 | -3.57621693 |
| 1 | 5.56529595 | 2.03477983 | -2.36528144 |
| 1 | 4.31463678 | 2.48477618 | -3.55589923 |
| 1 | 1.62206751 | 0.19521778 | -2.25849402 |
| 1 | 2.02581020 | 1.43270787 | -3.47073054 |
| 1 | 2.80483076 | -0.16500108 | -3.54189907 |
| 1 | 5.07440587 | 0.31014129 | -0.58260934 |
| 1 | 3.48572989 | -0.49380986 | -0.50059888 |
| 1 | 4.58197368 | -0.81211812 | -1.87257774 |
| 1 | -1.20803983 | 1.47283227 | 3.41168409 |

| | | | |
|---|---|---|---|
| 8 | -0.71718956 | -2.15402597 | 3.51161205 |
| 6 | -0.69257569 | -3.54052062 | 3.90226481 |
| 1 | -1.62990609 | -3.69885210 | 4.44509580 |
| 6 | 1.76613349 | -2.10850124 | 3.14124913 |
| 6 | 2.20040644 | -3.46339034 | 2.53789797 |
| 6 | 2.40327490 | -3.55929527 | 1.01301584 |
| 6 | 0.36829266 | -1.52266077 | 3.03368260 |
| 6 | 1.15056853 | -3.80902408 | 0.14988465 |
| 6 | 0.46319555 | -2.53303991 | -0.32704516 |
| 1 | 2.42085337 | -1.32065661 | 2.74666229 |
| 1 | 1.54549141 | -4.28245970 | 2.86357999 |
| 1 | 3.10202300 | -4.39549331 | 0.85136010 |
| 1 | 0.42639344 | -4.42992635 | 0.70548806 |
| 1 | 1.96030323 | -2.14857229 | 4.23124557 |
| 1 | 3.17530921 | -3.66996499 | 3.00933717 |
| 1 | 2.92356214 | -2.65667836 | 0.65257511 |
| 1 | 1.43112911 | -4.39048406 | -0.74382859 |
| 1 | -0.43035813 | -2.78739233 | -0.92684916 |
| 1 | 1.16153087 | -1.98432576 | -0.98808542 |
| 8 | 0.20030484 | -0.30017870 | 2.81039752 |
| 8 | 0.10839051 | -1.74441164 | 0.79824043 |
| 1 | -0.66874189 | -4.18390567 | 3.01384689 |
| 1 | 0.15636960 | -3.76087062 | 4.56223224 |
| 31 | -0.10718225 | 0.12545287 | 0.69683219 |

Electronic energy = -3523.843856 a.u.
DFT-D3(BJ) dispersion correction = -0.118084 a.u.
Thermal free energy = 0.601469 a.u.
Gibbs free energy = -3523.360470 a.u.
Number of imaginary frequencies = 0.

**Int2 eq CL isomer/conformer 4**

| Atomic N. | X | Y | Z |
|---|---|---|---|
| 7 | -1.49318604 | 1.92338223 | 0.82868619 |
| 8 | 0.00261654 | 0.67180605 | -1.35185654 |
| 8 | 0.55418389 | 0.05157151 | 1.77867026 |
| 6 | -2.87234681 | 2.15478278 | 0.33493913 |
| 6 | -0.22651950 | 1.84610454 | -1.97337864 |
| 6 | -0.13226472 | 1.92264191 | -3.39783479 |
| 6 | -0.37812300 | 3.16500865 | -3.99822417 |
| 6 | -0.70650024 | 4.30952456 | -3.26592493 |
| 6 | -0.78977237 | 4.22481483 | -1.88036077 |
| 6 | -0.54374020 | 3.01114916 | -1.22980437 |
| 6 | -0.55771449 | 2.95954695 | 0.27190965 |
| 1 | -0.89225535 | 5.25373894 | -3.77783666 |
| 6 | 0.23193040 | 0.68914702 | -4.24629064 |
| 6 | 0.81269111 | 1.00615301 | 2.68936209 |
| 6 | 2.06567955 | 1.00400094 | 3.37710140 |
| 6 | 2.28666677 | 2.01438142 | 4.32228199 |
| 6 | 1.34333667 | 3.00730531 | 4.60487696 |
| 6 | 0.12758065 | 2.99834611 | 3.93011087 |
| 6 | -0.15099055 | 2.00286388 | 2.98660650 |
| 6 | -1.50751148 | 1.97010893 | 2.33548941 |

| | | | |
|---|---|---|---|
| 1 | 1.56337055 | 3.77830316 | 5.34323888 |
| 6 | 3.13496671 | -0.06807087 | 3.08931872 |
| 6 | 0.27896568 | 1.01173830 | -5.75161777 |
| 6 | -0.82719375 | -0.42107083 | -4.04885008 |
| 6 | 1.63275427 | 0.16976234 | -3.84424755 |
| 6 | 4.38674523 | 0.11461291 | 3.96781344 |
| 6 | 3.59139630 | 0.02149959 | 1.61366789 |
| 6 | 2.56897791 | -1.47824705 | 3.38330342 |
| 1 | -3.19085581 | 3.17927066 | 0.59006733 |
| 1 | -2.90522703 | 2.03354875 | -0.75277140 |
| 1 | -0.84517924 | 3.94409660 | 0.67870525 |
| 1 | 0.44085013 | 2.72200227 | 0.66726661 |
| 1 | -0.31529536 | 3.24909706 | -5.08180601 |
| 1 | -1.03544519 | 5.10732931 | -1.28494640 |
| 1 | -2.08059932 | 1.08320209 | 2.64980434 |
| 1 | -2.07559313 | 2.86508225 | 2.64213394 |
| 1 | 3.23325087 | 2.03739854 | 4.85955397 |
| 1 | -0.62564147 | 3.76239884 | 4.13685129 |
| 1 | 0.54663960 | 0.09968666 | -6.30448455 |
| 1 | -0.69336977 | 1.35647164 | -6.13256790 |
| 1 | 1.03498796 | 1.77449604 | -5.98721176 |
| 1 | -0.89731055 | -0.72233455 | -2.99781145 |
| 1 | -1.81669770 | -0.07194100 | -4.37986170 |
| 1 | -0.56157231 | -1.30373037 | -4.65119254 |
| 1 | 2.39743328 | 0.93390368 | -4.04621779 |
| 1 | 1.67484631 | -0.08723799 | -2.78015794 |
| 1 | 1.88520753 | -0.72518310 | -4.43389457 |
| 1 | 5.11047845 | -0.67767019 | 3.72817675 |
| 1 | 4.15407524 | 0.03774869 | 5.03988986 |
| 1 | 4.88227216 | 1.07964480 | 3.78780868 |
| 1 | 2.75574193 | -0.13664808 | 0.92342803 |
| 1 | 4.03271114 | 1.00702518 | 1.40490814 |
| 1 | 4.35869628 | -0.74195384 | 1.41274603 |
| 1 | 2.28904380 | -1.56743483 | 4.44341399 |
| 1 | 1.68615328 | -1.68944830 | 2.76998824 |
| 1 | 3.33690244 | -2.23842660 | 3.17181441 |
| 1 | -3.54605259 | 1.43055928 | 0.80160662 |
| 8 | -2.33257529 | -2.43060278 | 2.04385834 |
| 6 | -2.34210550 | -3.83758533 | 2.35045676 |
| 1 | -2.32185755 | -3.88885595 | 3.44358362 |
| 6 | -3.14619994 | -2.81802465 | -0.30120287 |
| 6 | -2.52693202 | -4.13094001 | -0.83146329 |
| 6 | -1.23399183 | -4.05998269 | -1.66849106 |
| 6 | -2.53926989 | -1.96596704 | 0.80099343 |
| 6 | 0.09400575 | -3.95710269 | -0.89147477 |
| 6 | 0.53840262 | -2.51967039 | -0.62645957 |
| 1 | -3.27053238 | -2.12282629 | -1.14125662 |
| 1 | -2.39034777 | -4.86568942 | -0.02642817 |
| 1 | -1.20967622 | -4.98402240 | -2.26790031 |
| 1 | 0.00884161 | -4.50118552 | 0.06526451 |
| 1 | -4.16793712 | -3.05059738 | 0.05809606 |
| 1 | -3.31147374 | -4.55757568 | -1.47812194 |
| 1 | -1.30441887 | -3.23116189 | -2.39136892 |

| | | | |
|---|---|---|---|
| 1 | 0.89464410 | -4.45959744 | -1.45820944 |
| 1 | 1.42597252 | -2.51489128 | 0.03470425 |
| 1 | 0.83285880 | -2.05291266 | -1.58560769 |
| 8 | -2.56585532 | -0.70620940 | 0.72024921 |
| 8 | -0.53707947 | -1.80466907 | -0.05040380 |
| 1 | -1.44606136 | -4.32207257 | 1.94257463 |
| 1 | -3.25072228 | -4.32635808 | 1.97525778 |
| 31 | -0.63188351 | 0.05264083 | 0.31696054 |

Electronic energy = -3523.842630 a.u.
DFT-D3(BJ) dispersion correction = -0.117868 a.u.
Thermal free energy = 0.602928 a.u.
Gibbs free energy = -3523.357570 a.u.
Number of imaginary frequencies = 0.

**Int2 CL isomer/conformer 1**

| Atomic N. | X | Y | Z |
|---|---|---|---|
| 8 | -0.81292677 | 1.72512533 | -4.82145714 |
| 6 | -2.08056585 | 1.03833014 | -4.96545753 |
| 1 | -2.25602743 | 0.37163882 | -4.11176887 |
| 6 | 0.62245009 | 3.27006277 | -3.75996418 |
| 6 | 0.77315661 | 4.41977716 | -2.75652193 |
| 6 | 0.73044068 | 4.03185730 | -1.27012900 |
| 6 | -0.75106259 | 2.62767904 | -3.80342987 |
| 6 | 1.83202184 | 3.06732205 | -0.82033706 |
| 6 | 1.78401642 | 2.76575039 | 0.68133446 |
| 1 | 1.35068636 | 2.46589429 | -3.56926930 |
| 1 | -0.01575229 | 5.16252556 | -2.95121504 |
| 1 | 0.80767752 | 4.95722917 | -0.67396669 |
| 1 | 2.82680483 | 3.49239069 | -1.04605135 |
| 1 | 0.84578539 | 3.61970607 | -4.77998408 |
| 1 | 1.73179844 | 4.92093141 | -2.97104298 |
| 1 | -0.25108624 | 3.59806401 | -1.03710439 |
| 1 | 1.76231824 | 2.11517838 | -1.37180934 |
| 1 | 1.91874203 | 3.70631131 | 1.24465013 |
| 1 | 2.62910463 | 2.10479345 | 0.95171027 |
| 8 | -1.68948386 | 2.86555309 | -3.06096442 |
| 8 | 0.53885105 | 2.21927839 | 1.12109420 |
| 1 | -1.98822758 | 0.45739798 | -5.88802720 |
| 1 | -2.90162112 | 1.76211685 | -5.03925398 |
| 7 | -2.06568252 | 0.58706566 | 0.86385220 |
| 8 | 0.34390446 | -0.80267676 | 1.95741033 |
| 8 | 0.20591830 | -0.07226831 | -1.01721145 |
| 6 | -2.56813293 | 1.82973139 | 1.50569721 |
| 6 | -0.40221255 | -0.85259479 | 3.09344611 |
| 6 | 0.21858603 | -1.07425279 | 4.35601726 |
| 6 | -0.61785970 | -1.12283789 | 5.48111127 |
| 6 | -2.00474803 | -0.96676428 | 5.40391097 |
| 6 | -2.60025237 | -0.77041098 | 4.16224645 |
| 6 | -1.81383708 | -0.72512103 | 3.00624370 |
| 6 | -2.46794439 | -0.63416822 | 1.65312415 |
| 1 | -2.61025333 | -1.00600597 | 6.30936320 |
| 6 | 1.74364136 | -1.25536995 | 4.48620974 |

| | | | |
|---|---|---|---|
| 6 | -0.71349533 | -0.90378717 | -1.57176776 |
| 6 | -0.28878102 | -1.97100090 | -2.41693147 |
| 6 | -1.28606868 | -2.77738324 | -2.98412226 |
| 6 | -2.64894001 | -2.57812587 | -2.74679827 |
| 6 | -3.04939577 | -1.53176721 | -1.92344080 |
| 6 | -2.09812173 | -0.68417913 | -1.34251508 |
| 6 | -2.57170813 | 0.51439012 | -0.55852139 |
| 1 | -3.38641262 | -3.23668203 | -3.20540757 |
| 6 | 1.20369647 | -2.24071515 | -2.69513289 |
| 6 | 2.16969919 | -1.51827558 | 5.94296409 |
| 6 | 2.47367583 | 0.02545656 | 4.01807539 |
| 6 | 2.20761642 | -2.46787178 | 3.64428046 |
| 6 | 1.40430061 | -3.42778428 | -3.65644200 |
| 6 | 1.93333844 | -2.58940412 | -1.37635576 |
| 6 | 1.85942106 | -1.00385630 | -3.35344426 |
| 1 | -3.66994502 | 1.83416614 | 1.49560885 |
| 1 | -2.21125749 | 1.87460490 | 2.54001775 |
| 1 | -3.56580707 | -0.63177383 | 1.75649707 |
| 1 | -2.18581689 | -1.49780616 | 1.03346011 |
| 1 | -0.17348829 | -1.28303310 | 6.46178588 |
| 1 | -3.68461933 | -0.66926170 | 4.07654525 |
| 1 | -2.24585417 | 1.44689473 | -1.04697821 |
| 1 | -3.67387346 | 0.52103586 | -0.52647968 |
| 1 | -0.99300275 | -3.60168116 | -3.63148684 |
| 1 | -4.10993452 | -1.34998525 | -1.73604611 |
| 1 | 3.26023009 | -1.65260831 | 5.97693245 |
| 1 | 1.92008583 | -0.67782848 | 6.60676847 |
| 1 | 1.71120263 | -2.43147197 | 6.34917448 |
| 1 | 2.26115754 | 0.24990270 | 2.96739377 |
| 1 | 2.16885622 | 0.89047184 | 4.62444848 |
| 1 | 3.56087091 | -0.10376562 | 4.13138584 |
| 1 | 1.72726534 | -3.39133044 | 3.99933074 |
| 1 | 1.96821711 | -2.33723391 | 2.58341428 |
| 1 | 3.29647622 | -2.59430658 | 3.74415853 |
| 1 | 2.48102527 | -3.57154427 | -3.82443915 |
| 1 | 0.93769015 | -3.25049701 | -4.63637489 |
| 1 | 1.00714925 | -4.36694394 | -3.24513190 |
| 1 | 1.87454830 | -1.77180828 | -0.65022736 |
| 1 | 1.49789063 | -3.48888403 | -0.91803391 |
| 1 | 2.99501168 | -2.79388729 | -1.58210575 |
| 1 | 1.36591272 | -0.76064930 | -4.30564264 |
| 1 | 1.80314581 | -0.12768798 | -2.69883049 |
| 1 | 2.91891225 | -1.21388351 | -3.56461391 |
| 1 | -2.18535538 | 2.69820171 | 0.95886833 |
| 31 | -0.03374593 | 0.54434062 | 0.72370423 |

Electronic energy = -3523.875045 a.u.
DFT-D3(BJ) dispersion correction = -0.116024 a.u.
Thermal free energy = 0.599093 a.u.
Gibbs free energy = -3523.391977 a.u.
Number of imaginary frequencies = 0.

**Int2_CL isomer/conformer 2**

| Atomic N. | X | Y | Z |
|---|---|---|---|
| 8 | -4.70114995 | -1.19027616 | 2.28291040 |
| 6 | -5.20521189 | -2.52748355 | 2.51069157 |
| 1 | -4.39098623 | -3.26082639 | 2.45043996 |
| 6 | -3.35129865 | 0.65886304 | 2.90963196 |
| 6 | -2.05297734 | 1.06695547 | 3.61082915 |
| 6 | -0.79860697 | 0.44873400 | 2.98344600 |
| 6 | -3.76328416 | -0.77546380 | 3.18599898 |
| 6 | 0.48936088 | 0.85588269 | 3.70467306 |
| 6 | 1.76914833 | 0.31752993 | 3.06203869 |
| 1 | -3.28975295 | 0.80550185 | 1.82069066 |
| 1 | -2.11514812 | 0.77910213 | 4.67158797 |
| 1 | -0.88652026 | -0.64965277 | 2.98982402 |
| 1 | 0.45278552 | 0.49232149 | 4.74657368 |
| 1 | -4.18919082 | 1.29012475 | 3.25086541 |
| 1 | -1.96899782 | 2.16505664 | 3.58079976 |
| 1 | -0.74766481 | 0.76075631 | 1.92599903 |
| 1 | 0.56496449 | 1.95511653 | 3.75293755 |
| 1 | 1.69756544 | -0.78120865 | 2.95357742 |
| 1 | 2.62291230 | 0.52618783 | 3.72972755 |
| 8 | -3.35924647 | -1.47439380 | 4.09634668 |
| 8 | 2.06282538 | 0.95122321 | 1.80834625 |
| 1 | -5.94009716 | -2.69895375 | 1.71816600 |
| 1 | -5.67560891 | -2.59833272 | 3.49965050 |
| 7 | 3.28126564 | 0.03819505 | -0.95523408 |
| 8 | 0.55093262 | 1.23105936 | -0.90916661 |
| 8 | 1.08110927 | -1.57672690 | 0.32903828 |
| 6 | 4.40526531 | 0.87288002 | -0.46110985 |
| 6 | 1.12041184 | 2.15234961 | -1.73062115 |
| 6 | 0.50760357 | 3.42624418 | -1.91083779 |
| 6 | 1.13919094 | 4.32402235 | -2.78400062 |
| 6 | 2.32071198 | 4.01743015 | -3.46474499 |
| 6 | 2.90070047 | 2.76542015 | -3.29097121 |
| 6 | 2.30488866 | 1.82632724 | -2.44194274 |
| 6 | 2.87350739 | 0.43563314 | -2.35069620 |
| 1 | 2.77631725 | 4.75269719 | -4.12788048 |
| 6 | -0.79071009 | 3.81082092 | -1.17444465 |
| 6 | 1.34612529 | -2.43560302 | -0.68872530 |
| 6 | 0.38257621 | -3.41761987 | -1.06107775 |
| 6 | 0.72272129 | -4.28494569 | -2.10889860 |
| 6 | 1.94561386 | -4.21868340 | -2.78289861 |
| 6 | 2.88140359 | -3.26396927 | -2.40220245 |
| 6 | 2.59846799 | -2.37938639 | -1.35449479 |
| 6 | 3.66612253 | -1.42170905 | -0.89232991 |
| 1 | 2.16025688 | -4.91206311 | -3.59589912 |
| 6 | -0.97683899 | -3.52826658 | -0.34317617 |
| 6 | -1.28510090 | 5.21452401 | -1.57214438 |
| 6 | -0.54325886 | 3.82520234 | 0.35241400 |
| 6 | -1.92029059 | 2.81234879 | -1.52307632 |
| 6 | -1.83440048 | -4.67607243 | -0.90898236 |
| 6 | -1.78427816 | -2.22118328 | -0.52396723 |
| 6 | -0.75691655 | -3.81705638 | 1.16079598 |
| 1 | 5.30740924 | 0.68406188 | -1.06520276 |

| | | | |
|---|---|---|---|
| 1 | 4.13301716 | 1.93114794 | -0.53654582 |
| 1 | 3.75229019 | 0.33849278 | -3.01007349 |
| 1 | 2.12945091 | -0.30975628 | -2.66782594 |
| 1 | 0.69823671 | 5.30711661 | -2.93835700 |
| 1 | 3.81296219 | 2.49709606 | -3.82872773 |
| 1 | 3.92860911 | -1.61000921 | 0.16134589 |
| 1 | 4.57763735 | -1.56115148 | -1.49701910 |
| 1 | 0.00542610 | -5.04203359 | -2.42029565 |
| 1 | 3.84825604 | -3.20330603 | -2.90684910 |
| 1 | -2.21540569 | 5.43196064 | -1.02827919 |
| 1 | -0.56088794 | 6.00005030 | -1.31217697 |
| 1 | -1.50584326 | 5.28554219 | -2.64707034 |
| 1 | -0.19806525 | 2.85237020 | 0.71811833 |
| 1 | 0.21416588 | 4.57771059 | 0.61545248 |
| 1 | -1.47479954 | 4.08468101 | 0.87871593 |
| 1 | -2.12822256 | 2.82621386 | -2.60298555 |
| 1 | -1.65966520 | 1.78889756 | -1.23345956 |
| 1 | -2.84428583 | 3.10091149 | -0.99898340 |
| 1 | -2.78433097 | -4.71500412 | -0.35705853 |
| 1 | -1.34550751 | -5.65427035 | -0.79401280 |
| 1 | -2.07652536 | -4.52911178 | -1.97153949 |
| 1 | -1.26142142 | -1.35855424 | -0.09855697 |
| 1 | -1.96842858 | -2.02206116 | -1.58957235 |
| 1 | -2.76044214 | -2.31259174 | -0.02422124 |
| 1 | -0.22924337 | -4.77228294 | 1.29755817 |
| 1 | -0.17385453 | -3.02549285 | 1.64307752 |
| 1 | -1.72760404 | -3.89234467 | 1.67370259 |
| 1 | 4.59917995 | 0.63125280 | 0.59004437 |
| 31 | 1.61067457 | 0.20183109 | 0.22232055 |

Electronic energy = -3523.874557 a.u.
DFT-D3(BJ) dispersion correction = -0.113649 a.u.
Thermal free energy = 0.596571 a.u.
Gibbs free energy = -3523.391635 a.u.
Number of imaginary frequencies = 0.

**Int2 CL isomer/conformer 3**

| Atomic N. | X | Y | Z |
|---|---|---|---|
| 8 | -3.30031508 | 1.90370063 | -2.94477648 |
| 6 | -3.86986551 | 0.59751679 | -3.20568832 |
| 1 | -3.90440095 | 0.40123012 | -4.28464307 |
| 6 | -1.55591584 | 3.50017342 | -3.18296783 |
| 6 | -0.03214716 | 3.59210324 | -3.04296948 |
| 6 | 0.49936445 | 2.95305174 | -1.75497930 |
| 6 | -2.04931833 | 2.09140530 | -3.45832487 |
| 6 | 2.01051689 | 3.12916004 | -1.57967442 |
| 6 | 2.56560012 | 2.52718273 | -0.28747036 |
| 1 | -1.89375723 | 4.11323053 | -4.03583070 |
| 1 | 0.25440503 | 4.65605321 | -3.06870388 |
| 1 | -0.01974941 | 3.39586239 | -0.88688983 |
| 1 | 2.25859499 | 4.20586441 | -1.58324440 |
| 1 | -2.06829124 | 3.89417746 | -2.29368376 |
| 1 | 0.43625543 | 3.11298779 | -3.91605743 |

| | | | |
|---|---|---|---|
| 1 | 0.25454442 | 1.87900279 | -1.75828965 |
| 1 | 2.54392637 | 2.67610062 | -2.43135128 |
| 1 | 2.00976278 | 2.93685367 | 0.57748428 |
| 1 | 3.62142226 | 2.83173248 | -0.17298323 |
| 8 | -1.44488417 | 1.23700018 | -4.07937136 |
| 8 | 2.53531214 | 1.09415383 | -0.30584935 |
| 1 | -4.87889099 | 0.63257399 | -2.78363890 |
| 1 | -3.27294641 | -0.18252400 | -2.71679843 |
| 7 | 2.36213465 | -0.86816375 | 2.20978282 |
| 8 | 0.60287634 | -1.31953481 | -0.14925397 |
| 8 | 0.19217826 | 1.16111714 | 1.70039883 |
| 6 | 3.83235433 | -0.89184356 | 2.00706157 |
| 6 | 1.27823996 | -2.49877282 | -0.21717576 |
| 6 | 1.30153818 | -3.24361495 | -1.43132256 |
| 6 | 1.99990670 | -4.46020321 | -1.42074029 |
| 6 | 2.65814750 | -4.94967092 | -0.28879127 |
| 6 | 2.61924960 | -4.21637294 | 0.89236137 |
| 6 | 1.92505908 | -3.00243428 | 0.94113396 |
| 6 | 1.79329624 | -2.26449571 | 2.24607363 |
| 1 | 3.19229382 | -5.89871018 | -0.33489058 |
| 6 | 0.60106845 | -2.73513148 | -2.70598963 |
| 6 | -0.29951275 | 0.68386522 | 2.87342593 |
| 6 | -1.66775860 | 0.89265910 | 3.21262973 |
| 6 | -2.10722568 | 0.38580152 | 4.44404890 |
| 6 | -1.26723199 | -0.29944298 | 5.32656176 |
| 6 | 0.06857725 | -0.48470666 | 4.98924823 |
| 6 | 0.56333866 | 0.01043892 | 3.77688955 |
| 6 | 2.03459418 | -0.11199752 | 3.47543176 |
| 1 | -1.65893984 | -0.68059567 | 6.26948513 |
| 6 | -2.62843145 | 1.64609601 | 2.27166665 |
| 6 | 0.72676707 | -3.73549668 | -3.87069410 |
| 6 | 1.24408400 | -1.40635718 | -3.16866588 |
| 6 | -0.90996529 | -2.53139757 | -2.44117980 |
| 6 | -4.04411242 | 1.76277737 | 2.86766673 |
| 6 | -2.75871454 | 0.89715694 | 0.92423822 |
| 6 | -2.11129459 | 3.08451567 | 2.03146090 |
| 1 | 4.32384191 | -1.36537250 | 2.87245776 |
| 1 | 4.06573429 | -1.46075182 | 1.10073335 |
| 1 | 2.29612046 | -2.82221738 | 3.05402436 |
| 1 | 0.73505872 | -2.14813857 | 2.52183213 |
| 1 | 2.04067422 | -5.05101053 | -2.33404508 |
| 1 | 3.11205125 | -4.58821718 | 1.79367795 |
| 1 | 2.48866391 | 0.88408284 | 3.34859296 |
| 1 | 2.54642546 | -0.61124971 | 4.31503337 |
| 1 | -3.14863674 | 0.52315795 | 4.72909092 |
| 1 | 0.74521461 | -1.00577233 | 5.67026730 |
| 1 | 0.20351853 | -3.32687242 | -4.74671082 |
| 1 | 1.77349758 | -3.90491367 | -4.16224895 |
| 1 | 0.26883509 | -4.70701075 | -3.63331633 |
| 1 | 1.19023242 | -0.63423880 | -2.39435790 |
| 1 | 2.30196561 | -1.55973475 | -3.42753334 |
| 1 | 0.72155373 | -1.02804227 | -4.05920281 |
| 1 | -1.38550330 | -3.48296428 | -2.16026151 |

| | | | |
|---|---|---|---|
| 1 | -1.08285449 | -1.80488039 | -1.64012324 |
| 1 | -1.39533133 | -2.16295390 | -3.35723218 |
| 1 | -4.68229549 | 2.31476810 | 2.16315531 |
| 1 | -4.04889255 | 2.31340506 | 3.81947205 |
| 1 | -4.50778758 | 0.77919279 | 3.03122490 |
| 1 | -1.79601816 | 0.81626059 | 0.40906426 |
| 1 | -3.15317587 | -0.11730303 | 1.08072432 |
| 1 | -3.45596752 | 1.43680094 | 0.26560621 |
| 1 | -2.06699405 | 3.64276839 | 2.97806146 |
| 1 | -1.11251916 | 3.08228715 | 1.58170225 |
| 1 | -2.79786816 | 3.61769511 | 1.35637209 |
| 1 | 4.19832208 | 0.13399505 | 1.88538240 |
| 31 | 1.38039483 | 0.13315195 | 0.70439705 |

Electronic energy = -3523.874781 a.u.
DFT-D3(BJ) dispersion correction = -0.113595 a.u.
Thermal free energy = 0.598229 a.u.
Gibbs free energy = -3523.390147 a.u.
Number of imaginary frequencies = 0.

**Int2 CL isomer/conformer 4**

| Atomic N. | X | Y | Z |
|---|---|---|---|
| 8 | -6.16777451 | 0.88333685 | 0.22826102 |
| 6 | -5.98414909 | 1.89836223 | 1.24681670 |
| 1 | -6.87754126 | 1.84050449 | 1.87547545 |
| 6 | -5.42150864 | -0.27857979 | -1.71526925 |
| 6 | -4.23408504 | -1.24293429 | -1.87054363 |
| 6 | -4.05555188 | -2.17700137 | -0.66661229 |
| 6 | -5.15512034 | 0.78996222 | -0.67478360 |
| 6 | -2.80680275 | -3.07288701 | -0.75382551 |
| 6 | -1.52942780 | -2.44355214 | -0.19890869 |
| 1 | -6.34584743 | -0.81586038 | -1.46252168 |
| 1 | -3.31118447 | -0.66924038 | -2.02785443 |
| 1 | -4.00103322 | -1.58254441 | 0.26210968 |
| 1 | -2.63350468 | -3.38025843 | -1.79866798 |
| 1 | -5.59429516 | 0.25116929 | -2.66617122 |
| 1 | -4.39249161 | -1.84786523 | -2.77743343 |
| 1 | -4.95614341 | -2.80689694 | -0.57382702 |
| 1 | -2.97538635 | -3.99903049 | -0.17919806 |
| 1 | -0.72102139 | -3.19969468 | -0.22365742 |
| 1 | -1.69860083 | -2.17724395 | 0.86296091 |
| 8 | -4.16229565 | 1.50069755 | -0.63780868 |
| 8 | -1.15259646 | -1.28903019 | -0.96075255 |
| 1 | -5.89483032 | 2.89034196 | 0.78688179 |
| 1 | -5.08120795 | 1.68992882 | 1.83388859 |
| 7 | -0.17583508 | 1.74034512 | -0.98761025 |
| 8 | 1.85602866 | -0.42854747 | -0.68950782 |
| 8 | -0.15976184 | 0.14151670 | 1.57497782 |
| 6 | -1.03386588 | 1.79431390 | -2.20099610 |
| 6 | 2.32980855 | 0.07721550 | -1.85842785 |
| 6 | 3.18748976 | -0.70218022 | -2.68691063 |
| 6 | 3.64234640 | -0.11000233 | -3.87442152 |
| 6 | 3.29202134 | 1.18749491 | -4.25893784 |

| | | | |
|---|---|---|---|
| 6 | 2.47019647 | 1.94501669 | -3.43074052 |
| 6 | 1.99761423 | 1.40675253 | -2.22929083 |
| 6 | 1.20884549 | 2.26438514 | -1.27657793 |
| 1 | 3.66737473 | 1.60003539 | -5.19542225 |
| 6 | 3.60236708 | -2.13465005 | -2.29817738 |
| 6 | 0.19653396 | 1.33125946 | 2.12319401 |
| 6 | 0.80917233 | 1.36690006 | 3.40975327 |
| 6 | 1.14545280 | 2.62609570 | 3.92692340 |
| 6 | 0.90555607 | 3.81756792 | 3.23664673 |
| 6 | 0.29596452 | 3.76930261 | 1.98802221 |
| 6 | -0.07429573 | 2.54015070 | 1.42910196 |
| 6 | -0.83463104 | 2.52634320 | 0.12700680 |
| 1 | 1.19337803 | 4.77228759 | 3.67670868 |
| 6 | 1.09210735 | 0.07677861 | 4.20488295 |
| 6 | 4.56178685 | -2.75629260 | -3.33046802 |
| 6 | 2.35569435 | -3.04713679 | -2.22080123 |
| 6 | 4.33570395 | -2.12270977 | -0.93569799 |
| 6 | 1.73197659 | 0.37354946 | 5.57440731 |
| 6 | 2.07582400 | -0.82784956 | 3.42584533 |
| 6 | -0.23078850 | -0.68027556 | 4.47125553 |
| 1 | -1.11466475 | 2.83713926 | -2.54806197 |
| 1 | -0.58695788 | 1.18358034 | -2.99282790 |
| 1 | 1.11004893 | 3.28829920 | -1.67376919 |
| 1 | 1.71593206 | 2.32489301 | -0.30241257 |
| 1 | 4.29313030 | -0.68224820 | -4.53317101 |
| 1 | 2.20292733 | 2.96939575 | -3.70027558 |
| 1 | -1.83269921 | 2.07839304 | 0.24994391 |
| 1 | -0.97384645 | 3.55984844 | -0.23060902 |
| 1 | 1.62092797 | 2.68527360 | 4.90425721 |
| 1 | 0.08694882 | 4.68964439 | 1.43797150 |
| 1 | 4.83502692 | -3.76835110 | -2.99939716 |
| 1 | 4.09986188 | -2.84776326 | -4.32424987 |
| 1 | 5.49202690 | -2.17848344 | -3.43037032 |
| 1 | 1.64137607 | -2.69151498 | -1.47059798 |
| 1 | 1.84383158 | -3.08891172 | -3.19311886 |
| 1 | 2.65972772 | -4.07046380 | -1.95257855 |
| 1 | 5.24761503 | -1.51056131 | -0.99328082 |
| 1 | 3.69893113 | -1.72366002 | -0.13881869 |
| 1 | 4.63376591 | -3.14725085 | -0.66535207 |
| 1 | 1.90038808 | -0.57586702 | 6.10237146 |
| 1 | 1.08340687 | 0.99446633 | 6.20934420 |
| 1 | 2.70644284 | 0.87363353 | 5.47703812 |
| 1 | 1.66935180 | -1.12844681 | 2.45466152 |
| 1 | 3.02932072 | -0.30771791 | 3.25495304 |
| 1 | 2.28569185 | -1.73715124 | 4.00942760 |
| 1 | -0.91485224 | -0.05752049 | 5.06592981 |
| 1 | -0.73255488 | -0.95548519 | 3.53718066 |
| 1 | -0.02622165 | -1.59837041 | 5.04288629 |
| 1 | -2.03185925 | 1.41655141 | -1.95113896 |
| 31 | 0.06531040 | -0.14070112 | -0.24923131 |

Electronic energy = -3523.875432 a.u.
DFT-D3(BJ) dispersion correction = -0.111661 a.u.
Thermal free energy = 0.597131 a.u.

Gibbs free energy = -3523.389961 a.u.
Number of imaginary frequencies = 0.

**Int2 CL isomer/conformer 5**

| Atomic N. | X | Y | Z |
|---|---|---|---|
| 8 | -6.08991760 | 0.81125234 | 0.27286626 |
| 6 | -5.88139030 | 1.82758048 | 1.28539856 |
| 1 | -6.73620464 | 1.73943552 | 1.96231104 |
| 6 | -5.41047894 | -0.32654267 | -1.70961251 |
| 6 | -4.22489438 | -1.29357043 | -1.87022748 |
| 6 | -4.02610533 | -2.21103745 | -0.65675888 |
| 6 | -5.12544542 | 0.75113294 | -0.68400956 |
| 6 | -2.77251024 | -3.09968814 | -0.74900484 |
| 6 | -1.49529087 | -2.45670238 | -0.20968271 |
| 1 | -6.33020821 | -0.86176780 | -1.43681336 |
| 1 | -3.30509897 | -0.72147076 | -2.05045402 |
| 1 | -3.96343134 | -1.60472098 | 0.26370096 |
| 1 | -2.60627567 | -3.41281063 | -1.79327649 |
| 1 | -5.59454407 | 0.19258646 | -2.66383296 |
| 1 | -4.39775263 | -1.91085207 | -2.76610762 |
| 1 | -4.92164756 | -2.84479279 | -0.54438817 |
| 1 | -2.92920414 | -4.02310636 | -0.16672781 |
| 1 | -0.67984040 | -3.20502882 | -0.24021722 |
| 1 | -1.65638364 | -2.18853387 | 0.85301580 |
| 8 | -4.15388458 | 1.49162490 | -0.69816734 |
| 8 | -1.13756186 | -1.30112364 | -0.97924046 |
| 1 | -5.84863192 | 2.82354892 | 0.82665810 |
| 1 | -4.94116823 | 1.64496046 | 1.82027802 |
| 7 | -0.17364796 | 1.73414022 | -1.04188484 |
| 8 | 1.86413642 | -0.42013011 | -0.67776417 |
| 8 | -0.19034091 | 0.17439864 | 1.54413823 |
| 6 | -1.01718782 | 1.76387206 | -2.26639829 |
| 6 | 2.35042262 | 0.06504246 | -1.85034016 |
| 6 | 3.22194312 | -0.72582672 | -2.65303764 |
| 6 | 3.68782979 | -0.15456317 | -3.84650156 |
| 6 | 3.33548683 | 1.13329872 | -4.26047184 |
| 6 | 2.50041822 | 1.90276016 | -3.45687592 |
| 6 | 2.01636744 | 1.38552277 | -2.25072728 |
| 6 | 1.21299472 | 2.25746242 | -1.32340405 |
| 1 | 3.71968938 | 1.52929836 | -5.20049951 |
| 6 | 3.64039087 | -2.14776896 | -2.23087342 |
| 6 | 0.15099575 | 1.37315551 | 2.08183953 |
| 6 | 0.73344768 | 1.42876763 | 3.38167679 |
| 6 | 1.05395062 | 2.69648248 | 3.88818145 |
| 6 | 0.82596538 | 3.87723124 | 3.17578622 |
| 6 | 0.24423274 | 3.80934780 | 1.91480422 |
| 6 | -0.10970370 | 2.57116780 | 1.36543245 |
| 6 | -0.84835266 | 2.53673783 | 0.05099109 |
| 1 | 1.10096344 | 4.83903177 | 3.60857569 |
| 6 | 1.00603007 | 0.15113113 | 4.20009533 |
| 6 | 4.61484124 | -2.78456157 | -3.23956674 |
| 6 | 2.39826739 | -3.06595922 | -2.14871888 |

| | | | |
|---|---|---|---|
| 6 | 4.35807739 | -2.10387851 | -0.86073387 |
| 6 | 1.60949830 | 0.47047286 | 5.58102434 |
| 6 | 2.01660371 | -0.75319974 | 3.45573297 |
| 6 | -0.31502983 | -0.61697484 | 4.44328961 |
| 1 | -1.09923538 | 2.80038047 | -2.63152793 |
| 1 | -0.55758157 | 1.14253179 | -3.04253626 |
| 1 | 1.11590195 | 3.27376513 | -1.74012911 |
| 1 | 1.70758625 | 2.33699699 | -0.34416099 |
| 1 | 4.34929972 | -0.73612122 | -4.48617424 |
| 1 | 2.23144071 | 2.92032580 | -3.74949893 |
| 1 | -1.84819670 | 2.09047889 | 0.16571669 |
| 1 | -0.98320937 | 3.56477599 | -0.32375775 |
| 1 | 1.50678491 | 2.77142704 | 4.87513513 |
| 1 | 0.04358009 | 4.72117036 | 1.34771133 |
| 1 | 4.89019103 | -3.78800668 | -2.88487924 |
| 1 | 4.16451460 | -2.89898056 | -4.23629466 |
| 1 | 5.54281754 | -2.20340477 | -3.34096535 |
| 1 | 1.67435673 | -2.69976621 | -1.41298068 |
| 1 | 1.89683543 | -3.12990828 | -3.12524489 |
| 1 | 2.70526819 | -4.08197843 | -1.85710828 |
| 1 | 5.26695653 | -1.48744527 | -0.92031639 |
| 1 | 3.70985605 | -1.69273463 | -0.07934247 |
| 1 | 4.65895895 | -3.12093574 | -0.56633093 |
| 1 | 1.77143514 | -0.47063794 | 6.12556928 |
| 1 | 0.94042357 | 1.09468679 | 6.19093087 |
| 1 | 2.58240249 | 0.97667651 | 5.50155492 |
| 1 | 1.63685980 | -1.06783462 | 2.47810387 |
| 1 | 2.96928986 | -0.22614341 | 3.30250702 |
| 1 | 2.22007579 | -1.65427750 | 4.05415510 |
| 1 | -1.02235974 | 0.00208289 | 5.01457103 |
| 1 | -0.79010433 | -0.90844441 | 3.50033130 |
| 1 | -0.11330388 | -1.52643454 | 5.02953276 |
| 1 | -2.01626014 | 1.38497610 | -2.02266965 |
| 31 | 0.06499649 | -0.13405280 | -0.27137861 |

Electronic energy = -3523.875408 a.u.
DFT-D3(BJ) dispersion correction = -0.111754 a.u.
Thermal free energy = 0.597917 a.u.
Gibbs free energy = -3523.389244 a.u.
Number of imaginary frequencies = 0.

**Int2 CL isomer/conformer 6**

| Atomic N. | X | Y | Z |
|---|---|---|---|
| 8 | 0.20127091 | -3.60624119 | -5.42115140 |
| 6 | 1.11055263 | -4.49008545 | -4.71948370 |
| 1 | 1.67739105 | -3.93124389 | -3.96463506 |
| 6 | -1.66846460 | -2.13584750 | -5.45300507 |
| 6 | -2.05325994 | -0.83862582 | -4.72903912 |
| 6 | -0.88713484 | 0.15137193 | -4.62395486 |
| 6 | -0.73250649 | -3.00555453 | -4.63559196 |
| 6 | -1.20738272 | 1.42074028 | -3.82165072 |
| 6 | -1.37908309 | 1.18256182 | -2.31935988 |
| 1 | -1.21202825 | -1.93275387 | -6.43165434 |

| | | | |
|---|---|---|---|
| 1 | -2.42943326 | -1.09907389 | -3.72878745 |
| 1 | -0.02218010 | -0.33960785 | -4.15103355 |
| 1 | -2.12636549 | 1.89785506 | -4.20529182 |
| 1 | -2.56885166 | -2.74524319 | -5.63972465 |
| 1 | -2.89124528 | -0.37011577 | -5.26984232 |
| 1 | -0.57120952 | 0.43783410 | -5.64078636 |
| 1 | -0.39007036 | 2.14649653 | -3.95636267 |
| 1 | -2.24997474 | 0.52122559 | -2.14084251 |
| 1 | -1.60231199 | 2.14591137 | -1.82744942 |
| 8 | -0.80260011 | -3.18114470 | -3.42972172 |
| 8 | -0.17986100 | 0.60181698 | -1.80711640 |
| 1 | 1.77800354 | -4.88736477 | -5.48981231 |
| 1 | 0.55591114 | -5.29986270 | -4.22919918 |
| 7 | -0.77452494 | -1.59600558 | 0.51011563 |
| 8 | -0.56062266 | 1.29657192 | 1.27794380 |
| 8 | 1.88094653 | -0.22685539 | 0.14613852 |
| 6 | -1.89796269 | -2.01956947 | -0.36499692 |
| 6 | -1.77472217 | 1.07289183 | 1.84358668 |
| 6 | -2.60339247 | 2.17281335 | 2.21261367 |
| 6 | -3.86003352 | 1.87355811 | 2.75921139 |
| 6 | -4.30515724 | 0.56525956 | 2.96888512 |
| 6 | -3.46328682 | -0.49719903 | 2.65787066 |
| 6 | -2.19648996 | -0.25604154 | 2.11472851 |
| 6 | -1.23442186 | -1.40009789 | 1.92992131 |
| 1 | -5.29411009 | 0.38490901 | 3.39014384 |
| 6 | -2.13072405 | 3.63226196 | 2.05484086 |
| 6 | 2.29143936 | -1.13354306 | 1.06577851 |
| 6 | 3.49772694 | -0.91179977 | 1.79280286 |
| 6 | 3.87773561 | -1.89191427 | 2.72043704 |
| 6 | 3.12943521 | -3.05031541 | 2.95019872 |
| 6 | 1.96149327 | -3.26029015 | 2.22605224 |
| 6 | 1.54180632 | -2.32058206 | 1.27665317 |
| 6 | 0.33334198 | -2.62259413 | 0.42870550 |
| 1 | 3.46290284 | -3.77945355 | 3.68853177 |
| 6 | 4.35174491 | 0.35232384 | 1.57184838 |
| 6 | -3.18251278 | 4.63516543 | 2.56554461 |
| 6 | -1.86142929 | 3.96607283 | 0.56975064 |
| 6 | -0.84143173 | 3.85229423 | 2.88224204 |
| 6 | 5.61875978 | 0.34977411 | 2.44798396 |
| 6 | 3.53957124 | 1.61588047 | 1.94250641 |
| 6 | 4.81232109 | 0.43185518 | 0.09666966 |
| 1 | -2.29173275 | -2.98897137 | -0.01814318 |
| 1 | -2.69931705 | -1.27374483 | -0.31552739 |
| 1 | -1.69269416 | -2.34147775 | 2.27720241 |
| 1 | -0.32188876 | -1.23196755 | 2.52091289 |
| 1 | -4.52304822 | 2.69147526 | 3.03517452 |
| 1 | -3.77288111 | -1.52618255 | 2.85468474 |
| 1 | 0.60010244 | -2.67723147 | -0.63856025 |
| 1 | -0.08908567 | -3.59879250 | 0.71921969 |
| 1 | 4.79069055 | -1.74808838 | 3.29541546 |
| 1 | 1.36885432 | -4.16493209 | 2.38014677 |
| 1 | -2.78885140 | 5.65488682 | 2.44921297 |
| 1 | -4.12219778 | 4.57702460 | 1.99703869 |

| | | | |
|---|---|---|---|
| 1 | -3.41051421 | 4.48864337 | 3.63107820 |
| 1 | -1.05697323 | 3.34615367 | 0.15976902 |
| 1 | -2.76819136 | 3.81493822 | -0.03438861 |
| 1 | -1.56416321 | 5.02139364 | 0.47386172 |
| 1 | -1.03063892 | 3.66614573 | 3.94939886 |
| 1 | -0.03308847 | 3.19086568 | 2.55124130 |
| 1 | -0.50408339 | 4.89452571 | 2.77551495 |
| 1 | 6.19500667 | 1.26329396 | 2.24350597 |
| 1 | 6.27101864 | -0.50828009 | 2.22973274 |
| 1 | 5.38140543 | 0.34327186 | 3.52173674 |
| 1 | 2.64479477 | 1.72054357 | 1.32010260 |
| 1 | 3.22720115 | 1.58156631 | 2.99630060 |
| 1 | 4.16332639 | 2.51187523 | 1.80290580 |
| 1 | 5.42905158 | -0.44127343 | -0.16261013 |
| 1 | 3.96020177 | 0.47358575 | -0.59014000 |
| 1 | 5.42554798 | 1.33374599 | -0.05190150 |
| 1 | -1.54106541 | -2.12599458 | -1.39645488 |
| 31 | 0.08564474 | 0.17458211 | -0.06764285 |

Electronic energy = -3523.875421 a.u.
DFT-D3(BJ) dispersion correction = -0.111378 a.u.
Thermal free energy = 0.597776 a.u.
Gibbs free energy = -3523.389023 a.u.
Number of imaginary frequencies = 0.

**Int2 CL isomer/conformer 7**

| Atomic N. | X | Y | Z |
|---|---|---|---|
| 8 | -1.45398638 | -1.93784156 | 5.40456257 |
| 6 | -0.07674218 | -1.59153840 | 5.69055958 |
| 1 | 0.50316992 | -1.53470003 | 4.76055066 |
| 6 | -3.54269315 | -1.49970797 | 4.36712059 |
| 6 | -3.67018136 | -2.78334346 | 3.51675565 |
| 6 | -3.18597106 | -2.65944764 | 2.06251493 |
| 6 | -2.12615767 | -1.03960457 | 4.63596454 |
| 6 | -1.66944961 | -2.77336362 | 1.86588439 |
| 6 | -1.23961780 | -2.58543588 | 0.40951678 |
| 1 | -4.06272948 | -0.66658470 | 3.87631348 |
| 1 | -4.73839303 | -3.05037238 | 3.51484262 |
| 1 | -3.66591443 | -3.45512730 | 1.46831264 |
| 1 | -1.32766600 | -3.76791429 | 2.20318788 |
| 1 | -4.02280735 | -1.67484080 | 5.34249278 |
| 1 | -3.14621669 | -3.60869456 | 4.02379035 |
| 1 | -3.53683202 | -1.70761407 | 1.63267881 |
| 1 | -1.13156359 | -2.02983781 | 2.47474275 |
| 1 | -0.16190968 | -2.81033968 | 0.32140251 |
| 1 | -1.77769484 | -3.30893749 | -0.23048467 |
| 8 | -1.62811868 | 0.00188862 | 4.23494234 |
| 8 | -1.55312855 | -1.27426180 | -0.07655619 |
| 1 | 0.29683041 | -2.39766649 | 6.32874886 |
| 1 | -0.02395009 | -0.62641204 | 6.20911883 |
| 7 | -0.58101301 | 1.71081321 | 0.70282206 |
| 8 | -0.10245766 | 0.63145951 | -2.03255318 |
| 8 | 1.39086667 | -0.55751723 | 0.41916488 |

| | | | |
|---|---|---|---|
| 6 | -1.97576134 | 1.83559627 | 1.20192220 |
| 6 | -0.88621407 | 1.67664612 | -2.40648318 |
| 6 | -1.52538842 | 1.67829443 | -3.67948740 |
| 6 | -2.30927321 | 2.79659837 | -4.00014295 |
| 6 | -2.47747938 | 3.88039533 | -3.13334423 |
| 6 | -1.83404160 | 3.87297210 | -1.89999282 |
| 6 | -1.03060808 | 2.78760522 | -1.53519235 |
| 6 | -0.24361965 | 2.82720309 | -0.25321928 |
| 1 | -3.10234912 | 4.72359317 | -3.42774692 |
| 6 | -1.36725445 | 0.50323506 | -4.66390870 |
| 6 | 2.28270362 | 0.38341995 | 0.82534651 |
| 6 | 3.67549501 | 0.19038137 | 0.59284678 |
| 6 | 4.54708981 | 1.19088435 | 1.04633505 |
| 6 | 4.10291416 | 2.34298809 | 1.70113143 |
| 6 | 2.74239975 | 2.51302304 | 1.93149884 |
| 6 | 1.82756687 | 1.53926774 | 1.51290879 |
| 6 | 0.37268339 | 1.69528021 | 1.87728547 |
| 1 | 4.81902622 | 3.09705623 | 2.02757110 |
| 6 | 4.20835438 | -1.06302521 | -0.12953404 |
| 6 | -2.11636246 | 0.75610778 | -5.98589903 |
| 6 | -1.94899220 | -0.78957955 | -4.04460649 |
| 6 | 0.12625285 | 0.29976392 | -5.01359614 |
| 6 | 5.74550386 | -1.05856460 | -0.23129942 |
| 6 | 3.65329875 | -1.12326994 | -1.57228705 |
| 6 | 3.80427909 | -2.33817086 | 0.64831535 |
| 1 | -2.09218260 | 2.79073480 | 1.73894460 |
| 1 | -2.67105905 | 1.80792930 | 0.35604338 |
| 1 | -0.40732687 | 3.78521880 | 0.26813762 |
| 1 | 0.83282908 | 2.73143032 | -0.45800742 |
| 1 | -2.81661356 | 2.82586800 | -4.96279274 |
| 1 | -1.93480818 | 4.71863505 | -1.21577214 |
| 1 | 0.03156057 | 0.87194664 | 2.52338639 |
| 1 | 0.22992193 | 2.63349542 | 2.43838008 |
| 1 | 5.61622066 | 1.07544822 | 0.87804306 |
| 1 | 2.37564712 | 3.39971100 | 2.45343575 |
| 1 | -1.96041078 | -0.10290361 | -6.65394972 |
| 1 | -3.20037251 | 0.86376105 | -5.83524037 |
| 1 | -1.74617015 | 1.65143311 | -6.50621567 |
| 1 | -1.44305150 | -1.06042811 | -3.11171190 |
| 1 | -3.02096217 | -0.66816367 | -3.83129217 |
| 1 | -1.83736990 | -1.62373257 | -4.75420655 |
| 1 | 0.53729409 | 1.20059187 | -5.49247255 |
| 1 | 0.72244527 | 0.07681261 | -4.12233739 |
| 1 | 0.23035759 | -0.53626086 | -5.72197856 |
| 1 | 6.07157944 | -1.97624919 | -0.74114263 |
| 1 | 6.22589530 | -1.04099868 | 0.75775067 |
| 1 | 6.12004876 | -0.20607533 | -0.81611279 |
| 1 | 2.56001178 | -1.18310300 | -1.58550747 |
| 1 | 3.95708566 | -0.23357758 | -2.14235008 |
| 1 | 4.05374674 | -2.00933614 | -2.08792646 |
| 1 | 4.23507030 | -2.32629300 | 1.66036036 |
| 1 | 2.71605294 | -2.42877941 | 0.73368832 |
| 1 | 4.18974986 | -3.22833887 | 0.12829463 |

| | | | |
|---|---|---|---|
| 1 | -2.18822609 | 1.00882178 | 1.88857707 |
| 31 | -0.24286023 | -0.03835719 | -0.30084327 |

Electronic energy = -3523.874025 a.u.
DFT-D3(BJ) dispersion correction = -0.112428 a.u.
Thermal free energy = 0.597989 a.u.
Gibbs free energy = -3523.388464 a.u.
Number of imaginary frequencies = 0.

**Int2 CL isomer/conformer 8**

| Atomic N. | X | Y | Z |
|---|---|---|---|
| 8 | 3.92678345 | -1.10637489 | -7.90266196 |
| 6 | 4.54563439 | -2.12586602 | -8.72327503 |
| 1 | 5.16844724 | -2.78791845 | -8.10857180 |
| 6 | 2.50525346 | -0.44573314 | -6.12466558 |
| 6 | 1.60985050 | -0.91360351 | -4.97803778 |
| 6 | 1.00339282 | 0.25378458 | -4.19231817 |
| 6 | 3.10791189 | -1.58699073 | -6.92088336 |
| 6 | 0.10834921 | -0.20682315 | -3.03619878 |
| 6 | -0.50057197 | 0.95720302 | -2.25327168 |
| 1 | 3.33237750 | 0.18129161 | -5.75417095 |
| 1 | 2.19455284 | -1.55570243 | -4.30125962 |
| 1 | 1.81369998 | 0.89001314 | -3.79613935 |
| 1 | 0.70032056 | -0.83138771 | -2.34438249 |
| 1 | 1.94668481 | 0.19349839 | -6.82826510 |
| 1 | 0.80779172 | -1.55066289 | -5.38136247 |
| 1 | 0.41692782 | 0.89156418 | -4.87641156 |
| 1 | -0.71014472 | -0.83595856 | -3.42413276 |
| 1 | -1.08121381 | 1.59638989 | -2.94130653 |
| 1 | 0.31171694 | 1.58336185 | -1.83921336 |
| 8 | 2.90780742 | -2.77272850 | -6.73675517 |
| 8 | -1.40901876 | 0.53050068 | -1.23296805 |
| 1 | 5.15749601 | -1.58138732 | -9.44889946 |
| 1 | 3.78080470 | -2.72606870 | -9.23212007 |
| 7 | -1.11472529 | -1.95644250 | 0.79740139 |
| 8 | -1.76563579 | 0.76207459 | 1.82614496 |
| 8 | 1.03494385 | 0.15262026 | 0.61163441 |
| 6 | -2.03354490 | -2.59603008 | -0.17780577 |
| 6 | -2.90810989 | 0.15302705 | 2.24139032 |
| 6 | -4.06318751 | 0.92583861 | 2.55282202 |
| 6 | -5.20068489 | 0.22907907 | 2.98702764 |
| 6 | -5.23501587 | -1.16189340 | 3.12141608 |
| 6 | -4.09458431 | -1.90291996 | 2.82996357 |
| 6 | -2.92899011 | -1.25684970 | 2.40420126 |
| 6 | -1.66333705 | -2.04450107 | 2.19994457 |
| 1 | -6.14651645 | -1.65604593 | 3.45755166 |
| 6 | -4.06950266 | 2.46095956 | 2.41631008 |
| 6 | 1.66842325 | -0.64808710 | 1.50816262 |
| 6 | 2.73297651 | -0.12963777 | 2.30069928 |
| 6 | 3.35354727 | -1.01194054 | 3.19671085 |
| 6 | 2.96960235 | -2.34890861 | 3.33426701 |
| 6 | 1.93737433 | -2.84495540 | 2.54642521 |
| 6 | 1.29100834 | -2.01140562 | 1.62575825 |

| | | | |
|---|---|---|---|
| 6 | 0.24995407 | -2.59849739 | 0.70748742 |
| 1 | 3.47872644 | -2.99313937 | 4.05088033 |
| 6 | 3.18664539 | 1.33906308 | 2.18465949 |
| 6 | -5.41517776 | 3.07070111 | 2.85272943 |
| 6 | -3.83859240 | 2.86289200 | 0.94034943 |
| 6 | -2.97203464 | 3.08012366 | 3.31431450 |
| 6 | 4.37329206 | 1.64938393 | 3.11671355 |
| 6 | 2.03063513 | 2.28709982 | 2.58234538 |
| 6 | 3.64766046 | 1.64148213 | 0.73880368 |
| 1 | -2.11037450 | -3.67539110 | 0.03082144 |
| 1 | -3.02568973 | -2.13905223 | -0.09730251 |
| 1 | -1.83063480 | -3.10828781 | 2.43834380 |
| 1 | -0.86421339 | -1.67082826 | 2.85664183 |
| 1 | -6.10226357 | 0.79006288 | 3.22628596 |
| 1 | -4.09243193 | -2.98889190 | 2.94881911 |
| 1 | 0.55647772 | -2.49227050 | -0.34557246 |
| 1 | 0.13491182 | -3.67505079 | 0.91642647 |
| 1 | 4.16625336 | -0.64453039 | 3.82049463 |
| 1 | 1.63124610 | -3.89028470 | 2.62811019 |
| 1 | -5.36103778 | 4.16376221 | 2.74938916 |
| 1 | -6.25102369 | 2.72210120 | 2.22894365 |
| 1 | -5.64830342 | 2.84907188 | 3.90433942 |
| 1 | -2.87833424 | 2.49780578 | 0.56062559 |
| 1 | -4.63645870 | 2.45996034 | 0.29981024 |
| 1 | -3.85214497 | 3.95970675 | 0.84841785 |
| 1 | -3.15197540 | 2.83329362 | 4.37094266 |
| 1 | -1.97453997 | 2.72190812 | 3.03840730 |
| 1 | -2.98887251 | 4.17628787 | 3.21650721 |
| 1 | 4.66369679 | 2.70136017 | 2.98531543 |
| 1 | 5.25353430 | 1.03204763 | 2.88594218 |
| 1 | 4.11646870 | 1.50881195 | 4.17658766 |
| 1 | 1.16232528 | 2.16881586 | 1.92571038 |
| 1 | 1.70995813 | 2.09641931 | 3.61655410 |
| 1 | 2.36972197 | 3.33221303 | 2.51978182 |
| 1 | 4.49955570 | 1.00292047 | 0.46275168 |
| 1 | 2.84069588 | 1.47733805 | 0.01657267 |
| 1 | 3.97438706 | 2.68989396 | 0.66557079 |
| 1 | -1.65136225 | -2.44026191 | -1.19302181 |
| 31 | -0.80957581 | 0.03757674 | 0.40674605 |

Electronic energy = -3523.873924 a.u.
DFT-D3(BJ) dispersion correction = -0.108607 a.u.
Thermal free energy = 0.594084 a.u.
Gibbs free energy = -3523.388447 a.u.
Number of imaginary frequencies = 0.

**Int2 CL isomer/conformer 9**

| Atomic N. | X | Y | Z |
|---|---|---|---|
| 8 | -1.61877250 | 6.47107812 | -0.14426334 |
| 6 | -2.45431381 | 6.43556152 | -1.32578736 |
| 1 | -1.84870194 | 6.23857374 | -2.21931390 |
| 6 | -0.02580976 | 5.49147220 | 1.31409607 |
| 6 | 0.33655927 | 4.17141975 | 2.00181613 |

| | | | |
|---|---|---|---|
| 6 | -0.85391863 | 3.50803437 | 2.70478164 |
| 6 | -0.92060173 | 5.32773924 | 0.10144777 |
| 6 | -0.48578253 | 2.26542456 | 3.52832395 |
| 6 | 0.12754422 | 1.12114970 | 2.72107523 |
| 1 | 0.88662344 | 5.99538529 | 0.95129255 |
| 1 | 1.13200775 | 4.37665037 | 2.73680261 |
| 1 | -1.33206240 | 4.24330444 | 3.37429143 |
| 1 | -1.38987256 | 1.88762377 | 4.03175790 |
| 1 | -0.50440393 | 6.19484816 | 2.01116332 |
| 1 | 0.76505310 | 3.48862933 | 1.25388515 |
| 1 | -1.60913536 | 3.22339205 | 1.95675434 |
| 1 | 0.23085158 | 2.53882706 | 4.32291196 |
| 1 | 0.36220992 | 0.28761293 | 3.40986237 |
| 1 | 1.08386317 | 1.44657924 | 2.27084602 |
| 8 | -1.00411740 | 4.33367133 | -0.60132064 |
| 8 | -0.79707938 | 0.68585801 | 1.71720995 |
| 1 | -2.91750518 | 7.42515513 | -1.38185956 |
| 1 | -3.21974363 | 5.65517968 | -1.22990795 |
| 7 | -1.19823575 | 0.35011511 | -1.40967131 |
| 8 | -0.49021732 | -2.04931925 | 0.21406924 |
| 8 | 1.56677207 | 0.22367341 | -0.19707668 |
| 6 | -2.43925483 | 1.10839940 | -1.10221843 |
| 6 | -1.70038955 | -2.49514187 | -0.21445326 |
| 6 | -2.36528576 | -3.54924253 | 0.47546472 |
| 6 | -3.60632389 | -3.96306924 | -0.03079015 |
| 6 | -4.19450113 | -3.38963410 | -1.16186856 |
| 6 | -3.52612559 | -2.37299649 | -1.83639439 |
| 6 | -2.27992745 | -1.93008562 | -1.38082648 |
| 6 | -1.50422355 | -0.91276728 | -2.17436170 |
| 1 | -5.16436360 | -3.74310771 | -1.51193498 |
| 6 | -1.75087748 | -4.20953026 | 1.72521689 |
| 6 | 1.95588725 | 0.15747199 | -1.49680284 |
| 6 | 3.25677242 | -0.32306251 | -1.82537545 |
| 6 | 3.60448440 | -0.35711363 | -3.18346849 |
| 6 | 2.73647246 | 0.05453116 | -4.19865347 |
| 6 | 1.47531856 | 0.53380128 | -3.86278403 |
| 6 | 1.08017139 | 0.60362405 | -2.52152515 |
| 6 | -0.24397333 | 1.23888436 | -2.17854054 |
| 1 | 3.04979772 | 0.00111356 | -5.24124229 |
| 6 | 4.24437154 | -0.79097484 | -0.73808387 |
| 6 | -2.63531029 | -5.34795710 | 2.26791225 |
| 6 | -1.59839692 | -3.16616528 | 2.85725103 |
| 6 | -0.37285439 | -4.82222883 | 1.37909283 |
| 6 | 5.59234002 | -1.23410014 | -1.33793255 |
| 6 | 3.66124082 | -2.00349421 | 0.02542310 |
| 6 | 4.53876142 | 0.36549444 | 0.24686500 |
| 1 | -2.92750310 | 1.41420037 | -2.04170139 |
| 1 | -3.12287630 | 0.47360752 | -0.52822588 |
| 1 | -2.05726122 | -0.63562720 | -3.08736188 |
| 1 | -0.52958051 | -1.32202058 | -2.47842213 |
| 1 | -4.14339307 | -4.76166007 | 0.47788653 |
| 1 | -3.95755909 | -1.92580228 | -2.73492469 |
| 1 | -0.10728546 | 2.13314403 | -1.55102383 |

| | | | |
|---|---|---|---|
| 1 | -0.75253605 | 1.55601179 | -3.10399866 |
| 1 | 4.58875097 | -0.72511608 | -3.46716576 |
| 1 | 0.78750596 | 0.87505790 | -4.63977396 |
| 1 | -2.14665863 | -5.79075014 | 3.14750136 |
| 1 | -3.62508288 | -4.98960271 | 2.58616417 |
| 1 | -2.77602854 | -6.15006948 | 1.52892682 |
| 1 | -0.94526983 | -2.33824960 | 2.56140607 |
| 1 | -2.57731610 | -2.75011084 | 3.13601904 |
| 1 | -1.16719303 | -3.64616706 | 3.74906459 |
| 1 | -0.47762632 | -5.59597743 | 0.60447767 |
| 1 | 0.32739322 | -4.06140470 | 1.01798451 |
| 1 | 0.05741200 | -5.29706721 | 2.27408223 |
| 1 | 6.26061519 | -1.54455672 | -0.52216655 |
| 1 | 6.09116422 | -0.41899931 | -1.88204644 |
| 1 | 5.48224331 | -2.09119197 | -2.01790645 |
| 1 | 2.72055258 | -1.75380625 | 0.52718030 |
| 1 | 3.47415889 | -2.84124120 | -0.66164380 |
| 1 | 4.37947312 | -2.34352984 | 0.78697526 |
| 1 | 4.99693435 | 1.21506723 | -0.28049881 |
| 1 | 3.62595770 | 0.71412974 | 0.74183883 |
| 1 | 5.24685941 | 0.02532851 | 1.01783460 |
| 1 | -2.18050976 | 1.99773931 | -0.51760372 |
| 31 | -0.18036201 | -0.21162361 | 0.26396592 |

Electronic energy = -3523.876908 a.u.
DFT-D3(BJ) dispersion correction = -0.111427 a.u.
Thermal free energy = 0.600729 a.u.
Gibbs free energy = -3523.387606 a.u.
Number of imaginary frequencies = 0.

**Int2 CL isomer/conformer 10**

| Atomic N. | X | Y | Z |
|---|---|---|---|
| 7 | 0.33270628 | 1.32612304 | 2.74779124 |
| 8 | -0.61111166 | 1.22058403 | -0.07744718 |
| 8 | 2.33538965 | 0.50950342 | 0.65119543 |
| 6 | -0.45255908 | 0.60253788 | 3.77843591 |
| 6 | -1.73393804 | 1.77633683 | 0.45132289 |
| 6 | -2.96644756 | 1.71318883 | -0.25983791 |
| 6 | -4.08052277 | 2.32193571 | 0.33681848 |
| 6 | -4.02094358 | 2.96985169 | 1.57409632 |
| 6 | -2.80780696 | 3.03555364 | 2.25114730 |
| 6 | -1.66192141 | 2.45625030 | 1.69503616 |
| 6 | -0.32933623 | 2.62837734 | 2.37337020 |
| 1 | -4.91733892 | 3.42293894 | 1.99746264 |
| 6 | -3.07754251 | 1.00247631 | -1.62278380 |
| 6 | 2.93989275 | 1.67158980 | 1.01009648 |
| 6 | 3.88824390 | 2.28234375 | 0.13945558 |
| 6 | 4.48630813 | 3.47246870 | 0.57776003 |
| 6 | 4.18883144 | 4.06360421 | 1.80917417 |
| 6 | 3.27072457 | 3.45111032 | 2.65416258 |
| 6 | 2.65230833 | 2.25457778 | 2.27182551 |
| 6 | 1.74118483 | 1.55280912 | 3.24520304 |
| 1 | 4.67598162 | 4.99385191 | 2.10095210 |

| | | | |
|---|---|---|---|
| 6 | 4.24438049 | 1.66708265 | -1.22824218 |
| 6 | -4.49945904 | 1.09976367 | -2.20743499 |
| 6 | -2.74700607 | -0.50045598 | -1.46440115 |
| 6 | -2.11528314 | 1.65288470 | -2.64522432 |
| 6 | 5.31400148 | 2.49243717 | -1.96840849 |
| 6 | 2.99175720 | 1.62075696 | -2.13491150 |
| 6 | 4.81478465 | 0.24136087 | -1.03771265 |
| 1 | -0.47347279 | 1.18518609 | 4.71372734 |
| 1 | -1.47689036 | 0.45530537 | 3.41981044 |
| 1 | -0.44094792 | 3.23496424 | 3.28787468 |
| 1 | 0.38051916 | 3.14359001 | 1.70986317 |
| 1 | -5.03852827 | 2.28697887 | -0.17876609 |
| 1 | -2.73418604 | 3.55460152 | 3.20960029 |
| 1 | 2.13139487 | 0.55124787 | 3.48846319 |
| 1 | 1.68406267 | 2.12935504 | 4.18355886 |
| 1 | 5.21012771 | 3.96696367 | -0.06732810 |
| 1 | 3.03390850 | 3.88907762 | 3.62639680 |
| 1 | -4.51973109 | 0.58707166 | -3.17943061 |
| 1 | -5.24757654 | 0.61509510 | -1.56312899 |
| 1 | -4.80760865 | 2.14193131 | -2.37576550 |
| 1 | -1.72862418 | -0.65940228 | -1.09433234 |
| 1 | -3.44541221 | -0.97456197 | -0.75851627 |
| 1 | -2.84192401 | -1.01070805 | -2.43462509 |
| 1 | -2.37066532 | 2.71197739 | -2.79671394 |
| 1 | -1.07279840 | 1.59001670 | -2.31577788 |
| 1 | -2.20558289 | 1.14090116 | -3.61503588 |
| 1 | 5.53884562 | 2.00517831 | -2.92767540 |
| 1 | 6.25409394 | 2.55560953 | -1.40140408 |
| 1 | 4.97076547 | 3.51325722 | -2.19032010 |
| 1 | 2.19893387 | 1.00036803 | -1.70412704 |
| 1 | 2.59034554 | 2.63155904 | -2.29609271 |
| 1 | 3.25983863 | 1.20235750 | -3.11675316 |
| 1 | 5.73202010 | 0.26931651 | -0.43139262 |
| 1 | 4.09222095 | -0.41907386 | -0.54640805 |
| 1 | 5.07116501 | -0.18912528 | -2.01756124 |
| 1 | 0.00356771 | -0.37696082 | 3.96242832 |
| 8 | -3.36691419 | -5.14633228 | -1.40638798 |
| 6 | -4.56013188 | -4.39340726 | -1.72811783 |
| 1 | -4.34825644 | -3.31695254 | -1.71888469 |
| 6 | -1.13230046 | -5.80834383 | -1.87375059 |
| 6 | -0.61585750 | -5.53306150 | -0.44989536 |
| 6 | -0.06037299 | -4.11806086 | -0.25944159 |
| 6 | -2.32991967 | -4.96973431 | -2.27880579 |
| 6 | 0.47959833 | -3.88381839 | 1.15478589 |
| 6 | 1.08955942 | -2.49935868 | 1.36021718 |
| 1 | -0.34824848 | -5.62011648 | -2.61877844 |
| 1 | 0.17375590 | -6.26947470 | -0.22636573 |
| 1 | 0.74650921 | -3.94041835 | -0.99195572 |
| 1 | 1.25727302 | -4.63268243 | 1.38804022 |
| 1 | -1.42899679 | -6.86630844 | -1.95514664 |
| 1 | -1.42768001 | -5.71660936 | 0.27051012 |
| 1 | -0.84105537 | -3.37163927 | -0.47175305 |
| 1 | -0.32931319 | -4.02496438 | 1.89115153 |

| | | | |
|---|---|---|---|
| 1 | 1.54465934 | -2.45137575 | 2.36905242 |
| 1 | 1.90542171 | -2.34420409 | 0.62959456 |
| 8 | -2.39375930 | -4.24399941 | -3.25345044 |
| 8 | 0.07181266 | -1.49853167 | 1.22000648 |
| 1 | -5.28448953 | -4.65287164 | -0.94981641 |
| 1 | -4.93587182 | -4.67585317 | -2.71970757 |
| 31 | 0.53143857 | 0.24419772 | 1.00942959 |

Electronic energy = -3523.873085 a.u.
DFT-D3(BJ) dispersion correction = -0.110061 a.u.
Thermal free energy = 0.595649 a.u.
Gibbs free energy = -3523.387497 a.u.
Number of imaginary frequencies = 0.

**Int2 CL isomer/conformer 11**

| Atomic N. | X | Y | Z |
|---|---|---|---|
| 8 | -3.99843832 | -6.12003148 | -2.23406698 |
| 6 | -3.85937251 | -7.55960849 | -2.28088617 |
| 1 | -2.80072151 | -7.84544561 | -2.23808196 |
| 6 | -3.68828582 | -4.03499176 | -1.14914195 |
| 6 | -3.13604767 | -3.31065745 | 0.07798846 |
| 6 | -3.34462274 | -1.79539455 | 0.00050528 |
| 6 | -3.49455031 | -5.53758579 | -1.10413145 |
| 6 | -2.79201764 | -1.04843776 | 1.21791711 |
| 6 | -2.90185970 | 0.47218774 | 1.10078830 |
| 1 | -3.21666397 | -3.66259241 | -2.07322680 |
| 1 | -2.06280727 | -3.53523867 | 0.17773936 |
| 1 | -2.86627092 | -1.39833250 | -0.90867992 |
| 1 | -1.73210581 | -1.31757872 | 1.37390848 |
| 1 | -4.76638767 | -3.84042814 | -1.27370047 |
| 1 | -3.61341527 | -3.71455024 | 0.98453006 |
| 1 | -4.42270423 | -1.57451963 | -0.09594669 |
| 1 | -3.32873404 | -1.36494256 | 2.12970401 |
| 1 | -2.59972452 | 0.93782406 | 2.05637773 |
| 1 | -3.95500909 | 0.75126298 | 0.91657374 |
| 8 | -2.96986064 | -6.16791170 | -0.20580721 |
| 8 | -2.14287057 | 0.99778290 | 0.00167141 |
| 1 | -4.30445553 | -7.86299035 | -3.23350332 |
| 1 | -4.38816237 | -8.02488831 | -1.43931271 |
| 7 | -0.14183788 | 3.38934228 | -0.40649575 |
| 8 | 0.89234712 | 0.61754621 | -0.78393019 |
| 8 | 0.07154439 | 1.62263995 | 2.03372690 |
| 6 | -1.25167377 | 3.84966502 | -1.27882967 |
| 6 | 1.17295802 | 1.06646321 | -2.03636153 |
| 6 | 1.41282226 | 0.13891060 | -3.09096348 |
| 6 | 1.69579885 | 0.66888166 | -4.35848263 |
| 6 | 1.75552860 | 2.04209613 | -4.61169059 |
| 6 | 1.54795121 | 2.93628546 | -3.56691558 |
| 6 | 1.27200680 | 2.46166440 | -2.27993468 |
| 6 | 1.17867562 | 3.43018787 | -1.13111802 |
| 1 | 1.97289244 | 2.40387323 | -5.61657886 |
| 6 | 1.37508655 | -1.38369183 | -2.85384077 |
| 6 | 0.98794025 | 2.54785770 | 2.41822321 |

| | | | |
|---|---|---|---|
| 6 | 1.93912538 | 2.23039910 | 3.43081371 |
| 6 | 2.84905883 | 3.23392661 | 3.79258635 |
| 6 | 2.85316923 | 4.50286860 | 3.20598251 |
| 6 | 1.91236408 | 4.80353175 | 2.22797528 |
| 6 | 0.97174056 | 3.84287149 | 1.83678870 |
| 6 | -0.10311216 | 4.22566477 | 0.85214131 |
| 1 | 3.58632611 | 5.24677888 | 3.51723336 |
| 6 | 1.97205496 | 0.84348689 | 4.10279154 |
| 6 | 1.70537172 | -2.17422652 | -4.13394426 |
| 6 | -0.03675612 | -1.81840257 | -2.39593629 |
| 6 | 2.42262488 | -1.77866470 | -1.78553995 |
| 6 | 3.06514435 | 0.75493162 | 5.18460652 |
| 6 | 2.27628866 | -0.25086353 | 3.05247398 |
| 6 | 0.61777430 | 0.55453309 | 4.79338168 |
| 1 | -1.11930675 | 4.91590209 | -1.52372310 |
| 1 | -1.25408774 | 3.26287621 | -2.20383578 |
| 1 | 1.34836842 | 4.46066007 | -1.48571790 |
| 1 | 1.94220870 | 3.20426314 | -0.37225505 |
| 1 | 1.87417645 | -0.01401482 | -5.18704630 |
| 1 | 1.61803872 | 4.01326174 | -3.73608146 |
| 1 | -1.10220740 | 4.11823759 | 1.30467132 |
| 1 | 0.02186349 | 5.28073439 | 0.55705560 |
| 1 | 3.59192063 | 3.02027356 | 4.55881782 |
| 1 | 1.88940053 | 5.79337046 | 1.76661022 |
| 1 | 1.67737031 | -3.24915129 | -3.90584691 |
| 1 | 0.97740563 | -1.98818527 | -4.93694611 |
| 1 | 2.71076522 | -1.94330639 | -4.51458916 |
| 1 | -0.32420844 | -1.33220502 | -1.45774407 |
| 1 | -0.78651467 | -1.56801561 | -3.16080630 |
| 1 | -0.05736204 | -2.90824204 | -2.24191748 |
| 1 | 3.43562736 | -1.51350443 | -2.12163012 |
| 1 | 2.23181145 | -1.27999603 | -0.82920633 |
| 1 | 2.39490732 | -2.86680960 | -1.62310838 |
| 1 | 3.03749386 | -0.24623814 | 5.63744709 |
| 1 | 2.91037237 | 1.48670421 | 5.99068269 |
| 1 | 4.07288713 | 0.90071892 | 4.76948803 |
| 1 | 1.51153955 | -0.28753103 | 2.26956279 |
| 1 | 3.25066463 | -0.07176373 | 2.57554959 |
| 1 | 2.31495210 | -1.23529444 | 3.54272752 |
| 1 | 0.41271425 | 1.30348036 | 5.57230442 |
| 1 | -0.21013685 | 0.56257286 | 4.07637472 |
| 1 | 0.65142352 | -0.43374747 | 5.27632718 |
| 1 | -2.20507267 | 3.69772839 | -0.76035275 |
| 31 | -0.40514220 | 1.46573239 | 0.24380711 |

Electronic energy = -3523.874730 a.u.
DFT-D3(BJ) dispersion correction = -0.109996 a.u.
Thermal free energy = 0.597285 a.u.
Gibbs free energy = -3523.387441 a.u.
Number of imaginary frequencies = 0.

**Int2 CL isomer/conformer 12**

Atomic N. X Y Z

| | | | |
|---|---|---|---|
| 8 | -4.24603805 | 3.04732076 | 1.97822871 |
| 6 | -4.88512401 | 2.02160500 | 1.17506997 |
| 1 | -5.37143716 | 2.46893630 | 0.29956067 |
| 6 | -2.65640492 | 4.81105431 | 2.22448491 |
| 6 | -1.12908695 | 4.61864484 | 2.26996820 |
| 6 | -0.71225361 | 3.35782354 | 3.03750761 |
| 6 | -3.30835280 | 3.78197398 | 1.32151223 |
| 6 | 0.77669943 | 3.00352322 | 2.91009828 |
| 6 | 1.16267288 | 2.46431247 | 1.53025055 |
| 1 | -2.89307945 | 5.79932904 | 1.80056945 |
| 1 | -0.67351623 | 5.50698692 | 2.73590569 |
| 1 | -0.96599561 | 3.48850198 | 4.10211267 |
| 1 | 1.02904588 | 2.23331614 | 3.65567435 |
| 1 | -3.09382193 | 4.75758664 | 3.23042569 |
| 1 | -0.76199768 | 4.58271317 | 1.23317776 |
| 1 | -1.29383385 | 2.49250243 | 2.68344549 |
| 1 | 1.40624969 | 3.88330624 | 3.13108620 |
| 1 | 2.23848045 | 2.21193410 | 1.52538312 |
| 1 | 1.01228583 | 3.25150856 | 0.76395399 |
| 8 | -3.02989287 | 3.61646337 | 0.14512540 |
| 8 | 0.35628266 | 1.32008124 | 1.24099258 |
| 1 | -5.62226163 | 1.55722163 | 1.83669182 |
| 1 | -4.14492480 | 1.28350392 | 0.84203288 |
| 7 | -0.05850795 | 0.87564090 | -1.97264086 |
| 8 | -0.24816745 | -1.36898625 | -0.00767226 |
| 8 | 2.48176106 | 0.03676638 | -0.59872556 |
| 6 | -0.95902157 | 2.05062897 | -1.84269323 |
| 6 | -1.53854846 | -1.41213301 | -0.43243613 |
| 6 | -2.53617321 | -2.05067923 | 0.35919486 |
| 6 | -3.84481725 | -2.05915348 | -0.14669615 |
| 6 | -4.19032344 | -1.47814040 | -1.37062112 |
| 6 | -3.20197389 | -0.87515537 | -2.14214779 |
| 6 | -1.87842490 | -0.84982381 | -1.69070227 |
| 6 | -0.78977584 | -0.29903764 | -2.57283553 |
| 1 | -5.22324021 | -1.50790356 | -1.71771531 |
| 6 | -2.19863038 | -2.70253329 | 1.71439829 |
| 6 | 2.83199456 | -0.38107972 | -1.84367816 |
| 6 | 3.88625119 | -1.32425134 | -2.01226230 |
| 6 | 4.20108085 | -1.70420882 | -3.32498697 |
| 6 | 3.53132749 | -1.19904964 | -4.44320984 |
| 6 | 2.51505751 | -0.26729732 | -4.26487597 |
| 6 | 2.16740478 | 0.15586877 | -2.97658019 |
| 6 | 1.14378175 | 1.24815803 | -2.80773466 |
| 1 | 3.80876199 | -1.53213738 | -5.44318476 |
| 6 | 4.65202507 | -1.90557125 | -0.80740502 |
| 6 | -3.43049220 | -3.37240092 | 2.35215013 |
| 6 | -1.69018092 | -1.63048322 | 2.70712414 |
| 6 | -1.12532268 | -3.80090470 | 1.52489206 |
| 6 | 5.76595990 | -2.87411473 | -1.24781645 |
| 6 | 3.68817516 | -2.69889560 | 0.10618114 |
| 6 | 5.32408770 | -0.76541524 | -0.00565056 |
| 1 | -1.30187565 | 2.37060979 | -2.83989828 |
| 1 | -1.82583565 | 1.79939035 | -1.22491996 |

| | | | |
|---|---|---|---|
| 1 | -1.20709643 | 0.01607876 | -3.54392244 |
| 1 | -0.02346995 | -1.06465327 | -2.76330569 |
| 1 | -4.63162443 | -2.53500021 | 0.43601350 |
| 1 | -3.44627938 | -0.43307752 | -3.11061520 |
| 1 | 1.59113462 | 2.12024512 | -2.30441221 |
| 1 | 0.78323728 | 1.57842263 | -3.79620680 |
| 1 | 4.99683077 | -2.42883214 | -3.48736430 |
| 1 | 1.98966726 | 0.15195904 | -5.12587683 |
| 1 | -3.13211246 | -3.83336104 | 3.30440598 |
| 1 | -4.22944828 | -2.64952358 | 2.57288099 |
| 1 | -3.84567024 | -4.16709539 | 1.71539389 |
| 1 | -0.78480705 | -1.13165313 | 2.34559720 |
| 1 | -2.45993024 | -0.86340147 | 2.87756644 |
| 1 | -1.46190313 | -2.10092927 | 3.67570537 |
| 1 | -1.49307904 | -4.58950510 | 0.85210896 |
| 1 | -0.19855739 | -3.39213385 | 1.10860552 |
| 1 | -0.89667522 | -4.26580930 | 2.49596136 |
| 1 | 6.28692655 | -3.24749156 | -0.35480566 |
| 1 | 6.51442615 | -2.38163926 | -1.88540040 |
| 1 | 5.36865707 | -3.74672116 | -1.78621028 |
| 1 | 2.89452191 | -2.06246575 | 0.51130653 |
| 1 | 3.22010005 | -3.52580203 | -0.44697359 |
| 1 | 4.24770058 | -3.12968266 | 0.95030791 |
| 1 | 6.04467925 | -0.22262298 | -0.63484666 |
| 1 | 4.58558505 | -0.05134668 | 0.37431935 |
| 1 | 5.87373854 | -1.18750075 | 0.84936564 |
| 1 | -0.42105942 | 2.87571776 | -1.36283596 |
| 31 | 0.68445499 | 0.22996770 | -0.16687215 |

Electronic energy = -3523.875521 a.u.
DFT-D3(BJ) dispersion correction = -0.112655 a.u.
Thermal free energy = 0.600736 a.u.
Gibbs free energy = -3523.387439 a.u.
Number of imaginary frequencies = 0.

**Int2 CL isomer/conformer 13**

| Atomic N. | X | Y | Z |
|---|---|---|---|
| 8 | -2.60182844 | -3.75864483 | -5.36184305 |
| 6 | -3.49095963 | -4.55051951 | -4.53738356 |
| 1 | -2.98836374 | -5.46522699 | -4.19891839 |
| 6 | -0.56386778 | -2.59597888 | -5.71061817 |
| 6 | 0.51179750 | -1.76302108 | -5.00863326 |
| 6 | -0.04783545 | -0.54624214 | -4.26419252 |
| 6 | -1.44950987 | -3.36776605 | -4.75001301 |
| 6 | 1.02328416 | 0.23287646 | -3.49574224 |
| 6 | 0.45214212 | 1.35285589 | -2.62344114 |
| 1 | -0.10177221 | -3.34644091 | -6.37428871 |
| 1 | 1.24620850 | -1.43289390 | -5.76073494 |
| 1 | -0.54360673 | 0.12950367 | -4.98304816 |
| 1 | 1.74954465 | 0.67653511 | -4.19956401 |
| 1 | -1.20335278 | -1.97653873 | -6.35715655 |
| 1 | 1.05352667 | -2.40998585 | -4.30122855 |
| 1 | -0.82557071 | -0.86835859 | -3.55530093 |

| | | | |
|---|---|---|---|
| 1 | 1.59797065 | -0.45560716 | -2.85127572 |
| 1 | -0.11985208 | 2.05582408 | -3.25543530 |
| 1 | 1.28554689 | 1.92239340 | -2.17485472 |
| 8 | -1.18122977 | -3.64263285 | -3.59266201 |
| 8 | -0.45673809 | 0.87153767 | -1.62619493 |
| 1 | -4.34302002 | -4.79080494 | -5.18017736 |
| 1 | -3.81610242 | -3.97355185 | -3.66260334 |
| 7 | -0.17543871 | -1.57936218 | 0.48887310 |
| 8 | -0.79908798 | 1.18137255 | 1.42066886 |
| 8 | 1.98468142 | 0.50789308 | 0.20884265 |
| 6 | -1.12096729 | -2.26284273 | -0.43205166 |
| 6 | -1.93126857 | 0.59227529 | 1.88678748 |
| 6 | -3.07937145 | 1.37935540 | 2.18951456 |
| 6 | -4.20724472 | 0.70347340 | 2.67823908 |
| 6 | -4.23875828 | -0.68055008 | 2.87284679 |
| 6 | -3.10532708 | -1.43489323 | 2.58761716 |
| 6 | -1.94939996 | -0.80923548 | 2.10885640 |
| 6 | -0.68846477 | -1.60520365 | 1.90730133 |
| 1 | -5.14258590 | -1.15871142 | 3.25045793 |
| 6 | -3.08752413 | 2.90706711 | 1.98685194 |
| 6 | 2.62903299 | -0.27307729 | 1.11370225 |
| 6 | 3.71119852 | 0.25916465 | 1.87313679 |
| 6 | 4.34314690 | -0.60292722 | 2.78064837 |
| 6 | 3.95436489 | -1.93356130 | 2.96002474 |
| 6 | 2.90474080 | -2.44337199 | 2.20451183 |
| 6 | 2.24601532 | -1.63050437 | 1.27417636 |
| 6 | 1.18299133 | -2.23519819 | 0.39327785 |
| 1 | 4.47283492 | -2.56200283 | 3.68398808 |
| 6 | 4.17126118 | 1.72131599 | 1.70963875 |
| 6 | -4.42277876 | 3.53785196 | 2.42472591 |
| 6 | -2.88610047 | 3.24413194 | 0.49032564 |
| 6 | -1.97077815 | 3.56243626 | 2.83408908 |
| 6 | 5.37367043 | 2.05035534 | 2.61464569 |
| 6 | 3.02699651 | 2.68730953 | 2.09807934 |
| 6 | 4.61155619 | 1.98010384 | 0.24899382 |
| 1 | -1.23047559 | -3.31862159 | -0.13727394 |
| 1 | -2.09775493 | -1.76978762 | -0.38329775 |
| 1 | -0.84967519 | -2.65790392 | 2.19384848 |
| 1 | 0.12535492 | -1.20437919 | 2.52904046 |
| 1 | -5.10320618 | 1.27529352 | 2.91320590 |
| 1 | -3.10104120 | -2.51501277 | 2.75125813 |
| 1 | 1.46818623 | -2.16408510 | -0.66849008 |
| 1 | 1.06188499 | -3.30389037 | 0.63559449 |
| 1 | 5.16908522 | -0.22415765 | 3.37983420 |
| 1 | 2.59352175 | -3.48406781 | 2.31948233 |
| 1 | -4.37028824 | 4.62517472 | 2.27155179 |
| 1 | -5.27256188 | 3.16308159 | 1.83583078 |
| 1 | -4.63291680 | 3.36353748 | 3.49003339 |
| 1 | -1.93474798 | 2.85833141 | 0.10870162 |
| 1 | -3.69830955 | 2.81696540 | -0.11562814 |
| 1 | -2.89801200 | 4.33599551 | 0.35102295 |
| 1 | -2.12868027 | 3.36139383 | 3.90387884 |
| 1 | -0.97982192 | 3.19053110 | 2.55278787 |

| | | | |
|---|---|---|---|
| 1 | -1.98847107 | 4.65347622 | 2.68968516 |
| 1 | 5.66723165 | 3.09686202 | 2.45039803 |
| 1 | 6.24723151 | 1.42231672 | 2.38731326 |
| 1 | 5.13241758 | 1.93962091 | 3.68171681 |
| 1 | 2.14760421 | 2.55463868 | 1.45926310 |
| 1 | 2.72218931 | 2.52858391 | 3.14247588 |
| 1 | 3.37060843 | 3.72823821 | 1.99972982 |
| 1 | 5.45610135 | 1.32957111 | -0.02166164 |
| 1 | 3.79302128 | 1.79910828 | -0.45595318 |
| 1 | 4.94187575 | 3.02438008 | 0.14051077 |
| 1 | -0.74159242 | -2.21752038 | -1.45835100 |
| 31 | 0.13743445 | 0.39234931 | 0.02003212 |

Electronic energy = -3523.876153 a.u.
DFT-D3(BJ) dispersion correction = -0.110820 a.u.
Thermal free energy = 0.599848 a.u.
Gibbs free energy = -3523.387124 a.u.
Number of imaginary frequencies = 0.

**Int2 CL isomer/conformer 14**

| Atomic N. | X | Y | Z |
|---|---|---|---|
| 8 | -1.92683033 | -7.29888329 | -1.94864220 |
| 6 | -2.12461081 | -8.72970218 | -2.03590418 |
| 1 | -1.51457912 | -9.15386007 | -2.84354770 |
| 6 | -0.53624617 | -5.40774522 | -1.61568000 |
| 6 | 0.88049522 | -4.90592737 | -1.33902156 |
| 6 | 0.95561187 | -3.37721580 | -1.28010749 |
| 6 | -0.63966508 | -6.91828455 | -1.68904482 |
| 6 | 2.36918940 | -2.85939692 | -0.99866703 |
| 6 | 2.45777866 | -1.33701662 | -0.92400826 |
| 1 | -0.92440731 | -4.99913620 | -2.56315494 |
| 1 | 1.55913640 | -5.28912323 | -2.11713099 |
| 1 | 0.59864180 | -2.95271863 | -2.23554416 |
| 1 | 3.06013510 | -3.20456826 | -1.78766268 |
| 1 | -1.23778181 | -5.06000760 | -0.83975920 |
| 1 | 1.23791411 | -5.33469333 | -0.39007137 |
| 1 | 0.27658075 | -2.99984454 | -0.49923656 |
| 1 | 2.73748569 | -3.27819881 | -0.04742515 |
| 1 | 2.07963615 | -0.89681206 | -1.86575854 |
| 1 | 3.52111559 | -1.04043563 | -0.82873384 |
| 8 | 0.27356857 | -7.70853344 | -1.54323943 |
| 8 | 1.71339281 | -0.86506999 | 0.20790705 |
| 1 | -3.19057825 | -8.86273355 | -2.24466526 |
| 1 | -1.85040881 | -9.21432605 | -1.09026272 |
| 7 | 2.31410960 | 2.16644439 | 1.16640023 |
| 8 | -0.43766520 | 1.02156374 | 1.32529568 |
| 8 | 0.89951385 | 1.60910614 | -1.43270125 |
| 6 | 3.40345537 | 1.49986743 | 1.92190256 |
| 6 | -0.27760015 | 1.25448641 | 2.65551788 |
| 6 | -1.10097942 | 0.58201065 | 3.60349763 |
| 6 | -0.88927045 | 0.87778605 | 4.95822057 |
| 6 | 0.07720650 | 1.78826026 | 5.39514563 |
| 6 | 0.86211706 | 2.45090878 | 4.45755661 |

| | | | |
|---|---|---|---|
| 6 | 0.68441795 | 2.20232062 | 3.09205009 |
| 6 | 1.45363339 | 3.00540363 | 2.07750446 |
| 1 | 0.20649391 | 1.97870290 | 6.46049786 |
| 6 | -2.17935761 | -0.42833149 | 3.16521015 |
| 6 | 0.94776888 | 2.96033403 | -1.56216339 |
| 6 | 0.07403352 | 3.62285609 | -2.47199056 |
| 6 | 0.18500585 | 5.01756180 | -2.56567363 |
| 6 | 1.10142118 | 5.75898726 | -1.81416202 |
| 6 | 1.95724588 | 5.09868252 | -0.94025894 |
| 6 | 1.89779279 | 3.70536007 | -0.81623426 |
| 6 | 2.90531836 | 2.99956065 | 0.05336320 |
| 1 | 1.14346664 | 6.84307166 | -1.91794312 |
| 6 | -0.95223501 | 2.84536591 | -3.31950313 |
| 6 | -2.96456183 | -0.98771950 | 4.36668827 |
| 6 | -1.52175979 | -1.62990513 | 2.44599506 |
| 6 | -3.20163881 | 0.25781904 | 2.22784063 |
| 6 | -1.75457309 | 3.77707624 | -4.24749678 |
| 6 | -1.96803530 | 2.12658500 | -2.40039275 |
| 6 | -0.22765247 | 1.81483724 | -4.21804319 |
| 1 | 4.09894979 | 2.25561567 | 2.32129277 |
| 1 | 2.97805765 | 0.92542636 | 2.75243000 |
| 1 | 2.10082332 | 3.74140847 | 2.58359200 |
| 1 | 0.76787933 | 3.55544088 | 1.41652461 |
| 1 | -1.49746210 | 0.37552971 | 5.70826672 |
| 1 | 1.60832588 | 3.18260944 | 4.77569717 |
| 1 | 3.51198684 | 2.30117582 | -0.54562752 |
| 1 | 3.58890561 | 3.73768186 | 0.50511031 |
| 1 | -0.47277112 | 5.55371448 | -3.24716938 |
| 1 | 2.69057611 | 5.65737163 | -0.35441447 |
| 1 | -3.72560905 | -1.69034137 | 3.99862943 |
| 1 | -2.31825345 | -1.53840898 | 5.06538908 |
| 1 | -3.48708449 | -0.19672062 | 4.92396790 |
| 1 | -0.97167738 | -1.32080850 | 1.55079486 |
| 1 | -0.82277643 | -2.14905110 | 3.11765561 |
| 1 | -2.29784315 | -2.34969729 | 2.14366237 |
| 1 | -3.70352227 | 1.08868001 | 2.74499806 |
| 1 | -2.72261835 | 0.64924340 | 1.32409004 |
| 1 | -3.97328843 | -0.46734644 | 1.92771571 |
| 1 | -2.45986944 | 3.17276790 | -4.83533739 |
| 1 | -1.10716242 | 4.31260576 | -4.95689960 |
| 1 | -2.34337754 | 4.51637398 | -3.68550762 |
| 1 | -1.47938682 | 1.39894735 | -1.74398194 |
| 1 | -2.50598819 | 2.85177688 | -1.77308042 |
| 1 | -2.70991505 | 1.59286518 | -3.01345753 |
| 1 | 0.46571198 | 2.32084630 | -4.90568327 |
| 1 | 0.33833156 | 1.08969576 | -3.62348076 |
| 1 | -0.96557717 | 1.26856934 | -4.82489663 |
| 1 | 3.94474178 | 0.81592834 | 1.25887540 |
| 31 | 1.05664698 | 0.82333073 | 0.24408201 |

Electronic energy = -3523.874389 a.u.
DFT-D3(BJ) dispersion correction = -0.108239 a.u.
Thermal free energy = 0.595644 a.u.
Gibbs free energy = -3523.386984 a.u.

Number of imaginary frequencies = 0.

**Int2_CL isomer/conformer 15**

| Atomic N. | X | Y | Z |
|---|---|---|---|
| 8 | -1.71520808 | -7.70799370 | 0.63185790 |
| 6 | -2.25328563 | -9.04450477 | 0.49686235 |
| 1 | -2.90458506 | -9.28475221 | 1.34680993 |
| 6 | -1.99734788 | -5.36117576 | 0.82013414 |
| 6 | -2.99236847 | -4.20244252 | 0.87276872 |
| 6 | -2.29939305 | -2.84419172 | 1.01790401 |
| 6 | -2.65731969 | -6.71837104 | 0.67773477 |
| 6 | -3.28649845 | -1.67381306 | 1.05729167 |
| 6 | -2.61313793 | -0.30890391 | 1.17626860 |
| 1 | -1.36968400 | -5.38757521 | 1.72610568 |
| 1 | -3.69207126 | -4.36277239 | 1.70795238 |
| 1 | -1.69065050 | -2.83586890 | 1.93964996 |
| 1 | -3.97597823 | -1.79110495 | 1.91159678 |
| 1 | -1.29269753 | -5.23988442 | -0.01877729 |
| 1 | -3.60702918 | -4.21196461 | -0.04065933 |
| 1 | -1.59963345 | -2.69063080 | 0.18138605 |
| 1 | -3.90623207 | -1.67931114 | 0.14512111 |
| 1 | -1.98145873 | -0.28270466 | 2.08394722 |
| 1 | -3.39201761 | 0.46926511 | 1.30005706 |
| 8 | -3.85308908 | -6.93188777 | 0.61182557 |
| 8 | -1.83714843 | -0.05324287 | -0.00317000 |
| 1 | -1.38236896 | -9.70682732 | 0.47663188 |
| 1 | -2.83147249 | -9.13440815 | -0.43152866 |
| 7 | -1.17015078 | 3.01239721 | -0.76184933 |
| 8 | 0.86062624 | 0.86332319 | -1.16343161 |
| 8 | -0.00118031 | 1.73928348 | 1.70379963 |
| 6 | -2.46521628 | 2.89924943 | -1.47794598 |
| 6 | 0.75685905 | 1.23924106 | -2.46621913 |
| 6 | 1.19260372 | 0.36244782 | -3.50074435 |
| 6 | 1.06649416 | 0.81853994 | -4.82124794 |
| 6 | 0.54262570 | 2.07340584 | -5.14492758 |
| 6 | 0.13746921 | 2.92645409 | -4.12392986 |
| 6 | 0.25203721 | 2.52612010 | -2.78809696 |
| 6 | -0.07877268 | 3.49437896 | -1.68407321 |
| 1 | 0.46025278 | 2.37870116 | -6.18798456 |
| 6 | 1.77857375 | -1.02848988 | -3.18834103 |
| 6 | 0.51245540 | 2.98075023 | 1.90361115 |
| 6 | 1.61811221 | 3.16329170 | 2.78363737 |
| 6 | 2.09049992 | 4.47231504 | 2.95551660 |
| 6 | 1.52609321 | 5.57486998 | 2.30738745 |
| 6 | 0.43948426 | 5.38447337 | 1.46170924 |
| 6 | -0.08061200 | 4.09983671 | 1.26301630 |
| 6 | -1.32361699 | 3.92949439 | 0.42880683 |
| 1 | 1.93472556 | 6.57221946 | 2.46892739 |
| 6 | 2.27242908 | 1.97616452 | 3.51761213 |
| 6 | 2.21431005 | -1.76861013 | -4.46712811 |
| 6 | 0.71665881 | -1.90809926 | -2.48687314 |
| 6 | 3.03049372 | -0.88823013 | -2.28971214 |

| | | | |
|---|---|---|---|
| 6 | 3.42367782 | 2.43146983 | 4.43432970 |
| 6 | 2.86661849 | 0.97901970 | 2.49489825 |
| 6 | 1.22922106 | 1.26261796 | 4.41032007 |
| 1 | -2.79678971 | 3.89455226 | -1.81566227 |
| 1 | -2.34624081 | 2.24237515 | -2.34614108 |
| 1 | -0.38711780 | 4.46540614 | -2.10681562 |
| 1 | 0.79829822 | 3.66719958 | -1.04333402 |
| 1 | 1.38388810 | 0.16943545 | -5.63528439 |
| 1 | -0.25438542 | 3.92004592 | -4.35337066 |
| 1 | -2.13768765 | 3.49488041 | 1.03124014 |
| 1 | -1.66345227 | 4.91246934 | 0.06214340 |
| 1 | 2.93800789 | 4.64399251 | 3.61644869 |
| 1 | -0.02549154 | 6.23409504 | 0.95667828 |
| 1 | 2.63532592 | -2.74481250 | -4.18767251 |
| 1 | 1.37050669 | -1.95601829 | -5.14689883 |
| 1 | 2.99117344 | -1.21929830 | -5.01847381 |
| 1 | 0.38186431 | -1.46958219 | -1.54074147 |
| 1 | -0.16228894 | -2.04476715 | -3.13350015 |
| 1 | 1.14008313 | -2.90223598 | -2.27657784 |
| 1 | 3.80714365 | -0.29851498 | -2.79833003 |
| 1 | 2.79246171 | -0.40220942 | -1.33755968 |
| 1 | 3.44803561 | -1.88443047 | -2.07850476 |
| 1 | 3.84514052 | 1.55138221 | 4.94031866 |
| 1 | 3.08182757 | 3.12828660 | 5.21329491 |
| 1 | 4.23858512 | 2.90928123 | 3.87155781 |
| 1 | 2.09638618 | 0.56282191 | 1.83713320 |
| 1 | 3.62682728 | 1.46944081 | 1.87007645 |
| 1 | 3.35109459 | 0.14643371 | 3.02704693 |
| 1 | 0.83329866 | 1.95258313 | 5.16981825 |
| 1 | 0.39138676 | 0.87590977 | 3.82030590 |
| 1 | 1.70481316 | 0.42032238 | 4.93525104 |
| 1 | -3.21654649 | 2.46809967 | -0.80636511 |
| 31 | -0.53824451 | 1.21128706 | 0.00508093 |

Electronic energy = -3523.874397 a.u.
DFT-D3(BJ) dispersion correction = -0.108192 a.u.
Thermal free energy = 0.595932 a.u.
Gibbs free energy = -3523.386657 a.u.
Number of imaginary frequencies = 0.

**Int2 CL isomer/conformer 16**

| Atomic N. | X | Y | Z |
|---|---|---|---|
| 8 | 0.37947139 | 5.62119613 | 7.46498085 |
| 6 | -0.12339745 | 6.68129960 | 8.31258939 |
| 1 | -0.16645878 | 7.62805828 | 7.75943347 |
| 6 | 0.23817894 | 4.26352074 | 5.52679097 |
| 6 | -0.58799262 | 3.89258817 | 4.29584491 |
| 6 | 0.06477243 | 2.78174898 | 3.46613794 |
| 6 | -0.37492273 | 5.37834467 | 6.35172313 |
| 6 | -0.75662012 | 2.40214871 | 2.22917267 |
| 6 | -0.10134444 | 1.30114686 | 1.40066224 |
| 1 | 1.25369039 | 4.58319720 | 5.23938635 |
| 1 | -0.73445179 | 4.78838124 | 3.67323690 |

| | | | |
|---|---|---|---|
| 1 | 1.07347549 | 3.10429487 | 3.15372879 |
| 1 | -0.90119424 | 3.28704498 | 1.58790725 |
| 1 | 0.38264802 | 3.39376441 | 6.18797426 |
| 1 | -1.59433422 | 3.57908052 | 4.61369808 |
| 1 | 0.20988560 | 1.88888594 | 4.09905113 |
| 1 | -1.75999234 | 2.06204314 | 2.53239907 |
| 1 | 0.04610395 | 0.40496272 | 2.03466909 |
| 1 | 0.89992185 | 1.63553846 | 1.06714321 |
| 8 | -1.39080080 | 5.99199294 | 6.08548011 |
| 8 | -0.93943861 | 0.99010004 | 0.28088967 |
| 1 | 0.58600460 | 6.74928349 | 9.14300622 |
| 1 | -1.12832893 | 6.43628872 | 8.67894329 |
| 7 | -1.36845604 | 0.40135890 | -2.74126191 |
| 8 | -0.65387984 | -1.90110555 | -0.97986969 |
| 8 | 1.42342323 | 0.29696285 | -1.58625488 |
| 6 | -2.58488471 | 1.20337804 | -2.45140711 |
| 6 | -1.88532240 | -2.34897569 | -1.34517803 |
| 6 | -2.54895051 | -3.34268743 | -0.57010840 |
| 6 | -3.81158988 | -3.76321315 | -1.01360259 |
| 6 | -4.42193349 | -3.25257840 | -2.16272068 |
| 6 | -3.75487750 | -2.29599439 | -2.92076671 |
| 6 | -2.48783481 | -1.84964798 | -2.52998383 |
| 6 | -1.71901766 | -0.90310577 | -3.41305761 |
| 1 | -5.40797702 | -3.60804106 | -2.46159763 |
| 6 | -1.91060026 | -3.93574541 | 0.70125962 |
| 6 | 1.77728317 | 0.12636804 | -2.88616266 |
| 6 | 3.05441586 | -0.41452213 | -3.21353578 |
| 6 | 3.36166495 | -0.55532277 | -4.57432522 |
| 6 | 2.47758149 | -0.19112385 | -5.59409681 |
| 6 | 1.24081871 | 0.34927992 | -5.26160066 |
| 6 | 0.88668166 | 0.52393455 | -3.91824990 |
| 6 | -0.41144323 | 1.21370787 | -3.58477605 |
| 1 | 2.76045587 | -0.32761144 | -6.63772145 |
| 6 | 4.06003481 | -0.82983237 | -2.12159498 |
| 6 | -2.80224560 | -5.01573887 | 1.34267894 |
| 6 | -1.70014448 | -2.82655237 | 1.75878011 |
| 6 | -0.55750964 | -4.60140604 | 0.35442030 |
| 6 | 5.37868146 | -1.35172765 | -2.72311882 |
| 6 | 3.46835110 | -1.96752062 | -1.25633003 |
| 6 | 4.41067903 | 0.38659570 | -1.23178623 |
| 1 | -3.06421846 | 1.51099722 | -3.39469468 |
| 1 | -3.28664090 | 0.59924112 | -1.86650224 |
| 1 | -2.29399373 | -0.67789208 | -4.32669186 |
| 1 | -0.76043582 | -1.34916420 | -3.71581234 |
| 1 | -4.34807250 | -4.51624425 | -0.43922444 |
| 1 | -4.20374962 | -1.90019781 | -3.83469771 |
| 1 | -0.22501675 | 2.13413669 | -3.00841695 |
| 1 | -0.93236318 | 1.49590289 | -4.51464490 |
| 1 | 4.32680123 | -0.97201458 | -4.85633216 |
| 1 | 0.54153608 | 0.65511074 | -6.04305185 |
| 1 | -2.29623208 | -5.41260551 | 2.23419713 |
| 1 | -3.77360431 | -4.61424540 | 1.66599726 |
| 1 | -2.98308946 | -5.86005306 | 0.66185499 |

| | | | |
|---|---|---|---|
| 1 | -1.02964065 | -2.04157495 | 1.39291455 |
| 1 | -2.65893892 | -2.36292363 | 2.03222853 |
| 1 | -1.26003837 | -3.26023416 | 2.66960310 |
| 1 | -0.70323663 | -5.42080759 | -0.36452064 |
| 1 | 0.14729925 | -3.88219140 | -0.07651399 |
| 1 | -0.10939703 | -5.02752521 | 1.26496368 |
| 1 | 6.06179338 | -1.62017765 | -1.90484402 |
| 1 | 5.88231224 | -0.59206942 | -3.33832331 |
| 1 | 5.22792997 | -2.25272642 | -3.33515944 |
| 1 | 2.54954321 | -1.65660748 | -0.74820995 |
| 1 | 3.23980602 | -2.84736822 | -1.87480540 |
| 1 | 4.19978324 | -2.27115055 | -0.49208622 |
| 1 | 4.87475681 | 1.18340852 | -1.83128003 |
| 1 | 3.52175808 | 0.79482065 | -0.73893244 |
| 1 | 5.13187346 | 0.08373977 | -0.45751730 |
| 1 | -2.30437769 | 2.08624512 | -1.86608704 |
| 31 | -0.32042670 | -0.06987288 | -1.05629067 |

Electronic energy = -3523.874337 a.u.
DFT-D3(BJ) dispersion correction = -0.107208 a.u.
Thermal free energy = 0.595529 a.u.
Gibbs free energy = -3523.386016 a.u.
Number of imaginary frequencies = 0.

**Int2 CL isomer/conformer 17**

| Atomic N. | X | Y | Z |
|---|---|---|---|
| 8 | -1.80364985 | 6.24634352 | -1.11907504 |
| 6 | -2.89337957 | 6.10257124 | -0.17739746 |
| 1 | -3.83024771 | 5.88505277 | -0.70531404 |
| 6 | -0.37704074 | 5.37804591 | -2.80442910 |
| 6 | 0.96300792 | 5.46373779 | -2.03954151 |
| 6 | 1.31727997 | 4.22168656 | -1.20936221 |
| 6 | -1.55993493 | 5.14818635 | -1.88879226 |
| 6 | 1.52996374 | 2.94509613 | -2.02944729 |
| 6 | 1.94106491 | 1.74354787 | -1.17705286 |
| 1 | -0.36694482 | 4.55741912 | -3.53169700 |
| 1 | 0.93290212 | 6.34573494 | -1.38246667 |
| 1 | 2.24001097 | 4.43767323 | -0.64487740 |
| 1 | 2.31810481 | 3.11291191 | -2.78565262 |
| 1 | -0.53694796 | 6.31994606 | -3.34995762 |
| 1 | 1.75710285 | 5.65162843 | -2.78069521 |
| 1 | 0.53839489 | 4.03509963 | -0.45344424 |
| 1 | 0.61111182 | 2.68088285 | -2.57781774 |
| 1 | 2.87980649 | 1.97685001 | -0.63972593 |
| 1 | 2.14680022 | 0.88455555 | -1.83996244 |
| 8 | -2.21826631 | 4.12350537 | -1.81560880 |
| 8 | 0.93492646 | 1.43730313 | -0.20325158 |
| 1 | -2.95476155 | 7.06321017 | 0.34269123 |
| 1 | -2.68178167 | 5.29145770 | 0.53071996 |
| 7 | -1.83278572 | -0.17920488 | -0.30898629 |
| 8 | 0.32617318 | -1.05585583 | 1.54896339 |
| 8 | 0.68457735 | -1.31739820 | -1.52635708 |
| 6 | -2.41110589 | 1.17862555 | -0.13057300 |

| | | | |
|---|---|---|---|
| 6 | -0.62311325 | -0.82763554 | 2.49398279 |
| 6 | -0.24757384 | -0.63798124 | 3.85515417 |
| 6 | -1.27974771 | -0.42148993 | 4.77996653 |
| 6 | -2.62932143 | -0.38652821 | 4.41726536 |
| 6 | -2.98268370 | -0.58647788 | 3.08683388 |
| 6 | -1.99367793 | -0.81900952 | 2.12509767 |
| 6 | -2.38464458 | -1.14529379 | 0.70854787 |
| 1 | -3.39408303 | -0.20990710 | 5.17362136 |
| 6 | 1.22889542 | -0.66508130 | 4.29660526 |
| 6 | -0.16108038 | -2.28686662 | -1.96043717 |
| 6 | 0.35511207 | -3.55017153 | -2.37150533 |
| 6 | -0.56792210 | -4.50463538 | -2.82199776 |
| 6 | -1.94352955 | -4.26269096 | -2.87792640 |
| 6 | -2.43266020 | -3.02240193 | -2.48456625 |
| 6 | -1.55471065 | -2.02748184 | -2.03776986 |
| 6 | -2.09903807 | -0.65882404 | -1.71744034 |
| 1 | -2.62171251 | -5.04008847 | -3.22950146 |
| 6 | 1.86429730 | -3.86094708 | -2.32407868 |
| 6 | 1.37707005 | -0.47671423 | 5.81824796 |
| 6 | 2.00791938 | 0.48328307 | 3.61238543 |
| 6 | 1.86777932 | -2.02803635 | 3.93817098 |
| 6 | 2.17663556 | -5.27617251 | -2.84631217 |
| 6 | 2.37874424 | -3.78552302 | -0.86706024 |
| 6 | 2.64127559 | -2.86187259 | -3.21422702 |
| 1 | -3.50049382 | 1.14185517 | -0.29201959 |
| 1 | -2.20876165 | 1.52743394 | 0.88788716 |
| 1 | -3.48284462 | -1.16013930 | 0.60861874 |
| 1 | -2.00471499 | -2.13645896 | 0.42038513 |
| 1 | -1.02548537 | -0.26778332 | 5.82715783 |
| 1 | -4.03242648 | -0.58203392 | 2.78446080 |
| 1 | -1.65299094 | 0.10469930 | -2.37456119 |
| 1 | -3.18926586 | -0.64518257 | -1.88079202 |
| 1 | -0.20519782 | -5.48122492 | -3.13719724 |
| 1 | -3.50263223 | -2.80804699 | -2.53270696 |
| 1 | 2.44406234 | -0.51555876 | 6.07995336 |
| 1 | 0.99007124 | 0.49560396 | 6.15614969 |
| 1 | 0.86843762 | -1.27034402 | 6.38462708 |
| 1 | 1.98189163 | 0.40188099 | 2.52042327 |
| 1 | 1.58668195 | 1.45900371 | 3.89517101 |
| 1 | 3.05986776 | 0.46232643 | 3.93593084 |
| 1 | 1.34471493 | -2.84735587 | 4.45281391 |
| 1 | 1.83558994 | -2.21801680 | 2.86010016 |
| 1 | 2.91898986 | -2.04151194 | 4.26424149 |
| 1 | 3.26250893 | -5.44101511 | -2.80084949 |
| 1 | 1.86549455 | -5.40944328 | -3.89257680 |
| 1 | 1.69914176 | -6.05781980 | -2.23793457 |
| 1 | 2.23605908 | -2.78902030 | -0.43622959 |
| 1 | 1.85489469 | -4.51442906 | -0.23211460 |
| 1 | 3.45314860 | -4.02267429 | -0.83973498 |
| 1 | 2.31277552 | -2.93993268 | -4.26098439 |
| 1 | 2.49707808 | -1.82883212 | -2.88007267 |
| 1 | 3.71676531 | -3.09327270 | -3.18006701 |
| 1 | -1.96189745 | 1.87451488 | -0.84730989 |

31 0.20192642 -0.21728651 -0.10671527
Electronic energy = -3523.874628 a.u.
DFT-D3(BJ) dispersion correction = -0.110635 a.u.
Thermal free energy = 0.599977 a.u.
Gibbs free energy = -3523.385286 a.u.
Number of imaginary frequencies = 0.

**Int2 CL isomer/conformer 18**

| Atomic N. | X | Y | Z |
|---|---|---|---|
| 7 | 0.80914878 | 1.27155662 | 1.61344911 |
| 8 | -1.58314432 | 0.57207644 | -0.05851005 |
| 8 | 1.27928988 | 0.17573211 | -1.15757250 |
| 6 | 0.89780002 | 0.74440821 | 3.00159129 |
| 6 | -2.26483808 | 1.21389878 | 0.92701308 |
| 6 | -3.67509148 | 1.04510860 | 1.04738791 |
| 6 | -4.30993978 | 1.70465790 | 2.11034335 |
| 6 | -3.62543013 | 2.51925161 | 3.01660402 |
| 6 | -2.25965723 | 2.72280445 | 2.85070710 |
| 6 | -1.57658831 | 2.09320961 | 1.80444271 |
| 6 | -0.13604886 | 2.44167933 | 1.53691073 |
| 1 | -4.16372321 | 3.00326497 | 3.83140173 |
| 6 | -4.48192977 | 0.20655528 | 0.03535790 |
| 6 | 1.81968801 | 1.40410389 | -1.37121487 |
| 6 | 1.97678310 | 1.88710686 | -2.70326632 |
| 6 | 2.56381995 | 3.15055482 | -2.86379915 |
| 6 | 2.98046675 | 3.93489819 | -1.78505109 |
| 6 | 2.81984666 | 3.45200162 | -0.49141705 |
| 6 | 2.25557287 | 2.18950335 | -0.27157780 |
| 6 | 2.19262953 | 1.65567796 | 1.13801963 |
| 1 | 3.42438362 | 4.91479969 | -1.96005706 |
| 6 | 1.51096447 | 1.06733675 | -3.92372830 |
| 6 | -5.98902613 | 0.21620515 | 0.35384202 |
| 6 | -4.01950540 | -1.26828625 | 0.04726449 |
| 6 | -4.30481710 | 0.79765756 | -1.38401243 |
| 6 | 1.81541337 | 1.78791739 | -5.25093762 |
| 6 | -0.01903847 | 0.84680402 | -3.86103257 |
| 6 | 2.23901842 | -0.29703321 | -3.96625427 |
| 1 | 1.30082948 | 1.52582458 | 3.66601819 |
| 1 | -0.10305358 | 0.46522233 | 3.34970734 |
| 1 | 0.20814690 | 3.20822186 | 2.25132878 |
| 1 | -0.02480132 | 2.85248519 | 0.52233764 |
| 1 | -5.38337378 | 1.58135386 | 2.24111083 |
| 1 | -1.71276603 | 3.39048714 | 3.52030345 |
| 1 | 2.80373109 | 0.74641694 | 1.25145077 |
| 1 | 2.58821628 | 2.41359221 | 1.83438185 |
| 1 | 2.69514484 | 3.54780383 | -3.86850277 |
| 1 | 3.14503378 | 4.04644855 | 0.36527799 |
| 1 | -6.51778094 | -0.37645282 | -0.40609136 |
| 1 | -6.20893085 | -0.23193199 | 1.33382648 |
| 1 | -6.41013758 | 1.23158684 | 0.33277516 |
| 1 | -2.97149173 | -1.36162476 | -0.25530504 |
| 1 | -4.13734136 | -1.70698220 | 1.04900451 |

| | | | |
|---|---|---|---|
| 1 | -4.63351363 | -1.85499445 | -0.65283087 |
| 1 | -4.67494235 | 1.83248517 | -1.42133116 |
| 1 | -3.25377591 | 0.79215153 | -1.69378583 |
| 1 | -4.88408288 | 0.20533157 | -2.10862315 |
| 1 | 1.47197873 | 1.15922207 | -6.08468068 |
| 1 | 2.89214567 | 1.96293694 | -5.39003577 |
| 1 | 1.29268274 | 2.75201610 | -5.32892423 |
| 1 | -0.31372266 | 0.29340598 | -2.96318673 |
| 1 | -0.55014464 | 1.80947871 | -3.86362806 |
| 1 | -0.34894638 | 0.27557413 | -4.74207482 |
| 1 | 3.32751978 | -0.15357644 | -4.03619745 |
| 1 | 2.01885540 | -0.89354862 | -3.07462116 |
| 1 | 1.91560902 | -0.86351761 | -4.85268703 |
| 1 | 1.55841370 | -0.13118058 | 3.02315728 |
| 8 | 3.74465818 | -1.50217221 | 0.97388620 |
| 6 | 3.98592347 | -1.82732235 | -0.41413954 |
| 1 | 4.51168056 | -0.95993460 | -0.82610679 |
| 6 | 2.81604706 | -3.77533028 | 1.23227118 |
| 6 | 2.05539026 | -4.70978454 | 2.18382830 |
| 6 | 0.51922776 | -4.65165114 | 2.09972061 |
| 6 | 3.14109283 | -2.41072196 | 1.80682605 |
| 6 | -0.17072777 | -3.38656166 | 2.66504268 |
| 6 | -0.74071825 | -2.42969357 | 1.61924104 |
| 1 | 2.25406999 | -3.63450413 | 0.29678526 |
| 1 | 2.37785447 | -4.51050923 | 3.21669761 |
| 1 | 0.21176118 | -4.79800522 | 1.04988772 |
| 1 | 0.52048242 | -2.83853610 | 3.32238304 |
| 1 | 3.77785685 | -4.23370003 | 0.94397853 |
| 1 | 2.36116324 | -5.74174363 | 1.95136633 |
| 1 | 0.14032120 | -5.52977257 | 2.64545617 |
| 1 | -1.02447702 | -3.69336618 | 3.29228608 |
| 1 | -1.35500209 | -1.66838999 | 2.13605036 |
| 1 | -1.42487267 | -2.98685893 | 0.95142643 |
| 8 | 2.94863873 | -2.06892113 | 2.95853631 |
| 8 | 0.29668919 | -1.82532561 | 0.83132608 |
| 1 | 3.04066755 | -1.96362892 | -0.95412600 |
| 1 | 4.62194771 | -2.71775548 | -0.51104592 |
| 31 | 0.12477620 | -0.10749834 | 0.26434415 |

Electronic energy = -3523.864240 a.u.
DFT-D3(BJ) dispersion correction = -0.117301 a.u.
Thermal free energy = 0.601064 a.u.
Gibbs free energy = -3523.380477 a.u.
Number of imaginary frequencies = 0.

**Int2 CL isomer/conformer 19**

| Atomic N. | X | Y | Z |
|---|---|---|---|
| 7 | 0.82418648 | 1.26419329 | 1.61347122 |
| 8 | -1.57695360 | 0.57691752 | -0.05107765 |
| 8 | 1.27926698 | 0.17393899 | -1.16243935 |
| 6 | 0.92182280 | 0.73217155 | 2.99919201 |
| 6 | -2.25347917 | 1.21788182 | 0.93851457 |
| 6 | -3.66419708 | 1.05488608 | 1.06100872 |

| | | | |
|---|---|---|---|
| 6 | -4.29402194 | 1.71292688 | 2.12787351 |
| 6 | -3.60402835 | 2.52060046 | 3.03615749 |
| 6 | -2.23768038 | 2.71876372 | 2.86835801 |
| 6 | -1.55945544 | 2.09059418 | 1.81808251 |
| 6 | -0.11886485 | 2.43657986 | 1.54719772 |
| 1 | -4.13853411 | 3.00369644 | 3.85398746 |
| 6 | -4.47656912 | 0.22335697 | 0.04760395 |
| 6 | 1.81522679 | 1.40417850 | -1.37744124 |
| 6 | 1.96265402 | 1.89057904 | -2.70917930 |
| 6 | 2.54440448 | 3.15649321 | -2.87015481 |
| 6 | 2.96546000 | 3.93935930 | -1.79199036 |
| 6 | 2.81542352 | 3.45248091 | -0.49847657 |
| 6 | 2.25626582 | 2.18781419 | -0.27860707 |
| 6 | 2.20518957 | 1.64594433 | 1.12832492 |
| 1 | 3.40511316 | 4.92111652 | -1.96739208 |
| 6 | 1.49382966 | 1.07121339 | -3.92872910 |
| 6 | -5.98326126 | 0.24022647 | 0.36773745 |
| 6 | -4.02194335 | -1.25415469 | 0.05606228 |
| 6 | -4.29819816 | 0.81670364 | -1.37065493 |
| 6 | 1.78689126 | 1.79636996 | -5.25601068 |
| 6 | -0.03449585 | 0.84181214 | -3.85813330 |
| 6 | 2.22936441 | -0.28892525 | -3.97823503 |
| 1 | 1.33153765 | 1.51045085 | 3.66321187 |
| 1 | -0.07720479 | 0.45433893 | 3.35365319 |
| 1 | 0.22980485 | 3.19872113 | 2.26415352 |
| 1 | -0.01086613 | 2.85242427 | 0.53435103 |
| 1 | -5.36776160 | 1.59396004 | 2.26017558 |
| 1 | -1.68660975 | 3.38158739 | 3.53935547 |
| 1 | 2.81238737 | 0.73223233 | 1.22450356 |
| 1 | 2.61190610 | 2.39609003 | 1.82649020 |
| 1 | 2.66815483 | 3.55673414 | -3.87463365 |
| 1 | 3.14503747 | 4.04538462 | 0.35759403 |
| 1 | -6.51600267 | -0.34697354 | -0.39365457 |
| 1 | -6.20470181 | -0.20997371 | 1.34642171 |
| 1 | -6.39875985 | 1.25799180 | 0.35047314 |
| 1 | -2.97541558 | -1.35279911 | -0.24943267 |
| 1 | -4.13989183 | -1.69393284 | 1.05723082 |
| 1 | -4.64120808 | -1.83704108 | -0.64276134 |
| 1 | -4.66364499 | 1.85329349 | -1.40526102 |
| 1 | -3.24760257 | 0.80703645 | -1.68174422 |
| 1 | -4.88107980 | 0.22866478 | -2.09586091 |
| 1 | 1.44222284 | 1.16765637 | -6.08923114 |
| 1 | 2.86186561 | 1.97747261 | -5.40078604 |
| 1 | 1.25854269 | 2.75780679 | -5.32884373 |
| 1 | -0.32069733 | 0.28285279 | -2.96101212 |
| 1 | -0.57111881 | 1.80140162 | -3.85352236 |
| 1 | -0.36618096 | 0.27241059 | -4.73969259 |
| 1 | 3.31653500 | -0.13908352 | -4.05498614 |
| 1 | 2.01857959 | -0.88839573 | -3.08623991 |
| 1 | 1.90347819 | -0.85576299 | -4.86353303 |
| 1 | 1.58034098 | -0.14511875 | 3.01335875 |
| 8 | 3.74649204 | -1.52121971 | 0.96295231 |
| 6 | 3.98056470 | -1.84522641 | -0.42669657 |

| | | | |
|---|---|---|---|
| 1 | 4.51327574 | -0.98179378 | -0.83805545 |
| 6 | 2.80960428 | -3.79114712 | 1.22202983 |
| 6 | 2.04691598 | -4.72271337 | 2.17477830 |
| 6 | 0.51083593 | -4.65709489 | 2.09534043 |
| 6 | 3.14174242 | -2.42834055 | 1.79673361 |
| 6 | -0.17161977 | -3.38847178 | 2.66185735 |
| 6 | -0.73999160 | -2.42943664 | 1.61716094 |
| 1 | 2.24670301 | -3.64777894 | 0.28750303 |
| 1 | 2.37348845 | -4.52634857 | 3.20691901 |
| 1 | 0.19944567 | -4.80270559 | 1.04652726 |
| 1 | 0.52365745 | -2.84349436 | 3.31741917 |
| 1 | 3.76916563 | -4.25345249 | 0.93240830 |
| 1 | 2.34678039 | -5.75594578 | 1.94021378 |
| 1 | 0.12943761 | -5.53311092 | 2.64271957 |
| 1 | -1.02530143 | -3.69106874 | 3.29123143 |
| 1 | -1.35037530 | -1.66583453 | 2.13516307 |
| 1 | -1.42776106 | -2.98410006 | 0.95095194 |
| 8 | 2.95463744 | -2.08720619 | 2.94951729 |
| 8 | 0.29789476 | -1.82892985 | 0.82665914 |
| 1 | 3.03220437 | -1.97103589 | -0.96386061 |
| 1 | 4.60754150 | -2.74143455 | -0.52806487 |
| 31 | 0.12959891 | -0.10925847 | 0.26376396 |

Electronic energy = -3523.864275 a.u.
DFT-D3(BJ) dispersion correction = -0.117292 a.u.
Thermal free energy = 0.601544 a.u.
Gibbs free energy = -3523.380023 a.u.
Number of imaginary frequencies = 0.

**Int2 CL isomer/conformer 20**

| Atomic N. | X | Y | Z |
|---|---|---|---|
| 8 | -4.29531803 | 1.69720832 | 1.19677731 |
| 6 | -4.07840682 | 1.00850706 | 2.44831005 |
| 1 | -3.95046146 | 1.80003832 | 3.19359026 |
| 6 | -4.35485846 | -0.52488868 | 0.11077274 |
| 6 | -4.64426044 | -1.22345994 | -1.22074610 |
| 6 | -4.22005356 | -2.70621751 | -1.23124818 |
| 6 | -4.43189096 | 0.98552374 | 0.03047203 |
| 6 | -2.78879227 | -3.00084174 | -1.71530899 |
| 6 | -1.62737659 | -2.40957152 | -0.91345280 |
| 1 | -3.33331278 | -0.76269689 | 0.45011305 |
| 1 | -4.13441085 | -0.68229624 | -2.02817832 |
| 1 | -4.36280327 | -3.14228991 | -0.22670445 |
| 1 | -2.67532716 | -2.66170646 | -2.75848003 |
| 1 | -5.03133704 | -0.89324887 | 0.89866780 |
| 1 | -5.72209833 | -1.14290422 | -1.42814661 |
| 1 | -4.90373512 | -3.26154395 | -1.89268575 |
| 1 | -2.65132734 | -4.09569968 | -1.72742274 |
| 1 | -0.70102926 | -2.94087562 | -1.19795631 |
| 1 | -1.78630265 | -2.59307762 | 0.16881651 |
| 8 | -4.55616865 | 1.61890878 | -1.00092333 |
| 8 | -1.50225795 | -1.00785455 | -1.18445105 |
| 1 | -3.17058233 | 0.39177640 | 2.41119664 |

| | | | |
|---|---|---|---|
| 1 | -4.94715923 | 0.39250332 | 2.71992933 |
| 7 | -0.37068603 | 1.89919955 | -1.19909972 |
| 8 | 1.59239000 | -0.34258852 | -0.79839421 |
| 8 | -0.49397997 | 0.33755355 | 1.37386634 |
| 6 | -1.22453714 | 1.96530095 | -2.41689443 |
| 6 | 2.12151261 | 0.11930190 | -1.96532230 |
| 6 | 3.01942661 | -0.68937066 | -2.71946197 |
| 6 | 3.50579841 | -0.15505345 | -3.92211467 |
| 6 | 3.15932104 | 1.11777791 | -4.38330682 |
| 6 | 2.31996329 | 1.91538299 | -3.61285157 |
| 6 | 1.81227277 | 1.43484973 | -2.40110526 |
| 6 | 1.03595260 | 2.35581352 | -1.49759618 |
| 1 | 3.55774679 | 1.48355257 | -5.32954587 |
| 6 | 3.46358038 | -2.08246839 | -2.23119581 |
| 6 | -0.05513394 | 1.50639558 | 1.91670050 |
| 6 | 0.55690639 | 1.51229863 | 3.20318582 |
| 6 | 0.96147416 | 2.75388208 | 3.71570813 |
| 6 | 0.79421205 | 3.95235100 | 3.01715592 |
| 6 | 0.19543048 | 3.93156583 | 1.76250721 |
| 6 | -0.24255268 | 2.72278230 | 1.20860668 |
| 6 | -0.98482290 | 2.74614276 | -0.10468703 |
| 1 | 1.13184687 | 4.89183603 | 3.45445628 |
| 6 | 0.78262405 | 0.21191733 | 4.00130175 |
| 6 | 4.46544042 | -2.74007909 | -3.19893283 |
| 6 | 2.24749395 | -3.02907356 | -2.11476579 |
| 6 | 4.16261532 | -1.95524603 | -0.85649278 |
| 6 | 1.43055736 | 0.48349181 | 5.37240391 |
| 6 | 1.73179121 | -0.72943752 | 3.22292927 |
| 6 | -0.56523786 | -0.49960553 | 4.26619642 |
| 1 | -1.28451513 | 3.00798094 | -2.76701814 |
| 1 | -0.78422126 | 1.34216351 | -3.20254675 |
| 1 | 0.97960885 | 3.36452257 | -1.93901703 |
| 1 | 1.53575301 | 2.43971856 | -0.52124823 |
| 1 | 4.18192530 | -0.75409323 | -4.52927295 |
| 1 | 2.06739250 | 2.92824486 | -3.93451577 |
| 1 | -2.01487259 | 2.38049492 | 0.01682020 |
| 1 | -1.04614190 | 3.78178802 | -0.47672512 |
| 1 | 1.43491616 | 2.79205015 | 4.69493818 |
| 1 | 0.04731019 | 4.85860498 | 1.20441413 |
| 1 | 4.76127809 | -3.71872213 | -2.79504796 |
| 1 | 4.03002673 | -2.91143674 | -4.19413580 |
| 1 | 5.37998810 | -2.14138695 | -3.31710677 |
| 1 | 1.53086940 | -2.66268200 | -1.37233719 |
| 1 | 1.73272099 | -3.12621029 | -3.08174960 |
| 1 | 2.58228546 | -4.03089380 | -1.80558754 |
| 1 | 5.05815724 | -1.32199164 | -0.93534890 |
| 1 | 3.49478148 | -1.52108395 | -0.10405134 |
| 1 | 4.48156437 | -2.94925401 | -0.50760037 |
| 1 | 1.55958051 | -0.47147045 | 5.90128788 |
| 1 | 0.80557653 | 1.13043036 | 6.00496973 |
| 1 | 2.42437725 | 0.94391406 | 5.27758171 |
| 1 | 1.31667938 | -1.01176979 | 2.24967058 |
| 1 | 2.70507772 | -0.24678815 | 3.05492297 |

| | | | |
|---|---|---|---|
| 1 | 1.90460734 | -1.64782393 | 3.80435844 |
| 1 | -1.23952093 | 0.15006388 | 4.84383338 |
| 1 | -1.06087292 | -0.77799031 | 3.32970236 |
| 1 | -0.39367419 | -1.41402962 | 4.85409773 |
| 1 | -2.23102935 | 1.60238478 | -2.18016878 |
| 31 | -0.20176400 | 0.02149959 | -0.43931115 |

Electronic energy = -3523.863566 a.u.
DFT-D3(BJ) dispersion correction = -0.116124 a.u.
Thermal free energy = 0.599743 a.u.
Gibbs free energy = -3523.379947 a.u.
Number of imaginary frequencies = 0.

**Int2 CL isomer/conformer 21**

| Atomic N. | X | Y | Z |
|---|---|---|---|
| 7 | -0.03056698 | 1.81530188 | 1.34175524 |
| 8 | -1.15227584 | -0.34038584 | -0.40773807 |
| 8 | 0.94221782 | 1.62564829 | -1.52034654 |
| 6 | 0.56602992 | 1.50527757 | 2.66898988 |
| 6 | -1.94721913 | -0.57949289 | 0.66890579 |
| 6 | -2.63032299 | -1.82333764 | 0.79657960 |
| 6 | -3.42930382 | -2.00067058 | 1.93633254 |
| 6 | -3.57530088 | -1.01912717 | 2.92085431 |
| 6 | -2.92615209 | 0.20204322 | 2.76847083 |
| 6 | -2.12745696 | 0.43709990 | 1.64434508 |
| 6 | -1.53803728 | 1.80409719 | 1.41108076 |
| 1 | -4.20181042 | -1.20802283 | 3.79253985 |
| 6 | -2.51348429 | -2.92345768 | -0.27723958 |
| 6 | 0.27786493 | 2.80462526 | -1.65231319 |
| 6 | -0.08839124 | 3.27732219 | -2.94516515 |
| 6 | -0.76412406 | 4.50434160 | -3.01071670 |
| 6 | -1.08051691 | 5.25833528 | -1.87704482 |
| 6 | -0.70286358 | 4.79308920 | -0.62275896 |
| 6 | -0.01490783 | 3.57980979 | -0.49958085 |
| 6 | 0.48514512 | 3.15378381 | 0.85722695 |
| 1 | -1.61402481 | 6.20304897 | -1.98015767 |
| 6 | 0.24204041 | 2.47916704 | -4.22187850 |
| 6 | -3.37655581 | -4.15324439 | 0.06229572 |
| 6 | -1.04940364 | -3.40791364 | -0.39560009 |
| 6 | -3.00017278 | -2.38072112 | -1.64227994 |
| 6 | -0.22516589 | 3.21111303 | -5.49421686 |
| 6 | -0.47106523 | 1.10665594 | -4.19382853 |
| 6 | 1.77188938 | 2.28001194 | -4.34150000 |
| 1 | 0.33992424 | 2.31695155 | 3.37799845 |
| 1 | 0.14145245 | 0.57135288 | 3.05112123 |
| 1 | -1.85080168 | 2.49702678 | 2.20936154 |
| 1 | -1.88507292 | 2.21170214 | 0.45031697 |
| 1 | -3.95994060 | -2.94216384 | 2.06717754 |
| 1 | -3.04927833 | 0.99174597 | 3.51279013 |
| 1 | 1.58267375 | 3.05807324 | 0.85489419 |
| 1 | 0.21402958 | 3.91274343 | 1.60910139 |
| 1 | -1.06400438 | 4.88953806 | -3.98349101 |
| 1 | -0.92492035 | 5.37538376 | 0.27426788 |

| | | | |
|---|---|---|---|
| 1 | -3.26756479 | -4.89807863 | -0.73868917 |
| 1 | -3.06666238 | -4.63253506 | 1.00246863 |
| 1 | -4.44411883 | -3.90100029 | 0.13590701 |
| 1 | -0.37699636 | -2.59242810 | -0.68266567 |
| 1 | -0.70038197 | -3.83037649 | 0.55846506 |
| 1 | -0.97899276 | -4.19778744 | -1.15862440 |
| 1 | -4.05384259 | -2.07206644 | -1.58142953 |
| 1 | -2.40467898 | -1.52195815 | -1.97059073 |
| 1 | -2.92365548 | -3.17068503 | -2.40478617 |
| 1 | 0.04598626 | 2.60783409 | -6.37216621 |
| 1 | 0.25601976 | 4.19336701 | -5.60698402 |
| 1 | -1.31548964 | 3.35204256 | -5.51550914 |
| 1 | -0.14845519 | 0.49815075 | -3.34250747 |
| 1 | -1.56122038 | 1.23577867 | -4.13245546 |
| 1 | -0.24683202 | 0.55321716 | -5.11829063 |
| 1 | 2.28441331 | 3.25067249 | -4.40962091 |
| 1 | 2.17569056 | 1.73358623 | -3.48233133 |
| 1 | 2.00175131 | 1.71267705 | -5.25615106 |
| 1 | 1.65106019 | 1.39300959 | 2.57003417 |
| 8 | 0.75249021 | -1.76830026 | 4.72641010 |
| 6 | -0.01409827 | -2.78300308 | 4.04540795 |
| 1 | -0.98007632 | -2.81037971 | 4.55962893 |
| 6 | 2.52559337 | -2.13799104 | 3.03986988 |
| 6 | 3.96245074 | -1.74738683 | 2.68679351 |
| 6 | 4.42955079 | -2.24659052 | 1.30441572 |
| 6 | 2.00875948 | -1.43172915 | 4.27594257 |
| 6 | 4.15705407 | -1.32148434 | 0.10233347 |
| 6 | 2.72523216 | -1.21920302 | -0.42388397 |
| 1 | 1.85526490 | -1.86911604 | 2.20483672 |
| 1 | 4.06064125 | -0.65391956 | 2.74017090 |
| 1 | 4.00733166 | -3.24644666 | 1.09980882 |
| 1 | 4.51568299 | -0.30395870 | 0.33175724 |
| 1 | 2.44041821 | -3.22898161 | 3.17164755 |
| 1 | 4.63219334 | -2.14475196 | 3.46439244 |
| 1 | 5.52050825 | -2.39162481 | 1.35093191 |
| 1 | 4.76792095 | -1.68023814 | -0.74446062 |
| 1 | 2.75690509 | -0.70855843 | -1.40365468 |
| 1 | 2.32034444 | -2.23547065 | -0.59785366 |
| 8 | 2.60513100 | -0.55914466 | 4.87535787 |
| 8 | 1.87301516 | -0.51296397 | 0.49229554 |
| 1 | -0.17627081 | -2.52750144 | 2.98942341 |
| 1 | 0.46828379 | -3.76801839 | 4.12277835 |
| 31 | 0.48951667 | 0.49659067 | -0.11897234 |

Electronic energy = -3523.862957 a.u.
DFT-D3(BJ) dispersion correction = -0.115228 a.u.
Thermal free energy = 0.598564 a.u.
Gibbs free energy = -3523.379622 a.u.
Number of imaginary frequencies = 0.

**Int2_CL isomer/conformer 22**

| Atomic N. | X | Y | Z |
|---|---|---|---|
| 7 | 0.71358871 | 1.11038700 | 1.68842561 |

| | | | |
|---|---|---|---|
| 8 | -1.75688397 | -0.11286227 | 0.49969090 |
| 8 | 0.63066929 | 0.71592215 | -1.29788788 |
| 6 | 1.39637311 | 0.43924041 | 2.82619549 |
| 6 | -2.25549938 | 0.05457739 | 1.75300405 |
| 6 | -3.43887791 | -0.63332801 | 2.14993957 |
| 6 | -3.87675656 | -0.44466874 | 3.46926477 |
| 6 | -3.21959594 | 0.39232101 | 4.37549555 |
| 6 | -2.10054990 | 1.10379569 | 3.95593633 |
| 6 | -1.62354530 | 0.95712238 | 2.64880095 |
| 6 | -0.51526892 | 1.84744497 | 2.15209265 |
| 1 | -3.59545686 | 0.49876093 | 5.39301960 |
| 6 | -4.23073401 | -1.51862004 | 1.16645349 |
| 6 | 0.66878836 | 2.07268315 | -1.31855020 |
| 6 | 0.27274497 | 2.77568313 | -2.49340041 |
| 6 | 0.34353557 | 4.17575888 | -2.45862457 |
| 6 | 0.77798193 | 4.88443406 | -1.33498245 |
| 6 | 1.17414877 | 4.18517562 | -0.20033724 |
| 6 | 1.13821083 | 2.78557126 | -0.18415638 |
| 6 | 1.68814048 | 2.05007676 | 1.01175746 |
| 1 | 0.80733970 | 5.97381633 | -1.35312423 |
| 6 | -0.21584138 | 2.03261314 | -3.75283173 |
| 6 | -5.49531648 | -2.11073297 | 1.81685936 |
| 6 | -3.36863536 | -2.70410917 | 0.67592726 |
| 6 | -4.68746584 | -0.66834585 | -0.04340394 |
| 6 | -0.55127757 | 3.00440447 | -4.90000377 |
| 6 | -1.50205386 | 1.23338780 | -3.43637290 |
| 6 | 0.88695305 | 1.07686800 | -4.26801608 |
| 1 | 1.72565640 | 1.19557259 | 3.55684019 |
| 1 | 0.69837859 | -0.24810781 | 3.31722084 |
| 1 | -0.21283993 | 2.55543745 | 2.94188391 |
| 1 | -0.85722034 | 2.43381964 | 1.28621174 |
| 1 | -4.76743423 | -0.96994972 | 3.80910600 |
| 1 | -1.59828536 | 1.79813696 | 4.63335023 |
| 1 | 2.55062551 | 1.42662836 | 0.72898389 |
| 1 | 2.03245824 | 2.77647426 | 1.76622878 |
| 1 | 0.04158228 | 4.74174913 | -3.33795980 |
| 1 | 1.53235124 | 4.71960489 | 0.68235557 |
| 1 | -6.03143174 | -2.71363176 | 1.07023863 |
| 1 | -5.25697596 | -2.77015723 | 2.66416732 |
| 1 | -6.18467866 | -1.32918983 | 2.16721900 |
| 1 | -2.48785462 | -2.35766287 | 0.12467890 |
| 1 | -3.03601449 | -3.32098340 | 1.52376808 |
| 1 | -3.96294366 | -3.34399346 | 0.00613081 |
| 1 | -5.34200706 | 0.15174087 | 0.28564977 |
| 1 | -3.83333650 | -0.23887226 | -0.57875834 |
| 1 | -5.25824016 | -1.29568881 | -0.74490851 |
| 1 | -0.88040073 | 2.42457480 | -5.77401472 |
| 1 | 0.32085516 | 3.59979474 | -5.20698870 |
| 1 | -1.36700011 | 3.69251090 | -4.63526539 |
| 1 | -1.33011133 | 0.47674232 | -2.66375098 |
| 1 | -2.30121978 | 1.90525319 | -3.09167497 |
| 1 | -1.85616921 | 0.72396213 | -4.34549023 |
| 1 | 1.79284612 | 1.64166814 | -4.53446154 |

| | | | |
|---|---|---|---|
| 1 | 1.14852283 | 0.32554708 | -3.51484729 |
| 1 | 0.53584139 | 0.55699280 | -5.17236757 |
| 1 | 2.27332118 | -0.10753469 | 2.45895363 |
| 8 | 4.35949288 | 0.11313473 | -0.30543265 |
| 6 | 4.39452172 | -0.01376604 | -1.74513323 |
| 1 | 4.07867658 | 0.96337422 | -2.12449516 |
| 6 | 4.96347780 | -2.29644702 | -0.11466692 |
| 6 | 3.67146820 | -3.12514055 | -0.32837396 |
| 6 | 3.06286277 | -3.66413690 | 0.97785948 |
| 6 | 4.63913433 | -0.95391633 | 0.50993797 |
| 6 | 1.57708691 | -4.04240811 | 0.84044200 |
| 6 | 0.62526728 | -2.87139401 | 1.09507780 |
| 1 | 5.63332905 | -2.81644819 | 0.58356422 |
| 1 | 2.91791039 | -2.51260042 | -0.84163795 |
| 1 | 3.64732353 | -4.53839137 | 1.30697480 |
| 1 | 1.38109936 | -4.46132657 | -0.16072105 |
| 1 | 5.49340775 | -2.17570236 | -1.06874633 |
| 1 | 3.90934083 | -3.96672646 | -0.99810309 |
| 1 | 3.16773350 | -2.91092446 | 1.77438431 |
| 1 | 1.31742231 | -4.83037331 | 1.56660479 |
| 1 | -0.41783998 | -3.21284970 | 0.97074302 |
| 1 | 0.74282294 | -2.55201877 | 2.15001160 |
| 8 | 4.56467469 | -0.77009909 | 1.71193471 |
| 8 | 0.92444882 | -1.79480646 | 0.20297651 |
| 1 | 3.69224728 | -0.77875414 | -2.09908036 |
| 1 | 5.41256281 | -0.23141972 | -2.09638626 |
| 31 | 0.08853764 | -0.18467486 | 0.22859521 |

Electronic energy = -3523.863701 a.u.
DFT-D3(BJ) dispersion correction = -0.115506 a.u.
Thermal free energy = 0.601088 a.u.
Gibbs free energy = -3523.378119 a.u.
Number of imaginary frequencies = 0.

**Int2_CL isomer/conformer 23**

| Atomic N. | X | Y | Z |
|---|---|---|---|
| 7 | -0.15541265 | 1.82211816 | 1.13472142 |
| 8 | -1.57640776 | 0.09668474 | -0.87165107 |
| 8 | 1.49924227 | 0.10532916 | -0.70283865 |
| 6 | -0.59428609 | 1.79506124 | 2.55429318 |
| 6 | -2.66263449 | 0.81290128 | -0.47736771 |
| 6 | -3.97759338 | 0.33780226 | -0.75450193 |
| 6 | -5.05089564 | 1.12418935 | -0.31033635 |
| 6 | -4.87748203 | 2.33313301 | 0.36900791 |
| 6 | -3.58943703 | 2.80350727 | 0.60089028 |
| 6 | -2.48260255 | 2.06376152 | 0.17071788 |
| 6 | -1.10081740 | 2.64741377 | 0.29704126 |
| 1 | -5.74499370 | 2.90426894 | 0.69945634 |
| 6 | -4.21998713 | -0.97761323 | -1.52060079 |
| 6 | 1.93821202 | 1.30753370 | -1.16202015 |
| 6 | 2.55811221 | 1.39839838 | -2.44192836 |
| 6 | 3.00443985 | 2.66347045 | -2.85067956 |
| 6 | 2.86201523 | 3.80860039 | -2.06260569 |

| | | | |
|---|---|---|---|
| 6 | 2.26291664 | 3.70362888 | -0.81255546 |
| 6 | 1.81018842 | 2.46310382 | -0.34784107 |
| 6 | 1.25868129 | 2.36328081 | 1.05170753 |
| 1 | 3.22011438 | 4.77142461 | -2.42677865 |
| 6 | 2.73803203 | 0.16210039 | -3.34550974 |
| 6 | -5.72011741 | -1.25068962 | -1.73895240 |
| 6 | -3.64647846 | -2.17187534 | -0.72428963 |
| 6 | -3.55655301 | -0.90525873 | -2.91669307 |
| 6 | 3.44634958 | 0.51313076 | -4.66781435 |
| 6 | 1.36063924 | -0.43972186 | -3.71086721 |
| 6 | 3.60834993 | -0.89613575 | -2.62699412 |
| 1 | -0.60551389 | 2.82034836 | 2.95689204 |
| 1 | -1.60067772 | 1.36782595 | 2.61841458 |
| 1 | -1.15159889 | 3.65748098 | 0.73612111 |
| 1 | -0.62737184 | 2.73261933 | -0.69204077 |
| 1 | -6.06715550 | 0.78213557 | -0.49731112 |
| 1 | -3.43021825 | 3.76200741 | 1.10015201 |
| 1 | 1.87109911 | 1.69694452 | 1.67957199 |
| 1 | 1.26295885 | 3.36001505 | 1.52200558 |
| 1 | 3.47787854 | 2.76634007 | -3.82526082 |
| 1 | 2.15381757 | 4.58379169 | -0.17509056 |
| 1 | -5.83350033 | -2.18829873 | -2.30157147 |
| 1 | -6.26362873 | -1.36764934 | -0.79012691 |
| 1 | -6.20451946 | -0.45438051 | -2.32220560 |
| 1 | -2.56614407 | -2.06932869 | -0.57799100 |
| 1 | -4.12567899 | -2.24867411 | 0.26252937 |
| 1 | -3.83518307 | -3.10984592 | -1.26842028 |
| 1 | -3.98990125 | -0.08544221 | -3.50786412 |
| 1 | -2.47541897 | -0.74547223 | -2.84177897 |
| 1 | -3.73420978 | -1.84474254 | -3.46198325 |
| 1 | 3.55594617 | -0.40206536 | -5.26690384 |
| 1 | 4.45348106 | 0.92221184 | -4.50236180 |
| 1 | 2.87138699 | 1.23319901 | -5.26783139 |
| 1 | 0.80986317 | -0.76514072 | -2.82229436 |
| 1 | 0.74530390 | 0.29708811 | -4.24702073 |
| 1 | 1.49872178 | -1.30980624 | -4.37063548 |
| 1 | 4.60867869 | -0.49307204 | -2.41192024 |
| 1 | 3.15438098 | -1.21590584 | -1.68290468 |
| 1 | 3.73088285 | -1.77872427 | -3.27315831 |
| 1 | 0.10509411 | 1.18806677 | 3.13716499 |
| 8 | 1.61496349 | -0.02215731 | 5.63231190 |
| 6 | 0.95136088 | -1.06517480 | 6.37441068 |
| 1 | 0.55240291 | -0.57399961 | 7.26801577 |
| 6 | 1.99343366 | -1.68263472 | 3.83433143 |
| 6 | 2.85267846 | -1.94679305 | 2.59433016 |
| 6 | 2.47372245 | -3.25018362 | 1.86255262 |
| 6 | 2.15589571 | -0.28667058 | 4.39711078 |
| 6 | 1.39977585 | -3.13516607 | 0.76580603 |
| 6 | -0.00629014 | -2.68983070 | 1.18474119 |
| 1 | 0.92457970 | -1.77835364 | 3.57398954 |
| 1 | 2.77574237 | -1.09382346 | 1.90739306 |
| 1 | 2.16216570 | -4.01277721 | 2.59862638 |
| 1 | 1.75959732 | -2.45793063 | -0.02726143 |

| | | | |
|---|---|---|---|
| 1 | 2.19724997 | -2.43584242 | 4.61095825 |
| 1 | 3.90688740 | -1.99276188 | 2.90774374 |
| 1 | 3.37936536 | -3.65547632 | 1.38462299 |
| 1 | 1.29145802 | -4.12727446 | 0.29336435 |
| 1 | -0.71219410 | -2.94205467 | 0.37173436 |
| 1 | -0.33136678 | -3.26907725 | 2.06854499 |
| 8 | 2.68501904 | 0.63838932 | 3.81033354 |
| 8 | -0.11851586 | -1.30425583 | 1.53944501 |
| 1 | 0.12526185 | -1.50812344 | 5.80097569 |
| 1 | 1.65733338 | -1.85055758 | 6.67932441 |
| 31 | -0.09089404 | -0.00854437 | 0.25540874 |

Electronic energy = -3523.861613 a.u.
DFT-D3(BJ) dispersion correction = -0.114972 a.u.
Thermal free energy = 0.598663 a.u.
Gibbs free energy = -3523.377921 a.u.
Number of imaginary frequencies = 0.

**Int2 CL isomer/conformer 24**

| Atomic N. | X | Y | Z |
|---|---|---|---|
| 8 | -0.48437096 | -5.90449092 | -3.14671556 |
| 6 | -0.26021849 | -7.28587548 | -2.80773256 |
| 1 | -1.00060799 | -7.84923709 | -3.38533817 |
| 1 | -0.42438866 | -7.46944408 | -1.73613095 |
| 1 | 0.74843526 | -7.61768876 | -3.09212354 |
| 6 | 0.34145396 | -4.92349026 | -2.65154795 |
| 8 | 0.11564869 | -3.77697928 | -2.97422311 |
| 7 | -0.88014359 | 1.48507391 | 2.79464797 |
| 8 | -1.23422909 | 0.80088148 | -0.08181420 |
| 8 | 1.61072193 | 1.30711702 | 1.11407555 |
| 6 | -1.55101741 | 0.60855620 | 3.78667904 |
| 6 | -2.55056616 | 0.98207774 | 0.21004673 |
| 6 | -3.55260480 | 0.42903234 | -0.63857225 |
| 6 | -4.88949804 | 0.67231333 | -0.29036925 |
| 6 | -5.25993153 | 1.41629840 | 0.83366543 |
| 6 | -4.27111648 | 1.95779442 | 1.64780103 |
| 6 | -2.92081749 | 1.75834797 | 1.33876258 |
| 6 | -1.85866356 | 2.43975332 | 2.15859313 |
| 1 | -6.31409486 | 1.57160921 | 1.06348555 |
| 6 | -3.19033018 | -0.41049116 | -1.87867652 |
| 6 | 1.71253575 | 2.62634689 | 1.41710413 |
| 6 | 2.53281682 | 3.47959275 | 0.62315484 |
| 6 | 2.60722990 | 4.82833045 | 0.99919508 |
| 6 | 1.91898199 | 5.34937346 | 2.09881982 |
| 6 | 1.13099825 | 4.50404398 | 2.87127242 |
| 6 | 1.03070026 | 3.14537922 | 2.54849910 |
| 6 | 0.25477316 | 2.22839654 | 3.45807514 |
| 1 | 2.00360844 | 6.40759076 | 2.34553496 |
| 6 | 3.30573335 | 2.94868137 | -0.60028258 |
| 6 | -4.44257249 | -0.85883825 | -2.65597508 |
| 6 | -2.43492101 | -1.68952895 | -1.44781202 |
| 6 | -2.32065305 | 0.41895556 | -2.85359442 |
| 6 | 4.14229757 | 4.05248087 | -1.27468996 |

| | | | |
|---|---|---|---|
| 6 | 2.31792194 | 2.40851350 | -1.66128283 |
| 6 | 4.28403361 | 1.83053695 | -0.16780670 |
| 1 | -1.92988495 | 1.21246868 | 4.62725117 |
| 1 | -2.38812940 | 0.09002955 | 3.30703907 |
| 1 | -2.32142596 | 3.04420328 | 2.95693215 |
| 1 | -1.25478667 | 3.11284532 | 1.53249795 |
| 1 | -5.67863229 | 0.25978452 | -0.91635836 |
| 1 | -4.53727769 | 2.55763236 | 2.52123591 |
| 1 | 0.91163562 | 1.44868547 | 3.87672107 |
| 1 | -0.16092872 | 2.80592810 | 4.30071338 |
| 1 | 3.22062367 | 5.50675875 | 0.40918140 |
| 1 | 0.59443067 | 4.88801601 | 3.74181138 |
| 1 | -4.12507588 | -1.44494318 | -3.52972528 |
| 1 | -5.09977264 | -1.50041667 | -2.05096043 |
| 1 | -5.03022224 | -0.00477982 | -3.02370073 |
| 1 | -1.51384193 | -1.45593707 | -0.90356361 |
| 1 | -3.07046724 | -2.31221808 | -0.80065262 |
| 1 | -2.16008251 | -2.28213285 | -2.33223329 |
| 1 | -2.86910945 | 1.30698322 | -3.20138791 |
| 1 | -1.38756624 | 0.74748187 | -2.38420980 |
| 1 | -2.06952867 | -0.19255366 | -3.73285089 |
| 1 | 4.67984219 | 3.61915363 | -2.12996673 |
| 1 | 4.89315560 | 4.47811212 | -0.59322372 |
| 1 | 3.51653719 | 4.87064275 | -1.65960269 |
| 1 | 1.71999673 | 1.57603223 | -1.27618140 |
| 1 | 1.63239263 | 3.20131272 | -1.99344422 |
| 1 | 2.87549193 | 2.05273871 | -2.54074209 |
| 1 | 5.02086984 | 2.21731106 | 0.55143467 |
| 1 | 3.75571959 | 0.98943976 | 0.29372007 |
| 1 | 4.83309096 | 1.45850498 | -1.04605280 |
| 1 | -0.83595113 | -0.13621355 | 4.15432176 |
| 31 | -0.01724593 | 0.41782807 | 1.26125388 |
| 6 | 1.50002287 | -5.35289131 | -1.76233194 |
| 6 | 2.21466289 | -4.17506318 | -1.09169845 |
| 6 | 1.43846538 | -3.58511514 | 0.09145733 |
| 6 | 2.17878368 | -2.42436083 | 0.76175090 |
| 6 | 1.46842486 | -1.86506743 | 1.99564682 |
| 1 | 1.14284774 | -6.06802492 | -1.00394628 |
| 1 | 2.39348254 | -3.39285995 | -1.84427523 |
| 1 | 0.45057118 | -3.23911036 | -0.24526678 |
| 1 | 2.33146480 | -1.60778211 | 0.03524621 |
| 1 | 2.20203883 | -5.91989587 | -2.39805501 |
| 1 | 3.20199397 | -4.52047202 | -0.74365909 |
| 1 | 1.26056517 | -4.37781029 | 0.84082396 |
| 1 | 3.18558870 | -2.75449658 | 1.07642034 |
| 1 | 2.12139578 | -1.10935982 | 2.47059980 |
| 1 | 1.31831184 | -2.67858956 | 2.72918732 |
| 8 | 0.17037764 | -1.33281231 | 1.70411793 |

Electronic energy = -3523.862942 a.u.
DFT-D3(BJ) dispersion correction = -0.110965 a.u.
Thermal free energy = 0.596445 a.u.
Gibbs free energy = -3523.377462 a.u.
Number of imaginary frequencies = 0.

**Int2 CL isomer/conformer 25**

| Atomic N. | X | Y | Z |
|---|---|---|---|
| 7 | -1.67942810 | 1.50698893 | 0.47441115 |
| 8 | 0.60060236 | 1.14484336 | -1.45084271 |
| 8 | 0.58657997 | -0.31709430 | 1.25851335 |
| 6 | -3.08595818 | 1.30791418 | 0.03229814 |
| 6 | -0.02436427 | 2.13856958 | -2.13838019 |
| 6 | 0.30179213 | 2.38690891 | -3.50321955 |
| 6 | -0.40274035 | 3.41021417 | -4.15483389 |
| 6 | -1.37650774 | 4.18589015 | -3.52003286 |
| 6 | -1.64779838 | 3.96758054 | -2.17349959 |
| 6 | -0.96904431 | 2.96465597 | -1.47327394 |
| 6 | -1.14902031 | 2.84160298 | 0.01665823 |
| 1 | -1.90238769 | 4.96389796 | -4.07333640 |
| 6 | 1.39934786 | 1.58828403 | -4.23396461 |
| 6 | 0.82012484 | 0.64140733 | 2.19560011 |
| 6 | 2.09112199 | 0.72025755 | 2.83456715 |
| 6 | 2.26258541 | 1.72158601 | 3.80145472 |
| 6 | 1.25128544 | 2.62184209 | 4.14683769 |
| 6 | 0.01238003 | 2.52590426 | 3.52300710 |
| 6 | -0.21940203 | 1.53670986 | 2.55995091 |
| 6 | -1.60435097 | 1.38410399 | 1.98307256 |
| 1 | 1.43492765 | 3.38809705 | 4.89991072 |
| 6 | 3.23523750 | -0.25275471 | 2.48598925 |
| 6 | 1.59703915 | 2.07167083 | -5.68300832 |
| 6 | 1.02799244 | 0.08959178 | -4.29563775 |
| 6 | 2.74968494 | 1.76401084 | -3.49881874 |
| 6 | 4.49931621 | 0.02037309 | 3.32309867 |
| 6 | 3.63122565 | -0.10350374 | 0.99814767 |
| 6 | 2.79917625 | -1.70846112 | 2.77721905 |
| 1 | -3.71992203 | 2.10125630 | 0.45966122 |
| 1 | -3.13471141 | 1.35184251 | -1.06098278 |
| 1 | -1.83370042 | 3.62406271 | 0.38361503 |
| 1 | -0.18320556 | 2.96958194 | 0.52760144 |
| 1 | -0.18771314 | 3.61341425 | -5.20230951 |
| 1 | -2.37472382 | 4.58924744 | -1.64585621 |
| 1 | -2.03399467 | 0.39857370 | 2.21884477 |
| 1 | -2.27032816 | 2.14979846 | 2.41353467 |
| 1 | 3.22390787 | 1.81142591 | 4.30385203 |
| 1 | -0.79621895 | 3.21015247 | 3.78943713 |
| 1 | 2.40084217 | 1.48365407 | -6.14848043 |
| 1 | 0.69231218 | 1.93562809 | -6.29324266 |
| 1 | 1.89247992 | 3.12969216 | -5.72913216 |
| 1 | 0.95181134 | -0.33901023 | -3.29095312 |
| 1 | 0.06796131 | -0.05529346 | -4.81213383 |
| 1 | 1.80044854 | -0.46455108 | -4.85037046 |
| 1 | 3.05337286 | 2.82088553 | -3.49564852 |
| 1 | 2.69114885 | 1.41566858 | -2.46159123 |
| 1 | 3.53285778 | 1.18897202 | -4.01616647 |
| 1 | 5.27608630 | -0.70532224 | 3.04279120 |
| 1 | 4.31462085 | -0.09290054 | 4.40111897 |

| | | | |
|---|---|---|---|
| 1 | 4.90669432 | 1.02562583 | 3.14274667 |
| 1 | 2.79517578 | -0.33366823 | 0.32970845 |
| 1 | 3.96789720 | 0.92167627 | 0.78715613 |
| 1 | 4.46092015 | -0.78778383 | 0.76385012 |
| 1 | 2.56856093 | -1.83660528 | 3.84489534 |
| 1 | 1.91333622 | -1.98622475 | 2.19584508 |
| 1 | 3.61681032 | -2.40009805 | 2.52271341 |
| 1 | -3.43628953 | 0.33183210 | 0.38550369 |
| 8 | -4.57802636 | -3.23436177 | 2.90280833 |
| 6 | -4.74918211 | -4.65149591 | 2.69714316 |
| 1 | -5.33406069 | -4.99784662 | 3.55577284 |
| 6 | -3.03842914 | -3.19024120 | 0.93949240 |
| 6 | -1.61270232 | -3.58222577 | 1.39172288 |
| 6 | -0.77485516 | -4.24418059 | 0.27894932 |
| 6 | -3.79334884 | -2.49873842 | 2.05525561 |
| 6 | 0.01241626 | -3.31650559 | -0.66341725 |
| 6 | -0.78363739 | -2.38688653 | -1.58707880 |
| 1 | -2.95864856 | -2.46119788 | 0.12218555 |
| 1 | -1.09743253 | -2.68501617 | 1.76605762 |
| 1 | -0.04071008 | -4.90943038 | 0.76107851 |
| 1 | 0.72055083 | -2.70914202 | -0.07347760 |
| 1 | -3.56838758 | -4.08122488 | 0.57468116 |
| 1 | -1.68307757 | -4.27935158 | 2.24241417 |
| 1 | -1.42423403 | -4.90509162 | -0.32212112 |
| 1 | 0.63344645 | -3.95664099 | -1.31428944 |
| 1 | -0.12007704 | -2.05125899 | -2.40482712 |
| 1 | -1.60463040 | -2.95369897 | -2.06299989 |
| 8 | -3.71468313 | -1.30095029 | 2.26878742 |
| 8 | -1.38753230 | -1.26445397 | -0.93746689 |
| 1 | -3.78691462 | -5.18044079 | 2.67711643 |
| 1 | -5.30947100 | -4.85350086 | 1.77347629 |
| 31 | -0.40222736 | 0.11125753 | -0.25915942 |

Electronic energy = -3523.861822 a.u.
DFT-D3(BJ) dispersion correction = -0.115001 a.u.
Thermal free energy = 0.599751 a.u.
Gibbs free energy = -3523.377072 a.u.
Number of imaginary frequencies = 0.

**Int2 CL isomer/conformer 26**

| Atomic N. | X | Y | Z |
|---|---|---|---|
| 8 | -2.18936767 | -5.20172936 | -0.46002533 |
| 6 | -2.29982405 | -4.84190754 | 0.92895927 |
| 1 | -3.34217950 | -5.04275600 | 1.19868260 |
| 1 | -2.07960836 | -3.77687783 | 1.08907439 |
| 1 | -1.63893197 | -5.45452708 | 1.55955285 |
| 6 | -0.98746843 | -5.04849810 | -1.11834942 |
| 8 | -0.94351675 | -5.36033371 | -2.28943922 |
| 7 | -0.66807369 | 0.86863573 | 2.43881041 |
| 8 | -0.95437144 | 0.59037778 | -0.52148885 |
| 8 | 1.82609879 | 1.17610414 | 0.78177784 |
| 6 | -1.23477405 | -0.20301652 | 3.29432153 |
| 6 | -2.28399028 | 0.59541821 | -0.22786260 |

| | | | |
|---|---|---|---|
| 6 | -3.22491490 | 0.10705869 | -1.17912121 |
| 6 | -4.57846432 | 0.13749643 | -0.81170973 |
| 6 | -5.02124035 | 0.62576911 | 0.42090033 |
| 6 | -4.09376954 | 1.12734759 | 1.32746892 |
| 6 | -2.73098837 | 1.13039648 | 1.00832729 |
| 6 | -1.74882752 | 1.79283449 | 1.93675462 |
| 1 | -6.08460748 | 0.62223269 | 0.66012397 |
| 6 | -2.78396940 | -0.42934681 | -2.55506625 |
| 6 | 1.79364820 | 2.44731566 | 1.26107091 |
| 6 | 2.52518143 | 3.47687716 | 0.60210464 |
| 6 | 2.45998599 | 4.76308029 | 1.15699482 |
| 6 | 1.71934728 | 5.05850835 | 2.30520932 |
| 6 | 1.02063125 | 4.04157152 | 2.94526684 |
| 6 | 1.06076256 | 2.73592774 | 2.44118942 |
| 6 | 0.38661099 | 1.62910244 | 3.20936804 |
| 1 | 1.69505184 | 6.07671434 | 2.69300011 |
| 6 | 3.35342854 | 3.19615516 | -0.66733823 |
| 6 | -3.98741196 | -0.84870266 | -3.42064433 |
| 6 | -1.89162643 | -1.67946978 | -2.37662262 |
| 6 | -2.01858805 | 0.66778618 | -3.33284278 |
| 6 | 4.07748225 | 4.45904778 | -1.17119170 |
| 6 | 2.43051634 | 2.70957855 | -1.80956553 |
| 6 | 4.43751183 | 2.13239383 | -0.37110966 |
| 1 | -1.66592247 | 0.23415790 | 4.20946440 |
| 1 | -2.02030299 | -0.73126682 | 2.74299946 |
| 1 | -2.27419186 | 2.21689693 | 2.80894387 |
| 1 | -1.22488414 | 2.61414199 | 1.42612177 |
| 1 | -5.32140596 | -0.23851377 | -1.51251623 |
| 1 | -4.42024140 | 1.54200055 | 2.28398686 |
| 1 | 1.12193882 | 0.87164394 | 3.52575317 |
| 1 | -0.08457173 | 2.04039483 | 4.11759076 |
| 1 | 3.00258692 | 5.57333423 | 0.67369083 |
| 1 | 0.44723357 | 4.24819230 | 3.85167349 |
| 1 | -3.61831326 | -1.20754708 | -4.39154643 |
| 1 | -4.55985452 | -1.66956229 | -2.96540158 |
| 1 | -4.67063236 | -0.00908679 | -3.61493364 |
| 1 | -0.98462451 | -1.45332006 | -1.80491751 |
| 1 | -2.44557525 | -2.47720957 | -1.86160831 |
| 1 | -1.59038859 | -2.07000646 | -3.35958427 |
| 1 | -2.66191528 | 1.54408525 | -3.50033411 |
| 1 | -1.12012157 | 0.99315231 | -2.79754676 |
| 1 | -1.71594282 | 0.27860195 | -4.31660771 |
| 1 | 4.66031814 | 4.20182774 | -2.06686111 |
| 1 | 4.77839176 | 4.86011279 | -0.42482609 |
| 1 | 3.37446383 | 5.25589092 | -1.45377574 |
| 1 | 1.91560601 | 1.77868535 | -1.54982207 |
| 1 | 1.67223134 | 3.46934963 | -2.04736647 |
| 1 | 3.02640608 | 2.53068562 | -2.71719522 |
| 1 | 5.12441587 | 2.48671031 | 0.41137655 |
| 1 | 3.99481097 | 1.18567791 | -0.04333104 |
| 1 | 5.02969282 | 1.94424558 | -1.27935609 |
| 1 | -0.44352073 | -0.91348657 | 3.55980563 |
| 31 | 0.28877256 | 0.13368121 | 0.77221428 |

| | | | |
|---|---|---|---|
| 6 | 0.18122766 | -4.50215576 | -0.32210510 |
| 6 | 1.43561915 | -4.31833060 | -1.17766882 |
| 6 | 2.70144418 | -3.94496164 | -0.38875030 |
| 6 | 2.85554229 | -2.49468887 | 0.10662178 |
| 6 | 1.99728845 | -2.03887002 | 1.29456201 |
| 1 | -0.10615359 | -3.54754177 | 0.14798110 |
| 1 | 1.23789597 | -3.56023306 | -1.95113338 |
| 1 | 2.81188659 | -4.62928582 | 0.47208770 |
| 1 | 2.69695487 | -1.78960397 | -0.72698930 |
| 1 | 0.38573867 | -5.19946390 | 0.51018964 |
| 1 | 1.62260602 | -5.25515217 | -1.72262146 |
| 1 | 3.56525752 | -4.15280504 | -1.04078914 |
| 1 | 3.90770364 | -2.36526688 | 0.41443194 |
| 1 | 2.52353712 | -1.21061266 | 1.80132260 |
| 1 | 1.90854254 | -2.86372598 | 2.02562605 |
| 8 | 0.66070689 | -1.64133661 | 0.93456332 |

Electronic energy = -3523.859751 a.u.
DFT-D3(BJ) dispersion correction = -0.113667 a.u.
Thermal free energy = 0.597052 a.u.
Gibbs free energy = -3523.376366 a.u.
Number of imaginary frequencies = 0.

**Int2_CL isomer/conformer 27**

| Atomic N. | X | Y | Z |
|---|---|---|---|
| 8 | -5.16436092 | -0.51841735 | 2.35124508 |
| 6 | -6.09302896 | -1.43599150 | 2.96603003 |
| 1 | -5.99180884 | -1.26760908 | 4.04295066 |
| 6 | -5.67365276 | -1.55374193 | 0.13994426 |
| 6 | -4.92770745 | -2.90915513 | 0.03567740 |
| 6 | -3.47810487 | -2.84174364 | -0.47915571 |
| 6 | -4.98959014 | -0.50323014 | 0.99543203 |
| 6 | -2.42086023 | -2.62888829 | 0.61130590 |
| 6 | -0.99170083 | -2.60526921 | 0.06428576 |
| 1 | -5.74368195 | -1.11391094 | -0.86341342 |
| 1 | -4.94602522 | -3.43162960 | 1.00586180 |
| 1 | -3.25062169 | -3.79070278 | -0.99272573 |
| 1 | -2.59881157 | -1.69188283 | 1.16139843 |
| 1 | -6.69804430 | -1.73582631 | 0.49676230 |
| 1 | -5.52943038 | -3.52707451 | -0.64852192 |
| 1 | -3.38282450 | -2.05300995 | -1.24078596 |
| 1 | -2.48633323 | -3.45003327 | 1.34804928 |
| 1 | -0.28723420 | -2.56778016 | 0.91428286 |
| 1 | -0.79308534 | -3.54654929 | -0.48160257 |
| 8 | -4.26574211 | 0.36024419 | 0.53176028 |
| 8 | -0.78020964 | -1.52879044 | -0.85568123 |
| 1 | -5.84776511 | -2.48119977 | 2.73838179 |
| 1 | -7.12559087 | -1.21457009 | 2.66147875 |
| 7 | -0.76467429 | 1.69465070 | -0.72319258 |
| 8 | 1.74486697 | 0.20538362 | -1.32311650 |
| 8 | 0.44779989 | 0.02804984 | 1.48650275 |
| 6 | -1.91697838 | 1.55505677 | -1.65289596 |
| 6 | 1.69709830 | 0.91507052 | -2.47998139 |

| | | | |
|---|---|---|---|
| 6 | 2.43193660 | 0.48081530 | -3.62080019 |
| 6 | 2.34450776 | 1.26967969 | -4.77736338 |
| 6 | 1.57958869 | 2.43789117 | -4.84382182 |
| 6 | 0.87932436 | 2.85867541 | -3.71783761 |
| 6 | 0.94157376 | 2.11551741 | -2.53442729 |
| 6 | 0.27673765 | 2.62881148 | -1.28556477 |
| 1 | 1.53978451 | 3.01318138 | -5.76882286 |
| 6 | 3.28539385 | -0.80207164 | -3.59016871 |
| 6 | 0.60334421 | 1.23653463 | 2.08651375 |
| 6 | 1.53314104 | 1.38222609 | 3.15696197 |
| 6 | 1.64300581 | 2.65022696 | 3.74523238 |
| 6 | 0.88870132 | 3.74851769 | 3.32334012 |
| 6 | -0.02230053 | 3.58963695 | 2.28518075 |
| 6 | -0.18397550 | 2.34233416 | 1.67009841 |
| 6 | -1.25832145 | 2.17614110 | 0.62459460 |
| 1 | 1.01493399 | 4.71718693 | 3.80685990 |
| 6 | 2.38667349 | 0.19761422 | 3.65196751 |
| 6 | 4.01564080 | -1.03919515 | -4.92558018 |
| 6 | 2.38380246 | -2.03280654 | -3.33325968 |
| 6 | 4.36628007 | -0.69577229 | -2.48807339 |
| 6 | 3.29102891 | 0.59404434 | 4.83441095 |
| 6 | 3.31008363 | -0.30657391 | 2.51781943 |
| 6 | 1.47072077 | -0.94992884 | 4.14043946 |
| 1 | -2.39272567 | 2.53827616 | -1.79928171 |
| 1 | -1.56269910 | 1.17777159 | -2.61830936 |
| 1 | -0.20586175 | 3.60113293 | -1.48025081 |
| 1 | 1.01658441 | 2.77185393 | -0.48429324 |
| 1 | 2.88924282 | 0.96151006 | -5.66802999 |
| 1 | 0.29112629 | 3.77896856 | -3.74144084 |
| 1 | -2.01777393 | 1.44427297 | 0.94042483 |
| 1 | -1.77299893 | 3.13846647 | 0.46828150 |
| 1 | 2.34868892 | 2.79432533 | 4.56131495 |
| 1 | -0.63171210 | 4.43181688 | 1.94981744 |
| 1 | 4.61656451 | -1.95628974 | -4.84532144 |
| 1 | 3.31624456 | -1.17565035 | -5.76321247 |
| 1 | 4.70054717 | -0.21548632 | -5.17418449 |
| 1 | 1.85079223 | -1.95701628 | -2.37941943 |
| 1 | 1.63947981 | -2.14135683 | -4.13553593 |
| 1 | 2.99832505 | -2.94602147 | -3.31558956 |
| 1 | 5.03750561 | 0.15284085 | -2.68582397 |
| 1 | 3.91971009 | -0.56220900 | -1.49701966 |
| 1 | 4.97572876 | -1.61236220 | -2.47658556 |
| 1 | 3.86646008 | -0.28649975 | 5.15394626 |
| 1 | 2.71038553 | 0.94229831 | 5.70091187 |
| 1 | 4.01238356 | 1.37741031 | 4.56039264 |
| 1 | 2.73747777 | -0.65221879 | 1.65087282 |
| 1 | 3.98969589 | 0.49160370 | 2.18621173 |
| 1 | 3.92364129 | -1.14426094 | 2.88305451 |
| 1 | 0.84764876 | -0.61523533 | 4.98269647 |
| 1 | 0.81216167 | -1.30624204 | 3.34104427 |
| 1 | 2.08608711 | -1.79321447 | 4.48965034 |
| 1 | -2.64627986 | 0.86027679 | -1.22253696 |
| 31 | 0.18006580 | -0.07289730 | -0.34975995 |

Electronic energy = -3523.861866 a.u.
DFT-D3(BJ) dispersion correction = -0.112494 a.u.
Thermal free energy = 0.598285 a.u.
Gibbs free energy = -3523.376075 a.u.
Number of imaginary frequencies = 0.

## Initiation step of LA addition

**RC vdW LA isomer/conformer 1**

| Atomic N. | X | Y | Z |
|---|---|---|---|
| 7 | 0.54747041 | 1.32345215 | 1.68328063 |
| 8 | -1.87554148 | 0.48861198 | 0.12003196 |
| 8 | 0.99003157 | -0.02449978 | -0.97779617 |
| 6 | 0.67265337 | 0.91302361 | 3.10743354 |
| 6 | -2.54057293 | 1.20442570 | 1.06673094 |
| 6 | -3.94707147 | 1.04092477 | 1.22595187 |
| 6 | -4.56424554 | 1.77923928 | 2.24678725 |
| 6 | -3.86567492 | 2.66304998 | 3.07406733 |
| 6 | -2.50412317 | 2.85697562 | 2.86717352 |
| 6 | -1.83850109 | 2.14996109 | 1.85964409 |
| 6 | -0.40547734 | 2.48170403 | 1.53551990 |
| 6 | -4.76872680 | 0.12444429 | 0.29638400 |
| 6 | 1.48898760 | 1.19459559 | -1.32252561 |
| 6 | 1.59331953 | 1.55945584 | -2.69569367 |
| 6 | 2.14669150 | 2.81584430 | -2.98319244 |
| 6 | 2.57770458 | 3.70027457 | -1.99083823 |
| 6 | 2.46603168 | 3.33211481 | -0.65509823 |
| 6 | 1.93579682 | 2.08305560 | -0.30981529 |
| 6 | 1.91686221 | 1.66914364 | 1.14014630 |
| 6 | 1.10948066 | 0.62712966 | -3.82491522 |
| 6 | -6.27069918 | 0.16097601 | 0.63646562 |
| 6 | -4.30623915 | -1.34560720 | 0.41939634 |
| 6 | -4.61506923 | 0.60012781 | -1.16842834 |
| 6 | 1.35174809 | 1.23871489 | -5.21802377 |
| 6 | -0.41123886 | 0.37661703 | -3.69095212 |
| 6 | 1.87155255 | -0.71810580 | -3.77689568 |
| 1 | 1.09413561 | 1.74459067 | 3.69485096 |
| 1 | -0.31862370 | 0.66629201 | 3.50398856 |
| 1 | -0.04996782 | 3.30296998 | 2.17998774 |
| 1 | -0.31809016 | 2.81145707 | 0.48967349 |
| 1 | -5.63461132 | 1.66426936 | 2.40676816 |
| 1 | -1.94797380 | 3.57651793 | 3.47225241 |
| 1 | 2.53374725 | 0.77169039 | 1.30427287 |
| 1 | 2.33137758 | 2.47970638 | 1.76176706 |
| 1 | 2.23950472 | 3.12584164 | -4.02219434 |
| 1 | 2.80272712 | 4.00631287 | 0.13540039 |
| 1 | -6.81094546 | -0.48979185 | -0.06555356 |
| 1 | -6.47624086 | -0.20724818 | 1.65218926 |
| 1 | -6.69197322 | 1.17193480 | 0.54056058 |
| 1 | -3.26067497 | -1.46608611 | 0.11622475 |
| 1 | -4.41979680 | -1.70488127 | 1.45294312 |

| | | | |
|---|---|---|---|
| 1 | -4.92586824 | -1.98446726 | -0.22788481 |
| 1 | -4.98465373 | 1.62958658 | -1.28155040 |
| 1 | -3.57000486 | 0.56710489 | -1.49586110 |
| 1 | -5.20797689 | -0.04640496 | -1.83300513 |
| 1 | 0.99790878 | 0.53328835 | -5.98300776 |
| 1 | 2.41846659 | 1.42538380 | -5.40893241 |
| 1 | 0.80194337 | 2.18033215 | -5.35906660 |
| 1 | -0.66163752 | -0.11456093 | -2.74474717 |
| 1 | -0.96804645 | 1.32292675 | -3.74772838 |
| 1 | -0.75453239 | -0.26835411 | -4.51384649 |
| 1 | 2.95217274 | -0.55624651 | -3.90202370 |
| 1 | 1.70034933 | -1.23965955 | -2.82900109 |
| 1 | 1.53078234 | -1.36806137 | -4.59673729 |
| 1 | 1.33374595 | 0.04097626 | 3.18475112 |
| 8 | 5.10175246 | -4.11316275 | -0.76946216 |
| 8 | -0.01308953 | -1.84920519 | 1.12256343 |
| 6 | -0.88987458 | -2.30464947 | 2.15645553 |
| 1 | -1.86427424 | -1.78857041 | 2.15854188 |
| 1 | -1.08671706 | -3.37798916 | 2.00401710 |
| 1 | -0.42690493 | -2.17618566 | 3.15077176 |
| 31 | -0.16455753 | -0.16016026 | 0.46462727 |
| 1 | -4.39052523 | 3.20768892 | 3.85875014 |
| 1 | 2.99569328 | 4.66852513 | -2.26562443 |
| 8 | 3.70961550 | -4.38793822 | 0.95916789 |
| 6 | 4.26664262 | -3.63921100 | -0.03382390 |
| 6 | 3.74975289 | -2.20144206 | -0.12084200 |
| 8 | 3.60452756 | -1.63104239 | 1.22474974 |
| 6 | 2.95209251 | -2.38106640 | 2.15270062 |
| 6 | 2.59501478 | -3.79347019 | 1.69766662 |
| 8 | 2.69328894 | -1.91474680 | 3.24296549 |
| 6 | 2.26451546 | -4.72515548 | 2.84548148 |
| 6 | 4.67645791 | -1.29650783 | -0.90470112 |
| 1 | 2.74526796 | -2.21645633 | -0.58048882 |
| 1 | 1.73312633 | -3.69322601 | 1.01391458 |
| 1 | 2.00444841 | -5.71627765 | 2.45443388 |
| 1 | 1.41454798 | -4.32737479 | 3.41146491 |
| 1 | 3.11968085 | -4.81916068 | 3.52576857 |
| 1 | 4.79382405 | -1.69168445 | -1.92015264 |
| 1 | 5.66689685 | -1.25794109 | -0.43483955 |
| 1 | 4.25840416 | -0.28465903 | -0.96343503 |

Electronic energy = -3673.047581 a.u.
DFT-D3(BJ) dispersion correction = -0.117295 a.u.
Thermal free energy = 0.578290 a.u.
Gibbs free energy = -3672.586587 a.u.
Number of imaginary frequencies = 0.

**RC vdW LA isomer/conformer 2**

| Atomic N. | X | Y | Z |
|---|---|---|---|
| 7 | -0.59822521 | 1.01663178 | 1.87239681 |
| 8 | -1.46275402 | -1.29465747 | 0.15688897 |
| 8 | 0.54660921 | 0.83130776 | -0.90676820 |
| 6 | -0.07741863 | 0.75440332 | 3.24079633 |

| | | | |
|---|---|---|---|
| 6 | -2.35129862 | -1.50820779 | 1.16608276 |
| 6 | -3.02806117 | -2.75791730 | 1.26807218 |
| 6 | -3.89901500 | -2.92551526 | 2.35514327 |
| 6 | -4.13567463 | -1.92409833 | 3.30081312 |
| 6 | -3.51555819 | -0.68816144 | 3.15268062 |
| 6 | -2.64018489 | -0.46430149 | 2.08418704 |
| 6 | -2.10180482 | 0.92086535 | 1.83836826 |
| 1 | -4.81592898 | -2.10731383 | 4.13238185 |
| 6 | -2.84942806 | -3.86847614 | 0.21287544 |
| 6 | -0.18521251 | 1.96426589 | -1.10129022 |
| 6 | -0.53856070 | 2.36770878 | -2.42096052 |
| 6 | -1.25793420 | 3.56485922 | -2.55225332 |
| 6 | -1.63924008 | 4.34473642 | -1.45734949 |
| 6 | -1.29632021 | 3.93249141 | -0.17478248 |
| 6 | -0.56416744 | 2.75433417 | 0.01507397 |
| 6 | -0.12727908 | 2.37790384 | 1.40861896 |
| 1 | -2.20233615 | 5.26500687 | -1.61152072 |
| 6 | -0.16791837 | 1.52491270 | -3.65856708 |
| 6 | -3.72260131 | -5.09933518 | 0.52175827 |
| 6 | -1.38139045 | -4.34917340 | 0.15870568 |
| 6 | -3.27559699 | -3.33499484 | -1.17612923 |
| 6 | -0.64657197 | 2.18374091 | -4.96643770 |
| 6 | -0.83986044 | 0.13413849 | -3.57237650 |
| 6 | 1.36655654 | 1.36272274 | -3.76358229 |
| 1 | -0.45656564 | 1.52321770 | 3.93298861 |
| 1 | -0.42200899 | -0.22918572 | 3.57905211 |
| 1 | -2.50786530 | 1.62432967 | 2.58427319 |
| 1 | -2.40342552 | 1.27494777 | 0.84148403 |
| 1 | -4.41763253 | -3.87502987 | 2.47292921 |
| 1 | -3.72218033 | 0.12380428 | 3.85347812 |
| 1 | 0.97055528 | 2.35550189 | 1.48813443 |
| 1 | -0.49860145 | 3.12622180 | 2.12785134 |
| 1 | -1.54332972 | 3.90231908 | -3.54668105 |
| 1 | -1.58248740 | 4.52918961 | 0.69399822 |
| 1 | -3.57449288 | -5.84871764 | -0.26858555 |
| 1 | -3.45255832 | -5.57063401 | 1.47803907 |
| 1 | -4.79350948 | -4.85186598 | 0.54531712 |
| 1 | -0.70721001 | -3.54220851 | -0.14630944 |
| 1 | -1.05782340 | -4.72870451 | 1.13906720 |
| 1 | -1.28654897 | -5.16953312 | -0.56867299 |
| 1 | -4.33327362 | -3.03440050 | -1.16761301 |
| 1 | -2.67089817 | -2.47308363 | -1.47973983 |
| 1 | -3.15561899 | -4.12658617 | -1.93125962 |
| 1 | -0.35218958 | 1.54830525 | -5.81354839 |
| 1 | -0.19276418 | 3.17313564 | -5.12245846 |
| 1 | -1.74013535 | 2.29199014 | -5.00057121 |
| 1 | -0.50351493 | -0.42732644 | -2.69435869 |
| 1 | -1.93369162 | 0.23250035 | -3.52183472 |
| 1 | -0.59355209 | -0.45289495 | -4.47000337 |
| 1 | 1.85471320 | 2.34382055 | -3.85647406 |
| 1 | 1.77425416 | 0.84661166 | -2.88761364 |
| 1 | 1.61799030 | 0.77536198 | -4.65937960 |
| 1 | 1.01849914 | 0.78261069 | 3.23043628 |

| | | | |
|---|---|---|---|
| 8 | 1.59660757 | -1.17877575 | 1.00986029 |
| 6 | 1.52115044 | -2.43055464 | 1.69600113 |
| 1 | 0.60007672 | -2.54932959 | 2.29251197 |
| 8 | 3.39554634 | 1.20317673 | 3.02649266 |
| 1 | 1.57442087 | -3.27481948 | 0.98880459 |
| 1 | 2.37635763 | -2.50516654 | 2.38671573 |
| 31 | 0.08718247 | -0.31373711 | 0.47635721 |
| 8 | 3.52444144 | 2.15260031 | 1.01124417 |
| 6 | 3.81839292 | 1.15714250 | 1.88981826 |
| 6 | 4.66941958 | 0.02749003 | 1.31842878 |
| 8 | 5.73182232 | 0.56375187 | 0.46691271 |
| 6 | 5.37617442 | 1.48675541 | -0.46985850 |
| 6 | 3.92344461 | 1.95940918 | -0.38896737 |
| 8 | 6.17773914 | 1.87465614 | -1.28872605 |
| 6 | 3.69569919 | 3.26995701 | -1.11222794 |
| 6 | 5.33134489 | -0.81781109 | 2.38716563 |
| 1 | 3.99312918 | -0.58760335 | 0.69696276 |
| 1 | 3.27447353 | 1.16938208 | -0.80813117 |
| 1 | 2.63908378 | 3.55680249 | -1.05292586 |
| 1 | 3.97531952 | 3.15518035 | -2.16561198 |
| 1 | 4.31538460 | 4.06345426 | -0.67674636 |
| 1 | 4.56620724 | -1.24263013 | 3.04665724 |
| 1 | 6.01170515 | -0.20881015 | 2.99496077 |
| 1 | 5.89791923 | -1.63209473 | 1.91953026 |

Electronic energy = -3673.047227 a.u.
DFT-D3(BJ) dispersion correction = -0.116996 a.u.
Thermal free energy = 0.578202 a.u.
Gibbs free energy = -3672.586021 a.u.
Number of imaginary frequencies = 0.

**RC vdW LA isomer/conformer 3**

| Atomic N. | X | Y | Z |
|---|---|---|---|
| 7 | 0.34397165 | 2.69449117 | 1.35897224 |
| 8 | -1.07028850 | 0.12980791 | 0.71952175 |
| 8 | 0.87892262 | 1.47443201 | -1.33126087 |
| 6 | 1.03952076 | 2.76416227 | 2.66849247 |
| 6 | -1.78134245 | 0.43814767 | 1.83971259 |
| 6 | -2.53307551 | -0.57327032 | 2.50409050 |
| 6 | -3.24603055 | -0.19164428 | 3.65015365 |
| 6 | -3.24547878 | 1.11501334 | 4.14588624 |
| 6 | -2.52688101 | 2.09701168 | 3.47355246 |
| 6 | -1.80717898 | 1.77419218 | 2.31713230 |
| 6 | -1.14550476 | 2.87117004 | 1.52712928 |
| 1 | -3.81157561 | 1.35934963 | 5.04457024 |
| 6 | -2.56711015 | -2.02664405 | 1.99309025 |
| 6 | 0.32490997 | 2.62758644 | -1.78835808 |
| 6 | -0.17554067 | 2.69866964 | -3.12029695 |
| 6 | -0.72132069 | 3.92226037 | -3.53395846 |
| 6 | -0.78955097 | 5.04363467 | -2.70199329 |
| 6 | -0.28823129 | 4.96325729 | -1.40818208 |
| 6 | 0.27974007 | 3.76926448 | -0.94710565 |
| 6 | 0.91138715 | 3.73625570 | 0.42019838 |

| | | | |
|---|---|---|---|
| 1 | -1.22999609 | 5.97045212 | -3.06891687 |
| 6 | -0.12390597 | 1.48552364 | -4.07014795 |
| 6 | -3.47885989 | -2.92084181 | 2.85446671 |
| 6 | -1.14772306 | -2.63831094 | 2.04410656 |
| 6 | -3.11340288 | -2.07272477 | 0.54595219 |
| 6 | -0.69593198 | 1.82001820 | -5.46075459 |
| 6 | -0.96642998 | 0.32244721 | -3.49490947 |
| 6 | 1.34100918 | 1.03034042 | -4.27324844 |
| 1 | 0.89282430 | 3.75879379 | 3.11928746 |
| 1 | 0.63348390 | 2.00054770 | 3.34044146 |
| 1 | -1.32298943 | 3.84712448 | 2.00894313 |
| 1 | -1.55619502 | 2.92022675 | 0.50781034 |
| 1 | -3.82384268 | -0.94362237 | 4.18411973 |
| 1 | -2.53435982 | 3.13040392 | 3.82747901 |
| 1 | 1.98783048 | 3.51195843 | 0.34684957 |
| 1 | 0.80493250 | 4.72208171 | 0.90234316 |
| 1 | -1.11724720 | 4.00778000 | -4.54410829 |
| 1 | -0.32033496 | 5.83162788 | -0.74645151 |
| 1 | -3.47382774 | -3.93790498 | 2.43823843 |
| 1 | -3.13138033 | -2.98944375 | 3.89556887 |
| 1 | -4.52053869 | -2.56881535 | 2.85639999 |
| 1 | -0.44813276 | -2.07158514 | 1.41915076 |
| 1 | -0.76616430 | -2.64332626 | 3.07600186 |
| 1 | -1.17474937 | -3.67722313 | 1.68496755 |
| 1 | -4.14666282 | -1.69753921 | 0.51212902 |
| 1 | -2.50402472 | -1.47073290 | -0.13651442 |
| 1 | -3.11775247 | -3.11173849 | 0.18480070 |
| 1 | -0.62523139 | 0.92810772 | -6.09913584 |
| 1 | -0.13517712 | 2.62581311 | -5.95600934 |
| 1 | -1.75587016 | 2.10885669 | -5.41529457 |
| 1 | -0.58896190 | -0.01203317 | -2.52274596 |
| 1 | -2.01603729 | 0.62545937 | -3.37153296 |
| 1 | -0.94099250 | -0.53239655 | -4.18742030 |
| 1 | 1.93839359 | 1.83630785 | -4.72374440 |
| 1 | 1.80830346 | 0.74004098 | -3.32601936 |
| 1 | 1.36988931 | 0.16818042 | -4.95670612 |
| 1 | 2.11156700 | 2.58550491 | 2.52449116 |
| 8 | 1.97433301 | -0.13696391 | 0.98933704 |
| 6 | 3.30034016 | 0.27058653 | 0.62162221 |
| 1 | 3.61669802 | 1.18395781 | 1.15921920 |
| 8 | 0.36717348 | -5.02587783 | -0.81719539 |
| 1 | 3.39875934 | 0.45809014 | -0.46041848 |
| 1 | 4.00030641 | -0.53108494 | 0.89769136 |
| 31 | 0.58579506 | 0.89424361 | 0.40845515 |
| 8 | 1.66869936 | -4.61002352 | 0.95494719 |
| 6 | 1.23719324 | -4.35313979 | -0.31194623 |
| 6 | 1.93646455 | -3.17347723 | -0.98841397 |
| 8 | 3.37800926 | -3.23099245 | -0.73137427 |
| 6 | 3.76262109 | -3.39423448 | 0.56516358 |
| 6 | 2.61217734 | -3.65341364 | 1.53774174 |
| 8 | 4.93055834 | -3.32042411 | 0.87719795 |
| 6 | 3.07222380 | -4.21587802 | 2.86609538 |
| 6 | 1.73551967 | -3.14355166 | -2.48849479 |

| | | | |
|---|---|---|---|
| 1 | 1.55385329 | -2.24431320 | -0.52630726 |
| 1 | 2.08414475 | -2.69286341 | 1.68486999 |
| 1 | 2.20777210 | -4.36868511 | 3.52367333 |
| 1 | 3.76590815 | -3.51205565 | 3.34053037 |
| 1 | 3.59321978 | -5.17040059 | 2.72392942 |
| 1 | 0.66416543 | -3.07596282 | -2.71125767 |
| 1 | 2.12675887 | -4.05798000 | -2.95050386 |
| 1 | 2.24645198 | -2.27206200 | -2.91579627 |

Electronic energy = -3673.042932 a.u.
DFT-D3(BJ) dispersion correction = -0.114976 a.u.
Thermal free energy = 0.576847 a.u.
Gibbs free energy = -3672.581061 a.u.
Number of imaginary frequencies = 0.

**RC vdW LA isomer/conformer 4**

| Atomic N. | X | Y | Z |
|---|---|---|---|
| 7 | 0.91245674 | 1.54739850 | 1.31849271 |
| 8 | -1.71888231 | 0.55265246 | 0.26738601 |
| 8 | 0.86319719 | 0.31193173 | -1.42996992 |
| 6 | 1.37294054 | 1.10403338 | 2.65992948 |
| 6 | -2.22269276 | 1.19319093 | 1.35467565 |
| 6 | -3.55584072 | 0.92789846 | 1.78439850 |
| 6 | -3.99713198 | 1.58540863 | 2.94234005 |
| 6 | -3.20200637 | 2.48921584 | 3.65275781 |
| 6 | -1.92844576 | 2.79051630 | 3.18194424 |
| 6 | -1.43837847 | 2.16640931 | 2.02940619 |
| 6 | -0.13364903 | 2.62394829 | 1.43096105 |
| 6 | -4.49415303 | -0.00517624 | 0.99157924 |
| 6 | 1.18855762 | 1.57249694 | -1.82127878 |
| 6 | 0.97712741 | 1.97585907 | -3.17152864 |
| 6 | 1.35011270 | 3.28325853 | -3.51603835 |
| 6 | 1.90354607 | 4.18076176 | -2.59885321 |
| 6 | 2.10921574 | 3.77185293 | -1.28613055 |
| 6 | 1.76946602 | 2.47260499 | -0.89013619 |
| 6 | 2.10094700 | 2.01924008 | 0.50900364 |
| 6 | 0.35879503 | 1.02422479 | -4.21547324 |
| 6 | -5.89698080 | -0.07962978 | 1.62373641 |
| 6 | -3.93595613 | -1.44569013 | 0.94328953 |
| 6 | -4.66456465 | 0.53636966 | -0.44841706 |
| 6 | 0.25891682 | 1.68254164 | -5.60474858 |
| 6 | -1.07498832 | 0.62672952 | -3.79172062 |
| 6 | 1.23366753 | -0.24236843 | -4.36977501 |
| 1 | 1.84773635 | 1.94810419 | 3.18603537 |
| 1 | 0.51305932 | 0.75842398 | 3.24480842 |
| 1 | 0.28672818 | 3.45234525 | 2.02579838 |
| 1 | -0.29401690 | 2.99255491 | 0.40680793 |
| 1 | -5.00318139 | 1.38929686 | 3.30848330 |
| 1 | -1.30922232 | 3.53178455 | 3.69232677 |
| 1 | 2.80152080 | 1.17025027 | 0.49569572 |
| 1 | 2.57927016 | 2.84462624 | 1.06209598 |
| 1 | 1.19763215 | 3.62309613 | -4.53864044 |
| 1 | 2.55064296 | 4.45394260 | -0.55604422 |

| | | | |
|---|---|---|---|
| 1 | -6.52996168 | -0.73580300 | 1.00971954 |
| 1 | -5.87340285 | -0.50052990 | 2.63957203 |
| 1 | -6.38447133 | 0.90495351 | 1.66594247 |
| 1 | -2.96992202 | -1.48643649 | 0.42904136 |
| 1 | -3.81472728 | -1.85169968 | 1.95845439 |
| 1 | -4.63846189 | -2.09781801 | 0.40266190 |
| 1 | -5.10718578 | 1.54293008 | -0.43347453 |
| 1 | -3.70574370 | 0.58474534 | -0.97650763 |
| 1 | -5.34044125 | -0.12180345 | -1.01522633 |
| 1 | -0.17736565 | 0.96099912 | -6.30993417 |
| 1 | 1.24415330 | 1.97387891 | -5.99688178 |
| 1 | -0.39016319 | 2.57007767 | -5.59799010 |
| 1 | -1.08364997 | 0.09557930 | -2.83421090 |
| 1 | -1.71407666 | 1.51679211 | -3.70012820 |
| 1 | -1.51796128 | -0.03183073 | -4.55414227 |
| 1 | 2.24816833 | 0.02540134 | -4.70006747 |
| 1 | 1.30625646 | -0.79646760 | -3.42811256 |
| 1 | 0.79480042 | -0.90605637 | -5.13015382 |
| 1 | 2.09895571 | 0.29090413 | 2.54348802 |
| 8 | 1.87693333 | -5.73348137 | 0.95142169 |
| 8 | 0.50939127 | -1.62890125 | 0.80310842 |
| 6 | -0.19030882 | -2.24650892 | 1.88355993 |
| 1 | -1.20785995 | -1.84813684 | 2.03031943 |
| 1 | -0.28356112 | -3.32405115 | 1.67355541 |
| 1 | 0.36383953 | -2.12370276 | 2.83200077 |
| 31 | 0.07289806 | 0.03345707 | 0.22213185 |
| 1 | -3.58880274 | 2.96820325 | 4.55215054 |
| 1 | 2.17026099 | 5.18960591 | -2.91366338 |
| 8 | 2.81876663 | -4.03649038 | -0.15877886 |
| 6 | 2.61989450 | -4.77846076 | 0.96090779 |
| 6 | 3.41875729 | -4.32269675 | 2.18481202 |
| 8 | 3.40517582 | -2.86474848 | 2.30423408 |
| 6 | 3.67312000 | -2.14637149 | 1.18308769 |
| 6 | 3.80958091 | -2.96585613 | -0.10080704 |
| 8 | 3.82757097 | -0.94484801 | 1.25080211 |
| 6 | 3.65407730 | -2.13100023 | -1.35541545 |
| 6 | 2.88903630 | -4.89479963 | 3.48319359 |
| 1 | 4.46873181 | -4.63544388 | 2.03086397 |
| 1 | 4.81168459 | -3.43662034 | -0.07526811 |
| 1 | 3.76828974 | -2.77007570 | -2.23941352 |
| 1 | 4.42313785 | -1.35003282 | -1.37701728 |
| 1 | 2.66759611 | -1.65274085 | -1.37543606 |
| 1 | 2.91863241 | -5.98952477 | 3.43926489 |
| 1 | 1.84894195 | -4.58552596 | 3.64201070 |
| 1 | 3.50397015 | -4.54916818 | 4.32312731 |

Electronic energy = -3673.043151 a.u.
DFT-D3(BJ) dispersion correction = -0.115201 a.u.
Thermal free energy = 0.578118 a.u.
Gibbs free energy = -3672.580234 a.u.
Number of imaginary frequencies = 0.

**RC_vdW_LA isomer/conformer 5**

| Atomic N. | X | Y | Z |
|---|---|---|---|
| 7 | 0.95879910 | 1.64031148 | 1.23826833 |
| 8 | -1.76040108 | 0.48353780 | 0.66288435 |
| 8 | 0.61917748 | -0.01195010 | -1.25577599 |
| 6 | 1.52901859 | 1.44521438 | 2.59734901 |
| 6 | -2.15498749 | 1.33105835 | 1.65150776 |
| 6 | -3.43330328 | 1.16737891 | 2.26091265 |
| 6 | -3.76075174 | 2.04394913 | 3.30606432 |
| 6 | -2.90792929 | 3.06276680 | 3.73917949 |
| 6 | -1.69288672 | 3.25292382 | 3.09039855 |
| 6 | -1.31635127 | 2.41005717 | 2.03859220 |
| 6 | -0.08010229 | 2.73069610 | 1.23937641 |
| 1 | -3.20566938 | 3.71269937 | 4.56187933 |
| 6 | -4.43882933 | 0.10510947 | 1.77168930 |
| 6 | 0.92985481 | 1.16065037 | -1.87211966 |
| 6 | 0.58285563 | 1.35967247 | -3.24012185 |
| 6 | 0.95788867 | 2.57766300 | -3.82605942 |
| 6 | 1.63360815 | 3.58017355 | -3.12572866 |
| 6 | 1.95963594 | 3.37494860 | -1.79014911 |
| 6 | 1.62532700 | 2.17135060 | -1.15799998 |
| 6 | 2.07575426 | 1.94450379 | 0.26304908 |
| 1 | 1.89744537 | 4.51287896 | -3.62406976 |
| 6 | -0.18449787 | 0.29454652 | -4.04990268 |
| 6 | -5.76354911 | 0.16349471 | 2.55601218 |
| 6 | -3.86641173 | -1.31988440 | 1.94308747 |
| 6 | -4.77204430 | 0.35775679 | 0.28142495 |
| 6 | -0.42198185 | 0.73997571 | -5.50563344 |
| 6 | -1.57266680 | 0.04228360 | -3.41537895 |
| 6 | 0.61837431 | -1.02643280 | -4.10292141 |
| 1 | 2.02303751 | 2.37393401 | 2.92541446 |
| 1 | 0.72379218 | 1.20237088 | 3.29960011 |
| 1 | 0.38919566 | 3.65248033 | 1.62183431 |
| 1 | -0.34032284 | 2.89952348 | 0.18383822 |
| 1 | -4.72128794 | 1.93219786 | 3.80533974 |
| 1 | -1.03214381 | 4.07335554 | 3.37938870 |
| 1 | 2.76817185 | 1.09134156 | 0.33304165 |
| 1 | 2.60787284 | 2.83818538 | 0.62819338 |
| 1 | 0.70680455 | 2.76058562 | -4.86899271 |
| 1 | 2.49136045 | 4.14396629 | -1.22550116 |
| 1 | -6.44897506 | -0.59590090 | 2.15393810 |
| 1 | -5.62280768 | -0.05214806 | 3.62524126 |
| 1 | -6.25941940 | 1.14000390 | 2.45966221 |
| 1 | -2.96933962 | -1.46496411 | 1.33314249 |
| 1 | -3.61344476 | -1.51663283 | 2.99532770 |
| 1 | -4.61736082 | -2.06171355 | 1.63197892 |
| 1 | -5.22736703 | 1.34992998 | 0.14928270 |
| 1 | -3.87643411 | 0.29889295 | -0.34720227 |
| 1 | -5.49349794 | -0.39489275 | -0.07110890 |
| 1 | -0.96657633 | -0.05385631 | -6.03607312 |
| 1 | 0.52044921 | 0.91205990 | -6.04556565 |
| 1 | -1.03045604 | 1.65371927 | -5.56598388 |
| 1 | -1.48989317 | -0.32753912 | -2.38794545 |
| 1 | -2.16775013 | 0.96674424 | -3.40355861 |

| | | | |
|---|---|---|---|
| 1 | -2.12098299 | -0.70627164 | -4.00700867 |
| 1 | 1.60421922 | -0.86411937 | -4.56315682 |
| 1 | 0.76119800 | -1.44718425 | -3.10215760 |
| 1 | 0.07816242 | -1.76631827 | -4.71230965 |
| 1 | 2.26316878 | 0.63219560 | 2.57243243 |
| 8 | 0.62618994 | -1.53639919 | 1.31477404 |
| 6 | -0.18883265 | -2.27891569 | 2.22400771 |
| 1 | -0.65552502 | -1.64432364 | 2.99732156 |
| 8 | 4.12456337 | -0.79266264 | 1.99039876 |
| 1 | -0.98792355 | -2.83092900 | 1.70261555 |
| 1 | 0.45250825 | -3.01725696 | 2.73003447 |
| 31 | 0.02623621 | -0.02017565 | 0.50362202 |
| 8 | 3.47931472 | -1.50460918 | -0.01022135 |
| 6 | 3.74591234 | -1.71472891 | 1.29016744 |
| 6 | 3.64896379 | -3.12305670 | 1.85464523 |
| 8 | 2.76344968 | -4.03310509 | 1.14929094 |
| 6 | 2.42001500 | -3.80899110 | -0.14349554 |
| 6 | 2.92382015 | -2.56201884 | -0.85564328 |
| 8 | 1.73108656 | -4.61161577 | -0.73411298 |
| 6 | 3.93736503 | -2.91434545 | -1.94156114 |
| 6 | 5.03895412 | -3.75038378 | 1.98031879 |
| 1 | 3.20103392 | -3.01693254 | 2.85161403 |
| 1 | 2.03550362 | -2.09723777 | -1.30508158 |
| 1 | 4.22018742 | -2.00573885 | -2.48701205 |
| 1 | 3.48085257 | -3.62307987 | -2.64295499 |
| 1 | 4.84249801 | -3.36943575 | -1.51662494 |
| 1 | 5.67132340 | -3.12173794 | 2.61955407 |
| 1 | 5.52006208 | -3.85415538 | 0.99842185 |
| 1 | 4.94769225 | -4.74544413 | 2.43301501 |

Electronic energy = -3673.042180 a.u.
DFT-D3(BJ) dispersion correction = -0.116888 a.u.
Thermal free energy = 0.579184 a.u.
Gibbs free energy = -3672.579884 a.u.
Number of imaginary frequencies = 0.

**RC vdW LA isomer/conformer 6**

| Atomic N. | X | Y | Z |
|---|---|---|---|
| 7 | -0.33773776 | 2.19013227 | -0.16742620 |
| 8 | -1.75312294 | -0.19968112 | 0.96767725 |
| 8 | 0.18347659 | -0.50720436 | -1.40779684 |
| 6 | 0.42237730 | 3.10295820 | 0.72832039 |
| 6 | -2.42248240 | 0.73702739 | 1.69241902 |
| 6 | -3.15600236 | 0.36057956 | 2.85474300 |
| 6 | -3.80755630 | 1.38292651 | 3.56055657 |
| 6 | -3.77270653 | 2.72244744 | 3.16352121 |
| 6 | -3.08903673 | 3.06990768 | 2.00335939 |
| 6 | -2.42876085 | 2.08853408 | 1.25630202 |
| 6 | -1.82079858 | 2.44544222 | -0.07385511 |
| 1 | -4.28926232 | 3.48083620 | 3.75174779 |
| 6 | -3.25128581 | -1.10781283 | 3.31377265 |
| 6 | -0.44411969 | 0.04432360 | -2.47989049 |
| 6 | -0.98717862 | -0.79261495 | -3.49748841 |

| | | | |
|---|---|---|---|
| 6 | -1.60388346 | -0.16079361 | -4.58719954 |
| 6 | -1.70377864 | 1.22846942 | -4.70057044 |
| 6 | -1.16431447 | 2.03314787 | -3.70323638 |
| 6 | -0.52361205 | 1.45711488 | -2.59974471 |
| 6 | 0.14911626 | 2.35505330 | -1.59138901 |
| 1 | -2.20008513 | 1.67254258 | -5.56338898 |
| 6 | -0.91053628 | -2.33003368 | -3.40821077 |
| 6 | -4.13197790 | -1.26420830 | 4.56771863 |
| 6 | -1.84877677 | -1.65369718 | 3.66532770 |
| 6 | -3.88476486 | -1.96707070 | 2.19350212 |
| 6 | -1.53817536 | -3.00955875 | -4.64019572 |
| 6 | -1.68364299 | -2.82950800 | -2.16486758 |
| 6 | 0.56491657 | -2.78993243 | -3.33397489 |
| 1 | 0.25387940 | 4.14693901 | 0.41881220 |
| 1 | 0.07816050 | 2.96921280 | 1.75937001 |
| 1 | -1.99913597 | 3.50954554 | -0.30150082 |
| 1 | -2.27763095 | 1.84620881 | -0.87529216 |
| 1 | -4.36468182 | 1.12906736 | 4.46049397 |
| 1 | -3.07879812 | 4.10488461 | 1.65397685 |
| 1 | 1.23352562 | 2.17106349 | -1.55442699 |
| 1 | -0.00230157 | 3.40907466 | -1.87730280 |
| 1 | -2.03240164 | -0.77273097 | -5.37872662 |
| 1 | -1.22237357 | 3.12137620 | -3.77796097 |
| 1 | -4.17855847 | -2.32823593 | 4.83992680 |
| 1 | -3.72418502 | -0.72003578 | 5.43199218 |
| 1 | -5.16241126 | -0.92177409 | 4.39477340 |
| 1 | -1.18821636 | -1.63904744 | 2.79233314 |
| 1 | -1.38541450 | -1.05689264 | 4.46431631 |
| 1 | -1.92992075 | -2.69252956 | 4.01965444 |
| 1 | -4.90226263 | -1.61694378 | 1.96652390 |
| 1 | -3.29097164 | -1.93044139 | 1.27347111 |
| 1 | -3.95386291 | -3.01510093 | 2.52268026 |
| 1 | -1.44813299 | -4.09959258 | -4.53037921 |
| 1 | -1.02864770 | -2.72778506 | -5.57311355 |
| 1 | -2.60774833 | -2.77627285 | -4.74288728 |
| 1 | -1.26522306 | -2.42626885 | -1.23672075 |
| 1 | -2.74165418 | -2.53599380 | -2.22209239 |
| 1 | -1.63847302 | -3.92807013 | -2.11545392 |
| 1 | 1.11603135 | -2.46788155 | -4.22995563 |
| 1 | 1.06777615 | -2.38511625 | -2.44927468 |
| 1 | 0.60939228 | -3.88863691 | -3.28738539 |
| 1 | 1.49044376 | 2.86827776 | 0.66365842 |
| 8 | 1.39041352 | 0.07851812 | 1.35974392 |
| 6 | 1.81654938 | -1.20493876 | 1.82419421 |
| 1 | 2.66918931 | -1.05510628 | 2.50367365 |
| 8 | 3.51905412 | 2.30612889 | -0.80255498 |
| 1 | 1.02949812 | -1.73234635 | 2.39101508 |
| 1 | 2.15024085 | -1.85913598 | 1.00006530 |
| 31 | -0.06774892 | 0.22859281 | 0.28574091 |
| 8 | 4.88413772 | 1.72201943 | 0.86216391 |
| 6 | 4.31001327 | 1.50646265 | -0.34852432 |
| 6 | 4.76146770 | 0.23229725 | -1.06429669 |
| 8 | 4.87267197 | -0.88057862 | -0.12371081 |

| | | | |
|---|---|---|---|
| 6 | 5.52187603 | -0.65159699 | 1.05046571 |
| 6 | 5.94261794 | 0.80235474 | 1.27943471 |
| 8 | 5.74166984 | -1.55548224 | 1.82324878 |
| 6 | 6.26133156 | 1.10174133 | 2.72959161 |
| 6 | 3.81846184 | -0.19444932 | -2.17051545 |
| 1 | 5.77043536 | 0.42895376 | -1.47492431 |
| 1 | 6.82699169 | 0.99788891 | 0.64435073 |
| 1 | 6.57347616 | 2.14772928 | 2.83560511 |
| 1 | 7.07061992 | 0.44563217 | 3.06982718 |
| 1 | 5.38153902 | 0.91947182 | 3.35827827 |
| 1 | 3.75042603 | 0.60096878 | -2.92200202 |
| 1 | 2.81432341 | -0.38538510 | -1.77129243 |
| 1 | 4.19825365 | -1.10536734 | -2.64882054 |

Electronic energy = -3673.042664 a.u.
DFT-D3(BJ) dispersion correction = -0.113841 a.u.
Thermal free energy = 0.580721 a.u.
Gibbs free energy = -3672.575784 a.u.
Number of imaginary frequencies = 0.

**RC vdW LA isomer/conformer 7**

| Atomic N. | X | Y | Z |
|---|---|---|---|
| 7 | -0.12564996 | 2.60911400 | 1.77236436 |
| 8 | -1.44216258 | 0.03722009 | 0.99931441 |
| 8 | 0.78337812 | 1.32945036 | -0.78754072 |
| 6 | 0.40653991 | 2.71037900 | 3.15353132 |
| 6 | -2.27999076 | 0.35165167 | 2.02280252 |
| 6 | -3.10380293 | -0.65616583 | 2.60240076 |
| 6 | -3.94411292 | -0.26774887 | 3.65623294 |
| 6 | -4.00441520 | 1.04317890 | 4.13744398 |
| 6 | -3.21840476 | 2.02308418 | 3.54128728 |
| 6 | -2.36893513 | 1.69263824 | 2.47936441 |
| 6 | -1.62269288 | 2.78133209 | 1.75620762 |
| 1 | -4.67005612 | 1.29240485 | 4.96377885 |
| 6 | -3.08588928 | -2.11021130 | 2.09173547 |
| 6 | 0.27123020 | 2.45618540 | -1.34425565 |
| 6 | -0.05604570 | 2.48209302 | -2.73104627 |
| 6 | -0.56679880 | 3.68244927 | -3.24533743 |
| 6 | -0.76088975 | 4.82389804 | -2.46158105 |
| 6 | -0.42697120 | 4.78839502 | -1.11279035 |
| 6 | 0.09855688 | 3.61921417 | -0.54950972 |
| 6 | 0.54913137 | 3.63128595 | 0.88775922 |
| 1 | -1.16803532 | 5.73093006 | -2.90813610 |
| 6 | 0.13954439 | 1.24658215 | -3.63154029 |
| 6 | -4.09089201 | -2.99703183 | 2.85096050 |
| 6 | -1.68269346 | -2.72964767 | 2.28685633 |
| 6 | -3.47395476 | -2.15003343 | 0.59416965 |
| 6 | -0.25604481 | 1.53404948 | -5.09239074 |
| 6 | -0.74338428 | 0.07664151 | -3.13686169 |
| 6 | 1.62754459 | 0.82140769 | -3.63351509 |
| 1 | 0.20882573 | 3.71547435 | 3.56045949 |
| 1 | -0.07818553 | 1.96153468 | 3.78886870 |
| 1 | -1.86269093 | 3.76431363 | 2.19570261 |

| | | | |
|---|---|---|---|
| 1 | -1.90840833 | 2.80821669 | 0.69432109 |
| 1 | -4.57790734 | -1.01674673 | 4.12731973 |
| 1 | -3.27135970 | 3.06008669 | 3.88081653 |
| 1 | 1.62758836 | 3.41621992 | 0.95862469 |
| 1 | 0.37500381 | 4.62925829 | 1.32382629 |
| 1 | -0.83253711 | 3.73292377 | -4.29959822 |
| 1 | -0.55878355 | 5.67271291 | -0.48524810 |
| 1 | -4.04842408 | -4.01501688 | 2.43840593 |
| 1 | -3.85764451 | -3.06578418 | 3.92361708 |
| 1 | -5.12491587 | -2.63911485 | 2.74126418 |
| 1 | -0.92101127 | -2.19843917 | 1.70777565 |
| 1 | -1.39460936 | -2.71498453 | 3.34844271 |
| 1 | -1.69441102 | -3.77772562 | 1.95095973 |
| 1 | -4.48470854 | -1.74254279 | 0.44508587 |
| 1 | -2.77041621 | -1.57876841 | -0.02043922 |
| 1 | -3.47303749 | -3.19177083 | 0.23975337 |
| 1 | -0.08563782 | 0.62797519 | -5.69084445 |
| 1 | 0.34589824 | 2.34133312 | -5.53461824 |
| 1 | -1.31914686 | 1.79771206 | -5.19019498 |
| 1 | -0.47195461 | -0.24632578 | -2.12701429 |
| 1 | -1.80500150 | 0.36341997 | -3.13896193 |
| 1 | -0.62387718 | -0.78679754 | -3.80792700 |
| 1 | 2.26200056 | 1.63084266 | -4.02418239 |
| 1 | 1.97068254 | 0.56086560 | -2.62663221 |
| 1 | 1.75887725 | -0.05759416 | -4.28253473 |
| 1 | 1.48720918 | 2.52685888 | 3.14372392 |
| 8 | 1.55538761 | -0.16383212 | 1.75795003 |
| 6 | 2.91862344 | 0.20663611 | 1.52982476 |
| 1 | 3.21519011 | 1.08069696 | 2.14064968 |
| 8 | 0.67836867 | -2.98414655 | -0.88330628 |
| 1 | 3.12693936 | 0.44457492 | 0.47264407 |
| 1 | 3.56091008 | -0.63665507 | 1.82350240 |
| 31 | 0.25256378 | 0.77664382 | 0.90525272 |
| 8 | 2.55759138 | -2.79361668 | -2.08101221 |
| 6 | 1.87464928 | -3.14296124 | -0.95181037 |
| 6 | 2.74139371 | -3.73977772 | 0.15994895 |
| 8 | 3.99788656 | -2.99811690 | 0.28634162 |
| 6 | 4.68724101 | -2.72884900 | -0.85645080 |
| 6 | 3.97361815 | -3.14639081 | -2.14450656 |
| 8 | 5.77676969 | -2.20438485 | -0.81216829 |
| 6 | 4.55475036 | -2.49081103 | -3.38030705 |
| 6 | 2.06091304 | -3.72406760 | 1.51294940 |
| 1 | 2.99246779 | -4.77638684 | -0.13440247 |
| 1 | 4.04760923 | -4.24737160 | -2.22561161 |
| 1 | 4.01992351 | -2.83780090 | -4.27272477 |
| 1 | 5.61591248 | -2.75058626 | -3.46966877 |
| 1 | 4.47026245 | -1.39975216 | -3.30969845 |
| 1 | 1.14121696 | -4.31883639 | 1.46545285 |
| 1 | 1.79525948 | -2.69724959 | 1.79730544 |
| 1 | 2.72903223 | -4.15784164 | 2.26736939 |

Electronic energy = -3673.038917 a.u.
DFT-D3(BJ) dispersion correction = -0.113755 a.u.
Thermal free energy = 0.578681 a.u.

Gibbs free energy = -3672.573991 a.u.
Number of imaginary frequencies = 0.

**RC vdW LA isomer/conformer 8**

| Atomic N. | X | Y | Z |
|---|---|---|---|
| 8 | 0.03711439 | -3.59383130 | -1.37280362 |
| 6 | -1.06403349 | -5.44806620 | -0.15588841 |
| 8 | -0.04652390 | -6.33921761 | -0.70001449 |
| 6 | 1.14020275 | -5.84026646 | -1.13101377 |
| 6 | 1.29260307 | -4.32951920 | -1.25324706 |
| 8 | 2.04767364 | -6.58771631 | -1.42370554 |
| 6 | 2.15497548 | -3.74533147 | -0.13529912 |
| 6 | -1.01482949 | -5.45377657 | 1.37116562 |
| 1 | -2.01103569 | -5.89595667 | -0.48630631 |
| 1 | 1.78828859 | -4.16008729 | -2.21812017 |
| 1 | 2.29884967 | -2.67207354 | -0.31134900 |
| 1 | 3.12936128 | -4.25057430 | -0.13461091 |
| 1 | 1.68453700 | -3.86627771 | 0.84839464 |
| 1 | -1.86806855 | -4.88582506 | 1.76265522 |
| 1 | -0.08904499 | -5.00161823 | 1.74903494 |
| 1 | -1.07776486 | -6.48781057 | 1.73229075 |
| 7 | 1.49964163 | 1.44991944 | 2.04169947 |
| 8 | -1.01557057 | 1.27843907 | 0.41418981 |
| 8 | 1.61313873 | -0.05197648 | -0.56065642 |
| 6 | 1.48379048 | 1.03840370 | 3.46704378 |
| 6 | -1.48998895 | 2.15181022 | 1.34324719 |
| 6 | -2.89239325 | 2.37446016 | 1.46434506 |
| 6 | -3.31337862 | 3.25910396 | 2.46866617 |
| 6 | -2.42473441 | 3.92956187 | 3.31401921 |
| 6 | -1.05633420 | 3.75046563 | 3.14241982 |
| 6 | -0.58017464 | 2.88264025 | 2.15309449 |
| 6 | 0.89677422 | 2.81878894 | 1.86432594 |
| 6 | -3.90713972 | 1.70783107 | 0.51336034 |
| 6 | 2.40084962 | 0.99362093 | -0.93340105 |
| 6 | 2.59762673 | 1.29110422 | -2.31281815 |
| 6 | 3.44468826 | 2.36617987 | -2.62024352 |
| 6 | 4.08058300 | 3.13732826 | -1.64336887 |
| 6 | 3.88078063 | 2.83553271 | -0.30134009 |
| 6 | 3.05505055 | 1.76512360 | 0.06171325 |
| 6 | 2.91514799 | 1.39990978 | 1.51613860 |
| 6 | 1.90879791 | 0.47977261 | -3.42834667 |
| 6 | -5.35109570 | 2.15185110 | 0.81511168 |
| 6 | -3.86200144 | 0.16862491 | 0.64462274 |
| 6 | -3.59325956 | 2.11642425 | -0.94626574 |
| 6 | 2.29491189 | 0.98719946 | -4.83061845 |
| 6 | 0.37135384 | 0.59639600 | -3.30344293 |
| 6 | 2.33075426 | -1.00562701 | -3.34322593 |
| 1 | 2.15425123 | 1.68731580 | 4.05361458 |
| 1 | 0.46474458 | 1.12799338 | 3.85978260 |
| 1 | 1.44233511 | 3.52616619 | 2.51152187 |
| 1 | 1.09294576 | 3.09712690 | 0.81860104 |
| 1 | -4.37900023 | 3.43695202 | 2.60014036 |

```
   1    -0.34245026   4.30145477   3.75906062
   1     3.25817489   0.36821898   1.69448530
   1     3.53372293   2.07393116   2.13202786
   1     3.61257017   2.62208166  -3.66458388
   1     4.37282411   3.42095749   0.47855527
   1    -6.02863027   1.66842253   0.09732595
   1    -5.67464120   1.85627228   1.82394198
   1    -5.48106228   3.23883017   0.71179433
   1    -2.89188880  -0.23803883   0.34250312
   1    -4.06968581  -0.14133314   1.67946193
   1    -4.62986491  -0.28165688  -0.00176473
   1    -3.66933381   3.20671602  -1.06887638
   1    -2.58796839   1.79973814  -1.24544610
   1    -4.32015405   1.64767648  -1.62653922
   1     1.78616882   0.36964355  -5.58414640
   1     3.37614553   0.91193745  -5.01656108
   1     1.98410207   2.02908763  -4.99470185
   1     0.00465235   0.17513313  -2.36191472
   1     0.05409782   1.64748309  -3.36182156
   1    -0.10903385   0.05077903  -4.12932080
   1     3.41914859  -1.11071522  -3.46211056
   1     2.03616373  -1.44217414  -2.38343523
   1     1.84453426  -1.57551939  -4.14938737
   1     1.80815863  -0.00536424   3.54681462
   8    -2.05431230  -3.33450185  -0.64333560
   8     0.17078117  -1.39185886   1.73584846
   6    -1.04091971  -1.62948366   2.45671140
   1    -1.41200394  -0.73808621   2.99417337
   1    -1.84494387  -1.98214159   1.79048032
   1    -0.84101409  -2.41156103   3.20701890
  31     0.41772373   0.17021193   0.83977709
   6    -1.06701775  -4.03437644  -0.72979110
   1    -2.80419035   4.60118099   4.08402820
   1     4.72474686   3.96656065  -1.93538510
Electronic energy = -3673.035876 a.u.
DFT-D3(BJ) dispersion correction = -0.115157 a.u.
Thermal free energy = 0.577532 a.u.
Gibbs free energy = -3672.573500 a.u.
Number of imaginary frequencies = 0.
```

**<u>RC vdW LA isomer/conformer 9</u>**

```
Atomic N.      X             Y             Z
   7    -0.53922616   3.27053935  -0.26467154
   8     0.61434283   0.70213069  -1.26983124
   8    -0.00905513   1.14525899   1.78270692
   6    -1.72234134   3.77540796  -1.00362593
   6     0.81831802   1.43113417  -2.39869254
   6     1.06379968   0.76737643  -3.63643659
   6     1.27049347   1.57016813  -4.76757026
   6     1.24848971   2.96658686  -4.72179986
   6     1.03316536   3.60199378  -3.50409541
   6     0.83351593   2.84945339  -2.34117224
```

| | | | |
|---|---|---|---|
| 6 | 0.73508770 | 3.55378768 | -1.01484801 |
| 1 | 1.40819110 | 3.54685635 | -5.63043957 |
| 6 | 1.10371759 | -0.76995205 | -3.73698594 |
| 6 | 0.83951256 | 2.07454014 | 2.28940563 |
| 6 | 1.88155633 | 1.67737072 | 3.17703454 |
| 6 | 2.71256288 | 2.68861561 | 3.68020955 |
| 6 | 2.55636200 | 4.03771892 | 3.34761548 |
| 6 | 1.52975930 | 4.41242869 | 2.48894220 |
| 6 | 0.66277957 | 3.44493082 | 1.96644182 |
| 6 | -0.50125148 | 3.88102378 | 1.11671049 |
| 1 | 3.23376725 | 4.78473896 | 3.76111725 |
| 6 | 2.09085460 | 0.20184549 | 3.57064577 |
| 6 | 1.42666426 | -1.24801147 | -5.16522830 |
| 6 | -0.27524617 | -1.35672866 | -3.35662223 |
| 6 | 2.19900180 | -1.33808655 | -2.80317574 |
| 6 | 3.26249647 | 0.03352218 | 4.55670610 |
| 6 | 2.41753771 | -0.64474423 | 2.31749520 |
| 6 | 0.82133035 | -0.35112406 | 4.26122712 |
| 1 | -1.67309344 | 4.87317032 | -1.08921319 |
| 1 | -1.74339485 | 3.33495216 | -2.00612673 |
| 1 | 0.81887632 | 4.64444066 | -1.15798335 |
| 1 | 1.55097430 | 3.24073511 | -0.34656603 |
| 1 | 1.45224356 | 1.09069690 | -5.72757009 |
| 1 | 1.03804598 | 4.69246081 | -3.43921005 |
| 1 | -1.45656519 | 3.60042206 | 1.58939813 |
| 1 | -0.49031370 | 4.97830038 | 1.00562260 |
| 1 | 3.52140645 | 2.41734385 | 4.35606833 |
| 1 | 1.38240682 | 5.46210630 | 2.22489381 |
| 1 | 1.45316015 | -2.34692520 | -5.17575887 |
| 1 | 0.66570958 | -0.92991857 | -5.89250710 |
| 1 | 2.40810655 | -0.89054938 | -5.50855515 |
| 1 | -0.55497011 | -1.08771298 | -2.33232922 |
| 1 | -1.05636031 | -0.98730348 | -4.03697396 |
| 1 | -0.24627748 | -2.45453431 | -3.44058004 |
| 1 | 3.18865479 | -0.95579204 | -3.09254487 |
| 1 | 2.01677461 | -1.07202509 | -1.75642251 |
| 1 | 2.22524640 | -2.43539600 | -2.88691217 |
| 1 | 3.36131786 | -1.03108876 | 4.81123208 |
| 1 | 3.09721931 | 0.58339105 | 5.49472999 |
| 1 | 4.21998263 | 0.35982459 | 4.12496489 |
| 1 | 1.58879764 | -0.64628737 | 1.60235795 |
| 1 | 3.31901156 | -0.26564223 | 1.81431499 |
| 1 | 2.60818679 | -1.68619640 | 2.61694163 |
| 1 | 0.58834920 | 0.22913133 | 5.16636582 |
| 1 | -0.04433442 | -0.32816815 | 3.59154932 |
| 1 | 0.99150544 | -1.39536991 | 4.56307285 |
| 1 | -2.63503161 | 3.48736903 | -0.46947219 |
| 8 | -2.28353238 | 0.57643265 | -0.22008448 |
| 6 | -3.14664636 | 0.47801809 | 0.91666429 |
| 1 | -3.46891502 | 1.46992719 | 1.28561704 |
| 8 | -1.01828131 | -2.31856184 | 1.43279996 |
| 1 | -2.68069539 | -0.06612301 | 1.75414657 |
| 1 | -4.05179683 | -0.06870623 | 0.60796369 |

| | | | |
|---|---|---|---|
| 31 | -0.60287920 | 1.23120241 | 0.02845844 |
| 8 | -1.86868559 | -4.31434658 | 1.93951882 |
| 6 | -1.49612971 | -3.36369033 | 1.04637543 |
| 6 | -1.67696296 | -3.64464670 | -0.43924615 |
| 8 | -1.76496105 | -5.06030714 | -0.79116852 |
| 6 | -2.32442589 | -5.94280658 | 0.06942379 |
| 6 | -2.60921128 | -5.49028874 | 1.49776333 |
| 8 | -2.57249151 | -7.07628663 | -0.28104536 |
| 6 | -4.10464860 | -5.30715256 | 1.75391778 |
| 6 | -2.84917959 | -2.86606398 | -1.03271946 |
| 1 | -0.74603888 | -3.31184428 | -0.91665105 |
| 1 | -2.23109650 | -6.29753378 | 2.13932789 |
| 1 | -4.26595862 | -5.05886738 | 2.81018228 |
| 1 | -4.62839282 | -6.24299321 | 1.52185600 |
| 1 | -4.52680098 | -4.50454161 | 1.13532705 |
| 1 | -2.68476051 | -1.78948617 | -0.88132648 |
| 1 | -3.80354116 | -3.15368969 | -0.57132921 |
| 1 | -2.90578884 | -3.06786066 | -2.10962119 |

Electronic energy = -3673.035856 a.u.
DFT-D3(BJ) dispersion correction = -0.113510 a.u.
Thermal free energy = 0.578954 a.u.
Gibbs free energy = -3672.570411 a.u.
Number of imaginary frequencies = 0.

## Initiation step with axial LA addition

**TS1 ax LA isomer/conformer 1**

| Atomic N. | X | Y | Z |
|---|---|---|---|
| 7 | 0.30682507 | 1.96146405 | 1.53897429 |
| 8 | -1.32642376 | -0.38757351 | 0.88427657 |
| 8 | 0.63330120 | 0.96321100 | -1.25613189 |
| 6 | 0.93847781 | 1.87572932 | 2.87749004 |
| 6 | -2.07642055 | 0.00398380 | 1.93640828 |
| 6 | -3.02564263 | -0.89600313 | 2.50930762 |
| 6 | -3.73969626 | -0.44610351 | 3.62936711 |
| 6 | -3.57738709 | 0.83163399 | 4.17350769 |
| 6 | -2.69815230 | 1.72066450 | 3.56458259 |
| 6 | -1.96442223 | 1.32211125 | 2.44231534 |
| 6 | -1.14579082 | 2.32313347 | 1.68348551 |
| 1 | -4.15199089 | 1.13026547 | 5.05022478 |
| 6 | -3.29323912 | -2.28739595 | 1.90247142 |
| 6 | 0.25759140 | 2.22007108 | -1.57908535 |
| 6 | -0.23568764 | 2.49230552 | -2.89013720 |
| 6 | -0.59365690 | 3.81583500 | -3.18117189 |
| 6 | -0.48904106 | 4.85241270 | -2.24867225 |
| 6 | -0.00215054 | 4.57509136 | -0.97657377 |
| 6 | 0.38342702 | 3.27268076 | -0.63656038 |
| 6 | 0.99693471 | 3.02027655 | 0.71631217 |
| 1 | -0.78768861 | 5.86492300 | -2.51983976 |
| 6 | -0.37144759 | 1.37757218 | -3.94647765 |
| 6 | -4.38967752 | -3.05021501 | 2.66898489 |

| | | | |
|---|---|---|---|
| 6 | -2.01368403 | -3.15316081 | 1.93600118 |
| 6 | -3.77230740 | -2.12894297 | 0.43922848 |
| 6 | -0.90487683 | 1.91913998 | -5.28621960 |
| 6 | -1.36481702 | 0.29667124 | -3.45758963 |
| 6 | 1.00919211 | 0.73585331 | -4.22505331 |
| 1 | 0.87663925 | 2.85175014 | 3.38753176 |
| 1 | 0.41244194 | 1.12811410 | 3.48140192 |
| 1 | -1.20501494 | 3.31160116 | 2.17184496 |
| 1 | -1.53233743 | 2.43664336 | 0.66009754 |
| 1 | -4.45712662 | -1.11600673 | 4.09988403 |
| 1 | -2.58955765 | 2.74088192 | 3.94015169 |
| 1 | 2.04371381 | 2.70073295 | 0.61452752 |
| 1 | 0.99145108 | 3.95802879 | 1.29860365 |
| 1 | -0.97697943 | 4.05334234 | -4.17183685 |
| 1 | 0.09403590 | 5.37145662 | -0.23489253 |
| 1 | -4.55205297 | -4.02330685 | 2.18390512 |
| 1 | -4.10709858 | -3.24470711 | 3.71404076 |
| 1 | -5.34933186 | -2.51367950 | 2.66219521 |
| 1 | -1.22348571 | -2.70616198 | 1.32331765 |
| 1 | -1.65143239 | -3.26906514 | 2.96890020 |
| 1 | -2.23393607 | -4.15709782 | 1.54128623 |
| 1 | -4.70933342 | -1.55468297 | 0.39926320 |
| 1 | -3.02101183 | -1.61721694 | -0.17238101 |
| 1 | -3.96317244 | -3.11998677 | -0.00000468 |
| 1 | -0.97509736 | 1.08908665 | -6.00348568 |
| 1 | -0.23791648 | 2.67732978 | -5.72187853 |
| 1 | -1.90968144 | 2.35477352 | -5.18687105 |
| 1 | -1.01699007 | -0.18858387 | -2.53983331 |
| 1 | -2.35482168 | 0.73641670 | -3.26814580 |
| 1 | -1.48128986 | -0.47658050 | -4.23215437 |
| 1 | 1.71522582 | 1.48759985 | -4.60807848 |
| 1 | 1.43071461 | 0.28491804 | -3.32020974 |
| 1 | 0.90394604 | -0.04868601 | -4.99011660 |
| 1 | 1.98838341 | 1.58257902 | 2.77900910 |
| 8 | 2.26690578 | -0.35445699 | 0.97792554 |
| 6 | 3.43000960 | 0.41302431 | 0.66363651 |
| 1 | 3.48353076 | 1.30750191 | 1.30463207 |
| 8 | 0.72580576 | -1.77870953 | -0.14627914 |
| 1 | 3.44146419 | 0.73217547 | -0.39051804 |
| 1 | 4.32670307 | -0.18535845 | 0.87941241 |
| 31 | 0.41026565 | 0.15392232 | 0.40855497 |
| 8 | 2.74050689 | -1.77300012 | -1.15709138 |
| 6 | 1.98193264 | -1.96222484 | -0.03675060 |
| 6 | 2.52464175 | -3.03478146 | 0.90903378 |
| 8 | 3.96519923 | -2.89073682 | 1.09490049 |
| 6 | 4.72414494 | -2.71693563 | -0.02149704 |
| 6 | 3.92975879 | -2.60524607 | -1.32327712 |
| 8 | 5.93109798 | -2.66265917 | 0.05426492 |
| 6 | 4.74048662 | -2.02582145 | -2.46421933 |
| 6 | 1.89378905 | -3.06495070 | 2.28474998 |
| 1 | 2.34106493 | -3.99822903 | 0.39449850 |
| 1 | 3.58318853 | -3.62316298 | -1.58517611 |
| 1 | 4.12087822 | -1.96830487 | -3.36725807 |

| | | | |
|---|---|---|---|
| 1 | 5.60855770 | -2.66595292 | -2.65957808 |
| 1 | 5.10278845 | -1.02257228 | -2.20967770 |
| 1 | 0.80957592 | -3.19427681 | 2.19335556 |
| 1 | 2.10179806 | -2.12863016 | 2.81432033 |
| 1 | 2.30547285 | -3.90574121 | 2.85653996 |

Electronic energy = -3673.022217 a.u.
DFT-D3(BJ) dispersion correction = -0.119086 a.u.
Thermal free energy = 0.585298 a.u.
Gibbs free energy = -3672.556004 a.u.
Number of imaginary frequencies = 1.

**TS1 ax LA isomer/conformer 2**

| Atomic N. | X | Y | Z |
|---|---|---|---|
| 7 | 0.79303670 | 2.06523085 | 1.32340753 |
| 8 | -1.25926685 | 0.22807597 | 0.07782372 |
| 8 | 1.60028446 | 0.75431067 | -1.18350422 |
| 6 | 1.00072920 | 2.01107502 | 2.78928328 |
| 6 | -2.10133719 | 0.87112212 | 0.92059678 |
| 6 | -3.38583350 | 0.31642973 | 1.20268953 |
| 6 | -4.19508362 | 1.00739288 | 2.11640406 |
| 6 | -3.80494714 | 2.20408177 | 2.72502995 |
| 6 | -2.57619238 | 2.76500320 | 2.39356518 |
| 6 | -1.72992146 | 2.11982679 | 1.48441577 |
| 6 | -0.47938445 | 2.80642939 | 1.00915945 |
| 6 | -3.88238311 | -0.97072947 | 0.51425749 |
| 6 | 1.69584012 | 2.01669669 | -1.65172923 |
| 6 | 1.67115426 | 2.26860714 | -3.05547190 |
| 6 | 1.80206347 | 3.60324121 | -3.46504211 |
| 6 | 1.95100689 | 4.66505766 | -2.56643629 |
| 6 | 1.98023069 | 4.40296555 | -1.20126796 |
| 6 | 1.86448085 | 3.08618808 | -0.74211413 |
| 6 | 1.97855651 | 2.78478575 | 0.72263533 |
| 6 | 1.50828004 | 1.12871134 | -4.07933521 |
| 6 | -5.32150126 | -1.32707775 | 0.93231767 |
| 6 | -2.98240113 | -2.17141008 | 0.88614291 |
| 6 | -3.88312483 | -0.77492517 | -1.02112043 |
| 6 | 1.53910589 | 1.65018308 | -5.52826834 |
| 6 | 0.15078624 | 0.41603020 | -3.87273264 |
| 6 | 2.66315794 | 0.10997231 | -3.92866802 |
| 1 | 1.16489983 | 3.02995038 | 3.17872572 |
| 1 | 0.11698559 | 1.59042180 | 3.27694225 |
| 1 | -0.41146895 | 3.81620169 | 1.44991362 |
| 1 | -0.49958956 | 2.92291713 | -0.08400405 |
| 1 | -5.17372799 | 0.60091507 | 2.36501932 |
| 1 | -2.27117205 | 3.72406840 | 2.81873870 |
| 1 | 2.84986544 | 2.14029503 | 0.92438650 |
| 1 | 2.12034845 | 3.72526526 | 1.28207970 |
| 1 | 1.78230906 | 3.83169317 | -4.52914858 |
| 1 | 2.10348797 | 5.21507215 | -0.48103231 |
| 1 | -5.63202429 | -2.23658705 | 0.39879167 |
| 1 | -5.40328550 | -1.53282213 | 2.00976181 |
| 1 | -6.03630304 | -0.53234363 | 0.67457664 |

| | | | |
|---|---|---|---|
| 1 | -1.94954669 | -2.02198982 | 0.55848318 |
| 1 | -2.99081492 | -2.33834553 | 1.97401571 |
| 1 | -3.36393213 | -3.08415198 | 0.40336010 |
| 1 | -4.55148983 | 0.05085845 | -1.30542219 |
| 1 | -2.87832952 | -0.55891830 | -1.39998329 |
| 1 | -4.24931335 | -1.68989944 | -1.51084721 |
| 1 | 1.42958498 | 0.79927313 | -6.21541166 |
| 1 | 2.48919177 | 2.14921308 | -5.76863146 |
| 1 | 0.71589446 | 2.34936237 | -5.73539829 |
| 1 | 0.08263450 | -0.04911801 | -2.88389611 |
| 1 | -0.68273187 | 1.12458909 | -3.98475003 |
| 1 | 0.02837889 | -0.37327722 | -4.62975740 |
| 1 | 3.63443542 | 0.59566414 | -4.10410070 |
| 1 | 2.66866231 | -0.34039021 | -2.93029976 |
| 1 | 2.54798865 | -0.69334376 | -4.67198896 |
| 1 | 1.87435174 | 1.38951838 | 3.01535463 |
| 8 | 1.06368434 | -1.67724764 | -0.26227573 |
| 8 | 1.05016565 | -0.91324276 | 1.95961893 |
| 6 | 0.16062076 | -1.14846742 | 3.05821538 |
| 1 | -0.67282164 | -0.43267566 | 3.05062222 |
| 1 | -0.28150883 | -2.15219116 | 2.96843863 |
| 1 | 0.70592761 | -1.06475699 | 4.01185513 |
| 31 | 0.59324658 | 0.17805503 | 0.28951856 |
| 1 | -4.46912003 | 2.69976664 | 3.43295574 |
| 1 | 2.04302979 | 5.68573570 | -2.93749809 |
| 8 | 0.52545482 | -3.31840539 | 1.16916478 |
| 6 | 1.37065935 | -2.29915881 | 0.82081735 |
| 6 | 2.84263778 | -2.58799720 | 1.18982971 |
| 8 | 3.16727924 | -3.95171714 | 0.76212120 |
| 6 | 2.35041451 | -4.93352556 | 1.23832750 |
| 6 | 1.09582365 | -4.41283512 | 1.94980109 |
| 8 | 2.62244081 | -6.10197592 | 1.08613622 |
| 6 | 0.02420662 | -5.47249937 | 2.10179949 |
| 6 | 3.82956076 | -1.66250694 | 0.51340938 |
| 1 | 2.95451069 | -2.54464865 | 2.28392029 |
| 1 | 1.40051138 | -4.03087234 | 2.94219732 |
| 1 | -0.84894252 | -5.05399990 | 2.61767530 |
| 1 | 0.42017835 | -6.31338024 | 2.68290257 |
| 1 | -0.28319842 | -5.84765959 | 1.11859667 |
| 1 | 3.69335914 | -0.63745880 | 0.88098019 |
| 1 | 3.67647958 | -1.66447556 | -0.57144588 |
| 1 | 4.85277462 | -1.98322606 | 0.74479997 |

Electronic energy = -3673.021191 a.u.
DFT-D3(BJ) dispersion correction = -0.119626 a.u.
Thermal free energy = 0.585753 a.u.
Gibbs free energy = -3672.555064 a.u.
Number of imaginary frequencies = 1.

**TS1 ax LA isomer/conformer 3**

| Atomic N. | X | Y | Z |
|---|---|---|---|
| 7 | 0.50082840 | 0.27300815 | 2.42160341 |
| 8 | 1.31275716 | 0.52639805 | -0.46373602 |

| | | | |
|---|---|---|---|
| 8 | -0.66114051 | -1.75619740 | 0.62529316 |
| 6 | 0.17992233 | 1.52440967 | 3.14919134 |
| 6 | 2.29291980 | 1.30248555 | 0.05715193 |
| 6 | 3.01869567 | 2.18746359 | -0.79415132 |
| 6 | 4.00037358 | 2.98961171 | -0.19412084 |
| 6 | 4.29646204 | 2.93482788 | 1.17118964 |
| 6 | 3.61551399 | 2.03024264 | 1.97894180 |
| 6 | 2.62830216 | 1.20290852 | 1.43148800 |
| 6 | 1.99325776 | 0.13218153 | 2.27473725 |
| 6 | 2.76278728 | 2.24185638 | -2.31396980 |
| 6 | -0.02137624 | -2.63284561 | 1.43207020 |
| 6 | 0.26723590 | -3.95312882 | 0.97716934 |
| 6 | 0.91040617 | -4.81155476 | 1.88050541 |
| 6 | 1.26928855 | -4.42355586 | 3.17542027 |
| 6 | 0.97830573 | -3.13368762 | 3.60535269 |
| 6 | 0.32737626 | -2.24226463 | 2.74531541 |
| 6 | -0.05903908 | -0.87567799 | 3.22894632 |
| 6 | -0.10524632 | -4.42068121 | -0.44313801 |
| 6 | 3.70110749 | 3.24071310 | -3.01731764 |
| 6 | 1.31125438 | 2.68386411 | -2.61252444 |
| 6 | 3.02096542 | 0.84805364 | -2.93567276 |
| 6 | 0.29240358 | -5.88839114 | -0.68788625 |
| 6 | 0.63024302 | -3.56031411 | -1.49783687 |
| 6 | -1.63435750 | -4.31423619 | -0.65247699 |
| 1 | 0.56750811 | 1.46186456 | 4.17981261 |
| 1 | 0.65386914 | 2.37634260 | 2.65332614 |
| 1 | 2.44329992 | 0.12653900 | 3.28249809 |
| 1 | 2.16384809 | -0.85916214 | 1.83057324 |
| 1 | 4.56207937 | 3.68665955 | -0.81325729 |
| 1 | 3.85870868 | 1.94136844 | 3.04026594 |
| 1 | -1.15329217 | -0.74507978 | 3.21069849 |
| 1 | 0.27592002 | -0.74668185 | 4.27215743 |
| 1 | 1.14970040 | -5.82529077 | 1.56448325 |
| 1 | 1.24397372 | -2.81030454 | 4.61435438 |
| 1 | 3.49107755 | 3.22759519 | -4.09616752 |
| 1 | 3.55063552 | 4.27156353 | -2.66434920 |
| 1 | 4.76044498 | 2.97822585 | -2.88385898 |
| 1 | 0.58103150 | 1.97035786 | -2.21609799 |
| 1 | 1.11299711 | 3.67825484 | -2.18459579 |
| 1 | 1.16244836 | 2.75284970 | -3.70078272 |
| 1 | 4.06223913 | 0.53646069 | -2.76818356 |
| 1 | 2.35509690 | 0.08883446 | -2.51101853 |
| 1 | 2.85096041 | 0.88957805 | -4.02219402 |
| 1 | -0.00329610 | -6.17251228 | -1.70774700 |
| 1 | -0.21224650 | -6.57574990 | 0.00650699 |
| 1 | 1.37788902 | -6.04324167 | -0.60428428 |
| 1 | 0.33628464 | -2.50716487 | -1.43961798 |
| 1 | 1.71959454 | -3.62817017 | -1.36428831 |
| 1 | 0.38975813 | -3.92619433 | -2.50750356 |
| 1 | -2.17031628 | -4.95110714 | 0.06653475 |
| 1 | -1.97802105 | -3.28111437 | -0.53266446 |
| 1 | -1.89525118 | -4.65409435 | -1.66625070 |
| 1 | -0.90535517 | 1.66935090 | 3.17461596 |

| | | | |
|---|---|---|---|
| 8 | -1.55247899 | 0.16451278 | -1.06720511 |
| 8 | -1.57912250 | 1.69240372 | 0.69610850 |
| 6 | -1.10653594 | 3.03349219 | 0.45314048 |
| 1 | -0.07923316 | 3.13703434 | 0.82273468 |
| 1 | -1.10068987 | 3.25085158 | -0.62348370 |
| 1 | -1.75269189 | 3.75089941 | 0.98071945 |
| 31 | -0.26038595 | 0.06415191 | 0.41220926 |
| 8 | -2.81236378 | 1.96494940 | -1.49318190 |
| 6 | -2.45085070 | 0.99104200 | -0.59717481 |
| 6 | -3.62791821 | 0.34646864 | 0.14382123 |
| 8 | -4.52739796 | 1.33195740 | 0.73569071 |
| 6 | -4.73917136 | 2.50231854 | 0.09149842 |
| 6 | -3.93849362 | 2.83173157 | -1.17030583 |
| 8 | -5.54545787 | 3.30144133 | 0.51999898 |
| 6 | -4.84745893 | 2.91487427 | -2.39529948 |
| 6 | -4.39889811 | -0.62192762 | -0.74543367 |
| 1 | -3.22158915 | -0.19496773 | 1.00825357 |
| 1 | -3.51445120 | 3.82981867 | -0.97721133 |
| 1 | -4.25604241 | 3.22645217 | -3.26501260 |
| 1 | -5.63711615 | 3.65324495 | -2.21228643 |
| 1 | -5.31002939 | 1.94428332 | -2.61500694 |
| 1 | -3.72640445 | -1.42173084 | -1.07746278 |
| 1 | -4.80892046 | -0.12183291 | -1.63239321 |
| 1 | -5.22602138 | -1.06170498 | -0.17431414 |
| 1 | 5.06597706 | 3.58309745 | 1.59019251 |
| 1 | 1.77291253 | -5.12832778 | 3.83689352 |

Electronic energy = -3673.020342 a.u.
DFT-D3(BJ) dispersion correction = -0.119746 a.u.
Thermal free energy = 0.585609 a.u.
Gibbs free energy = -3672.554479 a.u.
Number of imaginary frequencies = 1.

**TS1 ax LA isomer/conformer 4**

| Atomic N. | X | Y | Z |
|---|---|---|---|
| 7 | -0.91150878 | 0.86554684 | 2.11475385 |
| 8 | -0.78394602 | 0.87396517 | -0.81664647 |
| 8 | 1.71926155 | -0.03772426 | 1.09178023 |
| 6 | -2.22999892 | 0.34702355 | 2.55098874 |
| 6 | -1.77196062 | 1.79098915 | -0.71609925 |
| 6 | -2.57222895 | 2.09457721 | -1.85943093 |
| 6 | -3.59878610 | 3.03491636 | -1.69156644 |
| 6 | -3.85182474 | 3.67869990 | -0.47659803 |
| 6 | -3.04074920 | 3.40182620 | 0.61790274 |
| 6 | -1.99635327 | 2.47785235 | 0.50168686 |
| 6 | -1.04215798 | 2.28854739 | 1.64307992 |
| 1 | -4.66688244 | 4.39796607 | -0.39712010 |
| 6 | -2.31791873 | 1.43524321 | -3.22933255 |
| 6 | 2.19678090 | 0.92762095 | 1.91083573 |
| 6 | 3.51339266 | 1.43695375 | 1.70504304 |
| 6 | 3.96834752 | 2.41962281 | 2.59531694 |
| 6 | 3.18948316 | 2.90804458 | 3.64852293 |
| 6 | 1.90886641 | 2.40194328 | 3.83832091 |

| | | | |
|---|---|---|---|
| 6 | 1.41005665 | 1.40860851 | 2.98711667 |
| 6 | 0.05493974 | 0.81462183 | 3.26855128 |
| 1 | 3.58495092 | 3.67877923 | 4.30989756 |
| 6 | 4.40311242 | 0.93285523 | 0.55114538 |
| 6 | -3.26870000 | 1.97598118 | -4.31401179 |
| 6 | -2.54009832 | -0.09148775 | -3.13575485 |
| 6 | -0.87216337 | 1.72726827 | -3.69780521 |
| 6 | 5.78315862 | 1.61723894 | 0.54972746 |
| 6 | 3.73557198 | 1.23924757 | -0.81058779 |
| 6 | 4.64482110 | -0.58945676 | 0.68867798 |
| 1 | -2.62332291 | 0.96156789 | 3.37797779 |
| 1 | -2.93547449 | 0.38742934 | 1.71404173 |
| 1 | -1.35428077 | 2.90709330 | 2.50252024 |
| 1 | -0.02827444 | 2.60879314 | 1.36007744 |
| 1 | -4.23198277 | 3.27920510 | -2.54243767 |
| 1 | -3.19672601 | 3.91491888 | 1.56971116 |
| 1 | 0.14337231 | -0.24409358 | 3.55203958 |
| 1 | -0.40505360 | 1.34613260 | 4.11962420 |
| 1 | 4.96768667 | 2.82988526 | 2.46272098 |
| 1 | 1.28491316 | 2.76663111 | 4.65750982 |
| 1 | -3.03190449 | 1.48681870 | -5.26959824 |
| 1 | -4.32334437 | 1.76164814 | -4.08722165 |
| 1 | -3.15722684 | 3.06011870 | -4.46058138 |
| 1 | -1.85136444 | -0.55679642 | -2.42441058 |
| 1 | -3.57273701 | -0.31254977 | -2.82524154 |
| 1 | -2.37902120 | -0.55109909 | -4.12317267 |
| 1 | -0.70925452 | 2.81027509 | -3.79953129 |
| 1 | -0.13379748 | 1.32558939 | -2.99580155 |
| 1 | -0.70221886 | 1.26620729 | -4.68267941 |
| 1 | 6.37789750 | 1.21828123 | -0.28416206 |
| 1 | 6.34251059 | 1.42462668 | 1.47675271 |
| 1 | 5.70631540 | 2.70504627 | 0.40863373 |
| 1 | 2.77375918 | 0.72671418 | -0.91634634 |
| 1 | 3.57180325 | 2.32023201 | -0.92811425 |
| 1 | 4.39270635 | 0.90663916 | -1.62838960 |
| 1 | 5.14951157 | -0.81779562 | 1.63902313 |
| 1 | 3.70328414 | -1.14767108 | 0.64662125 |
| 1 | 5.29345238 | -0.93694614 | -0.12983638 |
| 1 | -2.12735608 | -0.69084259 | 2.88478345 |
| 8 | -0.67138062 | -2.06254901 | 1.18656656 |
| 6 | -0.02284814 | -2.64177581 | 2.33102554 |
| 1 | -0.52477629 | -2.28727997 | 3.24472226 |
| 8 | 0.43242454 | -1.60059606 | -0.88142498 |
| 1 | 1.04410332 | -2.37077622 | 2.38417041 |
| 1 | -0.12635105 | -3.73499139 | 2.30299796 |
| 31 | -0.04008962 | -0.22580938 | 0.50245462 |
| 8 | -1.46136091 | -2.78825911 | -1.04753084 |
| 6 | -0.21992534 | -2.63560248 | -0.48929476 |
| 6 | 0.56420473 | -3.93233896 | -0.33868311 |
| 8 | -0.22988441 | -5.03909626 | 0.18432047 |
| 6 | -1.58418201 | -5.05623556 | 0.05912018 |
| 6 | -2.30680902 | -3.83358986 | -0.50600724 |
| 8 | -2.20700952 | -6.01740405 | 0.45608282 |

| | | | |
|---|---|---|---|
| 6 | -3.30140814 | -4.22444796 | -1.59218267 |
| 6 | 1.18010406 | -4.33377540 | -1.67704495 |
| 1 | 1.35774835 | -3.77957149 | 0.40409466 |
| 1 | -2.84148375 | -3.39630721 | 0.35474562 |
| 1 | -3.85582553 | -3.33521101 | -1.91601004 |
| 1 | -4.00185213 | -4.96834210 | -1.19692341 |
| 1 | -2.78071365 | -4.65028814 | -2.45977549 |
| 1 | 1.87060548 | -3.55161325 | -2.01445938 |
| 1 | 0.40407533 | -4.47335084 | -2.44108777 |
| 1 | 1.73129593 | -5.27411843 | -1.55278387 |

Electronic energy = -3673.020735 a.u.
DFT-D3(BJ) dispersion correction = -0.118410 a.u.
Thermal free energy = 0.584726 a.u.
Gibbs free energy = -3672.554419 a.u.
Number of imaginary frequencies = 1.

**TS1 ax LA isomer/conformer 5**

| Atomic N. | X | Y | Z |
|---|---|---|---|
| 7 | 0.61339980 | 1.97742641 | 1.22842801 |
| 8 | -1.14530540 | -0.28789073 | 0.58895993 |
| 8 | 0.59868097 | 1.11210692 | -1.64918923 |
| 6 | 1.44066477 | 1.82437313 | 2.44981217 |
| 6 | -1.71290576 | -0.01288993 | 1.78438473 |
| 6 | -2.55934668 | -0.97180152 | 2.41741347 |
| 6 | -3.08072972 | -0.63075304 | 3.67427969 |
| 6 | -2.82602620 | 0.59359503 | 4.30008888 |
| 6 | -2.04893780 | 1.54234147 | 3.64389706 |
| 6 | -1.50842929 | 1.25310016 | 2.38670492 |
| 6 | -0.80422688 | 2.31900573 | 1.60124528 |
| 1 | -3.25182867 | 0.80553538 | 5.28083563 |
| 6 | -2.92155719 | -2.30932736 | 1.74223697 |
| 6 | 0.17322315 | 2.38065648 | -1.85672987 |
| 6 | -0.48518899 | 2.71121025 | -3.07830596 |
| 6 | -0.88758641 | 4.04269361 | -3.25201130 |
| 6 | -0.67451245 | 5.03151560 | -2.28708529 |
| 6 | -0.02949378 | 4.69680977 | -1.10243595 |
| 6 | 0.40736037 | 3.38457775 | -0.88214213 |
| 6 | 1.18564332 | 3.07731867 | 0.37137595 |
| 1 | -1.01362610 | 6.05192709 | -2.46484900 |
| 6 | -0.75108802 | 1.65077150 | -4.16567898 |
| 6 | -3.89916039 | -3.13817334 | 2.59660625 |
| 6 | -1.65663183 | -3.16827989 | 1.51775455 |
| 6 | -3.61250806 | -2.03410172 | 0.38479462 |
| 6 | -1.45450985 | 2.25439286 | -5.39614344 |
| 6 | -1.66961503 | 0.53604394 | -3.61116552 |
| 6 | 0.58401901 | 1.04000628 | -4.65466166 |
| 1 | 1.45794797 | 2.77239537 | 3.01293612 |
| 1 | 1.00860500 | 1.04256141 | 3.08460045 |
| 1 | -0.78910875 | 3.26619840 | 2.16789460 |
| 1 | -1.32901156 | 2.50544119 | 0.65286356 |
| 1 | -3.71597695 | -1.34754670 | 4.19147205 |
| 1 | -1.87367117 | 2.52411127 | 4.09021330 |

| | | | |
|---|---|---|---|
| 1 | 2.21396804 | 2.77042890 | 0.13073096 |
| 1 | 1.25124669 | 3.98855758 | 0.99049360 |
| 1 | -1.39569318 | 4.32454252 | -4.17228460 |
| 1 | 0.15152116 | 5.45528936 | -0.33739138 |
| 1 | -4.13999796 | -4.06716204 | 2.06057262 |
| 1 | -3.46493530 | -3.42111135 | 3.56672764 |
| 1 | -4.84446621 | -2.60689759 | 2.77941489 |
| 1 | -0.94628984 | -2.67072439 | 0.85071677 |
| 1 | -1.15486217 | -3.37765908 | 2.47411275 |
| 1 | -1.93981814 | -4.13204432 | 1.06740236 |
| 1 | -4.53770590 | -1.45731366 | 0.53007162 |
| 1 | -2.95551062 | -1.47822118 | -0.29323560 |
| 1 | -3.88004661 | -2.98772168 | -0.09521644 |
| 1 | -1.61237979 | 1.46137011 | -6.14073324 |
| 1 | -0.85255927 | 3.04123688 | -5.87358952 |
| 1 | -2.44032860 | 2.67257428 | -5.14648056 |
| 1 | -1.20884621 | 0.01013023 | -2.76868057 |
| 1 | -2.62995696 | 0.95449185 | -3.27705073 |
| 1 | -1.87858129 | -0.19949456 | -4.40268469 |
| 1 | 1.23257554 | 1.81834459 | -5.08316469 |
| 1 | 1.11982596 | 0.54608691 | -3.83692384 |
| 1 | 0.38507712 | 0.29583940 | -5.44074440 |
| 1 | 2.45900416 | 1.53794241 | 2.16764641 |
| 8 | 2.60065556 | -0.14642531 | 0.36751178 |
| 6 | 3.52177072 | 0.41162363 | -0.57417208 |
| 1 | 3.72074628 | 1.46279299 | -0.31423834 |
| 8 | 0.99227411 | -1.60403097 | -0.62520528 |
| 1 | 3.14128971 | 0.38018045 | -1.60778666 |
| 1 | 4.48573256 | -0.11936617 | -0.51582563 |
| 31 | 0.55484211 | 0.25002518 | -0.00098232 |
| 8 | 1.77764988 | -2.19916630 | 1.39142334 |
| 6 | 2.02841830 | -1.87744653 | 0.08140824 |
| 6 | 3.21379495 | -2.65406060 | -0.53362358 |
| 8 | 3.38234878 | -3.94723105 | 0.12780640 |
| 6 | 3.43354559 | -3.94926524 | 1.49244046 |
| 6 | 2.93945050 | -2.65206695 | 2.13749719 |
| 8 | 3.81194639 | -4.92655754 | 2.09565353 |
| 6 | 2.53415298 | -2.83537245 | 3.58582711 |
| 6 | 3.02930045 | -2.93555331 | -2.01179314 |
| 1 | 4.14222622 | -2.08847475 | -0.36406296 |
| 1 | 3.73050451 | -1.88370824 | 2.05065536 |
| 1 | 2.20578480 | -1.87769043 | 4.00786686 |
| 1 | 3.38815308 | -3.21137953 | 4.16081190 |
| 1 | 1.71812499 | -3.56366110 | 3.66549563 |
| 1 | 2.94117188 | -1.99466085 | -2.56794763 |
| 1 | 2.11389756 | -3.51487851 | -2.17775750 |
| 1 | 3.89241505 | -3.49746251 | -2.38911176 |

Electronic energy = -3673.018039 a.u.
DFT-D3(BJ) dispersion correction = -0.118629 a.u.
Thermal free energy = 0.584278 a.u.
Gibbs free energy = -3672.552390 a.u.
Number of imaginary frequencies = 1.

**TS1_ax LA isomer/conformer 6**

| Atomic N. | X | Y | Z |
|---|---|---|---|
| 7 | 0.97637862 | 1.78719747 | 1.10506964 |
| 8 | -1.41388309 | 0.07270603 | 0.47468203 |
| 8 | 1.05022073 | 0.39432040 | -1.49075997 |
| 6 | 1.43007588 | 1.74812675 | 2.51447916 |
| 6 | -2.02002549 | 0.87468523 | 1.38007677 |
| 6 | -3.27333927 | 0.48167115 | 1.93875408 |
| 6 | -3.83304191 | 1.32074881 | 2.91302156 |
| 6 | -3.23341298 | 2.51576376 | 3.32103229 |
| 6 | -2.04837990 | 2.92110586 | 2.71660042 |
| 6 | -1.44580603 | 2.12217212 | 1.73799551 |
| 6 | -0.25400305 | 2.64468288 | 0.98245758 |
| 6 | -4.01497936 | -0.78256792 | 1.45707166 |
| 6 | 1.25645125 | 1.62957609 | -1.98859882 |
| 6 | 1.00323856 | 1.89139843 | -3.36862397 |
| 6 | 1.25301087 | 3.19166160 | -3.82880092 |
| 6 | 1.72782564 | 4.21430492 | -3.00075674 |
| 6 | 1.97610188 | 3.94302130 | -1.66011810 |
| 6 | 1.75504887 | 2.65719032 | -1.15337181 |
| 6 | 2.10634017 | 2.34697270 | 0.27226973 |
| 6 | 0.47805756 | 0.79408908 | -4.31506205 |
| 6 | -5.36420727 | -0.96619248 | 2.17714763 |
| 6 | -3.17835212 | -2.05623150 | 1.71689391 |
| 6 | -4.31038523 | -0.65865606 | -0.05741117 |
| 6 | 0.30924246 | 1.31449950 | -5.75499153 |
| 6 | -0.90691519 | 0.29653379 | -3.83739686 |
| 6 | 1.47377670 | -0.38948870 | -4.36186171 |
| 1 | 1.75598180 | 2.75409555 | 2.82763314 |
| 1 | 0.60280502 | 1.43625295 | 3.15926290 |
| 1 | -0.00595172 | 3.66313267 | 1.32773197 |
| 1 | -0.47686580 | 2.70736599 | -0.09275642 |
| 1 | -4.77835894 | 1.03813899 | 3.37255454 |
| 1 | -1.58842504 | 3.87560678 | 2.98284578 |
| 1 | 2.91161799 | 1.59590960 | 0.32722399 |
| 1 | 2.46774006 | 3.26410079 | 0.76759082 |
| 1 | 1.06599605 | 3.42521429 | -4.87537098 |
| 1 | 2.35455227 | 4.72352171 | -0.99596214 |
| 1 | -5.85841703 | -1.86618578 | 1.78453362 |
| 1 | -5.24139643 | -1.10291529 | 3.26161289 |
| 1 | -6.04208374 | -0.11735942 | 2.00824976 |
| 1 | -2.23871708 | -2.03561902 | 1.15404320 |
| 1 | -2.95639944 | -2.16380024 | 2.78955460 |
| 1 | -3.74814463 | -2.94399166 | 1.40230334 |
| 1 | -4.94432545 | 0.21732280 | -0.25695017 |
| 1 | -3.38722539 | -0.56260175 | -0.63985264 |
| 1 | -4.84936142 | -1.55285239 | -0.40589386 |
| 1 | -0.05700862 | 0.49491164 | -6.38955212 |
| 1 | 1.25992823 | 1.66449845 | -6.18283939 |
| 1 | -0.42329291 | 2.13233638 | -5.81753111 |
| 1 | -0.85329670 | -0.15252927 | -2.84009290 |
| 1 | -1.62963259 | 1.12513173 | -3.81101608 |

| | | | |
|---|---|---|---|
| 1 | -1.28874576 | -0.46333224 | -4.53611135 |
| 1 | 2.45290160 | -0.05677742 | -4.73708487 |
| 1 | 1.61001527 | -0.83720732 | -3.37158728 |
| 1 | 1.09558535 | -1.16403222 | -5.04652739 |
| 1 | 2.26454949 | 1.04507613 | 2.61282945 |
| 8 | 0.45639217 | -2.01863694 | -0.33438656 |
| 8 | 1.37706804 | -1.20142078 | 1.69469094 |
| 6 | 0.59984386 | -1.50275874 | 2.87046313 |
| 1 | 0.61048567 | -0.62737101 | 3.53321242 |
| 1 | -0.44540507 | -1.74185681 | 2.62424207 |
| 1 | 1.06376386 | -2.34092045 | 3.40563774 |
| 31 | 0.42585683 | -0.07582574 | 0.20884420 |
| 1 | -3.70525551 | 3.13130045 | 4.08680439 |
| 1 | 1.89962292 | 5.21116209 | -3.40659547 |
| 8 | 2.70113230 | -2.02544403 | -0.12956683 |
| 6 | 1.46543062 | -2.40775704 | 0.36079678 |
| 6 | 1.50863934 | -3.83729768 | 0.91584945 |
| 8 | 2.44748402 | -3.90311599 | 2.02729869 |
| 6 | 3.62671828 | -3.23359513 | 1.86481249 |
| 6 | 3.87301922 | -2.64809966 | 0.46779186 |
| 8 | 4.43171453 | -3.19212842 | 2.76713324 |
| 6 | 5.00542355 | -1.63888514 | 0.45203838 |
| 6 | 0.19060378 | -4.44877005 | 1.34442961 |
| 1 | 1.91906011 | -4.43845940 | 0.08222216 |
| 1 | 4.13483953 | -3.51228547 | -0.17426518 |
| 1 | 5.14699221 | -1.26300550 | -0.56870073 |
| 1 | 5.93246841 | -2.11122417 | 0.79676211 |
| 1 | 4.77576828 | -0.79901165 | 1.11911881 |
| 1 | -0.53046083 | -4.35519838 | 0.52355021 |
| 1 | -0.22290948 | -3.95928907 | 2.23117375 |
| 1 | 0.33863991 | -5.51280880 | 1.56800318 |

Electronic energy = -3673.015381 a.u.
DFT-D3(BJ) dispersion correction = -0.119766 a.u.
Thermal free energy = 0.586111 a.u.
Gibbs free energy = -3672.549036 a.u.
Number of imaginary frequencies = 1.

**TS1 ax LA isomer/conformer 7**

| Atomic N. | X | Y | Z |
|---|---|---|---|
| 7 | -0.85252634 | 2.31749733 | -0.21426028 |
| 8 | 0.78563948 | 0.22752192 | -1.44763722 |
| 8 | 0.26986805 | 0.40670263 | 1.76538599 |
| 6 | -2.06891777 | 2.57607382 | -1.02051005 |
| 6 | 0.87517832 | 1.10532368 | -2.46999130 |
| 6 | 1.27451366 | 0.64905842 | -3.76246888 |
| 6 | 1.33770003 | 1.60371824 | -4.78764825 |
| 6 | 1.04069531 | 2.95549988 | -4.58820141 |
| 6 | 0.68280739 | 3.39134875 | -3.31700259 |
| 6 | 0.61206570 | 2.48010749 | -2.25797498 |
| 6 | 0.34138212 | 2.96362862 | -0.86458643 |
| 1 | 1.10118282 | 3.65744762 | -5.41984597 |
| 6 | 1.64328661 | -0.82448079 | -4.02246490 |

| | | | |
|---|---|---|---|
| 6 | 0.75876905 | 1.52911331 | 2.33874234 |
| 6 | 1.84674396 | 1.43427664 | 3.25666645 |
| 6 | 2.30163622 | 2.62578630 | 3.83841762 |
| 6 | 1.73924330 | 3.87340287 | 3.55158965 |
| 6 | 0.67638586 | 3.95081560 | 2.65899894 |
| 6 | 0.17363157 | 2.78970424 | 2.05969466 |
| 6 | -1.03617553 | 2.88731825 | 1.16826906 |
| 1 | 2.13355955 | 4.77301977 | 4.02393630 |
| 6 | 2.49793391 | 0.07915079 | 3.59752965 |
| 6 | 2.05588617 | -1.06598554 | -5.48640651 |
| 6 | 0.43582008 | -1.74307477 | -3.72235372 |
| 6 | 2.83966083 | -1.23063210 | -3.12890574 |
| 6 | 3.63524780 | 0.23252751 | 4.62507042 |
| 6 | 3.10827108 | -0.55583121 | 2.32542291 |
| 6 | 1.44773049 | -0.87800910 | 4.21087801 |
| 1 | -2.24421376 | 3.66194504 | -1.10352337 |
| 1 | -1.93903301 | 2.16220678 | -2.02622251 |
| 1 | 0.18265427 | 4.05604528 | -0.86530493 |
| 1 | 1.19724106 | 2.75518985 | -0.20620523 |
| 1 | 1.63005169 | 1.28496422 | -5.78652333 |
| 1 | 0.47237458 | 4.44691407 | -3.12966441 |
| 1 | -1.89046374 | 2.35375157 | 1.61038529 |
| 1 | -1.32602037 | 3.94733212 | 1.06310363 |
| 1 | 3.13296815 | 2.58649370 | 4.53987737 |
| 1 | 0.21811961 | 4.91463677 | 2.42537021 |
| 1 | 2.31634647 | -2.12641812 | -5.61271180 |
| 1 | 1.24191451 | -0.83903034 | -6.19050198 |
| 1 | 2.93731957 | -0.47345345 | -5.77092893 |
| 1 | 0.15573218 | -1.68429756 | -2.66485947 |
| 1 | -0.42893803 | -1.46406496 | -4.34348537 |
| 1 | 0.69591809 | -2.78713186 | -3.95400996 |
| 1 | 3.71905135 | -0.61040892 | -3.35567855 |
| 1 | 2.59949044 | -1.12365950 | -2.06545646 |
| 1 | 3.10736140 | -2.28081274 | -3.32070360 |
| 1 | 4.05867481 | -0.75916752 | 4.83827746 |
| 1 | 3.27966478 | 0.65203611 | 5.57748163 |
| 1 | 4.45189301 | 0.86558714 | 4.24869575 |
| 1 | 2.34454589 | -0.76551048 | 1.56939499 |
| 1 | 3.86614931 | 0.10818856 | 1.88483888 |
| 1 | 3.60097999 | -1.50482353 | 2.58571549 |
| 1 | 1.02908332 | -0.45264589 | 5.13505194 |
| 1 | 0.62897995 | -1.07628832 | 3.51087053 |
| 1 | 1.92598755 | -1.83567256 | 4.46693930 |
| 1 | -2.93603396 | 2.10522075 | -0.54625898 |
| 8 | -2.29801786 | -0.37738228 | 0.20134519 |
| 6 | -3.11846324 | -0.03262965 | 1.31864629 |
| 1 | -3.61363247 | 0.93428811 | 1.13386240 |
| 8 | -0.40517991 | -1.75555210 | -0.04662161 |
| 1 | -2.54163990 | 0.02438784 | 2.25438171 |
| 1 | -3.90801101 | -0.78837694 | 1.43582559 |
| 31 | -0.37471601 | 0.24348432 | 0.02485652 |
| 8 | -1.87161487 | -2.56308144 | 1.46338718 |
| 6 | -1.61508090 | -2.11056056 | 0.19762691 |

| | | | |
|---|---|---|---|
| 6 | -2.41169234 | -2.79077089 | -0.93728495 |
| 8 | -2.36122506 | -4.24432655 | -0.74648742 |
| 6 | -2.66144181 | -4.74588835 | 0.47778737 |
| 6 | -2.58386657 | -3.81442437 | 1.68577925 |
| 8 | -2.92702588 | -5.91963571 | 0.61859571 |
| 6 | -3.94127423 | -3.60122324 | 2.35288595 |
| 6 | -3.84902159 | -2.35844396 | -1.19050491 |
| 1 | -1.81804231 | -2.63742250 | -1.84576523 |
| 1 | -1.93829981 | -4.34974157 | 2.39837920 |
| 1 | -3.81196607 | -2.99620596 | 3.25894394 |
| 1 | -4.35183208 | -4.57877485 | 2.63595130 |
| 1 | -4.65976597 | -3.10229586 | 1.69061754 |
| 1 | -3.86859801 | -1.32335324 | -1.54852873 |
| 1 | -4.47828429 | -2.42884920 | -0.29486330 |
| 1 | -4.27632663 | -3.01137745 | -1.96312327 |

Electronic energy = -3673.009046 a.u.
DFT-D3(BJ) dispersion correction = -0.118591 a.u.
Thermal free energy = 0.584156 a.u.
Gibbs free energy = -3672.543481 a.u.
Number of imaginary frequencies = 1.

**TS1 ax LA isomer/conformer 8**

| Atomic N. | X | Y | Z |
|---|---|---|---|
| 8 | 1.13091792 | -3.09346156 | 0.28949530 |
| 6 | -1.01521026 | -4.09822451 | 0.61678151 |
| 8 | -0.69317387 | -5.07230209 | -0.44575799 |
| 6 | 0.51556777 | -5.07359700 | -1.08707745 |
| 6 | 1.46803822 | -3.88598453 | -0.88425063 |
| 8 | 0.77120914 | -5.97131334 | -1.85846949 |
| 6 | 2.91570736 | -4.32437274 | -0.75437149 |
| 6 | -0.71385328 | -4.73910158 | 1.95991103 |
| 1 | -2.08810092 | -3.91026607 | 0.48314570 |
| 1 | 1.34453491 | -3.24923867 | -1.77918270 |
| 1 | 3.56309045 | -3.44014786 | -0.71115327 |
| 1 | 3.18920382 | -4.93227856 | -1.62411643 |
| 1 | 3.05995664 | -4.92114983 | 0.15570206 |
| 1 | -1.14110546 | -4.14950766 | 2.77919493 |
| 1 | 0.36832190 | -4.82703477 | 2.11460140 |
| 1 | -1.16535388 | -5.73932023 | 1.98204344 |
| 7 | 1.38727722 | 1.02118240 | 1.92580542 |
| 8 | -1.16223373 | 0.92783237 | 0.32501938 |
| 8 | 1.52723039 | -0.21846752 | -0.77752639 |
| 6 | 1.37866359 | 0.61430164 | 3.35186569 |
| 6 | -1.59702874 | 1.80593910 | 1.26168065 |
| 6 | -2.99146247 | 2.08039681 | 1.38502790 |
| 6 | -3.38301632 | 2.95249944 | 2.41163114 |
| 6 | -2.47176804 | 3.57051293 | 3.27302393 |
| 6 | -1.10996585 | 3.35389721 | 3.09048904 |
| 6 | -0.66329837 | 2.49374607 | 2.08096534 |
| 6 | 0.80707825 | 2.40060163 | 1.77304903 |
| 6 | -4.02540591 | 1.48889575 | 0.40505278 |
| 6 | 2.34206728 | 0.83549014 | -1.03349966 |

| | | | |
|---|---|---|---|
| 6 | 2.56146615 | 1.26695651 | -2.37553862 |
| 6 | 3.44195224 | 2.34188916 | -2.56574080 |
| 6 | 4.09186585 | 2.99019373 | -1.51145772 |
| 6 | 3.86822113 | 2.55968830 | -0.20879321 |
| 6 | 3.00688781 | 1.48403702 | 0.03484957 |
| 6 | 2.81563152 | 0.98363889 | 1.43758264 |
| 6 | 1.86989511 | 0.58762045 | -3.57370010 |
| 6 | -5.45175706 | 1.98512929 | 0.70868890 |
| 6 | -4.04919608 | -0.05498031 | 0.47933897 |
| 6 | -3.67829219 | 1.93201753 | -1.03706528 |
| 6 | 2.26251652 | 1.23839470 | -4.91339810 |
| 6 | 0.33253632 | 0.69723443 | -3.44187161 |
| 6 | 2.28969111 | -0.89882460 | -3.64282499 |
| 1 | 2.05839521 | 1.26576071 | 3.92626395 |
| 1 | 0.36840344 | 0.71557430 | 3.76061264 |
| 1 | 1.37350850 | 3.09211509 | 2.42055215 |
| 1 | 0.99333952 | 2.69326252 | 0.72967220 |
| 1 | -4.44181953 | 3.16564165 | 2.54654682 |
| 1 | -0.37725740 | 3.87086851 | 3.71452146 |
| 1 | 3.13163774 | -0.06801498 | 1.52839929 |
| 1 | 3.42932886 | 1.58298245 | 2.13141903 |
| 1 | 3.62681335 | 2.69746430 | -3.57763059 |
| 1 | 4.36605661 | 3.04842338 | 0.63172620 |
| 1 | -6.14297937 | 1.55574272 | -0.03024847 |
| 1 | -5.79780646 | 1.67247982 | 1.70488608 |
| 1 | -5.53270252 | 3.07942280 | 0.63981565 |
| 1 | -3.08695515 | -0.48645332 | 0.18368941 |
| 1 | -4.29819718 | -0.39153093 | 1.49708675 |
| 1 | -4.82186437 | -0.44509636 | -0.20047203 |
| 1 | -3.69926117 | 3.02830752 | -1.12072271 |
| 1 | -2.68700956 | 1.57600148 | -1.33899476 |
| 1 | -4.42136170 | 1.52487687 | -1.73936820 |
| 1 | 1.74826412 | 0.71079670 | -5.72914453 |
| 1 | 3.34293779 | 1.17218299 | -5.10700570 |
| 1 | 1.96298135 | 2.29525729 | -4.96349956 |
| 1 | -0.03880641 | 0.18343309 | -2.54962758 |
| 1 | 0.02122409 | 1.75069574 | -3.39268950 |
| 1 | -0.14690623 | 0.24415400 | -4.32273189 |
| 1 | 3.37465310 | -0.99065678 | -3.79778868 |
| 1 | 2.02281055 | -1.42128656 | -2.71788381 |
| 1 | 1.78280029 | -1.39420107 | -4.48459481 |
| 1 | 1.70085615 | -0.42877359 | 3.43775979 |
| 8 | -0.71897584 | -1.92334224 | -0.43719511 |
| 8 | -0.03956286 | -1.63322942 | 1.92417729 |
| 6 | -1.24029904 | -1.47278716 | 2.69100983 |
| 1 | -1.36591821 | -0.43468460 | 3.03568102 |
| 1 | -2.13760411 | -1.72801049 | 2.10253232 |
| 1 | -1.18879287 | -2.12218751 | 3.57790899 |
| 31 | 0.27545562 | -0.22657975 | 0.60612193 |
| 6 | -0.23987380 | -2.83099787 | 0.27301822 |
| 1 | -2.82773970 | 4.23489364 | 4.06032808 |
| 1 | 4.76292102 | 3.82497297 | -1.71328099 |

Electronic energy = -3673.003547 a.u.

DFT-D3(BJ) dispersion correction = -0.120390 a.u.
Thermal free energy = 0.584673 a.u.
Gibbs free energy = -3672.539264 a.u.
Number of imaginary frequencies = 1.

**Int1 LA isomer/conformer 1**

| Atomic N. | X | Y | Z |
|---|---|---|---|
| 7 | 1.68970429 | 2.00030114 | -0.79329598 |
| 8 | 0.27044187 | -0.60971705 | -1.14956486 |
| 8 | 0.62943554 | 0.67684454 | 1.67877856 |
| 6 | 1.30576498 | 3.06690816 | -1.75149896 |
| 6 | 0.89911757 | -0.60055677 | -2.35467978 |
| 6 | 0.45766106 | -1.48076764 | -3.38694521 |
| 6 | 1.12112647 | -1.40956316 | -4.62048977 |
| 6 | 2.18702809 | -0.53862016 | -4.86022844 |
| 6 | 2.64051019 | 0.27680790 | -3.82962989 |
| 6 | 2.02004074 | 0.24153449 | -2.57562264 |
| 6 | 2.62589157 | 1.01352141 | -1.43438436 |
| 1 | 2.66538478 | -0.51465823 | -5.83920517 |
| 6 | -0.68154402 | -2.49458495 | -3.15886521 |
| 6 | 1.93263047 | 0.71645999 | 2.05342895 |
| 6 | 2.40314855 | -0.15381192 | 3.08117544 |
| 6 | 3.75406826 | -0.05152762 | 3.44178903 |
| 6 | 4.63472912 | 0.85131746 | 2.83925609 |
| 6 | 4.16318473 | 1.69665550 | 1.84201266 |
| 6 | 2.81952225 | 1.64572651 | 1.45176868 |
| 6 | 2.31620219 | 2.62855984 | 0.42838128 |
| 1 | 5.67849804 | 0.88838695 | 3.15057084 |
| 6 | 1.47091711 | -1.16705216 | 3.77423157 |
| 6 | -0.94411228 | -3.36132948 | -4.40462093 |
| 6 | -1.99857851 | -1.75918635 | -2.82373544 |
| 6 | -0.30554426 | -3.45166081 | -2.00211639 |
| 6 | 2.20667911 | -1.98544888 | 4.85209009 |
| 6 | 0.91049362 | -2.17169940 | 2.73970534 |
| 6 | 0.31143237 | -0.42144459 | 4.47628049 |
| 1 | 2.20082244 | 3.63350170 | -2.05640331 |
| 1 | 0.84696054 | 2.61559181 | -2.63759916 |
| 1 | 3.52220429 | 1.55616539 | -1.77979016 |
| 1 | 2.94026838 | 0.33006127 | -0.63163083 |
| 1 | 0.79600598 | -2.05881478 | -5.43117752 |
| 1 | 3.49780036 | 0.93658766 | -3.98127839 |
| 1 | 1.53699077 | 3.28023468 | 0.85577923 |
| 1 | 3.14763409 | 3.27180930 | 0.09469968 |
| 1 | 4.14252727 | -0.70541255 | 4.22026173 |
| 1 | 4.83184620 | 2.41483562 | 1.36223882 |
| 1 | -1.75321640 | -4.07088007 | -4.18067836 |
| 1 | -1.26221382 | -2.76090935 | -5.26920674 |
| 1 | -0.06043574 | -3.94804677 | -4.69369529 |
| 1 | -1.88585855 | -1.15914299 | -1.91455667 |
| 1 | -2.29695644 | -1.09749272 | -3.65030882 |
| 1 | -2.80794338 | -2.48793662 | -2.66647108 |
| 1 | 0.60589261 | -4.01527172 | -2.24846269 |

| | | | |
|---|---|---|---|
| 1 | -0.13397017 | -2.90668676 | -1.06711610 |
| 1 | -1.11741070 | -4.17708303 | -1.84088486 |
| 1 | 1.49630790 | -2.68494191 | 5.31514400 |
| 1 | 2.61121257 | -1.34844067 | 5.65181469 |
| 1 | 3.02910673 | -2.58144518 | 4.43090967 |
| 1 | 0.33190788 | -1.66902150 | 1.95754831 |
| 1 | 1.72709679 | -2.73014121 | 2.25981956 |
| 1 | 0.25604225 | -2.89873407 | 3.24429522 |
| 1 | 0.69981549 | 0.25761167 | 5.24926944 |
| 1 | -0.27685982 | 0.16488522 | 3.76223172 |
| 1 | -0.35476243 | -1.14615929 | 4.96881599 |
| 1 | 0.58161108 | 3.73687316 | -1.27644354 |
| 8 | -3.11538030 | 2.44306086 | 1.01044296 |
| 6 | -2.28558314 | 2.87292111 | 2.10574774 |
| 1 | -2.97171591 | 3.31941203 | 2.83361830 |
| 8 | -1.24657923 | 2.20568814 | -0.37079124 |
| 1 | -1.55752151 | 3.62356242 | 1.76939115 |
| 1 | -1.75340601 | 2.02795808 | 2.56624029 |
| 31 | 0.08208601 | 0.90647206 | -0.08594555 |
| 8 | -2.10328200 | 0.35691026 | 0.50570419 |
| 6 | -2.45810206 | 1.69803560 | 0.02445253 |
| 6 | -3.42286073 | 1.53006118 | -1.15813968 |
| 8 | -4.60514723 | 0.75992428 | -0.77469167 |
| 6 | -4.51324752 | -0.26165501 | 0.11137985 |
| 6 | -3.17861466 | -0.56897140 | 0.80765180 |
| 8 | -5.49026551 | -0.94070952 | 0.34853583 |
| 6 | -3.37524190 | -0.71754717 | 2.31564282 |
| 6 | -3.92054056 | 2.83895474 | -1.73922977 |
| 1 | -2.87866454 | 0.94887504 | -1.91758371 |
| 1 | -2.85103441 | -1.53314349 | 0.38672856 |
| 1 | -2.42651136 | -1.01265170 | 2.78035720 |
| 1 | -4.12919808 | -1.48935054 | 2.50947330 |
| 1 | -3.71644161 | 0.22618271 | 2.75867314 |
| 1 | -3.06552285 | 3.44064440 | -2.07317758 |
| 1 | -4.48281253 | 3.40230581 | -0.98564768 |
| 1 | -4.57067247 | 2.64006908 | -2.60054333 |

Electronic energy = -3673.042728 a.u.
DFT-D3(BJ) dispersion correction = -0.120009 a.u.
Thermal free energy = 0.586323 a.u.
Gibbs free energy = -3672.576414 a.u.
Number of imaginary frequencies = 0.

**Int1 LA isomer/conformer 2**

| Atomic N. | X | Y | Z |
|---|---|---|---|
| 7 | 0.18742354 | 2.62797637 | 0.70474594 |
| 8 | -1.32076600 | 0.12225586 | 0.03265767 |
| 8 | 1.69602037 | 0.39739440 | -0.64762785 |
| 6 | 0.02622995 | 3.17359944 | 2.07557888 |
| 6 | -2.41063366 | 0.90632070 | 0.24533641 |
| 6 | -3.69682446 | 0.30508318 | 0.38305837 |
| 6 | -4.77672707 | 1.15618687 | 0.66058674 |
| 6 | -4.64296348 | 2.54257243 | 0.77186596 |

| | | | |
|---|---|---|---|
| 6 | -3.39802707 | 3.12211327 | 0.55447171 |
| 6 | -2.28640932 | 2.32065530 | 0.27014805 |
| 6 | -0.99800697 | 2.97469148 | -0.15259970 |
| 1 | -5.51176450 | 3.16037021 | 0.99846269 |
| 6 | -3.91280543 | -1.20949428 | 0.18878464 |
| 6 | 1.85200327 | 1.33030612 | -1.62457622 |
| 6 | 2.19199614 | 0.92838085 | -2.95056046 |
| 6 | 2.34838336 | 1.93891429 | -3.90949650 |
| 6 | 2.19154613 | 3.29622227 | -3.61533735 |
| 6 | 1.87835346 | 3.67728877 | -2.31627492 |
| 6 | 1.71947821 | 2.70915718 | -1.31676975 |
| 6 | 1.46801431 | 3.15521021 | 0.09894539 |
| 1 | 2.31815193 | 4.04456413 | -4.39754591 |
| 6 | 2.40446041 | -0.55236688 | -3.31977765 |
| 6 | -5.39881582 | -1.59904856 | 0.30269069 |
| 6 | -3.14162646 | -2.01372312 | 1.25836532 |
| 6 | -3.43598209 | -1.62404928 | -1.22436335 |
| 6 | 2.76009458 | -0.73338448 | -4.80777422 |
| 6 | 1.11279753 | -1.36254219 | -3.06025600 |
| 6 | 3.57688770 | -1.12731563 | -2.49118143 |
| 1 | -0.00821569 | 4.27457279 | 2.03557122 |
| 1 | -0.90793292 | 2.80021445 | 2.50907848 |
| 1 | -1.11499364 | 4.07160142 | -0.15811397 |
| 1 | -0.73099717 | 2.66294069 | -1.17333205 |
| 1 | -5.76673634 | 0.72386485 | 0.79263442 |
| 1 | -3.28004178 | 4.20787906 | 0.57747083 |
| 1 | 2.27255890 | 2.81234393 | 0.76928302 |
| 1 | 1.43813051 | 4.25678597 | 0.14106156 |
| 1 | 2.60003281 | 1.66004039 | -4.93115315 |
| 1 | 1.76537403 | 4.73310449 | -2.06009958 |
| 1 | -5.49713927 | -2.68054956 | 0.13203115 |
| 1 | -5.81024914 | -1.38416155 | 1.29969215 |
| 1 | -6.01899663 | -1.08837252 | -0.44784701 |
| 1 | -2.06476537 | -1.82210477 | 1.20216850 |
| 1 | -3.49825652 | -1.75681788 | 2.26699238 |
| 1 | -3.31143890 | -3.09113962 | 1.10817752 |
| 1 | -4.00605090 | -1.08783703 | -1.99683430 |
| 1 | -2.37056312 | -1.41403527 | -1.36919526 |
| 1 | -3.60170609 | -2.70222934 | -1.37066611 |
| 1 | 2.90619706 | -1.80419348 | -5.00816914 |
| 1 | 3.69394145 | -0.21895849 | -5.07660025 |
| 1 | 1.96040077 | -0.37779399 | -5.47406110 |
| 1 | 0.80707713 | -1.31065110 | -2.01051575 |
| 1 | 0.28624573 | -0.98534140 | -3.68006749 |
| 1 | 1.28025564 | -2.41809561 | -3.32176956 |
| 1 | 4.51194912 | -0.60355452 | -2.73790574 |
| 1 | 3.40215173 | -1.03442179 | -1.41508934 |
| 1 | 3.71392484 | -2.19398416 | -2.72038980 |
| 1 | 0.86571344 | 2.84649440 | 2.69784883 |
| 8 | 0.65036722 | -1.34755999 | 3.95964087 |
| 6 | -0.69363687 | -0.88368785 | 4.17439111 |
| 1 | -1.36371435 | -1.20808886 | 3.36617921 |
| 8 | 0.97698932 | 0.32190761 | 2.38475908 |

```
   8     0.65929369  -1.76038191   1.62738692
   1    -1.01177181  -1.34064640   5.11810221
   1    -0.72353764   0.21085409   4.26294539
  31     0.37643496   0.57880240   0.64173315
   8     3.34503779  -1.80978717   1.67391173
   6     2.72522128  -2.84314811   1.02988512
   6     1.27053415  -3.06272103   1.45835707
   6     1.22479093  -0.98232025   2.72153164
   6     2.72824058  -1.35590605   2.91424588
   8     3.30291332  -3.47253314   0.17243978
   6     3.56479555  -0.20440874   3.43269085
   6     0.47558284  -3.86020769   0.44305534
   1     1.26032257  -3.58222075   2.43520336
   1     2.75457980  -2.19343122   3.62803821
   1     4.59745488  -0.53784772   3.59414928
   1     3.15353034   0.15256849   4.38545024
   1     3.55974896   0.62233863   2.71272757
   1     0.96035658  -4.82978015   0.27938290
   1     0.43531076  -3.33219338  -0.51751317
   1    -0.54520306  -4.02254008   0.80954867
Electronic energy = -3673.041321 a.u.
DFT-D3(BJ) dispersion correction = -0.119947 a.u.
Thermal free energy = 0.586906 a.u.
Gibbs free energy = -3672.574361 a.u.
Number of imaginary frequencies = 0.
```

**Int1 LA isomer/conformer 3**

```
Atomic N.      X            Y            Z
   7    -1.69606077  -0.12938011   1.35308099
   8    -0.67324744   0.81256164  -1.29529267
   8     1.23598126   0.55705103   1.10695027
   6    -2.52521635  -1.36087196   1.41820267
   6    -1.99452001   0.82971498  -1.61568059
   6    -2.40389063   0.83007096  -2.97973378
   6    -3.78363274   0.85252958  -3.23239867
   6    -4.74267066   0.87818888  -2.21570767
   6    -4.32815580   0.90066671  -0.88827265
   6    -2.96340856   0.89028090  -0.58050757
   6    -2.51209062   1.03816667   0.84711058
   1    -5.80352417   0.88854036  -2.46542103
   6    -1.38068556   0.80917062  -4.13218292
   6     0.90672672   1.53455648   1.99402886
   6     1.75675697   2.66803715   2.14792739
   6     1.37313433   3.63397301   3.08930681
   6     0.21202880   3.52487593   3.85928639
   6    -0.60391540   2.41051548   3.70425755
   6    -0.26249838   1.40772267   2.78857244
   6    -1.11147925   0.16436152   2.71771194
   1    -0.04678597   4.30676148   4.57298173
   6     3.04572043   2.83464883   1.31859596
   6    -2.06637268   0.86482625  -5.51049673
   6    -0.54969731  -0.49469405  -4.08363657
```

| | | | |
|---|---|---|---|
| 6 | -0.44562407 | 2.03826143 | -4.03429646 |
| 6 | 3.79941548 | 4.12880338 | 1.67981566 |
| 6 | 2.70647510 | 2.90906651 | -0.18890576 |
| 6 | 4.00699770 | 1.65369647 | 1.59143927 |
| 1 | -3.36302741 | -1.20677067 | 2.11630368 |
| 1 | -2.92156530 | -1.58929236 | 0.42337205 |
| 1 | -3.38133263 | 1.15851418 | 1.51437424 |
| 1 | -1.87090042 | 1.92426376 | 0.96134259 |
| 1 | -4.13004804 | 0.84483460 | -4.26423759 |
| 1 | -5.06001732 | 0.94550531 | -0.07858792 |
| 1 | -0.52119437 | -0.72803096 | 2.97666829 |
| 1 | -1.94527510 | 0.24230414 | 3.43412384 |
| 1 | 1.99905287 | 4.51366436 | 3.22628630 |
| 1 | -1.51015440 | 2.29982822 | 4.30377166 |
| 1 | -1.29400808 | 0.86444537 | -6.29267839 |
| 1 | -2.71468180 | -0.00557526 | -5.68789557 |
| 1 | -2.66564969 | 1.77795744 | -5.63797993 |
| 1 | 0.00691088 | -0.59109295 | -3.14549390 |
| 1 | -1.20221699 | -1.37380194 | -4.18918035 |
| 1 | 0.16977486 | -0.50664084 | -4.91659815 |
| 1 | -1.02368952 | 2.97081852 | -4.11035691 |
| 1 | 0.11000951 | 2.04961479 | -3.09047715 |
| 1 | 0.27663998 | 2.02083238 | -4.86461771 |
| 1 | 4.71087080 | 4.19200145 | 1.06864406 |
| 1 | 4.10757507 | 4.14833587 | 2.73514925 |
| 1 | 3.20175420 | 5.02841180 | 1.47372689 |
| 1 | 2.20794973 | 2.00018107 | -0.54137757 |
| 1 | 2.04910397 | 3.76552677 | -0.39638590 |
| 1 | 3.63103066 | 3.04399003 | -0.77050185 |
| 1 | 4.29539683 | 1.62629824 | 2.65237816 |
| 1 | 3.54720501 | 0.69398194 | 1.33245971 |
| 1 | 4.92408130 | 1.77504853 | 0.99521040 |
| 1 | -1.91316005 | -2.19862102 | 1.76570531 |
| 8 | 2.18723035 | -3.19944034 | -0.48154558 |
| 6 | 3.03039553 | -2.03956903 | -0.58087234 |
| 1 | 3.08084160 | -1.49852387 | 0.37558239 |
| 8 | 0.26019436 | -1.90274852 | -0.63860105 |
| 8 | 0.90404401 | -2.64002188 | 1.41080867 |
| 1 | 4.02448825 | -2.42021887 | -0.83992564 |
| 1 | 2.67974889 | -1.36306149 | -1.37342357 |
| 31 | -0.08908201 | -0.25808537 | 0.10464934 |
| 8 | -0.83210674 | -4.58180882 | 0.80703201 |
| 6 | -0.38721522 | -4.50875482 | 2.09652808 |
| 6 | 0.97612215 | -3.82467121 | 2.23800470 |
| 6 | 0.87562156 | -2.94769973 | -0.00289484 |
| 6 | 0.15751275 | -4.31659006 | -0.23622882 |
| 8 | -1.09093518 | -4.88131866 | 3.01103189 |
| 6 | -0.55653784 | -4.40636059 | -1.56812585 |
| 6 | 1.28890622 | -3.42275537 | 3.66533454 |
| 1 | 1.77190356 | -4.48969934 | 1.85434233 |
| 1 | 0.93125477 | -5.09557439 | -0.16804385 |
| 1 | -1.00938838 | -5.39945388 | -1.68138435 |
| 1 | 0.16287738 | -4.24913393 | -2.38149219 |

| | | | |
|---|---|---|---|
| 1 | -1.33389645 | -3.63722524 | -1.64138713 |
| 1 | 1.34493109 | -4.31659720 | 4.29829337 |
| 1 | 0.50291956 | -2.77411309 | 4.07173413 |
| 1 | 2.24997479 | -2.89520985 | 3.70070077 |

Electronic energy = -3673.042082 a.u.
DFT-D3(BJ) dispersion correction = -0.118239 a.u.
Thermal free energy = 0.586376 a.u.
Gibbs free energy = -3672.573944 a.u.
Number of imaginary frequencies = 0.

**Int1 LA isomer/conformer 4**

| Atomic N. | X | Y | Z |
|---|---|---|---|
| 7 | 0.40589841 | -0.10377474 | 2.26441693 |
| 8 | 1.08226233 | 1.36496264 | -0.24459201 |
| 8 | 0.03115589 | -1.55340583 | -0.35962792 |
| 6 | -0.50876721 | 0.50467914 | 3.26457895 |
| 6 | 1.52336430 | 2.31781283 | 0.62057542 |
| 6 | 1.70676073 | 3.66037136 | 0.18291090 |
| 6 | 2.16525453 | 4.58404593 | 1.13421091 |
| 6 | 2.44464560 | 4.23700826 | 2.45926719 |
| 6 | 2.28502856 | 2.91666817 | 2.86618600 |
| 6 | 1.84048103 | 1.95284045 | 1.95460679 |
| 6 | 1.78663307 | 0.50380251 | 2.35400243 |
| 1 | 2.79364855 | 4.99496171 | 3.16040294 |
| 6 | 1.41884328 | 4.08595153 | -1.27013993 |
| 6 | 1.05417807 | -2.32291861 | 0.10832761 |
| 6 | 1.81344298 | -3.12414482 | -0.79144762 |
| 6 | 2.84416401 | -3.90072833 | -0.24186942 |
| 6 | 3.14228818 | -3.91091385 | 1.12327389 |
| 6 | 2.38523455 | -3.13250764 | 1.99093253 |
| 6 | 1.33516776 | -2.34778785 | 1.49899219 |
| 6 | 0.45981046 | -1.60082294 | 2.47272786 |
| 1 | 3.95912762 | -4.52616658 | 1.49995427 |
| 6 | 1.52245743 | -3.14642707 | -2.30540388 |
| 6 | 1.74579259 | 5.57231658 | -1.50840103 |
| 6 | -0.07970658 | 3.88544399 | -1.59695148 |
| 6 | 2.29064196 | 3.26139195 | -2.24715564 |
| 6 | 2.45932612 | -4.11154978 | -3.05668994 |
| 6 | 1.73102062 | -1.73902694 | -2.91222850 |
| 6 | 0.07400907 | -3.62715031 | -2.55995575 |
| 1 | -0.13945883 | 0.29490686 | 4.28117434 |
| 1 | -0.54596365 | 1.58886129 | 3.11173780 |
| 1 | 2.14767546 | 0.37556620 | 3.38782822 |
| 1 | 2.42258902 | -0.10311098 | 1.69324163 |
| 1 | 2.30765615 | 5.62074573 | 0.83476607 |
| 1 | 2.52383689 | 2.61770593 | 3.88938724 |
| 1 | -0.58498266 | -1.93764317 | 2.39847482 |
| 1 | 0.80661726 | -1.78719771 | 3.50233941 |
| 1 | 3.44680439 | -4.52000617 | -0.90335866 |
| 1 | 2.59253875 | -3.13677318 | 3.06327441 |
| 1 | 1.53891129 | 5.81831398 | -2.55954156 |
| 1 | 1.12944828 | 6.23848656 | -0.88742322 |

| | | | |
|---|---|---|---|
| 1 | 2.80490476 | 5.79872565 | -1.31800833 |
| 1 | -0.38554799 | 2.83914974 | -1.49259254 |
| 1 | -0.70713030 | 4.49519232 | -0.93073567 |
| 1 | -0.27862271 | 4.20276015 | -2.63193939 |
| 1 | 3.35919902 | 3.42992330 | -2.04866115 |
| 1 | 2.08665617 | 2.18842936 | -2.16468526 |
| 1 | 2.08625429 | 3.57689196 | -3.28150831 |
| 1 | 2.20174891 | -4.09769861 | -4.12508932 |
| 1 | 2.35480719 | -5.14818699 | -2.70538190 |
| 1 | 3.51513861 | -3.81749201 | -2.96893181 |
| 1 | 1.05572271 | -0.99902186 | -2.47006352 |
| 1 | 2.76467466 | -1.39820183 | -2.75717391 |
| 1 | 1.54412596 | -1.77136514 | -3.99615761 |
| 1 | -0.07186208 | -4.64435119 | -2.16841163 |
| 1 | -0.65824986 | -2.96412926 | -2.08651108 |
| 1 | -0.12599095 | -3.64994925 | -3.64168226 |
| 1 | -1.51125689 | 0.08123657 | 3.13749238 |
| 8 | -4.11303365 | 0.92276766 | 0.41896586 |
| 6 | -4.00297257 | 1.68740981 | 1.62759735 |
| 1 | -4.94357011 | 2.24263871 | 1.70890649 |
| 8 | -1.84695263 | 0.91334704 | 0.03453327 |
| 1 | -3.16020461 | 2.39080144 | 1.57429653 |
| 1 | -3.89295937 | 1.03178868 | 2.50542011 |
| 31 | -0.20127249 | 0.15286980 | 0.33009299 |
| 8 | -2.77103468 | -0.88213480 | 1.10255077 |
| 6 | -2.99287763 | 0.14334542 | 0.09354072 |
| 6 | -3.30834593 | -0.52842808 | -1.26056328 |
| 8 | -4.51163239 | -1.34368319 | -1.13671613 |
| 6 | -4.58503408 | -2.16323342 | -0.05709132 |
| 6 | -3.34110091 | -2.19063012 | 0.84306869 |
| 8 | -5.55714403 | -2.86561846 | 0.11809994 |
| 6 | -3.63030891 | -2.86184130 | 2.17451891 |
| 6 | -3.55139525 | 0.45239837 | -2.39047326 |
| 1 | -2.45802947 | -1.19067619 | -1.49623303 |
| 1 | -2.58494773 | -2.77883449 | 0.28458544 |
| 1 | -2.71378322 | -2.92241877 | 2.77651019 |
| 1 | -4.00969483 | -3.87593950 | 2.00481548 |
| 1 | -4.39022453 | -2.29902632 | 2.73077276 |
| 1 | -2.66895332 | 1.08977430 | -2.52448699 |
| 1 | -4.41758119 | 1.08423297 | -2.16309061 |
| 1 | -3.73935420 | -0.09560958 | -3.32238379 |

Electronic energy = -3673.040980 a.u.
DFT-D3(BJ) dispersion correction = -0.118294 a.u.
Thermal free energy = 0.585616 a.u.
Gibbs free energy = -3672.573658 a.u.
Number of imaginary frequencies = 0.

**Int1_LA isomer/conformer 5**

| Atomic N. | X | Y | Z |
|---|---|---|---|
| 7 | -0.86676347 | 2.46406957 | -0.04648263 |
| 8 | 0.37415074 | 0.22803616 | 1.49944525 |
| 8 | -1.35493633 | -0.34112013 | -1.01285275 |

| | | | |
|---|---|---|---|
| 6 | 0.01959709 | 3.61830766 | -0.34044228 |
| 6 | 0.49612611 | 1.17633488 | 2.46331697 |
| 6 | 1.37266585 | 0.95150625 | 3.56570000 |
| 6 | 1.48763787 | 1.97656979 | 4.51586327 |
| 6 | 0.77235683 | 3.17393994 | 4.43036020 |
| 6 | -0.12021586 | 3.35705016 | 3.38000384 |
| 6 | -0.27938172 | 2.36397672 | 2.40699486 |
| 6 | -1.36213588 | 2.51257984 | 1.37141595 |
| 1 | 0.90229557 | 3.94492336 | 5.18968382 |
| 6 | 2.14021583 | -0.37543991 | 3.73261392 |
| 6 | -2.65254433 | 0.00370478 | -0.83366902 |
| 6 | -3.64523009 | -1.01424577 | -0.71322253 |
| 6 | -4.97402327 | -0.60228656 | -0.54256134 |
| 6 | -5.34971760 | 0.74293967 | -0.48625784 |
| 6 | -4.37489942 | 1.72544127 | -0.61388424 |
| 6 | -3.03327441 | 1.37022134 | -0.79891486 |
| 6 | -2.01411964 | 2.45511173 | -1.02821733 |
| 1 | -6.39589220 | 1.01398526 | -0.34547965 |
| 6 | -3.27703068 | -2.50972756 | -0.77209654 |
| 6 | 2.98823338 | -0.39338624 | 5.01825300 |
| 6 | 3.10087716 | -0.60262139 | 2.54329668 |
| 6 | 1.13560235 | -1.54921831 | 3.82605085 |
| 6 | -4.51485609 | -3.41654669 | -0.63646662 |
| 6 | -2.31864198 | -2.86850050 | 0.38811330 |
| 6 | -2.61740643 | -2.83506836 | -2.13340934 |
| 1 | -0.54787305 | 4.55796509 | -0.24033499 |
| 1 | 0.85656964 | 3.62677050 | 0.36601801 |
| 1 | -1.90072338 | 3.46362425 | 1.52190923 |
| 1 | -2.09501778 | 1.69715344 | 1.46367951 |
| 1 | 2.16186187 | 1.84036362 | 5.35935173 |
| 1 | -0.72075396 | 4.26728518 | 3.31542202 |
| 1 | -1.54777715 | 2.35417355 | -2.02154546 |
| 1 | -2.50879214 | 3.43990744 | -0.98566222 |
| 1 | -5.75164752 | -1.35737300 | -0.44300141 |
| 1 | -4.64725248 | 2.78283069 | -0.58369502 |
| 1 | 3.49906747 | -1.36381674 | 5.09486559 |
| 1 | 3.76200486 | 0.38805248 | 5.01618241 |
| 1 | 2.37309802 | -0.27195643 | 5.92135654 |
| 1 | 2.55214933 | -0.67056117 | 1.59774047 |
| 1 | 3.83001314 | 0.21813325 | 2.47221220 |
| 1 | 3.66044897 | -1.53924889 | 2.69073904 |
| 1 | 0.47533189 | -1.42393648 | 4.69641035 |
| 1 | 0.51484696 | -1.61948930 | 2.92583193 |
| 1 | 1.68124384 | -2.49722588 | 3.95000781 |
| 1 | -4.19573690 | -4.46704044 | -0.69406867 |
| 1 | -5.24200651 | -3.24758628 | -1.44370721 |
| 1 | -5.02578268 | -3.27950904 | 0.32746964 |
| 1 | -1.39433808 | -2.28280350 | 0.34670096 |
| 1 | -2.79974642 | -2.68171581 | 1.35902610 |
| 1 | -2.05973334 | -3.93743506 | 0.34040976 |
| 1 | -3.32314556 | -2.65167810 | -2.95667955 |
| 1 | -1.72450907 | -2.22361031 | -2.30251687 |
| 1 | -2.33170501 | -3.89786139 | -2.16532859 |

| | | | |
|---|---|---|---|
| 1 | 0.41161069 | 3.52303425 | -1.35829269 |
| 8 | 1.58557124 | -0.96277615 | -1.03087835 |
| 6 | 1.49623635 | -2.33377529 | -1.45843987 |
| 1 | 2.48788290 | -2.71910704 | -1.74260239 |
| 8 | 1.39848228 | 1.19772023 | -1.48933282 |
| 8 | 1.28814185 | -0.23921269 | -3.26334329 |
| 1 | 1.13292065 | -2.89420776 | -0.59021297 |
| 1 | 0.79002850 | -2.44032241 | -2.29105837 |
| 31 | 0.05842581 | 0.63267350 | -0.29013811 |
| 8 | 3.79448986 | 0.92065531 | -3.38482048 |
| 6 | 3.19317358 | 0.55753656 | -4.55463332 |
| 6 | 2.13589235 | -0.53889605 | -4.39655800 |
| 6 | 1.92716366 | 0.07296307 | -2.03398592 |
| 6 | 3.46298272 | 0.12064892 | -2.21011823 |
| 8 | 3.49954610 | 1.09071097 | -5.59725831 |
| 6 | 4.19716592 | 0.73191230 | -1.03579918 |
| 6 | 1.24350457 | -0.66539169 | -5.61619005 |
| 1 | 2.67013982 | -1.49596724 | -4.22887194 |
| 1 | 3.82369211 | -0.90703987 | -2.38863455 |
| 1 | 5.27502160 | 0.74166596 | -1.24069564 |
| 1 | 4.01450103 | 0.14255771 | -0.12921977 |
| 1 | 3.84897446 | 1.75698283 | -0.86668555 |
| 1 | 1.85077124 | -0.90295755 | -6.49747088 |
| 1 | 0.71867298 | 0.27904004 | -5.80289417 |
| 1 | 0.50622340 | -1.46239654 | -5.45781339 |

Electronic energy = -3673.041060 a.u.
DFT-D3(BJ) dispersion correction = -0.117761 a.u.
Thermal free energy = 0.586080 a.u.
Gibbs free energy = -3672.572740 a.u.
Number of imaginary frequencies = 0.

**Int1_LA isomer/conformer 6**

| Atomic N. | X | Y | Z |
|---|---|---|---|
| 7 | 0.16381937 | 1.90652808 | 1.59927207 |
| 8 | -1.61595736 | -0.25292718 | 0.55029485 |
| 8 | 0.58408195 | 0.97044786 | -1.24013247 |
| 6 | 0.83679852 | 1.70522131 | 2.90792775 |
| 6 | -2.28176570 | -0.04308751 | 1.71610291 |
| 6 | -3.17359443 | -1.03247448 | 2.22121452 |
| 6 | -3.82979452 | -0.74599545 | 3.42745110 |
| 6 | -3.64501359 | 0.44946517 | 4.12812041 |
| 6 | -2.78936104 | 1.41656161 | 3.61142355 |
| 6 | -2.11737044 | 1.18658583 | 2.40589410 |
| 6 | -1.28722959 | 2.27881427 | 1.78736803 |
| 1 | -4.17517709 | 0.62144717 | 5.06476481 |
| 6 | -3.41502240 | -2.35992847 | 1.47598697 |
| 6 | 0.22551405 | 2.24641294 | -1.53753410 |
| 6 | -0.20918271 | 2.56675528 | -2.85619806 |
| 6 | -0.55405900 | 3.90173824 | -3.11061308 |
| 6 | -0.48821117 | 4.90159543 | -2.13591488 |
| 6 | -0.05017737 | 4.57722360 | -0.85724285 |
| 6 | 0.32067763 | 3.26210699 | -0.55093265 |

| | | | |
|---|---|---|---|
| 6 | 0.90221905 | 2.96214619 | 0.80691370 |
| 1 | -0.77497381 | 5.92410150 | -2.38091606 |
| 6 | -0.29878192 | 1.49430773 | -3.96002898 |
| 6 | -4.45322697 | -3.24182615 | 2.19516350 |
| 6 | -2.10079946 | -3.17178083 | 1.39172999 |
| 6 | -3.95659568 | -2.07892927 | 0.05390137 |
| 6 | -0.75480699 | 2.09195204 | -5.30459571 |
| 6 | -1.33022029 | 0.41040072 | -3.56633749 |
| 6 | 1.08856008 | 0.84810442 | -4.19042928 |
| 1 | 0.83450263 | 2.64967135 | 3.47566653 |
| 1 | 0.29943829 | 0.94050641 | 3.47930719 |
| 1 | -1.32968245 | 3.19059040 | 2.40595729 |
| 1 | -1.66341246 | 2.53170690 | 0.78528151 |
| 1 | -4.51178331 | -1.48480138 | 3.84438518 |
| 1 | -2.64994836 | 2.36827875 | 4.12929820 |
| 1 | 1.93190048 | 2.58313273 | 0.71559152 |
| 1 | 0.92759708 | 3.88395161 | 1.41115981 |
| 1 | -0.89485662 | 4.17735433 | -4.10683712 |
| 1 | 0.02301121 | 5.34644922 | -0.08515151 |
| 1 | -4.59816208 | -4.16529176 | 1.61677928 |
| 1 | -4.12460996 | -3.53275270 | 3.20350637 |
| 1 | -5.43131089 | -2.74602431 | 2.27683884 |
| 1 | -1.31983113 | -2.62904129 | 0.84868251 |
| 1 | -1.72438853 | -3.40435276 | 2.39867284 |
| 1 | -2.28687211 | -4.12403420 | 0.87182428 |
| 1 | -4.91279068 | -1.53794340 | 0.10449421 |
| 1 | -3.25160960 | -1.48448124 | -0.53706476 |
| 1 | -4.13516356 | -3.03068864 | -0.46923529 |
| 1 | -0.78943837 | 1.29109461 | -6.05668644 |
| 1 | -0.06021691 | 2.86098377 | -5.67253598 |
| 1 | -1.76134120 | 2.53066422 | -5.24437906 |
| 1 | -1.04847571 | -0.10344050 | -2.64125789 |
| 1 | -2.32638498 | 0.85421091 | -3.42600273 |
| 1 | -1.40386053 | -0.33936001 | -4.36867555 |
| 1 | 1.81902471 | 1.60490235 | -4.51214589 |
| 1 | 1.46557191 | 0.36356443 | -3.28334207 |
| 1 | 1.01562460 | 0.09192555 | -4.98699061 |
| 1 | 1.86696205 | 1.37490949 | 2.73352146 |
| 8 | 3.04723503 | 0.11287231 | 0.92918481 |
| 6 | 4.36097325 | 0.39014868 | 0.40173976 |
| 1 | 4.58032920 | 1.42378251 | 0.69552276 |
| 8 | 1.18797079 | -1.15523436 | 1.04158277 |
| 1 | 4.37155942 | 0.30588181 | -0.69179468 |
| 1 | 5.12183980 | -0.27664791 | 0.83288085 |
| 31 | 0.16797425 | 0.24576608 | 0.40688496 |
| 8 | 2.73219788 | -1.49582566 | -0.67253770 |
| 6 | 2.52217578 | -1.19968551 | 0.70781840 |
| 6 | 3.15892852 | -2.30637339 | 1.60084123 |
| 8 | 4.39346714 | -2.82326271 | 1.02066014 |
| 6 | 4.30180312 | -3.27618633 | -0.26858375 |
| 6 | 2.99580324 | -2.88474933 | -0.96751116 |
| 8 | 5.22066928 | -3.88279930 | -0.77122849 |
| 6 | 3.07481290 | -3.03908216 | -2.47399851 |

| | | | |
|---|---|---|---|
| 6 | 3.46983806 | -1.85844510 | 3.01525547 |
| 1 | 2.42589981 | -3.12752465 | 1.62906992 |
| 1 | 2.17556101 | -3.51439194 | -0.57072160 |
| 1 | 2.11916700 | -2.74676539 | -2.92632420 |
| 1 | 3.29464152 | -4.08232167 | -2.72963902 |
| 1 | 3.87507557 | -2.40969391 | -2.88224466 |
| 1 | 2.55008361 | -1.49955988 | 3.49363513 |
| 1 | 4.21218846 | -1.05156469 | 3.02210763 |
| 1 | 3.85996269 | -2.70498938 | 3.59408382 |

Electronic energy = -3673.041383 a.u.
DFT-D3(BJ) dispersion correction = -0.116530 a.u.
Thermal free energy = 0.585588 a.u.
Gibbs free energy = -3672.572325 a.u.
Number of imaginary frequencies = 0.

**Int1 LA isomer/conformer 7**

| Atomic N. | X | Y | Z |
|---|---|---|---|
| 7 | -0.27549247 | 1.89700549 | 1.21350634 |
| 8 | -1.67111697 | 0.03544636 | -0.67224527 |
| 8 | 1.39501182 | 0.29946285 | -0.72768353 |
| 6 | -0.63134077 | 1.85477834 | 2.65586980 |
| 6 | -2.78372862 | 0.65840080 | -0.20085692 |
| 6 | -4.06289053 | 0.04942802 | -0.34670177 |
| 6 | -5.16656057 | 0.75283759 | 0.15809582 |
| 6 | -5.05411826 | 1.99962668 | 0.78018903 |
| 6 | -3.80132424 | 2.59172640 | 0.89775473 |
| 6 | -2.66802704 | 1.93846653 | 0.40099600 |
| 6 | -1.33271034 | 2.63148656 | 0.42065300 |
| 1 | -5.94295921 | 2.50230224 | 1.16124134 |
| 6 | -4.23365397 | -1.32178196 | -1.02951124 |
| 6 | 1.72252932 | 1.53035659 | -1.20199805 |
| 6 | 2.26895726 | 1.66275695 | -2.51176467 |
| 6 | 2.60498248 | 2.95536611 | -2.93886370 |
| 6 | 2.42054825 | 4.08950986 | -2.14336319 |
| 6 | 1.89211589 | 3.94521761 | -0.86607380 |
| 6 | 1.55285514 | 2.67602946 | -0.38104118 |
| 6 | 1.08189031 | 2.54380879 | 1.04545474 |
| 1 | 2.69042124 | 5.07448184 | -2.52371168 |
| 6 | 2.48019237 | 0.44195754 | -3.42961741 |
| 6 | -5.71280378 | -1.74533679 | -1.10475550 |
| 6 | -3.48242275 | -2.41279178 | -0.23034217 |
| 6 | -3.69829575 | -1.26294872 | -2.48019402 |
| 6 | 3.10953852 | 0.83912929 | -4.77851896 |
| 6 | 1.12293261 | -0.23372807 | -3.73737950 |
| 6 | 3.43807537 | -0.57136480 | -2.75914929 |
| 1 | -0.66736205 | 2.88078120 | 3.05585026 |
| 1 | -1.61207743 | 1.38282048 | 2.77599281 |
| 1 | -1.43193409 | 3.64430774 | 0.84464883 |
| 1 | -0.93023083 | 2.72891767 | -0.59818963 |
| 1 | -6.15748578 | 0.31130193 | 0.06967593 |
| 1 | -3.69329450 | 3.57588015 | 1.35935850 |
| 1 | 1.77349790 | 1.92066030 | 1.63369158 |

```
   1     1.03973673   3.54115509   1.51292231
   1     3.02106948   3.08950444  -3.93548538
   1     1.74933504   4.81748396  -0.22439041
   1    -5.77921741  -2.71850896  -1.61154048
   1    -6.16294399  -1.86173124  -0.10819071
   1    -6.31922128  -1.03086310  -1.68010145
   1    -2.40791885  -2.21040050  -0.17067321
   1    -3.87707800  -2.48422495   0.79367341
   1    -3.62400628  -3.39023498  -0.71641452
   1    -4.25511611  -0.51857564  -3.06800063
   1    -2.63459768  -1.00342725  -2.50867706
   1    -3.83150009  -2.24255524  -2.96391955
   1     3.24678259  -0.06554232  -5.38748777
   1     4.09701091   1.30655974  -4.65373835
   1     2.46793828   1.52566479  -5.34940026
   1     0.63002218  -0.59355748  -2.82812767
   1     0.44535899   0.46995982  -4.24184131
   1     1.27943743  -1.09214294  -4.40798090
   1     4.41810753  -0.11062734  -2.56711235
   1     3.03187753  -0.93733853  -1.81003575
   1     3.59443256  -1.43245011  -3.42620183
   1     0.11458320   1.26447148   3.19822099
   8     0.17722703  -1.29892162   3.56030538
   6     0.95480662  -1.35004097   4.77010987
   1     0.29013195  -0.97426508   5.55684520
   8    -0.07428818  -1.46449946   1.34179894
   1     1.84464826  -0.70744122   4.70262162
   1     1.26804989  -2.37379109   5.02106288
  31    -0.11064081   0.06658722   0.32991970
   8     1.96954639  -0.75966943   2.23242954
   6     0.83805931  -1.64803173   2.35403366
   6     1.31153543  -3.12799142   2.26764239
   8     2.56701743  -3.33448914   2.98339019
   6     3.59581217  -2.50925835   2.62522847
   6     3.18151232  -1.35481483   1.70421319
   8     4.71264770  -2.69904020   3.05291011
   6     4.24400080  -0.27711563   1.62023111
   6     0.29773654  -4.12528437   2.79229720
   1     1.49496340  -3.32208562   1.19933724
   1     2.98440555  -1.75811456   0.69269593
   1     3.93096866   0.50270370   0.91537715
   1     5.18569117  -0.71553658   1.26988078
   1     4.42202943   0.16562029   2.60845663
   1    -0.64399502  -4.00352659   2.24316982
   1     0.10132775  -3.97345598   3.85979686
   1     0.66899492  -5.14645533   2.63973176
Electronic energy = -3673.039710 a.u.
DFT-D3(BJ) dispersion correction = -0.118415 a.u.
Thermal free energy = 0.586702 a.u.
Gibbs free energy = -3672.571423 a.u.
Number of imaginary frequencies = 0.
```

**Int1 LA isomer/conformer 8**

| Atomic N. | X | Y | Z |
|---|---|---|---|
| 7 | 1.57843272 | -0.21173029 | -1.60351176 |
| 8 | -0.02457102 | 1.22714343 | 0.49574916 |
| 8 | -1.42646361 | -0.33676744 | -1.70614542 |
| 6 | 2.72565960 | -1.07795000 | -1.23214468 |
| 6 | 1.17318417 | 1.78738741 | 0.80857284 |
| 6 | 1.34766106 | 2.47207257 | 2.04573213 |
| 6 | 2.62390505 | 2.98915989 | 2.31561963 |
| 6 | 3.69321730 | 2.88149281 | 1.42193034 |
| 6 | 3.48885988 | 2.27270236 | 0.18791966 |
| 6 | 2.23435144 | 1.74369136 | -0.13375380 |
| 6 | 1.96734210 | 1.24455383 | -1.52857753 |
| 1 | 4.66771020 | 3.29407155 | 1.68282844 |
| 6 | 0.18122743 | 2.67684464 | 3.03262552 |
| 6 | -1.26089473 | 0.32159370 | -2.87654078 |
| 6 | -2.35141946 | 1.03169740 | -3.45934372 |
| 6 | -2.11509037 | 1.68125906 | -4.67882980 |
| 6 | -0.87550446 | 1.65651960 | -5.32573683 |
| 6 | 0.17609112 | 0.94996810 | -4.75407291 |
| 6 | -0.00864111 | 0.27195981 | -3.54275096 |
| 6 | 1.10898726 | -0.57740429 | -2.99803384 |
| 1 | -0.74071620 | 2.18408427 | -6.26982486 |
| 6 | -3.73358995 | 1.08523700 | -2.77958550 |
| 6 | 0.60214843 | 3.50950140 | 4.25806699 |
| 6 | -0.33506602 | 1.31702389 | 3.55207179 |
| 6 | -0.96625915 | 3.43993389 | 2.32790951 |
| 6 | -4.75428422 | 1.88120125 | -3.61470400 |
| 6 | -3.62269517 | 1.78205593 | -1.40270738 |
| 6 | -4.29638945 | -0.34575972 | -2.60582964 |
| 1 | 3.53527367 | -0.94945481 | -1.96852288 |
| 1 | 3.08712677 | -0.79252162 | -0.23937149 |
| 1 | 2.85552221 | 1.39304158 | -2.16484082 |
| 1 | 1.13266741 | 1.80114970 | -1.97935076 |
| 1 | 2.79489664 | 3.50147739 | 3.26062533 |
| 1 | 4.29491575 | 2.22512134 | -0.54786825 |
| 1 | 0.80395159 | -1.63428898 | -2.93318727 |
| 1 | 1.98061928 | -0.51670503 | -3.67014896 |
| 1 | -2.92635835 | 2.23686635 | -5.14564694 |
| 1 | 1.14849480 | 0.90553167 | -5.24982669 |
| 1 | -0.26878652 | 3.64058057 | 4.91582750 |
| 1 | 1.38786299 | 3.01439112 | 4.84716591 |
| 1 | 0.95701482 | 4.51124780 | 3.97609093 |
| 1 | -0.67752793 | 0.68082128 | 2.73019784 |
| 1 | 0.45808134 | 0.78432641 | 4.09698232 |
| 1 | -1.17449931 | 1.47736623 | 4.24586003 |
| 1 | -0.62239791 | 4.42696399 | 1.98601892 |
| 1 | -1.34844613 | 2.88588544 | 1.46348269 |
| 1 | -1.79532994 | 3.59763790 | 3.03429526 |
| 1 | -5.72142378 | 1.87861416 | -3.09228561 |
| 1 | -4.91374821 | 1.43585892 | -4.60743685 |
| 1 | -4.45426383 | 2.93080722 | -3.74725340 |
| 1 | -2.94883245 | 1.24362475 | -0.72808025 |

| | | | |
|---|---|---|---|
| 1 | -3.24972738 | 2.81020541 | -1.51668934 |
| 1 | -4.61602044 | 1.83221594 | -0.93145521 |
| 1 | -4.42042333 | -0.83349664 | -3.58384210 |
| 1 | -3.63826205 | -0.96583557 | -1.98778270 |
| 1 | -5.28539561 | -0.29864395 | -2.12513251 |
| 1 | 2.40441483 | -2.12484451 | -1.20450961 |
| 8 | 1.23618206 | -1.46676283 | 1.76124620 |
| 6 | 2.37048087 | -1.77367226 | 2.59511058 |
| 1 | 2.84670403 | -0.80864614 | 2.80250126 |
| 8 | -0.44925455 | -2.00712482 | 0.39420758 |
| 1 | 3.07612628 | -2.43647728 | 2.07597003 |
| 1 | 2.06178210 | -2.23894331 | 3.54209247 |
| 31 | -0.08676576 | -0.38912692 | -0.42098696 |
| 8 | 1.36682667 | -3.44991128 | 0.61259612 |
| 6 | 0.45395850 | -2.54954588 | 1.27190045 |
| 6 | -0.36186307 | -3.32803881 | 2.34375414 |
| 8 | 0.45540394 | -4.32741901 | 3.02743754 |
| 6 | 1.09527070 | -5.22516661 | 2.22005538 |
| 6 | 1.05324112 | -4.85416301 | 0.73388287 |
| 8 | 1.66431968 | -6.18362315 | 2.69319082 |
| 6 | 2.06197230 | -5.63824591 | -0.08349087 |
| 6 | -0.99829873 | -2.45184462 | 3.40358745 |
| 1 | -1.15126503 | -3.85713194 | 1.78708735 |
| 1 | 0.03346142 | -5.04676768 | 0.34785247 |
| 1 | 1.98502130 | -5.35715641 | -1.14144101 |
| 1 | 1.86673879 | -6.71227067 | 0.01786523 |
| 1 | 3.08080526 | -5.44211714 | 0.27315159 |
| 1 | -1.65821311 | -1.71974535 | 2.92293647 |
| 1 | -0.24318252 | -1.91274952 | 3.98673281 |
| 1 | -1.59604398 | -3.07283693 | 4.08251230 |

Electronic energy = -3673.039732 a.u.
DFT-D3(BJ) dispersion correction = -0.118342 a.u.
Thermal free energy = 0.587235 a.u.
Gibbs free energy = -3672.570838 a.u.
Number of imaginary frequencies = 0.

**Int1 LA isomer/conformer 9**

| Atomic N. | X | Y | Z |
|---|---|---|---|
| 7 | -0.50963475 | 1.27330309 | 1.61841721 |
| 8 | -1.00868748 | 0.56492403 | -1.24468847 |
| 8 | 1.76500936 | 0.10576918 | 0.02714500 |
| 6 | -1.45309966 | 0.81746702 | 2.67006612 |
| 6 | -2.19380028 | 1.19421319 | -1.02437012 |
| 6 | -3.30120773 | 0.97009382 | -1.89143398 |
| 6 | -4.48912590 | 1.65716499 | -1.59943910 |
| 6 | -4.61469908 | 2.53459539 | -0.51834346 |
| 6 | -3.51735231 | 2.76375838 | 0.30479514 |
| 6 | -2.30537772 | 2.11178883 | 0.05236599 |
| 6 | -1.08353212 | 2.45004076 | 0.86159656 |
| 1 | -5.56224569 | 3.03982122 | -0.33220058 |
| 6 | -3.20435809 | 0.01926438 | -3.10053672 |
| 6 | 2.30930576 | 1.33885079 | 0.22060323 |

| | | | |
|---|---|---|---|
| 6 | 3.35447118 | 1.80003682 | -0.63099486 |
| 6 | 3.88665808 | 3.06902599 | -0.35977718 |
| 6 | 3.43324229 | 3.87597186 | 0.68723787 |
| 6 | 2.41457944 | 3.41220898 | 1.51106979 |
| 6 | 1.85613235 | 2.14674817 | 1.29455710 |
| 6 | 0.81792149 | 1.62783225 | 2.25407757 |
| 1 | 3.87596118 | 4.85809814 | 0.85189819 |
| 6 | 3.88430356 | 0.95171731 | -1.80423891 |
| 6 | -4.51227099 | -0.01063432 | -3.91368608 |
| 6 | -2.92554396 | -1.42500498 | -2.62188389 |
| 6 | -2.08057825 | 0.48803720 | -4.05556349 |
| 6 | 5.01826461 | 1.66545869 | -2.56455188 |
| 6 | 2.75137378 | 0.68048850 | -2.82198074 |
| 6 | 4.45638741 | -0.38480184 | -1.27613813 |
| 1 | -1.61111335 | 1.62636590 | 3.40127645 |
| 1 | -2.41260979 | 0.55531524 | 2.21167122 |
| 1 | -1.31245152 | 3.24343415 | 1.59200891 |
| 1 | -0.27478542 | 2.81099253 | 0.21005998 |
| 1 | -5.35762992 | 1.50181230 | -2.23695814 |
| 1 | -3.58520503 | 3.46645706 | 1.13841589 |
| 1 | 1.15990404 | 0.70253222 | 2.74308470 |
| 1 | 0.62697549 | 2.37957851 | 3.03715699 |
| 1 | 4.68379951 | 3.45186210 | -0.99398595 |
| 1 | 2.05000403 | 4.02306661 | 2.33982109 |
| 1 | -4.38391991 | -0.68831228 | -4.76952134 |
| 1 | -5.36112318 | -0.38437593 | -3.32286128 |
| 1 | -4.77436691 | 0.98001566 | -4.31261206 |
| 1 | -1.98440363 | -1.49977878 | -2.06719735 |
| 1 | -3.73897497 | -1.78097355 | -1.97259908 |
| 1 | -2.86882297 | -2.09865548 | -3.49040036 |
| 1 | -2.29088504 | 1.49919535 | -4.43365404 |
| 1 | -1.10482573 | 0.49853874 | -3.55819098 |
| 1 | -2.02376901 | -0.19010896 | -4.92046590 |
| 1 | 5.36241982 | 1.01567187 | -3.38151468 |
| 1 | 5.88467471 | 1.87152903 | -1.91964459 |
| 1 | 4.68469712 | 2.61156648 | -3.01461042 |
| 1 | 1.92383431 | 0.12091952 | -2.37366319 |
| 1 | 2.35274237 | 1.62446888 | -3.22032720 |
| 1 | 3.14475778 | 0.09576726 | -3.66712044 |
| 1 | 5.29568387 | -0.20395161 | -0.58887546 |
| 1 | 3.69354664 | -0.96514018 | -0.74662228 |
| 1 | 4.83222402 | -0.98703177 | -2.11699607 |
| 1 | -1.03218779 | -0.06074869 | 3.17087472 |
| 8 | 0.66917213 | -3.71560460 | 0.77505823 |
| 6 | 1.79420304 | -3.17448401 | 0.06680534 |
| 1 | 2.34738560 | -2.44137515 | 0.67298690 |
| 8 | -0.69020534 | -1.84858709 | 0.40494896 |
| 8 | 0.36952607 | -2.03422212 | 2.40748049 |
| 1 | 2.43982657 | -4.03072221 | -0.15741854 |
| 1 | 1.47876496 | -2.70089337 | -0.87414039 |
| 31 | -0.06569882 | -0.12968922 | 0.19525036 |
| 6 | -0.23201787 | -2.76578623 | 1.31252680 |
| 6 | -1.39873543 | -3.61147682 | 1.86895875 |

| | | | |
|---|---|---|---|
| 8 | -0.95371669 | -4.45943613 | 2.97679164 |
| 6 | 0.12381587 | -4.14366389 | 3.73621988 |
| 6 | 0.96177088 | -2.90347969 | 3.39564930 |
| 8 | 0.43990117 | -4.86769049 | 4.65841953 |
| 6 | 1.24197752 | -2.07498829 | 4.64369552 |
| 6 | -2.60531803 | -2.80945598 | 2.32424655 |
| 1 | -1.68677568 | -4.32490464 | 1.08516838 |
| 1 | 1.91243573 | -3.30425977 | 3.00211005 |
| 1 | 1.93022275 | -1.25485995 | 4.40065485 |
| 1 | 1.70082502 | -2.71219003 | 5.40765466 |
| 1 | 0.31204617 | -1.65506983 | 5.05105228 |
| 1 | -3.02604264 | -2.25071520 | 1.47952951 |
| 1 | -2.33470010 | -2.10732923 | 3.12382399 |
| 1 | -3.36894891 | -3.49536607 | 2.71197562 |

Electronic energy = -3673.038978 a.u.
DFT-D3(BJ) dispersion correction = -0.118796 a.u.
Thermal free energy = 0.586973 a.u.
Gibbs free energy = -3672.570801 a.u.
Number of imaginary frequencies = 0.

**Int1 LA isomer/conformer 10**

| Atomic N. | X | Y | Z |
|---|---|---|---|
| 7 | -0.98311551 | 0.53754719 | 1.86419828 |
| 8 | -0.30583487 | 1.21141780 | -0.97758675 |
| 8 | 1.82000845 | 0.00397708 | 0.91358845 |
| 6 | -2.22565719 | -0.21706769 | 2.17184259 |
| 6 | -1.52882900 | 1.80518831 | -0.95312484 |
| 6 | -2.21124404 | 2.09683829 | -2.16833550 |
| 6 | -3.46652775 | 2.71414854 | -2.06230325 |
| 6 | -4.05014463 | 3.04633214 | -0.83613079 |
| 6 | -3.36247857 | 2.77501972 | 0.34186776 |
| 6 | -2.10208827 | 2.16965990 | 0.29226788 |
| 6 | -1.30322112 | 1.98347974 | 1.55252921 |
| 1 | -5.03098138 | 3.52080403 | -0.80882297 |
| 6 | -1.60262873 | 1.75285888 | -3.54182441 |
| 6 | 2.14668381 | 0.93199176 | 1.85017646 |
| 6 | 3.40538879 | 1.59686543 | 1.78748165 |
| 6 | 3.68623135 | 2.53070583 | 2.79519625 |
| 6 | 2.79259832 | 2.82485758 | 3.82905201 |
| 6 | 1.57100049 | 2.16397811 | 3.88124026 |
| 6 | 1.24458484 | 1.21103391 | 2.90856867 |
| 6 | -0.03821555 | 0.43367835 | 3.04470059 |
| 1 | 3.05522368 | 3.56492188 | 4.58473115 |
| 6 | 4.41832337 | 1.31130578 | 0.66102334 |
| 6 | -2.50849425 | 2.20874766 | -4.70128759 |
| 6 | -1.41845808 | 0.22248422 | -3.67465250 |
| 6 | -0.24116909 | 2.46808890 | -3.71311121 |
| 6 | 5.71052680 | 2.13299830 | 0.82864027 |
| 6 | 3.80936563 | 1.68708881 | -0.71054733 |
| 6 | 4.82026694 | -0.18276612 | 0.67130252 |
| 1 | -2.72223599 | 0.23894010 | 3.04330779 |
| 1 | -2.89910085 | -0.18654239 | 1.30963358 |

| | | | |
|---|---|---|---|
| 1 | -1.84370582 | 2.40243049 | 2.41704381 |
| 1 | -0.33350791 | 2.49668944 | 1.47798304 |
| 1 | -4.01870653 | 2.94338958 | -2.97184654 |
| 1 | -3.78867214 | 3.04848980 | 1.30978202 |
| 1 | 0.15957775 | -0.64353843 | 3.15914698 |
| 1 | -0.58388868 | 0.77598613 | 3.93903263 |
| 1 | 4.63737249 | 3.05917928 | 2.77339073 |
| 1 | 0.86247024 | 2.37134892 | 4.68621469 |
| 1 | -2.02269933 | 1.95442411 | -5.65395502 |
| 1 | -3.48690396 | 1.70694361 | -4.68650778 |
| 1 | -2.67391514 | 3.29585895 | -4.69440321 |
| 1 | -0.74202117 | -0.17605198 | -2.91123890 |
| 1 | -2.38550894 | -0.29328240 | -3.58340910 |
| 1 | -1.00093909 | -0.01483209 | -4.66514176 |
| 1 | -0.36830670 | 3.55931459 | -3.66114820 |
| 1 | 0.47505238 | 2.16419248 | -2.94220800 |
| 1 | 0.18345548 | 2.22477039 | -4.69888812 |
| 1 | 6.39895513 | 1.88507458 | 0.00843863 |
| 1 | 6.22633276 | 1.90619187 | 1.77294477 |
| 1 | 5.52267847 | 3.21547949 | 0.78464342 |
| 1 | 2.90951381 | 1.10341444 | -0.93129099 |
| 1 | 3.54497958 | 2.75391760 | -0.73927515 |
| 1 | 4.54535916 | 1.49938920 | -1.50686187 |
| 1 | 5.28806993 | -0.45162660 | 1.62971522 |
| 1 | 3.95339789 | -0.83263075 | 0.51059168 |
| 1 | 5.55244168 | -0.37534556 | -0.12736346 |
| 1 | -1.96721766 | -1.25793126 | 2.39048725 |
| 8 | -0.04982671 | -2.91061667 | 1.42968211 |
| 6 | 1.33048364 | -3.28929215 | 1.59038741 |
| 1 | 1.58882486 | -3.05024737 | 2.62856412 |
| 8 | -0.09919057 | -1.74774602 | -0.59724504 |
| 1 | 1.98700637 | -2.71262081 | 0.92253514 |
| 1 | 1.46587134 | -4.36935009 | 1.43032775 |
| 31 | 0.09170078 | -0.12051583 | 0.25059179 |
| 8 | -1.95401642 | -2.77896979 | 0.20564312 |
| 6 | -0.53866397 | -2.85132849 | 0.07139332 |
| 6 | -0.18899867 | -4.10590531 | -0.75523195 |
| 8 | -0.68605797 | -5.30634728 | -0.08581927 |
| 6 | -1.82899624 | -5.25875447 | 0.64988985 |
| 6 | -2.53506643 | -3.91608588 | 0.87805271 |
| 8 | -2.25635742 | -6.27496504 | 1.15678587 |
| 6 | -4.00163955 | -3.99579189 | 0.46416276 |
| 6 | -0.70619252 | -4.04890076 | -2.18492159 |
| 1 | 0.90104707 | -4.23898180 | -0.76483973 |
| 1 | -2.46893791 | -3.76000338 | 1.96948531 |
| 1 | -4.50889431 | -3.05730714 | 0.72217856 |
| 1 | -4.48735936 | -4.82580097 | 0.98963278 |
| 1 | -4.09121764 | -4.15468492 | -0.61843841 |
| 1 | -0.21934833 | -3.22678879 | -2.72286077 |
| 1 | -1.79072543 | -3.88376165 | -2.20776065 |
| 1 | -0.47688591 | -4.99506144 | -2.69169354 |

Electronic energy = -3673.038974 a.u.
DFT-D3(BJ) dispersion correction = -0.117611 a.u.

Thermal free energy = 0.586004 a.u.
Gibbs free energy = -3672.570581 a.u.
Number of imaginary frequencies = 0.

**Int1_LA isomer/conformer 11**

| Atomic N. | X | Y | Z |
|---|---|---|---|
| 7 | -0.08656108 | 2.29506958 | -0.31327961 |
| 8 | 0.11555592 | -0.10796771 | 1.45986295 |
| 8 | -1.69006854 | -0.16914144 | -1.02159925 |
| 6 | 1.15182301 | 2.95784555 | -0.79679340 |
| 6 | 0.82051077 | 0.74006642 | 2.25461701 |
| 6 | 1.67937550 | 0.22878423 | 3.26953711 |
| 6 | 2.37696022 | 1.16587608 | 4.04632316 |
| 6 | 2.25250409 | 2.54679496 | 3.86703811 |
| 6 | 1.38738198 | 3.03232823 | 2.89228849 |
| 6 | 0.66065439 | 2.14154396 | 2.09462330 |
| 6 | -0.36587610 | 2.66317317 | 1.12583787 |
| 1 | 2.82181099 | 3.23258061 | 4.49437087 |
| 6 | 1.83118777 | -1.28522973 | 3.51622207 |
| 6 | -2.74511448 | 0.65294893 | -0.78807226 |
| 6 | -4.03402893 | 0.09616123 | -0.54243752 |
| 6 | -5.08861159 | 0.99298680 | -0.32055847 |
| 6 | -4.91988488 | 2.38058062 | -0.32925441 |
| 6 | -3.65982240 | 2.91032993 | -0.58089283 |
| 6 | -2.57280863 | 2.06176723 | -0.82462207 |
| 6 | -1.24689658 | 2.66790058 | -1.20908499 |
| 1 | -5.76991088 | 3.03640537 | -0.14195563 |
| 6 | -4.26454259 | -1.42794568 | -0.51577688 |
| 6 | 2.77248031 | -1.58214420 | 4.69899396 |
| 6 | 2.42881066 | -1.97775312 | 2.26904251 |
| 6 | 0.45448907 | -1.90434631 | 3.85752924 |
| 6 | -5.74365833 | -1.78056395 | -0.26877707 |
| 6 | -3.44266988 | -2.07060770 | 0.62661835 |
| 6 | -3.86496554 | -2.05256877 | -1.87403879 |
| 1 | 1.00920570 | 4.05051433 | -0.79985120 |
| 1 | 1.98237209 | 2.70446905 | -0.12940894 |
| 1 | -0.43143368 | 3.76193148 | 1.18955145 |
| 1 | -1.35933491 | 2.24972401 | 1.35292592 |
| 1 | 3.04991720 | 0.80805040 | 4.82336132 |
| 1 | 1.25300422 | 4.10769294 | 2.75448555 |
| 1 | -0.94633353 | 2.34297983 | -2.21699565 |
| 1 | -1.33060786 | 3.76708633 | -1.21369514 |
| 1 | -6.08371976 | 0.59805953 | -0.12478968 |
| 1 | -3.50889382 | 3.99192924 | -0.60559326 |
| 1 | 2.83254954 | -2.67041500 | 4.84096243 |
| 1 | 3.79413019 | -1.21783866 | 4.51801967 |
| 1 | 2.40683883 | -1.14507618 | 5.63955998 |
| 1 | 1.79588669 | -1.85022708 | 1.38474313 |
| 1 | 3.42752973 | -1.57718699 | 2.04237118 |
| 1 | 2.53765065 | -3.05585185 | 2.46054934 |
| 1 | 0.03983994 | -1.44853151 | 4.76862449 |
| 1 | -0.26385229 | -1.76779119 | 3.04195897 |

| | | | |
|---|---|---|---|
| 1 | 0.57047196 | -2.98301599 | 4.04239710 |
| 1 | -5.85419842 | -2.87415824 | -0.27223792 |
| 1 | -6.40182483 | -1.37912317 | -1.05296662 |
| 1 | -6.09999034 | -1.41708791 | 0.70589207 |
| 1 | -2.36736576 | -1.90900526 | 0.49751461 |
| 1 | -3.73978372 | -1.65437674 | 1.59994410 |
| 1 | -3.62761597 | -3.15518151 | 0.65152698 |
| 1 | -4.47616810 | -1.63384196 | -2.68707738 |
| 1 | -2.80905834 | -1.87436954 | -2.10411731 |
| 1 | -4.03855382 | -3.13900127 | -1.84717462 |
| 1 | 1.38417814 | 2.60633113 | -1.80796188 |
| 8 | 0.65793919 | 0.37027306 | -3.07263205 |
| 6 | 0.77030654 | 0.71512697 | -4.46512086 |
| 1 | 0.58981092 | -0.15701845 | -5.11208316 |
| 8 | 1.42322457 | -0.55807852 | -1.16227203 |
| 8 | 2.89705387 | 0.42384623 | -2.67594446 |
| 1 | -0.01576664 | 1.45588540 | -4.65356056 |
| 1 | 1.75232170 | 1.15339633 | -4.68806138 |
| 31 | -0.01750443 | 0.25507764 | -0.35626316 |
| 8 | 3.09733746 | -2.40952914 | -2.60620625 |
| 6 | 4.15236104 | -1.69953639 | -2.12973912 |
| 6 | 4.12355066 | -0.16683733 | -2.19008399 |
| 6 | 1.72370899 | -0.37342303 | -2.48651597 |
| 6 | 1.94793070 | -1.72678253 | -3.19506193 |
| 8 | 5.11645533 | -2.28657360 | -1.68575514 |
| 6 | 0.77643510 | -2.68412584 | -3.09552678 |
| 6 | 4.53686331 | 0.43873707 | -0.84690455 |
| 1 | 4.88389099 | 0.09391093 | -2.94622743 |
| 1 | 2.20284444 | -1.52541660 | -4.24768544 |
| 1 | 1.01731931 | -3.62134969 | -3.61275733 |
| 1 | -0.11744545 | -2.24351692 | -3.55628321 |
| 1 | 0.55806840 | -2.90197939 | -2.04340136 |
| 1 | 5.50917587 | 0.03365950 | -0.54218639 |
| 1 | 3.79276003 | 0.20419265 | -0.07666206 |
| 1 | 4.61839414 | 1.52849855 | -0.95134588 |

Electronic energy = -3673.036652 a.u.
DFT-D3(BJ) dispersion correction = -0.118265 a.u.
Thermal free energy = 0.585383 a.u.
Gibbs free energy = -3672.569534 a.u.
Number of imaginary frequencies = 0.

**Int1 LA isomer/conformer 12**

| Atomic N. | X | Y | Z |
|---|---|---|---|
| 7 | -1.69117419 | -1.32628285 | -0.74491114 |
| 8 | -0.66113382 | 1.43531038 | -0.23358842 |
| 8 | -0.11857801 | -0.81115110 | 1.78370895 |
| 6 | -1.44997362 | -1.95426106 | -2.06920557 |
| 6 | -1.42868145 | 1.74458814 | -1.31174440 |
| 6 | -1.20014728 | 2.94954312 | -2.03609375 |
| 6 | -2.03509888 | 3.20169214 | -3.13475384 |
| 6 | -3.05892840 | 2.33467472 | -3.52794692 |
| 6 | -3.28578606 | 1.17306910 | -2.79778007 |

| | | | |
|---|---|---|---|
| 6 | -2.48898644 | 0.87824220 | -1.68593913 |
| 6 | -2.81018763 | -0.31324089 | -0.82545459 |
| 1 | -3.67573595 | 2.57480565 | -4.39391877 |
| 6 | -0.08700149 | 3.93724820 | -1.63519428 |
| 6 | -1.29041675 | -1.14225408 | 2.38229670 |
| 6 | -1.51965602 | -0.77091312 | 3.73953330 |
| 6 | -2.74201918 | -1.15036315 | 4.31153471 |
| 6 | -3.72053638 | -1.86053159 | 3.60996045 |
| 6 | -3.48023016 | -2.22525771 | 2.29052012 |
| 6 | -2.27002228 | -1.88612277 | 1.67279567 |
| 6 | -1.99522290 | -2.39540435 | 0.28046058 |
| 1 | -4.65949117 | -2.12453939 | 4.09618206 |
| 6 | -0.47173221 | 0.01896028 | 4.54861522 |
| 6 | -0.07504466 | 5.18523105 | -2.53826971 |
| 6 | 1.29859771 | 3.26188920 | -1.76491740 |
| 6 | -0.30740100 | 4.42376088 | -0.18275959 |
| 6 | -0.93391205 | 0.26998798 | 5.99657021 |
| 6 | -0.22874386 | 1.40181792 | 3.89871205 |
| 6 | 0.85475056 | -0.77470640 | 4.62203091 |
| 1 | -2.34701989 | -2.51200236 | -2.38306584 |
| 1 | -1.23064590 | -1.17523935 | -2.80692481 |
| 1 | -3.70881078 | -0.82884028 | -1.20274464 |
| 1 | -3.00691221 | -0.00260808 | 0.21103291 |
| 1 | -1.88052131 | 4.10936167 | -3.71523567 |
| 1 | -4.09619095 | 0.49366403 | -3.07193339 |
| 1 | -1.11584876 | -3.05768407 | 0.27353716 |
| 1 | -2.86267370 | -2.97277685 | -0.07945696 |
| 1 | -2.94802851 | -0.87638308 | 5.34452837 |
| 1 | -4.22633083 | -2.79118091 | 1.72807500 |
| 1 | 0.72341958 | 5.86084338 | -2.20034672 |
| 1 | 0.12943613 | 4.93510151 | -3.58938275 |
| 1 | -1.02252494 | 5.74148566 | -2.48920339 |
| 1 | 1.39162888 | 2.38509847 | -1.11595956 |
| 1 | 1.48043862 | 2.94316517 | -2.80134160 |
| 1 | 2.08695024 | 3.97897972 | -1.49027318 |
| 1 | -1.27228462 | 4.94378902 | -0.09041812 |
| 1 | -0.29097923 | 3.59175531 | 0.52934985 |
| 1 | 0.48654314 | 5.13432429 | 0.09300981 |
| 1 | -0.14824467 | 0.82346594 | 6.53007427 |
| 1 | -1.10815211 | -0.66749665 | 6.54437467 |
| 1 | -1.85062908 | 0.87559025 | 6.04063851 |
| 1 | 0.15217485 | 1.31167920 | 2.87607587 |
| 1 | -1.16005932 | 1.98549213 | 3.86963556 |
| 1 | 0.50623209 | 1.96639112 | 4.49221947 |
| 1 | 0.69999948 | -1.74042451 | 5.12540316 |
| 1 | 1.26666480 | -0.96311154 | 3.62476271 |
| 1 | 1.59492661 | -0.20564810 | 5.20478198 |
| 1 | -0.59232443 | -2.63208471 | -2.00099326 |
| 8 | 1.49285503 | -2.61556027 | -0.23705637 |
| 6 | 1.91215132 | -3.98221755 | -0.35997702 |
| 1 | 2.88996362 | -4.15479838 | 0.11700106 |
| 8 | 1.52952446 | -0.45427673 | -0.86697833 |
| 8 | 2.05352319 | -2.06666348 | -2.41590681 |

| | | | |
|---|---|---|---|
| 1 | 1.16016658 | -4.57788625 | 0.17099698 |
| 1 | 1.94818914 | -4.29389145 | -1.41408606 |
| 31 | -0.08434327 | -0.31310064 | 0.00171199 |
| 8 | 4.32105567 | -0.60294006 | -1.58595937 |
| 6 | 4.14676966 | -0.84978909 | -2.91469059 |
| 6 | 3.24899473 | -2.05268301 | -3.22720112 |
| 6 | 2.17350295 | -1.64347906 | -1.04863056 |
| 6 | 3.66866388 | -1.52427751 | -0.66431805 |
| 8 | 4.70423717 | -0.16946225 | -3.74651678 |
| 6 | 3.89412166 | -0.99115184 | 0.73599946 |
| 6 | 2.82107419 | -2.08742998 | -4.68275365 |
| 1 | 3.85372849 | -2.95626186 | -3.00080223 |
| 1 | 4.14749124 | -2.51148029 | -0.78014450 |
| 1 | 4.96937510 | -0.94926648 | 0.94986993 |
| 1 | 3.40900337 | -1.64438458 | 1.47270047 |
| 1 | 3.46978439 | 0.01538118 | 0.82469341 |
| 1 | 3.70432106 | -2.12562148 | -5.33087468 |
| 1 | 2.24829310 | -1.18583930 | -4.93105399 |
| 1 | 2.19981366 | -2.97341851 | -4.86668595 |

Electronic energy = -3673.036606 a.u.
DFT-D3(BJ) dispersion correction = -0.116414 a.u.
Thermal free energy = 0.583945 a.u.
Gibbs free energy = -3672.569075 a.u.
Number of imaginary frequencies = 0.

**Int1 LA isomer/conformer 13**

| Atomic N. | X | Y | Z |
|---|---|---|---|
| 7 | 2.04867658 | 1.05492720 | -1.36668786 |
| 8 | 0.84794032 | -0.97967785 | 0.48118072 |
| 8 | -0.48100364 | 1.79898844 | 0.07477618 |
| 6 | 2.46432136 | 0.56139852 | -2.70305029 |
| 6 | 2.06868023 | -1.56491362 | 0.35663888 |
| 6 | 2.23801504 | -2.92987685 | 0.73312524 |
| 6 | 3.51486402 | -3.48658417 | 0.56814160 |
| 6 | 4.60191168 | -2.76223435 | 0.07136137 |
| 6 | 4.43247603 | -1.42086407 | -0.25218463 |
| 6 | 3.18221538 | -0.81064542 | -0.09822036 |
| 6 | 3.05593187 | 0.67410165 | -0.31398193 |
| 1 | 5.57308181 | -3.24313193 | -0.04500045 |
| 6 | 1.07832586 | -3.76283518 | 1.31445315 |
| 6 | 0.29685430 | 2.79540132 | 0.57201434 |
| 6 | -0.09422065 | 3.47397072 | 1.76407824 |
| 6 | 0.73654346 | 4.50897537 | 2.21587926 |
| 6 | 1.90943084 | 4.88630405 | 1.55584417 |
| 6 | 2.27728994 | 4.22066078 | 0.39304191 |
| 6 | 1.47665464 | 3.18828134 | -0.11121723 |
| 6 | 1.85114975 | 2.55184038 | -1.42310604 |
| 1 | 2.52496280 | 5.69397429 | 1.95157395 |
| 6 | -1.37283969 | 3.08974442 | 2.53318924 |
| 6 | 1.53023362 | -5.18318224 | 1.70408537 |
| 6 | -0.05395503 | -3.90681529 | 0.27227570 |
| 6 | 0.53402708 | -3.08794676 | 2.59655460 |

| | | | |
|---|---|---|---|
| 6 | -1.59316898 | 3.98210269 | 3.76946382 |
| 6 | -1.26284245 | 1.63116482 | 3.03725563 |
| 6 | -2.61205565 | 3.25421842 | 1.62230748 |
| 1 | 3.41173068 | 1.04064085 | -2.99910174 |
| 1 | 2.60642348 | -0.52385088 | -2.66365423 |
| 1 | 4.03313790 | 1.10037333 | -0.59665543 |
| 1 | 2.72811150 | 1.17335643 | 0.60990495 |
| 1 | 3.67250241 | -4.52970871 | 0.83564828 |
| 1 | 5.27645217 | -0.82536532 | -0.60782774 |
| 1 | 1.06481433 | 2.71511708 | -2.17793800 |
| 1 | 2.78237109 | 3.00542491 | -1.80138709 |
| 1 | 0.46458645 | 5.04242374 | 3.12468624 |
| 1 | 3.18463396 | 4.50417410 | -0.14502720 |
| 1 | 0.67287946 | -5.72464934 | 2.12849459 |
| 1 | 1.88613339 | -5.75881961 | 0.83721738 |
| 1 | 2.32395917 | -5.17195738 | 2.46510159 |
| 1 | -0.46677573 | -2.93705704 | -0.02173056 |
| 1 | 0.31239681 | -4.41454765 | -0.63212524 |
| 1 | -0.86887973 | -4.51624139 | 0.69270864 |
| 1 | 1.32182227 | -3.01493222 | 3.36056362 |
| 1 | 0.15237746 | -2.08184193 | 2.39236132 |
| 1 | -0.28455183 | -3.69398765 | 3.01395673 |
| 1 | -2.51963545 | 3.66975078 | 4.27178441 |
| 1 | -1.70612262 | 5.04201954 | 3.49939463 |
| 1 | -0.77550682 | 3.89230000 | 4.49941640 |
| 1 | -1.12777671 | 0.92375538 | 2.21216361 |
| 1 | -0.41248723 | 1.52196086 | 3.72603393 |
| 1 | -2.17775734 | 1.35705887 | 3.58441641 |
| 1 | -2.72299162 | 4.30217645 | 1.30781746 |
| 1 | -2.54575418 | 2.63201489 | 0.72459512 |
| 1 | -3.52190324 | 2.97099847 | 2.17209882 |
| 1 | 1.68299633 | 0.79535085 | -3.43405005 |
| 8 | -1.59855557 | -2.20449448 | -2.58067205 |
| 6 | -0.96759012 | -2.55124444 | -3.81682952 |
| 1 | -0.03391496 | -1.98785050 | -3.95936835 |
| 8 | -0.48733576 | -0.16109496 | -2.37409490 |
| 8 | -2.05964704 | -0.77530610 | -0.89785273 |
| 1 | -0.73722971 | -3.62083241 | -3.74680846 |
| 1 | -1.63358410 | -2.39174753 | -4.68263705 |
| 31 | 0.24973861 | 0.29619638 | -0.72529840 |
| 8 | -3.58320763 | 0.84101402 | -2.39643191 |
| 6 | -4.10710325 | 0.44527357 | -1.19824926 |
| 6 | -3.48384809 | -0.83651845 | -0.63982356 |
| 6 | -1.69091448 | -0.80556550 | -2.27754244 |
| 6 | -2.81956922 | -0.15150897 | -3.14918748 |
| 8 | -4.95756449 | 1.10840747 | -0.64905242 |
| 6 | -2.31700349 | 0.57327771 | -4.38391258 |
| 6 | -3.70341861 | -0.99726912 | 0.85088646 |
| 1 | -3.90137258 | -1.70693386 | -1.17922667 |
| 1 | -3.51632268 | -0.95776305 | -3.42942686 |
| 1 | -3.16941039 | 0.99400502 | -4.93223466 |
| 1 | -1.77436238 | -0.10998789 | -5.04823480 |
| 1 | -1.64015801 | 1.38371752 | -4.08991609 |

```
   1    -4.77873196  -1.03908922   1.05997969
   1    -3.28104802  -0.14476102   1.39567340
   1    -3.22853297  -1.92320828   1.19760173
Electronic energy = -3673.035486 a.u.
DFT-D3(BJ) dispersion correction = -0.118707 a.u.
Thermal free energy = 0.585121 a.u.
Gibbs free energy = -3672.569072 a.u.
Number of imaginary frequencies = 0.
```

**Int1 LA isomer/conformer 14**

```
Atomic N.     X            Y            Z
   7    -0.99750947   1.80332086   0.91775640
   8     1.20354150   0.83614864  -0.86394646
   8     0.21836477  -0.89931902   1.49878402
   6    -2.24542432   2.30779133   0.28783512
   6     1.13587245   2.10301492  -1.35447889
   6     1.68348684   2.40454152  -2.63419701
   6     1.58496636   3.73238527  -3.07551404
   6     0.98487903   4.74206071  -2.31785481
   6     0.47227847   4.43755916  -1.06140620
   6     0.55438702   3.13105326  -0.56739704
   6     0.10517300   2.83169978   0.83786067
   1     0.92760500   5.75785147  -2.70866449
   6     2.35877078   1.32310918  -3.50053509
   6     0.58856254  -0.29796872   2.65982794
   6     1.62951417  -0.86948006   3.44691749
   6     1.96325163  -0.21439576   4.64078811
   6     1.32355583   0.95176660   5.07066728
   6     0.29976222   1.48992593   4.29967815
   6    -0.08468431   0.86945437   3.10465904
   6    -1.27538197   1.40507961   2.35094454
   1     1.62449406   1.42876711   6.00322540
   6     2.36024236  -2.15440137   3.00915912
   6     2.90900800   1.90215737  -4.81762179
   6     1.33585501   0.22397511  -3.87340857
   6     3.55260134   0.70012534  -2.73817960
   6     3.41467212  -2.59473400   4.04225976
   6     3.10033468  -1.91718287   1.67136633
   6     1.34981180  -3.31705583   2.85866014
   1    -2.60740115   3.19113799   0.83753513
   1    -2.04305541   2.58638030  -0.75175215
   1    -0.24911328   3.75215587   1.33053411
   1     0.93619172   2.42697724   1.43418216
   1     1.98694379   3.99410936  -4.05255706
   1     0.01930826   5.21601764  -0.44332854
   1    -2.07120739   0.64642678   2.28957713
   1    -1.68111196   2.28587847   2.87500526
   1     2.75834400  -0.62285299   5.26173383
   1    -0.22408866   2.39114017   4.62598097
   1     3.38959390   1.09503865  -5.38857360
   1     2.11503683   2.32544931  -5.44982531
   1     3.66669605   2.67953246  -4.64252322
```

| | | | |
|---|---|---|---|
| 1 | 0.92590980 | -0.26762524 | -2.98467470 |
| 1 | 0.50098448 | 0.65052429 | -4.44847562 |
| 1 | 1.82347264 | -0.53881376 | -4.49961154 |
| 1 | 4.30492410 | 1.46775998 | -2.50530533 |
| 1 | 3.23393929 | 0.23075829 | -1.80129833 |
| 1 | 4.03482285 | -0.06453677 | -3.36604850 |
| 1 | 3.89486301 | -3.51843765 | 3.68946117 |
| 1 | 2.96784191 | -2.80866828 | 5.02395817 |
| 1 | 4.20570322 | -1.84254428 | 4.17524314 |
| 1 | 2.40890544 | -1.65033204 | 0.86516145 |
| 1 | 3.83976513 | -1.10992648 | 1.77414926 |
| 1 | 3.63695811 | -2.83208267 | 1.37756643 |
| 1 | 0.85360828 | -3.52428467 | 3.81809618 |
| 1 | 0.58078207 | -3.09237445 | 2.11172443 |
| 1 | 1.88160623 | -4.23055128 | 2.55138944 |
| 1 | -3.00451647 | 1.51852707 | 0.31033262 |
| 8 | -1.76007091 | -2.79255260 | -0.46515527 |
| 6 | -0.93134230 | -3.45493596 | -1.42709513 |
| 1 | -0.19681244 | -2.76558388 | -1.87040899 |
| 8 | -1.38157794 | -0.61244482 | -1.22383160 |
| 8 | -3.00705664 | -1.08264910 | 0.29312126 |
| 1 | -0.40067238 | -4.23957989 | -0.87557981 |
| 1 | -1.52369878 | -3.92685248 | -2.23013779 |
| 31 | -0.25325127 | 0.12283259 | 0.03706873 |
| 8 | -4.57201309 | -0.96732986 | -1.85533260 |
| 6 | -5.19531776 | -1.05446287 | -0.64409462 |
| 6 | -4.32180182 | -1.66389221 | 0.45411785 |
| 6 | -2.32640138 | -1.53738459 | -0.87429120 |
| 6 | -3.35803514 | -1.76220746 | -2.04134100 |
| 8 | -6.31257780 | -0.61032998 | -0.49342588 |
| 6 | -2.84226054 | -1.40687810 | -3.42271744 |
| 6 | -4.83075000 | -1.35256875 | 1.84758351 |
| 1 | -4.25699740 | -2.75866778 | 0.31418550 |
| 1 | -3.65917044 | -2.82126161 | -2.00146554 |
| 1 | -3.62570660 | -1.60322465 | -4.16592011 |
| 1 | -1.95801888 | -2.00381400 | -3.67558217 |
| 1 | -2.56020011 | -0.34871937 | -3.45931423 |
| 1 | -5.83937520 | -1.76346799 | 1.97337532 |
| 1 | -4.88784969 | -0.26803106 | 2.00636236 |
| 1 | -4.16273917 | -1.79802679 | 2.59503637 |

Electronic energy = -3673.037297 a.u.
DFT-D3(BJ) dispersion correction = -0.116836 a.u.
Thermal free energy = 0.585213 a.u.
Gibbs free energy = -3672.568920 a.u.
Number of imaginary frequencies = 0.

**Int1 LA isomer/conformer 15**

| Atomic N. | X | Y | Z |
|---|---|---|---|
| 7 | -0.94862765 | 2.36181953 | -0.44406185 |
| 8 | 1.05571153 | 0.30777195 | -1.26931770 |
| 8 | 0.04824770 | 0.42583140 | 1.64928857 |
| 6 | -2.20398590 | 2.51592250 | -1.22327012 |

| | | | |
|---|---|---|---|
| 6 | 1.03669482 | 0.99582425 | -2.44314419 |
| 6 | 1.51134331 | 0.38988383 | -3.64116824 |
| 6 | 1.46564828 | 1.16563426 | -4.80916913 |
| 6 | 0.98644811 | 2.47868935 | -4.83262717 |
| 6 | 0.54580859 | 3.06485951 | -3.65089719 |
| 6 | 0.57918997 | 2.33910463 | -2.45509597 |
| 6 | 0.21608347 | 3.00806708 | -1.15720768 |
| 1 | 0.96718460 | 3.03636960 | -5.76889652 |
| 6 | 2.05333551 | -1.05282525 | -3.66071523 |
| 6 | 0.58191069 | 1.55107780 | 2.19640158 |
| 6 | 1.63713398 | 1.44258779 | 3.14732708 |
| 6 | 2.13486430 | 2.63570026 | 3.69056919 |
| 6 | 1.64398474 | 3.89471762 | 3.33359644 |
| 6 | 0.60827911 | 3.98499860 | 2.41098396 |
| 6 | 0.06200144 | 2.82500257 | 1.84798018 |
| 6 | -1.13381390 | 2.94717610 | 0.93738113 |
| 1 | 2.07058138 | 4.79394752 | 3.77761859 |
| 6 | 2.21695222 | 0.07514537 | 3.56126067 |
| 6 | 2.54637798 | -1.46190289 | -5.06160399 |
| 6 | 0.93918719 | -2.04696574 | -3.25695178 |
| 6 | 3.25532646 | -1.18053250 | -2.69453028 |
| 6 | 3.31802250 | 0.21861106 | 4.62913861 |
| 6 | 2.85360492 | -0.62389740 | 2.33670707 |
| 6 | 1.10690819 | -0.81854741 | 4.16451565 |
| 1 | -2.45356058 | 3.58488619 | -1.31881799 |
| 1 | -2.06797458 | 2.08611238 | -2.22155561 |
| 1 | -0.03417465 | 4.06825923 | -1.32784259 |
| 1 | 1.05807068 | 2.96655513 | -0.45084429 |
| 1 | 1.81264228 | 0.72942158 | -5.74403977 |
| 1 | 0.19021330 | 4.09776463 | -3.64175856 |
| 1 | -2.00282745 | 2.41914607 | 1.35847756 |
| 1 | -1.40399578 | 4.00981208 | 0.82185381 |
| 1 | 2.94424093 | 2.58710626 | 4.41651366 |
| 1 | 0.20239058 | 4.95867527 | 2.12757475 |
| 1 | 2.93361807 | -2.48954606 | -5.01613301 |
| 1 | 1.73762867 | -1.44836830 | -5.80651458 |
| 1 | 3.36171935 | -0.81609592 | -5.41880499 |
| 1 | 0.56572232 | -1.85187505 | -2.24633891 |
| 1 | 0.09105854 | -1.98665027 | -3.95369434 |
| 1 | 1.32852246 | -3.07554854 | -3.28857440 |
| 1 | 4.07057908 | -0.50800631 | -2.99928172 |
| 1 | 2.97304593 | -0.93994935 | -1.66387091 |
| 1 | 3.64109329 | -2.21094386 | -2.71894150 |
| 1 | 3.68754171 | -0.78154830 | 4.89606235 |
| 1 | 2.94342955 | 0.68928706 | 5.54969767 |
| 1 | 4.17711820 | 0.79987850 | 4.26441316 |
| 1 | 2.11949219 | -0.80998415 | 1.54575116 |
| 1 | 3.66430751 | -0.01093127 | 1.91753486 |
| 1 | 3.28322720 | -1.58973562 | 2.64273792 |
| 1 | 0.67081205 | -0.34564999 | 5.05678148 |
| 1 | 0.30522538 | -1.00938523 | 3.44286600 |
| 1 | 1.53638494 | -1.78413058 | 4.47191631 |
| 1 | -3.01349508 | 1.98925757 | -0.70530558 |

| | | | |
|---|---|---|---|
| 8 | -3.08680286 | -0.04176500 | 0.89042760 |
| 6 | -3.33856921 | -0.17114423 | 2.30699130 |
| 1 | -3.73839521 | 0.80353818 | 2.61366051 |
| 8 | -1.66050291 | -0.82162057 | -0.70984169 |
| 1 | -2.41117357 | -0.38170755 | 2.85314723 |
| 1 | -4.08168934 | -0.94956600 | 2.52247699 |
| 31 | -0.41493717 | 0.41290481 | -0.14254253 |
| 8 | -2.22978664 | -2.17212403 | 1.11242532 |
| 6 | -2.65519057 | -1.20086299 | 0.16548349 |
| 6 | -3.82596289 | -1.79216345 | -0.65255082 |
| 8 | -3.41113762 | -3.03538347 | -1.30294563 |
| 6 | -2.44427049 | -3.82460658 | -0.76295434 |
| 6 | -1.67800408 | -3.34342525 | 0.47606740 |
| 8 | -2.16509259 | -4.87637227 | -1.29965457 |
| 6 | -1.58582660 | -4.43619816 | 1.53332935 |
| 6 | -5.10014645 | -2.06036516 | 0.13446415 |
| 1 | -4.02743582 | -1.10724240 | -1.48808771 |
| 1 | -0.66662150 | -3.10282014 | 0.10336014 |
| 1 | -0.95471560 | -4.09197435 | 2.36239417 |
| 1 | -1.14617785 | -5.33711769 | 1.09149292 |
| 1 | -2.58183288 | -4.68181974 | 1.92524523 |
| 1 | -5.53378744 | -1.12006860 | 0.49829824 |
| 1 | -4.90688124 | -2.72291035 | 0.98860892 |
| 1 | -5.83151907 | -2.54706953 | -0.52326926 |

Electronic energy = -3673.036995 a.u.
DFT-D3(BJ) dispersion correction = -0.117712 a.u.
Thermal free energy = 0.585999 a.u.
Gibbs free energy = -3672.568708 a.u.
Number of imaginary frequencies = 0.

**Int1 LA isomer/conformer 16**

| Atomic N. | X | Y | Z |
|---|---|---|---|
| 7 | 0.25030681 | -0.00992594 | 2.75221943 |
| 8 | -0.60594487 | -1.18978363 | 0.12579459 |
| 8 | -0.16026042 | 1.84662821 | 0.41630701 |
| 6 | 1.37578605 | -0.67902963 | 3.45222115 |
| 6 | -0.90226934 | -2.27226864 | 0.89486563 |
| 6 | -1.13190147 | -3.53530265 | 0.27512806 |
| 6 | -1.40067275 | -4.62408366 | 1.11738576 |
| 6 | -1.47011572 | -4.50964757 | 2.50823841 |
| 6 | -1.30336520 | -3.26038656 | 3.09489723 |
| 6 | -1.04023772 | -2.13765591 | 2.30169674 |
| 6 | -1.03306911 | -0.77342604 | 2.93998035 |
| 1 | -1.67284908 | -5.38813269 | 3.12065314 |
| 6 | -1.12055678 | -3.70269993 | -1.25758704 |
| 6 | -1.09708857 | 2.39089297 | 1.23599950 |
| 6 | -2.13643380 | 3.20203414 | 0.69126504 |
| 6 | -3.06056056 | 3.75268216 | 1.59033450 |
| 6 | -3.00162219 | 3.53657204 | 2.96986323 |
| 6 | -1.98130980 | 2.74958315 | 3.48947189 |
| 6 | -1.02280940 | 2.18513167 | 2.63874324 |
| 6 | 0.12077243 | 1.41401531 | 3.24358207 |

| | | | |
|---|---|---|---|
| 1 | -3.74818759 | 3.98271338 | 3.62667972 |
| 6 | -2.24663813 | 3.47106231 | -0.82181119 |
| 6 | -1.45430027 | -5.14575462 | -1.68097772 |
| 6 | 0.27451927 | -3.36437853 | -1.82898782 |
| 6 | -2.18763738 | -2.77858153 | -1.89229437 |
| 6 | -3.44145549 | 4.38269893 | -1.16093393 |
| 6 | -2.46090588 | 2.14048743 | -1.58149743 |
| 6 | -0.96973260 | 4.18410120 | -1.32508872 |
| 1 | 1.18290024 | -0.69042240 | 4.53721562 |
| 1 | 1.47193736 | -1.70801618 | 3.08967784 |
| 1 | -1.23061052 | -0.86097880 | 4.02149431 |
| 1 | -1.82327390 | -0.14268715 | 2.50617935 |
| 1 | -1.56310106 | -5.60480679 | 0.67440741 |
| 1 | -1.39886098 | -3.13843024 | 4.17623699 |
| 1 | 1.08568181 | 1.89287126 | 3.01141452 |
| 1 | 0.01025371 | 1.38823581 | 4.34030980 |
| 1 | -3.86614587 | 4.37287440 | 1.20198242 |
| 1 | -1.90918021 | 2.57448299 | 4.56525682 |
| 1 | -1.45305757 | -5.20347882 | -2.77877946 |
| 1 | -0.71248212 | -5.86896576 | -1.31193490 |
| 1 | -2.44956593 | -5.45981455 | -1.33494461 |
| 1 | 0.55232733 | -2.32566463 | -1.62414008 |
| 1 | 1.04039481 | -4.02290680 | -1.39356017 |
| 1 | 0.27398642 | -3.51810139 | -2.91948688 |
| 1 | -3.19153727 | -3.03741780 | -1.52542623 |
| 1 | -1.99467341 | -1.72436675 | -1.66536531 |
| 1 | -2.18530921 | -2.90570261 | -2.98566143 |
| 1 | -3.46724898 | 4.54365651 | -2.24804902 |
| 1 | -3.35717197 | 5.36987961 | -0.68409945 |
| 1 | -4.40295979 | 3.93473028 | -0.87056210 |
| 1 | -1.63983188 | 1.43602642 | -1.41194820 |
| 1 | -3.39677326 | 1.65876025 | -1.26273672 |
| 1 | -2.53364049 | 2.33706083 | -2.66208559 |
| 1 | -0.85203365 | 5.15822157 | -0.82859385 |
| 1 | -0.06633814 | 3.59528298 | -1.13808817 |
| 1 | -1.04504254 | 4.36721149 | -2.40756079 |
| 1 | 2.30530932 | -0.14034155 | 3.24091933 |
| 8 | 3.30189019 | -1.55277834 | -0.52192136 |
| 6 | 3.74301771 | -2.13618482 | -1.75319207 |
| 1 | 4.74508661 | -1.78368534 | -2.05193912 |
| 8 | 2.37127817 | 0.10692079 | 0.65735604 |
| 8 | 1.98111126 | 0.06607285 | -1.59225474 |
| 1 | 3.79698582 | -3.21481287 | -1.56685748 |
| 1 | 3.02498739 | -1.95291521 | -2.56613998 |
| 31 | 0.52304782 | 0.15163835 | 0.73174829 |
| 8 | 3.81296435 | 2.08904772 | -1.03633901 |
| 6 | 2.92388461 | 2.28031082 | -2.05321032 |
| 6 | 2.35787314 | 0.98490225 | -2.64715406 |
| 6 | 2.95488061 | -0.16888182 | -0.54399848 |
| 6 | 4.20878950 | 0.71032398 | -0.77335309 |
| 8 | 2.64344096 | 3.39725123 | -2.42561515 |
| 6 | 5.14628285 | 0.73165042 | 0.41768294 |
| 6 | 1.14160633 | 1.22247755 | -3.52031131 |

| | | | |
|---|---|---|---|
| 1 | 3.16238187 | 0.52550526 | -3.25547772 |
| 1 | 4.74248272 | 0.34167898 | -1.66438381 |
| 1 | 6.02600366 | 1.34599922 | 0.18872634 |
| 1 | 5.47451512 | -0.28889753 | 0.65182664 |
| 1 | 4.63180985 | 1.14814832 | 1.29108240 |
| 1 | 1.40913267 | 1.88185068 | -4.35434556 |
| 1 | 0.34382091 | 1.70319683 | -2.94305026 |
| 1 | 0.77731317 | 0.26644316 | -3.91709903 |

Electronic energy = -3673.036122 a.u.
DFT-D3(BJ) dispersion correction = -0.118912 a.u.
Thermal free energy = 0.586437 a.u.
Gibbs free energy = -3672.568597 a.u.
Number of imaginary frequencies = 0.

**Int1 LA isomer/conformer 17**

| Atomic N. | X | Y | Z |
|---|---|---|---|
| 7 | 1.10357821 | 2.10047687 | 1.14244698 |
| 8 | -1.05076865 | 0.29191114 | 0.05514280 |
| 8 | 1.58706738 | 0.66803331 | -1.45443037 |
| 6 | 1.55099877 | 1.96237342 | 2.55020054 |
| 6 | -1.82658445 | 0.89168305 | 0.99667996 |
| 6 | -3.10880654 | 0.35377673 | 1.31075169 |
| 6 | -3.82645903 | 0.97534830 | 2.34395426 |
| 6 | -3.35546618 | 2.09907306 | 3.02853156 |
| 6 | -2.14771159 | 2.67092307 | 2.64266398 |
| 6 | -1.39163878 | 2.09318684 | 1.61678063 |
| 6 | -0.20580131 | 2.83945947 | 1.06307922 |
| 6 | -3.71625709 | -0.82165970 | 0.51783848 |
| 6 | 1.59585414 | 1.92316909 | -1.96561488 |
| 6 | 1.40098941 | 2.12869004 | -3.36256698 |
| 6 | 1.43413068 | 3.45157508 | -3.82574418 |
| 6 | 1.64744369 | 4.54730293 | -2.98355334 |
| 6 | 1.85227649 | 4.33224547 | -1.62572929 |
| 6 | 1.84148916 | 3.02983706 | -1.11151719 |
| 6 | 2.17604969 | 2.80717675 | 0.33954971 |
| 6 | 1.16737299 | 0.94973194 | -4.32732666 |
| 6 | -5.15582435 | -1.13489840 | 0.96834203 |
| 6 | -2.88242454 | -2.10717634 | 0.70876980 |
| 6 | -3.77661187 | -0.45395473 | -0.98489400 |
| 6 | 1.02134410 | 1.42237016 | -5.78614257 |
| 6 | -0.13571660 | 0.20441976 | -3.95324694 |
| 6 | 2.36881654 | -0.02427056 | -4.27620911 |
| 1 | 1.72693442 | 2.96052628 | 2.98265993 |
| 1 | 0.78441994 | 1.43637131 | 3.12752596 |
| 1 | -0.08884247 | 3.80466834 | 1.58368429 |
| 1 | -0.36118469 | 3.05179412 | -0.00529745 |
| 1 | -4.79816056 | 0.57340114 | 2.62474403 |
| 1 | -1.79348581 | 3.59040958 | 3.11435879 |
| 1 | 3.07544828 | 2.17869413 | 0.43993372 |
| 1 | 2.38092661 | 3.77455311 | 0.82737168 |
| 1 | 1.28230019 | 3.64197016 | -4.88658030 |
| 1 | 2.03756075 | 5.17241590 | -0.95261671 |

| | | | |
|---|---|---|---|
| 1 | -5.54964973 | -1.95653573 | 0.35333097 |
| 1 | -5.20224745 | -1.45946253 | 2.01819329 |
| 1 | -5.82726520 | -0.27376263 | 0.83974071 |
| 1 | -1.84182608 | -1.97080572 | 0.39917850 |
| 1 | -2.89544395 | -2.41992738 | 1.76349337 |
| 1 | -3.31932001 | -2.92282698 | 0.11185052 |
| 1 | -4.39597681 | 0.44143910 | -1.14002142 |
| 1 | -2.77897394 | -0.26102959 | -1.39422288 |
| 1 | -4.23079060 | -1.28215456 | -1.54934650 |
| 1 | 0.86991474 | 0.54514850 | -6.43091310 |
| 1 | 1.92086743 | 1.94391925 | -6.14404127 |
| 1 | 0.15512187 | 2.08586218 | -5.92352690 |
| 1 | -0.08853325 | -0.21448749 | -2.94261217 |
| 1 | -1.00064118 | 0.88131254 | -4.00853324 |
| 1 | -0.30571174 | -0.62139742 | -4.66052480 |
| 1 | 3.29349892 | 0.48631347 | -4.58265772 |
| 1 | 2.51459438 | -0.43545539 | -3.27169885 |
| 1 | 2.19841184 | -0.85892387 | -4.97293862 |
| 1 | 2.47788972 | 1.37853488 | 2.57843960 |
| 8 | 1.88375599 | -0.97814235 | 0.93098402 |
| 8 | 0.54666060 | -1.15313541 | 2.72698920 |
| 6 | -0.27507707 | -1.90963362 | 3.62858519 |
| 1 | -1.00051006 | -1.19912823 | 4.04155657 |
| 1 | -0.81374390 | -2.70689792 | 3.09921501 |
| 1 | 0.31476190 | -2.33698639 | 4.45670021 |
| 31 | 0.80122262 | 0.31507872 | 0.18591421 |
| 1 | -3.94738280 | 2.53934881 | 3.83087010 |
| 1 | 1.65735930 | 5.55747017 | -3.39260629 |
| 8 | 0.55274957 | -2.84655150 | 1.13650851 |
| 6 | 1.37662824 | -1.88933557 | 1.81242172 |
| 6 | 2.52602125 | -2.61333351 | 2.55394826 |
| 8 | 3.26630318 | -3.43923601 | 1.61051343 |
| 6 | 2.52911369 | -4.29806644 | 0.84849601 |
| 6 | 1.01622526 | -4.21322495 | 1.07877847 |
| 8 | 3.07394624 | -5.06852411 | 0.09067575 |
| 6 | 0.22721743 | -4.92333652 | -0.00567959 |
| 6 | 3.52680005 | -1.67734721 | 3.20164017 |
| 1 | 2.08293101 | -3.27931789 | 3.31388179 |
| 1 | 0.82250751 | -4.70538290 | 2.05527099 |
| 1 | -0.84583826 | -4.86056429 | 0.21459978 |
| 1 | 0.52663369 | -5.97674349 | -0.05432014 |
| 1 | 0.42232855 | -4.46132075 | -0.98090607 |
| 1 | 3.02458477 | -1.04632282 | 3.94614442 |
| 1 | 3.98900966 | -1.04042879 | 2.43857699 |
| 1 | 4.30861567 | -2.26030557 | 3.70451167 |

Electronic energy = -3673.035809 a.u.
DFT-D3(BJ) dispersion correction = -0.117826 a.u.
Thermal free energy = 0.585187 a.u.
Gibbs free energy = -3672.568449 a.u.
Number of imaginary frequencies = 0.

**<u>Int1_LA isomer/conformer 18</u>**

| Atomic N. | X | Y | Z |
|---|---|---|---|
| 8 | 1.25719228 | -2.90091021 | 1.15025734 |
| 6 | -0.80456837 | -4.14585493 | 0.86399807 |
| 8 | -0.42018163 | -4.54507375 | -0.48585788 |
| 6 | 0.82548071 | -4.26314450 | -0.94563405 |
| 6 | 1.73494077 | -3.31726032 | -0.15563276 |
| 8 | 1.18499250 | -4.70977289 | -2.01521529 |
| 6 | 3.11874800 | -3.92557855 | 0.04465244 |
| 6 | -0.47604029 | -5.27509707 | 1.83111195 |
| 1 | -1.89075003 | -4.00196525 | 0.78661772 |
| 1 | 1.81710547 | -2.42423949 | -0.80052873 |
| 1 | 3.78362608 | -3.18773804 | 0.51209297 |
| 1 | 3.53317086 | -4.21725407 | -0.92671658 |
| 1 | 3.06662363 | -4.80996944 | 0.69346161 |
| 1 | -0.91536786 | -5.08631785 | 2.81835151 |
| 1 | 0.60898478 | -5.38410580 | 1.95648243 |
| 1 | -0.88463959 | -6.21487122 | 1.43820150 |
| 7 | 1.26712896 | 0.78273411 | 1.69091484 |
| 8 | -1.23395513 | 1.12269758 | 0.06304530 |
| 8 | 1.29499924 | -0.17668587 | -1.15746516 |
| 6 | 1.19534306 | 0.23373528 | 3.07043171 |
| 6 | -1.62189868 | 1.88183843 | 1.12120707 |
| 6 | -3.00015457 | 2.18027218 | 1.32248052 |
| 6 | -3.32645345 | 2.96230450 | 2.44059193 |
| 6 | -2.36746258 | 3.45268081 | 3.33197409 |
| 6 | -1.02253479 | 3.18129834 | 3.10372414 |
| 6 | -0.64110269 | 2.41247672 | 1.99858557 |
| 6 | 0.81585254 | 2.22666075 | 1.67399251 |
| 6 | -4.08751326 | 1.68138434 | 0.35033734 |
| 6 | 2.24856468 | 0.78150043 | -1.30723804 |
| 6 | 2.56432150 | 1.27788180 | -2.60418543 |
| 6 | 3.57069456 | 2.25145773 | -2.68525077 |
| 6 | 4.25106817 | 2.73722938 | -1.56484487 |
| 6 | 3.93594310 | 2.23766376 | -0.30674351 |
| 6 | 2.94838372 | 1.25443390 | -0.16905499 |
| 6 | 2.69208340 | 0.65513684 | 1.18816628 |
| 6 | 1.83710909 | 0.77505863 | -3.86701454 |
| 6 | -5.48837983 | 2.18136350 | 0.75092605 |
| 6 | -4.13530741 | 0.13536958 | 0.34291069 |
| 6 | -3.80151996 | 2.20776638 | -1.07656921 |
| 6 | 2.37355672 | 1.45096070 | -5.14334262 |
| 6 | 0.32680820 | 1.09837049 | -3.77743446 |
| 6 | 2.04476048 | -0.74907697 | -4.03274825 |
| 1 | 1.88749829 | 0.79416411 | 3.71925437 |
| 1 | 0.17578244 | 0.34473949 | 3.45358946 |
| 1 | 1.44328193 | 2.78473311 | 2.38813806 |
| 1 | 1.03858455 | 2.60089779 | 0.66424484 |
| 1 | -4.37161139 | 3.20068354 | 2.62923246 |
| 1 | -0.25437486 | 3.58155258 | 3.76933677 |
| 1 | 2.90334836 | -0.42585689 | 1.19576418 |
| 1 | 3.34995108 | 1.13327247 | 1.93193752 |
| 1 | 3.83417872 | 2.65568352 | -3.66069297 |
| 1 | 4.46260536 | 2.59598983 | 0.58049146 |

| | | | |
|---|---|---|---|
| 1 | -6.22098983 | 1.81230649 | 0.01935000 |
| 1 | -5.79535783 | 1.81075170 | 1.73984191 |
| 1 | -5.54997693 | 3.27917651 | 0.75559689 |
| 1 | -3.18358896 | -0.30299059 | 0.02362423 |
| 1 | -4.38199220 | -0.24775735 | 1.34454116 |
| 1 | -4.91920857 | -0.20822660 | -0.34884106 |
| 1 | -3.80460179 | 3.30741705 | -1.09052430 |
| 1 | -2.83374825 | 1.85736218 | -1.45098940 |
| 1 | -4.58709047 | 1.85933371 | -1.76391471 |
| 1 | 1.82911739 | 1.05322838 | -6.01122987 |
| 1 | 3.44222379 | 1.24646029 | -5.30258762 |
| 1 | 2.22395435 | 2.54037676 | -5.13129600 |
| 1 | -0.14723453 | 0.60406326 | -2.92305603 |
| 1 | 0.16548795 | 2.18219930 | -3.68514837 |
| 1 | -0.17862183 | 0.75723783 | -4.69328195 |
| 1 | 3.11438269 | -0.98757801 | -4.12440797 |
| 1 | 1.63351319 | -1.31182466 | -3.18838552 |
| 1 | 1.54331067 | -1.09406577 | -4.94911324 |
| 1 | 1.46018687 | -0.82814895 | 3.05942978 |
| 8 | -0.67923113 | -1.80270412 | 0.42127514 |
| 8 | -0.32850323 | -2.50627439 | 2.62305382 |
| 6 | -1.68025938 | -2.23913356 | 3.02282231 |
| 1 | -1.61707739 | -1.83351413 | 4.03929527 |
| 1 | -2.16119449 | -1.50514821 | 2.36088835 |
| 1 | -2.28904302 | -3.15834488 | 3.04972921 |
| 31 | 0.10952213 | -0.14100017 | 0.26815532 |
| 6 | -0.15989399 | -2.78859499 | 1.23362679 |
| 1 | -2.67428089 | 4.05273372 | 4.18855039 |
| 1 | 5.02129880 | 3.49943974 | -1.68133907 |

Electronic energy = -3673.035326 a.u.
DFT-D3(BJ) dispersion correction = -0.119173 a.u.
Thermal free energy = 0.586342 a.u.
Gibbs free energy = -3672.568158 a.u.
Number of imaginary frequencies = 0.

**Int1 LA isomer/conformer 19**

| Atomic N. | X | Y | Z |
|---|---|---|---|
| 7 | 0.47192116 | -1.46908199 | -1.81928965 |
| 8 | 0.22524021 | -0.34107967 | 0.97159868 |
| 8 | 1.41885902 | 1.32841949 | -1.26561782 |
| 6 | -0.65737661 | -2.17154419 | -2.47452518 |
| 6 | -0.00153097 | -1.63502380 | 1.32218822 |
| 6 | -0.47207884 | -1.95323089 | 2.62887136 |
| 6 | -0.77633796 | -3.29889446 | 2.88604981 |
| 6 | -0.58584501 | -4.31605498 | 1.94665368 |
| 6 | -0.01135748 | -4.00517366 | 0.71869984 |
| 6 | 0.30583740 | -2.67795897 | 0.40915492 |
| 6 | 1.13346533 | -2.37491019 | -0.81081443 |
| 1 | -0.84824390 | -5.34468291 | 2.19307606 |
| 6 | -0.56213662 | -0.89226977 | 3.74481148 |
| 6 | 2.63093037 | 0.81839389 | -1.58461956 |
| 6 | 3.82106882 | 1.49599555 | -1.18802153 |

| | | | |
|---|---|---|---|
| 6 | 5.04216268 | 0.91849019 | -1.56276911 |
| 6 | 5.12903882 | -0.27032762 | -2.29422220 |
| 6 | 3.96173472 | -0.91426906 | -2.68682187 |
| 6 | 2.71438346 | -0.37375696 | -2.35005903 |
| 6 | 1.46442884 | -1.02462420 | -2.87817849 |
| 1 | 6.10387994 | -0.68195361 | -2.55489106 |
| 6 | 3.77152067 | 2.80885524 | -0.38234541 |
| 6 | -1.02476029 | -1.50054512 | 5.08193127 |
| 6 | -1.55439113 | 0.23168412 | 3.38039275 |
| 6 | 0.84377103 | -0.28450431 | 3.97140818 |
| 6 | 5.18051772 | 3.36533174 | -0.10358581 |
| 6 | 3.09300442 | 2.56785485 | 0.98696190 |
| 6 | 2.99729522 | 3.88927999 | -1.17475971 |
| 1 | -0.28141917 | -3.06328034 | -3.00119117 |
| 1 | -1.38948338 | -2.47239505 | -1.71911010 |
| 1 | 1.41198934 | -3.30993891 | -1.32460657 |
| 1 | 2.06222998 | -1.86362194 | -0.51758520 |
| 1 | -1.17033187 | -3.57087195 | 3.86346405 |
| 1 | 0.22473886 | -4.79367206 | 0.00042191 |
| 1 | 0.90543199 | -0.33153350 | -3.52747825 |
| 1 | 1.73273385 | -1.91047554 | -3.47675478 |
| 1 | 5.96853922 | 1.40895046 | -1.26956924 |
| 1 | 4.00641452 | -1.83569528 | -3.27182294 |
| 1 | -1.04703761 | -0.70878311 | 5.84404504 |
| 1 | -2.03766195 | -1.92412315 | 5.01691283 |
| 1 | -0.34174386 | -2.28400334 | 5.43995613 |
| 1 | -1.24427400 | 0.74730399 | 2.46650257 |
| 1 | -2.56685421 | -0.17169138 | 3.23423386 |
| 1 | -1.59893822 | 0.96738377 | 4.19772293 |
| 1 | 1.55938242 | -1.06273539 | 4.27341778 |
| 1 | 1.22153600 | 0.20432828 | 3.06587873 |
| 1 | 0.79945340 | 0.46509493 | 4.77591135 |
| 1 | 5.08759666 | 4.30354261 | 0.46149769 |
| 1 | 5.72712888 | 3.59097705 | -1.03067919 |
| 1 | 5.78772038 | 2.67568573 | 0.50053302 |
| 1 | 2.06172079 | 2.21753817 | 0.87311702 |
| 1 | 3.65021993 | 1.82081342 | 1.57065982 |
| 1 | 3.07786777 | 3.50532124 | 1.56322728 |
| 1 | 3.49715345 | 4.09908065 | -2.13172886 |
| 1 | 1.96721539 | 3.57894454 | -1.38031950 |
| 1 | 2.97151252 | 4.82514422 | -0.59618360 |
| 1 | -1.13374045 | -1.50129106 | -3.19867552 |
| 8 | -3.54261843 | 1.82573675 | -0.68414611 |
| 6 | -3.03079924 | 2.35566899 | 0.54890716 |
| 1 | -3.77342020 | 3.09033027 | 0.87920262 |
| 8 | -1.41079186 | 1.24538567 | -1.41475408 |
| 1 | -2.06369894 | 2.85206974 | 0.39071501 |
| 1 | -2.92828816 | 1.57202615 | 1.31245349 |
| 31 | -0.00397267 | 0.23820075 | -0.78145477 |
| 8 | -2.74852400 | -0.40831790 | -0.66492098 |
| 6 | -2.73054988 | 0.87414603 | -1.33103828 |
| 6 | -3.32808821 | 0.70034420 | -2.74242386 |
| 8 | -4.68480549 | 0.16405067 | -2.68133204 |

| | | | |
|---|---|---|---|
| 6 | -5.04859754 | -0.68065943 | -1.68467914 |
| 6 | -4.03429441 | -1.07109388 | -0.59917368 |
| 8 | -6.16866983 | -1.14734769 | -1.67466182 |
| 6 | -4.65844712 | -0.96310612 | 0.79188997 |
| 6 | -3.39348260 | 1.97983665 | -3.55328309 |
| 1 | -2.70457099 | -0.04739615 | -3.25734478 |
| 1 | -3.81589996 | -2.13442134 | -0.79918207 |
| 1 | -3.95103047 | -1.34629490 | 1.53769749 |
| 1 | -5.57739367 | -1.55994138 | 0.82737319 |
| 1 | -4.90627999 | 0.07880656 | 1.02760789 |
| 1 | -2.38683756 | 2.40604735 | -3.64813167 |
| 1 | -4.04479589 | 2.70840355 | -3.05736715 |
| 1 | -3.78824453 | 1.76710970 | -4.55502768 |

Electronic energy = -3673.033622 a.u.
DFT-D3(BJ) dispersion correction = -0.118684 a.u.
Thermal free energy = 0.584156 a.u.
Gibbs free energy = -3672.568149 a.u.
Number of imaginary frequencies = 0.

**Int1 LA isomer/conformer 20**

| Atomic N. | X | Y | Z |
|---|---|---|---|
| 7 | 1.44644896 | -0.68511108 | -1.72051331 |
| 8 | 0.15182866 | 0.93059290 | 0.48145324 |
| 8 | -1.54673148 | -0.57627846 | -1.57521052 |
| 6 | 2.59094216 | -1.57725939 | -1.41349444 |
| 6 | 1.38653716 | 1.49081240 | 0.59612833 |
| 6 | 1.69655100 | 2.30606376 | 1.72234775 |
| 6 | 3.01617600 | 2.77160962 | 1.82377078 |
| 6 | 3.99295795 | 2.51273878 | 0.85781754 |
| 6 | 3.64287592 | 1.81018736 | -0.28990778 |
| 6 | 2.34109032 | 1.31788979 | -0.43975592 |
| 6 | 1.90776828 | 0.75271305 | -1.76480898 |
| 1 | 5.00709957 | 2.88901187 | 0.99115157 |
| 6 | 0.62360604 | 2.72394617 | 2.74841102 |
| 6 | -1.43838726 | -0.01219946 | -2.79952432 |
| 6 | -2.52311401 | 0.73902115 | -3.33985669 |
| 6 | -2.34919555 | 1.28522777 | -4.61899708 |
| 6 | -1.17618255 | 1.12189387 | -5.36296869 |
| 6 | -0.13090248 | 0.37703613 | -4.83011609 |
| 6 | -0.25678868 | -0.20215766 | -3.56115065 |
| 6 | 0.84064976 | -1.09582229 | -3.04977876 |
| 1 | -1.08779489 | 1.57376004 | -6.35076331 |
| 6 | -3.83249220 | 0.94388533 | -2.55343590 |
| 6 | 1.19289946 | 3.68104812 | 3.81282353 |
| 6 | 0.04773034 | 1.50054230 | 3.49261853 |
| 6 | -0.51245892 | 3.47449149 | 2.01131239 |
| 6 | -4.86337972 | 1.75869006 | -3.35716191 |
| 6 | -3.55137900 | 1.72000943 | -1.24494103 |
| 6 | -4.47791402 | -0.42490072 | -2.22973077 |
| 1 | 3.34227939 | -1.49482946 | -2.21534737 |
| 1 | 3.03772392 | -1.28420096 | -0.45965000 |
| 1 | 2.73035370 | 0.82119591 | -2.49586236 |

```
   1     1.05778379   1.32589360  -2.16365893
   1     3.29661711   3.36634062   2.69090009
   1     4.36784961   1.66040508  -1.09332436
   1     0.47341520  -2.12372684  -2.89739180
   1     1.65761011  -1.13507682  -3.78867763
   1    -3.15693889   1.86906994  -5.05648093
   1     0.78899890   0.22579150  -5.39946819
   1     0.38366280   3.96960941   4.49844918
   1     1.97972491   3.20584662   4.41603133
   1     1.59692251   4.60334905   3.37058494
   1    -0.41218717   0.78764945   2.80138108
   1     0.82958045   0.98000365   4.06176734
   1    -0.72232405   1.83334236   4.20530909
   1    -0.12542725   4.37296845   1.50856056
   1    -0.99822603   2.83781668   1.26291976
   1    -1.27434471   3.79525007   2.73773195
   1    -5.77865138   1.86505872  -2.75786967
   1    -5.14058989   1.26216783  -4.29840600
   1    -4.50261975   2.77114564  -3.58966378
   1    -2.86114502   1.17746363  -0.59041108
   1    -3.11809950   2.70670998  -1.46367506
   1    -4.49306986   1.87772375  -0.69774948
   1    -4.71946476  -0.96715470  -3.15570853
   1    -3.81561346  -1.05021301  -1.62152704
   1    -5.41633790  -0.26942219  -1.67615308
   1     2.24256519  -2.61286956  -1.34184761
   8     0.02767911  -4.31548799   1.15256952
   6     0.23524177  -4.68505395  -0.21416499
   1     1.26753113  -4.46917402  -0.53658925
   8    -0.62115367  -2.18971764   0.53201622
   8     1.45968593  -2.45171601   1.38513413
   1     0.07287566  -5.76803863  -0.25560333
   1    -0.48057499  -4.17936116  -0.87721911
  31    -0.12097469  -0.68153634  -0.39099507
   8     0.23943863  -1.85385789   3.70452254
   6     1.60075840  -1.90165915   3.71758245
   6     2.20674364  -2.74185849   2.58950621
   6     0.09711384  -2.91773093   1.43624271
   6    -0.44240445  -2.86462117   2.90426588
   8     2.23456699  -1.27352386   4.53798408
   6    -1.92658390  -2.57586244   2.98818891
   6     3.67007159  -2.41751388   2.35788270
   1     2.09251500  -3.81448519   2.83680241
   1    -0.22176246  -3.84493896   3.35270174
   1    -2.24973302  -2.59951957   4.03651956
   1    -2.48312392  -3.33621902   2.42625696
   1    -2.15283721  -1.59244574   2.56017898
   1     4.23498476  -2.60151598   3.27904690
   1     3.79576151  -1.35961787   2.09357263
   1     4.07569157  -3.04929482   1.55711625
Electronic energy = -3673.034914 a.u.
DFT-D3(BJ) dispersion correction = -0.118505 a.u.
Thermal free energy = 0.586572 a.u.
```

Gibbs free energy = -3672.566848 a.u.
Number of imaginary frequencies = 0.

**Int1 LA isomer/conformer 21**

| Atomic N. | X | Y | Z |
|---|---|---|---|
| 7 | 0.57834407 | 1.73404945 | 1.25602138 |
| 8 | -1.47531186 | -0.25828814 | 0.35863572 |
| 8 | 0.45135503 | 1.05796005 | -1.66986269 |
| 6 | 1.42654144 | 1.44638387 | 2.44218441 |
| 6 | -1.92936720 | -0.11288967 | 1.63278415 |
| 6 | -2.77375301 | -1.10195116 | 2.21196355 |
| 6 | -3.20510784 | -0.87922754 | 3.52818831 |
| 6 | -2.84556472 | 0.25257250 | 4.26593319 |
| 6 | -2.03726205 | 1.22045584 | 3.67943171 |
| 6 | -1.58771863 | 1.05276964 | 2.36513218 |
| 6 | -0.81291295 | 2.14775804 | 1.68574912 |
| 1 | -3.20348252 | 0.37437110 | 5.28818328 |
| 6 | -3.19894629 | -2.36183486 | 1.43299924 |
| 6 | 0.12237626 | 2.36897660 | -1.80046737 |
| 6 | -0.52435688 | 2.82921293 | -2.98376644 |
| 6 | -0.82032668 | 4.19784123 | -3.05960999 |
| 6 | -0.51249379 | 5.09952071 | -2.03671553 |
| 6 | 0.12457874 | 4.63748180 | -0.89099371 |
| 6 | 0.45638110 | 3.28264367 | -0.76857144 |
| 6 | 1.23753357 | 2.82365148 | 0.43440025 |
| 1 | -0.77072509 | 6.15319924 | -2.14068638 |
| 6 | -0.89132356 | 1.86920753 | -4.13270349 |
| 6 | -4.14594215 | -3.25372559 | 2.25803192 |
| 6 | -1.95766597 | -3.21359843 | 1.07665218 |
| 6 | -3.95392597 | -1.95776539 | 0.14408099 |
| 6 | -1.55533434 | 2.60887765 | -5.30956028 |
| 6 | -1.89491995 | 0.80226672 | -3.63515142 |
| 6 | 0.38218056 | 1.18324206 | -4.68198459 |
| 1 | 1.50610692 | 2.35527696 | 3.06011871 |
| 1 | 0.97475418 | 0.64228102 | 3.03115225 |
| 1 | -0.71346352 | 3.02024395 | 2.35204911 |
| 1 | -1.32531679 | 2.47707732 | 0.77015928 |
| 1 | -3.84443097 | -1.62021624 | 4.00448672 |
| 1 | -1.76346699 | 2.12383342 | 4.22919692 |
| 1 | 2.21473187 | 2.40934251 | 0.14053481 |
| 1 | 1.42020718 | 3.68000698 | 1.10380662 |
| 1 | -1.31873035 | 4.58054658 | -3.94821432 |
| 1 | 0.38286188 | 5.32680929 | -0.08405707 |
| 1 | -4.42991438 | -4.12633456 | 1.65300401 |
| 1 | -3.66805618 | -3.63028139 | 3.17399168 |
| 1 | -5.07138561 | -2.72910631 | 2.53679863 |
| 1 | -1.25161228 | -2.66530752 | 0.44426239 |
| 1 | -1.43101245 | -3.53265417 | 1.98755197 |
| 1 | -2.27342791 | -4.11868384 | 0.53585297 |
| 1 | -4.86103358 | -1.38609331 | 0.38933680 |
| 1 | -3.32779454 | -1.34981402 | -0.51784978 |
| 1 | -4.26269790 | -2.86189120 | -0.40223110 |

| | | | |
|---|---|---|---|
| 1 | -1.78624842 | 1.88277279 | -6.10175721 |
| 1 | -0.89520233 | 3.37351772 | -5.74408958 |
| 1 | -2.50052425 | 3.08882086 | -5.01760218 |
| 1 | -1.47656422 | 0.19355809 | -2.82675218 |
| 1 | -2.81641309 | 1.27714298 | -3.26889627 |
| 1 | -2.16627170 | 0.13264551 | -4.46519917 |
| 1 | 1.08805755 | 1.93121953 | -5.07194630 |
| 1 | 0.88697597 | 0.59538257 | -3.90784864 |
| 1 | 0.11267599 | 0.50994839 | -5.50970690 |
| 1 | 2.41899139 | 1.12920172 | 2.10656579 |
| 8 | 3.35153844 | -0.30830748 | 0.00206400 |
| 6 | 3.66525566 | -0.10974275 | -1.38677417 |
| 1 | 4.11861625 | 0.88656428 | -1.45286844 |
| 8 | 1.27209394 | -1.37074732 | -0.16018762 |
| 1 | 2.76215624 | -0.13217787 | -2.01403494 |
| 1 | 4.39734708 | -0.84924379 | -1.75020155 |
| 31 | 0.27979474 | 0.17864937 | -0.04344482 |
| 8 | 2.50150068 | -1.46039523 | 1.73912801 |
| 6 | 2.54116105 | -1.47438314 | 0.31866581 |
| 6 | 3.23582030 | -2.78905694 | -0.19076380 |
| 8 | 3.12073793 | -3.87339441 | 0.78010174 |
| 6 | 3.50273642 | -3.60652718 | 2.06247968 |
| 6 | 3.62586556 | -2.11309980 | 2.36778292 |
| 8 | 3.66382024 | -4.50522548 | 2.85800253 |
| 6 | 3.58410731 | -1.82405867 | 3.85617487 |
| 6 | 2.67144456 | -3.34244479 | -1.48579758 |
| 1 | 4.31031042 | -2.56842015 | -0.29871891 |
| 1 | 4.57017174 | -1.73035129 | 1.93473951 |
| 1 | 3.71529447 | -0.74946836 | 4.03587152 |
| 1 | 4.38619853 | -2.37394688 | 4.36205273 |
| 1 | 2.62651693 | -2.14996662 | 4.28102604 |
| 1 | 2.76403446 | -2.61395475 | -2.29969168 |
| 1 | 1.60948606 | -3.57986622 | -1.35804584 |
| 1 | 3.21934622 | -4.25284145 | -1.76057625 |

Electronic energy = -3673.034953 a.u.
DFT-D3(BJ) dispersion correction = -0.117049 a.u.
Thermal free energy = 0.586398 a.u.
Gibbs free energy = -3672.565604 a.u.
Number of imaginary frequencies = 0.

**Int1 LA isomer/conformer 22**

| Atomic N. | X | Y | Z |
|---|---|---|---|
| 7 | -0.67430443 | -1.88414605 | 1.51757038 |
| 8 | -0.81230874 | 0.66036593 | -0.09680541 |
| 8 | 1.57643334 | 0.10220128 | 1.72571452 |
| 6 | -0.89990381 | -3.20602786 | 0.88170875 |
| 6 | -2.10702654 | 0.29235506 | -0.29983473 |
| 6 | -2.88906413 | 0.96054427 | -1.28458908 |
| 6 | -4.18953159 | 0.47930821 | -1.49792820 |
| 6 | -4.73919423 | -0.57751962 | -0.76689929 |
| 6 | -3.99746894 | -1.15142064 | 0.26019645 |
| 6 | -2.69476576 | -0.70898098 | 0.51709882 |

| | | | |
|---|---|---|---|
| 6 | -1.98365984 | -1.17644507 | 1.76037048 |
| 1 | -5.75062699 | -0.92262872 | -0.98073712 |
| 6 | -2.36220709 | 2.19663845 | -2.04201974 |
| 6 | 1.14846540 | 0.22101784 | 3.00654909 |
| 6 | 1.51427745 | 1.35941102 | 3.78202215 |
| 6 | 1.04829554 | 1.41057545 | 5.10336826 |
| 6 | 0.25710702 | 0.40653396 | 5.66997441 |
| 6 | -0.08084960 | -0.70537912 | 4.90751868 |
| 6 | 0.36762770 | -0.81307885 | 3.58537854 |
| 6 | 0.08821316 | -2.07564808 | 2.81346032 |
| 1 | -0.08585917 | 0.49706632 | 6.70047105 |
| 6 | 2.38631661 | 2.48823909 | 3.19818263 |
| 6 | -3.43352591 | 2.80027642 | -2.96989784 |
| 6 | -1.14361935 | 1.83561460 | -2.91906934 |
| 6 | -1.96915239 | 3.29212491 | -1.02094364 |
| 6 | 2.67393264 | 3.58810283 | 4.23755788 |
| 6 | 1.66234583 | 3.15941901 | 2.00711468 |
| 6 | 3.75069776 | 1.92116403 | 2.73820219 |
| 1 | -1.46466235 | -3.85437998 | 1.57065997 |
| 1 | -1.46156692 | -3.07173502 | -0.04739078 |
| 1 | -2.63542683 | -1.85161360 | 2.33937087 |
| 1 | -1.73956356 | -0.31541019 | 2.40034675 |
| 1 | -4.80442811 | 0.94829824 | -2.26378939 |
| 1 | -4.43267218 | -1.92944748 | 0.89154323 |
| 1 | 1.02826274 | -2.57331179 | 2.52594028 |
| 1 | -0.48778014 | -2.77487757 | 3.44157906 |
| 1 | 1.30509060 | 2.27022547 | 5.71956403 |
| 1 | -0.68489555 | -1.50858198 | 5.33550422 |
| 1 | -3.01622852 | 3.68972410 | -3.46293978 |
| 1 | -3.73991441 | 2.10020911 | -3.76065294 |
| 1 | -4.32979423 | 3.11770133 | -2.41764474 |
| 1 | -0.33549178 | 1.39182072 | -2.32954319 |
| 1 | -1.42869149 | 1.11995491 | -3.70254064 |
| 1 | -0.76318643 | 2.74308157 | -3.41274648 |
| 1 | -2.83704480 | 3.58483073 | -0.41217021 |
| 1 | -1.17141345 | 2.95363030 | -0.35053181 |
| 1 | -1.61593290 | 4.18628473 | -1.55647260 |
| 1 | 3.30656154 | 4.35881263 | 3.77495150 |
| 1 | 3.21385218 | 3.19926267 | 5.11304053 |
| 1 | 1.75482862 | 4.08161935 | 4.58519825 |
| 1 | 1.46860078 | 2.44904235 | 1.19655670 |
| 1 | 0.70211526 | 3.58877337 | 2.32748019 |
| 1 | 2.28264804 | 3.97670606 | 1.60906003 |
| 1 | 4.29301932 | 1.48169758 | 3.58815945 |
| 1 | 3.62909167 | 1.15221550 | 1.96772179 |
| 1 | 4.36965638 | 2.73331942 | 2.32754927 |
| 1 | 0.06622416 | -3.66737979 | 0.64797761 |
| 8 | 1.37014669 | -0.71588315 | -2.89253135 |
| 6 | 2.53117623 | 0.12835310 | -2.94136895 |
| 1 | 2.17380007 | 1.11795911 | -3.25237475 |
| 8 | 1.65785491 | -1.45256601 | -0.68554639 |
| 1 | 3.25810946 | -0.23489449 | -3.68289855 |
| 1 | 3.00775344 | 0.21203804 | -1.95437375 |

| | | | |
|---|---|---|---|
| 31 | 0.45927458 | -0.58564498 | 0.41523639 |
| 8 | 0.05717795 | -2.33004375 | -2.03631907 |
| 6 | 1.38320937 | -1.80965279 | -1.97847676 |
| 6 | 2.32974444 | -2.97525277 | -2.36960351 |
| 8 | 2.08898946 | -3.34869733 | -3.75636687 |
| 6 | 0.81813177 | -3.31198912 | -4.24171539 |
| 6 | -0.33387702 | -2.91958777 | -3.30341945 |
| 8 | 0.60804926 | -3.63777938 | -5.39033364 |
| 6 | -1.35644913 | -2.04522637 | -4.02337721 |
| 6 | 3.82544075 | -2.76225594 | -2.21431803 |
| 1 | 2.01958955 | -3.81842406 | -1.73026183 |
| 1 | -0.80988115 | -3.88076529 | -3.03632706 |
| 1 | -2.19662324 | -1.83558084 | -3.34918622 |
| 1 | -1.72666262 | -2.56579652 | -4.91444621 |
| 1 | -0.89610899 | -1.09803246 | -4.32590679 |
| 1 | 4.04309674 | -2.40554966 | -1.20024228 |
| 1 | 4.20968110 | -2.03959345 | -2.94187735 |
| 1 | 4.34647482 | -3.71529052 | -2.37348105 |

Electronic energy = -3673.031998 a.u.
DFT-D3(BJ) dispersion correction = -0.118230 a.u.
Thermal free energy = 0.585291 a.u.
Gibbs free energy = -3672.564937 a.u.
Number of imaginary frequencies = 0.

**Int1 LA isomer/conformer 23**

| Atomic N. | X | Y | Z |
|---|---|---|---|
| 7 | 0.51737828 | -1.91895363 | -1.63671762 |
| 8 | 0.14522848 | -0.92189090 | 1.17553089 |
| 8 | 1.10434541 | 0.98233230 | -1.05876534 |
| 6 | -0.58873614 | -2.70270210 | -2.24478845 |
| 6 | 0.07702172 | -2.25219652 | 1.46576731 |
| 6 | -0.44269144 | -2.67738720 | 2.72246575 |
| 6 | -0.51125358 | -4.05975689 | 2.95144663 |
| 6 | -0.08218224 | -5.00756737 | 2.01886495 |
| 6 | 0.47175907 | -4.57678673 | 0.81883583 |
| 6 | 0.57358189 | -3.20864367 | 0.54085812 |
| 6 | 1.32685202 | -2.76290115 | -0.68513117 |
| 1 | -0.16506397 | -6.07116465 | 2.24171694 |
| 6 | -0.89270484 | -1.67185973 | 3.80131661 |
| 6 | 2.36171398 | 0.63274386 | -1.44256446 |
| 6 | 3.47185802 | 1.44766966 | -1.07635131 |
| 6 | 4.73871628 | 1.04187786 | -1.52037085 |
| 6 | 4.94258993 | -0.10821827 | -2.28773349 |
| 6 | 3.85034470 | -0.88992136 | -2.64448133 |
| 6 | 2.56043096 | -0.52425007 | -2.24031960 |
| 6 | 1.38671109 | -1.33385103 | -2.72870146 |
| 1 | 5.94868735 | -0.38471298 | -2.60233449 |
| 6 | 3.29815598 | 2.72171344 | -0.22597325 |
| 6 | -1.32808343 | -2.37822677 | 5.09949805 |
| 6 | -2.10049448 | -0.85024930 | 3.29782848 |
| 6 | 0.27916720 | -0.72920876 | 4.16689537 |
| 6 | 4.63862035 | 3.44212611 | 0.01325950 |

| | | | |
|---|---|---|---|
| 6 | 2.71936001 | 2.35789017 | 1.16155350 |
| 6 | 2.36439138 | 3.72118235 | -0.94910702 |
| 1 | -0.17273757 | -3.50318250 | -2.87706443 |
| 1 | -1.19680348 | -3.14865097 | -1.45032508 |
| 1 | 1.70555109 | -3.63915739 | -1.23672734 |
| 1 | 2.19230624 | -2.14561608 | -0.40165328 |
| 1 | -0.91694645 | -4.41635552 | 3.89615981 |
| 1 | 0.85415135 | -5.29866605 | 0.09369027 |
| 1 | 0.70947265 | -0.71589619 | -3.33987430 |
| 1 | 1.74637017 | -2.16462348 | -3.35750619 |
| 1 | 5.60685168 | 1.64129794 | -1.25318649 |
| 1 | 3.98572389 | -1.78638318 | -3.25357103 |
| 1 | -1.61603682 | -1.61714179 | 5.83840628 |
| 1 | -2.19870234 | -3.03213826 | 4.94587269 |
| 1 | -0.51583968 | -2.97524888 | 5.53868056 |
| 1 | -1.87048619 | -0.29657588 | 2.38207572 |
| 1 | -2.95712314 | -1.51028580 | 3.09694487 |
| 1 | -2.40718740 | -0.12680878 | 4.06840735 |
| 1 | 1.12790544 | -1.30423046 | 4.56508959 |
| 1 | 0.62521375 | -0.15519953 | 3.30080930 |
| 1 | -0.04441520 | -0.02296864 | 4.94623782 |
| 1 | 4.45546767 | 4.34391891 | 0.61432596 |
| 1 | 5.11028055 | 3.76241715 | -0.92694674 |
| 1 | 5.35320152 | 2.81669846 | 0.56751122 |
| 1 | 1.74219383 | 1.87093759 | 1.07964025 |
| 1 | 3.39733524 | 1.68033050 | 1.70020213 |
| 1 | 2.60142262 | 3.26964883 | 1.76619208 |
| 1 | 2.79275878 | 4.02125501 | -1.91645764 |
| 1 | 1.37348580 | 3.29066891 | -1.12801337 |
| 1 | 2.24547971 | 4.62808102 | -0.33747635 |
| 1 | -1.21812349 | -2.03632755 | -2.84453552 |
| 8 | -3.44694335 | 0.66806981 | 0.24106517 |
| 6 | -4.23649487 | -0.53149442 | 0.17216225 |
| 1 | -4.67681001 | -0.64970160 | 1.16799536 |
| 8 | -1.78192374 | 0.00224299 | -1.25243572 |
| 1 | -3.60902104 | -1.40521101 | -0.05834513 |
| 1 | -5.03313044 | -0.43697761 | -0.57829790 |
| 31 | -0.12198197 | -0.32024740 | -0.55777086 |
| 8 | -3.66141218 | 1.01294055 | -2.01165916 |
| 6 | -2.73578385 | 0.97729710 | -0.92734711 |
| 6 | -2.07686440 | 2.37617347 | -0.72758802 |
| 8 | -2.98346506 | 3.45811820 | -1.09009043 |
| 6 | -3.54421412 | 3.38598310 | -2.33363188 |
| 6 | -3.37933428 | 2.01832419 | -3.00285333 |
| 8 | -4.14548273 | 4.33098487 | -2.79402232 |
| 6 | -4.33492753 | 1.82459949 | -4.16456666 |
| 6 | -1.62324566 | 2.66287739 | 0.69218614 |
| 1 | -1.21708970 | 2.41463556 | -1.41597421 |
| 1 | -2.33400807 | 1.91660042 | -3.35630027 |
| 1 | -4.17370986 | 0.83924179 | -4.61951125 |
| 1 | -4.16915841 | 2.60423733 | -4.91732017 |
| 1 | -5.37348995 | 1.89739384 | -3.81904926 |
| 1 | -0.94045639 | 1.88404772 | 1.05914243 |

| | | | |
|---|---|---|---|
| 1 | -2.48693236 | 2.70146090 | 1.36469197 |
| 1 | -1.09059422 | 3.62151926 | 0.72011408 |

Electronic energy = -3673.032629 a.u.
DFT-D3(BJ) dispersion correction = -0.117322 a.u.
Thermal free energy = 0.585878 a.u.
Gibbs free energy = -3672.564073 a.u.
Number of imaginary frequencies = 0.

**Int1 LA isomer/conformer 24**

| Atomic N. | X | Y | Z |
|---|---|---|---|
| 7 | 1.49067708 | 0.90369928 | -1.44429795 |
| 8 | 1.12173835 | -0.74836817 | 1.00318044 |
| 8 | -0.80920979 | 1.58316950 | 0.39474916 |
| 6 | 1.63663795 | 0.23563028 | -2.76364864 |
| 6 | 2.28153690 | -1.32700925 | 0.59516052 |
| 6 | 2.66221532 | -2.62223483 | 1.03974330 |
| 6 | 3.91024965 | -3.10160083 | 0.60139683 |
| 6 | 4.75546285 | -2.38684420 | -0.24505945 |
| 6 | 4.36419169 | -1.12379918 | -0.68319179 |
| 6 | 3.14646178 | -0.58722026 | -0.26162589 |
| 6 | 2.77172675 | 0.81869848 | -0.64819869 |
| 1 | 5.70861865 | -2.81287098 | -0.55740682 |
| 6 | 1.84540074 | -3.54652020 | 1.97714605 |
| 6 | -0.10773831 | 2.73347205 | 0.57493142 |
| 6 | -0.38175198 | 3.55602829 | 1.70525313 |
| 6 | 0.36474130 | 4.73583451 | 1.83476902 |
| 6 | 1.34400767 | 5.11894526 | 0.91375402 |
| 6 | 1.58972005 | 4.31416326 | -0.19272005 |
| 6 | 0.86381832 | 3.13146456 | -0.37977742 |
| 6 | 1.06314379 | 2.34049282 | -1.64752302 |
| 1 | 1.90373478 | 6.04212735 | 1.06284803 |
| 6 | -1.45294795 | 3.16990016 | 2.74443659 |
| 6 | 2.63280602 | -3.72303092 | 3.29725786 |
| 6 | 1.67202515 | -4.92749124 | 1.29890232 |
| 6 | 0.43086768 | -3.05315503 | 2.34245885 |
| 6 | -1.59165736 | 4.23551805 | 3.84816917 |
| 6 | -1.06905179 | 1.84049394 | 3.43644861 |
| 6 | -2.83437825 | 3.03335713 | 2.06016136 |
| 1 | 2.40364143 | 0.75490860 | -3.36004648 |
| 1 | 1.93829377 | -0.80642587 | -2.61448272 |
| 1 | 3.57598961 | 1.27995277 | -1.24466774 |
| 1 | 2.61167288 | 1.43916843 | 0.24548589 |
| 1 | 4.23537624 | -4.08717261 | 0.93553712 |
| 1 | 5.01173822 | -0.53813012 | -1.33951984 |
| 1 | 0.12478413 | 2.28314290 | -2.22105573 |
| 1 | 1.82062693 | 2.83415863 | -2.27818762 |
| 1 | 0.18349897 | 5.38310569 | 2.69080740 |
| 1 | 2.33831805 | 4.60423006 | -0.93336128 |
| 1 | 2.08944556 | -4.40139202 | 3.97251388 |
| 1 | 3.63267028 | -4.14472611 | 3.12665482 |
| 1 | 2.75519457 | -2.75848733 | 3.80999947 |
| 1 | 1.12004688 | -4.82938948 | 0.35307688 |

| | | | |
|---|---|---|---|
| 1 | 2.63037823 | -5.41672435 | 1.08187871 |
| 1 | 1.09989287 | -5.59576272 | 1.95956991 |
| 1 | 0.44891898 | -2.10631984 | 2.89217474 |
| 1 | -0.19551311 | -2.91695909 | 1.45181879 |
| 1 | -0.04340689 | -3.81528294 | 2.98027552 |
| 1 | -2.37434647 | 3.91928072 | 4.55220867 |
| 1 | -1.88699830 | 5.21433998 | 3.44338608 |
| 1 | -0.66290942 | 4.36155553 | 4.42329190 |
| 1 | -1.00229932 | 1.01344042 | 2.72178262 |
| 1 | -0.10008149 | 1.93431645 | 3.94756843 |
| 1 | -1.82706401 | 1.58383360 | 4.19204960 |
| 1 | -3.13678656 | 3.98851832 | 1.60637838 |
| 1 | -2.82620268 | 2.26519003 | 1.27936066 |
| 1 | -3.59290311 | 2.76313652 | 2.81071743 |
| 1 | 0.67576815 | 0.26239147 | -3.28869303 |
| 8 | -3.15656204 | -0.44464268 | -0.93202710 |
| 6 | -3.58268329 | -1.36282931 | 0.08104330 |
| 1 | -2.72708619 | -1.78196473 | 0.63200275 |
| 8 | -0.95471870 | -1.21656071 | -1.09980550 |
| 8 | -1.82698942 | 0.24002241 | -2.61026461 |
| 1 | -4.20372770 | -0.78021953 | 0.77117416 |
| 1 | -4.19183146 | -2.18478460 | -0.33353140 |
| 31 | 0.04734126 | 0.08369024 | -0.25516291 |
| 8 | -2.12762990 | -1.97089031 | -4.05993800 |
| 6 | -2.36914660 | -0.78399404 | -4.68962417 |
| 6 | -2.72892489 | 0.35603152 | -3.73496075 |
| 6 | -2.09209390 | -0.89316292 | -1.78662345 |
| 6 | -2.58060273 | -2.09707072 | -2.67478240 |
| 8 | -2.22843364 | -0.68383539 | -5.88891320 |
| 6 | -2.10758027 | -3.46168154 | -2.21123288 |
| 6 | -2.55702096 | 1.72393492 | -4.36498259 |
| 1 | -3.76980604 | 0.23295311 | -3.38313567 |
| 1 | -3.68140486 | -2.05499750 | -2.69215039 |
| 1 | -2.50393929 | -4.23302471 | -2.88399133 |
| 1 | -2.45431425 | -3.66980082 | -1.19202883 |
| 1 | -1.01250911 | -3.50191625 | -2.21408376 |
| 1 | -3.21395647 | 1.81433395 | -5.23805284 |
| 1 | -1.52360877 | 1.86661932 | -4.70613123 |
| 1 | -2.81356223 | 2.50304282 | -3.63663663 |

Electronic energy = -3673.031591 a.u.
DFT-D3(BJ) dispersion correction = -0.116387 a.u.
Thermal free energy = 0.584021 a.u.
Gibbs free energy = -3672.563957 a.u.
Number of imaginary frequencies = 0.

**Int1 LA isomer/conformer 25**

| Atomic N. | X | Y | Z |
|---|---|---|---|
| 7 | 0.45313494 | 0.31005524 | 2.33141717 |
| 8 | 1.53626622 | 0.24592194 | -0.46687578 |
| 8 | -0.65349309 | -1.75737815 | 0.45713674 |
| 6 | 0.02808067 | 1.59412506 | 2.94713749 |
| 6 | 2.41919838 | 1.15213405 | 0.03388952 |

| | | | |
|---|---|---|---|
| 6 | 3.14922032 | 2.00441837 | -0.84234852 |
| 6 | 4.04428323 | 2.90864264 | -0.25076801 |
| 6 | 4.23918866 | 2.99360764 | 1.13099947 |
| 6 | 3.53518661 | 2.13935777 | 1.97290782 |
| 6 | 2.63715055 | 1.21098385 | 1.43445264 |
| 6 | 1.96402228 | 0.20508536 | 2.32674821 |
| 6 | 2.96984321 | 1.94152346 | -2.37179593 |
| 6 | -0.05165831 | -2.62729279 | 1.31535055 |
| 6 | 0.25215054 | -3.95492741 | 0.89855097 |
| 6 | 0.84957526 | -4.79883685 | 1.84651124 |
| 6 | 1.15200697 | -4.38865932 | 3.14788521 |
| 6 | 0.84856137 | -3.09058240 | 3.54032094 |
| 6 | 0.23977145 | -2.20991978 | 2.63806006 |
| 6 | -0.16063138 | -0.83744291 | 3.10810838 |
| 6 | -0.05260236 | -4.45392620 | -0.52790181 |
| 6 | 3.91041235 | 2.92061323 | -3.09964277 |
| 6 | 1.52029970 | 2.32350506 | -2.75409026 |
| 6 | 3.29829248 | 0.52008273 | -2.88797990 |
| 6 | 0.36810134 | -5.92287202 | -0.72473562 |
| 6 | 0.72473733 | -3.61207969 | -1.56720044 |
| 6 | -1.57167620 | -4.37029767 | -0.80570916 |
| 1 | 0.33123750 | 1.61042605 | 4.00610425 |
| 1 | 0.51419301 | 2.42409829 | 2.42368794 |
| 1 | 2.31227839 | 0.31764229 | 3.36656619 |
| 1 | 2.19368213 | -0.81939689 | 2.00060882 |
| 1 | 4.61306450 | 3.58023396 | -0.89112973 |
| 1 | 3.69133035 | 2.17073976 | 3.05357403 |
| 1 | -1.25072541 | -0.69597519 | 3.03372210 |
| 1 | 0.12176219 | -0.71147107 | 4.16591437 |
| 1 | 1.09856362 | -5.81857808 | 1.55981164 |
| 1 | 1.06987461 | -2.75098323 | 4.55444964 |
| 1 | 3.75427192 | 2.82443156 | -4.18334476 |
| 1 | 3.70989900 | 3.96790398 | -2.83063392 |
| 1 | 4.96968472 | 2.70500255 | -2.89764729 |
| 1 | 0.78229538 | 1.64610954 | -2.31186232 |
| 1 | 1.29085698 | 3.34800003 | -2.42579454 |
| 1 | 1.40474511 | 2.28815013 | -3.84793776 |
| 1 | 4.33988047 | 0.25446392 | -2.65487384 |
| 1 | 2.63948189 | -0.23414476 | -2.44480856 |
| 1 | 3.17827239 | 0.48825630 | -3.98136582 |
| 1 | 0.12347292 | -6.22766378 | -1.75195040 |
| 1 | -0.16489610 | -6.60198382 | -0.04373006 |
| 1 | 1.44941361 | -6.06710944 | -0.58693635 |
| 1 | 0.43273229 | -2.55714443 | -1.54111140 |
| 1 | 1.80769460 | -3.67597315 | -1.38856258 |
| 1 | 0.52521758 | -3.99710269 | -2.57843147 |
| 1 | -2.13190696 | -4.99862760 | -0.09813227 |
| 1 | -1.93937879 | -3.34230663 | -0.72340039 |
| 1 | -1.78440913 | -4.73358773 | -1.82231258 |
| 1 | -1.05859321 | 1.70520545 | 2.86407146 |
| 8 | -1.31640832 | 1.04275331 | -0.52475864 |
| 8 | -2.34183214 | 2.54762037 | 0.94333484 |
| 6 | -1.52276736 | 3.66860489 | 0.56805740 |

| | | | |
|---|---|---|---|
| 1 | -1.43236344 | 4.28726833 | 1.46834843 |
| 1 | -0.52584138 | 3.34710236 | 0.23133314 |
| 1 | -1.98838406 | 4.25938575 | -0.23416538 |
| 31 | -0.09689484 | 0.00962130 | 0.37027132 |
| 8 | -3.07359077 | 2.17180285 | -1.26460624 |
| 6 | -2.50950019 | 1.57812186 | -0.10258257 |
| 6 | -3.50688627 | 0.53544363 | 0.44722822 |
| 8 | -4.78019093 | 1.16699092 | 0.78720084 |
| 6 | -5.21892101 | 2.23528212 | 0.07300167 |
| 6 | -4.32632520 | 2.84086252 | -1.01703145 |
| 8 | -6.29935729 | 2.72510967 | 0.33016048 |
| 6 | -5.06889948 | 2.92046489 | -2.34797797 |
| 6 | -3.75819865 | -0.62510120 | -0.50231667 |
| 1 | -3.13472497 | 0.16818364 | 1.41621181 |
| 1 | -4.12333374 | 3.86663879 | -0.65756867 |
| 1 | -4.44193680 | 3.43809593 | -3.08476600 |
| 1 | -6.00626716 | 3.47226107 | -2.21473658 |
| 1 | -5.29468755 | 1.91459562 | -2.72559714 |
| 1 | -2.84859617 | -1.22327577 | -0.62652689 |
| 1 | -4.07889809 | -0.26048087 | -1.48642338 |
| 1 | -4.54455353 | -1.26663129 | -0.08483991 |
| 1 | 4.94407697 | 3.71761543 | 1.53943612 |
| 1 | 1.62186433 | -5.08279266 | 3.84434061 |

Electronic energy = -3673.031742 a.u.
DFT-D3(BJ) dispersion correction = -0.118070 a.u.
Thermal free energy = 0.586195 a.u.
Gibbs free energy = -3672.563616 a.u.
Number of imaginary frequencies = 0.

**Int1 LA isomer/conformer 26**

| Atomic N. | X | Y | Z |
|---|---|---|---|
| 7 | -2.59891824 | 0.19139447 | -1.11020149 |
| 8 | 0.28433699 | -0.55145660 | -1.22465526 |
| 8 | -0.70549429 | 1.80865488 | 0.57659603 |
| 6 | -3.61967993 | -0.85204331 | -0.83411687 |
| 6 | -0.19737637 | -1.46871992 | -2.10768107 |
| 6 | 0.48490249 | -2.69964333 | -2.31830107 |
| 6 | -0.04141754 | -3.56519157 | -3.28936452 |
| 6 | -1.19611752 | -3.27114552 | -4.01979751 |
| 6 | -1.87944297 | -2.08481292 | -3.76950350 |
| 6 | -1.39020050 | -1.18270441 | -2.81977333 |
| 6 | -2.11596610 | 0.10528431 | -2.53922398 |
| 1 | -1.56507572 | -3.97650928 | -4.76424463 |
| 6 | 1.74872319 | -3.07126139 | -1.51731288 |
| 6 | -1.05051199 | 2.80202499 | -0.29179420 |
| 6 | -0.22627779 | 3.95761026 | -0.42094587 |
| 6 | -0.64749743 | 4.94801657 | -1.32026255 |
| 6 | -1.81868251 | 4.84258728 | -2.07496851 |
| 6 | -2.62086268 | 3.71749609 | -1.92835548 |
| 6 | -2.25396449 | 2.70439420 | -1.03462725 |
| 6 | -3.18954655 | 1.54796964 | -0.81222526 |
| 1 | -2.09835936 | 5.63780412 | -2.76577173 |

| | | | |
|---|---|---|---|
| 6 | 1.07410003 | 4.12491698 | 0.38754392 |
| 6 | 2.17127145 | -4.53414363 | -1.75907392 |
| 6 | 1.55444582 | -2.85715540 | 0.01689612 |
| 6 | 2.91488653 | -2.16207057 | -1.97415306 |
| 6 | 1.76789050 | 5.46906875 | 0.09523906 |
| 6 | 2.07364891 | 3.00573586 | 0.01224212 |
| 6 | 0.76062175 | 4.09230303 | 1.90206601 |
| 1 | -4.50401003 | -0.68457738 | -1.47040125 |
| 1 | -3.20147709 | -1.84119409 | -1.04361760 |
| 1 | -2.98692598 | 0.21235555 | -3.20706791 |
| 1 | -1.45810299 | 0.97265796 | -2.69067500 |
| 1 | 0.45640067 | -4.51469749 | -3.47749754 |
| 1 | -2.79546396 | -1.84629041 | -4.31458316 |
| 1 | -3.50769693 | 1.50517861 | 0.24188216 |
| 1 | -4.09243424 | 1.67310149 | -1.43289467 |
| 1 | -0.03537548 | 5.83915358 | -1.44449915 |
| 1 | -3.54967704 | 3.61984668 | -2.49473282 |
| 1 | 3.05828150 | -4.75536398 | -1.14885754 |
| 1 | 1.38630086 | -5.25306153 | -1.48348533 |
| 1 | 2.44581686 | -4.71517162 | -2.80775669 |
| 1 | 5.59302792 | -1.85305019 | 1.64300875 |
| 1 | 2.40279431 | -3.33335443 | 0.53476805 |
| 1 | 1.61114987 | -1.78662829 | 0.22817768 |
| 1 | 3.13725155 | -2.32100165 | -3.03902141 |
| 1 | 2.67180593 | -1.10353218 | -1.82007306 |
| 1 | 3.82413969 | -2.39674664 | -1.39916528 |
| 1 | 2.67917425 | 5.53797325 | 0.70603395 |
| 1 | 1.13201326 | 6.32790019 | 0.35419811 |
| 1 | 2.06946073 | 5.56181177 | -0.95821648 |
| 1 | 1.67029138 | 2.00951291 | 0.22069571 |
| 1 | 2.32758979 | 3.05705018 | -1.05642444 |
| 1 | 3.00435213 | 3.12914581 | 0.58678278 |
| 1 | 0.09704473 | 4.92564154 | 2.17504741 |
| 1 | 0.27578497 | 3.15751068 | 2.20188575 |
| 1 | 1.69075856 | 4.20297591 | 2.47959749 |
| 1 | -3.90806089 | -0.80583328 | 0.22148144 |
| 8 | 0.00554368 | -2.98280945 | 1.96573398 |
| 6 | 0.24428597 | -3.39978870 | 0.59745450 |
| 1 | 4.97853561 | -1.88078767 | 1.21187726 |
| 8 | -1.46174092 | -1.29874771 | 1.24799939 |
| 8 | 0.53083473 | -0.67502308 | 2.07545959 |
| 1 | 0.25347435 | -4.49680689 | 0.65713347 |
| 1 | -0.61570061 | -3.10412104 | -0.01818744 |
| 31 | -0.91107364 | 0.03922515 | 0.07908561 |
| 8 | -1.02107086 | -0.43097263 | 4.26416232 |
| 6 | 0.19303990 | 0.19818981 | 4.27992373 |
| 6 | 1.21912931 | -0.41805695 | 3.32248899 |
| 6 | -0.52013836 | -1.67943535 | 2.16911087 |
| 6 | -1.05985411 | -1.74172080 | 3.62734486 |
| 8 | 0.37197011 | 1.17617968 | 4.97020672 |
| 6 | -2.48833332 | -2.23926641 | 3.71398763 |
| 6 | 2.40556801 | 0.48832581 | 3.06820951 |
| 1 | 1.56881514 | -1.38194280 | 3.73841315 |

| | | | |
|---|---|---|---|
| 1 | -0.39141141 | -2.41412707 | 4.18663406 |
| 1 | -2.79737560 | -2.30146498 | 4.76491520 |
| 1 | -2.56039618 | -3.23690938 | 3.26292941 |
| 1 | -3.16028919 | -1.55808649 | 3.17899865 |
| 1 | 2.92479431 | 0.68399997 | 4.01408192 |
| 1 | 2.07710289 | 1.44761499 | 2.65399305 |
| 1 | 3.10064019 | 0.00681544 | 2.36922424 |

Electronic energy = -3673.012817 a.u.
DFT-D3(BJ) dispersion correction = -0.122214 a.u.
Thermal free energy = 0.577338 a.u.
Gibbs free energy = -3672.557694 a.u.
Number of imaginary frequencies = 0.

**Int1 LA isomer/conformer 27**

| Atomic N. | X | Y | Z |
|---|---|---|---|
| 7 | -2.57928203 | -0.11755329 | -0.93672628 |
| 8 | 0.41285905 | -0.20795177 | -0.95329012 |
| 8 | -1.05902667 | 1.82741467 | 0.78673506 |
| 6 | -3.41016054 | -1.32440244 | -0.70147354 |
| 6 | 0.25071827 | -1.08976621 | -1.97054661 |
| 6 | 1.32747320 | -1.91783530 | -2.40130219 |
| 6 | 1.04447956 | -2.84663475 | -3.41687956 |
| 6 | -0.20849055 | -2.96009936 | -4.02272432 |
| 6 | -1.22764604 | -2.09146632 | -3.64449680 |
| 6 | -1.00313339 | -1.14627086 | -2.63983247 |
| 6 | -2.04454865 | -0.09703857 | -2.34820259 |
| 1 | -0.37312123 | -3.70447265 | -4.80134328 |
| 6 | 2.75953440 | -1.82023505 | -1.82226915 |
| 6 | -1.55270294 | 2.74559870 | -0.08107936 |
| 6 | -0.95241982 | 4.03363385 | -0.18304455 |
| 6 | -1.52338457 | 4.93418515 | -1.09361929 |
| 6 | -2.63387559 | 4.61730481 | -1.88212395 |
| 6 | -3.22024193 | 3.36288593 | -1.76039428 |
| 6 | -2.69732161 | 2.42921085 | -0.85733769 |
| 6 | -3.40968556 | 1.11912901 | -0.65204922 |
| 1 | -3.03565643 | 5.35146464 | -2.58021859 |
| 6 | 0.26948849 | 4.42592408 | 0.67040555 |
| 6 | 3.79039931 | -2.49295406 | -2.75950728 |
| 6 | 2.89747966 | -2.60913718 | -0.49806341 |
| 6 | 3.22068281 | -0.35690607 | -1.64034044 |
| 6 | 0.71329975 | 5.87688758 | 0.40516497 |
| 6 | 1.47056945 | 3.50971645 | 0.33744362 |
| 6 | -0.07839793 | 4.31697401 | 2.17434708 |
| 1 | -4.32170375 | -1.27431554 | -1.31836162 |
| 1 | -2.83988367 | -2.21846725 | -0.97393489 |
| 1 | -2.89757730 | -0.20358855 | -3.03868205 |
| 1 | -1.61907081 | 0.90745469 | -2.48809460 |
| 1 | 1.83581668 | -3.51128904 | -3.75800543 |
| 1 | -2.19886358 | -2.12438843 | -4.14345534 |
| 1 | -3.73751629 | 1.01314994 | 0.39441189 |
| 1 | -4.30421933 | 1.07670879 | -1.29487641 |
| 1 | -1.08393228 | 5.92416260 | -1.20082656 |

| | | | |
|---|---|---|---|
| 1 | -4.09927558 | 3.10119784 | -2.35371649 |
| 1 | 4.80272878 | -2.30565419 | -2.37683467 |
| 1 | 3.66273567 | -3.58252717 | -2.82910085 |
| 1 | 3.73083798 | -2.07303334 | -3.77243655 |
| 1 | -0.29554185 | -2.82702906 | 0.28371776 |
| 1 | 2.61224329 | -3.65835832 | -0.66233117 |
| 1 | 3.93695667 | -2.55787699 | -0.14615102 |
| 1 | 3.17263367 | 0.17560538 | -2.60110133 |
| 1 | 2.60435928 | 0.18911889 | -0.92269991 |
| 1 | 4.26333208 | -0.34132220 | -1.29340730 |
| 1 | 1.57612283 | 6.10811492 | 1.04559363 |
| 1 | -0.07844242 | 6.60193783 | 0.64314274 |
| 1 | 1.02633484 | 6.03153427 | -0.63759737 |
| 1 | 1.25291281 | 2.45935057 | 0.55713561 |
| 1 | 1.74094569 | 3.59129408 | -0.72515571 |
| 1 | 2.34450403 | 3.81178385 | 0.93386334 |
| 1 | -0.90240210 | 4.99865482 | 2.43118701 |
| 1 | -0.37170944 | 3.29765155 | 2.44773525 |
| 1 | 0.79607435 | 4.60273592 | 2.77828665 |
| 1 | -3.68562785 | -1.37941620 | 0.35837501 |
| 8 | -1.64468887 | -2.55655197 | 3.26795421 |
| 6 | -1.67691410 | -1.74694218 | 4.45120866 |
| 1 | -2.47587518 | -2.16724307 | 5.07296677 |
| 8 | -1.06163386 | -0.84197630 | 1.87381888 |
| 1 | -1.90912197 | -0.69867126 | 4.21204582 |
| 1 | -0.72246183 | -1.80379264 | 4.99454662 |
| 31 | -0.94089078 | 0.04749072 | 0.28920975 |
| 8 | 0.58614370 | -2.19711313 | 2.87865884 |
| 6 | -0.73485381 | -2.13624605 | 2.26943449 |
| 6 | -0.85842035 | -3.18961547 | 1.15469163 |
| 8 | 2.03164886 | -2.14206646 | 0.56513937 |
| 6 | 2.61815863 | -1.56603258 | 1.64827101 |
| 6 | 1.55531144 | -1.16528891 | 2.69140010 |
| 8 | 3.81275487 | -1.36883909 | 1.76768289 |
| 6 | 2.20723649 | -0.85240824 | 4.03383875 |
| 6 | -0.36791064 | -4.58434286 | 1.54491003 |
| 1 | -1.92461615 | -3.23326874 | 0.88878691 |
| 1 | 1.06657619 | -0.25350588 | 2.30829109 |
| 1 | 1.45557013 | -0.44854667 | 4.72329170 |
| 1 | 3.01085826 | -0.11785413 | 3.90527607 |
| 1 | 2.63810320 | -1.76221260 | 4.47123984 |
| 1 | -0.91549108 | -4.96041631 | 2.41853663 |
| 1 | 0.70067033 | -4.56791754 | 1.79101734 |
| 1 | -0.52081431 | -5.28743413 | 0.71387166 |

Electronic energy = -3673.024887 a.u.
DFT-D3(BJ) dispersion correction = -0.120706 a.u.
Thermal free energy = 0.588709 a.u.
Gibbs free energy = -3672.556884 a.u.
Number of imaginary frequencies = 0.

**Int1 LA isomer/conformer 28**

Atomic N. X Y Z

| | | | |
|---|---|---|---|
| 7 | -0.96116260 | -1.97322110 | 1.41666528 |
| 8 | -0.94828406 | 0.56494321 | -0.19856032 |
| 8 | 1.39345932 | -0.12833385 | 1.64036034 |
| 6 | -1.34327713 | -3.24120558 | 0.75100213 |
| 6 | -2.28030298 | 0.34346372 | -0.31092052 |
| 6 | -3.06631256 | 1.09801491 | -1.23137576 |
| 6 | -4.41657009 | 0.73523065 | -1.36222138 |
| 6 | -5.01509996 | -0.27565391 | -0.60801024 |
| 6 | -4.26460411 | -0.92256130 | 0.36765156 |
| 6 | -2.91401829 | -0.60423702 | 0.54085904 |
| 6 | -2.17998168 | -1.14849882 | 1.73766877 |
| 1 | -6.06586338 | -0.52229906 | -0.75789400 |
| 6 | -2.51316986 | 2.29239161 | -2.04403534 |
| 6 | 1.02108016 | -0.06131996 | 2.93903593 |
| 6 | 1.47322568 | 1.01531523 | 3.75778087 |
| 6 | 1.06371524 | 1.01664470 | 5.09837241 |
| 6 | 0.24620219 | 0.02247079 | 5.64536979 |
| 6 | -0.17819409 | -1.02790005 | 4.84039394 |
| 6 | 0.21218989 | -1.08570826 | 3.49662317 |
| 6 | -0.17078989 | -2.28522631 | 2.67186596 |
| 1 | -0.05010599 | 0.07309836 | 6.69294986 |
| 6 | 2.37478150 | 2.13208720 | 3.19630926 |
| 6 | -3.65677429 | 3.21410784 | -2.53354029 |
| 6 | -1.83393111 | 1.82089040 | -3.35028346 |
| 6 | -1.57198615 | 3.18754723 | -1.20636399 |
| 6 | 2.75508749 | 3.16104283 | 4.27771326 |
| 6 | 1.63745796 | 2.89758986 | 2.07221572 |
| 6 | 3.69152678 | 1.52847692 | 2.65161945 |
| 1 | -1.95523160 | -3.85130778 | 1.43495535 |
| 1 | -1.92591526 | -3.02207636 | -0.15062911 |
| 1 | -2.85973184 | -1.76442285 | 2.35001736 |
| 1 | -1.82036225 | -0.32204754 | 2.36852436 |
| 1 | -5.03679316 | 1.26936522 | -2.07939881 |
| 1 | -4.72788912 | -1.66185103 | 1.02511618 |
| 1 | 0.72391171 | -2.82673664 | 2.32430924 |
| 1 | -0.77168430 | -2.97828737 | 3.28359125 |
| 1 | 1.38799384 | 1.82829306 | 5.74704195 |
| 1 | -0.80589173 | -1.82235595 | 5.25047595 |
| 1 | -3.22410207 | 4.10302582 | -3.01269512 |
| 1 | -4.31522306 | 2.73399501 | -3.27070545 |
| 1 | -4.27223972 | 3.55357045 | -1.68988579 |
| 1 | -0.41520325 | -3.14164325 | -2.10647844 |
| 1 | -2.57479053 | 1.31172081 | -3.98219105 |
| 1 | -1.41869529 | 2.68802185 | -3.88285844 |
| 1 | -2.09655886 | 3.54324594 | -0.30759617 |
| 1 | -0.66754103 | 2.66794946 | -0.88538217 |
| 1 | -1.27531597 | 4.06291874 | -1.79998912 |
| 1 | 3.40418092 | 3.92597064 | 3.82861991 |
| 1 | 3.31077318 | 2.70239822 | 5.10850627 |
| 1 | 1.87483864 | 3.67626418 | 4.68886191 |
| 1 | 1.37665311 | 2.24111835 | 1.23563972 |
| 1 | 0.71349918 | 3.35495163 | 2.45479762 |
| 1 | 2.28038551 | 3.70379495 | 1.68829472 |

| | | | |
|---|---|---|---|
| 1 | 4.24429076 | 1.01904316 | 3.45461445 |
| 1 | 3.50373390 | 0.80976536 | 1.84684936 |
| 1 | 4.33216633 | 2.33241133 | 2.25873287 |
| 1 | -0.43902491 | -3.79502891 | 0.47525365 |
| 8 | 2.79471389 | -2.18215935 | -2.50240174 |
| 6 | 3.62350178 | -2.93595061 | -1.61248156 |
| 1 | 3.29988543 | -3.98907996 | -1.54322730 |
| 8 | 1.40268761 | -1.64413066 | -0.77750639 |
| 1 | 3.64116754 | -2.49197451 | -0.60800599 |
| 1 | 4.62936027 | -2.90660773 | -2.04851282 |
| 31 | 0.25858811 | -0.74725510 | 0.30848226 |
| 8 | 0.84201359 | -1.12310884 | -3.00921644 |
| 6 | 1.42086986 | -2.05032319 | -2.11924792 |
| 6 | 0.63935627 | -3.36295663 | -2.32702364 |
| 8 | -0.78638515 | 0.83628546 | -3.17027620 |
| 6 | 0.49026819 | 1.27455118 | -3.26235200 |
| 6 | 1.51486909 | 0.13646299 | -3.17675373 |
| 8 | 0.82662720 | 2.43324800 | -3.43133487 |
| 6 | 2.37370726 | 0.14993785 | -4.44726163 |
| 6 | 0.76933935 | -3.95873385 | -3.72894961 |
| 1 | 0.98899525 | -4.07810726 | -1.56725625 |
| 1 | 2.14573725 | 0.34766948 | -2.29795521 |
| 1 | 3.17476422 | -0.59014430 | -4.35391954 |
| 1 | 2.80368704 | 1.14831229 | -4.59456329 |
| 1 | 1.75534773 | -0.10214139 | -5.31983962 |
| 1 | 1.81756566 | -4.18034779 | -3.96838048 |
| 1 | 0.38985484 | -3.25986102 | -4.48458834 |
| 1 | 0.19494659 | -4.89280181 | -3.80507160 |

Electronic energy = -3673.024611 a.u.
DFT-D3(BJ) dispersion correction = -0.118092 a.u.
Thermal free energy = 0.587886 a.u.
Gibbs free energy = -3672.554817 a.u.
Number of imaginary frequencies = 0.

**TS2 ax LA isomer/conformer 1**

| Atomic N. | X | Y | Z |
|---|---|---|---|
| 7 | 0.60299641 | 0.72826207 | -2.11924458 |
| 8 | 1.32392025 | 0.62730861 | 0.69452834 |
| 8 | -1.62412679 | -0.09584198 | -0.30338505 |
| 6 | 1.71411920 | 0.21109137 | -2.95500994 |
| 6 | 2.28013921 | 1.51326942 | 0.34804749 |
| 6 | 3.39232063 | 1.74025190 | 1.21538925 |
| 6 | 4.36268187 | 2.65767455 | 0.78777939 |
| 6 | 4.27539778 | 3.35063505 | -0.42366537 |
| 6 | 3.16924429 | 3.14835954 | -1.24160528 |
| 6 | 2.16868544 | 2.25009775 | -0.85596651 |
| 6 | 0.91276431 | 2.13048244 | -1.66420937 |
| 1 | 5.06060839 | 4.04907084 | -0.71269667 |
| 6 | 3.51724195 | 1.02822888 | 2.57612228 |
| 6 | -2.27212381 | 0.92320728 | -0.91849089 |
| 6 | -3.40152979 | 1.52590334 | -0.28694960 |
| 6 | -4.05603552 | 2.55283260 | -0.98154980 |

| | | | |
|---|---|---|---|
| 6 | -3.64120436 | 3.00438118 | -2.23714709 |
| 6 | -2.53065419 | 2.42040730 | -2.83419180 |
| 6 | -1.84758317 | 1.37981915 | -2.19312143 |
| 6 | -0.68435353 | 0.73393565 | -2.90282702 |
| 1 | -4.17974520 | 3.81044006 | -2.73505950 |
| 6 | -3.88859463 | 1.07937634 | 1.10664809 |
| 6 | 4.78047800 | 1.46295989 | 3.34212995 |
| 6 | 3.59934878 | -0.50203532 | 2.37287307 |
| 6 | 2.29594803 | 1.37218022 | 3.46200919 |
| 6 | -5.11013365 | 1.89159524 | 1.57780612 |
| 6 | -2.76997423 | 1.28566730 | 2.15551448 |
| 6 | -4.31596470 | -0.40648872 | 1.07102406 |
| 1 | 1.80003059 | 0.80495119 | -3.88013768 |
| 1 | 2.65201092 | 0.29450068 | -2.39454675 |
| 1 | 0.97216046 | 2.77245808 | -2.56001639 |
| 1 | 0.04279112 | 2.46096611 | -1.07888746 |
| 1 | 5.22689390 | 2.84359550 | 1.42291951 |
| 1 | 3.06131291 | 3.70030713 | -2.17827678 |
| 1 | -0.90288234 | -0.31416631 | -3.14961410 |
| 1 | -0.50145149 | 1.26444161 | -3.85282421 |
| 1 | -4.92120123 | 3.02968240 | -0.52511638 |
| 1 | -2.18465567 | 2.76229525 | -3.81221008 |
| 1 | 4.81177282 | 0.93558848 | 4.30621719 |
| 1 | 5.70296240 | 1.21214175 | 2.79829264 |
| 1 | 4.78472042 | 2.54157186 | 3.55554295 |
| 1 | 2.68959737 | -0.88346386 | 1.89761019 |
| 1 | 4.46826696 | -0.76395702 | 1.75016618 |
| 1 | 3.71978426 | -0.99981028 | 3.34770417 |
| 1 | 2.24403858 | 2.45538235 | 3.64477038 |
| 1 | 1.35912919 | 1.05053759 | 2.99400163 |
| 1 | 2.38972521 | 0.86911118 | 4.43655443 |
| 1 | -5.41290331 | 1.53422284 | 2.57223129 |
| 1 | -5.97308779 | 1.77091539 | 0.90712154 |
| 1 | -4.88469791 | 2.96398830 | 1.66718543 |
| 1 | -1.87843812 | 0.69351721 | 1.92429447 |
| 1 | -2.47914624 | 2.34482098 | 2.20736551 |
| 1 | -3.13465142 | 0.98730153 | 3.15009260 |
| 1 | -5.13857126 | -0.55832928 | 0.35721454 |
| 1 | -3.48059511 | -1.05274880 | 0.78217310 |
| 1 | -4.66887093 | -0.71786112 | 2.06582093 |
| 1 | 1.53485227 | -0.83829749 | -3.20441222 |
| 8 | 0.94617456 | -4.02782202 | 0.45222992 |
| 6 | 2.35678601 | -3.74281693 | 0.62066877 |
| 1 | 2.71033096 | -3.09893990 | -0.19482777 |
| 8 | 0.46564150 | -1.85481143 | 0.97362590 |
| 8 | 0.20646781 | -2.28242755 | -1.35649967 |
| 1 | 2.84873486 | -4.71863508 | 0.57561952 |
| 1 | 2.53791571 | -3.25756788 | 1.58590353 |
| 31 | 0.21475786 | -0.43802679 | -0.40124771 |
| 8 | -1.54894412 | -4.44852495 | -0.57282102 |
| 6 | -1.24058056 | -4.23323679 | -1.88079786 |
| 6 | -0.72294188 | -2.83115721 | -2.30305433 |
| 6 | 0.12602036 | -2.96676493 | 0.41876736 |

| | | | |
|---|---|---|---|
| 6 | -1.33866978 | -3.38744950 | 0.39258596 |
| 8 | -1.43773210 | -5.12286615 | -2.67982984 |
| 6 | -1.79668105 | -3.91823578 | 1.74375331 |
| 6 | -0.12425599 | -2.94010496 | -3.70452571 |
| 1 | -1.63599610 | -2.19994640 | -2.33441043 |
| 1 | -1.92464530 | -2.50063753 | 0.11044365 |
| 1 | -2.86259604 | -4.17307329 | 1.69408727 |
| 1 | -1.65406358 | -3.14852548 | 2.51283741 |
| 1 | -1.22816229 | -4.81545734 | 2.01759553 |
| 1 | -0.81324375 | -3.48186755 | -4.36215067 |
| 1 | 0.82074726 | -3.49736810 | -3.66620731 |
| 1 | 0.06216800 | -1.95047188 | -4.13773441 |

Electronic energy = -3673.024170 a.u.
DFT-D3(BJ) dispersion correction = -0.120600 a.u.
Thermal free energy = 0.586918 a.u.
Gibbs free energy = -3672.557852 a.u.
Number of imaginary frequencies = 1.

**TS2 ax LA isomer/conformer 2**

| Atomic N. | X | Y | Z |
|---|---|---|---|
| 7 | -1.55911207 | 0.03417682 | 1.55896533 |
| 8 | -0.83306903 | -0.11127213 | -1.23088551 |
| 8 | 1.25591707 | 0.69230503 | 1.15917301 |
| 6 | -2.70497441 | -0.90497571 | 1.60169959 |
| 6 | -2.00277686 | 0.53831840 | -1.42782712 |
| 6 | -2.58594728 | 0.53023666 | -2.73317957 |
| 6 | -3.79859781 | 1.21139526 | -2.90411496 |
| 6 | -4.44894505 | 1.87788796 | -1.86209524 |
| 6 | -3.87217116 | 1.87927175 | -0.59800720 |
| 6 | -2.65254283 | 1.22963214 | -0.37553254 |
| 6 | -2.01008773 | 1.34797823 | 0.97836846 |
| 1 | -5.39550686 | 2.38729715 | -2.04215574 |
| 6 | -1.92105865 | -0.20461911 | -3.91382241 |
| 6 | 1.02878439 | 1.57171345 | 2.15842843 |
| 6 | 1.88072288 | 2.71308231 | 2.29327464 |
| 6 | 1.64627790 | 3.57250214 | 3.37512827 |
| 6 | 0.60894299 | 3.37588429 | 4.29036093 |
| 6 | -0.24535228 | 2.29368567 | 4.11869907 |
| 6 | -0.04422528 | 1.38941860 | 3.06921768 |
| 6 | -0.99078959 | 0.22327386 | 2.94070888 |
| 1 | 0.46196765 | 4.07583189 | 5.11269665 |
| 6 | 3.00301170 | 3.01511574 | 1.27917266 |
| 6 | -2.70386672 | -0.01648902 | -5.22722960 |
| 6 | -1.87715721 | -1.72098112 | -3.61597657 |
| 6 | -0.49079576 | 0.33494744 | -4.15465498 |
| 6 | 3.75975370 | 4.31225061 | 1.62262738 |
| 6 | 2.40066385 | 3.19558620 | -0.13486928 |
| 6 | 4.03948975 | 1.86905539 | 1.26406431 |
| 1 | -3.52700520 | -0.46482214 | 2.18960404 |
| 1 | -3.06173587 | -1.10457218 | 0.58634865 |
| 1 | -2.71674681 | 1.81257832 | 1.68654430 |
| 1 | -1.12379360 | 1.99679625 | 0.93691117 |

| | | | |
|---|---|---|---|
| 1 | -4.26255608 | 1.22129440 | -3.88851047 |
| 1 | -4.35678148 | 2.39875674 | 0.23188412 |
| 1 | -0.49583036 | -0.72668260 | 3.19709516 |
| 1 | -1.82978976 | 0.36479685 | 3.64271545 |
| 1 | 2.28872800 | 4.44091034 | 3.50704813 |
| 1 | -1.08202362 | 2.13586712 | 4.80315924 |
| 1 | -2.18248248 | -0.55361378 | -6.03259134 |
| 1 | -3.72296524 | -0.42479053 | -5.16649675 |
| 1 | -2.76863575 | 1.04091549 | -5.52237940 |
| 1 | -1.33108282 | -1.93296421 | -2.69124651 |
| 1 | -2.89470410 | -2.12573123 | -3.51518774 |
| 1 | -1.38416231 | -2.25214863 | -4.44527340 |
| 1 | -0.51827204 | 1.40881944 | -4.39119577 |
| 1 | 0.15009134 | 0.18746959 | -3.27927446 |
| 1 | -0.03960176 | -0.18619783 | -5.01321697 |
| 1 | 4.53865337 | 4.48035145 | 0.86527938 |
| 1 | 4.25784016 | 4.25558281 | 2.60143375 |
| 1 | 3.09963679 | 5.19148493 | 1.61792660 |
| 1 | 1.88904786 | 2.28881788 | -0.47518173 |
| 1 | 1.68297935 | 4.02881098 | -0.14583759 |
| 1 | 3.20074749 | 3.43021774 | -0.85328853 |
| 1 | 4.49416590 | 1.74164093 | 2.25760913 |
| 1 | 3.56899261 | 0.92771101 | 0.96587634 |
| 1 | 4.84396887 | 2.10260391 | 0.54981703 |
| 1 | -2.40290260 | -1.84876943 | 2.06119990 |
| 8 | 2.91889858 | -2.92450547 | 0.14031690 |
| 6 | 3.80976152 | -1.86410642 | 0.57590508 |
| 1 | 3.35312343 | -1.32093775 | 1.41192079 |
| 8 | 1.53925562 | -1.33750820 | -0.76669711 |
| 8 | 0.61040235 | -2.29119730 | 1.24358225 |
| 1 | 4.72425270 | -2.37326908 | 0.89358425 |
| 1 | 4.01047468 | -1.17341542 | -0.25038946 |
| 31 | 0.07379727 | -0.52656198 | 0.35609722 |
| 8 | -0.40790451 | -3.77511144 | -0.81906134 |
| 6 | -0.93574023 | -3.90582514 | 0.44471765 |
| 6 | 0.04699645 | -3.56335092 | 1.56591034 |
| 6 | 1.72169375 | -2.52432036 | -0.33658215 |
| 6 | 1.03772044 | -3.76048565 | -0.96554822 |
| 8 | -2.10847783 | -4.17596531 | 0.58003223 |
| 6 | 1.33111250 | -3.79508185 | -2.45946336 |
| 6 | -0.53143704 | -3.68319893 | 2.96817493 |
| 1 | 0.88513219 | -4.28636789 | 1.51371038 |
| 1 | 1.45030522 | -4.65699196 | -0.48087743 |
| 1 | 0.84765732 | -4.67176819 | -2.90750194 |
| 1 | 2.41406274 | -3.86527252 | -2.62597942 |
| 1 | 0.95378375 | -2.88533330 | -2.94029617 |
| 1 | -0.80201304 | -4.72977257 | 3.16295743 |
| 1 | -1.43350255 | -3.08058929 | 3.11372828 |
| 1 | 0.22830991 | -3.37670684 | 3.69875336 |

Electronic energy = -3673.018503 a.u.
DFT-D3(BJ) dispersion correction = -0.121542 a.u.
Thermal free energy = 0.587319 a.u.
Gibbs free energy = -3672.552726 a.u.

Number of imaginary frequencies = 1.

**TS2_ax_LA isomer/conformer 3**

| Atomic N. | X | Y | Z |
|---|---|---|---|
| 7 | 0.43231621 | 0.71766555 | -2.08899736 |
| 8 | 1.34541261 | 0.79332429 | 0.68382412 |
| 8 | -1.65933263 | -0.16163653 | -0.23766989 |
| 6 | 1.66107213 | 0.40242341 | -2.85438728 |
| 6 | 1.99306941 | 1.91447854 | 0.29578730 |
| 6 | 3.04409719 | 2.43171096 | 1.11592889 |
| 6 | 3.70841193 | 3.58161092 | 0.66799605 |
| 6 | 3.38384581 | 4.23009157 | -0.52660024 |
| 6 | 2.34467363 | 3.73224759 | -1.30332088 |
| 6 | 1.63884676 | 2.59416986 | -0.89604008 |
| 6 | 0.43596336 | 2.16782761 | -1.68750942 |
| 1 | 3.93410587 | 5.11843061 | -0.83626026 |
| 6 | 3.44010305 | 1.76111507 | 2.44597888 |
| 6 | -2.46609521 | 0.57839131 | -1.02807295 |
| 6 | -3.72921538 | 1.01143885 | -0.52060223 |
| 6 | -4.54437256 | 1.76381421 | -1.37645745 |
| 6 | -4.16647911 | 2.10590100 | -2.67811608 |
| 6 | -2.93109727 | 1.68739474 | -3.15648246 |
| 6 | -2.08283091 | 0.91973215 | -2.34926963 |
| 6 | -0.78361630 | 0.42973828 | -2.93194914 |
| 1 | -4.83307695 | 2.69799495 | -3.30486488 |
| 6 | -4.18454123 | 0.66984016 | 0.91240758 |
| 6 | 4.55120754 | 2.53537369 | 3.17960858 |
| 6 | 3.97526050 | 0.33843490 | 2.16628551 |
| 6 | 2.22255182 | 1.69328213 | 3.39838672 |
| 6 | -5.57838821 | 1.24589360 | 1.22620153 |
| 6 | -3.19815421 | 1.26806784 | 1.94366205 |
| 6 | -4.27071238 | -0.86422467 | 1.09132051 |
| 1 | 1.74181175 | 1.06746542 | -3.73013997 |
| 1 | 2.54128599 | 0.54588902 | -2.21880221 |
| 1 | 0.35746881 | 2.77820969 | -2.60339141 |
| 1 | -0.48834008 | 2.33003807 | -1.11333776 |
| 1 | 4.51497269 | 3.99242091 | 1.27232766 |
| 1 | 2.05508041 | 4.23399305 | -2.22949052 |
| 1 | -0.80809075 | -0.65955299 | -3.09090853 |
| 1 | -0.63268507 | 0.89890778 | -3.91926527 |
| 1 | -5.51296139 | 2.10564661 | -1.01665711 |
| 1 | -2.61407447 | 1.94439912 | -4.16975498 |
| 1 | 4.78366280 | 2.01757407 | 4.12121773 |
| 1 | 5.48046112 | 2.58650279 | 2.59386015 |
| 1 | 4.24250841 | 3.55978441 | 3.43309665 |
| 1 | 3.21908951 | -0.26403511 | 1.65367770 |
| 1 | 4.87959909 | 0.38293034 | 1.54150403 |
| 1 | 4.24045324 | -0.15745278 | 3.11300778 |
| 1 | 1.85219193 | 2.70441794 | 3.62154675 |
| 1 | 1.40347433 | 1.10930169 | 2.96593881 |
| 1 | 2.52155113 | 1.22706676 | 4.34973240 |
| 1 | -5.85402584 | 0.97078723 | 2.25422883 |

| | | | |
|---|---|---|---|
| 1 | -6.35304832 | 0.84348696 | 0.55758089 |
| 1 | -5.59765577 | 2.34353042 | 1.16187656 |
| 1 | -2.19092369 | 0.85420448 | 1.82784975 |
| 1 | -3.14291716 | 2.36143494 | 1.83760393 |
| 1 | -3.54733706 | 1.04559004 | 2.96343541 |
| 1 | -5.00593424 | -1.29596341 | 0.39714864 |
| 1 | -3.30071449 | -1.33816290 | 0.90873080 |
| 1 | -4.59145784 | -1.10142148 | 2.11729789 |
| 1 | 1.62846005 | -0.63607556 | -3.19990492 |
| 8 | 1.27921009 | -3.65447211 | 1.12070215 |
| 6 | 2.47366285 | -3.04285693 | 1.66324854 |
| 1 | 3.00235319 | -2.49609351 | 0.87192094 |
| 8 | 0.21758367 | -1.63406718 | 1.26423252 |
| 8 | 0.82626051 | -2.16076350 | -0.93015641 |
| 1 | 3.08405328 | -3.87685776 | 2.02276564 |
| 1 | 2.21788359 | -2.36076427 | 2.48068023 |
| 31 | 0.19786619 | -0.34300441 | -0.26844865 |
| 6 | 0.30125758 | -2.80235362 | 0.72895628 |
| 6 | -1.00130737 | -3.60887170 | 0.43282291 |
| 8 | -1.64939320 | -3.34793711 | -0.84831905 |
| 6 | -0.99355769 | -3.35818768 | -2.04006243 |
| 6 | 0.48346114 | -2.95292425 | -2.06185079 |
| 8 | -1.60633552 | -3.58460474 | -3.06319118 |
| 6 | 1.42648077 | -4.14985323 | -2.22622466 |
| 6 | -0.91021985 | -5.11556292 | 0.64395070 |
| 1 | -1.72530222 | -3.18799877 | 1.14470768 |
| 1 | 0.57022333 | -2.33837271 | -2.97276402 |
| 1 | 2.44867396 | -3.78602624 | -2.39409947 |
| 1 | 1.11737752 | -4.74952936 | -3.09240723 |
| 1 | 1.43243587 | -4.78531981 | -1.33300400 |
| 1 | -0.60907358 | -5.33809853 | 1.67479300 |
| 1 | -0.18792932 | -5.58841228 | -0.03251175 |
| 1 | -1.90059602 | -5.55251694 | 0.46346211 |

Electronic energy = -3673.015464 a.u.
DFT-D3(BJ) dispersion correction = -0.120835 a.u.
Thermal free energy = 0.586783 a.u.
Gibbs free energy = -3672.549517 a.u.
Number of imaginary frequencies = 1.

**TS2 ax LA isomer/conformer 4**

| Atomic N. | X | Y | Z |
|---|---|---|---|
| 7 | 0.25220561 | 1.35015988 | 2.00628734 |
| 8 | -0.91734773 | 0.14713909 | -0.53831029 |
| 8 | 2.09800196 | 0.15361300 | 0.06888691 |
| 6 | -0.39292100 | 1.12616313 | 3.31884384 |
| 6 | -1.99183476 | 0.91291463 | -0.22603051 |
| 6 | -3.22205544 | 0.74617207 | -0.93040854 |
| 6 | -4.32426453 | 1.48719740 | -0.47805768 |
| 6 | -4.24636841 | 2.40424013 | 0.57360870 |
| 6 | -3.01133466 | 2.65127563 | 1.16283274 |
| 6 | -1.88047719 | 1.93423915 | 0.75517613 |
| 6 | -0.51879054 | 2.37342906 | 1.22063100 |

| | | | |
|---|---|---|---|
| 1 | -5.13521147 | 2.94704866 | 0.89454365 |
| 6 | -3.32974124 | -0.13883054 | -2.18907642 |
| 6 | 2.60075736 | 1.40230620 | -0.01387122 |
| 6 | 3.36567187 | 1.79921293 | -1.15178752 |
| 6 | 3.88795972 | 3.10043406 | -1.14679384 |
| 6 | 3.68439865 | 3.99993324 | -0.09499584 |
| 6 | 2.93542981 | 3.60053182 | 1.00603628 |
| 6 | 2.40339303 | 2.30726004 | 1.05522180 |
| 6 | 1.65568948 | 1.84708428 | 2.27069044 |
| 1 | 4.10812855 | 5.00287437 | -0.14404196 |
| 6 | 3.60720873 | 0.84703070 | -2.33940935 |
| 6 | -4.73768759 | -0.08851344 | -2.81127906 |
| 6 | -3.01651096 | -1.61506498 | -1.87075090 |
| 6 | -2.33128190 | 0.37959939 | -3.25270557 |
| 6 | 4.48157978 | 1.49678731 | -3.42829347 |
| 6 | 2.25904274 | 0.46448824 | -2.99428201 |
| 6 | 4.33704424 | -0.43091390 | -1.86225331 |
| 1 | -0.38554639 | 2.06245470 | 3.90155125 |
| 1 | -1.43081522 | 0.81106514 | 3.17904401 |
| 1 | -0.60836625 | 3.28638864 | 1.83470774 |
| 1 | 0.10840981 | 2.62017870 | 0.35193485 |
| 1 | -5.28504181 | 1.35362256 | -0.97155023 |
| 1 | -2.90508890 | 3.42615414 | 1.92555892 |
| 1 | 2.17725587 | 1.01136696 | 2.76517892 |
| 1 | 1.58867550 | 2.67514539 | 2.99677467 |
| 1 | 4.47440481 | 3.43560815 | -2.00033116 |
| 1 | 2.76430869 | 4.28446579 | 1.84050608 |
| 1 | -4.74852276 | -0.71153045 | -3.71685100 |
| 1 | -5.50689220 | -0.48243377 | -2.13091207 |
| 1 | -5.02424622 | 0.93042701 | -3.10809326 |
| 1 | -1.98930776 | -1.72650802 | -1.51155412 |
| 1 | -3.70922828 | -2.00677085 | -1.11067700 |
| 1 | -3.13469338 | -2.22555017 | -2.77900147 |
| 1 | -2.56277609 | 1.41843128 | -3.52872729 |
| 1 | -1.29838526 | 0.33650658 | -2.88821864 |
| 1 | -2.40277791 | -0.23622286 | -4.16206503 |
| 1 | 4.62745857 | 0.77676815 | -4.24602938 |
| 1 | 5.47643852 | 1.77368891 | -3.05011058 |
| 1 | 4.01074696 | 2.39251757 | -3.85854936 |
| 1 | 1.60158789 | -0.05761108 | -2.29132032 |
| 1 | 1.73683071 | 1.35994506 | -3.36125398 |
| 1 | 2.43895292 | -0.19873618 | -3.85401607 |
| 1 | 5.30509806 | -0.17898345 | -1.40461802 |
| 1 | 3.73299050 | -0.98250967 | -1.13400543 |
| 1 | 4.53126955 | -1.08986390 | -2.72208023 |
| 1 | 0.15657751 | 0.35530761 | 3.87060094 |
| 8 | 1.86109948 | -3.30166602 | 2.00527167 |
| 6 | 2.98028016 | -2.38839221 | 2.06639862 |
| 1 | 2.72628260 | -1.54419732 | 2.72336459 |
| 8 | 1.04041326 | -2.23962522 | 0.13894534 |
| 8 | -0.19807319 | -1.68739557 | 2.05758357 |
| 1 | 3.80050802 | -2.96328020 | 2.50643229 |
| 1 | 3.23882318 | -2.01946020 | 1.06810129 |

| | | | |
|---|---|---|---|
| 31 | 0.39374089 | -0.35437509 | 0.69856393 |
| 6 | 0.86049545 | -3.00159740 | 1.14569902 |
| 6 | -0.13248709 | -4.16857529 | 1.13919365 |
| 8 | -0.67542577 | -4.44738531 | 2.46544719 |
| 6 | -1.26426089 | -3.52550769 | 3.27847290 |
| 6 | -1.26122415 | -2.02283120 | 2.94057250 |
| 8 | -1.80516171 | -3.91206479 | 4.29378700 |
| 6 | -2.65862560 | -1.62363589 | 2.44370294 |
| 6 | -1.20649254 | -4.07272768 | 0.07593066 |
| 1 | 0.47472540 | -5.06831121 | 0.95303947 |
| 1 | -1.09474897 | -1.54560435 | 3.92164612 |
| 1 | -2.70743823 | -0.54532599 | 2.24556208 |
| 1 | -3.39854217 | -1.87222207 | 3.21527481 |
| 1 | -2.92770052 | -2.14683890 | 1.51876032 |
| 1 | -0.73868525 | -4.07711792 | -0.91665262 |
| 1 | -1.79245555 | -3.15374184 | 0.16350517 |
| 1 | -1.87504900 | -4.93925476 | 0.15509385 |

Electronic energy = -3673.010995 a.u.
DFT-D3(BJ) dispersion correction = -0.123166 a.u.
Thermal free energy = 0.587502 a.u.
Gibbs free energy = -3672.546659 a.u.
Number of imaginary frequencies = 1.

**Int2 ax LA isomer/conformer 1**

| Atomic N. | X | Y | Z |
|---|---|---|---|
| 7 | 0.48972341 | 1.19242068 | -2.30517960 |
| 8 | 1.46205237 | 0.56367846 | 0.38570173 |
| 8 | -1.60624881 | -0.04812037 | -0.58445353 |
| 6 | 1.54636561 | 0.83208841 | -3.28165834 |
| 6 | 2.38315238 | 1.50763634 | 0.08302942 |
| 6 | 3.60884539 | 1.54292959 | 0.81431582 |
| 6 | 4.52583326 | 2.55203507 | 0.48849010 |
| 6 | 4.28508268 | 3.50241531 | -0.50755527 |
| 6 | 3.08565619 | 3.45967335 | -1.20932391 |
| 6 | 2.13052987 | 2.48023642 | -0.91624150 |
| 6 | 0.80943769 | 2.49586405 | -1.63304729 |
| 1 | 5.03140309 | 4.26536329 | -0.72870752 |
| 6 | 3.91859804 | 0.51684402 | 1.92241390 |
| 6 | -2.31954074 | 1.03945595 | -0.97167947 |
| 6 | -3.43830392 | 1.47273650 | -0.19706946 |
| 6 | -4.15329261 | 2.58602571 | -0.66148776 |
| 6 | -3.81172783 | 3.27511254 | -1.82770749 |
| 6 | -2.71730747 | 2.84744779 | -2.56989752 |
| 6 | -1.97552206 | 1.73445965 | -2.15841403 |
| 6 | -0.83241961 | 1.26026612 | -3.01425161 |
| 1 | -4.39573841 | 4.13880383 | -2.14507846 |
| 6 | -3.85647829 | 0.75559398 | 1.10175567 |
| 6 | 5.28849280 | 0.77269479 | 2.57878048 |
| 6 | 3.95290336 | -0.91150498 | 1.32934689 |
| 6 | 2.85067014 | 0.60444204 | 3.03888741 |
| 6 | -5.08303947 | 1.41386313 | 1.76155683 |
| 6 | -2.70396483 | 0.80215986 | 2.13217013 |

| | | | |
|---|---|---|---|
| 6 | -4.23924111 | -0.70922797 | 0.78834056 |
| 1 | 1.60852879 | 1.60111390 | -4.06988701 |
| 1 | 2.51261072 | 0.76375066 | -2.77156473 |
| 1 | 0.80230127 | 3.29835165 | -2.39105244 |
| 1 | -0.01655495 | 2.69641380 | -0.93440266 |
| 1 | 5.47037142 | 2.60083268 | 1.02707905 |
| 1 | 2.87196334 | 4.19582817 | -1.98762722 |
| 1 | -1.02128378 | 0.24376024 | -3.39645634 |
| 1 | -0.72700133 | 1.92807930 | -3.88656927 |
| 1 | -5.00920483 | 2.93871959 | -0.08927988 |
| 1 | -2.42981309 | 3.37040042 | -3.48471714 |
| 1 | 5.45502672 | 0.01972263 | 3.36246502 |
| 1 | 6.11560020 | 0.68770791 | 1.85930211 |
| 1 | 5.34116058 | 1.76246035 | 3.05492881 |
| 1 | 2.99287393 | -1.18857344 | 0.88119300 |
| 1 | 4.73182252 | -0.99226482 | 0.55740646 |
| 1 | 4.19183049 | -1.63811442 | 2.12183298 |
| 1 | 2.84506337 | 1.60732142 | 3.49040999 |
| 1 | 1.84886941 | 0.39041744 | 2.65104525 |
| 1 | 3.08732266 | -0.11921694 | 3.83509238 |
| 1 | -5.33576152 | 0.85967888 | 2.67689460 |
| 1 | -5.96733011 | 1.39314732 | 1.10858931 |
| 1 | -4.88736644 | 2.45635313 | 2.05069661 |
| 1 | -1.79768587 | 0.31889500 | 1.75343821 |
| 1 | -2.45838779 | 1.84294779 | 2.38826372 |
| 1 | -3.01258684 | 0.29290828 | 3.05849146 |
| 1 | -5.10901324 | -0.74669117 | 0.11644313 |
| 1 | -3.41046966 | -1.23894312 | 0.30686622 |
| 1 | -4.50773836 | -1.23542448 | 1.71751064 |
| 1 | 1.31281020 | -0.14116806 | -3.72378072 |
| 8 | -0.29038750 | -3.32554012 | 2.52620257 |
| 6 | 0.54966141 | -2.66906945 | 3.51802509 |
| 1 | 1.56159266 | -2.53761646 | 3.11860620 |
| 8 | 0.05454181 | -1.56265963 | 1.16920879 |
| 8 | 0.78059708 | -1.76695002 | -1.68953284 |
| 1 | 0.54836669 | -3.34355024 | 4.37827902 |
| 1 | 0.12838539 | -1.69141820 | 3.77915130 |
| 31 | 0.25456132 | -0.25260209 | -0.77241530 |
| 8 | -0.33367184 | -4.33788957 | -0.33098316 |
| 6 | 0.20184618 | -4.07611138 | -1.58341936 |
| 6 | -0.09318806 | -2.72482994 | -2.27293860 |
| 6 | -0.44285203 | -2.66746326 | 1.37330366 |
| 6 | -1.26205719 | -3.44905165 | 0.34317838 |
| 8 | 0.91868697 | -4.92813883 | -2.05443668 |
| 6 | -2.36891167 | -4.30333853 | 0.93979164 |
| 6 | 0.16259927 | -2.86635088 | -3.77136595 |
| 1 | -1.15757958 | -2.45564945 | -2.13193281 |
| 1 | -1.67443659 | -2.70401999 | -0.34766559 |
| 1 | -2.92787936 | -4.78931797 | 0.12998657 |
| 1 | -3.06413907 | -3.68112355 | 1.51919737 |
| 1 | -1.95077268 | -5.07480814 | 1.59594295 |
| 1 | -0.45538007 | -3.66854179 | -4.19844789 |
| 1 | 1.21609452 | -3.11072559 | -3.95076706 |

```
   1    -0.08955661  -1.92704095  -4.28261608
Electronic energy = -3673.039346 a.u.
DFT-D3(BJ) dispersion correction = -0.119690 a.u.
Thermal free energy = 0.584096 a.u.
Gibbs free energy = -3672.574940 a.u.
Number of imaginary frequencies = 0.
```

## Initiation step with equatorial LA addition

**TS1_eq LA isomer/conformer 1**

| Atomic N. | X | Y | Z |
|---|---|---|---|
| 7 | -0.85166550 | 1.38781500 | 1.82185507 |
| 8 | -1.06658757 | -1.23469067 | 0.52863073 |
| 8 | 0.12862366 | 1.33237994 | -0.98078263 |
| 6 | -0.49368417 | 1.22557318 | 3.25272727 |
| 6 | -2.06726480 | -1.41720092 | 1.41591597 |
| 6 | -2.50258899 | -2.74077272 | 1.72721314 |
| 6 | -3.54281759 | -2.87445688 | 2.65757871 |
| 6 | -4.15497017 | -1.77855802 | 3.27339506 |
| 6 | -3.73035407 | -0.49384731 | 2.95136833 |
| 6 | -2.70202780 | -0.30463710 | 2.02221656 |
| 6 | -2.31023622 | 1.08556783 | 1.60923803 |
| 1 | -4.95815182 | -1.93368721 | 3.99360919 |
| 6 | -1.86342943 | -3.97478652 | 1.06084526 |
| 6 | -0.62971157 | 2.44181299 | -1.11088967 |
| 6 | -1.02091432 | 2.88220739 | -2.41216278 |
| 6 | -1.80589271 | 4.04026699 | -2.49405622 |
| 6 | -2.21095514 | 4.76281595 | -1.36814618 |
| 6 | -1.81623054 | 4.32955456 | -0.10771518 |
| 6 | -1.02072144 | 3.18611193 | 0.03096492 |
| 6 | -0.53267592 | 2.80096769 | 1.40186667 |
| 1 | -2.82760406 | 5.65428925 | -1.48135638 |
| 6 | -0.59400749 | 2.12063098 | -3.68221164 |
| 6 | -2.50110126 | -5.28939724 | 1.54790318 |
| 6 | -0.35372889 | -4.04417896 | 1.39399719 |
| 6 | -2.06246829 | -3.90620089 | -0.47210065 |
| 6 | -1.11523402 | 2.79465938 | -4.96537352 |
| 6 | -1.16267073 | 0.68221062 | -3.65748167 |
| 6 | 0.95013130 | 2.08511972 | -3.77917957 |
| 1 | -1.12620020 | 1.88491905 | 3.86964178 |
| 1 | -0.65747738 | 0.18776652 | 3.56110191 |
| 1 | -2.90299344 | 1.82879567 | 2.16899920 |
| 1 | -2.50613427 | 1.24718356 | 0.53893137 |
| 1 | -3.89283562 | -3.87155509 | 2.91928124 |
| 1 | -4.20391941 | 0.37798560 | 3.40872431 |
| 1 | 0.56267697 | 2.88705730 | 1.47296619 |
| 1 | -0.97442055 | 3.48312664 | 2.14784980 |
| 1 | -2.12139010 | 4.39566708 | -3.47333050 |
| 1 | -2.11290145 | 4.88352108 | 0.78589576 |
| 1 | -2.01541948 | -6.13316679 | 1.03709757 |
| 1 | -2.37039709 | -5.43777990 | 2.62969923 |

| | | | |
|---|---|---|---|
| 1 | -3.57507873 | -5.33809948 | 1.31759834 |
| 1 | 0.18001871 | -3.16355443 | 1.01920080 |
| 1 | -0.20179331 | -4.11499310 | 2.48154569 |
| 1 | 0.08734455 | -4.94181538 | 0.93379408 |
| 1 | -3.13314748 | -3.91622210 | -0.72279447 |
| 1 | -1.61729074 | -2.99785566 | -0.89302593 |
| 1 | -1.59545183 | -4.78139877 | -0.94948411 |
| 1 | -0.77423751 | 2.21669555 | -5.83628845 |
| 1 | -0.73393905 | 3.81974435 | -5.07874441 |
| 1 | -2.21377516 | 2.82574916 | -4.99998331 |
| 1 | -0.81253994 | 0.12393024 | -2.78305745 |
| 1 | -2.26193237 | 0.70285279 | -3.63627219 |
| 1 | -0.85276306 | 0.14160228 | -4.56490803 |
| 1 | 1.35298049 | 3.10443354 | -3.87156129 |
| 1 | 1.39400792 | 1.61605334 | -2.89405251 |
| 1 | 1.25629210 | 1.51841974 | -4.67198753 |
| 1 | 0.55917436 | 1.48722005 | 3.39209056 |
| 8 | 1.63771832 | -0.97982407 | -0.14785340 |
| 6 | 1.71523154 | -1.49680436 | -1.46849513 |
| 1 | 0.79244548 | -2.04535818 | -1.72243524 |
| 8 | 1.89053476 | 0.58438092 | 1.66125345 |
| 1 | 1.86553681 | -0.70302469 | -2.22004414 |
| 1 | 2.55151772 | -2.21514750 | -1.52889287 |
| 31 | 0.20126544 | 0.15308768 | 0.47476494 |
| 8 | 3.48172927 | 1.03658617 | 0.16515338 |
| 6 | 2.89309859 | 0.17200626 | 1.01328480 |
| 6 | 3.78681064 | -0.98424149 | 1.47717214 |
| 8 | 5.12142754 | -0.44846848 | 1.77481794 |
| 6 | 5.71087837 | 0.27586019 | 0.77983367 |
| 6 | 4.77294922 | 0.61984980 | -0.38436738 |
| 8 | 6.86936092 | 0.61286169 | 0.85417646 |
| 6 | 5.28966713 | 1.74500883 | -1.25452757 |
| 6 | 3.27822399 | -1.67991757 | 2.71955800 |
| 1 | 3.89234161 | -1.70151246 | 0.65071344 |
| 1 | 4.61245346 | -0.29235888 | -0.98615229 |
| 1 | 4.57628775 | 1.95056081 | -2.06184292 |
| 1 | 6.25419617 | 1.45604384 | -1.68788791 |
| 1 | 5.43964720 | 2.65417790 | -0.66030860 |
| 1 | 2.30344248 | -2.13475132 | 2.50567222 |
| 1 | 3.16371965 | -0.96736437 | 3.54478002 |
| 1 | 3.98199868 | -2.46750212 | 3.01482987 |

Electronic energy = -3673.030161 a.u.
DFT-D3(BJ) dispersion correction = -0.117784 a.u.
Thermal free energy = 0.584026 a.u.
Gibbs free energy = -3672.563919 a.u.
Number of imaginary frequencies = 1.

**TS1_eq_LA isomer/conformer 2**

| Atomic N. | X | Y | Z |
|---|---|---|---|
| 7 | -0.50164080 | 2.28206468 | -0.73304069 |
| 8 | -0.84355366 | 0.06840651 | 1.15196908 |
| 8 | 0.06997266 | -0.32622176 | -1.94433653 |

| | | | |
|---|---|---|---|
| 6 | -0.02664193 | 3.44224882 | 0.06012286 |
| 6 | -1.79986429 | 0.86712629 | 1.67267275 |
| 6 | -2.24119878 | 0.65478086 | 3.01515460 |
| 6 | -3.23953700 | 1.50509381 | 3.50996184 |
| 6 | -3.80310965 | 2.53539777 | 2.75189614 |
| 6 | -3.36523724 | 2.73264670 | 1.44714165 |
| 6 | -2.37945914 | 1.90438020 | 0.89966530 |
| 6 | -1.98037374 | 2.08368444 | -0.53793585 |
| 1 | -4.57533503 | 3.17326331 | 3.18174434 |
| 6 | -1.64461875 | -0.46271366 | 3.89266992 |
| 6 | -0.63481516 | 0.15759097 | -2.98590422 |
| 6 | -1.18586552 | -0.74621695 | -3.94539928 |
| 6 | -1.89155483 | -0.19282898 | -5.02230072 |
| 6 | -2.07909465 | 1.18381667 | -5.17909431 |
| 6 | -1.54475999 | 2.05250573 | -4.23440742 |
| 6 | -0.81612921 | 1.55414784 | -3.14811182 |
| 6 | -0.18422315 | 2.52483606 | -2.18666720 |
| 1 | -2.64254332 | 1.56671584 | -6.02986336 |
| 6 | -1.01862729 | -2.27196932 | -3.80220222 |
| 6 | -2.28991508 | -0.51035112 | 5.29052877 |
| 6 | -0.13046134 | -0.21705133 | 4.09566593 |
| 6 | -1.88145530 | -1.84155309 | 3.23224211 |
| 6 | -1.67871785 | -3.03646684 | -4.96520567 |
| 6 | -1.68997121 | -2.75575519 | -2.49493957 |
| 6 | 0.48187131 | -2.64968848 | -3.79776263 |
| 1 | -0.60143894 | 4.34114790 | -0.21765386 |
| 1 | -0.16674495 | 3.24656296 | 1.12818360 |
| 1 | -2.50700569 | 2.95231867 | -0.96810520 |
| 1 | -2.25821257 | 1.20471466 | -1.13812804 |
| 1 | -3.59349442 | 1.36519015 | 4.52978754 |
| 1 | -3.79489064 | 3.52695584 | 0.83232349 |
| 1 | 0.91504639 | 2.50033879 | -2.25647354 |
| 1 | -0.51246977 | 3.54741096 | -2.43946600 |
| 1 | -2.32131100 | -0.85795003 | -5.76915073 |
| 1 | -1.68010414 | 3.13188171 | -4.33576727 |
| 1 | -1.83132982 | -1.32596242 | 5.86790562 |
| 1 | -2.13274336 | 0.42164248 | 5.85262918 |
| 1 | -3.37023735 | -0.70848858 | 5.24001646 |
| 1 | 0.40257654 | -0.18268523 | 3.13932657 |
| 1 | 0.03704825 | 0.73466915 | 4.62149906 |
| 1 | 0.29981220 | -1.02354228 | 4.70970583 |
| 1 | -2.95869422 | -2.04314899 | 3.14078498 |
| 1 | -1.43476295 | -1.89213288 | 2.23377490 |
| 1 | -1.44158971 | -2.63547969 | 3.85521674 |
| 1 | -1.52712882 | -4.11490059 | -4.81501770 |
| 1 | -1.23702741 | -2.77250886 | -5.93707943 |
| 1 | -2.76281404 | -2.85963225 | -5.01578665 |
| 1 | -1.24927974 | -2.27616072 | -1.61417341 |
| 1 | -2.76709747 | -2.53417635 | -2.50799656 |
| 1 | -1.57000482 | -3.84521270 | -2.39411497 |
| 1 | 0.96081942 | -2.34024024 | -4.73839092 |
| 1 | 1.01005876 | -2.17604136 | -2.96341443 |
| 1 | 0.59031957 | -3.74127269 | -3.70705700 |

| | | | |
|---|---|---|---|
| 1 | 1.03609383 | 3.60418773 | -0.14099938 |
| 8 | 1.72253025 | -0.82416946 | 0.26928666 |
| 6 | 1.50000930 | -2.11775422 | 0.81814843 |
| 1 | 2.40838480 | -2.72547269 | 0.68545628 |
| 8 | 2.19447231 | 1.51534986 | -0.12578981 |
| 1 | 1.24775243 | -2.07717419 | 1.89104247 |
| 1 | 0.67589307 | -2.62643671 | 0.29012892 |
| 31 | 0.36676037 | 0.41802704 | -0.24673931 |
| 8 | 3.20853448 | 0.91540569 | 1.76688099 |
| 6 | 3.05251527 | 0.78706825 | 0.43677810 |
| 6 | 4.23645067 | 0.18444891 | -0.30914384 |
| 8 | 4.70228434 | -1.02633512 | 0.35192254 |
| 6 | 4.79344893 | -1.00583756 | 1.70811582 |
| 6 | 4.39896584 | 0.31639943 | 2.37520957 |
| 8 | 5.20209360 | -1.96891725 | 2.31517959 |
| 6 | 4.12877893 | 0.17174676 | 3.85857058 |
| 6 | 3.96525645 | -0.14857920 | -1.76084471 |
| 1 | 5.04828835 | 0.93731958 | -0.24102338 |
| 1 | 5.23368216 | 1.02226901 | 2.20429730 |
| 1 | 3.87602162 | 1.14854145 | 4.28852415 |
| 1 | 5.02147865 | -0.22347595 | 4.35636187 |
| 1 | 3.29894543 | -0.52406454 | 4.03039742 |
| 1 | 3.68185306 | 0.76203763 | -2.30197239 |
| 1 | 3.14611840 | -0.87223333 | -1.83154225 |
| 1 | 4.87294722 | -0.56353796 | -2.21588755 |

Electronic energy = -3673.028016 a.u.
DFT-D3(BJ) dispersion correction = -0.118961 a.u.
Thermal free energy = 0.583834 a.u.
Gibbs free energy = -3672.563143 a.u.
Number of imaginary frequencies = 1.

**TS1_eq_LA isomer/conformer 3**

| Atomic N. | X | Y | Z |
|---|---|---|---|
| 7 | -0.63867962 | 2.35204625 | 0.03793024 |
| 8 | -1.17746854 | -0.23847388 | 1.29763746 |
| 8 | 0.11001519 | 0.01950578 | -1.63110113 |
| 6 | -0.20316280 | 3.33342195 | 1.06233752 |
| 6 | -2.11592960 | 0.46139172 | 1.96908367 |
| 6 | -2.62781978 | -0.04123778 | 3.20356846 |
| 6 | -3.60359216 | 0.72554898 | 3.85575843 |
| 6 | -4.08065319 | 1.93802285 | 3.34834957 |
| 6 | -3.57869554 | 2.41319156 | 2.14134216 |
| 6 | -2.61089993 | 1.68151116 | 1.44522119 |
| 6 | -2.13009953 | 2.15669298 | 0.10405762 |
| 1 | -4.83867884 | 2.49955702 | 3.89434123 |
| 6 | -2.13316727 | -1.37526119 | 3.79528904 |
| 6 | -0.55205965 | 0.68575215 | -2.59963751 |
| 6 | -1.00644326 | -0.01707092 | -3.75711107 |
| 6 | -1.68310988 | 0.72145265 | -4.73725319 |
| 6 | -1.92589295 | 2.09315205 | -4.62006044 |
| 6 | -1.47456288 | 2.76692080 | -3.49093246 |
| 6 | -0.78046662 | 2.08071446 | -2.48755670 |

| | | | |
|---|---|---|---|
| 6 | -0.22598666 | 2.85693979 | -1.32287610 |
| 1 | -2.46361780 | 2.62371302 | -5.40567493 |
| 6 | -0.76396912 | -1.52967536 | -3.92675185 |
| 6 | -2.83188987 | -1.71198177 | 5.12614155 |
| 6 | -0.61365062 | -1.30375850 | 4.07961607 |
| 6 | -2.43643713 | -2.53082776 | 2.81215906 |
| 6 | -1.32170415 | -2.05954599 | -5.26116180 |
| 6 | -1.46888947 | -2.31096601 | -2.79271746 |
| 6 | 0.75483048 | -1.82642257 | -3.91597533 |
| 1 | -0.74295110 | 4.28361988 | 0.91701716 |
| 1 | -0.42530611 | 2.94976830 | 2.06368899 |
| 1 | -2.61560416 | 3.11223650 | -0.15683150 |
| 1 | -2.38262916 | 1.43286669 | -0.68467695 |
| 1 | -4.00968838 | 0.36922356 | 4.80086279 |
| 1 | -3.94410682 | 3.35241079 | 1.71944356 |
| 1 | 0.87503910 | 2.83880782 | -1.31965995 |
| 1 | -0.54762119 | 3.90902376 | -1.40414298 |
| 1 | -2.04306006 | 0.21034570 | -5.62837791 |
| 1 | -1.64735794 | 3.84003854 | -3.38093781 |
| 1 | -2.44783998 | -2.67260814 | 5.49818134 |
| 1 | -2.63626862 | -0.95553756 | 5.90006733 |
| 1 | -3.92013288 | -1.81493950 | 5.00745630 |
| 1 | -0.03930257 | -1.11376846 | 3.16605973 |
| 1 | -0.39525005 | -0.50543851 | 4.80467653 |
| 1 | -0.27133799 | -2.25540233 | 4.51431942 |
| 1 | -3.52071452 | -2.63033056 | 2.65749693 |
| 1 | -1.96333599 | -2.36358833 | 1.83829486 |
| 1 | -2.06519032 | -3.48118544 | 3.22540593 |
| 1 | -1.11442387 | -3.13688946 | -5.33235168 |
| 1 | -0.85132152 | -1.57394433 | -6.12839794 |
| 1 | -2.41071033 | -1.92771137 | -5.33634615 |
| 1 | -1.10279727 | -2.01024389 | -1.80549908 |
| 1 | -2.55485964 | -2.14090514 | -2.82481933 |
| 1 | -1.29243469 | -3.39039946 | -2.91618729 |
| 1 | 1.25086045 | -1.32606101 | -4.76066637 |
| 1 | 1.22042906 | -1.48212969 | -2.98584175 |
| 1 | 0.92509800 | -2.90922666 | -4.01727343 |
| 1 | 0.87310475 | 3.50063896 | 0.96452647 |
| 8 | 1.49658334 | -0.88839006 | 0.72608781 |
| 6 | 1.34880209 | -2.27789593 | 0.44666886 |
| 1 | 0.37551576 | -2.64031410 | 0.81720471 |
| 8 | 2.02391028 | 1.47986162 | 0.61382288 |
| 1 | 1.41168571 | -2.49437785 | -0.63364857 |
| 1 | 2.13719416 | -2.83716226 | 0.97443175 |
| 31 | 0.19923800 | 0.42358816 | 0.19763042 |
| 8 | 3.49271870 | 0.40630740 | -0.66378891 |
| 6 | 2.93681002 | 0.61352301 | 0.53631085 |
| 6 | 3.73539710 | 0.26237914 | 1.77538717 |
| 8 | 4.51069117 | -0.96157753 | 1.64873755 |
| 6 | 4.90029430 | -1.41094029 | 0.43251407 |
| 6 | 4.44316530 | -0.69072664 | -0.83434087 |
| 8 | 5.59569836 | -2.39934421 | 0.34255958 |
| 6 | 5.62995386 | -0.16522673 | -1.63476610 |

| | | | |
|---|---|---|---|
| 6 | 4.65798235 | 1.41723168 | 2.16930985 |
| 1 | 3.01221180 | 0.05545177 | 2.57183528 |
| 1 | 3.90370059 | -1.44757104 | -1.42376316 |
| 1 | 5.27292013 | 0.27323258 | -2.57450962 |
| 1 | 6.30919313 | -0.99550033 | -1.85947740 |
| 1 | 6.17891359 | 0.60064316 | -1.07094896 |
| 1 | 4.05912781 | 2.31072474 | 2.38638234 |
| 1 | 5.37067938 | 1.65574837 | 1.36805427 |
| 1 | 5.22000790 | 1.13735151 | 3.06879544 |

Electronic energy = -3673.027220 a.u.
DFT-D3(BJ) dispersion correction = -0.117913 a.u.
Thermal free energy = 0.583394 a.u.
Gibbs free energy = -3672.561738 a.u.
Number of imaginary frequencies = 1.

**Int1a LA isomer/conformer 1**

| Atomic N. | X | Y | Z |
|---|---|---|---|
| 7 | -0.51515709 | 2.47888814 | -0.84658116 |
| 8 | -0.95259963 | 0.12130814 | 0.93231853 |
| 8 | 0.14423066 | -0.18839325 | -2.00317960 |
| 6 | 0.11786464 | 3.61648342 | -0.13466888 |
| 6 | -1.79133656 | 0.97339816 | 1.57537206 |
| 6 | -2.21590464 | 0.67086600 | 2.90419991 |
| 6 | -3.07579862 | 1.58427455 | 3.53037073 |
| 6 | -3.52467666 | 2.75297501 | 2.90994547 |
| 6 | -3.12626511 | 3.02245556 | 1.60596865 |
| 6 | -2.27928350 | 2.13828728 | 0.92740739 |
| 6 | -1.98152847 | 2.38732671 | -0.52673651 |
| 1 | -4.18687278 | 3.43635892 | 3.44134935 |
| 6 | -1.75458004 | -0.60656587 | 3.63383501 |
| 6 | -0.69359674 | 0.21092489 | -2.98714901 |
| 6 | -1.27643558 | -0.76147363 | -3.85367492 |
| 6 | -2.11775186 | -0.29357923 | -4.87240474 |
| 6 | -2.40241071 | 1.06260397 | -5.05821805 |
| 6 | -1.82816109 | 2.00001752 | -4.20761516 |
| 6 | -0.96825227 | 1.58877931 | -3.18205319 |
| 6 | -0.29168192 | 2.63086532 | -2.33109023 |
| 1 | -3.06874232 | 1.37696709 | -5.86132799 |
| 6 | -0.99878695 | -2.26762917 | -3.67739882 |
| 6 | -2.40505292 | -0.74612844 | 5.02337465 |
| 6 | -0.22384397 | -0.56452883 | 3.84418724 |
| 6 | -2.14868457 | -1.86221657 | 2.81973364 |
| 6 | -1.71942169 | -3.11455679 | -4.74330892 |
| 6 | -1.50640280 | -2.74079235 | -2.29445771 |
| 6 | 0.51628257 | -2.55222108 | -3.81125840 |
| 1 | -0.33727966 | 4.56467636 | -0.46493292 |
| 1 | -0.03140380 | 3.50267473 | 0.94429899 |
| 1 | -2.47005951 | 3.32025242 | -0.85567676 |
| 1 | -2.37642349 | 1.57093698 | -1.14987937 |
| 1 | -3.40914980 | 1.38184445 | 4.54640865 |
| 1 | -3.48581972 | 3.91578994 | 1.09022301 |
| 1 | 0.80177203 | 2.60398495 | -2.46759840 |

| | | | |
|---|---|---|---|
| 1 | -0.64281195 | 3.63308157 | -2.62946222 |
| 1 | -2.57832589 | -1.01204430 | -5.54796069 |
| 1 | -2.03289555 | 3.06507667 | -4.33796374 |
| 1 | -2.05329526 | -1.67794403 | 5.48875882 |
| 1 | -2.13230184 | 0.08001109 | 5.69577660 |
| 1 | -3.50174213 | -0.79945313 | 4.96354556 |
| 1 | 0.31426897 | -0.47391495 | 2.89572639 |
| 1 | 0.05511972 | 0.28951202 | 4.47867896 |
| 1 | 0.10980672 | -1.48390923 | 4.35009644 |
| 1 | -3.24065569 | -1.91844320 | 2.70131952 |
| 1 | -1.69286134 | -1.85830827 | 1.82406275 |
| 1 | -1.82136223 | -2.76739438 | 3.35374955 |
| 1 | -1.48413911 | -4.17513622 | -4.57531831 |
| 1 | -1.39372059 | -2.86113656 | -5.76255506 |
| 1 | -2.81239450 | -3.00693018 | -4.68969945 |
| 1 | -1.00982320 | -2.20671720 | -1.47721213 |
| 1 | -2.59046500 | -2.58079277 | -2.20339823 |
| 1 | -1.31287877 | -3.81788434 | -2.17572783 |
| 1 | 0.87989725 | -2.25325563 | -4.80528182 |
| 1 | 1.09365147 | -2.01382572 | -3.05206670 |
| 1 | 0.70256387 | -3.63089481 | -3.69526432 |
| 1 | 1.19198671 | 3.62250036 | -0.34815936 |
| 8 | 1.79859087 | -0.96553165 | 0.22252874 |
| 6 | 1.95647111 | -2.25649461 | 0.84996415 |
| 1 | 2.89020662 | -2.73229731 | 0.52040490 |
| 8 | 2.04274296 | 1.22392145 | 0.08549715 |
| 1 | 1.94209225 | -2.16214196 | 1.94199712 |
| 1 | 1.09991757 | -2.84986392 | 0.51295973 |
| 31 | 0.29774420 | 0.64318828 | -0.34340149 |
| 8 | 2.86097313 | 0.12116338 | 1.96350145 |
| 6 | 2.70863245 | 0.12313816 | 0.55107324 |
| 6 | 4.08419438 | 0.01024917 | -0.15175815 |
| 8 | 4.98012734 | -0.89433271 | 0.56405330 |
| 6 | 5.16044997 | -0.63079342 | 1.89408811 |
| 6 | 4.18860848 | 0.41199461 | 2.45443011 |
| 8 | 6.00188117 | -1.22516846 | 2.52955719 |
| 6 | 4.13785984 | 0.40229463 | 3.96998703 |
| 6 | 4.00798573 | -0.46842293 | -1.58826971 |
| 1 | 4.52092732 | 1.02180189 | -0.11909871 |
| 1 | 4.50599485 | 1.41227546 | 2.09993383 |
| 1 | 3.43166436 | 1.16426893 | 4.32180744 |
| 1 | 5.13404777 | 0.61407143 | 4.37582271 |
| 1 | 3.81954151 | -0.58133609 | 4.33634823 |
| 1 | 3.32734331 | 0.17711397 | -2.15647748 |
| 1 | 3.63316208 | -1.49722044 | -1.64097760 |
| 1 | 5.00443359 | -0.42892924 | -2.04539853 |

Electronic energy = -3673.042917 a.u.
DFT-D3(BJ) dispersion correction = -0.118098 a.u.
Thermal free energy = 0.586137 a.u.
Gibbs free energy = -3672.574878 a.u.
Number of imaginary frequencies = 0.

**Int1a LA isomer/conformer 2**

| Atomic N. | X | Y | Z |
|---|---|---|---|
| 7 | -0.78582798 | 1.63014484 | 1.88574279 |
| 8 | -1.08810570 | -1.00074888 | 0.50070713 |
| 8 | 0.17828037 | 1.42759823 | -0.95568026 |
| 6 | -0.24209112 | 1.57782652 | 3.26627251 |
| 6 | -1.96803270 | -1.24622998 | 1.50498957 |
| 6 | -2.39109302 | -2.58424393 | 1.75958494 |
| 6 | -3.27368147 | -2.78716965 | 2.83036834 |
| 6 | -3.75691156 | -1.74308993 | 3.62352932 |
| 6 | -3.38096920 | -0.43729214 | 3.32852182 |
| 6 | -2.50726138 | -0.17671606 | 2.26689530 |
| 6 | -2.23936680 | 1.24897081 | 1.86394885 |
| 1 | -4.43487382 | -1.95244899 | 4.45082273 |
| 6 | -1.92991919 | -3.76141196 | 0.87676065 |
| 6 | -0.70958452 | 2.43194938 | -1.15009353 |
| 6 | -1.18433987 | 2.71218521 | -2.46626356 |
| 6 | -2.09129194 | 3.77071734 | -2.61302270 |
| 6 | -2.53667539 | 4.54271440 | -1.53617924 |
| 6 | -2.05981625 | 4.26613779 | -0.26030369 |
| 6 | -1.14287559 | 3.22755912 | -0.05792446 |
| 6 | -0.57262884 | 3.01370838 | 1.31954907 |
| 1 | -3.24803074 | 5.35205509 | -1.69958364 |
| 6 | -0.71767655 | 1.88977666 | -3.68340539 |
| 6 | -2.55206518 | -5.09707386 | 1.32554067 |
| 6 | -0.39421810 | -3.92301714 | 0.94215602 |
| 6 | -2.36896319 | -3.51666102 | -0.58707077 |
| 6 | -1.35198288 | 2.38745552 | -4.99594509 |
| 6 | -1.12464073 | 0.40705613 | -3.51275211 |
| 6 | 0.81687139 | 2.01026342 | -3.84118734 |
| 1 | -0.76574652 | 2.31190374 | 3.90025327 |
| 1 | -0.38973793 | 0.57483049 | 3.68083943 |
| 1 | -2.79228249 | 1.93849115 | 2.52431546 |
| 1 | -2.58323157 | 1.42735790 | 0.83416109 |
| 1 | -3.60058269 | -3.79970141 | 3.05953954 |
| 1 | -3.77963886 | 0.39857588 | 3.90794530 |
| 1 | 0.51895493 | 3.16506764 | 1.32179607 |
| 1 | -1.01631075 | 3.74193230 | 2.01894134 |
| 1 | -2.47376732 | 4.00380155 | -3.60506016 |
| 1 | -2.38568345 | 4.86362481 | 0.59408704 |
| 1 | -2.20393890 | -5.89564718 | 0.65497144 |
| 1 | -2.25411483 | -5.37097600 | 2.34807351 |
| 1 | -3.65020872 | -5.07941230 | 1.27570400 |
| 1 | 0.11495492 | -3.02764754 | 0.56955490 |
| 1 | -0.06840159 | -4.11239668 | 1.97576928 |
| 1 | -0.08415359 | -4.78255641 | 0.32799900 |
| 1 | -3.46420462 | -3.44593445 | -0.65501823 |
| 1 | -1.93533276 | -2.59353441 | -0.98769424 |
| 1 | -2.04525997 | -4.35829722 | -1.21848174 |
| 1 | -0.97854562 | 1.77352779 | -5.82801458 |
| 1 | -1.08855166 | 3.43270538 | -5.21268513 |
| 1 | -2.44790186 | 2.29869762 | -4.98663161 |
| 1 | -0.68746467 | -0.03220625 | -2.60997770 |

| | | | |
|---|---|---|---|
| 1 | -2.21797018 | 0.31119793 | -3.44597788 |
| 1 | -0.78800163 | -0.17430025 | -4.38488788 |
| 1 | 1.10320323 | 3.05415199 | -4.03554791 |
| 1 | 1.34019774 | 1.67143909 | -2.94058772 |
| 1 | 1.15288991 | 1.40420209 | -4.69674301 |
| 1 | 0.82967916 | 1.80023593 | 3.24121705 |
| 8 | 1.84197848 | -0.99676868 | -0.33047999 |
| 6 | 2.35474362 | -1.23338138 | -1.65388881 |
| 1 | 1.51908524 | -1.64780696 | -2.22819483 |
| 8 | 1.80689161 | 0.23293166 | 1.51332880 |
| 1 | 2.69525684 | -0.29857073 | -2.11599083 |
| 1 | 3.17185376 | -1.97116426 | -1.62944371 |
| 31 | 0.17936508 | 0.36132013 | 0.57115483 |
| 8 | 3.44730929 | 0.71041609 | -0.00506421 |
| 6 | 2.70802052 | -0.30708644 | 0.65451663 |
| 6 | 3.65688030 | -1.34048627 | 1.30567185 |
| 8 | 4.69620587 | -0.64291631 | 2.05605189 |
| 6 | 5.41386010 | 0.29292931 | 1.36972371 |
| 6 | 4.88662837 | 0.56934676 | -0.04122638 |
| 8 | 6.37014822 | 0.83274043 | 1.87893153 |
| 6 | 5.45759773 | 1.84239915 | -0.63555748 |
| 6 | 2.96985662 | -2.28262994 | 2.27110167 |
| 1 | 4.14853347 | -1.91492465 | 0.50165821 |
| 1 | 5.16798747 | -0.29392244 | -0.67769827 |
| 1 | 5.05474349 | 1.99641757 | -1.64445133 |
| 1 | 6.55020789 | 1.76983379 | -0.68843631 |
| 1 | 5.19867230 | 2.70403345 | -0.00881486 |
| 1 | 2.20425653 | -2.86713798 | 1.74691741 |
| 1 | 2.49060832 | -1.71360107 | 3.07554349 |
| 1 | 3.70823316 | -2.97111972 | 2.70080910 |

Electronic energy = -3673.041055 a.u.
DFT-D3(BJ) dispersion correction = -0.117755 a.u.
Thermal free energy = 0.586257 a.u.
Gibbs free energy = -3672.572553 a.u.
Number of imaginary frequencies = 0.

**Int1a LA isomer/conformer 3**

| Atomic N. | X | Y | Z |
|---|---|---|---|
| 7 | 1.15790607 | 2.17536254 | 0.89190595 |
| 8 | -1.27574711 | 0.45606976 | 0.77388660 |
| 8 | 0.79427554 | 0.50157131 | -1.56956261 |
| 6 | 1.89480889 | 2.14968109 | 2.17981637 |
| 6 | -1.68644387 | 1.26085002 | 1.78615699 |
| 6 | -2.75681646 | 0.84376951 | 2.63143637 |
| 6 | -3.13420675 | 1.71137843 | 3.66669576 |
| 6 | -2.51728154 | 2.94627893 | 3.88482087 |
| 6 | -1.49661121 | 3.35497841 | 3.03335358 |
| 6 | -1.08588501 | 2.53209486 | 1.97889027 |
| 6 | -0.06820052 | 3.03990786 | 0.99333102 |
| 1 | -2.84293380 | 3.58320970 | 4.70715623 |
| 6 | -3.48702131 | -0.49614696 | 2.41147115 |
| 6 | 0.84045713 | 1.70600798 | -2.19017867 |

| | | | |
|---|---|---|---|
| 6 | 0.31332787 | 1.84871220 | -3.50881428 |
| 6 | 0.40602085 | 3.11305857 | -4.10711896 |
| 6 | 0.98205500 | 4.21474219 | -3.46858407 |
| 6 | 1.49653399 | 4.06218918 | -2.18654164 |
| 6 | 1.44147200 | 2.81816531 | -1.54690898 |
| 6 | 2.08400291 | 2.66171084 | -0.19490311 |
| 1 | 1.02517517 | 5.17972516 | -3.97327426 |
| 6 | -0.33278029 | 0.66483935 | -4.25418335 |
| 6 | -4.61888568 | -0.71060306 | 3.43386151 |
| 6 | -2.50169521 | -1.67911604 | 2.55905388 |
| 6 | -4.12735509 | -0.51929189 | 1.00278984 |
| 6 | -0.81405509 | 1.05880227 | -5.66344931 |
| 6 | -1.56993787 | 0.15988554 | -3.47495867 |
| 6 | 0.70006715 | -0.47399474 | -4.42600871 |
| 1 | 2.23629181 | 3.16678290 | 2.43382411 |
| 1 | 1.23511990 | 1.78330305 | 2.97332241 |
| 1 | 0.24514640 | 4.06231648 | 1.26528150 |
| 1 | -0.49604385 | 3.08061429 | -0.01927237 |
| 1 | -3.94084228 | 1.41544232 | 4.33485453 |
| 1 | -1.01896578 | 4.32827992 | 3.16745056 |
| 1 | 2.90456255 | 1.92685293 | -0.23164855 |
| 1 | 2.51011903 | 3.62795971 | 0.12379966 |
| 1 | 0.00843704 | 3.25126772 | -5.11084757 |
| 1 | 1.95854500 | 4.90697462 | -1.67075260 |
| 1 | -5.11183473 | -1.67044123 | 3.22382689 |
| 1 | -4.24345387 | -0.75105404 | 4.46675956 |
| 1 | -5.38581131 | 0.07486328 | 3.37271608 |
| 1 | -1.70915578 | -1.63295597 | 1.80441824 |
| 1 | -2.04225567 | -1.67846616 | 3.55886174 |
| 1 | -3.04123440 | -2.63061359 | 2.43629701 |
| 1 | -4.86694705 | 0.28825027 | 0.90183515 |
| 1 | -3.37306719 | -0.40060214 | 0.21709549 |
| 1 | -4.64849305 | -1.47617386 | 0.84648613 |
| 1 | -1.25717061 | 0.17717129 | -6.14827483 |
| 1 | 0.01173426 | 1.40592615 | -6.30101190 |
| 1 | -1.58553106 | 1.84167913 | -5.63454102 |
| 1 | -1.30992330 | -0.15268875 | -2.45827758 |
| 1 | -2.33070546 | 0.95058042 | -3.40444978 |
| 1 | -2.02036880 | -0.69539897 | -4.00154214 |
| 1 | 1.54221910 | -0.13836663 | -5.04857858 |
| 1 | 1.09695349 | -0.80434042 | -3.46020185 |
| 1 | 0.23023181 | -1.33280956 | -4.92959807 |
| 1 | 2.75404383 | 1.47720685 | 2.08954015 |
| 8 | 0.37433953 | -1.92325049 | 0.05855094 |
| 6 | 0.18427064 | -2.76981673 | -1.09102025 |
| 1 | -0.76951758 | -2.46148787 | -1.53275147 |
| 8 | 1.75220961 | -0.69310171 | 1.29176825 |
| 1 | 0.98564808 | -2.62131874 | -1.82606604 |
| 1 | 0.11358147 | -3.82390527 | -0.78890151 |
| 31 | 0.50908976 | 0.30135347 | 0.26335059 |
| 8 | 2.75602199 | -2.26221060 | 0.01048515 |
| 6 | 1.66060795 | -1.96794762 | 0.84025245 |
| 6 | 1.51316340 | -2.96090307 | 2.00473432 |

| | | | |
|---|---|---|---|
| 8 | 1.51110652 | -4.34696248 | 1.53474113 |
| 6 | 2.39845194 | -4.71832489 | 0.57787425 |
| 6 | 3.14244640 | -3.64165948 | -0.21360295 |
| 8 | 2.55200375 | -5.89207629 | 0.31247285 |
| 6 | 4.65502454 | -3.76106450 | -0.02460366 |
| 6 | 2.55827931 | -2.77529494 | 3.09629186 |
| 1 | 0.50671034 | -2.82449108 | 2.41937509 |
| 1 | 2.90806590 | -3.87568540 | -1.26563120 |
| 1 | 5.15761764 | -3.03532141 | -0.67572714 |
| 1 | 4.97878029 | -4.77333240 | -0.29522905 |
| 1 | 4.94429382 | -3.55856457 | 1.01456354 |
| 1 | 2.43209689 | -1.78947338 | 3.55868518 |
| 1 | 3.58011430 | -2.84055442 | 2.70092733 |
| 1 | 2.42374772 | -3.55000444 | 3.86194816 |

Electronic energy = -3673.036767 a.u.
DFT-D3(BJ) dispersion correction = -0.118494 a.u.
Thermal free energy = 0.586740 a.u.
Gibbs free energy = -3672.568522 a.u.
Number of imaginary frequencies = 0.

**Int1b LA isomer/conformer 1**

| Atomic N. | X | Y | Z |
|---|---|---|---|
| 8 | 0.80216618 | -3.84261238 | 1.13569368 |
| 6 | 2.18774264 | -3.45213095 | 1.10482837 |
| 1 | 2.40915653 | -2.81338580 | 0.23751753 |
| 8 | 0.17603761 | -1.72164029 | 1.88717236 |
| 8 | -0.14050096 | -2.17188357 | -0.26137952 |
| 1 | 2.75264784 | -4.38762471 | 1.02958649 |
| 1 | 2.46215345 | -2.92310874 | 2.02758684 |
| 7 | 0.73384877 | 1.15915587 | 2.36722370 |
| 8 | -1.13033475 | 0.69432262 | 0.07316948 |
| 8 | 1.91237339 | -0.00099944 | -0.13322942 |
| 6 | 0.23599689 | 0.70763215 | 3.69067443 |
| 6 | -1.92982819 | 1.55161715 | 0.76127785 |
| 6 | -3.31593105 | 1.63870359 | 0.43492511 |
| 6 | -4.10207390 | 2.51632376 | 1.19556583 |
| 6 | -3.58127937 | 3.30312725 | 2.22604233 |
| 6 | -2.21948458 | 3.24620247 | 2.49970667 |
| 6 | -1.38641184 | 2.39345297 | 1.76643068 |
| 6 | 0.10273095 | 2.46581248 | 1.97304635 |
| 1 | -4.23511251 | 3.96445574 | 2.79432250 |
| 6 | -3.93103432 | 0.82794145 | -0.72350899 |
| 6 | 2.68846436 | 1.11036532 | -0.08000245 |
| 6 | 3.35563054 | 1.56784595 | -1.25500499 |
| 6 | 4.16181081 | 2.70837918 | -1.13374487 |
| 6 | 4.32649665 | 3.39740380 | 0.07120505 |
| 6 | 3.67422157 | 2.93944706 | 1.20973176 |
| 6 | 2.86662476 | 1.79737019 | 1.14815259 |
| 6 | 2.23856375 | 1.27516351 | 2.41286628 |
| 1 | 4.95959644 | 4.28350450 | 0.11290934 |
| 6 | 3.20469723 | 0.84218732 | -2.60655951 |
| 6 | -5.43085610 | 1.12607668 | -0.90722182 |

| | | | |
|---|---|---|---|
| 6 | -3.79251833 | -0.68721432 | -0.45373511 |
| 6 | -3.22253508 | 1.19198023 | -2.05048616 |
| 6 | 4.02530485 | 1.51936721 | -3.72052950 |
| 6 | 1.72463706 | 0.85358120 | -3.05700702 |
| 6 | 3.71351429 | -0.61343578 | -2.48213127 |
| 1 | 0.51824991 | 1.44021905 | 4.46447776 |
| 1 | -0.85513803 | 0.61873626 | 3.66071897 |
| 1 | 0.34012272 | 3.21557369 | 2.74676543 |
| 1 | 0.60530770 | 2.77588725 | 1.04479060 |
| 1 | -5.16583525 | 2.59246155 | 0.97864928 |
| 1 | -1.78264753 | 3.88156284 | 3.27349209 |
| 1 | 2.60430577 | 0.26230898 | 2.64704789 |
| 1 | 2.50599575 | 1.93345025 | 3.25639658 |
| 1 | 4.68045614 | 3.08302113 | -2.01417051 |
| 1 | 3.79384385 | 3.45823262 | 2.16347977 |
| 1 | -5.81116947 | 0.53260476 | -1.75067841 |
| 1 | -6.02064855 | 0.85362000 | -0.02003879 |
| 1 | -5.61656816 | 2.18487588 | -1.13777673 |
| 1 | -2.73820851 | -0.96833775 | -0.36016363 |
| 1 | -4.31760069 | -0.96818299 | 0.47116129 |
| 1 | -4.23746464 | -1.25992998 | -1.28130994 |
| 1 | -3.35303681 | 2.25996919 | -2.27725068 |
| 1 | -2.14924317 | 0.97558607 | -2.00727255 |
| 1 | -3.66322022 | 0.61521229 | -2.87781744 |
| 1 | 3.88547234 | 0.95994337 | -4.65638153 |
| 1 | 5.10191414 | 1.52479518 | -3.49721303 |
| 1 | 3.70154697 | 2.55412605 | -3.90341114 |
| 1 | 1.07616802 | 0.34765437 | -2.33402029 |
| 1 | 1.36476815 | 1.88522170 | -3.18029622 |
| 1 | 1.62761852 | 0.34441361 | -4.02799707 |
| 1 | 4.77923222 | -0.62895988 | -2.21106618 |
| 1 | 3.15340519 | -1.16890272 | -1.72219900 |
| 1 | 3.60428309 | -1.13112096 | -3.44737691 |
| 1 | 0.66765481 | -0.27090585 | 3.92532539 |
| 31 | 0.33113008 | -0.17152115 | 0.83478211 |
| 6 | -0.11381436 | -2.78624499 | 1.07218532 |
| 6 | -1.51087138 | -3.35903966 | 1.35060776 |
| 8 | -1.90778679 | -4.31660066 | 0.31929441 |
| 6 | -1.55251108 | -4.13200957 | -0.97566309 |
| 6 | -0.63574624 | -2.96428884 | -1.37116273 |
| 8 | -1.96737615 | -4.89093315 | -1.82646221 |
| 6 | 0.50861944 | -3.45037126 | -2.25939472 |
| 6 | -1.63065656 | -4.07328774 | 2.68256466 |
| 1 | -2.20695913 | -2.50838071 | 1.30095785 |
| 1 | -1.27733023 | -2.27821668 | -1.94713648 |
| 1 | 1.10463169 | -2.59108914 | -2.59041829 |
| 1 | 0.09564143 | -3.96399472 | -3.13538518 |
| 1 | 1.15452705 | -4.14969310 | -1.71433538 |
| 1 | -1.37240972 | -3.38124079 | 3.49435131 |
| 1 | -0.95332638 | -4.93436926 | 2.71659577 |
| 1 | -2.66184154 | -4.41923825 | 2.82765935 |

Electronic energy = -3673.042737 a.u.

DFT-D3(BJ) dispersion correction = -0.120011 a.u.

Thermal free energy = 0.586379 a.u.
Gibbs free energy = -3672.576369 a.u.
Number of imaginary frequencies = 0.

**Int1b_LA isomer/conformer 2**

| Atomic N. | X | Y | Z |
|---|---|---|---|
| 7 | -0.93846794 | 1.30934448 | 2.19457802 |
| 8 | -0.50390899 | 0.97129314 | -0.75437649 |
| 8 | 1.12879448 | -0.69156760 | 1.29970522 |
| 6 | -2.34387619 | 1.30944732 | 2.67148053 |
| 6 | -1.16423891 | 2.15702020 | -0.82932190 |
| 6 | -1.65136063 | 2.61458482 | -2.08963555 |
| 6 | -2.37523981 | 3.81596677 | -2.10043456 |
| 6 | -2.60284446 | 4.57672298 | -0.95077354 |
| 6 | -2.05468498 | 4.15903204 | 0.25666205 |
| 6 | -1.31955674 | 2.96994136 | 0.32452379 |
| 6 | -0.57658158 | 2.63783411 | 1.59098118 |
| 1 | -3.18002218 | 5.49934382 | -1.00964291 |
| 6 | -1.36131313 | 1.85432236 | -3.39968986 |
| 6 | 1.96576075 | 0.12501618 | 1.99311031 |
| 6 | 3.36999021 | 0.08590629 | 1.74506306 |
| 6 | 4.17930594 | 0.94528981 | 2.50151922 |
| 6 | 3.67027082 | 1.81464494 | 3.47043590 |
| 6 | 2.30268370 | 1.83147721 | 3.71468186 |
| 6 | 1.44824424 | 0.98736529 | 2.99434910 |
| 6 | -0.01641345 | 0.96428650 | 3.34149344 |
| 1 | 4.34199248 | 2.46721639 | 4.02794769 |
| 6 | 3.98439322 | -0.87244203 | 0.70741660 |
| 6 | -1.91865646 | 2.59369277 | -4.63081726 |
| 6 | -2.00826577 | 0.45211785 | -3.37151669 |
| 6 | 0.16840383 | 1.72285881 | -3.59637400 |
| 6 | 5.51505973 | -0.72378420 | 0.61752413 |
| 6 | 3.41666324 | -0.57686408 | -0.70069008 |
| 6 | 3.68567262 | -2.33426651 | 1.11446960 |
| 1 | -2.46309270 | 2.05299504 | 3.47638499 |
| 1 | -3.01069538 | 1.56818563 | 1.84176791 |
| 1 | -0.74840234 | 3.42286475 | 2.34670354 |
| 1 | 0.50546631 | 2.59392731 | 1.39683972 |
| 1 | -2.77747343 | 4.17924029 | -3.04419441 |
| 1 | -2.16963798 | 4.76624562 | 1.15743722 |
| 1 | -0.32731133 | -0.03843198 | 3.67634178 |
| 1 | -0.21348722 | 1.67232523 | 4.16364356 |
| 1 | 5.25371120 | 0.94236674 | 2.32797355 |
| 1 | 1.88303780 | 2.49245587 | 4.47637684 |
| 1 | -1.66345426 | 2.02223762 | -5.53456117 |
| 1 | -3.01389541 | 2.68824216 | -4.59934351 |
| 1 | -1.48518996 | 3.59797786 | -4.74143633 |
| 1 | -1.61666363 | -0.15104053 | -2.54544686 |
| 1 | -3.10075359 | 0.53232563 | -3.26870966 |
| 1 | -1.79909214 | -0.07368871 | -4.31596285 |
| 1 | 0.63629661 | 2.71601697 | -3.65889976 |
| 1 | 0.63550446 | 1.16914449 | -2.77458579 |

| | | | |
|---|---|---|---|
| 1 | 0.37743797 | 1.19200598 | -4.53758252 |
| 1 | 5.89608181 | -1.43371582 | -0.13012236 |
| 1 | 6.01051032 | -0.95461969 | 1.57160217 |
| 1 | 5.81786828 | 0.28519985 | 0.30103095 |
| 1 | 2.32900842 | -0.69579054 | -0.73135375 |
| 1 | 3.66119349 | 0.44961962 | -1.01122062 |
| 1 | 3.86231390 | -1.26854639 | -1.43117335 |
| 1 | 4.15965981 | -2.56702795 | 2.07934460 |
| 1 | 2.61165718 | -2.52387351 | 1.20220605 |
| 1 | 4.09002309 | -3.02606225 | 0.36145834 |
| 1 | -2.60175450 | 0.31152153 | 3.04110807 |
| 8 | -3.28611045 | -2.66925743 | -0.09334513 |
| 6 | -4.01295164 | -1.52025029 | -0.56144654 |
| 1 | -3.45326748 | -0.98058172 | -1.33800639 |
| 8 | -1.92478018 | -1.29047378 | 1.17993546 |
| 8 | -1.06309170 | -2.12815420 | -0.70744396 |
| 1 | -4.94046957 | -1.91711564 | -0.98900595 |
| 1 | -4.24789877 | -0.83805744 | 0.26691974 |
| 31 | -0.54667792 | -0.11140292 | 0.75746371 |
| 6 | -1.98394108 | -2.40560323 | 0.38725318 |
| 6 | -1.59229090 | -3.74897760 | 1.07936633 |
| 8 | -0.16768314 | -4.03410316 | 0.96017633 |
| 6 | 0.34788567 | -4.03748994 | -0.30508381 |
| 6 | -0.52588943 | -3.31691173 | -1.33724646 |
| 8 | 1.42509870 | -4.54055911 | -0.53224372 |
| 6 | 0.24512941 | -2.91895013 | -2.58060273 |
| 6 | -1.93052803 | -3.77701180 | 2.55561343 |
| 1 | -2.13890110 | -4.54498416 | 0.55090928 |
| 1 | -1.37000692 | -3.97669941 | -1.61353236 |
| 1 | -0.42855335 | -2.44439320 | -3.30416202 |
| 1 | 0.69363441 | -3.81075571 | -3.03370313 |
| 1 | 1.05309086 | -2.22158211 | -2.32752893 |
| 1 | -3.00425853 | -3.59651925 | 2.69297978 |
| 1 | -1.36970818 | -2.99977985 | 3.08786060 |
| 1 | -1.67850147 | -4.75828806 | 2.97657811 |

Electronic energy = -3673.041322 a.u.
DFT-D3(BJ) dispersion correction = -0.119972 a.u.
Thermal free energy = 0.586868 a.u.
Gibbs free energy = -3672.574426 a.u.
Number of imaginary frequencies = 0.

**Int1b LA isomer/conformer 3**

| Atomic N. | X | Y | Z |
|---|---|---|---|
| 7 | -0.96982671 | 2.04499403 | 1.64483511 |
| 8 | -0.77951616 | 0.45180817 | -0.89382384 |
| 8 | 1.32245296 | 0.11664309 | 1.32438978 |
| 6 | -2.29129028 | 2.10825171 | 2.31835851 |
| 6 | -1.63928582 | 1.38996491 | -1.37050421 |
| 6 | -2.29555581 | 1.17566797 | -2.61870241 |
| 6 | -3.23270303 | 2.13799185 | -3.02291894 |
| 6 | -3.50611483 | 3.29061611 | -2.28168664 |
| 6 | -2.78624484 | 3.53378607 | -1.11715383 |

| | | | |
|---|---|---|---|
| 6 | -1.83860063 | 2.60731982 | -0.66708073 |
| 6 | -0.92490841 | 2.98197598 | 0.47021659 |
| 1 | -4.25132050 | 4.00385536 | -2.63340028 |
| 6 | -1.94618822 | -0.02096358 | -3.52815537 |
| 6 | 2.06186174 | 1.24862380 | 1.44416157 |
| 6 | 3.40845548 | 1.27158073 | 0.97348698 |
| 6 | 4.13185248 | 2.45906991 | 1.15266979 |
| 6 | 3.58811828 | 3.59685717 | 1.75538404 |
| 6 | 2.27464388 | 3.56301699 | 2.20752034 |
| 6 | 1.51045504 | 2.39778270 | 2.06855095 |
| 6 | 0.11846640 | 2.36693833 | 2.64322403 |
| 1 | 4.18999466 | 4.49871154 | 1.86553726 |
| 6 | 4.04988143 | 0.04895121 | 0.28772383 |
| 6 | -2.73009167 | 0.01486388 | -4.85362654 |
| 6 | -2.27158758 | -1.36399848 | -2.83886217 |
| 6 | -0.43971409 | 0.03138563 | -3.88141996 |
| 6 | 5.51148729 | 0.31590037 | -0.11911194 |
| 6 | 3.27170386 | -0.30251730 | -1.00230035 |
| 6 | 4.05705753 | -1.16060688 | 1.25175330 |
| 1 | -2.45152515 | 3.11945355 | 2.72656452 |
| 1 | -3.08096912 | 1.88491773 | 1.59273022 |
| 1 | -1.16267283 | 3.99886663 | 0.82577323 |
| 1 | 0.12095851 | 2.98512346 | 0.12867211 |
| 1 | -3.76977850 | 1.98942029 | -3.95776594 |
| 1 | -2.93311269 | 4.46052183 | -0.55779706 |
| 1 | 0.03071439 | 1.59913477 | 3.42872646 |
| 1 | -0.11377463 | 3.34370395 | 3.09930066 |
| 1 | 5.16108637 | 2.50648102 | 0.80223555 |
| 1 | 1.83044725 | 4.43891019 | 2.68560114 |
| 1 | -2.42564646 | -0.84227053 | -5.47082543 |
| 1 | -3.81584570 | -0.06144878 | -4.69630043 |
| 1 | -2.52496228 | 0.92744754 | -5.43117336 |
| 1 | -1.70542979 | -1.47691626 | -1.90798183 |
| 1 | -3.34598018 | -1.43619039 | -2.61424475 |
| 1 | -2.01351752 | -2.19908795 | -3.50783089 |
| 1 | -0.20020342 | 0.96240778 | -4.41509084 |
| 1 | 0.18734014 | -0.02598240 | -2.98444198 |
| 1 | -0.18247447 | -0.81139193 | -4.54086086 |
| 1 | 5.91823152 | -0.58667912 | -0.59675811 |
| 1 | 6.14765912 | 0.54564058 | 0.74783120 |
| 1 | 5.59724316 | 1.13892493 | -0.84317287 |
| 1 | 2.22521089 | -0.54566959 | -0.79148054 |
| 1 | 3.29581590 | 0.53816456 | -1.71064628 |
| 1 | 3.73772068 | -1.17073095 | -1.49258762 |
| 1 | 4.64575713 | -0.93521251 | 2.15287025 |
| 1 | 3.04203102 | -1.43267352 | 1.55964335 |
| 1 | 4.51763001 | -2.02892159 | 0.75664370 |
| 1 | -2.32523072 | 1.36766931 | 3.12384556 |
| 8 | -2.80315763 | -2.71019072 | 1.58853131 |
| 6 | -3.66805730 | -1.93285000 | 0.74346541 |
| 1 | -3.17721748 | -1.65971626 | -0.20110361 |
| 8 | -1.56488822 | -0.80653365 | 2.10624144 |
| 8 | -0.66588151 | -2.24237021 | 0.66596034 |

| | | | |
|---|---|---|---|
| 1 | -4.52813735 | -2.57808299 | 0.53312455 |
| 1 | -4.00396229 | -1.02279637 | 1.25955048 |
| 31 | -0.49132909 | 0.17351023 | 0.92463934 |
| 6 | -1.53567796 | -2.14750290 | 1.83147481 |
| 6 | -0.91441019 | -3.00046739 | 2.95602427 |
| 8 | -0.68848303 | -4.37525759 | 2.50045553 |
| 6 | -0.46516157 | -4.67644448 | 1.19725706 |
| 6 | -0.51248439 | -3.57547224 | 0.12933244 |
| 8 | -0.26314226 | -5.82902740 | 0.87462596 |
| 6 | 0.73933983 | -3.59536702 | -0.73883570 |
| 6 | 0.37918770 | -2.44322513 | 3.52321638 |
| 1 | -1.66984650 | -3.10963824 | 3.74618114 |
| 1 | -1.39811806 | -3.81113549 | -0.48608840 |
| 1 | 0.64295939 | -2.85768261 | -1.54543885 |
| 1 | 0.86208965 | -4.59395264 | -1.17267265 |
| 1 | 1.62812624 | -3.35199943 | -0.14241671 |
| 1 | 0.18686520 | -1.49160523 | 4.03362025 |
| 1 | 1.12060166 | -2.26839056 | 2.73330159 |
| 1 | 0.79058007 | -3.15588538 | 4.24900964 |

Electronic energy = -3673.040073 a.u.
DFT-D3(BJ) dispersion correction = -0.121116 a.u.
Thermal free energy = 0.586864 a.u.
Gibbs free energy = -3672.574325 a.u.
Number of imaginary frequencies = 0.

**TS2 eq LA isomer/conformer 1**

| Atomic N. | X | Y | Z |
|---|---|---|---|
| 8 | 1.86696947 | -2.82827759 | 2.89563990 |
| 6 | 3.16910195 | -2.20228076 | 2.74145174 |
| 1 | 3.23825169 | -1.74402094 | 1.74719608 |
| 8 | 0.90874296 | -0.77443701 | 2.78427005 |
| 8 | 0.37115127 | -1.97074401 | 0.52602440 |
| 1 | 3.88866687 | -3.01877260 | 2.84456396 |
| 1 | 3.32164764 | -1.44393861 | 3.51832104 |
| 7 | 0.22292699 | 1.75834239 | 1.46176434 |
| 8 | -1.49815857 | 0.10865940 | -0.20571734 |
| 8 | 1.83400857 | 0.19817975 | -0.39612088 |
| 6 | -0.56226003 | 1.85646403 | 2.71746874 |
| 6 | -2.30823040 | 1.19639468 | -0.20170745 |
| 6 | -3.69085717 | 1.05058289 | -0.53681630 |
| 6 | -4.48408413 | 2.20635247 | -0.53078908 |
| 6 | -3.98578715 | 3.46891832 | -0.20155486 |
| 6 | -2.64428997 | 3.59629416 | 0.13657638 |
| 6 | -1.80258846 | 2.47817349 | 0.12953947 |
| 6 | -0.34057060 | 2.68033004 | 0.41208133 |
| 1 | -4.64364862 | 4.33775616 | -0.20692965 |
| 6 | -4.31054163 | -0.31985697 | -0.87298352 |
| 6 | 2.58978486 | 1.31374526 | -0.47727185 |
| 6 | 3.47404671 | 1.47698057 | -1.58630085 |
| 6 | 4.24904776 | 2.64399242 | -1.62466705 |
| 6 | 4.17832041 | 3.63331676 | -0.63932139 |
| 6 | 3.31005406 | 3.46291971 | 0.43278605 |

| | | | |
|---|---|---|---|
| 6 | 2.52494431 | 2.30779147 | 0.53005421 |
| 6 | 1.66030681 | 2.11838269 | 1.74907744 |
| 1 | 4.79500675 | 4.52859879 | -0.71690232 |
| 6 | 3.57524252 | 0.41484049 | -2.69923162 |
| 6 | -5.79849672 | -0.21177785 | -1.25820506 |
| 6 | -4.23199177 | -1.23149133 | 0.37419847 |
| 6 | -3.58098030 | -0.96725744 | -2.07278800 |
| 6 | 4.61216545 | 0.80236810 | -3.77038407 |
| 6 | 2.21118331 | 0.26261735 | -3.41347575 |
| 6 | 4.01350737 | -0.94508928 | -2.10442972 |
| 1 | -0.57755393 | 2.90279102 | 3.06277251 |
| 1 | -1.59189951 | 1.52923989 | 2.53773308 |
| 1 | -0.16716161 | 3.71822405 | 0.74122488 |
| 1 | 0.26513788 | 2.52725363 | -0.49396980 |
| 1 | -5.53794813 | 2.12155175 | -0.78838336 |
| 1 | -2.22891855 | 4.57203913 | 0.39825162 |
| 1 | 2.04133463 | 1.31360817 | 2.39481306 |
| 1 | 1.65875852 | 3.05212331 | 2.33616900 |
| 1 | 4.93277407 | 2.79628849 | -2.45769143 |
| 1 | 3.23929143 | 4.22347355 | 1.21385455 |
| 1 | -6.17904139 | -1.21649361 | -1.49136245 |
| 1 | -6.41345358 | 0.19118603 | -0.44083542 |
| 1 | -5.94886589 | 0.41380295 | -2.14970469 |
| 1 | -3.20164466 | -1.32297683 | 0.73717284 |
| 1 | -4.84740543 | -0.82105583 | 1.18805325 |
| 1 | -4.60891056 | -2.23764420 | 0.13660349 |
| 1 | -3.69338322 | -0.34283787 | -2.97100449 |
| 1 | -2.51208782 | -1.09518254 | -1.87694955 |
| 1 | -4.01982450 | -1.95270181 | -2.29016137 |
| 1 | 4.64805222 | 0.01262468 | -4.53408909 |
| 1 | 5.62350893 | 0.90212178 | -3.35024500 |
| 1 | 4.35214901 | 1.74176300 | -4.27944613 |
| 1 | 1.42831063 | -0.06236210 | -2.71983886 |
| 1 | 1.90316296 | 1.21521115 | -3.86880016 |
| 1 | 2.29262185 | -0.48528036 | -4.21661472 |
| 1 | 4.99595881 | -0.85486001 | -1.61774433 |
| 1 | 3.28891182 | -1.31497812 | -1.37105548 |
| 1 | 4.10421944 | -1.68968880 | -2.90976167 |
| 1 | -0.09944245 | 1.21900403 | 3.47576904 |
| 31 | 0.23138557 | -0.10514228 | 0.51154423 |
| 6 | 0.81865859 | -2.01416636 | 2.78982258 |
| 6 | -0.51556540 | -2.74974728 | 2.91365790 |
| 8 | -0.50109863 | -4.03362942 | 2.26010489 |
| 6 | -0.56799060 | -4.16468716 | 0.88571101 |
| 6 | -0.57852405 | -2.89053249 | 0.01585909 |
| 8 | -0.64218950 | -5.28596973 | 0.43749234 |
| 6 | -0.30038157 | -3.24201965 | -1.44386935 |
| 6 | -0.84826726 | -3.01511168 | 4.38186932 |
| 1 | -1.27695751 | -2.09359336 | 2.46833587 |
| 1 | -1.60799229 | -2.48661017 | 0.08849698 |
| 1 | -0.35621029 | -2.32922578 | -2.05137348 |
| 1 | -1.03258169 | -3.96776795 | -1.81822026 |
| 1 | 0.70305806 | -3.67449951 | -1.54358017 |

| | | | |
|---|---|---|---|
| 1 | -0.86185080 | -2.07324576 | 4.94671249 |
| 1 | -0.10533506 | -3.69043756 | 4.82413816 |
| 1 | -1.83739996 | -3.48391557 | 4.45312166 |

Electronic energy = -3673.031012 a.u.
DFT-D3(BJ) dispersion correction = -0.119727 a.u.
Thermal free energy = 0.586046 a.u.
Gibbs free energy = -3672.564694 a.u.
Number of imaginary frequencies = 1.

**TS2 eq LA isomer/conformer 2**

| Atomic N. | X | Y | Z |
|---|---|---|---|
| 7 | -0.85282868 | 1.03640997 | 1.87760365 |
| 8 | -0.69674909 | 0.77490807 | -1.03791296 |
| 8 | 1.32472730 | -0.72079211 | 0.95807326 |
| 6 | -2.26683116 | 1.29743731 | 2.24426150 |
| 6 | -1.09871614 | 2.06276202 | -1.02265799 |
| 6 | -1.65935087 | 2.63204741 | -2.20647693 |
| 6 | -2.09130788 | 3.96395254 | -2.13799596 |
| 6 | -1.98230720 | 4.73798370 | -0.97874314 |
| 6 | -1.40684235 | 4.18034267 | 0.15796505 |
| 6 | -0.95288920 | 2.85707736 | 0.14212355 |
| 6 | -0.23559310 | 2.30181360 | 1.34117496 |
| 1 | -2.33586717 | 5.76898575 | -0.97347552 |
| 6 | -1.76025498 | 1.82732558 | -3.51802540 |
| 6 | 2.03603220 | -0.04033693 | 1.89378953 |
| 6 | 3.45816040 | 0.06819240 | 1.78312218 |
| 6 | 4.13648510 | 0.73057783 | 2.81649232 |
| 6 | 3.48830199 | 1.30435205 | 3.91212058 |
| 6 | 2.10268474 | 1.24304116 | 3.97934127 |
| 6 | 1.37581587 | 0.57641542 | 2.98627067 |
| 6 | -0.12202635 | 0.53958857 | 3.10392404 |
| 1 | 4.06282663 | 1.81039369 | 4.68779469 |
| 6 | 4.24103212 | -0.49377996 | 0.58052075 |
| 6 | -2.38219309 | 2.65789437 | -4.65631819 |
| 6 | -2.64613509 | 0.57399833 | -3.32453966 |
| 6 | -0.34597260 | 1.39808154 | -3.97715998 |
| 6 | 5.74477625 | -0.16368438 | 0.65744013 |
| 6 | 3.71563601 | 0.13920915 | -0.73000723 |
| 6 | 4.11543131 | -2.03221154 | 0.54191506 |
| 1 | -2.30878830 | 2.15767789 | 2.93244576 |
| 1 | -2.84868097 | 1.53568733 | 1.34796607 |
| 1 | -0.21840464 | 3.04945016 | 2.15148664 |
| 1 | 0.80921370 | 2.06480742 | 1.09530783 |
| 1 | -2.53040242 | 4.42272568 | -3.02208853 |
| 1 | -1.28900659 | 4.77364588 | 1.06771100 |
| 1 | -0.50102431 | -0.47912061 | 3.28132868 |
| 1 | -0.42857841 | 1.16613257 | 3.95777130 |
| 1 | 5.21980906 | 0.81418937 | 2.76109171 |
| 1 | 1.56581545 | 1.71086514 | 4.80766535 |
| 1 | -2.42039561 | 2.04358697 | -5.56720829 |
| 1 | -3.41057014 | 2.97287154 | -4.42602777 |
| 1 | -1.78784990 | 3.55354929 | -4.88711786 |

| | | | |
|---|---|---|---|
| 1 | -2.21196103 | -0.11020342 | -2.58680463 |
| 1 | -3.65818596 | 0.86225098 | -3.00115013 |
| 1 | -2.74138212 | 0.03515899 | -4.27969837 |
| 1 | 0.28331470 | 2.27992296 | -4.16541862 |
| 1 | 0.14571507 | 0.77254426 | -3.22403765 |
| 1 | -0.41598555 | 0.82599223 | -4.91508150 |
| 1 | 6.24026632 | -0.57034296 | -0.23576733 |
| 1 | 6.22609520 | -0.62009829 | 1.53424931 |
| 1 | 5.93405247 | 0.91950864 | 0.67735428 |
| 1 | 2.64472675 | -0.03758981 | -0.87296712 |
| 1 | 3.88521647 | 1.22619689 | -0.72644538 |
| 1 | 4.25539017 | -0.28298938 | -1.59102237 |
| 1 | 4.57883120 | -2.47604465 | 1.43504333 |
| 1 | 3.07597494 | -2.36792350 | 0.50373107 |
| 1 | 4.63964844 | -2.43030429 | -0.34057242 |
| 1 | -2.68495107 | 0.41321644 | 2.73025274 |
| 8 | -3.59892321 | -2.49991345 | 0.10118303 |
| 6 | -4.29857159 | -1.24445355 | -0.09334970 |
| 1 | -3.65746093 | -0.54334188 | -0.64201975 |
| 8 | -2.15500617 | -1.44438815 | 1.51110506 |
| 8 | -0.90486813 | -1.95708227 | -0.62794971 |
| 1 | -5.17934084 | -1.49854457 | -0.68945599 |
| 1 | -4.59161711 | -0.81663114 | 0.87263024 |
| 31 | -0.43714312 | -0.40998629 | 0.39356291 |
| 6 | -2.48446393 | -2.44834709 | 0.84758252 |
| 6 | -1.95142102 | -3.86296773 | 1.06328177 |
| 8 | -0.54744542 | -3.89305782 | 1.37412584 |
| 6 | 0.38510588 | -3.78333759 | 0.35158440 |
| 6 | -0.09201877 | -3.07647824 | -0.93072116 |
| 8 | 1.49441850 | -4.22045469 | 0.54258090 |
| 6 | 1.06540895 | -2.75992656 | -1.87133324 |
| 6 | -2.67151546 | -4.50659037 | 2.24958014 |
| 1 | -2.15107584 | -4.43925142 | 0.14752048 |
| 1 | -0.75476867 | -3.80254555 | -1.44833708 |
| 1 | 0.66579545 | -2.30284715 | -2.78536725 |
| 1 | 1.61115563 | -3.67562079 | -2.13213420 |
| 1 | 1.76835525 | -2.06600547 | -1.39752686 |
| 1 | -3.75343633 | -4.54265785 | 2.06833124 |
| 1 | -2.47586989 | -3.93408823 | 3.16520166 |
| 1 | -2.29924202 | -5.52883148 | 2.38730764 |

Electronic energy = -3673.024989 a.u.
DFT-D3(BJ) dispersion correction = -0.121776 a.u.
Thermal free energy = 0.587309 a.u.
Gibbs free energy = -3672.559455 a.u.
Number of imaginary frequencies = 1.

**TS2 eq LA isomer/conformer 3**

| Atomic N. | X | Y | Z |
|---|---|---|---|
| 7 | -0.99857360 | 1.75151324 | 1.33199489 |
| 8 | -0.84557486 | 0.23166104 | -1.18343353 |
| 8 | 1.40019321 | 0.03166742 | 1.04640830 |
| 6 | -2.41307449 | 1.89420414 | 1.76178193 |

| | | | |
|---|---|---|---|
| 6 | -1.56891167 | 1.26567721 | -1.67015088 |
| 6 | -2.32156920 | 1.10438550 | -2.87287164 |
| 6 | -3.04997993 | 2.21185756 | -3.32955027 |
| 6 | -3.06575084 | 3.43969917 | -2.66142750 |
| 6 | -2.32426572 | 3.58559346 | -1.49430346 |
| 6 | -1.57005167 | 2.51520777 | -1.00164151 |
| 6 | -0.69861215 | 2.71045423 | 0.20528886 |
| 1 | -3.65089059 | 4.27033424 | -3.05570555 |
| 6 | -2.33921814 | -0.23021553 | -3.64277434 |
| 6 | 2.05327272 | 1.14320838 | 1.46746874 |
| 6 | 3.45023036 | 1.28589773 | 1.20385540 |
| 6 | 4.08247137 | 2.44183874 | 1.68281376 |
| 6 | 3.40580797 | 3.44442534 | 2.38157988 |
| 6 | 2.04406595 | 3.30185604 | 2.61684990 |
| 6 | 1.36445153 | 2.15942717 | 2.17720699 |
| 6 | -0.09666080 | 2.02197027 | 2.51547432 |
| 1 | 3.94059348 | 4.32842445 | 2.72831798 |
| 6 | 4.24561262 | 0.22473216 | 0.41780519 |
| 6 | -3.20363402 | -0.15544455 | -4.91542196 |
| 6 | -2.93421125 | -1.34459281 | -2.74976683 |
| 6 | -0.90536952 | -0.60638303 | -4.08515406 |
| 6 | 5.72859192 | 0.61065698 | 0.25710174 |
| 6 | 3.65864015 | 0.07329636 | -1.00534308 |
| 6 | 4.20854187 | -1.12793839 | 1.16592181 |
| 1 | -2.60020399 | 2.93811584 | 2.06085539 |
| 1 | -3.07950687 | 1.64236128 | 0.92975885 |
| 1 | -0.81086469 | 3.73589945 | 0.59419441 |
| 1 | 0.36235413 | 2.56950998 | -0.04708929 |
| 1 | -3.63460350 | 2.11697054 | -4.24280596 |
| 1 | -2.31041241 | 4.53882265 | -0.96084434 |
| 1 | -0.27702457 | 1.19412589 | 3.21655607 |
| 1 | -0.44390747 | 2.95385027 | 2.99114132 |
| 1 | 5.14664888 | 2.57393074 | 1.49790001 |
| 1 | 1.49144495 | 4.07508135 | 3.15526557 |
| 1 | -3.17482710 | -1.12955678 | -5.42415571 |
| 1 | -4.25640965 | 0.06828505 | -4.69005919 |
| 1 | -2.83115625 | 0.59821433 | -5.62400198 |
| 1 | -2.36002541 | -1.46372807 | -1.82434475 |
| 1 | -3.97719669 | -1.11198092 | -2.48778009 |
| 1 | -2.92873788 | -2.30313921 | -3.29029870 |
| 1 | -0.50193959 | 0.15481552 | -4.76856375 |
| 1 | -0.22884613 | -0.69247735 | -3.22827840 |
| 1 | -0.91889268 | -1.56795061 | -4.62034321 |
| 1 | 6.24151897 | -0.17939448 | -0.30960429 |
| 1 | 6.23998642 | 0.70644897 | 1.22576070 |
| 1 | 5.85494518 | 1.55051517 | -0.29926142 |
| 1 | 2.60275531 | -0.21396662 | -0.98116434 |
| 1 | 3.74669909 | 1.01885355 | -1.55991316 |
| 1 | 4.21573925 | -0.69653541 | -1.56030142 |
| 1 | 4.68473625 | -1.03604937 | 2.15295529 |
| 1 | 3.18160057 | -1.47793436 | 1.31105304 |
| 1 | 4.75991249 | -1.88918567 | 0.59363323 |
| 1 | -2.59692311 | 1.22205269 | 2.60388279 |

| | | | |
|---|---|---|---|
| 8 | -2.82955599 | -2.60949683 | 2.26325464 |
| 6 | -3.88060904 | -1.72376561 | 1.82215941 |
| 1 | -3.61399794 | -1.27628410 | 0.85467470 |
| 8 | -1.48241496 | -0.80471039 | 2.65665388 |
| 8 | -0.80479199 | -1.95508444 | 0.63965672 |
| 1 | -4.76400614 | -2.35907149 | 1.71034873 |
| 1 | -4.06315422 | -0.93759793 | 2.56498790 |
| 31 | -0.41147292 | -0.10960120 | 0.61605215 |
| 6 | -1.62591231 | -2.03868604 | 2.48558068 |
| 6 | -0.63432795 | -3.09993052 | 2.97205663 |
| 8 | -0.61769122 | -4.29614353 | 2.13759494 |
| 6 | -0.42302224 | -4.35144711 | 0.78226465 |
| 6 | -0.40921608 | -3.09092045 | -0.10418760 |
| 8 | -0.29442254 | -5.44627619 | 0.27574861 |
| 6 | 0.95357305 | -2.94974184 | -0.78624916 |
| 6 | 0.76046616 | -2.57078123 | 3.25163960 |
| 1 | -1.06083000 | -3.49261189 | 3.91099811 |
| 1 | -1.16576099 | -3.30666089 | -0.88105232 |
| 1 | 0.91991407 | -2.12000537 | -1.50449586 |
| 1 | 1.19054461 | -3.87447834 | -1.32616949 |
| 1 | 1.74476123 | -2.75020337 | -0.05232562 |
| 1 | 0.71419632 | -1.80231166 | 4.03430367 |
| 1 | 1.21273375 | -2.11952186 | 2.36187506 |
| 1 | 1.39200926 | -3.39403582 | 3.60815954 |

Electronic energy = -3673.021939 a.u.
DFT-D3(BJ) dispersion correction = -0.122936 a.u.
Thermal free energy = 0.587150 a.u.
Gibbs free energy = -3672.557725 a.u.
Number of imaginary frequencies = 1.

**TS2 eq LA isomer/conformer 4**

| Atomic N. | X | Y | Z |
|---|---|---|---|
| 8 | 1.82328534 | -3.54923892 | 1.16198206 |
| 6 | 3.02167153 | -2.72451520 | 1.14418340 |
| 1 | 2.90340233 | -1.92269313 | 0.40592876 |
| 8 | 0.66769272 | -1.81613123 | 2.07587910 |
| 8 | -0.00478959 | -2.04526806 | -0.29388216 |
| 1 | 3.82611728 | -3.40730309 | 0.85774785 |
| 1 | 3.20337987 | -2.30522037 | 2.14076281 |
| 7 | 0.41149446 | 1.01136053 | 2.19359374 |
| 8 | -1.51220810 | 0.40018132 | 0.09295833 |
| 8 | 1.72116780 | 0.12511598 | -0.25530094 |
| 6 | -0.34622824 | 0.69416714 | 3.42861629 |
| 6 | -2.14635253 | 1.51432669 | 0.53918463 |
| 6 | -3.51341248 | 1.74064839 | 0.18181968 |
| 6 | -4.12998581 | 2.90043211 | 0.67071241 |
| 6 | -3.47410679 | 3.82493544 | 1.48642492 |
| 6 | -2.14573336 | 3.59913635 | 1.82300615 |
| 6 | -1.47494328 | 2.46619749 | 1.34703696 |
| 6 | -0.00593835 | 2.34081101 | 1.63524103 |
| 1 | -3.99875569 | 4.70875740 | 1.84902287 |
| 6 | -4.30273581 | 0.74881786 | -0.69233215 |

| | | | |
|---|---|---|---|
| 6 | 2.64948487 | 1.04739344 | 0.06880593 |
| 6 | 3.53205991 | 1.52832782 | -0.94763517 |
| 6 | 4.50542545 | 2.46281123 | -0.57001966 |
| 6 | 4.62579393 | 2.94426274 | 0.73682141 |
| 6 | 3.74688339 | 2.48602057 | 1.71111917 |
| 6 | 2.76782775 | 1.53593349 | 1.39534497 |
| 6 | 1.88358116 | 1.02347171 | 2.50513124 |
| 1 | 5.39291382 | 3.67852306 | 0.98225391 |
| 6 | 3.41181898 | 1.05250621 | -2.41007805 |
| 6 | -5.73288059 | 1.23676205 | -0.99328619 |
| 6 | -4.42930555 | -0.59420693 | 0.06135815 |
| 6 | -3.60119867 | 0.56020862 | -2.05824804 |
| 6 | 4.47841978 | 1.69965816 | -3.31391931 |
| 6 | 2.02700424 | 1.44482327 | -2.97858858 |
| 6 | 3.60146284 | -0.48044154 | -2.50353956 |
| 1 | -0.18936324 | 1.48633122 | 4.17899847 |
| 1 | -1.41539574 | 0.62868482 | 3.19994736 |
| 1 | 0.29940009 | 3.12587857 | 2.34776330 |
| 1 | 0.58608496 | 2.49133229 | 0.72025526 |
| 1 | -5.16919279 | 3.09093857 | 0.40999588 |
| 1 | -1.60369456 | 4.31162930 | 2.44896317 |
| 1 | 2.13623905 | -0.01102505 | 2.78090572 |
| 1 | 2.03448248 | 1.65336847 | 3.39862967 |
| 1 | 5.19419050 | 2.84186292 | -1.32267332 |
| 1 | 3.81522655 | 2.85889411 | 2.73577428 |
| 1 | -6.23703527 | 0.48823336 | -1.62122595 |
| 1 | -6.33300924 | 1.35540712 | -0.07984031 |
| 1 | -5.73930836 | 2.18951988 | -1.54252195 |
| 1 | -3.45280218 | -0.96737462 | 0.38377669 |
| 1 | -5.05653524 | -0.46905234 | 0.95606959 |
| 1 | -4.89503288 | -1.35812259 | -0.57761884 |
| 1 | -3.59711909 | 1.50643253 | -2.61919069 |
| 1 | -2.56269431 | 0.23225471 | -1.94271934 |
| 1 | -4.14350653 | -0.18654889 | -2.65740633 |
| 1 | 4.34776402 | 1.32988131 | -4.34082079 |
| 1 | 5.50014639 | 1.44374776 | -2.99755120 |
| 1 | 4.38739300 | 2.79495955 | -3.34406471 |
| 1 | 1.21283495 | 0.98329180 | -2.40934467 |
| 1 | 1.89515102 | 2.53637028 | -2.95685816 |
| 1 | 1.94705403 | 1.11575723 | -4.02584076 |
| 1 | 4.58552790 | -0.77445894 | -2.10878778 |
| 1 | 2.82226849 | -1.01473832 | -1.94933510 |
| 1 | 3.55487013 | -0.79565477 | -3.55709457 |
| 1 | 0.00152565 | -0.26518315 | 3.82431889 |
| 31 | 0.12941538 | -0.35660174 | 0.61927235 |
| 6 | 0.68445677 | -2.95098567 | 1.51776087 |
| 6 | -0.45661882 | -3.96796584 | 1.67402613 |
| 8 | -1.74979031 | -3.45130992 | 1.29091549 |
| 6 | -2.08410645 | -3.33472419 | -0.05626284 |
| 6 | -1.13674676 | -2.54454303 | -0.97044277 |
| 8 | -3.16242862 | -3.73815131 | -0.42403120 |
| 6 | -0.73025739 | -3.35837650 | -2.20497322 |
| 6 | -0.19485319 | -5.34058285 | 1.06409204 |

| | | | |
|---|---|---|---|
| 1 | -0.55923814 | -4.08023500 | 2.76742077 |
| 1 | -1.77192760 | -1.70617151 | -1.30990589 |
| 1 | -0.21792363 | -2.69840264 | -2.91586852 |
| 1 | -1.61665046 | -3.79042768 | -2.68658185 |
| 1 | -0.03951141 | -4.16782093 | -1.93246162 |
| 1 | 0.66849351 | -5.81528711 | 1.54518461 |
| 1 | 0.00864148 | -5.28063250 | -0.01212834 |
| 1 | -1.07873738 | -5.97097015 | 1.22337401 |

Electronic energy = -3673.021011 a.u.
DFT-D3(BJ) dispersion correction = -0.121570 a.u.
Thermal free energy = 0.586893 a.u.
Gibbs free energy = -3672.555688 a.u.
Number of imaginary frequencies = 1.

**Int2 LA isomer/conformer 1**

| Atomic N. | X | Y | Z |
|---|---|---|---|
| 8 | 4.23744765 | -2.31194577 | -0.55942857 |
| 6 | 4.39798060 | -3.32239445 | 0.47183550 |
| 1 | 5.34447239 | -3.17399525 | 1.00568483 |
| 8 | 4.39866601 | -0.70150370 | 1.04849994 |
| 8 | 0.66905634 | 2.76618003 | 0.34247118 |
| 1 | 4.39251539 | -4.27779816 | -0.05995703 |
| 1 | 3.56408551 | -3.26606971 | 1.18173672 |
| 7 | -2.19334443 | 2.18361265 | 0.39919500 |
| 8 | -0.58051747 | -0.14896384 | 1.24877852 |
| 8 | -0.70186393 | 0.82939048 | -1.78653551 |
| 6 | -2.08737725 | 3.29666857 | 1.37421391 |
| 6 | -1.43602176 | -0.14115685 | 2.29594305 |
| 6 | -1.07792689 | -0.81778738 | 3.50164482 |
| 6 | -2.00262256 | -0.79551432 | 4.55511439 |
| 6 | -3.23668331 | -0.14530276 | 4.46721290 |
| 6 | -3.57822768 | 0.50216894 | 3.28498405 |
| 6 | -2.69815838 | 0.49991723 | 2.19734731 |
| 6 | -3.12933143 | 1.12068986 | 0.89679325 |
| 1 | -3.92062957 | -0.15036796 | 5.31593605 |
| 6 | 0.27327372 | -1.54534352 | 3.64751159 |
| 6 | -1.90904341 | 0.80711946 | -2.38592505 |
| 6 | -2.15061786 | -0.10983284 | -3.45391763 |
| 6 | -3.41663255 | -0.08275578 | -4.05440261 |
| 6 | -4.42988638 | 0.79076646 | -3.64771371 |
| 6 | -4.18164435 | 1.68112148 | -2.60899432 |
| 6 | -2.92989779 | 1.70571555 | -1.98335224 |
| 6 | -2.65676599 | 2.73766708 | -0.92216121 |
| 1 | -5.40161010 | 0.76991408 | -4.14100375 |
| 6 | -1.06392286 | -1.09543408 | -3.92753963 |
| 6 | 0.41858747 | -2.22496243 | 5.02221881 |
| 6 | 1.43630751 | -0.53594743 | 3.50692274 |
| 6 | 0.40072682 | -2.65170699 | 2.57310417 |
| 6 | -1.54834556 | -1.96758471 | -5.10109288 |
| 6 | -0.67200073 | -2.05008971 | -2.77466778 |
| 6 | 0.18259628 | -0.31932249 | -4.41612018 |
| 1 | -3.07612897 | 3.76415195 | 1.51837611 |

| | | | |
|---|---|---|---|
| 1 | -1.72923545 | 2.91232788 | 2.33485459 |
| 1 | -4.13640804 | 1.55946381 | 1.00537606 |
| 1 | -3.18416516 | 0.36290847 | 0.10096151 |
| 1 | -1.75351617 | -1.30002284 | 5.48694394 |
| 1 | -4.54282360 | 1.00583893 | 3.18770403 |
| 1 | -1.86273553 | 3.43163034 | -1.24296388 |
| 1 | -3.57092186 | 3.33022238 | -0.74514877 |
| 1 | -3.63015758 | -0.77284837 | -4.86879791 |
| 1 | -4.95658488 | 2.37686719 | -2.27886781 |
| 1 | 1.39422855 | -2.72983310 | 5.06897095 |
| 1 | 0.38282226 | -1.50091937 | 5.84903529 |
| 1 | -0.35731951 | -2.98559382 | 5.19197444 |
| 1 | 1.41555706 | -0.04005370 | 2.53137928 |
| 1 | 1.37911518 | 0.23110433 | 4.29333006 |
| 1 | 2.40243495 | -1.05308533 | 3.60552400 |
| 1 | -0.40727654 | -3.39017725 | 2.68050490 |
| 1 | 0.36020630 | -2.23464790 | 1.56109778 |
| 1 | 1.35840852 | -3.18055936 | 2.70141510 |
| 1 | -0.73585668 | -2.64412084 | -5.40287586 |
| 1 | -1.82137594 | -1.36517662 | -5.97967714 |
| 1 | -2.41104427 | -2.59093996 | -4.82497325 |
| 1 | -0.26617436 | -1.50333560 | -1.91717273 |
| 1 | -1.54453284 | -2.62851196 | -2.43793541 |
| 1 | 0.09055483 | -2.76284013 | -3.12516182 |
| 1 | -0.07351060 | 0.32864142 | -5.26723994 |
| 1 | 0.60045713 | 0.30280755 | -3.61697970 |
| 1 | 0.95406358 | -1.02956787 | -4.75239936 |
| 1 | -1.37136808 | 4.03363135 | 0.99674092 |
| 31 | -0.35709083 | 1.23881978 | 0.01337567 |
| 6 | 4.25702697 | -1.03577693 | -0.10796685 |
| 6 | 4.12423318 | -0.07866277 | -1.30075620 |
| 8 | 3.71082530 | 1.23544116 | -0.82374472 |
| 6 | 2.44611804 | 1.34367054 | -0.40793792 |
| 6 | 2.04033123 | 2.73167486 | 0.07419685 |
| 8 | 1.64576439 | 0.40373218 | -0.42772112 |
| 6 | 2.87050302 | 3.13012913 | 1.30733932 |
| 6 | 5.45689774 | 0.11286789 | -2.01322153 |
| 1 | 3.35379165 | -0.46611877 | -1.98061323 |
| 1 | 2.29038759 | 3.42510739 | -0.75624465 |
| 1 | 2.55191160 | 4.12940772 | 1.62888363 |
| 1 | 3.94351140 | 3.15234127 | 1.07642306 |
| 1 | 2.69350519 | 2.42363677 | 2.12862531 |
| 1 | 5.80618278 | -0.85087109 | -2.40397952 |
| 1 | 6.21007735 | 0.51304585 | -1.32243789 |
| 1 | 5.33666762 | 0.80904164 | -2.85255218 |

Electronic energy = -3673.068305 a.u.
DFT-D3(BJ) dispersion correction = -0.114636 a.u.
Thermal free energy = 0.581614 a.u.
Gibbs free energy = -3672.601326 a.u.
Number of imaginary frequencies = 0.

**Int2_LA isomer/conformer 2**

| Atomic N. | X | Y | Z |
|---|---|---|---|
| 8 | -3.05885451 | -3.52076674 | -0.39565351 |
| 6 | -3.84594354 | -3.93070587 | 0.75469068 |
| 1 | -4.10018168 | -3.05669029 | 1.36580396 |
| 8 | -4.82037718 | -2.24456974 | -1.07369530 |
| 8 | 1.72609024 | -1.87914301 | -1.01559031 |
| 1 | -3.20411778 | -4.62091950 | 1.30893669 |
| 1 | -4.76673281 | -4.42601442 | 0.42402392 |
| 7 | 3.01379563 | 0.49168745 | 0.11040774 |
| 8 | 0.37525778 | -0.03031022 | 1.34720492 |
| 8 | 0.68954730 | 1.06552718 | -1.64660601 |
| 6 | 3.97883493 | -0.57580153 | 0.47042028 |
| 6 | 1.07355041 | 0.13110590 | 2.49362797 |
| 6 | 0.53747033 | -0.37367889 | 3.71744339 |
| 6 | 1.28767758 | -0.17487013 | 4.88483258 |
| 6 | 2.51850064 | 0.48691118 | 4.89103787 |
| 6 | 3.02559334 | 0.98344094 | 3.69541587 |
| 6 | 2.31207490 | 0.82405633 | 2.50247858 |
| 6 | 2.83414115 | 1.45973783 | 1.24299525 |
| 1 | 3.06861886 | 0.61306773 | 5.82350507 |
| 6 | -0.81546074 | -1.11115026 | 3.76174626 |
| 6 | 1.30545103 | 2.25357780 | -1.81254710 |
| 6 | 0.56014139 | 3.37475262 | -2.28882310 |
| 6 | 1.25009284 | 4.58258986 | -2.45988001 |
| 6 | 2.61299635 | 4.72324206 | -2.18083190 |
| 6 | 3.32831206 | 3.62368932 | -1.71991457 |
| 6 | 2.69142171 | 2.38968389 | -1.54473575 |
| 6 | 3.50461478 | 1.19348057 | -1.12848252 |
| 1 | 3.10456803 | 5.68561274 | -2.32296075 |
| 6 | -0.94595486 | 3.26543063 | -2.59917664 |
| 6 | -1.19442094 | -1.53792873 | 5.19259661 |
| 6 | -0.74577936 | -2.39568492 | 2.90295863 |
| 6 | -1.94470484 | -0.19014422 | 3.24177248 |
| 6 | -1.52652274 | 4.59564940 | -3.11497970 |
| 6 | -1.72752321 | 2.88737676 | -1.31851024 |
| 6 | -1.18740134 | 2.20292314 | -3.69819473 |
| 1 | 4.96515596 | -0.13226927 | 0.68786549 |
| 1 | 3.62337733 | -1.11047787 | 1.35738960 |
| 1 | 3.80131003 | 1.95207321 | 1.44431874 |
| 1 | 2.14137764 | 2.23327363 | 0.87899692 |
| 1 | 0.90370683 | -0.55311957 | 5.83048158 |
| 1 | 3.97898434 | 1.51658291 | 3.67715907 |
| 1 | 3.50774870 | 0.42447322 | -1.91819687 |
| 1 | 4.54957421 | 1.50412561 | -0.95645472 |
| 1 | 0.70683819 | 5.45500483 | -2.81872091 |
| 1 | 4.39521528 | 3.70919803 | -1.50089359 |
| 1 | -2.16871611 | -2.04702770 | 5.16694143 |
| 1 | -0.46600949 | -2.23969108 | 5.62439258 |
| 1 | -1.29018308 | -0.67618093 | 5.86860427 |
| 1 | -0.55243665 | -2.16015509 | 1.85106767 |
| 1 | 0.04839444 | -3.06427825 | 3.26650456 |
| 1 | -1.70133848 | -2.93842823 | 2.97150907 |
| 1 | -2.02893932 | 0.70846299 | 3.87004328 |

| | | | |
|---|---|---|---|
| 1 | -1.76394811 | 0.12309258 | 2.20810069 |
| 1 | -2.90853284 | -0.72121058 | 3.28512551 |
| 1 | -2.59674191 | 4.46008968 | -3.32709881 |
| 1 | -1.04329482 | 4.92524587 | -4.04623109 |
| 1 | -1.43726186 | 5.40161781 | -2.37237108 |
| 1 | -1.40597451 | 1.92009470 | -0.91856696 |
| 1 | -1.58595486 | 3.65082762 | -0.53983316 |
| 1 | -2.80384156 | 2.82990675 | -1.54249675 |
| 1 | -0.67808596 | 2.48754431 | -4.63065930 |
| 1 | -0.81962407 | 1.21909135 | -3.38633344 |
| 1 | -2.26506825 | 2.12741208 | -3.91134523 |
| 1 | 4.05638457 | -1.28100237 | -0.36327990 |
| 31 | 1.10433084 | -0.24045822 | -0.36350310 |
| 6 | -3.68682327 | -2.65675117 | -1.21977388 |
| 6 | -2.83239743 | -2.23270629 | -2.41874829 |
| 8 | -1.47801711 | -2.78463212 | -2.37683590 |
| 6 | -0.57556865 | -2.13096001 | -1.64242703 |
| 6 | 0.83135463 | -2.72032074 | -1.68202514 |
| 8 | -0.83247269 | -1.11325054 | -0.99208271 |
| 6 | 0.83239765 | -4.13602079 | -1.07858862 |
| 6 | -3.45965901 | -2.69398360 | -3.72795344 |
| 1 | -2.76186225 | -1.13686557 | -2.37648556 |
| 1 | 1.09571788 | -2.79775943 | -2.75756218 |
| 1 | 1.85445046 | -4.53171554 | -1.12595874 |
| 1 | 0.16259878 | -4.80774098 | -1.63147235 |
| 1 | 0.52244994 | -4.09804959 | -0.02584569 |
| 1 | -4.49045659 | -2.32431077 | -3.78052072 |
| 1 | -3.47043592 | -3.78995019 | -3.79043955 |
| 1 | -2.89249864 | -2.29396552 | -4.57711236 |

Electronic energy = -3673.067305 a.u.
DFT-D3(BJ) dispersion correction = -0.114489 a.u.
Thermal free energy = 0.582108 a.u.
Gibbs free energy = -3672.599686 a.u.
Number of imaginary frequencies = 0.

**Int2 LA isomer/conformer 3**

| Atomic N. | X | Y | Z |
|---|---|---|---|
| 8 | -4.22647237 | -0.26144962 | 1.99052974 |
| 6 | -4.57073679 | 0.63149242 | 3.08366416 |
| 1 | -4.09857883 | 1.60924616 | 2.93195053 |
| 8 | -5.24935152 | 1.18733482 | 0.56014670 |
| 8 | 0.03738211 | -2.66690431 | 0.65658091 |
| 1 | -4.18190402 | 0.14571941 | 3.98275109 |
| 1 | -5.65880232 | 0.75558144 | 3.14052737 |
| 7 | 2.63250516 | -1.53625059 | -0.08065570 |
| 8 | 0.81776623 | 0.51565045 | 1.03431039 |
| 8 | 0.38507285 | -0.77620395 | -1.86790561 |
| 6 | 3.02187001 | -2.50237274 | 0.97544856 |
| 6 | 1.90533528 | 0.83092267 | 1.77257895 |
| 6 | 1.74091605 | 1.59415784 | 2.96868220 |
| 6 | 2.89427237 | 1.90958657 | 3.70039178 |
| 6 | 4.17405385 | 1.50937591 | 3.30660777 |

| | | | |
|---|---|---|---|
| 6 | 4.32314879 | 0.77598697 | 2.13485989 |
| 6 | 3.20682386 | 0.44468919 | 1.35911597 |
| 6 | 3.40867988 | -0.25820505 | 0.04443606 |
| 1 | 5.04152045 | 1.77382047 | 3.91118465 |
| 6 | 0.34965628 | 2.05825909 | 3.44363632 |
| 6 | 1.36504279 | -0.62619705 | -2.78160489 |
| 6 | 1.12587154 | 0.16058089 | -3.94926507 |
| 6 | 2.17106870 | 0.27499358 | -4.87581093 |
| 6 | 3.41436041 | -0.33892984 | -4.69563174 |
| 6 | 3.63381175 | -1.10554631 | -3.55667632 |
| 6 | 2.61907080 | -1.26507294 | -2.60584914 |
| 6 | 2.85111617 | -2.17299264 | -1.42774772 |
| 1 | 4.19974894 | -0.21449059 | -5.44106193 |
| 6 | -0.22753175 | 0.86092903 | -4.18291921 |
| 6 | 0.42902824 | 2.88845663 | 4.73876424 |
| 6 | -0.54525469 | 0.83147516 | 3.73850065 |
| 6 | -0.31506669 | 2.94843365 | 2.36670225 |
| 6 | -0.25529104 | 1.63193758 | -5.51609041 |
| 6 | -0.50059733 | 1.88285653 | -3.05379871 |
| 6 | -1.36645271 | -0.18568074 | -4.23467232 |
| 1 | 4.09133420 | -2.75445841 | 0.87804632 |
| 1 | 2.84696365 | -2.06050174 | 1.96197307 |
| 1 | 4.48018980 | -0.47918124 | -0.10181409 |
| 1 | 3.08790907 | 0.37913218 | -0.79332282 |
| 1 | 2.79714361 | 2.48724891 | 4.61788127 |
| 1 | 5.31492213 | 0.46415308 | 1.79919234 |
| 1 | 2.16858023 | -3.03806825 | -1.45595145 |
| 1 | 3.88479967 | -2.55817745 | -1.46211941 |
| 1 | 2.01817310 | 0.87120689 | -5.77367018 |
| 1 | 4.59526570 | -1.59968264 | -3.39856060 |
| 1 | -0.58556113 | 3.20075459 | 5.02537047 |
| 1 | 0.84707494 | 2.31285362 | 5.57740675 |
| 1 | 1.03038172 | 3.79961604 | 4.60861980 |
| 1 | -0.70399565 | 0.22905310 | 2.83771602 |
| 1 | -0.09097369 | 0.19540667 | 4.51243289 |
| 1 | -1.52437196 | 1.16977066 | 4.11186790 |
| 1 | 0.29565279 | 3.84263168 | 2.17498091 |
| 1 | -0.44681718 | 2.40768204 | 1.42361855 |
| 1 | -1.30213373 | 3.28588250 | 2.71975170 |
| 1 | -1.24125206 | 2.10344198 | -5.63576316 |
| 1 | -0.09694753 | 0.97084656 | -6.38051941 |
| 1 | 0.49841357 | 2.43193356 | -5.54704133 |
| 1 | -0.54694097 | 1.39910632 | -2.07251291 |
| 1 | 0.28738407 | 2.64941738 | -3.02746895 |
| 1 | -1.45977295 | 2.39159583 | -3.23583576 |
| 1 | -1.20918111 | -0.88901526 | -5.06560660 |
| 1 | -1.42282538 | -0.75617871 | -3.30096544 |
| 1 | -2.32986864 | 0.32159054 | -4.39910649 |
| 1 | 2.40975299 | -3.40444743 | 0.87498296 |
| 31 | 0.60130374 | -1.01594655 | -0.01809767 |
| 6 | -4.62773141 | 0.16587951 | 0.77544919 |
| 6 | -4.24122176 | -0.78683413 | -0.36058181 |
| 8 | -3.43744697 | -1.92132788 | 0.09536447 |

| | | | |
|---|---|---|---|
| 6 | -2.12717461 | -1.71840786 | 0.24488558 |
| 6 | -1.33136282 | -2.94777274 | 0.67430811 |
| 8 | -1.57650105 | -0.63182514 | 0.04032871 |
| 6 | -1.79064609 | -3.41758695 | 2.06587474 |
| 6 | -5.47849415 | -1.36290219 | -1.03679462 |
| 1 | -3.64011997 | -0.19763795 | -1.06709961 |
| 1 | -1.57795884 | -3.74065238 | -0.06287474 |
| 1 | -1.20316455 | -4.30112459 | 2.34453592 |
| 1 | -2.85647848 | -3.68116724 | 2.06826241 |
| 1 | -1.60831952 | -2.63006360 | 2.80910396 |
| 1 | -6.12293579 | -0.53897012 | -1.36516003 |
| 1 | -6.04111717 | -2.00046884 | -0.34243837 |
| 1 | -5.18601228 | -1.95799443 | -1.91032904 |

Electronic energy = -3673.067279 a.u.
DFT-D3(BJ) dispersion correction = -0.114427 a.u.
Thermal free energy = 0.582278 a.u.
Gibbs free energy = -3672.599428 a.u.
Number of imaginary frequencies = 0.

**Int2 LA isomer/conformer 4**

| Atomic N. | X | Y | Z |
|---|---|---|---|
| 8 | -3.06603902 | -1.26655150 | -3.97320394 |
| 6 | -3.60263098 | -2.60385851 | -3.78781991 |
| 1 | -3.15979690 | -3.06329253 | -2.89645854 |
| 8 | -4.02409347 | -0.65853847 | -1.99633165 |
| 8 | -1.55419306 | 2.17105305 | 1.15091871 |
| 1 | -3.32138524 | -3.15246013 | -4.69107127 |
| 1 | -4.69263213 | -2.56308924 | -3.67407316 |
| 7 | 0.45415272 | 0.96272872 | 2.86280918 |
| 8 | 1.21437251 | 0.82213664 | 0.00416642 |
| 8 | -1.08046297 | -1.00944447 | 1.24547855 |
| 6 | 0.43373964 | 2.36464326 | 3.34643548 |
| 6 | 2.37483358 | 1.34085722 | 0.46244634 |
| 6 | 3.26968588 | 1.97984584 | -0.45009840 |
| 6 | 4.46418986 | 2.49906363 | 0.06822300 |
| 6 | 4.80367201 | 2.41263564 | 1.42108487 |
| 6 | 3.93310128 | 1.77394739 | 2.29736660 |
| 6 | 2.73267761 | 1.22724570 | 1.83121661 |
| 6 | 1.86088580 | 0.44768478 | 2.77723565 |
| 1 | 5.74169408 | 2.83669695 | 1.77935305 |
| 6 | 2.94340552 | 2.09399753 | -1.95248292 |
| 6 | -0.71924641 | -1.85532461 | 2.23224371 |
| 6 | -0.75656983 | -3.26516953 | 2.00467729 |
| 6 | -0.37819114 | -4.10184586 | 3.06351718 |
| 6 | 0.02484577 | -3.61406284 | 4.31005788 |
| 6 | 0.04669678 | -2.24082780 | 4.52399273 |
| 6 | -0.33158912 | -1.36014968 | 3.50405456 |
| 6 | -0.37577938 | 0.11607071 | 3.79072429 |
| 1 | 0.31616524 | -4.30497516 | 5.10111363 |
| 6 | -1.21104217 | -3.85167860 | 0.65386402 |
| 6 | 4.06956268 | 2.78985133 | -2.73988301 |
| 6 | 1.65849583 | 2.93142846 | -2.15165459 |

| | | | |
|---|---|---|---|
| 6 | 2.75734571 | 0.68540951 | -2.56552897 |
| 6 | -1.16965579 | -5.39171865 | 0.65195760 |
| 6 | -0.28379877 | -3.36635632 | -0.48531547 |
| 6 | -2.67093735 | -3.42887555 | 0.36694341 |
| 1 | 0.88236060 | 2.41934126 | 4.35289327 |
| 1 | 1.00717849 | 2.99949854 | 2.66323732 |
| 1 | 2.30291848 | 0.45627533 | 3.78873257 |
| 1 | 1.78528108 | -0.60319516 | 2.46057144 |
| 1 | 5.16088419 | 2.99509784 | -0.60519029 |
| 1 | 4.18464411 | 1.67833468 | 3.35621429 |
| 1 | -1.40324303 | 0.50565360 | 3.70421575 |
| 1 | -0.03222648 | 0.29768788 | 4.82374757 |
| 1 | -0.39235616 | -5.18009071 | 2.91531451 |
| 1 | 0.34930982 | -1.83569166 | 5.49242382 |
| 1 | 3.78835240 | 2.83438543 | -3.80200971 |
| 1 | 4.23902803 | 3.82158707 | -2.39922484 |
| 1 | 5.01975893 | 2.24091717 | -2.67235575 |
| 1 | 0.80595065 | 2.46685080 | -1.64530581 |
| 1 | 1.79295381 | 3.94804304 | -1.75377122 |
| 1 | 1.42837346 | 3.01545863 | -3.22529398 |
| 1 | 3.68336690 | 0.09985284 | -2.47098747 |
| 1 | 1.94808882 | 0.13691799 | -2.07138119 |
| 1 | 2.52316722 | 0.77330301 | -3.63806831 |
| 1 | -1.50292151 | -5.75604340 | -0.33056063 |
| 1 | -1.83896226 | -5.82536750 | 1.40902880 |
| 1 | -0.15471170 | -5.77986962 | 0.82103047 |
| 1 | -0.30255418 | -2.27624709 | -0.58438774 |
| 1 | 0.75361187 | -3.68281505 | -0.30300745 |
| 1 | -0.60618565 | -3.80983562 | -1.44068800 |
| 1 | -3.33877208 | -3.77803514 | 1.16803343 |
| 1 | -2.77012993 | -2.34197016 | 0.27758356 |
| 1 | -3.01285022 | -3.88857112 | -0.57369096 |
| 1 | -0.60202799 | 2.71717320 | 3.37374082 |
| 31 | -0.40139417 | 0.71343617 | 0.95730925 |
| 6 | -3.36565343 | -0.39702150 | -2.97913913 |
| 6 | -2.83184780 | 1.00137729 | -3.33332416 |
| 8 | -2.92577203 | 1.86569515 | -2.15835470 |
| 6 | -2.19189007 | 1.57469057 | -1.08228672 |
| 6 | -2.41613283 | 2.50774583 | 0.10468765 |
| 8 | -1.38964404 | 0.63842034 | -1.02158526 |
| 6 | -2.26125672 | 3.98296434 | -0.29924495 |
| 6 | -1.47053535 | 1.05686001 | -4.01724778 |
| 1 | -3.58837150 | 1.44459355 | -3.99954352 |
| 1 | -3.47262488 | 2.33495671 | 0.40325534 |
| 1 | -2.44091013 | 4.60493452 | 0.58617864 |
| 1 | -2.97910843 | 4.26232734 | -1.08130353 |
| 1 | -1.24030189 | 4.17499229 | -0.65610773 |
| 1 | -1.54132924 | 0.55758647 | -4.99102271 |
| 1 | -0.69378946 | 0.56618202 | -3.42320336 |
| 1 | -1.18920443 | 2.10453590 | -4.18587051 |

Electronic energy = -3673.063398 a.u.
DFT-D3(BJ) dispersion correction = -0.116319 a.u.
Thermal free energy = 0.582924 a.u.

Gibbs free energy = -3672.596793 a.u.
Number of imaginary frequencies = 0.

**Int2 LA isomer/conformer 5**

| Atomic N. | X | Y | Z |
|---|---|---|---|
| 8 | -1.64836557 | -1.07234900 | 4.40887460 |
| 6 | -2.58742798 | -2.17924914 | 4.47804411 |
| 1 | -3.32268542 | -1.99688273 | 5.27123903 |
| 8 | -3.39595315 | 0.32010669 | 3.96263800 |
| 8 | 2.27433642 | -0.72606285 | 1.52363104 |
| 1 | -1.97603267 | -3.05741841 | 4.70329333 |
| 1 | -3.10185331 | -2.29908119 | 3.51827534 |
| 7 | 2.28702454 | -1.39316637 | -1.29363068 |
| 8 | 0.67736374 | 1.04269145 | -0.71460653 |
| 8 | -0.35363973 | -1.86425809 | -0.02129672 |
| 6 | 3.72709861 | -1.19975946 | -0.99621854 |
| 6 | 1.45078603 | 1.57849007 | -1.68574506 |
| 6 | 1.54658205 | 2.99883971 | -1.80681846 |
| 6 | 2.36579057 | 3.50939682 | -2.82345673 |
| 6 | 3.07195652 | 2.69194808 | -3.70994908 |
| 6 | 2.94848867 | 1.31123869 | -3.60109668 |
| 6 | 2.13594293 | 0.74790609 | -2.61111679 |
| 6 | 1.92127625 | -0.74133274 | -2.59523131 |
| 1 | 3.70197772 | 3.13501202 | -4.48119582 |
| 6 | 0.77142985 | 3.94377785 | -0.86690280 |
| 6 | -0.52457163 | -2.69016619 | -1.07580580 |
| 6 | -1.84373225 | -3.05311978 | -1.48621275 |
| 6 | -1.96392593 | -3.94472156 | -2.56103578 |
| 6 | -0.85968419 | -4.46860819 | -3.23956727 |
| 6 | 0.41892091 | -4.09373984 | -2.84319465 |
| 6 | 0.59630640 | -3.21864535 | -1.76543360 |
| 6 | 1.99137112 | -2.87025819 | -1.32353412 |
| 1 | -1.00415508 | -5.15474710 | -4.07404051 |
| 6 | -3.09158804 | -2.47788052 | -0.78904600 |
| 6 | 1.00091591 | 5.42632959 | -1.21604509 |
| 6 | 1.23645966 | 3.73930290 | 0.59346543 |
| 6 | -0.74830429 | 3.67474988 | -0.97863216 |
| 6 | -4.39769110 | -3.02144700 | -1.39807765 |
| 6 | -3.11890532 | -0.93804708 | -0.93881882 |
| 6 | -3.08877687 | -2.86024457 | 0.70933406 |
| 1 | 4.33705735 | -1.65245427 | -1.79636391 |
| 1 | 3.95092335 | -0.12984490 | -0.93557394 |
| 1 | 2.50662105 | -1.21325611 | -3.40345513 |
| 1 | 0.86253921 | -0.98104323 | -2.77272632 |
| 1 | 2.46251955 | 4.58814262 | -2.93200411 |
| 1 | 3.47060989 | 0.65205504 | -4.29853940 |
| 1 | 2.18766114 | -3.23042332 | -0.30047026 |
| 1 | 2.71959786 | -3.35480951 | -1.99650426 |
| 1 | -2.95768671 | -4.23956093 | -2.89326180 |
| 1 | 1.29697104 | -4.48374564 | -3.36320595 |
| 1 | 0.41691484 | 6.05066468 | -0.52455577 |
| 1 | 2.05612897 | 5.71901214 | -1.11497286 |

| | | | |
|---|---|---|---|
| 1 | 0.66992527 | 5.66552962 | -2.23679213 |
| 1 | 1.05472517 | 2.71000496 | 0.91949205 |
| 1 | 2.30983068 | 3.95822951 | 0.69347245 |
| 1 | 0.68734912 | 4.42185371 | 1.26058590 |
| 1 | -1.09793775 | 3.86396790 | -2.00388825 |
| 1 | -0.99360324 | 2.64055879 | -0.71379945 |
| 1 | -1.29762682 | 4.35136199 | -0.30563728 |
| 1 | -5.25089562 | -2.58291753 | -0.86107186 |
| 1 | -4.47453164 | -4.11468650 | -1.30615856 |
| 1 | -4.50296408 | -2.75310988 | -2.45916801 |
| 1 | -2.23858372 | -0.47146367 | -0.48465649 |
| 1 | -3.15529121 | -0.65420222 | -2.00062010 |
| 1 | -4.01664438 | -0.53207435 | -0.44832139 |
| 1 | -3.11821364 | -3.95341775 | 0.83062887 |
| 1 | -2.19429447 | -2.47422650 | 1.21031525 |
| 1 | -3.98152532 | -2.43867704 | 1.19716764 |
| 1 | 3.95946714 | -1.66523054 | -0.03347788 |
| 31 | 1.00150513 | -0.58444979 | 0.16332750 |
| 6 | -2.21064687 | 0.12600760 | 4.13897351 |
| 6 | -1.18646464 | 1.26870332 | 4.21162407 |
| 8 | 0.19551467 | 0.80774870 | 4.02064583 |
| 6 | 0.55257582 | 0.29343251 | 2.84435340 |
| 6 | 1.99092991 | -0.21570531 | 2.79182358 |
| 8 | -0.19684081 | 0.20462898 | 1.86677797 |
| 6 | 2.99400063 | 0.87356133 | 3.20670748 |
| 6 | -1.55464603 | 2.47175076 | 3.35675385 |
| 1 | -1.16867234 | 1.56808111 | 5.27150552 |
| 1 | 2.02952915 | -1.02942680 | 3.54775665 |
| 1 | 4.00399350 | 0.44754594 | 3.16686915 |
| 1 | 2.79881900 | 1.23001097 | 4.22658011 |
| 1 | 2.94712012 | 1.72111574 | 2.51000594 |
| 1 | -2.53707391 | 2.83743725 | 3.67899359 |
| 1 | -1.60946311 | 2.21503552 | 2.29463801 |
| 1 | -0.81595862 | 3.26968061 | 3.50414127 |

Electronic energy = -3673.063689 a.u.
DFT-D3(BJ) dispersion correction = -0.116638 a.u.
Thermal free energy = 0.583734 a.u.
Gibbs free energy = -3672.596592 a.u.
Number of imaginary frequencies = 0.

**Int2 LA isomer/conformer 6**

| Atomic N. | X | Y | Z |
|---|---|---|---|
| 8 | -0.69874591 | -5.23350099 | -3.10500035 |
| 6 | -1.38033599 | -6.41606017 | -3.59892439 |
| 1 | -0.81375438 | -7.31798172 | -3.33742998 |
| 8 | -0.91908083 | -6.05082578 | -0.98800453 |
| 8 | 0.02977558 | -1.17202891 | 2.51239389 |
| 1 | -1.42982969 | -6.28638645 | -4.68360995 |
| 1 | -2.38580637 | -6.48145705 | -3.16614810 |
| 7 | 0.31030096 | 1.73206678 | 2.66857410 |
| 8 | -1.17058849 | 0.89880084 | 0.24191625 |
| 8 | 1.97081381 | 0.44046044 | 0.56029561 |

| | | | |
|---|---|---|---|
| 6 | -0.42387169 | 1.41357644 | 3.91788377 |
| 6 | -2.07924231 | 1.79160499 | 0.69448407 |
| 6 | -3.42408964 | 1.73202414 | 0.21806917 |
| 6 | -4.32803817 | 2.68256064 | 0.71295260 |
| 6 | -3.96297539 | 3.66768340 | 1.63480888 |
| 6 | -2.64578619 | 3.72732352 | 2.07702171 |
| 6 | -1.70007789 | 2.80982931 | 1.60700498 |
| 6 | -0.25881077 | 2.95615792 | 2.01159490 |
| 1 | -4.70314970 | 4.38206495 | 1.99522497 |
| 6 | -3.86994792 | 0.67064058 | -0.80749861 |
| 6 | 2.70474713 | 1.56707524 | 0.67010867 |
| 6 | 3.59178861 | 1.94115695 | -0.38453957 |
| 6 | 4.34176803 | 3.11261089 | -0.21242528 |
| 6 | 4.25026777 | 3.91002719 | 0.93239431 |
| 6 | 3.38827645 | 3.53161688 | 1.95564011 |
| 6 | 2.62528543 | 2.36394795 | 1.84065290 |
| 6 | 1.76718864 | 1.92625861 | 2.99709033 |
| 1 | 4.84936361 | 4.81656831 | 1.01761700 |
| 6 | 3.72227146 | 1.09183920 | -1.66426792 |
| 6 | -5.35372233 | 0.82445173 | -1.19111954 |
| 6 | -3.69227953 | -0.74790969 | -0.21690374 |
| 6 | -3.04144874 | 0.80685767 | -2.10731984 |
| 6 | 4.75001725 | 1.68298135 | -2.64753768 |
| 6 | 2.36395859 | 1.02589795 | -2.40200595 |
| 6 | 4.19894047 | -0.33570337 | -1.30464893 |
| 1 | -0.33079322 | 2.25067879 | 4.63034440 |
| 1 | -1.48270894 | 1.25108387 | 3.69157330 |
| 1 | -0.14601940 | 3.81083451 | 2.70109879 |
| 1 | 0.37333819 | 3.15463761 | 1.13329558 |
| 1 | -5.36138077 | 2.65667200 | 0.37166634 |
| 1 | -2.33266002 | 4.50022447 | 2.78282822 |
| 1 | 2.10963151 | 0.95736836 | 3.39547597 |
| 1 | 1.84049917 | 2.66982746 | 3.80940122 |
| 1 | 5.02279133 | 3.42474932 | -1.00224455 |
| 1 | 3.30677237 | 4.13493791 | 2.86277596 |
| 1 | -5.61404128 | 0.05133454 | -1.92830664 |
| 1 | -6.02183992 | 0.69588264 | -0.32716002 |
| 1 | -5.56211482 | 1.80189942 | -1.64948361 |
| 1 | -2.64368326 | -0.94007633 | 0.03320864 |
| 1 | -4.29987997 | -0.86955502 | 0.69185624 |
| 1 | -4.02195136 | -1.50153605 | -0.94879564 |
| 1 | -3.19150034 | 1.79895470 | -2.55747859 |
| 1 | -1.97166781 | 0.66849679 | -1.91703195 |
| 1 | -3.36967938 | 0.05181053 | -2.83847179 |
| 1 | 4.80929357 | 1.03599015 | -3.53455816 |
| 1 | 5.75745516 | 1.73818264 | -2.21019011 |
| 1 | 4.46403510 | 2.68773037 | -2.99066145 |
| 1 | 1.58843650 | 0.56440263 | -1.78203103 |
| 1 | 2.02846424 | 2.03406772 | -2.68529137 |
| 1 | 2.47034688 | 0.43455291 | -3.32474803 |
| 1 | 5.19070463 | -0.30476381 | -0.83007503 |
| 1 | 3.49986060 | -0.82503063 | -0.61734510 |
| 1 | 4.28191806 | -0.94237341 | -2.22006759 |

| | | | |
|---|---|---|---|
| 1 | -0.00917711 | 0.49847479 | 4.35222628 |
| 31 | 0.24224434 | 0.18510280 | 1.24515946 |
| 6 | -0.53327094 | -5.19862640 | -1.75740134 |
| 6 | 0.23285675 | -3.92452866 | -1.39033343 |
| 8 | 0.13568524 | -3.80433319 | 0.06181625 |
| 6 | 0.11227475 | -2.57133454 | 0.56608231 |
| 6 | 0.01562787 | -2.50318952 | 2.08726989 |
| 8 | 0.16675634 | -1.54726339 | -0.12397140 |
| 6 | -1.23522335 | -3.24654107 | 2.58621374 |
| 6 | 1.69799393 | -3.98765023 | -1.81426695 |
| 1 | -0.26368191 | -3.05905536 | -1.84911836 |
| 1 | 0.91274566 | -3.04029637 | 2.46302450 |
| 1 | -1.25824257 | -3.19070794 | 3.68144560 |
| 1 | -1.22178574 | -4.30072153 | 2.28058021 |
| 1 | -2.14201119 | -2.76532337 | 2.19569610 |
| 1 | 1.76063588 | -4.11640241 | -2.90193412 |
| 1 | 2.20416905 | -4.82981432 | -1.32484466 |
| 1 | 2.20559426 | -3.05300316 | -1.54557191 |

Electronic energy = -3673.066221 a.u.
DFT-D3(BJ) dispersion correction = -0.113377 a.u.
Thermal free energy = 0.583413 a.u.
Gibbs free energy = -3672.596185 a.u.
Number of imaginary frequencies = 0.

**Int2 LA isomer/conformer 7**

| Atomic N. | X | Y | Z |
|---|---|---|---|
| 8 | 1.92229188 | 4.08069160 | 2.12126785 |
| 6 | 2.97215458 | 4.47684123 | 1.19969404 |
| 1 | 2.57265726 | 4.54019321 | 0.18067573 |
| 8 | 0.79857289 | 5.99130661 | 1.59089700 |
| 8 | -0.97836419 | -0.10713565 | 1.99198864 |
| 1 | 3.72775763 | 3.68976727 | 1.27149405 |
| 1 | 3.38445846 | 5.45029554 | 1.49154965 |
| 7 | -2.01128409 | 0.68147766 | -1.08631369 |
| 8 | -0.86549186 | -1.94869968 | -0.31091460 |
| 8 | 0.94569253 | 0.53183377 | -0.45769501 |
| 6 | -3.22336878 | 1.20546374 | -0.40516736 |
| 6 | -2.10054324 | -2.41223031 | -0.62516597 |
| 6 | -2.54512440 | -3.67958263 | -0.14916506 |
| 6 | -3.83592684 | -4.08461779 | -0.52019227 |
| 6 | -4.67537985 | -3.30864589 | -1.32536801 |
| 6 | -4.21871687 | -2.08540150 | -1.80566483 |
| 6 | -2.93530617 | -1.63946096 | -1.47369461 |
| 6 | -2.38223118 | -0.38472634 | -2.08959824 |
| 1 | -5.67271529 | -3.66732587 | -1.57988865 |
| 6 | -1.64946259 | -4.57027866 | 0.73334856 |
| 6 | 1.06689937 | 0.81758474 | -1.78103896 |
| 6 | 2.29367268 | 0.54634470 | -2.45530959 |
| 6 | 2.36591772 | 0.87224943 | -3.81724663 |
| 6 | 1.29807421 | 1.43779381 | -4.51869219 |
| 6 | 0.11048346 | 1.70479345 | -3.84734233 |
| 6 | -0.01377233 | 1.41355587 | -2.48354557 |

| | | | |
|---|---|---|---|
| 6 | -1.27722522 | 1.81744681 | -1.76456680 |
| 1 | 1.39926077 | 1.66599880 | -5.57961012 |
| 6 | 3.49880572 | -0.07913943 | -1.72454088 |
| 6 | -2.32694919 | -5.91157057 | 1.07187876 |
| 6 | -1.34335834 | -3.85605753 | 2.07087367 |
| 6 | -0.33057582 | -4.89565918 | -0.00772473 |
| 6 | 4.70877115 | -0.26374755 | -2.66013062 |
| 6 | 3.12904840 | -1.47710928 | -1.17517748 |
| 6 | 3.95168900 | 0.84497171 | -0.56918164 |
| 1 | -3.89306484 | 1.66915970 | -1.14744283 |
| 1 | -3.74818268 | 0.37977520 | 0.08772747 |
| 1 | -3.10954526 | 0.05234227 | -2.79401008 |
| 1 | -1.45939422 | -0.60512274 | -2.64540980 |
| 1 | -4.20927421 | -5.04358287 | -0.16507793 |
| 1 | -4.84656306 | -1.47531820 | -2.45933592 |
| 1 | -1.06035805 | 2.54242219 | -0.96814678 |
| 1 | -1.97298704 | 2.28597543 | -2.48017769 |
| 1 | 3.28774111 | 0.67120482 | -4.35980671 |
| 1 | -0.73289588 | 2.15709363 | -4.37392378 |
| 1 | -1.64137597 | -6.51198649 | 1.68680024 |
| 1 | -3.25317118 | -5.77530191 | 1.64888519 |
| 1 | -2.56094055 | -6.49596463 | 0.17011776 |
| 1 | -0.83126577 | -2.90041322 | 1.91622298 |
| 1 | -2.27239492 | -3.66083021 | 2.62614703 |
| 1 | -0.70359662 | -4.49821951 | 2.69586732 |
| 1 | -0.53560417 | -5.44015997 | -0.94118729 |
| 1 | 0.22945801 | -3.98633433 | -0.25095763 |
| 1 | 0.30143433 | -5.53723039 | 0.62525244 |
| 1 | 5.53887812 | -0.70080193 | -2.08704200 |
| 1 | 5.06097159 | 0.69108089 | -3.07663797 |
| 1 | 4.48765402 | -0.94692872 | -3.49272478 |
| 1 | 2.30950751 | -1.42767391 | -0.45116258 |
| 1 | 2.82538511 | -2.14612095 | -1.99302811 |
| 1 | 4.00390176 | -1.92418353 | -0.67902503 |
| 1 | 4.28310171 | 1.81735214 | -0.96324021 |
| 1 | 3.14066836 | 1.01596340 | 0.14709403 |
| 1 | 4.80014809 | 0.38905646 | -0.03638174 |
| 1 | -2.92113570 | 1.94820147 | 0.33999648 |
| 31 | -0.65120152 | -0.16245003 | 0.19372747 |
| 6 | 0.88130138 | 4.93728749 | 2.19056120 |
| 6 | -0.19907400 | 4.48151759 | 3.17799246 |
| 8 | -0.13695970 | 3.06209060 | 3.48996096 |
| 6 | -0.47221787 | 2.22025070 | 2.48096168 |
| 6 | -0.33288328 | 0.75248765 | 2.89494834 |
| 8 | -0.83839598 | 2.59773602 | 1.37881430 |
| 6 | 1.15094869 | 0.40326338 | 3.10655320 |
| 6 | -0.07089735 | 5.23191000 | 4.49903310 |
| 1 | -1.16065848 | 4.69669302 | 2.69260837 |
| 1 | -0.85298851 | 0.65614364 | 3.86601787 |
| 1 | 1.22032110 | -0.64695804 | 3.41686645 |
| 1 | 1.60101029 | 1.03732714 | 3.88146351 |
| 1 | 1.71267027 | 0.53309065 | 2.17112897 |
| 1 | -0.11450554 | 6.31158222 | 4.30996411 |

| | | | |
|---|---|---|---|
| 1 | 0.88081715 | 4.99262507 | 4.99091706 |
| 1 | -0.89479764 | 4.95472771 | 5.16835678 |

Electronic energy = -3673.061232 a.u.
DFT-D3(BJ) dispersion correction = -0.114989 a.u.
Thermal free energy = 0.582169 a.u.
Gibbs free energy = -3672.594052 a.u.
Number of imaginary frequencies = 0.

**Int2 LA isomer/conformer 8**

| Atomic N. | X | Y | Z |
|---|---|---|---|
| 7 | -1.48557771 | 1.10436561 | 1.36818047 |
| 8 | -0.57687473 | 1.63018255 | -1.41199482 |
| 8 | 1.08097147 | -0.19431231 | 0.42906058 |
| 6 | -2.87730912 | 0.65379790 | 1.62544606 |
| 6 | -1.60787884 | 2.51034933 | -1.42299158 |
| 6 | -2.14099058 | 2.99043431 | -2.65438976 |
| 6 | -3.20609610 | 3.90011259 | -2.57870007 |
| 6 | -3.74377796 | 4.34368037 | -1.36654708 |
| 6 | -3.19716347 | 3.88711552 | -0.17145357 |
| 6 | -2.12671220 | 2.98710605 | -0.19095073 |
| 6 | -1.44593454 | 2.58733494 | 1.08831704 |
| 1 | -4.57613341 | 5.04747400 | -1.36302543 |
| 6 | -1.57430942 | 2.53292587 | -4.01232520 |
| 6 | 1.63638213 | 0.61827502 | 1.36515708 |
| 6 | 3.03262872 | 0.90399152 | 1.31600952 |
| 6 | 3.55266057 | 1.74443679 | 2.31060661 |
| 6 | 2.76551156 | 2.29832009 | 3.32379432 |
| 6 | 1.40844137 | 2.00064785 | 3.36799229 |
| 6 | 0.83704675 | 1.15552658 | 2.40885211 |
| 6 | -0.61198163 | 0.76249599 | 2.55479268 |
| 1 | 3.21450487 | 2.95464972 | 4.06922288 |
| 6 | 3.94229815 | 0.31281106 | 0.22055570 |
| 6 | -2.27451284 | 3.23196608 | -5.19288561 |
| 6 | -1.78141103 | 1.01002358 | -4.18752789 |
| 6 | -0.06825832 | 2.87668416 | -4.10219640 |
| 6 | 5.40733519 | 0.76038317 | 0.38551925 |
| 6 | 3.47758397 | 0.78355745 | -1.17792053 |
| 6 | 3.92456193 | -1.23220421 | 0.29850490 |
| 1 | -3.27372418 | 1.16658514 | 2.51690898 |
| 1 | -3.50397457 | 0.89762716 | 0.76063392 |
| 1 | -1.90340329 | 3.10662369 | 1.94711653 |
| 1 | -0.38006638 | 2.85600282 | 1.05626407 |
| 1 | -3.64177982 | 4.27866637 | -3.50173821 |
| 1 | -3.58294325 | 4.24123900 | 0.78746128 |
| 1 | -0.71078004 | -0.32403172 | 2.68520004 |
| 1 | -1.04018992 | 1.26024093 | 3.44084611 |
| 1 | 4.61367783 | 1.98641752 | 2.29432703 |
| 1 | 0.77692640 | 2.41225775 | 4.15868506 |
| 1 | -1.82286950 | 2.88289739 | -6.13246433 |
| 1 | -3.34818247 | 2.99794798 | -5.23675723 |
| 1 | -2.15734417 | 4.32480546 | -5.15496974 |
| 1 | -1.28485789 | 0.43651273 | -3.39764865 |

| | | | |
|---|---|---|---|
| 1 | -2.85253881 | 0.76112117 | -4.16982781 |
| 1 | -1.37500326 | 0.69004678 | -5.15947277 |
| 1 | 0.08629572 | 3.96257687 | -4.01938259 |
| 1 | 0.50565098 | 2.38212948 | -3.31125015 |
| 1 | 0.32798873 | 2.55286152 | -5.07678512 |
| 1 | 6.01047206 | 0.30354992 | -0.41196101 |
| 1 | 5.83344717 | 0.43873140 | 1.34687328 |
| 1 | 5.52011250 | 1.85099246 | 0.30091470 |
| 1 | 2.45826592 | 0.45340653 | -1.40199891 |
| 1 | 3.50760935 | 1.88050280 | -1.24780967 |
| 1 | 4.15055676 | 0.37642065 | -1.94779209 |
| 1 | 4.32126196 | -1.57506045 | 1.26524156 |
| 1 | 2.91255672 | -1.63365209 | 0.18182874 |
| 1 | 4.56066744 | -1.65269626 | -0.49521875 |
| 1 | -2.87748009 | -0.42909354 | 1.78688315 |
| 8 | -0.05314078 | -4.81551715 | 3.82547983 |
| 6 | 1.24380715 | -4.75672108 | 4.47534810 |
| 1 | 1.74745273 | -3.81532681 | 4.22535636 |
| 8 | 1.00924462 | -4.65602517 | 1.81428049 |
| 8 | -1.56071507 | -1.13774232 | -1.08172580 |
| 1 | 1.03178086 | -4.81461552 | 5.54672156 |
| 1 | 1.86936558 | -5.59841957 | 4.15368802 |
| 31 | -0.61142479 | 0.17705287 | -0.23781304 |
| 6 | -0.00494776 | -4.75504201 | 2.47056640 |
| 6 | -1.42359800 | -4.89319334 | 1.90063391 |
| 8 | -1.46557791 | -4.33888288 | 0.56273462 |
| 6 | -1.34709240 | -2.98814911 | 0.48100168 |
| 6 | -1.29520963 | -2.51293888 | -0.97282934 |
| 8 | -1.28852743 | -2.25575087 | 1.45596904 |
| 6 | 0.04649030 | -2.92550541 | -1.60384386 |
| 6 | -1.82605796 | -6.35940439 | 1.78674356 |
| 1 | -2.11582717 | -4.33589009 | 2.54604606 |
| 1 | -2.10682492 | -3.04428606 | -1.50291234 |
| 1 | 0.05769975 | -2.58816592 | -2.64805822 |
| 1 | 0.18006783 | -4.01451673 | -1.57460591 |
| 1 | 0.88327554 | -2.45639351 | -1.06833281 |
| 1 | -1.82390954 | -6.82387619 | 2.78097304 |
| 1 | -1.12609884 | -6.90049537 | 1.13725597 |
| 1 | -2.83558878 | -6.43795550 | 1.36411243 |

Electronic energy = -3673.060709 a.u.
DFT-D3(BJ) dispersion correction = -0.113851 a.u.
Thermal free energy = 0.581250 a.u.
Gibbs free energy = -3672.593310 a.u.
Number of imaginary frequencies = 0.

**Int2 LA isomer/conformer 9**

| Atomic N. | X | Y | Z |
|---|---|---|---|
| 7 | -0.12384673 | 1.77459485 | -1.40066355 |
| 8 | -0.78921005 | -0.54059981 | 0.43460578 |
| 8 | -0.56267528 | -0.94576007 | -2.52313255 |
| 6 | 0.94191851 | 2.75744570 | -1.08392406 |
| 6 | -1.03095033 | 0.43961093 | 1.33570659 |

| | | | |
|---|---|---|---|
| 6 | -1.12633000 | 0.13089566 | 2.72381563 |
| 6 | -1.33886717 | 1.19969480 | 3.60704376 |
| 6 | -1.47577618 | 2.52283786 | 3.17695604 |
| 6 | -1.43686984 | 2.80248918 | 1.81412552 |
| 6 | -1.23402473 | 1.77284954 | 0.88983622 |
| 6 | -1.36344219 | 2.05309950 | -0.58408363 |
| 1 | -1.63500597 | 3.32012917 | 3.90305693 |
| 6 | -1.02897854 | -1.32075026 | 3.23512147 |
| 6 | -1.58577928 | -0.36543636 | -3.17214013 |
| 6 | -2.64097898 | -1.15915020 | -3.71711333 |
| 6 | -3.66943223 | -0.48713045 | -4.39089596 |
| 6 | -3.69754536 | 0.90332160 | -4.54533778 |
| 6 | -2.65899791 | 1.66534168 | -4.02256970 |
| 6 | -1.60040411 | 1.04230594 | -3.35078234 |
| 6 | -0.43301307 | 1.86518945 | -2.88355498 |
| 1 | -4.52450378 | 1.37881386 | -5.07263317 |
| 6 | -2.64810255 | -2.69265704 | -3.56923115 |
| 6 | -1.20864815 | -1.40716991 | 4.76242118 |
| 6 | 0.35259636 | -1.93008128 | 2.90082805 |
| 6 | -2.14440222 | -2.17698810 | 2.58865958 |
| 6 | -3.86300421 | -3.33289834 | -4.26656362 |
| 6 | -2.71735157 | -3.08040249 | -2.07305018 |
| 6 | -1.37558589 | -3.29421982 | -4.21231464 |
| 1 | 0.61693419 | 3.76235422 | -1.39951542 |
| 1 | 1.14006739 | 2.75214323 | -0.00969067 |
| 1 | -1.64891127 | 3.10593104 | -0.74747093 |
| 1 | -2.15130304 | 1.42193554 | -1.02011447 |
| 1 | -1.40236408 | 0.99841998 | 4.67507977 |
| 1 | -1.58983707 | 3.82246919 | 1.45331062 |
| 1 | 0.49498258 | 1.55712569 | -3.39235366 |
| 1 | -0.61320697 | 2.92673109 | -3.12102675 |
| 1 | -4.49021140 | -1.06589195 | -4.81121298 |
| 1 | -2.65285557 | 2.75142251 | -4.14220460 |
| 1 | -1.14838564 | -2.46065720 | 5.07089300 |
| 1 | -0.42485188 | -0.85808372 | 5.30489682 |
| 1 | -2.18717256 | -1.02375339 | 5.08506054 |
| 1 | 0.52589965 | -1.95290647 | 1.81957373 |
| 1 | 1.15690336 | -1.35138820 | 3.37995487 |
| 1 | 0.40755177 | -2.96108588 | 3.28209286 |
| 1 | -3.13592837 | -1.78301800 | 2.85472521 |
| 1 | -2.05732668 | -2.19192829 | 1.49668359 |
| 1 | -2.08178441 | -3.21200108 | 2.95811815 |
| 1 | -3.81297052 | -4.42386341 | -4.14011063 |
| 1 | -3.87686600 | -3.12514359 | -5.34645713 |
| 1 | -4.81587832 | -2.99412139 | -3.83437753 |
| 1 | -1.85845974 | -2.69248233 | -1.51544202 |
| 1 | -3.63578124 | -2.68738571 | -1.61301042 |
| 1 | -2.73034984 | -4.17665091 | -1.97337338 |
| 1 | -1.33881666 | -3.06084186 | -5.28669398 |
| 1 | -0.46619082 | -2.90781786 | -3.73971243 |
| 1 | -1.38861779 | -4.38981380 | -4.10538274 |
| 1 | 1.86093092 | 2.48892785 | -1.61827547 |
| 8 | 3.71575351 | 3.06157164 | 3.14230077 |

| | | | |
|---|---|---|---|
| 6 | 3.99447349 | 4.44475668 | 2.80496836 |
| 1 | 5.06646701 | 4.58326177 | 2.61874349 |
| 8 | 4.56549317 | 2.44308456 | 1.11961233 |
| 8 | 1.93941318 | -1.02603821 | -1.36453739 |
| 1 | 3.67058533 | 5.02361258 | 3.67435042 |
| 1 | 3.43040440 | 4.73552499 | 1.91042679 |
| 31 | 0.32306570 | -0.21671395 | -1.03561724 |
| 6 | 4.06414790 | 2.16354203 | 2.18808720 |
| 6 | 3.75298900 | 0.74800805 | 2.69361279 |
| 8 | 3.83929525 | -0.19399423 | 1.59356411 |
| 6 | 2.85714665 | -0.12105355 | 0.67428808 |
| 6 | 2.99545673 | -1.13460256 | -0.45513935 |
| 8 | 1.91848913 | 0.66353743 | 0.74639004 |
| 6 | 4.34019652 | -0.97001336 | -1.18027187 |
| 6 | 4.75521477 | 0.29057997 | 3.74718247 |
| 1 | 2.72762534 | 0.74495947 | 3.08909258 |
| 1 | 2.98330097 | -2.13125190 | 0.03626715 |
| 1 | 4.38772745 | -1.70628605 | -1.99168961 |
| 1 | 5.18283201 | -1.13093119 | -0.49611412 |
| 1 | 4.41775924 | 0.03556839 | -1.61426449 |
| 1 | 4.71743995 | 0.96498519 | 4.61116557 |
| 1 | 5.77389904 | 0.28791519 | 3.33834793 |
| 1 | 4.50483569 | -0.72402380 | 4.08108410 |

Electronic energy = -3673.058632 a.u.
DFT-D3(BJ) dispersion correction = -0.115674 a.u.
Thermal free energy = 0.581408 a.u.
Gibbs free energy = -3672.592899 a.u.
Number of imaginary frequencies = 0.

**Int2 LA isomer/conformer 10**

| Atomic N. | X | Y | Z |
|---|---|---|---|
| 7 | -0.32020656 | 0.22841166 | 2.29266583 |
| 8 | 0.72199743 | -0.68198252 | -0.40230239 |
| 8 | -1.81036918 | -1.84308118 | 0.76210675 |
| 6 | -0.36150266 | 1.66355433 | 2.66815605 |
| 6 | 1.86378018 | -0.07477129 | 0.00282261 |
| 6 | 2.89022919 | 0.21997603 | -0.94044436 |
| 6 | 4.02654598 | 0.89066232 | -0.46426106 |
| 6 | 4.18883081 | 1.24969128 | 0.87673136 |
| 6 | 3.20646751 | 0.89977663 | 1.79771780 |
| 6 | 2.05586833 | 0.22485193 | 1.37740644 |
| 6 | 1.08799082 | -0.30752668 | 2.40053630 |
| 1 | 5.08601007 | 1.77951140 | 1.19642658 |
| 6 | 2.77721578 | -0.20804444 | -2.41781575 |
| 6 | -1.54465444 | -2.62474426 | 1.82433432 |
| 6 | -1.62416678 | -4.04611868 | 1.71456804 |
| 6 | -1.34547503 | -4.79589645 | 2.86519752 |
| 6 | -1.00017271 | -4.21260216 | 4.08920520 |
| 6 | -0.93778383 | -2.82737697 | 4.18786346 |
| 6 | -1.21929510 | -2.03126370 | 3.07115003 |
| 6 | -1.25118596 | -0.53575574 | 3.21610845 |
| 1 | -0.78575749 | -4.84098590 | 4.95355758 |

| | | | |
|---|---|---|---|
| 6 | -1.99986465 | -4.72831071 | 0.38522632 |
| 6 | 4.03066846 | 0.17581181 | -3.22650856 |
| 6 | 1.56730240 | 0.47359623 | -3.09752112 |
| 6 | 2.62364280 | -1.74564562 | -2.50389933 |
| 6 | -2.05517260 | -6.26155286 | 0.52034947 |
| 6 | -0.94432864 | -4.39840118 | -0.69676506 |
| 6 | -3.39798362 | -4.25410948 | -0.07845385 |
| 1 | -0.09887622 | 1.76894898 | 3.73334742 |
| 1 | 0.34571457 | 2.22531400 | 2.05385423 |
| 1 | 1.45567448 | -0.09340861 | 3.41804173 |
| 1 | 0.99605931 | -1.39898890 | 2.30195485 |
| 1 | 4.82115951 | 1.14602074 | -1.16315031 |
| 1 | 3.33551742 | 1.12758920 | 2.85840462 |
| 1 | -2.25824516 | -0.14082282 | 3.00448356 |
| 1 | -0.98999980 | -0.26112376 | 4.25143268 |
| 1 | -1.39197598 | -5.88218847 | 2.81035526 |
| 1 | -0.68227305 | -2.34943710 | 5.13657950 |
| 1 | 3.90649184 | -0.16765301 | -4.26344936 |
| 1 | 4.18969832 | 1.26384508 | -3.25484942 |
| 1 | 4.93953766 | -0.29732826 | -2.82829077 |
| 1 | 0.62814540 | 0.19422670 | -2.60806294 |
| 1 | 1.67450277 | 1.56833809 | -3.06923542 |
| 1 | 1.51087487 | 0.16815577 | -4.15338164 |
| 1 | 3.49924149 | -2.24565959 | -2.06532031 |
| 1 | 1.72640958 | -2.09031082 | -1.97802018 |
| 1 | 2.55081757 | -2.05494907 | -3.55779647 |
| 1 | -2.33700329 | -6.69526715 | -0.44974344 |
| 1 | -2.80439132 | -6.58500794 | 1.25759127 |
| 1 | -1.08183651 | -6.68886916 | 0.80225463 |
| 1 | -0.87739894 | -3.32098614 | -0.88065854 |
| 1 | 0.04859887 | -4.76081702 | -0.39302804 |
| 1 | -1.21253939 | -4.89590772 | -1.64137464 |
| 1 | -4.16303500 | -4.52323144 | 0.66472958 |
| 1 | -3.42435844 | -3.16962679 | -0.22974126 |
| 1 | -3.66093087 | -4.74725693 | -1.02688108 |
| 1 | -1.37241328 | 2.05461242 | 2.50185500 |
| 8 | -1.09875476 | 5.40181725 | -0.22990029 |
| 6 | -1.32080744 | 6.36576965 | 0.83189249 |
| 1 | -0.69416224 | 6.12443104 | 1.69909879 |
| 8 | 1.07993049 | 6.06258746 | -0.28376547 |
| 8 | -2.29537648 | 0.57095194 | -0.53363612 |
| 1 | -2.38277739 | 6.28061620 | 1.07937770 |
| 1 | -1.07932920 | 7.37700026 | 0.48304834 |
| 31 | -0.92077590 | -0.24085596 | 0.36896904 |
| 6 | 0.17372837 | 5.36076055 | -0.68718898 |
| 6 | 0.37850955 | 4.34800697 | -1.81839915 |
| 8 | -0.77640975 | 3.48890259 | -2.03432671 |
| 6 | -0.93755421 | 2.47256318 | -1.16141507 |
| 6 | -2.15165047 | 1.59965948 | -1.47154385 |
| 8 | -0.17740255 | 2.24779762 | -0.22883341 |
| 6 | -3.43863656 | 2.43604262 | -1.51989241 |
| 6 | 0.65821017 | 5.06130638 | -3.13634406 |
| 1 | 1.23181302 | 3.72446720 | -1.51700472 |

| | | | |
|---|---|---|---|
| 1 | -1.96748297 | 1.18575899 | -2.48623642 |
| 1 | -4.27928305 | 1.76815689 | -1.74356082 |
| 1 | -3.37909103 | 3.21306333 | -2.29206122 |
| 1 | -3.62008670 | 2.90813195 | -0.54499404 |
| 1 | 1.51371499 | 5.73522369 | -3.00869198 |
| 1 | -0.21385536 | 5.64646936 | -3.45625162 |
| 1 | 0.89573257 | 4.32548726 | -3.91439915 |

Electronic energy = -3673.057198 a.u.
DFT-D3(BJ) dispersion correction = -0.115369 a.u.
Thermal free energy = 0.581539 a.u.
Gibbs free energy = -3672.591028 a.u.
Number of imaginary frequencies = 0.

**Int2 LA isomer/conformer 11**

| Atomic N. | X | Y | Z |
|---|---|---|---|
| 8 | 2.10606549 | -3.35237390 | 5.03415318 |
| 6 | 2.23588054 | -2.72995922 | 6.33798092 |
| 1 | 2.75623133 | -1.76827779 | 6.25061280 |
| 8 | 0.89244872 | -1.55245294 | 4.35569110 |
| 8 | -1.60811148 | -1.58600978 | 0.58088416 |
| 1 | 2.81966600 | -3.43564938 | 6.93564571 |
| 1 | 1.24599651 | -2.56829832 | 6.78147553 |
| 7 | -0.50242471 | 1.15080601 | 1.67795204 |
| 8 | -1.49188226 | 0.97826433 | -1.12813320 |
| 8 | 1.16482302 | -0.37791381 | -0.33028865 |
| 6 | -1.39452480 | 0.76013835 | 2.80235013 |
| 6 | -2.43841913 | 1.84647015 | -0.68222721 |
| 6 | -3.65260608 | 2.02577341 | -1.40584818 |
| 6 | -4.57881751 | 2.94223824 | -0.88616725 |
| 6 | -4.35340052 | 3.66850095 | 0.28654557 |
| 6 | -3.15412381 | 3.50084851 | 0.97094877 |
| 6 | -2.19066487 | 2.60840037 | 0.48954126 |
| 6 | -0.84442381 | 2.52327924 | 1.15620910 |
| 1 | -5.10884696 | 4.36362363 | 0.65279735 |
| 6 | -3.94271293 | 1.24898425 | -2.70477912 |
| 6 | 2.05439867 | 0.62671765 | -0.10810504 |
| 6 | 3.10521318 | 0.86244095 | -1.04182136 |
| 6 | 3.99767471 | 1.90548609 | -0.75511954 |
| 6 | 3.89054886 | 2.70132155 | 0.38841929 |
| 6 | 2.86910433 | 2.45077471 | 1.29769416 |
| 6 | 1.95723422 | 1.41264767 | 1.07068786 |
| 6 | 0.93739323 | 1.09065534 | 2.13469600 |
| 1 | 4.60330934 | 3.50719556 | 0.56304516 |
| 6 | 3.26290227 | 0.00902507 | -2.31623366 |
| 6 | -5.29310986 | 1.65003420 | -3.32778029 |
| 6 | -4.00317481 | -0.26775480 | -2.40914954 |
| 6 | -2.84588713 | 1.54153671 | -3.75624599 |
| 6 | 4.48586596 | 0.43775300 | -3.14925261 |
| 6 | 2.01753227 | 0.16289453 | -3.22087439 |
| 6 | 3.46733146 | -1.47702327 | -1.93641736 |
| 1 | -1.26740809 | 1.46860728 | 3.63652297 |
| 1 | -2.43587662 | 0.77759168 | 2.46385195 |

| | | | |
|---|---|---|---|
| 1 | -0.78788945 | 3.23243659 | 1.99877798 |
| 1 | -0.04676537 | 2.77874300 | 0.44300856 |
| 1 | -5.51964772 | 3.09551201 | -1.41172910 |
| 1 | -2.94593134 | 4.07627121 | 1.87590983 |
| 1 | 1.07412504 | 0.07282198 | 2.52897587 |
| 1 | 1.05197862 | 1.79070647 | 2.97870153 |
| 1 | 4.80653706 | 2.11435919 | -1.45276630 |
| 1 | 2.77663031 | 3.05154979 | 2.20532921 |
| 1 | -5.44244299 | 1.07733769 | -4.25422356 |
| 1 | -6.14007674 | 1.42750664 | -2.66270102 |
| 1 | -5.32648694 | 2.71752732 | -3.58960233 |
| 1 | -3.06076072 | -0.63078836 | -1.98581510 |
| 1 | -4.80963768 | -0.49294658 | -1.69638509 |
| 1 | -4.20545993 | -0.82163959 | -3.33873909 |
| 1 | -2.82329735 | 2.61325212 | -4.00241939 |
| 1 | -1.85402896 | 1.24647969 | -3.39738758 |
| 1 | -3.06255298 | 0.98737883 | -4.68230304 |
| 1 | 4.55705587 | -0.20813832 | -4.03590754 |
| 1 | 5.42615656 | 0.33440216 | -2.58855785 |
| 1 | 4.40310276 | 1.47537569 | -3.50341909 |
| 1 | 1.10266258 | -0.16263866 | -2.71529752 |
| 1 | 1.88776708 | 1.21140086 | -3.52497810 |
| 1 | 2.14338990 | -0.44107975 | -4.13231042 |
| 1 | 4.38387778 | -1.60024172 | -1.34073138 |
| 1 | 2.62257974 | -1.86459935 | -1.35697736 |
| 1 | 3.57366943 | -2.08388290 | -2.84833115 |
| 1 | -1.12978770 | -0.24925127 | 3.13480753 |
| 31 | -0.62544680 | -0.12659278 | 0.09981877 |
| 6 | 1.40615165 | -2.63011138 | 4.12403606 |
| 6 | 1.40420865 | -3.37015501 | 2.78841183 |
| 8 | 0.38462088 | -2.75596794 | 1.96354676 |
| 6 | -0.27436166 | -3.61639239 | 1.13162325 |
| 6 | -1.31110539 | -2.91839231 | 0.23503602 |
| 8 | -0.07047124 | -4.81606741 | 1.09301701 |
| 6 | -0.89860317 | -3.10997409 | -1.23339317 |
| 6 | 2.76655817 | -3.28980266 | 2.09654455 |
| 1 | 1.13348089 | -4.42162935 | 2.95939651 |
| 1 | -2.23409392 | -3.50361467 | 0.40423874 |
| 1 | -1.69827121 | -2.73526137 | -1.88506811 |
| 1 | -0.72868443 | -4.17214657 | -1.45081889 |
| 1 | 0.02797522 | -2.55727007 | -1.45080280 |
| 1 | 3.54138721 | -3.72654171 | 2.73934479 |
| 1 | 3.02795217 | -2.24758386 | 1.87265583 |
| 1 | 2.73073135 | -3.85664739 | 1.15822272 |

Electronic energy = -3673.056403 a.u.
DFT-D3(BJ) dispersion correction = -0.115731 a.u.
Thermal free energy = 0.581115 a.u.
Gibbs free energy = -3672.591018 a.u.
Number of imaginary frequencies = 0.

**Int2 LA isomer/conformer 12**

Atomic N. X Y Z

| | | | |
|---|---|---|---|
| 8 | 4.47989237 | 1.81161911 | 4.21569455 |
| 6 | 5.68211523 | 2.34890331 | 3.60767463 |
| 1 | 5.62742718 | 2.25504291 | 2.51637077 |
| 8 | 3.28730589 | 3.40012597 | 3.10405410 |
| 8 | -0.65027582 | 1.92691942 | 0.71740574 |
| 1 | 6.50186088 | 1.74684734 | 4.01004181 |
| 1 | 5.80398489 | 3.40615203 | 3.87322135 |
| 7 | 1.66805236 | 0.37115079 | -1.11890277 |
| 8 | -1.25049824 | 0.32677183 | -1.67160893 |
| 8 | -0.13256892 | -1.22461475 | 0.72409517 |
| 6 | 2.40839813 | 1.65693569 | -1.03275951 |
| 6 | -0.90134361 | 1.00701980 | -2.79085178 |
| 6 | -1.86934605 | 1.75418099 | -3.52264748 |
| 6 | -1.42288810 | 2.42989819 | -4.66802794 |
| 6 | -0.09593881 | 2.38966817 | -5.10719141 |
| 6 | 0.83304988 | 1.63336318 | -4.39969217 |
| 6 | 0.43734109 | 0.93216138 | -3.25614824 |
| 6 | 1.39596580 | 0.00774096 | -2.55943414 |
| 1 | 0.20064817 | 2.93864635 | -6.00093378 |
| 6 | -3.34369207 | 1.81810459 | -3.07939713 |
| 6 | 0.53966100 | -2.27748130 | 0.19095320 |
| 6 | -0.00963925 | -3.58979776 | 0.28995718 |
| 6 | 0.72885680 | -4.64167413 | -0.27025175 |
| 6 | 1.95591779 | -4.44910751 | -0.91044660 |
| 6 | 2.48538294 | -3.16632844 | -0.99083555 |
| 6 | 1.79928294 | -2.07946126 | -0.43520578 |
| 6 | 2.46117591 | -0.72296119 | -0.44025621 |
| 1 | 2.48871683 | -5.29740862 | -1.33994661 |
| 6 | -1.36198877 | -3.85020905 | 0.98339579 |
| 6 | -4.20475255 | 2.63552895 | -4.06072987 |
| 6 | -3.45241552 | 2.49551240 | -1.69308592 |
| 6 | -3.94167765 | 0.39213091 | -3.02068060 |
| 6 | -1.73190627 | -5.34570236 | 0.98188275 |
| 6 | -2.49571298 | -3.09858000 | 0.24652520 |
| 6 | -1.29512931 | -3.39773969 | 2.46155148 |
| 1 | 3.38467127 | 1.55515434 | -1.53408968 |
| 1 | 1.82868571 | 2.44243470 | -1.52961728 |
| 1 | 2.36271137 | -0.01657500 | -3.08974918 |
| 1 | 0.99309141 | -1.01520284 | -2.53718816 |
| 1 | -2.13550785 | 3.01821001 | -5.24353851 |
| 1 | 1.86889167 | 1.56414158 | -4.74028768 |
| 1 | 2.63052502 | -0.36779595 | 0.58510376 |
| 1 | 3.43678919 | -0.79381324 | -0.94960937 |
| 1 | 0.33134038 | -5.65338279 | -0.21519743 |
| 1 | 3.44873325 | -2.99531005 | -1.47677132 |
| 1 | -5.24581131 | 2.63473702 | -3.70734651 |
| 1 | -3.87954232 | 3.68412378 | -4.12470533 |
| 1 | -4.19821517 | 2.20623520 | -5.07323586 |
| 1 | -2.88645977 | 1.95348679 | -0.92810093 |
| 1 | -3.07025732 | 3.52586453 | -1.73532091 |
| 1 | -4.50811594 | 2.53772461 | -1.38307734 |
| 1 | -3.90618589 | -0.08344733 | -4.01187985 |
| 1 | -3.40049443 | -0.24229009 | -2.31049092 |

| | | | |
|---|---|---|---|
| 1 | -4.99624423 | 0.44512320 | -2.70948554 |
| 1 | -2.69473626 | -5.47484795 | 1.49646364 |
| 1 | -0.98835037 | -5.95746993 | 1.51306310 |
| 1 | -1.84698142 | -5.74306907 | -0.03687463 |
| 1 | -2.33571252 | -2.01560902 | 0.24839998 |
| 1 | -2.56727011 | -3.43370974 | -0.79816271 |
| 1 | -3.45900290 | -3.30783451 | 0.73620843 |
| 1 | -0.53106523 | -3.97105971 | 3.00689343 |
| 1 | -1.05398562 | -2.33250272 | 2.54418559 |
| 1 | -2.26498317 | -3.57686272 | 2.95026260 |
| 1 | 2.55515137 | 1.92134156 | 0.01951353 |
| 31 | -0.14570492 | 0.40758066 | -0.16436029 |
| 6 | 3.33795339 | 2.45483151 | 3.86226913 |
| 6 | 2.13976135 | 1.89080502 | 4.64534633 |
| 8 | 0.90025138 | 2.21046072 | 3.95380229 |
| 6 | 0.75377180 | 1.73433784 | 2.69339201 |
| 6 | -0.61102367 | 2.10594387 | 2.10800430 |
| 8 | 1.59764687 | 1.07640247 | 2.10567382 |
| 6 | -1.72128784 | 1.32949292 | 2.83978694 |
| 6 | 2.21922711 | 0.42384352 | 5.05316816 |
| 1 | 2.07070207 | 2.51498710 | 5.55075981 |
| 1 | -0.74891535 | 3.18435596 | 2.30621057 |
| 1 | -2.68801175 | 1.61372845 | 2.40580708 |
| 1 | -1.72960503 | 1.56281104 | 3.91288246 |
| 1 | -1.58336930 | 0.24661278 | 2.70889460 |
| 1 | 3.07621442 | 0.27281099 | 5.72028715 |
| 1 | 2.33009528 | -0.23034119 | 4.18248724 |
| 1 | 1.30536324 | 0.15018346 | 5.59643393 |

Electronic energy = -3673.056798 a.u.
DFT-D3(BJ) dispersion correction = -0.114472 a.u.
Thermal free energy = 0.580841 a.u.
Gibbs free energy = -3672.590429 a.u.
Number of imaginary frequencies = 0.

**Int2 LA isomer/conformer 13**

| Atomic N. | X | Y | Z |
|---|---|---|---|
| 8 | 2.19213359 | -5.32849119 | -1.08001589 |
| 6 | 3.44712869 | -5.34638518 | -1.80386619 |
| 1 | 3.52442060 | -6.25643382 | -2.41152438 |
| 8 | 1.11047057 | -5.43298008 | -3.08125212 |
| 8 | 0.84986077 | -1.32158742 | 1.35251010 |
| 1 | 4.22407091 | -5.32357828 | -1.03429676 |
| 1 | 3.51936329 | -4.46915840 | -2.45879487 |
| 7 | 1.47305705 | 0.42073466 | -1.41872447 |
| 8 | 0.54477726 | 1.61600154 | 1.13937753 |
| 8 | -1.37601497 | -0.10935190 | -0.54790267 |
| 6 | 2.73245634 | -0.36601432 | -1.46498673 |
| 6 | 1.75191824 | 2.23396297 | 1.11576940 |
| 6 | 2.30536774 | 2.79314322 | 2.30379519 |
| 6 | 3.55769505 | 3.41560666 | 2.19399000 |
| 6 | 4.25891276 | 3.50755071 | 0.98780182 |
| 6 | 3.69526287 | 2.98033098 | -0.16970142 |

| | | | |
|---|---|---|---|
| 6 | 2.44354970 | 2.35840319 | -0.11715016 |
| 6 | 1.76802676 | 1.90124346 | -1.37987702 |
| 1 | 5.23226615 | 3.99724005 | 0.95769539 |
| 6 | 1.56365371 | 2.71975310 | 3.65244886 |
| 6 | -1.67124616 | 0.64023249 | -1.64366446 |
| 6 | -2.96071022 | 1.23459721 | -1.76995483 |
| 6 | -3.21049836 | 1.99019111 | -2.92453016 |
| 6 | -2.25773624 | 2.17584782 | -3.92956107 |
| 6 | -1.00817759 | 1.58099053 | -3.79901462 |
| 6 | -0.70805497 | 0.80350091 | -2.67393354 |
| 6 | 0.61517298 | 0.08112800 | -2.61836198 |
| 1 | -2.49622158 | 2.77919263 | -4.80534548 |
| 6 | -4.04475889 | 1.05694693 | -0.68795357 |
| 6 | 2.33455510 | 3.44254779 | 4.77310221 |
| 6 | 1.39424794 | 1.24421988 | 4.08478065 |
| 6 | 0.17873987 | 3.39982855 | 3.53440140 |
| 6 | -5.35370860 | 1.77968037 | -1.05937396 |
| 6 | -3.56326214 | 1.64826409 | 0.65794626 |
| 6 | -4.38294516 | -0.44360723 | -0.52177095 |
| 1 | 3.28571571 | -0.12450514 | -2.38704966 |
| 1 | 3.35023659 | -0.11281586 | -0.59642963 |
| 1 | 2.38733470 | 2.14615560 | -2.25891924 |
| 1 | 0.79606025 | 2.40177974 | -1.49810931 |
| 1 | 4.01212261 | 3.84496677 | 3.08518874 |
| 1 | 4.21230482 | 3.06530366 | -1.12841804 |
| 1 | 0.46630647 | -1.00652738 | -2.57273234 |
| 1 | 1.19929507 | 0.31355286 | -3.52416086 |
| 1 | -4.18399799 | 2.46131430 | -3.04629917 |
| 1 | -0.25348528 | 1.70219394 | -4.57935288 |
| 1 | 1.75612275 | 3.37360290 | 5.70544742 |
| 1 | 3.31786142 | 2.98744982 | 4.96125117 |
| 1 | 2.48104397 | 4.50992044 | 4.55242061 |
| 1 | 0.82634805 | 0.66528990 | 3.34871030 |
| 1 | 2.37529372 | 0.76451169 | 4.21494810 |
| 1 | 0.86434104 | 1.19893908 | 5.04883002 |
| 1 | 0.29031448 | 4.46064877 | 3.26599112 |
| 1 | -0.44579064 | 2.91327605 | 2.77751837 |
| 1 | -0.34218295 | 3.34989239 | 4.50280151 |
| 1 | -6.09189204 | 1.61118343 | -0.26239256 |
| 1 | -5.78616444 | 1.39931778 | -1.99607942 |
| 1 | -5.21431490 | 2.86613854 | -1.15517141 |
| 1 | -2.66143974 | 1.14753508 | 1.02460475 |
| 1 | -3.34287243 | 2.72031307 | 0.55333812 |
| 1 | -4.35366802 | 1.53735846 | 1.41580487 |
| 1 | -4.78747085 | -0.85449194 | -1.45836359 |
| 1 | -3.49810858 | -1.02571056 | -0.24248624 |
| 1 | -5.14662373 | -0.56975471 | 0.26074418 |
| 1 | 2.48670316 | -1.43269643 | -1.44410855 |
| 31 | 0.31269453 | 0.00828543 | 0.21830272 |
| 6 | 1.08989030 | -5.34882391 | -1.87015166 |
| 6 | -0.19618150 | -5.38749632 | -1.03086809 |
| 8 | -0.09912367 | -4.60523543 | 0.20063662 |
| 6 | 0.24183582 | -3.30082920 | 0.07424191 |

| | | | |
|---|---|---|---|
| 6 | 0.24795996 | -2.58732783 | 1.43130943 |
| 8 | 0.51561442 | -2.76775879 | -0.98969631 |
| 6 | -1.17162825 | -2.54511121 | 2.02366131 |
| 6 | -1.44073202 | -5.04978810 | -1.83642075 |
| 1 | -0.26989782 | -6.41619975 | -0.64422248 |
| 1 | 0.88035067 | -3.20817055 | 2.09303729 |
| 1 | -1.12798268 | -2.04120840 | 2.99734635 |
| 1 | -1.57307266 | -3.55733188 | 2.16360535 |
| 1 | -1.84738007 | -1.98056329 | 1.36569448 |
| 1 | -1.52532073 | -5.74652426 | -2.67914411 |
| 1 | -1.39068845 | -4.03134782 | -2.23575828 |
| 1 | -2.32950413 | -5.15226193 | -1.20116168 |

Electronic energy = -3673.057618 a.u.
DFT-D3(BJ) dispersion correction = -0.114752 a.u.
Thermal free energy = 0.582018 a.u.
Gibbs free energy = -3672.590352 a.u.
Number of imaginary frequencies = 0.

**Int2 LA isomer/conformer 14**

| Atomic N. | X | Y | Z |
|---|---|---|---|
| 8 | 3.06112040 | -1.49879245 | -2.99824931 |
| 6 | 1.92943121 | -1.04978317 | -3.78383057 |
| 1 | 1.16531901 | -0.59267984 | -3.14247863 |
| 8 | 4.57085506 | -1.06993233 | -1.42372107 |
| 8 | 1.44829635 | 1.66407949 | 1.84393058 |
| 1 | 1.52831904 | -1.96010727 | -4.23979959 |
| 1 | 2.24062719 | -0.35708818 | -4.57888001 |
| 7 | -1.29549760 | 0.99447105 | 2.59459337 |
| 8 | -0.11091398 | -1.10080462 | 0.87804928 |
| 8 | -0.92390507 | 1.74831144 | -0.26356144 |
| 6 | -0.63034953 | 1.04514211 | 3.92000350 |
| 6 | -0.57535245 | -1.92036000 | 1.84910503 |
| 6 | -0.03439954 | -3.23578686 | 1.97515105 |
| 6 | -0.55937046 | -4.05479623 | 2.98458532 |
| 6 | -1.56976146 | -3.63374077 | 3.85400403 |
| 6 | -2.09274365 | -2.35260961 | 3.71654354 |
| 6 | -1.61508850 | -1.49788312 | 2.71685826 |
| 6 | -2.26155529 | -0.15332529 | 2.52140828 |
| 1 | -1.94168723 | -4.30536175 | 4.62780963 |
| 6 | 1.08775561 | -3.73872406 | 1.04542101 |
| 6 | -2.21816236 | 2.11481270 | -0.17383095 |
| 6 | -2.98907715 | 2.27466714 | -1.36580742 |
| 6 | -4.32621628 | 2.66941351 | -1.22452480 |
| 6 | -4.91943190 | 2.90201288 | 0.01980678 |
| 6 | -4.15841441 | 2.74693704 | 1.17298977 |
| 6 | -2.81335902 | 2.36897767 | 1.08879477 |
| 6 | -1.99047549 | 2.30711067 | 2.34842253 |
| 1 | -5.96593315 | 3.20031205 | 0.08080532 |
| 6 | -2.37843914 | 2.01574662 | -2.75761779 |
| 6 | 1.48434505 | -5.19516699 | 1.35213309 |
| 6 | 2.35388934 | -2.86933091 | 1.22885647 |
| 6 | 0.62351031 | -3.69201261 | -0.43032651 |

| | | | |
|---|---|---|---|
| 6 | -3.38973527 | 2.27499969 | -3.89003921 |
| 6 | -1.92970332 | 0.53933194 | -2.87027437 |
| 6 | -1.17186147 | 2.95496896 | -2.99558429 |
| 1 | -1.38338158 | 1.19738316 | 4.71148216 |
| 1 | -0.10345650 | 0.10301368 | 4.10356202 |
| 1 | -3.04869639 | -0.00036640 | 3.27988549 |
| 1 | -2.73960021 | -0.08881509 | 1.53246688 |
| 1 | -0.16352472 | -5.06155454 | 3.10589558 |
| 1 | -2.89121599 | -2.00606601 | 4.37680706 |
| 1 | -1.18879033 | 3.06349323 | 2.33148456 |
| 1 | -2.63720175 | 2.52239761 | 3.21641079 |
| 1 | -4.93743383 | 2.79612169 | -2.11634433 |
| 1 | -4.59945489 | 2.93039434 | 2.15549125 |
| 1 | 2.28055176 | -5.50038074 | 0.65832405 |
| 1 | 1.87402093 | -5.31307145 | 2.37367660 |
| 1 | 0.64250349 | -5.89006211 | 1.21815751 |
| 1 | 2.14802358 | -1.81796416 | 1.00393323 |
| 1 | 2.72483345 | -2.94088346 | 2.26204401 |
| 1 | 3.15185722 | -3.21253142 | 0.55415674 |
| 1 | -0.24744425 | -4.34679671 | -0.58146326 |
| 1 | 0.35143663 | -2.67457027 | -0.73089024 |
| 1 | 1.43451767 | -4.04699512 | -1.08425431 |
| 1 | -2.90224365 | 2.08244245 | -4.85669555 |
| 1 | -3.74243900 | 3.31641407 | -3.89894092 |
| 1 | -4.26436524 | 1.61207421 | -3.82407975 |
| 1 | -1.18079647 | 0.28867659 | -2.11104863 |
| 1 | -2.78764547 | -0.13706482 | -2.74632788 |
| 1 | -1.49964586 | 0.35661403 | -3.86809894 |
| 1 | -1.48834735 | 4.00762600 | -2.96033788 |
| 1 | -0.39306571 | 2.80052089 | -2.24069636 |
| 1 | -0.74417275 | 2.76587557 | -3.99287157 |
| 1 | 0.09416043 | 1.86576902 | 3.92378048 |
| 31 | 0.04672453 | 0.75707823 | 1.00354104 |
| 6 | 3.74368546 | -0.64191509 | -2.19720612 |
| 6 | 3.51397009 | 0.87488788 | -2.37190768 |
| 8 | 3.58443133 | 1.51034701 | -1.05384315 |
| 6 | 2.54765157 | 1.27236763 | -0.24909477 |
| 6 | 2.63036145 | 1.90080896 | 1.13667409 |
| 8 | 1.57384463 | 0.59351882 | -0.59558587 |
| 6 | 3.86013665 | 1.36298082 | 1.88985290 |
| 6 | 4.63147887 | 1.48041167 | -3.20908345 |
| 1 | 2.52709018 | 1.09973716 | -2.79073923 |
| 1 | 2.77027343 | 2.98948898 | 0.96784352 |
| 1 | 3.88847362 | 1.83187408 | 2.88108895 |
| 1 | 4.79010708 | 1.59553465 | 1.35514247 |
| 1 | 3.78152931 | 0.27553827 | 2.01785857 |
| 1 | 4.61409504 | 1.05505031 | -4.22183562 |
| 1 | 5.60481738 | 1.26048892 | -2.75384504 |
| 1 | 4.50031133 | 2.56709851 | -3.28511508 |

Electronic energy = -3673.056183 a.u.
DFT-D3(BJ) dispersion correction = -0.116559 a.u.
Thermal free energy = 0.583041 a.u.
Gibbs free energy = -3672.589701 a.u.

Number of imaginary frequencies = 0.

**Int2_LA isomer/conformer 15**

| Atomic N. | X | Y | Z |
|---|---|---|---|
| 8 | -4.56141311 | -0.32928635 | -0.67825348 |
| 6 | -5.46511357 | 0.43511722 | 0.16660127 |
| 1 | -5.58276550 | 1.44959684 | -0.23235649 |
| 8 | -5.98205221 | -0.02044095 | -2.43562497 |
| 8 | -0.86733386 | 1.45692785 | -1.69172725 |
| 1 | -4.98728481 | 0.45091354 | 1.14970088 |
| 1 | -6.44377030 | -0.05713107 | 0.20870262 |
| 7 | 1.11305690 | 2.51449820 | 0.15460327 |
| 8 | 1.67003453 | -0.32523432 | -0.47916889 |
| 8 | -0.79128652 | 0.56688421 | 1.34908318 |
| 6 | 1.30440962 | 3.38792870 | -1.02871207 |
| 6 | 2.91656223 | 0.14352703 | -0.70974828 |
| 6 | 3.83043273 | -0.64157980 | -1.47683639 |
| 6 | 5.10947327 | -0.11199976 | -1.69813201 |
| 6 | 5.51402235 | 1.12809510 | -1.19588293 |
| 6 | 4.62483412 | 1.86985296 | -0.42590225 |
| 6 | 3.33841406 | 1.38459826 | -0.16644230 |
| 6 | 2.43404736 | 2.14569784 | 0.76397228 |
| 1 | 6.51797255 | 1.50067030 | -1.39933876 |
| 6 | 3.43884608 | -2.02766995 | -2.02721408 |
| 6 | -0.48378179 | 1.20719657 | 2.49476432 |
| 6 | -0.73487095 | 0.57210500 | 3.74904310 |
| 6 | -0.41449129 | 1.28825114 | 4.91046313 |
| 6 | 0.13491381 | 2.57381065 | 4.88158990 |
| 6 | 0.37188986 | 3.18293649 | 3.65441391 |
| 6 | 0.05744390 | 2.51793346 | 2.46357443 |
| 6 | 0.23982158 | 3.23102323 | 1.15079043 |
| 1 | 0.37490428 | 3.08780001 | 5.81232989 |
| 6 | -1.33002653 | -0.84775834 | 3.82605397 |
| 6 | 4.59575742 | -2.69108648 | -2.79775455 |
| 6 | 2.24731280 | -1.90165439 | -3.00510039 |
| 6 | 3.05909907 | -2.97069209 | -0.86048786 |
| 6 | -1.53052629 | -1.31013583 | 5.28152940 |
| 6 | -0.38022815 | -1.86183248 | 3.14543164 |
| 6 | -2.71552180 | -0.88288875 | 3.13701187 |
| 1 | 1.81269867 | 4.31979623 | -0.72802411 |
| 1 | 1.91827514 | 2.87043461 | -1.77332039 |
| 1 | 2.93559110 | 3.06820609 | 1.10475558 |
| 1 | 2.20335079 | 1.54672499 | 1.65760850 |
| 1 | 5.82352271 | -0.68503267 | -2.28691239 |
| 1 | 4.92669492 | 2.83043097 | -0.00190107 |
| 1 | -0.72749795 | 3.37324250 | 0.64201170 |
| 1 | 0.67456551 | 4.22879346 | 1.33445159 |
| 1 | -0.59129162 | 0.82741843 | 5.88072355 |
| 1 | 0.79524493 | 4.18902715 | 3.60789523 |
| 1 | 4.26613229 | -3.67629004 | -3.15756385 |
| 1 | 4.90169720 | -2.10329676 | -3.67557876 |
| 1 | 5.47806610 | -2.85020062 | -2.16130111 |

| | | | |
|---|---|---|---|
| 1 | 1.36636389 | -1.49749144 | -2.49566011 |
| 1 | 2.50530915 | -1.24536086 | -3.84976740 |
| 1 | 1.99013534 | -2.89232319 | -3.41016985 |
| 1 | 3.91118549 | -3.10091535 | -0.17756710 |
| 1 | 2.20969592 | -2.58037974 | -0.28960964 |
| 1 | 2.78833675 | -3.96170839 | -1.25603079 |
| 1 | -1.96296028 | -2.32089184 | 5.27845801 |
| 1 | -2.22097862 | -0.65559680 | 5.83335467 |
| 1 | -0.58144877 | -1.35940353 | 5.83462286 |
| 1 | -0.23635568 | -1.63296983 | 2.08451341 |
| 1 | 0.60217227 | -1.86305501 | 3.63961984 |
| 1 | -0.80040081 | -2.87604374 | 3.22544120 |
| 1 | -3.41112418 | -0.19193015 | 3.63725436 |
| 1 | -2.63701344 | -0.60805023 | 2.07946277 |
| 1 | -3.13827456 | -1.89700564 | 3.20442724 |
| 1 | 0.32739155 | 3.61457781 | -1.46693044 |
| 31 | 0.12148117 | 0.70826567 | -0.28271051 |
| 6 | -4.95670654 | -0.46605601 | -1.95801995 |
| 6 | -3.96130292 | -1.27056708 | -2.80813627 |
| 8 | -2.76298681 | -1.64892637 | -2.07412784 |
| 6 | -1.83918593 | -0.73784062 | -1.73266816 |
| 6 | -1.72225363 | 0.62970469 | -2.41775139 |
| 8 | -1.01498461 | -1.06274795 | -0.87597676 |
| 6 | -1.19138586 | 0.43231797 | -3.85667877 |
| 6 | -4.59547201 | -2.56914411 | -3.29425767 |
| 1 | -3.70396942 | -0.63777652 | -3.66852278 |
| 1 | -2.72988046 | 1.08766863 | -2.46423059 |
| 1 | -1.09632292 | 1.42255725 | -4.31867849 |
| 1 | -1.84843594 | -0.18446334 | -4.48625284 |
| 1 | -0.19794498 | -0.03316290 | -3.82272630 |
| 1 | -5.53094874 | -2.34164228 | -3.81836476 |
| 1 | -4.80912504 | -3.23093968 | -2.44540025 |
| 1 | -3.91244099 | -3.08392781 | -3.98073670 |

Electronic energy = -3673.055565 a.u.
DFT-D3(BJ) dispersion correction = -0.115097 a.u.
Thermal free energy = 0.581965 a.u.
Gibbs free energy = -3672.588697 a.u.
Number of imaginary frequencies = 0.

**Int2 LA isomer/conformer 16**

| Atomic N. | X | Y | Z |
|---|---|---|---|
| 7 | 0.64594004 | 1.98874433 | -2.45131898 |
| 8 | 1.21904857 | 0.88279662 | 0.26030180 |
| 8 | -1.60102138 | 0.55766520 | -1.03288261 |
| 6 | 1.60751930 | 1.65685331 | -3.53340402 |
| 6 | 2.36640245 | 1.60175015 | 0.11977774 |
| 6 | 3.49991671 | 1.30083805 | 0.92896660 |
| 6 | 4.64818401 | 2.08431299 | 0.74225106 |
| 6 | 4.71289811 | 3.12736124 | -0.18543326 |
| 6 | 3.59270483 | 3.42412333 | -0.95410000 |
| 6 | 2.41724004 | 2.68060137 | -0.80116800 |
| 6 | 1.17977143 | 3.07943571 | -1.55967340 |

| | | | |
|---|---|---|---|
| 1 | 5.63124977 | 3.70345860 | -0.29761451 |
| 6 | 3.46987997 | 0.16918822 | 1.97461034 |
| 6 | -2.19941313 | 1.77542651 | -1.10653286 |
| 6 | -3.29447329 | 2.08087964 | -0.24701184 |
| 6 | -3.88181050 | 3.34687489 | -0.38242852 |
| 6 | -3.43701153 | 4.29578986 | -1.30740758 |
| 6 | -2.37429502 | 3.98163871 | -2.14612008 |
| 6 | -1.75932779 | 2.72628787 | -2.06384797 |
| 6 | -0.68366340 | 2.37238481 | -3.05766895 |
| 1 | -3.92286622 | 5.26942340 | -1.36891014 |
| 6 | -3.81229696 | 1.07033062 | 0.79520970 |
| 6 | 4.80148644 | 0.05457548 | 2.74034004 |
| 6 | 3.21977761 | -1.19112397 | 1.28235352 |
| 6 | 2.36288741 | 0.45106266 | 3.01846737 |
| 6 | -5.02134098 | 1.61874344 | 1.57652287 |
| 6 | -2.70054409 | 0.76146881 | 1.82544888 |
| 6 | -4.26763328 | -0.23423052 | 0.09894984 |
| 1 | 1.72898676 | 2.52200113 | -4.20500985 |
| 1 | 2.57711197 | 1.40033395 | -3.09347973 |
| 1 | 1.38252228 | 3.96866345 | -2.17967948 |
| 1 | 0.36148332 | 3.33006594 | -0.86853875 |
| 1 | 5.53301523 | 1.87232580 | 1.33955750 |
| 1 | 3.61381103 | 4.24898431 | -1.66993594 |
| 1 | -0.98659585 | 1.50582066 | -3.66767539 |
| 1 | -0.51515565 | 3.22193587 | -3.74004274 |
| 1 | -4.71740560 | 3.61121886 | 0.26287102 |
| 1 | -2.01910594 | 4.70345538 | -2.88499091 |
| 1 | 4.72027467 | -0.75742874 | 3.47690668 |
| 1 | 5.64314786 | -0.18697163 | 2.07527128 |
| 1 | 5.04381812 | 0.97564966 | 3.28979191 |
| 1 | 2.26770088 | -1.20232614 | 0.74155083 |
| 1 | 4.02333482 | -1.41797220 | 0.56715214 |
| 1 | 3.20388297 | -1.99389713 | 2.03563551 |
| 1 | 2.56776966 | 1.38945182 | 3.55408202 |
| 1 | 1.37620591 | 0.53094864 | 2.54886398 |
| 1 | 2.33550132 | -0.36082362 | 3.76147331 |
| 1 | -5.35600295 | 0.85640189 | 2.29417039 |
| 1 | -5.87140118 | 1.84665546 | 0.91723283 |
| 1 | -4.77129827 | 2.52339250 | 2.14956231 |
| 1 | -1.82238839 | 0.31213445 | 1.35033973 |
| 1 | -2.38505685 | 1.67808294 | 2.34458341 |
| 1 | -3.08175040 | 0.05851815 | 2.58178714 |
| 1 | -5.08114852 | -0.02991832 | -0.61246862 |
| 1 | -3.44471259 | -0.71475154 | -0.44012062 |
| 1 | -4.64808635 | -0.94229979 | 0.85041025 |
| 1 | 1.23387287 | 0.79355358 | -4.09500322 |
| 8 | -0.41728931 | -5.71898034 | 1.88303345 |
| 6 | -1.16397667 | -6.96068004 | 1.91685613 |
| 1 | -2.17506026 | -6.78911579 | 2.30686413 |
| 8 | -2.00724820 | -4.86871321 | 0.48816260 |
| 8 | 0.74924146 | -1.09772833 | -2.15750272 |
| 1 | -0.59669662 | -7.62079022 | 2.57947334 |
| 1 | -1.23292246 | -7.38579041 | 0.90819642 |

| | | | |
|---|---|---|---|
| 31 | 0.23471952 | 0.39027037 | -1.23659893 |
| 8 | -0.33984834 | -2.65199773 | 0.03352535 |
| 6 | 0.12861728 | -3.19184429 | -1.13505549 |
| 6 | -0.12448074 | -2.22462078 | -2.30275555 |
| 6 | -0.97169849 | -4.75080582 | 1.10819775 |
| 6 | -0.12872223 | -3.47246835 | 1.20667799 |
| 8 | 0.68199913 | -4.27066474 | -1.19947008 |
| 6 | -0.54791132 | -2.64711200 | 2.41771113 |
| 6 | 0.14472039 | -2.90270356 | -3.63686408 |
| 1 | -1.18498597 | -1.91684019 | -2.24128414 |
| 1 | 0.93086359 | -3.75735419 | 1.26001943 |
| 1 | 0.04450909 | -1.72528768 | 2.46530474 |
| 1 | -0.37703659 | -3.22365996 | 3.33596078 |
| 1 | -1.61118896 | -2.38324570 | 2.35351796 |
| 1 | -0.49741628 | -3.78513052 | -3.75978960 |
| 1 | 1.19114100 | -3.22687378 | -3.69306549 |
| 1 | -0.06086929 | -2.19772944 | -4.45308911 |

Electronic energy = -3673.054994 a.u.
DFT-D3(BJ) dispersion correction = -0.114767 a.u.
Thermal free energy = 0.581512 a.u.
Gibbs free energy = -3672.588250 a.u.
Number of imaginary frequencies = 0.

**Int2 LA isomer/conformer 17**

| Atomic N. | X | Y | Z |
|---|---|---|---|
| 7 | 0.58419277 | 2.18257636 | -2.18957287 |
| 8 | 1.22346882 | 0.89817408 | 0.42607949 |
| 8 | -1.55722105 | 0.47136013 | -0.92663657 |
| 6 | 1.58331366 | 2.01903652 | -3.27640766 |
| 6 | 2.30988707 | 1.71696138 | 0.36206920 |
| 6 | 3.46062044 | 1.43708849 | 1.15418591 |
| 6 | 4.54137291 | 2.32527075 | 1.05198917 |
| 6 | 4.52469338 | 3.44917145 | 0.22177217 |
| 6 | 3.38842125 | 3.72061064 | -0.53231780 |
| 6 | 2.27718324 | 2.87325038 | -0.46022550 |
| 6 | 1.01729128 | 3.23380148 | -1.20071973 |
| 1 | 5.39301975 | 4.10603440 | 0.17287581 |
| 6 | 3.52004766 | 0.21642025 | 2.09296580 |
| 6 | -2.24672773 | 1.64254043 | -0.91432620 |
| 6 | -3.36737908 | 1.79543607 | -0.04707773 |
| 6 | -4.05234217 | 3.01806309 | -0.09344431 |
| 6 | -3.67791277 | 4.06666709 | -0.93833421 |
| 6 | -2.58715401 | 3.90211735 | -1.78373970 |
| 6 | -1.87549411 | 2.69621142 | -1.78968643 |
| 6 | -0.76335844 | 2.50858898 | -2.78889741 |
| 1 | -4.23862455 | 5.00118044 | -0.93205520 |
| 6 | -3.80909177 | 0.67156911 | 0.91108232 |
| 6 | 4.85515409 | 0.14034117 | 2.85744277 |
| 6 | 3.38312453 | -1.09126934 | 1.27835444 |
| 6 | 2.39202349 | 0.31260641 | 3.14781824 |
| 6 | -5.06129987 | 1.06366586 | 1.71807715 |
| 6 | -2.68170534 | 0.37256056 | 1.92711400 |

| | | | |
|---|---|---|---|
| 6 | -4.15767965 | -0.60808476 | 0.11408429 |
| 1 | 1.64310095 | 2.94390640 | -3.87242846 |
| 1 | 2.56487700 | 1.80482049 | -2.84047676 |
| 1 | 1.15130343 | 4.18596777 | -1.74073726 |
| 1 | 0.17483515 | 3.35716781 | -0.50422206 |
| 1 | 5.43769453 | 2.13351658 | 1.63882199 |
| 1 | 3.34570386 | 4.60431823 | -1.17300384 |
| 1 | -0.98880618 | 1.67325191 | -3.47170765 |
| 1 | -0.65468018 | 3.42109154 | -3.39822086 |
| 1 | -4.91005877 | 3.16653782 | 0.55981148 |
| 1 | -2.28412867 | 4.70465567 | -2.45988046 |
| 1 | 4.83907277 | -0.73969915 | 3.51592059 |
| 1 | 5.71487837 | 0.02978963 | 2.18091392 |
| 1 | 5.02047387 | 1.02375382 | 3.49088274 |
| 1 | 2.43549261 | -1.13185904 | 0.73109856 |
| 1 | 4.20284425 | -1.18511690 | 0.55177643 |
| 1 | 3.43399332 | -1.95782841 | 1.95533534 |
| 1 | 2.51908849 | 1.21246648 | 3.76713921 |
| 1 | 1.40273047 | 0.35440203 | 2.67868021 |
| 1 | 2.42952597 | -0.56239408 | 3.81471670 |
| 1 | -5.33781613 | 0.22685459 | 2.37496093 |
| 1 | -5.92366073 | 1.27258744 | 1.06854102 |
| 1 | -4.88589015 | 1.94038295 | 2.35813906 |
| 1 | -1.76822540 | 0.03083302 | 1.42951071 |
| 1 | -2.44202593 | 1.26881388 | 2.51726179 |
| 1 | -3.00981059 | -0.41338501 | 2.62436377 |
| 1 | -4.98031350 | -0.41381291 | -0.58966598 |
| 1 | -3.29581367 | -0.98047104 | -0.44942549 |
| 1 | -4.48706213 | -1.39696158 | 0.80723164 |
| 1 | 1.28754046 | 1.17858073 | -3.91395833 |
| 8 | -1.50029318 | -5.01352065 | 0.03764335 |
| 6 | -2.15378468 | -6.30191677 | -0.08118771 |
| 1 | -1.45540587 | -7.04216940 | -0.49025505 |
| 8 | -0.04613394 | -5.96066688 | 1.51317466 |
| 8 | 0.91488311 | -0.90231975 | -2.15425029 |
| 1 | -2.99231616 | -6.13753353 | -0.76416886 |
| 1 | -2.50782191 | -6.64209654 | 0.89973641 |
| 31 | 0.28852139 | 0.46233076 | -1.11795279 |
| 8 | -0.07809267 | -2.74229732 | -0.12682842 |
| 6 | 0.45691467 | -3.11892232 | -1.32932613 |
| 6 | 0.12513975 | -2.07141822 | -2.40525510 |
| 6 | -0.42972663 | -5.00621263 | 0.86598270 |
| 6 | 0.22469985 | -3.62619601 | 0.98426612 |
| 8 | 1.11425250 | -4.12767232 | -1.48615950 |
| 6 | -0.24574684 | -2.92883572 | 2.25599970 |
| 6 | 0.43978391 | -2.60227647 | -3.79511196 |
| 1 | -0.95468106 | -1.84790048 | -2.31913807 |
| 1 | 1.30785930 | -3.80863935 | 1.00766913 |
| 1 | 0.27606012 | -1.97136716 | 2.37244871 |
| 1 | -0.02289721 | -3.56395934 | 3.12241149 |
| 1 | -1.32649273 | -2.74050255 | 2.21878219 |
| 1 | -0.13719094 | -3.51354741 | -4.00330860 |
| 1 | 1.50649889 | -2.84482075 | -3.87480138 |

```
   1     0.18328311  -1.84166002  -4.54421896
Electronic energy = -3673.054287 a.u.
DFT-D3(BJ) dispersion correction = -0.114922 a.u.
Thermal free energy = 0.581471 a.u.
Gibbs free energy = -3672.587738 a.u.
Number of imaginary frequencies = 0.
```

**Int2 LA isomer/conformer 18**

| Atomic N. | X | Y | Z |
|---|---|---|---|
| 8 | 5.71542380 | -1.61164174 | 0.12134882 |
| 6 | 4.76079965 | -1.52150048 | -0.96134997 |
| 1 | 4.06167958 | -2.36585296 | -0.92983814 |
| 8 | 6.24842284 | -1.35060449 | 2.25816154 |
| 8 | -0.33918779 | -2.61462954 | 0.61444490 |
| 1 | 5.36130758 | -1.57918371 | -1.87455888 |
| 1 | 4.21079505 | -0.57177997 | -0.95261721 |
| 7 | -2.77711970 | -1.15188304 | -0.06980356 |
| 8 | -0.36320485 | 0.01191365 | -1.33807856 |
| 8 | -0.96495816 | 0.29031951 | 1.79413909 |
| 6 | -3.06558761 | -2.50276617 | -0.61118892 |
| 6 | -1.12492277 | -0.06023188 | -2.45367002 |
| 6 | -0.49821502 | -0.00924994 | -3.73594958 |
| 6 | -1.32566421 | -0.07866907 | -4.86546105 |
| 6 | -2.71565407 | -0.19327427 | -4.78010798 |
| 6 | -3.31596934 | -0.22847369 | -3.52645178 |
| 6 | -2.53847724 | -0.15028946 | -2.36593018 |
| 6 | -3.22051433 | -0.08332047 | -1.02672998 |
| 1 | -3.31788115 | -0.24931226 | -5.68679505 |
| 6 | 1.03111179 | 0.11924742 | -3.88265261 |
| 6 | -2.09653148 | 0.94199754 | 2.13853036 |
| 6 | -2.01197069 | 2.20573271 | 2.79762135 |
| 6 | -3.21572047 | 2.83313938 | 3.14663782 |
| 6 | -4.46844399 | 2.27574012 | 2.87356043 |
| 6 | -4.53764415 | 1.04276389 | 2.23508401 |
| 6 | -3.36573153 | 0.36674236 | 1.87615162 |
| 6 | -3.46546128 | -1.00361384 | 1.26224615 |
| 1 | -5.37738378 | 2.80519206 | 3.15888022 |
| 6 | -0.65227442 | 2.85845640 | 3.11569619 |
| 6 | 1.46832194 | 0.18459773 | -5.35816400 |
| 6 | 1.72645243 | -1.11037956 | -3.25254196 |
| 6 | 1.52793257 | 1.41460277 | -3.19767984 |
| 6 | -0.81467145 | 4.20882701 | 3.83860071 |
| 6 | 0.12984503 | 3.12773245 | 1.80820118 |
| 6 | 0.17113380 | 1.93557145 | 4.04528526 |
| 1 | -4.15136953 | -2.62014551 | -0.76552631 |
| 1 | -2.55113805 | -2.63406928 | -1.56872269 |
| 1 | -4.31373756 | -0.15872415 | -1.15812186 |
| 1 | -3.01468140 | 0.87723283 | -0.53112527 |
| 1 | -0.87320997 | -0.04630910 | -5.85487509 |
| 1 | -4.40207520 | -0.30162348 | -3.43464259 |
| 1 | -3.00669122 | -1.76351040 | 1.91566018 |
| 1 | -4.52839145 | -1.26936515 | 1.13113843 |

| | | | |
|---|---|---|---|
| 1 | -3.18172966 | 3.79930564 | 3.64687427 |
| 1 | -5.50545230 | 0.58516207 | 2.01744434 |
| 1 | 2.56238089 | 0.28490330 | -5.40296164 |
| 1 | 1.19668624 | -0.72479256 | -5.91345562 |
| 1 | 1.03597346 | 1.05149556 | -5.87783550 |
| 1 | 1.49985126 | -1.18407256 | -2.18324730 |
| 1 | 1.40001408 | -2.03756724 | -3.74591921 |
| 1 | 2.81718801 | -1.02602540 | -3.38197492 |
| 1 | 1.07095490 | 2.29756111 | -3.66734134 |
| 1 | 1.28384481 | 1.42377680 | -2.12993275 |
| 1 | 2.61985024 | 1.50153485 | -3.31251564 |
| 1 | 0.18154061 | 4.62405553 | 4.04804216 |
| 1 | -1.33788018 | 4.10389536 | 4.80002938 |
| 1 | -1.35622037 | 4.94389455 | 3.22584254 |
| 1 | 0.33435403 | 2.20182665 | 1.26067610 |
| 1 | -0.43674209 | 3.80314961 | 1.15089367 |
| 1 | 1.08975417 | 3.61310718 | 2.04292971 |
| 1 | -0.34953126 | 1.78557770 | 5.00238722 |
| 1 | 0.33823123 | 0.95631204 | 3.58319360 |
| 1 | 1.14689834 | 2.39794483 | 4.26100040 |
| 1 | -2.70068744 | -3.25518023 | 0.09518713 |
| 31 | -0.74195847 | -0.81664089 | 0.29881107 |
| 6 | 5.39613174 | -1.27815452 | 1.40077751 |
| 6 | 3.96507712 | -0.79076218 | 1.70539994 |
| 8 | 3.08954327 | -1.96789479 | 1.51230559 |
| 6 | 1.84369614 | -1.73683949 | 1.08633018 |
| 6 | 0.93860536 | -2.96713468 | 1.05133615 |
| 8 | 1.43028126 | -0.62193084 | 0.75699794 |
| 6 | 1.54277591 | -4.08166700 | 0.18093568 |
| 6 | 3.82876776 | -0.28298101 | 3.12631110 |
| 1 | 3.65517005 | -0.02121205 | 0.98521132 |
| 1 | 0.90599772 | -3.32711037 | 2.10244348 |
| 1 | 0.86878315 | -4.94647919 | 0.20876470 |
| 1 | 2.52995014 | -4.39057487 | 0.54950128 |
| 1 | 1.62630040 | -3.74499005 | -0.86141242 |
| 1 | 4.49764795 | 0.57501643 | 3.27058775 |
| 1 | 4.11712915 | -1.05922700 | 3.84381309 |
| 1 | 2.79808017 | 0.04331203 | 3.31376387 |

Electronic energy = -3673.053863 a.u.
DFT-D3(BJ) dispersion correction = -0.116187 a.u.
Thermal free energy = 0.582462 a.u.
Gibbs free energy = -3672.587588 a.u.
Number of imaginary frequencies = 0.

**Int2 LA isomer/conformer 19**

| Atomic N. | X | Y | Z |
|---|---|---|---|
| 7 | 0.95370758 | 0.70616494 | 1.41074590 |
| 8 | -1.02411852 | 1.04248441 | -0.82338877 |
| 8 | 1.90539752 | 0.15204033 | -1.38726141 |
| 6 | 0.52178616 | -0.10094703 | 2.58119008 |
| 6 | -1.75697098 | 1.67208724 | 0.13450742 |
| 6 | -3.14695267 | 1.90682947 | -0.07785969 |

| | | | |
|---|---|---|---|
| 6 | -3.85879998 | 2.52399154 | 0.96118599 |
| 6 | -3.25990065 | 2.92508035 | 2.15826684 |
| 6 | -1.89226698 | 2.74142652 | 2.32826687 |
| 6 | -1.13129730 | 2.13656980 | 1.32153999 |
| 6 | 0.36845813 | 2.09241859 | 1.45604177 |
| 1 | -3.85870206 | 3.39182754 | 2.94019653 |
| 6 | -3.84101893 | 1.53449218 | -1.40389356 |
| 6 | 2.75304366 | 1.17913231 | -1.12921214 |
| 6 | 3.38812167 | 1.86306708 | -2.20649068 |
| 6 | 4.26423581 | 2.90651409 | -1.87598261 |
| 6 | 4.52709060 | 3.28816251 | -0.55696419 |
| 6 | 3.91055641 | 2.60493093 | 0.48480817 |
| 6 | 3.03598755 | 1.54592393 | 0.21242892 |
| 6 | 2.46387221 | 0.75194680 | 1.35816659 |
| 1 | 5.21119281 | 4.11146973 | -0.35187974 |
| 6 | 3.12577614 | 1.47546811 | -3.67515881 |
| 6 | -5.33101009 | 1.92524945 | -1.40250033 |
| 6 | -3.76675616 | 0.01109772 | -1.65419651 |
| 6 | -3.16521259 | 2.28995391 | -2.57340757 |
| 6 | 3.94868637 | 2.33653194 | -4.65208291 |
| 6 | 1.63313416 | 1.68608379 | -4.02351059 |
| 6 | 3.52357855 | -0.00062113 | -3.91480256 |
| 1 | 0.90497296 | 0.35102027 | 3.50976812 |
| 1 | -0.57241550 | -0.12807168 | 2.62611208 |
| 1 | 0.67653775 | 2.56597277 | 2.40323609 |
| 1 | 0.84445007 | 2.64693361 | 0.63349158 |
| 1 | -4.92547306 | 2.70025238 | 0.83673324 |
| 1 | -1.39570632 | 3.08436049 | 3.23873093 |
| 1 | 2.78513467 | -0.30055109 | 1.30424702 |
| 1 | 2.82389334 | 1.16610876 | 2.31449991 |
| 1 | 4.76002839 | 3.45203709 | -2.67676503 |
| 1 | 4.11245924 | 2.87781804 | 1.52304608 |
| 1 | -5.77056139 | 1.65643371 | -2.37349263 |
| 1 | -5.89872798 | 1.39423556 | -0.62462281 |
| 1 | -5.47516936 | 3.00619147 | -1.26283343 |
| 1 | -2.72908808 | -0.32228326 | -1.75954714 |
| 1 | -4.23683969 | -0.54481463 | -0.82970038 |
| 1 | -4.30434315 | -0.24044258 | -2.58099767 |
| 1 | -3.25439926 | 3.37710778 | -2.43448287 |
| 1 | -2.10268154 | 2.03551346 | -2.65686041 |
| 1 | -3.66073748 | 2.02869382 | -3.52083750 |
| 1 | 3.73400747 | 2.01224676 | -5.68021495 |
| 1 | 5.03075777 | 2.22651635 | -4.49008363 |
| 1 | 3.69216218 | 3.40363438 | -4.58285777 |
| 1 | 0.97943759 | 1.06143223 | -3.40573498 |
| 1 | 1.34524415 | 2.73762806 | -3.87999340 |
| 1 | 1.45874246 | 1.42819097 | -5.07912550 |
| 1 | 4.59448136 | -0.14948441 | -3.71288167 |
| 1 | 2.95066180 | -0.68199865 | -3.27685560 |
| 1 | 3.33856881 | -0.26788928 | -4.96636849 |
| 1 | 0.90087642 | -1.12405546 | 2.49158138 |
| 8 | -0.57916678 | -4.96249817 | 4.86520917 |
| 6 | -0.84961364 | -4.43334923 | 6.18810191 |

| | | | |
|---|---|---|---|
| 1 | -1.61887391 | -3.65396734 | 6.13020307 |
| 8 | 0.09409841 | -2.88269146 | 4.22832414 |
| 8 | 0.05878346 | -1.88767687 | -0.30158915 |
| 1 | -1.20422946 | -5.28710043 | 6.77249779 |
| 1 | 0.06385790 | -4.01172151 | 6.62557040 |
| 31 | 0.37750219 | -0.10049436 | -0.37604408 |
| 6 | -0.11047298 | -4.05393835 | 3.97655925 |
| 6 | 0.19167800 | -4.74478988 | 2.64196065 |
| 8 | 0.12087700 | -3.79354349 | 1.55496903 |
| 6 | -1.13878796 | -3.35825849 | 1.26511587 |
| 6 | -1.19351674 | -2.46166489 | 0.01810818 |
| 8 | -2.12657810 | -3.69110841 | 1.89446854 |
| 6 | -1.69696265 | -3.32062814 | -1.15371762 |
| 6 | 1.60600502 | -5.31649556 | 2.63812249 |
| 1 | -0.55992133 | -5.53046908 | 2.48557516 |
| 1 | -1.96468141 | -1.70655266 | 0.25463762 |
| 1 | -1.84587269 | -2.67963335 | -2.03198541 |
| 1 | -2.64724925 | -3.81003750 | -0.90239559 |
| 1 | -0.94686002 | -4.08295042 | -1.40375220 |
| 1 | 1.70948037 | -6.06509673 | 3.43417231 |
| 1 | 2.34405344 | -4.51974153 | 2.79665353 |
| 1 | 1.80855907 | -5.79770610 | 1.67329134 |

Electronic energy = -3673.055066 a.u.
DFT-D3(BJ) dispersion correction = -0.112832 a.u.
Thermal free energy = 0.580687 a.u.
Gibbs free energy = -3672.587211 a.u.
Number of imaginary frequencies = 0.

**Int2 LA isomer/conformer 20**

| Atomic N. | X | Y | Z |
|---|---|---|---|
| 8 | -3.78563587 | 1.53616385 | -4.60676987 |
| 6 | -2.95703355 | 0.37721100 | -4.86067421 |
| 1 | -1.92357585 | 0.54647663 | -4.52856374 |
| 8 | -4.59956459 | 3.03140794 | -3.17499416 |
| 8 | -0.72565636 | 2.29309351 | 1.52828854 |
| 1 | -2.97049842 | 0.25469018 | -5.94810396 |
| 1 | -3.36428064 | -0.52922002 | -4.39228383 |
| 7 | 1.45590078 | 0.72055626 | 2.68019400 |
| 8 | 1.32158403 | 0.42910017 | -0.26406904 |
| 8 | -0.83557996 | -0.89269369 | 1.67464851 |
| 6 | 1.81433401 | 2.10143540 | 3.08804173 |
| 6 | 2.63330603 | 0.74593626 | -0.18706492 |
| 6 | 3.31282808 | 1.21737373 | -1.35113229 |
| 6 | 4.67458034 | 1.52576179 | -1.22494615 |
| 6 | 5.37404883 | 1.38785738 | -0.02281141 |
| 6 | 4.70519667 | 0.91266822 | 1.10013673 |
| 6 | 3.34856733 | 0.57898015 | 1.02694788 |
| 6 | 2.67000367 | -0.03448389 | 2.22145804 |
| 1 | 6.43178507 | 1.64590418 | 0.02820665 |
| 6 | 2.58354111 | 1.37943085 | -2.70018060 |
| 6 | -0.36447279 | -1.81373203 | 2.54011736 |
| 6 | -0.73853232 | -3.18328897 | 2.38773452 |

| | | | |
|---|---|---|---|
| 6 | -0.23276691 | -4.09849885 | 3.32092986 |
| 6 | 0.61022604 | -3.72235654 | 4.37104508 |
| 6 | 0.96290671 | -2.38487317 | 4.51235061 |
| 6 | 0.47489753 | -1.42704889 | 3.61604363 |
| 6 | 0.79589603 | 0.02597509 | 3.84273490 |
| 1 | 0.98424393 | -4.47191426 | 5.06832552 |
| 6 | -1.66092666 | -3.64145136 | 1.24056058 |
| 6 | 3.52759163 | 1.87239098 | -3.81283279 |
| 6 | 1.44619006 | 2.41940333 | -2.56760738 |
| 6 | 2.00191753 | 0.02049931 | -3.15803205 |
| 6 | -1.93961751 | -5.15536397 | 1.29384200 |
| 6 | -0.99979848 | -3.34226523 | -0.12595808 |
| 6 | -3.02617513 | -2.91920022 | 1.33570491 |
| 1 | 2.52600503 | 2.07008723 | 3.93024833 |
| 1 | 2.27801516 | 2.62556096 | 2.24596439 |
| 1 | 3.38055366 | -0.10394137 | 3.06328995 |
| 1 | 2.32813088 | -1.05505961 | 1.99324376 |
| 1 | 5.21640242 | 1.89269074 | -2.09483283 |
| 1 | 5.23563076 | 0.78040538 | 2.04599629 |
| 1 | -0.12022133 | 0.60363445 | 4.04724267 |
| 1 | 1.45751153 | 0.12162930 | 4.72082580 |
| 1 | -0.49810577 | -5.15008553 | 3.22732478 |
| 1 | 1.61390393 | -2.06733506 | 5.33018527 |
| 1 | 2.95987027 | 1.96209152 | -4.75019991 |
| 1 | 3.95278231 | 2.86132886 | -3.58866917 |
| 1 | 4.35484096 | 1.17092260 | -3.99284034 |
| 1 | 0.71202425 | 2.10404579 | -1.81869988 |
| 1 | 1.84795356 | 3.40039347 | -2.27489996 |
| 1 | 0.93416315 | 2.53982107 | -3.53522060 |
| 1 | 2.80577229 | -0.71749119 | -3.29381231 |
| 1 | 1.28601062 | -0.37479399 | -2.42913367 |
| 1 | 1.49209834 | 0.14295675 | -4.12691946 |
| 1 | -2.60593786 | -5.42600140 | 0.46214298 |
| 1 | -2.43876044 | -5.45181606 | 2.22770677 |
| 1 | -1.02062666 | -5.74949127 | 1.18682301 |
| 1 | -0.81704699 | -2.27095088 | -0.26035928 |
| 1 | -0.04088685 | -3.87284241 | -0.21629748 |
| 1 | -1.65518545 | -3.69036169 | -0.93966642 |
| 1 | -3.52812506 | -3.16226217 | 2.28364063 |
| 1 | -2.90439076 | -1.83200128 | 1.27529108 |
| 1 | -3.68020226 | -3.25068279 | 0.51384428 |
| 1 | 0.90437028 | 2.63469250 | 3.38084221 |
| 31 | 0.06506757 | 0.65242119 | 1.10912343 |
| 6 | -3.99557313 | 1.99800127 | -3.33950003 |
| 6 | -3.50971946 | 1.11934517 | -2.17697214 |
| 8 | -3.18484105 | 2.04453458 | -1.08867049 |
| 6 | -2.13719691 | 1.73008902 | -0.32670429 |
| 6 | -1.83179690 | 2.72381516 | 0.79044068 |
| 8 | -1.45065529 | 0.71487686 | -0.49109900 |
| 6 | -1.62213100 | 4.13724310 | 0.22163435 |
| 6 | -4.61647457 | 0.17190441 | -1.71926082 |
| 1 | -2.59723273 | 0.56661521 | -2.42447701 |
| 1 | -2.74224991 | 2.73652292 | 1.42732576 |

| | | | |
|---|---|---|---|
| 1 | -1.42293401 | 4.82057850 | 1.05613166 |
| 1 | -2.51005574 | 4.48477608 | -0.32202477 |
| 1 | -0.75405882 | 4.15012039 | -0.45113278 |
| 1 | -4.91687058 | -0.49360366 | -2.54029911 |
| 1 | -5.49367852 | 0.74961624 | -1.40280372 |
| 1 | -4.26157926 | -0.44792550 | -0.88605917 |

Electronic energy = -3673.053103 a.u.
DFT-D3(BJ) dispersion correction = -0.114521 a.u.
Thermal free energy = 0.581284 a.u.
Gibbs free energy = -3672.586339 a.u.
Number of imaginary frequencies = 0.

**Int2 LA isomer/conformer 21**

| Atomic N. | X | Y | Z |
|---|---|---|---|
| 7 | 2.20940690 | -0.61594542 | 0.30656862 |
| 8 | -0.51975481 | 0.63945490 | 0.54770659 |
| 8 | 1.55739192 | 1.66939504 | -1.51494603 |
| 6 | 2.24080115 | -2.10108087 | 0.27950877 |
| 6 | -0.51810153 | 0.03772439 | 1.76518077 |
| 6 | -1.73931644 | -0.07546777 | 2.49458914 |
| 6 | -1.68865044 | -0.72712698 | 3.73554587 |
| 6 | -0.50765214 | -1.24548478 | 4.27423169 |
| 6 | 0.68348348 | -1.08396426 | 3.57562673 |
| 6 | 0.69548962 | -0.42932898 | 2.33831178 |
| 6 | 2.02330656 | -0.09686203 | 1.70715788 |
| 1 | -0.52138667 | -1.75274853 | 5.23892377 |
| 6 | -3.05865417 | 0.52974775 | 1.97276916 |
| 6 | 2.63891775 | 2.23912983 | -0.93242966 |
| 6 | 2.82129439 | 3.65162727 | -1.00068946 |
| 6 | 3.96864220 | 4.18038265 | -0.39326507 |
| 6 | 4.91505642 | 3.38711147 | 0.26259869 |
| 6 | 4.73021174 | 2.01051457 | 0.31422094 |
| 6 | 3.60815053 | 1.42761796 | -0.28758348 |
| 6 | 3.48280811 | -0.07320608 | -0.30674954 |
| 1 | 5.78811588 | 3.84703407 | 0.72524679 |
| 6 | 1.80153799 | 4.56280351 | -1.71166620 |
| 6 | -4.22498682 | 0.29603629 | 2.95180464 |
| 6 | -3.46134470 | -0.10616094 | 0.62293551 |
| 6 | -2.89547511 | 2.05948554 | 1.80403295 |
| 6 | 2.23076188 | 6.04179577 | -1.67733917 |
| 6 | 0.42486701 | 4.46803891 | -1.01187268 |
| 6 | 1.67046458 | 4.15574960 | -3.19886903 |
| 1 | 3.12482573 | -2.46141134 | 0.82980586 |
| 1 | 1.33317100 | -2.49582415 | 0.74664902 |
| 1 | 2.84361415 | -0.48865907 | 2.33141887 |
| 1 | 2.15057146 | 0.99432350 | 1.64099185 |
| 1 | -2.60678100 | -0.83883051 | 4.30926792 |
| 1 | 1.62547766 | -1.44115371 | 3.99764478 |
| 1 | 3.49600583 | -0.45783615 | -1.33924178 |
| 1 | 4.33352553 | -0.52254048 | 0.23148872 |
| 1 | 4.13347771 | 5.25580840 | -0.42459635 |
| 1 | 5.46328467 | 1.37106375 | 0.81111513 |

| | | | |
|---|---|---|---|
| 1 | -5.13223355 | 0.76110588 | 2.54111489 |
| 1 | -4.43823410 | -0.77339403 | 3.09488986 |
| 1 | -4.03909197 | 0.75011054 | 3.93564099 |
| 1 | -2.72675971 | 0.12460409 | -0.15547478 |
| 1 | -3.55032066 | -1.19862379 | 0.71032999 |
| 1 | -4.43655792 | 0.28989726 | 0.30197710 |
| 1 | -2.66295550 | 2.53296706 | 2.76885020 |
| 1 | -2.09625870 | 2.30376823 | 1.09527451 |
| 1 | -3.83514434 | 2.49512650 | 1.43181011 |
| 1 | 1.47673300 | 6.64390376 | -2.20385583 |
| 1 | 3.19434494 | 6.20468941 | -2.18148726 |
| 1 | 2.30408089 | 6.42948946 | -0.65089280 |
| 1 | 0.02561438 | 3.44856724 | -1.03748399 |
| 1 | 0.50072507 | 4.78496935 | 0.03843657 |
| 1 | -0.29370685 | 5.13283188 | -1.51481138 |
| 1 | 2.63570836 | 4.26337575 | -3.71499980 |
| 1 | 1.33233517 | 3.11959369 | -3.30605110 |
| 1 | 0.94501819 | 4.81431567 | -3.70035669 |
| 1 | 2.26751861 | -2.45033317 | -0.75785751 |
| 8 | -2.04721045 | -4.11888340 | 0.75402262 |
| 6 | -1.56441872 | -4.35896287 | 2.10016749 |
| 1 | -2.10383076 | -5.20122850 | 2.55146307 |
| 8 | -1.37757618 | -6.22455794 | 0.20449955 |
| 8 | 0.17393551 | -0.82752227 | -2.19880227 |
| 1 | -1.75277225 | -3.43013366 | 2.64631631 |
| 1 | -0.49197812 | -4.58893029 | 2.07969562 |
| 31 | 0.69539664 | 0.19549923 | -0.79226082 |
| 6 | -1.85037674 | -5.14877916 | -0.10407188 |
| 6 | -2.42793818 | -4.84022491 | -1.49352528 |
| 8 | -2.29924192 | -3.43843900 | -1.87649532 |
| 6 | -1.04308275 | -2.90859696 | -1.84744974 |
| 6 | -1.06957707 | -1.46420021 | -2.37771452 |
| 8 | -0.06071044 | -3.51071403 | -1.45970497 |
| 6 | -1.40424357 | -1.47061861 | -3.87739964 |
| 6 | -1.90635906 | -5.78383407 | -2.56699408 |
| 1 | -3.51912452 | -4.95512606 | -1.39538495 |
| 1 | -1.88908269 | -0.95510836 | -1.83776811 |
| 1 | -1.44456641 | -0.43430540 | -4.23530897 |
| 1 | -2.37028992 | -1.95546245 | -4.06660112 |
| 1 | -0.61712120 | -1.99789020 | -4.43325338 |
| 1 | -2.14249494 | -6.81753054 | -2.28711920 |
| 1 | -0.81958433 | -5.69687091 | -2.67267835 |
| 1 | -2.39112531 | -5.55575734 | -3.52477920 |

Electronic energy = -3673.052654 a.u.
DFT-D3(BJ) dispersion correction = -0.115161 a.u.
Thermal free energy = 0.581498 a.u.
Gibbs free energy = -3672.586318 a.u.
Number of imaginary frequencies = 0.

**Int2_LA isomer/conformer 22**

| Atomic N. | X | Y | Z |
|---|---|---|---|
| 7 | 0.50412965 | 0.84281496 | -2.27131300 |

| | | | |
|---|---|---|---|
| 8 | -0.61841735 | -0.84575336 | -0.02703599 |
| 8 | 2.02199323 | -1.53370064 | -1.30458321 |
| 6 | 0.56529213 | 2.32563400 | -2.25169599 |
| 6 | -1.75076435 | -0.13802383 | -0.24736698 |
| 6 | -2.80810529 | -0.16054926 | 0.70690502 |
| 6 | -3.92429577 | 0.64778481 | 0.44334379 |
| 6 | -4.03982189 | 1.42752601 | -0.71163649 |
| 6 | -3.03332964 | 1.37664161 | -1.67212108 |
| 6 | -1.89980459 | 0.58610774 | -1.45943587 |
| 6 | -0.90275511 | 0.37851177 | -2.56904169 |
| 1 | -4.92474724 | 2.04451438 | -0.86798474 |
| 6 | -2.75555310 | -1.07904621 | 1.94521408 |
| 6 | 1.77217022 | -2.02501762 | -2.53246614 |
| 6 | 1.87661278 | -3.42508123 | -2.79030647 |
| 6 | 1.60904529 | -3.85845939 | -4.09601186 |
| 6 | 1.25419927 | -2.98607553 | -5.13083471 |
| 6 | 1.17249806 | -1.62257407 | -4.87220552 |
| 6 | 1.44093616 | -1.13645366 | -3.58709513 |
| 6 | 1.46078525 | 0.34510867 | -3.33994173 |
| 1 | 1.04958973 | -3.37526121 | -6.12814793 |
| 6 | 2.26793768 | -4.41927249 | -1.68032452 |
| 6 | -4.02969161 | -0.96111569 | 2.80237750 |
| 6 | -1.55539547 | -0.73397169 | 2.85707810 |
| 6 | -2.64169710 | -2.55134786 | 1.48070942 |
| 6 | 2.35204908 | -5.86456062 | -2.20590716 |
| 6 | 1.20996895 | -4.39920178 | -0.55178628 |
| 6 | 3.65773924 | -4.05372170 | -1.10636348 |
| 1 | 0.35356258 | 2.71143480 | -3.26224144 |
| 1 | -0.16910309 | 2.71503409 | -1.54348354 |
| 1 | -1.24104485 | 0.89034237 | -3.48565914 |
| 1 | -0.81572719 | -0.69392566 | -2.79623525 |
| 1 | -4.74228854 | 0.67017933 | 1.16159046 |
| 1 | -3.13222071 | 1.93160547 | -2.60809856 |
| 1 | 2.45964938 | 0.67319173 | -3.00955478 |
| 1 | 1.21975286 | 0.87838338 | -4.27438751 |
| 1 | 1.67301593 | -4.92154410 | -4.32178865 |
| 1 | 0.91277309 | -0.92078937 | -5.66835289 |
| 1 | -3.94971657 | -1.64562649 | 3.65884761 |
| 1 | -4.16785984 | 0.05436719 | 3.20233808 |
| 1 | -4.93306044 | -1.23879083 | 2.24078124 |
| 1 | -0.60499523 | -0.84695972 | 2.32488555 |
| 1 | -1.63312010 | 0.29538420 | 3.23577966 |
| 1 | -1.54535445 | -1.40864760 | 3.72651528 |
| 1 | -3.51018981 | -2.83206342 | 0.86754318 |
| 1 | -1.73162531 | -2.71661503 | 0.89293832 |
| 1 | -2.61576646 | -3.21788172 | 2.35628409 |
| 1 | 2.64539158 | -6.52800053 | -1.37984159 |
| 1 | 3.10471694 | -5.97257787 | -3.00044473 |
| 1 | 1.38604514 | -6.22356651 | -2.58992090 |
| 1 | 1.12751021 | -3.40823224 | -0.09334292 |
| 1 | 0.22175478 | -4.68506184 | -0.94062870 |
| 1 | 1.48859392 | -5.12126499 | 0.23096949 |
| 1 | 4.42583375 | -4.10389319 | -1.89219108 |

| | | | |
|---|---|---|---|
| 1 | 3.66241527 | -3.04619318 | -0.67689047 |
| 1 | 3.93379137 | -4.77153660 | -0.31874833 |
| 1 | 1.56779445 | 2.64679664 | -1.94434919 |
| 8 | -0.61857102 | 6.23968594 | 2.96589277 |
| 6 | -0.26497126 | 7.48840223 | 3.61468193 |
| 1 | -0.28213518 | 7.36878271 | 4.70479894 |
| 8 | 1.18522117 | 5.27333105 | 3.96916118 |
| 8 | 2.31284401 | 0.36706329 | 0.69900108 |
| 1 | -1.02301255 | 8.20631665 | 3.28893554 |
| 1 | 0.73656186 | 7.80798064 | 3.30215081 |
| 31 | 1.04181601 | -0.14175171 | -0.52014130 |
| 6 | 0.21042662 | 5.20036215 | 3.25379257 |
| 6 | -0.30425081 | 3.94523865 | 2.54237169 |
| 8 | 0.74725353 | 2.94980255 | 2.67606856 |
| 6 | 0.91649563 | 2.09960360 | 1.64611685 |
| 6 | 2.07927696 | 1.12966136 | 1.84965927 |
| 8 | 0.21326896 | 2.08579702 | 0.64215108 |
| 6 | 3.36474163 | 1.86707915 | 2.24990306 |
| 6 | -1.61268492 | 3.43643992 | 3.14245169 |
| 1 | -0.44308831 | 4.16927893 | 1.47566858 |
| 1 | 1.77340819 | 0.48104353 | 2.69931417 |
| 1 | 4.15956363 | 1.12594646 | 2.39700396 |
| 1 | 3.22456313 | 2.43879534 | 3.17531909 |
| 1 | 3.67461228 | 2.54952836 | 1.44676978 |
| 1 | -2.37161353 | 4.22750843 | 3.09985797 |
| 1 | -1.46564096 | 3.13833939 | 4.18878140 |
| 1 | -1.97518195 | 2.57675994 | 2.56595411 |

Electronic energy = -3673.054748 a.u.
DFT-D3(BJ) dispersion correction = -0.114989 a.u.
Thermal free energy = 0.583557 a.u.
Gibbs free energy = -3672.586181 a.u.
Number of imaginary frequencies = 0.

**Int2_LA isomer/conformer 23**

| Atomic N. | X | Y | Z |
|---|---|---|---|
| 7 | -0.80978090 | 1.64318769 | 2.10833030 |
| 8 | -0.76620152 | 1.37509446 | -0.84464900 |
| 8 | 1.42063683 | 0.00398763 | 1.02287223 |
| 6 | -2.18415698 | 1.53569141 | 2.65408491 |
| 6 | -1.50364270 | 2.50556290 | -0.79254053 |
| 6 | -2.26466386 | 2.90156367 | -1.93492083 |
| 6 | -3.02567430 | 4.07428388 | -1.83026930 |
| 6 | -3.05450599 | 4.85886102 | -0.67382020 |
| 6 | -2.28249386 | 4.48165653 | 0.41963911 |
| 6 | -1.49789946 | 3.32447734 | 0.36669822 |
| 6 | -0.57845933 | 3.00055032 | 1.51242800 |
| 1 | -3.66648460 | 5.76018560 | -0.63803491 |
| 6 | -2.23602975 | 2.08843794 | -3.24472261 |
| 6 | 2.18350935 | 0.83275407 | 1.76214617 |
| 6 | 3.57726055 | 0.95368188 | 1.47488954 |
| 6 | 4.32868986 | 1.81879740 | 2.28173205 |
| 6 | 3.77084755 | 2.55259537 | 3.33357230 |

| | | | |
|---|---|---|---|
| 6 | 2.41426249 | 2.42102895 | 3.60771860 |
| 6 | 1.61967957 | 1.56045061 | 2.84117311 |
| 6 | 0.17517446 | 1.36384627 | 3.21378807 |
| 1 | 4.39572647 | 3.21882721 | 3.92843599 |
| 6 | 4.23174280 | 0.16467080 | 0.32386319 |
| 6 | -3.11351702 | 2.72446678 | -4.33913297 |
| 6 | -2.76735721 | 0.65639863 | -3.00338327 |
| 6 | -0.78961882 | 2.02199396 | -3.79089296 |
| 6 | 5.74235593 | 0.44784723 | 0.21965742 |
| 6 | 3.59441170 | 0.56449565 | -1.02815014 |
| 6 | 4.06305212 | -1.35575377 | 0.55760360 |
| 1 | -2.33201917 | 2.28859734 | 3.44699531 |
| 1 | -2.91293675 | 1.70956118 | 1.85552032 |
| 1 | -0.68725675 | 3.75856155 | 2.30765154 |
| 1 | 0.47145376 | 3.01636438 | 1.18378092 |
| 1 | -3.62304930 | 4.39389856 | -2.68230292 |
| 1 | -2.26701597 | 5.09370340 | 1.32453646 |
| 1 | -0.01428254 | 0.32138611 | 3.51747533 |
| 1 | -0.07388128 | 2.01586626 | 4.06892761 |
| 1 | 5.39276627 | 1.93443607 | 2.08315560 |
| 1 | 1.95818948 | 2.97800841 | 4.42963347 |
| 1 | -3.04743646 | 2.11230717 | -5.25002624 |
| 1 | -4.17299436 | 2.77129636 | -4.04743443 |
| 1 | -2.77931242 | 3.73932822 | -4.59831692 |
| 1 | -2.13623474 | 0.12377027 | -2.28451010 |
| 1 | -3.79986626 | 0.68375344 | -2.62373274 |
| 1 | -2.76760803 | 0.09372044 | -3.94957296 |
| 1 | -0.41240054 | 3.03229756 | -4.00621060 |
| 1 | -0.11322371 | 1.54111864 | -3.07595626 |
| 1 | -0.77183269 | 1.44706865 | -4.72958205 |
| 1 | 6.15883153 | -0.14263711 | -0.60893948 |
| 1 | 6.28157436 | 0.16089663 | 1.13428666 |
| 1 | 5.95268059 | 1.50663622 | 0.00907695 |
| 1 | 2.52417771 | 0.33585700 | -1.05467047 |
| 1 | 3.72776389 | 1.63989864 | -1.21677303 |
| 1 | 4.08460950 | 0.01424615 | -1.84584087 |
| 1 | 4.55124976 | -1.65868005 | 1.49575897 |
| 1 | 3.00745831 | -1.64299191 | 0.60477637 |
| 1 | 4.53874525 | -1.91151142 | -0.26505499 |
| 1 | -2.33051097 | 0.52870226 | 3.05715593 |
| 8 | -0.53624923 | -6.37901209 | 0.92164434 |
| 6 | 0.20778498 | -6.87955147 | 2.06618438 |
| 1 | 0.13433677 | -6.17357757 | 2.90197125 |
| 8 | 0.79489248 | -4.52766759 | 0.92670835 |
| 8 | -1.58439810 | -1.03364087 | 1.28375499 |
| 1 | -0.26152589 | -7.83478354 | 2.31686348 |
| 1 | 1.26213851 | -7.01823270 | 1.79941008 |
| 31 | -0.38542613 | 0.23772943 | 0.59647907 |
| 6 | -0.11362331 | -5.17729682 | 0.45771248 |
| 6 | -0.97938874 | -4.75937883 | -0.74374053 |
| 8 | -0.52779968 | -3.47520053 | -1.23416962 |
| 6 | -0.71901511 | -2.34645516 | -0.52899783 |
| 6 | -1.76992182 | -2.22276735 | 0.58229577 |

| | | | |
|---|---|---|---|
| 8 | -0.07292470 | -1.35561813 | -0.86998538 |
| 6 | -3.18314047 | -2.26153210 | -0.04526252 |
| 6 | -0.85943745 | -5.72971530 | -1.91453850 |
| 1 | -2.02462658 | -4.70561646 | -0.40720209 |
| 1 | -1.65268708 | -3.08108449 | 1.27203163 |
| 1 | -3.91338139 | -2.14830885 | 0.76539927 |
| 1 | -3.40216889 | -3.19506385 | -0.58331749 |
| 1 | -3.30190431 | -1.42008292 | -0.73981323 |
| 1 | -1.18085416 | -6.73046804 | -1.60420095 |
| 1 | 0.17929866 | -5.77716635 | -2.26501060 |
| 1 | -1.49448018 | -5.39061031 | -2.74213984 |

Electronic energy = -3673.053800 a.u.
DFT-D3(BJ) dispersion correction = -0.114120 a.u.
Thermal free energy = 0.581850 a.u.
Gibbs free energy = -3672.586070 a.u.
Number of imaginary frequencies = 0.

**Int2 LA isomer/conformer 24**

| Atomic N. | X | Y | Z |
|---|---|---|---|
| 8 | -1.82972215 | -3.48623982 | 4.38283476 |
| 6 | -3.09473521 | -3.72683931 | 5.04928395 |
| 1 | -3.54283188 | -4.65775932 | 4.68130782 |
| 8 | -1.05701494 | -5.42779473 | 5.28851238 |
| 8 | -0.05411214 | -1.24042626 | 1.09708955 |
| 1 | -3.72099699 | -2.86648329 | 4.79629795 |
| 1 | -2.94708467 | -3.80036575 | 6.13377884 |
| 7 | 0.60045270 | 1.71165330 | 1.41285608 |
| 8 | -0.92924523 | 0.94460742 | -1.05368914 |
| 8 | 2.03121898 | 0.15741093 | -0.73702271 |
| 6 | 0.05375212 | 1.38331925 | 2.75521161 |
| 6 | -1.89425825 | 1.72887785 | -0.49874879 |
| 6 | -3.24515611 | 1.62241857 | -0.93754615 |
| 6 | -4.18770362 | 2.45595175 | -0.31723438 |
| 6 | -3.85016256 | 3.37040170 | 0.68424066 |
| 6 | -2.52172359 | 3.48857506 | 1.07861382 |
| 6 | -1.53885764 | 2.68786232 | 0.48655705 |
| 6 | -0.08810047 | 2.91639049 | 0.82124299 |
| 1 | -4.62133645 | 3.99128441 | 1.13998282 |
| 6 | -3.65892071 | 0.64802182 | -2.05820391 |
| 6 | 2.74498980 | 1.30648482 | -0.86569636 |
| 6 | 3.49169678 | 1.55426050 | -2.05371459 |
| 6 | 4.21723381 | 2.75257709 | -2.11623361 |
| 6 | 4.23064525 | 3.68751904 | -1.07715092 |
| 6 | 3.50960190 | 3.42673806 | 0.08231043 |
| 6 | 2.77765046 | 2.23940325 | 0.20376743 |
| 6 | 2.09444466 | 1.92729848 | 1.51029435 |
| 1 | 4.80481870 | 4.60839329 | -1.17758104 |
| 6 | 3.50498818 | 0.55213193 | -3.22495358 |
| 6 | -5.16389407 | 0.73831305 | -2.37341608 |
| 6 | -3.36402897 | -0.81128619 | -1.64089067 |
| 6 | -2.89242859 | 0.98823176 | -3.35870552 |
| 6 | 4.41020382 | 1.02648374 | -4.37754605 |

| | | | |
|---|---|---|---|
| 6 | 2.07852150 | 0.38743230 | -3.80041350 |
| 6 | 4.04729797 | -0.81631588 | -2.74795969 |
| 1 | 0.28475320 | 2.19817590 | 3.46000988 |
| 1 | -1.03181864 | 1.25919050 | 2.68331863 |
| 1 | 0.01278881 | 3.75472102 | 1.53026740 |
| 1 | 0.48065148 | 3.16915367 | -0.08560090 |
| 1 | -5.23044026 | 2.39055139 | -0.62229848 |
| 1 | -2.23116653 | 4.21911385 | 1.83702805 |
| 1 | 2.49437041 | 0.99783014 | 1.94607456 |
| 1 | 2.27435872 | 2.74393652 | 2.22877668 |
| 1 | 4.79239584 | 2.97404577 | -3.01330522 |
| 1 | 3.51915615 | 4.13650120 | 0.91256323 |
| 1 | -5.40084641 | 0.03610712 | -3.18517199 |
| 1 | -5.78672695 | 0.46598091 | -1.50899518 |
| 1 | -5.45578292 | 1.74326756 | -2.71061046 |
| 1 | -2.29242575 | -0.96900997 | -1.47920615 |
| 1 | -3.90100035 | -1.07058338 | -0.71688620 |
| 1 | -3.69708123 | -1.50043703 | -2.43139511 |
| 1 | -3.13321316 | 2.00757151 | -3.69355638 |
| 1 | -1.80829124 | 0.91535843 | -3.21841273 |
| 1 | -3.18850648 | 0.29166230 | -4.15776391 |
| 1 | 4.39301313 | 0.27254695 | -5.17724405 |
| 1 | 5.45592226 | 1.14732695 | -4.05972478 |
| 1 | 4.06565237 | 1.97575263 | -4.81261600 |
| 1 | 1.37828808 | 0.00711706 | -3.04937002 |
| 1 | 1.69596684 | 1.34894956 | -4.17194967 |
| 1 | 2.09777137 | -0.31868312 | -4.64429701 |
| 1 | 5.07927740 | -0.71668038 | -2.38091744 |
| 1 | 3.43228802 | -1.23652466 | -1.94508717 |
| 1 | 4.05616790 | -1.52538708 | -3.58957981 |
| 1 | 0.49322221 | 0.44286827 | 3.10533055 |
| 31 | 0.35870271 | 0.20209389 | 0.06909122 |
| 6 | -0.90448619 | -4.45312099 | 4.57821554 |
| 6 | 0.41796463 | -4.17297032 | 3.85674777 |
| 8 | 0.31120069 | -3.17273398 | 2.81246197 |
| 6 | -0.36367021 | -3.57805926 | 1.70325903 |
| 6 | -0.42617523 | -2.50937302 | 0.60184745 |
| 8 | -0.85497917 | -4.68423448 | 1.57250696 |
| 6 | 0.42619567 | -2.98582488 | -0.58419712 |
| 6 | 1.46884843 | -3.67479982 | 4.84405134 |
| 1 | 0.71851172 | -5.13020526 | 3.40861893 |
| 1 | -1.48790430 | -2.50988537 | 0.28887606 |
| 1 | 0.27631895 | -2.31686997 | -1.44528570 |
| 1 | 0.13465370 | -4.00009877 | -0.88554856 |
| 1 | 1.49194282 | -2.97581944 | -0.31779760 |
| 1 | 1.58315590 | -4.40403493 | 5.65547123 |
| 1 | 1.17313958 | -2.70749970 | 5.27126418 |
| 1 | 2.43263364 | -3.55551896 | 4.33356115 |

Electronic energy = -3673.051941 a.u.
DFT-D3(BJ) dispersion correction = -0.111821 a.u.
Thermal free energy = 0.578448 a.u.
Gibbs free energy = -3672.585313 a.u.
Number of imaginary frequencies = 0.

**Int2 LA isomer/conformer 25**

| Atomic N. | X | Y | Z |
|---|---|---|---|
| 7 | -1.55425007 | -1.78400201 | 0.28574913 |
| 8 | 0.37179878 | 0.51383425 | 0.62993712 |
| 8 | 1.35114773 | -2.37182069 | 0.67268228 |
| 6 | -2.56686845 | -1.78797093 | -0.79980288 |
| 6 | -0.75694906 | 1.24382317 | 0.81697224 |
| 6 | -0.69719213 | 2.66645664 | 0.78137156 |
| 6 | -1.90370740 | 3.36205809 | 0.94533663 |
| 6 | -3.12632450 | 2.71866376 | 1.16110219 |
| 6 | -3.15850526 | 1.33065143 | 1.25738263 |
| 6 | -1.98203751 | 0.58859698 | 1.10911495 |
| 6 | -1.98786881 | -0.88562103 | 1.41840860 |
| 1 | -4.03982635 | 3.30273803 | 1.27631119 |
| 6 | 0.64213865 | 3.41821608 | 0.63268138 |
| 6 | 0.93375353 | -2.96248477 | 1.81279230 |
| 6 | 1.85508474 | -3.21985277 | 2.87105415 |
| 6 | 1.35318088 | -3.84703791 | 4.01975763 |
| 6 | 0.01214577 | -4.21962978 | 4.15895056 |
| 6 | -0.87316224 | -3.97611486 | 3.11520007 |
| 6 | -0.42114611 | -3.36327768 | 1.94028501 |
| 6 | -1.36203909 | -3.20558540 | 0.77749837 |
| 1 | -0.32990526 | -4.69861652 | 5.07628425 |
| 6 | 3.34047976 | -2.82565745 | 2.75856226 |
| 6 | 0.44397474 | 4.94597089 | 0.62905582 |
| 6 | 1.35588887 | 3.04971399 | -0.68842874 |
| 6 | 1.55395644 | 3.06679723 | 1.83318798 |
| 6 | 4.14176395 | -3.24010872 | 4.00694893 |
| 6 | 3.47350183 | -1.29113779 | 2.61386087 |
| 6 | 3.98606494 | -3.52991061 | 1.54123218 |
| 1 | -3.50370336 | -2.23029962 | -0.42376618 |
| 1 | -2.74284183 | -0.76464289 | -1.13972331 |
| 1 | -2.99510650 | -1.20245583 | 1.73700427 |
| 1 | -1.29353465 | -1.09839833 | 2.24478421 |
| 1 | -1.89408850 | 4.45000805 | 0.90411762 |
| 1 | -4.09411992 | 0.81190950 | 1.47897632 |
| 1 | -1.00043963 | -3.76891591 | -0.09780698 |
| 1 | -2.35491529 | -3.60088862 | 1.04877603 |
| 1 | 2.03043980 | -4.05070968 | 4.84731268 |
| 1 | -1.92179797 | -4.27159780 | 3.19650662 |
| 1 | 1.42619610 | 5.43107970 | 0.53855939 |
| 1 | -0.16835158 | 5.27820627 | -0.22161819 |
| 1 | -0.01724772 | 5.30874283 | 1.55904595 |
| 1 | 1.62055190 | 1.98715529 | -0.71031580 |
| 1 | 0.72244584 | 3.28017763 | -1.55541619 |
| 1 | 2.28404442 | 3.63477277 | -0.77729950 |
| 1 | 1.08567168 | 3.37177968 | 2.78026385 |
| 1 | 1.76200857 | 1.99139736 | 1.87769425 |
| 1 | 2.51174765 | 3.60139240 | 1.74139491 |
| 1 | 5.19235093 | -2.94698980 | 3.86971340 |
| 1 | 4.12036823 | -4.32748423 | 4.16992674 |

| | | | |
|---|---|---|---|
| 1 | 3.77754059 | -2.74365148 | 4.91811220 |
| 1 | 2.96158241 | -0.92251458 | 1.71878571 |
| 1 | 3.04967682 | -0.77984373 | 3.49034170 |
| 1 | 4.53676660 | -1.01585254 | 2.54189725 |
| 1 | 3.93506900 | -4.62275484 | 1.65533982 |
| 1 | 3.48825091 | -3.25332401 | 0.60566454 |
| 1 | 5.04759572 | -3.24753329 | 1.47016076 |
| 1 | -2.19279363 | -2.38186575 | -1.64217072 |
| 8 | -3.23263595 | 2.74319343 | -3.64895504 |
| 6 | -3.68627338 | 3.75564379 | -2.71179443 |
| 1 | -3.29920689 | 3.53532710 | -1.70940131 |
| 8 | -1.09841186 | 3.43686591 | -3.26760631 |
| 8 | 0.89458741 | -1.36076400 | -1.96419281 |
| 1 | -4.77835026 | 3.69465554 | -2.73241388 |
| 1 | -3.34215552 | 4.74779103 | -3.02888075 |
| 31 | 0.31963225 | -1.12260355 | -0.25060023 |
| 6 | -1.89005363 | 2.69139529 | -3.80804001 |
| 6 | -1.48597937 | 1.64008001 | -4.85612422 |
| 8 | -0.25311375 | 0.96913104 | -4.45137315 |
| 6 | -0.25861585 | 0.35257064 | -3.24389488 |
| 6 | 1.06647025 | -0.36427604 | -2.94332892 |
| 8 | -1.20133941 | 0.38277241 | -2.46989800 |
| 6 | 1.69405980 | -1.00543801 | -4.18440193 |
| 6 | -2.55625565 | 0.64180435 | -5.27484145 |
| 1 | -1.15642188 | 2.21875859 | -5.73263203 |
| 1 | 1.74354712 | 0.44211438 | -2.58872540 |
| 1 | 2.62823936 | -1.49779235 | -3.88824978 |
| 1 | 1.91141158 | -0.25080054 | -4.94959972 |
| 1 | 1.02105720 | -1.76406810 | -4.60611077 |
| 1 | -3.39627461 | 1.17372274 | -5.73842846 |
| 1 | -2.93563343 | 0.07788099 | -4.41638684 |
| 1 | -2.13674851 | -0.05355483 | -6.01386973 |

Electronic energy = -3673.051872 a.u.
DFT-D3(BJ) dispersion correction = -0.116509 a.u.
Thermal free energy = 0.585079 a.u.
Gibbs free energy = -3672.583301 a.u.
Number of imaginary frequencies = 0.

**Int2 LA isomer/conformer 26**

| Atomic N. | X | Y | Z |
|---|---|---|---|
| 8 | 4.47785442 | -1.64911122 | -1.46518260 |
| 6 | 4.21196848 | -3.05903312 | -1.28033101 |
| 1 | 4.58348973 | -3.43470017 | -0.32089513 |
| 8 | 4.76918817 | 0.40975956 | -0.70120449 |
| 8 | -0.57030920 | -2.33984606 | 1.13758366 |
| 1 | 4.74293203 | -3.55561015 | -2.09962597 |
| 1 | 3.13711578 | -3.25624443 | -1.37209113 |
| 7 | -2.89383724 | -0.59122562 | 0.75650532 |
| 8 | -0.72364685 | -0.02814719 | -1.18147869 |
| 8 | -0.49799425 | 0.74351366 | 1.91861504 |
| 6 | -3.47879601 | -1.94161731 | 0.56890749 |
| 6 | -1.72135985 | -0.17664392 | -2.08257700 |

| | | | |
|---|---|---|---|
| 6 | -1.40136669 | -0.44026757 | -3.44872211 |
| 6 | -2.46932194 | -0.57881806 | -4.34643135 |
| 6 | -3.80694355 | -0.46685011 | -3.95648993 |
| 6 | -4.10494102 | -0.19291385 | -2.62600656 |
| 6 | -3.07721223 | -0.03437464 | -1.68998969 |
| 6 | -3.41290204 | 0.36521129 | -0.27908154 |
| 1 | -4.60345948 | -0.58711702 | -4.69080575 |
| 6 | 0.05958156 | -0.55908409 | -3.92624802 |
| 6 | -1.40396815 | 1.64785579 | 2.34891330 |
| 6 | -0.97403082 | 2.95438964 | 2.73043750 |
| 6 | -1.95435766 | 3.84703647 | 3.18532794 |
| 6 | -3.30813744 | 3.50785899 | 3.27154129 |
| 6 | -3.71317444 | 2.23030985 | 2.90161735 |
| 6 | -2.77328541 | 1.29493937 | 2.45333842 |
| 6 | -3.21770770 | -0.10991591 | 2.14700921 |
| 1 | -4.03434589 | 4.23998901 | 3.62458206 |
| 6 | 0.50696972 | 3.37293826 | 2.64198846 |
| 6 | 0.15336169 | -0.81671769 | -5.44191966 |
| 6 | 0.75669679 | -1.74397158 | -3.21613391 |
| 6 | 0.82529489 | 0.75327459 | -3.63329276 |
| 6 | 0.72751343 | 4.81732906 | 3.12971248 |
| 6 | 0.99410012 | 3.30234336 | 1.17525626 |
| 6 | 1.37469032 | 2.45123771 | 3.53230558 |
| 1 | -4.57523636 | -1.89330670 | 0.67956478 |
| 1 | -3.23553361 | -2.31260379 | -0.43207405 |
| 1 | -4.50705529 | 0.45117739 | -0.16183845 |
| 1 | -2.97637200 | 1.34566273 | -0.03672571 |
| 1 | -2.25525594 | -0.78504742 | -5.39361288 |
| 1 | -5.14237121 | -0.08275357 | -2.30170007 |
| 1 | -2.73425518 | -0.83105264 | 2.82603612 |
| 1 | -4.30885371 | -0.18913734 | 2.29217267 |
| 1 | -1.65725772 | 4.85211055 | 3.47941763 |
| 1 | -4.76456298 | 1.94105962 | 2.96822248 |
| 1 | 1.21301193 | -0.88470662 | -5.72680511 |
| 1 | -0.33037042 | -1.76006200 | -5.73496602 |
| 1 | -0.29390091 | -0.00018815 | -6.02677032 |
| 1 | 0.75563123 | -1.60330393 | -2.12995089 |
| 1 | 0.24866842 | -2.69117418 | -3.45161166 |
| 1 | 1.80014667 | -1.81897268 | -3.55902950 |
| 1 | 0.36180903 | 1.59534818 | -4.16762948 |
| 1 | 0.83649140 | 0.98341302 | -2.56285652 |
| 1 | 1.86572080 | 0.66368029 | -3.98037438 |
| 1 | 1.79696293 | 5.06022305 | 3.05328003 |
| 1 | 0.43143220 | 4.94994987 | 4.18057364 |
| 1 | 0.17946341 | 5.54875899 | 2.51841275 |
| 1 | 0.91849319 | 2.28830642 | 0.76977081 |
| 1 | 0.40311168 | 3.97665849 | 0.53871274 |
| 1 | 2.04662830 | 3.61838547 | 1.11590528 |
| 1 | 1.06903148 | 2.53053282 | 4.58604387 |
| 1 | 1.28931425 | 1.40408383 | 3.22177073 |
| 1 | 2.43043668 | 2.75548282 | 3.46227362 |
| 1 | -3.05364529 | -2.62170509 | 1.31391745 |
| 31 | -0.80070982 | -0.55902897 | 0.60870999 |

| | | | |
|---|---|---|---|
| 6 | 4.42845456 | -0.72949161 | -0.46869897 |
| 6 | 4.04438718 | -1.08294597 | 0.98479363 |
| 8 | 3.03168429 | -2.13709439 | 1.16276069 |
| 6 | 1.75574531 | -1.77798684 | 0.96839422 |
| 6 | 0.71639730 | -2.82282077 | 1.37360384 |
| 8 | 1.42633015 | -0.67557884 | 0.52787326 |
| 6 | 0.94749282 | -4.16451835 | 0.65973665 |
| 6 | 5.26878346 | -1.50399978 | 1.78953307 |
| 1 | 3.62082830 | -0.15251058 | 1.38081478 |
| 1 | 0.88298611 | -2.97827892 | 2.46245526 |
| 1 | 0.19180902 | -4.87758664 | 1.01120792 |
| 1 | 1.94430490 | -4.57148362 | 0.87644655 |
| 1 | 0.82431751 | -4.04217625 | -0.42484536 |
| 1 | 6.02822458 | -0.71541967 | 1.71799729 |
| 1 | 5.70238935 | -2.44203375 | 1.41976469 |
| 1 | 4.99523275 | -1.63364139 | 2.84418891 |

Electronic energy = -3673.049068 a.u.
DFT-D3(BJ) dispersion correction = -0.116006 a.u.
Thermal free energy = 0.581960 a.u.
Gibbs free energy = -3672.583114 a.u.
Number of imaginary frequencies = 0.

**Int2_LA isomer/conformer 27**

| Atomic N. | X | Y | Z |
|---|---|---|---|
| 7 | -1.84450841 | -1.41152025 | 0.09964261 |
| 8 | 0.35204249 | 0.61851393 | 0.58698671 |
| 8 | 0.70028959 | -2.24243380 | 1.39924628 |
| 6 | -2.59775724 | -1.47265667 | -1.17868273 |
| 6 | -0.63167628 | 1.51651954 | 0.36515608 |
| 6 | -0.30960254 | 2.88138755 | 0.10255762 |
| 6 | -1.37602627 | 3.75934698 | -0.14246788 |
| 6 | -2.71357365 | 3.35094723 | -0.12511552 |
| 6 | -3.01628680 | 2.02605116 | 0.17742059 |
| 6 | -1.99192976 | 1.11096222 | 0.44047690 |
| 6 | -2.33424077 | -0.26486510 | 0.95039750 |
| 1 | -3.50842818 | 4.07052988 | -0.32280084 |
| 6 | 1.14873961 | 3.38238805 | 0.12149794 |
| 6 | -0.09214147 | -2.56356653 | 2.43576304 |
| 6 | 0.45740890 | -2.72226783 | 3.74457294 |
| 6 | -0.42627435 | -3.06857237 | 4.77534997 |
| 6 | -1.79726031 | -3.26024872 | 4.57123953 |
| 6 | -2.31970094 | -3.11542439 | 3.29122851 |
| 6 | -1.47789194 | -2.78237979 | 2.22314195 |
| 6 | -2.03642158 | -2.72963947 | 0.82921748 |
| 1 | -2.44374493 | -3.52292182 | 5.40850258 |
| 6 | 1.96120155 | -2.52067545 | 4.01215685 |
| 6 | 1.24226956 | 4.89583756 | -0.14873262 |
| 6 | 1.98746673 | 2.66955963 | -0.96501521 |
| 6 | 1.77258652 | 3.12156645 | 1.51369987 |
| 6 | 2.32317393 | -2.77837241 | 5.48683296 |
| 6 | 2.36734580 | -1.06412806 | 3.68479474 |
| 6 | 2.79003461 | -3.50574550 | 3.15338087 |

| | | | |
|---|---|---|---|
| 1 | -3.65379926 | -1.70649464 | -0.96858070 |
| 1 | -2.52971388 | -0.51355496 | -1.69638025 |
| 1 | -3.42677088 | -0.36656913 | 1.06177405 |
| 1 | -1.88314294 | -0.41910858 | 1.94149342 |
| 1 | -1.16140761 | 4.80611467 | -0.35173670 |
| 1 | -4.05602653 | 1.69646518 | 0.23973496 |
| 1 | -1.56379643 | -3.48860281 | 0.18469222 |
| 1 | -3.11863607 | -2.93745097 | 0.85880226 |
| 1 | -0.03670612 | -3.19054891 | 5.78461732 |
| 1 | -3.38487677 | -3.27267256 | 3.10549078 |
| 1 | 2.29744850 | 5.20162470 | -0.11031243 |
| 1 | 0.85606927 | 5.16566715 | -1.14297206 |
| 1 | 0.70041112 | 5.48443868 | 0.60524081 |
| 1 | 2.00896199 | 1.58652023 | -0.80385355 |
| 1 | 1.57894102 | 2.87419426 | -1.96660154 |
| 1 | 3.02217550 | 3.04362430 | -0.94260238 |
| 1 | 1.21375753 | 3.65865590 | 2.29358514 |
| 1 | 1.77506295 | 2.05411606 | 1.75966104 |
| 1 | 2.81099282 | 3.48611915 | 1.53073508 |
| 1 | 3.40461476 | -2.63174428 | 5.61965675 |
| 1 | 2.08817173 | -3.80708384 | 5.79661928 |
| 1 | 1.81006284 | -2.08369336 | 6.16776978 |
| 1 | 2.17785380 | -0.81887544 | 2.63451653 |
| 1 | 1.80851980 | -0.35536260 | 4.31332601 |
| 1 | 3.44031440 | -0.92331939 | 3.88660648 |
| 1 | 2.53960358 | -4.54586575 | 3.40918219 |
| 1 | 2.60896837 | -3.35605056 | 2.08358232 |
| 1 | 3.86282091 | -3.35859092 | 3.35177471 |
| 1 | -2.17467099 | -2.25494315 | -1.81962809 |
| 8 | -2.54832810 | 1.29674389 | -4.53353220 |
| 6 | -2.44399080 | 2.59646496 | -3.89936230 |
| 1 | -2.00801733 | 2.51106240 | -2.89559546 |
| 8 | -1.57203420 | -0.59652577 | -5.16784842 |
| 8 | 1.33180920 | -1.92505030 | -1.26292024 |
| 1 | -3.47637737 | 2.94983033 | -3.81585776 |
| 1 | -1.87255210 | 3.30341062 | -4.51692238 |
| 31 | 0.20633058 | -1.14199053 | -0.03929256 |
| 6 | -1.44414117 | 0.53904300 | -4.76656114 |
| 6 | -0.06025056 | 1.19620993 | -4.59172969 |
| 8 | 0.89786373 | 0.18484547 | -4.16430872 |
| 6 | 0.65547251 | -0.34776784 | -2.95413065 |
| 6 | 1.65784942 | -1.40829052 | -2.51968886 |
| 8 | -0.28408806 | 0.00166203 | -2.24480781 |
| 6 | 1.74594842 | -2.53936093 | -3.55616312 |
| 6 | 0.44389621 | 1.73777062 | -5.92351961 |
| 1 | -0.08327511 | 1.97535200 | -3.82062751 |
| 1 | 2.64088822 | -0.89071252 | -2.48267320 |
| 1 | 2.46307220 | -3.28511309 | -3.19263773 |
| 1 | 2.07986503 | -2.16082091 | -4.53037757 |
| 1 | 0.76752446 | -3.02278140 | -3.67639811 |
| 1 | -0.22566048 | 2.52613070 | -6.29301375 |
| 1 | 0.47962902 | 0.93256451 | -6.66763030 |
| 1 | 1.44826899 | 2.16212287 | -5.80104304 |

Electronic energy = -3673.048266 a.u.
DFT-D3(BJ) dispersion correction = -0.118515 a.u.
Thermal free energy = 0.583826 a.u.
Gibbs free energy = -3672.582955 a.u.
Number of imaginary frequencies = 0.

**Int2 LA isomer/conformer 28**

| Atomic N. | X | Y | Z |
|---|---|---|---|
| 8 | 2.55285665 | 1.00159670 | -3.91969150 |
| 6 | 2.07285132 | 0.06898159 | -4.91192644 |
| 1 | 1.55309372 | -0.76962478 | -4.43135764 |
| 8 | 2.30274268 | 2.80753801 | -2.65916875 |
| 8 | -1.58211904 | -1.57490904 | -1.55403463 |
| 1 | 2.97300012 | -0.30309127 | -5.41244451 |
| 1 | 1.41540395 | 0.54472449 | -5.65087987 |
| 7 | -1.10504328 | -2.47935571 | 1.18662090 |
| 8 | -1.04082625 | 0.47524391 | 0.94155753 |
| 8 | 1.29447774 | -1.40373375 | -0.20771651 |
| 6 | -2.45475299 | -3.02684090 | 0.90610143 |
| 6 | -1.91914740 | 0.40937745 | 1.96790691 |
| 6 | -2.74778558 | 1.53601829 | 2.25757812 |
| 6 | -3.63911831 | 1.42416653 | 3.33383120 |
| 6 | -3.73967375 | 0.27176646 | 4.11806505 |
| 6 | -2.91517757 | -0.81222839 | 3.83746793 |
| 6 | -1.99908896 | -0.75022665 | 2.78180735 |
| 6 | -1.04205965 | -1.89057909 | 2.56655644 |
| 1 | -4.45192909 | 0.22990175 | 4.94207732 |
| 6 | -2.66686340 | 2.83223504 | 1.42684225 |
| 6 | 1.96066063 | -2.12261459 | 0.72314308 |
| 6 | 3.32522988 | -1.81860341 | 1.01273655 |
| 6 | 3.96898531 | -2.60784587 | 1.97594624 |
| 6 | 3.33345676 | -3.65679302 | 2.64753695 |
| 6 | 2.00640623 | -3.94656820 | 2.35188082 |
| 6 | 1.31942068 | -3.19757280 | 1.38923369 |
| 6 | -0.08904510 | -3.58038381 | 1.02431837 |
| 1 | 3.87560598 | -4.23718717 | 3.39410807 |
| 6 | 4.06602523 | -0.67273902 | 0.29674414 |
| 6 | -3.63219964 | 3.91439069 | 1.94602534 |
| 6 | -3.05104288 | 2.54209162 | -0.04310402 |
| 6 | -1.23709254 | 3.42006566 | 1.49315865 |
| 6 | 5.52789388 | -0.54839926 | 0.76611446 |
| 6 | 3.37349151 | 0.67960051 | 0.58832678 |
| 6 | 4.09408558 | -0.93599870 | -1.22771386 |
| 1 | -2.69005138 | -3.82899667 | 1.62585461 |
| 1 | -3.20196401 | -2.23189319 | 0.99836418 |
| 1 | -1.23976896 | -2.69111770 | 3.30030639 |
| 1 | -0.00266017 | -1.56023304 | 2.71173293 |
| 1 | -4.28606927 | 2.26618540 | 3.57325617 |
| 1 | -2.96222700 | -1.71675854 | 4.44832029 |
| 1 | -0.15390042 | -3.88527597 | -0.03284054 |
| 1 | -0.40859539 | -4.43652130 | 1.64306233 |
| 1 | 5.00813488 | -2.39591904 | 2.22126761 |

| | | | |
|---|---|---|---|
| 1 | 1.49117145 | -4.76583499 | 2.85872797 |
| 1 | -3.52653614 | 4.81517591 | 1.32445787 |
| 1 | -4.68333132 | 3.59573326 | 1.89091334 |
| 1 | -3.40964769 | 4.20146650 | 2.98371684 |
| 1 | -2.37205642 | 1.80586596 | -0.48609285 |
| 1 | -4.07954835 | 2.15699294 | -0.10687153 |
| 1 | -2.99853101 | 3.46957677 | -0.63430041 |
| 1 | -0.97180470 | 3.66859224 | 2.53097394 |
| 1 | -0.49293494 | 2.71727240 | 1.10352281 |
| 1 | -1.18742048 | 4.34788997 | 0.90236643 |
| 1 | 6.00683439 | 0.27607946 | 0.21915117 |
| 1 | 6.10788915 | -1.46102216 | 0.56477975 |
| 1 | 5.60046145 | -0.31890274 | 1.83921055 |
| 1 | 2.33513360 | 0.68744956 | 0.24286391 |
| 1 | 3.38504739 | 0.89433971 | 1.66704085 |
| 1 | 3.90757685 | 1.49085364 | 0.07208687 |
| 1 | 4.63462037 | -1.86891851 | -1.44782450 |
| 1 | 3.08087429 | -1.01133828 | -1.63610998 |
| 1 | 4.61091494 | -0.11058491 | -1.73899093 |
| 1 | -2.47582967 | -3.41832736 | -0.11590911 |
| 31 | -0.52118078 | -0.96172645 | -0.14016216 |
| 6 | 1.79208104 | 2.00789983 | -3.40938396 |
| 6 | 0.33295035 | 2.18074349 | -3.89551333 |
| 8 | -0.47403417 | 0.94397812 | -3.87265372 |
| 6 | -0.60544163 | 0.28181162 | -2.71827050 |
| 6 | -1.55692349 | -0.91276945 | -2.78028290 |
| 8 | -0.00687443 | 0.58057531 | -1.68414136 |
| 6 | -2.96544560 | -0.46640048 | -3.21165066 |
| 6 | -0.37511483 | 3.31491263 | -3.17574500 |
| 1 | 0.37395542 | 2.39211052 | -4.97536323 |
| 1 | -1.14784735 | -1.57786843 | -3.57188409 |
| 1 | -3.61236270 | -1.35157466 | -3.24275209 |
| 1 | -2.95292271 | 0.00077122 | -4.20527001 |
| 1 | -3.38094932 | 0.24036757 | -2.48087607 |
| 1 | 0.15307046 | 4.25394158 | -3.37944907 |
| 1 | -0.37620107 | 3.15788557 | -2.09228035 |
| 1 | -1.40526303 | 3.40110941 | -3.54500629 |

Electronic energy = -3673.048475 a.u.
DFT-D3(BJ) dispersion correction = -0.117669 a.u.
Thermal free energy = 0.583280 a.u.
Gibbs free energy = -3672.582864 a.u.
Number of imaginary frequencies = 0.

**Int2 LA isomer/conformer 29**

| Atomic N. | X | Y | Z |
|---|---|---|---|
| 8 | -0.89783602 | 1.53099759 | -6.32202770 |
| 6 | -1.20440929 | 0.12464292 | -6.48018609 |
| 1 | -0.37230538 | -0.51895149 | -6.17399102 |
| 8 | 0.05461001 | 3.23048246 | -5.26987623 |
| 8 | -1.94788237 | -1.54458033 | -0.77607765 |
| 1 | -1.38806607 | -0.00061680 | -7.55240422 |
| 1 | -2.10534456 | -0.13807029 | -5.91447665 |

| | | | |
|---|---|---|---|
| 7 | -1.15899986 | -1.95945180 | 2.00944510 |
| 8 | -0.67874099 | 0.82911306 | 1.12760720 |
| 8 | 1.10656930 | -1.61323644 | 0.10376810 |
| 6 | -2.60141342 | -2.30625372 | 2.03508935 |
| 6 | -1.39169721 | 1.12412495 | 2.23917373 |
| 6 | -1.98712232 | 2.41486342 | 2.37215428 |
| 6 | -2.71213643 | 2.67335878 | 3.54402918 |
| 6 | -2.86386615 | 1.73121081 | 4.56527241 |
| 6 | -2.26242222 | 0.48442189 | 4.43248694 |
| 6 | -1.51860765 | 0.17732664 | 3.28798380 |
| 6 | -0.78659001 | -1.13424193 | 3.20810682 |
| 1 | -3.44172300 | 1.97732329 | 5.45610521 |
| 6 | -1.83473527 | 3.49120314 | 1.27882154 |
| 6 | 1.80662541 | -2.25417244 | 1.06420779 |
| 6 | 3.23127125 | -2.16310132 | 1.08312958 |
| 6 | 3.90895717 | -2.85741311 | 2.09467863 |
| 6 | 3.24970495 | -3.61821763 | 3.06479804 |
| 6 | 1.86265203 | -3.70672765 | 3.03043272 |
| 6 | 1.13731828 | -3.04331050 | 2.03402539 |
| 6 | -0.35408349 | -3.23156496 | 1.95953618 |
| 1 | 3.82022573 | -4.13450482 | 3.83673333 |
| 6 | 3.99876223 | -1.33360804 | 0.03479065 |
| 6 | -2.53942961 | 4.80670976 | 1.65992611 |
| 6 | -2.46524266 | 2.99965713 | -0.04549373 |
| 6 | -0.33794682 | 3.81673439 | 1.05924265 |
| 6 | 5.52266454 | -1.39980661 | 0.24861773 |
| 6 | 3.58601312 | 0.15480175 | 0.12470353 |
| 6 | 3.71033860 | -1.87997879 | -1.38394747 |
| 1 | -2.82665113 | -2.90228529 | 2.93550565 |
| 1 | -3.20028931 | -1.38986135 | 2.05268443 |
| 1 | -0.97811803 | -1.72902007 | 4.11796698 |
| 1 | 0.29946589 | -0.97106296 | 3.14225261 |
| 1 | -3.18245660 | 3.64694218 | 3.66964570 |
| 1 | -2.35078249 | -0.26101789 | 5.22624221 |
| 1 | -0.64070948 | -3.72279399 | 1.01546911 |
| 1 | -0.68330964 | -3.87921702 | 2.79023913 |
| 1 | 4.99522742 | -2.80141788 | 2.13704726 |
| 1 | 1.32707934 | -4.30332453 | 3.77251683 |
| 1 | -2.39185374 | 5.53765506 | 0.85214813 |
| 1 | -3.62312976 | 4.67452922 | 1.79250590 |
| 1 | -2.12703229 | 5.24387521 | 2.58061355 |
| 1 | -1.97297169 | 2.08703422 | -0.39759677 |
| 1 | -3.53797471 | 2.79517188 | 0.08725762 |
| 1 | -2.35994700 | 3.77480029 | -0.81988614 |
| 1 | 0.11078069 | 4.20678648 | 1.98436312 |
| 1 | 0.22398938 | 2.93020514 | 0.74614819 |
| 1 | -0.23543454 | 4.58967093 | 0.28221452 |
| 1 | 6.01904775 | -0.80114721 | -0.52857022 |
| 1 | 5.90781515 | -2.42691350 | 0.17168996 |
| 1 | 5.82187006 | -0.98780022 | 1.22311918 |
| 1 | 2.51720701 | 0.29237133 | -0.06915901 |
| 1 | 3.81244778 | 0.55886668 | 1.12193446 |
| 1 | 4.15271915 | 0.74342056 | -0.61329881 |

| | | | |
|---|---|---|---|
| 1 | 4.05468981 | -2.92059953 | -1.47518153 |
| 1 | 2.63882981 | -1.84781243 | -1.61100179 |
| 1 | 4.25159956 | -1.27936754 | -2.13172651 |
| 1 | -2.84806508 | -2.87559658 | 1.13335518 |
| 31 | -0.59470552 | -0.85458137 | 0.31659375 |
| 6 | -0.24086375 | 2.05424554 | -5.25541302 |
| 6 | 0.20787420 | 1.20313003 | -4.05510059 |
| 8 | -0.82479331 | 0.20655714 | -3.71744614 |
| 6 | -0.89433710 | -0.17251076 | -2.43694275 |
| 6 | -2.01255209 | -1.16224567 | -2.11608063 |
| 8 | -0.12290176 | 0.23402816 | -1.56498373 |
| 6 | -3.38642108 | -0.56005525 | -2.46225708 |
| 6 | 1.56586374 | 0.54698754 | -4.29277465 |
| 1 | 0.27567369 | 1.90231579 | -3.21412777 |
| 1 | -1.83671092 | -2.03716441 | -2.77819722 |
| 1 | -4.15845140 | -1.29976919 | -2.21769570 |
| 1 | -3.46183841 | -0.30811569 | -3.52858644 |
| 1 | -3.56780927 | 0.34198638 | -1.86283842 |
| 1 | 2.29101990 | 1.32754528 | -4.55803920 |
| 1 | 1.53216999 | -0.18871236 | -5.10610028 |
| 1 | 1.90962793 | 0.05510431 | -3.37489433 |

Electronic energy = -3673.049707 a.u.
DFT-D3(BJ) dispersion correction = -0.114952 a.u.
Thermal free energy = 0.582114 a.u.
Gibbs free energy = -3672.582545 a.u.
Number of imaginary frequencies = 0.

**Int2 LA isomer/conformer 30**

| Atomic N. | X | Y | Z |
|---|---|---|---|
| 8 | 4.39250773 | -4.74022777 | 1.48494230 |
| 6 | 5.69830721 | -4.20884144 | 1.83841513 |
| 1 | 6.39675687 | -4.33397884 | 1.00239253 |
| 8 | 4.28296579 | -3.21815434 | -0.20900304 |
| 8 | 0.99884084 | -0.90270888 | 0.82824805 |
| 1 | 6.01910911 | -4.79138165 | 2.70637399 |
| 1 | 5.61666938 | -3.14431149 | 2.08825169 |
| 7 | 0.74353548 | 0.91040467 | -2.11533686 |
| 8 | -0.16301495 | 1.69961449 | 0.59619882 |
| 8 | -1.65410472 | -0.49859366 | -0.90279164 |
| 6 | 2.17228723 | 0.60818103 | -2.39205929 |
| 6 | 0.75167710 | 2.68675720 | 0.46417702 |
| 6 | 1.23574734 | 3.39703135 | 1.60190276 |
| 6 | 2.18970458 | 4.39940002 | 1.37164750 |
| 6 | 2.66157151 | 4.72431441 | 0.09580558 |
| 6 | 2.15506758 | 4.04818896 | -1.00983423 |
| 6 | 1.19585446 | 3.04587883 | -0.83510616 |
| 6 | 0.53357442 | 2.40365172 | -2.02007989 |
| 1 | 3.40826542 | 5.50887487 | -0.02717475 |
| 6 | 0.73072343 | 3.08404538 | 3.02350763 |
| 6 | -2.31230628 | 0.11273374 | -1.91923067 |
| 6 | -3.73013187 | 0.25757085 | -1.85389919 |
| 6 | -4.36527274 | 0.88976536 | -2.93185221 |

| | | | |
|---|---|---|---|
| 6 | -3.66944555 | 1.37584879 | -4.04180629 |
| 6 | -2.28931878 | 1.22091125 | -4.09747704 |
| 6 | -1.60291580 | 0.58272613 | -3.05698068 |
| 6 | -0.12233786 | 0.33434435 | -3.21508276 |
| 1 | -4.20596765 | 1.86913775 | -4.85222009 |
| 6 | -4.54053332 | -0.25744260 | -0.64738939 |
| 6 | 1.34670455 | 4.02697735 | 4.07421927 |
| 6 | 1.11216360 | 1.63788040 | 3.41743742 |
| 6 | -0.80503344 | 3.26194772 | 3.09010477 |
| 6 | -6.05081715 | -0.00686554 | -0.81880076 |
| 6 | -4.09982807 | 0.47133489 | 0.64420432 |
| 6 | -4.34764702 | -1.78477190 | -0.49021093 |
| 1 | 2.46877709 | 1.07385718 | -3.34589957 |
| 1 | 2.79254612 | 1.01537597 | -1.58605188 |
| 1 | 0.89858222 | 2.85355417 | -2.95859963 |
| 1 | -0.55528175 | 2.54886647 | -1.97290876 |
| 1 | 2.58772573 | 4.95234086 | 2.22089300 |
| 1 | 2.48367663 | 4.30738835 | -2.01916296 |
| 1 | 0.09618541 | -0.74124730 | -3.23095431 |
| 1 | 0.21957894 | 0.77026291 | -4.16852417 |
| 1 | -5.44572344 | 1.01848862 | -2.90626873 |
| 1 | -1.72712083 | 1.58338308 | -4.96116566 |
| 1 | 0.93995612 | 3.77272282 | 5.06356056 |
| 1 | 2.44041341 | 3.92687596 | 4.13186960 |
| 1 | 1.10362612 | 5.08125295 | 3.87685731 |
| 1 | 0.68671031 | 0.90048805 | 2.72849502 |
| 1 | 2.20530874 | 1.51463416 | 3.41902286 |
| 1 | 0.74541515 | 1.42196708 | 4.43319212 |
| 1 | -1.08511117 | 4.29857487 | 2.85153734 |
| 1 | -1.31749918 | 2.59288407 | 2.39047626 |
| 1 | -1.15991946 | 3.04218357 | 4.10879307 |
| 1 | -6.57908505 | -0.40308964 | 0.06013612 |
| 1 | -6.45752397 | -0.51437904 | -1.70557515 |
| 1 | -6.28794104 | 1.06444177 | -0.89054352 |
| 1 | -3.04172130 | 0.30059694 | 0.86750045 |
| 1 | -4.26102604 | 1.55496298 | 0.55114660 |
| 1 | -4.69759954 | 0.11180399 | 1.49582604 |
| 1 | -4.71203086 | -2.31428711 | -1.38265493 |
| 1 | -3.29339971 | -2.04159591 | -0.34201721 |
| 1 | -4.92284340 | -2.14582903 | 0.37608762 |
| 1 | 2.31021343 | -0.47558003 | -2.45222712 |
| 31 | 0.02432265 | 0.09740563 | -0.36854277 |
| 6 | 3.80560648 | -4.13692038 | 0.42159203 |
| 6 | 2.44780865 | -4.80291108 | 0.14239073 |
| 8 | 1.74018171 | -4.06110339 | -0.87376734 |
| 6 | 1.25315384 | -2.81536861 | -0.62846072 |
| 6 | 1.06872651 | -2.29874158 | 0.80418093 |
| 8 | 0.88228958 | -2.15166013 | -1.58254027 |
| 6 | -0.20033222 | -2.94844877 | 1.39860342 |
| 6 | 2.62024098 | -6.21936099 | -0.40107472 |
| 1 | 1.87591927 | -4.83281043 | 1.08127899 |
| 1 | 1.94206406 | -2.58837649 | 1.41456988 |
| 1 | -0.34409128 | -2.55611785 | 2.41293493 |

| | | | |
|---|---|---|---|
| 1 | -0.13453269 | -4.04529599 | 1.45029527 |
| 1 | -1.07446766 | -2.68013482 | 0.78993455 |
| 1 | 3.15612202 | -6.83883609 | 0.32767061 |
| 1 | 3.18362416 | -6.19947793 | -1.34260919 |
| 1 | 1.63511683 | -6.66298324 | -0.59078960 |

Electronic energy = -3673.049081 a.u.
DFT-D3(BJ) dispersion correction = -0.114388 a.u.
Thermal free energy = 0.581322 a.u.
Gibbs free energy = -3672.582147 a.u.
Number of imaginary frequencies = 0.

**Int2 LA isomer/conformer 31**

| Atomic N. | X | Y | Z |
|---|---|---|---|
| 8 | -3.75958030 | -4.19323791 | -2.76653012 |
| 6 | -2.78711612 | -3.96056057 | -1.72146259 |
| 1 | -1.88938569 | -3.48684221 | -2.13556775 |
| 8 | -5.37672500 | -3.47910210 | -4.10458542 |
| 8 | -0.64106604 | 1.02704404 | -1.78234871 |
| 1 | -2.53374268 | -4.95469760 | -1.34017530 |
| 1 | -3.19159067 | -3.35180593 | -0.90275968 |
| 7 | -1.25597475 | 1.48499877 | 1.42061734 |
| 8 | 1.48577431 | 1.50362159 | 0.26662217 |
| 8 | -0.10150512 | -1.13999542 | 0.45106223 |
| 6 | -2.28355695 | 2.43259117 | 0.91595602 |
| 6 | 1.47958922 | 2.80183789 | 0.66636455 |
| 6 | 2.40303103 | 3.73454017 | 0.11298667 |
| 6 | 2.33411108 | 5.05538214 | 0.58058215 |
| 6 | 1.41622435 | 5.47284955 | 1.54898182 |
| 6 | 0.53597749 | 4.54615406 | 2.09807533 |
| 6 | 0.57008445 | 3.21333260 | 1.67480660 |
| 6 | -0.29014151 | 2.18104525 | 2.34990432 |
| 1 | 1.40009255 | 6.51317370 | 1.87374177 |
| 6 | 3.43495600 | 3.31590764 | -0.95225322 |
| 6 | -0.14070461 | -1.45208393 | 1.77545558 |
| 6 | 0.65260765 | -2.52537952 | 2.27582003 |
| 6 | 0.56214465 | -2.80743160 | 3.64660325 |
| 6 | -0.25815214 | -2.08470782 | 4.51645169 |
| 6 | -1.03317800 | -1.04613356 | 4.01340557 |
| 6 | -0.99468511 | -0.72973250 | 2.65005080 |
| 6 | -1.93236167 | 0.32711926 | 2.12050527 |
| 1 | -0.28756514 | -2.33578025 | 5.57657961 |
| 6 | 1.57720737 | -3.34832347 | 1.35628902 |
| 6 | 4.35299564 | 4.48551187 | -1.35461129 |
| 6 | 2.71158316 | 2.83524819 | -2.23234779 |
| 6 | 4.34077067 | 2.18942798 | -0.40002359 |
| 6 | 2.31271414 | -4.46273154 | 2.12466718 |
| 6 | 2.65700916 | -2.43678086 | 0.72711193 |
| 6 | 0.74518488 | -4.03318236 | 0.24615245 |
| 1 | -2.88189596 | 2.81350773 | 1.75927602 |
| 1 | -1.78909889 | 3.27241425 | 0.41581524 |
| 1 | -0.87434191 | 2.63968618 | 3.16496978 |
| 1 | 0.33259141 | 1.38489869 | 2.78326537 |

| | | | |
|---|---|---|---|
| 1 | 3.02010229 | 5.79507246 | 0.17168835 |
| 1 | -0.17204011 | 4.84454798 | 2.87472136 |
| 1 | -2.62207285 | -0.09825161 | 1.37810089 |
| 1 | -2.52848671 | 0.73935220 | 2.95140801 |
| 1 | 1.16033343 | -3.61772565 | 4.05892862 |
| 1 | -1.68995408 | -0.47585720 | 4.67411424 |
| 1 | 5.07637697 | 4.12992437 | -2.10211302 |
| 1 | 3.79316350 | 5.31656634 | -1.80765225 |
| 1 | 4.92489649 | 4.87531415 | -0.50003248 |
| 1 | 2.06006002 | 1.97728582 | -2.03524902 |
| 1 | 2.09674352 | 3.64268099 | -2.65586177 |
| 1 | 3.45460066 | 2.54206573 | -2.98994539 |
| 1 | 4.89092302 | 2.53552153 | 0.48726682 |
| 1 | 3.75943867 | 1.30282451 | -0.12497433 |
| 1 | 5.07939775 | 1.90050739 | -1.16323279 |
| 1 | 2.94631083 | -5.02190802 | 1.42166931 |
| 1 | 1.61641544 | -5.17912690 | 2.58405866 |
| 1 | 2.96835339 | -4.06064915 | 2.91026045 |
| 1 | 2.21581029 | -1.63972663 | 0.11997866 |
| 1 | 3.27408660 | -1.97135002 | 1.50885987 |
| 1 | 3.31979229 | -3.03564045 | 0.08423501 |
| 1 | 0.01684848 | -4.73118510 | 0.68505753 |
| 1 | 0.20426398 | -3.29584941 | -0.35691966 |
| 1 | 1.40807640 | -4.60952783 | -0.41681241 |
| 1 | -2.93066279 | 1.91055733 | 0.20330766 |
| 31 | -0.13383607 | 0.64527036 | -0.06721267 |
| 6 | -4.57367224 | -3.20977968 | -3.23755155 |
| 6 | -4.46795439 | -1.80095058 | -2.62365225 |
| 8 | -3.15187690 | -1.27464887 | -3.00573771 |
| 6 | -2.52103195 | -0.49173391 | -2.08933511 |
| 6 | -1.22111750 | 0.09755441 | -2.65863035 |
| 8 | -2.94840888 | -0.27962752 | -0.96672685 |
| 6 | -0.24539480 | -1.00664616 | -3.09849313 |
| 6 | -5.55752322 | -0.87936511 | -3.13878032 |
| 1 | -4.50083179 | -1.85858151 | -1.52594359 |
| 1 | -1.53319794 | 0.65587696 | -3.56218717 |
| 1 | 0.64472297 | -0.53158350 | -3.52941219 |
| 1 | -0.69816358 | -1.66195235 | -3.85399370 |
| 1 | 0.06634639 | -1.61257869 | -2.23576283 |
| 1 | -6.54157628 | -1.28221587 | -2.86770034 |
| 1 | -5.51344882 | -0.80177710 | -4.23093929 |
| 1 | -5.44809340 | 0.11553275 | -2.68825759 |

Electronic energy = -3673.047435 a.u.
DFT-D3(BJ) dispersion correction = -0.115995 a.u.
Thermal free energy = 0.581742 a.u.
Gibbs free energy = -3672.581688 a.u.
Number of imaginary frequencies = 0.

**Int2 LA isomer/conformer 32**

| Atomic N. | X | Y | Z |
|---|---|---|---|
| 8 | 0.91924655 | 2.01797332 | 5.66230371 |
| 6 | 1.97079492 | 2.98026403 | 5.42773151 |

| | | | |
|---|---|---|---|
| 1 | 2.25829573 | 2.99504507 | 4.36773324 |
| 8 | -1.08544741 | 1.22273180 | 5.12213254 |
| 8 | -1.15958347 | 1.74276872 | -0.19374696 |
| 1 | 2.81498701 | 2.62938203 | 6.02993342 |
| 1 | 1.68634782 | 3.98747949 | 5.76433827 |
| 7 | 1.95178956 | 0.79296863 | -0.63129061 |
| 8 | -0.33848706 | -0.35482386 | -2.14161925 |
| 8 | 0.03267370 | -1.01694369 | 0.84838723 |
| 6 | 2.20976439 | 2.25467342 | -0.67533650 |
| 6 | 0.27906083 | 0.21305006 | -3.20775992 |
| 6 | -0.43689510 | 0.45504356 | -4.41574819 |
| 6 | 0.27342002 | 1.04169317 | -5.47353142 |
| 6 | 1.62640174 | 1.38296915 | -5.38473376 |
| 6 | 2.31961168 | 1.11919605 | -4.20773895 |
| 6 | 1.66116137 | 0.52551425 | -3.12581861 |
| 6 | 2.42971863 | 0.12401959 | -1.89713862 |
| 1 | 2.13095849 | 1.84233140 | -6.23475706 |
| 6 | -1.92694217 | 0.08883201 | -4.55817939 |
| 6 | 1.11976480 | -1.83141785 | 0.91582775 |
| 6 | 0.95065164 | -3.22045974 | 1.18699011 |
| 6 | 2.10953568 | -4.00730813 | 1.25249443 |
| 6 | 3.39250647 | -3.48654155 | 1.06382660 |
| 6 | 3.54355163 | -2.12806308 | 0.81151452 |
| 6 | 2.42217242 | -1.29202753 | 0.74861534 |
| 6 | 2.62554454 | 0.19281387 | 0.58318168 |
| 1 | 4.26222162 | -4.14137096 | 1.11644192 |
| 6 | -0.44350835 | -3.84107426 | 1.40627727 |
| 6 | -2.46336442 | 0.39626476 | -5.96908315 |
| 6 | -2.77280014 | 0.90712501 | -3.55428651 |
| 6 | -2.12764741 | -1.42528444 | -4.30988442 |
| 6 | -0.36177122 | -5.35353473 | 1.68862779 |
| 6 | -1.31777063 | -3.66067400 | 0.14280503 |
| 6 | -1.12681852 | -3.18379562 | 2.62858163 |
| 1 | 3.29478136 | 2.43774984 | -0.74023597 |
| 1 | 1.71808305 | 2.68458700 | -1.55477042 |
| 1 | 3.50119711 | 0.35461485 | -2.02042281 |
| 1 | 2.33266273 | -0.95663561 | -1.71815081 |
| 1 | -0.24734797 | 1.24756699 | -6.40705979 |
| 1 | 3.38362591 | 1.35316165 | -4.12511812 |
| 1 | 2.21485108 | 0.74161545 | 1.44280609 |
| 1 | 3.70413140 | 0.41125366 | 0.51192865 |
| 1 | 2.01219536 | -5.07272536 | 1.45206659 |
| 1 | 4.53800958 | -1.69755054 | 0.67332875 |
| 1 | -3.52125213 | 0.10148223 | -6.01982145 |
| 1 | -2.40838575 | 1.46777896 | -6.21083909 |
| 1 | -1.92584917 | -0.16489361 | -6.74728702 |
| 1 | -2.47207457 | 0.71907599 | -2.51816211 |
| 1 | -2.67076678 | 1.98435141 | -3.75076022 |
| 1 | -3.83580882 | 0.64190668 | -3.66261071 |
| 1 | -1.56216965 | -2.01609170 | -5.04540321 |
| 1 | -1.80175514 | -1.71584270 | -3.30543207 |
| 1 | -3.19275577 | -1.68051454 | -4.41945857 |
| 1 | -1.37841448 | -5.74097704 | 1.84575143 |

| | | | |
|---|---|---|---|
| 1 | 0.21921281 | -5.57490297 | 2.59554270 |
| 1 | 0.07714670 | -5.90978582 | 0.84760829 |
| 1 | -1.47827764 | -2.60432256 | -0.09546054 |
| 1 | -0.84806236 | -4.14258948 | -0.72682208 |
| 1 | -2.29944234 | -4.13199439 | 0.30222623 |
| 1 | -0.54445070 | -3.36664744 | 3.54304802 |
| 1 | -1.23269662 | -2.10146641 | 2.50154474 |
| 1 | -2.12795173 | -3.61743867 | 2.77347031 |
| 1 | 1.80559404 | 2.71515874 | 0.23229975 |
| 31 | -0.03213338 | 0.32426977 | -0.43129518 |
| 6 | -0.27805262 | 2.11676404 | 5.01690516 |
| 6 | -0.59583603 | 3.42181055 | 4.25871742 |
| 8 | -1.43798629 | 3.11533927 | 3.11737715 |
| 6 | -0.82811614 | 2.42253469 | 2.12014252 |
| 6 | -1.81569875 | 2.06446112 | 1.00594828 |
| 8 | 0.35799065 | 2.12980940 | 2.13566950 |
| 6 | -2.77077304 | 0.96443294 | 1.50181166 |
| 6 | -1.39503585 | 4.36175583 | 5.15482964 |
| 1 | 0.31536103 | 3.90301191 | 3.88442194 |
| 1 | -2.40843250 | 2.97764768 | 0.81517935 |
| 1 | -3.48591989 | 0.73797191 | 0.70062313 |
| 1 | -3.32056612 | 1.28983400 | 2.39422416 |
| 1 | -2.21132879 | 0.05210505 | 1.75034244 |
| 1 | -0.80313716 | 4.64740311 | 6.03516660 |
| 1 | -2.31122916 | 3.86468374 | 5.49661556 |
| 1 | -1.66228086 | 5.27142046 | 4.60242343 |

Electronic energy = -3673.047405 a.u.
DFT-D3(BJ) dispersion correction = -0.114638 a.u.
Thermal free energy = 0.581585 a.u.
Gibbs free energy = -3672.580458 a.u.
Number of imaginary frequencies = 0.

**Int2 LA isomer/conformer 33**

| Atomic N. | X | Y | Z |
|---|---|---|---|
| 8 | 2.61434440 | 4.61416736 | 1.04050417 |
| 6 | 2.62401954 | 5.61031714 | -0.01780367 |
| 1 | 1.59668539 | 5.85835275 | -0.30982028 |
| 8 | 1.49173394 | 6.09905083 | 2.35535929 |
| 8 | -0.08504154 | 1.60544385 | 0.43380547 |
| 1 | 3.16329403 | 5.14182584 | -0.84545504 |
| 1 | 3.13593513 | 6.51805246 | 0.32270092 |
| 7 | 1.90176517 | -0.74502816 | -1.15894251 |
| 8 | -1.03901955 | -0.41604560 | -1.36840528 |
| 8 | 0.18891896 | -1.46087218 | 1.23531294 |
| 6 | 2.82406213 | 0.34625837 | -1.56572336 |
| 6 | -0.77555809 | -0.15709063 | -2.66978523 |
| 6 | -1.73052545 | 0.50827844 | -3.49336957 |
| 6 | -1.36986404 | 0.74239269 | -4.82867260 |
| 6 | -0.14163548 | 0.34595304 | -5.36753081 |
| 6 | 0.76736918 | -0.33367626 | -4.56255524 |
| 6 | 0.45275147 | -0.59946987 | -3.22615676 |
| 6 | 1.35682669 | -1.44917929 | -2.37994038 |

| | | | |
|---|---|---|---|
| 1 | 0.09042242 | 0.55661427 | -6.41154852 |
| 6 | -3.10248583 | 0.94594394 | -2.94591662 |
| 6 | 0.63571483 | -2.71406753 | 0.96473448 |
| 6 | -0.06495717 | -3.84223258 | 1.48514535 |
| 6 | 0.44511532 | -5.11298003 | 1.18416846 |
| 6 | 1.58875965 | -5.30559178 | 0.40482723 |
| 6 | 2.26610036 | -4.19764972 | -0.09066802 |
| 6 | 1.81145194 | -2.90356530 | 0.19099308 |
| 6 | 2.63668415 | -1.72870126 | -0.27305536 |
| 1 | 1.94224311 | -6.31405981 | 0.19044042 |
| 6 | -1.33470331 | -3.68119259 | 2.34509172 |
| 6 | -3.98260809 | 1.58193141 | -4.03828901 |
| 6 | -2.91901259 | 1.99789245 | -1.82700697 |
| 6 | -3.86741460 | -0.28193104 | -2.39607052 |
| 6 | -1.89998075 | -5.04077940 | 2.79875453 |
| 6 | -2.44570927 | -2.97346395 | 1.53397983 |
| 6 | -1.00979162 | -2.87318249 | 3.62362579 |
| 1 | 3.66550820 | -0.07703657 | -2.13805820 |
| 1 | 2.28407041 | 1.05937960 | -2.19858766 |
| 1 | 2.21575433 | -1.80872577 | -2.97098853 |
| 1 | 0.81267925 | -2.32604646 | -2.00085078 |
| 1 | -2.07279629 | 1.25810523 | -5.48080004 |
| 1 | 1.71880647 | -0.68329567 | -4.97054513 |
| 1 | 3.00120639 | -1.14180763 | 0.58025277 |
| 1 | 3.50898986 | -2.09655574 | -0.83844976 |
| 1 | -0.07161892 | -5.99237072 | 1.56386370 |
| 1 | 3.16797626 | -4.32522584 | -0.69373176 |
| 1 | -4.95196077 | 1.85804371 | -3.59919972 |
| 1 | -3.53523435 | 2.49796632 | -4.45097762 |
| 1 | -4.18008269 | 0.88684940 | -4.86734700 |
| 1 | -2.32308478 | 1.61026882 | -0.99400074 |
| 1 | -2.41757434 | 2.89488161 | -2.21964683 |
| 1 | -3.90394280 | 2.30328027 | -1.44077810 |
| 1 | -4.03962079 | -1.01956915 | -3.19363989 |
| 1 | -3.31600339 | -0.76863133 | -1.58437901 |
| 1 | -4.84906480 | 0.03475875 | -2.01155437 |
| 1 | -2.79471918 | -4.86690702 | 3.41338851 |
| 1 | -1.18224830 | -5.60477700 | 3.41190298 |
| 1 | -2.20149979 | -5.66881770 | 1.94806553 |
| 1 | -2.14306654 | -1.97121395 | 1.21397191 |
| 1 | -2.70237509 | -3.55722544 | 0.63829335 |
| 1 | -3.35332673 | -2.88198724 | 2.14999385 |
| 1 | -0.26420772 | -3.40122632 | 4.23589481 |
| 1 | -0.61679158 | -1.88010176 | 3.38124932 |
| 1 | -1.92064157 | -2.75154139 | 4.22955855 |
| 1 | 3.20241259 | 0.85304510 | -0.67279071 |
| 31 | 0.27464669 | -0.12869023 | -0.05870813 |
| 6 | 1.99544647 | 5.00767566 | 2.17406228 |
| 6 | 1.99335839 | 3.92846755 | 3.26562592 |
| 8 | 2.48470322 | 2.64044183 | 2.81569638 |
| 6 | 1.76612301 | 1.86140905 | 1.96599265 |
| 6 | 0.29048994 | 2.15704324 | 1.66540859 |
| 8 | 2.31582989 | 0.87986069 | 1.49234445 |

| | | | |
|---|---|---|---|
| 6 | -0.57003619 | 1.62239372 | 2.82974758 |
| 6 | 2.89260201 | 4.35011238 | 4.42526378 |
| 1 | 0.95527286 | 3.84733555 | 3.61798535 |
| 1 | 0.14357542 | 3.24815495 | 1.58338917 |
| 1 | -1.62434430 | 1.81443680 | 2.59488830 |
| 1 | -0.33105549 | 2.10187289 | 3.79033614 |
| 1 | -0.42233769 | 0.53907258 | 2.93488618 |
| 1 | 2.58094643 | 5.33534045 | 4.79161547 |
| 1 | 3.93921282 | 4.39974807 | 4.09884224 |
| 1 | 2.81213277 | 3.62009333 | 5.23978626 |

Electronic energy = -3673.048742 a.u.
DFT-D3(BJ) dispersion correction = -0.114745 a.u.
Thermal free energy = 0.583409 a.u.
Gibbs free energy = -3672.580079 a.u.
Number of imaginary frequencies = 0.

**Int2 LA isomer/conformer 34**

| Atomic N. | X | Y | Z |
|---|---|---|---|
| 7 | 0.38993589 | 0.34968808 | -2.11601482 |
| 8 | 0.41389721 | 0.82257035 | 0.84403782 |
| 8 | -2.26470889 | 0.49048482 | -0.71585371 |
| 6 | 1.29255183 | -0.68455042 | -2.69082693 |
| 6 | 1.73539910 | 1.04823358 | 0.62750488 |
| 6 | 2.66276427 | 0.98013158 | 1.70719664 |
| 6 | 4.00932917 | 1.23812779 | 1.40962728 |
| 6 | 4.45712059 | 1.55346208 | 0.12304813 |
| 6 | 3.53763891 | 1.63347487 | -0.91803877 |
| 6 | 2.18085854 | 1.39574770 | -0.67399256 |
| 6 | 1.16914009 | 1.59691002 | -1.76893530 |
| 1 | 5.51521929 | 1.74568329 | -0.05543413 |
| 6 | 2.21165985 | 0.64233829 | 3.14168883 |
| 6 | -2.43678204 | 1.58949857 | -1.49859004 |
| 6 | -3.41467482 | 2.56495076 | -1.15018166 |
| 6 | -3.55553898 | 3.66442030 | -2.00947429 |
| 6 | -2.78534537 | 3.82904297 | -3.16407837 |
| 6 | -1.84054378 | 2.86480017 | -3.49539738 |
| 6 | -1.66693946 | 1.74011442 | -2.67990661 |
| 6 | -0.70834717 | 0.66085974 | -3.11053396 |
| 1 | -2.92859599 | 4.70495873 | -3.79656195 |
| 6 | -4.28387793 | 2.42577932 | 0.11549364 |
| 6 | 3.38722338 | 0.67552084 | 4.13650852 |
| 6 | 1.60892398 | -0.78192654 | 3.18860002 |
| 6 | 1.16835168 | 1.67675778 | 3.62745104 |
| 6 | -5.27794629 | 3.59356576 | 0.26069352 |
| 6 | -3.39098446 | 2.42289837 | 1.37889051 |
| 6 | -5.11333086 | 1.12128963 | 0.04933458 |
| 1 | 1.81655936 | -0.26534920 | -3.56468484 |
| 1 | 2.02705804 | -0.99475711 | -1.94091375 |
| 1 | 1.66378289 | 1.95232559 | -2.68795051 |
| 1 | 0.42300742 | 2.34882223 | -1.47324641 |
| 1 | 4.74394152 | 1.19076240 | 2.21154452 |
| 1 | 3.86165499 | 1.90261287 | -1.92601534 |

| | | | |
|---|---|---|---|
| 1 | -1.23240054 | -0.29373936 | -3.27594608 |
| 1 | -0.22896867 | 0.94880395 | -4.06047354 |
| 1 | -4.29062689 | 4.43014309 | -1.76915097 |
| 1 | -1.23612246 | 2.96833134 | -4.39919047 |
| 1 | 3.00982003 | 0.44017913 | 5.14166344 |
| 1 | 4.15900969 | -0.06900995 | 3.89277085 |
| 1 | 3.86077813 | 1.66682059 | 4.18493937 |
| 1 | 0.74209603 | -0.88215044 | 2.52646675 |
| 1 | 2.36166600 | -1.52985961 | 2.89752817 |
| 1 | 1.28781359 | -1.01395046 | 4.21549405 |
| 1 | 1.60066831 | 2.68804120 | 3.63346411 |
| 1 | 0.27767086 | 1.68286163 | 2.99020666 |
| 1 | 0.85995599 | 1.43579345 | 4.65605727 |
| 1 | -5.87665542 | 3.44046944 | 1.16970429 |
| 1 | -5.97492482 | 3.65233157 | -0.58777910 |
| 1 | -4.76808418 | 4.56258046 | 0.36148335 |
| 1 | -2.69108242 | 1.58079990 | 1.37973321 |
| 1 | -2.81169613 | 3.35478531 | 1.44841507 |
| 1 | -4.02100566 | 2.34771983 | 2.27801705 |
| 1 | -5.78250974 | 1.13132453 | -0.82335556 |
| 1 | -4.46914921 | 0.23793394 | -0.01622601 |
| 1 | -5.73692747 | 1.03225249 | 0.95181374 |
| 1 | 0.70502309 | -1.55529385 | -3.00248481 |
| 8 | 3.14983088 | -2.95282558 | -0.55661296 |
| 6 | 4.51532055 | -2.48011771 | -0.40495945 |
| 1 | 4.65844896 | -2.05550699 | 0.59561703 |
| 8 | 3.54437256 | -4.39002329 | 1.15915633 |
| 8 | -0.53482639 | -1.89451287 | 0.25263145 |
| 1 | 4.64051403 | -1.70959912 | -1.17099555 |
| 1 | 5.22083453 | -3.30568357 | -0.55732312 |
| 31 | -0.56839928 | -0.17592831 | -0.37496338 |
| 8 | 0.87277586 | -4.12105725 | -1.16676157 |
| 6 | -0.33593018 | -3.55404953 | -1.45075612 |
| 6 | -1.20479735 | -3.03548330 | -0.28798704 |
| 6 | 2.80095467 | -3.92429895 | 0.31667228 |
| 6 | 1.32421961 | -4.32684282 | 0.19957811 |
| 8 | -0.63535512 | -3.40832901 | -2.62254519 |
| 6 | 1.10677329 | -5.77701008 | 0.60907875 |
| 6 | -2.63985644 | -2.79448463 | -0.73135025 |
| 1 | -1.22094049 | -3.78998600 | 0.51926503 |
| 1 | 0.79724444 | -3.63397807 | 0.87344124 |
| 1 | 0.03364803 | -6.00745256 | 0.63376443 |
| 1 | 1.53286729 | -5.94947332 | 1.60415406 |
| 1 | 1.59592467 | -6.45378291 | -0.10305184 |
| 1 | -3.10259043 | -3.73843178 | -1.05165145 |
| 1 | -2.68847035 | -2.09185646 | -1.57173913 |
| 1 | -3.21531346 | -2.38570368 | 0.10919892 |

Electronic energy = -3673.044328 a.u.
DFT-D3(BJ) dispersion correction = -0.117876 a.u.
Thermal free energy = 0.582443 a.u.
Gibbs free energy = -3672.579761 a.u.
Number of imaginary frequencies = 0.

**Int2_LA isomer/conformer 35**

| Atomic N. | X | Y | Z |
|---|---|---|---|
| 8 | 0.78278727 | -5.82846051 | -1.07824822 |
| 6 | -0.28114351 | -6.75277120 | -1.39497905 |
| 1 | -0.69720155 | -7.20435773 | -0.48349976 |
| 8 | 1.53634907 | -4.15360843 | 0.17353734 |
| 8 | -0.02274591 | -0.44684052 | 2.78943483 |
| 1 | 0.19354958 | -7.53468449 | -1.99571321 |
| 1 | -1.07738278 | -6.28276240 | -1.98681954 |
| 7 | 0.78458631 | 2.25266609 | 2.03246844 |
| 8 | -1.06082384 | 1.02792080 | 0.05931430 |
| 8 | 1.98422394 | 0.13162045 | 0.32864444 |
| 6 | 0.13584040 | 2.46402951 | 3.34926201 |
| 6 | -1.75158605 | 2.17324187 | 0.24594991 |
| 6 | -3.12756689 | 2.23730755 | -0.13326477 |
| 6 | -3.80300261 | 3.44739316 | 0.07823368 |
| 6 | -3.18615760 | 4.57228568 | 0.63255693 |
| 6 | -1.84076609 | 4.50500071 | 0.97842313 |
| 6 | -1.11611292 | 3.32524160 | 0.77881662 |
| 6 | 0.36281698 | 3.30935767 | 1.05387411 |
| 1 | -3.75390759 | 5.49043215 | 0.78335956 |
| 6 | -3.84757237 | 1.02665195 | -0.76036876 |
| 6 | 2.90295051 | 1.08276024 | 0.06483880 |
| 6 | 3.72843149 | 0.96617391 | -1.09487570 |
| 6 | 4.67412092 | 1.97655618 | -1.31650160 |
| 6 | 4.83270124 | 3.07003349 | -0.45930607 |
| 6 | 4.02957656 | 3.16739400 | 0.67124270 |
| 6 | 3.07659851 | 2.17985066 | 0.94673321 |
| 6 | 2.27944943 | 2.25947069 | 2.22017965 |
| 1 | 5.57888727 | 3.83387959 | -0.67818052 |
| 6 | 3.59381985 | -0.22623063 | -2.06231252 |
| 6 | -5.31215923 | 1.34340350 | -1.11658458 |
| 6 | -3.86243020 | -0.15605579 | 0.23620983 |
| 6 | -3.13620904 | 0.60364075 | -2.06780865 |
| 6 | 4.59670226 | -0.13695501 | -3.22818804 |
| 6 | 2.17514689 | -0.25718665 | -2.67926510 |
| 6 | 3.87391174 | -1.54983839 | -1.31207891 |
| 1 | 0.42984755 | 3.44585929 | 3.75775260 |
| 1 | -0.95245411 | 2.43432605 | 3.23368069 |
| 1 | 0.68432478 | 4.29349125 | 1.43700723 |
| 1 | 0.92780382 | 3.11502117 | 0.13007911 |
| 1 | -4.85397557 | 3.52119868 | -0.19573059 |
| 1 | -1.33173351 | 5.37760025 | 1.39444055 |
| 1 | 2.49674831 | 1.40023263 | 2.87547873 |
| 1 | 2.55324841 | 3.17840824 | 2.76684278 |
| 1 | 5.31398380 | 1.91649636 | -2.19512458 |
| 1 | 4.14166084 | 4.00779112 | 1.36034009 |
| 1 | -5.77167665 | 0.45195437 | -1.56764219 |
| 1 | -5.90700501 | 1.61011250 | -0.23098137 |
| 1 | -5.39090403 | 2.16119863 | -1.84714552 |
| 1 | -2.84303026 | -0.45996091 | 0.49619541 |
| 1 | -4.39419267 | 0.11934593 | 1.15883067 |

| | | | |
|---|---|---|---|
| 1 | -4.38567454 | -1.01582285 | -0.21169728 |
| 1 | -3.15870248 | 1.42319491 | -2.80062057 |
| 1 | -2.09152755 | 0.32999139 | -1.88438333 |
| 1 | -3.65604031 | -0.25949229 | -2.51276084 |
| 1 | 4.46149537 | -1.01111616 | -3.88110966 |
| 1 | 5.63927301 | -0.14476442 | -2.87841164 |
| 1 | 4.44186611 | 0.76236966 | -3.84221266 |
| 1 | 1.40411329 | -0.36966751 | -1.91009393 |
| 1 | 1.97592131 | 0.66678903 | -3.24189353 |
| 1 | 2.09518536 | -1.10347229 | -3.37912774 |
| 1 | 4.89332154 | -1.54955272 | -0.89854697 |
| 1 | 3.16491406 | -1.71141289 | -0.49390510 |
| 1 | 3.79445960 | -2.39930525 | -2.00668874 |
| 1 | 0.44176195 | 1.66387934 | 4.03056464 |
| 31 | 0.32780970 | 0.39377511 | 1.15669278 |
| 6 | 0.58225678 | -4.74952510 | -0.26461248 |
| 6 | -0.87302041 | -4.32909450 | 0.02382559 |
| 8 | -0.88459464 | -3.59761261 | 1.28819198 |
| 6 | -0.46762634 | -2.32775179 | 1.36804553 |
| 6 | -0.38169801 | -1.79627387 | 2.79854342 |
| 8 | -0.18475035 | -1.61444976 | 0.40505648 |
| 6 | -1.69086925 | -2.02371518 | 3.57162222 |
| 6 | -1.54423863 | -3.60289201 | -1.14691180 |
| 1 | -1.46355726 | -5.21833077 | 0.28314739 |
| 1 | 0.41726604 | -2.40590527 | 3.27366309 |
| 1 | -1.56482051 | -1.63238588 | 4.58852151 |
| 1 | -1.94309602 | -3.09047075 | 3.63074896 |
| 1 | -2.51570161 | -1.48001025 | 3.09156924 |
| 1 | -1.77845452 | -4.32461613 | -1.94037685 |
| 1 | -0.91309354 | -2.81102399 | -1.55712891 |
| 1 | -2.49050590 | -3.16097121 | -0.80881888 |

Electronic energy = -3673.045671 a.u.
DFT-D3(BJ) dispersion correction = -0.115912 a.u.
Thermal free energy = 0.582442 a.u.
Gibbs free energy = -3672.579141 a.u.
Number of imaginary frequencies = 0.

**Int2 LA isomer/conformer 36**

| Atomic N. | X | Y | Z |
|---|---|---|---|
| 8 | 1.73860107 | -5.95337087 | 0.64163467 |
| 6 | 0.97241881 | -7.18424999 | 0.55306832 |
| 1 | 1.18861306 | -7.82477689 | 1.41655026 |
| 8 | 0.68388428 | -5.49358688 | 2.60607921 |
| 8 | -1.18395138 | -1.51301125 | 1.14835812 |
| 1 | 1.29727027 | -7.65711378 | -0.37763393 |
| 1 | -0.10160647 | -6.96262389 | 0.52632115 |
| 7 | -2.33963449 | 1.15028265 | 0.91958101 |
| 8 | -0.62908388 | 0.54160608 | -1.42539868 |
| 8 | 0.58466812 | 1.07212873 | 1.47299855 |
| 6 | -3.55280911 | 0.29808473 | 0.96466319 |
| 6 | -1.70952255 | 0.96832288 | -2.11704431 |
| 6 | -1.82952215 | 0.64279517 | -3.50246362 |

| | | | |
|---|---|---|---|
| 6 | -2.97436371 | 1.09319629 | -4.17480911 |
| 6 | -3.97400256 | 1.84485700 | -3.55077279 |
| 6 | -3.82773580 | 2.18645286 | -2.21074762 |
| 6 | -2.70082253 | 1.76989275 | -1.49367009 |
| 6 | -2.49991054 | 2.25454949 | -0.08373025 |
| 1 | -4.84952042 | 2.16739856 | -4.11431882 |
| 6 | -0.73484166 | -0.15125173 | -4.24332036 |
| 6 | 0.36181040 | 2.29848851 | 1.98736289 |
| 6 | 1.45412833 | 3.20130328 | 2.16440760 |
| 6 | 1.17239329 | 4.45755713 | 2.71753809 |
| 6 | -0.11736560 | 4.84756262 | 3.09111806 |
| 6 | -1.17237706 | 3.95786929 | 2.92037403 |
| 6 | -0.94374310 | 2.68503188 | 2.38473053 |
| 6 | -2.08319838 | 1.70542980 | 2.29650152 |
| 1 | -0.28997814 | 5.83859206 | 3.51081871 |
| 6 | 2.89077002 | 2.81259403 | 1.76067436 |
| 6 | -1.07593151 | -0.35407052 | -5.73153599 |
| 6 | -0.56396620 | -1.55355500 | -3.61473844 |
| 6 | 0.60722143 | 0.61656509 | -4.17622285 |
| 6 | 3.89975343 | 3.93542292 | 2.06740895 |
| 6 | 2.96008628 | 2.53109275 | 0.24099902 |
| 6 | 3.34404385 | 1.55949582 | 2.54782964 |
| 1 | -4.42390883 | 0.90257324 | 1.26931704 |
| 1 | -3.74147463 | -0.12622608 | -0.02709949 |
| 1 | -3.35092244 | 2.88809867 | 0.22139242 |
| 1 | -1.58955920 | 2.86836518 | -0.01121820 |
| 1 | -3.09730606 | 0.85028387 | -5.22873759 |
| 1 | -4.58168054 | 2.79758152 | -1.70906576 |
| 1 | -1.89649465 | 0.82706751 | 2.93546442 |
| 1 | -3.00998758 | 2.18958834 | 2.64981900 |
| 1 | 1.98559274 | 5.16747074 | 2.85981232 |
| 1 | -2.18657076 | 4.23919049 | 3.21341192 |
| 1 | -0.25995015 | -0.91323983 | -6.21135506 |
| 1 | -2.00078858 | -0.93283165 | -5.87086278 |
| 1 | -1.18001833 | 0.60142532 | -6.26545994 |
| 1 | -0.24421552 | -1.47646996 | -2.57045865 |
| 1 | -1.50725911 | -2.11829498 | -3.66011059 |
| 1 | 0.19886734 | -2.12092174 | -4.16958135 |
| 1 | 0.51230257 | 1.60151197 | -4.65609332 |
| 1 | 0.93442650 | 0.76167901 | -3.14094412 |
| 1 | 1.38589856 | 0.05259218 | -4.71236000 |
| 1 | 4.90427727 | 3.60290280 | 1.76899462 |
| 1 | 3.93624421 | 4.17925894 | 3.13921365 |
| 1 | 3.67793433 | 4.85606324 | 1.50852237 |
| 1 | 2.30875051 | 1.69960449 | -0.04711248 |
| 1 | 2.66340896 | 3.42213483 | -0.33094861 |
| 1 | 3.99243642 | 2.27538731 | -0.04282244 |
| 1 | 3.34198614 | 1.76052653 | 3.62930756 |
| 1 | 2.68558720 | 0.70727314 | 2.34721578 |
| 1 | 4.37013304 | 1.28864069 | 2.25497627 |
| 1 | -3.39032603 | -0.51735485 | 1.67650408 |
| 31 | -0.59758508 | 0.09739808 | 0.39244601 |
| 6 | 1.47464483 | -5.19666944 | 1.73263410 |

| | | | |
|---|---|---|---|
| 6 | 2.36760235 | -3.94107689 | 1.73593204 |
| 8 | 2.11297863 | -3.02710495 | 0.61342040 |
| 6 | 1.02865807 | -2.22877246 | 0.54353149 |
| 6 | -0.36487350 | -2.63832770 | 1.02971547 |
| 8 | 1.16760631 | -1.15554384 | -0.04567584 |
| 6 | -0.95174720 | -3.63227134 | -0.00026762 |
| 6 | 2.37975696 | -3.22132709 | 3.07234751 |
| 1 | 3.38045450 | -4.28863095 | 1.48975692 |
| 1 | -0.28363731 | -3.14604250 | 2.00655573 |
| 1 | -1.93569238 | -3.95284149 | 0.36315849 |
| 1 | -0.32008412 | -4.51738709 | -0.15115493 |
| 1 | -1.08125792 | -3.12443778 | -0.96471928 |
| 1 | 2.76931918 | -3.89928231 | 3.84300008 |
| 1 | 1.37796105 | -2.90572000 | 3.38504035 |
| 1 | 3.03673686 | -2.34480476 | 3.01194698 |

Electronic energy = -3673.046282 a.u.
DFT-D3(BJ) dispersion correction = -0.115532 a.u.
Thermal free energy = 0.583202 a.u.
Gibbs free energy = -3672.578612 a.u.
Number of imaginary frequencies = 0.

**Int2 LA isomer/conformer 37**

| Atomic N. | X | Y | Z |
|---|---|---|---|
| 8 | 2.26061886 | -2.85090120 | -4.50569704 |
| 6 | 3.58964177 | -2.52658188 | -4.96580764 |
| 1 | 3.60738171 | -1.62153321 | -5.58797187 |
| 8 | 0.24306398 | -2.26012872 | -3.80502018 |
| 8 | -2.01979348 | 1.23318806 | -1.61486851 |
| 1 | 3.89760685 | -3.38223214 | -5.57546184 |
| 1 | 4.27888571 | -2.42102559 | -4.11832692 |
| 7 | -2.74150705 | 0.91766006 | 1.19358757 |
| 8 | 0.18336946 | 1.21388386 | 0.80089649 |
| 8 | -1.37144049 | -1.46045987 | 0.05196200 |
| 6 | -3.48115777 | 2.14238809 | 0.80310641 |
| 6 | 0.04065940 | 2.18279280 | 1.73047380 |
| 6 | 1.05714102 | 3.17634129 | 1.87512269 |
| 6 | 0.86713038 | 4.16156362 | 2.85406629 |
| 6 | -0.25977558 | 4.20127737 | 3.67969901 |
| 6 | -1.23340615 | 3.21753177 | 3.54371318 |
| 6 | -1.08875588 | 2.20425026 | 2.59020261 |
| 6 | -2.09665076 | 1.08846365 | 2.53785563 |
| 1 | -0.36691018 | 4.99071008 | 4.42359881 |
| 6 | 2.32295129 | 3.16753301 | 0.99475403 |
| 6 | -1.97569308 | -2.10151405 | 1.07260997 |
| 6 | -1.48109146 | -3.37091352 | 1.50168392 |
| 6 | -2.15378695 | -4.00135825 | 2.55729156 |
| 6 | -3.26705327 | -3.44071493 | 3.19122697 |
| 6 | -3.74250648 | -2.20736146 | 2.76075012 |
| 6 | -3.11540211 | -1.53944801 | 1.70250281 |
| 6 | -3.69762997 | -0.24674980 | 1.19903249 |
| 1 | -3.75282764 | -3.96760138 | 4.01253796 |
| 6 | -0.25952234 | -4.02794511 | 0.82938833 |

| | | | |
|---|---|---|---|
| 6 | 3.28857597 | 4.31188724 | 1.35576985 |
| 6 | 1.93519758 | 3.34521281 | -0.49194675 |
| 6 | 3.09294243 | 1.83850659 | 1.18255592 |
| 6 | 0.07889057 | -5.39234381 | 1.45944842 |
| 6 | 0.98815559 | -3.12694990 | 0.99021495 |
| 6 | -0.54588737 | -4.27065833 | -0.67142741 |
| 1 | -4.27559641 | 2.35035331 | 1.53992685 |
| 1 | -2.79325486 | 2.99334178 | 0.76670547 |
| 1 | -2.88705828 | 1.26083683 | 3.28898760 |
| 1 | -1.62192009 | 0.12473443 | 2.77501058 |
| 1 | 1.62361748 | 4.93402674 | 2.98078681 |
| 1 | -2.11347222 | 3.21642655 | 4.19109076 |
| 1 | -4.04209174 | -0.34843267 | 0.15693163 |
| 1 | -4.56991283 | 0.02720632 | 1.81732729 |
| 1 | -1.79669308 | -4.96807685 | 2.90788257 |
| 1 | -4.61500518 | -1.75295082 | 3.23608240 |
| 1 | 4.17437715 | 4.25095204 | 0.70696991 |
| 1 | 2.83486225 | 5.30201284 | 1.20400620 |
| 1 | 3.63608978 | 4.24484917 | 2.39666378 |
| 1 | 1.28015240 | 2.53295635 | -0.82422676 |
| 1 | 1.41536385 | 4.30214118 | -0.64640420 |
| 1 | 2.84208364 | 3.34816976 | -1.11734473 |
| 1 | 3.41231546 | 1.72337898 | 2.22847932 |
| 1 | 2.47446884 | 0.97612802 | 0.91111532 |
| 1 | 3.99690979 | 1.83926588 | 0.55320686 |
| 1 | 0.95102599 | -5.81663580 | 0.94161890 |
| 1 | -0.74698460 | -6.11126586 | 1.35741245 |
| 1 | 0.33603640 | -5.30603413 | 2.52537467 |
| 1 | 0.84187367 | -2.14904225 | 0.51994538 |
| 1 | 1.22122102 | -2.97202514 | 2.05399766 |
| 1 | 1.85756987 | -3.61317927 | 0.52088072 |
| 1 | -1.41051053 | -4.93920058 | -0.79574662 |
| 1 | -0.74791052 | -3.33596104 | -1.20422744 |
| 1 | 0.32311849 | -4.75493192 | -1.14270117 |
| 1 | -3.91586597 | 1.99537687 | -0.19057868 |
| 31 | -1.21232481 | 0.39205540 | -0.15352490 |
| 6 | 1.36744527 | -1.91332471 | -4.07717119 |
| 6 | 1.82929164 | -0.44033869 | -3.99207683 |
| 8 | 0.63066357 | 0.38766009 | -3.89511705 |
| 6 | -0.07110300 | 0.42596211 | -2.75576574 |
| 6 | -1.39842417 | 1.17322970 | -2.86437330 |
| 8 | 0.29597111 | -0.07325929 | -1.69086144 |
| 6 | -1.21671173 | 2.57444541 | -3.46989254 |
| 6 | 2.88587262 | -0.13211033 | -2.92539527 |
| 1 | 2.21531823 | -0.14406461 | -4.97823326 |
| 1 | -2.00337077 | 0.56187485 | -3.56842295 |
| 1 | -2.20092864 | 3.05444480 | -3.53316419 |
| 1 | -0.78135211 | 2.52382747 | -4.47638842 |
| 1 | -0.57353470 | 3.18784123 | -2.82454483 |
| 1 | 3.87695463 | -0.43792817 | -3.28146953 |
| 1 | 2.67200787 | -0.62367383 | -1.97275718 |
| 1 | 2.91800062 | 0.95216844 | -2.75753855 |

Electronic energy = -3673.045721 a.u.

DFT-D3(BJ) dispersion correction = -0.116218 a.u.
Thermal free energy = 0.583343 a.u.
Gibbs free energy = -3672.578597 a.u.
Number of imaginary frequencies = 0.

**Int2 LA isomer/conformer 38**

| Atomic N. | X | Y | Z |
|---|---|---|---|
| 8 | 2.08497693 | -3.02614605 | 2.51955960 |
| 6 | 3.35299158 | -2.50803746 | 2.03519982 |
| 1 | 3.17372562 | -1.66862710 | 1.35158970 |
| 8 | 1.75610682 | -1.06140615 | 3.61883806 |
| 8 | -0.49540584 | -1.49981197 | 1.43678394 |
| 1 | 3.81851797 | -3.34515316 | 1.50721865 |
| 1 | 3.97488874 | -2.17883816 | 2.87606863 |
| 7 | 0.04926866 | 1.59287054 | 1.67408796 |
| 8 | -1.34723551 | 0.58233058 | -0.79529358 |
| 8 | 1.63301435 | -0.06987149 | -0.26471117 |
| 6 | -0.57303201 | 1.38710035 | 3.00900295 |
| 6 | -2.33949935 | 1.42075750 | -0.38483320 |
| 6 | -3.65384933 | 1.29434064 | -0.92039405 |
| 6 | -4.63159391 | 2.17304140 | -0.43027537 |
| 6 | -4.36117721 | 3.15494415 | 0.52647365 |
| 6 | -3.06235276 | 3.30194623 | 1.00063668 |
| 6 | -2.04662460 | 2.45646807 | 0.54092599 |
| 6 | -0.61928384 | 2.72607273 | 0.93559381 |
| 1 | -5.15863431 | 3.80803059 | 0.88048689 |
| 6 | -3.99320476 | 0.25927136 | -2.01138707 |
| 6 | 2.32865601 | 1.08768417 | -0.43998320 |
| 6 | 3.12793165 | 1.27038908 | -1.60494166 |
| 6 | 3.83831289 | 2.47518531 | -1.71143770 |
| 6 | 3.78255139 | 3.47690070 | -0.73885092 |
| 6 | 3.00139435 | 3.28266439 | 0.39463884 |
| 6 | 2.28345575 | 2.09232405 | 0.56090572 |
| 6 | 1.53114623 | 1.86131715 | 1.84673634 |
| 1 | 4.34761222 | 4.39940530 | -0.87116638 |
| 6 | 3.21109923 | 0.19860089 | -2.71038376 |
| 6 | -5.46363653 | 0.35474660 | -2.46005821 |
| 6 | -3.76523106 | -1.17586195 | -1.48602367 |
| 6 | -3.11227585 | 0.50856672 | -3.25911554 |
| 6 | 4.16382701 | 0.61645392 | -3.84661349 |
| 6 | 1.81565773 | -0.02298175 | -3.33982418 |
| 6 | 3.74789233 | -1.13109010 | -2.12929659 |
| 1 | -0.47842761 | 2.30978184 | 3.60365947 |
| 1 | -1.63419131 | 1.14542288 | 2.88506875 |
| 1 | -0.56376132 | 3.62506524 | 1.57127301 |
| 1 | -0.00285490 | 2.90587991 | 0.04234579 |
| 1 | -5.64913461 | 2.09074252 | -0.80748397 |
| 1 | -2.81847935 | 4.09030153 | 1.71632693 |
| 1 | 1.92521598 | 0.98826199 | 2.38936232 |
| 1 | 1.63627958 | 2.74344415 | 2.49936792 |
| 1 | 4.45600534 | 2.64719224 | -2.59079002 |
| 1 | 2.95220843 | 4.04877147 | 1.17156438 |

| | | | |
|---|---|---|---|
| 1 | -5.64398216 | -0.38898198 | -3.24905494 |
| 1 | -6.16346060 | 0.14068830 | -1.63926256 |
| 1 | -5.70566999 | 1.34321372 | -2.87613552 |
| 1 | -2.71557370 | -1.33576214 | -1.21959472 |
| 1 | -4.38768802 | -1.37315747 | -0.60113613 |
| 1 | -4.03778105 | -1.90717966 | -2.26155593 |
| 1 | -3.30037620 | 1.51078435 | -3.67068974 |
| 1 | -2.04548180 | 0.42429850 | -3.02365144 |
| 1 | -3.35615880 | -0.22811658 | -4.03947099 |
| 1 | 4.19225496 | -0.18346823 | -4.59993793 |
| 1 | 5.19219862 | 0.77073776 | -3.48908703 |
| 1 | 3.82917398 | 1.53331577 | -4.35276397 |
| 1 | 1.08514925 | -0.36368644 | -2.59873222 |
| 1 | 1.44068848 | 0.90713039 | -3.79033588 |
| 1 | 1.88143074 | -0.78191308 | -4.13400321 |
| 1 | 4.75350039 | -0.99074417 | -1.70618149 |
| 1 | 3.08938226 | -1.52138030 | -1.34599821 |
| 1 | 3.82062365 | -1.88345085 | -2.92906133 |
| 1 | -0.05611955 | 0.56672752 | 3.52034752 |
| 31 | -0.08561977 | -0.01672061 | 0.43972763 |
| 6 | 1.37735344 | -2.17450481 | 3.28230201 |
| 6 | 0.06189902 | -2.76360298 | 3.78710611 |
| 8 | -0.40232318 | -3.92340953 | 3.05728130 |
| 6 | -0.92010170 | -3.93420339 | 1.78909622 |
| 6 | -0.94651792 | -2.70910059 | 0.84311575 |
| 8 | -1.38085803 | -4.98390828 | 1.39130612 |
| 6 | -0.17278184 | -3.07816201 | -0.43090181 |
| 6 | 0.22959458 | -3.23144499 | 5.23392466 |
| 1 | -0.67944482 | -1.95896482 | 3.72540780 |
| 1 | -2.01801112 | -2.62198356 | 0.58249833 |
| 1 | -0.32245143 | -2.29996768 | -1.19551003 |
| 1 | -0.53604372 | -4.02922726 | -0.83711014 |
| 1 | 0.90122400 | -3.16088703 | -0.21833630 |
| 1 | 0.58592996 | -2.40134025 | 5.85679220 |
| 1 | 0.94889583 | -4.05885853 | 5.28889347 |
| 1 | -0.73570453 | -3.58121991 | 5.61986028 |

Electronic energy = -3673.043774 a.u.
DFT-D3(BJ) dispersion correction = -0.118843 a.u.
Thermal free energy = 0.584237 a.u.
Gibbs free energy = -3672.578380 a.u.
Number of imaginary frequencies = 0.

**Int2 LA isomer/conformer 39**

| Atomic N. | X | Y | Z |
|---|---|---|---|
| 8 | 0.17438142 | -2.32388307 | 4.79255239 |
| 6 | 1.36717633 | -1.57075179 | 4.47222631 |
| 1 | 2.26148097 | -2.20215191 | 4.42625543 |
| 8 | -1.12953209 | -4.10351955 | 4.61835872 |
| 8 | 1.06948337 | -1.76394565 | -0.72417353 |
| 1 | 1.46906115 | -0.84570405 | 5.28644411 |
| 1 | 1.22983714 | -1.03342602 | 3.52598171 |
| 7 | -2.15245333 | -0.85246690 | -0.53414854 |

| | | | |
|---|---|---|---|
| 8 | -0.05884874 | 0.65106851 | -2.02174826 |
| 8 | -0.02019455 | 0.56520573 | 1.07357871 |
| 6 | -2.45942072 | -2.25904125 | -0.90579049 |
| 6 | -0.83256445 | 0.36098843 | -3.09879473 |
| 6 | -0.29554327 | 0.43181916 | -4.41638867 |
| 6 | -1.15993053 | 0.11907268 | -5.47612723 |
| 6 | -2.49630716 | -0.24507164 | -5.28589454 |
| 6 | -3.01415967 | -0.28649788 | -3.99540538 |
| 6 | -2.19755835 | 0.02675600 | -2.90384214 |
| 6 | -2.77828822 | 0.10673299 | -1.51942576 |
| 1 | -3.12472892 | -0.48440952 | -6.14371188 |
| 6 | 1.16968880 | 0.83522876 | -4.67145699 |
| 6 | -1.07436291 | 1.33240468 | 1.47034855 |
| 6 | -0.84670316 | 2.62842317 | 2.01760558 |
| 6 | -1.97142221 | 3.35917022 | 2.42813984 |
| 6 | -3.27532997 | 2.87213037 | 2.31168730 |
| 6 | -3.48261436 | 1.60774670 | 1.77263712 |
| 6 | -2.39621026 | 0.82711440 | 1.36066452 |
| 6 | -2.65164234 | -0.57596662 | 0.86855703 |
| 1 | -4.11815167 | 3.48052523 | 2.63923506 |
| 6 | 0.57166267 | 3.21948746 | 2.15125108 |
| 6 | 1.49766473 | 0.88935087 | -6.17550802 |
| 6 | 2.12511565 | -0.19663839 | -4.02701098 |
| 6 | 1.44078748 | 2.24365933 | -4.09074245 |
| 6 | 0.55429030 | 4.62499493 | 2.78210492 |
| 6 | 1.22886868 | 3.35216532 | 0.75723533 |
| 6 | 1.43945582 | 2.32188132 | 3.06395556 |
| 1 | -3.54932553 | -2.41780001 | -0.87663480 |
| 1 | -2.09422771 | -2.45393740 | -1.92006065 |
| 1 | -3.86219610 | -0.09417850 | -1.54425467 |
| 1 | -2.62815899 | 1.11070377 | -1.09679194 |
| 1 | -0.77838910 | 0.15501274 | -6.49500680 |
| 1 | -4.06228160 | -0.54316756 | -3.82497996 |
| 1 | -2.15444715 | -1.31683657 | 1.50954254 |
| 1 | -3.73486610 | -0.77931745 | 0.88381727 |
| 1 | -1.83037951 | 4.35286017 | 2.84874363 |
| 1 | -4.49363510 | 1.20548491 | 1.67854303 |
| 1 | 2.54597969 | 1.19573092 | -6.30097616 |
| 1 | 1.38028271 | -0.08996212 | -6.66186044 |
| 1 | 0.87306485 | 1.62048472 | -6.70905746 |
| 1 | 1.97791269 | -0.27000200 | -2.94421854 |
| 1 | 1.96819784 | -1.19398809 | -4.46316712 |
| 1 | 3.16928686 | 0.09571252 | -4.21788385 |
| 1 | 0.79419978 | 2.99171364 | -4.57248639 |
| 1 | 1.26381834 | 2.27489200 | -3.01026046 |
| 1 | 2.48667014 | 2.52843116 | -4.28229900 |
| 1 | 1.58720632 | 4.99304626 | 2.86010343 |
| 1 | 0.12747946 | 4.61973450 | 3.79544759 |
| 1 | -0.00692975 | 5.34622367 | 2.17096438 |
| 1 | 1.32966717 | 2.38236255 | 0.25911114 |
| 1 | 0.63288102 | 4.01052208 | 0.10924672 |
| 1 | 2.23140938 | 3.79475612 | 0.85933756 |
| 1 | 1.00386894 | 2.25819649 | 4.07196757 |

| | | | |
|---|---|---|---|
| 1 | 1.52569179 | 1.30904598 | 2.65621120 |
| 1 | 2.44965838 | 2.74870344 | 3.15694999 |
| 1 | -1.96515300 | -2.93176993 | -0.19824878 |
| 31 | -0.15283084 | -0.42048420 | -0.49912643 |
| 6 | -0.11273213 | -3.54084391 | 4.26893879 |
| 6 | 0.85393802 | -4.31340350 | 3.34370069 |
| 8 | 1.64291572 | -3.55072835 | 2.37532655 |
| 6 | 0.92937926 | -2.88130489 | 1.43282430 |
| 6 | 1.82370192 | -2.33077180 | 0.31288010 |
| 8 | -0.28397216 | -2.76422350 | 1.47520497 |
| 6 | 2.90886028 | -1.38627312 | 0.85632213 |
| 6 | 1.84228608 | -5.13628175 | 4.16418469 |
| 1 | 0.18808379 | -4.97338998 | 2.77311670 |
| 1 | 2.33044780 | -3.22236340 | -0.10591793 |
| 1 | 3.54439796 | -1.07053709 | 0.01969706 |
| 1 | 3.53398870 | -1.88624188 | 1.60787589 |
| 1 | 2.45323204 | -0.49155089 | 1.30272625 |
| 1 | 1.28577693 | -5.77792267 | 4.85859596 |
| 1 | 2.52106823 | -4.49763603 | 4.74412132 |
| 1 | 2.44118508 | -5.77115309 | 3.49908024 |

Electronic energy = -3673.043905 a.u.
DFT-D3(BJ) dispersion correction = -0.116935 a.u.
Thermal free energy = 0.582763 a.u.
Gibbs free energy = -3672.578077 a.u.
Number of imaginary frequencies = 0.

**Int2 LA isomer/conformer 40**

| Atomic N. | X | Y | Z |
|---|---|---|---|
| 8 | -3.37064581 | 5.03490602 | 2.29798170 |
| 6 | -4.38513307 | 5.20506868 | 3.32459431 |
| 1 | -4.70022147 | 4.22773389 | 3.70891152 |
| 8 | -2.04687112 | 4.04121195 | 3.85935565 |
| 8 | -2.05468445 | 0.36709005 | 0.70342576 |
| 1 | -5.21387493 | 5.71515746 | 2.82594607 |
| 1 | -3.99029086 | 5.80919413 | 4.15019802 |
| 7 | -1.56869707 | -1.86919486 | -1.09191421 |
| 8 | 0.78158335 | -1.43240195 | 0.66606093 |
| 8 | 0.00050371 | 0.59887446 | -1.65136837 |
| 6 | -2.75205282 | -2.34987851 | -0.33804560 |
| 6 | 0.68168538 | -2.76772184 | 0.85701147 |
| 6 | 1.35049542 | -3.36923897 | 1.96633761 |
| 6 | 1.19913227 | -4.75242804 | 2.13837404 |
| 6 | 0.44558099 | -5.54719772 | 1.26994447 |
| 6 | -0.16418552 | -4.95650913 | 0.16873961 |
| 6 | -0.04019550 | -3.58079639 | -0.05494429 |
| 6 | -0.59327340 | -2.98586600 | -1.32110182 |
| 1 | 0.35190507 | -6.61827853 | 1.44932230 |
| 6 | 2.23212532 | -2.54292398 | 2.92443015 |
| 6 | 0.03710232 | 0.10244073 | -2.90429925 |
| 6 | 1.03000110 | 0.56523592 | -3.82047417 |
| 6 | 1.01109334 | 0.03115362 | -5.11612218 |
| 6 | 0.07562506 | -0.92224975 | -5.53003650 |

| | | | |
|---|---|---|---|
| 6 | -0.88803047 | -1.36291186 | -4.62983894 |
| 6 | -0.92347601 | -0.85205817 | -3.32714448 |
| 6 | -2.02933114 | -1.27719048 | -2.39851194 |
| 1 | 0.10620650 | -1.31392954 | -6.54676229 |
| 6 | 2.08614584 | 1.60729962 | -3.40223176 |
| 6 | 2.86793234 | -3.41723952 | 4.02165819 |
| 6 | 1.39507145 | -1.45267375 | 3.63175162 |
| 6 | 3.38570402 | -1.88119564 | 2.13282405 |
| 6 | 3.03299432 | 1.96562285 | -4.56305304 |
| 6 | 2.95658210 | 1.05043366 | -2.25071767 |
| 6 | 1.39412008 | 2.91648150 | -2.95242680 |
| 1 | -3.27117516 | -3.13319874 | -0.91595023 |
| 1 | -2.43148693 | -2.76763352 | 0.62220727 |
| 1 | -1.08870169 | -3.76991218 | -1.91993560 |
| 1 | 0.21610524 | -2.56414683 | -1.93567988 |
| 1 | 1.68687129 | -5.23531825 | 2.98317315 |
| 1 | -0.73050812 | -5.56219892 | -0.54263356 |
| 1 | -2.66381681 | -0.41929244 | -2.12274899 |
| 1 | -2.66764366 | -2.02053180 | -2.90666461 |
| 1 | 1.75838071 | 0.36270038 | -5.83493349 |
| 1 | -1.63232376 | -2.10293044 | -4.93321186 |
| 1 | 3.49377025 | -2.78232483 | 4.66492185 |
| 1 | 2.11199804 | -3.89433536 | 4.66252281 |
| 1 | 3.51424291 | -4.20190494 | 3.60270053 |
| 1 | 0.98655548 | -0.74203803 | 2.90618195 |
| 1 | 0.56609503 | -1.90299329 | 4.19797880 |
| 1 | 2.02769704 | -0.89722824 | 4.34069054 |
| 1 | 4.01681426 | -2.64650693 | 1.65797819 |
| 1 | 3.00456912 | -1.20985668 | 1.35556707 |
| 1 | 4.01941230 | -1.29687170 | 2.81745471 |
| 1 | 3.75762515 | 2.71514630 | -4.21405350 |
| 1 | 2.49459636 | 2.39813159 | -5.41901786 |
| 1 | 3.60396741 | 1.09441293 | -4.91537089 |
| 1 | 2.35810156 | 0.82793612 | -1.36138741 |
| 1 | 3.47127963 | 0.13084707 | -2.56502424 |
| 1 | 3.72388871 | 1.78948153 | -1.97385930 |
| 1 | 0.80413323 | 3.34556194 | -3.77596733 |
| 1 | 0.73099892 | 2.73780570 | -2.09905083 |
| 1 | 2.15464230 | 3.65610081 | -2.65834211 |
| 1 | -3.42492829 | -1.50654869 | -0.15344795 |
| 31 | -0.51917902 | -0.33576488 | -0.11016013 |
| 6 | -2.23210232 | 4.43573511 | 2.72725431 |
| 6 | -1.17114615 | 4.41934332 | 1.61403554 |
| 8 | -0.21543751 | 3.32775377 | 1.77144125 |
| 6 | -0.51416972 | 2.06432489 | 1.40838491 |
| 6 | -1.88757321 | 1.41539537 | 1.61510446 |
| 8 | 0.40618651 | 1.37267807 | 0.96995865 |
| 6 | -1.95746253 | 0.90460092 | 3.07185240 |
| 6 | -1.66701712 | 4.53681126 | 0.17751952 |
| 1 | -0.53765413 | 5.29273833 | 1.83888024 |
| 1 | -2.68563684 | 2.16488057 | 1.45934659 |
| 1 | -2.93554372 | 0.42568773 | 3.20880348 |
| 1 | -1.84193589 | 1.70856902 | 3.80838245 |

| | | | |
|---|---|---|---|
| 1 | -1.17810944 | 0.14791049 | 3.23001471 |
| 1 | -2.21150784 | 5.47988104 | 0.05179930 |
| 1 | -2.33763209 | 3.71523861 | -0.10202773 |
| 1 | -0.80586588 | 4.53584389 | -0.50256134 |

Electronic energy = -3673.044944 a.u.
DFT-D3(BJ) dispersion correction = -0.115532 a.u.
Thermal free energy = 0.582753 a.u.
Gibbs free energy = -3672.577722 a.u.
Number of imaginary frequencies = 0.

**Int2 LA isomer/conformer 41**

| Atomic N. | X | Y | Z |
|---|---|---|---|
| 8 | -2.73114925 | 0.35525895 | 5.29875838 |
| 6 | -1.93782040 | 1.49138578 | 5.70665991 |
| 1 | -1.04203542 | 1.58487413 | 5.07946511 |
| 8 | -4.39898463 | -0.53134713 | 4.13781845 |
| 8 | 0.42498562 | 1.40652923 | 1.36209631 |
| 1 | -1.63883198 | 1.27637189 | 6.73825893 |
| 1 | -2.50700099 | 2.42917185 | 5.68157593 |
| 7 | 0.09356793 | -1.92462899 | 0.95817371 |
| 8 | 1.89307114 | -0.16737597 | -0.63094073 |
| 8 | -1.16351244 | 0.01481612 | -0.99607009 |
| 6 | 0.23658655 | -2.02152389 | 2.43451166 |
| 6 | 2.87538198 | -0.91155210 | -0.06497640 |
| 6 | 4.21966456 | -0.44012479 | -0.03476708 |
| 6 | 5.17442948 | -1.27086926 | 0.57046238 |
| 6 | 4.85922993 | -2.51580802 | 1.12353508 |
| 6 | 3.54788481 | -2.97654100 | 1.06175096 |
| 6 | 2.55913722 | -2.19120602 | 0.46034201 |
| 6 | 1.16946125 | -2.73069591 | 0.26844500 |
| 1 | 5.63847534 | -3.12055324 | 1.58739967 |
| 6 | 4.61232898 | 0.92055540 | -0.64206515 |
| 6 | -1.52121973 | -1.12936788 | -1.63963260 |
| 6 | -1.89449747 | -1.08196398 | -3.01450980 |
| 6 | -2.26261738 | -2.29048234 | -3.62312290 |
| 6 | -2.27190031 | -3.51005786 | -2.94157251 |
| 6 | -1.91459456 | -3.54021022 | -1.59857912 |
| 6 | -1.55207487 | -2.36165254 | -0.93530732 |
| 6 | -1.27851654 | -2.41595371 | 0.54748828 |
| 1 | -2.55818066 | -4.42492396 | -3.46017260 |
| 6 | -1.89917873 | 0.23965574 | -3.80886978 |
| 6 | 6.12712584 | 1.18037709 | -0.54004387 |
| 6 | 3.89451804 | 2.06529065 | 0.11094581 |
| 6 | 4.23909633 | 0.95862436 | -2.14329373 |
| 6 | -2.35097709 | 0.03404971 | -5.26738834 |
| 6 | -0.47657940 | 0.84559641 | -3.85330840 |
| 6 | -2.88589508 | 1.24047246 | -3.16223008 |
| 1 | 0.13639123 | -3.07349675 | 2.74625331 |
| 1 | 1.22470203 | -1.65112549 | 2.72882886 |
| 1 | 1.10331631 | -3.76706713 | 0.63924382 |
| 1 | 0.90311236 | -2.73293728 | -0.79842908 |
| 1 | 6.20909984 | -0.93580996 | 0.61857271 |

| | | | |
|---|---|---|---|
| 1 | 3.28414729 | -3.95864321 | 1.46115607 |
| 1 | -1.99270067 | -1.79285649 | 1.10237144 |
| 1 | -1.38014410 | -3.45486941 | 0.90172132 |
| 1 | -2.54747168 | -2.28690890 | -4.67350775 |
| 1 | -1.92746902 | -4.48095087 | -1.04369820 |
| 1 | 6.35401003 | 2.15235817 | -1.00094211 |
| 1 | 6.47220347 | 1.22205079 | 0.50333037 |
| 1 | 6.71433262 | 0.41701770 | -1.07094403 |
| 1 | 2.80483724 | 1.96778807 | 0.06001282 |
| 1 | 4.18895838 | 2.07453589 | 1.17064223 |
| 1 | 4.17975299 | 3.03383767 | -0.32827061 |
| 1 | 4.77585989 | 0.17320456 | -2.69537571 |
| 1 | 3.16353392 | 0.81743756 | -2.29466349 |
| 1 | 4.52826896 | 1.93020744 | -2.57237946 |
| 1 | -2.34545980 | 1.00544317 | -5.78206464 |
| 1 | -3.37209375 | -0.36864297 | -5.33253755 |
| 1 | -1.67591175 | -0.63543610 | -5.81969235 |
| 1 | -0.09912600 | 1.07534508 | -2.85171358 |
| 1 | 0.22591838 | 0.14983049 | -4.33414650 |
| 1 | -0.48779556 | 1.77586299 | -4.44148246 |
| 1 | -3.91074767 | 0.84199095 | -3.18440634 |
| 1 | -2.61901400 | 1.44999432 | -2.12078192 |
| 1 | -2.87860316 | 2.18743460 | -3.72322523 |
| 1 | -0.54348962 | -1.42093551 | 2.91238684 |
| 31 | 0.23818069 | -0.01681666 | 0.22220827 |
| 6 | -3.67540444 | 0.42232775 | 4.31440615 |
| 6 | -3.86530804 | 1.74653414 | 3.53927049 |
| 8 | -2.61765937 | 2.33014359 | 3.03098852 |
| 6 | -1.80625427 | 1.52044183 | 2.30446269 |
| 6 | -0.60896797 | 2.28024083 | 1.72618198 |
| 8 | -2.00707587 | 0.32876511 | 2.14232133 |
| 6 | -1.07681132 | 3.18513876 | 0.57160356 |
| 6 | -4.90017497 | 1.61218701 | 2.43471736 |
| 1 | -4.19533361 | 2.50588696 | 4.26576772 |
| 1 | -0.22528048 | 2.92613652 | 2.53777462 |
| 1 | -0.20499693 | 3.72114444 | 0.17604108 |
| 1 | -1.81910604 | 3.91798334 | 0.91587354 |
| 1 | -1.51468729 | 2.58194637 | -0.23591493 |
| 1 | -5.86460656 | 1.32517668 | 2.87033236 |
| 1 | -4.60978789 | 0.83730772 | 1.71726234 |
| 1 | -5.01452185 | 2.57460534 | 1.91960706 |

Electronic energy = -3673.043820 a.u.
DFT-D3(BJ) dispersion correction = -0.115894 a.u.
Thermal free energy = 0.582017 a.u.
Gibbs free energy = -3672.577696 a.u.
Number of imaginary frequencies = 0.

**Int2_LA isomer/conformer 42**

| Atomic N. | X | Y | Z |
|---|---|---|---|
| 7 | -2.37660965 | 0.19296114 | -0.97708220 |
| 8 | 0.60564279 | 0.52005733 | -0.61032345 |
| 8 | -1.38425449 | 2.19111600 | 1.02401769 |

| | | | |
|---|---|---|---|
| 6 | -3.04058049 | -1.13490046 | -0.92308311 |
| 6 | 0.68181056 | -0.26579319 | -1.71811939 |
| 6 | 1.92606734 | -0.84913110 | -2.09048525 |
| 6 | 1.92068558 | -1.70852248 | -3.19955509 |
| 6 | 0.77101088 | -1.97064510 | -3.95007918 |
| 6 | -0.41611092 | -1.32148686 | -3.62658322 |
| 6 | -0.46689781 | -0.45399131 | -2.53027295 |
| 6 | -1.69015421 | 0.39637947 | -2.30530769 |
| 1 | 0.81577753 | -2.65486413 | -4.79726205 |
| 6 | 3.23792427 | -0.49738657 | -1.35761547 |
| 6 | -1.90487810 | 3.09595836 | 0.15908623 |
| 6 | -1.49755251 | 4.46058839 | 0.21117027 |
| 6 | -2.08617511 | 5.33942366 | -0.70931097 |
| 6 | -3.03595284 | 4.93077032 | -1.65076494 |
| 6 | -3.43725279 | 3.60011857 | -1.67961451 |
| 6 | -2.88952168 | 2.68295591 | -0.77470277 |
| 6 | -3.41285164 | 1.27261055 | -0.73893010 |
| 1 | -3.45812130 | 5.65251176 | -2.34984069 |
| 6 | -0.45645081 | 4.95415403 | 1.23457017 |
| 6 | 4.44904832 | -1.22765215 | -1.96795319 |
| 6 | 3.17930597 | -0.88037320 | 0.13848695 |
| 6 | 3.49557720 | 1.02359609 | -1.48815197 |
| 6 | -0.20882653 | 6.46991028 | 1.11744997 |
| 6 | 0.89928467 | 4.24727826 | 0.99919920 |
| 6 | -0.95553800 | 4.67746349 | 2.67292323 |
| 1 | -3.84469533 | -1.17195785 | -1.67558219 |
| 1 | -2.30507013 | -1.92039634 | -1.11352370 |
| 1 | -2.42994471 | 0.21494239 | -3.10269990 |
| 1 | -1.41661452 | 1.46155865 | -2.33566885 |
| 1 | 2.84950187 | -2.19196997 | -3.49701030 |
| 1 | -1.31009139 | -1.46394507 | -4.23795640 |
| 1 | -3.84797543 | 1.04473300 | 0.24712514 |
| 1 | -4.20441676 | 1.14915955 | -1.49634330 |
| 1 | -1.79071225 | 6.38698895 | -0.70050436 |
| 1 | -4.19013799 | 3.26268329 | -2.39557415 |
| 1 | 5.35687681 | -0.92901219 | -1.42495218 |
| 1 | 4.35881405 | -2.32066090 | -1.88465484 |
| 1 | 4.59818173 | -0.97025353 | -3.02617733 |
| 1 | 2.39196689 | -0.32475185 | 0.65851094 |
| 1 | 3.00043254 | -1.95748639 | 0.26423333 |
| 1 | 4.14077952 | -0.64359280 | 0.61843332 |
| 1 | 3.58866247 | 1.31217495 | -2.54497706 |
| 1 | 2.68536074 | 1.60832189 | -1.03710452 |
| 1 | 4.43636953 | 1.28700081 | -0.98127000 |
| 1 | 0.52854149 | 6.77016698 | 1.87536290 |
| 1 | -1.12339551 | 7.05358310 | 1.29663292 |
| 1 | 0.19666948 | 6.74987466 | 0.13435723 |
| 1 | 0.81751348 | 3.16213818 | 1.12091895 |
| 1 | 1.27593401 | 4.45483886 | -0.01282890 |
| 1 | 1.64204361 | 4.62036697 | 1.72059087 |
| 1 | -1.89715296 | 5.21249156 | 2.86476876 |
| 1 | -1.12145424 | 3.60818002 | 2.84306139 |
| 1 | -0.21024401 | 5.03579104 | 3.39926585 |

| | | | |
|---|---|---|---|
| 1 | -3.46277001 | -1.28718677 | 0.07703125 |
| 8 | -0.15066878 | -6.51923448 | 2.81439189 |
| 6 | -1.40076232 | -5.79716431 | 2.77647557 |
| 1 | -1.32100496 | -4.86342584 | 3.34625183 |
| 8 | 1.92077682 | -6.84331821 | 2.09549525 |
| 8 | -1.04667471 | -0.58171785 | 1.91511844 |
| 1 | -2.12807418 | -6.46015999 | 3.25634472 |
| 1 | -1.72751997 | -5.58595275 | 1.74966772 |
| 31 | -0.92862799 | 0.48665875 | 0.44324958 |
| 6 | 0.90962915 | -6.17953267 | 2.02649103 |
| 6 | 0.76340010 | -4.99030397 | 1.05977075 |
| 8 | 0.63913349 | -3.79386446 | 1.90060588 |
| 6 | -0.13832351 | -2.78776588 | 1.40191734 |
| 6 | -0.11950330 | -1.55535723 | 2.32555729 |
| 8 | -0.74514625 | -2.86322751 | 0.35036227 |
| 6 | -0.42921604 | -1.92703228 | 3.78145735 |
| 6 | 1.95619431 | -4.86935060 | 0.13085610 |
| 1 | -0.16239370 | -5.08382888 | 0.47283008 |
| 1 | 0.92406341 | -1.18254371 | 2.28038660 |
| 1 | -0.35444782 | -1.02253922 | 4.39718329 |
| 1 | 0.27198083 | -2.67971506 | 4.16154689 |
| 1 | -1.45660066 | -2.30850771 | 3.86100639 |
| 1 | 2.05579087 | -5.78836134 | -0.46073854 |
| 1 | 2.88101039 | -4.73492197 | 0.70347510 |
| 1 | 1.80960559 | -4.02572104 | -0.55503317 |

Electronic energy = -3673.042099 a.u.
DFT-D3(BJ) dispersion correction = -0.115814 a.u.
Thermal free energy = 0.580251 a.u.
Gibbs free energy = -3672.577662 a.u.
Number of imaginary frequencies = 0.

**Int2_LA isomer/conformer 43**

| Atomic N. | X | Y | Z |
|---|---|---|---|
| 7 | -1.93851157 | -1.77998891 | -0.51779312 |
| 8 | -0.06392905 | 0.57430427 | -0.89869569 |
| 8 | -1.84924126 | 0.42624170 | 1.46547105 |
| 6 | -1.50581069 | -3.16614380 | -0.81517041 |
| 6 | -0.02764796 | 0.14168782 | -2.18139423 |
| 6 | 0.97703290 | 0.61801801 | -3.07286809 |
| 6 | 0.98321117 | 0.09540850 | -4.37421690 |
| 6 | 0.04266200 | -0.83602237 | -4.82310369 |
| 6 | -0.97359904 | -1.24121571 | -3.96409643 |
| 6 | -1.02850407 | -0.74667267 | -2.65681209 |
| 6 | -2.23053074 | -1.03781987 | -1.80050512 |
| 1 | 0.09487861 | -1.21915026 | -5.84220083 |
| 6 | 1.99562661 | 1.69091616 | -2.64115001 |
| 6 | -3.14967463 | 0.56308222 | 1.16988766 |
| 6 | -3.83548951 | 1.77997770 | 1.47385671 |
| 6 | -5.19628213 | 1.85188044 | 1.14936731 |
| 6 | -5.89352875 | 0.79672902 | 0.55010010 |
| 6 | -5.22068775 | -0.38629054 | 0.26735781 |
| 6 | -3.86266710 | -0.51549940 | 0.58290351 |

| | | | |
|---|---|---|---|
| 6 | -3.17615030 | -1.83355496 | 0.35880284 |
| 1 | -6.95140039 | 0.90518019 | 0.31083692 |
| 6 | -3.10525258 | 2.96495179 | 2.13423408 |
| 6 | 2.94790050 | 2.07658774 | -3.78878414 |
| 6 | 2.86768450 | 1.17866346 | -1.47265069 |
| 6 | 1.24581676 | 2.97419140 | -2.20968651 |
| 6 | -4.04985822 | 4.15421832 | 2.38956388 |
| 6 | -1.96804778 | 3.46810872 | 1.21370743 |
| 6 | -2.52423477 | 2.53283543 | 3.50190997 |
| 1 | -2.31862890 | -3.70155532 | -1.33244842 |
| 1 | -0.61441785 | -3.14888040 | -1.44781192 |
| 1 | -2.97126029 | -1.62375657 | -2.37023957 |
| 1 | -2.71272420 | -0.09855769 | -1.49394021 |
| 1 | 1.75144341 | 0.42493468 | -5.07149794 |
| 1 | -1.75120236 | -1.92682093 | -4.30931955 |
| 1 | -2.83808747 | -2.27055489 | 1.31307971 |
| 1 | -3.88336268 | -2.54056224 | -0.10574345 |
| 1 | -5.74163208 | 2.76921126 | 1.36494722 |
| 1 | -5.74634078 | -1.22720908 | -0.19150028 |
| 1 | 3.63644767 | 2.85742928 | -3.43495035 |
| 1 | 3.55727405 | 1.22517038 | -4.12546566 |
| 1 | 2.40723214 | 2.48223238 | -4.65585860 |
| 1 | 2.25954763 | 0.95545396 | -0.58963987 |
| 1 | 3.41341764 | 0.26873768 | -1.76023745 |
| 1 | 3.60493660 | 1.95256025 | -1.20496546 |
| 1 | 0.65446314 | 3.37600193 | -3.04518553 |
| 1 | 0.57202677 | 2.78130073 | -1.36747087 |
| 1 | 1.97018840 | 3.74622331 | -1.90731624 |
| 1 | -3.48162621 | 4.96515282 | 2.86730919 |
| 1 | -4.87711076 | 3.88818074 | 3.06370104 |
| 1 | -4.47504117 | 4.55385693 | 1.45736114 |
| 1 | -1.23016200 | 2.68325784 | 1.01747666 |
| 1 | -2.37284929 | 3.81201393 | 0.25059955 |
| 1 | -1.45487065 | 4.31856259 | 1.68842157 |
| 1 | -3.32961267 | 2.21448827 | 4.18028164 |
| 1 | -1.81257551 | 1.70741438 | 3.39218749 |
| 1 | -2.00569918 | 3.38373064 | 3.97007299 |
| 1 | -1.27660542 | -3.68757498 | 0.12227607 |
| 8 | 4.56861895 | -0.68806065 | 1.09654301 |
| 6 | 5.21075020 | 0.54663658 | 1.52097020 |
| 1 | 4.80726572 | 0.86300383 | 2.49023437 |
| 8 | 5.46403425 | -1.72819066 | 2.91703587 |
| 8 | 0.39494650 | -1.00846846 | 1.99773205 |
| 1 | 4.97048309 | 1.27350024 | 0.74094580 |
| 1 | 6.29322668 | 0.39621064 | 1.60751675 |
| 31 | -0.58246364 | -0.57049981 | 0.48732048 |
| 6 | 4.78489665 | -1.73980114 | 1.90812739 |
| 6 | 4.10846364 | -3.02840545 | 1.42262635 |
| 8 | 3.11842704 | -2.83058427 | 0.37472411 |
| 6 | 1.92525755 | -2.25093417 | 0.61920382 |
| 6 | 1.43640184 | -1.93132720 | 2.03535033 |
| 8 | 1.19954223 | -2.01728099 | -0.34159653 |
| 6 | 0.96972190 | -3.23035097 | 2.73236061 |

| | | | |
|---|---|---|---|
| 6 | 5.15186055 | -3.97944503 | 0.84166552 |
| 1 | 3.64498619 | -3.48330726 | 2.30751793 |
| 1 | 2.27515844 | -1.49992982 | 2.61482083 |
| 1 | 0.57323449 | -2.95660547 | 3.71743998 |
| 1 | 1.76967292 | -3.97104905 | 2.87070853 |
| 1 | 0.16106988 | -3.69509648 | 2.15216252 |
| 1 | 5.93862969 | -4.15650765 | 1.58460218 |
| 1 | 5.59848450 | -3.54925013 | -0.06370013 |
| 1 | 4.68039801 | -4.93532288 | 0.58269385 |

Electronic energy = -3673.043549 a.u.
DFT-D3(BJ) dispersion correction = -0.116128 a.u.
Thermal free energy = 0.582145 a.u.
Gibbs free energy = -3672.577531 a.u.
Number of imaginary frequencies = 0.

**Int2 LA isomer/conformer 44**

| Atomic N. | X | Y | Z |
|---|---|---|---|
| 7 | 0.86101651 | 1.14330278 | -2.32210036 |
| 8 | 0.63883826 | -0.95882358 | -0.15185280 |
| 8 | 1.53950067 | 1.79876943 | 0.49189825 |
| 6 | -0.16827988 | 1.27025603 | -3.38110280 |
| 6 | 0.63364463 | -1.81990953 | -1.19778309 |
| 6 | 0.22410181 | -3.17249115 | -1.01369234 |
| 6 | 0.20951937 | -3.99805370 | -2.14726621 |
| 6 | 0.59484164 | -3.55474725 | -3.41594403 |
| 6 | 1.05034593 | -2.24952692 | -3.56977447 |
| 6 | 1.09109802 | -1.38585586 | -2.47002976 |
| 6 | 1.75517414 | -0.04242354 | -2.60153007 |
| 1 | 0.55728538 | -4.23401974 | -4.26769175 |
| 6 | -0.16340758 | -3.71677681 | 0.37526446 |
| 6 | 2.74542163 | 2.10001809 | -0.01035479 |
| 6 | 3.88835043 | 2.16762019 | 0.84562156 |
| 6 | 5.11554821 | 2.50037530 | 0.25765436 |
| 6 | 5.25936632 | 2.76167011 | -1.10967510 |
| 6 | 4.14062616 | 2.70211470 | -1.93258644 |
| 6 | 2.88741574 | 2.38733466 | -1.39352320 |
| 6 | 1.67336038 | 2.42378059 | -2.27899760 |
| 1 | 6.23838360 | 3.00897583 | -1.51999974 |
| 6 | 3.77415656 | 1.88196689 | 2.35512753 |
| 6 | -0.52404744 | -5.21354880 | 0.32595825 |
| 6 | -1.39254117 | -2.96466444 | 0.93172499 |
| 6 | 1.03178849 | -3.56135486 | 1.34627523 |
| 6 | 5.12481710 | 2.04756895 | 3.07655516 |
| 6 | 3.30381398 | 0.42581695 | 2.58391671 |
| 6 | 2.77696393 | 2.86802092 | 3.00948766 |
| 1 | 0.32307002 | 1.45305410 | -4.35077264 |
| 1 | -0.76015205 | 0.35280149 | -3.43465521 |
| 1 | 2.17210919 | 0.07922447 | -3.61546627 |
| 1 | 2.58618213 | 0.04089162 | -1.88656717 |
| 1 | -0.11700987 | -5.03121172 | -2.04231340 |
| 1 | 1.40368823 | -1.89586222 | -4.54140418 |
| 1 | 0.96802975 | 3.20478487 | -1.94960538 |

| | | | |
|---|---|---|---|
| 1 | 1.98049904 | 2.65866694 | -3.31171126 |
| 1 | 6.00353727 | 2.55364778 | 0.88539127 |
| 1 | 4.22680356 | 2.91093260 | -3.00170474 |
| 1 | -0.77109764 | -5.55372650 | 1.34181642 |
| 1 | -1.40083864 | -5.40702845 | -0.30932486 |
| 1 | 0.31169483 | -5.83015769 | -0.03574237 |
| 1 | -1.18348652 | -1.89925450 | 1.06963945 |
| 1 | -2.25412704 | -3.06297587 | 0.25708078 |
| 1 | -1.67849527 | -3.38585670 | 1.90749735 |
| 1 | 1.90109576 | -4.13102783 | 0.98635101 |
| 1 | 1.32462790 | -2.51136776 | 1.45711350 |
| 1 | 0.75788833 | -3.95275384 | 2.33808810 |
| 1 | 4.98479213 | 1.84225854 | 4.14754598 |
| 1 | 5.52068034 | 3.06954424 | 2.98409720 |
| 1 | 5.88375817 | 1.34496295 | 2.70261593 |
| 1 | 2.32528252 | 0.24073002 | 2.12862585 |
| 1 | 4.02514303 | -0.28561325 | 2.15599211 |
| 1 | 3.22904726 | 0.22444832 | 3.66364575 |
| 1 | 3.12501775 | 3.90482989 | 2.89020942 |
| 1 | 1.77877073 | 2.78088454 | 2.56711649 |
| 1 | 2.70297275 | 2.65896504 | 4.08787644 |
| 1 | -0.82810405 | 2.11581503 | -3.15084818 |
| 8 | -5.89364733 | 0.28392369 | 2.20574944 |
| 6 | -5.86885419 | -0.23919476 | 3.56194130 |
| 1 | -6.10068299 | -1.31095739 | 3.55845284 |
| 8 | -4.20021545 | -1.14880595 | 1.67249297 |
| 8 | -1.17362797 | 1.76314450 | 0.47541816 |
| 1 | -6.63505891 | 0.32504883 | 4.10062179 |
| 1 | -4.87941163 | -0.08284566 | 4.00779746 |
| 31 | 0.17689982 | 0.83478932 | -0.38471574 |
| 6 | -4.99319350 | -0.28707899 | 1.36522411 |
| 6 | -5.16072211 | 0.32241468 | -0.03560128 |
| 8 | -4.05192700 | -0.05339124 | -0.88716794 |
| 6 | -2.80941907 | 0.44882095 | -0.70777137 |
| 6 | -2.53309159 | 1.64675441 | 0.20580076 |
| 8 | -1.89785892 | -0.04444940 | -1.36014936 |
| 6 | -3.04369853 | 2.94004645 | -0.47195903 |
| 6 | -6.41771747 | -0.21155798 | -0.71840600 |
| 1 | -5.22516626 | 1.41391508 | 0.06590447 |
| 1 | -3.07758249 | 1.50093204 | 1.15881316 |
| 1 | -2.78575934 | 3.78387190 | 0.17924640 |
| 1 | -4.12855102 | 2.95120176 | -0.64799161 |
| 1 | -2.53341778 | 3.07995397 | -1.43447039 |
| 1 | -7.30275739 | 0.04004602 | -0.12191152 |
| 1 | -6.35430479 | -1.30128880 | -0.83033215 |
| 1 | -6.51871653 | 0.23970518 | -1.71315348 |

Electronic energy = -3673.042538 a.u.
DFT-D3(BJ) dispersion correction = -0.115328 a.u.
Thermal free energy = 0.580626 a.u.
Gibbs free energy = -3672.577240 a.u.
Number of imaginary frequencies = 0.

**Int2 LA isomer/conformer 45**

| Atomic N. | X | Y | Z |
|---|---|---|---|
| 7 | 2.01380383 | -1.76492538 | -0.19362029 |
| 8 | 0.20121684 | 0.37474567 | 0.96353277 |
| 8 | 1.66872194 | 0.84062784 | -1.57075900 |
| 6 | 1.61989322 | -3.19395497 | -0.18216170 |
| 6 | 0.32954773 | -0.34981959 | 2.10041837 |
| 6 | -0.55950847 | -0.13579987 | 3.19360974 |
| 6 | -0.39497043 | -0.94646396 | 4.32604302 |
| 6 | 0.60336258 | -1.91970460 | 4.42441509 |
| 6 | 1.50336900 | -2.07647038 | 3.37548702 |
| 6 | 1.38759932 | -1.28911003 | 2.22516658 |
| 6 | 2.46960633 | -1.33474586 | 1.18065209 |
| 1 | 0.68511881 | -2.53118112 | 5.32300993 |
| 6 | -1.64151103 | 0.96097863 | 3.15056892 |
| 6 | 2.99693610 | 0.94217396 | -1.42145270 |
| 6 | 3.63889864 | 2.21611531 | -1.51531669 |
| 6 | 5.03084304 | 2.25018408 | -1.36183191 |
| 6 | 5.79924322 | 1.10517742 | -1.12312169 |
| 6 | 5.16739268 | -0.13028835 | -1.04211451 |
| 6 | 3.77940772 | -0.22220239 | -1.20161016 |
| 6 | 3.12672836 | -1.57602623 | -1.20747317 |
| 1 | 6.87960439 | 1.18564808 | -1.00357585 |
| 6 | 2.82952158 | 3.50128827 | -1.77343453 |
| 6 | -2.44563818 | 1.02952198 | 4.46258376 |
| 6 | -2.64803249 | 0.68925834 | 2.00942067 |
| 6 | -0.97573128 | 2.34236344 | 2.94244303 |
| 6 | 3.73480080 | 4.74389433 | -1.86546733 |
| 6 | 1.83285623 | 3.74102152 | -0.61450088 |
| 6 | 2.06380603 | 3.38705174 | -3.11341435 |
| 1 | 2.49299779 | -3.81377235 | 0.07947313 |
| 1 | 0.82230118 | -3.35316619 | 0.54826432 |
| 1 | 3.27749142 | -2.01486620 | 1.49897549 |
| 1 | 2.90848310 | -0.33572994 | 1.04613210 |
| 1 | -1.07143242 | -0.81905270 | 5.16933456 |
| 1 | 2.32227338 | -2.79596220 | 3.45052408 |
| 1 | 2.66688459 | -1.78892022 | -2.18683738 |
| 1 | 3.88795047 | -2.35028197 | -1.01533473 |
| 1 | 5.54459199 | 3.20812770 | -1.42351369 |
| 1 | 5.74759429 | -1.03922242 | -0.86542193 |
| 1 | -3.18615678 | 1.83873713 | 4.38609266 |
| 1 | -2.99348360 | 0.09699365 | 4.66210251 |
| 1 | -1.80527364 | 1.24911872 | 5.32881694 |
| 1 | -2.15447557 | 0.70142918 | 1.03177292 |
| 1 | -3.13437486 | -0.28773327 | 2.14464929 |
| 1 | -3.42840974 | 1.46660704 | 2.01805462 |
| 1 | -0.29120055 | 2.57019348 | 3.77233049 |
| 1 | -0.40992587 | 2.37817546 | 2.00489396 |
| 1 | -1.74653822 | 3.12809345 | 2.91559330 |
| 1 | 3.10963122 | 5.62742735 | -2.05863143 |
| 1 | 4.46141345 | 4.66636016 | -2.68741570 |
| 1 | 4.28461402 | 4.92683279 | -0.93074370 |
| 1 | 1.12771705 | 2.90973254 | -0.51002356 |

| | | | |
|---|---|---|---|
| 1 | 2.36910027 | 3.86251857 | 0.33804222 |
| 1 | 1.26048744 | 4.66279066 | -0.80099998 |
| 1 | 2.76694436 | 3.26084262 | -3.94999908 |
| 1 | 1.37085453 | 2.53880451 | -3.10807873 |
| 1 | 1.48941253 | 4.30919771 | -3.29187981 |
| 1 | 1.26324764 | -3.48245969 | -1.17861841 |
| 8 | -4.68528190 | -0.41465127 | -0.84195372 |
| 6 | -5.54036527 | 0.65775578 | -0.36175549 |
| 1 | -6.39055045 | 0.79298499 | -1.04106259 |
| 8 | -6.46112186 | -1.83579816 | -0.72954884 |
| 8 | -0.61141143 | -0.51625365 | -2.15133129 |
| 1 | -4.90238680 | 1.54510489 | -0.34420069 |
| 1 | -5.90732651 | 0.42144938 | 0.64382836 |
| 31 | 0.53918207 | -0.39826288 | -0.70711291 |
| 6 | -5.28983598 | -1.61705927 | -0.96120715 |
| 6 | -4.29386734 | -2.66862178 | -1.48815482 |
| 8 | -3.12376560 | -2.69902521 | -0.61222562 |
| 6 | -1.97873557 | -2.04332109 | -0.88915035 |
| 6 | -1.70896031 | -1.36947173 | -2.23841436 |
| 8 | -1.11010896 | -2.03981558 | -0.02259976 |
| 6 | -1.45144778 | -2.44761393 | -3.31588234 |
| 6 | -4.89129284 | -4.06181517 | -1.50222104 |
| 1 | -4.00070027 | -2.36006465 | -2.50058456 |
| 1 | -2.60092940 | -0.77803378 | -2.51793660 |
| 1 | -1.20647472 | -1.93440372 | -4.25366735 |
| 1 | -2.31113630 | -3.10992835 | -3.49186968 |
| 1 | -0.59008059 | -3.06506388 | -3.02729610 |
| 1 | -5.80337878 | -4.06740471 | -2.11080108 |
| 1 | -5.15726384 | -4.37551659 | -0.48591833 |
| 1 | -4.17236556 | -4.77534311 | -1.92398442 |

Electronic energy = -3673.043158 a.u.
DFT-D3(BJ) dispersion correction = -0.115951 a.u.
Thermal free energy = 0.582933 a.u.
Gibbs free energy = -3672.576176 a.u.
Number of imaginary frequencies = 0.

**Int2 LA isomer/conformer 46**

| Atomic N. | X | Y | Z |
|---|---|---|---|
| 8 | -1.86661563 | -0.72727112 | 5.41727726 |
| 6 | -1.94484564 | -1.41867095 | 4.14685694 |
| 1 | -1.92932982 | -0.73036884 | 3.29512092 |
| 8 | -1.17351215 | 0.95690456 | 6.67802500 |
| 8 | 1.80708837 | -1.84872724 | 0.97625314 |
| 1 | -2.90684501 | -1.94045726 | 4.16749548 |
| 1 | -1.13426682 | -2.15352350 | 4.06465201 |
| 7 | 1.30276255 | -1.89615217 | -1.89630748 |
| 8 | 0.95838835 | 0.82190957 | -0.75701005 |
| 8 | -1.13780289 | -1.51981756 | -0.22886331 |
| 6 | 2.70136305 | -2.38330254 | -1.80676380 |
| 6 | 1.81332282 | 1.16461260 | -1.74775588 |
| 6 | 2.51226291 | 2.40789027 | -1.67744439 |
| 6 | 3.38828649 | 2.71758944 | -2.72735444 |

| | | | |
|---|---|---|---|
| 6 | 3.59106181 | 1.87031684 | -3.82010307 |
| 6 | 2.88608715 | 0.67364406 | -3.88953642 |
| 6 | 1.99114707 | 0.32011306 | -2.87398761 |
| 6 | 1.15042334 | -0.91789409 | -3.02543339 |
| 1 | 4.28740145 | 2.15186457 | -4.60991451 |
| 6 | 2.30860093 | 3.38364164 | -0.50102243 |
| 6 | -1.76645228 | -1.96813352 | -1.33795271 |
| 6 | -3.15827217 | -1.70732538 | -1.52153220 |
| 6 | -3.76170856 | -2.20737308 | -2.68363498 |
| 6 | -3.06081006 | -2.93709254 | -3.64812930 |
| 6 | -1.70768441 | -3.19106465 | -3.45505188 |
| 6 | -1.05830630 | -2.72402200 | -2.30656369 |
| 6 | 0.38679732 | -3.07784289 | -2.08172045 |
| 1 | -3.57262192 | -3.30008122 | -4.53927290 |
| 6 | -3.97313237 | -0.91376739 | -0.48168781 |
| 6 | 3.14066747 | 4.66949202 | -0.66356578 |
| 6 | 2.74675112 | 2.71708022 | 0.82400579 |
| 6 | 0.82218018 | 3.80440004 | -0.41148458 |
| 6 | -5.45034838 | -0.76624562 | -0.89170995 |
| 6 | -3.39635153 | 0.51280758 | -0.32114440 |
| 6 | -3.94428208 | -1.65512389 | 0.87583476 |
| 1 | 2.97823303 | -2.89718371 | -2.74273282 |
| 1 | 3.37711610 | -1.53645449 | -1.64892815 |
| 1 | 1.40116741 | -1.42996726 | -3.97058292 |
| 1 | 0.08208409 | -0.65833378 | -3.06422452 |
| 1 | 3.93969232 | 3.65556804 | -2.69590213 |
| 1 | 3.01070893 | 0.00613322 | -4.74532096 |
| 1 | 0.50696844 | -3.68878438 | -1.17242968 |
| 1 | 0.75653251 | -3.66900841 | -2.93703926 |
| 1 | -4.82046916 | -2.01857248 | -2.85173841 |
| 1 | -1.14118547 | -3.76512391 | -4.19186771 |
| 1 | 2.94963049 | 5.33006686 | 0.19435659 |
| 1 | 4.22085088 | 4.46517046 | -0.68934382 |
| 1 | 2.86975376 | 5.22345731 | -1.57370267 |
| 1 | 2.15691848 | 1.81524903 | 1.01933945 |
| 1 | 3.81113689 | 2.44201262 | 0.78773731 |
| 1 | 2.60728853 | 3.41930424 | 1.66083150 |
| 1 | 0.51101734 | 4.31734470 | -1.33300417 |
| 1 | 0.16870007 | 2.93862563 | -0.25835189 |
| 1 | 0.68296969 | 4.50537589 | 0.42633796 |
| 1 | -5.98314684 | -0.20262874 | -0.11242106 |
| 1 | -5.95091628 | -1.73964032 | -0.99610658 |
| 1 | -5.56422984 | -0.21312029 | -1.83510873 |
| 1 | -2.35618028 | 0.49364156 | 0.02033362 |
| 1 | -3.43721656 | 1.05578488 | -1.27638787 |
| 1 | -3.99573473 | 1.07563391 | 0.41131631 |
| 1 | -4.41826458 | -2.64352491 | 0.78608508 |
| 1 | -2.91477969 | -1.79344640 | 1.22453503 |
| 1 | -4.50406052 | -1.07895660 | 1.62924487 |
| 1 | 2.78769096 | -3.06947482 | -0.95850311 |
| 31 | 0.64051432 | -0.92527437 | -0.15508153 |
| 6 | -1.08316631 | 0.35528494 | 5.62785045 |
| 6 | -0.10021904 | 0.89557026 | 4.56508147 |

| | | | |
|---|---|---|---|
| 8 | 0.69490549 | -0.20184381 | 3.96358528 |
| 6 | 0.77259712 | -0.48945711 | 2.66562866 |
| 6 | 1.82767376 | -1.55069266 | 2.33901578 |
| 8 | 0.07233452 | 0.00225886 | 1.77670225 |
| 6 | 3.22370015 | -1.09614170 | 2.79987342 |
| 6 | -0.76511160 | 1.91770931 | 3.64554685 |
| 1 | 0.65861086 | 1.41034302 | 5.16648701 |
| 1 | 1.54142717 | -2.44522840 | 2.93297024 |
| 1 | 3.94359518 | -1.88505726 | 2.55013205 |
| 1 | 3.25136871 | -0.91960225 | 3.88304450 |
| 1 | 3.51903548 | -0.17901544 | 2.27261130 |
| 1 | -1.15852560 | 2.70637640 | 4.30249781 |
| 1 | -1.58754629 | 1.50815927 | 3.05222918 |
| 1 | -0.03487423 | 2.36945407 | 2.96483014 |

Electronic energy = -3673.042194 a.u.
DFT-D3(BJ) dispersion correction = -0.117392 a.u.
Thermal free energy = 0.583741 a.u.
Gibbs free energy = -3672.575845 a.u.
Number of imaginary frequencies = 0.

**Int2 LA isomer/conformer 47**

| Atomic N. | X | Y | Z |
|---|---|---|---|
| 7 | -0.27965093 | 1.43544350 | 2.55450754 |
| 8 | -0.18549313 | 1.38874973 | -0.44091761 |
| 8 | 1.49474289 | -0.62948402 | 1.28360412 |
| 6 | -1.61147635 | 1.41155751 | 3.20912828 |
| 6 | -0.91054467 | 2.53406892 | -0.31299361 |
| 6 | -1.66315249 | 3.02862596 | -1.41639794 |
| 6 | -2.38414929 | 4.21549564 | -1.21829356 |
| 6 | -2.38388519 | 4.91153211 | -0.00639201 |
| 6 | -1.62558139 | 4.42981685 | 1.05499570 |
| 6 | -0.87813206 | 3.25585745 | 0.90846493 |
| 6 | 0.03603974 | 2.81028539 | 2.01808412 |
| 1 | -2.96594076 | 5.82698233 | 0.09838324 |
| 6 | -1.68678116 | 2.29901819 | -2.77393506 |
| 6 | 2.48755535 | 0.07920609 | 1.88638815 |
| 6 | 3.83387352 | -0.06547917 | 1.44624261 |
| 6 | 4.80756601 | 0.69026701 | 2.11530096 |
| 6 | 4.50639046 | 1.55430224 | 3.17229616 |
| 6 | 3.19022160 | 1.67075590 | 3.60332966 |
| 6 | 2.17922877 | 0.93071507 | 2.97803260 |
| 6 | 0.78025137 | 0.97885185 | 3.53346863 |
| 1 | 5.29990769 | 2.12447030 | 3.65499700 |
| 6 | 4.21180357 | -1.01434434 | 0.29191783 |
| 6 | -2.53402290 | 3.05164000 | -3.81723063 |
| 6 | -2.30487748 | 0.89089944 | -2.60929883 |
| 6 | -0.25164094 | 2.18611541 | -3.34155170 |
| 6 | 5.72823751 | -1.00936525 | 0.02046119 |
| 6 | 3.51260347 | -0.57239366 | -1.01547355 |
| 6 | 3.81051306 | -2.46623407 | 0.64598782 |
| 1 | -1.61516926 | 2.09073031 | 4.07677327 |
| 1 | -2.37750771 | 1.73181475 | 2.49500113 |

| | | | |
|---|---|---|---|
| 1 | -0.00627858 | 3.52446313 | 2.85731481 |
| 1 | 1.07798668 | 2.76124552 | 1.66885059 |
| 1 | -2.97588783 | 4.61534054 | -2.03959061 |
| 1 | -1.58988201 | 4.97195358 | 2.00264443 |
| 1 | 0.46253308 | -0.02224634 | 3.86660578 |
| 1 | 0.74826280 | 1.65145867 | 4.40651736 |
| 1 | 5.84577684 | 0.60849083 | 1.79927101 |
| 1 | 2.93602460 | 2.32539890 | 4.43990244 |
| 1 | -2.50555750 | 2.49729285 | -4.76599442 |
| 1 | -3.58821890 | 3.13408358 | -3.51541707 |
| 1 | -2.14712248 | 4.06204746 | -4.01311743 |
| 1 | -1.72762267 | 0.27678820 | -1.91028814 |
| 1 | -3.33750413 | 0.96053955 | -2.23789249 |
| 1 | -2.32887724 | 0.37935226 | -3.58345597 |
| 1 | 0.18010353 | 3.18476240 | -3.50181833 |
| 1 | 0.40750558 | 1.62716494 | -2.66866334 |
| 1 | -0.27703571 | 1.67041957 | -4.31348211 |
| 1 | 5.94402860 | -1.70671556 | -0.80117072 |
| 1 | 6.30574601 | -1.34216028 | 0.89494981 |
| 1 | 6.09392121 | -0.01760955 | -0.28347247 |
| 1 | 2.42195357 | -0.60797077 | -0.92411975 |
| 1 | 3.80500259 | 0.45192972 | -1.28859097 |
| 1 | 3.81041006 | -1.24080467 | -1.83722995 |
| 1 | 4.33917183 | -2.80427244 | 1.54892071 |
| 1 | 2.73422522 | -2.56734311 | 0.81921242 |
| 1 | 4.09094090 | -3.13847114 | -0.17875262 |
| 1 | -1.83566233 | 0.39226936 | 3.54493735 |
| 8 | -3.18092559 | -3.73035367 | -2.62837245 |
| 6 | -4.31170099 | -4.59381688 | -2.90551978 |
| 1 | -5.01797668 | -4.57928077 | -2.06600426 |
| 8 | -2.72577053 | -5.02663843 | -0.81349198 |
| 8 | -1.51709423 | -1.03135797 | 0.83191465 |
| 1 | -4.77250229 | -4.18429789 | -3.80921581 |
| 1 | -3.97309806 | -5.62372154 | -3.07181560 |
| 31 | -0.14035196 | 0.16577539 | 0.94938748 |
| 6 | -2.46443507 | -4.07822680 | -1.52319280 |
| 6 | -1.33568436 | -3.06551630 | -1.28949906 |
| 8 | -0.44643910 | -3.58511086 | -0.27642594 |
| 6 | -0.61445297 | -3.37381326 | 1.06473713 |
| 6 | -1.51511883 | -2.23370843 | 1.60774865 |
| 8 | 0.00653356 | -4.07113534 | 1.83837263 |
| 6 | -2.95655759 | -2.70973691 | 1.80751758 |
| 6 | -0.48843932 | -2.80931435 | -2.53284273 |
| 1 | -1.79913053 | -2.12834582 | -0.94573927 |
| 1 | -1.07570144 | -2.05981383 | 2.60577800 |
| 1 | -3.51659496 | -1.94120438 | 2.35759932 |
| 1 | -2.97556726 | -3.64615560 | 2.38039230 |
| 1 | -3.45874966 | -2.88064416 | 0.84795689 |
| 1 | -1.11585740 | -2.44401741 | -3.35355092 |
| 1 | 0.01509028 | -3.73192450 | -2.84993831 |
| 1 | 0.27565199 | -2.05494892 | -2.30674648 |

Electronic energy = -3673.039253 a.u.
DFT-D3(BJ) dispersion correction = -0.115531 a.u.

Thermal free energy = 0.579038 a.u.
Gibbs free energy = -3672.575745 a.u.
Number of imaginary frequencies = 0.

**Int2_LA isomer/conformer 48**

| Atomic N. | X | Y | Z |
|---|---|---|---|
| 7 | -1.50944093 | -1.42068779 | -1.24393597 |
| 8 | 0.87211197 | -0.47573348 | 0.37951612 |
| 8 | 0.68508759 | 0.06569538 | -2.59627583 |
| 6 | -2.89804749 | -1.27827599 | -0.73576260 |
| 6 | 0.40433086 | -1.36080601 | 1.29416667 |
| 6 | 0.86713042 | -1.30799601 | 2.64066324 |
| 6 | 0.31280702 | -2.22413215 | 3.54686435 |
| 6 | -0.64078870 | -3.17552406 | 3.17512614 |
| 6 | -1.04000472 | -3.25380959 | 1.84492763 |
| 6 | -0.51285886 | -2.36954688 | 0.89828588 |
| 6 | -0.81404289 | -2.57629244 | -0.56231212 |
| 1 | -1.05211915 | -3.85763372 | 3.91890161 |
| 6 | 1.95442624 | -0.30785645 | 3.08340966 |
| 6 | 0.81869670 | -1.06041389 | -3.32098778 |
| 6 | 2.02635165 | -1.31953341 | -4.03694168 |
| 6 | 2.09050792 | -2.50601577 | -4.77992032 |
| 6 | 1.03615518 | -3.42361435 | -4.84137808 |
| 6 | -0.13910930 | -3.15543555 | -4.14901436 |
| 6 | -0.26017780 | -1.97758370 | -3.40200532 |
| 6 | -1.57075926 | -1.64966209 | -2.74336790 |
| 1 | 1.13865821 | -4.33624677 | -5.42834601 |
| 6 | 3.21182064 | -0.33639546 | -3.99327039 |
| 6 | 2.32159917 | -0.47541197 | 4.56984527 |
| 6 | 1.47005160 | 1.14806152 | 2.89268084 |
| 6 | 3.24376359 | -0.53898206 | 2.25930231 |
| 6 | 4.38663056 | -0.81188191 | -4.86844555 |
| 6 | 3.73908536 | -0.20627549 | -2.54451246 |
| 6 | 2.77255584 | 1.04988534 | -4.52225714 |
| 1 | -3.48151227 | -2.16808382 | -1.02192581 |
| 1 | -2.88810036 | -1.17788968 | 0.35173302 |
| 1 | -1.43920414 | -3.47460930 | -0.69721938 |
| 1 | 0.11902365 | -2.73233382 | -1.12321048 |
| 1 | 0.63300749 | -2.19852311 | 4.58697838 |
| 1 | -1.75176813 | -4.01806719 | 1.52465796 |
| 1 | -2.00159285 | -0.72503717 | -3.16095092 |
| 1 | -2.28706597 | -2.46786925 | -2.92376603 |
| 1 | 3.00090017 | -2.73377788 | -5.33172619 |
| 1 | -0.97969557 | -3.85204436 | -4.19175605 |
| 1 | 3.11039584 | 0.24590825 | 4.82717920 |
| 1 | 1.46606699 | -0.28200743 | 5.23359497 |
| 1 | 2.70912782 | -1.48068440 | 4.78805637 |
| 1 | 1.25181887 | 1.35592368 | 1.83964394 |
| 1 | 0.56550896 | 1.33522314 | 3.49107758 |
| 1 | 2.24875850 | 1.84911908 | 3.22971743 |
| 1 | 3.62699890 | -1.55674268 | 2.42131647 |
| 1 | 3.06577787 | -0.40104041 | 1.18708858 |

| | | | |
|---|---|---|---|
| 1 | 4.02316132 | 0.17000403 | 2.57812350 |
| 1 | 5.19767062 | -0.07203020 | -4.80963177 |
| 1 | 4.10192116 | -0.90748472 | -5.92639340 |
| 1 | 4.79391892 | -1.77531189 | -4.52883525 |
| 1 | 2.96675424 | 0.17058076 | -1.86576663 |
| 1 | 4.08565162 | -1.18018854 | -2.16918281 |
| 1 | 4.59196332 | 0.48927818 | -2.52053610 |
| 1 | 2.43631120 | 0.97561327 | -5.56698521 |
| 1 | 1.95767342 | 1.46878771 | -3.92209260 |
| 1 | 3.62510678 | 1.74562414 | -4.49102382 |
| 1 | -3.35872538 | -0.38604124 | -1.17588359 |
| 8 | -5.05872932 | 2.36189980 | 2.26092034 |
| 6 | -4.92857718 | 3.80004325 | 2.14793963 |
| 1 | -5.28116243 | 4.27128633 | 3.07606793 |
| 8 | -4.52989072 | 0.50292399 | 3.35260230 |
| 8 | -0.67162098 | 1.96689199 | -1.09996128 |
| 1 | -5.59346294 | 4.08547726 | 1.32622543 |
| 1 | -3.90544631 | 4.11428251 | 1.92240069 |
| 31 | -0.23583786 | 0.18566790 | -0.96951982 |
| 6 | -4.17574991 | 1.59198262 | 2.95740043 |
| 6 | -2.73242277 | 2.06655837 | 3.23646569 |
| 8 | -2.11859114 | 2.78565086 | 2.11532684 |
| 6 | -1.69701550 | 2.01429131 | 1.08375874 |
| 6 | -0.98793509 | 2.79669013 | -0.02139248 |
| 8 | -1.84029215 | 0.80170726 | 1.04714196 |
| 6 | -1.82954193 | 3.98172757 | -0.51569200 |
| 6 | -2.62422812 | 2.95348609 | 4.47020277 |
| 1 | -2.17339957 | 1.13465887 | 3.38262801 |
| 1 | -0.06730921 | 3.20105039 | 0.45403662 |
| 1 | -1.27919220 | 4.48352766 | -1.32054591 |
| 1 | -2.01597146 | 4.70393058 | 0.28970359 |
| 1 | -2.78708428 | 3.62832201 | -0.92201046 |
| 1 | -3.06018735 | 2.42792486 | 5.32982552 |
| 1 | -3.14337814 | 3.91115372 | 4.34254424 |
| 1 | -1.56724785 | 3.15467426 | 4.68669363 |

Electronic energy = -3673.039872 a.u.
DFT-D3(BJ) dispersion correction = -0.116521 a.u.
Thermal free energy = 0.580836 a.u.
Gibbs free energy = -3672.575557 a.u.
Number of imaginary frequencies = 0.

**Int2 LA isomer/conformer 49**

| Atomic N. | X | Y | Z |
|---|---|---|---|
| 8 | -2.17541647 | 4.25431464 | 5.40431442 |
| 6 | -0.94528594 | 4.88860008 | 4.97907728 |
| 1 | -0.05786038 | 4.36239971 | 5.34787648 |
| 8 | -3.40265696 | 2.48026039 | 5.89970984 |
| 8 | 1.12662544 | 1.22980816 | 0.82818013 |
| 1 | -0.98499365 | 5.89193284 | 5.41635536 |
| 1 | -0.90592910 | 4.95822677 | 3.88635808 |
| 7 | 0.82745225 | -2.07042973 | 0.82844160 |
| 8 | 1.29767655 | -0.44255729 | -1.61665947 |

| | | | |
|---|---|---|---|
| 8 | -1.43548134 | -0.33548585 | -0.18090059 |
| 6 | 1.78750145 | -2.00980159 | 1.96050765 |
| 6 | 2.47212245 | -1.12145876 | -1.65798492 |
| 6 | 3.56748540 | -0.62919365 | -2.42457464 |
| 6 | 4.74586358 | -1.39028958 | -2.41711078 |
| 6 | 4.87455867 | -2.58661549 | -1.70503503 |
| 6 | 3.78898346 | -3.06861825 | -0.98078001 |
| 6 | 2.58688550 | -2.35362959 | -0.96340263 |
| 6 | 1.37145164 | -2.92768748 | -0.28961335 |
| 1 | 5.81475354 | -3.13758663 | -1.72803087 |
| 6 | 3.46627728 | 0.68101489 | -3.22945170 |
| 6 | -2.02209094 | -1.53899207 | -0.42706682 |
| 6 | -3.10944597 | -1.62569675 | -1.34411597 |
| 6 | -3.67803073 | -2.89137404 | -1.54780415 |
| 6 | -3.22219231 | -4.04130127 | -0.89817452 |
| 6 | -2.16743363 | -3.94028449 | 0.00143641 |
| 6 | -1.57036175 | -2.69942870 | 0.25438417 |
| 6 | -0.50211859 | -2.60558255 | 1.31501824 |
| 1 | -3.69254828 | -5.00438905 | -1.09584486 |
| 6 | -3.64962057 | -0.38526890 | -2.08360765 |
| 6 | 4.75729305 | 0.97373928 | -4.01700147 |
| 6 | 3.22736831 | 1.87387710 | -2.27388551 |
| 6 | 2.31267649 | 0.58565503 | -4.25638882 |
| 6 | -4.83028113 | -0.73530257 | -3.00944528 |
| 6 | -2.54467159 | 0.23706424 | -2.96941343 |
| 6 | -4.16378286 | 0.65518068 | -1.06076225 |
| 1 | 1.93819275 | -3.02078792 | 2.37238299 |
| 1 | 2.74657577 | -1.61948045 | 1.60281745 |
| 1 | 1.59551066 | -3.92437505 | 0.12572973 |
| 1 | 0.54758111 | -3.03635411 | -1.00977518 |
| 1 | 5.60430895 | -1.03727395 | -2.98585170 |
| 1 | 3.85826599 | -4.01485886 | -0.43910109 |
| 1 | -0.81116293 | -1.92792853 | 2.12329538 |
| 1 | -0.32383122 | -3.60400804 | 1.74751648 |
| 1 | -4.50657383 | -2.99069971 | -2.24638671 |
| 1 | -1.80376791 | -4.82447595 | 0.52972194 |
| 1 | 4.62585262 | 1.90716276 | -4.58268911 |
| 1 | 5.62576352 | 1.10872704 | -3.35599222 |
| 1 | 4.98994135 | 0.17850965 | -4.74006873 |
| 1 | 2.30460958 | 1.75552121 | -1.69596900 |
| 1 | 4.06387319 | 1.97672530 | -1.56731632 |
| 1 | 3.15811175 | 2.80624967 | -2.85528701 |
| 1 | 2.49367713 | -0.23475322 | -4.96625749 |
| 1 | 1.34877565 | 0.41446351 | -3.76528440 |
| 1 | 2.25079292 | 1.52195698 | -4.83181911 |
| 1 | -5.18127250 | 0.18313154 | -3.50103172 |
| 1 | -5.68050255 | -1.15849612 | -2.45537577 |
| 1 | -4.54147770 | -1.44282102 | -3.80006014 |
| 1 | -1.68633691 | 0.57100318 | -2.37759038 |
| 1 | -2.18781100 | -0.49017099 | -3.71280910 |
| 1 | -2.94973667 | 1.10472987 | -3.51195935 |
| 1 | -4.99339716 | 0.24030653 | -0.47015445 |
| 1 | -3.37173913 | 0.96607631 | -0.37115430 |

| | | | |
|---|---|---|---|
| 1 | -4.53688808 | 1.54549939 | -1.58935206 |
| 1 | 1.38578843 | -1.34747880 | 2.73425421 |
| 31 | 0.40859125 | -0.24163759 | 0.01037585 |
| 6 | -2.37305483 | 2.91044733 | 5.42303725 |
| 6 | -1.31762042 | 1.90743499 | 4.92583219 |
| 8 | -0.61813766 | 2.41475335 | 3.74666488 |
| 6 | -0.24287711 | 1.48586446 | 2.82340193 |
| 6 | 0.40467053 | 2.15029942 | 1.60178852 |
| 8 | -0.41039839 | 0.28605539 | 2.95797563 |
| 6 | -0.65816270 | 2.91946141 | 0.79611364 |
| 6 | -0.33540082 | 1.53016325 | 6.03506689 |
| 1 | -1.88470287 | 1.01978228 | 4.62290379 |
| 1 | 1.13300356 | 2.88047371 | 2.00117837 |
| 1 | -0.16748981 | 3.39707478 | -0.06138344 |
| 1 | -1.14034109 | 3.69508816 | 1.40610106 |
| 1 | -1.43000612 | 2.23187714 | 0.42322102 |
| 1 | -0.89823094 | 1.16760175 | 6.90515201 |
| 1 | 0.27988407 | 2.38381596 | 6.34673199 |
| 1 | 0.32237671 | 0.72424451 | 5.68680065 |

Electronic energy = -3673.042907 a.u.
DFT-D3(BJ) dispersion correction = -0.114164 a.u.
Thermal free energy = 0.581636 a.u.
Gibbs free energy = -3672.575435 a.u.
Number of imaginary frequencies = 0.

**Int2 LA isomer/conformer 50**

| Atomic N. | X | Y | Z |
|---|---|---|---|
| 7 | 0.17487967 | 0.95755747 | -1.85969934 |
| 8 | 0.17643001 | 1.31654227 | 1.10524625 |
| 8 | -2.04146850 | -0.37347723 | -0.29749129 |
| 6 | 1.42004840 | 0.50074131 | -2.53172259 |
| 6 | 1.19223098 | 2.18069099 | 0.83021837 |
| 6 | 2.10769436 | 2.55838501 | 1.85328803 |
| 6 | 3.12086802 | 3.46257431 | 1.50082136 |
| 6 | 3.25535178 | 3.98938107 | 0.21311996 |
| 6 | 2.34463783 | 3.62295982 | -0.77208102 |
| 6 | 1.30725203 | 2.73346762 | -0.47145060 |
| 6 | 0.25915403 | 2.42334275 | -1.50600210 |
| 1 | 4.06394240 | 4.68506642 | -0.01077615 |
| 6 | 1.99670057 | 2.00140827 | 3.28608287 |
| 6 | -2.82768976 | 0.50310688 | -0.98202806 |
| 6 | -4.13770632 | 0.80177805 | -0.51078922 |
| 6 | -4.90018912 | 1.70086229 | -1.27061144 |
| 6 | -4.42573813 | 2.29752301 | -2.44206574 |
| 6 | -3.14909166 | 1.98747260 | -2.89600827 |
| 6 | -2.34905988 | 1.08529340 | -2.18410390 |
| 6 | -1.01287183 | 0.68196523 | -2.75423596 |
| 1 | -5.05496023 | 2.99632245 | -2.99298231 |
| 6 | -4.69997087 | 0.17042564 | 0.77839152 |
| 6 | 3.07444950 | 2.59011865 | 4.21603442 |
| 6 | 2.18609393 | 0.46601229 | 3.27555494 |
| 6 | 0.61952359 | 2.36240983 | 3.89231688 |

| | | | |
|---|---|---|---|
| 6 | -6.14585153 | 0.62406386 | 1.05572173 |
| 6 | -3.84487677 | 0.59401118 | 1.99604697 |
| 6 | -4.71930426 | -1.37169255 | 0.65281547 |
| 1 | 1.54136525 | 1.03198506 | -3.48919521 |
| 1 | 2.28060488 | 0.71907712 | -1.89038239 |
| 1 | 0.45533078 | 2.98851426 | -2.43217390 |
| 1 | -0.74063258 | 2.70283884 | -1.14287101 |
| 1 | 3.84085647 | 3.76756114 | 2.25790424 |
| 1 | 2.41954437 | 4.03808749 | -1.77965764 |
| 1 | -0.98385658 | -0.40106606 | -2.95427684 |
| 1 | -0.84457633 | 1.20339693 | -3.71078639 |
| 1 | -5.90379469 | 1.95492752 | -0.93515503 |
| 1 | -2.76491074 | 2.43212678 | -3.81664143 |
| 1 | 2.94345219 | 2.17025216 | 5.22332393 |
| 1 | 4.09225558 | 2.33834649 | 3.88443467 |
| 1 | 2.99549341 | 3.68365869 | 4.30072981 |
| 1 | 1.42631537 | -0.03550333 | 2.66676642 |
| 1 | 3.17768103 | 0.20056904 | 2.88041911 |
| 1 | 2.11664169 | 0.07568757 | 4.30202877 |
| 1 | 0.49186563 | 3.45377056 | 3.93967597 |
| 1 | -0.20384435 | 1.94093556 | 3.30591881 |
| 1 | 0.55281918 | 1.97023925 | 4.91838197 |
| 1 | -6.50108000 | 0.13798439 | 1.97524116 |
| 1 | -6.83197413 | 0.33886929 | 0.24513249 |
| 1 | -6.21823643 | 1.71051861 | 1.20875523 |
| 1 | -2.80590806 | 0.26093624 | 1.90200001 |
| 1 | -3.84812644 | 1.68734899 | 2.11191896 |
| 1 | -4.26376628 | 0.15497568 | 2.91393872 |
| 1 | -5.35632980 | -1.68401378 | -0.18756727 |
| 1 | -3.71406531 | -1.77754216 | 0.49739829 |
| 1 | -5.13345511 | -1.81304780 | 1.57173531 |
| 1 | 1.37689480 | -0.57853970 | -2.71595826 |
| 8 | 2.90405542 | -4.81229435 | -2.13450767 |
| 6 | 2.78261089 | -5.35452339 | -3.47374193 |
| 1 | 1.87975029 | -4.96642971 | -3.96144309 |
| 8 | 2.98282358 | -2.72824251 | -3.04381601 |
| 8 | 0.79445497 | -1.52037994 | 0.10009020 |
| 1 | 2.71681752 | -6.43767215 | -3.33844034 |
| 1 | 3.66052197 | -5.08622575 | -4.07411353 |
| 31 | -0.23084193 | -0.02283585 | -0.10909288 |
| 6 | 2.99454081 | -3.46706164 | -2.07182900 |
| 6 | 3.16050148 | -2.94271732 | -0.64445346 |
| 8 | 2.60450480 | -3.83366616 | 0.35451220 |
| 6 | 1.27625333 | -3.87381462 | 0.69128476 |
| 6 | 0.24551233 | -2.83343262 | 0.18643265 |
| 8 | 0.90587292 | -4.77762463 | 1.41059174 |
| 6 | -0.38980329 | -3.32152868 | -1.12488484 |
| 6 | 4.64655798 | -2.79927354 | -0.31820105 |
| 1 | 2.65365883 | -1.97336111 | -0.60295664 |
| 1 | -0.52837157 | -2.87357688 | 0.97058431 |
| 1 | -1.26053919 | -2.69266910 | -1.35783499 |
| 1 | -0.73458281 | -4.35916661 | -1.01969209 |
| 1 | 0.31923368 | -3.26444462 | -1.96187404 |

| | | | |
|---|---|---|---|
| 1 | 5.13607232 | -2.16172642 | -1.06585164 |
| 1 | 5.13502053 | -3.78221120 | -0.30549317 |
| 1 | 4.75833303 | -2.33632890 | 0.67008125 |

Electronic energy = -3673.040910 a.u.
DFT-D3(BJ) dispersion correction = -0.114791 a.u.
Thermal free energy = 0.580434 a.u.
Gibbs free energy = -3672.575267 a.u.
Number of imaginary frequencies = 0.

**Int2 LA isomer/conformer 51**

| Atomic N. | X | Y | Z |
|---|---|---|---|
| 7 | 0.36881218 | 2.08827664 | 1.79232517 |
| 8 | -0.72592930 | 1.02478239 | -0.79770110 |
| 8 | 2.17805560 | 0.49005450 | -0.01435036 |
| 6 | -0.34050286 | 1.84722377 | 3.07209843 |
| 6 | -1.78057920 | 1.83582572 | -0.52096721 |
| 6 | -2.99430244 | 1.70660784 | -1.25720988 |
| 6 | -4.06685583 | 2.52262789 | -0.86609058 |
| 6 | -3.97313248 | 3.45987063 | 0.16646779 |
| 6 | -2.75417448 | 3.64064455 | 0.81148371 |
| 6 | -1.65118984 | 2.85338966 | 0.46219297 |
| 6 | -0.28979127 | 3.19346133 | 1.01041391 |
| 1 | -4.83954285 | 4.06263211 | 0.43809116 |
| 6 | -3.11314763 | 0.76412432 | -2.47312480 |
| 6 | 2.83304994 | 1.67073053 | -0.13340895 |
| 6 | 3.72553030 | 1.88550559 | -1.22382311 |
| 6 | 4.38023246 | 3.12388647 | -1.28158732 |
| 6 | 4.19164607 | 4.12917662 | -0.32813997 |
| 6 | 3.33115547 | 3.90158710 | 0.73928381 |
| 6 | 2.66023305 | 2.67760350 | 0.85248295 |
| 6 | 1.82218330 | 2.40768818 | 2.07413605 |
| 1 | 4.71974586 | 5.07789360 | -0.42206032 |
| 6 | 3.96563842 | 0.80413955 | -2.29555629 |
| 6 | -4.49607318 | 0.86702532 | -3.14411092 |
| 6 | -2.91077790 | -0.71210662 | -2.06617558 |
| 6 | -2.05392278 | 1.16057098 | -3.53009302 |
| 6 | 4.99537371 | 1.25764351 | -3.34761729 |
| 6 | 2.64857931 | 0.49385647 | -3.04557020 |
| 6 | 4.51590726 | -0.48330098 | -1.63649141 |
| 1 | -0.28222172 | 2.74456388 | 3.70921519 |
| 1 | -1.39416048 | 1.62099704 | 2.87218335 |
| 1 | -0.35186468 | 4.08488686 | 1.65707872 |
| 1 | 0.40118234 | 3.42324359 | 0.18587726 |
| 1 | -5.01639873 | 2.43000672 | -1.38975145 |
| 1 | -2.63902902 | 4.41475397 | 1.57365367 |
| 1 | 2.20968508 | 1.53655935 | 2.62653038 |
| 1 | 1.85311406 | 3.27992771 | 2.74799690 |
| 1 | 5.06285884 | 3.32071869 | -2.10616262 |
| 1 | 3.18254622 | 4.66660197 | 1.50474500 |
| 1 | -4.51852216 | 0.19938140 | -4.01695249 |
| 1 | -5.30831711 | 0.55634264 | -2.47082164 |
| 1 | -4.70916068 | 1.88487548 | -3.50091747 |

| | | | |
|---|---|---|---|
| 1 | -1.91561599 | -0.87993599 | -1.64189836 |
| 1 | -3.66539362 | -1.02089698 | -1.32836822 |
| 1 | -3.02111477 | -1.35672280 | -2.95127545 |
| 1 | -2.21116766 | 2.19502285 | -3.86833150 |
| 1 | -1.03622617 | 1.07392647 | -3.13301861 |
| 1 | -2.14049414 | 0.50140948 | -4.40704345 |
| 1 | 5.13877440 | 0.44820652 | -4.07729426 |
| 1 | 5.97571757 | 1.47628663 | -2.90002324 |
| 1 | 4.65922359 | 2.14553304 | -3.90258005 |
| 1 | 1.87847236 | 0.10529336 | -2.37107185 |
| 1 | 2.25732564 | 1.39732643 | -3.53538597 |
| 1 | 2.83546715 | -0.26022168 | -3.82503868 |
| 1 | 5.47446232 | -0.28188786 | -1.13616441 |
| 1 | 3.81609157 | -0.89039510 | -0.89890340 |
| 1 | 4.69284273 | -1.24739342 | -2.40860977 |
| 1 | 0.12480269 | 1.00237675 | 3.59312305 |
| 8 | -1.94620697 | -5.59266738 | 1.72108466 |
| 6 | -3.15030072 | -6.33846944 | 2.02349917 |
| 1 | -3.42168658 | -6.20418459 | 3.07786516 |
| 8 | -0.84154532 | -6.90518373 | 3.21829556 |
| 8 | 0.23653940 | -1.12390734 | 1.45233180 |
| 1 | -3.92015173 | -5.92174159 | 1.36724893 |
| 1 | -3.00052609 | -7.40638064 | 1.82152158 |
| 31 | 0.41815611 | 0.44505682 | 0.55633038 |
| 6 | -0.86074374 | -5.97064595 | 2.44307106 |
| 6 | 0.38224730 | -5.15769546 | 2.05275060 |
| 8 | 0.08281127 | -3.77133250 | 1.71335601 |
| 6 | -0.59392594 | -3.05805311 | 2.64243863 |
| 6 | -0.86677452 | -1.61496754 | 2.18548222 |
| 8 | -0.99923823 | -3.50464596 | 3.70130632 |
| 6 | -2.19718066 | -1.60032073 | 1.42325432 |
| 6 | 1.50305959 | -5.27204785 | 3.07505765 |
| 1 | 0.71755740 | -5.56516737 | 1.08583064 |
| 1 | -0.99702134 | -1.05854999 | 3.13252580 |
| 1 | -2.47584430 | -0.56826905 | 1.16457793 |
| 1 | -2.99921752 | -2.02506583 | 2.04273501 |
| 1 | -2.11206793 | -2.18307017 | 0.49773123 |
| 1 | 1.78707692 | -6.32543997 | 3.18655735 |
| 1 | 1.18193301 | -4.89680334 | 4.05261678 |
| 1 | 2.37395010 | -4.70265543 | 2.72684292 |

Electronic energy = -3673.044484 a.u.
DFT-D3(BJ) dispersion correction = -0.113091 a.u.
Thermal free energy = 0.582467 a.u.
Gibbs free energy = -3672.575107 a.u.
Number of imaginary frequencies = 0.

**Int2 LA isomer/conformer 52**

| Atomic N. | X | Y | Z |
|---|---|---|---|
| 8 | -0.75088416 | -5.95904114 | -0.65887709 |
| 6 | 0.20771370 | -6.98210497 | -0.28011499 |
| 1 | 0.12570258 | -7.84467635 | -0.95221733 |
| 8 | 0.25032213 | -5.87083650 | -2.70161208 |

| | | | |
|---|---|---|---|
| 8 | -0.00776604 | -1.44194193 | -0.82340289 |
| 1 | -0.05773229 | -7.25701872 | 0.74459127 |
| 1 | 1.22819536 | -6.58272357 | -0.32716213 |
| 7 | -2.21815974 | 0.95382013 | -0.00971214 |
| 8 | 0.64742081 | 1.41690008 | -0.70867675 |
| 8 | -0.11599150 | 0.05626083 | 1.95781588 |
| 6 | -3.12318221 | 0.34661029 | -1.02085155 |
| 6 | 0.11559458 | 2.14346472 | -1.72387750 |
| 6 | 0.89661961 | 2.45729320 | -2.87352529 |
| 6 | 0.27821948 | 3.21576356 | -3.87859609 |
| 6 | -1.04242714 | 3.66584191 | -3.78831806 |
| 6 | -1.78594443 | 3.37199773 | -2.64992790 |
| 6 | -1.21390066 | 2.62700021 | -1.61334596 |
| 6 | -1.96826515 | 2.40858294 | -0.33103286 |
| 1 | -1.47770899 | 4.24872817 | -4.60001658 |
| 6 | 2.35787161 | 1.98748362 | -3.01055725 |
| 6 | -0.70079301 | 0.99300787 | 2.75163205 |
| 6 | 0.00889361 | 1.51419955 | 3.87225259 |
| 6 | -0.65324047 | 2.46135143 | 4.66676315 |
| 6 | -1.95152473 | 2.90240283 | 4.39762054 |
| 6 | -2.63260714 | 2.38242626 | 3.30312608 |
| 6 | -2.02605362 | 1.42293671 | 2.48368521 |
| 6 | -2.82787051 | 0.80168921 | 1.36841807 |
| 1 | -2.42131962 | 3.64602994 | 5.04121834 |
| 6 | 1.44669263 | 1.06587898 | 4.20331957 |
| 6 | 3.00445260 | 2.49313920 | -4.31383989 |
| 6 | 2.41997964 | 0.44197877 | -3.03259079 |
| 6 | 3.20345180 | 2.53458762 | -1.83564890 |
| 6 | 1.98796830 | 1.75870939 | 5.46847202 |
| 6 | 2.39990525 | 1.42828450 | 3.03998274 |
| 6 | 1.48384356 | -0.45841655 | 4.46509236 |
| 1 | -4.07248516 | 0.90566943 | -1.04527164 |
| 1 | -2.65398234 | 0.39638499 | -2.00950692 |
| 1 | -2.94487222 | 2.91929224 | -0.36721932 |
| 1 | -1.40537383 | 2.81338422 | 0.52248025 |
| 1 | 0.84511822 | 3.46558475 | -4.77368943 |
| 1 | -2.81105693 | 3.73527588 | -2.54658668 |
| 1 | -2.94210804 | -0.28213574 | 1.51794383 |
| 1 | -3.83259836 | 1.25472714 | 1.34164551 |
| 1 | -0.13662713 | 2.88087466 | 5.52786700 |
| 1 | -3.65163955 | 2.70634443 | 3.08018732 |
| 1 | 4.04581427 | 2.14325763 | -4.35410061 |
| 1 | 2.49307155 | 2.10823831 | -5.20814644 |
| 1 | 3.02192800 | 3.59138021 | -4.36798035 |
| 1 | 2.00790690 | 0.00216376 | -2.11808404 |
| 1 | 1.85720982 | 0.04524011 | -3.89047983 |
| 1 | 3.46596210 | 0.11439957 | -3.13492755 |
| 1 | 3.19822289 | 3.63445638 | -1.83464661 |
| 1 | 2.82588870 | 2.18384564 | -0.86923368 |
| 1 | 4.24711031 | 2.20224278 | -1.94447647 |
| 1 | 3.00862500 | 1.39922256 | 5.66184694 |
| 1 | 1.38283944 | 1.52952319 | 6.35757039 |
| 1 | 2.03965051 | 2.85100770 | 5.35346388 |

| | | | |
|---|---|---|---|
| 1 | 2.11283458 | 0.93176880 | 2.10724623 |
| 1 | 2.40405717 | 2.51390980 | 2.86584519 |
| 1 | 3.42649430 | 1.12207326 | 3.29242229 |
| 1 | 0.83666766 | -0.72184824 | 5.31439330 |
| 1 | 1.15412087 | -1.02509088 | 3.58807473 |
| 1 | 2.51058136 | -0.76714206 | 4.71403764 |
| 1 | -3.31988006 | -0.69563807 | -0.74883986 |
| 31 | -0.36202027 | 0.08922493 | 0.12000196 |
| 6 | -0.59858083 | -5.48906642 | -1.91995227 |
| 6 | -1.69968265 | -4.48716313 | -2.29912764 |
| 8 | -2.36143252 | -3.84518899 | -1.17428722 |
| 6 | -1.72217581 | -3.02054549 | -0.29447258 |
| 6 | -0.20387819 | -2.77259695 | -0.36280283 |
| 8 | -2.41408301 | -2.43484780 | 0.52162937 |
| 6 | 0.43940908 | -3.07015020 | 0.99115038 |
| 6 | -1.28357633 | -3.50603893 | -3.38933808 |
| 1 | -2.50867668 | -5.12339235 | -2.69611111 |
| 1 | 0.26527555 | -3.41658910 | -1.12246039 |
| 1 | 1.51031181 | -2.83406285 | 0.93622121 |
| 1 | 0.32434084 | -4.13133622 | 1.25402417 |
| 1 | -0.02196278 | -2.46250683 | 1.77882627 |
| 1 | -0.87453942 | -4.06484966 | -4.23938192 |
| 1 | -0.52511945 | -2.79428750 | -3.04121196 |
| 1 | -2.16456525 | -2.94462481 | -3.72462822 |

Electronic energy = -3673.041710 a.u.
DFT-D3(BJ) dispersion correction = -0.116144 a.u.
Thermal free energy = 0.583086 a.u.
Gibbs free energy = -3672.574768 a.u.
Number of imaginary frequencies = 0.

**Int2 LA isomer/conformer 53**

| Atomic N. | X | Y | Z |
|---|---|---|---|
| 7 | 1.58920033 | 1.41966986 | -1.19411366 |
| 8 | 0.32140960 | -0.96534316 | 0.18395082 |
| 8 | 1.51524927 | 1.32597270 | 1.78139747 |
| 6 | 0.94006788 | 2.05868795 | -2.36726426 |
| 6 | 0.45502046 | -1.54624562 | -1.03511789 |
| 6 | -0.25115535 | -2.74708774 | -1.33311789 |
| 6 | -0.10742641 | -3.27250541 | -2.62578263 |
| 6 | 0.70325885 | -2.68166012 | -3.59844953 |
| 6 | 1.43595433 | -1.54502311 | -3.27324874 |
| 6 | 1.33534347 | -0.98357464 | -1.99613978 |
| 6 | 2.26636819 | 0.13146614 | -1.60001679 |
| 1 | 0.77139215 | -3.11982171 | -4.59391183 |
| 6 | -1.11251532 | -3.46393372 | -0.27338913 |
| 6 | 2.85784822 | 1.36357332 | 1.67990346 |
| 6 | 3.67592956 | 0.95175735 | 2.77462333 |
| 6 | 5.06466646 | 1.02868695 | 2.60275087 |
| 6 | 5.66137319 | 1.48423244 | 1.42224982 |
| 6 | 4.85440734 | 1.89359476 | 0.36694341 |
| 6 | 3.46057260 | 1.84901748 | 0.49124829 |
| 6 | 2.60124050 | 2.39219109 | -0.61620233 |

| | | | |
|---|---|---|---|
| 1 | 6.74732811 | 1.51825667 | 1.33602785 |
| 6 | 3.05878771 | 0.44177317 | 4.09109031 |
| 6 | -1.73116659 | -4.76547120 | -0.81721784 |
| 6 | -2.27928833 | -2.56083462 | 0.18831953 |
| 6 | -0.23441242 | -3.84214447 | 0.94354471 |
| 6 | 4.13650663 | 0.08975619 | 5.13362216 |
| 6 | 2.23515679 | -0.84136292 | 3.82849514 |
| 6 | 2.15419306 | 1.53315015 | 4.71170967 |
| 1 | 1.71432742 | 2.35961657 | -3.09109840 |
| 1 | 0.24790764 | 1.35598245 | -2.83624496 |
| 1 | 2.95873634 | 0.35896399 | -2.42755473 |
| 1 | 2.87181844 | -0.17008637 | -0.73265904 |
| 1 | -0.64868297 | -4.17887270 | -2.89112055 |
| 1 | 2.11098028 | -1.09367903 | -4.00393031 |
| 1 | 2.00957910 | 3.25495499 | -0.26918123 |
| 1 | 3.24257593 | 2.73351898 | -1.44521813 |
| 1 | 5.71476251 | 0.71578136 | 3.41796599 |
| 1 | 5.29855320 | 2.26415639 | -0.55988870 |
| 1 | -2.31689141 | -5.24390051 | -0.01925638 |
| 1 | -2.41070571 | -4.58107372 | -1.66221120 |
| 1 | -0.96385452 | -5.48348747 | -1.13973396 |
| 1 | -1.90641444 | -1.63706053 | 0.64355472 |
| 1 | -2.92878594 | -2.30573695 | -0.66261820 |
| 1 | -2.89158009 | -3.09149380 | 0.93324590 |
| 1 | 0.57396149 | -4.52220802 | 0.63863263 |
| 1 | 0.21306920 | -2.95637990 | 1.40785990 |
| 1 | -0.84532710 | -4.36089105 | 1.69813465 |
| 1 | 3.64362204 | -0.25668755 | 6.05319990 |
| 1 | 4.75612086 | 0.95910496 | 5.39765533 |
| 1 | 4.79838138 | -0.71840706 | 4.78984969 |
| 1 | 1.41592613 | -0.65901139 | 3.12503147 |
| 1 | 2.87516225 | -1.63551514 | 3.41721615 |
| 1 | 1.80599040 | -1.20736852 | 4.77377111 |
| 1 | 2.74028566 | 2.43481584 | 4.94295516 |
| 1 | 1.33842001 | 1.81101047 | 4.03568643 |
| 1 | 1.71941789 | 1.16305505 | 5.65289357 |
| 1 | 0.38525079 | 2.94546884 | -2.03886014 |
| 8 | -4.24956285 | 2.45655781 | -3.73007669 |
| 6 | -4.93264228 | 3.10549956 | -2.63213098 |
| 1 | -5.63485944 | 2.43923638 | -2.12036466 |
| 8 | -3.42664509 | 0.66799560 | -4.74326814 |
| 8 | -1.09504521 | 1.85524060 | 0.93689028 |
| 1 | -5.48163442 | 3.93476670 | -3.09174232 |
| 1 | -4.20649495 | 3.50115242 | -1.91210660 |
| 31 | 0.36078769 | 0.88881762 | 0.37604125 |
| 6 | -3.87280701 | 1.14957403 | -3.72436733 |
| 6 | -4.05207987 | 0.23791340 | -2.49194627 |
| 8 | -3.85931221 | 0.88424333 | -1.19236558 |
| 6 | -2.57037057 | 1.05897381 | -0.80739442 |
| 6 | -2.42714580 | 1.55699807 | 0.63307001 |
| 8 | -1.61280624 | 0.80289874 | -1.52037563 |
| 6 | -3.29691590 | 2.78734207 | 0.92042348 |
| 6 | -5.42756491 | -0.42084285 | -2.47870414 |

| | | | |
|---|---|---|---|
| 1 | -3.26616528 | -0.51762289 | -2.60963406 |
| 1 | -2.80311141 | 0.71773786 | 1.25898260 |
| 1 | -3.16528888 | 3.06821394 | 1.97226572 |
| 1 | -4.35876572 | 2.58025511 | 0.73661052 |
| 1 | -2.97240808 | 3.63378625 | 0.29959357 |
| 1 | -5.57959960 | -0.94916037 | -3.42845530 |
| 1 | -6.23749774 | 0.30861777 | -2.35113135 |
| 1 | -5.47998125 | -1.14954292 | -1.65979517 |

Electronic energy = -3673.040094 a.u.
DFT-D3(BJ) dispersion correction = -0.116461 a.u.
Thermal free energy = 0.581883 a.u.
Gibbs free energy = -3672.574672 a.u.
Number of imaginary frequencies = 0.

**Int2 LA isomer/conformer 54**

| Atomic N. | X | Y | Z |
|---|---|---|---|
| 7 | -2.45449431 | -0.92483745 | -0.11899266 |
| 8 | 0.06609577 | 0.50157346 | 0.76121474 |
| 8 | -0.28839424 | -2.46298598 | 1.24057572 |
| 6 | -3.07238812 | -0.76335571 | -1.45959729 |
| 6 | -0.64704424 | 1.62384907 | 0.50347033 |
| 6 | 0.01111642 | 2.88357562 | 0.40936622 |
| 6 | -0.77900114 | 3.99431114 | 0.07594418 |
| 6 | -2.15899400 | 3.90795930 | -0.13107470 |
| 6 | -2.79885476 | 2.68388825 | 0.04173812 |
| 6 | -2.05891081 | 1.54576672 | 0.37714625 |
| 6 | -2.76609211 | 0.26982823 | 0.75258729 |
| 1 | -2.72838236 | 4.79828090 | -0.39763165 |
| 6 | 1.51532881 | 3.03231183 | 0.71818909 |
| 6 | -1.20187979 | -2.62345944 | 2.21841372 |
| 6 | -0.79733974 | -2.99687688 | 3.53484096 |
| 6 | -1.80615024 | -3.15207306 | 4.49544282 |
| 6 | -3.16287217 | -2.95998753 | 4.21304659 |
| 6 | -3.54630722 | -2.61296748 | 2.92266171 |
| 6 | -2.57874343 | -2.45667348 | 1.92289471 |
| 6 | -3.00756426 | -2.19307879 | 0.50719003 |
| 1 | -3.90803069 | -3.08662109 | 4.99827651 |
| 6 | 0.68574126 | -3.22086553 | 3.88721026 |
| 6 | 1.98633119 | 4.49313540 | 0.59107150 |
| 6 | 2.37773684 | 2.18681398 | -0.24765462 |
| 6 | 1.78335645 | 2.58178452 | 2.17463102 |
| 6 | 0.86947124 | -3.65528073 | 5.35331932 |
| 6 | 1.48229512 | -1.90931863 | 3.69083234 |
| 6 | 1.27995857 | -4.33755190 | 2.99584781 |
| 1 | -4.16949635 | -0.77627627 | -1.35725109 |
| 1 | -2.75166237 | 0.18185539 | -1.90236470 |
| 1 | -3.85822084 | 0.42228669 | 0.73950444 |
| 1 | -2.48163115 | -0.02848108 | 1.77223475 |
| 1 | -0.30514326 | 4.96945124 | -0.02388967 |
| 1 | -3.88406211 | 2.60754972 | -0.05827595 |
| 1 | -2.68368622 | -3.00941783 | -0.15845338 |
| 1 | -4.10725737 | -2.13250725 | 0.45976590 |

| | | | |
|---|---|---|---|
| 1 | -1.52854603 | -3.42910220 | 5.51097319 |
| 1 | -4.60134144 | -2.47359198 | 2.67550329 |
| 1 | 3.05643736 | 4.54721889 | 0.83695212 |
| 1 | 1.86098184 | 4.88219660 | -0.43025635 |
| 1 | 1.45446138 | 5.16061564 | 1.28383656 |
| 1 | 2.13469999 | 1.12182376 | -0.16902256 |
| 1 | 2.23249007 | 2.51188839 | -1.28808568 |
| 1 | 3.44278122 | 2.31626229 | -0.00222243 |
| 1 | 1.21596750 | 3.20298496 | 2.88241508 |
| 1 | 1.50317191 | 1.53352460 | 2.32820185 |
| 1 | 2.85309099 | 2.69344115 | 2.40882250 |
| 1 | 1.93998405 | -3.81339312 | 5.54706850 |
| 1 | 0.35051666 | -4.59966510 | 5.57284257 |
| 1 | 0.51594347 | -2.89080311 | 6.06043355 |
| 1 | 1.43931413 | -1.56231253 | 2.65310868 |
| 1 | 1.08694845 | -1.11400894 | 4.33936625 |
| 1 | 2.53751848 | -2.07224209 | 3.95848645 |
| 1 | 0.74761072 | -5.28621876 | 3.15882499 |
| 1 | 1.21667427 | -4.07961860 | 1.93321647 |
| 1 | 2.33783917 | -4.49568842 | 3.25589823 |
| 1 | -2.75942217 | -1.58945746 | -2.10916749 |
| 8 | 1.82553670 | 1.53655279 | -6.60341273 |
| 6 | 3.02459514 | 1.32012867 | -5.82094750 |
| 1 | 3.05584805 | 1.94809425 | -4.92403043 |
| 8 | -0.33965456 | 1.94597195 | -6.81178168 |
| 8 | 0.67171585 | -1.90973402 | -1.31791089 |
| 1 | 3.84670857 | 1.59517944 | -6.49004667 |
| 1 | 3.11134329 | 0.26525934 | -5.53745056 |
| 31 | -0.39159916 | -1.11568043 | -0.05271969 |
| 6 | 0.58675062 | 1.69247862 | -6.06975706 |
| 6 | 0.32435735 | 1.63056629 | -4.55511953 |
| 8 | 1.09622492 | 0.53759336 | -3.95066109 |
| 6 | 0.54260207 | -0.05500548 | -2.86816104 |
| 6 | 1.33881428 | -1.25895547 | -2.36054789 |
| 8 | -0.48773747 | 0.32970287 | -2.33424966 |
| 6 | 1.61531947 | -2.26733636 | -3.48536818 |
| 6 | 0.58042138 | 2.97331662 | -3.87405472 |
| 1 | -0.73677085 | 1.37423467 | -4.45928471 |
| 1 | 2.31126823 | -0.84583598 | -2.01431459 |
| 1 | 2.17448543 | -3.11148434 | -3.06439966 |
| 1 | 2.20309934 | -1.81735387 | -4.29523433 |
| 1 | 0.67021967 | -2.64915649 | -3.89434619 |
| 1 | 0.00262091 | 3.74848331 | -4.39498743 |
| 1 | 1.64078214 | 3.25427381 | -3.89865813 |
| 1 | 0.24109823 | 2.93324709 | -2.83198200 |

Electronic energy = -3673.039511 a.u.
DFT-D3(BJ) dispersion correction = -0.116768 a.u.
Thermal free energy = 0.581768 a.u.
Gibbs free energy = -3672.574510 a.u.
Number of imaginary frequencies = 0.

**Int2_LA isomer/conformer 55**

| Atomic N. | X | Y | Z |
|---|---|---|---|
| 7 | 2.24932063 | -1.19282104 | 0.66726048 |
| 8 | -0.09111420 | 0.72283792 | 0.54889151 |
| 8 | 2.21634471 | 0.96955710 | -1.39027607 |
| 6 | 2.10727276 | -2.66703738 | 0.77558671 |
| 6 | -0.40941188 | 0.18690713 | 1.75145353 |
| 6 | -1.70061276 | 0.40463155 | 2.31206334 |
| 6 | -1.98490524 | -0.23281909 | 3.52941238 |
| 6 | -1.05387319 | -1.02858100 | 4.20366645 |
| 6 | 0.22895789 | -1.16750574 | 3.68147484 |
| 6 | 0.56592705 | -0.55047580 | 2.47273025 |
| 6 | 1.99818812 | -0.53839206 | 2.00593318 |
| 1 | -1.32523690 | -1.51056479 | 5.14282121 |
| 6 | -2.72206136 | 1.34656678 | 1.64166412 |
| 6 | 3.28304861 | 1.44184082 | -0.71531020 |
| 6 | 3.69900392 | 2.79760472 | -0.87299082 |
| 6 | 4.82126798 | 3.21261865 | -0.14303520 |
| 6 | 5.53104925 | 2.36229918 | 0.71166510 |
| 6 | 5.12702321 | 1.03867291 | 0.84334134 |
| 6 | 4.01877428 | 0.57034038 | 0.12739665 |
| 6 | 3.65669576 | -0.88679079 | 0.18578190 |
| 1 | 6.39373530 | 2.73663400 | 1.26260473 |
| 6 | 2.94793057 | 3.76561787 | -1.80714680 |
| 6 | -4.02435103 | 1.45561381 | 2.45665481 |
| 6 | -3.10585613 | 0.85022940 | 0.22828133 |
| 6 | -2.11596837 | 2.76730012 | 1.54102107 |
| 6 | 3.60640217 | 5.15738517 | -1.84326658 |
| 6 | 1.49296478 | 3.95558057 | -1.31698807 |
| 6 | 2.94975394 | 3.21527569 | -3.25344484 |
| 1 | 2.90312102 | -3.06126298 | 1.42787939 |
| 1 | 1.12870081 | -2.91381920 | 1.19255912 |
| 1 | 2.64489001 | -1.03200831 | 2.75044331 |
| 1 | 2.34601812 | 0.49911959 | 1.89538120 |
| 1 | -2.96916610 | -0.10380909 | 3.97664279 |
| 1 | 0.98819795 | -1.73704419 | 4.22247833 |
| 1 | 3.72747165 | -1.34794850 | -0.81257586 |
| 1 | 4.35719328 | -1.41459830 | 0.85363407 |
| 1 | 5.15862021 | 4.24346678 | -0.23612176 |
| 1 | 5.67618926 | 0.35230417 | 1.49215545 |
| 1 | -4.70871887 | 2.14890046 | 1.94721817 |
| 1 | -4.53972918 | 0.48788802 | 2.54694672 |
| 1 | -3.84753815 | 1.84971205 | 3.46748011 |
| 1 | -2.22982688 | 0.80100861 | -0.42716540 |
| 1 | -3.57380047 | -0.14374590 | 0.27687699 |
| 1 | -3.83451692 | 1.54158538 | -0.22165679 |
| 1 | -1.87768994 | 3.15783448 | 2.54080103 |
| 1 | -1.20095412 | 2.77124882 | 0.93795416 |
| 1 | -2.84226231 | 3.45075025 | 1.07521492 |
| 1 | 3.04115308 | 5.80173629 | -2.53162752 |
| 1 | 4.64353668 | 5.11475392 | -2.20648247 |
| 1 | 3.60215463 | 5.64459092 | -0.85734681 |
| 1 | 0.94203203 | 3.00922915 | -1.31131405 |
| 1 | 1.47724580 | 4.37240423 | -0.29942474 |

| | | | |
|---|---|---|---|
| 1 | 0.96671729 | 4.65918149 | -1.98007896 |
| 1 | 3.97933780 | 3.11600158 | -3.62763922 |
| 1 | 2.46121625 | 2.23667244 | -3.31148795 |
| 1 | 2.41606376 | 3.91350753 | -3.91630111 |
| 1 | 2.19815898 | -3.11790314 | -0.21979800 |
| 8 | -4.47808891 | -5.14189357 | -1.72259282 |
| 6 | -5.14710201 | -3.91534867 | -2.10554227 |
| 1 | -5.84625289 | -3.60617444 | -1.31687584 |
| 8 | -3.16277203 | -6.29085271 | -0.36162648 |
| 8 | 0.32695145 | -0.90779224 | -2.22355894 |
| 1 | -5.71550579 | -4.17616415 | -3.00396470 |
| 1 | -4.44121586 | -3.11091304 | -2.33140151 |
| 31 | 0.97961781 | -0.22399389 | -0.65291769 |
| 6 | -3.50203161 | -5.20297641 | -0.77870536 |
| 6 | -2.82667592 | -3.93861745 | -0.21370225 |
| 8 | -2.43619415 | -3.06504245 | -1.32959950 |
| 6 | -1.31063767 | -2.33447000 | -1.15520078 |
| 6 | -0.91038099 | -1.53625610 | -2.39773260 |
| 8 | -0.67619101 | -2.30087756 | -0.11123683 |
| 6 | -0.85319306 | -2.42622706 | -3.64760382 |
| 6 | -3.66990210 | -3.20769559 | 0.82658509 |
| 1 | -1.90491123 | -4.29929412 | 0.25606838 |
| 1 | -1.72045728 | -0.78750908 | -2.53911958 |
| 1 | -0.55703661 | -1.80588283 | -4.50194910 |
| 1 | -1.82601886 | -2.88745915 | -3.85915960 |
| 1 | -0.10069759 | -3.21555580 | -3.51746777 |
| 1 | -3.99095486 | -3.92487240 | 1.59456080 |
| 1 | -4.55987857 | -2.74249195 | 0.38641225 |
| 1 | -3.06455580 | -2.43392196 | 1.31443948 |

Electronic energy = -3673.039057 a.u.
DFT-D3(BJ) dispersion correction = -0.116550 a.u.
Thermal free energy = 0.581555 a.u.
Gibbs free energy = -3672.574052 a.u.
Number of imaginary frequencies = 0.

**Int2 LA isomer/conformer 56**

| Atomic N. | X | Y | Z |
|---|---|---|---|
| 7 | 0.06233735 | 1.81682531 | 2.39247683 |
| 8 | -0.47185100 | 1.27116830 | -0.49344477 |
| 8 | 1.32476859 | -0.65654952 | 1.23627123 |
| 6 | -1.10765175 | 2.11041071 | 3.25981081 |
| 6 | -1.02293604 | 2.51567162 | -0.45113674 |
| 6 | -1.95157782 | 2.92057981 | -1.45169284 |
| 6 | -2.46379598 | 4.22315355 | -1.35716663 |
| 6 | -2.10461924 | 5.11002976 | -0.33835205 |
| 6 | -1.19081377 | 4.70345827 | 0.62750839 |
| 6 | -0.63816557 | 3.41900177 | 0.57225706 |
| 6 | 0.42810885 | 3.02012315 | 1.55463004 |
| 1 | -2.53676801 | 6.11005157 | -0.30687565 |
| 6 | -2.39084490 | 1.97154136 | -2.58374589 |
| 6 | 2.48506038 | -0.00642675 | 1.52147437 |
| 6 | 3.69731445 | -0.40039250 | 0.88635326 |

| | | | |
|---|---|---|---|
| 6 | 4.85921485 | 0.30372079 | 1.23392089 |
| 6 | 4.86335566 | 1.35246002 | 2.15796729 |
| 6 | 3.67469580 | 1.71868216 | 2.77838637 |
| 6 | 2.48676978 | 1.04073484 | 2.47854073 |
| 6 | 1.23369291 | 1.38120124 | 3.24352940 |
| 1 | 5.79217280 | 1.87357040 | 2.38898978 |
| 6 | 3.73715319 | -1.54772516 | -0.14270672 |
| 6 | -3.36689722 | 2.65561983 | -3.55972426 |
| 6 | -3.11906525 | 0.74437860 | -1.98673173 |
| 6 | -1.16306916 | 1.51472374 | -3.40713390 |
| 6 | 5.16477610 | -1.80422007 | -0.66150533 |
| 6 | 2.85963252 | -1.19690901 | -1.36765108 |
| 6 | 3.24206205 | -2.86239476 | 0.50610319 |
| 1 | -0.85546223 | 2.91647459 | 3.96718006 |
| 1 | -1.95285713 | 2.42626620 | 2.63902957 |
| 1 | 0.65118623 | 3.85607482 | 2.23819679 |
| 1 | 1.35917106 | 2.75037642 | 1.03492483 |
| 1 | -3.17984873 | 4.56213382 | -2.10334476 |
| 1 | -0.88342078 | 5.38528942 | 1.42363889 |
| 1 | 0.87747829 | 0.50832234 | 3.81369465 |
| 1 | 1.44655393 | 2.18729856 | 3.96482359 |
| 1 | 5.80150864 | 0.03143956 | 0.76241564 |
| 1 | 3.65929743 | 2.52632263 | 3.51349584 |
| 1 | -3.63868802 | 1.94129040 | -4.34968895 |
| 1 | -4.29824183 | 2.96753402 | -3.06562989 |
| 1 | -2.91850284 | 3.53367973 | -4.04694073 |
| 1 | -2.47097816 | 0.17065479 | -1.31582828 |
| 1 | -4.00822392 | 1.05593623 | -1.42039267 |
| 1 | -3.45635158 | 0.07911871 | -2.79581889 |
| 1 | -0.65243318 | 2.37961904 | -3.85546922 |
| 1 | -0.44307757 | 0.96608481 | -2.79064595 |
| 1 | -1.49358265 | 0.85890631 | -4.22716846 |
| 1 | 5.13898659 | -2.63641311 | -1.37908405 |
| 1 | 5.85461988 | -2.08637874 | 0.14689516 |
| 1 | 5.57932749 | -0.93090590 | -1.18531514 |
| 1 | 1.80902097 | -1.05665400 | -1.09265928 |
| 1 | 3.21581092 | -0.27544915 | -1.85016772 |
| 1 | 2.91446424 | -2.00936451 | -2.10783727 |
| 1 | 3.88833568 | -3.14602503 | 1.34950158 |
| 1 | 2.21424231 | -2.76751270 | 0.87269793 |
| 1 | 3.27616992 | -3.67667575 | -0.23350379 |
| 1 | -1.38743733 | 1.20859285 | 3.81586660 |
| 8 | -1.19881348 | -2.77411827 | -2.72565609 |
| 6 | -1.54912016 | -2.67875861 | -4.12825243 |
| 1 | -2.39749309 | -1.99693158 | -4.26298338 |
| 8 | -3.31743912 | -3.50423735 | -2.30230498 |
| 8 | -1.69552401 | -0.80423877 | 1.34954717 |
| 1 | -0.65507049 | -2.28916410 | -4.62378107 |
| 1 | -1.81577377 | -3.66742639 | -4.52279384 |
| 31 | -0.26375127 | 0.28929678 | 1.06195792 |
| 6 | -2.20459076 | -3.22242148 | -1.92103327 |
| 6 | -1.66478053 | -3.34894766 | -0.49029251 |
| 8 | -2.80648800 | -3.43962331 | 0.38557437 |

| | | | |
|---|---|---|---|
| 6 | -2.79703041 | -2.89587740 | 1.64144519 |
| 6 | -1.58476734 | -2.03453896 | 2.07470299 |
| 8 | -3.75195031 | -3.09430300 | 2.35981083 |
| 6 | -1.60730837 | -1.83381551 | 3.58464518 |
| 6 | -0.79958059 | -4.60587950 | -0.36300434 |
| 1 | -1.08484290 | -2.44198437 | -0.28024188 |
| 1 | -0.65279276 | -2.56838843 | 1.80870226 |
| 1 | -0.71796930 | -1.26538820 | 3.89534348 |
| 1 | -1.59057842 | -2.80023400 | 4.10653467 |
| 1 | -2.51814311 | -1.30195931 | 3.88464777 |
| 1 | 0.04302743 | -4.56004968 | -1.06476906 |
| 1 | -1.40039555 | -5.49930214 | -0.57711561 |
| 1 | -0.39895088 | -4.69023414 | 0.65609851 |

Electronic energy = -3673.038505 a.u.
DFT-D3(BJ) dispersion correction = -0.116200 a.u.
Thermal free energy = 0.580743 a.u.
Gibbs free energy = -3672.573962 a.u.
Number of imaginary frequencies = 0.

**Int2 LA isomer/conformer 57**

| Atomic N. | X | Y | Z |
|---|---|---|---|
| 7 | -2.27933668 | 0.58258061 | 2.04433620 |
| 8 | -1.17588069 | 0.58893394 | -0.73926388 |
| 8 | 0.72297963 | 0.47925457 | 1.67081313 |
| 6 | -3.36835515 | -0.37730052 | 2.36078066 |
| 6 | -2.45788031 | 0.91149645 | -1.06969755 |
| 6 | -2.90325097 | 0.76975330 | -2.41539084 |
| 6 | -4.23942385 | 1.10267603 | -2.68365794 |
| 6 | -5.11905692 | 1.57220729 | -1.70455508 |
| 6 | -4.65694793 | 1.75086500 | -0.40563141 |
| 6 | -3.33075296 | 1.44147953 | -0.08330783 |
| 6 | -2.80570456 | 1.77461355 | 1.28789557 |
| 1 | -6.15129322 | 1.80739974 | -1.96388913 |
| 6 | -1.96180945 | 0.29588619 | -3.54034956 |
| 6 | 0.67494599 | 1.65875450 | 2.33876162 |
| 6 | 1.77792865 | 2.56031909 | 2.27030962 |
| 6 | 1.67944478 | 3.75738841 | 2.99328361 |
| 6 | 0.55924602 | 4.09083349 | 3.75950956 |
| 6 | -0.50590997 | 3.20065597 | 3.82651543 |
| 6 | -0.45525989 | 1.98349251 | 3.13621506 |
| 6 | -1.58196077 | 0.99827085 | 3.31823180 |
| 1 | 0.52568058 | 5.03814250 | 4.29730664 |
| 6 | 3.03117526 | 2.24112691 | 1.43152399 |
| 6 | -2.67327115 | 0.26385095 | -4.90641200 |
| 6 | -1.44368317 | -1.13134631 | -3.25464831 |
| 6 | -0.76711950 | 1.27222640 | -3.66413965 |
| 6 | 4.09571906 | 3.34922789 | 1.54258312 |
| 6 | 2.65048986 | 2.11767742 | -0.06276417 |
| 6 | 3.68318737 | 0.92908366 | 1.92880331 |
| 1 | -4.09300580 | 0.09488219 | 3.04364632 |
| 1 | -3.87616736 | -0.66845610 | 1.43518227 |
| 1 | -3.59574011 | 2.24888716 | 1.89375446 |

| | | | |
|---|---|---|---|
| 1 | -1.96465131 | 2.48027978 | 1.21827662 |
| 1 | -4.61549691 | 0.98828259 | -3.69845406 |
| 1 | -5.31564837 | 2.15155330 | 0.36832644 |
| 1 | -1.21398924 | 0.06143192 | 3.76681886 |
| 1 | -2.33815003 | 1.42450036 | 3.99816555 |
| 1 | 2.50416590 | 4.46650878 | 2.95475552 |
| 1 | -1.38657001 | 3.43580936 | 4.42845818 |
| 1 | -1.95414916 | -0.06022177 | -5.67196989 |
| 1 | -3.51099550 | -0.44852127 | -4.91985077 |
| 1 | -3.05015333 | 1.25338551 | -5.20404418 |
| 1 | -0.82265364 | -1.16407527 | -2.35540553 |
| 1 | -2.27378005 | -1.83944905 | -3.12631091 |
| 1 | -0.83034821 | -1.47544115 | -4.10125573 |
| 1 | -1.11522576 | 2.28608122 | -3.91152112 |
| 1 | -0.18754844 | 1.31655706 | -2.73542039 |
| 1 | -0.09941158 | 0.93669817 | -4.47222181 |
| 1 | 4.96937118 | 3.06635475 | 0.93808270 |
| 1 | 4.44103678 | 3.49015637 | 2.57702445 |
| 1 | 3.73155222 | 4.31434324 | 1.16211356 |
| 1 | 1.94599699 | 1.29869372 | -0.24222595 |
| 1 | 2.19401272 | 3.05113884 | -0.42125220 |
| 1 | 3.55512988 | 1.93563998 | -0.66376886 |
| 1 | 3.98572198 | 1.02137747 | 2.98217385 |
| 1 | 2.99610508 | 0.08055424 | 1.83799348 |
| 1 | 4.58916663 | 0.71781539 | 1.33935817 |
| 1 | -2.93684624 | -1.27097869 | 2.82388458 |
| 8 | 3.84311137 | -2.79716111 | -1.20105496 |
| 6 | 4.41939382 | -1.50524486 | -1.53324239 |
| 1 | 4.07501818 | -0.74430104 | -0.82325349 |
| 8 | 1.77885804 | -1.88183448 | -1.47893185 |
| 8 | -0.99288391 | -2.00407467 | 1.25122160 |
| 1 | 5.50138019 | -1.64814466 | -1.46190048 |
| 1 | 4.12853022 | -1.21261312 | -2.54915956 |
| 31 | -0.81337977 | -0.22677491 | 0.88503501 |
| 8 | 0.58252758 | -4.35094459 | -1.10174561 |
| 6 | -0.40652007 | -3.67007341 | -0.41129431 |
| 6 | -0.05637153 | -3.01145040 | 0.94139733 |
| 6 | 2.48850415 | -2.83140774 | -1.21499246 |
| 6 | 1.98174255 | -4.22549627 | -0.80288061 |
| 8 | -1.52791089 | -3.71348524 | -0.85411854 |
| 6 | 2.68331586 | -5.36154436 | -1.54462769 |
| 6 | -0.13649963 | -4.09591224 | 2.02854088 |
| 1 | 0.96796351 | -2.60614372 | 0.91158492 |
| 1 | 2.17149522 | -4.32929575 | 0.27925087 |
| 1 | 2.27828490 | -6.32208741 | -1.20328585 |
| 1 | 3.76169828 | -5.33692626 | -1.35221034 |
| 1 | 2.50908750 | -5.27501357 | -2.62461696 |
| 1 | 0.54385239 | -4.93616631 | 1.82640126 |
| 1 | -1.16270866 | -4.48254380 | 2.07762872 |
| 1 | 0.12588232 | -3.65469809 | 2.99877893 |

Electronic energy = -3673.038744 a.u.
DFT-D3(BJ) dispersion correction = -0.115220 a.u.
Thermal free energy = 0.580630 a.u.

Gibbs free energy = -3672.573335 a.u.
Number of imaginary frequencies = 0.

**Int2 LA isomer/conformer 58**

| Atomic N. | X | Y | Z |
|---|---|---|---|
| 8 | 2.74740627 | 3.13475951 | -3.70962269 |
| 6 | 1.30674600 | 3.33148828 | -3.72588163 |
| 1 | 0.83518724 | 2.84521828 | -4.58625168 |
| 8 | 4.57241853 | 1.92520202 | -4.05663340 |
| 8 | 2.36873748 | -0.69883004 | -0.22009800 |
| 1 | 1.18168555 | 4.41643722 | -3.79947702 |
| 1 | 0.85619353 | 2.96872469 | -2.79598966 |
| 7 | 1.27108788 | -0.78201279 | 2.47050283 |
| 8 | -0.81530345 | -1.36153201 | 0.43891934 |
| 8 | 0.39528617 | 1.55479183 | 0.84319378 |
| 6 | 2.32203045 | -1.82747877 | 2.52789500 |
| 6 | -1.04419604 | -2.43989208 | 1.22277225 |
| 6 | -1.77814209 | -3.54712324 | 0.69874882 |
| 6 | -1.97818267 | -4.64763410 | 1.54412765 |
| 6 | -1.50346186 | -4.69064655 | 2.85798163 |
| 6 | -0.82080314 | -3.59172226 | 3.36801097 |
| 6 | -0.60040046 | -2.46357482 | 2.57036687 |
| 6 | 0.02784599 | -1.23882255 | 3.17715750 |
| 1 | -1.67872252 | -5.57166692 | 3.47535623 |
| 6 | -2.35155683 | -3.53167402 | -0.73253477 |
| 6 | 0.16008841 | 2.10557968 | 2.05351717 |
| 6 | -0.72218853 | 3.22250647 | 2.16601799 |
| 6 | -0.91798861 | 3.76413590 | 3.44388969 |
| 6 | -0.29310323 | 3.25653849 | 4.58685330 |
| 6 | 0.56837784 | 2.17233624 | 4.46381770 |
| 6 | 0.80891070 | 1.60068855 | 3.20911521 |
| 6 | 1.80906261 | 0.48294466 | 3.08803314 |
| 1 | -0.48200960 | 3.70822343 | 5.56063395 |
| 6 | -1.43234358 | 3.81509910 | 0.93310598 |
| 6 | -3.12695716 | -4.82189815 | -1.05856816 |
| 6 | -1.21095939 | -3.40624688 | -1.76910152 |
| 6 | -3.33484081 | -2.34753611 | -0.89298871 |
| 6 | -2.32017497 | 5.01888316 | 1.30056636 |
| 6 | -2.34549236 | 2.75227875 | 0.27731961 |
| 6 | -0.38204969 | 4.31308258 | -0.08795633 |
| 1 | 2.58315941 | -2.03684487 | 3.57899028 |
| 1 | 1.95326658 | -2.74654013 | 2.06067159 |
| 1 | 0.27401029 | -1.42752088 | 4.23652064 |
| 1 | -0.67135298 | -0.38993238 | 3.14707962 |
| 1 | -2.52510999 | -5.51015673 | 1.16757490 |
| 1 | -0.46532321 | -3.59076641 | 4.40104420 |
| 1 | 2.65881429 | 0.78125313 | 2.45294798 |
| 1 | 2.20487044 | 0.23585134 | 4.08808140 |
| 1 | -1.58881360 | 4.61348322 | 3.56011157 |
| 1 | 1.07362556 | 1.76363105 | 5.34195686 |
| 1 | -3.52168122 | -4.75187841 | -2.08231532 |
| 1 | -2.48657281 | -5.71477157 | -1.01112400 |

| | | | |
|---|---|---|---|
| 1 | -3.98230922 | -4.97132541 | -0.38419924 |
| 1 | -0.66377104 | -2.46761824 | -1.63344090 |
| 1 | -0.50820382 | -4.24796567 | -1.67743912 |
| 1 | -1.62852984 | -3.42142145 | -2.78753853 |
| 1 | -4.17426701 | -2.44579188 | -0.18943325 |
| 1 | -2.83886168 | -1.38745170 | -0.71361656 |
| 1 | -3.74799174 | -2.34239140 | -1.91338988 |
| 1 | -2.79277784 | 5.40618100 | 0.38649555 |
| 1 | -1.74120350 | 5.84023666 | 1.74710445 |
| 1 | -3.12547513 | 4.74204657 | 1.99619546 |
| 1 | -1.77526206 | 1.88341149 | -0.06661823 |
| 1 | -3.11019721 | 2.40748080 | 0.98838310 |
| 1 | -2.86397710 | 3.19224503 | -0.58846704 |
| 1 | 0.23367757 | 5.11325983 | 0.34820839 |
| 1 | 0.27859285 | 3.49748216 | -0.40209298 |
| 1 | -0.89048703 | 4.72227209 | -0.97511187 |
| 1 | 3.20398177 | -1.47873945 | 1.98169353 |
| 31 | 0.70028499 | -0.26505200 | 0.51541130 |
| 6 | 3.35991465 | 1.96423742 | -3.99106841 |
| 6 | 2.58032690 | 0.66112827 | -4.28146087 |
| 8 | 1.34323817 | 0.52110895 | -3.51686290 |
| 6 | 1.34970903 | 0.08765559 | -2.24279839 |
| 6 | 2.55123965 | -0.61404208 | -1.59916481 |
| 8 | 0.31000070 | 0.24070788 | -1.59957756 |
| 6 | 2.71379171 | -2.02651360 | -2.20817269 |
| 6 | 2.21726520 | 0.53986637 | -5.75781283 |
| 1 | 3.27909499 | -0.13748598 | -4.01377299 |
| 1 | 3.45961522 | -0.01814886 | -1.81687254 |
| 1 | 3.56814014 | -2.50558545 | -1.71494791 |
| 1 | 2.89660741 | -2.02182654 | -3.29193054 |
| 1 | 1.81437180 | -2.62149840 | -2.00452170 |
| 1 | 3.12786907 | 0.64075653 | -6.36151415 |
| 1 | 1.50150864 | 1.31151703 | -6.06619836 |
| 1 | 1.77300336 | -0.44517047 | -5.94980665 |

Electronic energy = -3673.040115 a.u.
DFT-D3(BJ) dispersion correction = -0.115983 a.u.
Thermal free energy = 0.582845 a.u.
Gibbs free energy = -3672.573253 a.u.
Number of imaginary frequencies = 0.

**Int2 LA isomer/conformer 59**

| Atomic N. | X | Y | Z |
|---|---|---|---|
| 8 | 6.96196314 | 0.34666344 | 1.31959018 |
| 6 | 7.79919313 | 1.52313100 | 1.27509866 |
| 1 | 8.03217439 | 1.80225338 | 0.23806071 |
| 8 | 5.18904107 | -0.75669483 | 0.56692959 |
| 8 | 1.09614132 | -0.16115563 | -1.71768177 |
| 1 | 8.72398108 | 1.23138142 | 1.78258388 |
| 1 | 7.35281408 | 2.37303422 | 1.80788106 |
| 7 | 0.34251849 | -1.69503412 | 1.22118130 |
| 8 | -1.53637250 | -1.10868845 | -1.00208655 |
| 8 | -0.50257786 | 1.17726906 | 0.72582299 |

| | | | |
|---|---|---|---|
| 6 | 1.56714941 | -2.51250948 | 1.01235736 |
| 6 | -1.60456167 | -2.45599437 | -1.11425189 |
| 6 | -2.09596132 | -3.06990397 | -2.30322299 |
| 6 | -2.12467491 | -4.47212908 | -2.33369525 |
| 6 | -1.70758303 | -5.26658454 | -1.26105933 |
| 6 | -1.26170433 | -4.65471235 | -0.09364655 |
| 6 | -1.22208712 | -3.25955594 | -0.00859332 |
| 6 | -0.87921805 | -2.58143499 | 1.28729218 |
| 1 | -1.74434843 | -6.35319538 | -1.33926156 |
| 6 | -2.57904633 | -2.23305988 | -3.50337940 |
| 6 | -1.10718741 | 1.02906874 | 1.93066575 |
| 6 | -2.13718910 | 1.93762862 | 2.31709851 |
| 6 | -2.72704456 | 1.74899230 | 3.57463845 |
| 6 | -2.34975185 | 0.71959691 | 4.44111298 |
| 6 | -1.33790512 | -0.15288095 | 4.05748065 |
| 6 | -0.70072011 | -0.00272121 | 2.81943560 |
| 6 | 0.47290167 | -0.89744066 | 2.49882147 |
| 1 | -2.84517214 | 0.60549507 | 5.40523220 |
| 6 | -2.59142348 | 3.08362946 | 1.39070625 |
| 6 | -3.12477631 | -3.11786888 | -4.63998324 |
| 6 | -1.40649107 | -1.40656036 | -4.08144305 |
| 6 | -3.72468084 | -1.29017001 | -3.06407184 |
| 6 | -3.69317769 | 3.94474599 | 2.03721071 |
| 6 | -3.17130105 | 2.51067358 | 0.07582962 |
| 6 | -1.40056024 | 4.02237040 | 1.08241217 |
| 1 | 1.68929667 | -3.21357636 | 1.85383965 |
| 1 | 1.46659791 | -3.08258869 | 0.08231317 |
| 1 | -0.70902308 | -3.32812186 | 2.08094989 |
| 1 | -1.70439345 | -1.92734263 | 1.60422886 |
| 1 | -2.48358041 | -4.97182622 | -3.23191974 |
| 1 | -0.96067927 | -5.25427636 | 0.76876413 |
| 1 | 1.39290463 | -0.30967977 | 2.38528874 |
| 1 | 0.62101396 | -1.61347860 | 3.32439106 |
| 1 | -3.51963404 | 2.42376678 | 3.89273946 |
| 1 | -1.02011066 | -0.95756580 | 4.72456485 |
| 1 | -3.47088329 | -2.47197506 | -5.45966692 |
| 1 | -2.35542809 | -3.78770447 | -5.05091938 |
| 1 | -3.98020441 | -3.72789730 | -4.31487068 |
| 1 | -0.97813516 | -0.72815645 | -3.33609742 |
| 1 | -0.60592944 | -2.07047117 | -4.43926698 |
| 1 | -1.76002405 | -0.81002862 | -4.93694876 |
| 1 | -4.58095833 | -1.87008710 | -2.68924126 |
| 1 | -3.39883705 | -0.60177220 | -2.27686763 |
| 1 | -4.06900256 | -0.69856706 | -3.92625763 |
| 1 | -3.96989412 | 4.75015281 | 1.34194261 |
| 1 | -3.35643700 | 4.41570639 | 2.97222045 |
| 1 | -4.60295560 | 3.36415743 | 2.24748921 |
| 1 | -2.43050948 | 1.92054939 | -0.47327481 |
| 1 | -4.03807199 | 1.86696190 | 0.28355921 |
| 1 | -3.50853206 | 3.33473694 | -0.57139683 |
| 1 | -1.01844283 | 4.47823387 | 2.00777792 |
| 1 | -0.58093435 | 3.48226573 | 0.59636450 |
| 1 | -1.72983160 | 4.83476585 | 0.41657800 |

| | | | |
|---|---|---|---|
| 1 | 2.44216557 | -1.85749550 | 0.94940673 |
| 31 | -0.00454090 | -0.31721780 | -0.26201098 |
| 6 | 5.73142769 | 0.31171810 | 0.72259408 |
| 6 | 5.09866313 | 1.65860066 | 0.32084484 |
| 8 | 4.16059409 | 1.44522729 | -0.76874827 |
| 6 | 2.94645913 | 0.87744230 | -0.54743046 |
| 6 | 2.15166280 | 0.74929866 | -1.84874636 |
| 8 | 2.50831188 | 0.53890495 | 0.53777981 |
| 6 | 1.67070413 | 2.14318763 | -2.29619048 |
| 6 | 4.51751205 | 2.42806246 | 1.51347607 |
| 1 | 5.86432039 | 2.27987282 | -0.16391082 |
| 1 | 2.84811223 | 0.35456300 | -2.61058728 |
| 1 | 1.09253033 | 2.02963009 | -3.22150037 |
| 1 | 2.51695404 | 2.81794691 | -2.48451242 |
| 1 | 1.01842502 | 2.58604167 | -1.52998548 |
| 1 | 5.32878590 | 2.77941669 | 2.16439837 |
| 1 | 3.83128673 | 1.81099481 | 2.09812828 |
| 1 | 3.97688366 | 3.30998540 | 1.14564552 |

Electronic energy = -3673.039971 a.u.
DFT-D3(BJ) dispersion correction = -0.114872 a.u.
Thermal free energy = 0.581649 a.u.
Gibbs free energy = -3672.573194 a.u.
Number of imaginary frequencies = 0.

**Int2 LA isomer/conformer 60**

| Atomic N. | X | Y | Z |
|---|---|---|---|
| 8 | 1.66389312 | 2.59943154 | 6.56001879 |
| 6 | 3.05109972 | 2.95578080 | 6.80449740 |
| 1 | 3.71783619 | 2.18841137 | 6.39318334 |
| 8 | 2.09744160 | 2.65215832 | 4.32340645 |
| 8 | -0.71654400 | -0.51361933 | 1.53621181 |
| 1 | 3.14496692 | 3.01281696 | 7.89247550 |
| 1 | 3.28004925 | 3.92221523 | 6.33982759 |
| 7 | -1.98935136 | 1.35737233 | -1.11283897 |
| 8 | -0.89372468 | -1.39562841 | -1.17867123 |
| 8 | 0.98695840 | 0.96972354 | -0.71141333 |
| 6 | -3.18658491 | 1.68158523 | -0.29520094 |
| 6 | -2.13800371 | -1.73226803 | -1.58433866 |
| 6 | -2.59717729 | -3.08017125 | -1.50465486 |
| 6 | -3.90495493 | -3.33734816 | -1.94192577 |
| 6 | -4.74839824 | -2.34404294 | -2.45000177 |
| 6 | -4.27673658 | -1.03913466 | -2.55501219 |
| 6 | -2.97709839 | -0.73180774 | -2.14060371 |
| 6 | -2.40047421 | 0.63469409 | -2.37416454 |
| 1 | -5.75869905 | -2.59704925 | -2.77174148 |
| 6 | -1.69679507 | -4.20850036 | -0.96614691 |
| 6 | 1.06046573 | 1.64292538 | -1.88619663 |
| 6 | 2.25038921 | 1.56886873 | -2.67002919 |
| 6 | 2.27789178 | 2.29125614 | -3.87107337 |
| 6 | 1.20046073 | 3.06104172 | -4.31761443 |
| 6 | 0.04958710 | 3.13075076 | -3.54138244 |
| 6 | -0.02872926 | 2.44222587 | -2.32460802 |

| | | | |
|---|---|---|---|
| 6 | -1.25164028 | 2.63265669 | -1.46179485 |
| 1 | 1.26578246 | 3.59874342 | -5.26345185 |
| 6 | 3.46369426 | 0.72997906 | -2.22105545 |
| 6 | -2.38824344 | -5.58279155 | -1.04194564 |
| 6 | -1.34662199 | -3.94614883 | 0.51742865 |
| 6 | -0.40093628 | -4.29859989 | -1.80702506 |
| 6 | 4.62811958 | 0.81670374 | -3.22622412 |
| 6 | 3.07136644 | -0.76204229 | -2.10555603 |
| 6 | 3.99298301 | 1.24955949 | -0.86346216 |
| 1 | -3.86848507 | 2.32176401 | -0.87836633 |
| 1 | -3.70682652 | 0.75473700 | -0.02898284 |
| 1 | -3.12426378 | 1.27459591 | -2.90637286 |
| 1 | -1.49116572 | 0.56522437 | -2.98816911 |
| 1 | -4.28904000 | -4.35443183 | -1.88351518 |
| 1 | -4.90542993 | -0.25287191 | -2.97991016 |
| 1 | -0.98785314 | 3.09418918 | -0.50107348 |
| 1 | -1.96356441 | 3.29613073 | -1.98050304 |
| 1 | 3.17103515 | 2.24916219 | -4.49168800 |
| 1 | -0.80076910 | 3.73460726 | -3.86652054 |
| 1 | -1.69777829 | -6.35059785 | -0.66432175 |
| 1 | -3.29738344 | -5.62502806 | -0.42444258 |
| 1 | -2.65523130 | -5.85529699 | -2.07345523 |
| 1 | -0.82247859 | -2.99390699 | 0.65190818 |
| 1 | -2.25972702 | -3.92515843 | 1.13062037 |
| 1 | -0.70400705 | -4.75683216 | 0.89515206 |
| 1 | -0.63617830 | -4.52544694 | -2.85734512 |
| 1 | 0.16678632 | -3.36256590 | -1.77150854 |
| 1 | 0.23584938 | -5.10945064 | -1.42105802 |
| 1 | 5.46578031 | 0.21011357 | -2.85332682 |
| 1 | 4.99444132 | 1.84630142 | -3.34866070 |
| 1 | 4.35172706 | 0.42457571 | -4.21568827 |
| 1 | 2.27856992 | -0.91920926 | -1.36723837 |
| 1 | 2.72047606 | -1.14620137 | -3.07423020 |
| 1 | 3.94933632 | -1.35380537 | -1.80434228 |
| 1 | 4.33363325 | 2.29130226 | -0.95414355 |
| 1 | 3.22150026 | 1.20596174 | -0.08757168 |
| 1 | 4.85062884 | 0.64008925 | -0.53976958 |
| 1 | -2.87274969 | 2.20660317 | 0.61203685 |
| 31 | -0.59865864 | 0.16333451 | -0.16821619 |
| 6 | 1.33453044 | 2.47935152 | 5.24817082 |
| 6 | -0.15289737 | 2.10611099 | 5.13377199 |
| 8 | -0.60922802 | 2.36118182 | 3.78643833 |
| 6 | -0.48288092 | 1.60147118 | 2.66251754 |
| 6 | -0.12629163 | 0.10197875 | 2.65317529 |
| 8 | -0.76756907 | 2.15777069 | 1.61228866 |
| 6 | 1.39583058 | -0.12005034 | 2.67687486 |
| 6 | -0.47870581 | 0.75426363 | 5.77487045 |
| 1 | -0.70076252 | 2.87549635 | 5.69791357 |
| 1 | -0.58198570 | -0.36373678 | 3.54009094 |
| 1 | 1.57536687 | -1.19844057 | 2.58125506 |
| 1 | 1.87212406 | 0.24543715 | 3.59448202 |
| 1 | 1.86267604 | 0.39323431 | 1.82576474 |
| 1 | -0.35133658 | 0.85954875 | 6.85977788 |

| | | | |
|---|---|---|---|
| 1 | 0.17518453 | -0.05651852 | 5.43585746 |
| 1 | -1.52450092 | 0.48526478 | 5.57853741 |

Electronic energy = -3673.037118 a.u.
DFT-D3(BJ) dispersion correction = -0.114935 a.u.
Thermal free energy = 0.579480 a.u.
Gibbs free energy = -3672.572572 a.u.
Number of imaginary frequencies = 0.

**Int2 LA isomer/conformer 61**

| Atomic N. | X | Y | Z |
|---|---|---|---|
| 8 | -3.43062884 | 2.23019425 | -3.23888913 |
| 6 | -2.81538461 | 3.26463902 | -4.05223955 |
| 1 | -3.54867529 | 3.67801751 | -4.75502931 |
| 8 | -4.89646304 | 3.78378742 | -2.44703004 |
| 8 | -1.69038538 | 0.30671462 | 1.00360652 |
| 1 | -2.00034211 | 2.76340036 | -4.58116865 |
| 1 | -2.43063426 | 4.06817404 | -3.41269398 |
| 7 | -0.23665338 | -2.67153857 | -0.14296268 |
| 8 | 0.93811622 | -0.61927250 | 1.63702348 |
| 8 | 0.46307448 | 0.03752326 | -1.31158442 |
| 6 | -1.45675080 | -3.41965616 | 0.25589890 |
| 6 | 0.99829319 | -1.62780226 | 2.53498903 |
| 6 | 1.15462056 | -1.36379136 | 3.92768053 |
| 6 | 1.19865115 | -2.47146092 | 4.78723580 |
| 6 | 1.10764840 | -3.79194869 | 4.33527762 |
| 6 | 0.99116933 | -4.03764765 | 2.97068940 |
| 6 | 0.95132587 | -2.96800359 | 2.07084278 |
| 6 | 0.96985690 | -3.21056591 | 0.58950762 |
| 1 | 1.14194206 | -4.61667459 | 5.04719185 |
| 6 | 1.27460345 | 0.07400558 | 4.46820818 |
| 6 | 1.35810895 | -0.68813528 | -2.02497993 |
| 6 | 2.45789258 | -0.03111662 | -2.65340926 |
| 6 | 3.35549916 | -0.82472002 | -3.38094824 |
| 6 | 3.21162492 | -2.20896260 | -3.50690439 |
| 6 | 2.12848737 | -2.83561354 | -2.90166539 |
| 6 | 1.19150596 | -2.09110131 | -2.17454024 |
| 6 | -0.03099024 | -2.79485700 | -1.63734992 |
| 1 | 3.94088466 | -2.78644108 | -4.07486928 |
| 6 | 2.65645857 | 1.49394578 | -2.54283620 |
| 6 | 1.50627759 | 0.09956084 | 5.99073471 |
| 6 | -0.02749439 | 0.85861685 | 4.18329151 |
| 6 | 2.47753125 | 0.78907181 | 3.80775294 |
| 6 | 3.89862715 | 1.97240083 | -3.31833521 |
| 6 | 2.85411670 | 1.90525613 | -1.06467309 |
| 6 | 1.43368343 | 2.22936718 | -3.14248128 |
| 1 | -1.33612421 | -4.48521221 | 0.00146306 |
| 1 | -1.60312465 | -3.32470395 | 1.33763628 |
| 1 | 1.03723678 | -4.29027288 | 0.37446782 |
| 1 | 1.84000727 | -2.71712749 | 0.13319875 |
| 1 | 1.30559768 | -2.30298833 | 5.85752659 |
| 1 | 0.95278749 | -5.06145851 | 2.59079780 |
| 1 | -0.94587535 | -2.39760851 | -2.09631451 |

| | | | |
|---|---|---|---|
| 1 | 0.03514448 | -3.86976424 | -1.87462279 |
| 1 | 4.20762689 | -0.35062235 | -3.86448559 |
| 1 | 1.98982769 | -3.91482737 | -2.99906325 |
| 1 | 1.60301355 | 1.14436469 | 6.31912633 |
| 1 | 0.66699507 | -0.34373145 | 6.54627740 |
| 1 | 2.42937913 | -0.42498199 | 6.27760728 |
| 1 | -0.24815214 | 0.91016280 | 3.11174605 |
| 1 | -0.88191921 | 0.38316054 | 4.68729156 |
| 1 | 0.06597960 | 1.88477539 | 4.57196148 |
| 1 | 3.41510736 | 0.26186191 | 4.03789087 |
| 1 | 2.36540674 | 0.84156465 | 2.71944003 |
| 1 | 2.56122232 | 1.81375115 | 4.20133850 |
| 1 | 3.98489038 | 3.06358305 | -3.21537747 |
| 1 | 3.83050490 | 1.74538499 | -4.39215394 |
| 1 | 4.82611188 | 1.53040414 | -2.92687236 |
| 1 | 1.98768352 | 1.64319816 | -0.44909192 |
| 1 | 3.73903496 | 1.40892075 | -0.64121401 |
| 1 | 3.01242566 | 2.99265748 | -0.99943413 |
| 1 | 1.32994850 | 1.99376005 | -4.21212878 |
| 1 | 0.50775885 | 1.94307318 | -2.63169842 |
| 1 | 1.56881425 | 3.31770786 | -3.04588925 |
| 1 | -2.32145247 | -3.00864713 | -0.27388637 |
| 31 | -0.31672903 | -0.64736596 | 0.23588408 |
| 6 | -4.45708625 | 2.64984940 | -2.47258588 |
| 6 | -5.09018370 | 1.50299985 | -1.66720095 |
| 8 | -4.26148938 | 0.30870934 | -1.62453081 |
| 6 | -3.10759680 | 0.12045740 | -0.93599091 |
| 6 | -2.64316565 | 0.99972198 | 0.24377988 |
| 8 | -2.45478736 | -0.86745564 | -1.24451097 |
| 6 | -2.09415179 | 2.36849785 | -0.20065318 |
| 6 | -5.70193566 | 1.97131702 | -0.34991625 |
| 1 | -5.91789590 | 1.15199698 | -2.30525166 |
| 1 | -3.52284447 | 1.15378568 | 0.89256819 |
| 1 | -1.65931891 | 2.84401265 | 0.68725932 |
| 1 | -2.86309651 | 3.04183510 | -0.60155370 |
| 1 | -1.30266539 | 2.23963766 | -0.95035166 |
| 1 | -6.52585062 | 2.65675397 | -0.58226584 |
| 1 | -4.99500455 | 2.51247603 | 0.28727697 |
| 1 | -6.10765249 | 1.11102046 | 0.19716567 |

Electronic energy = -3673.038861 a.u.
DFT-D3(BJ) dispersion correction = -0.116507 a.u.
Thermal free energy = 0.583231 a.u.
Gibbs free energy = -3672.572138 a.u.
Number of imaginary frequencies = 0.

**Int2 LA isomer/conformer 62**

| Atomic N. | X | Y | Z |
|---|---|---|---|
| 8 | -3.86595424 | 4.49119539 | -0.22600717 |
| 6 | -3.82054822 | 3.20722307 | 0.43749592 |
| 1 | -3.35830381 | 2.45193041 | -0.20828675 |
| 8 | -2.93306643 | 6.39221787 | -0.86592591 |
| 8 | -0.12153840 | 1.14544523 | 2.09245935 |

| | | | |
|---|---|---|---|
| 1 | -4.86804098 | 2.94407006 | 0.61403984 |
| 1 | -3.30251203 | 3.26519480 | 1.40457528 |
| 7 | 0.33306944 | -1.73089870 | 2.18536762 |
| 8 | 1.52547640 | -0.52933694 | -0.24867488 |
| 8 | -1.63798858 | -0.83420043 | 0.14349655 |
| 6 | 1.00989327 | -1.27285120 | 3.42322477 |
| 6 | 2.62351146 | -1.20646174 | 0.15806182 |
| 6 | 3.90433188 | -0.84136355 | -0.35669725 |
| 6 | 5.01374570 | -1.56803741 | 0.09804913 |
| 6 | 4.90826962 | -2.62017565 | 1.01209809 |
| 6 | 3.65213670 | -2.98386464 | 1.48491530 |
| 6 | 2.51007531 | -2.29813666 | 1.05694239 |
| 6 | 1.15179136 | -2.77925520 | 1.48823078 |
| 1 | 5.80106823 | -3.15251043 | 1.34009767 |
| 6 | 4.06703147 | 0.29445236 | -1.38664108 |
| 6 | -2.08403834 | -2.10499052 | 0.24592538 |
| 6 | -2.87922525 | -2.66479854 | -0.79924999 |
| 6 | -3.33082052 | -3.98104423 | -0.63042280 |
| 6 | -3.03129545 | -4.74704807 | 0.50021393 |
| 6 | -2.25835488 | -4.18952028 | 1.51241930 |
| 6 | -1.79264912 | -2.87427301 | 1.40091647 |
| 6 | -1.03016844 | -2.26238459 | 2.54483093 |
| 1 | -3.39991627 | -5.76949017 | 0.58214070 |
| 6 | -3.22649659 | -1.86023364 | -2.06729994 |
| 6 | 5.53545859 | 0.48369293 | -1.81112257 |
| 6 | 3.58525357 | 1.63618887 | -0.78670171 |
| 6 | 3.25447043 | -0.02919171 | -2.66318240 |
| 6 | -4.10892118 | -2.66630553 | -3.03887918 |
| 6 | -1.93399260 | -1.47765142 | -2.82663068 |
| 6 | -4.01174346 | -0.58267436 | -1.68624523 |
| 1 | 1.13901838 | -2.12222103 | 4.11514115 |
| 1 | 1.99376434 | -0.86140452 | 3.17485974 |
| 1 | 1.25609857 | -3.64882965 | 2.16005485 |
| 1 | 0.55986212 | -3.10304054 | 0.61914689 |
| 1 | 6.00360253 | -1.30632308 | -0.27143436 |
| 1 | 3.54231239 | -3.81860607 | 2.18109509 |
| 1 | -1.57659646 | -1.40408522 | 2.96838007 |
| 1 | -0.90653959 | -3.01224472 | 3.34505978 |
| 1 | -3.93593315 | -4.43561955 | -1.41276964 |
| 1 | -2.01648106 | -4.76749492 | 2.40736384 |
| 1 | 5.59142501 | 1.29564356 | -2.55020405 |
| 1 | 6.18062556 | 0.76129077 | -0.96476420 |
| 1 | 5.94873716 | -0.41996688 | -2.28159606 |
| 1 | 2.52392114 | 1.58522983 | -0.52198716 |
| 1 | 4.16705245 | 1.89088605 | 0.11187567 |
| 1 | 3.72220005 | 2.44434765 | -1.52140054 |
| 1 | 3.61336472 | -0.96203518 | -3.12174765 |
| 1 | 2.18677680 | -0.13598195 | -2.44343967 |
| 1 | 3.38040644 | 0.77936521 | -3.39944461 |
| 1 | -4.33297861 | -2.04407924 | -3.91700708 |
| 1 | -5.06774244 | -2.95629102 | -2.58501510 |
| 1 | -3.60508319 | -3.57499465 | -3.39872587 |
| 1 | -1.27677283 | -0.84790189 | -2.21828790 |

| | | | |
|---|---|---|---|
| 1 | -1.37799055 | -2.37918292 | -3.12215961 |
| 1 | -2.19111048 | -0.92385972 | -3.74236568 |
| 1 | -4.95757534 | -0.84293168 | -1.18833251 |
| 1 | -3.42317975 | 0.05206672 | -1.01484878 |
| 1 | -4.25248289 | -0.00809762 | -2.59360459 |
| 1 | 0.40449743 | -0.49174810 | 3.89373248 |
| 31 | 0.00306808 | -0.19644730 | 0.78497333 |
| 6 | -2.78994085 | 5.30745436 | -0.34629906 |
| 6 | -1.40926885 | 4.86264866 | 0.21177759 |
| 8 | -1.05290839 | 3.60838029 | -0.45505019 |
| 6 | -0.57398449 | 2.53204650 | 0.18883245 |
| 6 | -0.29753060 | 2.47060809 | 1.70365668 |
| 8 | -0.36787104 | 1.53256579 | -0.50278381 |
| 6 | 0.93732081 | 3.31041881 | 2.09153805 |
| 6 | -0.35943738 | 5.91424091 | -0.10301916 |
| 1 | -1.50097317 | 4.69559559 | 1.29546715 |
| 1 | -1.18409250 | 2.89061513 | 2.22361954 |
| 1 | 1.15007653 | 3.09970796 | 3.14670565 |
| 1 | 0.77914113 | 4.39089410 | 1.98136004 |
| 1 | 1.80945870 | 3.00929091 | 1.49672344 |
| 1 | -0.61590395 | 6.85457155 | 0.39945986 |
| 1 | -0.33104552 | 6.10555792 | -1.18129106 |
| 1 | 0.63068411 | 5.58504871 | 0.23322137 |

Electronic energy = -3673.038275 a.u.
DFT-D3(BJ) dispersion correction = -0.116252 a.u.
Thermal free energy = 0.582402 a.u.
Gibbs free energy = -3672.572125 a.u.
Number of imaginary frequencies = 0.

**Int2 LA isomer/conformer 63**

| Atomic N. | X | Y | Z |
|---|---|---|---|
| 7 | -0.97553810 | 1.55730559 | 1.28200705 |
| 8 | -0.52974684 | 0.37305130 | -1.44192947 |
| 8 | 1.52858095 | -0.02787739 | 0.86528696 |
| 6 | -2.36117317 | 1.43229866 | 1.80555750 |
| 6 | -1.45962426 | 1.29606453 | -1.80947273 |
| 6 | -2.14237231 | 1.15955485 | -3.05389891 |
| 6 | -3.09556510 | 2.13754707 | -3.37346165 |
| 6 | -3.38580242 | 3.21864020 | -2.53716642 |
| 6 | -2.69097571 | 3.35624212 | -1.34110269 |
| 6 | -1.72087082 | 2.41544305 | -0.97693541 |
| 6 | -0.88710896 | 2.65734175 | 0.25222729 |
| 1 | -4.14036986 | 3.94910621 | -2.82838097 |
| 6 | -1.84944383 | -0.00551062 | -4.02008305 |
| 6 | 2.13269173 | 1.11485920 | 1.28025596 |
| 6 | 3.51050295 | 1.33460034 | 0.98742126 |
| 6 | 4.08428804 | 2.52453223 | 1.45726900 |
| 6 | 3.36531216 | 3.47952712 | 2.18139622 |
| 6 | 2.02378030 | 3.25035458 | 2.46324463 |
| 6 | 1.40293064 | 2.07179062 | 2.03156591 |
| 6 | -0.02561848 | 1.81272773 | 2.43544215 |
| 1 | 3.85479803 | 4.39272215 | 2.51933787 |

| | | | |
|---|---|---|---|
| 6 | 4.34234807 | 0.31171962 | 0.18832637 |
| 6 | -2.67280401 | 0.09870902 | -5.31792910 |
| 6 | -2.21486724 | -1.35104030 | -3.35285519 |
| 6 | -0.35585837 | 0.00225082 | -4.42452786 |
| 6 | 5.80300972 | 0.76880982 | 0.01322336 |
| 6 | 3.74673596 | 0.12946756 | -1.22776183 |
| 6 | 4.37299624 | -1.04455215 | 0.93231980 |
| 1 | -2.66840314 | 2.38914164 | 2.25749552 |
| 1 | -3.04267952 | 1.19421161 | 0.98128001 |
| 1 | -1.18988030 | 3.60053229 | 0.73568879 |
| 1 | 0.17815946 | 2.74415135 | -0.00869732 |
| 1 | -3.63900703 | 2.05481876 | -4.31272349 |
| 1 | -2.87956230 | 4.20903028 | -0.68510639 |
| 1 | -0.11133968 | 0.92732554 | 3.08540144 |
| 1 | -0.40996870 | 2.67927071 | 2.99763693 |
| 1 | 5.13350334 | 2.72259246 | 1.24671550 |
| 1 | 1.44558087 | 3.97970393 | 3.03484112 |
| 1 | -2.41393039 | -0.74588309 | -5.97204498 |
| 1 | -3.75496787 | 0.04900089 | -5.12941996 |
| 1 | -2.45689877 | 1.02396176 | -5.87125802 |
| 1 | -1.64137251 | -1.50924702 | -2.43397820 |
| 1 | -3.28568613 | -1.38432681 | -3.10462132 |
| 1 | -2.00115686 | -2.18301299 | -4.04040077 |
| 1 | -0.10245959 | 0.93805906 | -4.94357130 |
| 1 | 0.30177173 | -0.09857483 | -3.55452359 |
| 1 | -0.15369177 | -0.83112478 | -5.11416373 |
| 1 | 6.35001576 | 0.00304101 | -0.55475140 |
| 1 | 6.31643938 | 0.89404758 | 0.97746029 |
| 1 | 5.87835415 | 1.71166731 | -0.54750585 |
| 1 | 2.71761687 | -0.24244096 | -1.19091360 |
| 1 | 3.75049661 | 1.08277222 | -1.77580319 |
| 1 | 4.35451198 | -0.58996483 | -1.79692440 |
| 1 | 4.84333710 | -0.93308864 | 1.92015290 |
| 1 | 3.36546716 | -1.45044886 | 1.07204486 |
| 1 | 4.96549584 | -1.77150660 | 0.35650399 |
| 1 | -2.38893022 | 0.64052364 | 2.56307265 |
| 8 | -3.02009403 | -2.79879094 | 2.87516257 |
| 6 | -4.20588941 | -1.97258685 | 2.91235754 |
| 1 | -4.15156602 | -1.20792181 | 2.12694997 |
| 8 | -1.82996942 | -1.12097554 | 3.85619853 |
| 8 | -1.02453052 | -1.64705331 | 0.94120081 |
| 1 | -5.03848151 | -2.65433339 | 2.71619523 |
| 1 | -4.31267152 | -1.49269754 | 3.89269593 |
| 31 | -0.25409570 | -0.09183172 | 0.34090059 |
| 6 | -1.89045800 | -2.23005065 | 3.35039504 |
| 6 | -0.72877285 | -3.22502348 | 3.34132472 |
| 8 | -0.85987140 | -4.27826541 | 2.35277379 |
| 6 | -1.09752805 | -4.15380280 | 1.00897201 |
| 6 | -0.92500926 | -2.85150460 | 0.19177174 |
| 8 | -1.37303874 | -5.17049586 | 0.40586644 |
| 6 | 0.40046625 | -2.98342794 | -0.57785133 |
| 6 | 0.65272531 | -2.59051386 | 3.39586290 |
| 1 | -0.87028969 | -3.79430833 | 4.27827950 |

| | | | |
|---|---|---|---|
| 1 | -1.75425350 | -2.90602252 | -0.53495635 |
| 1 | 0.49147887 | -2.16402882 | -1.30808033 |
| 1 | 0.42644807 | -3.92952705 | -1.13227175 |
| 1 | 1.25793027 | -2.94445081 | 0.10766221 |
| 1 | 0.71605208 | -1.94060083 | 4.27815717 |
| 1 | 0.86354118 | -1.98986879 | 2.50568080 |
| 1 | 1.40682684 | -3.38183552 | 3.49348974 |

Electronic energy = -3673.035593 a.u.
DFT-D3(BJ) dispersion correction = -0.119476 a.u.
Thermal free energy = 0.583138 a.u.
Gibbs free energy = -3672.571931 a.u.
Number of imaginary frequencies = 0.

**Int2 LA isomer/conformer 64**

| Atomic N. | X | Y | Z |
|---|---|---|---|
| 8 | 2.56713084 | -3.77869519 | 0.81620898 |
| 6 | 3.70707295 | -2.88765094 | 0.94523764 |
| 1 | 3.41691358 | -1.87541623 | 0.64093709 |
| 8 | 1.55413472 | -2.67585354 | 2.52654219 |
| 8 | -0.53031191 | -2.06330655 | 0.32903365 |
| 1 | 4.46257880 | -3.29145345 | 0.26528440 |
| 1 | 4.07099277 | -2.88345288 | 1.97983177 |
| 7 | 0.19793719 | 0.59053676 | 2.21023696 |
| 8 | -1.20921262 | 0.91202474 | -0.41973479 |
| 8 | 1.67930187 | -0.18458033 | -0.30354383 |
| 6 | -0.45053224 | -0.13379342 | 3.33776395 |
| 6 | -2.13013194 | 1.59107403 | 0.31676149 |
| 6 | -3.40916062 | 1.89158671 | -0.23439520 |
| 6 | -4.32985094 | 2.53772719 | 0.60428738 |
| 6 | -4.02942238 | 2.91542346 | 1.91578191 |
| 6 | -2.74998870 | 2.68909063 | 2.41152784 |
| 6 | -1.79230464 | 2.05059345 | 1.61628756 |
| 6 | -0.36190758 | 1.98375329 | 2.07699050 |
| 1 | -4.78474635 | 3.40423119 | 2.53079109 |
| 6 | -3.75877445 | 1.57032626 | -1.70118958 |
| 6 | 2.46305011 | 0.87800087 | 0.02850915 |
| 6 | 3.29093393 | 1.48551797 | -0.95878889 |
| 6 | 4.09573950 | 2.55826917 | -0.54773306 |
| 6 | 4.10397307 | 3.03977626 | 0.76407234 |
| 6 | 3.29277514 | 2.43459517 | 1.71730012 |
| 6 | 2.48012771 | 1.34929900 | 1.36713419 |
| 6 | 1.69405205 | 0.64300294 | 2.44414125 |
| 1 | 4.74135102 | 3.88192499 | 1.03320069 |
| 6 | 3.30267145 | 0.99637446 | -2.42137619 |
| 6 | -5.17759141 | 2.04167730 | -2.07133450 |
| 6 | -3.69535384 | 0.04943017 | -1.96283274 |
| 6 | -2.76699961 | 2.30160150 | -2.63770492 |
| 6 | 4.29890793 | 1.79355398 | -3.28480999 |
| 6 | 1.90178000 | 1.17527448 | -3.05281065 |
| 6 | 3.72595702 | -0.49042142 | -2.48782714 |
| 1 | -0.24590838 | 0.39941191 | 4.27987473 |
| 1 | -1.53413038 | -0.16978973 | 3.18033352 |

| | | | |
|---|---|---|---|
| 1 | -0.25187785 | 2.48658589 | 3.05220198 |
| 1 | 0.29015272 | 2.49986571 | 1.35668149 |
| 1 | -5.32451470 | 2.75902219 | 0.22200518 |
| 1 | -2.47452511 | 3.02679955 | 3.41309210 |
| 1 | 2.00954237 | -0.40581027 | 2.54695078 |
| 1 | 1.86081878 | 1.14673036 | 3.41053653 |
| 1 | 4.73745409 | 3.04690900 | -1.27837224 |
| 1 | 3.29142847 | 2.79083715 | 2.74977913 |
| 1 | -5.36578052 | 1.80904663 | -3.12905772 |
| 1 | -5.95167694 | 1.53288227 | -1.47861782 |
| 1 | -5.29931272 | 3.12698295 | -1.94508361 |
| 1 | -2.68347393 | -0.33678715 | -1.80087634 |
| 1 | -4.39203480 | -0.48852454 | -1.30328246 |
| 1 | -3.98335521 | -0.16225892 | -3.00379295 |
| 1 | -2.83236975 | 3.39019612 | -2.49695217 |
| 1 | -1.73339949 | 1.98740672 | -2.45315183 |
| 1 | -3.01453899 | 2.08100929 | -3.68717863 |
| 1 | 4.27478446 | 1.40071059 | -4.31108540 |
| 1 | 5.33173130 | 1.69989721 | -2.91901965 |
| 1 | 4.04232061 | 2.86148946 | -3.33512400 |
| 1 | 1.14005999 | 0.59022091 | -2.52688608 |
| 1 | 1.59987782 | 2.23228747 | -3.03477145 |
| 1 | 1.92322547 | 0.84673033 | -4.10292965 |
| 1 | 4.73353937 | -0.62478188 | -2.06691544 |
| 1 | 3.02734385 | -1.13281768 | -1.94099377 |
| 1 | 3.75292878 | -0.82154831 | -3.53697215 |
| 1 | -0.04499128 | -1.14967710 | 3.39797899 |
| 31 | -0.03656185 | -0.29980293 | 0.38015830 |
| 6 | 1.55154833 | -3.54738918 | 1.67627488 |
| 6 | 0.47363285 | -4.62465740 | 1.50883147 |
| 8 | -0.69518005 | -4.32889066 | 2.30661023 |
| 6 | -1.66834002 | -3.37687150 | 2.12259076 |
| 6 | -1.77996474 | -2.47319393 | 0.87367672 |
| 8 | -2.52294968 | -3.30561168 | 2.98301399 |
| 6 | -2.66618578 | -3.17159836 | -0.17079166 |
| 6 | 0.22181560 | -5.07656660 | 0.07287585 |
| 1 | 0.88364178 | -5.49180742 | 2.05660128 |
| 1 | -2.35816136 | -1.61389733 | 1.25504481 |
| 1 | -2.82585027 | -2.49102946 | -1.01548153 |
| 1 | -3.64001310 | -3.41879809 | 0.27299381 |
| 1 | -2.20637307 | -4.09159061 | -0.54758179 |
| 1 | 1.12235857 | -5.57717586 | -0.30335163 |
| 1 | -0.00601724 | -4.23170639 | -0.58368273 |
| 1 | -0.60181041 | -5.80248075 | 0.06006553 |

Electronic energy = -3673.036455 a.u.
DFT-D3(BJ) dispersion correction = -0.120058 a.u.
Thermal free energy = 0.584706 a.u.
Gibbs free energy = -3672.571807 a.u.
Number of imaginary frequencies = 0.

**Int2 LA isomer/conformer 65**

Atomic N.     X     Y     Z

| | | | |
|---|---|---|---|
| 8 | 1.30885761 | -0.25170425 | -6.16947656 |
| 6 | 1.41678431 | 1.07098706 | -6.74221012 |
| 1 | 0.81518204 | 1.79798404 | -6.17863785 |
| 8 | 1.47942743 | -1.59479746 | -4.40366044 |
| 8 | -1.00735347 | 0.87230131 | -1.79101587 |
| 1 | 1.00650047 | 0.97532007 | -7.75226190 |
| 1 | 2.45963555 | 1.40895869 | -6.81004983 |
| 7 | -2.75541190 | 0.12376774 | 0.40887095 |
| 8 | -0.07880917 | 0.96381949 | 1.38266335 |
| 8 | -0.55558162 | -1.80803578 | -0.11433536 |
| 6 | -3.50201488 | 1.23936155 | -0.22084824 |
| 6 | -0.80710005 | 1.76132077 | 2.19474440 |
| 6 | -0.17788007 | 2.86897459 | 2.84101188 |
| 6 | -0.97693808 | 3.67863385 | 3.66043553 |
| 6 | -2.33689809 | 3.43516416 | 3.87218592 |
| 6 | -2.92931592 | 2.33175980 | 3.26763881 |
| 6 | -2.17665135 | 1.48585907 | 2.44579621 |
| 6 | -2.80342738 | 0.22971448 | 1.90497751 |
| 1 | -2.91869837 | 4.09656306 | 4.51422047 |
| 6 | 1.32791536 | 3.15381218 | 2.67044973 |
| 6 | -1.38017466 | -2.67620659 | 0.50611942 |
| 6 | -0.85321517 | -3.88782000 | 1.04731232 |
| 6 | -1.75642108 | -4.76098825 | 1.66875596 |
| 6 | -3.12421836 | -4.48966493 | 1.77556523 |
| 6 | -3.62596719 | -3.30961941 | 1.23849850 |
| 6 | -2.76978305 | -2.40797229 | 0.59549753 |
| 6 | -3.34589499 | -1.18188112 | -0.05884850 |
| 1 | -3.78555200 | -5.19804360 | 2.27457528 |
| 6 | 0.64763514 | -4.22558347 | 0.95249857 |
| 6 | 1.77974239 | 4.38116562 | 3.48389749 |
| 6 | 1.66052359 | 3.43700420 | 1.18706207 |
| 6 | 2.15108398 | 1.94061467 | 3.16579263 |
| 6 | 0.97076043 | -5.58911649 | 1.59235280 |
| 6 | 1.48341939 | -3.15807946 | 1.69808061 |
| 6 | 1.08434320 | -4.29728419 | -0.53024616 |
| 1 | -4.55916452 | 1.20599914 | 0.09309542 |
| 1 | -3.06693368 | 2.19550477 | 0.08827258 |
| 1 | -3.85658231 | 0.16505497 | 2.22958867 |
| 1 | -2.28441618 | -0.65995741 | 2.29186525 |
| 1 | -0.52567906 | 4.53593814 | 4.15676319 |
| 1 | -3.98339275 | 2.10366340 | 3.44234538 |
| 1 | -3.18155351 | -1.20373771 | -1.14846535 |
| 1 | -4.43474650 | -1.14923670 | 0.11863137 |
| 1 | -1.38302123 | -5.69079697 | 2.09422557 |
| 1 | -4.69233248 | -3.08053536 | 1.30229470 |
| 1 | 2.85892833 | 4.53004690 | 3.33550153 |
| 1 | 1.27099278 | 5.30228882 | 3.16371808 |
| 1 | 1.61006743 | 4.24728265 | 4.56185475 |
| 1 | 1.43014853 | 2.56586273 | 0.56500853 |
| 1 | 1.08955573 | 4.30288645 | 0.81918604 |
| 1 | 2.73208923 | 3.66618063 | 1.08173093 |
| 1 | 1.95615260 | 1.75213305 | 4.23150832 |
| 1 | 1.90872754 | 1.03421785 | 2.60042157 |

| | | | |
|---|---|---|---|
| 1 | 3.22618283 | 2.14735549 | 3.05003729 |
| 1 | 2.04816603 | -5.78305376 | 1.49130488 |
| 1 | 0.43838628 | -6.41495846 | 1.09830131 |
| 1 | 0.73025179 | -5.61183287 | 2.66535956 |
| 1 | 1.35443636 | -2.16347641 | 1.25932026 |
| 1 | 1.19742997 | -3.11281359 | 2.75923917 |
| 1 | 2.55095545 | -3.42131182 | 1.64630060 |
| 1 | 0.52567217 | -5.08239928 | -1.06067574 |
| 1 | 0.92234928 | -3.34544970 | -1.04655253 |
| 1 | 2.15484787 | -4.54677342 | -0.58916013 |
| 1 | -3.42867328 | 1.14919714 | -1.30914629 |
| 31 | -0.72038517 | 0.05286562 | -0.12410095 |
| 6 | 1.71817307 | -0.51722202 | -4.89286046 |
| 6 | 2.54022485 | 0.56635763 | -4.16573296 |
| 8 | 2.49042409 | 0.34950218 | -2.73230376 |
| 6 | 1.34272114 | 0.46200176 | -2.04061310 |
| 6 | 0.09006780 | 1.15018705 | -2.59947556 |
| 8 | 1.34301022 | 0.04599949 | -0.88282379 |
| 6 | 0.32750542 | 2.67709264 | -2.67116339 |
| 6 | 4.01779193 | 0.43822183 | -4.53563627 |
| 1 | 2.17017763 | 1.57250472 | -4.39866846 |
| 1 | -0.09562177 | 0.75946631 | -3.62040980 |
| 1 | -0.58948719 | 3.14133026 | -3.05473303 |
| 1 | 1.16527871 | 2.96821318 | -3.32130138 |
| 1 | 0.51694248 | 3.06729722 | -1.66306036 |
| 1 | 4.16765626 | 0.56989504 | -5.61468382 |
| 1 | 4.38115307 | -0.55616630 | -4.24930800 |
| 1 | 4.60101871 | 1.19888561 | -4.00241221 |

Electronic energy = -3673.038522 a.u.
DFT-D3(BJ) dispersion correction = -0.115381 a.u.
Thermal free energy = 0.582236 a.u.
Gibbs free energy = -3672.571667 a.u.
Number of imaginary frequencies = 0.

**Int2 LA isomer/conformer 66**

| Atomic N. | X | Y | Z |
|---|---|---|---|
| 7 | 0.94479772 | -1.88184171 | 1.34765591 |
| 8 | -0.87266908 | -0.11756132 | -0.30358776 |
| 8 | -0.40677293 | 0.43695419 | 2.65561153 |
| 6 | 2.26996811 | -2.29352295 | 0.81960804 |
| 6 | -0.82181726 | -1.15309603 | -1.18433565 |
| 6 | -1.26272316 | -0.96566389 | -2.52421725 |
| 6 | -1.14028234 | -2.05610369 | -3.39762788 |
| 6 | -0.63749370 | -3.29594786 | -2.99563370 |
| 6 | -0.27474925 | -3.48418240 | -1.66655392 |
| 6 | -0.38018842 | -2.43037123 | -0.75246161 |
| 6 | -0.15848270 | -2.69472634 | 0.71283231 |
| 1 | -0.54622670 | -4.10831391 | -3.71620443 |
| 6 | -1.88397743 | 0.36591424 | -2.99225003 |
| 6 | -0.97750775 | -0.51212756 | 3.42595081 |
| 6 | -2.16529088 | -0.22338916 | 4.16203401 |
| 6 | -2.69722961 | -1.25193313 | 4.95169695 |

| | | | |
|---|---|---|---|
| 6 | -2.11666742 | -2.52151964 | 5.03916284 |
| 6 | -0.95303045 | -2.78736764 | 4.32664976 |
| 6 | -0.37264811 | -1.79106046 | 3.53244678 |
| 6 | 0.94235332 | -2.06050531 | 2.85350803 |
| 1 | -2.57355239 | -3.29019129 | 5.66230700 |
| 6 | -2.83823648 | 1.16106347 | 4.09137347 |
| 6 | -2.30563636 | 0.31327535 | -4.47283309 |
| 6 | -0.87237245 | 1.52580733 | -2.84753880 |
| 6 | -3.15108957 | 0.66765121 | -2.15699270 |
| 6 | -4.08156057 | 1.24334911 | 4.99665582 |
| 6 | -3.30175389 | 1.45225814 | 2.64432074 |
| 6 | -1.85167334 | 2.25644406 | 4.56206019 |
| 1 | 2.47286016 | -3.33540779 | 1.11577354 |
| 1 | 2.27424016 | -2.20737092 | -0.26979165 |
| 1 | 0.06884596 | -3.76141845 | 0.87584599 |
| 1 | -1.06730130 | -2.45604022 | 1.28479472 |
| 1 | -1.44155532 | -1.93721032 | -4.43655428 |
| 1 | 0.07629859 | -4.45901651 | -1.32031438 |
| 1 | 1.72461579 | -1.38058700 | 3.22824793 |
| 1 | 1.26185650 | -3.09406265 | 3.06581112 |
| 1 | -3.60628401 | -1.06359641 | 5.52017712 |
| 1 | -0.47567190 | -3.76800811 | 4.38981903 |
| 1 | -2.75702159 | 1.27669927 | -4.75041067 |
| 1 | -1.44839701 | 0.14191875 | -5.13976487 |
| 1 | -3.05530614 | -0.46840962 | -4.66122274 |
| 1 | -0.59288200 | 1.67794312 | -1.79940542 |
| 1 | 0.03314600 | 1.31348641 | -3.43250572 |
| 1 | -1.31930307 | 2.45835095 | -3.22571601 |
| 1 | -3.89930447 | -0.12718654 | -2.28935538 |
| 1 | -2.91833828 | 0.75174952 | -1.08933051 |
| 1 | -3.60187862 | 1.61499429 | -2.49060785 |
| 1 | -4.51434842 | 2.25081628 | 4.91747121 |
| 1 | -3.83506689 | 1.07131691 | 6.05449293 |
| 1 | -4.86010945 | 0.52598636 | 4.69900548 |
| 1 | -2.46110308 | 1.45440216 | 1.94250161 |
| 1 | -4.02931440 | 0.69807567 | 2.31088797 |
| 1 | -3.79121145 | 2.43728838 | 2.60206724 |
| 1 | -1.55142032 | 2.08285722 | 5.60592780 |
| 1 | -0.95080765 | 2.28071598 | 3.93956969 |
| 1 | -2.34008467 | 3.24165783 | 4.51112152 |
| 1 | 3.04489268 | -1.63788251 | 1.23432114 |
| 8 | 2.35126583 | 0.91392703 | -4.70066637 |
| 6 | 2.45168486 | 2.35629914 | -4.75599155 |
| 1 | 2.36043649 | 2.81436601 | -3.76470989 |
| 8 | 3.31431787 | -1.02916387 | -4.21296863 |
| 8 | 1.69897883 | 1.37772733 | 1.01512751 |
| 1 | 1.61572783 | 2.67356088 | -5.38693080 |
| 1 | 3.39543827 | 2.66189651 | -5.23050145 |
| 31 | 0.41593997 | 0.07677659 | 1.01964417 |
| 6 | 3.26157902 | 0.16207007 | -4.01317660 |
| 6 | 4.23885975 | 0.89109705 | -3.06270892 |
| 8 | 3.57390400 | 1.71636104 | -2.04391392 |
| 6 | 2.72465862 | 1.10791464 | -1.17025210 |

| | | | |
|---|---|---|---|
| 6 | 2.22360781 | 2.07333000 | -0.08350072 |
| 8 | 2.37719080 | -0.05512944 | -1.25241641 |
| 6 | 3.31698625 | 3.02130987 | 0.42121437 |
| 6 | 5.25191444 | -0.06050333 | -2.44896755 |
| 1 | 4.76592700 | 1.65485984 | -3.65494482 |
| 1 | 1.44084326 | 2.68273152 | -0.58733908 |
| 1 | 2.88573620 | 3.66888079 | 1.19414573 |
| 1 | 3.71496187 | 3.64358005 | -0.38929077 |
| 1 | 4.13908844 | 2.44839637 | 0.87115989 |
| 1 | 5.86193338 | -0.50227261 | -3.24684660 |
| 1 | 4.75403902 | -0.87578482 | -1.91539162 |
| 1 | 5.91144746 | 0.49241569 | -1.76726782 |

Electronic energy = -3673.036762 a.u.
DFT-D3(BJ) dispersion correction = -0.116598 a.u.
Thermal free energy = 0.582011 a.u.
Gibbs free energy = -3672.571348 a.u.
Number of imaginary frequencies = 0.

**Int2 LA isomer/conformer 67**

| Atomic N. | X | Y | Z |
|---|---|---|---|
| 8 | 3.89040654 | 1.64560624 | -4.77386016 |
| 6 | 2.71340230 | 1.75688772 | -5.61439143 |
| 1 | 1.81341803 | 1.39594433 | -5.10373940 |
| 8 | 4.97673302 | 2.18557060 | -2.91455900 |
| 8 | 2.35636635 | -0.16645270 | 0.06624951 |
| 1 | 2.92790272 | 1.12466497 | -6.48109836 |
| 1 | 2.57624428 | 2.79412560 | -5.95039676 |
| 7 | 0.94281309 | -1.20241147 | 2.39314886 |
| 8 | -0.72015083 | -1.44928904 | -0.05184239 |
| 8 | -0.10210617 | 1.36342893 | 1.30039898 |
| 6 | 2.13542343 | -2.08377266 | 2.35077162 |
| 6 | -0.87704160 | -2.72013340 | 0.38317693 |
| 6 | -1.34432471 | -3.71904422 | -0.52411370 |
| 6 | -1.47804965 | -5.02555196 | -0.03355317 |
| 6 | -1.18668094 | -5.37245347 | 1.28866144 |
| 6 | -0.76616355 | -4.38377844 | 2.17145917 |
| 6 | -0.62366399 | -3.06135625 | 1.73711878 |
| 6 | -0.28949519 | -1.98720923 | 2.73625949 |
| 1 | -1.29993828 | -6.40394105 | 1.62226493 |
| 6 | -1.71232039 | -3.37718778 | -1.98205400 |
| 6 | -0.56270416 | 1.53046479 | 2.55675862 |
| 6 | -1.61331462 | 2.46469933 | 2.80578030 |
| 6 | -2.04499744 | 2.61186266 | 4.13105390 |
| 6 | -1.49507455 | 1.88691488 | 5.19277635 |
| 6 | -0.46902901 | 0.98379308 | 4.93844721 |
| 6 | 0.00979376 | 0.80783745 | 3.63501083 |
| 6 | 1.18284334 | -0.10540132 | 3.39804873 |
| 1 | -1.86977261 | 2.03066844 | 6.20617166 |
| 6 | -2.25253243 | 3.27867839 | 1.66331525 |
| 6 | -2.21551142 | -4.61083574 | -2.75491373 |
| 6 | -0.47796857 | -2.83260734 | -2.73769704 |
| 6 | -2.84472295 | -2.32268901 | -2.00526543 |

| | | | |
|---|---|---|---|
| 6 | -3.34568825 | 4.23374416 | 2.17847558 |
| 6 | -2.91541774 | 2.32829074 | 0.63810505 |
| 6 | -1.17901299 | 4.14479120 | 0.96132627 |
| 1 | 2.29699637 | -2.54242113 | 3.34088562 |
| 1 | 1.98148098 | -2.87590838 | 1.61049048 |
| 1 | -0.15647005 | -2.43305734 | 3.73728064 |
| 1 | -1.10844905 | -1.25578789 | 2.80744348 |
| 1 | -1.82201124 | -5.80939777 | -0.70594600 |
| 1 | -0.55954892 | -4.62561896 | 3.21662347 |
| 1 | 2.04860631 | 0.45801171 | 3.01380588 |
| 1 | 1.48073227 | -0.57307531 | 4.35225956 |
| 1 | -2.84730670 | 3.31416710 | 4.35031496 |
| 1 | -0.01927408 | 0.41229952 | 5.75372519 |
| 1 | -2.47412434 | -4.30864806 | -3.77990048 |
| 1 | -1.45048158 | -5.39790327 | -2.82483208 |
| 1 | -3.11845571 | -5.04308159 | -2.30034740 |
| 1 | -0.11790450 | -1.90579278 | -2.27910306 |
| 1 | 0.33688375 | -3.57186995 | -2.73696837 |
| 1 | -0.74488875 | -2.62191842 | -3.78479938 |
| 1 | -3.74444786 | -2.71204707 | -1.50698639 |
| 1 | -2.54069975 | -1.39701036 | -1.50467422 |
| 1 | -3.11118682 | -2.08554024 | -3.04681343 |
| 1 | -3.76040292 | 4.79354285 | 1.32796639 |
| 1 | -2.95093460 | 4.96676364 | 2.89704622 |
| 1 | -4.17686475 | 3.69325483 | 2.65403495 |
| 1 | -2.18540998 | 1.65212599 | 0.18148812 |
| 1 | -3.69748826 | 1.72406841 | 1.12044095 |
| 1 | -3.38904180 | 2.91712602 | -0.16236499 |
| 1 | -0.72961089 | 4.85495154 | 1.67119786 |
| 1 | -0.38415337 | 3.52185582 | 0.53675607 |
| 1 | -1.64361233 | 4.72531067 | 0.14938433 |
| 1 | 3.00840587 | -1.49014721 | 2.06163936 |
| 31 | 0.55693314 | -0.23316483 | 0.57258069 |
| 6 | 3.93993811 | 2.21919085 | -3.54148325 |
| 6 | 2.66531421 | 2.91013012 | -3.00829960 |
| 8 | 1.54319595 | 1.98004003 | -2.80063889 |
| 6 | 1.47651502 | 1.10843537 | -1.76626946 |
| 6 | 2.68869850 | 0.38291761 | -1.17497000 |
| 8 | 0.34730237 | 0.82214335 | -1.36969189 |
| 6 | 3.11584309 | -0.72774047 | -2.16399194 |
| 6 | 2.92901375 | 3.78123574 | -1.79337580 |
| 1 | 2.26379406 | 3.53858031 | -3.81621398 |
| 1 | 3.52746078 | 1.09072616 | -1.05949954 |
| 1 | 3.96345065 | -1.25819453 | -1.71345022 |
| 1 | 3.43238622 | -0.34159259 | -3.14141757 |
| 1 | 2.29094607 | -1.43881377 | -2.30238602 |
| 1 | 3.62007741 | 4.58654378 | -2.07426386 |
| 1 | 3.39155049 | 3.22224730 | -0.97311072 |
| 1 | 1.98757805 | 4.22920479 | -1.45086057 |

Electronic energy = -3673.035494 a.u.
DFT-D3(BJ) dispersion correction = -0.116228 a.u.
Thermal free energy = 0.583527 a.u.
Gibbs free energy = -3672.568196 a.u.

Number of imaginary frequencies = 0.

**Int2_LA isomer/conformer 68**

| Atomic N. | X | Y | Z |
|---|---|---|---|
| 8 | 0.95971735 | 1.93626517 | 6.10860243 |
| 6 | 0.75589417 | 3.35989344 | 5.96244247 |
| 1 | 0.78318704 | 3.65713129 | 4.90474098 |
| 8 | 0.38192937 | -0.14472063 | 5.57881249 |
| 8 | 0.60037725 | 0.61525777 | 1.35045596 |
| 1 | 1.60011771 | 3.82093715 | 6.48434964 |
| 1 | -0.18022005 | 3.69431046 | 6.42947237 |
| 7 | -0.29036614 | -2.50977319 | 0.15892417 |
| 8 | 1.31619705 | -0.43024834 | -1.22110597 |
| 8 | -1.67660092 | 0.01493573 | -0.78990307 |
| 6 | 0.16087346 | -3.09607909 | 1.44849630 |
| 6 | 2.29938374 | -1.35920899 | -1.17705700 |
| 6 | 3.66490576 | -0.99097250 | -1.35554368 |
| 6 | 4.61805298 | -2.01800464 | -1.28709596 |
| 6 | 4.28127908 | -3.35697271 | -1.06505335 |
| 6 | 2.94212692 | -3.70799508 | -0.92599074 |
| 6 | 1.95197888 | -2.72306163 | -0.99545466 |
| 6 | 0.49772716 | -3.09823563 | -0.98857075 |
| 1 | 5.06229876 | -4.11560614 | -1.01411594 |
| 6 | 4.07980352 | 0.46981227 | -1.61637448 |
| 6 | -2.31353743 | -0.85043255 | -1.61929958 |
| 6 | -2.97262654 | -0.35527991 | -2.78350662 |
| 6 | -3.62350391 | -1.28810512 | -3.60287894 |
| 6 | -3.64245995 | -2.65725368 | -3.32473001 |
| 6 | -3.00231610 | -3.12614573 | -2.18353768 |
| 6 | -2.34974800 | -2.23848549 | -1.31916842 |
| 6 | -1.76772545 | -2.77755949 | -0.03480934 |
| 1 | -4.15416733 | -3.34602186 | -3.99680452 |
| 6 | -2.97295176 | 1.14596978 | -3.13592379 |
| 6 | 5.59757492 | 0.60595392 | -1.84102914 |
| 6 | 3.71017963 | 1.35238923 | -0.40109375 |
| 6 | 3.37703116 | 1.00087017 | -2.88864962 |
| 6 | -3.76266988 | 1.43531180 | -4.42670712 |
| 6 | -1.52584872 | 1.64261987 | -3.36548954 |
| 6 | -3.64176520 | 1.95707584 | -2.00085336 |
| 1 | 0.00753503 | -4.18714520 | 1.42996517 |
| 1 | 1.22664682 | -2.88605279 | 1.59039205 |
| 1 | 0.38128449 | -4.19410074 | -0.94703144 |
| 1 | 0.00625144 | -2.73739840 | -1.90356774 |
| 1 | 5.67078927 | -1.76800362 | -1.40754409 |
| 1 | 2.65254075 | -4.75184510 | -0.78344626 |
| 1 | -2.26536311 | -2.33567680 | 0.83812651 |
| 1 | -1.91369680 | -3.86988964 | 0.00072602 |
| 1 | -4.13064611 | -0.93918282 | -4.50045911 |
| 1 | -3.01391575 | -4.19144245 | -1.94200643 |
| 1 | 5.83527970 | 1.66074897 | -2.04049263 |
| 1 | 6.17725229 | 0.29882818 | -0.95836006 |
| 1 | 5.94205325 | 0.01941001 | -2.70519141 |

| | | | |
|---|---|---|---|
| 1 | 2.63500029 | 1.33263098 | -0.19358625 |
| 1 | 4.23964940 | 1.00918456 | 0.49986876 |
| 1 | 4.01119601 | 2.39384429 | -0.59519426 |
| 1 | 3.66621651 | 0.40560330 | -3.76719474 |
| 1 | 2.28662283 | 0.96518930 | -2.78959715 |
| 1 | 3.68014896 | 2.04329932 | -3.07098868 |
| 1 | -3.73844250 | 2.51656928 | -4.62410583 |
| 1 | -4.81801757 | 1.13795302 | -4.34278209 |
| 1 | -3.32655623 | 0.93091025 | -5.30098273 |
| 1 | -0.90524787 | 1.51802707 | -2.47227486 |
| 1 | -1.05406370 | 1.09095945 | -4.19126876 |
| 1 | -1.53822785 | 2.70992406 | -3.63472149 |
| 1 | -4.69056308 | 1.65151870 | -1.87321318 |
| 1 | -3.12187002 | 1.81448642 | -1.04749936 |
| 1 | -3.62953347 | 3.02894280 | -2.25135402 |
| 1 | -0.41446845 | -2.65701466 | 2.26909826 |
| 31 | -0.08078649 | -0.47079305 | 0.03105207 |
| 6 | 0.09556210 | 1.02908462 | 5.56383788 |
| 6 | -1.24980164 | 1.55235144 | 5.01990403 |
| 8 | -1.81681252 | 0.59028077 | 4.10068951 |
| 6 | -1.23787505 | 0.33212032 | 2.89692992 |
| 6 | -0.17918549 | 1.27218486 | 2.30490622 |
| 8 | -1.64252162 | -0.62948268 | 2.26639961 |
| 6 | -0.89296572 | 2.50726796 | 1.71208595 |
| 6 | -2.26357355 | 1.66712671 | 6.15934320 |
| 1 | -1.12765991 | 2.52170912 | 4.51862287 |
| 1 | 0.50697065 | 1.59845069 | 3.10786552 |
| 1 | -0.13380713 | 3.16619046 | 1.27242933 |
| 1 | -1.46498586 | 3.07503639 | 2.46111117 |
| 1 | -1.58323230 | 2.18890520 | 0.91915585 |
| 1 | -1.91762255 | 2.36635474 | 6.93119019 |
| 1 | -2.41011567 | 0.68185735 | 6.61838822 |
| 1 | -3.22357033 | 2.02250587 | 5.76522446 |

Electronic energy = -3673.034309 a.u.
DFT-D3(BJ) dispersion correction = -0.115589 a.u.
Thermal free energy = 0.582054 a.u.
Gibbs free energy = -3672.567844 a.u.
Number of imaginary frequencies = 0.

**Int2 LA isomer/conformer 69**

| Atomic N. | X | Y | Z |
|---|---|---|---|
| 8 | -4.00695405 | -4.82439726 | -1.32090776 |
| 6 | -2.79016797 | -5.52782433 | -1.68259247 |
| 1 | -2.63858587 | -5.56213345 | -2.76649518 |
| 8 | -5.50703103 | -3.21811808 | -1.60586590 |
| 8 | -1.61603427 | -0.60429330 | 0.02593983 |
| 1 | -2.93687340 | -6.54279122 | -1.29854955 |
| 1 | -1.91808373 | -5.06855410 | -1.20411804 |
| 7 | 1.47770037 | -1.51527323 | 1.02235732 |
| 8 | 0.17780670 | 1.09522692 | 1.59306306 |
| 8 | 1.09986315 | 0.43346816 | -1.26347873 |
| 6 | 0.85111372 | -2.78385463 | 1.47487464 |

| | | | |
|---|---|---|---|
| 6 | 0.26037590 | 0.68646854 | 2.88330092 |
| 6 | -0.55014163 | 1.28501219 | 3.89087711 |
| 6 | -0.40328993 | 0.80109117 | 5.19938238 |
| 6 | 0.49374970 | -0.21632692 | 5.53924899 |
| 6 | 1.29992845 | -0.77195315 | 4.55094385 |
| 6 | 1.19948520 | -0.31869210 | 3.23156859 |
| 6 | 2.15110691 | -0.81555295 | 2.18012751 |
| 1 | 0.56458614 | -0.55977512 | 6.57128824 |
| 6 | -1.54296472 | 2.41680256 | 3.56284360 |
| 6 | 2.45884368 | 0.46802361 | -1.23341811 |
| 6 | 3.15380445 | 1.55356474 | -1.84290351 |
| 6 | 4.55483228 | 1.53150796 | -1.79002169 |
| 6 | 5.27421049 | 0.50642998 | -1.17047936 |
| 6 | 4.58439684 | -0.54986064 | -0.58715205 |
| 6 | 3.18520341 | -0.58818483 | -0.62286926 |
| 6 | 2.47747930 | -1.80351916 | -0.07696888 |
| 1 | 6.36340313 | 0.53817284 | -1.14759034 |
| 6 | 2.40514993 | 2.70953810 | -2.53639316 |
| 6 | -2.25867846 | 2.93741968 | 4.82350704 |
| 6 | -2.63178703 | 1.90450789 | 2.59101554 |
| 6 | -0.79129902 | 3.61387300 | 2.93307650 |
| 6 | 3.37400160 | 3.75230044 | -3.12610680 |
| 6 | 1.50130737 | 3.44953867 | -1.52226220 |
| 6 | 1.55677899 | 2.16141069 | -3.70811564 |
| 1 | 1.62659778 | -3.45628069 | 1.87612434 |
| 1 | 0.12020744 | -2.56807976 | 2.26190726 |
| 1 | 2.88050864 | -1.51659043 | 2.61907651 |
| 1 | 2.70775688 | 0.02343267 | 1.73834000 |
| 1 | -1.01554142 | 1.22988681 | 5.99080245 |
| 1 | 2.02874992 | -1.54739228 | 4.79842073 |
| 1 | 1.90736837 | -2.31257851 | -0.86586352 |
| 1 | 3.22161817 | -2.51060537 | 0.32556645 |
| 1 | 5.11249504 | 2.34945935 | -2.24211964 |
| 1 | 5.12733420 | -1.36711616 | -0.10702496 |
| 1 | -2.93956678 | 3.75129996 | 4.53617777 |
| 1 | -2.86374130 | 2.15818992 | 5.30932330 |
| 1 | -1.55234101 | 3.34216204 | 5.56292193 |
| 1 | -2.20316965 | 1.54338137 | 1.65016303 |
| 1 | -3.20064202 | 1.08090927 | 3.04653128 |
| 1 | -3.33783408 | 2.71822065 | 2.36389963 |
| 1 | -0.04214711 | 4.01205392 | 3.63312363 |
| 1 | -0.28373289 | 3.32783816 | 2.00550822 |
| 1 | -1.50443884 | 4.42130757 | 2.70694072 |
| 1 | 2.78943463 | 4.54489027 | -3.61453712 |
| 1 | 4.03982787 | 3.31731120 | -3.88543150 |
| 1 | 3.99200090 | 4.22851010 | -2.35134464 |
| 1 | 0.74214609 | 2.78814265 | -1.09252319 |
| 1 | 2.10112208 | 3.86408363 | -0.69937025 |
| 1 | 0.98975418 | 4.28567779 | -2.02286925 |
| 1 | 2.20098542 | 1.69001152 | -4.46462264 |
| 1 | 0.82921541 | 1.41883500 | -3.36361466 |
| 1 | 1.01290789 | 2.98633627 | -4.19306963 |
| 1 | 0.35126766 | -3.25933754 | 0.62514193 |

| | | | |
|---|---|---|---|
| 31 | 0.15537909 | -0.17038665 | 0.21811845 |
| 6 | -4.41784972 | -3.66843374 | -1.89776876 |
| 6 | -3.56578901 | -2.91756273 | -2.94512937 |
| 8 | -2.13142877 | -2.99144656 | -2.71631185 |
| 6 | -1.50204585 | -2.19766519 | -1.79688508 |
| 6 | -2.21796558 | -0.98484970 | -1.18128167 |
| 8 | -0.34577218 | -2.47046183 | -1.52661253 |
| 6 | -2.24329603 | 0.16515651 | -2.21014486 |
| 6 | -3.83156543 | -3.43760056 | -4.35596911 |
| 1 | -3.91022234 | -1.87971997 | -2.88048784 |
| 1 | -3.25620684 | -1.26994510 | -0.93556114 |
| 1 | -2.74637120 | 1.02496249 | -1.75043760 |
| 1 | -2.77797210 | -0.09918315 | -3.13371806 |
| 1 | -1.21670321 | 0.45300935 | -2.47433265 |
| 1 | -4.90790325 | -3.38957590 | -4.56410065 |
| 1 | -3.49368766 | -4.47366651 | -4.48014097 |
| 1 | -3.30198001 | -2.80984278 | -5.08394101 |

Electronic energy = -3673.033004 a.u.
DFT-D3(BJ) dispersion correction = -0.115486 a.u.
Thermal free energy = 0.581328 a.u.
Gibbs free energy = -3672.567162 a.u.
Number of imaginary frequencies = 0.

**Int2 LA isomer/conformer 70**

| Atomic N. | X | Y | Z |
|---|---|---|---|
| 7 | 1.07693213 | 0.18106685 | -2.21104407 |
| 8 | -0.78612478 | -0.69426425 | -0.00825952 |
| 8 | 1.83531758 | -2.13083627 | -0.47400722 |
| 6 | 1.47232293 | 1.59558148 | -2.41956872 |
| 6 | -1.66451302 | 0.14940462 | -0.61116437 |
| 6 | -2.84623508 | 0.55477570 | 0.06892637 |
| 6 | -3.67733510 | 1.47191566 | -0.59129476 |
| 6 | -3.40468996 | 1.95690627 | -1.87387300 |
| 6 | -2.28510971 | 1.49045211 | -2.55602650 |
| 6 | -1.42226763 | 0.57642820 | -1.94238761 |
| 6 | -0.31713948 | -0.06595274 | -2.73801620 |
| 1 | -4.07938739 | 2.67461451 | -2.34089209 |
| 6 | -3.22984546 | -0.03022992 | 1.44359720 |
| 6 | 1.71033375 | -2.84474261 | -1.61151192 |
| 6 | 1.54778600 | -4.26151175 | -1.56367041 |
| 6 | 1.43539065 | -4.93621639 | -2.78712717 |
| 6 | 1.47763478 | -4.28298144 | -4.02338891 |
| 6 | 1.65211382 | -2.90417332 | -4.05805271 |
| 6 | 1.78127849 | -2.18345651 | -2.86486742 |
| 6 | 2.08045273 | -0.71071537 | -2.91545350 |
| 1 | 1.37805081 | -4.85321781 | -4.94691343 |
| 6 | 1.49740762 | -5.02021845 | -0.22353303 |
| 6 | -4.55479807 | 0.55806636 | 1.96429260 |
| 6 | -2.15065016 | 0.26407680 | 2.51115230 |
| 6 | -3.41931314 | -1.56001843 | 1.30195827 |
| 6 | 1.35487457 | -6.54019690 | -0.42783929 |
| 6 | 0.27997779 | -4.55071907 | 0.60746797 |

| | | | |
|---|---|---|---|
| 6 | 2.80367938 | -4.78121134 | 0.57086807 |
| 1 | 1.53932191 | 1.80199606 | -3.50030595 |
| 1 | 0.73128299 | 2.25240442 | -1.95755217 |
| 1 | -0.35042571 | 0.27907080 | -3.78516079 |
| 1 | -0.44012380 | -1.15885611 | -2.73976329 |
| 1 | -4.57456744 | 1.82693772 | -0.08780803 |
| 1 | -2.08446506 | 1.81276624 | -3.58065945 |
| 1 | 3.04861238 | -0.49269566 | -2.43638081 |
| 1 | 2.13913544 | -0.38093263 | -3.96608104 |
| 1 | 1.30274244 | -6.01672446 | -2.78276614 |
| 1 | 1.70274680 | -2.37481176 | -5.01246976 |
| 1 | -4.79357405 | 0.09597668 | 2.93278987 |
| 1 | -4.48664162 | 1.64382319 | 2.12294914 |
| 1 | -5.39527392 | 0.35361797 | 1.28523456 |
| 1 | -1.19895305 | -0.21347858 | 2.25452170 |
| 1 | -1.99156976 | 1.34445113 | 2.61939685 |
| 1 | -2.48171378 | -0.13375995 | 3.48285791 |
| 1 | -4.21888368 | -1.78864642 | 0.58213017 |
| 1 | -2.49720667 | -2.04851581 | 0.96559556 |
| 1 | -3.70466629 | -1.99124174 | 2.27376821 |
| 1 | 1.33667340 | -7.03196567 | 0.55521159 |
| 1 | 2.19824254 | -6.96238113 | -0.99352882 |
| 1 | 0.42111649 | -6.80371748 | -0.94555933 |
| 1 | 0.33389622 | -3.48139798 | 0.83672726 |
| 1 | -0.65731878 | -4.74095604 | 0.06478424 |
| 1 | 0.24138404 | -5.10685866 | 1.55661425 |
| 1 | 3.67209955 | -5.15306542 | 0.00728977 |
| 1 | 2.95513858 | -3.71755401 | 0.78389046 |
| 1 | 2.76199417 | -5.32759330 | 1.52560371 |
| 1 | 2.44913526 | 1.77204597 | -1.95303587 |
| 8 | -1.37067821 | 5.95208760 | 2.73113418 |
| 6 | -0.60063353 | 7.16142793 | 2.90103077 |
| 1 | 0.15251683 | 7.04279187 | 3.69265137 |
| 8 | -1.42217038 | 3.74409737 | 2.49760403 |
| 8 | 2.19524460 | 0.28647664 | 0.95818250 |
| 1 | -1.32741587 | 7.91899930 | 3.21092465 |
| 1 | -0.12356633 | 7.48703150 | 1.96690134 |
| 31 | 1.04053217 | -0.45461732 | -0.24673077 |
| 6 | -0.78319965 | 4.76263833 | 2.39172854 |
| 6 | 0.65723088 | 4.80789239 | 1.85143369 |
| 8 | 1.32094721 | 3.54979395 | 2.14924072 |
| 6 | 1.15311914 | 2.43995174 | 1.37763218 |
| 6 | 1.90480487 | 1.23064228 | 1.95847350 |
| 8 | 0.49578002 | 2.37870289 | 0.35595593 |
| 6 | 3.21025485 | 1.59782421 | 2.66908010 |
| 6 | 0.71370755 | 5.24926378 | 0.38351434 |
| 1 | 1.24407905 | 5.51365185 | 2.45559934 |
| 1 | 1.20182401 | 0.81408635 | 2.71271057 |
| 1 | 3.67470963 | 0.67625643 | 3.04056821 |
| 1 | 3.03046336 | 2.27386790 | 3.51336369 |
| 1 | 3.90706088 | 2.07625926 | 1.96752053 |
| 1 | 0.45425238 | 6.31404611 | 0.30980971 |
| 1 | 0.03001796 | 4.66874038 | -0.24004527 |

| | | | |
|---|---|---|---|
| 1 | 1.73683592 | 5.12752389 | 0.00405139 |

Electronic energy = -3673.032298 a.u.
DFT-D3(BJ) dispersion correction = -0.115535 a.u.
Thermal free energy = 0.580690 a.u.
Gibbs free energy = -3672.567143 a.u.
Number of imaginary frequencies = 0.

**Int2 LA isomer/conformer 71**

| Atomic N. | X | Y | Z |
|---|---|---|---|
| 8 | -6.08282915 | 2.98485024 | -0.36242387 |
| 6 | -4.72543860 | 3.37261764 | -0.66388422 |
| 1 | -4.04293883 | 3.08042651 | 0.14332377 |
| 8 | -7.66963702 | 1.44918991 | -0.19632999 |
| 8 | -1.17369246 | 0.87324089 | -0.90476175 |
| 1 | -4.75322459 | 4.46399268 | -0.74000662 |
| 1 | -4.38672888 | 2.96010326 | -1.62407360 |
| 7 | 0.41725619 | 1.02946285 | 2.15357355 |
| 8 | 1.67115759 | 0.60621585 | -0.50534323 |
| 8 | -0.07290201 | -1.60137435 | 0.73831861 |
| 6 | -0.33235041 | 2.27919491 | 2.44363520 |
| 6 | 2.31017748 | 1.76752217 | -0.22787374 |
| 6 | 2.92043654 | 2.53128937 | -1.26516808 |
| 6 | 3.55704484 | 3.72391651 | -0.89044124 |
| 6 | 3.61872808 | 4.17167268 | 0.43291458 |
| 6 | 3.04675467 | 3.40058100 | 1.43988496 |
| 6 | 2.40775654 | 2.19823282 | 1.12053468 |
| 6 | 1.90553417 | 1.28840491 | 2.20494223 |
| 1 | 4.12226454 | 5.10893393 | 0.66966823 |
| 6 | 2.88861253 | 2.06859196 | -2.73457900 |
| 6 | 0.63193704 | -2.10196207 | 1.78741896 |
| 6 | 1.22622220 | -3.39443009 | 1.68907406 |
| 6 | 1.93309290 | -3.86437592 | 2.80508301 |
| 6 | 2.07376275 | -3.12285428 | 3.98085552 |
| 6 | 1.48318080 | -1.86761655 | 4.06603103 |
| 6 | 0.75234201 | -1.35363992 | 2.98787213 |
| 6 | 0.03607854 | -0.03702372 | 3.15917639 |
| 1 | 2.64017330 | -3.52781504 | 4.81931247 |
| 6 | 1.10135198 | -4.25051259 | 0.41258124 |
| 6 | 3.66772178 | 3.02559528 | -3.65634046 |
| 6 | 1.42911603 | 2.01960086 | -3.24462665 |
| 6 | 3.54150694 | 0.67167123 | -2.86438063 |
| 6 | 1.81813507 | -5.60680273 | 0.55527637 |
| 6 | 1.74371548 | -3.51946377 | -0.78989957 |
| 6 | -0.38816358 | -4.55023229 | 0.12189332 |
| 1 | -0.06543814 | 2.64376248 | 3.44870407 |
| 1 | -0.06586483 | 3.04269985 | 1.70429860 |
| 1 | 2.13928207 | 1.70509989 | 3.19880593 |
| 1 | 2.38326704 | 0.30107006 | 2.12889166 |
| 1 | 4.02426664 | 4.33547769 | -1.66041717 |
| 1 | 3.11057402 | 3.71396403 | 2.48461702 |
| 1 | -1.04998748 | -0.16316672 | 3.05751301 |
| 1 | 0.24549201 | 0.36471679 | 4.16427918 |

| | | | |
|---|---|---|---|
| 1 | 2.40155384 | -4.84555068 | 2.75773359 |
| 1 | 1.57168506 | -1.27564102 | 4.97975376 |
| 1 | 3.62775688 | 2.64090402 | -4.68544607 |
| 1 | 3.23572555 | 4.03692535 | -3.66582363 |
| 1 | 4.72732696 | 3.10226128 | -3.37230105 |
| 1 | 0.81254450 | 1.33309602 | -2.65452908 |
| 1 | 0.97159550 | 3.01901048 | -3.19946968 |
| 1 | 1.41476829 | 1.68846197 | -4.29468999 |
| 1 | 4.59238245 | 0.70435865 | -2.54137491 |
| 1 | 3.01529100 | -0.07636191 | -2.26164809 |
| 1 | 3.52207103 | 0.34988214 | -3.91688838 |
| 1 | 1.68948064 | -6.17693870 | -0.37579072 |
| 1 | 1.40180726 | -6.21215253 | 1.37336427 |
| 1 | 2.89838600 | -5.48895128 | 0.72296111 |
| 1 | 1.25301658 | -2.56269097 | -0.99556888 |
| 1 | 2.80936400 | -3.32574446 | -0.60104699 |
| 1 | 1.66850708 | -4.14892279 | -1.68968439 |
| 1 | -0.83599881 | -5.12340268 | 0.94670049 |
| 1 | -0.96337666 | -3.62758648 | -0.00953541 |
| 1 | -0.47603271 | -5.15186061 | -0.79560853 |
| 1 | -1.40625037 | 2.07365262 | 2.39912600 |
| 31 | 0.03448198 | 0.20372462 | 0.31109969 |
| 6 | -6.50090327 | 1.69037133 | -0.39969060 |
| 6 | -5.47472561 | 0.56950061 | -0.70942433 |
| 8 | -4.55247961 | 0.53994823 | 0.42530426 |
| 6 | -3.20207994 | 0.40719789 | 0.31016589 |
| 6 | -2.48112255 | 0.38894955 | -1.05154079 |
| 8 | -2.56616273 | 0.32993108 | 1.34857019 |
| 6 | -2.51432081 | -1.02117700 | -1.66914739 |
| 6 | -6.18023797 | -0.77004831 | -0.82831891 |
| 1 | -4.93247101 | 0.81524581 | -1.63384087 |
| 1 | -2.99379972 | 1.08632988 | -1.73787354 |
| 1 | -1.90553489 | -1.00157992 | -2.58192098 |
| 1 | -3.52999373 | -1.34230598 | -1.93693054 |
| 1 | -2.08109816 | -1.75210931 | -0.97337473 |
| 1 | -6.88390093 | -0.74565997 | -1.66999097 |
| 1 | -6.75081753 | -0.97693365 | 0.08340004 |
| 1 | -5.45418341 | -1.57496152 | -0.99464982 |

Electronic energy = -3673.031640 a.u.
DFT-D3(BJ) dispersion correction = -0.115851 a.u.
Thermal free energy = 0.580941 a.u.
Gibbs free energy = -3672.566550 a.u.
Number of imaginary frequencies = 0.

**Int2 LA isomer/conformer 72**

| Atomic N. | X | Y | Z |
|---|---|---|---|
| 7 | 0.00316502 | 1.62806584 | 2.03384813 |
| 8 | -1.08830767 | 0.57705341 | -0.56592106 |
| 8 | 1.85085797 | 0.11751285 | 0.20155178 |
| 6 | -0.67587519 | 1.39329901 | 3.33210211 |
| 6 | -2.17704411 | 1.32441529 | -0.24333854 |
| 6 | -3.40990159 | 1.13325483 | -0.93110078 |

| | | | |
|---|---|---|---|
| 6 | -4.50411066 | 1.89951294 | -0.50119369 |
| 6 | -4.41733806 | 2.84064526 | 0.52860746 |
| 6 | -3.18807750 | 3.07248718 | 1.13665080 |
| 6 | -2.06377500 | 2.33676353 | 0.74587188 |
| 6 | -0.70128885 | 2.71598092 | 1.26046416 |
| 1 | -5.30045340 | 3.40353033 | 0.83017313 |
| 6 | -3.53286202 | 0.16907726 | -2.12840233 |
| 6 | 2.44031555 | 1.33415184 | 0.05975521 |
| 6 | 3.27537721 | 1.60411456 | -1.06269207 |
| 6 | 3.86840685 | 2.87388269 | -1.12383813 |
| 6 | 3.66992222 | 3.85665031 | -0.14946607 |
| 6 | 2.85725061 | 3.57795762 | 0.94271404 |
| 6 | 2.25055027 | 2.32143335 | 1.06057938 |
| 6 | 1.44977922 | 2.00213155 | 2.29363722 |
| 1 | 4.15028630 | 4.82984388 | -0.24919406 |
| 6 | 3.52255943 | 0.55735913 | -2.16704281 |
| 6 | -4.94269543 | 0.20051032 | -2.74804888 |
| 6 | -3.25184791 | -1.28816266 | -1.69855550 |
| 6 | -2.53259114 | 0.59014342 | -3.23213143 |
| 6 | 4.46913452 | 1.09163707 | -3.25888740 |
| 6 | 2.18946755 | 0.18351787 | -2.85716293 |
| 6 | 4.17542250 | -0.70979728 | -1.56634693 |
| 1 | -0.65684013 | 2.31629547 | 3.93391857 |
| 1 | -1.71838864 | 1.10374253 | 3.16027662 |
| 1 | -0.77053855 | 3.60503594 | 1.90928639 |
| 1 | -0.03477800 | 2.96147444 | 0.42098364 |
| 1 | -5.46676460 | 1.76173155 | -0.98985445 |
| 1 | -3.08372759 | 3.84546957 | 1.90145723 |
| 1 | 1.88143902 | 1.14270567 | 2.83130123 |
| 1 | 1.45543573 | 2.86774239 | 2.97631423 |
| 1 | 4.50848175 | 3.11451002 | -1.97038471 |
| 1 | 2.69550975 | 4.32731950 | 1.72077946 |
| 1 | -4.96886572 | -0.48366539 | -3.60788344 |
| 1 | -5.71577838 | -0.13134551 | -2.03977793 |
| 1 | -5.21160867 | 1.20172222 | -3.11414016 |
| 1 | -2.24092843 | -1.39809981 | -1.29210857 |
| 1 | -3.97657521 | -1.61618966 | -0.93930246 |
| 1 | -3.34786408 | -1.95594045 | -2.56785383 |
| 1 | -2.74422008 | 1.61243815 | -3.57757709 |
| 1 | -1.49704766 | 0.54889764 | -2.87652102 |
| 1 | -2.62878551 | -0.08466617 | -4.09601372 |
| 1 | 4.61457759 | 0.30811703 | -4.01564692 |
| 1 | 5.46000135 | 1.35061119 | -2.85811483 |
| 1 | 4.05827311 | 1.97345638 | -3.77188959 |
| 1 | 1.48519267 | -0.27725820 | -2.15714246 |
| 1 | 1.71460827 | 1.07161992 | -3.29898115 |
| 1 | 2.38321574 | -0.53639542 | -3.66626263 |
| 1 | 5.12631624 | -0.45895249 | -1.07295561 |
| 1 | 3.51734704 | -1.20585653 | -0.84566251 |
| 1 | 4.39340521 | -1.42778834 | -2.37110915 |
| 1 | -0.15644886 | 0.59952818 | 3.88097400 |
| 8 | 2.18845471 | -3.42244313 | 2.22029907 |
| 6 | 3.42948622 | -2.67651537 | 2.15572223 |

| | | | |
|---|---|---|---|
| 1 | 3.25323657 | -1.69290798 | 1.70184214 |
| 8 | 2.10822117 | -3.34623001 | -0.05421650 |
| 8 | -0.13255174 | -1.65271270 | 1.49950095 |
| 1 | 3.75981940 | -2.58067220 | 3.19449750 |
| 1 | 4.17242583 | -3.22158151 | 1.56001168 |
| 31 | 0.09009762 | 0.00409181 | 0.75891036 |
| 6 | 1.61866441 | -3.65337863 | 1.01597861 |
| 6 | 0.34711189 | -4.49888559 | 1.13003280 |
| 8 | -0.29039151 | -4.47176502 | 2.43632552 |
| 6 | -0.90689830 | -3.43199619 | 3.06536096 |
| 6 | -1.08993641 | -2.00968086 | 2.48160013 |
| 8 | -1.38793537 | -3.65490648 | 4.15976989 |
| 6 | -2.54677372 | -1.86880734 | 2.01187514 |
| 6 | -0.60496470 | -4.32154478 | -0.04416615 |
| 1 | 0.71805892 | -5.53853062 | 1.10643326 |
| 1 | -0.96650345 | -1.39064180 | 3.38676270 |
| 1 | -2.75114428 | -0.82875525 | 1.71718079 |
| 1 | -3.23164347 | -2.14066437 | 2.82507701 |
| 1 | -2.74745774 | -2.51076828 | 1.14546853 |
| 1 | -0.07907333 | -4.58390765 | -0.97018287 |
| 1 | -0.94736637 | -3.28575698 | -0.12892045 |
| 1 | -1.46390639 | -4.99427120 | 0.07609487 |

Electronic energy = -3673.029559 a.u.
DFT-D3(BJ) dispersion correction = -0.119723 a.u.
Thermal free energy = 0.583507 a.u.
Gibbs free energy = -3672.565775 a.u.
Number of imaginary frequencies = 0.

**Int2 LA isomer/conformer 73**

| Atomic N. | X | Y | Z |
|---|---|---|---|
| 7 | -0.76379793 | 2.08123882 | 0.97484481 |
| 8 | -0.58740524 | -0.49612825 | -0.57349250 |
| 8 | 0.27733405 | 2.15394064 | -1.83410206 |
| 6 | -0.16500272 | 2.23978247 | 2.32359869 |
| 6 | -1.24821796 | -1.01540626 | 0.49862531 |
| 6 | -1.31278308 | -2.42441652 | 0.68320031 |
| 6 | -1.95489095 | -2.88411350 | 1.84264593 |
| 6 | -2.55172772 | -2.02589527 | 2.77090181 |
| 6 | -2.55026806 | -0.65482209 | 2.53331505 |
| 6 | -1.91749639 | -0.14273048 | 1.39556228 |
| 6 | -2.06279686 | 1.31550628 | 1.04698252 |
| 1 | -3.03727901 | -2.43476410 | 3.65739908 |
| 6 | -0.76632843 | -3.40828571 | -0.37212168 |
| 6 | -0.87646720 | 2.81734556 | -2.08786328 |
| 6 | -1.37116510 | 2.92135489 | -3.42052002 |
| 6 | -2.56597280 | 3.63123564 | -3.60643101 |
| 6 | -3.26878240 | 4.22620992 | -2.55406234 |
| 6 | -2.76648486 | 4.13244831 | -1.26111772 |
| 6 | -1.57085863 | 3.44495810 | -1.02135693 |
| 6 | -0.97657083 | 3.44887202 | 0.36143405 |
| 1 | -4.19803310 | 4.76078044 | -2.75063260 |
| 6 | -0.62743161 | 2.28269269 | -4.60962206 |

| | | | |
|---|---|---|---|
| 6 | -0.96703037 | -4.87406316 | 0.05664353 |
| 6 | 0.74761121 | -3.21172798 | -0.61640047 |
| 6 | -1.53774350 | -3.19299331 | -1.69699901 |
| 6 | -1.33503567 | 2.56446082 | -5.94850723 |
| 6 | -0.56528655 | 0.74675142 | -4.43658235 |
| 6 | 0.80351190 | 2.86249537 | -4.71305961 |
| 1 | -0.83019516 | 2.85290764 | 2.95311050 |
| 1 | -0.02073341 | 1.25345742 | 2.77290699 |
| 1 | -2.71567463 | 1.81942659 | 1.77924624 |
| 1 | -2.52267369 | 1.42253489 | 0.05351107 |
| 1 | -1.99035837 | -3.95454916 | 2.03463205 |
| 1 | -3.06165681 | 0.02866440 | 3.21548132 |
| 1 | 0.01860196 | 3.92142680 | 0.35313787 |
| 1 | -1.62194109 | 4.02698498 | 1.04355682 |
| 1 | -2.97413745 | 3.72165940 | -4.61145190 |
| 1 | -3.29028111 | 4.60415764 | -0.42652146 |
| 1 | -0.58207708 | -5.53194823 | -0.73549851 |
| 1 | -0.41669519 | -5.10859671 | 0.97872161 |
| 1 | -2.02832621 | -5.12173951 | 0.20337385 |
| 1 | 0.96020070 | -2.20998209 | -1.00505746 |
| 1 | 1.31820717 | -3.37750889 | 0.30709104 |
| 1 | 1.09351459 | -3.94534330 | -1.36055242 |
| 1 | -2.61303641 | -3.37533567 | -1.55540612 |
| 1 | -1.40413287 | -2.17372611 | -2.07879616 |
| 1 | -1.17196190 | -3.89833090 | -2.45872501 |
| 1 | -0.75780192 | 2.10087971 | -6.76107295 |
| 1 | -1.40177656 | 3.64102041 | -6.16272888 |
| 1 | -2.34854175 | 2.13893404 | -5.98026116 |
| 1 | -0.02467720 | 0.46193083 | -3.52796733 |
| 1 | -1.57717749 | 0.31973414 | -4.38433712 |
| 1 | -0.05117611 | 0.29667046 | -5.29941913 |
| 1 | 0.76915527 | 3.94898640 | -4.88099208 |
| 1 | 1.38323785 | 2.66839358 | -3.80424136 |
| 1 | 1.32746986 | 2.40584188 | -5.56658300 |
| 1 | 0.81090895 | 2.73063545 | 2.22978483 |
| 8 | 1.14542402 | -3.02315085 | 4.41141706 |
| 6 | 0.73476332 | -1.86255126 | 5.17139791 |
| 1 | 1.35661040 | -1.73404358 | 6.06672025 |
| 8 | 2.08803961 | -3.84861958 | 2.59077025 |
| 8 | 2.18330486 | 1.06234955 | 0.10682403 |
| 1 | -0.29487873 | -2.08033017 | 5.47499553 |
| 1 | 0.75096738 | -0.96607487 | 4.54399349 |
| 31 | 0.41754225 | 1.04830734 | -0.34546439 |
| 6 | 2.11694122 | -3.00483529 | 3.46313424 |
| 6 | 3.38147857 | -2.10231100 | 3.54602471 |
| 8 | 3.61588073 | -1.46419023 | 2.24842382 |
| 6 | 2.63630483 | -0.65217654 | 1.78042717 |
| 6 | 3.00257706 | -0.03876727 | 0.41685654 |
| 8 | 1.58104548 | -0.46227108 | 2.36321314 |
| 6 | 4.46439667 | 0.41831509 | 0.35677653 |
| 6 | 3.62240171 | -1.11181274 | 4.67739073 |
| 1 | 4.18683518 | -2.85081831 | 3.57270796 |
| 1 | 2.86877465 | -0.87134488 | -0.30477418 |

| | | | |
|---|---|---|---|
| 1 | 4.66108961 | 0.82657538 | -0.64192413 |
| 1 | 5.15153439 | -0.41414297 | 0.54836046 |
| 1 | 4.64460265 | 1.21172971 | 1.09496265 |
| 1 | 3.57100741 | -1.62503733 | 5.64694169 |
| 1 | 2.91479582 | -0.27780117 | 4.67308905 |
| 1 | 4.63869677 | -0.70891081 | 4.57051081 |

Electronic energy = -3673.031385 a.u.
DFT-D3(BJ) dispersion correction = -0.117992 a.u.
Thermal free energy = 0.583779 a.u.
Gibbs free energy = -3672.565598 a.u.
Number of imaginary frequencies = 0.

**Int2 LA isomer/conformer 74**

| Atomic N. | X | Y | Z |
|---|---|---|---|
| 8 | 2.61457306 | -4.65571484 | 3.62275100 |
| 6 | 3.35958056 | -5.42710417 | 2.64654120 |
| 1 | 3.33984663 | -4.95821762 | 1.65648661 |
| 8 | 2.35119279 | -2.78664960 | 4.79065812 |
| 8 | 0.92787359 | -0.11203981 | 1.44287062 |
| 1 | 2.85438500 | -6.39696750 | 2.61188084 |
| 1 | 4.39611251 | -5.57289912 | 2.98177573 |
| 7 | 1.46497487 | 1.06350736 | -1.66976050 |
| 8 | -0.91069841 | 1.65983215 | 0.01725517 |
| 8 | -0.54721510 | -1.14072188 | -1.19277245 |
| 6 | 2.82853715 | 1.41563326 | -1.19675697 |
| 6 | -0.44566087 | 2.93165902 | -0.03982753 |
| 6 | -0.87085586 | 3.90616833 | 0.90916354 |
| 6 | -0.34123643 | 5.19857039 | 0.77860835 |
| 6 | 0.56065408 | 5.55403675 | -0.22921553 |
| 6 | 0.94597567 | 4.60026284 | -1.16587750 |
| 6 | 0.43941149 | 3.29898503 | -1.08655680 |
| 6 | 0.74337519 | 2.29570997 | -2.16332704 |
| 1 | 0.94651285 | 6.57205537 | -0.28293474 |
| 6 | -1.86960648 | 3.55998492 | 2.03026693 |
| 6 | -0.75595471 | -1.00568974 | -2.52910455 |
| 6 | -1.95968524 | -1.49999585 | -3.11203984 |
| 6 | -2.11364825 | -1.34618733 | -4.49729771 |
| 6 | -1.15089357 | -0.73435966 | -5.30419763 |
| 6 | 0.02023220 | -0.26169809 | -4.72363098 |
| 6 | 0.23475736 | -0.40190602 | -3.34719073 |
| 6 | 1.55998439 | 0.02756175 | -2.76917520 |
| 1 | -1.32029079 | -0.63067287 | -6.37587382 |
| 6 | -3.05507562 | -2.17639078 | -2.26323711 |
| 6 | -2.21937788 | 4.78997162 | 2.88904458 |
| 6 | -1.26141972 | 2.49508044 | 2.97293159 |
| 6 | -3.19113117 | 3.03512061 | 1.41980441 |
| 6 | -4.24941392 | -2.63368210 | -3.12242783 |
| 6 | -3.60393600 | -1.18646055 | -1.20877122 |
| 6 | -2.48530060 | -3.43474997 | -1.56691724 |
| 1 | 3.40280665 | 1.86093388 | -2.02534983 |
| 1 | 2.75466631 | 2.14204646 | -0.38001848 |
| 1 | 1.36034024 | 2.75329759 | -2.95468866 |

| | | | |
|---|---|---|---|
| 1 | -0.18688652 | 1.93294665 | -2.62364546 |
| 1 | -0.63855188 | 5.96381003 | 1.49354692 |
| 1 | 1.62667414 | 4.86275429 | -1.97920511 |
| 1 | 2.08944483 | -0.82533685 | -2.32284320 |
| 1 | 2.19061512 | 0.44728488 | -3.57041901 |
| 1 | -3.02404652 | -1.70952825 | -4.97015684 |
| 1 | 0.79008651 | 0.21182154 | -5.33709685 |
| 1 | -2.94458891 | 4.49184380 | 3.65959880 |
| 1 | -1.33948914 | 5.19954746 | 3.40625108 |
| 1 | -2.67902513 | 5.59274893 | 2.29423990 |
| 1 | -1.00977896 | 1.57178993 | 2.44042482 |
| 1 | -0.34590523 | 2.87736900 | 3.44766252 |
| 1 | -1.97998711 | 2.25243713 | 3.77095500 |
| 1 | -3.65242916 | 3.80098072 | 0.77904732 |
| 1 | -3.02793008 | 2.13254892 | 0.82116850 |
| 1 | -3.90200559 | 2.79645208 | 2.22559399 |
| 1 | -4.99295018 | -3.11418998 | -2.47073087 |
| 1 | -3.95445734 | -3.36872485 | -3.88527193 |
| 1 | -4.74640426 | -1.79061638 | -3.62368383 |
| 1 | -2.82346629 | -0.85135087 | -0.51809089 |
| 1 | -4.03547144 | -0.30060786 | -1.69650278 |
| 1 | -4.39934550 | -1.67193818 | -0.62311457 |
| 1 | -2.14105728 | -4.16706996 | -2.31180784 |
| 1 | -1.64368393 | -3.18438767 | -0.91261295 |
| 1 | -3.26952977 | -3.91215081 | -0.95992137 |
| 1 | 3.33132500 | 0.50939977 | -0.84466448 |
| 31 | 0.26279027 | 0.22631340 | -0.23024130 |
| 6 | 2.90696571 | -3.34996224 | 3.87207070 |
| 6 | 3.95458347 | -2.65408169 | 2.97599693 |
| 8 | 3.57665983 | -2.61361119 | 1.55994593 |
| 6 | 2.55771248 | -1.85099692 | 1.04138030 |
| 6 | 1.37741675 | -1.36707228 | 1.89624591 |
| 8 | 2.59844537 | -1.64036816 | -0.15728886 |
| 6 | 0.27945554 | -2.44764555 | 1.86011431 |
| 6 | 4.38745741 | -1.29977171 | 3.51290330 |
| 1 | 4.83598222 | -3.31246363 | 2.93960555 |
| 1 | 1.69348632 | -1.22903035 | 2.94030828 |
| 1 | -0.57400359 | -2.08382793 | 2.44619965 |
| 1 | 0.61521603 | -3.39814865 | 2.29705689 |
| 1 | -0.04388835 | -2.62332514 | 0.82524412 |
| 1 | 4.83498683 | -1.43152173 | 4.50644297 |
| 1 | 3.54914347 | -0.60309257 | 3.61891286 |
| 1 | 5.14204292 | -0.86753735 | 2.84329277 |

Electronic energy = -3673.030008 a.u.
DFT-D3(BJ) dispersion correction = -0.116277 a.u.
Thermal free energy = 0.582524 a.u.
Gibbs free energy = -3672.563761 a.u.
Number of imaginary frequencies = 0.

**Int2_LA isomer/conformer 75**

| Atomic N. | X | Y | Z |
|---|---|---|---|
| 7 | 0.05864537 | 2.13116582 | 1.58077410 |

| | | | |
|---|---|---|---|
| 8 | 0.19744972 | 0.37605462 | -0.88289303 |
| 8 | 2.36052070 | 0.29047210 | 1.20518921 |
| 6 | -1.17415383 | 2.31848751 | 2.38488892 |
| 6 | -0.79516048 | 1.17869455 | -1.33219671 |
| 6 | -1.58236187 | 0.78854750 | -2.45343115 |
| 6 | -2.62292993 | 1.64836951 | -2.83537052 |
| 6 | -2.88783051 | 2.85561172 | -2.18118029 |
| 6 | -2.07036744 | 3.25355234 | -1.12719835 |
| 6 | -1.01638138 | 2.43501885 | -0.70917608 |
| 6 | -0.02389426 | 2.93829974 | 0.30468452 |
| 1 | -3.71188590 | 3.48745314 | -2.51282383 |
| 6 | -1.28031875 | -0.50457212 | -3.23723406 |
| 6 | 3.08500118 | 1.42102449 | 1.14788694 |
| 6 | 4.39059409 | 1.41847592 | 0.56941842 |
| 6 | 5.08948815 | 2.63304593 | 0.55731478 |
| 6 | 4.56465902 | 3.81976673 | 1.08126938 |
| 6 | 3.29818631 | 3.80762074 | 1.65445015 |
| 6 | 2.56273357 | 2.61735901 | 1.70228825 |
| 6 | 1.24158938 | 2.58663465 | 2.41580896 |
| 1 | 5.14707542 | 4.74016321 | 1.04082896 |
| 6 | 5.00502749 | 0.13379152 | -0.01828001 |
| 6 | -2.23873464 | -0.69275463 | -4.42806048 |
| 6 | -1.42658603 | -1.74864811 | -2.33003393 |
| 6 | 0.15853967 | -0.44146831 | -3.80387751 |
| 6 | 6.43092459 | 0.36913732 | -0.55070925 |
| 6 | 4.14691364 | -0.37079207 | -1.20252570 |
| 6 | 5.09255761 | -0.96018506 | 1.07272801 |
| 1 | -1.24336888 | 3.37071557 | 2.70577099 |
| 1 | -2.05010464 | 2.05651710 | 1.78715349 |
| 1 | -0.25477786 | 3.98131992 | 0.57941258 |
| 1 | 0.98970991 | 2.91989490 | -0.12092100 |
| 1 | -3.25334511 | 1.37242444 | -3.67915883 |
| 1 | -2.22848784 | 4.21680766 | -0.63630614 |
| 1 | 1.27511640 | 1.89499523 | 3.27350572 |
| 1 | 1.00773177 | 3.59324250 | 2.80039918 |
| 1 | 6.08445673 | 2.66391824 | 0.11644450 |
| 1 | 2.87303426 | 4.71906230 | 2.08154458 |
| 1 | -1.97175592 | -1.61685504 | -4.95995675 |
| 1 | -3.28623634 | -0.78858803 | -4.10633426 |
| 1 | -2.17043729 | 0.13536785 | -5.14801802 |
| 1 | -0.72550119 | -1.71080814 | -1.48902146 |
| 1 | -2.45309038 | -1.82441692 | -1.94323822 |
| 1 | -1.22193165 | -2.66078006 | -2.91004669 |
| 1 | 0.26776105 | 0.41365230 | -4.48653167 |
| 1 | 0.90114537 | -0.34600084 | -3.00385821 |
| 1 | 0.37534944 | -1.35852464 | -4.37259067 |
| 1 | 6.82479712 | -0.57789574 | -0.94641530 |
| 1 | 7.11666423 | 0.70860976 | 0.23927540 |
| 1 | 6.45235015 | 1.10289045 | -1.36963154 |
| 1 | 3.12460974 | -0.60650430 | -0.88899222 |
| 1 | 4.09983840 | 0.38690058 | -1.99823356 |
| 1 | 4.59798684 | -1.28085996 | -1.62679079 |
| 1 | 5.73495924 | -0.62845438 | 1.90185450 |

| | | | |
|---|---|---|---|
| 1 | 4.10327456 | -1.20667731 | 1.47317786 |
| 1 | 5.53597530 | -1.87336034 | 0.64694708 |
| 1 | -1.13552849 | 1.67410364 | 3.27156808 |
| 8 | -4.76722000 | -3.21538510 | -0.79830357 |
| 6 | -5.30087567 | -1.88809740 | -0.54512937 |
| 1 | -5.72533689 | -1.80085756 | 0.46102169 |
| 8 | -3.65735261 | -5.04044077 | -0.20877030 |
| 8 | 0.35049120 | -1.44723243 | 1.79454688 |
| 1 | -6.09327667 | -1.76882865 | -1.29095656 |
| 1 | -4.52770610 | -1.12644457 | -0.69066146 |
| 31 | 0.50133207 | 0.19004371 | 0.95216719 |
| 6 | -3.98809468 | -3.90480349 | 0.06571710 |
| 6 | -3.54849989 | -3.33400381 | 1.43353280 |
| 8 | -3.28047707 | -1.90203525 | 1.37984439 |
| 6 | -2.03488844 | -1.38078173 | 1.45919103 |
| 6 | -0.82004834 | -2.18624975 | 1.93494903 |
| 8 | -1.91264466 | -0.19493875 | 1.17419857 |
| 6 | -0.98366260 | -2.58909348 | 3.41643234 |
| 6 | -4.60463828 | -3.60299626 | 2.50267356 |
| 1 | -2.63179715 | -3.88334078 | 1.66740541 |
| 1 | -0.75738709 | -3.10434766 | 1.31442210 |
| 1 | -0.05549724 | -3.08234751 | 3.72844014 |
| 1 | -1.82088760 | -3.27706376 | 3.59458798 |
| 1 | -1.12008383 | -1.69166055 | 4.03454881 |
| 1 | -4.82702013 | -4.67745235 | 2.52935002 |
| 1 | -5.53373855 | -3.05657876 | 2.29918828 |
| 1 | -4.23018555 | -3.29608672 | 3.48805560 |

Electronic energy = -3673.028426 a.u.
DFT-D3(BJ) dispersion correction = -0.117246 a.u.
Thermal free energy = 0.582611 a.u.
Gibbs free energy = -3672.563060 a.u.
Number of imaginary frequencies = 0.

**Int2_LA isomer/conformer 76**

| Atomic N. | X | Y | Z |
|---|---|---|---|
| 7 | 0.39819896 | 1.31810899 | 2.24937684 |
| 8 | 1.03843993 | -0.70353219 | 0.08728972 |
| 8 | -1.49881908 | -0.88427343 | 1.64252366 |
| 6 | 0.66939722 | 2.76349756 | 2.06317892 |
| 6 | 2.29798143 | -0.22107686 | 0.22211283 |
| 6 | 3.28060102 | -0.48997139 | -0.77375601 |
| 6 | 4.55274380 | 0.06959277 | -0.58512870 |
| 6 | 4.88516928 | 0.84492211 | 0.52977697 |
| 6 | 3.93330112 | 1.05025405 | 1.52303293 |
| 6 | 2.65033987 | 0.50906933 | 1.38751136 |
| 6 | 1.68469917 | 0.57870618 | 2.53879678 |
| 1 | 5.88680118 | 1.26437353 | 0.62530025 |
| 6 | 2.97193355 | -1.37834987 | -1.99490524 |
| 6 | -1.30456966 | -1.22347076 | 2.92581728 |
| 6 | -1.62249846 | -2.54017348 | 3.38164518 |
| 6 | -1.40857418 | -2.82185890 | 4.73719764 |
| 6 | -0.90359766 | -1.88004521 | 5.64073329 |

| | | | |
|---|---|---|---|
| 6 | -0.60562210 | -0.59943980 | 5.18964756 |
| 6 | -0.81366274 | -0.26136335 | 3.84699104 |
| 6 | -0.57980594 | 1.15339139 | 3.39737767 |
| 1 | -0.74781017 | -2.15155324 | 6.68476633 |
| 6 | -2.17475859 | -3.60828264 | 2.41851970 |
| 6 | 4.20277717 | -1.55106414 | -2.90496564 |
| 6 | 1.84863932 | -0.75624412 | -2.85393791 |
| 6 | 2.54874054 | -2.78826086 | -1.51672923 |
| 6 | -2.47210477 | -4.93599747 | 3.14029230 |
| 6 | -1.13806244 | -3.90744609 | 1.30966482 |
| 6 | -3.49969271 | -3.11990447 | 1.78529706 |
| 1 | 1.08063294 | 3.18264951 | 2.99605104 |
| 1 | 1.38459576 | 2.90462659 | 1.24879862 |
| 1 | 2.16223606 | 1.05887281 | 3.40946066 |
| 1 | 1.38065356 | -0.43379292 | 2.84063509 |
| 1 | 5.32008531 | -0.10181841 | -1.33798204 |
| 1 | 4.18508112 | 1.61176582 | 2.42598830 |
| 1 | -1.51794478 | 1.61672133 | 3.04921307 |
| 1 | -0.20206573 | 1.74923959 | 4.24484653 |
| 1 | -1.63678920 | -3.81877516 | 5.11060467 |
| 1 | -0.21854191 | 0.15522330 | 5.87859407 |
| 1 | 3.93393240 | -2.20550108 | -3.74635089 |
| 1 | 4.54211066 | -0.59384334 | -3.32718082 |
| 1 | 5.04673829 | -2.01907725 | -2.37750113 |
| 1 | 0.91228918 | -0.67577339 | -2.29254485 |
| 1 | 2.12913647 | 0.24560856 | -3.20662730 |
| 1 | 1.66299286 | -1.38325735 | -3.73879849 |
| 1 | 3.35809175 | -3.26345568 | -0.94309552 |
| 1 | 1.65304413 | -2.74642204 | -0.88695677 |
| 1 | 2.33153199 | -3.42564843 | -2.38749525 |
| 1 | -2.87078139 | -5.65644429 | 2.41179463 |
| 1 | -3.22440536 | -4.81633566 | 3.93367154 |
| 1 | -1.56785753 | -5.37978238 | 3.58171171 |
| 1 | -0.90330421 | -3.01324341 | 0.72294344 |
| 1 | -0.20407110 | -4.29050152 | 1.74605676 |
| 1 | -1.53389377 | -4.67599128 | 0.62817027 |
| 1 | -4.25793422 | -2.94519191 | 2.56305326 |
| 1 | -3.35613208 | -2.19191024 | 1.22145386 |
| 1 | -3.88904767 | -3.88954545 | 1.10118984 |
| 1 | -0.26564826 | 3.28341726 | 1.82067230 |
| 8 | -2.27539582 | 1.95217280 | -5.37582837 |
| 6 | -3.38285365 | 2.86993599 | -5.23737395 |
| 1 | -3.82048709 | 2.81578213 | -4.23053609 |
| 8 | -0.30201092 | 1.20584595 | -4.67099550 |
| 8 | -1.84891398 | 0.83957139 | -0.42520562 |
| 1 | -4.12474868 | 2.52930061 | -5.96633052 |
| 1 | -3.09574921 | 3.90337794 | -5.47511739 |
| 31 | -0.43255660 | 0.29982455 | 0.63875977 |
| 6 | -1.14678271 | 2.06596557 | -4.61240010 |
| 6 | -0.97639955 | 3.34768156 | -3.77248763 |
| 8 | -0.05694642 | 3.11383091 | -2.67516273 |
| 6 | -0.38695150 | 2.34276205 | -1.61355553 |
| 6 | -1.82093920 | 1.86569585 | -1.36087515 |

| | | | |
|---|---|---|---|
| 8 | 0.49581953 | 2.07756596 | -0.80878412 |
| 6 | -2.68063695 | 3.04868306 | -0.85554218 |
| 6 | -0.31845520 | 4.43386461 | -4.62356296 |
| 1 | -1.93795314 | 3.69942446 | -3.38064732 |
| 1 | -2.24126435 | 1.49152690 | -2.31614463 |
| 1 | -3.68491848 | 2.66478941 | -0.63889843 |
| 1 | -2.76626833 | 3.87976434 | -1.57021168 |
| 1 | -2.25504476 | 3.43969283 | 0.07837965 |
| 1 | -0.93316305 | 4.67270646 | -5.50106472 |
| 1 | 0.66262274 | 4.08408197 | -4.96716088 |
| 1 | -0.18492304 | 5.34473151 | -4.02705791 |

Electronic energy = -3673.027334 a.u.
DFT-D3(BJ) dispersion correction = -0.116528 a.u.
Thermal free energy = 0.581926 a.u.
Gibbs free energy = -3672.561936 a.u.
Number of imaginary frequencies = 0.

**Int2 LA isomer/conformer 77**

| Atomic N. | X | Y | Z |
|---|---|---|---|
| 7 | 0.37069999 | 0.97059608 | -2.48207010 |
| 8 | 0.95638994 | 1.04798140 | 0.46526134 |
| 8 | -1.67366966 | -0.22439331 | -0.65379092 |
| 6 | 1.47459104 | 0.51443782 | -3.36128063 |
| 6 | 1.88977293 | 1.96410918 | 0.09318664 |
| 6 | 2.91638630 | 2.34024698 | 1.00850263 |
| 6 | 3.87645171 | 3.25630516 | 0.55393061 |
| 6 | 3.84613245 | 3.81978556 | -0.72445040 |
| 6 | 2.80049856 | 3.49706123 | -1.58159790 |
| 6 | 1.81234729 | 2.59169823 | -1.17768608 |
| 6 | 0.59879383 | 2.39358240 | -2.04418564 |
| 1 | 4.62106799 | 4.52155854 | -1.03244241 |
| 6 | 2.96033743 | 1.79782279 | 2.45027839 |
| 6 | -2.46781957 | 0.74557760 | -1.17386683 |
| 6 | -3.64658202 | 1.14572393 | -0.48046246 |
| 6 | -4.43472591 | 2.13767296 | -1.08130630 |
| 6 | -4.10896089 | 2.73092105 | -2.30478618 |
| 6 | -2.96020171 | 2.32540465 | -2.97362076 |
| 6 | -2.14260388 | 1.33006640 | -2.42454280 |
| 6 | -0.95220471 | 0.84099276 | -3.20590681 |
| 1 | -4.75309923 | 3.50208778 | -2.72702797 |
| 6 | -4.04621861 | 0.51616168 | 0.86855380 |
| 6 | 4.12980330 | 2.39550383 | 3.25525177 |
| 6 | 3.13593090 | 0.26345427 | 2.44722250 |
| 6 | 1.65236108 | 2.17219354 | 3.18844121 |
| 6 | -5.35892576 | 1.11290226 | 1.41074530 |
| 6 | -2.95097407 | 0.77553831 | 1.93024167 |
| 6 | -4.26486885 | -1.00604455 | 0.69689447 |
| 1 | 1.52357367 | 1.14961357 | -4.26063955 |
| 1 | 2.42580250 | 0.58057091 | -2.82233358 |
| 1 | 0.67113444 | 3.02385606 | -2.94663442 |
| 1 | -0.31232067 | 2.68940806 | -1.50336243 |
| 1 | 4.68362508 | 3.54689679 | 1.22358421 |

| | | | |
|---|---|---|---|
| 1 | 2.72542840 | 3.96411315 | -2.56629136 |
| 1 | -1.05756482 | -0.22813957 | -3.45099654 |
| 1 | -0.87653437 | 1.39877702 | -4.15422361 |
| 1 | -5.33946517 | 2.46934079 | -0.57541087 |
| 1 | -2.69262583 | 2.76808813 | -3.93569296 |
| 1 | 4.10008141 | 1.99127161 | 4.27697064 |
| 1 | 5.10767328 | 2.13343297 | 2.82519831 |
| 1 | 4.06483107 | 3.49050615 | 3.33140521 |
| 1 | 2.28290445 | -0.23776151 | 1.97954954 |
| 1 | 4.05716869 | -0.01863830 | 1.91491171 |
| 1 | 3.21786053 | -0.09952344 | 3.48340041 |
| 1 | 1.53153755 | 3.26445294 | 3.23198106 |
| 1 | 0.77364048 | 1.74088971 | 2.69668036 |
| 1 | 1.68926429 | 1.79450380 | 4.22136783 |
| 1 | -5.60172086 | 0.62664381 | 2.36610385 |
| 1 | -6.20475719 | 0.94152729 | 0.72916861 |
| 1 | -5.27711415 | 2.19299135 | 1.60165365 |
| 1 | -2.00078390 | 0.30250647 | 1.66262895 |
| 1 | -2.78158439 | 1.85436362 | 2.06120762 |
| 1 | -3.27323747 | 0.36467188 | 2.89887922 |
| 1 | -5.07166673 | -1.20358015 | -0.02387983 |
| 1 | -3.35738702 | -1.51116896 | 0.34956266 |
| 1 | -4.56003036 | -1.44582188 | 1.66199004 |
| 1 | 1.30060547 | -0.52427079 | -3.66443425 |
| 8 | 1.62243713 | -3.97464846 | 1.34056448 |
| 6 | 2.81141104 | -3.30649327 | 1.83197976 |
| 1 | 3.03733763 | -2.44705120 | 1.19015362 |
| 8 | 0.37854985 | -2.19088291 | 2.02263368 |
| 8 | 0.97017502 | -1.76960106 | -0.89987562 |
| 1 | 3.60758403 | -4.05532491 | 1.77925890 |
| 1 | 2.65901016 | -2.96467951 | 2.86236921 |
| 31 | 0.17259324 | -0.12073330 | -0.73850275 |
| 6 | 0.47171193 | -3.26940044 | 1.47170866 |
| 6 | -0.73879624 | -4.04575519 | 0.91710793 |
| 8 | -1.40232631 | -3.36769007 | -0.18515752 |
| 6 | -0.94063344 | -3.30758377 | -1.48261958 |
| 6 | 0.44632758 | -2.70313079 | -1.82939329 |
| 8 | -1.69829967 | -3.61556653 | -2.37779566 |
| 6 | 1.48535001 | -3.79987257 | -2.09406258 |
| 6 | -0.51209555 | -5.51899002 | 0.60797688 |
| 1 | -1.49290355 | -3.95385383 | 1.71236596 |
| 1 | 0.22928066 | -2.22864536 | -2.80605768 |
| 1 | 2.38236809 | -3.34045843 | -2.53015773 |
| 1 | 1.08995772 | -4.54309817 | -2.79945593 |
| 1 | 1.77758976 | -4.29786400 | -1.16181082 |
| 1 | -0.16427255 | -6.04027762 | 1.50957677 |
| 1 | 0.23803129 | -5.66768315 | -0.17772043 |
| 1 | -1.45943609 | -5.96918543 | 0.28508305 |

Electronic energy = -3673.024473 a.u.
DFT-D3(BJ) dispersion correction = -0.119023 a.u.
Thermal free energy = 0.584233 a.u.
Gibbs free energy = -3672.559263 a.u.
Number of imaginary frequencies = 0.

**Int2 LA isomer/conformer 78**

| Atomic N. | X | Y | Z |
|---|---|---|---|
| 7 | 1.90498947 | -1.85857495 | 0.72366801 |
| 8 | -0.81172562 | -0.80430269 | -0.10982522 |
| 8 | 0.46158485 | 0.05205218 | 2.47249028 |
| 6 | 3.02783295 | -1.98931150 | -0.23703244 |
| 6 | -0.76746440 | -1.83139637 | -0.99342959 |
| 6 | -1.65479099 | -1.85907236 | -2.10679163 |
| 6 | -1.53184100 | -2.93557015 | -2.99722771 |
| 6 | -0.60281581 | -3.96500804 | -2.81985689 |
| 6 | 0.22236151 | -3.95219776 | -1.69984604 |
| 6 | 0.13656423 | -2.90416913 | -0.77742461 |
| 6 | 0.90582897 | -2.97496942 | 0.51364480 |
| 1 | -0.54064551 | -4.77587200 | -3.54567832 |
| 6 | -2.72248909 | -0.76649744 | -2.31517088 |
| 6 | 0.49534183 | -0.94701643 | 3.36856149 |
| 6 | -0.40492568 | -0.95658093 | 4.47779352 |
| 6 | -0.30196592 | -2.02316010 | 5.38060065 |
| 6 | 0.63038113 | -3.05658899 | 5.23577273 |
| 6 | 1.50811592 | -3.03273093 | 4.15794603 |
| 6 | 1.45559890 | -1.98250225 | 3.23384156 |
| 6 | 2.47198556 | -1.91430478 | 2.12975143 |
| 1 | 0.66478499 | -3.86841982 | 5.96222713 |
| 6 | -1.44912044 | 0.15953506 | 4.67099684 |
| 6 | -3.57702038 | -1.03017487 | -3.56931276 |
| 6 | -2.05950207 | 0.61909982 | -2.49863314 |
| 6 | -3.67824836 | -0.73517619 | -1.09866050 |
| 6 | -2.28318879 | -0.04698046 | 5.94917270 |
| 6 | -2.43181976 | 0.17755127 | 3.47613718 |
| 6 | -0.74386383 | 1.53138721 | 4.79506988 |
| 1 | 3.57121532 | -2.92730456 | -0.03737652 |
| 1 | 2.64276508 | -1.99654590 | -1.25940765 |
| 1 | 1.44998173 | -3.93190692 | 0.58209509 |
| 1 | 0.21760923 | -2.91922274 | 1.36915376 |
| 1 | -2.18199141 | -2.97560922 | -3.86933701 |
| 1 | 0.92561116 | -4.76874898 | -1.51931755 |
| 1 | 3.09899288 | -1.01262654 | 2.22702228 |
| 1 | 3.13390453 | -2.79434345 | 2.18615275 |
| 1 | -0.97920700 | -2.06004171 | 6.23227053 |
| 1 | 2.25064814 | -3.82392208 | 4.02917793 |
| 1 | -4.33143764 | -0.23573692 | -3.66039278 |
| 1 | -2.97461129 | -1.02007073 | -4.48900385 |
| 1 | -4.11189352 | -1.98913506 | -3.51066672 |
| 1 | -1.49881295 | 0.90716572 | -1.60174426 |
| 1 | -1.38293447 | 0.61469912 | -3.36447730 |
| 1 | -2.83620736 | 1.37802367 | -2.67967323 |
| 1 | -4.19220412 | -1.70084687 | -0.98511517 |
| 1 | -3.13943188 | -0.51629857 | -0.16990236 |
| 1 | -4.44525420 | 0.04030516 | -1.24797107 |
| 1 | -3.00265205 | 0.77902040 | 6.04318565 |
| 1 | -1.65922801 | -0.04759255 | 6.85486846 |

| | | | |
|---|---|---|---|
| 1 | -2.85857487 | -0.98379717 | 5.92204627 |
| 1 | -1.91385372 | 0.36320354 | 2.52943796 |
| 1 | -2.96389593 | -0.78168152 | 3.39748554 |
| 1 | -3.18166321 | 0.96998704 | 3.62350244 |
| 1 | -0.07360318 | 1.54356795 | 5.66735295 |
| 1 | -0.15705172 | 1.76207555 | 3.89934281 |
| 1 | -1.49567659 | 2.32317594 | 4.93632036 |
| 1 | 3.71492771 | -1.14312160 | -0.11496993 |
| 8 | -0.18129983 | 4.38684805 | -4.50002612 |
| 6 | 0.24903512 | 5.46890703 | -3.64526975 |
| 1 | 0.09696853 | 5.22387188 | -2.58456765 |
| 8 | -0.03307951 | 2.25756250 | -5.12478235 |
| 8 | 1.24630693 | 1.65914795 | 0.43938478 |
| 1 | -0.39563789 | 6.31270408 | -3.91034805 |
| 1 | 1.29607459 | 5.74839347 | -3.82855461 |
| 31 | 0.76217404 | -0.11949114 | 0.62168638 |
| 6 | 0.41916852 | 3.15903331 | -4.46289056 |
| 6 | 1.71337111 | 3.01675157 | -3.63290506 |
| 8 | 1.80366065 | 1.63493768 | -3.19537373 |
| 6 | 1.64691168 | 1.26804581 | -1.90638359 |
| 6 | 1.61823528 | 2.27564264 | -0.74783198 |
| 8 | 1.57589746 | 0.06843297 | -1.66771622 |
| 6 | 3.01158276 | 2.91957532 | -0.56852757 |
| 6 | 2.91953229 | 3.29185126 | -4.52920238 |
| 1 | 1.70709371 | 3.68784262 | -2.76984264 |
| 1 | 0.87890226 | 3.06602622 | -0.99754471 |
| 1 | 2.96554065 | 3.57721518 | 0.30781409 |
| 1 | 3.34477725 | 3.50876496 | -1.43437646 |
| 1 | 3.75498656 | 2.13618230 | -0.36954386 |
| 1 | 2.86925024 | 4.30431363 | -4.95258860 |
| 1 | 2.93098032 | 2.56943831 | -5.35443473 |
| 1 | 3.85015396 | 3.19628341 | -3.95501855 |

Electronic energy = -3673.027004 a.u.
DFT-D3(BJ) dispersion correction = -0.116264 a.u.
Thermal free energy = 0.584632 a.u.
Gibbs free energy = -3672.558636 a.u.
Number of imaginary frequencies = 0.

**Int2 LA isomer/conformer 79**

| Atomic N. | X | Y | Z |
|---|---|---|---|
| 8 | 6.69591345 | 3.20825279 | -1.03133976 |
| 6 | 6.93704691 | 4.16306980 | -2.08879043 |
| 1 | 6.76329086 | 3.71303917 | -3.07574857 |
| 8 | 5.35715193 | 1.73524615 | -0.04807990 |
| 8 | 0.33077085 | 1.60323568 | -0.21757353 |
| 1 | 7.99541285 | 4.42607375 | -1.99581003 |
| 1 | 6.33177558 | 5.07232462 | -1.96972626 |
| 7 | -0.77814166 | -0.95660069 | -2.17894053 |
| 8 | -1.98423059 | -0.03058108 | 0.37255806 |
| 8 | 0.69561403 | -1.51414196 | 0.40308178 |
| 6 | -0.65761883 | -0.03374792 | -3.33694667 |
| 6 | -3.04783484 | 0.27073665 | -0.40753188 |

| | | | |
|---|---|---|---|
| 6 | -4.05036179 | 1.17970854 | 0.04144305 |
| 6 | -5.11756455 | 1.43741943 | -0.83170691 |
| 6 | -5.23213592 | 0.84137197 | -2.09163818 |
| 6 | -4.26286587 | -0.06705060 | -2.50559128 |
| 6 | -3.18305219 | -0.36596953 | -1.66881766 |
| 6 | -2.20216165 | -1.44133212 | -2.04081836 |
| 1 | -6.07992870 | 1.07981830 | -2.73396465 |
| 6 | -3.96965239 | 1.84936957 | 1.42676340 |
| 6 | 0.42226580 | -2.80445189 | 0.08081200 |
| 6 | 0.47611748 | -3.81205134 | 1.08887794 |
| 6 | 0.19700611 | -5.12928610 | 0.69814545 |
| 6 | -0.12895488 | -5.47796608 | -0.61516996 |
| 6 | -0.17172162 | -4.48727539 | -1.58912781 |
| 6 | 0.11307076 | -3.15656222 | -1.25954959 |
| 6 | 0.16147691 | -2.12925509 | -2.36296058 |
| 1 | -0.34617113 | -6.51554442 | -0.86864758 |
| 6 | 0.82718333 | -3.47665965 | 2.55237256 |
| 6 | -5.19563809 | 2.73809439 | 1.70904926 |
| 6 | -2.71516527 | 2.75059651 | 1.50855131 |
| 6 | -3.91905888 | 0.77040201 | 2.53482270 |
| 6 | 0.83061744 | -4.73176429 | 3.44598602 |
| 6 | -0.21567728 | -2.49891026 | 3.14373834 |
| 6 | 2.24274193 | -2.85761060 | 2.62900223 |
| 1 | -0.95461297 | -0.55934360 | -4.25910576 |
| 1 | -1.31859129 | 0.82697574 | -3.18644142 |
| 1 | -2.49038081 | -1.91431961 | -2.99458613 |
| 1 | -2.17838052 | -2.22201850 | -1.26705505 |
| 1 | -5.89444990 | 2.13454514 | -0.52220551 |
| 1 | -4.34745376 | -0.56843363 | -3.47273632 |
| 1 | 1.16492017 | -1.69125298 | -2.44923311 |
| 1 | -0.09080657 | -2.61096220 | -3.32227356 |
| 1 | 0.22619870 | -5.91873433 | 1.44677508 |
| 1 | -0.41463313 | -4.73897916 | -2.62402423 |
| 1 | -5.09512952 | 3.17584183 | 2.71258045 |
| 1 | -5.27980069 | 3.56939503 | 0.99389119 |
| 1 | -6.13457150 | 2.16579149 | 1.68970870 |
| 1 | -1.79257621 | 2.18391317 | 1.34346990 |
| 1 | -2.76587094 | 3.55067373 | 0.75528419 |
| 1 | -2.66278469 | 3.22338724 | 2.50170689 |
| 1 | -4.82634031 | 0.14897529 | 2.51123189 |
| 1 | -3.04683436 | 0.11775200 | 2.42112426 |
| 1 | -3.86743448 | 1.25497839 | 3.52191776 |
| 1 | 1.09409588 | -4.43701189 | 4.47177438 |
| 1 | 1.57123268 | -5.47424127 | 3.11519130 |
| 1 | -0.15593165 | -5.21594381 | 3.48492638 |
| 1 | -0.24442272 | -1.55218016 | 2.59484725 |
| 1 | -1.22139245 | -2.94266198 | 3.11787853 |
| 1 | 0.03274218 | -2.28337148 | 4.19416402 |
| 1 | 2.99697191 | -3.56962953 | 2.26353713 |
| 1 | 2.31494142 | -1.94249501 | 2.03213699 |
| 1 | 2.48667143 | -2.61313757 | 3.67409563 |
| 1 | 0.38000824 | 0.30363914 | -3.42159338 |
| 31 | -0.26408034 | -0.12431539 | -0.36954885 |

| | | | |
|---|---|---|---|
| 6 | 5.47061512 | 2.63701487 | -0.84533143 |
| 6 | 4.26833473 | 3.21602727 | -1.61802683 |
| 8 | 3.41615910 | 2.12208373 | -2.03120909 |
| 6 | 2.44646393 | 1.42907162 | -1.35199968 |
| 6 | 1.68977849 | 1.96805914 | -0.12083413 |
| 8 | 2.11098958 | 0.37060398 | -1.85500496 |
| 6 | 2.33189711 | 1.47263182 | 1.18338607 |
| 6 | 3.65204685 | 4.38357418 | -0.84218808 |
| 1 | 4.60225015 | 3.59697823 | -2.59175237 |
| 1 | 1.70440549 | 3.06603336 | -0.14788403 |
| 1 | 1.72263657 | 1.83666109 | 2.02086655 |
| 1 | 3.36645392 | 1.81737320 | 1.30029464 |
| 1 | 2.33796178 | 0.37425558 | 1.20098751 |
| 1 | 4.36476811 | 5.22081926 | -0.84598881 |
| 1 | 3.44221191 | 4.13278776 | 0.20297734 |
| 1 | 2.73220324 | 4.72719070 | -1.33245528 |

Electronic energy = -3673.022235 a.u.
DFT-D3(BJ) dispersion correction = -0.115375 a.u.
Thermal free energy = 0.581226 a.u.
Gibbs free energy = -3672.556384 a.u.
Number of imaginary frequencies = 0.

**Int2_LA isomer/conformer 80**

| Atomic N. | X | Y | Z |
|---|---|---|---|
| 7 | 1.60198959 | -2.20120243 | -0.22047760 |
| 8 | -0.94197512 | -0.56047299 | -0.03289076 |
| 8 | 0.87901982 | -0.87023456 | 2.32716953 |
| 6 | 2.49760740 | -2.12726979 | -1.40137449 |
| 6 | -1.26208755 | -1.20923585 | -1.17559085 |
| 6 | -2.31582184 | -0.72654415 | -2.00504720 |
| 6 | -2.57656741 | -1.42984278 | -3.18998408 |
| 6 | -1.86280281 | -2.57130241 | -3.56628606 |
| 6 | -0.86912466 | -3.06045688 | -2.72391898 |
| 6 | -0.57431129 | -2.40137542 | -1.52632094 |
| 6 | 0.37710553 | -3.02633459 | -0.54140338 |
| 1 | -2.09776327 | -3.07985131 | -4.50132496 |
| 6 | -3.15243456 | 0.50618868 | -1.60730165 |
| 6 | 0.86829847 | -2.11651197 | 2.82303620 |
| 6 | 0.18735051 | -2.40328117 | 4.04622074 |
| 6 | 0.22792874 | -3.72421993 | 4.51137108 |
| 6 | 0.89721290 | -4.74998053 | 3.83437500 |
| 6 | 1.56577683 | -4.45829459 | 2.65086828 |
| 6 | 1.56679987 | -3.15141636 | 2.14902233 |
| 6 | 2.37524889 | -2.82769913 | 0.92495214 |
| 1 | 0.89333503 | -5.76367884 | 4.23488930 |
| 6 | -0.56289524 | -1.30292538 | 4.82056044 |
| 6 | -4.24905614 | 0.81745266 | -2.64303034 |
| 6 | -2.25507052 | 1.76213157 | -1.49826066 |
| 6 | -3.85285139 | 0.24913427 | -0.25175381 |
| 6 | -1.18855962 | -1.83870769 | 6.12190011 |
| 6 | -1.71275900 | -0.73620822 | 3.95415645 |
| 6 | 0.41316973 | -0.16688455 | 5.20972495 |

| | | | |
|---|---|---|---|
| 1 | 2.86838836 | -3.13715719 | -1.64130333 |
| 1 | 1.95225824 | -1.72101599 | -2.25628781 |
| 1 | 0.71767936 | -4.00781630 | -0.91194212 |
| 1 | -0.12884411 | -3.19014698 | 0.42091390 |
| 1 | -3.36597474 | -1.07762055 | -3.85187404 |
| 1 | -0.32777248 | -3.97470481 | -2.97864023 |
| 1 | 3.17784485 | -2.10975013 | 1.16112465 |
| 1 | 2.84669429 | -3.74836525 | 0.54306098 |
| 1 | -0.28706880 | -3.97295661 | 5.43783721 |
| 1 | 2.10436860 | -5.24051758 | 2.11047320 |
| 1 | -4.82565764 | 1.69004845 | -2.30367578 |
| 1 | -3.82987958 | 1.05895372 | -3.63094977 |
| 1 | -4.95370103 | -0.01804675 | -2.76055551 |
| 1 | -1.50215021 | 1.63772502 | -0.71179119 |
| 1 | -1.74555163 | 1.95375971 | -2.45458268 |
| 1 | -2.87389603 | 2.63968730 | -1.25340376 |
| 1 | -4.53613735 | -0.60895097 | -0.32788401 |
| 1 | -3.12597836 | 0.04582252 | 0.54241192 |
| 1 | -4.44732450 | 1.13016977 | 0.03563636 |
| 1 | -1.70066711 | -1.01384774 | 6.63780846 |
| 1 | -0.43057906 | -2.24007620 | 6.81033549 |
| 1 | -1.93561464 | -2.62293160 | 5.93101123 |
| 1 | -1.33781314 | -0.29815661 | 3.02317970 |
| 1 | -2.43259203 | -1.52797064 | 3.69999221 |
| 1 | -2.25058718 | 0.04409200 | 4.51447195 |
| 1 | 1.21240432 | -0.55085577 | 5.86085525 |
| 1 | 0.87122192 | 0.28842680 | 4.32506150 |
| 1 | -0.12909959 | 0.61342485 | 5.76579753 |
| 1 | 3.34937999 | -1.47476409 | -1.17487934 |
| 8 | 2.50602796 | 5.21175052 | -4.58319738 |
| 6 | 1.25175396 | 5.61452565 | -5.17903098 |
| 1 | 0.75394660 | 4.76522488 | -5.66633472 |
| 8 | 3.64981985 | 3.86311887 | -3.24221472 |
| 8 | 1.64472853 | 1.23830816 | 0.78677871 |
| 1 | 1.52535106 | 6.35388923 | -5.93822077 |
| 1 | 0.58028188 | 6.08560665 | -4.44810363 |
| 31 | 0.84786365 | -0.40036864 | 0.50275089 |
| 6 | 2.56850542 | 4.27045231 | -3.59796612 |
| 6 | 1.24727849 | 3.82281626 | -2.94060656 |
| 8 | 1.32209066 | 2.39007068 | -2.71104123 |
| 6 | 1.46188561 | 1.66060381 | -1.58050067 |
| 6 | 1.89391209 | 2.18917953 | -0.20501907 |
| 8 | 1.24394002 | 0.45880108 | -1.70799867 |
| 6 | 3.39502400 | 2.53860284 | -0.21719249 |
| 6 | 0.89017138 | 4.74844231 | -1.77878881 |
| 1 | 0.43302722 | 3.87395019 | -3.67537503 |
| 1 | 1.30991069 | 3.09720471 | 0.02000597 |
| 1 | 3.66523335 | 2.87474871 | 0.79137386 |
| 1 | 3.65483629 | 3.30948282 | -0.95206544 |
| 1 | 3.97689430 | 1.63549752 | -0.44558415 |
| 1 | 0.67640672 | 5.74573365 | -2.18964860 |
| 1 | 1.70770517 | 4.85567860 | -1.05836198 |
| 1 | -0.01670851 | 4.39924558 | -1.26914117 |

```
Electronic energy = -3673.019535 a.u.
DFT-D3(BJ) dispersion correction = -0.116500 a.u.
Thermal free energy = 0.584980 a.u.
Gibbs free energy = -3672.551054 a.u.
Number of imaginary frequencies = 0.
```

## Propagation step of CL addition to the product of CL addition on initiation step

**Int2 CL vdW CL isomer/conformer 1**

| Atomic N. | X | Y | Z |
|---|---|---|---|
| 7 | -0.24317908 | 1.71539183 | 2.10187375 |
| 8 | -0.99994230 | 1.51054737 | -0.78980760 |
| 8 | 1.97090930 | 1.71520926 | 0.05372383 |
| 6 | -0.74952767 | 0.72566462 | 3.08946797 |
| 6 | -2.26584871 | 1.64647666 | -0.31412745 |
| 6 | -3.38122596 | 1.31446050 | -1.13642286 |
| 6 | -4.65591113 | 1.43720754 | -0.56372871 |
| 6 | -4.86133435 | 1.88878361 | 0.74322695 |
| 6 | -3.76653658 | 2.26919337 | 1.51262985 |
| 6 | -2.47264915 | 2.16870413 | 0.98994164 |
| 6 | -1.30449746 | 2.72789862 | 1.75760695 |
| 6 | -3.20357279 | 0.87105232 | -2.60256472 |
| 6 | 2.14202746 | 2.99553454 | 0.47643857 |
| 6 | 2.86313300 | 3.92003611 | -0.33313909 |
| 6 | 3.01999609 | 5.22217815 | 0.16334010 |
| 6 | 2.50211036 | 5.62987425 | 1.39567683 |
| 6 | 1.81076485 | 4.71355563 | 2.18044675 |
| 6 | 1.63591893 | 3.39591293 | 1.74078555 |
| 6 | 0.98957424 | 2.39195324 | 2.66225951 |
| 6 | 3.44947232 | 3.51193739 | -1.69953513 |
| 6 | -4.55702556 | 0.62515601 | -3.29590371 |
| 6 | -2.40370154 | -0.44939347 | -2.68298576 |
| 6 | -2.47012309 | 1.98038657 | -3.39388252 |
| 6 | 4.21275202 | 4.67170240 | -2.36698297 |
| 6 | 2.31532854 | 3.09279881 | -2.66446023 |
| 6 | 4.45115942 | 2.34551500 | -1.52214878 |
| 1 | -1.01662333 | 1.24297882 | 4.02531794 |
| 1 | -1.64270767 | 0.23422140 | 2.68765624 |
| 1 | -1.65138084 | 3.19167309 | 2.69633492 |
| 1 | -0.79620905 | 3.50274140 | 1.16472562 |
| 1 | -5.52757091 | 1.16826319 | -1.15735555 |
| 1 | -3.90715601 | 2.66836434 | 2.51980666 |
| 1 | 1.68294366 | 1.57354459 | 2.91297047 |
| 1 | 0.69913538 | 2.88884641 | 3.60295126 |
| 1 | 3.56094488 | 5.95370389 | -0.43409667 |
| 1 | 1.41123877 | 5.00826289 | 3.15341363 |
| 1 | -4.37389747 | 0.33689654 | -4.34084623 |
| 1 | -5.12179652 | -0.19130414 | -2.82352930 |
| 1 | -5.18544631 | 1.52740782 | -3.30738047 |
| 1 | -1.40221600 | -0.34201844 | -2.25187886 |

| | | | |
|---|---|---|---|
| 1 | -2.93673409 | -1.25411491 | -2.15708766 |
| 1 | -2.29360342 | -0.75194532 | -3.73560869 |
| 1 | -3.05223595 | 2.91343181 | -3.38211164 |
| 1 | -1.47710264 | 2.18361089 | -2.97755722 |
| 1 | -2.35103030 | 1.67021439 | -4.44323266 |
| 1 | 4.61873570 | 4.32518713 | -3.32801534 |
| 1 | 5.05884660 | 5.01700332 | -1.75525935 |
| 1 | 3.56005785 | 5.53151293 | -2.57599533 |
| 1 | 1.75646921 | 2.23082410 | -2.28547841 |
| 1 | 1.60987040 | 3.92207429 | -2.81754133 |
| 1 | 2.74071868 | 2.82453480 | -3.64354366 |
| 1 | 5.28196898 | 2.64640025 | -0.86681096 |
| 1 | 3.96526593 | 1.46354771 | -1.09071886 |
| 1 | 4.87486588 | 2.06812517 | -2.49945507 |
| 1 | 0.03259501 | -0.01487177 | 3.29472355 |
| 8 | 2.34638670 | -0.63822417 | 3.43719770 |
| 8 | 2.75560758 | -2.71920911 | 2.81726593 |
| 6 | 2.94419163 | -1.37662785 | 2.67064386 |
| 6 | 3.89582266 | -0.87647163 | 1.60280401 |
| 1 | 3.57035937 | -1.25069633 | 0.61918390 |
| 1 | 3.77128147 | 0.21157570 | 1.58109725 |
| 6 | 5.37275675 | -1.24390861 | 1.85872836 |
| 1 | 5.61494671 | -1.07300450 | 2.92029939 |
| 1 | 5.99683999 | -0.54304568 | 1.28462037 |
| 6 | 5.74228646 | -2.67464353 | 1.45413792 |
| 1 | 5.60144996 | -2.77995858 | 0.36448486 |
| 1 | 6.81419836 | -2.84081099 | 1.64386932 |
| 6 | 4.92944573 | -3.76529298 | 2.15981846 |
| 1 | 5.12343357 | -3.76140859 | 3.24489838 |
| 1 | 5.25349370 | -4.75164959 | 1.78946691 |
| 6 | 3.42191797 | -3.66964763 | 1.94139634 |
| 1 | 3.17869440 | -3.43768443 | 0.89233134 |
| 1 | 2.93838146 | -4.62009813 | 2.19961899 |
| 31 | 0.34768271 | 0.86592654 | 0.33099616 |
| 1 | -5.87240252 | 1.95833454 | 1.14438530 |
| 1 | 2.64308200 | 6.65581935 | 1.73566409 |
| 8 | -5.75433323 | -5.98385642 | -0.47870125 |
| 6 | -7.12504597 | -5.84077941 | -0.92181101 |
| 1 | -7.59462141 | -4.97206618 | -0.44342760 |
| 6 | -3.54039618 | -5.16221146 | -0.26368223 |
| 6 | -2.60015556 | -3.98267377 | -0.50762894 |
| 6 | -1.17656754 | -4.25836300 | -0.00819139 |
| 6 | -4.95177889 | -4.91493017 | -0.76072185 |
| 6 | -0.17778433 | -3.12429290 | -0.28647270 |
| 6 | -0.45377276 | -1.84668057 | 0.50666618 |
| 1 | -3.59894624 | -5.41157538 | 0.80833242 |
| 1 | -3.01632149 | -3.08976644 | -0.01884342 |
| 1 | -1.19962447 | -4.46443015 | 1.07658685 |
| 1 | -0.16472073 | -2.88403261 | -1.36193803 |
| 1 | -3.16779450 | -6.07563235 | -0.75653621 |
| 1 | -2.57734224 | -3.75498081 | -1.58438455 |
| 1 | -0.80363421 | -5.18048352 | -0.48485223 |
| 1 | 0.83788764 | -3.46283982 | -0.02685349 |

| | | | |
|---|---|---|---|
| 1 | -0.55747315 | -2.10599485 | 1.57907263 |
| 1 | -1.41281316 | -1.40400436 | 0.18236794 |
| 8 | -5.34382766 | -3.91677586 | -1.33506840 |
| 8 | 0.63334560 | -0.92959387 | 0.33883842 |
| 1 | -7.62474117 | -6.76659805 | -0.62080538 |
| 1 | -7.16541263 | -5.71356584 | -2.01098036 |

Electronic energy = -3908.675273 a.u.
DFT-D3(BJ) dispersion correction = -0.135842 a.u.
Thermal free energy = 0.732789 a.u.
Gibbs free energy = -3908.078326 a.u.
Number of imaginary frequencies = 0.

**Int2 CL vdW CL isomer/conformer 2**

| Atomic N. | X | Y | Z |
|---|---|---|---|
| 7 | -0.44475038 | 3.54468643 | -0.40653159 |
| 8 | -2.09212582 | 1.14547936 | -1.09784476 |
| 8 | 0.96537815 | 1.22749268 | -1.70489782 |
| 6 | -0.53691895 | 4.08181021 | 0.97424281 |
| 6 | -3.12399038 | 1.95933574 | -0.74612629 |
| 6 | -4.39497731 | 1.40379837 | -0.42169037 |
| 6 | -5.41687399 | 2.30028382 | -0.07601414 |
| 6 | -5.23298631 | 3.68532604 | -0.04242516 |
| 6 | -3.99258415 | 4.21553453 | -0.38021801 |
| 6 | -2.94149779 | 3.36691343 | -0.74503201 |
| 6 | -1.64205461 | 3.95291532 | -1.22837263 |
| 6 | -4.64319221 | -0.11723043 | -0.44448491 |
| 6 | 1.14823044 | 2.11548596 | -2.71655582 |
| 6 | 1.46658101 | 1.65214517 | -4.02588501 |
| 6 | 1.65405423 | 2.62185233 | -5.02121640 |
| 6 | 1.54278061 | 3.99341855 | -4.77561360 |
| 6 | 1.24592659 | 4.43266079 | -3.49073251 |
| 6 | 1.05925514 | 3.50785189 | -2.45627615 |
| 6 | 0.84073270 | 4.00819171 | -1.05214583 |
| 6 | 1.60620358 | 0.14960765 | -4.34068317 |
| 6 | -6.10391991 | -0.46815788 | -0.10411579 |
| 6 | -3.74599346 | -0.81360868 | 0.60539915 |
| 6 | -4.35145950 | -0.68461329 | -1.85424825 |
| 6 | 1.97724159 | -0.09756572 | -5.81518742 |
| 6 | 0.26765932 | -0.57993203 | -4.07731952 |
| 6 | 2.72877060 | -0.46834720 | -3.47375836 |
| 1 | -0.52613191 | 5.18348076 | 0.95066524 |
| 1 | -1.46676675 | 3.73803318 | 1.43948046 |
| 1 | -1.69800144 | 5.05444934 | -1.23177385 |
| 1 | -1.42541617 | 3.62251892 | -2.25477477 |
| 1 | -6.39755186 | 1.90635945 | 0.18422084 |
| 1 | -3.83341239 | 5.29625451 | -0.38344613 |
| 1 | 1.64728970 | 3.66085101 | -0.38645420 |
| 1 | 0.84890838 | 5.11086184 | -1.04471579 |
| 1 | 1.89178762 | 2.29943563 | -6.03316925 |
| 1 | 1.16854202 | 5.50044913 | -3.27422815 |
| 1 | -6.22731781 | -1.55972353 | -0.14643136 |
| 1 | -6.38417917 | -0.14445731 | 0.90860732 |

| | | | |
|---|---|---|---|
| 1 | -6.81264090 | -0.02750992 | -0.82007328 |
| 1 | -2.68271653 | -0.63238172 | 0.41535976 |
| 1 | -3.97962212 | -0.45302549 | 1.61744623 |
| 1 | -3.92663392 | -1.89975540 | 0.58403780 |
| 1 | -5.01461437 | -0.22438488 | -2.60115235 |
| 1 | -3.31350288 | -0.50646616 | -2.15521734 |
| 1 | -4.53783855 | -1.76951079 | -1.86255665 |
| 1 | 2.07460256 | -1.17976397 | -5.98165782 |
| 1 | 2.93780517 | 0.36518477 | -6.08350201 |
| 1 | 1.20594260 | 0.27386282 | -6.50524835 |
| 1 | -0.04110371 | -0.49819996 | -3.02977775 |
| 1 | -0.53304532 | -0.16409847 | -4.70559806 |
| 1 | 0.37192449 | -1.64712358 | -4.32643865 |
| 1 | 3.69483032 | 0.00899237 | -3.69312281 |
| 1 | 2.52174544 | -0.35404158 | -2.40434780 |
| 1 | 2.82586216 | -1.54126734 | -3.70015875 |
| 1 | 0.31289612 | 3.72025657 | 1.56489417 |
| 31 | -0.38550616 | 1.49101585 | -0.45352672 |
| 1 | -6.05780526 | 4.33963668 | 0.23930208 |
| 1 | 1.69146872 | 4.70770803 | -5.58514816 |
| 8 | 5.27485896 | -2.56207947 | 7.09125099 |
| 6 | 5.38685117 | -3.14616905 | 8.40979136 |
| 1 | 5.15598557 | -2.40160627 | 9.18246243 |
| 6 | 4.02069070 | -1.47206944 | 5.39968522 |
| 6 | 2.62332310 | -1.08703662 | 4.91547040 |
| 6 | 2.65355856 | -0.40734381 | 3.54252851 |
| 6 | 4.03216469 | -2.07784344 | 6.78860706 |
| 6 | 1.25011661 | -0.09687098 | 3.00993100 |
| 6 | 1.27510422 | 0.56437745 | 1.63593021 |
| 1 | 4.68607602 | -0.59180312 | 5.42259785 |
| 1 | 2.14662756 | -0.42783105 | 5.65693706 |
| 1 | 3.24469583 | 0.52387392 | 3.60737819 |
| 1 | 0.70809189 | 0.55721874 | 3.71249628 |
| 1 | 4.49693500 | -2.18413422 | 4.70819906 |
| 1 | 1.99880953 | -1.99165031 | 4.86192859 |
| 1 | 3.17604979 | -1.06354212 | 2.82695487 |
| 1 | 0.67126922 | -1.03073196 | 2.93727767 |
| 1 | 1.88088090 | -0.04428238 | 0.93903816 |
| 1 | 1.78029196 | 1.54915823 | 1.71319948 |
| 8 | 3.09064239 | -2.13230451 | 7.55719878 |
| 8 | -0.06961899 | 0.70946681 | 1.15162743 |
| 1 | 6.42585731 | -3.48012348 | 8.49169691 |
| 1 | 4.69735076 | -3.99341502 | 8.51468397 |
| 8 | 2.80069969 | -3.53235889 | 1.49934814 |
| 8 | 0.59855355 | -3.61083016 | 1.73618753 |
| 6 | 1.71255640 | -3.39776797 | 0.97280483 |
| 6 | 1.53921159 | -3.02262530 | -0.49005519 |
| 1 | 0.94989959 | -2.09427986 | -0.57074221 |
| 1 | 2.54958696 | -2.80087195 | -0.85231909 |
| 6 | 0.88972855 | -4.12374378 | -1.35410872 |
| 1 | 1.33160002 | -5.09934496 | -1.09409236 |
| 1 | 1.16133561 | -3.93259787 | -2.40332025 |
| 6 | -0.63748923 | -4.18440863 | -1.24690479 |

| | | | |
|---|---|---|---|
| 1 | -1.05483346 | -3.23119733 | -1.61467742 |
| 1 | -1.01648713 | -4.96832508 | -1.92109420 |
| 6 | -1.16667180 | -4.43606027 | 0.16897306 |
| 1 | -0.86125280 | -5.43098481 | 0.53227473 |
| 1 | -2.26864696 | -4.43842010 | 0.14427843 |
| 6 | -0.73714219 | -3.39524656 | 1.19860935 |
| 1 | -0.81459849 | -2.37058436 | 0.80306918 |
| 1 | -1.37194989 | -3.45658557 | 2.09113710 |

Electronic energy = -3908.672027 a.u.
DFT-D3(BJ) dispersion correction = -0.134098 a.u.
Thermal free energy = 0.728846 a.u.
Gibbs free energy = -3908.077279 a.u.
Number of imaginary frequencies = 0.

**Int2 CL vdW CL isomer/conformer 3**

| Atomic N. | X | Y | Z |
|---|---|---|---|
| 7 | -1.39451210 | 1.26278149 | 1.14104709 |
| 8 | -2.95165321 | 1.71774964 | -1.36554833 |
| 8 | 0.12107271 | 2.26268243 | -1.25915178 |
| 6 | -1.53674813 | -0.04240077 | 1.83869435 |
| 6 | -4.01347875 | 1.47611322 | -0.55419691 |
| 6 | -5.27128471 | 1.10470302 | -1.11099787 |
| 6 | -6.32694238 | 0.88049347 | -0.21499341 |
| 6 | -6.18736710 | 1.00751694 | 1.17017299 |
| 6 | -4.95784631 | 1.38667375 | 1.69899541 |
| 6 | -3.87460903 | 1.63437553 | 0.84932704 |
| 6 | -2.57903225 | 2.15419398 | 1.41197727 |
| 6 | -5.46650614 | 0.95046824 | -2.63195257 |
| 6 | 0.31572647 | 3.35665455 | -0.47921356 |
| 6 | 0.69593399 | 4.59500171 | -1.07379822 |
| 6 | 0.89372857 | 5.68586775 | -0.21518993 |
| 6 | 0.73148912 | 5.59866783 | 1.17030953 |
| 6 | 0.37061216 | 4.38160195 | 1.73768375 |
| 6 | 0.17387538 | 3.25578112 | 0.92935414 |
| 6 | -0.10659342 | 1.92455013 | 1.57829480 |
| 6 | 0.88097259 | 4.73602947 | -2.59778809 |
| 6 | -6.91974386 | 0.58659771 | -2.99072264 |
| 6 | -4.56086878 | -0.18209221 | -3.17105895 |
| 6 | -5.13670471 | 2.28144871 | -3.34892855 |
| 6 | 1.32265006 | 6.15766444 | -2.99416344 |
| 6 | -0.45388885 | 4.45048442 | -3.32572125 |
| 6 | 1.97477468 | 3.76051452 | -3.09406567 |
| 1 | -1.62786689 | 0.12851066 | 2.92361534 |
| 1 | -2.43292139 | -0.55721868 | 1.47656188 |
| 1 | -2.66351559 | 2.28866733 | 2.50356309 |
| 1 | -2.32928480 | 3.12962692 | 0.96896316 |
| 1 | -7.29906924 | 0.58858388 | -0.60839579 |
| 1 | -4.83154171 | 1.51131559 | 2.77695957 |
| 1 | 0.68942423 | 1.19713758 | 1.35925984 |
| 1 | -0.14718147 | 2.04817419 | 2.67326471 |
| 1 | 1.17878286 | 6.64677064 | -0.63969372 |
| 1 | 0.25153095 | 4.28828890 | 2.81951021 |

| | | | |
|---|---|---|---|
| 1 | -7.00634771 | 0.50211501 | -4.08340761 |
| 1 | -7.22483003 | -0.37842724 | -2.56050621 |
| 1 | -7.63252030 | 1.35632471 | -2.66058345 |
| 1 | -3.49961200 | 0.01871649 | -2.98982277 |
| 1 | -4.81558285 | -1.14040639 | -2.69546573 |
| 1 | -4.71142434 | -0.29228675 | -4.25602376 |
| 1 | -5.80422648 | 3.08361227 | -3.00127211 |
| 1 | -4.10080281 | 2.58916490 | -3.17114728 |
| 1 | -5.28543249 | 2.16482753 | -4.43334817 |
| 1 | 1.45084174 | 6.19978065 | -4.08518049 |
| 1 | 2.28359684 | 6.43503543 | -2.53697448 |
| 1 | 0.57465878 | 6.91592618 | -2.72076456 |
| 1 | -0.80884292 | 3.43170371 | -3.13851631 |
| 1 | -1.23288606 | 5.15399633 | -2.99830153 |
| 1 | -0.31926312 | 4.57469264 | -4.41106104 |
| 1 | 2.93887233 | 3.98466315 | -2.61431304 |
| 1 | 1.71221321 | 2.71917869 | -2.87895602 |
| 1 | 2.10487165 | 3.87091259 | -4.18151670 |
| 1 | -0.64869947 | -0.65521578 | 1.64855543 |
| 8 | 1.92331040 | -0.92607880 | 2.14832918 |
| 8 | 1.61110635 | -2.80558044 | 3.27286815 |
| 6 | 2.43710411 | -1.88375672 | 2.70384422 |
| 6 | 3.93835622 | -2.07969831 | 2.80180425 |
| 1 | 4.21428538 | -3.05879031 | 2.37481973 |
| 1 | 4.37587415 | -1.29924009 | 2.16834932 |
| 6 | 4.48754117 | -1.96799145 | 4.24132196 |
| 1 | 4.01540889 | -1.11149675 | 4.74949531 |
| 1 | 5.56000921 | -1.73177902 | 4.17397023 |
| 6 | 4.31816091 | -3.24342398 | 5.07352701 |
| 1 | 4.89413360 | -4.05363035 | 4.59559054 |
| 1 | 4.76413698 | -3.08791191 | 6.06827174 |
| 6 | 2.86700902 | -3.70843544 | 5.23706973 |
| 1 | 2.27742128 | -2.97014253 | 5.80522643 |
| 1 | 2.85382781 | -4.63787526 | 5.82937897 |
| 6 | 2.14690708 | -3.99829275 | 3.92372270 |
| 1 | 2.78825720 | -4.54619256 | 3.21633044 |
| 1 | 1.24570228 | -4.59630606 | 4.10626452 |
| 31 | -1.26819792 | 1.07969894 | -0.90015955 |
| 1 | -7.03763531 | 0.81636738 | 1.82496331 |
| 1 | 0.88870051 | 6.47737806 | 1.79583002 |
| 8 | 4.70043202 | -6.77443502 | -0.46615709 |
| 6 | 5.09344365 | -7.84359839 | 0.43022400 |
| 1 | 5.93240731 | -7.52434282 | 1.06082003 |
| 6 | 3.90064226 | -4.56149718 | -0.84475496 |
| 6 | 2.44301104 | -4.10948942 | -0.64269291 |
| 6 | 2.09734767 | -2.84401316 | -1.43343373 |
| 6 | 4.29794882 | -5.63072617 | 0.14866891 |
| 6 | 0.67797507 | -2.34481828 | -1.14365328 |
| 6 | 0.35850717 | -1.02689823 | -1.84775716 |
| 1 | 4.57006072 | -3.70342750 | -0.66950618 |
| 1 | 2.27443084 | -3.92640996 | 0.42897090 |
| 1 | 2.82035283 | -2.05198272 | -1.17158060 |
| 1 | 0.55933180 | -2.19736350 | -0.05835049 |

| | | | |
|---|---|---|---|
| 1 | 4.07041180 | -4.91664713 | -1.86928382 |
| 1 | 1.76283313 | -4.92768901 | -0.93014539 |
| 1 | 2.22009053 | -3.03282136 | -2.51370212 |
| 1 | -0.06455196 | -3.10007690 | -1.44833057 |
| 1 | 0.42191984 | -1.16369552 | -2.94375624 |
| 1 | 1.11826389 | -0.27123590 | -1.57453979 |
| 8 | 4.26358167 | -5.50293122 | 1.36391884 |
| 8 | -0.96653009 | -0.59580283 | -1.51870557 |
| 1 | 5.38929737 | -8.67128914 | -0.22099036 |
| 1 | 4.25137636 | -8.13392447 | 1.07055173 |

Electronic energy = -3908.677412 a.u.
DFT-D3(BJ) dispersion correction = -0.131516 a.u.
Thermal free energy = 0.732284 a.u.
Gibbs free energy = -3908.076644 a.u.
Number of imaginary frequencies = 0.

**Int2 CL vdW CL isomer/conformer 4**

| Atomic N. | X | Y | Z |
|---|---|---|---|
| 7 | -0.66562921 | 1.92662355 | 1.18557374 |
| 8 | -2.62243247 | 1.38151294 | -1.02943828 |
| 8 | 0.39833172 | 1.25739310 | -1.55349669 |
| 6 | -0.78790723 | 1.20662240 | 2.48204819 |
| 6 | -3.53407330 | 1.80851140 | -0.11599987 |
| 6 | -4.91988101 | 1.53005761 | -0.29887203 |
| 6 | -5.80222164 | 1.98251401 | 0.69332118 |
| 6 | -5.37809429 | 2.69302688 | 1.81955991 |
| 6 | -4.02847895 | 2.99781175 | 1.96264112 |
| 6 | -3.10579439 | 2.57787621 | 0.99872659 |
| 6 | -1.67472281 | 3.04079816 | 1.08341911 |
| 6 | -5.43833971 | 0.78446466 | -1.54460459 |
| 6 | 0.86090250 | 2.53392278 | -1.50731353 |
| 6 | 1.22839351 | 3.20064004 | -2.71243733 |
| 6 | 1.71236008 | 4.51196039 | -2.60054278 |
| 6 | 1.83988961 | 5.16945830 | -1.37405276 |
| 6 | 1.48727589 | 4.50369121 | -0.20540040 |
| 6 | 1.01162929 | 3.18798093 | -0.25609128 |
| 6 | 0.74464924 | 2.45705227 | 1.03585080 |
| 6 | 1.10085669 | 2.51446332 | -4.08725680 |
| 6 | -6.97138489 | 0.63563448 | -1.53147180 |
| 6 | -4.83880835 | -0.63848418 | -1.61329616 |
| 6 | -5.06066963 | 1.57435916 | -2.82062237 |
| 6 | 1.58492145 | 3.42405941 | -5.23247107 |
| 6 | -0.37862814 | 2.16579346 | -4.37437301 |
| 6 | 1.96595145 | 1.23209659 | -4.12389473 |
| 1 | -0.58729937 | 1.90605126 | 3.30928445 |
| 1 | -1.80223208 | 0.80509594 | 2.57909012 |
| 1 | -1.54057791 | 3.70438667 | 1.95379053 |
| 1 | -1.40406185 | 3.60806837 | 0.18048562 |
| 1 | -6.86523562 | 1.77267298 | 0.58922536 |
| 1 | -3.67953771 | 3.58389557 | 2.81592522 |
| 1 | 1.40422251 | 1.58400704 | 1.15215456 |
| 1 | 0.94262539 | 3.12723726 | 1.88810255 |

| | | | |
|---|---|---|---|
| 1 | 1.99617909 | 5.04923741 | -3.50361493 |
| 1 | 1.59324322 | 4.99412614 | 0.76481733 |
| 1 | -7.28701604 | 0.11428936 | -2.44644018 |
| 1 | -7.32343484 | 0.04253877 | -0.67484675 |
| 1 | -7.48107707 | 1.60967857 | -1.51347956 |
| 1 | -3.74841803 | -0.60390454 | -1.70696534 |
| 1 | -5.09517960 | -1.21502438 | -0.71247860 |
| 1 | -5.24399717 | -1.17286442 | -2.48614937 |
| 1 | -5.52259924 | 2.57224903 | -2.80800763 |
| 1 | -3.97540945 | 1.69457879 | -2.91195433 |
| 1 | -5.42937071 | 1.04252077 | -3.71111853 |
| 1 | 1.48230749 | 2.88379613 | -6.18445977 |
| 1 | 2.64319059 | 3.70151526 | -5.12158654 |
| 1 | 0.98943544 | 4.34516129 | -5.31030941 |
| 1 | -0.78812472 | 1.48258882 | -3.62304178 |
| 1 | -0.99617202 | 3.07548800 | -4.38465763 |
| 1 | -0.46344450 | 1.68736372 | -5.36211350 |
| 1 | 3.02716150 | 1.47729121 | -3.97067813 |
| 1 | 1.65985278 | 0.51914365 | -3.35093167 |
| 1 | 1.87001166 | 0.74679583 | -5.10733888 |
| 1 | -0.06108823 | 0.38830333 | 2.51428017 |
| 8 | 2.73391484 | 0.30432336 | 2.85359549 |
| 8 | 1.61596660 | -1.43161743 | 3.64823317 |
| 6 | 2.79177756 | -0.81323525 | 3.33841794 |
| 6 | 4.09801825 | -1.52663998 | 3.63137151 |
| 1 | 4.12177300 | -2.48968075 | 3.09435947 |
| 1 | 4.87906313 | -0.88543532 | 3.20632198 |
| 6 | 4.36068700 | -1.75996051 | 5.13538475 |
| 1 | 4.10059255 | -0.85014690 | 5.70072671 |
| 1 | 5.44356409 | -1.90401961 | 5.26783636 |
| 6 | 3.63128092 | -2.97581467 | 5.71680197 |
| 1 | 3.99186011 | -3.88142024 | 5.20051557 |
| 1 | 3.90670976 | -3.08976549 | 6.77702260 |
| 6 | 2.10467718 | -2.91996593 | 5.59422283 |
| 1 | 1.69207933 | -2.08684118 | 6.18690900 |
| 1 | 1.67709223 | -3.84364165 | 6.01745509 |
| 6 | 1.59356479 | -2.79725091 | 4.16197029 |
| 1 | 2.14235595 | -3.45699494 | 3.47262697 |
| 1 | 0.52850411 | -3.05530914 | 4.11232049 |
| 31 | -0.97949928 | 0.72771244 | -0.41974890 |
| 1 | -6.10243316 | 3.01588823 | 2.56736083 |
| 1 | 2.21440267 | 6.19246756 | -1.33802512 |
| 8 | 4.07327873 | -5.66285838 | -0.17061806 |
| 6 | 4.43762485 | -6.79171127 | 0.66057948 |
| 1 | 5.26927948 | -6.52489230 | 1.32450490 |
| 6 | 3.24075513 | -3.45200519 | -0.42784947 |
| 6 | 1.71239104 | -3.45595097 | -0.64344887 |
| 6 | 1.26486416 | -2.28697812 | -1.52721145 |
| 6 | 3.66183333 | -4.55811434 | 0.50943911 |
| 6 | -0.23783716 | -2.27813975 | -1.84080310 |
| 6 | -1.15144683 | -2.13018427 | -0.61903219 |
| 1 | 3.54264087 | -2.49691374 | 0.02487998 |
| 1 | 1.21726421 | -3.39936464 | 0.33727257 |

| | | | |
|---|---|---|---|
| 1 | 1.53452134 | -1.33819753 | -1.03823561 |
| 1 | -0.52186226 | -3.20923340 | -2.36290477 |
| 1 | 3.75608637 | -3.56407531 | -1.39140501 |
| 1 | 1.41339083 | -4.41256126 | -1.10322822 |
| 1 | 1.82359159 | -2.31643976 | -2.47724709 |
| 1 | -0.44621187 | -1.45422945 | -2.54558336 |
| 1 | -1.03076167 | -3.00406481 | 0.04483645 |
| 1 | -2.20407320 | -2.13721637 | -0.95858330 |
| 8 | 3.61540410 | -4.50883315 | 1.73026704 |
| 8 | -0.87628085 | -0.98459042 | 0.18228962 |
| 1 | 4.73585275 | -7.57961006 | -0.03739085 |
| 1 | 3.58169451 | -7.11261589 | 1.26730468 |

Electronic energy = -3908.676864 a.u.
DFT-D3(BJ) dispersion correction = -0.133898 a.u.
Thermal free energy = 0.734852 a.u.
Gibbs free energy = -3908.075910 a.u.
Number of imaginary frequencies = 0.

**Int2 CL vdW CL isomer/conformer 5**

| Atomic N. | X | Y | Z |
|---|---|---|---|
| 7 | -1.05949342 | 1.99218270 | 2.35800066 |
| 8 | -2.23747541 | 2.15332004 | -0.38681001 |
| 8 | 0.80921244 | 1.83631804 | -0.01116241 |
| 6 | -1.52534005 | 0.98590617 | 3.35081802 |
| 6 | -3.39960789 | 2.35385741 | 0.29141885 |
| 6 | -4.65572436 | 2.16925158 | -0.35419890 |
| 6 | -5.80909450 | 2.39079772 | 0.41317488 |
| 6 | -5.76544792 | 2.78326158 | 1.75391208 |
| 6 | -4.53212088 | 2.98954887 | 2.36352221 |
| 6 | -3.35087146 | 2.79314289 | 1.64080134 |
| 6 | -2.02299178 | 3.14779207 | 2.25520177 |
| 6 | -4.75036556 | 1.75287499 | -1.83510268 |
| 6 | 1.21093285 | 3.05990724 | 0.42983205 |
| 6 | 1.92061209 | 3.93377227 | -0.44287386 |
| 6 | 2.31634578 | 5.17603230 | 0.07426326 |
| 6 | 2.03997875 | 5.57199516 | 1.38545386 |
| 6 | 1.35703422 | 4.70334267 | 2.22982937 |
| 6 | 0.95073406 | 3.44442842 | 1.77170829 |
| 6 | 0.32334626 | 2.47508152 | 2.74306565 |
| 6 | 2.24228863 | 3.53789978 | -1.89799316 |
| 6 | -6.21084871 | 1.65847806 | -2.31524336 |
| 6 | -4.10728533 | 0.36178167 | -2.04147869 |
| 6 | -4.03828957 | 2.80022375 | -2.72407556 |
| 6 | 3.04587594 | 4.63111005 | -2.62758605 |
| 6 | 0.93395660 | 3.32131193 | -2.69424400 |
| 6 | 3.09719465 | 2.24808339 | -1.92277987 |
| 1 | -1.57912975 | 1.45059163 | 4.34827389 |
| 1 | -2.51802225 | 0.62347667 | 3.06294430 |
| 1 | -2.16688960 | 3.56218503 | 3.26678617 |
| 1 | -1.51197979 | 3.90722808 | 1.64508612 |
| 1 | -6.78471779 | 2.24808759 | -0.04803524 |
| 1 | -4.47503829 | 3.32457667 | 3.40170730 |

| | | | |
|---|---|---|---|
| 1 | 0.93336327 | 1.56405688 | 2.84881799 |
| 1 | 0.24726384 | 2.94595442 | 3.73705660 |
| 1 | 2.85491473 | 5.86920372 | -0.56928247 |
| 1 | 1.14538699 | 4.98802838 | 3.26289899 |
| 1 | -6.21965839 | 1.37459549 | -3.37727826 |
| 1 | -6.78039873 | 0.89567342 | -1.76471810 |
| 1 | -6.73811263 | 2.61916317 | -2.22534160 |
| 1 | -3.04472259 | 0.37051885 | -1.77639715 |
| 1 | -4.61302614 | -0.39655213 | -1.42637386 |
| 1 | -4.19874854 | 0.06169168 | -3.09658348 |
| 1 | -4.51644836 | 3.78500640 | -2.61897362 |
| 1 | -2.97937630 | 2.89797919 | -2.46084574 |
| 1 | -4.11000486 | 2.50144607 | -3.78109923 |
| 1 | 3.25880031 | 4.29394695 | -3.65204868 |
| 1 | 4.00963492 | 4.83295302 | -2.13803144 |
| 1 | 2.48803114 | 5.57570333 | -2.70106455 |
| 1 | 0.32715012 | 2.51589443 | -2.26751183 |
| 1 | 0.32941015 | 4.23947174 | -2.70376676 |
| 1 | 1.17161766 | 3.06063985 | -3.73692637 |
| 1 | 4.04978810 | 2.40621047 | -1.39557866 |
| 1 | 2.57049755 | 1.41052216 | -1.45215207 |
| 1 | 3.32877258 | 1.97527723 | -2.96372920 |
| 1 | -0.81860558 | 0.14918112 | 3.37347731 |
| 8 | 1.73191940 | -0.50332750 | 3.43202473 |
| 8 | 2.66859016 | -2.50378126 | 3.35307916 |
| 6 | 2.38127944 | -1.30171982 | 2.77789016 |
| 6 | 2.89690561 | -1.00887750 | 1.38127921 |
| 1 | 2.56715775 | -1.79935569 | 0.68705972 |
| 1 | 2.40817167 | -0.07806081 | 1.06955427 |
| 6 | 4.43236294 | -0.86636173 | 1.30700803 |
| 1 | 4.78496483 | -0.26178744 | 2.15801306 |
| 1 | 4.67377573 | -0.29132988 | 0.40079031 |
| 6 | 5.17913423 | -2.20358124 | 1.25768335 |
| 1 | 4.89246337 | -2.73311726 | 0.33241878 |
| 1 | 6.26065079 | -2.01237642 | 1.17968899 |
| 6 | 4.91576255 | -3.12821000 | 2.45149910 |
| 1 | 5.28594457 | -2.67775171 | 3.38694066 |
| 1 | 5.48144544 | -4.06374687 | 2.31219706 |
| 6 | 3.45162025 | -3.50961546 | 2.65120261 |
| 1 | 2.96330472 | -3.76077473 | 1.69584003 |
| 1 | 3.37051427 | -4.38345783 | 3.30876768 |
| 31 | -0.87567850 | 1.22130013 | 0.48957786 |
| 1 | -6.69098493 | 2.93472652 | 2.30933648 |
| 1 | 2.36040421 | 6.55169931 | 1.73978791 |
| 8 | 0.60067046 | -8.51783686 | -2.34637471 |
| 6 | 1.02202591 | -9.85504486 | -1.98224697 |
| 1 | 2.11128900 | -9.89263938 | -1.85501538 |
| 6 | 0.48144215 | -6.18454066 | -1.91232137 |
| 6 | 0.08919464 | -5.23958257 | -0.77240843 |
| 6 | -0.11135888 | -3.79540620 | -1.24263322 |
| 6 | 0.90242131 | -7.55746135 | -1.42472993 |
| 6 | -0.53267775 | -2.85517754 | -0.10821368 |
| 6 | -0.59900046 | -1.39291673 | -0.54245256 |

| | | | |
|---|---|---|---|
| 1 | 1.34787998 | -5.77076546 | -2.45831357 |
| 1 | 0.86892286 | -5.27702055 | 0.00313578 |
| 1 | 0.82886665 | -3.43198981 | -1.69491883 |
| 1 | 0.17286389 | -2.94080075 | 0.73479091 |
| 1 | -0.32510586 | -6.29178295 | -2.65079307 |
| 1 | -0.83449607 | -5.60626386 | -0.29622975 |
| 1 | -0.86765033 | -3.76675994 | -2.04558272 |
| 1 | -1.51932166 | -3.15051345 | 0.28302322 |
| 1 | -1.31390606 | -1.29368957 | -1.38314851 |
| 1 | 0.39342149 | -1.07994780 | -0.92473082 |
| 8 | 1.45970436 | -7.79482017 | -0.36887789 |
| 8 | -1.00500657 | -0.58993465 | 0.56616925 |
| 1 | 0.70585922 | -10.49389310 | -2.81237435 |
| 1 | 0.54194604 | -10.16611840 | -1.04615669 |

Electronic energy = -3908.674939 a.u.
DFT-D3(BJ) dispersion correction = -0.132424 a.u.
Thermal free energy = 0.731967 a.u.
Gibbs free energy = -3908.075396 a.u.
Number of imaginary frequencies = 0.

**Int2 CL vdW CL isomer/conformer 6**

| Atomic N. | X | Y | Z |
|---|---|---|---|
| 7 | 0.36312958 | 2.28708041 | 0.34431999 |
| 8 | -2.45993881 | 1.61178001 | 1.12001462 |
| 8 | -1.27096551 | 0.55887573 | -1.50648567 |
| 6 | 1.47738641 | 2.05420784 | 1.30237987 |
| 6 | -2.23683905 | 2.51421456 | 2.11157251 |
| 6 | -3.09137518 | 2.55670670 | 3.25161357 |
| 6 | -2.79012367 | 3.50014576 | 4.24524180 |
| 6 | -1.71447616 | 4.38734700 | 4.14856517 |
| 6 | -0.91544958 | 4.36572427 | 3.01008511 |
| 6 | -1.17602932 | 3.45072667 | 1.98443289 |
| 6 | -0.40457174 | 3.53545912 | 0.69378314 |
| 6 | -4.31187483 | 1.62422926 | 3.38347757 |
| 6 | -1.16060682 | 1.62854396 | -2.33668969 |
| 6 | -2.06593280 | 1.77878283 | -3.42763476 |
| 6 | -1.89168049 | 2.89443990 | -4.25928516 |
| 6 | -0.88448859 | 3.84141032 | -4.05441290 |
| 6 | -0.00278197 | 3.67777058 | -2.99170490 |
| 6 | -0.12034178 | 2.57458476 | -2.13771792 |
| 6 | 0.91661282 | 2.36772836 | -1.06274632 |
| 6 | -3.19425003 | 0.76077853 | -3.68788460 |
| 6 | -5.09897342 | 1.88407811 | 4.68176215 |
| 6 | -3.86332991 | 0.14509089 | 3.40590438 |
| 6 | -5.28024130 | 1.86035008 | 2.19960912 |
| 6 | -4.02269424 | 1.12461514 | -4.93474704 |
| 6 | -4.16798347 | 0.72484533 | -2.48626432 |
| 6 | -2.59642804 | -0.64599445 | -3.92928332 |
| 1 | 2.18203250 | 2.89996263 | 1.25680813 |
| 1 | 1.07180606 | 1.96914810 | 2.31621644 |
| 1 | 0.30837053 | 4.37590510 | 0.73087168 |
| 1 | -1.09058145 | 3.71055405 | -0.14833072 |

| | | | |
|---|---|---|---|
| 1 | -3.41614719 | 3.54732257 | 5.13457515 |
| 1 | -0.09200598 | 5.07461356 | 2.89662004 |
| 1 | 1.47873311 | 1.43408784 | -1.21451655 |
| 1 | 1.64665066 | 3.19310428 | -1.08853076 |
| 1 | -2.57054632 | 3.03927277 | -5.09780025 |
| 1 | 0.80009162 | 4.39877275 | -2.82217047 |
| 1 | -5.96400703 | 1.20639929 | 4.71602071 |
| 1 | -4.49342549 | 1.69278594 | 5.57970668 |
| 1 | -5.48302858 | 2.91306731 | 4.73344775 |
| 1 | -3.36396182 | -0.13089032 | 2.47126011 |
| 1 | -3.17209965 | -0.04155944 | 4.24080690 |
| 1 | -4.73916394 | -0.50885302 | 3.53609755 |
| 1 | -5.64691734 | 2.89715945 | 2.20122493 |
| 1 | -4.79358362 | 1.66494810 | 1.23751510 |
| 1 | -6.15145413 | 1.19380220 | 2.29213643 |
| 1 | -4.80315700 | 0.36369752 | -5.07855941 |
| 1 | -3.40947300 | 1.14640459 | -5.84731679 |
| 1 | -4.52541131 | 2.09689047 | -4.82906529 |
| 1 | -3.66198119 | 0.42572599 | -1.56243501 |
| 1 | -4.62185547 | 1.71330366 | -2.32496903 |
| 1 | -4.97901699 | 0.00775698 | -2.68575229 |
| 1 | -1.94305803 | -0.64236740 | -4.81418355 |
| 1 | -2.01154144 | -0.98674551 | -3.06818140 |
| 1 | -3.40715185 | -1.36825082 | -4.11116281 |
| 1 | 2.00127094 | 1.13075819 | 1.03422604 |
| 8 | 3.75960953 | 0.42000722 | -1.01155713 |
| 8 | 4.13962796 | -0.61750021 | 0.90500752 |
| 6 | 4.38460516 | -0.44823036 | -0.42592176 |
| 6 | 5.42728175 | -1.31997224 | -1.09925747 |
| 1 | 5.15304302 | -2.38168387 | -0.98098488 |
| 1 | 5.36532897 | -1.07331490 | -2.16568247 |
| 6 | 6.86430378 | -1.08972224 | -0.58195290 |
| 1 | 7.04843134 | -0.00836358 | -0.47330205 |
| 1 | 7.56033383 | -1.44276107 | -1.35776402 |
| 6 | 7.18165049 | -1.81890385 | 0.72789225 |
| 1 | 7.08993377 | -2.90466739 | 0.55609579 |
| 1 | 8.23257104 | -1.63306735 | 0.99987669 |
| 6 | 6.27892086 | -1.43373008 | 1.90483833 |
| 1 | 6.41981230 | -0.37524060 | 2.17940291 |
| 1 | 6.57019502 | -2.02504974 | 2.78839993 |
| 6 | 4.79282011 | -1.67960009 | 1.66360937 |
| 1 | 4.61114319 | -2.64661573 | 1.17023833 |
| 1 | 4.24425510 | -1.66835467 | 2.61357854 |
| 31 | -0.98957712 | 0.77594139 | 0.32092797 |
| 1 | -1.51680125 | 5.09684190 | 4.95214588 |
| 1 | -0.79342882 | 4.69664532 | -4.72409110 |
| 8 | 2.79945157 | -6.27846524 | -1.14212003 |
| 6 | 3.82227726 | -7.15457044 | -0.60891980 |
| 1 | 4.78998961 | -6.94445227 | -1.08123072 |
| 6 | 1.88131313 | -4.11299854 | -1.47235697 |
| 6 | 0.86104836 | -3.80224463 | -0.35630830 |
| 6 | -0.26413113 | -2.89174938 | -0.85904650 |
| 6 | 3.02329209 | -4.94997197 | -0.94925429 |

| | | | |
|---|---|---|---|
| 6 | -1.35380775 | -2.60863619 | 0.18441422 |
| 6 | -0.87198918 | -1.86561461 | 1.43535652 |
| 1 | 2.30240411 | -3.17283347 | -1.85531158 |
| 1 | 1.38863081 | -3.32321850 | 0.48192989 |
| 1 | 0.16534599 | -1.93619131 | -1.19737458 |
| 1 | -1.81899016 | -3.55514913 | 0.51296336 |
| 1 | 1.38298498 | -4.63913777 | -2.29811077 |
| 1 | 0.43978393 | -4.74797532 | 0.02307017 |
| 1 | -0.73372009 | -3.35179530 | -1.74424834 |
| 1 | -2.15631596 | -2.02070720 | -0.29434157 |
| 1 | -0.14421140 | -2.48961680 | 1.98299243 |
| 1 | -1.73216666 | -1.71751756 | 2.11488087 |
| 8 | 4.01900503 | -4.51952183 | -0.38526320 |
| 8 | -0.21042243 | -0.63284417 | 1.16504223 |
| 1 | 3.48643839 | -8.16825246 | -0.84672944 |
| 1 | 3.91459085 | -7.01949228 | 0.47600203 |

Electronic energy = -3908.676874 a.u.
DFT-D3(BJ) dispersion correction = -0.133855 a.u.
Thermal free energy = 0.735701 a.u.
Gibbs free energy = -3908.075029 a.u.
Number of imaginary frequencies = 0.

**Int2_CL_vdW_CL isomer/conformer 7**

| Atomic N. | X | Y | Z |
|---|---|---|---|
| 7 | -0.51224247 | 1.78551426 | 1.34963680 |
| 8 | -2.47210053 | 1.73401842 | -0.92537944 |
| 8 | 0.53231817 | 1.39079858 | -1.45036902 |
| 6 | -0.71782786 | 0.91004983 | 2.53513534 |
| 6 | -3.35710225 | 2.11029660 | 0.03524359 |
| 6 | -4.75899780 | 1.98327103 | -0.18794876 |
| 6 | -5.61321247 | 2.37579574 | 0.85317432 |
| 6 | -5.14540807 | 2.88696500 | 2.06688923 |
| 6 | -3.77638617 | 3.04531168 | 2.25559208 |
| 6 | -2.87976720 | 2.67813883 | 1.24666267 |
| 6 | -1.41426200 | 2.99143113 | 1.39873266 |
| 6 | -5.32124163 | 1.45742784 | -1.52350682 |
| 6 | 1.10373916 | 2.60407128 | -1.23306926 |
| 6 | 1.54720320 | 3.38923399 | -2.33697634 |
| 6 | 2.14285420 | 4.62565934 | -2.04914845 |
| 6 | 2.30856433 | 5.10048465 | -0.74532074 |
| 6 | 1.88095630 | 4.31949633 | 0.32267134 |
| 6 | 1.29249957 | 3.06983870 | 0.09489016 |
| 6 | 0.94229935 | 2.19970108 | 1.27592038 |
| 6 | 1.38201886 | 2.90511590 | -3.79148473 |
| 6 | -6.86140660 | 1.44695379 | -1.53708426 |
| 6 | -4.84929758 | 0.00703887 | -1.77451639 |
| 6 | -4.85433043 | 2.36968166 | -2.68296928 |
| 6 | 1.96128702 | 3.91190205 | -4.80344762 |
| 6 | -0.11748271 | 2.72938343 | -4.12828412 |
| 6 | 2.13315970 | 1.56775023 | -3.99503392 |
| 1 | -0.46942582 | 1.47008141 | 3.45084225 |
| 1 | -1.76495710 | 0.59135528 | 2.57289430 |

| | | | |
|---|---|---|---|
| 1 | -1.23377323 | 3.51452522 | 2.35254234 |
| 1 | -1.07917564 | 3.64932296 | 0.58315759 |
| 1 | -6.68889652 | 2.27844441 | 0.71780315 |
| 1 | -3.39024426 | 3.47595840 | 3.18231781 |
| 1 | 1.51806546 | 1.26206235 | 1.27396322 |
| 1 | 1.18779827 | 2.72878107 | 2.21111473 |
| 1 | 2.48703217 | 5.25039627 | -2.87134403 |
| 1 | 2.01479346 | 4.66538356 | 1.35008109 |
| 1 | -7.20701357 | 1.07937886 | -2.51385368 |
| 1 | -7.27882837 | 0.78196548 | -0.76700616 |
| 1 | -7.28285933 | 2.45243955 | -1.39453001 |
| 1 | -3.75819388 | -0.04655624 | -1.84957107 |
| 1 | -5.17703477 | -0.65697930 | -0.96139936 |
| 1 | -5.27972817 | -0.36844077 | -2.71542548 |
| 1 | -5.22576262 | 3.39502464 | -2.54117114 |
| 1 | -3.76144658 | 2.40243066 | -2.75336463 |
| 1 | -5.25490393 | 1.99425285 | -3.63710145 |
| 1 | 1.82640926 | 3.51376942 | -5.81931727 |
| 1 | 3.03795554 | 4.07716731 | -4.65283244 |
| 1 | 1.44987561 | 4.88434516 | -4.75986961 |
| 1 | -0.59649110 | 1.99141164 | -3.47645234 |
| 1 | -0.65346609 | 3.68327136 | -4.01961648 |
| 1 | -0.22809963 | 2.39526564 | -5.17127588 |
| 1 | 3.20935212 | 1.69666660 | -3.80813680 |
| 1 | 1.75585224 | 0.78902042 | -3.32368493 |
| 1 | 2.00938718 | 1.22599676 | -5.03413800 |
| 1 | -0.06620368 | 0.03345201 | 2.45769167 |
| 8 | 2.66707023 | -0.38785843 | 2.78873543 |
| 8 | 1.40485724 | -2.07593312 | 3.46090108 |
| 6 | 2.63057507 | -1.52639877 | 3.22471433 |
| 6 | 3.87400386 | -2.33772743 | 3.53606990 |
| 1 | 3.85553470 | -3.27579995 | 2.95661055 |
| 1 | 4.71240661 | -1.73359338 | 3.17009501 |
| 6 | 4.05798535 | -2.65002935 | 5.03731256 |
| 1 | 3.83405883 | -1.74901718 | 5.63146083 |
| 1 | 5.12263934 | -2.87138836 | 5.20587666 |
| 6 | 3.22683412 | -3.83695117 | 5.53598719 |
| 1 | 3.54854320 | -4.74279710 | 4.99484698 |
| 1 | 3.44997825 | -4.01321735 | 6.59993033 |
| 6 | 1.71365780 | -3.67329016 | 5.35694614 |
| 1 | 1.33281871 | -2.83959983 | 5.96971379 |
| 1 | 1.20899899 | -4.58264050 | 5.72236569 |
| 6 | 1.27085866 | -3.45689716 | 3.91311694 |
| 1 | 1.80067583 | -4.12274214 | 3.21510286 |
| 1 | 0.19376177 | -3.63902244 | 3.81268335 |
| 31 | -0.90923460 | 0.84809062 | -0.40493535 |
| 1 | -5.84996156 | 3.17052186 | 2.84876812 |
| 1 | 2.77088303 | 6.07218014 | -0.57159261 |
| 8 | 3.49911427 | -5.96438400 | -0.72295406 |
| 6 | 3.69623703 | -7.23252993 | -0.05205773 |
| 1 | 4.54923491 | -7.17275744 | 0.63547805 |
| 6 | 2.95921202 | -3.65244633 | -0.68475598 |
| 6 | 1.45849117 | -3.49510487 | -1.01018602 |

| | | | |
|---|---|---|---|
| 6 | 1.17752102 | -2.18259653 | -1.74956153 |
| 6 | 3.21816605 | -4.91639120 | 0.09911356 |
| 6 | -0.28777463 | -1.99969459 | -2.16850370 |
| 6 | -1.28760551 | -1.93139039 | -1.00869981 |
| 1 | 3.29379559 | -2.80281566 | -0.07367553 |
| 1 | 0.88682616 | -3.52898626 | -0.07071453 |
| 1 | 1.47690521 | -1.33581654 | -1.11309799 |
| 1 | -0.59803971 | -2.82656032 | -2.83197572 |
| 1 | 3.54187429 | -3.66900261 | -1.61620037 |
| 1 | 1.12879195 | -4.35079626 | -1.62219563 |
| 1 | 1.81112316 | -2.13247764 | -2.65051110 |
| 1 | -0.37031884 | -1.07593790 | -2.76719541 |
| 1 | -1.29449997 | -2.89191103 | -0.46447702 |
| 1 | -2.30463562 | -1.79842719 | -1.42320878 |
| 8 | 3.15382499 | -5.02560174 | 1.31516615 |
| 8 | -0.99141677 | -0.93041822 | -0.03857489 |
| 1 | 3.89264052 | -7.95405277 | -0.85068854 |
| 1 | 2.79773619 | -7.51131262 | 0.51244004 |

Electronic energy = -3908.676868 a.u.
DFT-D3(BJ) dispersion correction = -0.133856 a.u.
Thermal free energy = 0.735763 a.u.
Gibbs free energy = -3908.074961 a.u.
Number of imaginary frequencies = 0.

**Int2 CL vdW CL isomer/conformer 8**

| Atomic N. | X | Y | Z |
|---|---|---|---|
| 7 | -0.54943607 | 3.54394123 | 0.50949196 |
| 8 | -0.86709807 | 1.11282361 | 2.22182326 |
| 8 | -1.86426441 | 1.14114058 | -0.73324247 |
| 6 | 0.79428115 | 4.16907515 | 0.42491205 |
| 6 | -0.43844433 | 1.95176398 | 3.20327609 |
| 6 | 0.08185008 | 1.42356561 | 4.41961379 |
| 6 | 0.49782607 | 2.34473825 | 5.39218467 |
| 6 | 0.41807514 | 3.72828136 | 5.21137042 |
| 6 | -0.11025104 | 4.23047976 | 4.02708666 |
| 6 | -0.55171610 | 3.35572410 | 3.02814057 |
| 6 | -1.23492249 | 3.90378955 | 1.80407363 |
| 6 | 0.18868951 | -0.09471708 | 4.66261790 |
| 6 | -2.94676185 | 1.96045657 | -0.78128304 |
| 6 | -4.25302042 | 1.41138381 | -0.93160371 |
| 6 | -5.32530483 | 2.31329984 | -0.98546948 |
| 6 | -5.15748036 | 3.69835160 | -0.89945356 |
| 6 | -3.87662706 | 4.22172473 | -0.76696435 |
| 6 | -2.76869157 | 3.36710294 | -0.71903910 |
| 6 | -1.38369180 | 3.95887560 | -0.68038599 |
| 6 | -4.48437687 | -0.10913548 | -1.03505822 |
| 6 | 0.73674276 | -0.41678338 | 6.06567545 |
| 6 | 1.15570177 | -0.72678717 | 3.63431959 |
| 6 | -1.20801713 | -0.75180463 | 4.55353897 |
| 6 | -5.97493703 | -0.45382772 | -1.21472193 |
| 6 | -4.00467429 | -0.81279368 | 0.25648836 |
| 6 | -3.72996686 | -0.67568921 | -2.26124915 |

| | | | |
|---|---|---|---|
| 1 | 0.69853205 | 5.26688311 | 0.41938725 |
| 1 | 1.39583612 | 3.86177420 | 1.28687096 |
| 1 | -1.30193517 | 5.00305939 | 1.86446578 |
| 1 | -2.25726585 | 3.50711525 | 1.71992001 |
| 1 | 0.90695902 | 1.97249328 | 6.32955657 |
| 1 | -0.20377642 | 5.30806907 | 3.87426080 |
| 1 | -0.80591767 | 3.65216360 | -1.56718394 |
| 1 | -1.44918761 | 5.05961492 | -0.68486939 |
| 1 | -6.33619871 | 1.92468546 | -1.09325795 |
| 1 | -3.72218068 | 5.30162634 | -0.71242424 |
| 1 | 0.78053832 | -1.50826665 | 6.18925762 |
| 1 | 1.75447416 | -0.02798014 | 6.21408565 |
| 1 | 0.09179247 | -0.01950018 | 6.86270660 |
| 1 | 0.81811242 | -0.56324583 | 2.60535492 |
| 1 | 2.16442212 | -0.30133763 | 3.73569412 |
| 1 | 1.22756390 | -1.81118199 | 3.81246990 |
| 1 | -1.89069952 | -0.33668709 | 5.30922686 |
| 1 | -1.65116745 | -0.59794236 | 3.56377577 |
| 1 | -1.12333024 | -1.83416026 | 4.73561971 |
| 1 | -6.08142204 | -1.54498922 | -1.29472048 |
| 1 | -6.39404275 | -0.01356198 | -2.13086981 |
| 1 | -6.58307896 | -0.12523817 | -0.35960150 |
| 1 | -2.93360159 | -0.66214454 | 0.42824147 |
| 1 | -4.55029457 | -0.43366659 | 1.13246494 |
| 1 | -4.19585522 | -1.89435181 | 0.18226247 |
| 1 | -4.10718550 | -0.22489204 | -3.19059480 |
| 1 | -2.65343508 | -0.48461311 | -2.19627391 |
| 1 | -3.89035193 | -1.76291145 | -2.32636235 |
| 1 | 1.29340012 | 3.84049120 | -0.49414747 |
| 31 | -0.47042243 | 1.49145053 | 0.44798310 |
| 1 | 0.76097970 | 4.40279603 | 5.99575623 |
| 1 | -6.02429368 | 4.35765511 | -0.93973137 |
| 8 | 6.41822429 | -2.30597744 | -6.13872242 |
| 6 | 7.73979704 | -2.81782189 | -6.42809396 |
| 1 | 8.48745182 | -2.01731042 | -6.35779347 |
| 6 | 4.87356833 | -1.23777684 | -4.69068493 |
| 6 | 4.56712545 | -0.85355883 | -3.24350593 |
| 6 | 3.17509697 | -0.23126394 | -3.09224993 |
| 6 | 6.27864031 | -1.76972407 | -4.88846017 |
| 6 | 2.82233151 | 0.06986805 | -1.63121814 |
| 6 | 1.43190807 | 0.67617997 | -1.47366084 |
| 1 | 4.76005288 | -0.36720693 | -5.35994983 |
| 1 | 5.33777470 | -0.15821104 | -2.87753623 |
| 1 | 3.12132392 | 0.69628175 | -3.69025645 |
| 1 | 3.56414290 | 0.75591547 | -1.19047149 |
| 1 | 4.16118632 | -1.99082207 | -5.06051099 |
| 1 | 4.63627542 | -1.75132187 | -2.61055516 |
| 1 | 2.42451765 | -0.92110027 | -3.51222876 |
| 1 | 2.86614433 | -0.86094512 | -1.04453809 |
| 1 | 0.68514022 | 0.03479391 | -1.97784360 |
| 1 | 1.40085724 | 1.65875713 | -1.98795322 |
| 8 | 7.18369778 | -1.72914065 | -4.07635277 |
| 8 | 1.12796064 | 0.81507510 | -0.07646162 |

| | | | |
|---|---|---|---|
| 1 | 7.68300146 | -3.20634783 | -7.44966208 |
| 1 | 8.00563606 | -3.61592411 | -5.72341743 |
| 8 | 1.39189381 | -3.49414436 | -2.95330992 |
| 8 | 1.85574277 | -3.51572358 | -0.78623830 |
| 6 | 0.97248886 | -3.36534241 | -1.81890247 |
| 6 | -0.48340885 | -3.06419124 | -1.50170724 |
| 1 | -0.55471111 | -2.13072928 | -0.91936970 |
| 1 | -0.95774602 | -2.88078852 | -2.47262296 |
| 6 | -1.21566719 | -4.19739162 | -0.75292067 |
| 1 | -0.94827619 | -5.16592487 | -1.20583570 |
| 1 | -2.29590986 | -4.06780955 | -0.91855794 |
| 6 | -0.95101012 | -4.22340215 | 0.75600458 |
| 1 | -1.32559438 | -3.28317792 | 1.19619650 |
| 1 | -1.53972010 | -5.03436080 | 1.21256245 |
| 6 | 0.52284767 | -4.39039078 | 1.14053295 |
| 1 | 0.90661415 | -5.37022306 | 0.81221828 |
| 1 | 0.61034005 | -4.37395143 | 2.23917615 |
| 6 | 1.44547699 | -3.30481162 | 0.59467048 |
| 1 | 1.00450022 | -2.30129059 | 0.69925545 |
| 1 | 2.39983279 | -3.30695068 | 1.13550973 |

Electronic energy = -3908.672023 a.u.
DFT-D3(BJ) dispersion correction = -0.134102 a.u.
Thermal free energy = 0.732600 a.u.
Gibbs free energy = -3908.073525 a.u.
Number of imaginary frequencies = 0.

**Int2 CL vdW CL isomer/conformer 9**

| Atomic N. | X | Y | Z |
|---|---|---|---|
| 7 | -1.64057558 | 2.10802545 | 1.86397044 |
| 8 | -2.27013705 | 1.33907715 | -0.96273910 |
| 8 | 0.47468164 | 2.53769707 | -0.24620591 |
| 6 | -1.88059898 | 1.13714464 | 2.96592166 |
| 6 | -3.52086512 | 1.15605332 | -0.46502291 |
| 6 | -4.47392784 | 0.37128991 | -1.17715871 |
| 6 | -5.74466679 | 0.22604434 | -0.60120281 |
| 6 | -6.09917568 | 0.81587741 | 0.61535600 |
| 6 | -5.16848395 | 1.60155892 | 1.28720008 |
| 6 | -3.88988150 | 1.78853498 | 0.75170033 |
| 6 | -2.93168698 | 2.74375065 | 1.41107908 |
| 6 | -4.13071747 | -0.28928686 | -2.52675386 |
| 6 | 0.28137372 | 3.84710531 | 0.05585051 |
| 6 | 0.71050266 | 4.85613191 | -0.85471083 |
| 6 | 0.49299012 | 6.19093109 | -0.48470044 |
| 6 | -0.11786965 | 6.55292309 | 0.71911674 |
| 6 | -0.52032754 | 5.55860577 | 1.60371465 |
| 6 | -0.31473562 | 4.20905136 | 1.29276727 |
| 6 | -0.65097928 | 3.16063667 | 2.32361477 |
| 6 | 1.38577946 | 4.49988446 | -2.19404538 |
| 6 | -5.33371616 | -1.04417529 | -3.12285803 |
| 6 | -2.98969228 | -1.31614840 | -2.33971729 |
| 6 | -3.70871907 | 0.78924937 | -3.55283648 |
| 6 | 1.79117716 | 5.75711418 | -2.98663300 |

| | | | |
|---|---|---|---|
| 6 | 0.41221686 | 3.69127974 | -3.08369563 |
| 6 | 2.67501920 | 3.68272296 | -1.93944187 |
| 1 | -2.34356481 | 1.65902025 | 3.81871043 |
| 1 | -2.55393986 | 0.34733558 | 2.61610998 |
| 1 | -3.40394025 | 3.21492829 | 2.28891640 |
| 1 | -2.64292264 | 3.54234281 | 0.71186287 |
| 1 | -6.49161561 | -0.37489114 | -1.11685470 |
| 1 | -5.43239845 | 2.09432709 | 2.22576184 |
| 1 | 0.24562330 | 2.60444930 | 2.63694556 |
| 1 | -1.07308411 | 3.64577705 | 3.21887268 |
| 1 | 0.80457009 | 6.98415862 | -1.16183495 |
| 1 | -0.98517937 | 5.82081756 | 2.55678529 |
| 1 | -5.03813242 | -1.48166714 | -4.08717124 |
| 1 | -5.66951462 | -1.86757133 | -2.47588506 |
| 1 | -6.18796302 | -0.37759589 | -3.30985658 |
| 1 | -2.08304722 | -0.83767837 | -1.95465399 |
| 1 | -3.28998496 | -2.10736424 | -1.63745518 |
| 1 | -2.75113929 | -1.78910819 | -3.30473125 |
| 1 | -4.52968596 | 1.50062117 | -3.72441332 |
| 1 | -2.83055501 | 1.34813048 | -3.21173409 |
| 1 | -3.46857807 | 0.31278439 | -4.51567446 |
| 1 | 2.27736463 | 5.44710399 | -3.92264999 |
| 1 | 2.50674544 | 6.38226644 | -2.43313617 |
| 1 | 0.92290004 | 6.37590379 | -3.25555562 |
| 1 | 0.11195047 | 2.75212856 | -2.60729450 |
| 1 | -0.49470176 | 4.27539946 | -3.29648760 |
| 1 | 0.89561433 | 3.45399354 | -4.04375020 |
| 1 | 3.39253805 | 4.26826209 | -1.34609313 |
| 1 | 2.46081213 | 2.75073326 | -1.40528089 |
| 1 | 3.15331854 | 3.43532239 | -2.89957448 |
| 1 | -0.92570388 | 0.70473971 | 3.28361286 |
| 8 | 1.46373585 | 0.95544827 | 3.85054083 |
| 8 | 1.10861169 | -1.17003486 | 4.34369210 |
| 6 | 1.84769817 | -0.19593851 | 3.73947027 |
| 6 | 3.11078651 | -0.57898276 | 2.99147228 |
| 1 | 2.86849517 | -1.30899394 | 2.20197522 |
| 1 | 3.44373075 | 0.34014606 | 2.49616947 |
| 6 | 4.22839161 | -1.13960094 | 3.89668536 |
| 1 | 4.30474189 | -0.52295178 | 4.80669174 |
| 1 | 5.18418440 | -1.02160414 | 3.36470028 |
| 6 | 4.05128509 | -2.61596041 | 4.26671186 |
| 1 | 4.08995854 | -3.21859935 | 3.34275972 |
| 1 | 4.90548948 | -2.93991362 | 4.88116020 |
| 6 | 2.74788506 | -2.93777852 | 5.00577845 |
| 1 | 2.71778207 | -2.43615228 | 5.98682285 |
| 1 | 2.70887676 | -4.02028891 | 5.20861949 |
| 6 | 1.47899730 | -2.57375878 | 4.24084648 |
| 1 | 1.54645764 | -2.86097338 | 3.17953354 |
| 1 | 0.61150437 | -3.08727944 | 4.67279147 |
| 31 | -0.81385331 | 1.27864760 | 0.20918587 |
| 1 | -7.09843010 | 0.66713072 | 1.02451980 |
| 1 | -0.27265472 | 7.60471862 | 0.95949670 |
| 8 | 3.50921495 | -7.49141434 | -2.77719705 |

| | | | |
|---|---|---|---|
| 6 | 3.84058042 | -8.88188851 | -2.54671185 |
| 1 | 4.58307285 | -8.97191829 | -1.74371585 |
| 6 | 2.69640442 | -5.39431022 | -2.02803133 |
| 6 | 1.85551748 | -4.68201604 | -0.96873262 |
| 6 | 1.66560716 | -3.19218662 | -1.27261723 |
| 6 | 2.97424437 | -6.84821697 | -1.69803591 |
| 6 | 0.80436070 | -2.47694878 | -0.22647115 |
| 6 | 0.67581380 | -0.97829686 | -0.48560920 |
| 1 | 3.67444105 | -4.89689511 | -2.14693632 |
| 1 | 2.33398409 | -4.80699252 | 0.01459021 |
| 1 | 2.65456306 | -2.70447533 | -1.33373058 |
| 1 | 1.23691014 | -2.62531667 | 0.77758346 |
| 1 | 2.22111250 | -5.34777855 | -3.01989297 |
| 1 | 0.87336923 | -5.17438105 | -0.89166861 |
| 1 | 1.20420442 | -3.07474307 | -2.26817658 |
| 1 | -0.20512525 | -2.91722825 | -0.20002396 |
| 1 | 0.24266255 | -0.82916193 | -1.49471651 |
| 1 | 1.68226774 | -0.51484931 | -0.49876585 |
| 8 | 2.77292207 | -7.39025543 | -0.62747831 |
| 8 | -0.13549986 | -0.38178136 | 0.53074648 |
| 1 | 4.24953001 | -9.24291111 | -3.49523465 |
| 1 | 2.94339790 | -9.44906207 | -2.26885738 |

Electronic energy = -3908.674067 a.u.
DFT-D3(BJ) dispersion correction = -0.130771 a.u.
Thermal free energy = 0.731861 a.u.
Gibbs free energy = -3908.072977 a.u.
Number of imaginary frequencies = 0.

**Int2 CL vdW CL isomer/conformer 10**

| Atomic N. | X | Y | Z |
|---|---|---|---|
| 7 | 0.56207485 | 4.27230645 | 1.37060870 |
| 8 | 2.56735735 | 2.09158259 | 1.05089925 |
| 8 | -0.46985876 | 1.46881580 | 0.93619475 |
| 6 | 0.69577378 | 5.48528570 | 0.52427441 |
| 6 | 3.44414545 | 3.07328951 | 1.39297996 |
| 6 | 4.82957502 | 2.93316603 | 1.09275878 |
| 6 | 5.67852384 | 3.97885434 | 1.48458255 |
| 6 | 5.22025878 | 5.12273395 | 2.14429378 |
| 6 | 3.86769347 | 5.23851809 | 2.44692982 |
| 6 | 2.97842886 | 4.21926072 | 2.08926205 |
| 6 | 1.53788218 | 4.29600465 | 2.51963550 |
| 6 | 5.37814529 | 1.68799292 | 0.36875191 |
| 6 | -0.96236517 | 1.63266214 | 2.19061956 |
| 6 | -1.33495178 | 0.49511989 | 2.96385387 |
| 6 | -1.85242291 | 0.73164173 | 4.24526792 |
| 6 | -2.00765047 | 2.01558095 | 4.77589627 |
| 6 | -1.64965220 | 3.11725410 | 4.00746421 |
| 6 | -1.14033993 | 2.93952284 | 2.71539729 |
| 6 | -0.86195428 | 4.15035235 | 1.86212528 |
| 6 | -1.17063946 | -0.93802193 | 2.42102169 |
| 6 | 6.90716147 | 1.75193573 | 0.19240639 |
| 6 | 4.75624440 | 1.57727662 | -1.04326956 |

| | | | |
|---|---|---|---|
| 6 | 5.06275675 | 0.41443427 | 1.18910730 |
| 6 | -1.67632343 | -1.99405974 | 3.42213273 |
| 6 | 0.32397981 | -1.23617970 | 2.15501214 |
| 6 | -1.98594730 | -1.11397719 | 1.11811285 |
| 1 | 0.40221546 | 6.37837692 | 1.09921750 |
| 1 | 1.73693563 | 5.58843345 | 0.20003498 |
| 1 | 1.36191027 | 5.21398244 | 3.10507003 |
| 1 | 1.27553139 | 3.43540209 | 3.15230221 |
| 1 | 6.74172366 | 3.90525893 | 1.26330841 |
| 1 | 3.49157442 | 6.11371694 | 2.98168398 |
| 1 | -1.48462210 | 4.13626180 | 0.95294617 |
| 1 | -1.10806271 | 5.06593849 | 2.42513924 |
| 1 | -2.14142289 | -0.11722071 | 4.86223997 |
| 1 | -1.77523809 | 4.12956531 | 4.39808611 |
| 1 | 7.24570164 | 0.83779096 | -0.31566562 |
| 1 | 7.21878491 | 2.60679421 | -0.42509715 |
| 1 | 7.43287496 | 1.80810350 | 1.15659322 |
| 1 | 3.66594498 | 1.48410084 | -1.00172216 |
| 1 | 5.00362330 | 2.46226347 | -1.64726752 |
| 1 | 5.15975300 | 0.69191588 | -1.55806696 |
| 1 | 5.53942508 | 0.46418272 | 2.17902610 |
| 1 | 3.98462398 | 0.28084153 | 1.32839475 |
| 1 | 5.46003615 | -0.46988157 | 0.66789318 |
| 1 | -1.54654450 | -2.99303995 | 2.98169656 |
| 1 | -2.74468035 | -1.86881236 | 3.65086549 |
| 1 | -1.11346704 | -1.97598964 | 4.36649935 |
| 1 | 0.74803463 | -0.55631748 | 1.40862999 |
| 1 | 0.91011604 | -1.14159695 | 3.08036998 |
| 1 | 0.43694827 | -2.26686159 | 1.78586611 |
| 1 | -3.05841088 | -0.96005544 | 1.30834695 |
| 1 | -1.66697706 | -0.41025661 | 0.34222061 |
| 1 | -1.85197220 | -2.13643415 | 0.73360878 |
| 1 | 0.05802571 | 5.38044959 | -0.36062792 |
| 31 | 0.91703988 | 2.54680747 | 0.32521521 |
| 1 | 5.92019784 | 5.91051298 | 2.42256854 |
| 1 | -2.40812439 | 2.14724346 | 5.78088846 |
| 8 | -2.61658477 | -2.67016894 | -5.60412432 |
| 6 | -3.96076428 | -3.13877686 | -5.30642404 |
| 1 | -3.93782392 | -4.17445241 | -4.94829149 |
| 6 | -0.36921592 | -2.22776754 | -4.98438168 |
| 6 | 0.38679940 | -1.45697415 | -3.89593581 |
| 6 | -0.19393476 | -0.06460473 | -3.62319546 |
| 6 | -1.75704705 | -2.66961224 | -4.55501446 |
| 6 | 0.62166670 | 0.73255717 | -2.59873491 |
| 6 | 0.02970492 | 2.11465156 | -2.32009309 |
| 1 | 0.17888551 | -3.15072544 | -5.23933624 |
| 1 | 1.43923684 | -1.35919202 | -4.20650571 |
| 1 | -0.24010269 | 0.50102889 | -4.57022326 |
| 1 | 1.65638214 | 0.86097914 | -2.95610897 |
| 1 | -0.44754117 | -1.64444812 | -5.91255203 |
| 1 | 0.38033228 | -2.04938126 | -2.96843091 |
| 1 | -1.23285175 | -0.16638021 | -3.26737969 |
| 1 | 0.67743249 | 0.16632427 | -1.65196721 |

| | | | |
|---|---|---|---|
| 1 | -0.99515671 | 2.00056246 | -1.91793347 |
| 1 | -0.05214569 | 2.67507075 | -3.26778733 |
| 8 | -2.06583880 | -3.01137330 | -3.42403475 |
| 8 | 0.84312950 | 2.91133253 | -1.45208607 |
| 1 | -4.50347990 | -3.06826079 | -6.25334070 |
| 1 | -4.42829962 | -2.50435358 | -4.54501391 |
| 8 | -6.09629146 | -4.07849960 | -2.71879233 |
| 8 | -4.46295590 | -5.56015402 | -2.51069419 |
| 6 | -5.09912244 | -4.41618426 | -2.10954608 |
| 6 | -4.54313358 | -3.64450539 | -0.92577370 |
| 1 | -3.50881680 | -3.33586959 | -1.14665700 |
| 1 | -5.15126317 | -2.73436492 | -0.86629468 |
| 6 | -4.59973981 | -4.40875872 | 0.41348128 |
| 1 | -5.57625991 | -4.91118732 | 0.50810621 |
| 1 | -4.55495975 | -3.66848899 | 1.22649490 |
| 6 | -3.46381859 | -5.41809095 | 0.60932730 |
| 1 | -2.50484022 | -4.87164216 | 0.61870654 |
| 1 | -3.56231958 | -5.88720878 | 1.60097553 |
| 6 | -3.39157542 | -6.51021069 | -0.46286888 |
| 1 | -4.28939360 | -7.14922213 | -0.43359025 |
| 1 | -2.53386702 | -7.16853730 | -0.24765861 |
| 6 | -3.21995070 | -5.98566470 | -1.88593374 |
| 1 | -2.48517000 | -5.16758295 | -1.94014360 |
| 1 | -2.87869942 | -6.79269207 | -2.54682715 |

Electronic energy = -3908.674257 a.u.
DFT-D3(BJ) dispersion correction = -0.132658 a.u.
Thermal free energy = 0.735438 a.u.
Gibbs free energy = -3908.071477 a.u.
Number of imaginary frequencies = 0.

**Int2 CL vdW CL isomer/conformer 11**

| Atomic N. | X | Y | Z |
|---|---|---|---|
| 7 | 1.36847346 | 3.99515220 | 0.33153776 |
| 8 | 1.44602226 | 1.21382825 | -0.75093378 |
| 8 | 0.13428652 | 1.83785904 | 2.02436855 |
| 6 | 0.85540155 | 5.05654155 | -0.56976128 |
| 6 | 2.25486542 | 1.78495758 | -1.68449346 |
| 6 | 2.46582286 | 1.14092717 | -2.93766934 |
| 6 | 3.32562195 | 1.77614022 | -3.84605803 |
| 6 | 3.96400071 | 2.98845178 | -3.56961003 |
| 6 | 3.75778114 | 3.60051369 | -2.33826145 |
| 6 | 2.91978650 | 3.00370401 | -1.38956992 |
| 6 | 2.78492831 | 3.61798215 | -0.02262974 |
| 6 | 1.77791637 | -0.19116578 | -3.29245494 |
| 6 | 1.11343277 | 2.18526928 | 2.89755518 |
| 6 | 1.48181342 | 1.29492384 | 3.94788442 |
| 6 | 2.49472360 | 1.71654479 | 4.82096297 |
| 6 | 3.13744785 | 2.95187010 | 4.69878233 |
| 6 | 2.75622258 | 3.81866955 | 3.68133451 |
| 6 | 1.74186993 | 3.45325663 | 2.78818377 |
| 6 | 1.26848965 | 4.45367584 | 1.76622886 |
| 6 | 0.79717191 | -0.07451918 | 4.12416974 |

| | | | |
|---|---|---|---|
| 6 | 2.19748193 | -0.70406746 | -4.68349089 |
| 6 | 0.24206580 | -0.00609209 | -3.31714772 |
| 6 | 2.16117588 | -1.28704707 | -2.26931018 |
| 6 | 1.34237737 | -0.83622667 | 5.34719899 |
| 6 | 1.04714948 | -0.96137974 | 2.88189271 |
| 6 | -0.72319061 | 0.11521313 | 4.34085374 |
| 1 | 1.44455844 | 5.97880936 | -0.43886072 |
| 1 | 0.93221427 | 4.72204284 | -1.60965709 |
| 1 | 3.41286743 | 4.52173527 | 0.05349444 |
| 1 | 3.11064280 | 2.91397063 | 0.75713690 |
| 1 | 3.50230419 | 1.31164634 | -4.81432433 |
| 1 | 4.25990800 | 4.53933007 | -2.09333701 |
| 1 | 0.20490331 | 4.69517984 | 1.92504783 |
| 1 | 1.84438135 | 5.38882753 | 1.86787129 |
| 1 | 2.80329475 | 1.05547721 | 5.62864178 |
| 1 | 3.23340471 | 4.79576755 | 3.57777978 |
| 1 | 1.69318334 | -1.66134281 | -4.87493357 |
| 1 | 1.90834788 | -0.01050375 | -5.48655893 |
| 1 | 3.28084585 | -0.88184542 | -4.74908505 |
| 1 | -0.15137003 | 0.30325966 | -2.34275038 |
| 1 | -0.04546380 | 0.75010717 | -4.06213148 |
| 1 | -0.23446774 | -0.95733460 | -3.59692423 |
| 1 | 3.24828755 | -1.45621526 | -2.27364197 |
| 1 | 1.85486566 | -1.01536115 | -1.25321080 |
| 1 | 1.66840873 | -2.23054244 | -2.54787548 |
| 1 | 0.81307817 | -1.79577226 | 5.43378280 |
| 1 | 1.18105922 | -0.28424852 | 6.28443244 |
| 1 | 2.41518042 | -1.05892365 | 5.25370835 |
| 1 | 0.62611192 | -0.51871889 | 1.97318970 |
| 1 | 2.12431209 | -1.11806422 | 2.72552605 |
| 1 | 0.57871273 | -1.94587785 | 3.03297658 |
| 1 | -0.91368146 | 0.70764672 | 5.24771212 |
| 1 | -1.19094218 | 0.61979106 | 3.48875328 |
| 1 | -1.20448113 | -0.86591582 | 4.47121967 |
| 1 | -0.19686668 | 5.25572885 | -0.33604687 |
| 31 | 0.26317962 | 2.26159059 | 0.21724619 |
| 1 | 4.61876880 | 3.44358908 | -4.31272743 |
| 1 | 3.92508545 | 3.23036801 | 5.39867311 |
| 8 | -4.22619571 | -2.02766375 | -2.54836687 |
| 6 | -3.19937632 | -2.89200074 | -3.10925748 |
| 1 | -2.20580639 | -2.45889068 | -2.95171850 |
| 6 | -5.19493235 | -0.85256099 | -0.71840760 |
| 6 | -4.66383514 | 0.47717140 | -0.15207704 |
| 6 | -4.10399609 | 1.41352843 | -1.22984589 |
| 6 | -4.08247171 | -1.74894974 | -1.22789280 |
| 6 | -3.73373090 | 2.80947910 | -0.70790837 |
| 6 | -2.49451270 | 2.85284908 | 0.18577605 |
| 1 | -5.92227390 | -0.67023980 | -1.52098088 |
| 1 | -3.89846170 | 0.25210087 | 0.60592268 |
| 1 | -3.21587934 | 0.96359908 | -1.70054050 |
| 1 | -4.58175518 | 3.23355597 | -0.14133093 |
| 1 | -5.70286629 | -1.41137014 | 0.08283219 |
| 1 | -5.49379373 | 0.97897034 | 0.37243029 |

| | | | |
|---|---|---|---|
| 1 | -4.85668707 | 1.52458868 | -2.02797549 |
| 1 | -3.55303740 | 3.48221827 | -1.56158110 |
| 1 | -2.61409695 | 2.17157993 | 1.04782568 |
| 1 | -2.39464138 | 3.87671138 | 0.59931383 |
| 8 | -3.16832353 | -2.17525858 | -0.53960816 |
| 8 | -1.34261711 | 2.50274354 | -0.59144179 |
| 1 | -3.43038033 | -2.95665775 | -4.17623344 |
| 1 | -3.23650006 | -3.88348982 | -2.64391107 |
| 8 | -0.23408607 | -4.55227533 | -3.01739583 |
| 8 | -1.48814136 | -5.77315725 | -1.65855397 |
| 6 | -0.34978425 | -5.05941128 | -1.91757279 |
| 6 | 0.69017763 | -4.91770262 | -0.82068405 |
| 1 | 0.98620764 | -5.91168242 | -0.44575316 |
| 1 | 1.56634957 | -4.47249006 | -1.30579046 |
| 6 | 0.22357453 | -4.03303644 | 0.35622983 |
| 1 | -0.29563526 | -3.14557936 | -0.03690942 |
| 1 | 1.11971995 | -3.66522860 | 0.87761340 |
| 6 | -0.66991640 | -4.76423110 | 1.36286701 |
| 1 | -0.08771316 | -5.57859621 | 1.83012882 |
| 1 | -0.93882599 | -4.07048823 | 2.17430769 |
| 6 | -1.95281474 | -5.35162908 | 0.76453691 |
| 1 | -2.60157986 | -4.54840914 | 0.38106855 |
| 1 | -2.51775001 | -5.86365546 | 1.56101759 |
| 6 | -1.72392561 | -6.36738886 | -0.35090730 |
| 1 | -0.90329435 | -7.06263924 | -0.10779623 |
| 1 | -2.63016328 | -6.96359729 | -0.51611912 |

Electronic energy = -3908.672240 a.u.
DFT-D3(BJ) dispersion correction = -0.134669 a.u.
Thermal free energy = 0.736421 a.u.
Gibbs free energy = -3908.070489 a.u.
Number of imaginary frequencies = 0.

**Int2 CL vdW CL isomer/conformer 12**

| Atomic N. | X | Y | Z |
|---|---|---|---|
| 7 | -0.52525319 | 3.40273944 | 1.92549841 |
| 8 | -2.32216401 | 1.14119448 | 1.16759511 |
| 8 | -0.16082215 | 2.18630339 | -0.80506663 |
| 6 | 0.20877355 | 3.32244532 | 3.21323470 |
| 6 | -2.93868471 | 1.45777429 | 2.33757322 |
| 6 | -3.79023861 | 0.51228766 | 2.97749563 |
| 6 | -4.40205709 | 0.91000389 | 4.17544411 |
| 6 | -4.20828754 | 2.17175049 | 4.74492416 |
| 6 | -3.38730758 | 3.09120921 | 4.10074421 |
| 6 | -2.76161530 | 2.75044434 | 2.89653684 |
| 6 | -1.96783255 | 3.78498613 | 2.14421396 |
| 6 | -4.03055894 | -0.89014260 | 2.38460834 |
| 6 | -0.56778829 | 3.41052771 | -1.22674587 |
| 6 | -1.05979147 | 3.58087206 | -2.55328836 |
| 6 | -1.45224180 | 4.87273378 | -2.93143211 |
| 6 | -1.37887028 | 5.97081542 | -2.06954540 |
| 6 | -0.88540301 | 5.79355506 | -0.78214594 |
| 6 | -0.46742075 | 4.52711726 | -0.35614214 |

| | | | |
|---|---|---|---|
| 6 | 0.16560010 | 4.37997708 | 1.00332110 |
| 6 | -1.16082206 | 2.39673195 | -3.53534653 |
| 6 | -5.02058046 | -1.71079508 | 3.23273109 |
| 6 | -2.70181604 | -1.68074374 | 2.33454399 |
| 6 | -4.63236546 | -0.77104110 | 0.96396401 |
| 6 | -1.68646068 | 2.83677726 | -4.91480793 |
| 6 | -2.14277796 | 1.33450020 | -2.98701579 |
| 6 | 0.23512610 | 1.76609161 | -3.75498157 |
| 1 | 0.24068280 | 4.31515498 | 3.69073380 |
| 1 | -0.29733523 | 2.61517263 | 3.87875608 |
| 1 | -1.99233225 | 4.74926545 | 2.67914980 |
| 1 | -2.38927483 | 3.94322810 | 1.14073702 |
| 1 | -5.05307304 | 0.20794197 | 4.69315074 |
| 1 | -3.23870008 | 4.09021538 | 4.51701614 |
| 1 | 1.20112957 | 4.01459511 | 0.91029956 |
| 1 | 0.19846995 | 5.36077664 | 1.50637602 |
| 1 | -1.83767721 | 5.03413764 | -3.93642733 |
| 1 | -0.80624098 | 6.64111960 | -0.09770658 |
| 1 | -5.16567409 | -2.69295442 | 2.76071194 |
| 1 | -4.64739436 | -1.88756063 | 4.25179301 |
| 1 | -6.00611329 | -1.22779268 | 3.30092649 |
| 1 | -1.94810370 | -1.17749017 | 1.71958618 |
| 1 | -2.28698182 | -1.80903159 | 3.34455914 |
| 1 | -2.88249539 | -2.68248038 | 1.91478198 |
| 1 | -5.59918292 | -0.24775051 | 0.99573040 |
| 1 | -3.96516209 | -0.22641769 | 0.28718842 |
| 1 | -4.80758093 | -1.77637471 | 0.55099010 |
| 1 | -1.72771353 | 1.96056334 | -5.57741533 |
| 1 | -1.02928220 | 3.57928583 | -5.38988776 |
| 1 | -2.70181774 | 3.25486685 | -4.85837570 |
| 1 | -1.81181639 | 0.93551629 | -2.02216746 |
| 1 | -3.14676071 | 1.76275895 | -2.85558807 |
| 1 | -2.22303329 | 0.49959708 | -3.70033434 |
| 1 | 0.93040122 | 2.50284915 | -4.18265749 |
| 1 | 0.66115613 | 1.39139552 | -2.81797401 |
| 1 | 0.15551154 | 0.92905265 | -4.46600617 |
| 1 | 1.23024664 | 2.96958963 | 3.02957467 |
| 31 | -0.54150761 | 1.60906291 | 0.92197305 |
| 1 | -4.70226087 | 2.43023177 | 5.68144872 |
| 1 | -1.70306212 | 6.95456207 | -2.40841046 |
| 8 | 7.82016630 | -4.22839181 | 0.37625600 |
| 6 | 8.51511812 | -5.46141994 | 0.67071243 |
| 1 | 8.71214506 | -5.54612811 | 1.74717742 |
| 6 | 5.94546317 | -2.79435820 | 0.60474779 |
| 6 | 4.63553718 | -2.52797585 | 1.34506885 |
| 6 | 3.99904615 | -1.19949938 | 0.92404003 |
| 6 | 6.60469594 | -4.10067692 | 0.99306711 |
| 6 | 2.61865130 | -0.97788425 | 1.55112401 |
| 6 | 1.95670511 | 0.30363528 | 1.05788904 |
| 1 | 6.67419636 | -1.98385295 | 0.76841063 |
| 1 | 4.81216407 | -2.54115426 | 2.43218427 |
| 1 | 4.67302428 | -0.36603045 | 1.19164185 |
| 1 | 2.68902888 | -0.95108116 | 2.65097497 |

| | | | |
|---|---|---|---|
| 1 | 5.76912582 | -2.82054908 | -0.48339218 |
| 1 | 3.93565272 | -3.35129135 | 1.13859230 |
| 1 | 3.89968246 | -1.18845998 | -0.17351563 |
| 1 | 1.96247442 | -1.82375604 | 1.29367876 |
| 1 | 1.95416774 | 0.31123930 | -0.04700622 |
| 1 | 2.55387830 | 1.17907786 | 1.38553523 |
| 8 | 6.14752099 | -4.94572600 | 1.73981803 |
| 8 | 0.61778696 | 0.38295500 | 1.57824775 |
| 1 | 9.45276808 | -5.40770456 | 0.10860222 |
| 1 | 7.91961458 | -6.32569167 | 0.34960609 |
| 8 | 3.40624513 | -2.93629661 | -2.37798681 |
| 8 | 1.48403723 | -2.36250865 | -1.43810495 |
| 6 | 2.19711111 | -2.86431185 | -2.48995068 |
| 6 | 1.44837207 | -3.32503872 | -3.72964218 |
| 1 | 0.83468338 | -2.49963182 | -4.12742410 |
| 1 | 2.22482241 | -3.54694283 | -4.47073264 |
| 6 | 0.56117086 | -4.56669171 | -3.49611155 |
| 1 | 1.11723378 | -5.30062339 | -2.89085777 |
| 1 | 0.38359160 | -5.04405056 | -4.47157222 |
| 6 | -0.79204882 | -4.25721539 | -2.84718731 |
| 1 | -1.37090641 | -3.61037674 | -3.52934821 |
| 1 | -1.36643274 | -5.19137651 | -2.74798213 |
| 6 | -0.70424422 | -3.57083244 | -1.47987188 |
| 1 | -0.22342646 | -4.22958448 | -0.73855462 |
| 1 | -1.72368869 | -3.37780364 | -1.10819866 |
| 6 | 0.03463094 | -2.23640664 | -1.48633602 |
| 1 | -0.25466612 | -1.61508648 | -2.34918843 |
| 1 | -0.19046007 | -1.67169928 | -0.57285374 |

Electronic energy = -3908.672130 a.u.
DFT-D3(BJ) dispersion correction = -0.131974 a.u.
Thermal free energy = 0.733866 a.u.
Gibbs free energy = -3908.070237 a.u.
Number of imaginary frequencies = 0.

**Int2 CL vdW CL isomer/conformer 13**

| Atomic N. | X | Y | Z |
|---|---|---|---|
| 7 | 1.44011260 | 4.30475047 | 1.17140790 |
| 8 | -0.02803397 | 2.67837693 | -0.85209509 |
| 8 | 1.87174824 | 1.32718580 | 1.23611317 |
| 6 | 0.64977069 | 5.27103520 | 1.97469064 |
| 6 | -0.32350444 | 3.89792640 | -1.37526482 |
| 6 | -1.41298666 | 4.04148851 | -2.28250698 |
| 6 | -1.66092247 | 5.32536674 | -2.78997583 |
| 6 | -0.89118857 | 6.43981738 | -2.44575905 |
| 6 | 0.18276349 | 6.28190838 | -1.57653019 |
| 6 | 0.48205919 | 5.02057794 | -1.05000779 |
| 6 | 1.71736778 | 4.83800493 | -0.21010141 |
| 6 | -2.28683964 | 2.84079243 | -2.69515472 |
| 6 | 3.14919269 | 1.66576850 | 0.92448994 |
| 6 | 4.03071513 | 0.69143521 | 0.37282566 |
| 6 | 5.33823138 | 1.10533910 | 0.08092379 |
| 6 | 5.79246047 | 2.40758605 | 0.31093851 |

| | | | |
|---|---|---|---|
| 6 | 4.92740834 | 3.34253023 | 0.86764591 |
| 6 | 3.61340350 | 2.98041323 | 1.18872867 |
| 6 | 2.72598220 | 3.97348139 | 1.89337325 |
| 6 | 3.57292760 | -0.75687504 | 0.11328619 |
| 6 | -3.36837255 | 3.23892517 | -3.71724853 |
| 6 | -3.01285043 | 2.26413005 | -1.45760631 |
| 6 | -1.41150951 | 1.74865173 | -3.35515450 |
| 6 | 4.70975858 | -1.62714507 | -0.45524597 |
| 6 | 2.42207239 | -0.77872936 | -0.92080653 |
| 6 | 3.11055875 | -1.41413978 | 1.43597647 |
| 1 | 1.23962275 | 6.18519633 | 2.15057028 |
| 1 | -0.26730979 | 5.52974210 | 1.43445856 |
| 1 | 2.25539478 | 5.79577075 | -0.11047558 |
| 1 | 2.40139141 | 4.11430556 | -0.67736713 |
| 1 | -2.49132516 | 5.46822447 | -3.47890940 |
| 1 | 0.81293769 | 7.13433101 | -1.31265305 |
| 1 | 2.41759574 | 3.58833523 | 2.87876808 |
| 1 | 3.27707153 | 4.91464356 | 2.05673537 |
| 1 | 6.03387746 | 0.38687772 | -0.34883306 |
| 1 | 5.26741024 | 4.36032908 | 1.07184773 |
| 1 | -3.95133047 | 2.34637182 | -3.98548931 |
| 1 | -4.07088640 | 3.98080060 | -3.31095834 |
| 1 | -2.93376472 | 3.64085553 | -4.64389242 |
| 1 | -2.30486806 | 1.92107083 | -0.69555147 |
| 1 | -3.66745062 | 3.02124292 | -1.00224532 |
| 1 | -3.64262790 | 1.41264396 | -1.75882402 |
| 1 | -0.91840423 | 2.14028942 | -4.25674311 |
| 1 | -0.63978688 | 1.38092278 | -2.67011646 |
| 1 | -2.04528840 | 0.90180608 | -3.66115849 |
| 1 | 4.33152832 | -2.64777123 | -0.60721893 |
| 1 | 5.56568244 | -1.69025436 | 0.23236721 |
| 1 | 5.06924877 | -1.25917135 | -1.42738305 |
| 1 | 1.55407539 | -0.20852541 | -0.57339035 |
| 1 | 2.75386957 | -0.35487526 | -1.87992640 |
| 1 | 2.10407582 | -1.81759730 | -1.09293078 |
| 1 | 3.94593597 | -1.47323548 | 2.14932124 |
| 1 | 2.29452609 | -0.85217895 | 1.90233844 |
| 1 | 2.75285618 | -2.43506122 | 1.23848027 |
| 1 | 0.37916983 | 4.81020038 | 2.93149726 |
| 31 | 0.47116281 | 2.50955942 | 0.93181979 |
| 1 | -1.12878579 | 7.41853207 | -2.86236433 |
| 1 | 6.81706425 | 2.68148903 | 0.05975246 |
| 8 | -2.49915533 | -3.44349872 | -1.59221259 |
| 6 | -1.43249483 | -2.67992168 | -2.20006543 |
| 1 | -1.26587181 | -3.14837507 | -3.17538262 |
| 6 | -2.43478409 | -1.82901801 | 0.27810151 |
| 6 | -3.09108962 | -1.51255091 | 1.62443376 |
| 6 | -2.67472314 | -0.15029296 | 2.19863084 |
| 6 | -2.95499425 | -3.10893918 | -0.34719895 |
| 6 | -1.21085529 | -0.06404627 | 2.64372787 |
| 6 | -0.80573403 | 1.32395703 | 3.14316729 |
| 1 | -1.34165557 | -1.92736507 | 0.37558959 |
| 1 | -2.85296240 | -2.31472037 | 2.34041096 |

| | | | |
|---|---|---|---|
| 1 | -2.87424220 | 0.64096064 | 1.45824369 |
| 1 | -0.53078178 | -0.34804533 | 1.82286583 |
| 1 | -2.59779554 | -0.99835651 | -0.42956295 |
| 1 | -4.18319847 | -1.53861458 | 1.49691766 |
| 1 | -3.31710092 | 0.07640020 | 3.06626588 |
| 1 | -1.02573686 | -0.78906138 | 3.45627439 |
| 1 | 0.21038419 | 1.26892055 | 3.57564471 |
| 1 | -1.49192600 | 1.64145116 | 3.94863911 |
| 8 | -3.75765059 | -3.85698194 | 0.18267237 |
| 8 | -0.88533716 | 2.33451650 | 2.12637799 |
| 1 | -0.51701202 | -2.74966731 | -1.59774974 |
| 1 | -1.72140572 | -1.62963272 | -2.34586266 |
| 8 | 0.30937586 | -4.05706587 | 0.58651899 |
| 8 | -0.26546839 | -5.78111157 | -0.67428419 |
| 6 | -0.07601863 | -5.21433878 | 0.54880853 |
| 6 | -0.35081267 | -6.03540143 | 1.79651691 |
| 1 | 0.20581634 | -6.98652264 | 1.74852101 |
| 1 | 0.06384535 | -5.44930027 | 2.62502048 |
| 6 | -1.84885015 | -6.31984230 | 2.04241919 |
| 1 | -2.43034116 | -5.39971624 | 1.87911312 |
| 1 | -1.96943264 | -6.58452382 | 3.10387184 |
| 6 | -2.41632937 | -7.45442338 | 1.18366242 |
| 1 | -1.91402620 | -8.39882097 | 1.45995982 |
| 1 | -3.48148760 | -7.58648655 | 1.42721906 |
| 6 | -2.27187541 | -7.24017879 | -0.32702043 |
| 1 | -2.83110145 | -6.34743129 | -0.64923047 |
| 1 | -2.71842256 | -8.09951630 | -0.85376304 |
| 6 | -0.83017334 | -7.11723020 | -0.81058714 |
| 1 | -0.16914227 | -7.84677502 | -0.31324162 |
| 1 | -0.77242564 | -7.29190531 | -1.89190104 |

Electronic energy = -3908.666153 a.u.
DFT-D3(BJ) dispersion correction = -0.136811 a.u.
Thermal free energy = 0.734862 a.u.
Gibbs free energy = -3908.068103 a.u.
Number of imaginary frequencies = 0.

## Propagation step of axial CL addition to the product of CL addition on initiation step

**Int2 CL C(O)ax CL isomer/conformer 1**

| Atomic N. | X | Y | Z |
|---|---|---|---|
| 7 | 0.56496588 | 2.91147832 | 0.79334702 |
| 8 | -1.46084790 | 0.83345407 | 0.59951571 |
| 8 | 0.60897787 | 1.66605175 | -1.86952910 |
| 6 | 1.00939079 | 2.77379196 | 2.19898286 |
| 6 | -2.15677327 | 1.66858055 | 1.40131963 |
| 6 | -3.25666839 | 1.14414196 | 2.15196717 |
| 6 | -3.98254259 | 2.03223014 | 2.95649916 |
| 6 | -3.66665129 | 3.38892924 | 3.06193040 |
| 6 | -2.58689535 | 3.88556397 | 2.34211290 |
| 6 | -1.83759035 | 3.04753807 | 1.50819240 |

| | | | |
|---|---|---|---|
| 6 | -0.72770388 | 3.65694786 | 0.69471801 |
| 6 | -3.62791327 | -0.35084757 | 2.10108593 |
| 6 | 0.79614344 | 2.93092246 | -2.29192811 |
| 6 | 0.52310297 | 3.25637488 | -3.65687714 |
| 6 | 0.74324063 | 4.57961281 | -4.06212667 |
| 6 | 1.20416634 | 5.57258700 | -3.19212434 |
| 6 | 1.46479411 | 5.24404350 | -1.86683766 |
| 6 | 1.27728406 | 3.93342208 | -1.41231096 |
| 6 | 1.64493093 | 3.59333318 | 0.00701211 |
| 6 | 0.00406457 | 2.19590167 | -4.64822656 |
| 6 | -4.86014717 | -0.67417578 | 2.96763491 |
| 6 | -2.45091357 | -1.19024739 | 2.65217675 |
| 6 | -3.96612144 | -0.77779455 | 0.65299993 |
| 6 | -0.19716528 | 2.77838961 | -6.05992053 |
| 6 | -1.36327889 | 1.64841655 | -4.17455190 |
| 6 | 1.02069308 | 1.03558625 | -4.77000826 |
| 1 | 1.19147077 | 3.76807658 | 2.64157764 |
| 1 | 0.23848786 | 2.25985578 | 2.78285791 |
| 1 | -0.56802147 | 4.70187237 | 1.01462369 |
| 1 | -0.98896906 | 3.68565002 | -0.37436794 |
| 1 | -4.82613036 | 1.65494708 | 3.53148192 |
| 1 | -2.31638495 | 4.94185014 | 2.40980267 |
| 1 | 2.51107506 | 2.91170865 | 0.04072702 |
| 1 | 1.93256867 | 4.51819745 | 0.53769671 |
| 1 | 0.54262844 | 4.85581668 | -5.09583797 |
| 1 | 1.82764572 | 6.00255657 | -1.16915495 |
| 1 | -5.07945011 | -1.74889097 | 2.88900790 |
| 1 | -4.69253109 | -0.44841298 | 4.03065030 |
| 1 | -5.75440230 | -0.12893097 | 2.63308554 |
| 1 | -1.53976139 | -1.03666802 | 2.06351962 |
| 1 | -2.24099716 | -0.91420121 | 3.69629778 |
| 1 | -2.70865933 | -2.26051054 | 2.63150262 |
| 1 | -4.83325982 | -0.21349168 | 0.27994828 |
| 1 | -3.12515961 | -0.60431826 | -0.02646473 |
| 1 | -4.22929929 | -1.84727912 | 0.63181523 |
| 1 | -0.56053192 | 1.98183794 | -6.72489399 |
| 1 | 0.73997374 | 3.16451065 | -6.48698607 |
| 1 | -0.94404223 | 3.58553278 | -6.07318702 |
| 1 | -1.28480679 | 1.15443184 | -3.20080916 |
| 1 | -2.10139909 | 2.46092666 | -4.10255895 |
| 1 | -1.74023593 | 0.91335069 | -4.90218524 |
| 1 | 1.98827536 | 1.40593182 | -5.14031328 |
| 1 | 1.17711239 | 0.54142207 | -3.80549323 |
| 1 | 0.64658162 | 0.28940567 | -5.48773327 |
| 1 | 1.92765380 | 2.17675151 | 2.22034964 |
| 8 | -0.39459441 | -0.76613899 | -1.38403244 |
| 8 | -0.55941266 | -2.61284988 | -2.53883972 |
| 6 | -0.24910905 | -1.98854983 | -1.38297382 |
| 6 | 0.20876490 | -2.78982395 | -0.19051069 |
| 1 | 1.11314185 | -3.36261364 | -0.45271558 |
| 1 | 0.50152801 | -2.05823975 | 0.57235064 |
| 6 | -0.86301226 | -3.76012937 | 0.35489053 |
| 1 | -1.83737784 | -3.24969343 | 0.38972148 |

| | | | |
|---|---|---|---|
| 1 | -0.58401746 | -3.99007899 | 1.39201221 |
| 6 | -0.97830331 | -5.07205928 | -0.42554396 |
| 1 | -0.01594382 | -5.60763710 | -0.35924121 |
| 1 | -1.72447497 | -5.71760655 | 0.06314858 |
| 6 | -1.35155497 | -4.90028431 | -1.90105079 |
| 1 | -2.35695654 | -4.46099198 | -2.00588552 |
| 1 | -1.39255957 | -5.88981954 | -2.38510881 |
| 6 | -0.37148944 | -4.05085374 | -2.70059751 |
| 1 | 0.67383670 | -4.30828876 | -2.46795928 |
| 1 | -0.52657526 | -4.18974338 | -3.77717144 |
| 31 | 0.24843932 | 1.02714672 | -0.14357050 |
| 1 | -4.25630796 | 4.04287888 | 3.70433536 |
| 1 | 1.35279658 | 6.59070602 | -3.55182442 |
| 8 | 2.64550031 | -3.67946789 | 4.41339683 |
| 6 | 1.29739906 | -3.88053211 | 4.90609665 |
| 1 | 0.60330826 | -3.19229202 | 4.40760428 |
| 6 | 4.23410250 | -3.67316729 | 2.63491628 |
| 6 | 4.31905097 | -2.65281133 | 1.48583353 |
| 6 | 4.00948663 | -1.21759546 | 1.92789587 |
| 6 | 2.80419922 | -3.90416399 | 3.07998736 |
| 6 | 4.03681639 | -0.20044806 | 0.77651103 |
| 6 | 2.79087612 | -0.22823798 | -0.11565655 |
| 1 | 4.84386730 | -3.35530882 | 3.49127412 |
| 1 | 3.63034094 | -2.96981725 | 0.68819120 |
| 1 | 3.01820016 | -1.17068886 | 2.40697068 |
| 1 | 4.92696451 | -0.36904440 | 0.14468370 |
| 1 | 4.61512982 | -4.64780431 | 2.29124963 |
| 1 | 5.33462173 | -2.69288269 | 1.06012212 |
| 1 | 4.74252209 | -0.92130413 | 2.69640126 |
| 1 | 4.13268319 | 0.81651655 | 1.19052166 |
| 1 | 2.67990696 | -1.23584403 | -0.56712139 |
| 1 | 2.93807615 | 0.47027744 | -0.96041390 |
| 8 | 1.89354102 | -4.25271768 | 2.34689598 |
| 8 | 1.63464640 | 0.09990172 | 0.65097200 |
| 1 | 1.34813496 | -3.67082781 | 5.97856523 |
| 1 | 0.97383397 | -4.91323058 | 4.72565698 |

Electronic energy = -3908.672972 a.u.
DFT-D3(BJ) dispersion correction = -0.139911 a.u.
Thermal free energy = 0.736956 a.u.
Gibbs free energy = -3908.075927 a.u.
Number of imaginary frequencies = 0.

**Int2 CL C(O)ax CL isomer/conformer 2**

| Atomic N. | X | Y | Z |
|---|---|---|---|
| 7 | -0.48675999 | 3.07275098 | 0.15571650 |
| 8 | -2.39687587 | 0.87287628 | -0.02028534 |
| 8 | 0.32949353 | 1.09650213 | -1.87593692 |
| 6 | -0.45411111 | 3.37707970 | 1.60506006 |
| 6 | -3.28642881 | 1.83925085 | 0.29613831 |
| 6 | -4.56287038 | 1.45501464 | 0.81495377 |
| 6 | -5.47862717 | 2.47200378 | 1.11507190 |
| 6 | -5.18907190 | 3.82743299 | 0.93888652 |

| | | | |
|---|---|---|---|
| 6 | -3.94267801 | 4.19066504 | 0.44329159 |
| 6 | -2.99528125 | 3.21563964 | 0.10999429 |
| 6 | -1.69504168 | 3.65988676 | -0.50400224 |
| 6 | -4.92223322 | -0.02421902 | 1.05750839 |
| 6 | 0.62889271 | 2.19212627 | -2.60084919 |
| 6 | 0.76675125 | 2.07300773 | -4.01846409 |
| 6 | 1.09281135 | 3.23193211 | -4.73551687 |
| 6 | 1.27644418 | 4.47593495 | -4.12404832 |
| 6 | 1.13946768 | 4.57919003 | -2.74469703 |
| 6 | 0.83099996 | 3.44967862 | -1.97721734 |
| 6 | 0.77493216 | 3.58070871 | -0.47859696 |
| 6 | 0.56076068 | 0.72447072 | -4.73663875 |
| 6 | -6.35849567 | -0.19319903 | 1.58873631 |
| 6 | -3.96907964 | -0.61634128 | 2.12211351 |
| 6 | -4.82585057 | -0.83083205 | -0.25945175 |
| 6 | 0.78465047 | 0.84207229 | -6.25616280 |
| 6 | -0.88668344 | 0.22280770 | -4.51996097 |
| 6 | 1.56617920 | -0.32456804 | -4.20426677 |
| 1 | -0.38798949 | 4.46672107 | 1.76469422 |
| 1 | -1.36521976 | 3.00134707 | 2.08259800 |
| 1 | -1.62640807 | 4.76140195 | -0.46596158 |
| 1 | -1.63896204 | 3.37059549 | -1.56485550 |
| 1 | -6.45691738 | 2.20243593 | 1.50858715 |
| 1 | -3.69170085 | 5.24364281 | 0.29601421 |
| 1 | 1.59436338 | 3.01830750 | -0.00090707 |
| 1 | 0.90026762 | 4.64265944 | -0.20281743 |
| 1 | 1.20251914 | 3.17019321 | -5.81673960 |
| 1 | 1.28150581 | 5.54012676 | -2.24455692 |
| 1 | -6.56093059 | -1.26404068 | 1.73564561 |
| 1 | -6.50595222 | 0.30610472 | 2.55720932 |
| 1 | -7.10767413 | 0.19082740 | 0.88149196 |
| 1 | -2.92406973 | -0.56308937 | 1.79716944 |
| 1 | -4.06503450 | -0.06994339 | 3.07180419 |
| 1 | -4.22819154 | -1.66948866 | 2.31366386 |
| 1 | -5.53770818 | -0.44076141 | -1.00148804 |
| 1 | -3.82003037 | -0.78450217 | -0.68983481 |
| 1 | -5.08524678 | -1.88454656 | -0.06958949 |
| 1 | 0.63397185 | -0.14545535 | -6.71531294 |
| 1 | 1.80580910 | 1.16905544 | -6.50111126 |
| 1 | 0.07422574 | 1.53667424 | -6.72758525 |
| 1 | -1.10020179 | 0.04789413 | -3.46064323 |
| 1 | -1.61125342 | 0.95146501 | -4.91253479 |
| 1 | -1.03487771 | -0.72489748 | -5.06014455 |
| 1 | 2.60050459 | -0.00103610 | -4.39382773 |
| 1 | 1.43868533 | -0.48698299 | -3.12889274 |
| 1 | 1.41036420 | -1.28264648 | -4.72364877 |
| 1 | 0.41192859 | 2.87608195 | 2.05136072 |
| 8 | -0.84058199 | -1.18199080 | -1.00017575 |
| 8 | -0.76199238 | -3.29416011 | -1.56454673 |
| 6 | -0.68131158 | -2.33681358 | -0.61392715 |
| 6 | -0.43553444 | -2.72015764 | 0.82741197 |
| 1 | 0.52305286 | -3.26190458 | 0.90026031 |
| 1 | -0.32083826 | -1.77071972 | 1.36736489 |

| | | | |
|---|---|---|---|
| 6 | -1.56227975 | -3.57512373 | 1.44845566 |
| 1 | -2.53795651 | -3.15298282 | 1.16418696 |
| 1 | -1.49106224 | -3.47011722 | 2.54100422 |
| 6 | -1.50411801 | -5.06201727 | 1.08737982 |
| 1 | -0.55687314 | -5.48553658 | 1.46431512 |
| 1 | -2.31062261 | -5.59351433 | 1.61559392 |
| 6 | -1.60977683 | -5.34921216 | -0.41333525 |
| 1 | -2.58878124 | -5.03159732 | -0.80716528 |
| 1 | -1.54478129 | -6.43655869 | -0.58117536 |
| 6 | -0.51891997 | -4.69793554 | -1.25481114 |
| 1 | 0.47260793 | -4.80089027 | -0.78588917 |
| 1 | -0.47379533 | -5.15596513 | -2.25000462 |
| 31 | -0.54222882 | 0.98941324 | -0.22228989 |
| 1 | -5.93055013 | 4.58546096 | 1.19146088 |
| 1 | 1.52068058 | 5.35168434 | -4.72530534 |
| 8 | 8.20761983 | -2.12511968 | 4.18482169 |
| 6 | 8.95429511 | -2.57306822 | 5.34146330 |
| 1 | 8.83877573 | -1.86412235 | 6.17089809 |
| 6 | 6.16704488 | -1.56248353 | 3.11620735 |
| 6 | 4.70895768 | -1.15575750 | 3.32959088 |
| 6 | 4.00631508 | -0.78190516 | 2.02013304 |
| 6 | 6.86262910 | -2.00179961 | 4.39012506 |
| 6 | 2.55003821 | -0.35128633 | 2.22478986 |
| 6 | 1.84426249 | -0.00217127 | 0.91490012 |
| 1 | 6.75247958 | -0.74597448 | 2.66583982 |
| 1 | 4.66706648 | -0.30662380 | 4.02974909 |
| 1 | 4.56278783 | 0.03195761 | 1.52353500 |
| 1 | 2.50685716 | 0.52469606 | 2.89265886 |
| 1 | 6.23662216 | -2.40181248 | 2.40299316 |
| 1 | 4.17199603 | -1.97958251 | 3.82344075 |
| 1 | 4.04303925 | -1.64202803 | 1.32833326 |
| 1 | 1.98686226 | -1.15381932 | 2.72974220 |
| 1 | 1.87731265 | -0.88009735 | 0.23654987 |
| 1 | 2.41380239 | 0.79669611 | 0.39803047 |
| 8 | 6.32471147 | -2.23245446 | 5.45652164 |
| 8 | 0.50345856 | 0.39785256 | 1.18121368 |
| 1 | 9.99701893 | -2.61903753 | 5.01286149 |
| 1 | 8.60463902 | -3.56143574 | 5.66563047 |

Electronic energy = -3908.671723 a.u.
DFT-D3(BJ) dispersion correction = -0.135294 a.u.
Thermal free energy = 0.734679 a.u.
Gibbs free energy = -3908.072338 a.u.
Number of imaginary frequencies = 0.

**Int2 CL C(O)ax CL isomer/conformer 3**

| Atomic N. | X | Y | Z |
|---|---|---|---|
| 7 | -0.69508138 | 2.70407570 | 0.90975055 |
| 8 | -2.56016749 | 0.43530320 | 0.74158595 |
| 8 | -0.09918197 | 0.82853041 | -1.31663748 |
| 6 | -0.33471167 | 2.95745368 | 2.32421274 |
| 6 | -3.34712199 | 1.25262314 | 1.47064498 |
| 6 | -4.42848992 | 0.69737886 | 2.22093375 |

| | | | |
|---|---|---|---|
| 6 | -5.23835421 | 1.58592035 | 2.94181695 |
| 6 | -5.02508446 | 2.96762985 | 2.95149563 |
| 6 | -3.97207615 | 3.49670878 | 2.21274041 |
| 6 | -3.14071665 | 2.65574642 | 1.46558496 |
| 6 | -2.05890347 | 3.24777715 | 0.60524371 |
| 6 | -4.69600753 | -0.82091810 | 2.24161893 |
| 6 | -0.13288061 | 1.95669001 | -2.05641569 |
| 6 | -0.32377679 | 1.87019727 | -3.47011788 |
| 6 | -0.32921376 | 3.06764261 | -4.19855074 |
| 6 | -0.16252884 | 4.31959967 | -3.60001554 |
| 6 | 0.02435969 | 4.39182052 | -2.22427840 |
| 6 | 0.05208513 | 3.22597838 | -1.45103137 |
| 6 | 0.34157171 | 3.32801637 | 0.02168564 |
| 6 | -0.52304761 | 0.51404937 | -4.17486034 |
| 6 | -5.93066033 | -1.17641810 | 3.09126476 |
| 6 | -3.48476773 | -1.56320203 | 2.85451196 |
| 6 | -4.95966933 | -1.33832370 | 0.80716956 |
| 6 | -0.67847564 | 0.67013821 | -5.69943765 |
| 6 | -1.80667093 | -0.17052819 | -3.64917973 |
| 6 | 0.70379232 | -0.39626969 | -3.93037158 |
| 1 | -0.33611884 | 4.04261739 | 2.52591996 |
| 1 | -1.05703181 | 2.46779809 | 2.98469754 |
| 1 | -2.04050998 | 4.34551532 | 0.72723540 |
| 1 | -2.24512608 | 3.03681956 | -0.45840574 |
| 1 | -6.06827875 | 1.19086840 | 3.52530480 |
| 1 | -3.79162367 | 4.57420270 | 2.19775654 |
| 1 | 1.29159450 | 2.82566737 | 0.26645543 |
| 1 | 0.44715595 | 4.39155271 | 0.30018655 |
| 1 | -0.47548328 | 3.02977018 | -5.27658086 |
| 1 | 0.16249657 | 5.35841296 | -1.73426554 |
| 1 | -6.07936331 | -2.26565583 | 3.06672009 |
| 1 | -5.80844269 | -0.88374908 | 4.14427890 |
| 1 | -6.84752023 | -0.70813593 | 2.70459852 |
| 1 | -2.57182304 | -1.39310287 | 2.27426769 |
| 1 | -3.30840060 | -1.22994722 | 3.88769511 |
| 1 | -3.68635643 | -2.64585183 | 2.87883654 |
| 1 | -5.83461009 | -0.83563941 | 0.36928224 |
| 1 | -4.09518895 | -1.16855152 | 0.15662152 |
| 1 | -5.17268846 | -2.41864091 | 0.83753594 |
| 1 | -0.80813911 | -0.32497757 | -6.14927404 |
| 1 | 0.20723743 | 1.13002534 | -6.16124520 |
| 1 | -1.56071772 | 1.26941724 | -5.96658813 |
| 1 | -1.75775331 | -0.35188841 | -2.57053062 |
| 1 | -2.68846567 | 0.45316665 | -3.85556220 |
| 1 | -1.95153541 | -1.13515227 | -4.16145746 |
| 1 | 1.61331577 | 0.06032491 | -4.34797498 |
| 1 | 0.86209814 | -0.57112195 | -2.86052489 |
| 1 | 0.55226787 | -1.36419754 | -4.43507241 |
| 1 | 0.66065735 | 2.54719741 | 2.52410465 |
| 8 | -1.11196092 | -1.58595062 | -0.25103946 |
| 8 | 0.93646913 | -2.38682569 | -0.51094488 |
| 6 | -0.39785584 | -2.56217403 | -0.45612138 |
| 6 | -0.99389277 | -3.93758590 | -0.66938953 |

| | | | |
|---|---|---|---|
| 1 | -0.57654336 | -4.63873498 | 0.07272561 |
| 1 | -2.06283581 | -3.82901520 | -0.45411249 |
| 6 | -0.78435271 | -4.49663381 | -2.09310910 |
| 1 | -0.99502850 | -3.70508652 | -2.82940365 |
| 1 | -1.53934596 | -5.27944265 | -2.25798365 |
| 6 | 0.60595016 | -5.09388770 | -2.33189032 |
| 1 | 0.74234613 | -5.95560533 | -1.65539077 |
| 1 | 0.65471492 | -5.49626583 | -3.35534759 |
| 6 | 1.76577505 | -4.11473703 | -2.12165965 |
| 1 | 1.72773618 | -3.29261777 | -2.85392673 |
| 1 | 2.71739128 | -4.64237941 | -2.29706572 |
| 6 | 1.84190564 | -3.51075984 | -0.72402709 |
| 1 | 1.67371501 | -4.26614024 | 0.05997462 |
| 1 | 2.82608712 | -3.05786066 | -0.55466474 |
| 31 | -0.72501817 | 0.61404257 | 0.44282806 |
| 8 | 0.38921264 | -0.03130429 | 1.75245880 |
| 1 | -5.67907724 | 3.61869654 | 3.53170950 |
| 1 | -0.18112711 | 5.22459683 | -4.20718598 |
| 8 | 7.99234131 | 0.50755787 | 2.28967732 |
| 6 | 8.56583875 | 1.83635385 | 2.23663141 |
| 1 | 8.69984712 | 2.15564289 | 1.19539901 |
| 6 | 6.28020258 | -1.04285939 | 1.74159067 |
| 6 | 4.76408616 | -1.17415656 | 1.56831444 |
| 6 | 3.97345380 | -0.69361163 | 2.79242300 |
| 6 | 6.76814925 | 0.39429165 | 1.69617558 |
| 6 | 2.45135584 | -0.85758628 | 2.67389013 |
| 6 | 1.80213079 | 0.03854312 | 1.61579122 |
| 1 | 6.80921640 | -1.57158837 | 0.93041933 |
| 1 | 4.52778074 | -2.23484011 | 1.37938773 |
| 1 | 4.32284820 | -1.25049357 | 3.67843368 |
| 1 | 1.98166581 | -0.61489693 | 3.64016656 |
| 1 | 6.62389189 | -1.50474534 | 2.67920922 |
| 1 | 4.46270984 | -0.61002530 | 0.67389927 |
| 1 | 4.20898427 | 0.36732516 | 2.97956510 |
| 1 | 2.19381918 | -1.90882699 | 2.46032296 |
| 1 | 2.12638449 | -0.26123537 | 0.60232187 |
| 1 | 2.17417486 | 1.07622080 | 1.77003960 |
| 8 | 6.18473309 | 1.32979898 | 1.18165307 |
| 1 | 9.53132021 | 1.75522228 | 2.74510816 |
| 1 | 7.91493402 | 2.55521678 | 2.74976739 |

Electronic energy = -3908.670003 a.u.
DFT-D3(BJ) dispersion correction = -0.137017 a.u.
Thermal free energy = 0.735748 a.u.
Gibbs free energy = -3908.071271 a.u.
Number of imaginary frequencies = 0.

**Int2 CL C(O)ax CL isomer/conformer 4**

| Atomic N. | X | Y | Z |
|---|---|---|---|
| 7 | -0.09637032 | 3.07013749 | 0.51529865 |
| 8 | -2.26797804 | 1.13666684 | 0.25053662 |
| 8 | 0.34215209 | 1.26116272 | -1.77357260 |
| 6 | 0.07065566 | 3.19730581 | 1.98173119 |

| | | | |
|---|---|---|---|
| 6 | -3.01005771 | 2.15259818 | 0.74305956 |
| 6 | -4.28640569 | 1.85631129 | 1.31679830 |
| 6 | -5.04885063 | 2.92734878 | 1.80113195 |
| 6 | -4.60826232 | 4.25239550 | 1.75425456 |
| 6 | -3.36300743 | 4.52867740 | 1.20284835 |
| 6 | -2.56637011 | 3.49942669 | 0.68779265 |
| 6 | -1.26626216 | 3.86321988 | 0.02298441 |
| 6 | -4.80704248 | 0.40918884 | 1.42105262 |
| 6 | 0.71837442 | 2.39349775 | -2.39981736 |
| 6 | 0.74454073 | 2.42391256 | -3.82848793 |
| 6 | 1.15524306 | 3.61501545 | -4.44185756 |
| 6 | 1.52580896 | 4.75257496 | -3.71813088 |
| 6 | 1.49672387 | 4.71056139 | -2.32909850 |
| 6 | 1.10982121 | 3.54094686 | -1.66408119 |
| 6 | 1.17159089 | 3.50347496 | -0.16044751 |
| 6 | 0.33396817 | 1.19742014 | -4.66750692 |
| 6 | -6.21519614 | 0.34230941 | 2.04229746 |
| 6 | -3.86547250 | -0.41075808 | 2.33395141 |
| 6 | -4.89656522 | -0.23952575 | 0.01908711 |
| 6 | 0.46692259 | 1.46412609 | -6.17885515 |
| 6 | -1.14405838 | 0.83395542 | -4.39024065 |
| 6 | 1.24422860 | -0.00839938 | -4.33239343 |
| 1 | 0.27849333 | 4.24644536 | 2.25243316 |
| 1 | -0.84510212 | 2.87313705 | 2.48732337 |
| 1 | -1.06202361 | 4.93778424 | 0.17501951 |
| 1 | -1.31724872 | 3.69610056 | -1.06407257 |
| 1 | -6.02428678 | 2.72541007 | 2.23966535 |
| 1 | -2.99584071 | 5.55639491 | 1.15352373 |
| 1 | 1.94781405 | 2.80134775 | 0.18658320 |
| 1 | 1.44209419 | 4.50569772 | 0.21625039 |
| 1 | 1.18293040 | 3.66657434 | -5.52880252 |
| 1 | 1.78529512 | 5.58595131 | -1.74247541 |
| 1 | -6.53691521 | -0.70836468 | 2.08505713 |
| 1 | -6.23669065 | 0.73570972 | 3.06883998 |
| 1 | -6.95654006 | 0.89103720 | 1.44376853 |
| 1 | -2.84549765 | -0.43813597 | 1.93473747 |
| 1 | -3.83117341 | 0.02505684 | 3.34320189 |
| 1 | -4.23803432 | -1.44295431 | 2.42708485 |
| 1 | -5.60223064 | 0.31527878 | -0.61631960 |
| 1 | -3.92295825 | -0.25729906 | -0.48170958 |
| 1 | -5.27019566 | -1.27182179 | 0.11070594 |
| 1 | 0.17057100 | 0.55848039 | -6.72720586 |
| 1 | 1.50053097 | 1.70365073 | -6.46864389 |
| 1 | -0.18719455 | 2.28182004 | -6.51489133 |
| 1 | -1.30455249 | 0.56344314 | -3.34173825 |
| 1 | -1.80367928 | 1.67681625 | -4.64431258 |
| 1 | -1.43774077 | -0.02424217 | -5.01406615 |
| 1 | 2.29471772 | 0.22379453 | -4.56262698 |
| 1 | 1.16720669 | -0.28260762 | -3.27504818 |
| 1 | 0.94788636 | -0.87615124 | -4.94158429 |
| 1 | 0.89775365 | 2.55328346 | 2.29996855 |
| 8 | -1.03895133 | -0.95674184 | -1.05939003 |
| 8 | -1.24904755 | -2.98486982 | -1.85246515 |

| | | | |
|---|---|---|---|
| 6 | -0.99351793 | -2.15904780 | -0.81343049 |
| 6 | -0.70177787 | -2.73181026 | 0.55464791 |
| 1 | 0.18861726 | -3.38079268 | 0.49372452 |
| 1 | -0.44065854 | -1.86983557 | 1.18310136 |
| 6 | -1.87894795 | -3.52351023 | 1.16597234 |
| 1 | -2.81426675 | -2.96634521 | 1.00572637 |
| 1 | -1.72476861 | -3.55417929 | 2.25457828 |
| 6 | -2.02071904 | -4.95579290 | 0.64346099 |
| 1 | -1.10756359 | -5.52190014 | 0.89722176 |
| 1 | -2.84811449 | -5.45292840 | 1.17282152 |
| 6 | -2.25749001 | -5.05345852 | -0.86657725 |
| 1 | -3.21566001 | -4.58631056 | -1.14606331 |
| 1 | -2.33274213 | -6.11448478 | -1.15517477 |
| 6 | -1.15448994 | -4.43330578 | -1.71571978 |
| 1 | -0.15366497 | -4.69887893 | -1.33964191 |
| 1 | -1.22905479 | -4.77492329 | -2.75492709 |
| 31 | -0.42949862 | 1.06426949 | -0.07907969 |
| 1 | -5.23300546 | 5.05385724 | 2.14863616 |
| 1 | 1.83027167 | 5.65957412 | -4.24038646 |
| 8 | 8.13179540 | -3.50142307 | 3.32595843 |
| 6 | 8.87838037 | -4.17712180 | 4.36615508 |
| 1 | 8.89023382 | -3.57470880 | 5.28314490 |
| 6 | 6.11969343 | -2.58200144 | 2.47226263 |
| 6 | 4.72966735 | -2.05481069 | 2.82830817 |
| 6 | 4.00688685 | -1.44399241 | 1.62305581 |
| 6 | 6.82317171 | -3.25789790 | 3.63327796 |
| 6 | 2.62141899 | -0.89120037 | 1.97396388 |
| 6 | 1.88760777 | -0.31323015 | 0.76442072 |
| 1 | 6.77113469 | -1.77982573 | 2.09221993 |
| 1 | 4.81965331 | -1.30331052 | 3.62841793 |
| 1 | 4.62531787 | -0.63691645 | 1.19317530 |
| 1 | 2.71351240 | -0.10310355 | 2.73925135 |
| 1 | 6.05810059 | -3.32172293 | 1.65558634 |
| 1 | 4.12951199 | -2.87428809 | 3.25160178 |
| 1 | 3.90975939 | -2.20778486 | 0.83124318 |
| 1 | 2.00058097 | -1.68564954 | 2.42074245 |
| 1 | 1.77670489 | -1.10251222 | -0.00803174 |
| 1 | 2.51638647 | 0.47346321 | 0.30023174 |
| 8 | 6.31710830 | -3.56486504 | 4.69603192 |
| 8 | 0.62153523 | 0.19860920 | 1.16939082 |
| 1 | 9.89015188 | -4.29490571 | 3.96637562 |
| 1 | 8.43121733 | -5.15457478 | 4.58717953 |

Electronic energy = -3908.671724 a.u.
DFT-D3(BJ) dispersion correction = -0.135284 a.u.
Thermal free energy = 0.736985 a.u.
Gibbs free energy = -3908.070023 a.u.
Number of imaginary frequencies = 0.

**Int2 CL C(O)ax CL isomer/conformer 5**

| Atomic N. | X | Y | Z |
|---|---|---|---|
| 7 | -0.49342068 | 2.78512938 | 0.80020037 |
| 8 | -2.28893212 | 0.47145457 | 1.02335574 |

| | | | |
|---|---|---|---|
| 8 | 0.05999531 | 0.76403355 | -1.26660404 |
| 6 | -0.17536519 | 3.17522511 | 2.19331295 |
| 6 | -3.12261802 | 1.38759744 | 1.55983926 |
| 6 | -4.21590286 | 0.94448712 | 2.36800155 |
| 6 | -5.07959052 | 1.91888992 | 2.88609408 |
| 6 | -4.90733361 | 3.28581092 | 2.65256934 |
| 6 | -3.83747603 | 3.70651463 | 1.87132064 |
| 6 | -2.95360188 | 2.77477086 | 1.31564708 |
| 6 | -1.85986805 | 3.26214026 | 0.40696312 |
| 6 | -4.43450091 | -0.54876405 | 2.67922058 |
| 6 | 0.06739261 | 1.83189727 | -2.09217530 |
| 6 | -0.10743618 | 1.63502716 | -3.49656695 |
| 6 | -0.06794166 | 2.77115864 | -4.31644512 |
| 6 | 0.12404432 | 4.06259910 | -3.81681701 |
| 6 | 0.29128736 | 4.24106988 | -2.44873836 |
| 6 | 0.27698918 | 3.13916976 | -1.58582206 |
| 6 | 0.54693529 | 3.35410377 | -0.12182054 |
| 6 | -0.34148712 | 0.23349642 | -4.09477212 |
| 6 | -5.68968092 | -0.78405914 | 3.54097654 |
| 6 | -3.22459006 | -1.09050158 | 3.47607057 |
| 6 | -4.61956462 | -1.35460717 | 1.37148070 |
| 6 | -0.46409639 | 0.27661404 | -5.62986446 |
| 6 | -1.65749376 | -0.36249862 | -3.54169911 |
| 6 | 0.84407046 | -0.70168698 | -3.75689984 |
| 1 | -0.21198847 | 4.27301418 | 2.29820715 |
| 1 | -0.89964824 | 2.72573747 | 2.88000714 |
| 1 | -1.85792290 | 4.36623680 | 0.38655660 |
| 1 | -2.02111324 | 2.91889830 | -0.62624857 |
| 1 | -5.91977759 | 1.60538772 | 3.50297411 |
| 1 | -3.68233023 | 4.76921544 | 1.67093508 |
| 1 | 1.50194225 | 2.88924146 | 0.17247800 |
| 1 | 0.63201558 | 4.43705266 | 0.07705222 |
| 1 | -0.19868262 | 2.65109618 | -5.39031269 |
| 1 | 0.44730192 | 5.24005203 | -2.03493163 |
| 1 | -5.79904143 | -1.86263130 | 3.72539569 |
| 1 | -5.62188222 | -0.28724274 | 4.51958920 |
| 1 | -6.60534584 | -0.43993815 | 3.03881852 |
| 1 | -2.29322777 | -0.98988925 | 2.90794521 |
| 1 | -3.11216269 | -0.54516761 | 4.42459563 |
| 1 | -3.38055958 | -2.15376362 | 3.71707259 |
| 1 | -5.50589792 | -1.00136477 | 0.82446349 |
| 1 | -3.74883395 | -1.26283701 | 0.71370619 |
| 1 | -4.77613247 | -2.41872845 | 1.60977124 |
| 1 | -0.62053025 | -0.74593713 | -6.00225290 |
| 1 | 0.44562296 | 0.66838973 | -6.10801182 |
| 1 | -1.32020900 | 0.88211743 | -5.96120280 |
| 1 | -1.62305583 | -0.48376594 | -2.45440608 |
| 1 | -2.51138221 | 0.28085194 | -3.80064085 |
| 1 | -1.83302974 | -1.35281993 | -3.98946421 |
| 1 | 1.78325138 | -0.30152214 | -4.16695854 |
| 1 | 0.95636104 | -0.82510728 | -2.67489700 |
| 1 | 0.67231235 | -1.69210810 | -4.20571874 |
| 1 | 0.82697009 | 2.81578068 | 2.45034912 |

| | | | |
|---|---|---|---|
| 8 | -0.85660160 | -1.54821056 | -0.16007365 |
| 8 | -0.76287466 | -3.64516902 | -0.78177928 |
| 6 | -0.54896360 | -2.69243565 | 0.15658597 |
| 6 | 0.00790657 | -3.07812142 | 1.50789443 |
| 1 | 0.98360990 | -3.57497296 | 1.37159493 |
| 1 | 0.19206630 | -2.12892565 | 2.02820584 |
| 6 | -0.92932950 | -3.99418852 | 2.32585594 |
| 1 | -1.96134248 | -3.61952054 | 2.25059622 |
| 1 | -0.64590682 | -3.89667451 | 3.38421005 |
| 6 | -0.87364619 | -5.47283899 | 1.93111579 |
| 1 | 0.14866232 | -5.85153940 | 2.10555390 |
| 1 | -1.53333714 | -6.05014908 | 2.59724398 |
| 6 | -1.25968638 | -5.75105242 | 0.47513475 |
| 1 | -2.31180633 | -5.48133596 | 0.28860439 |
| 1 | -1.17569283 | -6.83205985 | 0.27714696 |
| 6 | -0.39143202 | -5.03503910 | -0.55302694 |
| 1 | 0.67736922 | -5.08995226 | -0.29131419 |
| 1 | -0.52136693 | -5.48291384 | -1.54551652 |
| 31 | -0.49916711 | 0.67402964 | 0.51975655 |
| 1 | -5.60238571 | 4.00815251 | 3.08058732 |
| 1 | 0.13973083 | 4.91592104 | -4.49478821 |
| 8 | 8.29080414 | 0.77242432 | -0.56454406 |
| 6 | 8.83254262 | 2.06081221 | -0.94283276 |
| 1 | 8.81487821 | 2.17992793 | -2.03352682 |
| 6 | 6.53966373 | -0.82914077 | -0.56699852 |
| 6 | 5.01812384 | -1.00026003 | -0.57331475 |
| 6 | 4.31078049 | -0.24840544 | 0.56005714 |
| 6 | 6.99617726 | 0.56718068 | -0.94939385 |
| 6 | 2.79663978 | -0.48687588 | 0.58069391 |
| 6 | 2.10139384 | 0.29799877 | 1.69880534 |
| 1 | 7.00718617 | -1.51598003 | -1.29272645 |
| 1 | 4.79099688 | -2.07596720 | -0.49582007 |
| 1 | 4.74551075 | -0.55572156 | 1.52810475 |
| 1 | 2.59265422 | -1.56197512 | 0.71858603 |
| 1 | 6.97372888 | -1.08557161 | 0.41127243 |
| 1 | 4.62228753 | -0.65925716 | -1.54165566 |
| 1 | 4.50991074 | 0.83029787 | 0.45274583 |
| 1 | 2.35809098 | -0.19988686 | -0.38886506 |
| 1 | 2.35218696 | 1.37318969 | 1.57285318 |
| 1 | 2.53368423 | -0.00076812 | 2.67219723 |
| 8 | 6.33495391 | 1.39930610 | -1.54093537 |
| 8 | 0.69802332 | 0.09190217 | 1.79499405 |
| 1 | 9.86091946 | 2.06356951 | -0.56886112 |
| 1 | 8.25021889 | 2.87087624 | -0.48612255 |

Electronic energy = -3908.669022 a.u.
DFT-D3(BJ) dispersion correction = -0.137689 a.u.
Thermal free energy = 0.737037 a.u.
Gibbs free energy = -3908.069675 a.u.
Number of imaginary frequencies = 0.

**Int2 CL C(O)ax CL isomer/conformer 6**

Atomic N. X Y Z

| | | | |
|---|---|---|---|
| 7 | 0.08886230 | 2.74776157 | 0.87491225 |
| 8 | 1.18441177 | 0.70329834 | -0.95646929 |
| 8 | 0.53667434 | 0.08067825 | 2.13233353 |
| 6 | -1.00926471 | 3.53503447 | 0.26992679 |
| 6 | 1.48967388 | 1.78045373 | -1.71013370 |
| 6 | 1.78559664 | 1.60641159 | -3.09674402 |
| 6 | 2.08668234 | 2.75736876 | -3.83893815 |
| 6 | 2.11787865 | 4.03686278 | -3.27563767 |
| 6 | 1.86670254 | 4.18687064 | -1.91536095 |
| 6 | 1.56996774 | 3.07061413 | -1.12598604 |
| 6 | 1.41522756 | 3.22235369 | 0.36268800 |
| 6 | 1.79871113 | 0.20960451 | -3.75101671 |
| 6 | 1.30299104 | 0.79566315 | 2.98529565 |
| 6 | 2.28853196 | 0.13445692 | 3.78009196 |
| 6 | 3.03920599 | 0.92042930 | 4.66553941 |
| 6 | 2.86251947 | 2.30120962 | 4.79095663 |
| 6 | 1.89693562 | 2.93298273 | 4.01569595 |
| 6 | 1.11016491 | 2.19314978 | 3.12584899 |
| 6 | 0.00869583 | 2.88164972 | 2.36838591 |
| 6 | 2.53124459 | -1.38354689 | 3.66940612 |
| 6 | 2.20074681 | 0.27466236 | -5.23655390 |
| 6 | 0.39529609 | -0.43737173 | -3.68240072 |
| 6 | 2.82901940 | -0.69534969 | -3.03315592 |
| 6 | 3.61365816 | -1.87012188 | 4.65152517 |
| 6 | 3.01200258 | -1.73608699 | 2.24224960 |
| 6 | 1.23232291 | -2.15615117 | 4.00039548 |
| 1 | -0.90819298 | 4.59896695 | 0.54644974 |
| 1 | -0.97020746 | 3.44596666 | -0.82003532 |
| 1 | 1.55149995 | 4.28037391 | 0.64975869 |
| 1 | 2.17897539 | 2.63403610 | 0.89266912 |
| 1 | 2.30605450 | 2.65808121 | -4.90058938 |
| 1 | 1.91735961 | 5.17283353 | -1.44740605 |
| 1 | -0.97323177 | 2.47011293 | 2.65380982 |
| 1 | 0.00557759 | 3.95634897 | 2.62320111 |
| 1 | 3.79944348 | 0.44282757 | 5.28094838 |
| 1 | 1.73474184 | 4.00988426 | 4.10164884 |
| 1 | 2.20796658 | -0.74450012 | -5.64871774 |
| 1 | 1.49014938 | 0.86457564 | -5.83335069 |
| 1 | 3.20745536 | 0.69507542 | -5.37498441 |
| 1 | 0.05150241 | -0.54038076 | -2.64776518 |
| 1 | -0.33856087 | 0.15553379 | -4.24694155 |
| 1 | 0.42872797 | -1.43958830 | -4.13730317 |
| 1 | 3.83783629 | -0.26208083 | -3.09954595 |
| 1 | 2.57443515 | -0.83174597 | -1.97653471 |
| 1 | 2.85440718 | -1.68289957 | -3.51967195 |
| 1 | 3.73706710 | -2.95685128 | 4.53761576 |
| 1 | 3.34041906 | -1.67555406 | 5.69874875 |
| 1 | 4.59072810 | -1.40604026 | 4.45475339 |
| 1 | 2.27454633 | -1.45532659 | 1.48333872 |
| 1 | 3.95590123 | -1.22017509 | 2.01448611 |
| 1 | 3.19706338 | -2.81955525 | 2.16785894 |
| 1 | 0.91147995 | -1.94917472 | 5.03204578 |
| 1 | 0.41912882 | -1.87882849 | 3.32077148 |

| | | | |
|---|---|---|---|
| 1 | 1.41554363 | -3.23954342 | 3.91780567 |
| 1 | -1.97216081 | 3.15219706 | 0.62356878 |
| 8 | 0.13433426 | -1.60400272 | -0.10316399 |
| 8 | -1.44481022 | -2.59048353 | 1.09428353 |
| 6 | -0.50217322 | -2.62716087 | 0.13530688 |
| 6 | -0.22315789 | -3.91325323 | -0.61140158 |
| 1 | -1.14707692 | -4.25828249 | -1.10516676 |
| 1 | 0.48944365 | -3.64561129 | -1.39952937 |
| 6 | 0.34981085 | -5.04025030 | 0.27507548 |
| 1 | 1.13323950 | -4.62617684 | 0.92926141 |
| 1 | 0.85118488 | -5.76093326 | -0.38747065 |
| 6 | -0.70336162 | -5.78227089 | 1.10393813 |
| 1 | -1.40963778 | -6.27932701 | 0.41650511 |
| 1 | -0.21265578 | -6.58608523 | 1.67373994 |
| 6 | -1.49741162 | -4.89548009 | 2.06893567 |
| 1 | -0.84175052 | -4.46486290 | 2.84244729 |
| 1 | -2.23779097 | -5.51535748 | 2.60009061 |
| 6 | -2.26419124 | -3.76045979 | 1.40098971 |
| 1 | -2.77679582 | -4.09274885 | 0.48472620 |
| 1 | -3.01833873 | -3.34940394 | 2.08232688 |
| 31 | -0.05150392 | 0.64077385 | 0.44144588 |
| 1 | 2.35237391 | 4.90222843 | -3.89570509 |
| 1 | 3.47632707 | 2.87122116 | 5.48826276 |
| 8 | -2.72977549 | 1.88947018 | -4.57827761 |
| 6 | -1.94407095 | 2.87077887 | -3.87404190 |
| 1 | -1.27258056 | 3.30078965 | -4.62419725 |
| 6 | -3.75288967 | 1.31103085 | -2.39660660 |
| 6 | -4.89831044 | 0.49530282 | -1.79622594 |
| 6 | -5.05640759 | 0.61714831 | -0.27021590 |
| 6 | -3.62721350 | 1.10298789 | -3.89340629 |
| 6 | -4.10571783 | -0.23016703 | 0.60530117 |
| 6 | -2.79670194 | 0.40971941 | 1.07816399 |
| 1 | -2.79838205 | 1.04813825 | -1.90707529 |
| 1 | -4.76805799 | -0.56257359 | -2.06953880 |
| 1 | -4.98886469 | 1.67687439 | 0.03446146 |
| 1 | -3.86776776 | -1.17439862 | 0.08739505 |
| 1 | -3.90267545 | 2.38445759 | -2.19055658 |
| 1 | -5.83650134 | 0.80902433 | -2.27872153 |
| 1 | -6.08653633 | 0.30868219 | -0.03111182 |
| 1 | -4.64810908 | -0.50814395 | 1.52598996 |
| 1 | -2.38724008 | -0.21313767 | 1.89472335 |
| 1 | -3.04235580 | 1.39804702 | 1.52858801 |
| 8 | -4.25799372 | 0.28271307 | -4.52872446 |
| 8 | -1.84766015 | 0.55851336 | 0.02578104 |
| 1 | -1.34195349 | 2.40903842 | -3.08024298 |
| 1 | -2.57840790 | 3.66617456 | -3.45584642 |

Electronic energy = -3908.659316 a.u.
DFT-D3(BJ) dispersion correction = -0.143440 a.u.
Thermal free energy = 0.741654 a.u.
Gibbs free energy = -3908.061102 a.u.
Number of imaginary frequencies = 0.

**TS3 ax CL isomer/conformer 1**

| Atomic N. | X | Y | Z |
|---|---|---|---|
| 7 | -0.11753414 | 2.43843603 | 0.20069116 |
| 8 | -2.59558702 | 1.03976393 | -0.50081074 |
| 8 | 0.09737338 | 0.72910178 | -2.22770500 |
| 6 | 0.21671547 | 2.42556620 | 1.64427161 |
| 6 | -3.17249894 | 1.92627513 | 0.33397236 |
| 6 | -4.47494841 | 1.67979360 | 0.86481637 |
| 6 | -5.00137138 | 2.64062834 | 1.74050903 |
| 6 | -4.31747246 | 3.80927920 | 2.09115100 |
| 6 | -3.06570339 | 4.05575895 | 1.53625143 |
| 6 | -2.49999428 | 3.13120151 | 0.65292865 |
| 6 | -1.21631777 | 3.43589687 | -0.05682878 |
| 6 | -5.28076744 | 0.42436141 | 0.47949752 |
| 6 | 0.41236159 | 1.89319205 | -2.83340669 |
| 6 | 0.30635005 | 2.00501919 | -4.25225687 |
| 6 | 0.66241980 | 3.23211217 | -4.82857132 |
| 6 | 1.10584784 | 4.32446909 | -4.07620096 |
| 6 | 1.21279669 | 4.19981337 | -2.69579816 |
| 6 | 0.88224334 | 2.99179697 | -2.07016373 |
| 6 | 1.10058451 | 2.84470916 | -0.58876294 |
| 6 | -0.18210369 | 0.82686454 | -5.11797619 |
| 6 | -6.66842937 | 0.40275511 | 1.14713979 |
| 6 | -4.53027773 | -0.85337466 | 0.92188400 |
| 6 | -5.50129414 | 0.39335427 | -1.05187857 |
| 6 | -0.18707860 | 1.18082881 | -6.61713886 |
| 6 | -1.62989247 | 0.44310293 | -4.72976017 |
| 6 | 0.75186419 | -0.39335611 | -4.93438435 |
| 1 | 0.52854490 | 3.43327212 | 1.96766031 |
| 1 | -0.66261458 | 2.12449241 | 2.22363997 |
| 1 | -0.84395164 | 4.43369436 | 0.23476405 |
| 1 | -1.37085915 | 3.44182825 | -1.14539278 |
| 1 | -5.98756027 | 2.47597861 | 2.17125297 |
| 1 | -2.52557492 | 4.97701454 | 1.76758981 |
| 1 | 1.86773300 | 2.08250618 | -0.38665989 |
| 1 | 1.46747053 | 3.80102777 | -0.17646180 |
| 1 | 0.58631647 | 3.34979796 | -5.90805578 |
| 1 | 1.56460679 | 5.03763056 | -2.08914280 |
| 1 | -7.20405722 | -0.50229597 | 0.82665515 |
| 1 | -6.60069942 | 0.37548396 | 2.24467921 |
| 1 | -7.28071070 | 1.26920855 | 0.85803044 |
| 1 | -3.56491876 | -0.94455850 | 0.41214862 |
| 1 | -4.36526394 | -0.84721702 | 2.01030469 |
| 1 | -5.13497353 | -1.74078107 | 0.67801064 |
| 1 | -6.06751490 | 1.27769482 | -1.37912035 |
| 1 | -4.54842567 | 0.36712167 | -1.59174359 |
| 1 | -6.08183527 | -0.50087845 | -1.32601595 |
| 1 | -0.53255814 | 0.30729559 | -7.18809938 |
| 1 | 0.81637549 | 1.44077075 | -6.98478413 |
| 1 | -0.86809790 | 2.01383901 | -6.84468079 |
| 1 | -1.69543147 | 0.10521204 | -3.69043684 |
| 1 | -2.30802917 | 1.29836428 | -4.86409950 |
| 1 | -1.98241007 | -0.37357655 | -5.37800264 |

| | | | |
|---|---|---|---|
| 1 | 1.77882600 | -0.14428622 | -5.24052382 |
| 1 | 0.76655400 | -0.73377883 | -3.89352846 |
| 1 | 0.40325791 | -1.22375870 | -5.56729412 |
| 1 | 1.02984071 | 1.71737230 | 1.83082139 |
| 8 | -1.26695848 | -1.33072281 | -0.65491128 |
| 8 | 0.49825045 | -2.70289540 | -0.67388397 |
| 6 | -0.39278251 | -1.97273266 | 0.08075699 |
| 6 | -0.91656250 | -2.59591198 | 1.37194574 |
| 1 | -0.09544117 | -2.82281780 | 2.06756496 |
| 1 | -1.52997625 | -1.81778419 | 1.84197557 |
| 6 | -1.76023722 | -3.85877681 | 1.11425567 |
| 1 | -2.39054775 | -3.69842839 | 0.22581685 |
| 1 | -2.44876909 | -3.99332333 | 1.96190858 |
| 6 | -0.91757876 | -5.13129091 | 0.96125776 |
| 1 | -0.36659071 | -5.30324125 | 1.90270996 |
| 1 | -1.58503091 | -5.99795008 | 0.83430660 |
| 6 | 0.08709906 | -5.10202074 | -0.19675866 |
| 1 | -0.43541402 | -5.07359982 | -1.16621077 |
| 1 | 0.67757583 | -6.03319645 | -0.18511310 |
| 6 | 1.06981575 | -3.93339133 | -0.16120230 |
| 1 | 1.48313260 | -3.78540754 | 0.85023743 |
| 1 | 1.90944970 | -4.12334108 | -0.84295017 |
| 31 | -0.77534306 | 0.54499227 | -0.58922064 |
| 1 | -4.77060795 | 4.52228689 | 2.77996540 |
| 1 | 1.36298037 | 5.26127529 | -4.57047129 |
| 8 | 8.08388805 | -1.69935846 | 4.72113132 |
| 6 | 8.74764824 | -2.07801962 | 5.95155382 |
| 1 | 8.42995834 | -1.42617345 | 6.77494335 |
| 6 | 6.12493753 | -1.37354064 | 3.42815804 |
| 6 | 4.59677744 | -1.38542020 | 3.42361856 |
| 6 | 4.01196051 | -0.99502605 | 2.06216407 |
| 6 | 6.72234201 | -1.78014624 | 4.76211071 |
| 6 | 2.47873950 | -0.99404812 | 2.04873872 |
| 6 | 1.91730583 | -0.62538540 | 0.67526913 |
| 1 | 6.51815844 | -0.37688458 | 3.17055917 |
| 1 | 4.22548103 | -0.70012188 | 4.20112562 |
| 1 | 4.38131189 | 0.00557241 | 1.77664101 |
| 1 | 2.09604454 | -0.28609583 | 2.80221558 |
| 1 | 6.52878475 | -2.05452466 | 2.66079640 |
| 1 | 4.24141216 | -2.38662171 | 3.71144032 |
| 1 | 4.38503933 | -1.68942547 | 1.28926015 |
| 1 | 2.10873127 | -1.98870790 | 2.34560061 |
| 1 | 2.25026345 | -1.34196031 | -0.09440980 |
| 1 | 2.30931211 | 0.35733458 | 0.36904505 |
| 8 | 6.09934282 | -2.13565612 | 5.74450254 |
| 8 | 0.48401839 | -0.56640142 | 0.65986860 |
| 1 | 9.81671429 | -1.95817101 | 5.75196171 |
| 1 | 8.51403904 | -3.11876416 | 6.20874214 |

Electronic energy = -3908.650328 a.u.
DFT-D3(BJ) dispersion correction = -0.137160 a.u.
Thermal free energy = 0.737442 a.u.
Gibbs free energy = -3908.050047 a.u.
Number of imaginary frequencies = 1.

**TS3 ax CL isomer/conformer 2**

| Atomic N. | X | Y | Z |
|---|---|---|---|
| 7 | -0.30397114 | 2.15802407 | 0.97531551 |
| 8 | -2.63283253 | 0.41840988 | 0.69575161 |
| 8 | -0.29111484 | 0.41571736 | -1.44686353 |
| 6 | 0.14745380 | 2.24818587 | 2.38382506 |
| 6 | -3.23670125 | 1.32570398 | 1.48557985 |
| 6 | -4.45223045 | 0.99214274 | 2.15895796 |
| 6 | -5.01617193 | 1.97063470 | 2.98987198 |
| 6 | -4.45207691 | 3.23840523 | 3.16510963 |
| 6 | -3.29229045 | 3.56574869 | 2.47046185 |
| 6 | -2.69342089 | 2.62712574 | 1.62399042 |
| 6 | -1.52285123 | 3.01570868 | 0.77275169 |
| 6 | -5.13526726 | -0.37506908 | 1.96113265 |
| 6 | -0.08068701 | 1.58410549 | -2.08625269 |
| 6 | -0.34620664 | 1.66956425 | -3.48720431 |
| 6 | -0.09401590 | 2.89441085 | -4.11914301 |
| 6 | 0.39317808 | 4.01334906 | -3.43668008 |
| 6 | 0.64825064 | 3.91803813 | -2.07391834 |
| 6 | 0.42808723 | 2.71383333 | -1.39364958 |
| 6 | 0.79795790 | 2.62984729 | 0.06553970 |
| 6 | -0.89115506 | 0.46227562 | -4.27632713 |
| 6 | -6.44401693 | -0.48786145 | 2.76501417 |
| 6 | -4.20381546 | -1.51865363 | 2.42522526 |
| 6 | -5.48855209 | -0.56949270 | 0.46703362 |
| 6 | -1.07863343 | 0.79061449 | -5.76970959 |
| 6 | -2.27267194 | 0.04166312 | -3.72053647 |
| 6 | 0.09663375 | -0.72582865 | -4.18688393 |
| 1 | 0.39695352 | 3.29337549 | 2.63336921 |
| 1 | -0.65425700 | 1.90837371 | 3.04839039 |
| 1 | -1.24004543 | 4.06514740 | 0.96690673 |
| 1 | -1.77890599 | 2.93425012 | -0.29367793 |
| 1 | -5.93563461 | 1.74066150 | 3.52550149 |
| 1 | -2.85067415 | 4.56090879 | 2.56321120 |
| 1 | 1.64059496 | 1.93974710 | 0.22147909 |
| 1 | 1.12338090 | 3.62608838 | 0.41205671 |
| 1 | -0.28940916 | 2.98831058 | -5.18585157 |
| 1 | 1.03358030 | 4.77850580 | -1.52204335 |
| 1 | -6.89461040 | -1.47281814 | 2.57623911 |
| 1 | -6.27423477 | -0.40371907 | 3.84856772 |
| 1 | -7.17931652 | 0.27393404 | 2.46824074 |
| 1 | -3.29026723 | -1.55165207 | 1.82198596 |
| 1 | -3.93433762 | -1.39278126 | 3.48521924 |
| 1 | -4.72265339 | -2.48437643 | 2.32179308 |
| 1 | -6.18365526 | 0.21293446 | 0.12911895 |
| 1 | -4.59193945 | -0.53812432 | -0.16174504 |
| 1 | -5.98016119 | -1.54405856 | 0.32395372 |
| 1 | -1.46199131 | -0.10150511 | -6.28512621 |
| 1 | -0.13204417 | 1.07159019 | -6.25412893 |
| 1 | -1.80495524 | 1.60094941 | -5.92838430 |
| 1 | -2.21098232 | -0.26656005 | -2.67174911 |

| | | | |
|---|---|---|---|
| 1 | -2.99098754 | 0.87064385 | -3.80033755 |
| 1 | -2.66567636 | -0.80404407 | -4.30518770 |
| 1 | 1.07403839 | -0.44767588 | -4.60871649 |
| 1 | 0.23964010 | -1.05131173 | -3.15113449 |
| 1 | -0.29300460 | -1.57532346 | -4.76846552 |
| 1 | 1.02939093 | 1.61729085 | 2.53104639 |
| 8 | -1.04337502 | -1.76687467 | 0.39859000 |
| 8 | 0.75791073 | -3.07584262 | 0.45644641 |
| 6 | -0.08546018 | -2.23963165 | 1.14053750 |
| 6 | -0.39586025 | -2.57506633 | 2.59597731 |
| 1 | 0.52146888 | -2.57557416 | 3.20404744 |
| 1 | -1.02299488 | -1.75329518 | 2.96159840 |
| 6 | -1.13899815 | -3.91734481 | 2.74732518 |
| 1 | -1.87953854 | -4.01654959 | 1.93842852 |
| 1 | -1.71124256 | -3.88916278 | 3.68664527 |
| 6 | -0.20292765 | -5.13176441 | 2.77916622 |
| 1 | 0.47431797 | -5.02591372 | 3.64472151 |
| 1 | -0.79273468 | -6.04433632 | 2.95748401 |
| 6 | 0.64258599 | -5.31894732 | 1.51446748 |
| 1 | 0.00407505 | -5.55740690 | 0.64887947 |
| 1 | 1.31774938 | -6.17946625 | 1.65387034 |
| 6 | 1.50390446 | -4.11326981 | 1.14612150 |
| 1 | 2.01978111 | -3.70372391 | 2.02913380 |
| 1 | 2.26846051 | -4.40232134 | 0.41303343 |
| 31 | -0.79198593 | 0.17926759 | 0.33217698 |
| 1 | -4.92739296 | 3.96251750 | 3.82684851 |
| 1 | 0.56766909 | 4.94777203 | -3.96990538 |
| 8 | 8.39325619 | 0.48195058 | -0.89972419 |
| 6 | 9.09649754 | 1.67115080 | -0.46626532 |
| 1 | 9.00218487 | 2.46755552 | -1.21513987 |
| 6 | 6.42145920 | -0.62849736 | -1.60821152 |
| 6 | 4.89140463 | -0.61110914 | -1.58418334 |
| 6 | 4.29647207 | -0.64410639 | -0.17155290 |
| 6 | 7.05488586 | 0.65631360 | -1.10541534 |
| 6 | 2.76356769 | -0.66655838 | -0.17932110 |
| 6 | 2.20075989 | -0.60560453 | 1.24259210 |
| 1 | 6.79019022 | -0.77667105 | -2.63686752 |
| 1 | 4.52358198 | -1.47773921 | -2.15603852 |
| 1 | 4.67678833 | -1.53224337 | 0.36448336 |
| 1 | 2.39242315 | -1.57187223 | -0.67965043 |
| 1 | 6.82836437 | -1.46840739 | -1.02398026 |
| 1 | 4.53416920 | 0.28857893 | -2.10715699 |
| 1 | 4.65211535 | 0.23771797 | 0.38629103 |
| 1 | 2.38481426 | 0.18379383 | -0.76646239 |
| 1 | 2.55126047 | 0.31835037 | 1.73994839 |
| 1 | 2.60965204 | -1.44046450 | 1.83998156 |
| 8 | 6.48294497 | 1.71499538 | -0.92383814 |
| 8 | 0.77359235 | -0.65991610 | 1.36205411 |
| 1 | 10.14111996 | 1.36510992 | -0.35501301 |
| 1 | 8.69236851 | 2.02924180 | 0.48895243 |

Electronic energy = -3908.648497 a.u.
DFT-D3(BJ) dispersion correction = -0.139668 a.u.
Thermal free energy = 0.739531 a.u.

Gibbs free energy = -3908.048633 a.u.
Number of imaginary frequencies = 1.

**TS3 ax CL isomer/conformer 3**

| Atomic N. | X | Y | Z |
|---|---|---|---|
| 7 | 0.51218176 | 2.37749887 | 0.88574749 |
| 8 | -2.08092380 | 1.35865045 | 0.02707153 |
| 8 | 0.33103114 | 1.38819158 | -1.95192218 |
| 6 | 0.98440379 | 1.94910347 | 2.22412848 |
| 6 | -2.54967499 | 1.98395610 | 1.12235641 |
| 6 | -3.83516407 | 1.66227603 | 1.65744829 |
| 6 | -4.23856068 | 2.34732938 | 2.81298661 |
| 6 | -3.45322680 | 3.32232833 | 3.43689156 |
| 6 | -2.22262025 | 3.65860319 | 2.88156796 |
| 6 | -1.77833450 | 3.00923014 | 1.72594440 |
| 6 | -0.52963901 | 3.45396256 | 1.02813935 |
| 6 | -4.75221443 | 0.62051922 | 0.98871762 |
| 6 | 0.67094177 | 2.66211510 | -2.23933077 |
| 6 | 0.41535237 | 3.18331099 | -3.54367852 |
| 6 | 0.80768776 | 4.50436783 | -3.79852057 |
| 6 | 1.42282116 | 5.31208086 | -2.83701372 |
| 6 | 1.66881776 | 4.79258871 | -1.57136762 |
| 6 | 1.31034458 | 3.47387838 | -1.26598871 |
| 6 | 1.67242122 | 2.91252208 | 0.08564435 |
| 6 | -0.26763341 | 2.32972026 | -4.63065958 |
| 6 | -6.10798645 | 0.49753317 | 1.70918071 |
| 6 | -4.08312941 | -0.77300984 | 1.01275492 |
| 6 | -5.04170513 | 1.03591323 | -0.47353515 |
| 6 | -0.41729361 | 3.10091877 | -5.95574713 |
| 6 | -1.68777025 | 1.92014682 | -4.17293739 |
| 6 | 0.57576698 | 1.06620896 | -4.92604494 |
| 1 | 1.47919428 | 2.79114890 | 2.73713851 |
| 1 | 0.12459883 | 1.62733710 | 2.82309389 |
| 1 | -0.06973316 | 4.30386639 | 1.56232774 |
| 1 | -0.76210308 | 3.78592205 | 0.00631187 |
| 1 | -5.20790100 | 2.11458540 | 3.25068760 |
| 1 | -1.60634351 | 4.44362736 | 3.32674766 |
| 1 | 2.38335133 | 2.08060122 | -0.01313144 |
| 1 | 2.16274357 | 3.69909596 | 0.68522137 |
| 1 | 0.62224990 | 4.92935562 | -4.78336239 |
| 1 | 2.15194964 | 5.40481281 | -0.80618745 |
| 1 | -6.72689629 | -0.24090192 | 1.17952001 |
| 1 | -5.99680567 | 0.15083052 | 2.74715066 |
| 1 | -6.66056776 | 1.44832325 | 1.71735656 |
| 1 | -3.14010715 | -0.77523339 | 0.45580605 |
| 1 | -3.88707089 | -1.08869481 | 2.04904604 |
| 1 | -4.75660086 | -1.51572645 | 0.55689073 |
| 1 | -5.55274534 | 2.00946689 | -0.50497371 |
| 1 | -4.11846447 | 1.10587811 | -1.05885565 |
| 1 | -5.70052385 | 0.29237211 | -0.94783074 |
| 1 | -0.90149617 | 2.44746852 | -6.69536495 |
| 1 | 0.55485123 | 3.40613794 | -6.36984301 |

| | | | |
|---|---|---|---|
| 1 | -1.04576838 | 3.99660850 | -5.84549427 |
| 1 | -1.66178787 | 1.31217027 | -3.26285768 |
| 1 | -2.30511737 | 2.80955386 | -3.98021173 |
| 1 | -2.17722940 | 1.33331776 | -4.96513748 |
| 1 | 1.57721615 | 1.34508348 | -5.28629684 |
| 1 | 0.68397045 | 0.43912101 | -4.03474140 |
| 1 | 0.08763884 | 0.47108173 | -5.71292543 |
| 1 | 1.68221259 | 1.11293542 | 2.11650348 |
| 8 | -0.91597408 | -1.01364326 | -0.55991209 |
| 8 | 0.55051172 | -2.57247353 | -1.16026962 |
| 6 | 0.01847085 | -1.83147418 | -0.15301649 |
| 6 | -0.14975999 | -2.47372150 | 1.21590400 |
| 1 | 0.80987227 | -2.84448981 | 1.60195589 |
| 1 | -0.48671326 | -1.66999459 | 1.88255394 |
| 6 | -1.19302797 | -3.61097956 | 1.19171500 |
| 1 | -2.04590726 | -3.30896401 | 0.56403077 |
| 1 | -1.58875096 | -3.73480487 | 2.21113133 |
| 6 | -0.61952561 | -4.95476675 | 0.72391766 |
| 1 | 0.16538073 | -5.26750231 | 1.43207181 |
| 1 | -1.40946341 | -5.72098446 | 0.76950854 |
| 6 | -0.02047826 | -4.93926048 | -0.68812716 |
| 1 | -0.80066085 | -4.76588917 | -1.44686329 |
| 1 | 0.41298795 | -5.92966604 | -0.90429425 |
| 6 | 1.08277893 | -3.90455651 | -0.89349532 |
| 1 | 1.77622485 | -3.88272953 | -0.04004273 |
| 1 | 1.65419865 | -4.12670708 | -1.80408108 |
| 31 | -0.29154909 | 0.79724526 | -0.29385969 |
| 1 | -3.81307817 | 3.82152510 | 4.33666897 |
| 1 | 1.70261776 | 6.33704376 | -3.08037543 |
| 8 | 4.27603102 | -4.88731241 | 3.87581038 |
| 6 | 3.23880577 | -5.64740276 | 4.54447222 |
| 1 | 2.37650847 | -5.00567722 | 4.76455212 |
| 6 | 5.09422731 | -3.66487336 | 2.01603341 |
| 6 | 4.68676567 | -2.65331817 | 0.94062424 |
| 6 | 4.01029062 | -1.39770639 | 1.50617266 |
| 6 | 3.92487383 | -4.37968922 | 2.66430354 |
| 6 | 3.72165060 | -0.32236075 | 0.44765863 |
| 6 | 2.50630879 | -0.56769228 | -0.45622090 |
| 1 | 5.70031071 | -3.19403148 | 2.80409789 |
| 1 | 4.03151178 | -3.15015244 | 0.21002574 |
| 1 | 3.07281446 | -1.66813171 | 2.01768255 |
| 1 | 4.60198784 | -0.21107975 | -0.21032862 |
| 1 | 5.72902393 | -4.45356131 | 1.57731688 |
| 1 | 5.59522581 | -2.35411882 | 0.39341760 |
| 1 | 4.67106771 | -0.96643764 | 2.27714252 |
| 1 | 3.60155749 | 0.65072358 | 0.94929320 |
| 1 | 2.57865286 | -1.55471385 | -0.94354236 |
| 1 | 2.49765825 | 0.17038833 | -1.27587759 |
| 8 | 2.81707454 | -4.52432442 | 2.17381763 |
| 8 | 1.25990653 | -0.51690751 | 0.25300282 |
| 1 | 3.69761086 | -6.00829649 | 5.46961689 |
| 1 | 2.91337252 | -6.48662090 | 3.91738605 |

Electronic energy = -3908.650102 a.u.

DFT-D3(BJ) dispersion correction = -0.141722 a.u.
Thermal free energy = 0.743277 a.u.
Gibbs free energy = -3908.048547 a.u.
Number of imaginary frequencies = 1.

**TS3 ax CL isomer/conformer 4**

| Atomic N. | X | Y | Z |
|---|---|---|---|
| 7 | -0.45626861 | 2.04530621 | 0.97595119 |
| 8 | -2.69249058 | 0.31704694 | 0.19447498 |
| 8 | -0.15497069 | 0.84792393 | -1.72446656 |
| 6 | -0.03877374 | 1.86585104 | 2.38649631 |
| 6 | -3.35127783 | 0.96077824 | 1.17690754 |
| 6 | -4.54365540 | 0.40234143 | 1.72995079 |
| 6 | -5.16832733 | 1.11870730 | 2.76108003 |
| 6 | -4.68251085 | 2.33810019 | 3.24439526 |
| 6 | -3.54022098 | 2.88867712 | 2.67195392 |
| 6 | -2.88378620 | 2.21613669 | 1.63653970 |
| 6 | -1.72772932 | 2.85112691 | 0.92505175 |
| 6 | -5.13441038 | -0.92005074 | 1.20417690 |
| 6 | -0.07069091 | 2.13476944 | -2.12014937 |
| 6 | -0.29895875 | 2.47039127 | -3.48856258 |
| 6 | -0.18286105 | 3.82033134 | -3.84733534 |
| 6 | 0.14027829 | 4.82495260 | -2.92959738 |
| 6 | 0.36796591 | 4.48369837 | -1.60133445 |
| 6 | 0.27694926 | 3.14821076 | -1.19190621 |
| 6 | 0.62271106 | 2.78581953 | 0.22661567 |
| 6 | -0.66033018 | 1.39402080 | -4.53108454 |
| 6 | -6.43376160 | -1.30266619 | 1.93691301 |
| 6 | -4.12845850 | -2.07762074 | 1.40434575 |
| 6 | -5.47177362 | -0.78066099 | -0.29966119 |
| 6 | -0.83349299 | 1.99494863 | -5.93881607 |
| 6 | -1.99675632 | 0.71201521 | -4.15307713 |
| 6 | 0.46443215 | 0.33446193 | -4.61575651 |
| 1 | 0.09113193 | 2.84992123 | 2.86809587 |
| 1 | -0.80553180 | 1.30424333 | 2.93052959 |
| 1 | -1.51617479 | 3.84719086 | 1.35196877 |
| 1 | -1.95847416 | 2.98845291 | -0.14120680 |
| 1 | -6.07321262 | 0.71350908 | 3.21085548 |
| 1 | -3.15855050 | 3.85500121 | 3.01033616 |
| 1 | 1.51601768 | 2.14416027 | 0.25241071 |
| 1 | 0.85851240 | 3.70439482 | 0.79172224 |
| 1 | -0.35652518 | 4.10792017 | -4.88276434 |
| 1 | 0.63056660 | 5.25072145 | -0.86897403 |
| 1 | -6.81775188 | -2.24234104 | 1.51461315 |
| 1 | -6.27098131 | -1.46547532 | 3.01253057 |
| 1 | -7.21756935 | -0.54103994 | 1.81542671 |
| 1 | -3.21119571 | -1.90959609 | 0.82944322 |
| 1 | -3.87284374 | -2.18833733 | 2.46935344 |
| 1 | -4.58026981 | -3.02305889 | 1.06573451 |
| 1 | -6.21624279 | 0.01349153 | -0.45697436 |
| 1 | -4.57818604 | -0.54617262 | -0.88848674 |
| 1 | -5.89825726 | -1.72380674 | -0.67447740 |

| | | | |
|---|---|---|---|
| 1 | -1.08027124 | 1.18751538 | -6.64285517 |
| 1 | 0.08651140 | 2.47837710 | -6.29872036 |
| 1 | -1.65137708 | 2.72910881 | -5.97897387 |
| 1 | -1.93030334 | 0.19658199 | -3.18941164 |
| 1 | -2.80904269 | 1.45159400 | -4.10050678 |
| 1 | -2.26371551 | -0.03011735 | -4.92089081 |
| 1 | 1.41386437 | 0.80125850 | -4.91736650 |
| 1 | 0.61020631 | -0.17286687 | -3.65615582 |
| 1 | 0.20396069 | -0.42083439 | -5.37307596 |
| 1 | 0.90757835 | 1.31835592 | 2.42795396 |
| 8 | -0.98175579 | -1.66901958 | -0.53976512 |
| 8 | 1.02449787 | -2.60520029 | -0.87392622 |
| 6 | 0.04037095 | -2.25794721 | 0.02784233 |
| 6 | -0.27891085 | -3.23950267 | 1.15342605 |
| 1 | 0.61151344 | -3.44595909 | 1.76472652 |
| 1 | -0.99751109 | -2.71434522 | 1.79544330 |
| 6 | -0.88428879 | -4.55435228 | 0.62978142 |
| 1 | -1.58840120 | -4.32821560 | -0.18595579 |
| 1 | -1.48025477 | -5.00692606 | 1.43654454 |
| 6 | 0.17119974 | -5.56927872 | 0.17277236 |
| 1 | 0.79901260 | -5.83802652 | 1.04077470 |
| 1 | -0.32853362 | -6.49848413 | -0.14295682 |
| 6 | 1.08390117 | -5.08400488 | -0.95990694 |
| 1 | 0.50946593 | -4.93666744 | -1.88832045 |
| 1 | 1.83478808 | -5.86236525 | -1.17518210 |
| 6 | 1.83624876 | -3.78737640 | -0.66510803 |
| 1 | 2.26952934 | -3.79574585 | 0.34892324 |
| 1 | 2.65956569 | -3.65759277 | -1.38044024 |
| 31 | -0.83164263 | 0.21959506 | -0.10311923 |
| 8 | 0.64638793 | -0.85757667 | 0.89287132 |
| 1 | -5.20403576 | 2.85360026 | 4.05092955 |
| 1 | 0.21165489 | 5.86239433 | -3.25623369 |
| 8 | 8.02118206 | 1.05180025 | 1.94007075 |
| 6 | 8.35548496 | 2.44804764 | 1.74493754 |
| 1 | 8.37208462 | 2.69240570 | 0.67552269 |
| 6 | 6.57294893 | -0.80318296 | 1.65116608 |
| 6 | 5.13003588 | -1.23529840 | 1.37855804 |
| 6 | 4.14008951 | -0.73973966 | 2.44168043 |
| 6 | 6.80591869 | 0.68347722 | 1.44465125 |
| 6 | 2.69107580 | -1.20251107 | 2.22433257 |
| 6 | 2.06599331 | -0.64385200 | 0.94296771 |
| 1 | 7.26958370 | -1.32693112 | 0.97596675 |
| 1 | 5.09782362 | -2.33641553 | 1.33448505 |
| 1 | 4.47604370 | -1.08865857 | 3.43246293 |
| 1 | 2.07324839 | -0.88001567 | 3.07753992 |
| 1 | 6.89135456 | -1.06823289 | 2.67131329 |
| 1 | 4.83790064 | -0.86594671 | 0.38498747 |
| 1 | 4.16648531 | 0.36149123 | 2.47040558 |
| 1 | 2.65089989 | -2.30430007 | 2.21269417 |
| 1 | 2.51598096 | -1.08062935 | 0.03773061 |
| 1 | 2.25733900 | 0.43939498 | 0.89013511 |
| 8 | 6.04182243 | 1.45663893 | 0.89678621 |
| 1 | 9.34852028 | 2.57158160 | 2.18717146 |

| | | | |
|---|---|---|---|
| 1 | 7.62267780 | 3.09110260 | 2.24807525 |

Electronic energy = -3908.649132 a.u.
DFT-D3(BJ) dispersion correction = -0.138564 a.u.
Thermal free energy = 0.742289 a.u.
Gibbs free energy = -3908.045406 a.u.
Number of imaginary frequencies = 1.

**TS3 ax CL isomer/conformer 5**

| Atomic N. | X | Y | Z |
|---|---|---|---|
| 7 | 0.20486821 | 2.38812184 | 0.44920191 |
| 8 | -2.47105694 | 1.37000120 | -0.17834166 |
| 8 | 0.03036767 | 0.95534188 | -2.15405107 |
| 6 | 0.63764334 | 2.17388654 | 1.85011733 |
| 6 | -2.87185931 | 2.21660447 | 0.79028827 |
| 6 | -4.15231657 | 2.06192517 | 1.40299273 |
| 6 | -4.49097729 | 2.97101808 | 2.41589165 |
| 6 | -3.64480782 | 4.00669098 | 2.82551670 |
| 6 | -2.41642094 | 4.17008257 | 2.19309115 |
| 6 | -2.03491855 | 3.29307032 | 1.17309165 |
| 6 | -0.77871889 | 3.52729130 | 0.39102441 |
| 6 | -5.13274813 | 0.95908523 | 0.95976567 |
| 6 | 0.44114250 | 2.13754201 | -2.65875220 |
| 6 | 0.24480180 | 2.42397857 | -4.04304075 |
| 6 | 0.70706695 | 3.66006303 | -4.51541710 |
| 6 | 1.33760905 | 4.59975624 | -3.69362974 |
| 6 | 1.52993202 | 4.30530119 | -2.34870553 |
| 6 | 1.09865224 | 3.07955027 | -1.82772267 |
| 6 | 1.40391040 | 2.73876023 | -0.39436662 |
| 6 | -0.44944161 | 1.41801071 | -4.98222733 |
| 6 | -6.45972013 | 1.02023721 | 1.73901021 |
| 6 | -4.51521683 | -0.43841639 | 1.19902992 |
| 6 | -5.46715117 | 1.12891138 | -0.54161978 |
| 6 | -0.52343488 | 1.94165313 | -6.42900562 |
| 6 | -1.90017438 | 1.15992546 | -4.51016426 |
| 6 | 0.33774242 | 0.08593988 | -5.01266146 |
| 1 | 1.09133244 | 3.09694123 | 2.24942636 |
| 1 | -0.22774269 | 1.90947688 | 2.46713090 |
| 1 | -0.26637748 | 4.43672132 | 0.75119883 |
| 1 | -1.01013446 | 3.67368174 | -0.67380768 |
| 1 | -5.45564461 | 2.87225056 | 2.91074443 |
| 1 | -1.75175238 | 4.99155378 | 2.47123098 |
| 1 | 2.08263969 | 1.87457848 | -0.34045160 |
| 1 | 1.91588581 | 3.59240770 | 0.08345279 |
| 1 | 0.56618714 | 3.90955257 | -5.56557989 |
| 1 | 2.02685976 | 5.02141809 | -1.68993926 |
| 1 | -7.12462234 | 0.22463913 | 1.37341166 |
| 1 | -6.31547499 | 0.86070973 | 2.81773901 |
| 1 | -6.98041201 | 1.97813475 | 1.59574306 |
| 1 | -3.60849595 | -0.58052850 | 0.60091937 |
| 1 | -4.27132177 | -0.57599306 | 2.26370263 |
| 1 | -5.24099302 | -1.21691668 | 0.91652870 |
| 1 | -5.94282103 | 2.10367155 | -0.72406775 |

| | | | |
|---|---|---|---|
| 1 | -4.56687069 | 1.05602980 | -1.16157389 |
| 1 | -6.17173290 | 0.34408775 | -0.85692430 |
| 1 | -1.01637614 | 1.18556893 | -7.05651426 |
| 1 | 0.47389087 | 2.12586808 | -6.85449553 |
| 1 | -1.11142755 | 2.86787152 | -6.50564241 |
| 1 | -1.92844546 | 0.71235508 | -3.51141047 |
| 1 | -2.47533250 | 2.09706664 | -4.49112988 |
| 1 | -2.39908099 | 0.47068742 | -5.20836926 |
| 1 | 1.36118567 | 0.24944295 | -5.38174963 |
| 1 | 0.38969305 | -0.37162450 | -4.01895094 |
| 1 | -0.15827860 | -0.62065411 | -5.69557333 |
| 1 | 1.37066174 | 1.36251938 | 1.89244986 |
| 8 | -1.45705497 | -1.10272396 | -0.69900787 |
| 8 | 0.12498878 | -2.65282559 | -1.00842762 |
| 6 | -0.61866188 | -1.92007434 | -0.11008233 |
| 6 | -1.12584496 | -2.62684631 | 1.14435697 |
| 1 | -0.29332304 | -3.02564216 | 1.74228597 |
| 1 | -1.60637486 | -1.84451222 | 1.74412727 |
| 6 | -2.13276315 | -3.74673176 | 0.82052547 |
| 1 | -2.79762769 | -3.41272616 | 0.00889488 |
| 1 | -2.77345347 | -3.90088224 | 1.70168889 |
| 6 | -1.46382082 | -5.07980728 | 0.46240312 |
| 1 | -0.87578458 | -5.42270327 | 1.33195353 |
| 1 | -2.23901439 | -5.84411144 | 0.29621750 |
| 6 | -0.54327524 | -5.02888680 | -0.76287550 |
| 1 | -1.12239051 | -4.82736921 | -1.67812383 |
| 1 | -0.07157925 | -6.01605177 | -0.90032864 |
| 6 | 0.57499731 | -3.99189329 | -0.67979085 |
| 1 | 1.06993759 | -4.01156759 | 0.30529699 |
| 1 | 1.33768046 | -4.19488096 | -1.44330168 |
| 31 | -0.73693907 | 0.68484360 | -0.47525138 |
| 1 | -3.95513558 | 4.68406677 | 3.62115216 |
| 1 | 1.67207325 | 5.55129051 | -4.10684252 |
| 8 | 8.10977650 | -3.03229952 | 3.93337083 |
| 6 | 8.79728031 | -3.62632823 | 5.06141376 |
| 1 | 8.55126381 | -3.08969450 | 5.98619843 |
| 6 | 6.12408829 | -2.42121005 | 2.79317594 |
| 6 | 4.60655785 | -2.27185607 | 2.89582348 |
| 6 | 3.98953104 | -1.67341280 | 1.62731731 |
| 6 | 6.74857903 | -3.04354358 | 4.02780151 |
| 6 | 2.46887732 | -1.50375485 | 1.72578228 |
| 6 | 1.87182176 | -0.92214715 | 0.44405147 |
| 1 | 6.61257315 | -1.44998884 | 2.61556125 |
| 1 | 4.36122036 | -1.64060569 | 3.76399803 |
| 1 | 4.45422173 | -0.69360280 | 1.41942418 |
| 1 | 2.21776915 | -0.84799582 | 2.57568192 |
| 1 | 6.40049648 | -3.05044675 | 1.93055737 |
| 1 | 4.15907145 | -3.25558114 | 3.10373616 |
| 1 | 4.23229074 | -2.31536269 | 0.76261920 |
| 1 | 2.00367975 | -2.47909021 | 1.94184995 |
| 1 | 2.06632161 | -1.58099210 | -0.41895238 |
| 1 | 2.36020350 | 0.03772115 | 0.21299741 |
| 8 | 6.14454556 | -3.50191569 | 4.97895813 |

| | | | |
|---|---|---|---|
| 8 | 0.45820418 | -0.69876879 | 0.54308522 |
| 1 | 9.86298180 | -3.53491449 | 4.83116817 |
| 1 | 8.51223755 | -4.68002510 | 5.17269802 |

Electronic energy = -3908.650363 a.u.
DFT-D3(BJ) dispersion correction = -0.137161 a.u.
Thermal free energy = 0.744682 a.u.
Gibbs free energy = -3908.042842 a.u.
Number of imaginary frequencies = 1.

**TS3 ax CL isomer/conformer 6**

| Atomic N. | X | Y | Z |
|---|---|---|---|
| 7 | -0.11298153 | 2.34507823 | 0.76556253 |
| 8 | 1.65957546 | 0.56218696 | -0.75276995 |
| 8 | 0.86147141 | -0.01504125 | 2.29905438 |
| 6 | -1.31458092 | 2.84399390 | 0.05889585 |
| 6 | 1.71968818 | 1.58799076 | -1.62265921 |
| 6 | 2.16065645 | 1.37978017 | -2.96490026 |
| 6 | 2.16886568 | 2.49654579 | -3.81392813 |
| 6 | 1.78596330 | 3.77597380 | -3.39622235 |
| 6 | 1.40294600 | 3.97324133 | -2.07264900 |
| 6 | 1.38372743 | 2.89309096 | -1.18486440 |
| 6 | 1.09924030 | 3.09416533 | 0.27299377 |
| 6 | 2.63066363 | -0.00301790 | -3.45661020 |
| 6 | 1.41661310 | 0.92064857 | 3.09851837 |
| 6 | 2.47929692 | 0.56367540 | 3.98069143 |
| 6 | 3.00341201 | 1.57726049 | 4.79497910 |
| 6 | 2.52961373 | 2.89308190 | 4.77118301 |
| 6 | 1.48531163 | 3.22438097 | 3.91546655 |
| 6 | 0.91803426 | 2.24674559 | 3.08952355 |
| 6 | -0.27870390 | 2.58926177 | 2.24665022 |
| 6 | 3.03063536 | -0.87446040 | 4.03709602 |
| 6 | 3.11380553 | 0.04031212 | -4.91835785 |
| 6 | 1.47539401 | -1.02843332 | -3.37777638 |
| 6 | 3.81761146 | -0.48810604 | -2.59002781 |
| 6 | 4.15192938 | -1.01742673 | 5.08337879 |
| 6 | 3.62507701 | -1.27281189 | 2.66528344 |
| 6 | 1.90424299 | -1.86137342 | 4.42764568 |
| 1 | -1.44752598 | 3.92122579 | 0.25743395 |
| 1 | -1.19251549 | 2.69827199 | -1.01902974 |
| 1 | 0.94827586 | 4.16534185 | 0.49326459 |
| 1 | 1.94355488 | 2.74165082 | 0.88216430 |
| 1 | 2.48983240 | 2.36978245 | -4.84657574 |
| 1 | 1.13905013 | 4.97049570 | -1.71239853 |
| 1 | -1.15080595 | 1.99549687 | 2.55912185 |
| 1 | -0.53223640 | 3.65466475 | 2.38485932 |
| 1 | 3.81984377 | 1.33848894 | 5.47419500 |
| 1 | 1.09175479 | 4.24320316 | 3.88855720 |
| 1 | 3.45406485 | -0.96317941 | -5.21083021 |
| 1 | 2.31183147 | 0.33081201 | -5.61291885 |
| 1 | 3.95980620 | 0.72944337 | -5.05507803 |
| 1 | 1.11984754 | -1.15847480 | -2.34969497 |
| 1 | 0.63371187 | -0.71659905 | -4.01486063 |

| | | | |
|---|---|---|---|
| 1 | 1.82455850 | -2.00539589 | -3.74613571 |
| 1 | 4.66139984 | 0.21381596 | -2.66133237 |
| 1 | 3.53090310 | -0.58638668 | -1.53734684 |
| 1 | 4.16248226 | -1.46937191 | -2.94991922 |
| 1 | 4.50106955 | -2.05971408 | 5.09122038 |
| 1 | 3.80419993 | -0.77731287 | 6.09876013 |
| 1 | 5.01873112 | -0.38127449 | 4.85231447 |
| 1 | 2.86569023 | -1.26817799 | 1.87656903 |
| 1 | 4.43406725 | -0.58577651 | 2.37746906 |
| 1 | 4.04834461 | -2.28697014 | 2.72617054 |
| 1 | 1.49569619 | -1.61060548 | 5.41788626 |
| 1 | 1.08903360 | -1.84954083 | 3.69613981 |
| 1 | 2.31060624 | -2.88305402 | 4.47961521 |
| 1 | -2.20182467 | 2.30300546 | 0.39946210 |
| 8 | 0.11627407 | -1.56462467 | -0.07319789 |
| 8 | -1.27315187 | -2.60207176 | 1.34962773 |
| 6 | -1.13150001 | -1.92117214 | 0.15747061 |
| 6 | -1.88782942 | -2.44378495 | -1.06024623 |
| 1 | -2.97391605 | -2.44921184 | -0.89071310 |
| 1 | -1.68591285 | -1.72147143 | -1.86018026 |
| 6 | -1.41193795 | -3.84343648 | -1.49475384 |
| 1 | -0.31535935 | -3.89395952 | -1.41118860 |
| 1 | -1.64942789 | -3.96689057 | -2.56170344 |
| 6 | -2.07036686 | -4.98611641 | -0.71226728 |
| 1 | -3.15975070 | -4.95279026 | -0.88898373 |
| 1 | -1.72418725 | -5.94974041 | -1.11731148 |
| 6 | -1.81793940 | -4.95918369 | 0.79956937 |
| 1 | -0.75184333 | -5.12539673 | 1.02197957 |
| 1 | -2.37109351 | -5.78765059 | 1.27238238 |
| 6 | -2.24660254 | -3.66730666 | 1.49134672 |
| 1 | -3.24269772 | -3.34151125 | 1.14915919 |
| 1 | -2.29757881 | -3.81642032 | 2.57803917 |
| 31 | 0.31541461 | 0.24367216 | 0.53553087 |
| 1 | 1.81028509 | 4.60992718 | -4.09777880 |
| 1 | 2.97646904 | 3.64702439 | 5.41911411 |
| 8 | -2.75653267 | 0.72840458 | -4.42082930 |
| 6 | -1.75911760 | 1.66350853 | -3.96199036 |
| 1 | -1.09574687 | 1.82537436 | -4.81696844 |
| 6 | -3.69945383 | 0.75684321 | -2.12564421 |
| 6 | -4.92431164 | 0.27808201 | -1.34323645 |
| 6 | -4.91627550 | 0.58321768 | 0.16702859 |
| 6 | -3.72161126 | 0.26379672 | -3.56523204 |
| 6 | -4.20305681 | -0.43727243 | 1.08408725 |
| 6 | -2.74477577 | -0.18843085 | 1.48329997 |
| 1 | -2.76628160 | 0.41223133 | -1.64925349 |
| 1 | -5.05120039 | -0.80311614 | -1.50056648 |
| 1 | -4.52084351 | 1.59718621 | 0.35362795 |
| 1 | -4.31147766 | -1.44719720 | 0.65766698 |
| 1 | -3.64853120 | 1.85813999 | -2.11927676 |
| 1 | -5.81469488 | 0.73934036 | -1.79596901 |
| 1 | -5.96814013 | 0.62134820 | 0.48922929 |
| 1 | -4.74496603 | -0.47038010 | 2.04495621 |
| 1 | -2.46895862 | -0.86023390 | 2.31229162 |

| | | | |
|---|---|---|---|
| 1 | -2.65802121 | 0.83995545 | 1.86841011 |
| 8 | -4.53462791 | -0.52709544 | -3.99779272 |
| 8 | -1.76660740 | -0.33658734 | 0.43711823 |
| 1 | -1.17059135 | 1.25226271 | -3.13080406 |
| 1 | -2.21248055 | 2.62213063 | -3.67128754 |

Electronic energy = -3908.633149 a.u.
DFT-D3(BJ) dispersion correction = -0.146442 a.u.
Thermal free energy = 0.744692 a.u.
Gibbs free energy = -3908.034900 a.u.
Number of imaginary frequencies = 1.

**Int3 CL isomer/conformer 1**

| Atomic N. | X | Y | Z |
|---|---|---|---|
| 7 | -0.62754880 | 0.62967964 | 1.55749045 |
| 8 | -2.08812431 | 1.13354759 | -0.99761479 |
| 8 | 0.79951094 | 2.10478444 | -0.64377544 |
| 6 | -0.67680995 | -0.71399709 | 2.18871569 |
| 6 | -3.15932785 | 0.71047383 | -0.28435302 |
| 6 | -4.34058874 | 0.26547136 | -0.94664214 |
| 6 | -5.40638537 | -0.15676793 | -0.13840942 |
| 6 | -5.35203684 | -0.14892093 | 1.25891162 |
| 6 | -4.20329712 | 0.31303891 | 1.89296692 |
| 6 | -3.11454485 | 0.75448148 | 1.13357139 |
| 6 | -1.92074254 | 1.37245759 | 1.80596174 |
| 6 | -4.44732677 | 0.25013025 | -2.48388022 |
| 6 | 0.80699996 | 3.11238266 | 0.26495597 |
| 6 | 0.98640005 | 4.46095162 | -0.16429638 |
| 6 | 1.01133122 | 5.45189146 | 0.82752152 |
| 6 | 0.86135659 | 5.16999037 | 2.18820161 |
| 6 | 0.68241257 | 3.85234709 | 2.59245690 |
| 6 | 0.66462210 | 2.82052352 | 1.64636095 |
| 6 | 0.55001571 | 1.39575212 | 2.12133246 |
| 6 | 1.13914138 | 4.82721973 | -1.65418121 |
| 6 | -5.83607329 | -0.21711372 | -2.95938839 |
| 6 | -3.40299182 | -0.72213205 | -3.08117051 |
| 6 | -4.22546006 | 1.67515703 | -3.04507181 |
| 6 | 1.32188597 | 6.34332998 | -1.85857719 |
| 6 | -0.12686097 | 4.40937002 | -2.43906304 |
| 6 | 2.38586121 | 4.13165041 | -2.24896316 |
| 1 | -0.80286222 | -0.60546483 | 3.27762250 |
| 1 | -1.52627986 | -1.27492356 | 1.78403345 |
| 1 | -2.07582395 | 1.42443706 | 2.89635074 |
| 1 | -1.75739530 | 2.39529409 | 1.43748206 |
| 1 | -6.31920869 | -0.51112268 | -0.61407716 |
| 1 | -4.14856371 | 0.35439289 | 2.98334904 |
| 1 | 1.43733786 | 0.80894306 | 1.84006928 |
| 1 | 0.46516088 | 1.37882653 | 3.22006304 |
| 1 | 1.14463027 | 6.49048311 | 0.53091523 |
| 1 | 0.56523372 | 3.60815664 | 3.65066428 |
| 1 | -5.86328678 | -0.19118129 | -4.05808088 |
| 1 | -6.05694481 | -1.24898932 | -2.64926747 |
| 1 | -6.64069482 | 0.43684862 | -2.59284199 |

| | | | |
|---|---|---|---|
| 1 | -2.37811914 | -0.43537683 | -2.82311031 |
| 1 | -3.57804114 | -1.74609328 | -2.71892961 |
| 1 | -3.49266965 | -0.73373544 | -4.17830496 |
| 1 | -4.98829907 | 2.36650580 | -2.65779128 |
| 1 | -3.23639875 | 2.06365171 | -2.77978092 |
| 1 | -4.31097022 | 1.65824255 | -4.14230614 |
| 1 | 1.42967775 | 6.54640347 | -2.93350969 |
| 1 | 2.22438972 | 6.72377346 | -1.35848472 |
| 1 | 0.45556476 | 6.91769056 | -1.49987060 |
| 1 | -0.30156774 | 3.32974016 | -2.38872766 |
| 1 | -1.01532126 | 4.92165866 | -2.04260997 |
| 1 | -0.01687296 | 4.69210582 | -3.49699856 |
| 1 | 3.29711060 | 4.45288518 | -1.72328433 |
| 1 | 2.30836553 | 3.04184767 | -2.17824316 |
| 1 | 2.49325798 | 4.40402196 | -3.30991388 |
| 1 | 0.25563365 | -1.24703090 | 1.97918995 |
| 8 | 0.04335254 | -0.78088235 | -1.54850984 |
| 8 | 1.65781600 | -1.12783041 | -0.02938588 |
| 6 | 1.19839182 | -1.51556430 | -1.35704918 |
| 6 | 2.24461080 | -1.28556327 | -2.46653355 |
| 1 | 2.87362180 | -2.18601176 | -2.53839713 |
| 1 | 1.66122740 | -1.23867163 | -3.39618247 |
| 6 | 3.12640403 | -0.03949052 | -2.31863483 |
| 1 | 2.52722141 | 0.80839695 | -1.95245853 |
| 1 | 3.48499058 | 0.24598562 | -3.31972346 |
| 6 | 4.34960609 | -0.25510001 | -1.41423313 |
| 1 | 4.91056713 | -1.13049489 | -1.78656257 |
| 1 | 5.02835631 | 0.60727968 | -1.50875193 |
| 6 | 4.02683687 | -0.46197724 | 0.07035764 |
| 1 | 3.66146088 | 0.48150189 | 0.50778633 |
| 1 | 4.94981554 | -0.72300380 | 0.61570076 |
| 6 | 2.98739536 | -1.54406767 | 0.35412008 |
| 1 | 3.23356351 | -2.49402595 | -0.14807466 |
| 1 | 2.93517290 | -1.74612295 | 1.43540387 |
| 31 | -0.35770494 | 0.65713062 | -0.48316150 |
| 1 | -6.20729371 | -0.49201641 | 1.84108591 |
| 1 | 0.88114063 | 5.97745254 | 2.91997899 |
| 8 | 2.85200631 | -5.20575090 | 4.88005393 |
| 6 | 3.08303124 | -4.50598783 | 6.12740851 |
| 1 | 3.77676450 | -3.66969668 | 5.97505570 |
| 6 | 2.16721215 | -5.23539312 | 2.60428301 |
| 6 | 0.99209673 | -4.75819313 | 1.74629864 |
| 6 | 1.01212620 | -5.33572834 | 0.32802753 |
| 6 | 2.31292774 | -4.44221396 | 3.88856124 |
| 6 | -0.16293730 | -4.86849565 | -0.54545594 |
| 6 | -0.25600718 | -3.35750528 | -0.73517594 |
| 1 | 3.11154666 | -5.09696271 | 2.04808071 |
| 1 | 1.02683056 | -3.66145074 | 1.70655431 |
| 1 | 1.95611900 | -5.05249013 | -0.16433473 |
| 1 | -1.11570895 | -5.20507018 | -0.10225263 |
| 1 | 2.10361228 | -6.30648433 | 2.84082623 |
| 1 | 0.04469017 | -5.02174998 | 2.24590734 |
| 1 | 1.00290406 | -6.43735394 | 0.37449590 |

| | | | |
|---|---|---|---|
| 1 | -0.09120906 | -5.34465817 | -1.53578827 |
| 1 | -0.35689131 | -2.84914092 | 0.23617939 |
| 1 | -1.13666733 | -3.09867312 | -1.34262735 |
| 8 | 2.01990351 | -3.26758255 | 4.03380721 |
| 8 | 0.93836205 | -2.91274882 | -1.40421436 |
| 1 | 3.51575332 | -5.25174023 | 6.80089195 |
| 1 | 2.13852068 | -4.11954368 | 6.52997594 |

Electronic energy = -3908.668252 a.u.
DFT-D3(BJ) dispersion correction = -0.140833 a.u.
Thermal free energy = 0.740745 a.u.
Gibbs free energy = -3908.068340 a.u.
Number of imaginary frequencies = 0.

**Int3 CL isomer/conformer 2**

| Atomic N. | X | Y | Z |
|---|---|---|---|
| 7 | 0.44234494 | 2.08917276 | 1.07597186 |
| 8 | -2.26228403 | 1.47560894 | -0.01712316 |
| 8 | 0.21507520 | 1.22249077 | -1.80589499 |
| 6 | 0.89291805 | 1.44177289 | 2.33397928 |
| 6 | -2.68057007 | 1.94253993 | 1.18634468 |
| 6 | -3.98556231 | 1.63287129 | 1.66789029 |
| 6 | -4.34776141 | 2.15513812 | 2.91830329 |
| 6 | -3.49373586 | 2.95362759 | 3.68486539 |
| 6 | -2.23145723 | 3.26871821 | 3.19232663 |
| 6 | -1.82364361 | 2.78242044 | 1.94577762 |
| 6 | -0.51072626 | 3.22304882 | 1.35644668 |
| 6 | -4.96217863 | 0.76444519 | 0.85090617 |
| 6 | 0.64807248 | 2.48524785 | -2.04409132 |
| 6 | 0.45966651 | 3.06367934 | -3.33353925 |
| 6 | 0.94143743 | 4.36547631 | -3.53019375 |
| 6 | 1.58278807 | 5.09691448 | -2.52610844 |
| 6 | 1.77136549 | 4.51627831 | -1.27711813 |
| 6 | 1.32323669 | 3.21315643 | -1.02918568 |
| 6 | 1.64254443 | 2.56490137 | 0.29356473 |
| 6 | -0.24620545 | 2.29333395 | -4.46710120 |
| 6 | -6.32304264 | 0.61092297 | 1.55606692 |
| 6 | -4.37995317 | -0.65548676 | 0.65733583 |
| 6 | -5.22881179 | 1.41903069 | -0.52569578 |
| 6 | -0.29232350 | 3.10922750 | -5.77293298 |
| 6 | -1.70804682 | 1.98133968 | -4.06809780 |
| 6 | 0.51081109 | 0.97830535 | -4.77235693 |
| 1 | 1.46509505 | 2.16341276 | 2.93977233 |
| 1 | 0.01923439 | 1.10306711 | 2.90096568 |
| 1 | -0.00797604 | 3.93664821 | 2.03085567 |
| 1 | -0.67173770 | 3.72347672 | 0.39039057 |
| 1 | -5.33479614 | 1.92915305 | 3.31796450 |
| 1 | -1.55762068 | 3.91244392 | 3.76262068 |
| 1 | 2.26162496 | 1.66613116 | 0.14664220 |
| 1 | 2.20667540 | 3.27032648 | 0.92646893 |
| 1 | 0.80758577 | 4.83618106 | -4.50246201 |
| 1 | 2.28278656 | 5.06490320 | -0.48283077 |
| 1 | -6.98364660 | -0.00174471 | 0.92611252 |

| | | | |
|---|---|---|---|
| 1 | -6.23278248 | 0.10527937 | 2.52849430 |
| 1 | -6.81955673 | 1.57971926 | 1.71184906 |
| 1 | -3.42688906 | -0.63601619 | 0.11846505 |
| 1 | -4.21552387 | -1.14254997 | 1.62963232 |
| 1 | -5.09008736 | -1.27202062 | 0.08485553 |
| 1 | -5.68366758 | 2.41252104 | -0.39970266 |
| 1 | -4.30567992 | 1.52953009 | -1.10469471 |
| 1 | -5.93078275 | 0.79719045 | -1.10208819 |
| 1 | -0.79249398 | 2.51190823 | -6.54846587 |
| 1 | 0.71356351 | 3.35628811 | -6.14257773 |
| 1 | -0.86117109 | 4.04341524 | -5.65903315 |
| 1 | -1.75996508 | 1.35326283 | -3.17275012 |
| 1 | -2.26380488 | 2.90981108 | -3.87295408 |
| 1 | -2.21159110 | 1.45127614 | -4.89089298 |
| 1 | 1.54127341 | 1.19098105 | -5.09350630 |
| 1 | 0.54478476 | 0.32051328 | -3.89709672 |
| 1 | 0.00631011 | 0.44424402 | -5.59212774 |
| 1 | 1.51851461 | 0.57644277 | 2.08689073 |
| 8 | -0.51372658 | -0.91252261 | 0.52544836 |
| 8 | 0.09549478 | -1.99903155 | -1.38166311 |
| 6 | 0.40069510 | -1.80168574 | 0.00094235 |
| 6 | 0.43323351 | -3.06020429 | 0.88007369 |
| 1 | 1.23283070 | -3.73720899 | 0.54163966 |
| 1 | 0.74300559 | -2.69827201 | 1.86898923 |
| 6 | -0.89824160 | -3.81716234 | 0.99539550 |
| 1 | -1.71433463 | -3.08766356 | 1.11031335 |
| 1 | -0.87394664 | -4.40793380 | 1.92417427 |
| 6 | -1.18871793 | -4.76461992 | -0.17392307 |
| 1 | -0.39652156 | -5.53339850 | -0.21391233 |
| 1 | -2.12940619 | -5.30421566 | 0.02088553 |
| 6 | -1.27456686 | -4.07971340 | -1.54134154 |
| 1 | -2.12356510 | -3.37806730 | -1.57453339 |
| 1 | -1.46369778 | -4.84290973 | -2.31529308 |
| 6 | -0.01168324 | -3.31625309 | -1.94514586 |
| 1 | 0.89007454 | -3.91700547 | -1.72720228 |
| 1 | -0.02264044 | -3.12634922 | -3.02803714 |
| 31 | -0.53644598 | 0.78579641 | -0.17123418 |
| 1 | -3.82121276 | 3.33054505 | 4.65381205 |
| 1 | 1.93183509 | 6.11007783 | -2.72522954 |
| 8 | 4.78212732 | -3.52309121 | 4.69699936 |
| 6 | 3.76109055 | -3.82869611 | 5.67816198 |
| 1 | 2.89838257 | -3.16253293 | 5.55319140 |
| 6 | 5.53648386 | -3.40314475 | 2.44728801 |
| 6 | 5.07745170 | -2.94105817 | 1.06075804 |
| 6 | 4.54584740 | -1.50214910 | 1.04964749 |
| 6 | 4.38450944 | -3.66704949 | 3.40003650 |
| 6 | 4.06079462 | -1.03326759 | -0.33106389 |
| 6 | 2.72872296 | -1.62885332 | -0.77889328 |
| 1 | 6.22794393 | -2.68388014 | 2.90969096 |
| 1 | 4.30572236 | -3.63759698 | 0.70140130 |
| 1 | 3.71841974 | -1.40661047 | 1.77029196 |
| 1 | 4.81607170 | -1.28013400 | -1.09652956 |
| 1 | 6.08984672 | -4.35428187 | 2.36757153 |

| | | | |
|---|---|---|---|
| 1 | 5.92862923 | -3.01926735 | 0.36523892 |
| 1 | 5.34690063 | -0.82805498 | 1.39628708 |
| 1 | 3.96488638 | 0.06428434 | -0.33938622 |
| 1 | 2.76614500 | -2.73114884 | -0.72253690 |
| 1 | 2.51546895 | -1.35089215 | -1.82270615 |
| 8 | 3.25745993 | -3.99590759 | 3.07787677 |
| 8 | 1.69187839 | -1.12046167 | 0.07751201 |
| 1 | 4.23745271 | -3.66958463 | 6.65018992 |
| 1 | 3.42766876 | -4.86880062 | 5.57367729 |

Electronic energy = -3908.663223 a.u.
DFT-D3(BJ) dispersion correction = -0.137972 a.u.
Thermal free energy = 0.737673 a.u.
Gibbs free energy = -3908.063522 a.u.
Number of imaginary frequencies = 0.

**Int3 CL isomer/conformer 3**

| Atomic N. | X | Y | Z |
|---|---|---|---|
| 7 | -1.04224385 | -0.42047490 | 1.93966485 |
| 8 | -1.30673947 | 0.93047580 | -0.71227590 |
| 8 | 0.66314298 | 1.98634163 | 1.31594718 |
| 6 | -0.92350999 | -1.90037947 | 1.98022225 |
| 6 | -2.44236339 | 0.19922535 | -0.80772778 |
| 6 | -3.07327737 | 0.02424995 | -2.07430434 |
| 6 | -4.25622310 | -0.72870419 | -2.10324379 |
| 6 | -4.81848141 | -1.30288783 | -0.95916326 |
| 6 | -4.20185839 | -1.10972638 | 0.27265587 |
| 6 | -3.03120563 | -0.34982855 | 0.36203638 |
| 6 | -2.47316878 | 0.00125027 | 1.71620365 |
| 6 | -2.48957001 | 0.64833741 | -3.35747778 |
| 6 | -0.02524479 | 2.51700523 | 2.35835995 |
| 6 | -0.12189120 | 3.93382867 | 2.50270134 |
| 6 | -0.81142303 | 4.42096473 | 3.62220950 |
| 6 | -1.40384175 | 3.58639504 | 4.57362355 |
| 6 | -1.31400031 | 2.20881694 | 4.41538110 |
| 6 | -0.62469128 | 1.66590801 | 3.32443004 |
| 6 | -0.49239167 | 0.16880347 | 3.21995580 |
| 6 | 0.49426326 | 4.90250412 | 1.47327240 |
| 6 | -3.36058701 | 0.34621973 | -4.59154569 |
| 6 | -1.07894082 | 0.07883687 | -3.63908009 |
| 6 | -2.41789344 | 2.18742960 | -3.21222697 |
| 6 | 0.24427405 | 6.37662307 | 1.84496587 |
| 6 | -0.13525444 | 4.67081396 | 0.07931731 |
| 6 | 2.02562828 | 4.70134356 | 1.40542252 |
| 1 | -1.53916487 | -2.29751251 | 2.80350718 |
| 1 | -1.25850958 | -2.33768768 | 1.03327231 |
| 1 | -3.08977487 | -0.45263279 | 2.50995444 |
| 1 | -2.48839375 | 1.09139227 | 1.86034879 |
| 1 | -4.76195302 | -0.87974497 | -3.05568987 |
| 1 | -4.63768763 | -1.52923831 | 1.18217099 |
| 1 | 0.56501581 | -0.13975070 | 3.25184149 |
| 1 | -1.01282413 | -0.30827525 | 4.06665914 |
| 1 | -0.90113908 | 5.49704212 | 3.75771421 |

| | | | |
|---|---|---|---|
| 1 | -1.77221185 | 1.53644060 | 5.14415432 |
| 1 | -2.90555049 | 0.81859656 | -5.47375836 |
| 1 | -3.43196095 | -0.73231922 | -4.79560068 |
| 1 | -4.37868888 | 0.74889196 | -4.48876371 |
| 1 | -0.38668126 | 0.27621815 | -2.81402851 |
| 1 | -1.12258110 | -1.00876232 | -3.79759412 |
| 1 | -0.67066598 | 0.53560301 | -4.55364714 |
| 1 | -3.42199930 | 2.60822797 | -3.05595119 |
| 1 | -1.78187371 | 2.48347962 | -2.37113304 |
| 1 | -2.00674945 | 2.62827586 | -4.13328837 |
| 1 | 0.70447568 | 7.01842626 | 1.08028794 |
| 1 | 0.69307102 | 6.64290305 | 2.81291666 |
| 1 | -0.82751558 | 6.61991498 | 1.87821673 |
| 1 | 0.03861011 | 3.65300321 | -0.28437681 |
| 1 | -1.22068060 | 4.84329200 | 0.11108030 |
| 1 | 0.29843468 | 5.37666900 | -0.64520807 |
| 1 | 2.48892182 | 4.91705575 | 2.37941016 |
| 1 | 2.28042424 | 3.67610609 | 1.11910386 |
| 1 | 2.46013367 | 5.38938228 | 0.66430383 |
| 1 | 0.12600370 | -2.16681044 | 2.14819286 |
| 8 | 1.16053000 | -0.73959349 | -0.39524910 |
| 8 | 2.73151032 | -0.41426716 | 1.18102030 |
| 6 | 2.53676051 | -0.81117829 | -0.18659617 |
| 6 | 3.32052129 | 0.03291439 | -1.21887993 |
| 1 | 4.31433153 | -0.41978600 | -1.35207218 |
| 1 | 2.78421188 | -0.10378795 | -2.16761053 |
| 6 | 3.47406574 | 1.52390363 | -0.89468861 |
| 1 | 2.53531177 | 1.93377142 | -0.48938018 |
| 1 | 3.65630916 | 2.06112808 | -1.83848256 |
| 6 | 4.63782420 | 1.82870878 | 0.06244906 |
| 1 | 5.55954200 | 1.39735786 | -0.36626083 |
| 1 | 4.79850341 | 2.91780420 | 0.10279442 |
| 6 | 4.45855823 | 1.30762601 | 1.49387325 |
| 1 | 3.66994367 | 1.87914334 | 2.00784333 |
| 1 | 5.39052661 | 1.47083487 | 2.06161412 |
| 6 | 4.09315328 | -0.17084310 | 1.59409384 |
| 1 | 4.78077267 | -0.80805268 | 1.01299869 |
| 1 | 4.14368987 | -0.49964849 | 2.64349955 |
| 31 | 0.06235707 | 0.44839217 | 0.45769307 |
| 8 | 3.00418664 | -2.13678268 | -0.40873534 |
| 1 | -5.73574864 | -1.88723832 | -1.03407589 |
| 1 | -1.93457074 | 4.01472708 | 5.42372513 |
| 8 | -1.82134952 | -5.16157792 | -2.56939645 |
| 6 | -2.81378727 | -4.11814939 | -2.75433896 |
| 1 | -3.64173043 | -4.24982724 | -2.04711647 |
| 6 | -0.23755789 | -6.30676110 | -1.21200798 |
| 6 | 0.95594158 | -5.94529076 | -0.32166249 |
| 6 | 2.02872899 | -5.10608432 | -1.03183712 |
| 6 | -1.21630062 | -5.15473442 | -1.35323600 |
| 6 | 3.02949257 | -4.49464965 | -0.03644949 |
| 6 | 2.57168632 | -3.14670788 | 0.52228109 |
| 1 | -0.80960126 | -7.13610102 | -0.76254647 |
| 1 | 1.41873888 | -6.86957103 | 0.05878477 |

| | | | |
|---|---|---|---|
| 1 | 2.55277833 | -5.74049731 | -1.76439807 |
| 1 | 4.01127680 | -4.34057804 | -0.50860275 |
| 1 | 0.07712953 | -6.64644172 | -2.20869899 |
| 1 | 0.57182265 | -5.40113789 | 0.55514336 |
| 1 | 1.56088307 | -4.29102018 | -1.60787478 |
| 1 | 3.19058949 | -5.19544468 | 0.79968856 |
| 1 | 3.02219488 | -2.95288725 | 1.50987368 |
| 1 | 1.47474206 | -3.12000409 | 0.62446768 |
| 8 | -1.46385262 | -4.33642074 | -0.48347464 |
| 1 | -3.16057566 | -4.23144214 | -3.78593500 |
| 1 | -2.36453243 | -3.13136353 | -2.59565622 |

Electronic energy = -3908.663104 a.u.
DFT-D3(BJ) dispersion correction = -0.142362 a.u.
Thermal free energy = 0.742476 a.u.
Gibbs free energy = -3908.062991 a.u.
Number of imaginary frequencies = 0.

**Int3 CL isomer/conformer 4**

| Atomic N. | X | Y | Z |
|---|---|---|---|
| 7 | -0.61653098 | 2.23459598 | 0.89877465 |
| 8 | -2.99020839 | 0.47861113 | 0.46865193 |
| 8 | -0.58551851 | 0.30908352 | -1.42523465 |
| 6 | 0.00918044 | 2.28802709 | 2.24470091 |
| 6 | -3.53590594 | 1.24573066 | 1.44585051 |
| 6 | -4.68020604 | 0.79263022 | 2.16480825 |
| 6 | -5.19064568 | 1.64074227 | 3.15860000 |
| 6 | -4.63044758 | 2.88670254 | 3.45542455 |
| 6 | -3.52929520 | 3.32829672 | 2.72929284 |
| 6 | -2.98706704 | 2.52645910 | 1.71939066 |
| 6 | -1.87885939 | 3.05756195 | 0.85071222 |
| 6 | -5.33468801 | -0.56996833 | 1.86420572 |
| 6 | -0.55292488 | 1.45184331 | -2.15406711 |
| 6 | -0.90831161 | 1.41505380 | -3.53452816 |
| 6 | -0.84442972 | 2.62061616 | -4.24706530 |
| 6 | -0.45477546 | 3.82841866 | -3.66067652 |
| 6 | -0.09782250 | 3.84764832 | -2.31726596 |
| 6 | -0.12845359 | 2.66984058 | -1.56047924 |
| 6 | 0.37650048 | 2.69827301 | -0.14015400 |
| 6 | -1.34652155 | 0.10494876 | -4.21881420 |
| 6 | -6.58274069 | -0.81396675 | 2.73328045 |
| 6 | -4.33718326 | -1.71494606 | 2.15873924 |
| 6 | -5.78587172 | -0.62777042 | 0.38515693 |
| 6 | -1.63750437 | 0.30988338 | -5.71757860 |
| 6 | -2.64530131 | -0.42674154 | -3.56745122 |
| 6 | -0.22563317 | -0.95667398 | -4.10828401 |
| 1 | 0.31589364 | 3.32201812 | 2.47169183 |
| 1 | -0.71425315 | 1.95064546 | 2.99463472 |
| 1 | -1.62684937 | 4.09131387 | 1.14125594 |
| 1 | -2.18861060 | 3.06760063 | -0.20462851 |
| 1 | -6.05881754 | 1.31948763 | 3.73152872 |
| 1 | -3.09145732 | 4.30969689 | 2.92605363 |
| 1 | 1.24620661 | 2.03382047 | -0.02242012 |

| | | | |
|---|---|---|---|
| 1 | 0.69063376 | 3.72267683 | 0.12087590 |
| 1 | -1.11541402 | 2.62534367 | -5.30127173 |
| 1 | 0.22344776 | 4.77789125 | -1.84311504 |
| 1 | -7.01583672 | -1.78934249 | 2.46930613 |
| 1 | -6.34376527 | -0.83893697 | 3.80642963 |
| 1 | -7.35773341 | -0.05177307 | 2.56687046 |
| 1 | -3.43157724 | -1.63034222 | 1.54884646 |
| 1 | -4.04165124 | -1.70725758 | 3.21817721 |
| 1 | -4.81202757 | -2.68514322 | 1.94575658 |
| 1 | -6.52749348 | 0.15726288 | 0.17683832 |
| 1 | -4.94101476 | -0.49848850 | -0.29972399 |
| 1 | -6.25780571 | -1.60065602 | 0.17908553 |
| 1 | -1.93186012 | -0.65327373 | -6.15815091 |
| 1 | -0.75394961 | 0.66834782 | -6.26556828 |
| 1 | -2.46333115 | 1.01582551 | -5.88764088 |
| 1 | -2.50568688 | -0.64778076 | -2.50417037 |
| 1 | -3.45725420 | 0.30830609 | -3.66483526 |
| 1 | -2.96241666 | -1.35115608 | -4.07386272 |
| 1 | 0.69030186 | -0.60395175 | -4.60520808 |
| 1 | 0.01029752 | -1.18839239 | -3.06405636 |
| 1 | -0.54469577 | -1.88335733 | -4.60956618 |
| 1 | 0.88127548 | 1.62439323 | 2.25977374 |
| 8 | -0.58142283 | -0.91363171 | 1.57221345 |
| 8 | 0.49850007 | -2.37213116 | 0.14471723 |
| 6 | 0.60245602 | -1.58329839 | 1.33448032 |
| 6 | 1.01343963 | -2.34481997 | 2.61070192 |
| 1 | 1.88907534 | -2.97785376 | 2.39629741 |
| 1 | 1.34736488 | -1.56397590 | 3.30881819 |
| 6 | -0.10135431 | -3.16598183 | 3.27435996 |
| 1 | -0.99808076 | -2.53389087 | 3.34699300 |
| 1 | 0.20915706 | -3.39474537 | 4.30614426 |
| 6 | -0.44101510 | -4.47947391 | 2.56296188 |
| 1 | 0.42002995 | -5.16710247 | 2.64100132 |
| 1 | -1.27307360 | -4.96964571 | 3.09300092 |
| 6 | -0.79978314 | -4.32609881 | 1.08079995 |
| 1 | -1.67586842 | -3.67174924 | 0.95024282 |
| 1 | -1.08148686 | -5.31429166 | 0.68079132 |
| 6 | 0.34838710 | -3.79933452 | 0.21549162 |
| 1 | 1.30056071 | -4.26659032 | 0.53281322 |
| 1 | 0.18264427 | -4.07865735 | -0.83439392 |
| 31 | -1.13495423 | 0.33217010 | 0.34085165 |
| 1 | -5.06096658 | 3.50617665 | 4.24211030 |
| 1 | -0.43017418 | 4.74221666 | -4.25425445 |
| 8 | 9.28339874 | -0.28173397 | -0.76329572 |
| 6 | 10.28383695 | -0.45652138 | 0.26912173 |
| 1 | 10.64300863 | 0.51748442 | 0.62463127 |
| 6 | 7.21723151 | 0.59790783 | -1.53126452 |
| 6 | 5.79453186 | 0.95639605 | -1.09460113 |
| 6 | 5.04507766 | -0.20978148 | -0.44008958 |
| 6 | 8.17499090 | 0.40952380 | -0.36855908 |
| 6 | 3.62167580 | 0.16876739 | -0.01640745 |
| 6 | 2.87584259 | -1.00240551 | 0.60986902 |
| 1 | 7.64223356 | 1.40278547 | -2.15439604 |

| | | | |
|---|---|---|---|
| 1 | 5.23353175 | 1.30055884 | -1.97788734 |
| 1 | 5.00436165 | -1.05733349 | -1.14617714 |
| 1 | 3.05144742 | 0.52106865 | -0.89127307 |
| 1 | 7.23603719 | -0.30732797 | -2.15641181 |
| 1 | 5.84104532 | 1.80297643 | -0.39274638 |
| 1 | 5.61023208 | -0.55535094 | 0.44058453 |
| 1 | 3.66290761 | 1.00126671 | 0.70542877 |
| 1 | 3.43585485 | -1.38185530 | 1.48444328 |
| 1 | 2.76744373 | -1.81919392 | -0.12080084 |
| 8 | 8.01601566 | 0.83042857 | 0.76231357 |
| 8 | 1.57221139 | -0.55097027 | 1.02104651 |
| 1 | 11.09365427 | -1.01905745 | -0.20518316 |
| 1 | 9.86660466 | -1.01460124 | 1.11661594 |

Electronic energy = -3908.664345 a.u.
DFT-D3(BJ) dispersion correction = -0.136199 a.u.
Thermal free energy = 0.739434 a.u.
Gibbs free energy = -3908.061110 a.u.
Number of imaginary frequencies = 0.

**Int3 CL isomer/conformer 5**

| Atomic N. | X | Y | Z |
|---|---|---|---|
| 7 | -0.35691561 | 2.07931707 | 1.36213414 |
| 8 | -2.68212156 | 1.55707757 | -0.43065104 |
| 8 | 0.12132836 | 0.59349120 | -1.22251264 |
| 6 | -0.42889113 | 1.69397877 | 2.79457023 |
| 6 | -3.34253434 | 2.33991616 | 0.46017220 |
| 6 | -4.76539328 | 2.31163257 | 0.53056067 |
| 6 | -5.37684135 | 3.15130454 | 1.47323804 |
| 6 | -4.65391678 | 3.99679642 | 2.31998517 |
| 6 | -3.26662827 | 4.03314868 | 2.22376084 |
| 6 | -2.60678945 | 3.22372974 | 1.29304385 |
| 6 | -1.11975016 | 3.35345780 | 1.10038831 |
| 6 | -5.60119825 | 1.40154069 | -0.39062445 |
| 6 | 0.83685499 | 1.70394921 | -1.53214920 |
| 6 | 1.15345382 | 1.98027706 | -2.89439418 |
| 6 | 1.90910777 | 3.13153166 | -3.15743653 |
| 6 | 2.34867550 | 3.99656241 | -2.15110300 |
| 6 | 2.04368519 | 3.70851408 | -0.82574720 |
| 6 | 1.30415604 | 2.56397268 | -0.50362388 |
| 6 | 1.08846896 | 2.21388511 | 0.94684676 |
| 6 | 0.68429043 | 1.05509533 | -4.03490277 |
| 6 | -7.11340122 | 1.58434576 | -0.16125523 |
| 6 | -5.26936908 | -0.08426792 | -0.11605308 |
| 6 | -5.31640594 | 1.74223097 | -1.87295551 |
| 6 | 1.18426303 | 1.53979809 | -5.40898585 |
| 6 | -0.86139816 | 1.02383729 | -4.09298490 |
| 6 | 1.23471083 | -0.37602172 | -3.82362495 |
| 1 | 0.05836336 | 2.46543329 | 3.41276944 |
| 1 | -1.47854036 | 1.59826844 | 3.09214425 |
| 1 | -0.71450682 | 4.13860064 | 1.76060980 |
| 1 | -0.88960252 | 3.63063296 | 0.06119049 |
| 1 | -6.46212652 | 3.14653399 | 1.55776760 |

| | | | |
|---|---|---|---|
| 1 | -2.68352614 | 4.70553852 | 2.85737931 |
| 1 | 1.54594982 | 1.24123223 | 1.18527895 |
| 1 | 1.55895374 | 2.97859205 | 1.58714306 |
| 1 | 2.16223078 | 3.37237453 | -4.18829761 |
| 1 | 2.39016551 | 4.36331634 | -0.02297508 |
| 1 | -7.66064335 | 0.92659728 | -0.85152904 |
| 1 | -7.41469620 | 1.31327233 | 0.86109274 |
| 1 | -7.43968611 | 2.61580887 | -0.35855817 |
| 1 | -4.21361792 | -0.30751962 | -0.30256466 |
| 1 | -5.49762078 | -0.34528336 | 0.92772268 |
| 1 | -5.88074514 | -0.72693440 | -0.76821693 |
| 1 | -5.58993652 | 2.78540681 | -2.08906579 |
| 1 | -4.25959270 | 1.60040070 | -2.12317888 |
| 1 | -5.92041999 | 1.09307598 | -2.52520426 |
| 1 | 0.83398503 | 0.84123757 | -6.18210571 |
| 1 | 2.28226068 | 1.57023012 | -5.46297720 |
| 1 | 0.79494038 | 2.53590401 | -5.66470816 |
| 1 | -1.29547384 | 0.63366421 | -3.16660899 |
| 1 | -1.26350685 | 2.03241355 | -4.26632945 |
| 1 | -1.18807107 | 0.38118323 | -4.92472139 |
| 1 | 2.33483582 | -0.37005493 | -3.81884189 |
| 1 | 0.88181443 | -0.80784816 | -2.88080042 |
| 1 | 0.90516269 | -1.02372832 | -4.65044229 |
| 1 | 0.07409949 | 0.72984823 | 2.93126621 |
| 8 | -1.65816156 | -0.82950477 | 1.08208907 |
| 8 | -0.77420733 | -2.42372230 | -0.27634621 |
| 6 | -0.82512819 | -1.92532401 | 1.06150033 |
| 6 | -1.28876727 | -2.92091120 | 2.13747716 |
| 1 | -0.56154267 | -3.74388366 | 2.22525205 |
| 1 | -1.23408216 | -2.35139623 | 3.07509342 |
| 6 | -2.70724893 | -3.47978611 | 1.94705348 |
| 1 | -3.36855331 | -2.66540748 | 1.61443062 |
| 1 | -3.08393247 | -3.80425307 | 2.92969823 |
| 6 | -2.78982145 | -4.66689453 | 0.98063489 |
| 1 | -2.17070337 | -5.49184420 | 1.37640861 |
| 1 | -3.82437541 | -5.04490870 | 0.95845850 |
| 6 | -2.33662712 | -4.35656658 | -0.44928496 |
| 1 | -3.00821342 | -3.62055744 | -0.91960611 |
| 1 | -2.39986450 | -5.27637365 | -1.05505926 |
| 6 | -0.90574160 | -3.82686658 | -0.55829559 |
| 1 | -0.21908080 | -4.42633410 | 0.06638239 |
| 1 | -0.55697004 | -3.91105250 | -1.59761917 |
| 31 | -1.14914000 | 0.65221115 | 0.12147683 |
| 1 | -5.17668384 | 4.62592144 | 3.04042039 |
| 1 | 2.92628338 | 4.88478386 | -2.40673140 |
| 8 | 9.04137457 | -2.65870577 | 1.55108236 |
| 6 | 10.31627137 | -2.13322428 | 1.99281537 |
| 1 | 10.46471256 | -1.11542585 | 1.61080756 |
| 6 | 6.67360610 | -2.58053282 | 1.47168304 |
| 6 | 5.42878483 | -1.74490295 | 1.76784372 |
| 6 | 4.14040640 | -2.42245189 | 1.28943211 |
| 6 | 7.95666965 | -1.94111369 | 1.96700750 |
| 6 | 2.89080467 | -1.58084849 | 1.57202577 |

| | | | |
|---|---|---|---|
| 6 | 1.61569474 | -2.25375039 | 1.08001662 |
| 1 | 6.78107353 | -2.77172779 | 0.39224644 |
| 1 | 5.53099276 | -0.75939255 | 1.28768269 |
| 1 | 4.21007483 | -2.62271842 | 0.20660621 |
| 1 | 2.99036471 | -0.60142657 | 1.07678098 |
| 1 | 6.59992430 | -3.57638083 | 1.94041656 |
| 1 | 5.37183808 | -1.54988250 | 2.84949083 |
| 1 | 4.03998503 | -3.40578742 | 1.78145277 |
| 1 | 2.80595436 | -1.38875044 | 2.65398158 |
| 1 | 1.50791717 | -3.24228473 | 1.56596547 |
| 1 | 1.65776874 | -2.40857047 | -0.00999026 |
| 8 | 8.03850995 | -0.93571591 | 2.64681722 |
| 8 | 0.49924859 | -1.41220212 | 1.40764074 |
| 1 | 11.06774751 | -2.81483865 | 1.58296952 |
| 1 | 10.36494837 | -2.11399317 | 3.08885758 |

Electronic energy = -3908.664237 a.u.
DFT-D3(BJ) dispersion correction = -0.135256 a.u.
Thermal free energy = 0.739813 a.u.
Gibbs free energy = -3908.059680 a.u.
Number of imaginary frequencies = 0.

**Int3 CL isomer/conformer 6**

| Atomic N. | X | Y | Z |
|---|---|---|---|
| 7 | -1.11407219 | 1.92656336 | 1.62873150 |
| 8 | -3.02902257 | 0.43997192 | -0.10363802 |
| 8 | -0.14766447 | 0.89654909 | -1.04246323 |
| 6 | -0.94095615 | 1.52770757 | 3.04835141 |
| 6 | -3.92999370 | 0.81723608 | 0.83663598 |
| 6 | -5.17981568 | 0.14213401 | 0.95424727 |
| 6 | -6.05998738 | 0.59212330 | 1.94941505 |
| 6 | -5.76085724 | 1.65759148 | 2.80395064 |
| 6 | -4.54832340 | 2.32458304 | 2.66216240 |
| 6 | -3.63855062 | 1.92293677 | 1.67852645 |
| 6 | -2.38410472 | 2.71798892 | 1.43876779 |
| 6 | -5.55324126 | -1.03081950 | 0.02691688 |
| 6 | -0.02768020 | 2.21881141 | -1.31902559 |
| 6 | 0.06024201 | 2.64969414 | -2.67525392 |
| 6 | 0.19621561 | 4.02590410 | -2.90473439 |
| 6 | 0.24473952 | 4.96624387 | -1.87146852 |
| 6 | 0.17137100 | 4.53159920 | -0.55321321 |
| 6 | 0.05032967 | 3.16668551 | -0.26478128 |
| 6 | 0.08987619 | 2.71733242 | 1.17392650 |
| 6 | 0.00729256 | 1.64674627 | -3.84485162 |
| 6 | -6.97099714 | -1.56087351 | 0.31279540 |
| 6 | -4.57028118 | -2.20739913 | 0.23298612 |
| 6 | -5.52400714 | -0.56955829 | -1.44987746 |
| 6 | 0.16413534 | 2.34693455 | -5.20806199 |
| 6 | -1.35621303 | 0.91592968 | -3.85764693 |
| 6 | 1.15806787 | 0.61927068 | -3.72084821 |
| 1 | -0.84031328 | 2.42680236 | 3.67802675 |
| 1 | -1.81555154 | 0.95395203 | 3.37343217 |
| 1 | -2.34777771 | 3.59078110 | 2.11222332 |

| | | | |
|---|---|---|---|
| 1 | -2.35397685 | 3.08615370 | 0.40287616 |
| 1 | -7.01913036 | 0.09157809 | 2.07087247 |
| 1 | -4.30513913 | 3.17684596 | 3.30108622 |
| 1 | 0.95189132 | 2.05728358 | 1.35467929 |
| 1 | 0.18871018 | 3.59622098 | 1.83277577 |
| 1 | 0.26117235 | 4.38576591 | -3.92991163 |
| 1 | 0.22254077 | 5.24826531 | 0.26970948 |
| 1 | -7.19158989 | -2.38219632 | -0.38390059 |
| 1 | -7.06783269 | -1.95931629 | 1.33328017 |
| 1 | -7.73900055 | -0.78745394 | 0.16670475 |
| 1 | -3.53777243 | -1.92202060 | 0.00583081 |
| 1 | -4.60803882 | -2.56529011 | 1.27237617 |
| 1 | -4.85207548 | -3.04447561 | -0.42435153 |
| 1 | -6.25055143 | 0.23920481 | -1.61674568 |
| 1 | -4.53078853 | -0.21070435 | -1.74030023 |
| 1 | -5.79786664 | -1.40982171 | -2.10613331 |
| 1 | 0.13167498 | 1.58869993 | -6.00334009 |
| 1 | 1.12465348 | 2.87557713 | -5.29352375 |
| 1 | -0.64713459 | 3.06332023 | -5.40295026 |
| 1 | -1.52114175 | 0.34672957 | -2.93692913 |
| 1 | -2.18062187 | 1.63435300 | -3.97320992 |
| 1 | -1.39396730 | 0.21692464 | -4.70707878 |
| 1 | 2.13411583 | 1.12563410 | -3.74534555 |
| 1 | 1.08606583 | 0.04409939 | -2.79154391 |
| 1 | 1.11963412 | -0.08104110 | -4.56932554 |
| 1 | -0.04381163 | 0.90451811 | 3.13627371 |
| 8 | -0.98384587 | -1.30174726 | 1.17464171 |
| 8 | 0.64798547 | -2.28544215 | -0.07362266 |
| 6 | 0.30392200 | -1.79367417 | 1.21469741 |
| 6 | 0.41006532 | -2.79749066 | 2.37574006 |
| 1 | 1.44753099 | -3.15152012 | 2.47302307 |
| 1 | 0.19774786 | -2.20028386 | 3.27275831 |
| 6 | -0.55974167 | -3.98690105 | 2.29839371 |
| 1 | -1.54157357 | -3.62245726 | 1.96111485 |
| 1 | -0.70513994 | -4.38098528 | 3.31639667 |
| 6 | -0.07327458 | -5.13007831 | 1.40050566 |
| 1 | 0.86817577 | -5.53280563 | 1.81526419 |
| 1 | -0.80135940 | -5.95600706 | 1.43692727 |
| 6 | 0.16708979 | -4.73397118 | -0.05978388 |
| 1 | -0.77483049 | -4.42709937 | -0.54173992 |
| 1 | 0.53539808 | -5.61329620 | -0.61466546 |
| 6 | 1.18459792 | -3.60748564 | -0.25047344 |
| 1 | 2.06885034 | -3.76832750 | 0.39308128 |
| 1 | 1.53689642 | -3.59700571 | -1.29179140 |
| 31 | -1.22212575 | 0.32420181 | 0.35263354 |
| 8 | 1.14344047 | -0.61579792 | 1.49619673 |
| 1 | -6.47738067 | 1.96492733 | 3.56565773 |
| 1 | 0.34248768 | 6.02720984 | -2.10111786 |
| 8 | 8.58244650 | 0.56154030 | -0.17447794 |
| 6 | 8.96176136 | 1.89266435 | -0.60120151 |
| 1 | 8.71171754 | 2.04248279 | -1.65900065 |
| 6 | 6.96378132 | -1.14834200 | 0.11643173 |
| 6 | 5.48190622 | -1.41832270 | 0.39036885 |

| | | | |
|---|---|---|---|
| 6 | 4.97259834 | -0.74975743 | 1.67469708 |
| 6 | 7.25146817 | 0.28473965 | -0.29500380 |
| 6 | 3.52313584 | -1.10778605 | 2.03968838 |
| 6 | 2.50476221 | -0.58204619 | 1.02312004 |
| 1 | 7.32556782 | -1.78567659 | -0.70785176 |
| 1 | 5.33592556 | -2.50858065 | 0.46544106 |
| 1 | 5.62776447 | -1.04240197 | 2.51242487 |
| 1 | 3.27914733 | -0.67776276 | 3.02405827 |
| 1 | 7.59032057 | -1.39792580 | 0.98620979 |
| 1 | 4.89716308 | -1.07477783 | -0.47500629 |
| 1 | 5.06122046 | 0.34410574 | 1.57317716 |
| 1 | 3.43303666 | -2.20170441 | 2.14223010 |
| 1 | 2.56742024 | -1.10518586 | 0.06011110 |
| 1 | 2.71051413 | 0.48220276 | 0.82541022 |
| 8 | 6.43442965 | 1.08758374 | -0.70494767 |
| 1 | 10.04344975 | 1.94869086 | -0.44620553 |
| 1 | 8.44266853 | 2.65002059 | -0.00061742 |

Electronic energy = -3908.660004 a.u.
DFT-D3(BJ) dispersion correction = -0.136927 a.u.
Thermal free energy = 0.738457 a.u.
Gibbs free energy = -3908.058474 a.u.
Number of imaginary frequencies = 0.

**Int3 CL isomer/conformer 7**

| Atomic N. | X | Y | Z |
|---|---|---|---|
| 7 | -0.83706879 | 2.54474774 | 0.66046053 |
| 8 | -2.94300962 | 1.12984498 | -0.90157222 |
| 8 | 0.00496541 | 0.76596529 | -1.63295743 |
| 6 | -0.82648638 | 2.45004289 | 2.14221767 |
| 6 | -3.78561726 | 1.88483025 | -0.15416619 |
| 6 | -5.15795417 | 1.52358102 | -0.02213806 |
| 6 | -5.96614103 | 2.35557746 | 0.76655144 |
| 6 | -5.48299259 | 3.50126291 | 1.40603579 |
| 6 | -4.14744847 | 3.85707172 | 1.24821096 |
| 6 | -3.30016857 | 3.06747877 | 0.46373218 |
| 6 | -1.89089237 | 3.51505178 | 0.18671863 |
| 6 | -5.73387863 | 0.27419658 | -0.71671034 |
| 6 | 0.42851555 | 1.93865888 | -2.16531160 |
| 6 | 0.67326019 | 2.02798962 | -3.56739279 |
| 6 | 1.12520994 | 3.25846749 | -4.06418386 |
| 6 | 1.33492136 | 4.37478552 | -3.24921509 |
| 6 | 1.10286830 | 4.27142724 | -1.88257012 |
| 6 | 0.66531622 | 3.06181221 | -1.32898004 |
| 6 | 0.53198636 | 2.95026526 | 0.16908635 |
| 6 | 0.44761859 | 0.82387592 | -4.50333206 |
| 6 | -7.24701069 | 0.12511737 | -0.46990115 |
| 6 | -5.04892814 | -1.00152562 | -0.17233343 |
| 6 | -5.52593606 | 0.37452094 | -2.24687740 |
| 6 | 0.81739375 | 1.15365120 | -5.96197554 |
| 6 | -1.04359747 | 0.41175963 | -4.48935785 |
| 6 | 1.33068865 | -0.36871570 | -4.06372124 |
| 1 | -0.56333677 | 3.42855272 | 2.57601824 |

| | | | |
|---|---|---|---|
| 1 | -1.82043771 | 2.15347678 | 2.49416852 |
| 1 | -1.69683516 | 4.49062253 | 0.66334749 |
| 1 | -1.72864564 | 3.62657782 | -0.89528112 |
| 1 | -7.01694666 | 2.10157297 | 0.89448950 |
| 1 | -3.75485625 | 4.76285565 | 1.71632764 |
| 1 | 1.21742050 | 2.18741255 | 0.56851862 |
| 1 | 0.78851975 | 3.91653846 | 0.63480815 |
| 1 | 1.31580864 | 3.35850104 | -5.13114458 |
| 1 | 1.27250347 | 5.12794457 | -1.22603823 |
| 1 | -7.60781869 | -0.76849021 | -0.99895552 |
| 1 | -7.48403220 | -0.00503960 | 0.59610298 |
| 1 | -7.81478828 | 0.98668856 | -0.85031792 |
| 1 | -3.96816412 | -0.99249849 | -0.34843235 |
| 1 | -5.22066732 | -1.10077157 | 0.90955203 |
| 1 | -5.47374347 | -1.88923629 | -0.66593359 |
| 1 | -6.04565711 | 1.25584779 | -2.65060974 |
| 1 | -4.46416210 | 0.44763247 | -2.50532519 |
| 1 | -5.94440232 | -0.51790510 | -2.73689169 |
| 1 | 0.64958390 | 0.26163327 | -6.58207271 |
| 1 | 1.87504165 | 1.43606410 | -6.06754476 |
| 1 | 0.19747638 | 1.96265318 | -6.37478056 |
| 1 | -1.37325756 | 0.11001877 | -3.48982075 |
| 1 | -1.67889626 | 1.24315371 | -4.82724437 |
| 1 | -1.20038899 | -0.43500618 | -5.17481123 |
| 1 | 2.39597136 | -0.09758810 | -4.10777789 |
| 1 | 1.09267056 | -0.69218018 | -3.04473474 |
| 1 | 1.17086021 | -1.21750590 | -4.74610140 |
| 1 | -0.09241343 | 1.69513208 | 2.44570944 |
| 8 | -1.40663362 | -0.66075294 | 0.90850216 |
| 8 | 0.00317927 | -2.24723584 | 0.09213160 |
| 6 | -0.26017210 | -1.37582319 | 1.18132321 |
| 6 | -0.40497300 | -2.03837059 | 2.56139488 |
| 1 | 0.53406212 | -2.54102059 | 2.83872746 |
| 1 | -0.52659130 | -1.19758969 | 3.25749508 |
| 6 | -1.59154367 | -3.00578624 | 2.69505039 |
| 1 | -2.46295735 | -2.57314374 | 2.18099068 |
| 1 | -1.86064288 | -3.07751758 | 3.76037719 |
| 6 | -1.30299729 | -4.41745732 | 2.17158583 |
| 1 | -0.47812981 | -4.85371205 | 2.76282062 |
| 1 | -2.18048201 | -5.05865865 | 2.35137779 |
| 6 | -0.92802712 | -4.48214447 | 0.68758646 |
| 1 | -1.77271271 | -4.16258031 | 0.05665490 |
| 1 | -0.70890878 | -5.52892950 | 0.41748948 |
| 6 | 0.29061100 | -3.64102703 | 0.30431727 |
| 1 | 1.10440314 | -3.77376334 | 1.04013558 |
| 1 | 0.67601588 | -3.96900330 | -0.67159717 |
| 31 | -1.23333739 | 0.75181426 | -0.25438985 |
| 1 | -6.15182777 | 4.11154135 | 2.01294758 |
| 1 | 1.67812037 | 5.31338226 | -3.68417002 |
| 8 | 9.08435222 | -2.35817458 | 3.03198503 |
| 6 | 10.03502491 | -2.77333475 | 4.04221359 |
| 1 | 10.04349143 | -2.06125304 | 4.87698462 |
| 6 | 6.87778475 | -1.83780761 | 2.33943620 |

| | | | |
|---|---|---|---|
| 6 | 5.41741228 | -1.69671856 | 2.76736759 |
| 6 | 4.50523156 | -1.27351107 | 1.61130818 |
| 6 | 7.79315639 | -2.27353517 | 3.46763202 |
| 6 | 3.03886831 | -1.12043442 | 2.03320866 |
| 6 | 2.15105948 | -0.71218725 | 0.85613589 |
| 1 | 7.26860572 | -0.88997002 | 1.93498187 |
| 1 | 5.34791205 | -0.96407124 | 3.58613861 |
| 1 | 4.86418244 | -0.31878497 | 1.18884313 |
| 1 | 2.95565071 | -0.36033343 | 2.82708700 |
| 1 | 6.98300281 | -2.56970903 | 1.52172783 |
| 1 | 5.06967912 | -2.65222045 | 3.18902716 |
| 1 | 4.57771097 | -2.01456364 | 0.79614660 |
| 1 | 2.68498472 | -2.06924677 | 2.46718364 |
| 1 | 2.12426157 | -1.49146529 | 0.08112519 |
| 1 | 2.56512225 | 0.19332987 | 0.38374851 |
| 8 | 7.45279703 | -2.52163247 | 4.60881054 |
| 8 | 0.80762618 | -0.35565465 | 1.23108981 |
| 1 | 11.00531721 | -2.79015563 | 3.53674138 |
| 1 | 9.77829045 | -3.76918605 | 4.42478440 |

Electronic energy = -3908.661646 a.u.
DFT-D3(BJ) dispersion correction = -0.135614 a.u.
Thermal free energy = 0.739699 a.u.
Gibbs free energy = -3908.057561 a.u.
Number of imaginary frequencies = 0.

**Int3 CL isomer/conformer 8**

| Atomic N. | X | Y | Z |
|---|---|---|---|
| 7 | -0.08202175 | 1.75081615 | 1.35090721 |
| 8 | 2.01531960 | 0.54744601 | -0.40315232 |
| 8 | 1.41733632 | -0.67636162 | 2.33147090 |
| 6 | -1.42263462 | 2.06690001 | 0.79468904 |
| 6 | 1.80229310 | 1.65854226 | -1.15158205 |
| 6 | 2.20833759 | 1.70136357 | -2.51684271 |
| 6 | 1.95422051 | 2.88670548 | -3.22271867 |
| 6 | 1.33670858 | 3.99989106 | -2.64426145 |
| 6 | 0.96858967 | 3.95410536 | -1.30363970 |
| 6 | 1.20907628 | 2.79981652 | -0.55081506 |
| 6 | 0.93056794 | 2.78939013 | 0.92707240 |
| 6 | 2.89802040 | 0.50036176 | -3.19276725 |
| 6 | 1.86673042 | 0.18918833 | 3.27466182 |
| 6 | 3.05198553 | -0.11126365 | 4.00835413 |
| 6 | 3.45071694 | 0.80826763 | 4.98889647 |
| 6 | 2.74619983 | 1.98516658 | 5.25753364 |
| 6 | 1.59304097 | 2.26672726 | 4.53439210 |
| 6 | 1.13882199 | 1.37578330 | 3.55434714 |
| 6 | -0.16782913 | 1.66105377 | 2.85813424 |
| 6 | 3.87483096 | -1.38596588 | 3.73257678 |
| 6 | 3.29153922 | 0.80934574 | -4.64969661 |
| 6 | 1.94243751 | -0.71573897 | -3.22034125 |
| 6 | 4.19688650 | 0.13735500 | -2.43399475 |
| 6 | 5.09101961 | -1.49903502 | 4.67154954 |
| 6 | 4.41447798 | -1.36444047 | 2.28276124 |

| | | | |
|---|---|---|---|
| 6 | 3.00688276 | -2.64713384 | 3.95540301 |
| 1 | -1.76787335 | 3.03525244 | 1.19177265 |
| 1 | -1.35843410 | 2.12471304 | -0.29688384 |
| 1 | 0.56291784 | 3.77542072 | 1.25631769 |
| 1 | 1.84682440 | 2.56229682 | 1.49091133 |
| 1 | 2.24531296 | 2.94860494 | -4.26985786 |
| 1 | 0.50994967 | 4.82173395 | -0.82362234 |
| 1 | -0.90347596 | 0.86692008 | 3.05660496 |
| 1 | -0.58298078 | 2.61248829 | 3.22947621 |
| 1 | 4.35252554 | 0.60767857 | 5.56422780 |
| 1 | 1.02418331 | 3.17804289 | 4.73229285 |
| 1 | 3.79269710 | -0.07059193 | -5.07750870 |
| 1 | 2.41644863 | 1.02817717 | -5.27890426 |
| 1 | 3.99079273 | 1.65524429 | -4.71934587 |
| 1 | 1.64285688 | -1.02725723 | -2.21436541 |
| 1 | 1.03228922 | -0.47928455 | -3.79079396 |
| 1 | 2.43940948 | -1.56572395 | -3.71281773 |
| 1 | 4.90021521 | 0.98296271 | -2.44406404 |
| 1 | 3.99355453 | -0.13372806 | -1.39251870 |
| 1 | 4.68764161 | -0.71545552 | -2.92736281 |
| 1 | 5.63579379 | -2.42484841 | 4.43843428 |
| 1 | 4.79483414 | -1.54674026 | 5.72956085 |
| 1 | 5.79343003 | -0.66292949 | 4.54327730 |
| 1 | 3.60514758 | -1.33112817 | 1.54587851 |
| 1 | 5.06190176 | -0.49026198 | 2.12334945 |
| 1 | 5.01402639 | -2.26842387 | 2.09647446 |
| 1 | 2.63859216 | -2.68710025 | 4.99111660 |
| 1 | 2.14699525 | -2.66636539 | 3.27750573 |
| 1 | 3.61146389 | -3.54923187 | 3.77680339 |
| 1 | -2.12325452 | 1.27285884 | 1.07353394 |
| 8 | -0.30070013 | -1.26992362 | -0.20939952 |
| 8 | -1.71524022 | -1.15981595 | 1.57469437 |
| 6 | -1.52876050 | -1.69586814 | 0.23821660 |
| 6 | -1.61262382 | -3.22879686 | 0.12519447 |
| 1 | -2.63941275 | -3.57685494 | 0.31726799 |
| 1 | -1.40874277 | -3.42171385 | -0.93693248 |
| 6 | -0.62183149 | -4.01179691 | 0.99972689 |
| 1 | 0.34995968 | -3.49480173 | 0.99489503 |
| 1 | -0.45067213 | -4.99259006 | 0.52984238 |
| 6 | -1.09976140 | -4.24160669 | 2.43848757 |
| 1 | -2.04803531 | -4.80769336 | 2.41104006 |
| 1 | -0.37529753 | -4.88420807 | 2.96321162 |
| 6 | -1.31085766 | -2.96152309 | 3.25313960 |
| 1 | -0.35401910 | -2.44112862 | 3.41473978 |
| 1 | -1.70212987 | -3.22831099 | 4.24944682 |
| 6 | -2.28694608 | -1.96738340 | 2.62713551 |
| 1 | -3.19889765 | -2.47577166 | 2.27050692 |
| 1 | -2.60196782 | -1.22935535 | 3.38018232 |
| 31 | 0.67617427 | -0.05203838 | 0.74628479 |
| 1 | 1.15766813 | 4.89603353 | -3.23831789 |
| 1 | 3.10157091 | 2.67272051 | 6.02485424 |
| 8 | -5.27511370 | 1.57601211 | -6.53324236 |
| 6 | -4.20564912 | 0.65638889 | -6.82374723 |

| | | | |
|---|---|---|---|
| 1 | -4.15749729 | 0.60418437 | -7.91630725 |
| 6 | -4.83130552 | 1.15829912 | -4.13129370 |
| 6 | -5.27564273 | 1.58908534 | -2.73134628 |
| 6 | -4.45049791 | 0.94731939 | -1.60677920 |
| 6 | -5.61292060 | 1.85824683 | -5.23125065 |
| 6 | -4.68494102 | -0.55775172 | -1.42943842 |
| 6 | -3.86981572 | -1.17701051 | -0.29789675 |
| 1 | -4.94084216 | 0.06992215 | -4.26715203 |
| 1 | -6.34378397 | 1.35583409 | -2.60262759 |
| 1 | -3.37836424 | 1.12485176 | -1.78608992 |
| 1 | -4.44321439 | -1.10088001 | -2.35675462 |
| 1 | -3.75603877 | 1.36326921 | -4.26907067 |
| 1 | -5.20077910 | 2.68413278 | -2.66629382 |
| 1 | -4.69817947 | 1.45359363 | -0.65833061 |
| 1 | -5.75219110 | -0.74251295 | -1.21903185 |
| 1 | -4.16665254 | -2.23372153 | -0.16898737 |
| 1 | -4.07089846 | -0.64796975 | 0.65241831 |
| 8 | -6.51444095 | 2.64447801 | -5.03402153 |
| 8 | -2.48299129 | -1.06826177 | -0.63968341 |
| 1 | -4.41207558 | -0.34628523 | -6.42303836 |
| 1 | -3.24298125 | 1.01959315 | -6.43668210 |

Electronic energy = -3908.651595 a.u.
DFT-D3(BJ) dispersion correction = -0.138869 a.u.
Thermal free energy = 0.740197 a.u.
Gibbs free energy = -3908.050266 a.u.
Number of imaginary frequencies = 0.

**Int3 CL isomer/conformer 9**

| Atomic N. | X | Y | Z |
|---|---|---|---|
| 7 | 1.91175198 | 2.52405372 | 1.39113951 |
| 8 | 1.12519169 | 0.03082968 | 2.82037499 |
| 8 | -0.78350314 | 1.28174274 | 0.80482033 |
| 6 | 3.22671596 | 2.63058590 | 0.71011683 |
| 6 | 2.26557146 | 0.25290137 | 3.52004173 |
| 6 | 2.87877894 | -0.79512064 | 4.26599788 |
| 6 | 4.05737964 | -0.48135122 | 4.95900689 |
| 6 | 4.63032152 | 0.79412101 | 4.94952044 |
| 6 | 4.00509063 | 1.81606153 | 4.24251968 |
| 6 | 2.82239129 | 1.55872333 | 3.54132354 |
| 6 | 2.06897290 | 2.68238039 | 2.88531172 |
| 1 | 5.55109440 | 0.98360549 | 5.50118682 |
| 6 | 2.27460215 | -2.21184201 | 4.31665328 |
| 6 | -1.24176542 | 2.42113092 | 1.38347326 |
| 6 | -2.57474653 | 2.47651422 | 1.88778562 |
| 6 | -2.99670604 | 3.68535253 | 2.45913431 |
| 6 | -2.17239183 | 4.81016978 | 2.55144919 |
| 6 | -0.87780743 | 4.74572199 | 2.04993074 |
| 6 | -0.40754689 | 3.56819799 | 1.45586414 |
| 6 | 0.96640892 | 3.56411805 | 0.83495596 |
| 1 | -2.54412556 | 5.72505034 | 3.01255222 |
| 6 | -3.52107834 | 1.26162206 | 1.81259289 |
| 6 | 3.08303042 | -3.14939699 | 5.23319397 |

| | | | |
|---|---|---|---|
| 6 | 2.26855404 | -2.84036543 | 2.90350835 |
| 6 | 0.83308712 | -2.15227557 | 4.87635199 |
| 6 | -4.90655284 | 1.57220327 | 2.41050419 |
| 6 | -2.93415352 | 0.07397922 | 2.61130297 |
| 6 | -3.74112009 | 0.85366935 | 0.33657548 |
| 1 | 3.65823491 | 3.62875473 | 0.88923732 |
| 1 | 3.90500292 | 1.86808593 | 1.10859110 |
| 1 | 2.57149704 | 3.64579559 | 3.07357661 |
| 1 | 1.04770332 | 2.74578698 | 3.28754264 |
| 1 | 4.55618664 | -1.26289647 | 5.52946570 |
| 1 | 4.41782758 | 2.82768598 | 4.24590792 |
| 1 | 0.91043744 | 3.35507897 | -0.24389436 |
| 1 | 1.43426213 | 4.55354922 | 0.96867045 |
| 1 | -4.00712930 | 3.75559725 | 2.85736510 |
| 1 | -0.21935640 | 5.61567732 | 2.10390265 |
| 1 | 2.60051097 | -4.13706562 | 5.24691257 |
| 1 | 4.11361040 | -3.29157048 | 4.87677200 |
| 1 | 3.11970635 | -2.78441232 | 6.26996267 |
| 1 | 1.68085633 | -2.24864164 | 2.19422388 |
| 1 | 3.29285141 | -2.92433121 | 2.51229967 |
| 1 | 1.84010880 | -3.85335207 | 2.95076482 |
| 1 | 0.83112965 | -1.74554803 | 5.89831732 |
| 1 | 0.18381121 | -1.52865585 | 4.25236371 |
| 1 | 0.40935009 | -3.16734908 | 4.91655371 |
| 1 | -5.54043248 | 0.67843850 | 2.32256230 |
| 1 | -5.41393638 | 2.39044800 | 1.87923239 |
| 1 | -4.84887396 | 1.83144265 | 3.47746941 |
| 1 | -1.96584990 | -0.24685269 | 2.21386200 |
| 1 | -2.79829031 | 0.34633466 | 3.66775539 |
| 1 | -3.62700771 | -0.78009618 | 2.56835669 |
| 1 | -4.21088646 | 1.67372626 | -0.22611378 |
| 1 | -2.79547767 | 0.59471577 | -0.15178590 |
| 1 | -4.41130914 | -0.01802225 | 0.28670860 |
| 1 | 3.08547274 | 2.47143889 | -0.36463592 |
| 6 | 3.12723189 | -0.55479488 | -1.95020614 |
| 6 | 3.25686254 | -0.40576255 | -3.46863526 |
| 6 | 2.77923556 | 0.93561724 | -4.04712158 |
| 6 | 1.73149385 | -0.32827434 | -1.34902496 |
| 6 | 1.25034291 | 1.08218859 | -4.03222565 |
| 6 | 0.64482325 | 1.51106567 | -2.68859648 |
| 1 | 3.79322430 | 0.15904795 | -1.44273467 |
| 1 | 4.31837995 | -0.55104662 | -3.72517775 |
| 1 | 3.12452906 | 1.00512439 | -5.09047258 |
| 1 | 0.93573828 | 1.83448865 | -4.77382471 |
| 1 | 3.44320553 | -1.56258909 | -1.64452022 |
| 1 | 2.70392780 | -1.21946640 | -3.96401885 |
| 1 | 3.25226966 | 1.77412038 | -3.50776519 |
| 1 | 0.80902674 | 0.12600898 | -4.34792191 |
| 1 | -0.40944021 | 1.19649401 | -2.63018333 |
| 1 | 0.66598602 | 2.60887901 | -2.60840018 |
| 8 | 1.77849908 | -0.52538316 | 0.01666142 |
| 8 | 1.34693303 | 1.08531390 | -1.50088963 |
| 31 | 0.96858336 | 0.72902678 | 1.09389210 |

| | | | |
|---|---|---|---|
| 8 | -4.18752959 | -2.84888058 | -7.65085013 |
| 6 | -4.99343534 | -3.72244735 | -8.47725296 |
| 1 | -4.44418472 | -4.64265811 | -8.71317231 |
| 6 | -2.98425784 | -2.39657695 | -5.65837007 |
| 6 | -2.51448739 | -2.94116596 | -4.31002375 |
| 6 | -1.67959402 | -1.92417127 | -3.52538621 |
| 6 | -3.81780471 | -3.38571061 | -6.44982079 |
| 6 | -1.20116862 | -2.46969889 | -2.17596009 |
| 6 | -0.41039556 | -1.47900314 | -1.33318490 |
| 1 | -2.12930995 | -2.09831976 | -6.28691060 |
| 1 | -1.92857992 | -3.85879606 | -4.47390431 |
| 1 | -0.80568159 | -1.62249929 | -4.12494039 |
| 1 | -0.58514257 | -3.36989849 | -2.33371536 |
| 1 | -3.58537055 | -1.48141688 | -5.52909350 |
| 1 | -3.39071720 | -3.24580296 | -3.71705847 |
| 1 | -2.27547176 | -1.00972315 | -3.35654794 |
| 1 | -2.07233815 | -2.78587647 | -1.57775678 |
| 1 | -0.18715126 | -1.91352020 | -0.34744175 |
| 1 | -0.98686682 | -0.55000942 | -1.17793617 |
| 8 | -4.13150247 | -4.50521041 | -6.09274794 |
| 8 | 0.82945893 | -1.18844213 | -2.01654779 |
| 1 | -5.20072798 | -3.15054640 | -9.38686741 |
| 1 | -5.92604917 | -3.98582988 | -7.96248041 |

Electronic energy = -3908.665578 a.u.
DFT-D3(BJ) dispersion correction = -0.138107 a.u.
Thermal free energy = 0.739486 a.u.
Gibbs free energy = -3908.064199 a.u.
Number of imaginary frequencies = 0.

**Int3 CL isomer/conformer 10**

| Atomic N. | X | Y | Z |
|---|---|---|---|
| 7 | 2.44228978 | 2.35768142 | 0.28831976 |
| 8 | 1.32397424 | 0.11508864 | 1.90123350 |
| 8 | -0.41466406 | 1.49601306 | -0.19191768 |
| 6 | 3.74509464 | 2.22153272 | -0.40946048 |
| 6 | 2.49581873 | 0.21520376 | 2.57449269 |
| 6 | 2.96062176 | -0.85554317 | 3.39248518 |
| 6 | 4.18390612 | -0.67110386 | 4.05380122 |
| 6 | 4.93910486 | 0.50074432 | 3.94611544 |
| 6 | 4.45986691 | 1.55012210 | 3.16867313 |
| 6 | 3.24036362 | 1.42171560 | 2.49585765 |
| 6 | 2.64922667 | 2.59526243 | 1.76545191 |
| 1 | 5.88699846 | 0.59075680 | 4.47669662 |
| 6 | 2.15613126 | -2.16052854 | 3.54908635 |
| 6 | -0.68994502 | 2.72646809 | 0.31255634 |
| 6 | -1.99154258 | 3.01228420 | 0.82059559 |
| 6 | -2.21843816 | 4.30525618 | 1.31307139 |
| 6 | -1.23466560 | 5.29826580 | 1.32395626 |
| 6 | 0.02684716 | 5.00962330 | 0.81712420 |
| 6 | 0.30593341 | 3.73838843 | 0.30013549 |
| 6 | 1.65086200 | 3.48671307 | -0.33192930 |
| 1 | -1.45851812 | 6.28649725 | 1.72558028 |

| | | | |
|---|---|---|---|
| 6 | -3.10800348 | 1.95116661 | 0.82556924 |
| 6 | 2.83083233 | -3.13779586 | 4.53013675 |
| 6 | 2.04109937 | -2.88137775 | 2.18543821 |
| 6 | 0.74557748 | -1.85147452 | 4.10517022 |
| 6 | -4.42306506 | 2.49952377 | 1.41243256 |
| 6 | -2.69737409 | 0.73590737 | 1.69142578 |
| 6 | -3.41010617 | 1.49613444 | -0.62261333 |
| 1 | 4.32368942 | 3.15353443 | -0.30232353 |
| 1 | 4.31020641 | 1.39371578 | 0.03291607 |
| 1 | 3.29166804 | 3.48437677 | 1.87956855 |
| 1 | 1.65606326 | 2.83784013 | 2.16982844 |
| 1 | 4.57117620 | -1.47450578 | 4.67803396 |
| 1 | 5.01750393 | 2.48680354 | 3.09461866 |
| 1 | 1.54299849 | 3.21394142 | -1.39249555 |
| 1 | 2.26362197 | 4.40162881 | -0.27351191 |
| 1 | -3.20064524 | 4.55085571 | 1.71266834 |
| 1 | 0.80773823 | 5.77350007 | 0.80602364 |
| 1 | 2.20897163 | -4.04011492 | 4.61825790 |
| 1 | 3.82460715 | -3.45558516 | 4.18244793 |
| 1 | 2.93490508 | -2.70752436 | 5.53685497 |
| 1 | 1.53765956 | -2.26195807 | 1.43601574 |
| 1 | 3.03747641 | -3.14219395 | 1.80001559 |
| 1 | 1.46990390 | -3.81473619 | 2.30727099 |
| 1 | 0.81521955 | -1.37730444 | 5.09526163 |
| 1 | 0.18641367 | -1.18511576 | 3.43969680 |
| 1 | 0.17953086 | -2.78869568 | 4.21883478 |
| 1 | -5.17948702 | 1.70287852 | 1.38413317 |
| 1 | -4.81389197 | 3.34978828 | 0.83436028 |
| 1 | -4.31106871 | 2.81223631 | 2.46087439 |
| 1 | -1.78317527 | 0.26224617 | 1.31996230 |
| 1 | -2.52467878 | 1.04491766 | 2.73280002 |
| 1 | -3.50179659 | -0.01326251 | 1.67938683 |
| 1 | -3.77420335 | 2.34188089 | -1.22496321 |
| 1 | -2.51664469 | 1.08608353 | -1.10583054 |
| 1 | -4.18678398 | 0.71783626 | -0.61094671 |
| 1 | 3.56163426 | 2.01668341 | -1.47003712 |
| 6 | 3.17165494 | -1.04362920 | -2.80728011 |
| 6 | 3.29943251 | -1.01455718 | -4.33356103 |
| 6 | 2.95111003 | 0.32243997 | -5.00768564 |
| 6 | 1.81260046 | -0.62584333 | -2.22401342 |
| 6 | 1.44585564 | 0.63021494 | -4.99374919 |
| 6 | 0.91278337 | 1.22072858 | -3.68060407 |
| 1 | 3.91614959 | -0.37316375 | -2.35264542 |
| 1 | 4.33875598 | -1.28211733 | -4.58315375 |
| 1 | 3.28607492 | 0.27677703 | -6.05574875 |
| 1 | 1.20225368 | 1.35480107 | -5.78786604 |
| 1 | 3.38009636 | -2.05651261 | -2.43346784 |
| 1 | 2.66404608 | -1.80300056 | -4.76689974 |
| 1 | 3.51654146 | 1.14551291 | -4.53784526 |
| 1 | 0.90098136 | -0.29439762 | -5.23116702 |
| 1 | -0.16741381 | 1.02541789 | -3.58593975 |
| 1 | 1.05052007 | 2.31285693 | -3.68234707 |
| 8 | 1.83463951 | -0.73636967 | -0.84849159 |

| | | | |
|---|---|---|---|
| 8 | 1.59123175 | 0.80878926 | -2.47437663 |
| 31 | 1.23504156 | 0.70598963 | 0.12882857 |
| 8 | -6.40953974 | -3.46013118 | -0.61367436 |
| 6 | -7.44904980 | -2.53956732 | -0.20227423 |
| 1 | -7.35835675 | -2.31071129 | 0.86703351 |
| 6 | -4.12215543 | -4.05880665 | -0.81705904 |
| 6 | -2.74126539 | -3.49647412 | -1.16949395 |
| 6 | -2.72894026 | -2.68744545 | -2.47516031 |
| 6 | -5.13773153 | -3.00208850 | -0.42533468 |
| 6 | -1.32608103 | -2.42287934 | -3.04293528 |
| 6 | -0.44409244 | -1.54877898 | -2.15877341 |
| 1 | -4.04396021 | -4.74117290 | 0.04652929 |
| 1 | -2.04394553 | -4.34529697 | -1.26172632 |
| 1 | -3.30078482 | -3.24016307 | -3.23965107 |
| 1 | -1.41863365 | -1.94036002 | -4.02882891 |
| 1 | -4.53546785 | -4.65823651 | -1.64162720 |
| 1 | -2.38369692 | -2.87949942 | -0.33256861 |
| 1 | -3.25831657 | -1.73366103 | -2.32114330 |
| 1 | -0.80759358 | -3.38141004 | -3.20940734 |
| 1 | -0.27859719 | -2.00833430 | -1.17329301 |
| 1 | -0.90524439 | -0.55788811 | -2.00253908 |
| 8 | -4.88468093 | -1.90085261 | 0.02766450 |
| 8 | 0.82272392 | -1.42080167 | -2.84187136 |
| 1 | -8.39125634 | -3.05560571 | -0.41006904 |
| 1 | -7.38252791 | -1.60604824 | -0.77499044 |

Electronic energy = -3908.664631 a.u.
DFT-D3(BJ) dispersion correction = -0.140052 a.u.
Thermal free energy = 0.742250 a.u.
Gibbs free energy = -3908.062433 a.u.
Number of imaginary frequencies = 0.

**Int3_CL isomer/conformer 11**

| Atomic N. | X | Y | Z |
|---|---|---|---|
| 7 | 2.38194976 | 2.12748797 | 0.46939897 |
| 8 | 1.49346900 | -0.13150765 | 2.20173535 |
| 8 | -0.44187448 | 1.08572946 | 0.19863629 |
| 6 | 3.66222170 | 2.05139767 | -0.27814473 |
| 6 | 2.68634772 | 0.07019167 | 2.81316189 |
| 6 | 3.26317304 | -0.94054870 | 3.63603574 |
| 6 | 4.49992455 | -0.65174390 | 4.23166008 |
| 6 | 5.16465785 | 0.56569732 | 4.05538303 |
| 6 | 4.57736831 | 1.55475436 | 3.27318769 |
| 6 | 3.33979300 | 1.32183001 | 2.66414158 |
| 6 | 2.63615482 | 2.42819766 | 1.92836095 |
| 1 | 6.12741796 | 0.73738557 | 4.53672906 |
| 6 | 2.56155574 | -2.29308068 | 3.86666527 |
| 6 | -0.75324423 | 2.31931790 | 0.67314691 |
| 6 | -2.02557108 | 2.55236152 | 1.27450415 |
| 6 | -2.30058897 | 3.85453268 | 1.71552453 |
| 6 | -1.38697412 | 4.90538504 | 1.59819889 |
| 6 | -0.14775248 | 4.66429747 | 1.01731996 |
| 6 | 0.17472262 | 3.38650759 | 0.54446933 |

| | | | |
|---|---|---|---|
| 6 | 1.49413375 | 3.18102370 | -0.15419942 |
| 1 | -1.64544439 | 5.89930471 | 1.96303264 |
| 6 | -3.05985340 | 1.42294036 | 1.44966492 |
| 6 | 3.34936444 | -3.19115780 | 4.83921480 |
| 6 | 2.43250264 | -3.06089589 | 2.53027174 |
| 6 | 1.16104066 | -2.06573909 | 4.48452203 |
| 6 | -4.36039847 | 1.92747287 | 2.10352033 |
| 6 | -2.48609349 | 0.31543526 | 2.36477070 |
| 6 | -3.44058222 | 0.82890393 | 0.07377215 |
| 1 | 4.18245588 | 3.02140541 | -0.22215175 |
| 1 | 4.29727606 | 1.27705611 | 0.16622768 |
| 1 | 3.21981369 | 3.36205798 | 1.98543968 |
| 1 | 1.64787674 | 2.61636406 | 2.37156118 |
| 1 | 4.97153786 | -1.40695401 | 4.85797139 |
| 1 | 5.06408505 | 2.52460681 | 3.14568175 |
| 1 | 1.34472155 | 2.85390210 | -1.19407885 |
| 1 | 2.05467970 | 4.13030937 | -0.16975686 |
| 1 | -3.26336622 | 4.06146900 | 2.17881599 |
| 1 | 0.58158469 | 5.47090876 | 0.91386820 |
| 1 | 2.79791189 | -4.13148115 | 4.98156875 |
| 1 | 4.34565997 | -3.44954715 | 4.45184893 |
| 1 | 3.46984939 | -2.72483002 | 5.82788453 |
| 1 | 1.85133638 | -2.50168736 | 1.78978695 |
| 1 | 3.42542851 | -3.26406650 | 2.10338510 |
| 1 | 1.93519825 | -4.02742498 | 2.70505622 |
| 1 | 1.24469054 | -1.55856679 | 5.45700811 |
| 1 | 0.52541860 | -1.45948691 | 3.83028238 |
| 1 | 0.66844339 | -3.03594333 | 4.65070813 |
| 1 | -5.06135481 | 1.08571010 | 2.19600948 |
| 1 | -4.85320167 | 2.70373459 | 1.50038453 |
| 1 | -4.18961159 | 2.32596729 | 3.11392316 |
| 1 | -1.57723137 | -0.12973861 | 1.94665907 |
| 1 | -2.24457926 | 0.71976027 | 3.35819275 |
| 1 | -3.23375435 | -0.48165767 | 2.49626469 |
| 1 | -3.88503414 | 1.59783337 | -0.57475776 |
| 1 | -2.56517410 | 0.40998190 | -0.43357863 |
| 1 | -4.18558593 | 0.03005660 | 0.21141115 |
| 1 | 3.45058742 | 1.79990437 | -1.32304093 |
| 6 | 3.26037799 | -1.33770786 | -2.46197129 |
| 6 | 3.44343384 | -1.36096887 | -3.98204634 |
| 6 | 3.07771468 | -0.06103290 | -4.71494834 |
| 6 | 1.86649272 | -0.95657812 | -1.93535920 |
| 6 | 1.56456695 | 0.19955994 | -4.75019518 |
| 6 | 0.97823263 | 0.83119224 | -3.48161169 |
| 1 | 3.96207877 | -0.62270930 | -2.00725983 |
| 1 | 4.49883257 | -1.60414768 | -4.18440753 |
| 1 | 3.44281933 | -0.13389030 | -5.75126014 |
| 1 | 1.31757260 | 0.88388789 | -5.57832942 |
| 1 | 3.49433715 | -2.32675420 | -2.04259668 |
| 1 | 2.84814009 | -2.18373905 | -4.40807631 |
| 1 | 3.60559863 | 0.79549655 | -4.26091104 |
| 1 | 1.05272907 | -0.74958339 | -4.96263741 |
| 1 | -0.10754540 | 0.64689729 | -3.43798030 |

| | | | |
|---|---|---|---|
| 1 | 1.12458722 | 1.92206806 | -3.51348156 |
| 8 | 1.85663365 | -1.03794336 | -0.55525942 |
| 8 | 1.58646027 | 0.45750446 | -2.22440091 |
| 31 | 1.27224970 | 0.40257491 | 0.42398670 |
| 8 | -7.11132479 | -1.06645556 | -2.26079389 |
| 6 | -8.06972234 | -0.08056435 | -2.71349072 |
| 1 | -7.68860803 | 0.93329745 | -2.53794514 |
| 6 | -4.97617452 | -2.09189260 | -2.36418394 |
| 6 | -3.53111981 | -1.93622652 | -2.83649771 |
| 6 | -2.62757790 | -3.06322001 | -2.32075040 |
| 6 | -5.89744149 | -1.00614705 | -2.88479814 |
| 6 | -1.16058123 | -2.93530276 | -2.76101539 |
| 6 | -0.38299438 | -1.88734470 | -1.97201885 |
| 1 | -5.04130433 | -2.10150006 | -1.26509169 |
| 1 | -3.15481464 | -0.95519303 | -2.51129820 |
| 1 | -2.67972346 | -3.10986233 | -1.21889841 |
| 1 | -1.11604354 | -2.68605786 | -3.83378238 |
| 1 | -5.39276884 | -3.05991004 | -2.69192192 |
| 1 | -3.51015465 | -1.92003504 | -3.93708865 |
| 1 | -3.02214585 | -4.02688609 | -2.68248617 |
| 1 | -0.64637865 | -3.90075190 | -2.63973827 |
| 1 | -0.30412779 | -2.18332506 | -0.91433687 |
| 1 | -0.87202576 | -0.89981907 | -2.01528553 |
| 8 | -5.62650238 | -0.18846152 | -3.74366877 |
| 8 | 0.93517408 | -1.81541479 | -2.55506344 |
| 1 | -8.97317455 | -0.26301847 | -2.12367039 |
| 1 | -8.27285997 | -0.20515960 | -3.78468911 |

Electronic energy = -3908.664152 a.u.
DFT-D3(BJ) dispersion correction = -0.139278 a.u.
Thermal free energy = 0.741661 a.u.
Gibbs free energy = -3908.061769 a.u.
Number of imaginary frequencies = 0.

**Int3_CL isomer/conformer 12**

| Atomic N. | X | Y | Z |
|---|---|---|---|
| 7 | 2.22168811 | 2.54277191 | 0.00943633 |
| 8 | 1.23684655 | 0.29866772 | 1.70438575 |
| 8 | -0.58107896 | 1.48553943 | -0.41856295 |
| 6 | 3.52578282 | 2.46496232 | -0.69562430 |
| 6 | 2.40691234 | 0.48243025 | 2.36539338 |
| 6 | 2.93224208 | -0.53424063 | 3.21440933 |
| 6 | 4.14879382 | -0.26301689 | 3.85805772 |
| 6 | 4.83999912 | 0.94280926 | 3.70435232 |
| 6 | 4.30053802 | 1.93916533 | 2.89718342 |
| 6 | 3.08470531 | 1.72363915 | 2.23987664 |
| 6 | 2.42495910 | 2.83904566 | 1.47711465 |
| 1 | 5.78571580 | 1.10026861 | 4.22295305 |
| 6 | 2.19803003 | -1.87301648 | 3.42247410 |
| 6 | -0.92707479 | 2.71012505 | 0.05535744 |
| 6 | -2.23563275 | 2.92869301 | 0.57922335 |
| 6 | -2.53897894 | 4.21927463 | 1.03549233 |
| 6 | -1.62182876 | 5.27302748 | 0.99836560 |

| | | | |
|---|---|---|---|
| 6 | -0.35163069 | 5.04889395 | 0.48055179 |
| 6 | 0.00193812 | 3.78289004 | -0.00163709 |
| 6 | 1.35493642 | 3.59707555 | -0.64024326 |
| 1 | -1.90298877 | 6.25714442 | 1.37288034 |
| 6 | -3.28303507 | 1.79911771 | 0.64339759 |
| 6 | 2.92971346 | -2.78001545 | 4.42985895 |
| 6 | 2.10915861 | -2.64596008 | 2.08574602 |
| 6 | 0.77798317 | -1.61742411 | 3.98200382 |
| 6 | -4.61904579 | 2.28305651 | 1.23867010 |
| 6 | -2.77522438 | 0.64535262 | 1.53997568 |
| 6 | -3.57965433 | 1.27696984 | -0.78192955 |
| 1 | 4.04439562 | 3.43492894 | -0.62576273 |
| 1 | 4.14491441 | 1.69066288 | -0.22930917 |
| 1 | 3.01788809 | 3.76539067 | 1.55735898 |
| 1 | 1.42258617 | 3.03871713 | 1.88209548 |
| 1 | 4.58236470 | -1.02343866 | 4.50523655 |
| 1 | 4.80733830 | 2.90086162 | 2.78730960 |
| 1 | 1.25535226 | 3.28196686 | -1.68985443 |
| 1 | 1.90725025 | 4.55113393 | -0.61920471 |
| 1 | -3.52817969 | 4.41431792 | 1.44529430 |
| 1 | 0.37856178 | 5.86005339 | 0.43485094 |
| 1 | 2.35591627 | -3.70944634 | 4.55353516 |
| 1 | 3.93595679 | -3.05820795 | 4.08410776 |
| 1 | 3.01881236 | -2.31093223 | 5.42061156 |
| 1 | 1.57045751 | -2.07889892 | 1.31976744 |
| 1 | 3.11443595 | -2.87076527 | 1.70086175 |
| 1 | 1.58472681 | -3.60061763 | 2.24393390 |
| 1 | 0.83124843 | -1.10826369 | 4.95563129 |
| 1 | 0.18078291 | -1.00273051 | 3.29994157 |
| 1 | 0.26047299 | -2.57733811 | 4.13013301 |
| 1 | -5.32666052 | 1.44196529 | 1.25416797 |
| 1 | -5.07324983 | 3.08661398 | 0.64106284 |
| 1 | -4.50685092 | 2.63813684 | 2.27318929 |
| 1 | -1.84684182 | 0.20909462 | 1.15776609 |
| 1 | -2.58949062 | 1.00226937 | 2.56315045 |
| 1 | -3.53538858 | -0.14813070 | 1.59330669 |
| 1 | -3.99327420 | 2.07912511 | -1.41058713 |
| 1 | -2.67437881 | 0.89102995 | -1.26280081 |
| 1 | -4.32487371 | 0.46802222 | -0.73465459 |
| 1 | 3.34860538 | 2.21046637 | -1.74645456 |
| 6 | 3.15342572 | -0.94316165 | -2.97690890 |
| 6 | 3.27303581 | -0.98955818 | -4.50294005 |
| 6 | 2.86988684 | 0.29695930 | -5.24078680 |
| 6 | 1.78048787 | -0.55798847 | -2.40464178 |
| 6 | 1.35409089 | 0.54608095 | -5.22681759 |
| 6 | 0.80734226 | 1.18601316 | -3.94357970 |
| 1 | 3.86862975 | -0.21678777 | -2.56291148 |
| 1 | 4.32058436 | -1.23000179 | -4.74514311 |
| 1 | 3.19818740 | 0.21137198 | -6.28839752 |
| 1 | 1.07451341 | 1.21789278 | -6.05486693 |
| 1 | 3.40891174 | -1.92439877 | -2.55167450 |
| 1 | 2.66567727 | -1.82237735 | -4.89078830 |
| 1 | 3.40665337 | 1.16372628 | -4.81803580 |

| | | | |
|---|---|---|---|
| 1 | 0.84382744 | -0.41010335 | -5.40958938 |
| 1 | -0.26884801 | 0.97041242 | -3.84298318 |
| 1 | 0.91983660 | 2.27976717 | -3.99986139 |
| 8 | 1.82134133 | -0.60612500 | -1.02503039 |
| 8 | 1.48794334 | 0.85165891 | -2.71408043 |
| 31 | 1.12309441 | 0.81500786 | -0.08759510 |
| 8 | -5.48003136 | -3.56455304 | 1.41463393 |
| 6 | -5.68165402 | -4.18563264 | 2.70748893 |
| 1 | -5.80653026 | -5.27019848 | 2.59703436 |
| 6 | -4.27339207 | -3.34835014 | -0.62105390 |
| 6 | -2.82542348 | -3.18957423 | -1.09475435 |
| 6 | -2.74209197 | -2.61788236 | -2.51525018 |
| 6 | -4.37863541 | -4.00962455 | 0.73935443 |
| 6 | -1.31411342 | -2.49975764 | -3.07024507 |
| 6 | -0.44289062 | -1.52677976 | -2.28509367 |
| 1 | -4.83168127 | -3.98827122 | -1.32713766 |
| 1 | -2.32453283 | -4.16851660 | -1.05070567 |
| 1 | -3.32545854 | -3.26270333 | -3.19350838 |
| 1 | -1.35889510 | -2.16300866 | -4.11798001 |
| 1 | -4.80047707 | -2.38418238 | -0.59552135 |
| 1 | -2.29183759 | -2.53750519 | -0.38656331 |
| 1 | -3.22813264 | -1.62754209 | -2.54390035 |
| 1 | -0.82867410 | -3.48880587 | -3.07463617 |
| 1 | -0.26651036 | -1.88739468 | -1.26150456 |
| 1 | -0.92839958 | -0.53661610 | -2.22271334 |
| 8 | -3.61158342 | -4.84142777 | 1.18573566 |
| 8 | 0.82137270 | -1.42489069 | -2.97379972 |
| 1 | -6.59114296 | -3.72875076 | 3.10927174 |
| 1 | -4.82455435 | -3.98965417 | 3.36363399 |

Electronic energy = -3908.664582 a.u.
DFT-D3(BJ) dispersion correction = -0.139960 a.u.
Thermal free energy = 0.743544 a.u.
Gibbs free energy = -3908.060997 a.u.
Number of imaginary frequencies = 0.

**Int3 CL isomer/conformer 13**

| Atomic N. | X | Y | Z |
|---|---|---|---|
| 7 | 2.44855672 | 2.55916269 | 0.72167088 |
| 8 | 1.43868954 | 0.21310916 | 2.25690256 |
| 8 | -0.37074919 | 1.57924803 | 0.23097818 |
| 6 | 3.75362976 | 2.51026404 | 0.01574646 |
| 6 | 2.60955568 | 0.34002981 | 2.92894137 |
| 6 | 3.12571041 | -0.73806522 | 3.70493086 |
| 6 | 4.34409096 | -0.52373653 | 4.36636958 |
| 6 | 5.04598761 | 0.68366004 | 4.29807817 |
| 6 | 4.51602533 | 1.73856847 | 3.56220067 |
| 6 | 3.29911331 | 1.58054861 | 2.89088127 |
| 6 | 2.65174536 | 2.75219008 | 2.20625143 |
| 1 | 5.99243669 | 0.79612718 | 4.82690174 |
| 6 | 2.37911325 | -2.08130447 | 3.81956837 |
| 6 | -0.69644960 | 2.77738594 | 0.78057989 |
| 6 | -2.00225120 | 2.98461564 | 1.31569381 |

| | | | |
|---|---|---|---|
| 6 | -2.28551069 | 4.24905800 | 1.85122848 |
| 6 | -1.35097082 | 5.28765055 | 1.88122781 |
| 6 | -0.08338959 | 5.07493630 | 1.35250518 |
| 6 | 0.25028456 | 3.83589662 | 0.79222500 |
| 6 | 1.60118002 | 3.66970716 | 0.14418377 |
| 1 | -1.61668225 | 6.25108335 | 2.31596162 |
| 6 | -3.06752951 | 1.87022976 | 1.30935712 |
| 6 | 3.10378769 | -3.06353212 | 4.75918600 |
| 6 | 2.28125145 | -2.75721105 | 2.43188708 |
| 6 | 0.96247801 | -1.85163630 | 4.39841062 |
| 6 | -4.39467010 | 2.33712573 | 1.93712655 |
| 6 | -2.57708778 | 0.65328174 | 2.12896942 |
| 6 | -3.37416569 | 1.44385329 | -0.14552725 |
| 1 | 4.28776065 | 3.46404574 | 0.15599467 |
| 1 | 4.35880232 | 1.69467933 | 0.42668416 |
| 1 | 3.25390107 | 3.66471647 | 2.35054476 |
| 1 | 1.65079487 | 2.93417958 | 2.62296528 |
| 1 | 4.77046983 | -1.33135271 | 4.95882941 |
| 1 | 5.03135168 | 2.70107256 | 3.52003717 |
| 1 | 1.49893837 | 3.42842748 | -0.92450070 |
| 1 | 2.16992904 | 4.61033959 | 0.23053104 |
| 1 | -3.27232636 | 4.43468213 | 2.27095743 |
| 1 | 0.66036027 | 5.87497350 | 1.35875062 |
| 1 | 2.52226491 | -3.99458506 | 4.81850321 |
| 1 | 4.10709178 | -3.32496616 | 4.39259900 |
| 1 | 3.19814699 | -2.66628989 | 5.78032282 |
| 1 | 1.74372496 | -2.13444673 | 1.70944698 |
| 1 | 3.28386564 | -2.96036506 | 2.02848502 |
| 1 | 1.75251752 | -3.71839034 | 2.52480586 |
| 1 | 1.02215034 | -1.40996539 | 5.40400505 |
| 1 | 0.36948460 | -1.18691155 | 3.76097392 |
| 1 | 0.43692259 | -2.81502420 | 4.48394024 |
| 1 | -5.11669432 | 1.50904292 | 1.89997904 |
| 1 | -4.83593329 | 3.18474525 | 1.39310115 |
| 1 | -4.27566493 | 2.62328285 | 2.99205901 |
| 1 | -1.66091956 | 0.22356708 | 1.71155101 |
| 1 | -2.37743649 | 0.94247065 | 3.17067146 |
| 1 | -3.35438816 | -0.12591639 | 2.13674605 |
| 1 | -3.77974490 | 2.28921404 | -0.72052738 |
| 1 | -2.47458890 | 1.07884316 | -0.65286769 |
| 1 | -4.12779910 | 0.64135167 | -0.14732155 |
| 1 | 3.57561010 | 2.33498673 | -1.05095810 |
| 6 | 3.32838531 | -0.70791725 | -2.49522475 |
| 6 | 3.45604076 | -0.63087553 | -4.01949514 |
| 6 | 3.07067105 | 0.71563646 | -4.65261766 |
| 6 | 1.95652507 | -0.35326904 | -1.90127898 |
| 6 | 1.55744493 | 0.97954212 | -4.62804339 |
| 6 | 1.00822103 | 1.51715705 | -3.29953140 |
| 1 | 4.05021720 | -0.02724648 | -2.01964069 |
| 1 | 4.50221800 | -0.86249814 | -4.27576342 |
| 1 | 3.40496228 | 0.71137137 | -5.70179059 |
| 1 | 1.29163322 | 1.72043461 | -5.39970300 |
| 1 | 3.56920641 | -1.72391555 | -2.15062218 |

| | | | |
|---|---|---|---|
| 1 | 2.84190902 | -1.42249326 | -4.47670750 |
| 1 | 3.61360425 | 1.53965494 | -4.15835274 |
| 1 | 1.03838985 | 0.04769133 | -4.89303643 |
| 1 | -0.06911343 | 1.29942217 | -3.21917620 |
| 1 | 1.12606313 | 2.61126209 | -3.26661328 |
| 8 | 1.98415044 | -0.51133133 | -0.52926730 |
| 8 | 1.68437689 | 1.08084396 | -2.09963843 |
| 31 | 1.32272508 | 0.85890990 | 0.50615790 |
| 8 | -6.94848286 | -4.52762617 | -2.65736855 |
| 6 | -7.92364545 | -5.25571533 | -3.44120859 |
| 1 | -8.21666437 | -4.67554340 | -4.32535564 |
| 6 | -4.84145363 | -3.46726171 | -2.42175269 |
| 6 | -3.48437064 | -3.18976158 | -3.06734795 |
| 6 | -2.55712226 | -2.38406735 | -2.15146600 |
| 6 | -5.78912797 | -4.23854815 | -3.31976069 |
| 6 | -1.19085266 | -2.10784308 | -2.78881871 |
| 6 | -0.27706583 | -1.30645040 | -1.87171137 |
| 1 | -5.34343385 | -2.52660214 | -2.13997405 |
| 1 | -3.63704985 | -2.65106157 | -4.01495531 |
| 1 | -3.04278331 | -1.42857539 | -1.88895679 |
| 1 | -1.32449685 | -1.56212817 | -3.73705061 |
| 1 | -4.72943799 | -4.03136850 | -1.48192138 |
| 1 | -3.00732978 | -4.14481486 | -3.33665213 |
| 1 | -2.41500038 | -2.92770958 | -1.20150959 |
| 1 | -0.69474315 | -3.05944690 | -3.03811728 |
| 1 | -0.12588323 | -1.83235093 | -0.91562563 |
| 1 | -0.71480386 | -0.31749091 | -1.65134095 |
| 8 | -5.57787848 | -4.56424962 | -4.47249772 |
| 8 | 0.99184860 | -1.15857657 | -2.54361117 |
| 1 | -8.77837163 | -5.40446905 | -2.77433172 |
| 1 | -7.51225253 | -6.21937185 | -3.76706590 |

Electronic energy = -3908.665874 a.u.
DFT-D3(BJ) dispersion correction = -0.137749 a.u.
Thermal free energy = 0.742692 a.u.
Gibbs free energy = -3908.060931 a.u.
Number of imaginary frequencies = 0.

**Int3 CL isomer/conformer 14**

| Atomic N. | X | Y | Z |
|---|---|---|---|
| 7 | 1.36268683 | 3.19532728 | 0.97793469 |
| 8 | 0.89586220 | 0.65153906 | 2.45808524 |
| 8 | -0.88045956 | 1.36708784 | 0.09640759 |
| 6 | 2.71770412 | 3.56434368 | 0.49657763 |
| 6 | 1.83434331 | 1.14701704 | 3.30201532 |
| 6 | 2.52364568 | 0.29131131 | 4.20949084 |
| 6 | 3.48042559 | 0.88476123 | 5.04626047 |
| 6 | 3.76682491 | 2.25322388 | 5.02556094 |
| 6 | 3.06532520 | 3.08264477 | 4.15655438 |
| 6 | 2.09377998 | 2.54293294 | 3.30719613 |
| 6 | 1.23755781 | 3.44528961 | 2.46269081 |
| 1 | 4.52454584 | 2.66298009 | 5.69342234 |
| 6 | 2.23146477 | -1.22033632 | 4.27547032 |

| | | | |
|---|---|---|---|
| 6 | -1.65403100 | 2.40109012 | 0.51578304 |
| 6 | -3.03060706 | 2.18350761 | 0.81940939 |
| 6 | -3.78319888 | 3.29358688 | 1.22817694 |
| 6 | -3.24067152 | 4.57573239 | 1.34949887 |
| 6 | -1.89920206 | 4.77597676 | 1.04636671 |
| 6 | -1.10322036 | 3.70595119 | 0.61975423 |
| 6 | 0.32185766 | 3.97686127 | 0.20949055 |
| 1 | -3.86525016 | 5.40573054 | 1.67934991 |
| 6 | -3.67536749 | 0.78783571 | 0.70538737 |
| 6 | 3.05656870 | -1.91811692 | 5.37326350 |
| 6 | 2.59198419 | -1.89220714 | 2.92982225 |
| 6 | 0.73769603 | -1.45715506 | 4.60290922 |
| 6 | -5.16994946 | 0.81050503 | 1.07847794 |
| 6 | -2.97963616 | -0.20529021 | 1.66591462 |
| 6 | -3.57517921 | 0.27859643 | -0.75171560 |
| 1 | 2.89232371 | 4.63918328 | 0.66659874 |
| 1 | 3.46860329 | 2.98611116 | 1.04619110 |
| 1 | 1.48814632 | 4.50258237 | 2.65095207 |
| 1 | 0.17445582 | 3.29851587 | 2.70121851 |
| 1 | 4.03197771 | 0.25646731 | 5.74344911 |
| 1 | 3.25023094 | 4.15945188 | 4.14503996 |
| 1 | 0.48489003 | 3.71344863 | -0.84634352 |
| 1 | 0.54347501 | 5.04983820 | 0.33339049 |
| 1 | -4.83523356 | 3.15666642 | 1.47090569 |
| 1 | -1.45610335 | 5.77117436 | 1.12699992 |
| 1 | 2.79743596 | -2.98637146 | 5.38875154 |
| 1 | 4.13839031 | -1.84467542 | 5.18982718 |
| 1 | 2.84400595 | -1.51118423 | 6.37259320 |
| 1 | 2.01816176 | -1.47456729 | 2.09613713 |
| 1 | 3.66113081 | -1.76354725 | 2.70701440 |
| 1 | 2.38592484 | -2.97210451 | 2.98822261 |
| 1 | 0.48128441 | -1.01965101 | 5.57897712 |
| 1 | 0.08358336 | -1.01786588 | 3.84203113 |
| 1 | 0.53708112 | -2.53821714 | 4.65484777 |
| 1 | -5.57857182 | -0.20448465 | 0.97239569 |
| 1 | -5.75226664 | 1.47148833 | 0.42034806 |
| 1 | -5.33224371 | 1.12356108 | 2.11990705 |
| 1 | -1.91643915 | -0.32259200 | 1.43301109 |
| 1 | -3.06742827 | 0.13672532 | 2.70707510 |
| 1 | -3.46217220 | -1.19180261 | 1.59208322 |
| 1 | -4.11787064 | 0.94806647 | -1.43515608 |
| 1 | -2.53276013 | 0.21604943 | -1.08216404 |
| 1 | -4.02792954 | -0.72194367 | -0.82834352 |
| 1 | 2.78976741 | 3.33868527 | -0.57305728 |
| 6 | 3.76016988 | 0.37508635 | -1.86275656 |
| 6 | 4.12150220 | 0.49236810 | -3.34646199 |
| 6 | 3.45254202 | 1.64961024 | -4.10521141 |
| 6 | 2.26465711 | 0.27587445 | -1.52491826 |
| 6 | 1.95257294 | 1.42165510 | -4.34645871 |
| 6 | 1.04032081 | 1.75533713 | -3.15823230 |
| 1 | 4.13900099 | 1.24786890 | -1.31025753 |
| 1 | 5.21558581 | 0.60377273 | -3.41251689 |
| 1 | 3.94913146 | 1.75366619 | -5.08250390 |

| | | | |
|---|---|---|---|
| 1 | 1.60776214 | 2.04194232 | -5.18991571 |
| 1 | 4.24178325 | -0.51346893 | -1.42970821 |
| 1 | 3.87417196 | -0.45158125 | -3.85698415 |
| 1 | 3.61668146 | 2.60184133 | -3.57191485 |
| 1 | 1.80427358 | 0.37405980 | -4.64405332 |
| 1 | 0.08722515 | 1.20925022 | -3.24748011 |
| 1 | 0.80494949 | 2.83082061 | -3.16379377 |
| 8 | 2.09999621 | 0.13083059 | -0.16106437 |
| 8 | 1.59752725 | 1.55091745 | -1.84099912 |
| 31 | 0.88104712 | 1.22461756 | 0.67849582 |
| 8 | -4.67411944 | -6.42224540 | -3.59775564 |
| 6 | -5.21267759 | -7.42546980 | -4.49155566 |
| 1 | -5.44733529 | -6.98381683 | -5.46828819 |
| 6 | -3.04393140 | -4.79525020 | -3.03705266 |
| 6 | -1.73510007 | -4.11947207 | -3.44383266 |
| 6 | -1.28425122 | -3.06327790 | -2.42935659 |
| 6 | -3.52265045 | -5.83135183 | -4.03533239 |
| 6 | 0.02665403 | -2.37926139 | -2.83289594 |
| 6 | 0.46866558 | -1.33199076 | -1.81994850 |
| 1 | -3.85027247 | -4.05358072 | -2.91305599 |
| 1 | -1.85771912 | -3.65731430 | -4.43526920 |
| 1 | -2.07705895 | -2.30426779 | -2.31514819 |
| 1 | -0.08955925 | -1.90352968 | -3.82032921 |
| 1 | -2.94917706 | -5.28982045 | -2.05669715 |
| 1 | -0.95202500 | -4.88412874 | -3.56224285 |
| 1 | -1.16312106 | -3.53302646 | -1.43798404 |
| 1 | 0.82533972 | -3.13111996 | -2.93545331 |
| 1 | 0.60332057 | -1.78412913 | -0.82451964 |
| 1 | -0.28573073 | -0.53122360 | -1.73132223 |
| 8 | -2.98172753 | -6.11667100 | -5.08676186 |
| 8 | 1.72720952 | -0.79136390 | -2.27550325 |
| 1 | -6.12095024 | -7.79101224 | -4.00286138 |
| 1 | -4.49203308 | -8.24088002 | -4.63255720 |

Electronic energy = -3908.665867 a.u.
DFT-D3(BJ) dispersion correction = -0.137732 a.u.
Thermal free energy = 0.742821 a.u.
Gibbs free energy = -3908.060778 a.u.
Number of imaginary frequencies = 0.

**TS4 ax CL isomer/conformer 1**

| Atomic N. | X | Y | Z |
|---|---|---|---|
| 7 | 0.97835767 | 3.10923076 | 0.73155212 |
| 8 | 1.47052503 | 0.78881192 | 2.43142056 |
| 8 | -0.93350971 | 0.86238188 | 0.34280899 |
| 6 | 2.25205064 | 3.71250343 | 0.27335802 |
| 6 | 2.08680964 | 1.68339550 | 3.22829747 |
| 6 | 3.01234221 | 1.23574245 | 4.21997261 |
| 6 | 3.62879205 | 2.21523428 | 5.01147318 |
| 6 | 3.36782670 | 3.58222961 | 4.87211800 |
| 6 | 2.44487143 | 4.00310421 | 3.92051363 |
| 6 | 1.79600561 | 3.06507111 | 3.11094117 |
| 6 | 0.71920168 | 3.50094128 | 2.16229868 |

| | | | |
|---|---|---|---|
| 1 | 3.87710881 | 4.30446005 | 5.51019669 |
| 6 | 3.31193495 | -0.26179048 | 4.42631149 |
| 6 | -1.84756243 | 1.83134139 | 0.57102931 |
| 6 | -3.15669107 | 1.48279595 | 1.02495050 |
| 6 | -4.07850504 | 2.52455926 | 1.19860792 |
| 6 | -3.76278448 | 3.86575341 | 0.96411669 |
| 6 | -2.47833920 | 4.19564962 | 0.54824984 |
| 6 | -1.52260339 | 3.19372988 | 0.34367341 |
| 6 | -0.15242909 | 3.58214211 | -0.14565544 |
| 1 | -4.51318216 | 4.64082813 | 1.11915851 |
| 6 | -3.54698777 | 0.02309313 | 1.33163917 |
| 6 | 4.32223606 | -0.49525386 | 5.56507826 |
| 6 | 3.91283011 | -0.87334234 | 3.13890576 |
| 6 | 2.01024771 | -1.01065290 | 4.80001640 |
| 6 | -5.00364637 | -0.09568262 | 1.81850183 |
| 6 | -2.64080977 | -0.54501384 | 2.44976044 |
| 6 | -3.41615462 | -0.84284770 | 0.05764844 |
| 1 | 2.19754195 | 4.81144667 | 0.35229003 |
| 1 | 3.07467294 | 3.35463428 | 0.90158814 |
| 1 | 0.59583312 | 4.59713793 | 2.20190430 |
| 1 | -0.24499027 | 3.04925370 | 2.43519688 |
| 1 | 4.34537649 | 1.90520322 | 5.77007198 |
| 1 | 2.20563650 | 5.06334686 | 3.80857968 |
| 1 | 0.04258140 | 3.17030406 | -1.14748311 |
| 1 | -0.09194323 | 4.68162918 | -0.21984485 |
| 1 | -5.08402920 | 2.28701067 | 1.54073679 |
| 1 | -2.20391750 | 5.23837137 | 0.37256619 |
| 1 | 4.49021149 | -1.57599485 | 5.67641544 |
| 1 | 5.29656267 | -0.02895166 | 5.35748816 |
| 1 | 3.95406818 | -0.11687164 | 6.52971268 |
| 1 | 3.20531511 | -0.82028067 | 2.30457067 |
| 1 | 4.84048510 | -0.35171467 | 2.85831571 |
| 1 | 4.16172123 | -1.93162727 | 3.31282282 |
| 1 | 1.59030080 | -0.61322761 | 5.73565245 |
| 1 | 1.25675881 | -0.91926658 | 4.01018476 |
| 1 | 2.22672725 | -2.07905388 | 4.95302248 |
| 1 | -5.22574234 | -1.15305579 | 2.02133608 |
| 1 | -5.72344732 | 0.25456008 | 1.06441879 |
| 1 | -5.17602062 | 0.46198243 | 2.75028038 |
| 1 | -1.58599782 | -0.54965931 | 2.15584755 |
| 1 | -2.74338150 | 0.04829996 | 3.36985183 |
| 1 | -2.93902588 | -1.57917130 | 2.67995763 |
| 1 | -4.08334446 | -0.47138011 | -0.73438871 |
| 1 | -2.38760138 | -0.83602339 | -0.31651080 |
| 1 | -3.70105290 | -1.88231623 | 0.28181738 |
| 1 | 2.44465303 | 3.43338203 | -0.76654977 |
| 6 | 3.56615591 | -0.98193687 | -0.95779812 |
| 6 | 4.22531796 | -0.80053383 | -2.32952976 |
| 6 | 4.04423809 | 0.57214481 | -3.00269628 |
| 6 | 2.05029464 | -0.85121256 | -0.89792633 |
| 6 | 2.62178826 | 0.83873457 | -3.52826762 |
| 6 | 1.65581036 | 1.43398833 | -2.49327421 |
| 1 | 3.97357059 | -0.26736921 | -0.22979467 |

| | | | |
|---|---|---|---|
| 1 | 5.30338240 | -0.98496205 | -2.19832706 |
| 1 | 4.73949385 | 0.61665809 | -3.85535550 |
| 1 | 2.65888667 | 1.55117714 | -4.36812639 |
| 1 | 3.79286575 | -1.98982716 | -0.57202053 |
| 1 | 3.86067009 | -1.58156466 | -3.01487231 |
| 1 | 4.35301352 | 1.37444758 | -2.31161618 |
| 1 | 2.21155524 | -0.09765308 | -3.93030572 |
| 1 | 0.61002463 | 1.28635275 | -2.81787634 |
| 1 | 1.82975876 | 2.52266073 | -2.43734479 |
| 8 | 1.46462333 | -0.86551625 | 0.27021837 |
| 8 | 1.85676301 | 0.90313882 | -1.17708504 |
| 31 | 0.91469491 | 0.98088932 | 0.64064533 |
| 8 | -5.58271790 | -5.07705021 | -5.42533302 |
| 6 | -6.11269808 | -5.80098629 | -6.56157494 |
| 1 | -6.00242090 | -5.20951986 | -7.47923374 |
| 6 | -3.80012512 | -4.00724983 | -4.28724241 |
| 6 | -2.30955744 | -3.67051339 | -4.30188560 |
| 6 | -1.87806189 | -2.88218307 | -3.06099987 |
| 6 | -4.25582170 | -4.76774216 | -5.51760435 |
| 6 | -0.38258842 | -2.54470873 | -3.06828690 |
| 6 | 0.03150060 | -1.75940502 | -1.83372259 |
| 1 | -4.41179657 | -3.09282827 | -4.21459055 |
| 1 | -2.07668042 | -3.09592819 | -5.21144962 |
| 1 | -2.46631360 | -1.95121527 | -2.99399972 |
| 1 | -0.13710880 | -1.96259713 | -3.97137356 |
| 1 | -4.06529617 | -4.60661888 | -3.40134764 |
| 1 | -1.72693396 | -4.60169554 | -4.37578678 |
| 1 | -2.11883259 | -3.46177673 | -2.15346169 |
| 1 | 0.20936821 | -3.47260857 | -3.11804795 |
| 1 | -0.17052504 | -2.31509018 | -0.90750909 |
| 1 | -0.49644160 | -0.79520291 | -1.76897109 |
| 8 | -3.56170940 | -5.07083797 | -6.46947527 |
| 8 | 1.46298516 | -1.51721454 | -1.93477380 |
| 1 | -7.16953993 | -5.96684170 | -6.33161592 |
| 1 | -5.58747911 | -6.75618505 | -6.68715429 |

Electronic energy = -3908.656336 a.u.
DFT-D3(BJ) dispersion correction = -0.138924 a.u.
Thermal free energy = 0.739400 a.u.
Gibbs free energy = -3908.055860 a.u.
Number of imaginary frequencies = 1.

**TS4 ax CL isomer/conformer 2**

| Atomic N. | X | Y | Z |
|---|---|---|---|
| 7 | 1.87621343 | 2.13028479 | 0.37630451 |
| 8 | 1.94191527 | -0.31653386 | 1.96593225 |
| 8 | -0.66813940 | 0.59408242 | 0.37595806 |
| 6 | 3.17261791 | 2.34411192 | -0.30885398 |
| 6 | 2.92656755 | 0.31459668 | 2.63435125 |
| 6 | 3.83215261 | -0.43693820 | 3.44415927 |
| 6 | 4.84350777 | 0.27392405 | 4.10569525 |
| 6 | 4.98648882 | 1.66188776 | 4.00940371 |
| 6 | 4.08159590 | 2.38517189 | 3.23965001 |

| | | | |
|---|---|---|---|
| 6 | 3.04938650 | 1.72445512 | 2.56555867 |
| 6 | 2.00515604 | 2.50904655 | 1.82805920 |
| 1 | 5.79301405 | 2.16722775 | 4.54070091 |
| 6 | 3.69576812 | -1.96386147 | 3.60237360 |
| 6 | -1.18090618 | 1.78301620 | 0.76230049 |
| 6 | -2.42385435 | 1.82761657 | 1.46513200 |
| 6 | -2.93396139 | 3.09081149 | 1.79558432 |
| 6 | -2.27326536 | 4.28223038 | 1.48339975 |
| 6 | -1.05012345 | 4.22549629 | 0.82577038 |
| 6 | -0.50228888 | 2.99125028 | 0.45805687 |
| 6 | 0.80808657 | 2.96528244 | -0.28329015 |
| 1 | -2.70832205 | 5.24068165 | 1.76612663 |
| 6 | -3.17394948 | 0.54194450 | 1.86691058 |
| 6 | 4.77324533 | -2.54440856 | 4.53722572 |
| 6 | 3.84349871 | -2.66390395 | 2.23116183 |
| 6 | 2.31626320 | -2.30571699 | 4.21471548 |
| 6 | -4.47740698 | 0.84728587 | 2.62898517 |
| 6 | -2.28863382 | -0.32052588 | 2.79761910 |
| 6 | -3.55778265 | -0.26728216 | 0.60663497 |
| 1 | 3.47363472 | 3.40180349 | -0.22211176 |
| 1 | 3.94208717 | 1.71949100 | 0.15713719 |
| 1 | 2.22879982 | 3.58850694 | 1.88363540 |
| 1 | 1.01381683 | 2.35336065 | 2.27646589 |
| 1 | 5.55418396 | -0.27273321 | 4.72311211 |
| 1 | 4.15705490 | 3.47287035 | 3.16828108 |
| 1 | 0.68253952 | 2.55899191 | -1.29856467 |
| 1 | 1.19006133 | 3.99594879 | -0.38157320 |
| 1 | -3.88124108 | 3.15422392 | 2.32781172 |
| 1 | -0.50766736 | 5.14274073 | 0.58537102 |
| 1 | 4.62334061 | -3.63014245 | 4.62292385 |
| 1 | 5.78991175 | -2.38044548 | 4.15073967 |
| 1 | 4.71411180 | -2.12163568 | 5.55063295 |
| 1 | 3.04731107 | -2.36168814 | 1.54258251 |
| 1 | 4.81921577 | -2.42955017 | 1.77902460 |
| 1 | 3.78817582 | -3.75537467 | 2.36453295 |
| 1 | 2.20962238 | -1.84687877 | 5.20859337 |
| 1 | 1.49858963 | -1.95431685 | 3.57600307 |
| 1 | 2.22166109 | -3.39604354 | 4.33316898 |
| 1 | -4.96774626 | -0.10134947 | 2.89019585 |
| 1 | -5.18598175 | 1.42996216 | 2.02251887 |
| 1 | -4.29134989 | 1.39113533 | 3.56624079 |
| 1 | -1.36116147 | -0.63075733 | 2.30512857 |
| 1 | -2.02917933 | 0.23667455 | 3.70938015 |
| 1 | -2.83787179 | -1.22517979 | 3.09991312 |
| 1 | -4.22278976 | 0.32268253 | -0.04173710 |
| 1 | -2.66695952 | -0.54942220 | 0.03617228 |
| 1 | -4.09313917 | -1.18339467 | 0.90042245 |
| 1 | 3.08445001 | 2.07945991 | -1.36634946 |
| 6 | 2.73509312 | -2.48793888 | -1.69088733 |
| 6 | 3.19173479 | -2.45975947 | -3.15360808 |
| 6 | 3.35077143 | -1.06999385 | -3.79645252 |
| 6 | 1.36829901 | -1.89110744 | -1.38236308 |
| 6 | 2.02188635 | -0.34686533 | -4.08246136 |

| | | | |
|---|---|---|---|
| 6 | 1.47175276 | 0.47431251 | -2.90749288 |
| 1 | 3.45774817 | -1.97067571 | -1.04538310 |
| 1 | 4.16234159 | -2.97869134 | -3.20040870 |
| 1 | 3.88085341 | -1.20588219 | -4.75194979 |
| 1 | 2.14902329 | 0.35550079 | -4.92191744 |
| 1 | 2.68979168 | -3.53235483 | -1.34015036 |
| 1 | 2.49490356 | -3.05473328 | -3.76443172 |
| 1 | 4.00240850 | -0.43771151 | -3.17014360 |
| 1 | 1.27716565 | -1.08734059 | -4.40478897 |
| 1 | 0.39717498 | 0.68194884 | -3.05752325 |
| 1 | 1.99229634 | 1.44724166 | -2.88608384 |
| 8 | 1.00263679 | -1.77565229 | -0.13299999 |
| 8 | 1.69348967 | -0.15523753 | -1.63805354 |
| 31 | 1.14761353 | 0.12831546 | 0.31530610 |
| 8 | -6.95536852 | 0.78393281 | -3.28806043 |
| 6 | -7.48802996 | 2.05497670 | -3.73105788 |
| 1 | -6.88655090 | 2.88195944 | -3.33341527 |
| 6 | -5.21832895 | -0.82718593 | -3.20099711 |
| 6 | -3.71117401 | -1.04204392 | -3.33741832 |
| 6 | -3.28683162 | -2.46130109 | -2.93994117 |
| 6 | -5.67135572 | 0.53800499 | -3.68167806 |
| 6 | -1.77829707 | -2.72177887 | -3.08024335 |
| 6 | -0.95368189 | -2.04312634 | -1.99510539 |
| 1 | -5.55289125 | -0.95909685 | -2.16083479 |
| 1 | -3.18981266 | -0.29429823 | -2.72166109 |
| 1 | -3.60330820 | -2.67168593 | -1.90389729 |
| 1 | -1.43466675 | -2.37573886 | -4.06870461 |
| 1 | -5.77314377 | -1.57841146 | -3.78953314 |
| 1 | -3.41212535 | -0.84312838 | -4.37791872 |
| 1 | -3.82609439 | -3.18363619 | -3.57439685 |
| 1 | -1.58140886 | -3.80391741 | -3.03675270 |
| 1 | -1.18033254 | -2.44843245 | -0.99951988 |
| 1 | -1.11904633 | -0.95567745 | -1.96322811 |
| 8 | -5.01097965 | 1.31943548 | -4.34000731 |
| 8 | 0.44535807 | -2.29586363 | -2.30376148 |
| 1 | -8.50860596 | 2.09367371 | -3.33829331 |
| 1 | -7.49063253 | 2.10979176 | -4.82705879 |

Electronic energy = -3908.655189 a.u.
DFT-D3(BJ) dispersion correction = -0.140787 a.u.
Thermal free energy = 0.740580 a.u.
Gibbs free energy = -3908.055396 a.u.
Number of imaginary frequencies = 1.

**TS4 ax CL isomer/conformer 3**

| Atomic N. | X | Y | Z |
|---|---|---|---|
| 7 | 1.99310875 | 2.66736269 | -0.12519661 |
| 8 | 1.98648036 | 0.30597743 | 1.58971274 |
| 8 | -0.59565991 | 1.20004618 | -0.04258772 |
| 6 | 3.28500533 | 2.80862188 | -0.83727717 |
| 6 | 3.00546026 | 0.93108350 | 2.21105146 |
| 6 | 3.89504886 | 0.18816869 | 3.04598475 |
| 6 | 4.94190454 | 0.89365548 | 3.65620804 |

| | | | |
|---|---|---|---|
| 6 | 5.13448000 | 2.26857853 | 3.48659587 |
| 6 | 4.24519396 | 2.98531461 | 2.69286299 |
| 6 | 3.17907929 | 2.32996821 | 2.06817436 |
| 6 | 2.15269613 | 3.11267567 | 1.30450976 |
| 1 | 5.96714783 | 2.76958871 | 3.98036194 |
| 6 | 3.70599127 | -1.32267964 | 3.28348947 |
| 6 | -1.07327497 | 2.41852641 | 0.29517347 |
| 6 | -2.31144309 | 2.52846575 | 0.99957174 |
| 6 | -2.78333735 | 3.81860566 | 1.27894092 |
| 6 | -2.09005594 | 4.97656059 | 0.91582865 |
| 6 | -0.87265319 | 4.85718155 | 0.25608867 |
| 6 | -0.36238843 | 3.59336424 | -0.06244855 |
| 6 | 0.94100499 | 3.50083375 | -0.81085199 |
| 1 | -2.49571872 | 5.95803213 | 1.16086054 |
| 6 | -3.09800363 | 1.28378975 | 1.45628941 |
| 6 | 4.77313042 | -1.89357972 | 4.23605537 |
| 6 | 3.81308699 | -2.09598827 | 1.94822204 |
| 6 | 2.32278967 | -1.58349168 | 3.92665434 |
| 6 | -4.38819408 | 1.65906823 | 2.20983672 |
| 6 | -2.23548818 | 0.43285191 | 2.41855097 |
| 6 | -3.51257372 | 0.43535343 | 0.23209421 |
| 1 | 3.61465836 | 3.86108327 | -0.81302679 |
| 1 | 4.04411793 | 2.19037700 | -0.34637022 |
| 1 | 2.41276908 | 4.18541765 | 1.30387890 |
| 1 | 1.16231203 | 3.01223516 | 1.77048540 |
| 1 | 5.64137125 | 0.35291117 | 4.29136992 |
| 1 | 4.35938025 | 4.06438828 | 2.56457090 |
| 1 | 0.79598701 | 3.05507803 | -1.80659652 |
| 1 | 1.34878874 | 4.51602364 | -0.95626342 |
| 1 | -3.72580338 | 3.93121934 | 1.81150043 |
| 1 | -0.30491036 | 5.74754524 | -0.02385554 |
| 1 | 4.58503485 | -2.96719813 | 4.37932396 |
| 1 | 5.79034042 | -1.78723645 | 3.83125520 |
| 1 | 4.74151421 | -1.41743743 | 5.22678947 |
| 1 | 3.02205992 | -1.79968619 | 1.25109637 |
| 1 | 4.79244471 | -1.92234397 | 1.47694218 |
| 1 | 3.71794391 | -3.17635536 | 2.13769221 |
| 1 | 2.24456525 | -1.07142055 | 4.89690256 |
| 1 | 1.50999498 | -1.23613477 | 3.27966762 |
| 1 | 2.19141364 | -2.66226649 | 4.10154772 |
| 1 | -4.90471840 | 0.73664051 | 2.51114035 |
| 1 | -5.08242846 | 2.23752022 | 1.58296871 |
| 1 | -4.18154716 | 2.23465347 | 3.12352252 |
| 1 | -1.31875837 | 0.07668680 | 1.93729722 |
| 1 | -1.95753217 | 1.01734948 | 3.30746245 |
| 1 | -2.80966592 | -0.44332436 | 2.75576544 |
| 1 | -4.15963364 | 1.01696718 | -0.44152075 |
| 1 | -2.63357663 | 0.09835687 | -0.32673737 |
| 1 | -4.07537079 | -0.44927597 | 0.56825286 |
| 1 | 3.17539215 | 2.48715901 | -1.87695742 |
| 6 | 2.73299289 | -2.02669644 | -2.01135325 |
| 6 | 3.15818739 | -2.06075954 | -3.48347425 |
| 6 | 3.31268001 | -0.69946361 | -4.18585348 |

| | | | |
|---|---|---|---|
| 6 | 1.37798584 | -1.40639424 | -1.69817758 |
| 6 | 1.98235953 | 0.01970770 | -4.47532463 |
| 6 | 1.45759904 | 0.88818091 | -3.32196498 |
| 1 | 3.47376227 | -1.49106121 | -1.40228570 |
| 1 | 4.12390137 | -2.58869004 | -3.52994156 |
| 1 | 3.82271194 | -0.87782258 | -5.14530134 |
| 1 | 2.09744978 | 0.68753272 | -5.34430599 |
| 1 | 2.68624377 | -3.05613947 | -1.61887133 |
| 1 | 2.44419432 | -2.67462516 | -4.05456257 |
| 1 | 3.98032689 | -0.04632740 | -3.59920907 |
| 1 | 1.22884130 | -0.72957325 | -4.75396538 |
| 1 | 0.37781441 | 1.08351386 | -3.45120525 |
| 1 | 1.97193933 | 1.86432934 | -3.35578299 |
| 8 | 1.04050422 | -1.23428082 | -0.44746029 |
| 8 | 1.71777749 | 0.31490314 | -2.03471565 |
| 31 | 1.20793748 | 0.68596733 | -0.08415802 |
| 8 | -7.34983635 | -3.37455320 | -0.67860079 |
| 6 | -8.01162815 | -4.17058897 | 0.33353117 |
| 1 | -7.88449717 | -5.24033594 | 0.12490056 |
| 6 | -5.40282774 | -2.70007586 | -1.85015142 |
| 6 | -3.89319181 | -2.87902665 | -2.00636721 |
| 6 | -3.32124972 | -1.98643255 | -3.11475253 |
| 6 | -6.00248909 | -3.57969713 | -0.77026647 |
| 6 | -1.81243634 | -2.15933800 | -3.35089827 |
| 6 | -0.96118248 | -1.61112416 | -2.21410656 |
| 1 | -5.93000507 | -2.91830587 | -2.79354906 |
| 1 | -3.67293978 | -3.93477130 | -2.22917533 |
| 1 | -3.84607720 | -2.20971751 | -4.05827999 |
| 1 | -1.52717018 | -1.64412236 | -4.28157520 |
| 1 | -5.65483189 | -1.65452826 | -1.60859406 |
| 1 | -3.40858555 | -2.66606331 | -1.04286063 |
| 1 | -3.53938246 | -0.92847008 | -2.88861346 |
| 1 | -1.57693911 | -3.22633147 | -3.49380493 |
| 1 | -1.15623486 | -2.11521554 | -1.25882947 |
| 1 | -1.13061678 | -0.53256077 | -2.06935072 |
| 8 | -5.39496613 | -4.36532879 | -0.06820315 |
| 8 | 0.42987129 | -1.83568203 | -2.58123350 |
| 1 | -9.06730938 | -3.88770151 | 0.27970862 |
| 1 | -7.59964371 | -3.94919872 | 1.32612395 |

Electronic energy = -3908.655040 a.u.
DFT-D3(BJ) dispersion correction = -0.140493 a.u.
Thermal free energy = 0.740476 a.u.
Gibbs free energy = -3908.055058 a.u.
Number of imaginary frequencies = 1.

**TS4 ax CL isomer/conformer 4**

| Atomic N. | X | Y | Z |
|---|---|---|---|
| 7 | 2.03943491 | 2.61749172 | 0.51584995 |
| 8 | 2.02057838 | 0.18644181 | 2.12963033 |
| 8 | -0.53335321 | 1.12179613 | 0.47219530 |
| 6 | 3.34830284 | 2.80084014 | -0.15456423 |
| 6 | 3.01340032 | 0.79837018 | 2.80412793 |

| | | | |
|---|---|---|---|
| 6 | 3.89105988 | 0.03234494 | 3.63077760 |
| 6 | 4.91074371 | 0.72479284 | 4.29911995 |
| 6 | 5.08786583 | 2.10811758 | 4.19330168 |
| 6 | 4.21026945 | 2.84616542 | 3.40613055 |
| 6 | 3.17089868 | 2.20418549 | 2.72503686 |
| 6 | 2.15539193 | 3.00562286 | 1.96615684 |
| 1 | 5.89955711 | 2.59863997 | 4.73058844 |
| 6 | 3.71808767 | -1.48982549 | 3.79785013 |
| 6 | -1.03510725 | 2.32128716 | 0.84108275 |
| 6 | -2.29569149 | 2.39062810 | 1.51006842 |
| 6 | -2.79049039 | 3.66385603 | 1.82501292 |
| 6 | -2.09974527 | 4.84228897 | 1.52908552 |
| 6 | -0.86189795 | 4.76154566 | 0.90240318 |
| 6 | -0.32771161 | 3.51655078 | 0.55058455 |
| 6 | 0.99697757 | 3.46767259 | -0.16389690 |
| 1 | -2.52387571 | 5.80912018 | 1.79969013 |
| 6 | -3.08252120 | 1.12081075 | 1.89159262 |
| 6 | 4.76765585 | -2.08708882 | 4.75371218 |
| 6 | 3.87225151 | -2.20268226 | 2.43383622 |
| 6 | 2.32216072 | -1.79719520 | 4.39100170 |
| 6 | -4.40081978 | 1.45343900 | 2.61580086 |
| 6 | -2.24134183 | 0.24270731 | 2.84837842 |
| 6 | -3.44584107 | 0.31666934 | 0.62220442 |
| 1 | 3.66539097 | 3.85468268 | -0.07866504 |
| 1 | 4.10090017 | 2.17068768 | 0.33117968 |
| 1 | 2.40213203 | 4.08034849 | 2.01623893 |
| 1 | 1.15418971 | 2.87522554 | 2.40062261 |
| 1 | 5.60064697 | 0.16672835 | 4.92974234 |
| 1 | 4.31282711 | 3.93106699 | 3.32659388 |
| 1 | 0.88442558 | 3.06195235 | -1.18073606 |
| 1 | 1.39711702 | 4.49206829 | -0.25636381 |
| 1 | -3.75015283 | 3.74584699 | 2.33183527 |
| 1 | -0.29663804 | 5.66809368 | 0.67417073 |
| 1 | 4.59190416 | -3.16844916 | 4.84524822 |
| 1 | 5.79361677 | -1.94921350 | 4.38190889 |
| 1 | 4.70248222 | -1.65504348 | 5.76284027 |
| 1 | 3.09636426 | -1.88597035 | 1.72867942 |
| 1 | 4.86146975 | -1.99600315 | 1.99788499 |
| 1 | 3.78724837 | -3.29147816 | 2.57352018 |
| 1 | 2.21031976 | -1.32876551 | 5.37983227 |
| 1 | 1.52207720 | -1.43303692 | 3.73740292 |
| 1 | 2.20235848 | -2.88430524 | 4.51586008 |
| 1 | -4.91717291 | 0.51503354 | 2.86335564 |
| 1 | -5.08020353 | 2.04908681 | 1.98884535 |
| 1 | -4.23122120 | 1.99481118 | 3.55763578 |
| 1 | -1.30636621 | -0.08615398 | 2.38283992 |
| 1 | -1.99732792 | 0.79557401 | 3.76702023 |
| 1 | -2.81667471 | -0.65078813 | 3.13485527 |
| 1 | -4.07509089 | 0.91754860 | -0.05117851 |
| 1 | -2.54480100 | 0.01007264 | 0.08176459 |
| 1 | -4.01187944 | -0.58542866 | 0.90110672 |
| 1 | 3.27022457 | 2.52135134 | -1.20916796 |
| 6 | 2.85157561 | -2.00831985 | -1.55005550 |

| | | | |
|---|---|---|---|
| 6 | 3.30506349 | -1.97865272 | -3.01378012 |
| 6 | 3.47198677 | -0.58893508 | -3.65468812 |
| 6 | 1.49189305 | -1.40013623 | -1.23447418 |
| 6 | 2.14658189 | 0.14193378 | -3.93726444 |
| 6 | 1.60428441 | 0.96478200 | -2.75940490 |
| 1 | 3.58126140 | -1.50530684 | -0.90108252 |
| 1 | 4.27163982 | -2.50460529 | -3.06434011 |
| 1 | 3.99919057 | -0.72709757 | -4.61142445 |
| 1 | 2.27566814 | 0.84362561 | -4.77705479 |
| 1 | 2.79423928 | -3.05466104 | -1.20616841 |
| 1 | 2.60236192 | -2.56719637 | -3.62417793 |
| 1 | 4.12867355 | 0.03858516 | -3.02898574 |
| 1 | 1.39727211 | -0.59477258 | -4.25762272 |
| 1 | 0.52834064 | 1.17210782 | -2.90136695 |
| 1 | 2.12507915 | 1.93797982 | -2.74276328 |
| 8 | 1.13349187 | -1.27968776 | 0.01606334 |
| 8 | 1.83572280 | 0.33647427 | -1.49216044 |
| 31 | 1.27630341 | 0.62826186 | 0.45523772 |
| 8 | -7.72828197 | -2.94180775 | -4.26560545 |
| 6 | -8.64272690 | -3.43701100 | -5.27289152 |
| 1 | -8.53933048 | -2.86184883 | -6.20165777 |
| 6 | -5.53681803 | -2.46543074 | -3.49874783 |
| 6 | -4.04086971 | -2.59886646 | -3.78126669 |
| 6 | -3.18089581 | -2.01344681 | -2.65646553 |
| 6 | -6.40899134 | -3.01682067 | -4.61010265 |
| 6 | -1.67888093 | -2.15519524 | -2.92899370 |
| 6 | -0.83713037 | -1.57443571 | -1.80364013 |
| 1 | -5.81774616 | -1.40979946 | -3.34824276 |
| 1 | -3.80568433 | -2.09926009 | -4.73342752 |
| 1 | -3.43259454 | -0.94831246 | -2.51734161 |
| 1 | -1.42284846 | -1.65193772 | -3.87546062 |
| 1 | -5.81433153 | -2.97745156 | -2.56341982 |
| 1 | -3.79205751 | -3.66125488 | -3.92831659 |
| 1 | -3.42911530 | -2.51301455 | -1.70443797 |
| 1 | -1.42168999 | -3.21905899 | -3.05533290 |
| 1 | -1.04462194 | -2.06411910 | -0.84215599 |
| 1 | -1.01568699 | -0.49521393 | -1.67669880 |
| 8 | -6.01786995 | -3.46541476 | -5.67100477 |
| 8 | 0.55929297 | -1.79216707 | -2.15098763 |
| 1 | -9.64168262 | -3.30733514 | -4.84559774 |
| 1 | -8.44447327 | -4.49531221 | -5.48432159 |

Electronic energy = -3908.656304 a.u.
DFT-D3(BJ) dispersion correction = -0.138892 a.u.
Thermal free energy = 0.741751 a.u.
Gibbs free energy = -3908.053445 a.u.
Number of imaginary frequencies = 1.

**TS4_ax_CL isomer/conformer 5**

| Atomic N. | X | Y | Z |
|---|---|---|---|
| 7 | 1.58389807 | 2.50981259 | 1.16195309 |
| 8 | 1.50870287 | -0.00378813 | 2.64006305 |
| 8 | -0.97966635 | 1.00769901 | 0.92740971 |

| | | | |
|---|---|---|---|
| 6 | 2.90988684 | 2.73564529 | 0.53985697 |
| 6 | 2.48740578 | 0.56301373 | 3.37223506 |
| 6 | 3.34206653 | -0.25185910 | 4.17574739 |
| 6 | 4.34515285 | 0.39839441 | 4.90864038 |
| 6 | 4.52784252 | 1.78489745 | 4.88602781 |
| 6 | 3.67326784 | 2.56946874 | 4.11866665 |
| 6 | 2.65079594 | 1.97028542 | 3.37592030 |
| 6 | 1.65724480 | 2.81442761 | 2.63507414 |
| 1 | 5.32599115 | 2.24128389 | 5.47152042 |
| 6 | 3.16406727 | -1.78074551 | 4.24899101 |
| 6 | -1.50324917 | 2.17739773 | 1.35746002 |
| 6 | -2.79149532 | 2.19781113 | 1.97485304 |
| 6 | -3.30180883 | 3.44460654 | 2.36252427 |
| 6 | -2.60295153 | 4.64149237 | 2.18213010 |
| 6 | -1.34234142 | 4.60741997 | 1.59801328 |
| 6 | -0.79078609 | 3.39132977 | 1.17827690 |
| 6 | 0.55633432 | 3.39554501 | 0.50510359 |
| 1 | -3.04012322 | 5.58577108 | 2.50631046 |
| 6 | -3.59488297 | 0.90472782 | 2.21941686 |
| 6 | 4.18563366 | -2.43453026 | 5.19822025 |
| 6 | 3.35839581 | -2.41204882 | 2.85025954 |
| 6 | 1.75180924 | -2.12061214 | 4.78263426 |
| 6 | -4.94142151 | 1.18334198 | 2.91395807 |
| 6 | -2.79668975 | -0.05417276 | 3.13438678 |
| 6 | -3.90748715 | 0.21182036 | 0.87279785 |
| 1 | 3.21749616 | 3.78567481 | 0.68017662 |
| 1 | 3.65258574 | 2.08469892 | 1.01335430 |
| 1 | 1.90091956 | 3.88453913 | 2.75294113 |
| 1 | 0.64381820 | 2.65952134 | 3.03147721 |
| 1 | 5.01731396 | -0.19685732 | 5.52425671 |
| 1 | 3.78029490 | 3.65674949 | 4.10278511 |
| 1 | 0.47824731 | 3.05458570 | -0.53829199 |
| 1 | 0.95168388 | 4.42586517 | 0.49055535 |
| 1 | -4.28164291 | 3.48931646 | 2.83417988 |
| 1 | -0.77266413 | 5.52871943 | 1.45609951 |
| 1 | 4.00696898 | -3.51902366 | 5.22168875 |
| 1 | 5.22192097 | -2.27741575 | 4.86465788 |
| 1 | 4.09156895 | -2.06163120 | 6.22845602 |
| 1 | 2.60388494 | -2.05389977 | 2.14166713 |
| 1 | 4.36010313 | -2.18112278 | 2.45694947 |
| 1 | 3.27028465 | -3.50710917 | 2.92304230 |
| 1 | 1.61150122 | -1.71119881 | 5.79379129 |
| 1 | 0.97057068 | -1.71752095 | 4.12914658 |
| 1 | 1.62900233 | -3.21303010 | 4.83974218 |
| 1 | -5.46878052 | 0.23033558 | 3.06277132 |
| 1 | -5.59396267 | 1.83098495 | 2.31055212 |
| 1 | -4.80950403 | 1.64543653 | 3.90291071 |
| 1 | -1.84434414 | -0.35018137 | 2.68241429 |
| 1 | -2.58856249 | 0.42033213 | 4.10425949 |
| 1 | -3.38671708 | -0.96383423 | 3.32344580 |
| 1 | -4.51518917 | 0.86639172 | 0.23073450 |
| 1 | -2.98544264 | -0.04352531 | 0.34102872 |
| 1 | -4.47794819 | -0.71248251 | 1.05157220 |

| | | | |
|---|---|---|---|
| 1 | 2.86354351 | 2.51154184 | -0.52978158 |
| 6 | 2.45837498 | -1.99068213 | -1.18712878 |
| 6 | 2.91736126 | -1.87431324 | -2.64443064 |
| 6 | 3.09562421 | -0.44792110 | -3.19503880 |
| 6 | 1.09753489 | -1.40351081 | -0.83880788 |
| 6 | 1.77571273 | 0.30639860 | -3.43768358 |
| 6 | 1.22937322 | 1.05766332 | -2.21532393 |
| 1 | 3.18492055 | -1.52179933 | -0.50977534 |
| 1 | 3.88081312 | -2.40233183 | -2.72416472 |
| 1 | 3.62868690 | -0.52705735 | -4.15526676 |
| 1 | 1.91230226 | 1.05788410 | -4.23199129 |
| 1 | 2.40300560 | -3.05492783 | -0.90364730 |
| 1 | 2.21350622 | -2.41914749 | -3.29312348 |
| 1 | 3.75124645 | 0.13474439 | -2.52631807 |
| 1 | 1.02421510 | -0.40487707 | -3.80720806 |
| 1 | 0.15766147 | 1.28642917 | -2.35615063 |
| 1 | 1.76175499 | 2.02126217 | -2.13134575 |
| 8 | 0.74391323 | -1.35789168 | 0.41776824 |
| 8 | 1.43831086 | 0.34787434 | -0.98829120 |
| 31 | 0.83291411 | 0.52584809 | 0.96153402 |
| 8 | -3.68138170 | -1.73361957 | -8.47322559 |
| 6 | -4.52240419 | -2.29924655 | -9.50719357 |
| 1 | -4.27394152 | -3.35568118 | -9.66919231 |
| 6 | -2.87427735 | -1.68066156 | -6.24529457 |
| 6 | -2.96286035 | -2.29729486 | -4.84987593 |
| 6 | -2.01971960 | -1.61970747 | -3.85079598 |
| 6 | -3.79350471 | -2.34099793 | -7.25486231 |
| 6 | -2.10231733 | -2.23370886 | -2.44922352 |
| 6 | -1.23463190 | -1.55107582 | -1.40495145 |
| 1 | -1.84671772 | -1.73957050 | -6.64038229 |
| 1 | -2.73239279 | -3.37189960 | -4.91297245 |
| 1 | -0.98305660 | -1.68894041 | -4.21850300 |
| 1 | -1.83941531 | -3.30319023 | -2.49077916 |
| 1 | -3.11763668 | -0.60587007 | -6.22167683 |
| 1 | -4.00119972 | -2.23282099 | -4.48944378 |
| 1 | -2.25859022 | -0.54322189 | -3.78878736 |
| 1 | -3.14240360 | -2.18154049 | -2.08594704 |
| 1 | -1.39466751 | -1.97562492 | -0.40594277 |
| 1 | -1.44139171 | -0.47016361 | -1.35213757 |
| 8 | -4.53546095 | -3.28038335 | -7.04017258 |
| 8 | 0.16219856 | -1.74294150 | -1.77488494 |
| 1 | -4.31353855 | -1.70956850 | -10.40495777 |
| 1 | -5.58046293 | -2.22020936 | -9.22743988 |

Electronic energy = -3908.656082 a.u.
DFT-D3(BJ) dispersion correction = -0.139156 a.u.
Thermal free energy = 0.742775 a.u.
Gibbs free energy = -3908.052463 a.u.
Number of imaginary frequencies = 1.

**TS4 ax CL isomer/conformer 6**

| Atomic N. | X | Y | Z |
|---|---|---|---|
| 7 | 2.23418689 | 2.51246572 | 0.19782174 |

| | | | |
|---|---|---|---|
| 8 | 1.81266975 | 0.09655925 | 1.78036177 |
| 8 | -0.49887836 | 1.30318570 | -0.03214705 |
| 6 | 3.55165577 | 2.56932855 | -0.47767034 |
| 6 | 2.89229584 | 0.52292866 | 2.46491575 |
| 6 | 3.62654233 | -0.38201198 | 3.29092336 |
| 6 | 4.74513769 | 0.12911366 | 3.96504664 |
| 6 | 5.15032244 | 1.46417069 | 3.86750555 |
| 6 | 4.40754795 | 2.34295249 | 3.08573079 |
| 6 | 3.27858877 | 1.88463867 | 2.39964294 |
| 6 | 2.39764905 | 2.84322429 | 1.65691650 |
| 1 | 6.03081656 | 1.80976331 | 4.40927792 |
| 6 | 3.20427036 | -1.85521305 | 3.45130515 |
| 6 | -0.88234669 | 2.51923084 | 0.42213243 |
| 6 | -2.16021585 | 2.68360519 | 1.03851032 |
| 6 | -2.50720358 | 3.97142792 | 1.47032654 |
| 6 | -1.66453242 | 5.07665157 | 1.32132649 |
| 6 | -0.42483804 | 4.90546417 | 0.71704304 |
| 6 | -0.03097901 | 3.64258718 | 0.25912678 |
| 6 | 1.29266524 | 3.49937916 | -0.44347402 |
| 1 | -1.97769070 | 6.05733776 | 1.67942441 |
| 6 | -3.13327193 | 1.50225282 | 1.21190774 |
| 6 | 4.12379789 | -2.61529636 | 4.42559004 |
| 6 | 3.26786494 | -2.58248186 | 2.08793402 |
| 6 | 1.76645958 | -1.93168032 | 4.01818800 |
| 6 | -4.43767309 | 1.92233086 | 1.91594505 |
| 6 | -2.49127483 | 0.38751543 | 2.07151747 |
| 6 | -3.52309823 | 0.94392163 | -0.17660186 |
| 1 | 3.97510290 | 3.58446431 | -0.39214647 |
| 1 | 4.23738241 | 1.86009538 | -0.00137300 |
| 1 | 2.79608417 | 3.86998129 | 1.73398697 |
| 1 | 1.38513839 | 2.84562135 | 2.08452392 |
| 1 | 5.33064365 | -0.53877324 | 4.59453344 |
| 1 | 4.68535614 | 3.39748621 | 3.01649833 |
| 1 | 1.15232277 | 3.16158056 | -1.48098326 |
| 1 | 1.79666948 | 4.48082924 | -0.47589976 |
| 1 | -3.47277093 | 4.12288332 | 1.94934916 |
| 1 | 0.25097305 | 5.75414801 | 0.58831137 |
| 1 | 3.76911497 | -3.65217042 | 4.51402950 |
| 1 | 5.16464281 | -2.65145659 | 4.07176924 |
| 1 | 4.11393404 | -2.17512679 | 5.43332386 |
| 1 | 2.57452822 | -2.14042044 | 1.36444759 |
| 1 | 4.28829956 | -2.54466176 | 1.67697895 |
| 1 | 2.99834132 | -3.64188552 | 2.22061777 |
| 1 | 1.71431446 | -1.45158005 | 5.00642395 |
| 1 | 1.04779708 | -1.44197452 | 3.35219336 |
| 1 | 1.46963286 | -2.98496127 | 4.13759089 |
| 1 | -5.09090281 | 1.04266131 | 2.00207877 |
| 1 | -4.98701000 | 2.68879557 | 1.34984934 |
| 1 | -4.25605392 | 2.30263901 | 2.93180370 |
| 1 | -1.58095479 | -0.00934790 | 1.61114538 |
| 1 | -2.23658395 | 0.76841110 | 3.07133436 |
| 1 | -3.20573449 | -0.44005176 | 2.19332218 |
| 1 | -4.04427862 | 1.71167421 | -0.76830262 |

| | | | |
|---|---|---|---|
| 1 | -2.63522077 | 0.62238699 | -0.73159832 |
| 1 | -4.19570112 | 0.08179584 | -0.05991181 |
| 1 | 3.44127083 | 2.30853868 | -1.53435624 |
| 6 | 2.72638845 | -2.02592111 | -1.98790765 |
| 6 | 3.16911697 | -1.97564006 | -3.45504475 |
| 6 | 3.36937928 | -0.57847673 | -4.07145166 |
| 6 | 1.37733161 | -1.40322590 | -1.65652394 |
| 6 | 2.06668997 | 0.20318469 | -4.32536745 |
| 6 | 1.56858933 | 1.01956236 | -3.12297940 |
| 1 | 3.46851850 | -1.54512000 | -1.33642721 |
| 1 | 4.12105131 | -2.52568436 | -3.52502489 |
| 1 | 3.88144350 | -0.71623421 | -5.03658772 |
| 1 | 2.21145892 | 0.91486275 | -5.15409660 |
| 1 | 2.65514898 | -3.07810593 | -1.66406751 |
| 1 | 2.44617581 | -2.53320289 | -4.07099771 |
| 1 | 4.05363703 | 0.01540202 | -3.44249082 |
| 1 | 1.28796625 | -0.50067037 | -4.64989042 |
| 1 | 0.49891192 | 1.26891351 | -3.24151945 |
| 1 | 2.12484336 | 1.97358501 | -3.09200215 |
| 8 | 1.01643324 | -1.28181469 | -0.41137540 |
| 8 | 1.79841554 | 0.34678286 | -1.88164127 |
| 31 | 1.24542046 | 0.62455547 | 0.05736354 |
| 8 | -7.16262627 | -2.47635555 | -1.83826768 |
| 6 | -7.98557186 | -1.56391525 | -1.07011425 |
| 1 | -8.12354565 | -1.93976796 | -0.04856618 |
| 6 | -5.14369774 | -3.69153333 | -2.15575051 |
| 6 | -3.63756561 | -3.40953827 | -2.22356153 |
| 6 | -3.28669024 | -2.20248222 | -3.10577989 |
| 6 | -5.91102600 | -2.67321134 | -1.33354592 |
| 6 | -1.78341925 | -2.06001759 | -3.39627624 |
| 6 | -0.96020997 | -1.61764717 | -2.19343805 |
| 1 | -5.31755924 | -4.66557932 | -1.66803586 |
| 1 | -3.13917637 | -4.30590153 | -2.62700701 |
| 1 | -3.80962396 | -2.30833268 | -4.07065821 |
| 1 | -1.63259017 | -1.33742535 | -4.21339273 |
| 1 | -5.58775139 | -3.75867987 | -3.15895391 |
| 1 | -3.26124048 | -3.26624346 | -1.20045567 |
| 1 | -3.67189360 | -1.27778184 | -2.64581847 |
| 1 | -1.38958240 | -3.02584672 | -3.75333738 |
| 1 | -1.13423669 | -2.24457002 | -1.30901194 |
| 1 | -1.16829562 | -0.57406205 | -1.91544569 |
| 8 | -5.49672604 | -2.11719728 | -0.33298987 |
| 8 | 0.43914822 | -1.74867666 | -2.58213997 |
| 1 | -8.94147205 | -1.52068758 | -1.60065341 |
| 1 | -7.51837254 | -0.57223362 | -1.02683461 |

Electronic energy = -3908.653921 a.u.
DFT-D3(BJ) dispersion correction = -0.140677 a.u.
Thermal free energy = 0.744005 a.u.
Gibbs free energy = -3908.050593 a.u.
Number of imaginary frequencies = 1.

**Int4_CL isomer/conformer 1**

| Atomic N. | X | Y | Z |
|---|---|---|---|
| 7 | 2.01584000 | 3.00620725 | 2.31117174 |
| 8 | 1.79212831 | 0.11292514 | 2.05111484 |
| 8 | -0.41482604 | 2.22637427 | 0.82157669 |
| 6 | 3.47150325 | 3.24923591 | 2.18430703 |
| 6 | 2.36852090 | 0.11331633 | 3.27315692 |
| 6 | 2.96208956 | -1.09658628 | 3.75436296 |
| 6 | 3.55367884 | -1.07262718 | 5.02423382 |
| 6 | 3.58566678 | 0.07330510 | 5.82285547 |
| 6 | 2.99703804 | 1.24012747 | 5.35034214 |
| 6 | 2.37771504 | 1.26980588 | 4.09537872 |
| 6 | 1.65853301 | 2.52553126 | 3.68114772 |
| 1 | 4.06385836 | 0.04787680 | 6.80207280 |
| 6 | 2.96025731 | -2.38802938 | 2.91189401 |
| 6 | -0.98973818 | 3.19402955 | 1.56247886 |
| 6 | -2.41165395 | 3.20533288 | 1.71004689 |
| 6 | -2.97639528 | 4.23366603 | 2.47618270 |
| 6 | -2.20955111 | 5.22509360 | 3.09512754 |
| 6 | -0.82776622 | 5.20365119 | 2.94566964 |
| 6 | -0.21189949 | 4.20837625 | 2.17780737 |
| 6 | 1.27929009 | 4.26835591 | 1.97363028 |
| 1 | -2.69238588 | 6.00126074 | 3.68864474 |
| 6 | -3.29647886 | 2.12687830 | 1.05330593 |
| 6 | 3.60977224 | -3.57029331 | 3.65589478 |
| 6 | 3.77124190 | -2.16231983 | 1.61513363 |
| 6 | 1.50988705 | -2.80791924 | 2.57221088 |
| 6 | -4.79281351 | 2.35549552 | 1.33837395 |
| 6 | -2.93005777 | 0.72970840 | 1.60778963 |
| 6 | -3.11350785 | 2.15114985 | -0.48309822 |
| 1 | 3.78686200 | 4.04571725 | 2.87971448 |
| 1 | 4.02073312 | 2.33167101 | 2.42127516 |
| 1 | 1.86818153 | 3.32804477 | 4.41052574 |
| 1 | 0.56857012 | 2.36997081 | 3.67957039 |
| 1 | 4.01309737 | -1.98092933 | 5.40965182 |
| 1 | 2.99834414 | 2.14624509 | 5.96058981 |
| 1 | 1.52756561 | 4.48527278 | 0.92171642 |
| 1 | 1.69426448 | 5.08910561 | 2.58500356 |
| 1 | -4.05687151 | 4.26532829 | 2.60495417 |
| 1 | -0.20857740 | 5.96966467 | 3.41830600 |
| 1 | 3.57213497 | -4.46080828 | 3.01188643 |
| 1 | 4.66605626 | -3.38132611 | 3.89551971 |
| 1 | 3.07946366 | -3.81312111 | 4.58795958 |
| 1 | 3.36724022 | -1.32521192 | 1.03711324 |
| 1 | 4.82289598 | -1.94382074 | 1.85169166 |
| 1 | 3.74630269 | -3.06918289 | 0.99072759 |
| 1 | 0.94751665 | -3.02410906 | 3.49235002 |
| 1 | 0.98192285 | -2.02142565 | 2.02258509 |
| 1 | 1.52130408 | -3.72490379 | 1.96242080 |
| 1 | -5.37553669 | 1.56468764 | 0.84433866 |
| 1 | -5.14645355 | 3.32035744 | 0.94709466 |
| 1 | -5.02150012 | 2.31100947 | 2.41295853 |
| 1 | -1.88756795 | 0.47235383 | 1.39280636 |
| 1 | -3.08346872 | 0.69312434 | 2.69617294 |

```
   1    -3.58097778  -0.03326071   1.15214567
   1    -3.41164235   3.12738818  -0.89274347
   1    -2.07217068   1.95771057  -0.76291847
   1    -3.75394359   1.38358012  -0.94581561
   1     3.69218894   3.53799472   1.15095197
   6     2.21146161  -0.95796622  -1.68864347
   6     2.31060846  -0.48394072  -3.16295343
   6     2.56502567   1.02102219  -3.33915281
   6     0.81700917  -0.90826797  -1.11714703
   6     1.47674474   1.96418549  -2.77750646
   6     1.93159210   2.77886739  -1.55863086
   1     2.86358177  -0.34552401  -1.05610891
   1     3.14352917  -1.03108812  -3.62994801
   1     2.69658466   1.20577544  -4.41665880
   1     1.17147652   2.69020622  -3.54941419
   1     2.53148493  -2.00840278  -1.62816152
   1     1.40355837  -0.78529413  -3.70963505
   1     3.52878370   1.26666228  -2.86483402
   1     0.56411637   1.40376157  -2.52093416
   1     1.09060554   3.42497832  -1.24166333
   1     2.75212657   3.45023557  -1.87836515
   8     0.39002196  -0.14216174  -0.24895336
   8     2.43272574   1.98810132  -0.48297432
  31     1.33118798   1.54372868   0.93000991
   8    -3.99054835  -4.42094230  -7.70309145
   6    -4.69463047  -5.41831051  -8.48196361
   1    -4.19294520  -6.39096529  -8.40339240
   6    -3.15994066  -3.59452145  -5.64333678
   6    -3.00554047  -3.85436836  -4.14542619
   6    -2.24695206  -2.73032473  -3.43307205
   6    -3.90847363  -4.69659698  -6.36859117
   6    -2.08707822  -2.98694981  -1.93035360
   6    -1.36473271  -1.87980856  -1.18379051
   1    -2.17825597  -3.47681775  -6.13027405
   1    -2.48315666  -4.81185781  -3.99577256
   1    -1.25142771  -2.60863974  -3.88974493
   1    -1.55990853  -3.93982159  -1.76207967
   1    -3.69446495  -2.64848822  -5.82945402
   1    -4.00190398  -3.98152597  -3.69463658
   1    -2.77658482  -1.77309267  -3.58173072
   1    -3.08056337  -3.09431325  -1.46384091
   1    -1.34295894  -2.05759545  -0.10022435
   1    -1.81819694  -0.89405685  -1.36091418
   8    -4.38065636  -5.69491183  -5.85885651
   8     0.01952728  -1.84085933  -1.66673838
   1    -4.66980520  -5.05105829  -9.51235575
   1    -5.72890798  -5.52010743  -8.13005101
Electronic energy = -3908.677004 a.u.
DFT-D3(BJ) dispersion correction = -0.137259 a.u.
Thermal free energy = 0.736134 a.u.
Gibbs free energy = -3908.078129 a.u.
Number of imaginary frequencies = 0.
```

**Int4_CL isomer/conformer 2**

| Atomic N. | X | Y | Z |
|---|---|---|---|
| 7 | 1.17605561 | 3.70699554 | 1.82079504 |
| 8 | 1.69330168 | 0.84179431 | 1.86682092 |
| 8 | -0.59725067 | 2.24723714 | -0.03688464 |
| 6 | 2.52627426 | 4.29518715 | 1.97950563 |
| 6 | 1.94732316 | 1.06494438 | 3.17481775 |
| 6 | 2.67465274 | 0.07770428 | 3.91252741 |
| 6 | 2.92612603 | 0.33016235 | 5.26756003 |
| 6 | 2.50020917 | 1.49407416 | 5.91235012 |
| 6 | 1.78612662 | 2.44190175 | 5.18917859 |
| 6 | 1.49317274 | 2.23557402 | 3.83606827 |
| 6 | 0.62406113 | 3.24161423 | 3.12990810 |
| 1 | 2.72500568 | 1.65186633 | 6.96714143 |
| 6 | 3.17250899 | -1.22217377 | 3.24911264 |
| 6 | -1.53252431 | 3.09215173 | 0.43893932 |
| 6 | -2.91424684 | 2.76090672 | 0.28065765 |
| 6 | -3.86019769 | 3.66839435 | 0.77544539 |
| 6 | -3.50446455 | 4.86039990 | 1.41305283 |
| 6 | -2.15799024 | 5.17137615 | 1.56311543 |
| 6 | -1.17056593 | 4.30827224 | 1.07367309 |
| 6 | 0.27370383 | 4.72200725 | 1.18467259 |
| 1 | -4.27553951 | 5.53284128 | 1.78879794 |
| 6 | -3.35342439 | 1.45303126 | -0.40783927 |
| 6 | 3.88507687 | -2.15130477 | 4.25017231 |
| 6 | 4.19094272 | -0.88066926 | 2.13714389 |
| 6 | 1.98185463 | -2.01699071 | 2.66094325 |
| 6 | -4.88679066 | 1.32253226 | -0.47479202 |
| 6 | -2.82141660 | 0.23304321 | 0.38110548 |
| 6 | -2.82583424 | 1.41403191 | -1.86193474 |
| 1 | 2.47947600 | 5.18795007 | 2.62618770 |
| 1 | 3.19759989 | 3.55979909 | 2.43585146 |
| 1 | 0.46541216 | 4.11613453 | 3.78566191 |
| 1 | -0.36898381 | 2.81807331 | 2.91390373 |
| 1 | 3.47947429 | -0.40583779 | 5.84786650 |
| 1 | 1.43249830 | 3.35519476 | 5.67326387 |
| 1 | 0.70683508 | 4.92367910 | 0.19122987 |
| 1 | 0.33619757 | 5.65859233 | 1.76654243 |
| 1 | -4.91900025 | 3.43970170 | 0.66815639 |
| 1 | -1.85639889 | 6.09828551 | 2.05652081 |
| 1 | 4.20953327 | -3.06074405 | 3.72404040 |
| 1 | 4.78079563 | -1.68517577 | 4.68521962 |
| 1 | 3.22138804 | -2.46181581 | 5.06995760 |
| 1 | 3.75052247 | -0.21354914 | 1.38944948 |
| 1 | 5.07581860 | -0.38613769 | 2.56386650 |
| 1 | 4.52691380 | -1.80199158 | 1.63602251 |
| 1 | 1.28413486 | -2.30951217 | 3.45929225 |
| 1 | 1.43095436 | -1.42675852 | 1.92094281 |
| 1 | 2.34977808 | -2.93800900 | 2.18216840 |
| 1 | -5.14329251 | 0.37861943 | -0.97711346 |
| 1 | -5.34727272 | 2.13896022 | -1.04968803 |
| 1 | -5.34466359 | 1.29937290 | 0.52462384 |

| | | | |
|---|---|---|---|
| 1 | -1.72720563 | 0.22812411 | 0.42465109 |
| 1 | -3.21374028 | 0.23606639 | 1.40852518 |
| 1 | -3.15735041 | -0.69787073 | -0.10290315 |
| 1 | -3.23304592 | 2.25386307 | -2.44378765 |
| 1 | -1.73202928 | 1.46751545 | -1.88961886 |
| 1 | -3.14820798 | 0.48046483 | -2.34982423 |
| 1 | 2.91275005 | 4.56262137 | 0.99002590 |
| 6 | 3.20129016 | -0.32999998 | -1.54801682 |
| 6 | 3.53479415 | 0.05944255 | -3.01247398 |
| 6 | 3.46886032 | 1.56564622 | -3.30853341 |
| 6 | 1.73749250 | -0.58775274 | -1.29598025 |
| 6 | 2.09130510 | 2.23746002 | -3.10744934 |
| 6 | 2.04633067 | 3.21840661 | -1.92764569 |
| 1 | 3.53229364 | 0.46184605 | -0.86690227 |
| 1 | 4.55790604 | -0.28829224 | -3.22099315 |
| 1 | 3.80321450 | 1.70853040 | -4.34788120 |
| 1 | 1.81584430 | 2.81076287 | -4.00832119 |
| 1 | 3.72534272 | -1.26418815 | -1.29843455 |
| 1 | 2.87836792 | -0.49553713 | -3.70033918 |
| 1 | 4.21035367 | 2.07632430 | -2.67330117 |
| 1 | 1.29912515 | 1.48238681 | -2.98418447 |
| 1 | 1.02796718 | 3.64899852 | -1.87471708 |
| 1 | 2.73938181 | 4.05389575 | -2.14633636 |
| 8 | 0.95937753 | 0.09965606 | -0.62841887 |
| 8 | 2.45216687 | 2.65311026 | -0.68281862 |
| 31 | 1.18705579 | 2.03417313 | 0.51035433 |
| 8 | -5.19562765 | -7.14336703 | -3.55760851 |
| 6 | -5.59559448 | -8.34547534 | -4.25927074 |
| 1 | -5.47372132 | -8.21619956 | -5.34202179 |
| 6 | -3.57905265 | -5.50715456 | -2.98718100 |
| 6 | -2.11914948 | -5.07462945 | -3.11669700 |
| 6 | -1.82500560 | -3.77746541 | -2.35623083 |
| 6 | -3.89519530 | -6.77824101 | -3.75252890 |
| 6 | -0.35897961 | -3.34333168 | -2.47906010 |
| 6 | -0.09673847 | -2.05242675 | -1.72461289 |
| 1 | -4.25937642 | -4.72021047 | -3.35249900 |
| 1 | -1.87079863 | -4.94950380 | -4.18157143 |
| 1 | -2.47936947 | -2.97306171 | -2.73429581 |
| 1 | -0.09809271 | -3.20443603 | -3.54016585 |
| 1 | -3.85670583 | -5.66830501 | -1.93307374 |
| 1 | -1.46679540 | -5.88079174 | -2.74738311 |
| 1 | -2.08157146 | -3.90761560 | -1.29085606 |
| 1 | 0.29945078 | -4.13456220 | -2.08730201 |
| 1 | -0.29973925 | -2.15162905 | -0.64891705 |
| 1 | -0.70006519 | -1.21771949 | -2.10845110 |
| 8 | -3.11964126 | -7.40225907 | -4.45155657 |
| 8 | 1.31811071 | -1.71539847 | -1.89634113 |
| 1 | -6.64876153 | -8.49337231 | -4.00215183 |
| 1 | -4.99109115 | -9.20023491 | -3.93091961 |

Electronic energy = -3908.677057 a.u.
DFT-D3(BJ) dispersion correction = -0.137158 a.u.
Thermal free energy = 0.736427 a.u.
Gibbs free energy = -3908.077787 a.u.

Number of imaginary frequencies = 0.

**Int4_CL isomer/conformer 3**

| Atomic N. | X | Y | Z |
|---|---|---|---|
| 7 | 2.45794320 | 2.93290957 | 0.85455967 |
| 8 | 2.17059961 | 0.05106216 | 1.15953228 |
| 8 | -0.02162337 | 1.94046862 | -0.40549156 |
| 6 | 3.91778074 | 3.10223899 | 0.67202121 |
| 6 | 2.75006191 | 0.27502313 | 2.35882731 |
| 6 | 3.31581826 | -0.83321293 | 3.06628751 |
| 6 | 3.91388439 | -0.57904817 | 4.30748993 |
| 6 | 3.97805285 | 0.69860597 | 4.86891652 |
| 6 | 3.41644161 | 1.76615246 | 4.17884341 |
| 6 | 2.79226524 | 1.56865628 | 2.94160760 |
| 6 | 2.10400739 | 2.74066186 | 2.29371985 |
| 1 | 4.46025348 | 0.85146377 | 5.83441242 |
| 6 | 3.27769179 | -2.26303568 | 2.49025046 |
| 6 | -0.55362440 | 3.05738483 | 0.12971227 |
| 6 | -1.97058990 | 3.13847481 | 0.30161467 |
| 6 | -2.49088687 | 4.32252261 | 0.84054919 |
| 6 | -1.68587832 | 5.40085771 | 1.21955120 |
| 6 | -0.30873229 | 5.30684842 | 1.05668916 |
| 6 | 0.26400606 | 4.15390630 | 0.50632006 |
| 6 | 1.75247314 | 4.12421730 | 0.27721887 |
| 1 | -2.13549516 | 6.29943273 | 1.64213188 |
| 6 | -2.89641686 | 1.96721763 | -0.08084613 |
| 6 | 3.89876348 | -3.29531454 | 3.45055592 |
| 6 | 4.09032396 | -2.31432717 | 1.17601025 |
| 6 | 1.81587393 | -2.70309752 | 2.23574589 |
| 6 | -4.37992964 | 2.28939851 | 0.18286719 |
| 6 | -2.54425270 | 0.71628418 | 0.75970561 |
| 6 | -2.76182602 | 1.64398407 | -1.58839713 |
| 1 | 4.26461442 | 4.00998039 | 1.19480483 |
| 1 | 4.44416227 | 2.23292364 | 1.08073016 |
| 1 | 2.34509351 | 3.66320595 | 2.85127633 |
| 1 | 1.01014132 | 2.62104168 | 2.32987620 |
| 1 | 4.35244469 | -1.40680472 | 4.86172419 |
| 1 | 3.44367133 | 2.77306038 | 4.60182527 |
| 1 | 1.99041915 | 4.11640070 | -0.79919443 |
| 1 | 2.20106475 | 5.03776787 | 0.70608451 |
| 1 | -3.56695008 | 4.40977534 | 0.97968662 |
| 1 | 0.34073101 | 6.13582535 | 1.34772060 |
| 1 | 3.83577825 | -4.29253053 | 2.99132486 |
| 1 | 4.96047485 | -3.09125080 | 3.65055667 |
| 1 | 3.36575186 | -3.33860062 | 4.41130195 |
| 1 | 3.70765568 | -1.59343799 | 0.44670979 |
| 1 | 5.14833921 | -2.08349414 | 1.36845169 |
| 1 | 4.03831938 | -3.32344192 | 0.73786875 |
| 1 | 1.25125465 | -2.72162265 | 3.17948357 |
| 1 | 1.30662665 | -2.02484958 | 1.54295126 |
| 1 | 1.80192245 | -3.72107594 | 1.81556356 |
| 1 | -4.98683893 | 1.42067123 | -0.10804002 |

```
   1    -4.72595472   3.15259875  -0.40452409
   1    -4.57620759   2.49145059   1.24607085
   1    -1.51497073   0.38774676   0.57973320
   1    -2.66089265   0.92765778   1.83299464
   1    -3.23026400  -0.10390494   0.49940258
   1    -3.05348072   2.51279586  -2.19708894
   1    -1.73365078   1.36709176  -1.84574110
   1    -3.43217344   0.80984512  -1.84581586
   1     4.13247669   3.17566187  -0.39966907
   6     2.52650898  -1.72284873  -2.28338562
   6     2.64291013  -1.55476023  -3.82129149
   6     2.91308911  -0.11746720  -4.29216086
   6     1.13086579  -1.53057848  -1.74449122
   6     1.83082798   0.92661832  -3.93456627
   6     2.28952870   1.96359544  -2.89988951
   1     3.19038150  -1.01177476  -1.77947567
   1     3.47436584  -2.19114612  -4.16051758
   1     3.05001055  -0.15010928  -5.38428820
   1     1.53306704   1.48682431  -4.83657566
   1     2.82138394  -2.74723913  -2.01255437
   1     1.73737619  -1.95256675  -4.30491492
   1     3.87678019   0.20989027  -3.87004890
   1     0.91289705   0.43521175  -3.57597808
   1     1.44922478   2.65852384  -2.71014244
   1     3.10772120   2.56030579  -3.34814069
   8     0.72018871  -0.60491222  -1.03920949
   8     2.79718197   1.39713408  -1.69340096
  31     1.71080912   1.25158458  -0.20753853
   8    -7.27430408  -2.91075952  -0.57000133
   6    -8.11677700  -1.76633832  -0.85379306
   1    -8.00973228  -1.00767976  -0.06847307
   6    -5.15328878  -3.87641472  -0.12905235
   6    -3.64922888  -3.74064982  -0.38071709
   6    -3.28738312  -3.70312660  -1.87156116
   6    -5.94317518  -2.63100101  -0.49000681
   6    -1.77761177  -3.70505644  -2.15783063
   6    -1.07799848  -2.44665526  -1.66870297
   1    -5.34793229  -4.06898110   0.93918107
   1    -3.13917906  -4.59332574   0.09635243
   1    -3.72934124  -4.58287558  -2.36779916
   1    -1.61507903  -3.79324825  -3.24310971
   1    -5.58073154  -4.73384459  -0.67080600
   1    -3.29108694  -2.83125537   0.12313782
   1    -3.74704762  -2.81677054  -2.33702404
   1    -1.30646640  -4.58544573  -1.69159306
   1    -1.07926278  -2.35142060  -0.57534575
   1    -1.52564336  -1.53526090  -2.09072493
   8    -5.47999811  -1.51811823  -0.66300455
   8     0.31408188  -2.53100096  -2.11859169
   1    -9.13775615  -2.15870153  -0.87834953
   1    -7.84672053  -1.32257798  -1.82008808
Electronic energy = -3908.676606 a.u.
DFT-D3(BJ) dispersion correction = -0.139596 a.u.
```

Thermal free energy = 0.738604 a.u.
Gibbs free energy = -3908.077598 a.u.
Number of imaginary frequencies = 0.

**Int4_CL isomer/conformer 4**

| Atomic N. | X | Y | Z |
|---|---|---|---|
| 7 | 2.58270770 | 2.64701669 | 1.31453015 |
| 8 | 2.13905278 | -0.22379396 | 1.42058449 |
| 8 | 0.15389513 | 1.86744083 | -0.17869458 |
| 6 | 4.05922481 | 2.74210346 | 1.24759178 |
| 6 | 2.64044227 | -0.09972695 | 2.66852218 |
| 6 | 3.11081464 | -1.27283278 | 3.34011769 |
| 6 | 3.62445137 | -1.12216003 | 4.63487962 |
| 6 | 3.69570372 | 0.11424814 | 5.28150429 |
| 6 | 3.23071413 | 1.24640494 | 4.62292084 |
| 6 | 2.69324260 | 1.15277300 | 3.33390624 |
| 6 | 2.11100756 | 2.39310160 | 2.70993604 |
| 1 | 4.11002101 | 0.18575867 | 6.28721544 |
| 6 | 3.06293215 | -2.66055058 | 2.66958583 |
| 6 | -0.35496031 | 2.99117781 | 0.36477880 |
| 6 | -1.77403436 | 3.16178738 | 0.40468019 |
| 6 | -2.26959734 | 4.34194309 | 0.97443545 |
| 6 | -1.43983643 | 5.33731714 | 1.49867037 |
| 6 | -0.06184951 | 5.16355052 | 1.44907262 |
| 6 | 0.48898333 | 4.01030358 | 0.87722971 |
| 6 | 1.98762284 | 3.90803349 | 0.76349722 |
| 1 | -1.87147277 | 6.23570970 | 1.93987036 |
| 6 | -2.73109274 | 2.09218440 | -0.15784119 |
| 6 | 3.58013710 | -3.77522439 | 3.59866160 |
| 6 | 3.96190237 | -2.66058065 | 1.41160937 |
| 6 | 1.60756125 | -3.02538105 | 2.29008171 |
| 6 | -4.20851304 | 2.51054150 | -0.03152148 |
| 6 | -2.55934066 | 0.76695075 | 0.62210229 |
| 6 | -2.45168123 | 1.85986686 | -1.66326664 |
| 1 | 4.41394492 | 3.59738719 | 1.84771177 |
| 1 | 4.50673701 | 1.82234561 | 1.63933364 |
| 1 | 2.35244233 | 3.26926512 | 3.33763650 |
| 1 | 1.01304577 | 2.32855500 | 2.65821742 |
| 1 | 3.98891355 | -2.00080739 | 5.16387083 |
| 1 | 3.26764947 | 2.22347820 | 5.11018594 |
| 1 | 2.30999035 | 3.95209008 | -0.28983341 |
| 1 | 2.44732544 | 4.76722038 | 1.28332770 |
| 1 | -3.34648998 | 4.49394612 | 1.01850556 |
| 1 | 0.60712186 | 5.93042506 | 1.84666185 |
| 1 | 3.51579464 | -4.73787420 | 3.07098257 |
| 1 | 4.63150899 | -3.62487058 | 3.88296835 |
| 1 | 2.98022456 | -3.85969291 | 4.51624838 |
| 1 | 3.65546764 | -1.88168112 | 0.70625083 |
| 1 | 5.01143296 | -2.48309096 | 1.68849843 |
| 1 | 3.90602470 | -3.63779540 | 0.90678358 |
| 1 | 0.97974775 | -3.08461840 | 3.19124694 |
| 1 | 1.17091312 | -2.28219789 | 1.61444102 |

| | | | |
|---|---|---|---|
| 1 | 1.58673349 | -4.01158609 | 1.80018953 |
| 1 | -4.84010256 | 1.72270614 | -0.46568152 |
| 1 | -4.42106276 | 3.44094142 | -0.57786891 |
| 1 | -4.51252144 | 2.64603759 | 1.01687070 |
| 1 | -1.54324944 | 0.36996993 | 0.52203421 |
| 1 | -2.77436313 | 0.91588627 | 1.69052991 |
| 1 | -3.27087048 | 0.01925721 | 0.23810504 |
| 1 | -2.59534179 | 2.79170132 | -2.22948376 |
| 1 | -1.42817988 | 1.50573604 | -1.82855663 |
| 1 | -3.15998489 | 1.11613777 | -2.05905176 |
| 1 | 4.35661533 | 2.86383037 | 0.20027816 |
| 6 | 2.63453351 | -1.80909769 | -2.10373195 |
| 6 | 2.79726086 | -1.55310401 | -3.62641993 |
| 6 | 3.16643909 | -0.10944037 | -4.00088673 |
| 6 | 1.23017912 | -1.60719892 | -1.59422326 |
| 6 | 2.13909258 | 0.97782389 | -3.61179229 |
| 6 | 2.61960833 | 1.91528399 | -2.49556536 |
| 1 | 3.30211077 | -1.14561672 | -1.54252826 |
| 1 | 3.59604490 | -2.21779569 | -3.98880666 |
| 1 | 3.33539845 | -0.08857132 | -5.08886024 |
| 1 | 1.91403716 | 1.61314464 | -4.48461501 |
| 1 | 2.89473503 | -2.85496480 | -1.88635710 |
| 1 | 1.88165748 | -1.86261950 | -4.15384813 |
| 1 | 4.13537729 | 0.13223607 | -3.53489284 |
| 1 | 1.17849174 | 0.52570794 | -3.31948076 |
| 1 | 1.82391213 | 2.66066584 | -2.30503779 |
| 1 | 3.50174923 | 2.47197627 | -2.86716204 |
| 8 | 0.81428994 | -0.68832697 | -0.88309622 |
| 8 | 3.02064547 | 1.24583403 | -1.30141533 |
| 31 | 1.83147644 | 1.07543826 | 0.10093860 |
| 8 | -7.71783482 | -1.96702176 | -1.80533023 |
| 6 | -8.63473292 | -0.86771750 | -2.02916627 |
| 1 | -8.46600166 | -0.07121261 | -1.29366657 |
| 6 | -5.51956436 | -2.83423122 | -1.60290920 |
| 6 | -4.07441875 | -2.64758418 | -2.06685846 |
| 6 | -3.16820619 | -3.80168282 | -1.62215953 |
| 6 | -6.39893004 | -1.63527414 | -1.90388317 |
| 6 | -1.72053057 | -3.70035650 | -2.12829529 |
| 6 | -0.98699180 | -2.49308607 | -1.56609126 |
| 1 | -5.55406163 | -2.98682581 | -0.51004550 |
| 1 | -3.70472984 | -1.68871946 | -1.67770479 |
| 1 | -3.16507633 | -3.86134606 | -0.52001838 |
| 1 | -1.70990578 | -3.65402510 | -3.22903311 |
| 1 | -5.97924219 | -3.73117986 | -2.04419226 |
| 1 | -4.05173365 | -2.55981272 | -3.16529289 |
| 1 | -3.59088085 | -4.75534878 | -1.97776759 |
| 1 | -1.16991565 | -4.60882007 | -1.84002191 |
| 1 | -1.00178694 | -2.47987117 | -0.46643560 |
| 1 | -1.39480307 | -1.53880767 | -1.92373171 |
| 8 | -6.00663442 | -0.51606631 | -2.17731877 |
| 8 | 0.40667056 | -2.58811904 | -2.00572993 |
| 1 | -9.63427007 | -1.29749591 | -1.91383681 |
| 1 | -8.50096443 | -0.45743519 | -3.03779272 |

Electronic energy = -3908.676262 a.u.
DFT-D3(BJ) dispersion correction = -0.139199 a.u.
Thermal free energy = 0.739051 a.u.
Gibbs free energy = -3908.076409 a.u.
Number of imaginary frequencies = 0.

**Int4 CL isomer/conformer 5**

| Atomic N. | X | Y | Z |
|---|---|---|---|
| 7 | 2.29802520 | 3.15848166 | 0.63606586 |
| 8 | 2.07095251 | 0.27855698 | 1.01055981 |
| 8 | -0.10306577 | 2.06769786 | -0.70199031 |
| 6 | 3.75629361 | 3.37207836 | 0.48781059 |
| 6 | 2.62583304 | 0.54671532 | 2.21304313 |
| 6 | 3.20822550 | -0.52771596 | 2.95728581 |
| 6 | 3.77769409 | -0.22534533 | 4.20122789 |
| 6 | 3.79771078 | 1.06744225 | 4.73069370 |
| 6 | 3.21917835 | 2.10162490 | 4.00470652 |
| 6 | 2.62250547 | 1.85484898 | 2.76277907 |
| 6 | 1.91310118 | 2.98752276 | 2.07071223 |
| 1 | 4.25866739 | 1.25743697 | 5.70004346 |
| 6 | 3.21616211 | -1.97265907 | 2.41890682 |
| 6 | -0.69154145 | 3.17106927 | -0.20036995 |
| 6 | -2.11653262 | 3.21279738 | -0.09263803 |
| 6 | -2.69481527 | 4.37965804 | 0.42438209 |
| 6 | -1.93894937 | 5.48144820 | 0.83492500 |
| 6 | -0.55432891 | 5.43107689 | 0.72303636 |
| 6 | 0.07533721 | 4.29569016 | 0.19979202 |
| 6 | 1.57045134 | 4.31461225 | 0.01758410 |
| 1 | -2.43232098 | 6.36552919 | 1.23853461 |
| 6 | -2.99112367 | 2.02147430 | -0.53202155 |
| 6 | 3.85141800 | -2.96181796 | 3.41445339 |
| 6 | 4.04739563 | -2.03708039 | 1.11686369 |
| 6 | 1.77003079 | -2.45773325 | 2.15731905 |
| 6 | -4.49265063 | 2.30446203 | -0.33717432 |
| 6 | -2.64627859 | 0.76786026 | 0.30611691 |
| 6 | -2.77060227 | 1.72869701 | -2.03545249 |
| 1 | 4.05858570 | 4.30320701 | 0.99679223 |
| 1 | 4.29907462 | 2.53093706 | 0.93228597 |
| 1 | 2.10936944 | 3.93063402 | 2.61114437 |
| 1 | 0.82322564 | 2.83179266 | 2.08182546 |
| 1 | 4.22835170 | -1.02659907 | 4.78390672 |
| 1 | 3.21087603 | 3.11918592 | 4.40223246 |
| 1 | 1.84015991 | 4.29868665 | -1.05113565 |
| 1 | 1.97387922 | 5.24995337 | 0.44432422 |
| 1 | -3.77776772 | 4.43623167 | 0.51871169 |
| 1 | 0.05671743 | 6.28183459 | 1.03350385 |
| 1 | 3.82206435 | -3.97195157 | 2.98101215 |
| 1 | 4.90420012 | -2.72323343 | 3.62356144 |
| 1 | 3.30574418 | -2.99516733 | 4.36839782 |
| 1 | 3.65259065 | -1.34982806 | 0.36195173 |
| 1 | 5.09550252 | -1.76881808 | 1.31502594 |
| 1 | 4.03109116 | -3.05969011 | 0.70838709 |

| | | | |
|---|---|---|---|
| 1 | 1.19182629 | -2.46541250 | 3.09271454 |
| 1 | 1.25339318 | -1.81290458 | 1.43852343 |
| 1 | 1.78779290 | -3.48690016 | 1.76599051 |
| 1 | -5.06739740 | 1.42837208 | -0.67087762 |
| 1 | -4.83198935 | 3.16717487 | -0.92847734 |
| 1 | -4.74673455 | 2.48374594 | 0.71745428 |
| 1 | -1.60107710 | 0.46939058 | 0.17379647 |
| 1 | -2.82503235 | 0.95485104 | 1.37495092 |
| 1 | -3.29203953 | -0.07111994 | 0.00085772 |
| 1 | -3.05431340 | 2.59923299 | -2.64484464 |
| 1 | -1.72335842 | 1.48302528 | -2.24310736 |
| 1 | -3.40223525 | 0.88143657 | -2.34659310 |
| 1 | 3.99827760 | 3.42641272 | -0.57916372 |
| 6 | 2.57041792 | -1.57653672 | -2.41048678 |
| 6 | 2.70932302 | -1.43040830 | -3.94869144 |
| 6 | 2.96876823 | 0.00289432 | -4.43752835 |
| 6 | 1.16351235 | -1.40337112 | -1.89672080 |
| 6 | 1.86744734 | 1.03904825 | -4.11684728 |
| 6 | 2.29321795 | 2.09877019 | -3.09119963 |
| 1 | 3.21155936 | -0.84475758 | -1.90665354 |
| 1 | 3.55431737 | -2.06065785 | -4.26491316 |
| 1 | 3.12575688 | -0.04708265 | -5.52627824 |
| 1 | 1.58029211 | 1.58075597 | -5.03351203 |
| 1 | 2.88079501 | -2.59044296 | -2.11818710 |
| 1 | 1.81720714 | -1.84754774 | -4.44085381 |
| 1 | 3.92079882 | 0.34959553 | -4.00443466 |
| 1 | 0.94889233 | 0.54192799 | -3.76738608 |
| 1 | 1.44478466 | 2.79324590 | -2.93922945 |
| 1 | 3.12061613 | 2.69042598 | -3.52905933 |
| 8 | 0.72526147 | -0.47620460 | -1.20993609 |
| 8 | 2.76963749 | 1.55991206 | -1.85993737 |
| 31 | 1.63885479 | 1.42716892 | -0.40667991 |
| 8 | -6.74213160 | -3.89932799 | 1.37792659 |
| 6 | -7.17182934 | -4.19268028 | 2.72971640 |
| 1 | -6.99707949 | -5.24964743 | 2.96700967 |
| 6 | -5.07169764 | -3.81862652 | -0.30219715 |
| 6 | -3.56629613 | -3.77718966 | -0.56552359 |
| 6 | -3.24736024 | -3.56407190 | -2.05061619 |
| 6 | -5.41593512 | -4.12076953 | 1.14422081 |
| 6 | -1.74634339 | -3.56406907 | -2.38359896 |
| 6 | -1.02616524 | -2.34871664 | -1.82328704 |
| 1 | -5.55339266 | -4.59611882 | -0.91986137 |
| 1 | -3.11251950 | -4.71734717 | -0.21691823 |
| 1 | -3.72994499 | -4.36260676 | -2.63730437 |
| 1 | -1.61392547 | -3.57465122 | -3.47640433 |
| 1 | -5.55815661 | -2.87249874 | -0.58519123 |
| 1 | -3.12087413 | -2.97983557 | 0.04810308 |
| 1 | -3.69826696 | -2.61795787 | -2.39729352 |
| 1 | -1.27616310 | -4.48055747 | -1.99331607 |
| 1 | -1.03087911 | -2.31694291 | -0.72597192 |
| 1 | -1.45959597 | -1.40898099 | -2.19450938 |
| 8 | -4.64140922 | -4.51960331 | 1.99278367 |
| 8 | 0.36771092 | -2.42174217 | -2.26780242 |

| | | | |
|---|---|---|---|
| 1 | -8.24120523 | -3.96202342 | 2.74948198 |
| 1 | -6.62555045 | -3.56967937 | 3.44874159 |

Electronic energy = -3908.675643 a.u.
DFT-D3(BJ) dispersion correction = -0.138298 a.u.
Thermal free energy = 0.737677 a.u.
Gibbs free energy = -3908.076264 a.u.
Number of imaginary frequencies = 0.

**Int4 CL isomer/conformer 6**

| Atomic N. | X | Y | Z |
|---|---|---|---|
| 7 | 2.58418276 | 3.07300027 | 1.52940633 |
| 8 | 2.15450597 | 0.19260879 | 1.53872602 |
| 8 | 0.15373102 | 2.32574939 | 0.02439228 |
| 6 | 4.05781765 | 3.19512620 | 1.44205589 |
| 6 | 2.68406451 | 0.27283146 | 2.77906632 |
| 6 | 3.16886974 | -0.92237641 | 3.39900085 |
| 6 | 3.71310176 | -0.81517141 | 4.68563557 |
| 6 | 3.79959359 | 0.39851638 | 5.37215732 |
| 6 | 3.31688645 | 1.55162166 | 4.76504808 |
| 6 | 2.74889902 | 1.50121072 | 3.48670479 |
| 6 | 2.14243013 | 2.75804749 | 2.92249848 |
| 1 | 4.23799281 | 0.43602464 | 6.36936158 |
| 6 | 3.10288934 | -2.28709346 | 2.68430244 |
| 6 | -0.37004337 | 3.40328269 | 0.64055886 |
| 6 | -1.79118971 | 3.53378668 | 0.72535652 |
| 6 | -2.30167906 | 4.67330026 | 1.36103717 |
| 6 | -1.48315688 | 5.66411729 | 1.91083364 |
| 6 | -0.10272059 | 5.52621584 | 1.82368948 |
| 6 | 0.46150715 | 4.41546534 | 1.18542215 |
| 6 | 1.95928040 | 4.34569652 | 1.04056022 |
| 1 | -1.92532912 | 6.53016953 | 2.40312912 |
| 6 | -2.73296747 | 2.46193453 | 0.14086124 |
| 6 | 3.63743675 | -3.43255240 | 3.56482597 |
| 6 | 3.97378966 | -2.24698375 | 1.40751464 |
| 6 | 1.63872956 | -2.63665259 | 2.32465635 |
| 6 | -4.21633473 | 2.82738645 | 0.33781293 |
| 6 | -2.49541129 | 1.10511443 | 0.84544223 |
| 6 | -2.49450719 | 2.31681172 | -1.38110506 |
| 1 | 4.40884160 | 4.03153864 | 2.07027472 |
| 1 | 4.52596664 | 2.26790735 | 1.78965140 |
| 1 | 2.38606658 | 3.61184246 | 3.57952888 |
| 1 | 1.04456304 | 2.68199394 | 2.88889583 |
| 1 | 4.08994528 | -1.71095991 | 5.17573574 |
| 1 | 3.36293154 | 2.51107104 | 5.28546251 |
| 1 | 2.26093381 | 4.43836103 | -0.01568006 |
| 1 | 2.41407001 | 5.19058734 | 1.58737671 |
| 1 | -3.38035024 | 4.79651424 | 1.43953390 |
| 1 | 0.55666287 | 6.28909574 | 2.24416180 |
| 1 | 3.55862505 | -4.37729723 | 3.00768319 |
| 1 | 4.69530631 | -3.29399948 | 3.83045283 |
| 1 | 3.05771780 | -3.54560504 | 4.49224768 |
| 1 | 3.65156378 | -1.44607048 | 0.73466619 |

| | | | |
|---|---|---|---|
| 1 | 5.02929179 | -2.07800673 | 1.66638587 |
| 1 | 3.90601821 | -3.20756196 | 0.87315606 |
| 1 | 1.02927548 | -2.72046423 | 3.23640176 |
| 1 | 1.19030151 | -1.87379702 | 1.67932704 |
| 1 | 1.60467881 | -3.60821962 | 1.80720412 |
| 1 | -4.84053712 | 2.03505488 | -0.09999214 |
| 1 | -4.48038786 | 3.77058503 | -0.16194826 |
| 1 | -4.48520081 | 2.90967790 | 1.40080733 |
| 1 | -1.46882514 | 0.75175530 | 0.70231355 |
| 1 | -2.69024225 | 1.19042194 | 1.92445838 |
| 1 | -3.18629970 | 0.34929000 | 0.43906600 |
| 1 | -2.70243427 | 3.26545708 | -1.89737352 |
| 1 | -1.46164392 | 2.02135604 | -1.59538760 |
| 1 | -3.17468542 | 1.55456297 | -1.79321737 |
| 1 | 4.33632249 | 3.36270320 | 0.39599380 |
| 6 | 2.62236517 | -1.26785053 | -2.06046197 |
| 6 | 2.81556518 | -0.95095974 | -3.56703572 |
| 6 | 3.18673117 | 0.50780112 | -3.87506565 |
| 6 | 1.21581440 | -1.05637179 | -1.56079249 |
| 6 | 2.15006373 | 1.57510596 | -3.45604271 |
| 6 | 2.61715819 | 2.47386332 | -2.30266285 |
| 1 | 3.29671263 | -0.64768721 | -1.45953409 |
| 1 | 3.62427388 | -1.59821747 | -3.93879465 |
| 1 | 3.37170678 | 0.57534998 | -4.95842554 |
| 1 | 1.92793121 | 2.24009336 | -4.30727155 |
| 1 | 2.85441261 | -2.32862372 | -1.88573397 |
| 1 | 1.91179162 | -1.24233828 | -4.12411695 |
| 1 | 4.14722759 | 0.73202149 | -3.38384287 |
| 1 | 1.18962483 | 1.10750948 | -3.18807443 |
| 1 | 1.81398842 | 3.20425697 | -2.08652186 |
| 1 | 3.49516607 | 3.05263920 | -2.64957811 |
| 8 | 0.81950960 | -0.18272646 | -0.78404488 |
| 8 | 3.02031384 | 1.76210215 | -1.13450494 |
| 31 | 1.83994974 | 1.53433562 | 0.26629107 |
| 8 | -7.71656756 | -4.99062389 | -2.51100296 |
| 6 | -8.58402349 | -5.98999449 | -3.09932374 |
| 1 | -8.59974047 | -5.88911272 | -4.19179726 |
| 6 | -5.59227815 | -3.97373505 | -2.24790439 |
| 6 | -4.11174127 | -4.02481891 | -2.62272809 |
| 6 | -3.30088384 | -2.91149247 | -1.95153151 |
| 6 | -6.41430185 | -5.06157296 | -2.91292571 |
| 6 | -1.81347230 | -2.96185929 | -2.32296045 |
| 6 | -1.03436224 | -1.84654986 | -1.64923732 |
| 1 | -6.03790447 | -3.00338973 | -2.52224610 |
| 1 | -4.01130302 | -3.95433091 | -3.71650275 |
| 1 | -3.71761149 | -1.92989174 | -2.23610782 |
| 1 | -1.69815080 | -2.87826883 | -3.41519683 |
| 1 | -5.73194299 | -4.06273727 | -1.15842693 |
| 1 | -3.69990710 | -5.00708264 | -2.34467159 |
| 1 | -3.40878583 | -2.98558325 | -0.85577748 |
| 1 | -1.38705887 | -3.93363307 | -2.02826448 |
| 1 | -1.07835173 | -1.91298536 | -0.55299498 |
| 1 | -1.39587718 | -0.85126948 | -1.94397820 |

| | | | |
|---|---|---|---|
| 8 | -5.99789915 | -5.89145391 | -3.69867600 |
| 8 | 0.36546517 | -1.96938820 | -2.06188547 |
| 1 | -9.57497788 | -5.79705276 | -2.67750476 |
| 1 | -8.23947489 | -6.99845931 | -2.83827879 |

Electronic energy = -3908.677047 a.u.
DFT-D3(BJ) dispersion correction = -0.137150 a.u.
Thermal free energy = 0.740177 a.u.
Gibbs free energy = -3908.074019 a.u.
Number of imaginary frequencies = 0.

## Propagation step of equatorial CL addition to the product of CL addition on initiation step

**Int2 CL C(O)ax CL isomer/conformer 1**

| Atomic N. | X | Y | Z |
|---|---|---|---|
| 7 | -2.13349690 | 0.88787956 | 2.08657956 |
| 8 | -2.43471959 | 0.65312770 | -0.79433553 |
| 8 | 0.30445719 | 1.62766648 | 0.60461095 |
| 6 | -2.94567271 | -0.17471486 | 2.73365066 |
| 6 | -3.73143395 | 0.89900588 | -0.53654410 |
| 6 | -4.74238986 | 0.53102335 | -1.47916626 |
| 6 | -6.07260330 | 0.82288243 | -1.14692693 |
| 6 | -6.43986124 | 1.44789221 | 0.04898770 |
| 6 | -5.44852099 | 1.80580880 | 0.95665419 |
| 6 | -4.10565532 | 1.54502863 | 0.66772501 |
| 6 | -3.02897350 | 2.00005515 | 1.60499736 |
| 6 | -4.38862754 | -0.15753323 | -2.81004676 |
| 6 | 0.31545638 | 2.68980970 | 1.43216097 |
| 6 | 1.07465707 | 3.84919728 | 1.07955069 |
| 6 | 1.07486455 | 4.92322613 | 1.97955899 |
| 6 | 0.36170852 | 4.90410757 | 3.18167023 |
| 6 | -0.38125669 | 3.77567655 | 3.50925704 |
| 6 | -0.40320689 | 2.66629473 | 2.65568935 |
| 6 | -1.15709577 | 1.43599357 | 3.09293712 |
| 6 | 1.85904642 | 3.92494090 | -0.24598452 |
| 6 | -5.63614274 | -0.45153330 | -3.66432483 |
| 6 | -3.69123052 | -1.51012992 | -2.53379703 |
| 6 | -3.46791099 | 0.76210982 | -3.64631991 |
| 6 | 2.60862572 | 5.26222476 | -0.39745822 |
| 6 | 0.88702344 | 3.80433263 | -1.44367497 |
| 6 | 2.91801879 | 2.79868259 | -0.31023020 |
| 1 | -3.54423528 | 0.26080889 | 3.55095139 |
| 1 | -3.62451369 | -0.61926802 | 1.99824253 |
| 1 | -3.47475152 | 2.48499275 | 2.49003345 |
| 1 | -2.37288812 | 2.73751480 | 1.12049699 |
| 1 | -6.86156104 | 0.55008125 | -1.84589279 |
| 1 | -5.70669626 | 2.30366522 | 1.89447115 |
| 1 | -0.47260953 | 0.60858401 | 3.32782872 |
| 1 | -1.72124846 | 1.67199815 | 4.01172243 |
| 1 | 1.64531784 | 5.81754002 | 1.73525574 |
| 1 | -0.95037424 | 3.74054907 | 4.44133981 |

| | | | |
|---|---|---|---|
| 1 | -5.32173791 | -0.93701902 | -4.59956818 |
| 1 | -6.33481778 | -1.13255811 | -3.15687529 |
| 1 | -6.17879167 | 0.46606735 | -3.93419912 |
| 1 | -2.78026719 | -1.38186736 | -1.93938505 |
| 1 | -4.36823243 | -2.18692628 | -1.99142418 |
| 1 | -3.42289411 | -1.99411122 | -3.48577078 |
| 1 | -3.98907257 | 1.69623694 | -3.90258851 |
| 1 | -2.55319673 | 1.01673681 | -3.09999671 |
| 1 | -3.18876637 | 0.26076628 | -4.58593024 |
| 1 | 3.14995612 | 5.26147326 | -1.35440685 |
| 1 | 3.34858894 | 5.41533297 | 0.40165449 |
| 1 | 1.92366824 | 6.12225687 | -0.40671995 |
| 1 | 0.34012042 | 2.85578908 | -1.42602206 |
| 1 | 0.15690043 | 4.62659848 | -1.43220505 |
| 1 | 1.44881021 | 3.86414129 | -2.38844620 |
| 1 | 3.63274896 | 2.89223303 | 0.52107271 |
| 1 | 2.44941924 | 1.80989770 | -0.26318200 |
| 1 | 3.48356799 | 2.87240000 | -1.25179615 |
| 1 | -2.27948711 | -0.94663128 | 3.12849884 |
| 8 | -0.32559151 | -1.27511422 | 1.92281097 |
| 8 | 0.78030587 | -3.06386897 | 2.50061829 |
| 6 | 0.77987534 | -1.83423753 | 1.96533452 |
| 6 | 2.07109855 | -1.18921633 | 1.54217523 |
| 1 | 2.59088848 | -1.84074554 | 0.82208203 |
| 1 | 1.79694011 | -0.26700291 | 1.01983251 |
| 6 | 3.00395553 | -0.87946052 | 2.73786684 |
| 1 | 2.42003901 | -0.44428882 | 3.56543772 |
| 1 | 3.69975206 | -0.09326938 | 2.41081278 |
| 6 | 3.81405037 | -2.08342607 | 3.23000201 |
| 1 | 4.47024701 | -2.42212931 | 2.41177085 |
| 1 | 4.47338027 | -1.76509493 | 4.05241906 |
| 6 | 2.96397174 | -3.27050273 | 3.69640121 |
| 1 | 2.37005619 | -3.00864588 | 4.58777966 |
| 1 | 3.63060580 | -4.09380488 | 4.00102562 |
| 6 | 2.02511411 | -3.82606549 | 2.63200457 |
| 1 | 2.51246259 | -3.89258226 | 1.64875599 |
| 1 | 1.66066067 | -4.82036207 | 2.91605317 |
| 31 | -0.99160688 | 0.26689678 | 0.39801334 |
| 8 | -0.10825338 | -0.95874612 | -0.68539313 |
| 1 | -7.48950583 | 1.65078116 | 0.26172681 |
| 1 | 0.38381980 | 5.76742278 | 3.84667710 |
| 8 | 4.69639342 | -5.18402342 | -1.69645608 |
| 6 | 4.96034868 | -5.99909593 | -0.53036866 |
| 1 | 5.67292617 | -5.49759016 | 0.13666343 |
| 6 | 4.01979448 | -3.15057321 | -2.71172985 |
| 6 | 2.88623246 | -2.11861878 | -2.67190800 |
| 6 | 1.48867093 | -2.74538020 | -2.56783410 |
| 6 | 4.19037562 | -3.94727397 | -1.43584531 |
| 6 | 0.34398076 | -1.78743732 | -2.92871196 |
| 6 | 0.18566616 | -0.56333460 | -2.02229755 |
| 1 | 4.98619611 | -2.64416949 | -2.88080012 |
| 1 | 2.95213395 | -1.52207592 | -3.59658946 |
| 1 | 1.43650077 | -3.61388499 | -3.24731310 |

| | | | |
|---|---|---|---|
| 1 | -0.60510591 | -2.34575986 | -2.91791332 |
| 1 | 3.89051377 | -3.85288347 | -3.54765119 |
| 1 | 3.05738704 | -1.42096200 | -1.83950472 |
| 1 | 1.32802338 | -3.13334017 | -1.55039382 |
| 1 | 0.48777858 | -1.42344489 | -3.96206563 |
| 1 | -0.61618414 | 0.06832961 | -2.44450246 |
| 1 | 1.11326551 | 0.04520802 | -2.04475454 |
| 8 | 3.95215225 | -3.55294830 | -0.30448847 |
| 1 | 5.38303363 | -6.93052571 | -0.91850046 |
| 1 | 4.02963448 | -6.19709004 | 0.01637850 |

Electronic energy = -3908.666463 a.u.
DFT-D3(BJ) dispersion correction = -0.140696 a.u.
Thermal free energy = 0.740924 a.u.
Gibbs free energy = -3908.066234 a.u.
Number of imaginary frequencies = 0.

**TS3 eq CL isomer/conformer 1**

| Atomic N. | X | Y | Z |
|---|---|---|---|
| 7 | -1.90726554 | 1.28909290 | 2.36311388 |
| 8 | -1.72103071 | 0.25447732 | -0.37006485 |
| 8 | 0.50837499 | 2.23548198 | 0.95148373 |
| 6 | -2.50702739 | 0.22091119 | 3.19935274 |
| 6 | -3.06875467 | 0.23172274 | -0.26809230 |
| 6 | -3.82983208 | -0.61141294 | -1.13677204 |
| 6 | -5.22531891 | -0.58897847 | -1.00527716 |
| 6 | -5.88736773 | 0.21021140 | -0.06963630 |
| 6 | -5.13795757 | 1.02382886 | 0.77233070 |
| 6 | -3.74270058 | 1.04829395 | 0.67569172 |
| 6 | -2.96152878 | 1.98396957 | 1.55388010 |
| 6 | -3.15268683 | -1.51458240 | -2.18534946 |
| 6 | 0.13953371 | 3.43506455 | 1.45117438 |
| 6 | 0.62941897 | 4.64167547 | 0.85924125 |
| 6 | 0.22036459 | 5.85625315 | 1.42700827 |
| 6 | -0.63357335 | 5.92975092 | 2.53043246 |
| 6 | -1.10036039 | 4.75148439 | 3.10106254 |
| 6 | -0.71531683 | 3.51099777 | 2.58050919 |
| 6 | -1.19126129 | 2.25725937 | 3.26183414 |
| 6 | 1.58655727 | 4.62222099 | -0.34811103 |
| 6 | -4.17548847 | -2.33148527 | -2.99777555 |
| 6 | -2.21142340 | -2.52505541 | -1.48808646 |
| 6 | -2.36028123 | -0.64636034 | -3.19019699 |
| 6 | 1.95910609 | 6.03984070 | -0.82234126 |
| 6 | 0.92496437 | 3.90290403 | -1.54657996 |
| 6 | 2.90307426 | 3.91539836 | 0.05164599 |
| 1 | -3.28199172 | 0.65171474 | 3.85578680 |
| 1 | -2.96415496 | -0.54149610 | 2.56062841 |
| 1 | -3.64537287 | 2.51314926 | 2.24004769 |
| 1 | -2.44387436 | 2.74765992 | 0.95478755 |
| 1 | -5.82602119 | -1.22335231 | -1.65450752 |
| 1 | -5.63037968 | 1.66027367 | 1.51130283 |
| 1 | -0.35000691 | 1.69205558 | 3.69454455 |
| 1 | -1.87134874 | 2.53089952 | 4.08711004 |

| | | | |
|---|---|---|---|
| 1 | 0.57641679 | 6.78775597 | 0.99082214 |
| 1 | -1.76751316 | 4.78122091 | 3.96564078 |
| 1 | -3.63545656 | -2.95200562 | -3.72749639 |
| 1 | -4.76607037 | -3.00668287 | -2.36159611 |
| 1 | -4.86804056 | -1.68817091 | -3.55957437 |
| 1 | -1.42570126 | -2.01947021 | -0.91685796 |
| 1 | -2.78099608 | -3.17044544 | -0.80287051 |
| 1 | -1.73440683 | -3.16917491 | -2.24342704 |
| 1 | -3.03767800 | 0.03166993 | -3.72970200 |
| 1 | -1.59991753 | -0.04433567 | -2.68179107 |
| 1 | -1.86389172 | -1.29029036 | -3.93244481 |
| 1 | 2.64161539 | 5.96044922 | -1.68081379 |
| 1 | 2.47632456 | 6.61652899 | -0.04208396 |
| 1 | 1.07866347 | 6.61037207 | -1.15154254 |
| 1 | 0.64323819 | 2.87559271 | -1.29580867 |
| 1 | 0.01938214 | 4.43998384 | -1.86425889 |
| 1 | 1.61995745 | 3.87832713 | -2.39987564 |
| 1 | 3.42139769 | 4.48589993 | 0.83624506 |
| 1 | 2.71466064 | 2.90452695 | 0.42977539 |
| 1 | 3.57529020 | 3.84683347 | -0.81755495 |
| 1 | -1.72170663 | -0.24340478 | 3.80293918 |
| 8 | 0.12941273 | -0.68613189 | 2.35750771 |
| 8 | 1.06470168 | -2.57433701 | 1.71091747 |
| 6 | 1.21068680 | -1.25278401 | 1.91072309 |
| 6 | 2.56283593 | -0.70618588 | 2.33021092 |
| 1 | 3.30994725 | -0.85322934 | 1.53692746 |
| 1 | 2.42748976 | 0.37560636 | 2.45049191 |
| 6 | 3.07567191 | -1.35031760 | 3.63615632 |
| 1 | 2.24104381 | -1.47265625 | 4.34494543 |
| 1 | 3.78105879 | -0.64926952 | 4.10719156 |
| 6 | 3.79288554 | -2.68828773 | 3.40872288 |
| 1 | 4.65883350 | -2.50938106 | 2.74794388 |
| 1 | 4.20558357 | -3.04681969 | 4.36471462 |
| 6 | 2.92285323 | -3.79168034 | 2.79368877 |
| 1 | 2.14591551 | -4.11379433 | 3.50599885 |
| 1 | 3.54895997 | -4.67632866 | 2.59350395 |
| 6 | 2.23486447 | -3.41458941 | 1.48639870 |
| 1 | 2.91804338 | -2.92755604 | 0.77640390 |
| 1 | 1.82724965 | -4.29934216 | 0.98610795 |
| 31 | -0.44691300 | 0.59984982 | 0.97360158 |
| 8 | 1.00552464 | -0.47276056 | 0.15319723 |
| 1 | -6.97516775 | 0.19084635 | -0.00268961 |
| 1 | -0.92887163 | 6.89872551 | 2.93270350 |
| 8 | 0.88466859 | -5.24109793 | -3.20447612 |
| 6 | 0.23826483 | -6.19619846 | -2.32719326 |
| 1 | 0.95164472 | -6.96429110 | -2.00363183 |
| 6 | 2.57895756 | -3.62260294 | -3.63604236 |
| 6 | 2.77408290 | -2.22786927 | -3.01765776 |
| 6 | 1.44990122 | -1.50266147 | -2.74886346 |
| 6 | 1.95589709 | -4.60041714 | -2.65978289 |
| 6 | 1.61843264 | -0.08240281 | -2.19270515 |
| 6 | 2.03734064 | -0.01255304 | -0.72089034 |
| 1 | 3.55817008 | -4.04286432 | -3.91666484 |

| | | | |
|---|---|---|---|
| 1 | 3.38889003 | -1.62804496 | -3.70821762 |
| 1 | 0.87947404 | -1.45236182 | -3.69070888 |
| 1 | 0.67502415 | 0.47330362 | -2.30401683 |
| 1 | 1.96590900 | -3.56861115 | -4.54566240 |
| 1 | 3.35603213 | -2.33839631 | -2.08988762 |
| 1 | 0.83412498 | -2.08685446 | -2.04729033 |
| 1 | 2.37357163 | 0.46514231 | -2.78427720 |
| 1 | 2.32508826 | 1.02094853 | -0.46854877 |
| 1 | 2.92639232 | -0.64927948 | -0.56065100 |
| 8 | 2.35619068 | -4.80869770 | -1.52681541 |
| 1 | -0.56586802 | -6.63732958 | -2.92353272 |
| 1 | -0.16943482 | -5.68548679 | -1.44575226 |

Electronic energy = -3908.660227 a.u.
DFT-D3(BJ) dispersion correction = -0.142836 a.u.
Thermal free energy = 0.742940 a.u.
Gibbs free energy = -3908.060123 a.u.
Number of imaginary frequencies = 1.

**TS3 eq CL isomer/conformer 2**

| Atomic N. | X | Y | Z |
|---|---|---|---|
| 7 | -0.55524504 | 2.83434772 | 1.79135060 |
| 8 | -2.13406563 | 1.11400867 | 0.02731847 |
| 8 | 1.00238061 | 1.83113468 | -0.47401643 |
| 6 | -1.10114133 | 2.60485363 | 3.15061545 |
| 6 | -3.13768053 | 1.94506299 | 0.38671750 |
| 6 | -4.49560642 | 1.52139628 | 0.23279595 |
| 6 | -5.50460482 | 2.41821098 | 0.60954756 |
| 6 | -5.23676109 | 3.68841434 | 1.12586379 |
| 6 | -3.91434550 | 4.09032631 | 1.27271271 |
| 6 | -2.86761761 | 3.23971915 | 0.90057725 |
| 6 | -1.45368063 | 3.74031711 | 1.00356317 |
| 6 | -4.85038567 | 0.12963633 | -0.32488644 |
| 6 | 1.49636769 | 3.08245325 | -0.50652444 |
| 6 | 2.11998868 | 3.56267190 | -1.70048761 |
| 6 | 2.63812971 | 4.86434174 | -1.68301964 |
| 6 | 2.55808210 | 5.69712019 | -0.56282616 |
| 6 | 1.94137847 | 5.22310257 | 0.58949202 |
| 6 | 1.42136371 | 3.92443538 | 0.63207996 |
| 6 | 0.82447958 | 3.41999865 | 1.91839302 |
| 6 | 2.21393251 | 2.68669128 | -2.96513224 |
| 6 | -6.37203169 | -0.09218727 | -0.42023146 |
| 6 | -4.29135418 | -0.96736306 | 0.61218542 |
| 6 | -4.28243780 | -0.02523108 | -1.75568318 |
| 6 | 2.94488358 | 3.40540290 | -4.11476851 |
| 6 | 0.79330987 | 2.33982110 | -3.47032213 |
| 6 | 3.00040317 | 1.38961959 | -2.66024923 |
| 1 | -1.23411691 | 3.56998277 | 3.66802192 |
| 1 | -2.07030439 | 2.10007358 | 3.08361363 |
| 1 | -1.44649446 | 4.74038601 | 1.47043312 |
| 1 | -0.99815649 | 3.84397483 | 0.00760771 |
| 1 | -6.54481411 | 2.11644125 | 0.50153381 |
| 1 | -3.67733526 | 5.07996988 | 1.67013407 |

| | | | |
|---|---|---|---|
| 1 | 1.44291675 | 2.62266254 | 2.36147237 |
| 1 | 0.77975768 | 4.25056934 | 2.64410210 |
| 1 | 3.11896062 | 5.25337791 | -2.57885814 |
| 1 | 1.86379194 | 5.85674524 | 1.47621155 |
| 1 | -6.56061983 | -1.09550035 | -0.82902479 |
| 1 | -6.86217356 | -0.03885449 | 0.56263262 |
| 1 | -6.85478640 | 0.63369054 | -1.09032309 |
| 1 | -3.20938396 | -0.87298089 | 0.75067091 |
| 1 | -4.77067518 | -0.90390253 | 1.60017204 |
| 1 | -4.50586939 | -1.96393085 | 0.19599612 |
| 1 | -4.75544739 | 0.70018709 | -2.43371463 |
| 1 | -3.20008421 | 0.13967733 | -1.78185225 |
| 1 | -4.49673700 | -1.03491950 | -2.13885498 |
| 1 | 2.98654222 | 2.73527169 | -4.98550367 |
| 1 | 3.97932673 | 3.66634059 | -3.84788656 |
| 1 | 2.42475319 | 4.32203054 | -4.42841673 |
| 1 | 0.21741246 | 1.80087936 | -2.71022916 |
| 1 | 0.24480934 | 3.25475907 | -3.73791409 |
| 1 | 0.85919744 | 1.70976424 | -4.37078905 |
| 1 | 4.02769947 | 1.62707102 | -2.34626770 |
| 1 | 2.51655602 | 0.80903488 | -1.86770725 |
| 1 | 3.05996585 | 0.76822937 | -3.56735158 |
| 1 | -0.40652138 | 1.97299492 | 3.71193457 |
| 8 | 0.30630404 | 0.17696679 | 2.28593779 |
| 8 | -0.38378891 | -1.92370737 | 2.44409680 |
| 6 | 0.54601872 | -1.05643690 | 2.00198817 |
| 6 | 1.97723293 | -1.51427579 | 1.81715584 |
| 1 | 2.02970910 | -2.36463690 | 1.12441838 |
| 1 | 2.50072312 | -0.67357665 | 1.34794438 |
| 6 | 2.63575220 | -1.89734375 | 3.16159201 |
| 1 | 2.34964180 | -1.16654336 | 3.93525004 |
| 1 | 3.72607970 | -1.80924714 | 3.04225039 |
| 6 | 2.30621195 | -3.32673526 | 3.61165023 |
| 1 | 2.69089770 | -4.03108501 | 2.85544538 |
| 1 | 2.84456730 | -3.54598308 | 4.54698753 |
| 6 | 0.81266189 | -3.60933208 | 3.81868601 |
| 1 | 0.41155696 | -3.02608299 | 4.66371250 |
| 1 | 0.68459272 | -4.67159939 | 4.08262348 |
| 6 | -0.06008361 | -3.34114814 | 2.59694242 |
| 1 | 0.39965305 | -3.73019385 | 1.67714524 |
| 1 | -1.04667354 | -3.80557299 | 2.71288824 |
| 31 | -0.33969316 | 1.07785451 | 0.61278749 |
| 1 | -6.05507565 | 4.35031939 | 1.40926051 |
| 1 | 2.96905899 | 6.70598888 | -0.59874827 |
| 8 | 3.78220344 | -6.55644178 | -1.18689740 |
| 6 | 3.97822523 | -7.73174429 | -0.36188689 |
| 1 | 4.49651670 | -7.46443701 | 0.56747222 |
| 6 | 2.90577912 | -4.36538935 | -1.51498222 |
| 6 | 1.46870077 | -3.82565069 | -1.57320189 |
| 6 | 1.39320087 | -2.39578533 | -2.11681008 |
| 6 | 3.03037000 | -5.57679272 | -0.61402446 |
| 6 | -0.02650969 | -1.81522977 | -2.14486313 |
| 6 | -0.67431837 | -1.61573493 | -0.76848590 |

| | | | |
|---|---|---|---|
| 1 | 3.56385016 | -3.59246373 | -1.08126092 |
| 1 | 1.04901075 | -3.86694717 | -0.55793947 |
| 1 | 2.02937961 | -1.73897684 | -1.50158262 |
| 1 | -0.69077152 | -2.47045922 | -2.73636079 |
| 1 | 3.29752660 | -4.59720707 | -2.51377773 |
| 1 | 0.84989315 | -4.49784279 | -2.19057703 |
| 1 | 1.81423235 | -2.36655068 | -3.13530993 |
| 1 | -0.00623683 | -0.84238720 | -2.65984130 |
| 1 | -0.78657919 | -2.58551121 | -0.24853344 |
| 1 | -1.69191003 | -1.22007012 | -0.90521950 |
| 8 | 2.54150319 | -5.67416525 | 0.50000072 |
| 8 | 0.08311695 | -0.74956977 | 0.07028655 |
| 1 | 4.58854818 | -8.40968418 | -0.96586645 |
| 1 | 3.01301050 | -8.19091892 | -0.11526149 |

Electronic energy = -3908.660011 a.u.
DFT-D3(BJ) dispersion correction = -0.141260 a.u.
Thermal free energy = 0.741342 a.u.
Gibbs free energy = -3908.059928 a.u.
Number of imaginary frequencies = 1.

**TS3 eq CL isomer/conformer 3**

| Atomic N. | X | Y | Z |
|---|---|---|---|
| 7 | -0.79571372 | 2.95838833 | 1.47763407 |
| 8 | -2.23307443 | 0.91606349 | -0.04672300 |
| 8 | 0.82313210 | 1.84164071 | -0.68941331 |
| 6 | -1.30712998 | 2.83922672 | 2.86414647 |
| 6 | -3.30260658 | 1.69377542 | 0.23334724 |
| 6 | -4.61945868 | 1.13994515 | 0.15394591 |
| 6 | -5.69994402 | 1.98433053 | 0.44356143 |
| 6 | -5.54011202 | 3.32405996 | 0.80587876 |
| 6 | -4.25678253 | 3.85241532 | 0.88171166 |
| 6 | -3.14271808 | 3.05764461 | 0.59040034 |
| 6 | -1.77737892 | 3.68690252 | 0.60928613 |
| 6 | -4.85573721 | -0.33265388 | -0.23266561 |
| 6 | 1.20609641 | 3.11872458 | -0.87169534 |
| 6 | 1.77590489 | 3.51107788 | -2.12328410 |
| 6 | 2.17948103 | 4.84557867 | -2.26222968 |
| 6 | 2.03660035 | 5.79078960 | -1.24196982 |
| 6 | 1.47239649 | 5.40051460 | -0.03274548 |
| 6 | 1.06741107 | 4.07559490 | 0.16567585 |
| 6 | 0.52786422 | 3.67224169 | 1.51165462 |
| 6 | 1.93473375 | 2.50781155 | -3.28239346 |
| 6 | -6.35293198 | -0.69366956 | -0.27491942 |
| 6 | -4.19642687 | -1.26025522 | 0.81561798 |
| 6 | -4.28701735 | -0.60498512 | -1.64551079 |
| 6 | 2.59117770 | 3.15005255 | -4.51895618 |
| 6 | 0.54558367 | 1.98534095 | -3.71927762 |
| 6 | 2.83320451 | 1.32560635 | -2.84750485 |
| 1 | -1.51969254 | 3.84192944 | 3.27190161 |
| 1 | -2.22883344 | 2.24847841 | 2.87278080 |
| 1 | -1.85404682 | 4.73081827 | 0.95929933 |
| 1 | -1.34151745 | 3.71449661 | -0.40040624 |

| | | | |
|---|---|---|---|
| 1 | -6.71060419 | 1.58380187 | 0.38973975 |
| 1 | -4.10400391 | 4.89809656 | 1.15869963 |
| 1 | 1.21797776 | 2.98774886 | 2.03100157 |
| 1 | 0.41606203 | 4.57361221 | 2.13898754 |
| 1 | 2.61759186 | 5.16941261 | -3.20471168 |
| 1 | 1.34719312 | 6.12229586 | 0.77790660 |
| 1 | -6.45641613 | -1.75035763 | -0.56128734 |
| 1 | -6.83842850 | -0.56797063 | 0.70365065 |
| 1 | -6.90210676 | -0.09516893 | -1.01598203 |
| 1 | -3.12590146 | -1.05750704 | 0.92364866 |
| 1 | -4.67250013 | -1.12298536 | 1.79765427 |
| 1 | -4.32576418 | -2.31345487 | 0.52168471 |
| 1 | -4.82602978 | -0.00688717 | -2.39480042 |
| 1 | -3.22323012 | -0.35278088 | -1.70946932 |
| 1 | -4.41594410 | -1.66713798 | -1.90515351 |
| 1 | 2.68354082 | 2.39083028 | -5.30890942 |
| 1 | 3.60127163 | 3.52691221 | -4.30203438 |
| 1 | 1.99101055 | 3.97724891 | -4.92477608 |
| 1 | 0.02506970 | 1.48946905 | -2.89283919 |
| 1 | -0.08260302 | 2.81345010 | -4.07874060 |
| 1 | 0.65867341 | 1.26449096 | -4.54373503 |
| 1 | 3.83843613 | 1.68385231 | -2.58009958 |
| 1 | 2.40752745 | 0.79950708 | -1.98649812 |
| 1 | 2.93940806 | 0.61271232 | -3.67997575 |
| 1 | -0.55468941 | 2.33769274 | 3.47989392 |
| 8 | 0.30735362 | 0.46212906 | 2.25203037 |
| 8 | -0.16024849 | -1.66505551 | 2.66451120 |
| 6 | 0.66708088 | -0.76554543 | 2.10053968 |
| 6 | 2.13408875 | -1.09630573 | 1.92454314 |
| 1 | 2.26023054 | -2.01067162 | 1.32971096 |
| 1 | 2.55961823 | -0.26689085 | 1.34841597 |
| 6 | 2.85371423 | -1.25552475 | 3.28268385 |
| 1 | 2.50869012 | -0.47339025 | 3.97817183 |
| 1 | 3.92679787 | -1.07315409 | 3.12126160 |
| 6 | 2.68114734 | -2.64977860 | 3.89957571 |
| 1 | 3.12170315 | -3.39416099 | 3.21562028 |
| 1 | 3.25683379 | -2.70592618 | 4.83668709 |
| 6 | 1.22866964 | -3.05447698 | 4.18231678 |
| 1 | 0.78605413 | -2.42138553 | 4.96871424 |
| 1 | 1.21527767 | -4.08705807 | 4.56708384 |
| 6 | 0.30999628 | -3.01625681 | 2.96546507 |
| 1 | 0.78960264 | -3.46047282 | 2.08151531 |
| 1 | -0.62137115 | -3.56037760 | 3.16286612 |
| 31 | -0.43665507 | 1.10394561 | 0.50181800 |
| 1 | -6.41087151 | 3.94151855 | 1.02630937 |
| 1 | 2.35826826 | 6.82030916 | -1.39889741 |
| 8 | 4.32393456 | -6.29939365 | -0.56367850 |
| 6 | 4.63806343 | -7.35879755 | 0.37407935 |
| 1 | 5.15647316 | -6.95178270 | 1.25120556 |
| 6 | 3.25830007 | -4.23461008 | -1.09162509 |
| 6 | 1.77567530 | -3.83937502 | -1.16867840 |
| 6 | 1.55504739 | -2.49392557 | -1.86634529 |
| 6 | 3.50977755 | -5.32566738 | -0.07139220 |

| | | | |
|---|---|---|---|
| 6 | 0.08699226 | -2.05141163 | -1.91395426 |
| 6 | -0.54674011 | -1.75347161 | -0.54900789 |
| 1 | 3.84695077 | -3.36211777 | -0.75913268 |
| 1 | 1.38032603 | -3.80293226 | -0.14345445 |
| 1 | 2.13999987 | -1.71611774 | -1.34917390 |
| 1 | -0.52570087 | -2.82814503 | -2.40566754 |
| 1 | 3.65319324 | -4.53583384 | -2.07044888 |
| 1 | 1.21219063 | -4.63166094 | -1.68893754 |
| 1 | 1.94975781 | -2.54350376 | -2.89453387 |
| 1 | 0.00481346 | -1.14691544 | -2.53622103 |
| 1 | -0.55422360 | -2.66208196 | 0.08169849 |
| 1 | -1.60027492 | -1.47250414 | -0.69699460 |
| 8 | 3.06191158 | -5.33951712 | 1.06385398 |
| 8 | 0.14217287 | -0.72910970 | 0.16034375 |
| 1 | 5.28645754 | -8.04766178 | -0.17541631 |
| 1 | 3.72091508 | -7.86370182 | 0.70153695 |

Electronic energy = -3908.660004 a.u.
DFT-D3(BJ) dispersion correction = -0.141229 a.u.
Thermal free energy = 0.741419 a.u.
Gibbs free energy = -3908.059815 a.u.
Number of imaginary frequencies = 1.

**TS3_eq_CL isomer/conformer 4**

| Atomic N. | X | Y | Z |
|---|---|---|---|
| 7 | -1.57257199 | 2.86601949 | 1.94073606 |
| 8 | -2.10859108 | 0.58661568 | 0.17559911 |
| 8 | 0.65489650 | 2.31984043 | 0.12456706 |
| 6 | -2.24818540 | 2.56533766 | 3.22569156 |
| 6 | -3.38589978 | 1.00966811 | 0.28779244 |
| 6 | -4.45764256 | 0.10895242 | -0.00513370 |
| 6 | -5.76663303 | 0.59480298 | 0.11734998 |
| 6 | -6.05632401 | 1.90357530 | 0.51235175 |
| 6 | -5.00714350 | 2.77080894 | 0.79510504 |
| 6 | -3.68091607 | 2.34120560 | 0.67756867 |
| 6 | -2.56849289 | 3.32380700 | 0.91466421 |
| 6 | -4.19414711 | -1.34579146 | -0.44003701 |
| 6 | 0.67997497 | 3.65549302 | -0.02846083 |
| 6 | 1.33290493 | 4.22209692 | -1.16649199 |
| 6 | 1.34355319 | 5.61891317 | -1.27615678 |
| 6 | 0.74516892 | 6.45814514 | -0.33087182 |
| 6 | 0.11459801 | 5.89495468 | 0.77308518 |
| 6 | 0.08719348 | 4.50556374 | 0.93849450 |
| 6 | -0.52905869 | 3.92431664 | 2.18185043 |
| 6 | 2.00195765 | 3.33158493 | -2.23193765 |
| 6 | -5.49827242 | -2.11781049 | -0.71590805 |
| 6 | -3.44578266 | -2.10176420 | 0.68344861 |
| 6 | -3.36868548 | -1.36438656 | -1.74804139 |
| 6 | 2.65881896 | 4.16363764 | -3.34932280 |
| 6 | 0.94715458 | 2.41703486 | -2.89899850 |
| 6 | 3.11262155 | 2.46984124 | -1.58474183 |
| 1 | -2.80289698 | 3.45355844 | 3.57220960 |
| 1 | -2.94982600 | 1.73544025 | 3.09411907 |

| | | | |
|---|---|---|---|
| 1 | -2.98832870 | 4.29162931 | 1.23871005 |
| 1 | -2.00119090 | 3.50855541 | -0.00960018 |
| 1 | -6.59907770 | -0.07227688 | -0.09934675 |
| 1 | -5.20662737 | 3.80057311 | 1.10066891 |
| 1 | 0.23369171 | 3.44664907 | 2.81827235 |
| 1 | -0.99357623 | 4.73589420 | 2.76830149 |
| 1 | 1.83240247 | 6.07787037 | -2.13380766 |
| 1 | -0.35666087 | 6.53118467 | 1.52603352 |
| 1 | -5.24724054 | -3.14257169 | -1.02594459 |
| 1 | -6.13533306 | -2.18927765 | 0.17753150 |
| 1 | -6.08511972 | -1.66205931 | -1.52638376 |
| 1 | -2.49973917 | -1.61276031 | 0.93745571 |
| 1 | -4.06586885 | -2.14931631 | 1.59088492 |
| 1 | -3.23442221 | -3.13409305 | 0.36307725 |
| 1 | -3.93370533 | -0.89043236 | -2.56392002 |
| 1 | -2.41965723 | -0.83115017 | -1.63075030 |
| 1 | -3.15613246 | -2.40356708 | -2.04360604 |
| 1 | 3.12457275 | 3.48222160 | -4.07567549 |
| 1 | 3.44792700 | 4.82478333 | -2.96270442 |
| 1 | 1.92689753 | 4.77735615 | -3.89423776 |
| 1 | 0.46564680 | 1.76014078 | -2.16657209 |
| 1 | 0.16946934 | 3.01814795 | -3.39239264 |
| 1 | 1.42820704 | 1.79097545 | -3.66630101 |
| 1 | 3.89084172 | 3.11050272 | -1.14435756 |
| 1 | 2.70787883 | 1.82097208 | -0.80031139 |
| 1 | 3.58926344 | 1.84040439 | -2.35225058 |
| 1 | -1.49604177 | 2.28364038 | 3.96846223 |
| 8 | -0.04019170 | 0.66429794 | 2.93578315 |
| 8 | -0.32047695 | -1.52383220 | 3.11083317 |
| 6 | 0.50746912 | -0.48928243 | 2.87365723 |
| 6 | 1.99580514 | -0.63636053 | 3.11244774 |
| 1 | 2.41859412 | -1.41167665 | 2.46028805 |
| 1 | 2.43946886 | 0.31768253 | 2.80746341 |
| 6 | 2.32280707 | -0.95991111 | 4.58699179 |
| 1 | 1.68325627 | -0.36011913 | 5.25437355 |
| 1 | 3.35737300 | -0.64101779 | 4.78244829 |
| 6 | 2.19981551 | -2.45322633 | 4.91734362 |
| 1 | 2.91628861 | -3.00823665 | 4.28744459 |
| 1 | 2.51076007 | -2.62406230 | 5.95963860 |
| 6 | 0.80080974 | -3.04799628 | 4.71194506 |
| 1 | 0.08610581 | -2.62322927 | 5.43527079 |
| 1 | 0.83732671 | -4.13144732 | 4.91143703 |
| 6 | 0.22114007 | -2.86158943 | 3.31314683 |
| 1 | 0.95422524 | -3.08996320 | 2.52468491 |
| 1 | -0.65210557 | -3.50867176 | 3.16648459 |
| 31 | -0.55959982 | 1.21953273 | 1.04945207 |
| 1 | -7.09116316 | 2.23533916 | 0.59685731 |
| 1 | 0.77285850 | 7.53971624 | -0.46299681 |
| 8 | 3.95629549 | -5.89834642 | -4.90259123 |
| 6 | 5.14043283 | -6.62067652 | -5.31715250 |
| 1 | 5.98518801 | -5.93209171 | -5.44412422 |
| 6 | 2.78378034 | -4.54357100 | -3.34888983 |
| 6 | 2.93086696 | -3.54468584 | -2.20084500 |

| | | | |
|---|---|---|---|
| 6 | 1.59114504 | -2.93262863 | -1.77871656 |
| 6 | 4.08340502 | -5.23313332 | -3.71628046 |
| 6 | 1.73185468 | -1.90789139 | -0.64784086 |
| 6 | 0.38249904 | -1.34921932 | -0.19201450 |
| 1 | 2.37522388 | -4.06534243 | -4.25219488 |
| 1 | 3.62626100 | -2.74502301 | -2.50090551 |
| 1 | 1.11488819 | -2.44892406 | -2.64890218 |
| 1 | 2.36793709 | -1.06942022 | -0.97304988 |
| 1 | 2.06286216 | -5.33607578 | -3.08308840 |
| 1 | 3.40214825 | -4.04855156 | -1.34343970 |
| 1 | 0.90503716 | -3.73895049 | -1.46387887 |
| 1 | 2.24496579 | -2.37732935 | 0.20942183 |
| 1 | -0.26372877 | -2.17223883 | 0.17444429 |
| 1 | -0.14369994 | -0.91179579 | -1.05881810 |
| 8 | 5.10730743 | -5.22691298 | -3.05923700 |
| 8 | 0.53572696 | -0.35941309 | 0.81490761 |
| 1 | 4.87509823 | -7.08895350 | -6.26988649 |
| 1 | 5.40808916 | -7.38022947 | -4.57167768 |

Electronic energy = -3908.659675 a.u.
DFT-D3(BJ) dispersion correction = -0.136841 a.u.
Thermal free energy = 0.738051 a.u.
Gibbs free energy = -3908.058465 a.u.
Number of imaginary frequencies = 1.

**TS3 eq CL isomer/conformer 5**

| Atomic N. | X | Y | Z |
|---|---|---|---|
| 7 | 0.23315448 | 2.66503429 | 2.24421024 |
| 8 | -1.33332634 | 0.93983603 | 0.48792735 |
| 8 | 1.81564105 | 1.71512485 | -0.02582070 |
| 6 | -0.31609315 | 2.40655494 | 3.59707236 |
| 6 | -2.34486079 | 1.77498591 | 0.81978625 |
| 6 | -3.70003629 | 1.36208630 | 0.61744386 |
| 6 | -4.71397018 | 2.25556421 | 0.98932034 |
| 6 | -4.45543385 | 3.51439071 | 1.53725839 |
| 6 | -3.13592863 | 3.91245031 | 1.71437097 |
| 6 | -2.08417010 | 3.06410527 | 1.35068166 |
| 6 | -0.67466766 | 3.57098007 | 1.46729147 |
| 6 | -4.05035734 | -0.00650872 | 0.00372643 |
| 6 | 2.29496479 | 2.97160697 | -0.04053740 |
| 6 | 2.92635012 | 3.46512008 | -1.22425258 |
| 6 | 3.42852211 | 4.77279472 | -1.19277167 |
| 6 | 3.32440209 | 5.59706211 | -0.06798019 |
| 6 | 2.70123792 | 5.10830212 | 1.07489824 |
| 6 | 2.19690251 | 3.80294299 | 1.10408986 |
| 6 | 1.59933734 | 3.27943087 | 2.38381290 |
| 6 | 3.04603672 | 2.59446669 | -2.49108863 |
| 6 | -5.56977844 | -0.20613633 | -0.15769771 |
| 6 | -3.54233217 | -1.14014566 | 0.92541039 |
| 6 | -3.43195486 | -0.12542719 | -1.40970099 |
| 6 | 3.78300238 | 3.32918859 | -3.62677574 |
| 6 | 1.63736427 | 2.22861886 | -3.01653504 |
| 6 | 3.84262252 | 1.30359828 | -2.18434048 |

| | | | |
|---|---|---|---|
| 1 | -0.46379998 | 3.36080050 | 4.13012362 |
| 1 | -1.28002858 | 1.89338982 | 3.51520705 |
| 1 | -0.67568791 | 4.56423903 | 1.94849980 |
| 1 | -0.21712665 | 3.69380283 | 0.47422829 |
| 1 | -5.75179815 | 1.95972836 | 0.84808528 |
| 1 | -2.90430975 | 4.89704704 | 2.12726831 |
| 1 | 2.23061037 | 2.49457169 | 2.82888961 |
| 1 | 1.52834964 | 4.10639620 | 3.11155677 |
| 1 | 3.91521239 | 5.17380142 | -2.08012748 |
| 1 | 2.60646605 | 5.73573351 | 1.96441603 |
| 1 | -5.75016451 | -1.19022799 | -0.61291391 |
| 1 | -6.09747887 | -0.18318389 | 0.80700523 |
| 1 | -6.01742363 | 0.55019236 | -0.81846720 |
| 1 | -2.46559787 | -1.06213188 | 1.10724568 |
| 1 | -4.05944777 | -1.10269940 | 1.89577568 |
| 1 | -3.75283933 | -2.11847854 | 0.46686828 |
| 1 | -3.85402322 | 0.64344639 | -2.07357717 |
| 1 | -2.34452057 | 0.00589965 | -1.38804400 |
| 1 | -3.66594934 | -1.10958540 | -1.84224892 |
| 1 | 3.84441757 | 2.66522098 | -4.50094271 |
| 1 | 4.81047630 | 3.60190463 | -3.34483814 |
| 1 | 3.25519872 | 4.24108934 | -3.94152880 |
| 1 | 1.06848645 | 1.65758407 | -2.27487254 |
| 1 | 1.07085729 | 3.13659525 | -3.26984525 |
| 1 | 1.72641516 | 1.61994982 | -3.92954803 |
| 1 | 4.85635376 | 1.54883838 | -1.83398342 |
| 1 | 3.34112239 | 0.69870365 | -1.42112899 |
| 1 | 3.93938541 | 0.70125949 | -3.10091376 |
| 1 | 0.38383356 | 1.77464473 | 4.15171051 |
| 8 | 1.33519661 | 0.11177931 | 2.73372412 |
| 8 | 1.20702195 | -2.08198285 | 2.94412208 |
| 6 | 1.82432973 | -1.01666284 | 2.40590644 |
| 6 | 3.25088930 | -1.12997508 | 1.91126120 |
| 1 | 3.33442616 | -1.90110779 | 1.13466597 |
| 1 | 3.47866702 | -0.17017648 | 1.43358862 |
| 6 | 4.23691082 | -1.43083715 | 3.06193566 |
| 1 | 3.97150445 | -0.83424914 | 3.94965744 |
| 1 | 5.23603106 | -1.09198630 | 2.75045753 |
| 6 | 4.31157112 | -2.92177629 | 3.41528010 |
| 1 | 4.67375755 | -3.47131371 | 2.52929473 |
| 1 | 5.06686687 | -3.07463384 | 4.20166826 |
| 6 | 2.98582721 | -3.54495144 | 3.87084222 |
| 1 | 2.66487455 | -3.11999822 | 4.83569288 |
| 1 | 3.13580632 | -4.62347031 | 4.04189873 |
| 6 | 1.83181667 | -3.39852452 | 2.88349009 |
| 1 | 2.13842940 | -3.61707520 | 1.84945726 |
| 1 | 1.00923407 | -4.07405233 | 3.14742684 |
| 31 | 0.47365940 | 0.93217713 | 1.03629434 |
| 1 | -5.27845526 | 4.17261505 | 1.81568086 |
| 1 | 3.72266126 | 6.61138535 | -0.09287016 |
| 8 | -3.67991209 | -5.58867645 | -4.28041172 |
| 6 | -5.03291082 | -5.47235823 | -4.78291607 |
| 1 | -5.75614119 | -5.55609226 | -3.96186757 |

| | | | |
|---|---|---|---|
| 6 | -1.92118752 | -4.86938429 | -2.85715628 |
| 6 | -1.26538730 | -3.60813475 | -2.29060555 |
| 6 | 0.08167136 | -3.90572572 | -1.62099159 |
| 6 | -3.32853460 | -4.63327408 | -3.37027144 |
| 6 | 0.83986378 | -2.65828204 | -1.14319026 |
| 6 | 0.09876926 | -1.85080850 | -0.07179457 |
| 1 | -2.00170326 | -5.63954926 | -2.06939721 |
| 1 | -1.95757592 | -3.14325166 | -1.57576144 |
| 1 | -0.08676737 | -4.58186483 | -0.76331168 |
| 1 | 1.06004083 | -1.99677479 | -1.99675965 |
| 1 | -1.31860566 | -5.31991625 | -3.65930223 |
| 1 | -1.12289751 | -2.87450886 | -3.10074496 |
| 1 | 0.72379088 | -4.46061087 | -2.32560682 |
| 1 | 1.81617761 | -2.96701312 | -0.73368633 |
| 1 | -0.25039777 | -2.53146839 | 0.73464894 |
| 1 | -0.80391073 | -1.38705456 | -0.49686173 |
| 8 | -4.07489395 | -3.73836493 | -3.01921511 |
| 8 | 0.94034624 | -0.85036081 | 0.47730660 |
| 1 | -5.15104151 | -6.29907894 | -5.49001741 |
| 1 | -5.17343664 | -4.50718880 | -5.28526115 |

Electronic energy = -3908.657893 a.u.
DFT-D3(BJ) dispersion correction = -0.138974 a.u.
Thermal free energy = 0.739212 a.u.
Gibbs free energy = -3908.057655 a.u.
Number of imaginary frequencies = 1.

**TS3 eq CL isomer/conformer 6**

| Atomic N. | X | Y | Z |
|---|---|---|---|
| 7 | -0.22397059 | 3.09684253 | 2.18588257 |
| 8 | -1.76069307 | 1.10968375 | 0.67710316 |
| 8 | 1.25265586 | 2.08071184 | -0.12460350 |
| 6 | -0.61586750 | 2.92447305 | 3.60523343 |
| 6 | -2.81229138 | 1.84799850 | 1.09233522 |
| 6 | -4.12106371 | 1.27092147 | 1.10349631 |
| 6 | -5.18370199 | 2.07778049 | 1.53297591 |
| 6 | -5.01181269 | 3.40103817 | 1.94790816 |
| 6 | -3.73531437 | 3.95152521 | 1.93557227 |
| 6 | -2.64074349 | 3.19431853 | 1.50463414 |
| 6 | -1.28642130 | 3.84201884 | 1.43104088 |
| 6 | -4.36542463 | -0.18556605 | 0.66342521 |
| 6 | 1.58645082 | 3.37074089 | -0.30578962 |
| 6 | 2.05314445 | 3.80502319 | -1.58488250 |
| 6 | 2.40305686 | 5.15510321 | -1.72043741 |
| 6 | 2.30752707 | 6.07266283 | -0.66940922 |
| 6 | 1.85166693 | 5.63811779 | 0.57021850 |
| 6 | 1.50245774 | 4.29689932 | 0.76401412 |
| 6 | 1.08653617 | 3.83617210 | 2.13447356 |
| 6 | 2.16927075 | 2.82710052 | -2.77109551 |
| 6 | -5.85284805 | -0.57789862 | 0.74490035 |
| 6 | -3.58821130 | -1.15044093 | 1.58993995 |
| 6 | -3.92570710 | -0.37470490 | -0.80746865 |
| 6 | 2.71123099 | 3.51994491 | -4.03559685 |

| | | | |
|---|---|---|---|
| 6 | 0.77746421 | 2.25018024 | -3.12283468 |
| 6 | 3.14287233 | 1.67663217 | -2.42012644 |
| 1 | -0.81648833 | 3.90960169 | 4.05906677 |
| 1 | -1.51962173 | 2.31035399 | 3.67185664 |
| 1 | -1.34317720 | 4.87095070 | 1.82599509 |
| 1 | -0.93827039 | 3.91241002 | 0.38989076 |
| 1 | -6.18920279 | 1.66100943 | 1.55072689 |
| 1 | -3.57454348 | 4.98502827 | 2.25114655 |
| 1 | 1.83080971 | 3.14771867 | 2.56691337 |
| 1 | 1.01244652 | 4.71154499 | 2.80273080 |
| 1 | 2.76069760 | 5.51409388 | -2.68389177 |
| 1 | 1.77039945 | 6.33662415 | 1.40645063 |
| 1 | -5.96502447 | -1.62085044 | 0.41484743 |
| 1 | -6.24428225 | -0.51171798 | 1.77036595 |
| 1 | -6.48116255 | 0.04453506 | 0.09165666 |
| 1 | -2.51445055 | -0.93636996 | 1.58524454 |
| 1 | -3.95405245 | -1.06510210 | 2.62389016 |
| 1 | -3.74182606 | -2.19021463 | 1.26094055 |
| 1 | -4.53024006 | 0.25949639 | -1.47217381 |
| 1 | -2.87149429 | -0.11435514 | -0.94727474 |
| 1 | -4.07463646 | -1.42218399 | -1.11247230 |
| 1 | 2.77944517 | 2.78071928 | -4.84666967 |
| 1 | 3.71782041 | 3.93460727 | -3.88056445 |
| 1 | 2.05086994 | 4.32873917 | -4.38053465 |
| 1 | 0.34598249 | 1.70179939 | -2.27846217 |
| 1 | 0.08426691 | 3.05560493 | -3.40628386 |
| 1 | 0.86425197 | 1.56221461 | -3.97797203 |
| 1 | 4.14745617 | 2.07183576 | -2.20887446 |
| 1 | 2.79673243 | 1.11421061 | -1.54595554 |
| 1 | 3.22532105 | 0.98608404 | -3.27381992 |
| 1 | 0.19608898 | 2.42596626 | 4.14280844 |
| 8 | 0.89196754 | 0.54328823 | 2.81319523 |
| 8 | 0.12914701 | -1.51184082 | 3.11408710 |
| 6 | 1.09974682 | -0.70616460 | 2.64506745 |
| 6 | 2.51346684 | -1.22674489 | 2.49265885 |
| 1 | 2.54672837 | -2.05644298 | 1.77459705 |
| 1 | 3.08708692 | -0.40120268 | 2.05739999 |
| 6 | 3.11707473 | -1.67816877 | 3.84114671 |
| 1 | 2.84090233 | -0.96473420 | 4.63416386 |
| 1 | 4.21279192 | -1.63338768 | 3.75385928 |
| 6 | 2.71479392 | -3.10593367 | 4.23333597 |
| 1 | 3.08353281 | -3.79661012 | 3.45545435 |
| 1 | 3.23034334 | -3.38840222 | 5.16428137 |
| 6 | 1.20743668 | -3.32694340 | 4.41287756 |
| 1 | 0.83178490 | -2.76649952 | 5.28420544 |
| 1 | 1.02264953 | -4.39248419 | 4.62604189 |
| 6 | 0.35363731 | -2.94896436 | 3.20648694 |
| 1 | 0.77927572 | -3.32072616 | 2.26211858 |
| 1 | -0.66065115 | -3.35357141 | 3.30761504 |
| 31 | 0.06959825 | 1.28665304 | 1.10526311 |
| 1 | -5.86855698 | 3.98899388 | 2.27698255 |
| 1 | 2.58473444 | 7.11519098 | -0.82532960 |
| 8 | 0.82140142 | -6.63909864 | -5.47967386 |

| | | | |
|---|---|---|---|
| 6 | 1.61691988 | -7.65032101 | -6.14331007 |
| 1 | 2.55286932 | -7.21742868 | -6.51810884 |
| 6 | 0.50242049 | -5.04631805 | -3.75170088 |
| 6 | 1.20767891 | -4.14496613 | -2.73796892 |
| 6 | 0.23999819 | -3.20034933 | -2.01763248 |
| 6 | 1.42636621 | -6.05800581 | -4.40160465 |
| 6 | 0.94061917 | -2.27715135 | -1.01478899 |
| 6 | -0.04303355 | -1.38689923 | -0.25288299 |
| 1 | 0.01368002 | -4.45992661 | -4.54465818 |
| 1 | 1.98603499 | -3.55875802 | -3.25137830 |
| 1 | -0.29981720 | -2.58712745 | -2.75958705 |
| 1 | 1.66852665 | -1.63410521 | -1.53449786 |
| 1 | -0.30753201 | -5.61681461 | -3.26485777 |
| 1 | 1.73678315 | -4.77300310 | -2.00532985 |
| 1 | -0.52975476 | -3.79609942 | -1.49553835 |
| 1 | 1.51656592 | -2.88727570 | -0.29734841 |
| 1 | -0.76985300 | -2.01500654 | 0.30091769 |
| 1 | -0.63675511 | -0.79797685 | -0.97420335 |
| 8 | 2.54801440 | -6.34570074 | -4.02839565 |
| 8 | 0.62588912 | -0.50680059 | 0.63825256 |
| 1 | 0.99707621 | -8.00948811 | -6.97045755 |
| 1 | 1.85403061 | -8.46831703 | -5.45124674 |

Electronic energy = -3908.659672 a.u.
DFT-D3(BJ) dispersion correction = -0.136796 a.u.
Thermal free energy = 0.739885 a.u.
Gibbs free energy = -3908.056583 a.u.
Number of imaginary frequencies = 1.

**TS3 eq CL isomer/conformer 7**

| Atomic N. | X | Y | Z |
|---|---|---|---|
| 7 | -0.05613568 | 2.92333488 | 2.42593961 |
| 8 | -1.70742262 | 1.09620361 | 0.83651316 |
| 8 | 1.30216876 | 2.03984684 | -0.00805745 |
| 6 | -0.39940156 | 2.65704883 | 3.84339558 |
| 6 | -2.72002281 | 1.82958103 | 1.34644002 |
| 6 | -4.04393045 | 1.28867379 | 1.36753277 |
| 6 | -5.06500958 | 2.08965172 | 1.89695043 |
| 6 | -4.83846335 | 3.37324122 | 2.40072843 |
| 6 | -3.54746365 | 3.88848251 | 2.37807461 |
| 6 | -2.49293104 | 3.13628470 | 1.84948000 |
| 6 | -1.12352788 | 3.75078208 | 1.77004569 |
| 6 | -4.34813289 | -0.12428397 | 0.83290056 |
| 6 | 1.66886264 | 3.32985800 | -0.10774472 |
| 6 | 2.10376262 | 3.84397502 | -1.36855687 |
| 6 | 2.49053864 | 5.18942213 | -1.42154482 |
| 6 | 2.46046204 | 6.02963866 | -0.30415663 |
| 6 | 2.03566018 | 5.51872597 | 0.91747341 |
| 6 | 1.65218334 | 4.17739118 | 1.02822956 |
| 6 | 1.27276225 | 3.62936220 | 2.37703412 |
| 6 | 2.14682614 | 2.95249580 | -2.62641310 |
| 6 | -5.84244340 | -0.48134944 | 0.94566005 |
| 6 | -3.56515645 | -1.17541341 | 1.65482578 |

| | | | |
|---|---|---|---|
| 6 | -3.97058061 | -0.21648099 | -0.66431459 |
| 6 | 2.66858150 | 3.71865002 | -3.85686000 |
| 6 | 0.72532131 | 2.44594375 | -2.96748648 |
| 6 | 3.09189787 | 1.74911246 | -2.39573599 |
| 1 | -0.55309287 | 3.61092767 | 4.37554252 |
| 1 | -1.31800882 | 2.06444258 | 3.90088277 |
| 1 | -1.13471357 | 4.74872577 | 2.24110008 |
| 1 | -0.81251802 | 3.88871350 | 0.72390878 |
| 1 | -6.08082490 | 1.69945820 | 1.92398641 |
| 1 | -3.34424729 | 4.89085994 | 2.76214693 |
| 1 | 2.01185183 | 2.89159158 | 2.72978353 |
| 1 | 1.24899454 | 4.45527245 | 3.10891957 |
| 1 | 2.82495968 | 5.60715771 | -2.36978236 |
| 1 | 2.00622035 | 6.15683883 | 1.80388589 |
| 1 | -5.99823499 | -1.49377370 | 0.54566046 |
| 1 | -6.19212314 | -0.48028058 | 1.98820633 |
| 1 | -6.47640058 | 0.20419998 | 0.36507477 |
| 1 | -2.48628836 | -0.99157767 | 1.62120947 |
| 1 | -3.88734420 | -1.15575776 | 2.70647721 |
| 1 | -3.76337530 | -2.18370311 | 1.25854504 |
| 1 | -4.58114169 | 0.48063885 | -1.25656441 |
| 1 | -2.91526063 | 0.02535766 | -0.82722863 |
| 1 | -4.16181608 | -1.23440526 | -1.03803992 |
| 1 | 2.68467703 | 3.03808523 | -4.72028675 |
| 1 | 3.69309431 | 4.08986559 | -3.70940065 |
| 1 | 2.02428885 | 4.57007809 | -4.11975412 |
| 1 | 0.30324898 | 1.85421708 | -2.14791613 |
| 1 | 0.05141243 | 3.29093847 | -3.17023633 |
| 1 | 0.76022555 | 1.81691176 | -3.87036912 |
| 1 | 4.11607488 | 2.09607659 | -2.19466255 |
| 1 | 2.75777821 | 1.13586175 | -1.55160906 |
| 1 | 3.12048022 | 1.12059329 | -3.29938030 |
| 1 | 0.41681648 | 2.09813521 | 4.31051616 |
| 8 | 0.99548399 | 0.29430414 | 2.82148980 |
| 8 | 0.19135077 | -1.76005855 | 2.99114965 |
| 6 | 1.16452582 | -0.94270980 | 2.54846510 |
| 6 | 2.55813353 | -1.48230772 | 2.30324510 |
| 1 | 2.54493806 | -2.25082597 | 1.51949583 |
| 1 | 3.13818782 | -0.63759420 | 1.91588604 |
| 6 | 3.19558276 | -2.05689765 | 3.58779003 |
| 1 | 2.96504503 | -1.40524619 | 4.44589763 |
| 1 | 4.28847925 | -2.03003491 | 3.46494882 |
| 6 | 2.77053414 | -3.50266346 | 3.87640350 |
| 1 | 3.09365414 | -4.13473063 | 3.03130785 |
| 1 | 3.31105436 | -3.87304846 | 4.76121640 |
| 6 | 1.26523298 | -3.70306389 | 4.09329066 |
| 1 | 0.93560083 | -3.20925664 | 5.02168766 |
| 1 | 1.06012626 | -4.77816650 | 4.22390026 |
| 6 | 0.37938174 | -3.20499003 | 2.95563153 |
| 1 | 0.76047708 | -3.50572208 | 1.96797507 |
| 1 | -0.64128588 | -3.59202561 | 3.06152658 |
| 31 | 0.14136257 | 1.19030612 | 1.20587790 |
| 1 | -5.66437101 | 3.95786107 | 2.80593392 |

| | | | |
|---|---|---|---|
| 1 | 2.76382776 | 7.07265038 | -0.39424410 |
| 8 | 0.45188937 | -6.15699618 | -6.04103102 |
| 6 | 1.19480754 | -7.12651918 | -6.81811464 |
| 1 | 2.13070423 | -6.68754145 | -7.18590010 |
| 6 | 0.23661294 | -4.71564367 | -4.17024865 |
| 6 | 0.99865087 | -3.93181510 | -3.10146577 |
| 6 | 0.08202282 | -3.02612201 | -2.27247338 |
| 6 | 1.10800674 | -5.69139097 | -4.93705669 |
| 6 | 0.83897406 | -2.21443047 | -1.21558563 |
| 6 | -0.09471970 | -1.36503952 | -0.35097004 |
| 1 | -0.25391288 | -4.04464682 | -4.89184195 |
| 1 | 1.78227251 | -3.32619297 | -3.58337540 |
| 1 | -0.45854330 | -2.33486337 | -2.94174237 |
| 1 | 1.57162654 | -1.54988479 | -1.70036774 |
| 1 | -0.57835600 | -5.30291308 | -3.71265245 |
| 1 | 1.52563339 | -4.63908585 | -2.44330091 |
| 1 | -0.69140007 | -3.64262303 | -1.78077615 |
| 1 | 1.41542596 | -2.90093798 | -0.57125064 |
| 1 | -0.82061875 | -2.01751644 | 0.17511611 |
| 1 | -0.69463847 | -0.70463792 | -1.00187968 |
| 8 | 2.23117063 | -6.04295829 | -4.62879183 |
| 8 | 0.62633013 | -0.57680852 | 0.58452914 |
| 1 | 0.53803483 | -7.39404689 | -7.65145151 |
| 1 | 1.42956642 | -8.00879321 | -6.20921264 |

Electronic energy = -3908.659701 a.u.
DFT-D3(BJ) dispersion correction = -0.136807 a.u.
Thermal free energy = 0.740440 a.u.
Gibbs free energy = -3908.056068 a.u.
Number of imaginary frequencies = 1.

**Int3a CL isomer/conformer 1**

| Atomic N. | X | Y | Z |
|---|---|---|---|
| 7 | 0.59479268 | 2.93409444 | 2.23331060 |
| 8 | -1.12557971 | 1.05263950 | 0.70026032 |
| 8 | 1.84177429 | 1.67445199 | -0.15226948 |
| 6 | 0.39354543 | 2.76524026 | 3.69293409 |
| 6 | -2.12687171 | 1.78619632 | 1.24369631 |
| 6 | -3.45613403 | 1.26193365 | 1.25891025 |
| 6 | -4.45712973 | 2.05203639 | 1.84089460 |
| 6 | -4.20668989 | 3.31155181 | 2.39161573 |
| 6 | -2.91499339 | 3.82275586 | 2.34751011 |
| 6 | -1.87856066 | 3.08278989 | 1.76657755 |
| 6 | -0.52459305 | 3.72285233 | 1.61873518 |
| 6 | -3.79020947 | -0.11795576 | 0.65896967 |
| 6 | 2.17384915 | 2.94561942 | -0.45438847 |
| 6 | 2.52535777 | 3.28043101 | -1.79803991 |
| 6 | 2.87936298 | 4.61019746 | -2.06215816 |
| 6 | 2.89496340 | 5.60080821 | -1.07555566 |
| 6 | 2.55340356 | 5.26323419 | 0.22895284 |
| 6 | 2.20542726 | 3.94617727 | 0.55150754 |
| 6 | 1.92592227 | 3.59611708 | 1.98926057 |
| 6 | 2.51594636 | 2.22096961 | -2.91775727 |

| | | | |
|---|---|---|---|
| 6 | -5.29649440 | -0.43438942 | 0.73034386 |
| 6 | -3.05440747 | -1.22352939 | 1.45165682 |
| 6 | -3.39267590 | -0.16089348 | -0.83613121 |
| 6 | 2.94996532 | 2.81003169 | -4.27324711 |
| 6 | 1.08774255 | 1.65421601 | -3.09993999 |
| 6 | 3.50166832 | 1.07789440 | -2.57665683 |
| 1 | 0.38535193 | 3.75135987 | 4.18680390 |
| 1 | -0.56250888 | 2.26368504 | 3.87643030 |
| 1 | -0.53578223 | 4.72985288 | 2.07040001 |
| 1 | -0.26516258 | 3.84276982 | 0.55619839 |
| 1 | -5.47639309 | 1.67138465 | 1.87119353 |
| 1 | -2.69805386 | 4.81539457 | 2.74895877 |
| 1 | 2.67969489 | 2.89241545 | 2.37906368 |
| 1 | 1.97250077 | 4.51196702 | 2.60306579 |
| 1 | 3.14926167 | 4.89307372 | -3.07808559 |
| 1 | 2.56335674 | 6.01978443 | 1.01703903 |
| 1 | -5.47429995 | -1.41829163 | 0.27322463 |
| 1 | -5.66389318 | -0.47948369 | 1.76588996 |
| 1 | -5.89962952 | 0.30035054 | 0.17763084 |
| 1 | -1.97208129 | -1.06220160 | 1.47373064 |
| 1 | -3.41378836 | -1.25263077 | 2.49092828 |
| 1 | -3.25499120 | -2.20611770 | 0.99663155 |
| 1 | -3.96905009 | 0.58223110 | -1.40647688 |
| 1 | -2.32796500 | 0.05345318 | -0.97781003 |
| 1 | -3.61476272 | -1.15253510 | -1.25866714 |
| 1 | 2.93106753 | 2.01375664 | -5.03118696 |
| 1 | 3.97304015 | 3.21192362 | -4.24114561 |
| 1 | 2.27340724 | 3.60670860 | -4.61482548 |
| 1 | 0.71937381 | 1.18496348 | -2.18143261 |
| 1 | 0.38771839 | 2.45308003 | -3.38457965 |
| 1 | 1.08471866 | 0.90113833 | -3.90327978 |
| 1 | 4.52749213 | 1.46564120 | -2.49143945 |
| 1 | 3.23511734 | 0.58752216 | -1.63410844 |
| 1 | 3.49203339 | 0.32591987 | -3.38107253 |
| 1 | 1.20240134 | 2.14791556 | 4.09728274 |
| 8 | 1.44906197 | 0.12857454 | 2.61550565 |
| 8 | 0.46668644 | -1.91079481 | 2.77850167 |
| 6 | 1.48007047 | -1.15489521 | 2.15718192 |
| 6 | 2.89201309 | -1.75196241 | 2.17451200 |
| 1 | 2.90012851 | -2.73214258 | 1.67086285 |
| 1 | 3.48725353 | -1.07060233 | 1.55266875 |
| 6 | 3.51003101 | -1.86421873 | 3.57742244 |
| 1 | 3.26206135 | -0.95425686 | 4.14578077 |
| 1 | 4.60585093 | -1.87781188 | 3.47330807 |
| 6 | 3.07921873 | -3.11621774 | 4.35015478 |
| 1 | 3.42303388 | -4.00845537 | 3.79733335 |
| 1 | 3.59731286 | -3.14090509 | 5.32199802 |
| 6 | 1.56923083 | -3.23617951 | 4.58030453 |
| 1 | 1.20599010 | -2.42399900 | 5.23011993 |
| 1 | 1.36036657 | -4.18093934 | 5.10969630 |
| 6 | 0.73097614 | -3.22784601 | 3.30141068 |
| 1 | 1.18427780 | -3.87952379 | 2.53309113 |
| 1 | -0.27463385 | -3.61772760 | 3.51123858 |

| | | | |
|---|---|---|---|
| 31 | 0.67461526 | 1.12374014 | 1.20409923 |
| 1 | -5.01737814 | 3.88570450 | 2.84024379 |
| 1 | 3.17042030 | 6.62404652 | -1.33057345 |
| 8 | -2.69544805 | -4.85438126 | -5.31404335 |
| 6 | -4.01328650 | -4.70603847 | -5.89750333 |
| 1 | -4.78186609 | -5.07909490 | -5.20895800 |
| 6 | -1.13227309 | -4.43562586 | -3.57587205 |
| 6 | -0.62391778 | -3.29215331 | -2.69385176 |
| 6 | 0.68173195 | -3.65236128 | -1.97320953 |
| 6 | -2.51538406 | -4.17692156 | -4.14268218 |
| 6 | 1.31405141 | -2.49113514 | -1.18911793 |
| 6 | 0.42096472 | -1.97930500 | -0.06119465 |
| 1 | -1.20857416 | -5.36309342 | -2.98082579 |
| 1 | -1.41172687 | -3.03421135 | -1.97271261 |
| 1 | 0.49546797 | -4.49951123 | -1.28933495 |
| 1 | 1.54105852 | -1.65772205 | -1.87225749 |
| 1 | -0.43924408 | -4.65707303 | -4.39993262 |
| 1 | -0.46840693 | -2.39415674 | -3.31395001 |
| 1 | 1.41710844 | -4.01259188 | -2.71140874 |
| 1 | 2.27523415 | -2.81937652 | -0.76387349 |
| 1 | 0.12663691 | -2.78689760 | 0.62509173 |
| 1 | -0.50317993 | -1.53469351 | -0.45290314 |
| 8 | -3.37509651 | -3.48494285 | -3.63039284 |
| 8 | 1.07955701 | -0.93993248 | 0.70155574 |
| 1 | -3.99167484 | -5.30144450 | -6.81524330 |
| 1 | -4.21587596 | -3.65142546 | -6.12159454 |

Electronic energy = -3908.664288 a.u.
DFT-D3(BJ) dispersion correction = -0.138734 a.u.
Thermal free energy = 0.738140 a.u.
Gibbs free energy = -3908.064883 a.u.
Number of imaginary frequencies = 0.

**Int3a CL isomer/conformer 2**

| Atomic N. | X | Y | Z |
|---|---|---|---|
| 7 | -1.54950933 | 2.98797884 | 2.07568984 |
| 8 | -2.08936432 | 0.75679536 | 0.17969776 |
| 8 | 0.66582327 | 2.29198671 | 0.22901266 |
| 6 | -2.10175396 | 2.69319413 | 3.42027385 |
| 6 | -3.37908206 | 1.11614132 | 0.38623590 |
| 6 | -4.42839288 | 0.20819499 | 0.04535949 |
| 6 | -5.74691975 | 0.62148440 | 0.28205575 |
| 6 | -6.06533162 | 1.86799119 | 0.82682679 |
| 6 | -5.03795690 | 2.75248468 | 1.13374077 |
| 6 | -3.70359309 | 2.39732446 | 0.90561823 |
| 6 | -2.62929043 | 3.42688341 | 1.12973850 |
| 6 | -4.13409409 | -1.17775183 | -0.56139316 |
| 6 | 0.65803610 | 3.61501682 | -0.02969309 |
| 6 | 1.26142677 | 4.10096168 | -1.22962953 |
| 6 | 1.24510208 | 5.48535679 | -1.44595554 |
| 6 | 0.66564598 | 6.38635117 | -0.54721414 |
| 6 | 0.08317621 | 5.90185094 | 0.61841479 |
| 6 | 0.08403950 | 4.52885457 | 0.89117672 |

| | | | |
|---|---|---|---|
| 6 | -0.47779615 | 4.04026577 | 2.19993636 |
| 6 | 1.90586361 | 3.14141041 | -2.24959409 |
| 6 | -5.42307359 | -1.94613940 | -0.90975780 |
| 6 | -3.34893007 | -2.03819056 | 0.45605466 |
| 6 | -3.33243698 | -1.02388640 | -1.87590742 |
| 6 | 2.51087209 | 3.89574837 | -3.44855609 |
| 6 | 0.84061229 | 2.16853425 | -2.80905713 |
| 6 | 3.05172063 | 2.34370568 | -1.58224758 |
| 1 | -2.55672312 | 3.60292447 | 3.84658677 |
| 1 | -2.86530025 | 1.91227316 | 3.34117031 |
| 1 | -3.07858087 | 4.35822506 | 1.51554895 |
| 1 | -2.12396169 | 3.67367617 | 0.18404283 |
| 1 | -6.56315786 | -0.05537237 | 0.03645818 |
| 1 | -5.26174984 | 3.74170859 | 1.53971433 |
| 1 | 0.30949594 | 3.58578845 | 2.82356065 |
| 1 | -0.89773074 | 4.89411785 | 2.75872027 |
| 1 | 1.69595857 | 5.88357675 | -2.35322814 |
| 1 | -0.37091850 | 6.58803133 | 1.33707948 |
| 1 | -5.15090161 | -2.91582586 | -1.35115076 |
| 1 | -6.03866435 | -2.15024164 | -0.02191392 |
| 1 | -6.03910815 | -1.40879912 | -1.64522230 |
| 1 | -2.42416912 | -1.54920665 | 0.77735076 |
| 1 | -3.96143206 | -2.22736657 | 1.34982277 |
| 1 | -3.09552922 | -3.01180749 | 0.00792020 |
| 1 | -3.92113935 | -0.47012291 | -2.62188051 |
| 1 | -2.39156181 | -0.48715866 | -1.71519246 |
| 1 | -3.10783285 | -2.01720724 | -2.29430744 |
| 1 | 2.96077830 | 3.16764827 | -4.13862415 |
| 1 | 3.30336146 | 4.59319075 | -3.14080552 |
| 1 | 1.75140274 | 4.45796030 | -4.01096042 |
| 1 | 0.38954140 | 1.56527418 | -2.01386950 |
| 1 | 0.03988133 | 2.72257383 | -3.32009170 |
| 1 | 1.30394887 | 1.49078302 | -3.54267175 |
| 1 | 3.83603870 | 3.02412953 | -1.21943615 |
| 1 | 2.68665708 | 1.75043727 | -0.73686719 |
| 1 | 3.50964418 | 1.66508821 | -2.31857117 |
| 1 | -1.29503663 | 2.33619093 | 4.06884054 |
| 8 | -0.08858077 | 0.51248152 | 2.80452822 |
| 8 | -0.31957129 | -1.73564564 | 2.70003806 |
| 6 | 0.51897369 | -0.64514238 | 2.41639287 |
| 6 | 1.96486149 | -0.73941553 | 2.91749016 |
| 1 | 2.48681610 | -1.58310050 | 2.44047877 |
| 1 | 2.44595132 | 0.17632536 | 2.55009677 |
| 6 | 2.08792957 | -0.83511989 | 4.44689045 |
| 1 | 1.34509941 | -0.16640642 | 4.90904573 |
| 1 | 3.07733508 | -0.45232717 | 4.74043149 |
| 6 | 1.93988032 | -2.26302448 | 4.98688915 |
| 1 | 2.74442903 | -2.88602429 | 4.55775241 |
| 1 | 2.10314793 | -2.26084542 | 6.07625358 |
| 6 | 0.59149965 | -2.92518789 | 4.68340137 |
| 1 | -0.22439918 | -2.40889774 | 5.21419498 |
| 1 | 0.60631808 | -3.96187850 | 5.05972812 |
| 6 | 0.22493712 | -2.97510770 | 3.20015515 |

| | | | |
|---|---|---|---|
| 1 | 1.08261269 | -3.31034929 | 2.59124836 |
| 1 | -0.59227362 | -3.69248560 | 3.04131316 |
| 31 | -0.62553444 | 1.33975390 | 1.19216708 |
| 1 | -7.10601133 | 2.14208640 | 0.99969315 |
| 1 | 0.67142779 | 7.45479486 | -0.76242634 |
| 8 | 4.06843851 | -5.89494213 | -4.87997602 |
| 6 | 5.27624077 | -6.51879735 | -5.37883772 |
| 1 | 6.03820226 | -5.75987337 | -5.59615427 |
| 6 | 2.90800204 | -4.60391759 | -3.26559487 |
| 6 | 3.05493747 | -3.58019017 | -2.13987040 |
| 6 | 1.70180472 | -3.06765105 | -1.63589277 |
| 6 | 4.23166893 | -5.18421563 | -3.72557268 |
| 6 | 1.83825425 | -2.01725096 | -0.52675260 |
| 6 | 0.47296555 | -1.55655170 | -0.02216802 |
| 1 | 2.39505048 | -4.17357101 | -4.13940539 |
| 1 | 3.66411811 | -2.73382476 | -2.49386109 |
| 1 | 1.13367284 | -2.63461544 | -2.47717567 |
| 1 | 2.39275783 | -1.14334324 | -0.90297804 |
| 1 | 2.27887672 | -5.45073632 | -2.94147301 |
| 1 | 3.61966003 | -4.03302458 | -1.31112055 |
| 1 | 1.10302831 | -3.91928312 | -1.26752737 |
| 1 | 2.42934991 | -2.43634653 | 0.30357732 |
| 1 | -0.08439342 | -2.37373185 | 0.45897394 |
| 1 | -0.13864584 | -1.19326414 | -0.86048741 |
| 8 | 5.30112863 | -5.06253010 | -3.15887861 |
| 8 | 0.55951697 | -0.44395720 | 0.89635892 |
| 1 | 4.97616547 | -7.04025188 | -6.29278513 |
| 1 | 5.67634703 | -7.22565086 | -4.64101068 |

Electronic energy = -3908.666130 a.u.
DFT-D3(BJ) dispersion correction = -0.137288 a.u.
Thermal free energy = 0.739123 a.u.
Gibbs free energy = -3908.064294 a.u.
Number of imaginary frequencies = 0.

**Int3a CL isomer/conformer 3**

| Atomic N. | X | Y | Z |
|---|---|---|---|
| 7 | -0.66638545 | 3.17045277 | 1.48779355 |
| 8 | -2.08059953 | 1.00156476 | 0.04195586 |
| 8 | 0.90950412 | 1.78493904 | -0.60847182 |
| 6 | -1.10289324 | 3.13882324 | 2.90460994 |
| 6 | -3.16614897 | 1.77187812 | 0.29718882 |
| 6 | -4.47027486 | 1.19227725 | 0.20690895 |
| 6 | -5.57034450 | 2.02108168 | 0.46608462 |
| 6 | -5.44081232 | 3.36808793 | 0.81246151 |
| 6 | -4.16955173 | 3.92265268 | 0.89474694 |
| 6 | -3.03503621 | 3.14717903 | 0.62758012 |
| 6 | -1.69216509 | 3.82797904 | 0.61326058 |
| 6 | -4.67520777 | -0.29561077 | -0.14031620 |
| 6 | 1.26259879 | 3.04484927 | -0.94388451 |
| 6 | 1.77825298 | 3.30726054 | -2.25163533 |
| 6 | 2.15006587 | 4.62445822 | -2.55178258 |
| 6 | 2.02898555 | 5.67452394 | -1.63769061 |

| | | | |
|---|---|---|---|
| 6 | 1.52074969 | 5.41098448 | -0.37169759 |
| 6 | 1.14793106 | 4.11020829 | -0.01230309 |
| 6 | 0.66625829 | 3.86419398 | 1.39309619 |
| 6 | 1.92501153 | 2.18801625 | -3.30067053 |
| 6 | -6.16623391 | -0.66664119 | -0.25717642 |
| 6 | -4.07359983 | -1.16849225 | 0.98592071 |
| 6 | -4.02220352 | -0.63323156 | -1.50177300 |
| 6 | 2.50945568 | 2.70873451 | -4.62738798 |
| 6 | 0.53983913 | 1.57795257 | -3.61987281 |
| 6 | 2.88559534 | 1.09660963 | -2.77397465 |
| 1 | -1.23399139 | 4.16756458 | 3.27967892 |
| 1 | -2.05494714 | 2.60358089 | 2.98473271 |
| 1 | -1.80535401 | 4.87962606 | 0.92806685 |
| 1 | -1.26877292 | 3.83662354 | -0.40230224 |
| 1 | -6.57239700 | 1.60113166 | 0.40292485 |
| 1 | -4.04141402 | 4.97650920 | 1.15235654 |
| 1 | 1.37068950 | 3.22706215 | 1.95285250 |
| 1 | 0.59268091 | 4.82847961 | 1.92427392 |
| 1 | 2.54541865 | 4.84805954 | -3.54086742 |
| 1 | 1.41458821 | 6.21504186 | 0.36017220 |
| 1 | -6.24814119 | -1.73191211 | -0.51710996 |
| 1 | -6.70877566 | -0.51677911 | 0.68724515 |
| 1 | -6.67416595 | -0.09299201 | -1.04596142 |
| 1 | -3.01595737 | -0.94352567 | 1.16021921 |
| 1 | -4.61496062 | -1.00055679 | 1.92862145 |
| 1 | -4.16936501 | -2.23512024 | 0.72820448 |
| 1 | -4.49055226 | -0.04874264 | -2.30726825 |
| 1 | -2.94903962 | -0.41762949 | -1.50148858 |
| 1 | -4.17077977 | -1.69982096 | -1.73083151 |
| 1 | 2.59476925 | 1.87238141 | -5.33511020 |
| 1 | 3.51516197 | 3.13410808 | -4.49794597 |
| 1 | 1.86692050 | 3.46959549 | -5.09359089 |
| 1 | 0.06132107 | 1.17260255 | -2.72208592 |
| 1 | -0.12613451 | 2.33931060 | -4.05149519 |
| 1 | 0.64810972 | 0.76740705 | -4.35701206 |
| 1 | 3.88722187 | 1.51712507 | -2.60121411 |
| 1 | 2.52214302 | 0.66523682 | -1.83554487 |
| 1 | 2.98141017 | 0.29174377 | -3.51910188 |
| 1 | -0.34800847 | 2.61367638 | 3.49894535 |
| 8 | 0.33910568 | 0.51888827 | 2.26783762 |
| 8 | -0.72267847 | -1.45570830 | 2.59883047 |
| 6 | 0.40135779 | -0.82270819 | 2.02174845 |
| 6 | 1.77187496 | -1.41624768 | 2.36266919 |
| 1 | 1.84244532 | -2.45330591 | 1.99606717 |
| 1 | 2.48315778 | -0.82139871 | 1.77465586 |
| 6 | 2.13504711 | -1.35124305 | 3.85495852 |
| 1 | 1.80910608 | -0.37946910 | 4.25771842 |
| 1 | 3.23201190 | -1.37273715 | 3.94455114 |
| 6 | 1.55735944 | -2.50112338 | 4.68855767 |
| 1 | 1.96299171 | -3.45369325 | 4.30484630 |
| 1 | 1.91488383 | -2.41246201 | 5.72677129 |
| 6 | 0.02752496 | -2.58390248 | 4.68812962 |
| 1 | -0.41231924 | -1.70206443 | 5.18115413 |

| | | | |
|---|---|---|---|
| 1 | -0.28497883 | -3.46339255 | 5.27583914 |
| 6 | -0.59906806 | -2.70948720 | 3.29909440 |
| 1 | -0.05821150 | -3.45770131 | 2.69343004 |
| 1 | -1.63963341 | -3.05137122 | 3.38791859 |
| 31 | -0.34715056 | 1.28088590 | 0.68711921 |
| 1 | -6.32587200 | 3.97184388 | 1.01275951 |
| 1 | 2.32503131 | 6.68488187 | -1.91940705 |
| 8 | 5.11682997 | -5.83411704 | -1.66005436 |
| 6 | 5.68925759 | -7.05266688 | -1.12705027 |
| 1 | 5.99305959 | -6.90922778 | -0.08241557 |
| 6 | 3.51731740 | -4.08120908 | -1.58548700 |
| 6 | 2.02862571 | -3.82943480 | -1.33399034 |
| 6 | 1.59811753 | -2.41963515 | -1.75331974 |
| 6 | 4.00637520 | -5.39217359 | -1.00121201 |
| 6 | 0.10412271 | -2.13511108 | -1.54216692 |
| 6 | -0.33001706 | -2.04528520 | -0.08433481 |
| 1 | 4.11763644 | -3.28363042 | -1.11325802 |
| 1 | 1.82537387 | -4.00305662 | -0.26733873 |
| 1 | 2.18929914 | -1.67221919 | -1.20054809 |
| 1 | -0.49781019 | -2.93024789 | -2.01449678 |
| 1 | 3.76279695 | -4.04947457 | -2.65659912 |
| 1 | 1.43536061 | -4.57898927 | -1.88353203 |
| 1 | 1.83735052 | -2.26951269 | -2.81864420 |
| 1 | -0.17318321 | -1.19715456 | -2.04665793 |
| 1 | 0.01999328 | -2.92221170 | 0.48019265 |
| 1 | -1.42408588 | -2.00166191 | -0.00133402 |
| 8 | 3.51048833 | -5.97947764 | -0.05718413 |
| 8 | 0.24147977 | -0.85587657 | 0.51817681 |
| 1 | 6.55760348 | -7.26449662 | -1.75818418 |
| 1 | 4.96155343 | -7.87213246 | -1.17814527 |

Electronic energy = -3908.665537 a.u.
DFT-D3(BJ) dispersion correction = -0.140107 a.u.
Thermal free energy = 0.743522 a.u.
Gibbs free energy = -3908.062122 a.u.
Number of imaginary frequencies = 0.

**Int3a CL isomer/conformer 4**

| Atomic N. | X | Y | Z |
|---|---|---|---|
| 7 | -0.42243906 | 3.03939372 | 1.82095062 |
| 8 | -1.98362369 | 1.17146340 | 0.12754312 |
| 8 | 1.08242072 | 1.74648593 | -0.38459193 |
| 6 | -0.89483502 | 2.89582853 | 3.21917781 |
| 6 | -3.00400757 | 2.00459121 | 0.44644982 |
| 6 | -4.35109057 | 1.55717088 | 0.27283189 |
| 6 | -5.38086384 | 2.44762843 | 0.60591096 |
| 6 | -5.14316863 | 3.73227085 | 1.10021223 |
| 6 | -3.83086431 | 4.15931384 | 1.26094844 |
| 6 | -2.76195905 | 3.31927416 | 0.92683158 |
| 6 | -1.36504546 | 3.87510576 | 1.00702891 |
| 6 | -4.67676222 | 0.13958278 | -0.23774654 |
| 6 | 1.55117265 | 2.99847671 | -0.57845852 |
| 6 | 2.12337330 | 3.34873229 | -1.84133757 |

| | | | |
|---|---|---|---|
| 6 | 2.61251044 | 4.65277496 | -1.99448278 |
| 6 | 2.55569090 | 5.60915074 | -0.97711552 |
| 6 | 1.99424897 | 5.26067727 | 0.24514395 |
| 6 | 1.50328578 | 3.96699179 | 0.45861730 |
| 6 | 0.96727068 | 3.61812146 | 1.82189484 |
| 6 | 2.20716984 | 2.33410874 | -2.99790563 |
| 6 | -6.19091259 | -0.08038626 | -0.42073231 |
| 6 | -4.18578918 | -0.89893873 | 0.79794966 |
| 6 | -4.01885371 | -0.10952484 | -1.61576300 |
| 6 | 2.87064007 | 2.93422412 | -4.25190903 |
| 6 | 0.78644433 | 1.87913702 | -3.40675196 |
| 6 | 3.05930984 | 1.11759826 | -2.56712014 |
| 1 | -0.94475518 | 3.88617921 | 3.70176473 |
| 1 | -1.89171215 | 2.44249304 | 3.22632126 |
| 1 | -1.39405620 | 4.89314869 | 1.43199287 |
| 1 | -0.91725147 | 3.95388095 | 0.00505345 |
| 1 | -6.41379212 | 2.12793341 | 0.48225652 |
| 1 | -3.61783061 | 5.16366998 | 1.63414430 |
| 1 | 1.59971193 | 2.86530295 | 2.32067282 |
| 1 | 0.96449552 | 4.52353424 | 2.45249270 |
| 1 | 3.05213740 | 4.94202560 | -2.94723538 |
| 1 | 1.93788090 | 5.99045212 | 1.05610900 |
| 1 | -6.35893047 | -1.09925044 | -0.79859093 |
| 1 | -6.74333282 | 0.01381867 | 0.52514853 |
| 1 | -6.62511829 | 0.61934062 | -1.14947349 |
| 1 | -3.11779785 | -0.78984293 | 1.01517037 |
| 1 | -4.73652918 | -0.78429542 | 1.74320714 |
| 1 | -4.36719551 | -1.91909626 | 0.42424372 |
| 1 | -4.41164371 | 0.59669948 | -2.36194150 |
| 1 | -2.93132865 | 0.00694371 | -1.57305115 |
| 1 | -4.25429810 | -1.12809528 | -1.96104202 |
| 1 | 2.90524018 | 2.16649553 | -5.03825096 |
| 1 | 3.90414781 | 3.25670441 | -4.05962232 |
| 1 | 2.30689984 | 3.78987837 | -4.65080432 |
| 1 | 0.25025763 | 1.42784179 | -2.56530037 |
| 1 | 0.19844110 | 2.73371900 | -3.77204287 |
| 1 | 0.84920083 | 1.14038700 | -4.22062619 |
| 1 | 4.08655570 | 1.43245657 | -2.33142543 |
| 1 | 2.63455612 | 0.62600495 | -1.68577824 |
| 1 | 3.11032116 | 0.38691387 | -3.38909886 |
| 1 | -0.20363025 | 2.24473001 | 3.76434751 |
| 8 | 0.32766353 | 0.23860174 | 2.32439008 |
| 8 | -0.91727290 | -1.65207111 | 2.42524732 |
| 6 | 0.27412838 | -1.06947219 | 1.93652821 |
| 6 | 1.57564858 | -1.82086906 | 2.23392621 |
| 1 | 1.56113170 | -2.81485712 | 1.75779685 |
| 1 | 2.35338840 | -1.23562221 | 1.72586778 |
| 6 | 1.90337564 | -1.95000255 | 3.73022676 |
| 1 | 1.65586848 | -1.00103090 | 4.23120908 |
| 1 | 2.99117097 | -2.08233846 | 3.83523259 |
| 6 | 1.20239506 | -3.12423092 | 4.42367763 |
| 1 | 1.53009336 | -4.06341651 | 3.94436427 |
| 1 | 1.53913962 | -3.18137013 | 5.47099368 |

| | | | |
|---|---|---|---|
| 6 | -0.32813763 | -3.06434986 | 4.38917311 |
| 1 | -0.69923520 | -2.20401865 | 4.96908173 |
| 1 | -0.73418263 | -3.96942154 | 4.87141583 |
| 6 | -0.92690350 | -2.98026349 | 2.98483900 |
| 1 | -0.44076974 | -3.70580481 | 2.30925583 |
| 1 | -1.99622481 | -3.23123344 | 3.01878457 |
| 31 | -0.24908573 | 1.22445338 | 0.82624074 |
| 1 | -5.97694908 | 4.38713018 | 1.35305375 |
| 1 | 2.94327326 | 6.61379104 | -1.14535190 |
| 8 | 4.65233747 | -6.00131430 | -2.25761068 |
| 6 | 5.11283988 | -7.32046667 | -1.87679728 |
| 1 | 5.36358343 | -7.34604251 | -0.80881659 |
| 6 | 3.16631779 | -4.16680803 | -2.01470784 |
| 6 | 1.70242469 | -3.81983726 | -1.73533037 |
| 6 | 1.39666143 | -2.34051176 | -1.99418883 |
| 6 | 3.53577363 | -5.57687852 | -1.59722902 |
| 6 | -0.07017786 | -1.95507030 | -1.75471308 |
| 6 | -0.51161006 | -1.99204107 | -0.29685182 |
| 1 | 3.82932791 | -3.48661392 | -1.45163906 |
| 1 | 1.47549878 | -4.09299315 | -0.69459725 |
| 1 | 2.04270947 | -1.71224400 | -1.36060617 |
| 1 | -0.73180894 | -2.63827359 | -2.31422290 |
| 1 | 3.42468262 | -4.02730405 | -3.07441663 |
| 1 | 1.05262103 | -4.45078802 | -2.36396271 |
| 1 | 1.65904996 | -2.09338389 | -3.03575258 |
| 1 | -0.26163966 | -0.94648448 | -2.15138120 |
| 1 | -0.24197292 | -2.95309458 | 0.16597492 |
| 1 | -1.59902172 | -1.86588819 | -0.21158724 |
| 8 | 2.94980418 | -6.25228733 | -0.77126336 |
| 8 | 0.15090261 | -0.92996491 | 0.43606796 |
| 1 | 6.00118176 | -7.50434283 | -2.48857177 |
| 1 | 4.33803257 | -8.07019691 | -2.07996669 |

Electronic energy = -3908.665542 a.u.
DFT-D3(BJ) dispersion correction = -0.140117 a.u.
Thermal free energy = 0.743583 a.u.
Gibbs free energy = -3908.062076 a.u.
Number of imaginary frequencies = 0.

**Int3a CL isomer/conformer 5**

| Atomic N. | X | Y | Z |
|---|---|---|---|
| 7 | -1.89889218 | 1.25839399 | 2.46207063 |
| 8 | -1.69218544 | 0.36446445 | -0.36081287 |
| 8 | 0.50336764 | 2.20562652 | 0.98206936 |
| 6 | -2.40230200 | 0.15812529 | 3.32054336 |
| 6 | -3.03658625 | 0.24298314 | -0.23048319 |
| 6 | -3.76066780 | -0.58381437 | -1.14307317 |
| 6 | -5.15136418 | -0.66259072 | -0.98462769 |
| 6 | -5.84109543 | 0.02093397 | 0.01970375 |
| 6 | -5.12728534 | 0.82344613 | 0.90172657 |
| 6 | -3.73900524 | 0.95111689 | 0.77986235 |
| 6 | -3.01380103 | 1.90272854 | 1.69150559 |
| 6 | -3.04969547 | -1.37506319 | -2.25751260 |

| | | | |
|---|---|---|---|
| 6 | 0.11687993 | 3.41096240 | 1.45834026 |
| 6 | 0.58346155 | 4.60620800 | 0.82907096 |
| 6 | 0.17051147 | 5.82969517 | 1.37408036 |
| 6 | -0.66997405 | 5.91940628 | 2.48693852 |
| 6 | -1.11879961 | 4.75083343 | 3.09080730 |
| 6 | -0.72657085 | 3.50164422 | 2.59577792 |
| 6 | -1.17480753 | 2.26036388 | 3.32015656 |
| 6 | 1.50909541 | 4.56264010 | -0.40232991 |
| 6 | -4.04173428 | -2.16606614 | -3.13164628 |
| 6 | -2.07571418 | -2.39948209 | -1.62976941 |
| 6 | -2.28861020 | -0.40934692 | -3.19610190 |
| 6 | 1.87852634 | 5.97154858 | -0.90365209 |
| 6 | 0.80456504 | 3.83277872 | -1.56980022 |
| 6 | 2.83094973 | 3.84638030 | -0.03900737 |
| 1 | -3.11580269 | 0.55665394 | 4.06109190 |
| 1 | -2.90752344 | -0.59065975 | 2.70178441 |
| 1 | -3.72461451 | 2.35365333 | 2.40528174 |
| 1 | -2.55700302 | 2.72366950 | 1.11900832 |
| 1 | -5.72456157 | -1.28737962 | -1.66699276 |
| 1 | -5.64370340 | 1.37525010 | 1.69055524 |
| 1 | -0.31635734 | 1.71610079 | 3.74697949 |
| 1 | -1.83894468 | 2.54522509 | 4.15405873 |
| 1 | 0.51030216 | 6.75439907 | 0.91128752 |
| 1 | -1.77515073 | 4.79529495 | 3.96290060 |
| 1 | -3.47972504 | -2.70207830 | -3.90993771 |
| 1 | -4.59952457 | -2.91602452 | -2.55231167 |
| 1 | -4.76383216 | -1.50890671 | -3.63760966 |
| 1 | -1.31455475 | -1.91259739 | -1.01090937 |
| 1 | -2.62589366 | -3.11488318 | -1.00068834 |
| 1 | -1.56758922 | -2.96678777 | -2.42476569 |
| 1 | -2.98850272 | 0.28818277 | -3.67925129 |
| 1 | -1.54039918 | 0.17475666 | -2.65020061 |
| 1 | -1.78239660 | -0.98218786 | -3.98823955 |
| 1 | 2.53969574 | 5.87706449 | -1.77704792 |
| 1 | 2.41756334 | 6.55433635 | -0.14285242 |
| 1 | 0.99440803 | 6.54386739 | -1.21946938 |
| 1 | 0.52500991 | 2.81031150 | -1.29604652 |
| 1 | -0.10720429 | 4.37107256 | -1.86667901 |
| 1 | 1.47255252 | 3.79210507 | -2.44385568 |
| 1 | 3.37180928 | 4.40604091 | 0.73807827 |
| 1 | 2.64419371 | 2.83282817 | 0.33188207 |
| 1 | 3.48105882 | 3.78496157 | -0.92537246 |
| 1 | -1.55502888 | -0.31402532 | 3.82842250 |
| 8 | 0.17743730 | -0.79542975 | 2.09052849 |
| 8 | 1.30291479 | -2.55393012 | 1.23173459 |
| 6 | 1.34426985 | -1.17629762 | 1.47554394 |
| 6 | 2.58381345 | -0.64756934 | 2.21515425 |
| 1 | 3.50302600 | -0.83134803 | 1.63811836 |
| 1 | 2.44866602 | 0.44244103 | 2.25943817 |
| 6 | 2.73839763 | -1.21972427 | 3.63432308 |
| 1 | 1.74408144 | -1.28760670 | 4.10266681 |
| 1 | 3.31858230 | -0.50275368 | 4.23555771 |
| 6 | 3.44816742 | -2.57881295 | 3.67934343 |

| | | | |
|---|---|---|---|
| 1 | 4.46577935 | -2.45750097 | 3.26760043 |
| 1 | 3.57805472 | -2.88592761 | 4.72937513 |
| 6 | 2.73736559 | -3.69633287 | 2.90841369 |
| 1 | 1.77379286 | -3.94379854 | 3.38243695 |
| 1 | 3.35361544 | -4.61013533 | 2.94896200 |
| 6 | 2.46894730 | -3.38138782 | 1.43716748 |
| 1 | 3.36076395 | -2.94354399 | 0.95736073 |
| 1 | 2.23204884 | -4.29874545 | 0.88405090 |
| 31 | -0.49915238 | 0.61681259 | 1.05360564 |
| 8 | 1.19132915 | -0.47128922 | 0.14106576 |
| 1 | -6.92320466 | -0.07658693 | 0.10684807 |
| 1 | -0.96969892 | 6.89437806 | 2.87093786 |
| 8 | 0.63816708 | -5.12774278 | -3.03462319 |
| 6 | 0.04476668 | -6.00426829 | -2.04393173 |
| 1 | 0.69538493 | -6.86769142 | -1.85764080 |
| 6 | 2.41477535 | -3.71543897 | -3.76721558 |
| 6 | 2.78222550 | -2.33695926 | -3.19007926 |
| 6 | 1.55443598 | -1.50863823 | -2.79157432 |
| 6 | 1.86500599 | -4.64060739 | -2.69883528 |
| 6 | 1.90625101 | -0.15491664 | -2.15807988 |
| 6 | 2.34645046 | -0.24750780 | -0.70034868 |
| 1 | 3.31919693 | -4.19959449 | -4.16746079 |
| 1 | 3.36715183 | -1.78651655 | -3.94453189 |
| 1 | 0.93520183 | -1.34007243 | -3.68700557 |
| 1 | 1.04441365 | 0.52671739 | -2.21344728 |
| 1 | 1.69049173 | -3.61938891 | -4.58717470 |
| 1 | 3.44623384 | -2.49294115 | -2.32561176 |
| 1 | 0.92657081 | -2.07646688 | -2.08768130 |
| 1 | 2.71889001 | 0.32797566 | -2.72725484 |
| 1 | 2.85163143 | 0.67856781 | -0.38430016 |
| 1 | 3.04197838 | -1.08984187 | -0.56557416 |
| 8 | 2.43959305 | -4.92354271 | -1.66200942 |
| 1 | -0.91019630 | -6.32054498 | -2.47388230 |
| 1 | -0.11416473 | -5.45965094 | -1.10490813 |

Electronic energy = -3908.663676 a.u.
DFT-D3(BJ) dispersion correction = -0.143147 a.u.
Thermal free energy = 0.745598 a.u.
Gibbs free energy = -3908.061225 a.u.
Number of imaginary frequencies = 0.

**Int3a CL isomer/conformer 6**

| Atomic N. | X | Y | Z |
|---|---|---|---|
| 7 | -0.13264804 | 3.21229585 | 2.29178225 |
| 8 | -1.70733501 | 1.26697769 | 0.68137399 |
| 8 | 1.27060981 | 2.02528940 | -0.03576713 |
| 6 | -0.38969077 | 3.02727849 | 3.74072618 |
| 6 | -2.75845181 | 1.95235110 | 1.19190047 |
| 6 | -4.06211533 | 1.36758294 | 1.16628471 |
| 6 | -5.11905067 | 2.11377670 | 1.70608383 |
| 6 | -4.94618504 | 3.38571844 | 2.25757225 |
| 6 | -3.67720965 | 3.95276847 | 2.26083253 |
| 6 | -2.58785403 | 3.25859761 | 1.72213113 |

| | | | |
|---|---|---|---|
| 6 | -1.25712859 | 3.95520114 | 1.63097235 |
| 6 | -4.30948734 | -0.03397559 | 0.57451689 |
| 6 | 1.55567644 | 3.31133247 | -0.32240312 |
| 6 | 1.94555859 | 3.66613576 | -1.64988267 |
| 6 | 2.24984840 | 5.01160848 | -1.89610154 |
| 6 | 2.18084609 | 5.99760582 | -0.90699359 |
| 6 | 1.80208193 | 5.64004415 | 0.38194769 |
| 6 | 1.50090251 | 4.30731208 | 0.68643414 |
| 6 | 1.17656491 | 3.93565878 | 2.10901314 |
| 6 | 2.02866182 | 2.61105869 | -2.77075553 |
| 6 | -5.80010650 | -0.42252764 | 0.60149442 |
| 6 | -3.54572396 | -1.09120703 | 1.40509534 |
| 6 | -3.85947347 | -0.07591215 | -0.90519776 |
| 6 | 2.49017060 | 3.22369849 | -4.10650926 |
| 6 | 0.63590914 | 1.98135093 | -3.01140347 |
| 6 | 3.04872851 | 1.51052766 | -2.39259036 |
| 1 | -0.46567912 | 4.00942068 | 4.23667178 |
| 1 | -1.32769721 | 2.47999186 | 3.88107308 |
| 1 | -1.32947272 | 4.95965382 | 2.08253280 |
| 1 | -0.95857287 | 4.08867367 | 0.58043057 |
| 1 | -6.12036531 | 1.68710568 | 1.70299718 |
| 1 | -3.51929310 | 4.95410548 | 2.66806926 |
| 1 | 1.94422974 | 3.26431798 | 2.52774713 |
| 1 | 1.15464569 | 4.84830312 | 2.72886318 |
| 1 | 2.54800787 | 5.31072623 | -2.89940245 |
| 1 | 1.74603736 | 6.39311746 | 1.17141686 |
| 1 | -5.91570567 | -1.42099825 | 0.15557678 |
| 1 | -6.19897036 | -0.47127594 | 1.62496912 |
| 1 | -6.41989059 | 0.27413328 | 0.01868591 |
| 1 | -2.47278670 | -0.88027615 | 1.44893333 |
| 1 | -3.92934426 | -1.11835720 | 2.43551963 |
| 1 | -3.69025588 | -2.09034161 | 0.96456290 |
| 1 | -4.45189331 | 0.62893577 | -1.50681704 |
| 1 | -2.80189104 | 0.18651845 | -1.01319986 |
| 1 | -4.01877894 | -1.08509954 | -1.31544023 |
| 1 | 2.53828489 | 2.43006735 | -4.86584243 |
| 1 | 3.49188237 | 3.67110728 | -4.03194561 |
| 1 | 1.79288730 | 3.99043512 | -4.47405862 |
| 1 | 0.25578906 | 1.48870836 | -2.11000888 |
| 1 | -0.08889642 | 2.74930735 | -3.31834013 |
| 1 | 0.69919594 | 1.23483331 | -3.81819502 |
| 1 | 4.05249538 | 1.94160472 | -2.26471113 |
| 1 | 2.76563947 | 1.00496623 | -1.46296584 |
| 1 | 3.10423835 | 0.76260362 | -3.19877316 |
| 1 | 0.42891446 | 2.44669223 | 4.17857899 |
| 8 | 0.76655291 | 0.41914766 | 2.71028525 |
| 8 | -0.06135480 | -1.68592203 | 2.74994410 |
| 6 | 0.92957223 | -0.84300914 | 2.22070948 |
| 6 | 2.37929241 | -1.32856110 | 2.33440537 |
| 1 | 2.52118545 | -2.26272439 | 1.76963238 |
| 1 | 2.97090526 | -0.55839857 | 1.82273024 |
| 6 | 2.86337593 | -1.50511706 | 3.78283019 |
| 1 | 2.46478425 | -0.68257627 | 4.39694040 |

| | | | |
|---|---|---|---|
| 1 | 3.95922950 | -1.40261755 | 3.79800705 |
| 6 | 2.49364665 | -2.86310582 | 4.39238925 |
| 1 | 2.96941872 | -3.65874277 | 3.79223328 |
| 1 | 2.92724267 | -2.94118025 | 5.40197561 |
| 6 | 0.98844236 | -3.14074229 | 4.47051357 |
| 1 | 0.50077578 | -2.44922764 | 5.17616167 |
| 1 | 0.82980514 | -4.15829092 | 4.86548948 |
| 6 | 0.25105873 | -3.04130566 | 3.13511494 |
| 1 | 0.80387505 | -3.56712366 | 2.33720103 |
| 1 | -0.73725165 | -3.51466066 | 3.21811329 |
| 31 | 0.06804039 | 1.41104849 | 1.26027963 |
| 1 | -5.79786959 | 3.92481570 | 2.67233782 |
| 1 | 2.42053914 | 7.03314812 | -1.14807916 |
| 8 | 0.98661166 | -6.61365273 | -5.52957388 |
| 6 | 1.82760963 | -7.49404736 | -6.31341494 |
| 1 | 2.64011460 | -6.92800619 | -6.78590525 |
| 6 | 0.66397859 | -5.09530123 | -3.73785104 |
| 6 | 1.35365600 | -4.17649591 | -2.72965462 |
| 6 | 0.35497974 | -3.35413647 | -1.90857685 |
| 6 | 1.63231426 | -5.96563579 | -4.51592902 |
| 6 | 1.03944795 | -2.40959368 | -0.91288519 |
| 6 | 0.01884425 | -1.62652357 | -0.09070405 |
| 1 | 0.05926616 | -4.52250429 | -4.45805236 |
| 1 | 2.04131539 | -3.50151505 | -3.26257621 |
| 1 | -0.28446128 | -2.76403388 | -2.58754138 |
| 1 | 1.68371559 | -1.69783664 | -1.45241104 |
| 1 | -0.04526591 | -5.77076801 | -3.22972726 |
| 1 | 1.98359625 | -4.78193742 | -2.06062739 |
| 1 | -0.32208490 | -4.03695672 | -1.36562785 |
| 1 | 1.69654823 | -2.99292452 | -0.24774771 |
| 1 | -0.58935220 | -2.28581258 | 0.54607998 |
| 1 | -0.67077189 | -1.08685069 | -0.75549007 |
| 8 | 2.81946994 | -6.09641828 | -4.28529610 |
| 8 | 0.62661790 | -0.60761819 | 0.73495800 |
| 1 | 1.16645725 | -7.92820003 | -7.06949169 |
| 1 | 2.26027336 | -8.27761401 | -5.67865393 |

Electronic energy = -3908.666149 a.u.
DFT-D3(BJ) dispersion correction = -0.137267 a.u.
Thermal free energy = 0.742229 a.u.
Gibbs free energy = -3908.061187 a.u.
Number of imaginary frequencies = 0.

**Int3b CL isomer/conformer 1**

| Atomic N. | X | Y | Z |
|---|---|---|---|
| 6 | -2.89646034 | -0.75292612 | 2.29614745 |
| 6 | -3.76659960 | -1.86703061 | 1.70622085 |
| 6 | -3.78359702 | -1.95817129 | 0.17184542 |
| 6 | -1.40862979 | -0.77066461 | 1.93257460 |
| 6 | -2.45797560 | -2.45728227 | -0.42497874 |
| 6 | -1.38025136 | -1.39268760 | -0.61895989 |
| 1 | -3.27300226 | 0.23236416 | 1.98646770 |
| 1 | -4.79581903 | -1.70597621 | 2.06450215 |

| | | | |
|---|---|---|---|
| 1 | -4.58055066 | -2.66009086 | -0.11944339 |
| 1 | -2.63107739 | -2.88828802 | -1.42425516 |
| 1 | -2.94339296 | -0.78800227 | 3.39485141 |
| 1 | -3.44353724 | -2.83743349 | 2.11550402 |
| 1 | -4.05595329 | -0.98276248 | -0.26454210 |
| 1 | -2.07105060 | -3.27203607 | 0.20393720 |
| 1 | -0.40146702 | -1.86338406 | -0.79582794 |
| 1 | -1.61876733 | -0.76850075 | -1.49246374 |
| 8 | -0.72071217 | 0.27288671 | 2.49035682 |
| 8 | -1.25734581 | -0.42840764 | 0.46047071 |
| 7 | 1.06205185 | 2.60704993 | 2.18096460 |
| 8 | -0.88122067 | 2.42518740 | -0.06131578 |
| 8 | 1.52463068 | 0.43292568 | 0.18176793 |
| 6 | 0.43958584 | 2.83407540 | 3.50826018 |
| 6 | -1.15799453 | 3.70387329 | 0.28689989 |
| 6 | -2.36537859 | 4.31452188 | -0.16794097 |
| 6 | -2.61369982 | 5.63379714 | 0.23680665 |
| 6 | -1.73144537 | 6.35915789 | 1.04226212 |
| 6 | -0.53988843 | 5.76676947 | 1.44588900 |
| 6 | -0.23781450 | 4.45525540 | 1.06331656 |
| 6 | 1.11416264 | 3.88189820 | 1.38929698 |
| 1 | -1.97225709 | 7.38054447 | 1.33693622 |
| 6 | -3.35317496 | 3.56654801 | -1.08504871 |
| 6 | 2.73343509 | 1.02771906 | 0.06178953 |
| 6 | 3.52672926 | 0.80175213 | -1.10420254 |
| 6 | 4.77841015 | 1.42989252 | -1.16399018 |
| 6 | 5.26473416 | 2.25341278 | -0.14440626 |
| 6 | 4.48534512 | 2.46279905 | 0.98722932 |
| 6 | 3.23162215 | 1.85100057 | 1.10459495 |
| 6 | 2.44316097 | 2.03560215 | 2.37386915 |
| 1 | 6.24265205 | 2.72503265 | -0.24049032 |
| 6 | 3.03483558 | -0.09835258 | -2.25465463 |
| 6 | -4.56224324 | 4.44079816 | -1.46740092 |
| 6 | -3.89973556 | 2.30795518 | -0.37299234 |
| 6 | -2.64420289 | 3.16018705 | -2.39931843 |
| 6 | 4.06199695 | -0.18981435 | -3.39905375 |
| 6 | 1.72974550 | 0.47258233 | -2.85836772 |
| 6 | 2.80061923 | -1.53655562 | -1.73562397 |
| 1 | 1.04320602 | 3.55396962 | 4.08607809 |
| 1 | -0.57022604 | 3.23654729 | 3.37614853 |
| 1 | 1.71158466 | 4.62042330 | 1.95155382 |
| 1 | 1.66384055 | 3.64458339 | 0.46645609 |
| 1 | -3.53275016 | 6.12015081 | -0.08510175 |
| 1 | 0.18035875 | 6.32484256 | 2.04868379 |
| 1 | 2.29385992 | 1.07406253 | 2.89145326 |
| 1 | 2.99909285 | 2.70084601 | 3.05659785 |
| 1 | 5.40197609 | 1.27902416 | -2.04332520 |
| 1 | 4.84632697 | 3.09885568 | 1.79872638 |
| 1 | -5.22342161 | 3.86483622 | -2.13063274 |
| 1 | -5.15320831 | 4.73899175 | -0.58915950 |
| 1 | -4.26104398 | 5.34908230 | -2.00889008 |
| 1 | -3.09315943 | 1.60689973 | -0.13320612 |
| 1 | -4.41673557 | 2.58252710 | 0.55859671 |

| | | | |
|---|---|---|---|
| 1 | -4.62590724 | 1.79689528 | -1.02422696 |
| 1 | -2.29319649 | 4.05127096 | -2.93979996 |
| 1 | -1.78409705 | 2.50946058 | -2.20656135 |
| 1 | -3.34980796 | 2.62502694 | -3.05340843 |
| 1 | 3.66182864 | -0.84757904 | -4.18371821 |
| 1 | 5.01757297 | -0.61887438 | -3.06437612 |
| 1 | 4.26089988 | 0.78975760 | -3.85756518 |
| 1 | 0.93324156 | 0.54317351 | -2.11057717 |
| 1 | 1.90229347 | 1.47658076 | -3.27264509 |
| 1 | 1.38342859 | -0.17700574 | -3.67646970 |
| 1 | 3.74428139 | -1.97486608 | -1.37858663 |
| 1 | 2.07997429 | -1.54793497 | -0.91120885 |
| 1 | 2.41301703 | -2.17497847 | -2.54312670 |
| 1 | 0.37423550 | 1.88156284 | 4.04383703 |
| 31 | 0.03352403 | 1.20198156 | 1.01749533 |
| 8 | 1.05284948 | -7.16676266 | -2.22661404 |
| 6 | 1.34236592 | -7.18745427 | -3.64593083 |
| 1 | 0.48327435 | -6.81519385 | -4.21798474 |
| 6 | 0.41553046 | -6.00560419 | -0.25093878 |
| 6 | 0.92565493 | -4.79521908 | 0.53988423 |
| 6 | 0.34939811 | -4.74189915 | 1.95815157 |
| 6 | 0.76242676 | -5.93125691 | -1.72523653 |
| 6 | 0.86665954 | -3.57117867 | 2.80777240 |
| 6 | 0.53874412 | -2.17815555 | 2.28254871 |
| 1 | -0.68680172 | -6.04231992 | -0.19537328 |
| 1 | 0.66522704 | -3.88573530 | -0.01855739 |
| 1 | -0.74987170 | -4.68735842 | 1.90365095 |
| 1 | 1.96474287 | -3.63425899 | 2.89602305 |
| 1 | 0.78270367 | -6.95315999 | 0.16626856 |
| 1 | 2.02700277 | -4.82591701 | 0.58379261 |
| 1 | 0.58486644 | -5.68239150 | 2.48380909 |
| 1 | 0.46260933 | -3.66137515 | 3.82867492 |
| 1 | 0.97238457 | -2.00526752 | 1.28438169 |
| 1 | 0.94604524 | -1.41472823 | 2.96114948 |
| 8 | 0.76476406 | -4.91785917 | -2.40032055 |
| 8 | -0.90022772 | -2.04514342 | 2.22606878 |
| 1 | 1.54341050 | -8.23618288 | -3.88463986 |
| 1 | 2.21677956 | -6.56291375 | -3.86710508 |

Electronic energy = -3908.670204 a.u.
DFT-D3(BJ) dispersion correction = -0.142390 a.u.
Thermal free energy = 0.741792 a.u.
Gibbs free energy = -3908.070802 a.u.
Number of imaginary frequencies = 0.

**Int3b CL isomer/conformer 2**

| Atomic N. | X | Y | Z |
|---|---|---|---|
| 6 | -2.87600925 | -0.37049043 | 2.41692838 |
| 6 | -3.76221756 | -1.55276991 | 2.01291570 |
| 6 | -3.79923512 | -1.87623851 | 0.51063251 |
| 6 | -1.39306052 | -0.45835023 | 2.04397449 |
| 6 | -2.48471068 | -2.47151011 | -0.01885838 |
| 6 | -1.40223875 | -1.45825138 | -0.38460768 |

| | | | |
|---|---|---|---|
| 1 | -3.24823359 | 0.55985530 | 1.96482751 |
| 1 | -4.78559911 | -1.32925481 | 2.35402087 |
| 1 | -4.60443671 | -2.60768958 | 0.33925388 |
| 1 | -2.67341960 | -3.04738240 | -0.93918740 |
| 1 | -2.90985095 | -0.23746265 | 3.50859971 |
| 1 | -3.44179768 | -2.45271641 | 2.56153824 |
| 1 | -4.07116496 | -0.97671868 | -0.06640924 |
| 1 | -2.09549291 | -3.18506693 | 0.72163585 |
| 1 | -0.42939079 | -1.95899955 | -0.50092782 |
| 1 | -1.64860590 | -0.97079028 | -1.33920548 |
| 8 | -0.68918764 | 0.65012223 | 2.43034865 |
| 8 | -1.25771496 | -0.34327266 | 0.53542787 |
| 7 | 1.11584786 | 2.88801680 | 1.75253800 |
| 8 | -0.86053610 | 2.39678078 | -0.41283257 |
| 8 | 1.52761874 | 0.43255487 | 0.09739705 |
| 6 | 0.51582648 | 3.31819688 | 3.03926353 |
| 6 | -1.12138903 | 3.71578300 | -0.25380714 |
| 6 | -2.33019313 | 4.26564474 | -0.77725095 |
| 6 | -2.56037051 | 5.63305882 | -0.56929447 |
| 6 | -1.65923156 | 6.46002788 | 0.10696513 |
| 6 | -0.46686792 | 5.92084918 | 0.57727041 |
| 6 | -0.18262358 | 4.56383348 | 0.38933364 |
| 6 | 1.16848184 | 4.02954442 | 0.77849498 |
| 1 | -1.88636251 | 7.51641692 | 0.25017833 |
| 6 | -3.33864599 | 3.40112923 | -1.55941629 |
| 6 | 2.74029808 | 0.98797383 | -0.12698032 |
| 6 | 3.51487865 | 0.57805861 | -1.25476508 |
| 6 | 4.77096037 | 1.17626188 | -1.42588085 |
| 6 | 5.27921110 | 2.14008778 | -0.55014275 |
| 6 | 4.51853923 | 2.52716673 | 0.54690381 |
| 6 | 3.26142699 | 1.95386483 | 0.77237666 |
| 6 | 2.49415591 | 2.33592740 | 2.01011443 |
| 1 | 6.25959601 | 2.58115277 | -0.72988353 |
| 6 | 3.00062544 | -0.48444803 | -2.24589187 |
| 6 | -4.54603902 | 4.22219904 | -2.04963637 |
| 6 | -3.88460544 | 2.26738759 | -0.66143707 |
| 6 | -2.65375166 | 2.79740595 | -2.80902541 |
| 6 | 4.01024029 | -0.75973684 | -3.37637517 |
| 6 | 1.68947550 | -0.00497100 | -2.91274904 |
| 6 | 2.76704405 | -1.82276050 | -1.50628507 |
| 1 | 1.13538583 | 4.10880656 | 3.49484746 |
| 1 | -0.49175299 | 3.70874250 | 2.86213728 |
| 1 | 1.78185673 | 4.83653624 | 1.21555541 |
| 1 | 1.70139501 | 3.65030785 | -0.10596753 |
| 1 | -3.47997350 | 6.07647036 | -0.94683807 |
| 1 | 0.26768896 | 6.55403573 | 1.08033231 |
| 1 | 2.34379623 | 1.46454994 | 2.66801688 |
| 1 | 3.06669918 | 3.08950729 | 2.57761924 |
| 1 | 5.38048307 | 0.88631568 | -2.27982269 |
| 1 | 4.89703046 | 3.27496367 | 1.24756528 |
| 1 | -5.22272862 | 3.56160794 | -2.61040478 |
| 1 | -5.12021118 | 4.65381215 | -1.21687179 |
| 1 | -4.24563583 | 5.03682653 | -2.72422314 |

| | | | |
|---|---|---|---|
| 1 | -3.08014589 | 1.60186909 | -0.33059278 |
| 1 | -4.38659475 | 2.68189208 | 0.22552917 |
| 1 | -4.62325344 | 1.67219658 | -1.22055210 |
| 1 | -2.30271814 | 3.59499265 | -3.47987103 |
| 1 | -1.79694197 | 2.17234291 | -2.53422548 |
| 1 | -3.37433961 | 2.17986494 | -3.36716738 |
| 1 | 3.59454138 | -1.52734901 | -4.04454550 |
| 1 | 4.96807231 | -1.13999603 | -2.99271023 |
| 1 | 4.20826185 | 0.13578458 | -3.98316493 |
| 1 | 0.90356466 | 0.18212442 | -2.17403179 |
| 1 | 1.85938056 | 0.92352168 | -3.47711157 |
| 1 | 1.33091790 | -0.77008844 | -3.61786167 |
| 1 | 3.71369256 | -2.20552230 | -1.09692788 |
| 1 | 2.05775863 | -1.70065969 | -0.68093129 |
| 1 | 2.36576162 | -2.57771224 | -2.19839775 |
| 1 | 0.44893806 | 2.45716792 | 3.71199314 |
| 31 | 0.05604863 | 1.33714348 | 0.82587011 |
| 8 | 0.99592773 | -7.38650485 | -1.16717770 |
| 6 | 1.26462832 | -7.60652826 | -2.57370127 |
| 1 | 0.38731763 | -7.34263290 | -3.17745339 |
| 6 | 0.35723818 | -5.96436204 | 0.63054758 |
| 6 | 0.88716919 | -4.65674971 | 1.23191785 |
| 6 | 0.33533413 | -4.39646234 | 2.63687413 |
| 6 | 0.67674459 | -6.09945171 | -0.84553588 |
| 6 | 0.87152408 | -3.11927344 | 3.30122427 |
| 6 | 0.54713450 | -1.81387225 | 2.58390155 |
| 1 | -0.74396382 | -5.98355958 | 0.71021242 |
| 1 | 0.62097908 | -3.83491982 | 0.55332099 |
| 1 | -0.76443081 | -4.34318696 | 2.59239709 |
| 1 | 1.97009564 | -3.17841160 | 3.38518821 |
| 1 | 0.72907297 | -6.84485124 | 1.17176290 |
| 1 | 1.98895541 | -4.68893167 | 1.26189139 |
| 1 | 0.57574560 | -5.25298593 | 3.28870594 |
| 1 | 0.48032035 | -3.05752473 | 4.32899804 |
| 1 | 0.97303197 | -1.78876682 | 1.56796572 |
| 1 | 0.96407271 | -0.96321272 | 3.14310097 |
| 8 | 0.63761402 | -5.19596358 | -1.66106789 |
| 8 | -0.89155041 | -1.67933036 | 2.52041128 |
| 1 | 1.49040633 | -8.67353607 | -2.66140591 |
| 1 | 2.11841148 | -6.99945653 | -2.89941485 |

Electronic energy = -3908.670198 a.u.
DFT-D3(BJ) dispersion correction = -0.142500 a.u.
Thermal free energy = 0.742150 a.u.
Gibbs free energy = -3908.070549 a.u.
Number of imaginary frequencies = 0.

**Int3b CL isomer/conformer 3**

| Atomic N. | X | Y | Z |
|---|---|---|---|
| 6 | -3.45460551 | -1.10757249 | 1.81619392 |
| 6 | -4.34860199 | -2.27245020 | 1.37970732 |
| 6 | -4.44709138 | -2.50574205 | -0.13671550 |
| 6 | -1.99475555 | -1.14933729 | 1.35712090 |

| | | | |
|---|---|---|---|
| 6 | -3.15689721 | -3.07391978 | -0.74950794 |
| 6 | -2.07794670 | -2.04356140 | -1.08899019 |
| 1 | -3.86039575 | -0.15443360 | 1.44843585 |
| 1 | -5.35795200 | -2.07919093 | 1.77641545 |
| 1 | -5.26108557 | -3.22455547 | -0.31898787 |
| 1 | -3.38618789 | -3.59247202 | -1.69483570 |
| 1 | -3.43072491 | -1.04844443 | 2.91457398 |
| 1 | -3.99891340 | -3.20075730 | 1.85861727 |
| 1 | -4.73674289 | -1.57315473 | -0.64880527 |
| 1 | -2.74662199 | -3.83169332 | -0.06685514 |
| 1 | -1.10079591 | -2.53722703 | -1.20698990 |
| 1 | -2.31936637 | -1.53669421 | -2.03448287 |
| 8 | -1.28286394 | -0.05624986 | 1.77175665 |
| 8 | -1.95043666 | -0.94807662 | -0.14725739 |
| 7 | 0.52445319 | 2.19409478 | 1.16191188 |
| 8 | -1.59200240 | 1.88315610 | -0.91092309 |
| 8 | 0.79653789 | -0.11417248 | -0.72021051 |
| 6 | 0.02643388 | 2.51059575 | 2.52364995 |
| 6 | -1.84701099 | 3.17996277 | -0.61380990 |
| 6 | -3.08822276 | 3.76493888 | -1.00593175 |
| 6 | -3.30841325 | 5.10501031 | -0.65663090 |
| 6 | -2.36766136 | 5.87329962 | 0.03466158 |
| 6 | -1.14523832 | 5.30386122 | 0.37424351 |
| 6 | -0.87005127 | 3.97268005 | 0.04307474 |
| 6 | 0.50619803 | 3.41683614 | 0.28981834 |
| 1 | -2.58826419 | 6.90960222 | 0.29052568 |
| 6 | -4.14118534 | 2.96869397 | -1.80200970 |
| 6 | 1.98941210 | 0.46502396 | -0.99092663 |
| 6 | 2.66935625 | 0.16052316 | -2.20921018 |
| 6 | 3.91323825 | 0.77009895 | -2.42478670 |
| 6 | 4.49520345 | 1.65056571 | -1.50862432 |
| 6 | 3.82371666 | 1.94002476 | -0.32642366 |
| 6 | 2.58399334 | 1.34979500 | -0.05453328 |
| 6 | 1.91704093 | 1.62651577 | 1.26681054 |
| 1 | 5.46212971 | 2.10514562 | -1.72389070 |
| 6 | 2.06721617 | -0.80053577 | -3.25326473 |
| 6 | -5.37966535 | 3.82147842 | -2.13556436 |
| 6 | -4.62605584 | 1.74914398 | -0.98506733 |
| 6 | -3.53374836 | 2.49311089 | -3.14365563 |
| 6 | 2.98230452 | -0.97094324 | -4.48072558 |
| 6 | 0.71390994 | -0.25301114 | -3.76496703 |
| 6 | 1.87542783 | -2.20289905 | -2.62919405 |
| 1 | 0.68129601 | 3.26251437 | 2.99457195 |
| 1 | -0.99152822 | 2.90876921 | 2.45904932 |
| 1 | 1.14697504 | 4.18736030 | 0.75236101 |
| 1 | 0.97602344 | 3.12072138 | -0.65971176 |
| 1 | -4.25184753 | 5.57346377 | -0.93103575 |
| 1 | -0.38138830 | 5.89439824 | 0.88543065 |
| 1 | 1.81837516 | 0.70490500 | 1.86247816 |
| 1 | 2.53433680 | 2.33226020 | 1.84787363 |
| 1 | 4.45180075 | 0.55861702 | -3.34673134 |
| 1 | 4.26088403 | 2.62191757 | 0.40630012 |
| 1 | -6.08828500 | 3.21076718 | -2.71319333 |

| | | | |
|---|---|---|---|
| 1 | -5.90154467 | 4.16693503 | -1.23127983 |
| 1 | -5.12504046 | 4.69905163 | -2.74709422 |
| 1 | -3.80124110 | 1.06050489 | -0.77301297 |
| 1 | -5.07045862 | 2.07192753 | -0.03176878 |
| 1 | -5.39840644 | 1.20521219 | -1.55084102 |
| 1 | -3.22827116 | 3.35413004 | -3.75579657 |
| 1 | -2.65941220 | 1.85263315 | -2.98303221 |
| 1 | -4.28566612 | 1.92451363 | -3.71237797 |
| 1 | 2.50632627 | -1.66643666 | -5.18675852 |
| 1 | 3.96212393 | -1.39168658 | -4.21229328 |
| 1 | 3.14433836 | -0.02159914 | -5.01114764 |
| 1 | -0.00871973 | -0.13087486 | -2.95160539 |
| 1 | 0.85181889 | 0.72363914 | -4.25086284 |
| 1 | 0.28948459 | -0.94358337 | -4.50981758 |
| 1 | 2.84378557 | -2.62331236 | -2.32006881 |
| 1 | 1.21930130 | -2.16113929 | -1.75305331 |
| 1 | 1.43159418 | -2.88598977 | -3.36964007 |
| 1 | 0.01185964 | 1.59590691 | 3.12464418 |
| 31 | -0.61068711 | 0.73270615 | 0.18704249 |
| 8 | 6.34908009 | -3.12660406 | 3.84664456 |
| 6 | 7.26969949 | -2.24403608 | 4.53221638 |
| 1 | 7.40842592 | -1.31655003 | 3.96250792 |
| 6 | 4.22691373 | -3.58508540 | 2.89263375 |
| 6 | 2.73993395 | -3.23584909 | 2.95461536 |
| 6 | 1.88718144 | -4.14783132 | 2.06415348 |
| 6 | 5.09954143 | -2.61019661 | 3.65967011 |
| 6 | 0.37577180 | -3.88489381 | 2.15618592 |
| 6 | -0.00533208 | -2.47955868 | 1.71002325 |
| 1 | 4.58001037 | -3.57594124 | 1.84683972 |
| 1 | 2.61863436 | -2.18414009 | 2.66063956 |
| 1 | 2.21266872 | -4.03745261 | 1.01512502 |
| 1 | 0.02761658 | -4.04127670 | 3.19001524 |
| 1 | 4.42453169 | -4.60206273 | 3.26396129 |
| 1 | 2.39138613 | -3.30287846 | 3.99814762 |
| 1 | 2.07550003 | -5.20009316 | 2.33383661 |
| 1 | -0.15920045 | -4.61371268 | 1.52751392 |
| 1 | 0.38333911 | -2.27280093 | 0.69799673 |
| 1 | 0.40280079 | -1.71909661 | 2.39016532 |
| 8 | 4.76496355 | -1.50776380 | 4.05199969 |
| 8 | -1.44816961 | -2.38937908 | 1.72105143 |
| 1 | 8.20874339 | -2.80161614 | 4.59987594 |
| 1 | 6.89106517 | -1.99647682 | 5.53181318 |

Electronic energy = -3908.670755 a.u.
DFT-D3(BJ) dispersion correction = -0.140290 a.u.
Thermal free energy = 0.740550 a.u.
Gibbs free energy = -3908.070495 a.u.
Number of imaginary frequencies = 0.

**Int3b CL isomer/conformer 4**

| Atomic N. | X | Y | Z |
|---|---|---|---|
| 6 | -3.90118071 | -0.22408327 | 1.00970092 |
| 6 | -4.76188182 | -1.31410907 | 0.36295625 |

| | | | |
|---|---|---|---|
| 6 | -4.41661108 | -1.66164337 | -1.09446113 |
| 6 | -2.39009117 | -0.46829435 | 1.03939383 |
| 6 | -3.08773180 | -2.42027839 | -1.24162380 |
| 6 | -1.82977750 | -1.54939773 | -1.26542255 |
| 1 | -4.04353998 | 0.73494900 | 0.49159446 |
| 1 | -5.80950948 | -0.97672354 | 0.40942931 |
| 1 | -5.22216105 | -2.29821030 | -1.49234820 |
| 1 | -3.08070455 | -2.98583845 | -2.18768209 |
| 1 | -4.21070458 | -0.07793979 | 2.05539816 |
| 1 | -4.70402829 | -2.23217212 | 0.96878229 |
| 1 | -4.40859409 | -0.74932216 | -1.71360528 |
| 1 | -3.01276893 | -3.16087909 | -0.43277943 |
| 1 | -0.93923236 | -2.15625183 | -1.03956755 |
| 1 | -1.69354864 | -1.10250635 | -2.26077449 |
| 8 | -1.69882912 | 0.56818921 | 1.60829169 |
| 8 | -1.86146677 | -0.39703089 | -0.38471747 |
| 7 | 0.43809895 | 2.58813062 | 1.47345533 |
| 8 | -0.97839850 | 2.32031717 | -1.13281111 |
| 8 | 1.00553619 | 0.13257986 | -0.13564544 |
| 6 | -0.40489813 | 3.04792351 | 2.60451998 |
| 6 | -1.16319171 | 3.65433934 | -0.98779867 |
| 6 | -2.14731046 | 4.32230650 | -1.77625107 |
| 6 | -2.31595998 | 5.69854040 | -1.56687278 |
| 6 | -1.55697835 | 6.42452443 | -0.64485436 |
| 6 | -0.56795401 | 5.77125152 | 0.08232480 |
| 6 | -0.35257886 | 4.39986645 | -0.09252884 |
| 6 | 0.81600240 | 3.74072596 | 0.58804727 |
| 1 | -1.73073655 | 7.49234930 | -0.51225885 |
| 6 | -2.97712727 | 3.57327768 | -2.83736701 |
| 6 | 2.27810428 | 0.58710243 | -0.06412547 |
| 6 | 3.24807042 | 0.15108743 | -1.01746435 |
| 6 | 4.55738232 | 0.63032337 | -0.87124772 |
| 6 | 4.93489783 | 1.50859847 | 0.14848505 |
| 6 | 3.98018751 | 1.93306378 | 1.06534806 |
| 6 | 2.66067415 | 1.47482426 | 0.97454338 |
| 6 | 1.66305295 | 1.90039200 | 2.01886109 |
| 1 | 5.96469097 | 1.85904328 | 0.21625030 |
| 6 | 2.87847963 | -0.80446234 | -2.16935260 |
| 6 | -3.93903551 | 4.51339882 | -3.58804464 |
| 6 | -3.83468134 | 2.46981242 | -2.17691149 |
| 6 | -2.03376451 | 2.94385687 | -3.89071747 |
| 6 | 4.08797959 | -1.12402092 | -3.06830332 |
| 6 | 1.79827327 | -0.15939698 | -3.06958868 |
| 6 | 2.36588110 | -2.14738193 | -1.59893552 |
| 1 | 0.15934205 | 3.76643603 | 3.22240496 |
| 1 | -1.30493449 | 3.53605826 | 2.21640770 |
| 1 | 1.36800676 | 4.48160041 | 1.19207004 |
| 1 | 1.51414022 | 3.33496208 | -0.15878192 |
| 1 | -3.06981747 | 6.23119822 | -2.14373807 |
| 1 | 0.06318897 | 6.32472750 | 0.78152199 |
| 1 | 1.28646333 | 1.03292109 | 2.58491573 |
| 1 | 2.15162988 | 2.58239898 | 2.73562920 |
| 1 | 5.31678913 | 0.31582978 | -1.58477152 |

| | | | |
|---|---|---|---|
| 1 | 4.25147428 | 2.62168620 | 1.86873793 |
| 1 | -4.49076748 | 3.93170393 | -4.34036485 |
| 1 | -4.67955809 | 4.97222275 | -2.91683731 |
| 1 | -3.40466443 | 5.31546537 | -4.11723800 |
| 1 | -3.20754427 | 1.72342417 | -1.67752905 |
| 1 | -4.52482011 | 2.90370430 | -1.43796497 |
| 1 | -4.43844913 | 1.96283497 | -2.94547300 |
| 1 | -1.45330649 | 3.72412293 | -4.40416727 |
| 1 | -1.33517825 | 2.23561097 | -3.43167958 |
| 1 | -2.62656373 | 2.40995387 | -4.64936929 |
| 1 | 3.76754125 | -1.80798964 | -3.86709518 |
| 1 | 4.89735965 | -1.62055114 | -2.51371194 |
| 1 | 4.49638504 | -0.22338478 | -3.54893362 |
| 1 | 0.88576733 | 0.07001578 | -2.50972167 |
| 1 | 2.17259197 | 0.77427231 | -3.51347090 |
| 1 | 1.54080726 | -0.84456918 | -3.89170220 |
| 1 | 3.15178623 | -2.64163311 | -1.00905855 |
| 1 | 1.49151210 | -1.99933403 | -0.95629772 |
| 1 | 2.08717054 | -2.82306653 | -2.42193915 |
| 1 | -0.70332173 | 2.18443377 | 3.20741135 |
| 31 | -0.51044435 | 1.16675583 | 0.26107011 |
| 8 | 4.12504473 | -6.04204432 | 4.00854737 |
| 6 | 4.27614829 | -7.45083471 | 4.30681162 |
| 1 | 3.35305461 | -7.85147069 | 4.74415327 |
| 6 | 3.09362754 | -4.26583804 | 2.82018815 |
| 6 | 1.78211863 | -3.80242690 | 2.18071850 |
| 6 | 0.59281801 | -3.82895986 | 3.15027064 |
| 6 | 3.13203568 | -5.75389605 | 3.11685350 |
| 6 | -0.74937806 | -3.44004692 | 2.51244661 |
| 6 | -0.80412911 | -1.98573608 | 2.06245030 |
| 1 | 3.31122357 | -3.71558659 | 3.74795951 |
| 1 | 1.56117889 | -4.44500316 | 1.31447019 |
| 1 | 0.49494578 | -4.84329428 | 3.56592162 |
| 1 | -1.56073244 | -3.60478091 | 3.23833618 |
| 1 | 3.94182387 | -4.06201776 | 2.14524400 |
| 1 | 1.93423948 | -2.78453437 | 1.79223036 |
| 1 | 0.80086619 | -3.15883675 | 4.00322564 |
| 1 | -0.95546302 | -4.09789488 | 1.65266689 |
| 1 | -0.08764887 | -1.78210173 | 1.25127959 |
| 1 | -0.56725764 | -1.31078065 | 2.90108491 |
| 8 | 2.40976870 | -6.60029400 | 2.62400832 |
| 8 | -2.15335565 | -1.72461588 | 1.60969785 |
| 1 | 5.10151244 | -7.50821558 | 5.02294036 |
| 1 | 4.51263796 | -8.01343217 | 3.39485059 |

Electronic energy = -3908.670402 a.u.
DFT-D3(BJ) dispersion correction = -0.141114 a.u.
Thermal free energy = 0.741597 a.u.
Gibbs free energy = -3908.069919 a.u.
Number of imaginary frequencies = 0.

**Int3b CL isomer/conformer 5**

Atomic N. X Y Z

| | | | |
|---|---|---|---|
| 7 | 0.73666562 | 2.57302457 | 1.90967025 |
| 8 | -0.29522737 | 1.56744242 | -0.68647922 |
| 8 | 2.76440471 | 1.60183369 | -0.07837231 |
| 6 | -0.05018503 | 2.11563712 | 3.08093508 |
| 6 | -1.37535377 | 2.31977894 | -0.37195362 |
| 6 | -2.60175096 | 2.11752108 | -1.07460285 |
| 6 | -3.70329014 | 2.89379315 | -0.68726659 |
| 6 | -3.63616231 | 3.85180462 | 0.32812428 |
| 6 | -2.42206940 | 4.07734351 | 0.96746670 |
| 6 | -1.28868129 | 3.33601134 | 0.61551614 |
| 6 | 0.04538949 | 3.70374327 | 1.20727312 |
| 1 | -4.52218463 | 4.42558600 | 0.59948213 |
| 6 | -2.71005055 | 1.11078766 | -2.23776101 |
| 6 | 3.29300190 | 2.83102282 | 0.11681851 |
| 6 | 4.18650241 | 3.38328145 | -0.85149705 |
| 6 | 4.71787143 | 4.65406691 | -0.59240091 |
| 6 | 4.40700900 | 5.38644232 | 0.55666315 |
| 6 | 3.53929547 | 4.83846544 | 1.49385574 |
| 6 | 2.98871575 | 3.56718374 | 1.29197110 |
| 6 | 2.11621156 | 2.97380753 | 2.36675969 |
| 1 | 4.84074081 | 6.37423763 | 0.71147557 |
| 6 | 4.56108712 | 2.61599168 | -2.13465763 |
| 6 | -4.11674194 | 1.10245401 | -2.86521736 |
| 6 | -2.41717800 | -0.32473453 | -1.74532626 |
| 6 | -1.71025428 | 1.49489275 | -3.35504720 |
| 6 | 5.53941156 | 3.40927849 | -3.02167483 |
| 6 | 3.29562401 | 2.34483757 | -2.98232522 |
| 6 | 5.25871202 | 1.28560112 | -1.76494562 |
| 1 | -0.14413999 | 2.93596870 | 3.81205622 |
| 1 | -1.05087292 | 1.81233556 | 2.75425416 |
| 1 | -0.07555913 | 4.53965409 | 1.91794442 |
| 1 | 0.73501646 | 4.03880225 | 0.41826232 |
| 1 | -4.65538956 | 2.75027176 | -1.19507995 |
| 1 | -2.33545755 | 4.84927231 | 1.73569859 |
| 1 | 2.56461390 | 2.05651301 | 2.78135730 |
| 1 | 2.00857881 | 3.69942607 | 3.19097209 |
| 1 | 5.39846806 | 5.09869999 | -1.31614549 |
| 1 | 3.28578997 | 5.39004526 | 2.40205485 |
| 1 | -4.13236216 | 0.38298832 | -3.69637395 |
| 1 | -4.89005147 | 0.79429743 | -2.14653279 |
| 1 | -4.39336258 | 2.08505114 | -3.27347494 |
| 1 | -1.40121454 | -0.40752479 | -1.34417115 |
| 1 | -3.13114177 | -0.61829330 | -0.96117823 |
| 1 | -2.52210722 | -1.03480766 | -2.58017911 |
| 1 | -1.93878409 | 2.49529542 | -3.75020435 |
| 1 | -0.67802854 | 1.49428080 | -2.98750353 |
| 1 | -1.78712341 | 0.77771723 | -4.18676749 |
| 1 | 5.77364478 | 2.81351209 | -3.91555038 |
| 1 | 6.48806097 | 3.61967376 | -2.50709589 |
| 1 | 5.10926654 | 4.36174052 | -3.36334832 |
| 1 | 2.55138516 | 1.76418020 | -2.42771435 |
| 1 | 2.83007823 | 3.29154596 | -3.29241087 |
| 1 | 3.56737797 | 1.78869620 | -3.89263013 |

```
   1     6.19975138   1.48026939  -1.23007873
   1     4.62192625   0.66437433  -1.12567502
   1     5.50016383   0.72116754  -2.67877311
   1     0.45313570   1.25825523   3.53928466
   6     2.92866286  -1.85510293   1.26909191
   6     3.33548701  -3.22554378   0.71449602
   6     3.45058153  -3.30289730  -0.81214560
   6     1.46886587  -1.43166851   1.05529774
   6     2.19695471  -2.86937209  -1.58511276
   6     1.91820635  -1.36970318  -1.53973436
   1     3.56970142  -1.05083432   0.87573836
   1     4.31062896  -3.49385799   1.15080668
   1     3.70519892  -4.33707156  -1.09368331
   1     2.33872009  -3.11990706  -2.64926112
   1     3.05770587  -1.85254794   2.36141039
   1     2.61649793  -3.97686784   1.07149797
   1     4.29619805  -2.67690549  -1.14682505
   1     1.31006871  -3.42671247  -1.24696043
   1     1.25409081  -1.07893875  -2.36607230
   1     2.85893617  -0.80494096  -1.64332672
   8     1.10865548  -0.32822723   1.78441040
   8     1.21894686  -0.89330270  -0.35827061
  31     1.06163294   1.06598234   0.50131398
   8    -7.25986130  -5.83413022   2.22710238
   6    -8.04018158  -7.04865951   2.33551945
   1    -7.92769958  -7.65952485   1.43092912
   6    -5.19059969  -4.70702898   1.96576144
   6    -3.67908945  -4.85663161   1.79782361
   6    -2.96569847  -3.50507290   1.68785446
   6    -5.91754217  -6.03380265   2.07128543
   6    -1.44920905  -3.64891109   1.51774077
   6    -0.75387468  -2.29974911   1.40614868
   1    -5.63065686  -4.14979561   1.12251243
   1    -3.47166973  -5.46310537   0.90289704
   1    -3.37957295  -2.94007853   0.83469224
   1    -1.23007852  -4.24418820   0.61671355
   1    -5.43499022  -4.11761673   2.86462589
   1    -3.27321529  -5.42600812   2.64805205
   1    -3.17926911  -2.90291060   2.58809879
   1    -1.02718127  -4.20082606   2.37302620
   1    -0.93590117  -1.68493393   2.30088162
   1    -1.11635677  -1.74486279   0.52644193
   8    -5.40531492  -7.13609845   2.02666591
   8     0.66369458  -2.54903552   1.27684788
   1    -9.07641054  -6.71740908   2.45334973
   1    -7.71823813  -7.63574545   3.20487763
Electronic energy = -3908.670757 a.u.
DFT-D3(BJ) dispersion correction = -0.137894 a.u.
Thermal free energy = 0.738794 a.u.
Gibbs free energy = -3908.069857 a.u.
Number of imaginary frequencies = 0.
```

**Int3b CL isomer/conformer 6**

| Atomic N. | X | Y | Z |
|---|---|---|---|
| 6 | -3.38188516 | -0.47526230 | 2.12568726 |
| 6 | -4.44026371 | -1.43601137 | 1.57415327 |
| 6 | -4.55809289 | -1.49312048 | 0.04207757 |
| 6 | -1.94237387 | -0.69238006 | 1.65206277 |
| 6 | -3.36772461 | -2.19178199 | -0.63481011 |
| 6 | -2.13408766 | -1.31608688 | -0.86783723 |
| 1 | -3.63359547 | 0.56265453 | 1.86540164 |
| 1 | -5.41187297 | -1.13114894 | 1.99430495 |
| 1 | -5.47303547 | -2.05230326 | -0.20826390 |
| 1 | -3.66648541 | -2.56487206 | -1.62803147 |
| 1 | -3.35862700 | -0.53864623 | 3.22391772 |
| 1 | -4.24360809 | -2.45186881 | 1.95193165 |
| 1 | -4.69263036 | -0.47902251 | -0.36905220 |
| 1 | -3.09131109 | -3.07278576 | -0.03819341 |
| 1 | -1.24184964 | -1.94242206 | -1.02212937 |
| 1 | -2.27138342 | -0.69316063 | -1.76336325 |
| 8 | -1.07099797 | 0.22831808 | 2.16837422 |
| 8 | -1.86463930 | -0.34434418 | 0.17421740 |
| 7 | 1.01553429 | 2.27299034 | 1.76059546 |
| 8 | -1.11916979 | 2.46452872 | -0.30433507 |
| 8 | 0.97288954 | 0.14370209 | -0.33995198 |
| 6 | 0.56005428 | 2.52125165 | 3.15076222 |
| 6 | -1.18737516 | 3.75027972 | 0.11709788 |
| 6 | -2.33187790 | 4.54018141 | -0.20378829 |
| 6 | -2.36053353 | 5.85814826 | 0.27413809 |
| 6 | -1.32225953 | 6.41573865 | 1.02527379 |
| 6 | -0.19394011 | 5.64916708 | 1.29520043 |
| 6 | -0.10944306 | 4.33054380 | 0.83503163 |
| 6 | 1.17363930 | 3.56533971 | 1.01190235 |
| 1 | -1.39423042 | 7.44343476 | 1.38119261 |
| 6 | -3.48540840 | 3.98165846 | -1.06044604 |
| 6 | 2.23550841 | 0.57677573 | -0.56502202 |
| 6 | 2.87153185 | 0.30325876 | -1.81359553 |
| 6 | 4.18730208 | 0.75717286 | -1.98106110 |
| 6 | 4.88051581 | 1.45670776 | -0.98946936 |
| 6 | 4.25127313 | 1.71626951 | 0.22240220 |
| 6 | 2.94217688 | 1.27515156 | 0.44766599 |
| 6 | 2.31284249 | 1.50655681 | 1.79570955 |
| 1 | 5.90126363 | 1.79387598 | -1.16872760 |
| 6 | 2.14841663 | -0.46226313 | -2.93932935 |
| 6 | -4.58886229 | 5.02972094 | -1.29766228 |
| 6 | -4.14109557 | 2.77102646 | -0.35765719 |
| 6 | -2.94851891 | 3.55530763 | -2.44792919 |
| 6 | 3.03641414 | -0.63466617 | -4.18625783 |
| 6 | 0.88277098 | 0.30870420 | -3.38283488 |
| 6 | 1.76762880 | -1.87981183 | -2.45087543 |
| 1 | 1.31274724 | 3.12170027 | 3.68849086 |
| 1 | -0.38966472 | 3.06602210 | 3.13396732 |
| 1 | 1.91535960 | 4.18885437 | 1.54042178 |
| 1 | 1.60023864 | 3.29989092 | 0.03340149 |
| 1 | -3.22710755 | 6.47956142 | 0.05583797 |

| | | | |
|---|---|---|---|
| 1 | 0.64493859 | 6.07394795 | 1.85140121 |
| 1 | 2.07791431 | 0.55143251 | 2.29275810 |
| 1 | 3.02021131 | 2.05508827 | 2.44071358 |
| 1 | 4.69565922 | 0.56575711 | -2.92425639 |
| 1 | 4.77518601 | 2.25732405 | 1.01352686 |
| 1 | -5.37542440 | 4.58420775 | -1.92341655 |
| 1 | -5.05908641 | 5.35715001 | -0.35898757 |
| 1 | -4.21014046 | 5.91696865 | -1.82516766 |
| 1 | -3.42364780 | 1.95454023 | -0.22316262 |
| 1 | -4.53563152 | 3.06063777 | 0.62770658 |
| 1 | -4.98289166 | 2.40021591 | -0.96290248 |
| 1 | -2.52214054 | 4.41890386 | -2.97878556 |
| 1 | -2.17451483 | 2.78497922 | -2.35876476 |
| 1 | -3.77226744 | 3.15615186 | -3.05966281 |
| 1 | 2.47353192 | -1.18839574 | -4.95133684 |
| 1 | 3.94997492 | -1.20673649 | -3.96866690 |
| 1 | 3.32663909 | 0.33096089 | -4.62455697 |
| 1 | 0.18103306 | 0.44761237 | -2.55404887 |
| 1 | 1.15237706 | 1.30022068 | -3.77410866 |
| 1 | 0.37145958 | -0.24359290 | -4.18615955 |
| 1 | 2.67018359 | -2.45339674 | -2.19267546 |
| 1 | 1.12096772 | -1.83676752 | -1.56785068 |
| 1 | 1.23838351 | -2.42250315 | -3.24916396 |
| 1 | 0.40977338 | 1.56298444 | 3.65804780 |
| 31 | -0.30786890 | 1.08398354 | 0.65940321 |
| 8 | 3.50970238 | -6.02947738 | -0.19079203 |
| 6 | 4.36649995 | -5.58389348 | -1.26943800 |
| 1 | 4.53262242 | -4.50133980 | -1.20335879 |
| 6 | 3.04459791 | -6.28108954 | 2.13084596 |
| 6 | 1.58790721 | -5.81807588 | 1.96334947 |
| 6 | 1.42325980 | -4.29503563 | 2.00327918 |
| 6 | 3.99686167 | -5.77910230 | 1.06061055 |
| 6 | -0.04251725 | -3.85698970 | 1.90846947 |
| 6 | -0.18313790 | -2.34172075 | 1.87664122 |
| 1 | 3.08297127 | -7.38242146 | 2.11369307 |
| 1 | 0.98887888 | -6.27258183 | 2.76917185 |
| 1 | 1.86617846 | -3.90298138 | 2.93475274 |
| 1 | -0.60946945 | -4.25166636 | 2.76711993 |
| 1 | 3.45634928 | -5.95124329 | 3.09371438 |
| 1 | 1.18831162 | -6.20823330 | 1.01530186 |
| 1 | 1.99511606 | -3.84320085 | 1.17518367 |
| 1 | -0.50158596 | -4.28261379 | 1.00130619 |
| 1 | 0.30549970 | -1.92256060 | 0.98186353 |
| 1 | 0.28601764 | -1.88499976 | 2.76273479 |
| 8 | 5.06656662 | -5.23708673 | 1.26500368 |
| 8 | -1.59586924 | -2.03151906 | 1.87572053 |
| 1 | 3.83115328 | -5.83915481 | -2.18905155 |
| 1 | 5.33530965 | -6.09722272 | -1.22660079 |

Electronic energy = -3908.670148 a.u.
DFT-D3(BJ) dispersion correction = -0.140507 a.u.
Thermal free energy = 0.741258 a.u.
Gibbs free energy = -3908.069397 a.u.
Number of imaginary frequencies = 0.

**Int3b CL isomer/conformer 7**

| Atomic N. | X | Y | Z |
|---|---|---|---|
| 6 | -3.63908664 | 0.21429245 | 0.99850279 |
| 6 | -4.52721824 | -0.84308449 | 0.33500963 |
| 6 | -4.17030626 | -1.19914470 | -1.11755515 |
| 6 | -2.13831429 | -0.08280174 | 1.05267737 |
| 6 | -2.86876405 | -2.00729592 | -1.24426071 |
| 6 | -1.57809566 | -1.18487820 | -1.24220263 |
| 1 | -3.73897541 | 1.17850682 | 0.48003908 |
| 1 | -5.56277779 | -0.46871844 | 0.36518397 |
| 1 | -4.99231790 | -1.80332407 | -1.53190341 |
| 1 | -2.86679622 | -2.57032314 | -2.19185667 |
| 1 | -3.96052071 | 0.36981374 | 2.03923305 |
| 1 | -4.51220782 | -1.76398697 | 0.93917663 |
| 1 | -4.11651528 | -0.28581599 | -1.73285768 |
| 1 | -2.83722236 | -2.75202726 | -0.43619734 |
| 1 | -0.71574735 | -1.82436801 | -0.99767430 |
| 1 | -1.40448555 | -0.74492105 | -2.23481580 |
| 8 | -1.42021584 | 0.92795137 | 1.63230824 |
| 8 | -1.58478459 | -0.03123272 | -0.36365220 |
| 7 | 0.79387911 | 2.86938894 | 1.51962986 |
| 8 | -0.55893967 | 2.61652080 | -1.11704488 |
| 8 | 1.29883268 | 0.35643192 | -0.02025192 |
| 6 | -0.07400371 | 3.41781992 | 2.59078623 |
| 6 | -0.65246216 | 3.96386163 | -1.01394956 |
| 6 | -1.57835501 | 4.67197215 | -1.83769288 |
| 6 | -1.64680412 | 6.06383293 | -1.68252843 |
| 6 | -0.84831596 | 6.76641588 | -0.77597644 |
| 6 | 0.07510330 | 6.06928172 | -0.00479216 |
| 6 | 0.19180562 | 4.68023269 | -0.12602743 |
| 6 | 1.28660802 | 3.96344943 | 0.61638413 |
| 1 | -0.94331864 | 7.84850927 | -0.68624628 |
| 6 | -2.45992080 | 3.94511616 | -2.87257293 |
| 6 | 2.59227239 | 0.72205814 | 0.13261230 |
| 6 | 3.58978330 | 0.19035100 | -0.74004343 |
| 6 | 4.91716267 | 0.58376409 | -0.52015480 |
| 6 | 5.28824340 | 1.46527304 | 0.49896466 |
| 6 | 4.30842350 | 1.97861143 | 1.34057783 |
| 6 | 2.96858724 | 1.60870968 | 1.17459794 |
| 6 | 1.94316657 | 2.12519044 | 2.14885633 |
| 1 | 6.33337669 | 1.74689556 | 0.62633748 |
| 6 | 3.23032932 | -0.77668426 | -1.88550860 |
| 6 | -3.35542289 | 4.92256017 | -3.65683515 |
| 6 | -3.38992711 | 2.93066956 | -2.16809613 |
| 6 | -1.56753784 | 3.21062582 | -3.90173812 |
| 6 | 4.47142372 | -1.20870573 | -2.68939163 |
| 6 | 2.26230191 | -0.08926928 | -2.87742386 |
| 6 | 2.58790387 | -2.06140368 | -1.31174685 |
| 1 | 0.50176634 | 4.12249124 | 3.21383043 |
| 1 | -0.92211035 | 3.94551944 | 2.14212104 |
| 1 | 1.86680562 | 4.68294781 | 1.21959533 |

| | | | |
|---|---|---|---|
| 1 | 1.98253551 | 3.48570089 | -0.08901505 |
| 1 | -2.35239499 | 6.62814905 | -2.28949292 |
| 1 | 0.73088771 | 6.60011451 | 0.68922687 |
| 1 | 1.48341159 | 1.30002148 | 2.71676520 |
| 1 | 2.43401605 | 2.79728461 | 2.87328432 |
| 1 | 5.69743301 | 0.19563010 | -1.17217708 |
| 1 | 4.57513816 | 2.66805869 | 2.14479291 |
| 1 | -3.95010117 | 4.35442255 | -4.38642501 |
| 1 | -4.05878101 | 5.45746224 | -3.00217309 |
| 1 | -2.76749280 | 5.66450263 | -4.21608481 |
| 1 | -2.81290988 | 2.15918207 | -1.64718750 |
| 1 | -4.03860064 | 3.43903633 | -1.43925114 |
| 1 | -4.03699428 | 2.44010907 | -2.91176985 |
| 1 | -0.93451585 | 3.92633291 | -4.44602883 |
| 1 | -0.91856600 | 2.47373519 | -3.41543192 |
| 1 | -2.20070607 | 2.69137306 | -4.63758860 |
| 1 | 4.15786689 | -1.89792338 | -3.48645385 |
| 1 | 5.20658178 | -1.73743691 | -2.06566294 |
| 1 | 4.97112472 | -0.35490357 | -3.16917006 |
| 1 | 1.32635766 | 0.20912459 | -2.39386467 |
| 1 | 2.72719986 | 0.80985346 | -3.30665639 |
| 1 | 2.02572390 | -0.77510742 | -3.70538504 |
| 1 | 3.29721302 | -2.58781958 | -0.65624442 |
| 1 | 1.68632866 | -1.83075307 | -0.73397314 |
| 1 | 2.31570427 | -2.74313583 | -2.13209473 |
| 1 | -0.45302655 | 2.59455132 | 3.20465702 |
| 31 | -0.17710295 | 1.46945391 | 0.30775977 |
| 8 | 0.62563464 | -8.33259982 | 1.94987262 |
| 6 | 0.79571289 | -9.72158009 | 2.32081605 |
| 1 | 1.82808653 | -9.90998819 | 2.64150125 |
| 6 | 0.68840155 | -6.01946019 | 2.46288539 |
| 6 | 0.84364729 | -4.97641861 | 3.57079993 |
| 6 | 0.77158027 | -3.52807407 | 3.06631938 |
| 6 | 0.87652118 | -7.44189673 | 2.95487081 |
| 6 | -0.59744741 | -3.11856758 | 2.50734464 |
| 6 | -0.62507238 | -1.64982852 | 2.10880051 |
| 1 | 1.42484747 | -5.84842927 | 1.65929110 |
| 1 | 1.80719984 | -5.14016748 | 4.07455233 |
| 1 | 1.03024230 | -2.85777345 | 3.90259841 |
| 1 | -1.37930786 | -3.30065648 | 3.26284834 |
| 1 | -0.29688781 | -5.96258065 | 1.97599167 |
| 1 | 0.07110284 | -5.14250178 | 4.33798183 |
| 1 | 1.54652102 | -3.36950554 | 2.29645882 |
| 1 | -0.85593193 | -3.72819156 | 1.62734201 |
| 1 | 0.12598034 | -1.44094514 | 1.32938502 |
| 1 | -0.40170475 | -1.00523264 | 2.97379888 |
| 8 | 1.20849371 | -7.77472976 | 4.07664074 |
| 8 | -1.95394364 | -1.34880147 | 1.62330087 |
| 1 | 0.55970185 | -10.29330797 | 1.41815424 |
| 1 | 0.11398640 | -9.98686152 | 3.13866315 |

Electronic energy = -3908.670301 a.u.
DFT-D3(BJ) dispersion correction = -0.139482 a.u.
Thermal free energy = 0.740429 a.u.

Gibbs free energy = -3908.069354 a.u.
Number of imaginary frequencies = 0.

**Int3b CL isomer/conformer 8**

| Atomic N. | X | Y | Z |
|---|---|---|---|
| 6 | -3.43994468 | 0.10478903 | 1.10817980 |
| 6 | -4.32797650 | -0.97149908 | 0.47550224 |
| 6 | -3.96489843 | -1.37811144 | -0.96229199 |
| 6 | -1.94052581 | -0.19565543 | 1.17845289 |
| 6 | -2.66529953 | -2.19412477 | -1.05524270 |
| 6 | -1.37207852 | -1.37544426 | -1.07470232 |
| 1 | -3.53466001 | 1.05246626 | 0.55917635 |
| 1 | -5.36246360 | -0.59315352 | 0.48731270 |
| 1 | -4.78700274 | -1.99353159 | -1.35974480 |
| 1 | -2.66060037 | -2.78899049 | -1.98321741 |
| 1 | -3.76543679 | 0.29383578 | 2.14206594 |
| 1 | -4.31904080 | -1.87111962 | 1.11098021 |
| 1 | -3.90528366 | -0.48678278 | -1.60847261 |
| 1 | -2.63925585 | -2.90994565 | -0.22114105 |
| 1 | -0.51347414 | -2.00806635 | -0.80098375 |
| 1 | -1.19067339 | -0.97229807 | -2.08146383 |
| 8 | -1.22020685 | 0.83098046 | 1.72765576 |
| 8 | -1.38243997 | -0.19093981 | -0.23851480 |
| 7 | 0.98882952 | 2.77265486 | 1.55674852 |
| 8 | -0.35949263 | 2.43518231 | -1.07295363 |
| 8 | 1.50024491 | 0.21465675 | 0.09346089 |
| 6 | 0.12326233 | 3.34059528 | 2.61964711 |
| 6 | -0.46931884 | 3.78352233 | -1.00720145 |
| 6 | -1.39707685 | 4.45900416 | -1.85578087 |
| 6 | -1.48519755 | 5.85318525 | -1.73486009 |
| 6 | -0.70202216 | 6.58834151 | -0.84081534 |
| 6 | 0.22734031 | 5.92298619 | -0.04886349 |
| 6 | 0.36233973 | 4.53299920 | -0.13493142 |
| 6 | 1.46624153 | 3.84851735 | 0.62420910 |
| 1 | -0.81228325 | 7.67088983 | -0.77795846 |
| 6 | -2.25578329 | 3.69605379 | -2.88382178 |
| 6 | 2.79186857 | 0.59618223 | 0.21991043 |
| 6 | 3.78538981 | 0.04419320 | -0.64469789 |
| 6 | 5.11078098 | 0.45847809 | -0.45315559 |
| 6 | 5.48368281 | 1.37762487 | 0.53154296 |
| 6 | 4.50795699 | 1.90828183 | 1.36727054 |
| 6 | 3.17031008 | 1.51985902 | 1.22835682 |
| 6 | 2.14925802 | 2.05553736 | 2.19678372 |
| 1 | 6.52710753 | 1.67416844 | 0.63738645 |
| 6 | 3.42376329 | -0.96908851 | -1.74869090 |
| 6 | -3.15369810 | 4.64256265 | -3.70254947 |
| 6 | -3.18123944 | 2.68463344 | -2.16938086 |
| 6 | -1.33804949 | 2.95189270 | -3.88339968 |
| 6 | 4.66019545 | -1.41924098 | -2.54974555 |
| 6 | 2.43481964 | -0.33308197 | -2.75402836 |
| 6 | 2.80276005 | -2.23721874 | -1.11684807 |
| 1 | 0.69751392 | 4.06464017 | 3.22157966 |

| | | | |
|---|---|---|---|
| 1 | -0.73166470 | 3.85057120 | 2.16349664 |
| 1 | 2.03959660 | 4.59012250 | 1.20697532 |
| 1 | 2.16549685 | 3.36081147 | -0.07095210 |
| 1 | -2.19368181 | 6.39311036 | -2.36038699 |
| 1 | 0.87357904 | 6.47955677 | 0.63386520 |
| 1 | 1.70101445 | 1.24259441 | 2.79086585 |
| 1 | 2.64058844 | 2.75145951 | 2.89808279 |
| 1 | 5.88800634 | 0.05618905 | -1.10029329 |
| 1 | 4.77633273 | 2.62625231 | 2.14557744 |
| 1 | -3.73070038 | 4.04950227 | -4.42648353 |
| 1 | -3.87295258 | 5.18287016 | -3.06996886 |
| 1 | -2.56824849 | 5.37929145 | -4.27121818 |
| 1 | -2.60113403 | 1.93374841 | -1.62242797 |
| 1 | -3.84767783 | 3.20012545 | -1.46188107 |
| 1 | -3.81019247 | 2.16821808 | -2.91108017 |
| 1 | -0.70759110 | 3.66552211 | -4.43350399 |
| 1 | -0.68660426 | 2.23470377 | -3.37183080 |
| 1 | -1.95124717 | 2.40732470 | -4.61793074 |
| 1 | 4.34477129 | -2.14069155 | -3.31702474 |
| 1 | 5.40690910 | -1.91755402 | -1.91477400 |
| 1 | 5.14681912 | -0.57957127 | -3.06649834 |
| 1 | 1.50878487 | -0.01254422 | -2.26575279 |
| 1 | 2.88818676 | 0.54318371 | -3.23942027 |
| 1 | 2.18233760 | -1.06118365 | -3.54007316 |
| 1 | 3.52474313 | -2.72806394 | -0.44771989 |
| 1 | 1.90398908 | -1.99457143 | -0.53967066 |
| 1 | 2.53121927 | -2.95516917 | -1.90589561 |
| 1 | -0.24594808 | 2.52993520 | 3.25585083 |
| 31 | 0.02105484 | 1.33252262 | 0.38717401 |
| 8 | -0.87412754 | -6.73678843 | 1.11919627 |
| 6 | -2.24386256 | -7.08932194 | 0.80736554 |
| 1 | -2.55754504 | -7.96508586 | 1.38914874 |
| 6 | 0.81333331 | -6.13908364 | 2.68761713 |
| 6 | 1.04992118 | -5.05812732 | 3.75455172 |
| 6 | 0.96250212 | -3.61576695 | 3.23218101 |
| 6 | -0.65288361 | -6.43606601 | 2.43331928 |
| 6 | -0.41133409 | -3.20636953 | 2.68681949 |
| 6 | -0.43585948 | -1.73953394 | 2.28058417 |
| 1 | 1.24897772 | -7.09279637 | 3.03012077 |
| 1 | 2.05181380 | -5.21005242 | 4.18351555 |
| 1 | 1.23887647 | -2.93664979 | 4.05621656 |
| 1 | -1.18969652 | -3.38878476 | 3.44381766 |
| 1 | 1.31012576 | -5.89101080 | 1.73899673 |
| 1 | 0.32806351 | -5.20987166 | 4.57165274 |
| 1 | 1.72507221 | -3.47038194 | 2.44739517 |
| 1 | -0.67361474 | -3.81561453 | 1.80731577 |
| 1 | 0.31762799 | -1.53419566 | 1.50253989 |
| 1 | -0.21660988 | -1.08973818 | 3.14295655 |
| 8 | -1.52805252 | -6.44780752 | 3.27841504 |
| 8 | -1.76352494 | -1.44232160 | 1.78831129 |
| 1 | -2.24854221 | -7.31239500 | -0.26389495 |
| 1 | -2.91468978 | -6.25092372 | 1.03399307 |

Electronic energy = -3908.669192 a.u.

DFT-D3(BJ) dispersion correction = -0.141116 a.u.
Thermal free energy = 0.741444 a.u.
Gibbs free energy = -3908.068865 a.u.
Number of imaginary frequencies = 0.

**Int3b CL isomer/conformer 9**

| Atomic N. | X | Y | Z |
|---|---|---|---|
| 6 | -3.46065392 | 0.28154070 | 1.04327600 |
| 6 | -4.35811767 | -0.84302720 | 0.51674203 |
| 6 | -3.98421669 | -1.40535890 | -0.86471420 |
| 6 | -1.96778404 | -0.03334772 | 1.16712852 |
| 6 | -2.69657995 | -2.24525490 | -0.85368062 |
| 6 | -1.39006468 | -1.45234515 | -0.94089377 |
| 1 | -3.53189450 | 1.16561243 | 0.39389227 |
| 1 | -5.38655495 | -0.45068813 | 0.47414122 |
| 1 | -4.81102860 | -2.04772761 | -1.20582134 |
| 1 | -2.69014702 | -2.93373866 | -1.71451076 |
| 1 | -3.79615413 | 0.58584730 | 2.04599468 |
| 1 | -4.37092261 | -1.67003898 | 1.24413765 |
| 1 | -3.90304982 | -0.58865411 | -1.60091882 |
| 1 | -2.69198304 | -2.87079902 | 0.05039569 |
| 1 | -0.54530310 | -2.06506509 | -0.58973531 |
| 1 | -1.18957001 | -1.16085971 | -1.98199202 |
| 8 | -1.23703719 | 1.03375187 | 1.61645929 |
| 8 | -1.39175196 | -0.18581418 | -0.23491746 |
| 7 | 1.02633776 | 2.89142602 | 1.29346775 |
| 8 | -0.28665157 | 2.32322614 | -1.31433619 |
| 8 | 1.49307495 | 0.18625464 | 0.09415106 |
| 6 | 0.16863814 | 3.57216517 | 2.29510259 |
| 6 | -0.38891766 | 3.67289066 | -1.37302007 |
| 6 | -1.30120803 | 4.27514855 | -2.29044351 |
| 6 | -1.37867436 | 5.67524517 | -2.29749479 |
| 6 | -0.59923037 | 6.48262922 | -1.46459442 |
| 6 | 0.31538945 | 5.88516475 | -0.60420081 |
| 6 | 0.43909134 | 4.49218245 | -0.56209731 |
| 6 | 1.52693558 | 3.86923972 | 0.26945408 |
| 1 | -0.70083097 | 7.56722036 | -1.50177442 |
| 6 | -2.15594381 | 3.42831802 | -3.25400366 |
| 6 | 2.79125077 | 0.55697603 | 0.18349827 |
| 6 | 3.77619642 | -0.09709571 | -0.61726444 |
| 6 | 5.10769007 | 0.31511657 | -0.46804404 |
| 6 | 5.49466557 | 1.32521385 | 0.41702664 |
| 6 | 4.52750618 | 1.95250129 | 1.19369509 |
| 6 | 3.18408572 | 1.57196197 | 1.09387629 |
| 6 | 2.17167749 | 2.21684968 | 2.00285633 |
| 1 | 6.54238204 | 1.61581580 | 0.49261187 |
| 6 | 3.40018428 | -1.21615081 | -1.60855096 |
| 6 | -3.03618697 | 4.30296460 | -4.16642427 |
| 6 | -3.09806584 | 2.49553656 | -2.45912872 |
| 6 | -1.23508629 | 2.58702945 | -4.17013040 |
| 6 | 4.63034450 | -1.76817917 | -2.35346547 |
| 6 | 2.42225667 | -0.67667799 | -2.67897520 |

| | | | |
|---|---|---|---|
| 6 | 2.75873192 | -2.39990881 | -0.84668102 |
| 1 | 0.75703501 | 4.33430235 | 2.83301210 |
| 1 | -0.67136014 | 4.05870965 | 1.78840174 |
| 1 | 2.10428552 | 4.65509349 | 0.78664131 |
| 1 | 2.22562250 | 3.30943632 | -0.36920764 |
| 1 | -2.07552856 | 6.16145556 | -2.97776628 |
| 1 | 0.95871510 | 6.49670133 | 0.03272289 |
| 1 | 1.70743165 | 1.47283738 | 2.67035500 |
| 1 | 2.67498800 | 2.96706815 | 2.63637399 |
| 1 | 5.87855330 | -0.16340935 | -1.06926471 |
| 1 | 4.80706604 | 2.74168021 | 1.89533591 |
| 1 | -3.61090952 | 3.65064356 | -4.83941177 |
| 1 | -3.75713820 | 4.90502942 | -3.59443406 |
| 1 | -2.43788402 | 4.97946949 | -4.79354750 |
| 1 | -2.53047531 | 1.79418411 | -1.83832270 |
| 1 | -3.76671674 | 3.08039761 | -1.81003136 |
| 1 | -3.72455480 | 1.91733752 | -3.15600100 |
| 1 | -0.59318423 | 3.24129286 | -4.77783292 |
| 1 | -0.59493500 | 1.91564560 | -3.58714697 |
| 1 | -1.84604747 | 1.98118216 | -4.85700741 |
| 1 | 4.30470230 | -2.56337048 | -3.03927235 |
| 1 | 5.36904017 | -2.20624764 | -1.66680111 |
| 1 | 5.12990518 | -0.99642640 | -2.95656900 |
| 1 | 1.49914347 | -0.29563061 | -2.23052296 |
| 1 | 2.88777421 | 0.13829504 | -3.25181115 |
| 1 | 2.16319740 | -1.48051564 | -3.38505684 |
| 1 | 3.47232903 | -2.82782358 | -0.12724841 |
| 1 | 1.86331902 | -2.08330906 | -0.30095736 |
| 1 | 2.47675252 | -3.19362843 | -1.55540488 |
| 1 | -0.22273602 | 2.83162596 | 2.99972852 |
| 31 | 0.03906439 | 1.36254261 | 0.25553861 |
| 8 | -0.94201883 | -6.56149353 | 1.73761239 |
| 6 | -2.30497310 | -6.94541894 | 1.43361313 |
| 1 | -2.62946646 | -7.76325607 | 2.08910167 |
| 6 | 0.71489097 | -5.81880251 | 3.27604227 |
| 6 | 0.93232674 | -4.64954879 | 4.24975432 |
| 6 | 0.85981194 | -3.25806637 | 3.60211596 |
| 6 | -0.74650093 | -6.13887398 | 3.02192474 |
| 6 | -0.50104848 | -2.89527299 | 2.99442541 |
| 6 | -0.50659112 | -1.47194216 | 2.45491157 |
| 1 | 1.14411826 | -6.73896966 | 3.70705174 |
| 1 | 1.92464766 | -4.76409856 | 4.71129001 |
| 1 | 1.12127712 | -2.51008849 | 4.36945226 |
| 1 | -1.29529891 | -3.00459110 | 3.74896266 |
| 1 | 1.22870756 | -5.65324154 | 2.31850109 |
| 1 | 0.19316138 | -4.72898871 | 5.06160345 |
| 1 | 1.63877933 | -3.18275408 | 2.82357609 |
| 1 | -0.74933841 | -3.58142572 | 2.16916172 |
| 1 | 0.26599137 | -1.34435062 | 1.67916566 |
| 1 | -0.30127766 | -0.74624169 | 3.25820252 |
| 8 | -1.63814565 | -6.07246746 | 3.84699605 |
| 8 | -1.82016339 | -1.21209936 | 1.90668028 |
| 1 | -2.28834977 | -7.26849226 | 0.38829554 |

| | | | |
|---|---|---|---|
| 1 | -2.98082351 | -6.09118174 | 1.56697444 |

Electronic energy = -3908.669218 a.u.
DFT-D3(BJ) dispersion correction = -0.141215 a.u.
Thermal free energy = 0.741723 a.u.
Gibbs free energy = -3908.068711 a.u.
Number of imaginary frequencies = 0.

**Int3b CL isomer/conformer 10**

| Atomic N. | X | Y | Z |
|---|---|---|---|
| 7 | 0.70322819 | 2.33098956 | 2.21963220 |
| 8 | 0.09557546 | 1.61926916 | -0.59897781 |
| 8 | 2.92942982 | 1.12658563 | 0.60615982 |
| 6 | -0.36777929 | 1.95747765 | 3.17547210 |
| 6 | -0.90200775 | 2.52162737 | -0.44889417 |
| 6 | -1.97111687 | 2.55177985 | -1.39472388 |
| 6 | -3.00128327 | 3.47725773 | -1.17519521 |
| 6 | -3.00566581 | 4.36550218 | -0.09620066 |
| 6 | -1.93132489 | 4.36365902 | 0.78664496 |
| 6 | -0.87257247 | 3.46574273 | 0.61091425 |
| 6 | 0.34905730 | 3.58769701 | 1.48148367 |
| 1 | -3.83359253 | 5.06174394 | 0.03714457 |
| 6 | -1.98310594 | 1.62990893 | -2.63089860 |
| 6 | 3.58358322 | 2.24610333 | 0.99016493 |
| 6 | 4.72932061 | 2.68710576 | 0.25965056 |
| 6 | 5.37908456 | 3.84425410 | 0.71045340 |
| 6 | 4.95099009 | 4.56895556 | 1.82617884 |
| 6 | 3.83714401 | 4.12937611 | 2.53166865 |
| 6 | 3.15707718 | 2.97239490 | 2.13301933 |
| 6 | 2.00308795 | 2.48301280 | 2.96799910 |
| 1 | 5.48580892 | 5.46696631 | 2.13501357 |
| 6 | 5.24228578 | 1.92291186 | -0.97669735 |
| 6 | -3.21368817 | 1.87731084 | -3.52374001 |
| 6 | -2.01565762 | 0.14488508 | -2.20358539 |
| 6 | -0.72678315 | 1.89891058 | -3.49388939 |
| 6 | 6.48850403 | 2.58574970 | -1.59369255 |
| 6 | 4.15279002 | 1.89121274 | -2.07454895 |
| 6 | 5.64435117 | 0.48455927 | -0.57321791 |
| 1 | -0.48694272 | 2.75064021 | 3.93243647 |
| 1 | -1.31329090 | 1.83384813 | 2.63620642 |
| 1 | 0.20860166 | 4.40051939 | 2.21499919 |
| 1 | 1.22944294 | 3.84139580 | 0.87244451 |
| 1 | -3.83700609 | 3.51165298 | -1.87173480 |
| 1 | -1.89248739 | 5.07668898 | 1.61347483 |
| 1 | 2.21363591 | 1.48826584 | 3.39290180 |
| 1 | 1.83809970 | 3.18042228 | 3.80693930 |
| 1 | 6.25257031 | 4.20332678 | 0.16935256 |
| 1 | 3.48707144 | 4.67638328 | 3.41004973 |
| 1 | -3.16287604 | 1.20837031 | -4.39472388 |
| 1 | -4.15657239 | 1.66560615 | -2.99871841 |
| 1 | -3.25001762 | 2.90926292 | -3.90117210 |
| 1 | -1.12894887 | -0.11928007 | -1.61714681 |
| 1 | -2.91231956 | -0.06504457 | -1.60130208 |

| | | | |
|---|---|---|---|
| 1 | -2.04967933 | -0.49979216 | -3.09544716 |
| 1 | -0.71720600 | 2.94016517 | -3.84727606 |
| 1 | 0.19442420 | 1.71527532 | -2.92973665 |
| 1 | -0.73391559 | 1.24246174 | -4.37764670 |
| 1 | 6.81098820 | 1.99752383 | -2.46478735 |
| 1 | 7.33143221 | 2.62085705 | -0.88856200 |
| 1 | 6.28345589 | 3.60783595 | -1.94331666 |
| 1 | 3.23311250 | 1.41364954 | -1.72139082 |
| 1 | 3.90548457 | 2.91173336 | -2.40091925 |
| 1 | 4.52154134 | 1.33697012 | -2.95127792 |
| 1 | 6.47173356 | 0.50512361 | 0.15103377 |
| 1 | 4.80420519 | -0.05318324 | -0.12030444 |
| 1 | 5.98473177 | -0.07324885 | -1.45919889 |
| 1 | -0.10401288 | 1.01142745 | 3.65910564 |
| 6 | 2.28567312 | -2.36163192 | 1.70132272 |
| 6 | 2.59092606 | -3.75589311 | 1.14096768 |
| 6 | 3.01312677 | -3.78686824 | -0.33224056 |
| 6 | 0.98346631 | -1.69980034 | 1.22990936 |
| 6 | 2.02850199 | -3.12723440 | -1.30804174 |
| 6 | 1.97148091 | -1.60546777 | -1.20915574 |
| 1 | 3.10781669 | -1.65593391 | 1.50491818 |
| 1 | 3.40076922 | -4.19580821 | 1.74419867 |
| 1 | 3.16863599 | -4.83547464 | -0.63237657 |
| 1 | 2.35257924 | -3.35270802 | -2.33732014 |
| 1 | 2.18109673 | -2.42437529 | 2.79444192 |
| 1 | 1.70886013 | -4.39542434 | 1.28869007 |
| 1 | 3.99264273 | -3.28935874 | -0.44113489 |
| 1 | 1.01791261 | -3.54929110 | -1.19774842 |
| 1 | 1.54668775 | -1.17821200 | -2.12864478 |
| 1 | 2.98612650 | -1.19510436 | -1.07968553 |
| 8 | 0.64799179 | -0.58313676 | 1.95139213 |
| 8 | 1.11825140 | -1.07153415 | -0.16115625 |
| 31 | 1.08216895 | 0.85277211 | 0.79462096 |
| 8 | -8.35925287 | -4.62848896 | 0.22132603 |
| 6 | -9.32643462 | -5.69411152 | 0.06407598 |
| 1 | -9.11957269 | -6.26923286 | -0.84726340 |
| 6 | -6.13132774 | -3.86302982 | 0.48299740 |
| 6 | -4.66237188 | -4.26011316 | 0.62343174 |
| 6 | -3.74159363 | -3.04459804 | 0.77292458 |
| 6 | -7.06360231 | -5.04870941 | 0.32596287 |
| 6 | -2.26497104 | -3.43124843 | 0.91293599 |
| 6 | -1.36661252 | -2.21081617 | 1.05340745 |
| 1 | -6.28196263 | -3.20162342 | -0.38603235 |
| 1 | -4.36095492 | -4.85278306 | -0.25382131 |
| 1 | -3.86627786 | -2.38097311 | -0.10028378 |
| 1 | -1.94735372 | -4.01541934 | 0.03427126 |
| 1 | -6.47153519 | -3.28095335 | 1.35504880 |
| 1 | -4.54675466 | -4.92684035 | 1.49174884 |
| 1 | -4.05201682 | -2.45536670 | 1.65334193 |
| 1 | -2.12947735 | -4.08110110 | 1.79244570 |
| 1 | -1.64647927 | -1.61278682 | 1.93430600 |
| 1 | -1.44055689 | -1.56754535 | 0.16246925 |
| 8 | -6.73452704 | -6.21918604 | 0.29315679 |

| | | | |
|---|---|---|---|
| 8 | -0.00859400 | -2.68036831 | 1.20573958 |
| 1 | -10.29804412 | -5.19541735 | -0.00429497 |
| 1 | -9.29643766 | -6.37060916 | 0.92752825 |

Electronic energy = -3908.670759 a.u.
DFT-D3(BJ) dispersion correction = -0.137918 a.u.
Thermal free energy = 0.740224 a.u.
Gibbs free energy = -3908.068453 a.u.
Number of imaginary frequencies = 0.

**Int3b CL isomer/conformer 11**

| Atomic N. | X | Y | Z |
|---|---|---|---|
| 6 | -3.56643389 | -1.40489100 | 1.05607558 |
| 6 | -4.24587582 | -2.73677775 | 0.71406569 |
| 6 | -4.29471678 | -3.08066872 | -0.77868271 |
| 6 | -2.03579789 | -1.35614619 | 0.90099955 |
| 6 | -2.92926102 | -3.10745724 | -1.47823208 |
| 6 | -2.28415454 | -1.73476346 | -1.63551342 |
| 1 | -3.99360693 | -0.57372649 | 0.47423590 |
| 1 | -5.27677689 | -2.70511834 | 1.10085562 |
| 1 | -4.78096567 | -4.06174990 | -0.89942930 |
| 1 | -3.06379872 | -3.50334302 | -2.49829261 |
| 1 | -3.75691525 | -1.15777316 | 2.11085688 |
| 1 | -3.73345414 | -3.54747944 | 1.25399945 |
| 1 | -4.93918022 | -2.35146227 | -1.30016552 |
| 1 | -2.23196466 | -3.78605301 | -0.96377579 |
| 1 | -1.51065661 | -1.76094442 | -2.41467462 |
| 1 | -3.03938821 | -0.98805779 | -1.93190287 |
| 8 | -1.50660731 | -0.23001441 | 1.49610654 |
| 8 | -1.56291052 | -1.24185409 | -0.47732143 |
| 7 | 0.42466419 | 2.07234218 | 1.31319324 |
| 8 | -1.29543095 | 1.70035835 | -1.09146522 |
| 8 | 1.10525198 | -0.23338832 | -0.43256382 |
| 6 | -0.38324428 | 2.43974615 | 2.50237698 |
| 6 | -1.52132674 | 3.02695898 | -0.93341978 |
| 6 | -2.63694233 | 3.63534049 | -1.58727051 |
| 6 | -2.83613282 | 5.00882708 | -1.39018772 |
| 6 | -1.99622948 | 5.79038546 | -0.59307196 |
| 6 | -0.90042704 | 5.19362937 | 0.01925635 |
| 6 | -0.64516678 | 3.82861399 | -0.15498759 |
| 6 | 0.62577717 | 3.25162060 | 0.40624101 |
| 1 | -2.19601001 | 6.85389249 | -0.46276808 |
| 6 | -3.59768430 | 2.82097702 | -2.47572709 |
| 6 | 2.34731968 | 0.29117987 | -0.37851435 |
| 6 | 3.30963472 | -0.09166906 | -1.36268748 |
| 6 | 4.59377905 | 0.45958554 | -1.26005485 |
| 6 | 4.95230195 | 1.35811886 | -0.25028998 |
| 6 | 4.00754347 | 1.72014220 | 0.70348373 |
| 6 | 2.71384740 | 1.18688903 | 0.65884746 |
| 6 | 1.75089085 | 1.51939642 | 1.76865432 |
| 1 | 5.96121100 | 1.76955996 | -0.21672681 |
| 6 | 2.94961369 | -1.07039490 | -2.49825632 |
| 6 | -4.68489685 | 3.70168614 | -3.11989109 |

| | | | |
|---|---|---|---|
| 6 | -4.31802309 | 1.75795953 | -1.61568632 |
| 6 | -2.81928839 | 2.14252134 | -3.62794952 |
| 6 | 4.15116542 | -1.34994545 | -3.42062068 |
| 6 | 1.82605800 | -0.47163804 | -3.37760916 |
| 6 | 2.49560907 | -2.42853547 | -1.91114384 |
| 1 | 0.14592323 | 3.20555412 | 3.09301519 |
| 1 | -1.35132111 | 2.83792535 | 2.18043100 |
| 1 | 1.17630741 | 4.02989170 | 0.96221948 |
| 1 | 1.28271142 | 2.90148837 | -0.40437555 |
| 1 | -3.68294333 | 5.49313646 | -1.87289021 |
| 1 | -0.21487913 | 5.78771728 | 0.62790039 |
| 1 | 1.52084993 | 0.62747217 | 2.37198556 |
| 1 | 2.21474169 | 2.25436828 | 2.44726173 |
| 1 | 5.34691222 | 0.18788658 | -1.99766503 |
| 1 | 4.26749829 | 2.41426217 | 1.50589922 |
| 1 | -5.32976237 | 3.07015008 | -3.74786871 |
| 1 | -5.32725120 | 4.18550544 | -2.37006847 |
| 1 | -4.25523921 | 4.48113415 | -3.76545990 |
| 1 | -3.59529808 | 1.09919934 | -1.12256770 |
| 1 | -4.93298130 | 2.23889009 | -0.84105935 |
| 1 | -4.98249915 | 1.14651518 | -2.24592070 |
| 1 | -2.34474928 | 2.90022940 | -4.26831174 |
| 1 | -2.03885611 | 1.47375460 | -3.24984464 |
| 1 | -3.51266991 | 1.55983084 | -4.25395678 |
| 1 | 3.84263046 | -2.05569302 | -4.20509751 |
| 1 | 4.99211233 | -1.80552970 | -2.87788961 |
| 1 | 4.51184485 | -0.43883595 | -3.91955126 |
| 1 | 0.91995498 | -0.27408550 | -2.79488579 |
| 1 | 2.15492346 | 0.47205088 | -3.83651257 |
| 1 | 1.57469931 | -1.17223823 | -4.18880142 |
| 1 | 3.30655088 | -2.88351046 | -1.32298169 |
| 1 | 1.61669573 | -2.31549580 | -1.26708308 |
| 1 | 2.24569984 | -3.12160146 | -2.72923867 |
| 1 | -0.54800442 | 1.54572424 | 3.11306343 |
| 31 | -0.45990320 | 0.58898594 | 0.16437971 |
| 8 | 5.25364977 | -1.68939268 | 3.17421538 |
| 6 | 6.07873386 | -0.58066197 | 3.60452827 |
| 1 | 5.85300920 | 0.31410944 | 3.01054807 |
| 6 | 3.13690754 | -2.68610849 | 2.79884196 |
| 6 | 1.68862150 | -2.73897022 | 3.28554974 |
| 6 | 0.89731626 | -3.87225735 | 2.62611562 |
| 6 | 3.91454591 | -1.50860728 | 3.35017167 |
| 6 | -0.56044573 | -3.97307694 | 3.09953480 |
| 6 | -1.41817477 | -2.75094615 | 2.78326668 |
| 1 | 3.16589744 | -2.59998385 | 1.69885833 |
| 1 | 1.21154875 | -1.77235121 | 3.07230866 |
| 1 | 0.90760245 | -3.73441473 | 1.53384129 |
| 1 | -0.59279715 | -4.12668523 | 4.19169609 |
| 1 | 3.68295313 | -3.61095515 | 3.03912473 |
| 1 | 1.67358243 | -2.85507516 | 4.38196163 |
| 1 | 1.39765168 | -4.83426741 | 2.82759354 |
| 1 | -1.02867925 | -4.85888728 | 2.64151135 |
| 1 | -1.02855171 | -1.84590682 | 3.27477178 |

| | | | |
|---|---|---|---|
| 1 | -2.44796962 | -2.91947539 | 3.15052465 |
| 8 | 3.43537779 | -0.51588262 | 3.87096754 |
| 8 | -1.41339919 | -2.56183846 | 1.35820652 |
| 1 | 7.10990179 | -0.90520518 | 3.43498647 |
| 1 | 5.90762017 | -0.36372150 | 4.66638410 |

Electronic energy = -3908.668073 a.u.
DFT-D3(BJ) dispersion correction = -0.143196 a.u.
Thermal free energy = 0.742861 a.u.
Gibbs free energy = -3908.068407 a.u.
Number of imaginary frequencies = 0.

**Int3b CL isomer/conformer 12**

| Atomic N. | X | Y | Z |
|---|---|---|---|
| 6 | -3.82616085 | 0.03987674 | 0.85143249 |
| 6 | -4.69697435 | -1.04176189 | 0.20429604 |
| 6 | -4.35170641 | -1.39565769 | -1.25154938 |
| 6 | -2.31789956 | -0.21968668 | 0.88683595 |
| 6 | -3.03145971 | -2.17018221 | -1.39347224 |
| 6 | -1.76318443 | -1.31424151 | -1.41497130 |
| 1 | -3.95665783 | 0.99917313 | 0.33062550 |
| 1 | -5.74086862 | -0.69260451 | 0.24730836 |
| 1 | -5.16352259 | -2.02332603 | -1.65078326 |
| 1 | -3.02845845 | -2.73765079 | -2.33842456 |
| 1 | -4.13754795 | 0.19191880 | 1.89574658 |
| 1 | -4.65112041 | -1.95933174 | 0.81190982 |
| 1 | -4.33098359 | -0.48449689 | -1.87205520 |
| 1 | -2.96828313 | -2.91001649 | -0.58286319 |
| 1 | -0.88037172 | -1.93052044 | -1.18421356 |
| 1 | -1.61845266 | -0.87181138 | -2.41112848 |
| 8 | -1.61649797 | 0.81084290 | 1.45278000 |
| 8 | -1.78468017 | -0.15938988 | -0.53763268 |
| 7 | 0.53776834 | 2.81506472 | 1.29774514 |
| 8 | -0.84450824 | 2.51258632 | -1.31790270 |
| 8 | 1.09094875 | 0.31109020 | -0.24110591 |
| 6 | -0.32580173 | 3.33390158 | 2.38695631 |
| 6 | -0.98607802 | 3.85566928 | -1.21452763 |
| 6 | -1.94552567 | 4.52966127 | -2.02831606 |
| 6 | -2.06439520 | 5.91779599 | -1.86996837 |
| 6 | -1.28266005 | 6.64870955 | -0.97120270 |
| 6 | -0.32402173 | 5.98603840 | -0.21261998 |
| 6 | -0.15739926 | 4.60241225 | -0.33709844 |
| 6 | 0.97477419 | 3.92561499 | 0.38639095 |
| 1 | -1.41725370 | 7.72635825 | -0.87859823 |
| 6 | -2.80658550 | 3.77209670 | -3.05840590 |
| 6 | 2.37445809 | 0.72106221 | -0.12161208 |
| 6 | 3.36869264 | 0.21768189 | -1.01464590 |
| 6 | 4.68630809 | 0.65892264 | -0.82969004 |
| 6 | 5.05074102 | 1.55977965 | 0.17490722 |
| 6 | 4.07434456 | 2.04362727 | 1.03782430 |
| 6 | 2.74462085 | 1.62631543 | 0.90645942 |
| 6 | 1.72474838 | 2.11107741 | 1.90255358 |
| 1 | 6.08803581 | 1.87906788 | 0.27467033 |

| | | | |
|---|---|---|---|
| 6 | 3.01612487 | -0.77326936 | -2.14165844 |
| 6 | -3.74299737 | 4.71672140 | -3.83504281 |
| 6 | -3.69307030 | 2.72191393 | -2.35054401 |
| 6 | -1.89248076 | 3.07481101 | -4.09439552 |
| 6 | 4.25160091 | -1.17019658 | -2.97180719 |
| 6 | 1.99769155 | -0.13430950 | -3.11520610 |
| 6 | 2.43544027 | -2.07355161 | -1.53747156 |
| 1 | 0.23887118 | 4.05706975 | 2.99893988 |
| 1 | -1.19990463 | 3.83325712 | 1.95610473 |
| 1 | 1.53949231 | 4.66581821 | 0.97929153 |
| 1 | 1.67426127 | 3.47295959 | -0.33183812 |
| 1 | -2.79693667 | 6.45610945 | -2.46859269 |
| 1 | 0.32064357 | 6.54050232 | 0.47331020 |
| 1 | 1.30541266 | 1.27217608 | 2.48152426 |
| 1 | 2.20887075 | 2.80086357 | 2.61484734 |
| 1 | 5.46373725 | 0.29371573 | -1.49819978 |
| 1 | 4.33620710 | 2.74702365 | 1.83150938 |
| 1 | -4.32057533 | 4.12806552 | -4.56208906 |
| 1 | -4.46167620 | 5.22383309 | -3.17492330 |
| 1 | -3.18672281 | 5.48110228 | -4.39645813 |
| 1 | -3.08420222 | 1.97223807 | -1.83407084 |
| 1 | -4.35641856 | 3.20417111 | -1.61716518 |
| 1 | -4.32533300 | 2.20822023 | -3.09132262 |
| 1 | -1.29071280 | 3.81663705 | -4.63934798 |
| 1 | -1.21369250 | 2.36141580 | -3.61409224 |
| 1 | -2.50782604 | 2.53359304 | -4.82960307 |
| 1 | 3.94296212 | -1.87759538 | -3.75474350 |
| 1 | 5.01920129 | -1.66743661 | -2.36137655 |
| 1 | 4.71022972 | -0.30469166 | -3.47122982 |
| 1 | 1.07124575 | 0.14972403 | -2.60557751 |
| 1 | 2.42269444 | 0.76588304 | -3.58212073 |
| 1 | 1.75161678 | -0.84643202 | -3.91772284 |
| 1 | 3.17860846 | -2.56466457 | -0.89215692 |
| 1 | 1.53904577 | -1.86956073 | -0.94188389 |
| 1 | 2.17069473 | -2.77586552 | -2.34270458 |
| 1 | -0.66502621 | 2.49832711 | 3.00742371 |
| 31 | -0.41198373 | 1.38127652 | 0.10516915 |
| 8 | 3.53965742 | -7.21143567 | 4.60451978 |
| 6 | 3.73369661 | -8.58095503 | 5.03184643 |
| 1 | 3.07582353 | -8.81618664 | 5.87801648 |
| 6 | 2.16855995 | -5.46716311 | 3.76911141 |
| 6 | 0.78387167 | -5.08409948 | 3.24833045 |
| 6 | 0.69995355 | -3.60896928 | 2.84256596 |
| 6 | 2.27176090 | -6.92113550 | 4.18759188 |
| 6 | -0.68463265 | -3.21815377 | 2.31337706 |
| 6 | -0.74964960 | -1.75095951 | 1.91445416 |
| 1 | 2.45010391 | -4.84926762 | 4.63779678 |
| 1 | 0.03129558 | -5.30415827 | 4.02081703 |
| 1 | 0.95370680 | -2.97553884 | 3.71043423 |
| 1 | -1.44998459 | -3.41662329 | 3.08072422 |
| 1 | 2.94600145 | -5.27736041 | 3.01144097 |
| 1 | 0.52958292 | -5.72322409 | 2.38880391 |
| 1 | 1.46141030 | -3.39544386 | 2.07264725 |

| | | | |
|---|---|---|---|
| 1 | -0.93971923 | -3.84266229 | 1.44196507 |
| 1 | -0.02137228 | -1.52778526 | 1.11762465 |
| 1 | -0.51973645 | -1.09986626 | 2.77298709 |
| 8 | 1.36983892 | -7.73701716 | 4.16823932 |
| 8 | -2.09512574 | -1.47788361 | 1.45895462 |
| 1 | 4.78502766 | -8.64544404 | 5.32835590 |
| 1 | 3.51845453 | -9.27418951 | 4.20896595 |

Electronic energy = -3908.671511 a.u.
DFT-D3(BJ) dispersion correction = -0.139006 a.u.
Thermal free energy = 0.742482 a.u.
Gibbs free energy = -3908.068036 a.u.
Number of imaginary frequencies = 0.

**Int3b CL isomer/conformer 13**

| Atomic N. | X | Y | Z |
|---|---|---|---|
| 7 | 0.26880392 | 2.57309625 | 1.71156601 |
| 8 | -0.14454831 | 1.96341798 | -1.16491995 |
| 8 | 2.55207162 | 1.28624918 | 0.24779063 |
| 6 | -0.89272848 | 2.23544905 | 2.57009877 |
| 6 | -1.10865215 | 2.90892093 | -1.06597869 |
| 6 | -2.10109917 | 3.01140973 | -2.08704550 |
| 6 | -3.09812249 | 3.98345068 | -1.92250628 |
| 6 | -3.14025464 | 4.84872103 | -0.82579678 |
| 6 | -2.13824553 | 4.77324019 | 0.13539679 |
| 6 | -1.11460678 | 3.82652518 | 0.01708899 |
| 6 | 0.03975857 | 3.86377650 | 0.98195125 |
| 1 | -3.94072839 | 5.58322955 | -0.73779968 |
| 6 | -2.06714540 | 2.11398907 | -3.34047877 |
| 6 | 3.23038122 | 2.36074556 | 0.71105460 |
| 6 | 4.45201780 | 2.75613128 | 0.08482606 |
| 6 | 5.12193532 | 3.86699008 | 0.61550842 |
| 6 | 4.64346221 | 4.58903329 | 1.71228755 |
| 6 | 3.45558643 | 4.19399080 | 2.31598434 |
| 6 | 2.75147826 | 3.08360055 | 1.83514079 |
| 6 | 1.51077704 | 2.63816581 | 2.56344483 |
| 1 | 5.19693562 | 5.45062003 | 2.08532414 |
| 6 | 5.02257198 | 1.99300221 | -1.12661406 |
| 6 | -3.21736016 | 2.43603817 | -4.31295878 |
| 6 | -2.19938381 | 0.62514105 | -2.94718280 |
| 6 | -0.74059685 | 2.33772489 | -4.10571149 |
| 6 | 6.34650116 | 2.60181106 | -1.62622354 |
| 6 | 4.02423710 | 2.04314960 | -2.30732892 |
| 6 | 5.31687156 | 0.52691467 | -0.72981615 |
| 1 | -1.02852531 | 3.01426635 | 3.33907064 |
| 1 | -1.79810760 | 2.17879790 | 1.95598073 |
| 1 | -0.11508439 | 4.66613860 | 1.72400024 |
| 1 | 0.97722865 | 4.08295678 | 0.44957101 |
| 1 | -3.87646671 | 4.07376753 | -2.67800828 |
| 1 | -2.12797224 | 5.46521595 | 0.98074108 |
| 1 | 1.63507689 | 1.62423013 | 2.97687925 |
| 1 | 1.31565752 | 3.32431369 | 3.40522214 |
| 1 | 6.05314470 | 4.19085389 | 0.15434744 |

| | | | |
|---|---|---|---|
| 1 | 3.06478558 | 4.73954621 | 3.17792763 |
| 1 | -3.13597361 | 1.77989262 | -5.19133189 |
| 1 | -4.20442262 | 2.26165829 | -3.86053831 |
| 1 | -3.17738551 | 3.47454890 | -4.67171418 |
| 1 | -1.37181624 | 0.31059068 | -2.30238398 |
| 1 | -3.14681566 | 0.44628744 | -2.41720708 |
| 1 | -2.19596552 | -0.00193898 | -3.85211006 |
| 1 | -0.65675324 | 3.38339328 | -4.43554884 |
| 1 | 0.12790339 | 2.09984520 | -3.48135905 |
| 1 | -0.71463437 | 1.69811787 | -5.00138352 |
| 1 | 6.70698915 | 2.01608072 | -2.48400859 |
| 1 | 7.13103364 | 2.57698752 | -0.85625797 |
| 1 | 6.22276570 | 3.64055515 | -1.96479175 |
| 1 | 3.05537301 | 1.60913619 | -2.03995791 |
| 1 | 3.85770867 | 3.08214016 | -2.62654668 |
| 1 | 4.43245777 | 1.48826952 | -3.16599391 |
| 1 | 6.08485873 | 0.48774554 | 0.05642777 |
| 1 | 4.41705895 | 0.02561856 | -0.35671708 |
| 1 | 5.69619490 | -0.02956379 | -1.60070050 |
| 1 | -0.71873055 | 1.26415337 | 3.04435405 |
| 6 | 1.64957276 | -2.17690661 | 1.19548695 |
| 6 | 1.93993573 | -3.57230592 | 0.62975313 |
| 6 | 2.47979038 | -3.59499813 | -0.80477891 |
| 6 | 0.42434983 | -1.44430032 | 0.62976273 |
| 6 | 1.60954646 | -2.87026285 | -1.84129778 |
| 6 | 1.61568185 | -1.34974431 | -1.71243607 |
| 1 | 2.51644907 | -1.50664613 | 1.08715034 |
| 1 | 2.67746410 | -4.05975253 | 1.28687455 |
| 1 | 2.60919338 | -4.64393684 | -1.11560154 |
| 1 | 2.00459231 | -3.09179394 | -2.84630448 |
| 1 | 1.44862970 | -2.25660243 | 2.27391912 |
| 1 | 1.02185988 | -4.17442645 | 0.68980316 |
| 1 | 3.48700527 | -3.14332793 | -0.82308050 |
| 1 | 0.57472693 | -3.24495645 | -1.82199077 |
| 1 | 1.28826540 | -0.88455544 | -2.65305642 |
| 1 | 2.63398983 | -0.99136304 | -1.49091729 |
| 8 | 0.08113622 | -0.32901207 | 1.35152232 |
| 8 | 0.70456874 | -0.79525133 | -0.72518759 |
| 31 | 0.68346482 | 1.11111836 | 0.27950453 |
| 8 | -5.35903448 | -6.97334811 | 4.40872054 |
| 6 | -6.28500435 | -8.07546231 | 4.56157444 |
| 1 | -7.29771793 | -7.76983465 | 4.26973202 |
| 6 | -4.26712007 | -5.34301828 | 3.07802346 |
| 6 | -4.09281300 | -4.75925705 | 1.67647995 |
| 6 | -3.08760882 | -3.60384397 | 1.64540695 |
| 6 | -5.25537084 | -6.49164573 | 3.13412646 |
| 6 | -2.90416657 | -3.01574527 | 0.24219831 |
| 6 | -1.91513775 | -1.86026574 | 0.17825102 |
| 1 | -4.60746933 | -4.57292820 | 3.78972809 |
| 1 | -5.07064854 | -4.41964305 | 1.30088494 |
| 1 | -3.42048615 | -2.80472164 | 2.33096847 |
| 1 | -3.87210141 | -2.64413965 | -0.13424829 |
| 1 | -3.30651782 | -5.70596350 | 3.47869141 |

| | | | |
|---|---|---|---|
| 1 | -3.76775894 | -5.55673411 | 0.99047678 |
| 1 | -2.11172568 | -3.94824007 | 2.02311529 |
| 1 | -2.57997521 | -3.80593605 | -0.45501243 |
| 1 | -2.18092626 | -1.07071729 | 0.89763542 |
| 1 | -1.89470615 | -1.42146874 | -0.83118894 |
| 8 | -5.88288278 | -6.94353954 | 2.19522630 |
| 8 | -0.60426412 | -2.37892190 | 0.50121501 |
| 1 | -6.25006033 | -8.33796050 | 5.62330896 |
| 1 | -5.97622744 | -8.92577993 | 3.94049622 |

Electronic energy = -3908.670784 a.u.
DFT-D3(BJ) dispersion correction = -0.137910 a.u.
Thermal free energy = 0.741867 a.u.
Gibbs free energy = -3908.066828 a.u.
Number of imaginary frequencies = 0.

**Int3b CL isomer/conformer 14**

| Atomic N. | X | Y | Z |
|---|---|---|---|
| 7 | 0.13790213 | 2.57295324 | 2.63267112 |
| 8 | -0.33268359 | 2.05244096 | -0.25396582 |
| 8 | 2.42987601 | 1.44252969 | 1.05406727 |
| 6 | -0.97424557 | 2.14794585 | 3.51753499 |
| 6 | -1.33972252 | 2.94257308 | -0.09126097 |
| 6 | -2.36367946 | 3.03618612 | -1.08155726 |
| 6 | -3.40543416 | 3.94528512 | -0.84832387 |
| 6 | -3.46177478 | 4.75952779 | 0.28615274 |
| 6 | -2.42909697 | 4.69878259 | 1.21547550 |
| 6 | -1.36114188 | 3.81408117 | 1.02906619 |
| 6 | -0.18027098 | 3.87564480 | 1.96007564 |
| 1 | -4.29708632 | 5.44528833 | 0.42745938 |
| 6 | -2.31516943 | 2.19997143 | -2.37640907 |
| 6 | 3.07206819 | 2.53396700 | 1.52928801 |
| 6 | 4.25293448 | 3.00558059 | 0.87831387 |
| 6 | 4.88668921 | 4.13129322 | 1.42199439 |
| 6 | 4.41070928 | 4.79657396 | 2.55515977 |
| 6 | 3.26356202 | 4.32670058 | 3.18355222 |
| 6 | 2.59706161 | 3.19864930 | 2.69032947 |
| 6 | 1.40481879 | 2.67066909 | 3.44395944 |
| 1 | 4.93479436 | 5.67238680 | 2.93741523 |
| 6 | 4.81969426 | 2.30614045 | -0.37272676 |
| 6 | -3.50511721 | 2.50515335 | -3.30567438 |
| 6 | -2.35883824 | 0.68934232 | -2.05255565 |
| 6 | -1.02209088 | 2.52992521 | -3.16035067 |
| 6 | 6.09748535 | 2.99138619 | -0.89307147 |
| 6 | 3.78329869 | 2.34346681 | -1.52066725 |
| 6 | 5.19426091 | 0.84470379 | -0.03019287 |
| 1 | -1.12191479 | 2.89348641 | 4.31668451 |
| 1 | -1.89664299 | 2.06455588 | 2.93247167 |
| 1 | -0.35229809 | 4.64112229 | 2.73651951 |
| 1 | 0.72720696 | 4.16273811 | 1.40832230 |
| 1 | -4.20862584 | 4.02599929 | -1.57849026 |
| 1 | -2.43031991 | 5.35500069 | 2.08893286 |
| 1 | 1.59148617 | 1.65111813 | 3.81816852 |

| | | | |
|---|---|---|---|
| 1 | 1.20703818 | 3.31879321 | 4.31471661 |
| 1 | 5.78611638 | 4.51315359 | 0.94249448 |
| 1 | 2.87624304 | 4.82668818 | 4.07422212 |
| 1 | -3.41157296 | 1.89677046 | -4.21664105 |
| 1 | -4.46992358 | 2.25694236 | -2.83989069 |
| 1 | -3.52888872 | 3.56015816 | -3.61411722 |
| 1 | -1.49800890 | 0.38831202 | -1.44580965 |
| 1 | -3.28029344 | 0.43631361 | -1.50728468 |
| 1 | -2.34897571 | 0.10683545 | -2.98685936 |
| 1 | -1.00150053 | 3.59291606 | -3.44109575 |
| 1 | -0.12674229 | 2.30974293 | -2.56822786 |
| 1 | -0.98540868 | 1.93538565 | -4.08621638 |
| 1 | 6.45782854 | 2.44872562 | -1.77879670 |
| 1 | 6.90604986 | 2.98122631 | -0.14802217 |
| 1 | 5.91502946 | 4.03258657 | -1.19560227 |
| 1 | 2.84484276 | 1.85598627 | -1.23742390 |
| 1 | 3.55813158 | 3.38225572 | -1.80232424 |
| 1 | 4.18952259 | 1.83408726 | -2.40799885 |
| 1 | 5.98894196 | 0.81944286 | 0.72965937 |
| 1 | 4.33162483 | 0.29079752 | 0.35610852 |
| 1 | 5.57000819 | 0.33229187 | -0.92921158 |
| 1 | -0.73533428 | 1.17204755 | 3.95224652 |
| 6 | 1.67520720 | -2.11564159 | 1.88771764 |
| 6 | 1.98027807 | -3.47684225 | 1.25101942 |
| 6 | 2.48620175 | -3.42090978 | -0.19496701 |
| 6 | 0.41471297 | -1.39129889 | 1.39466706 |
| 6 | 1.57944865 | -2.66600987 | -1.17718579 |
| 6 | 1.54719492 | -1.15477228 | -0.96744395 |
| 1 | 2.52201255 | -1.41964798 | 1.78292872 |
| 1 | 2.74339271 | -3.97824351 | 1.86702218 |
| 1 | 2.62970551 | -4.45100944 | -0.55838939 |
| 1 | 1.96352982 | -2.82399416 | -2.19836780 |
| 1 | 1.51053669 | -2.24796251 | 2.96705131 |
| 1 | 1.07651429 | -4.10080647 | 1.30557287 |
| 1 | 3.48361644 | -2.94796929 | -0.21383520 |
| 1 | 0.55476329 | -3.06797835 | -1.16267980 |
| 1 | 1.19493691 | -0.64971756 | -1.87802446 |
| 1 | 2.55861222 | -0.78055558 | -0.74017837 |
| 8 | 0.06803167 | -0.31692102 | 2.17274254 |
| 8 | 0.63413448 | -0.67930676 | 0.05749918 |
| 31 | 0.57276603 | 1.18443963 | 1.13456669 |
| 8 | -4.18809282 | -7.89820054 | -2.69297141 |
| 6 | -5.08023547 | -9.02586201 | -2.86003108 |
| 1 | -5.00754944 | -9.70398689 | -2.00037464 |
| 6 | -3.45471793 | -5.98431557 | -1.50178106 |
| 6 | -3.67754645 | -5.08299433 | -0.28797916 |
| 6 | -2.66918903 | -3.93175248 | -0.22153709 |
| 6 | -4.43923395 | -7.13438860 | -1.58809604 |
| 6 | -2.88602302 | -3.02335227 | 0.99354449 |
| 6 | -1.93682458 | -1.83575842 | 1.06596457 |
| 1 | -2.43890390 | -6.41253017 | -1.49707717 |
| 1 | -3.61579614 | -5.68865542 | 0.62935584 |
| 1 | -1.64515511 | -4.33745279 | -0.19167874 |

| | | | |
|---|---|---|---|
| 1 | -2.79548922 | -3.61125219 | 1.92162683 |
| 1 | -3.52555198 | -5.41119973 | -2.44081095 |
| 1 | -4.70341261 | -4.68388605 | -0.31627092 |
| 1 | -2.73752903 | -3.32565176 | -1.14204243 |
| 1 | -3.91361724 | -2.62262503 | 0.97443475 |
| 1 | -2.19684799 | -1.17621347 | 1.90627397 |
| 1 | -1.97693898 | -1.24681816 | 0.13523378 |
| 8 | -5.33622511 | -7.37061331 | -0.80137144 |
| 8 | -0.59622771 | -2.34429500 | 1.25722678 |
| 1 | -4.74868422 | -9.52414790 | -3.77611240 |
| 1 | -6.11822737 | -8.68352913 | -2.95753590 |

Electronic energy = -3908.670733 a.u.
DFT-D3(BJ) dispersion correction = -0.138520 a.u.
Thermal free energy = 0.742755 a.u.
Gibbs free energy = -3908.066498 a.u.
Number of imaginary frequencies = 0.

**Int3b CL isomer/conformer 15**

| Atomic N. | X | Y | Z |
|---|---|---|---|
| 7 | 0.90360133 | 1.87432626 | 1.45841241 |
| 8 | 0.04983562 | 1.01677271 | -1.25632297 |
| 8 | 2.93520204 | 0.42628569 | -0.21222411 |
| 6 | -0.09612678 | 1.62317238 | 2.52517149 |
| 6 | -0.90393436 | 1.96199995 | -1.08516854 |
| 6 | -2.04744234 | 1.97271914 | -1.93986519 |
| 6 | -3.02533790 | 2.94759431 | -1.69537384 |
| 6 | -2.90969727 | 3.90049060 | -0.67942355 |
| 6 | -1.76465924 | 3.91404941 | 0.10969996 |
| 6 | -0.75415413 | 2.96807209 | -0.09488205 |
| 6 | 0.54077555 | 3.09310084 | 0.66174790 |
| 1 | -3.70059632 | 4.63418301 | -0.52386878 |
| 6 | -2.19307377 | 0.97825390 | -3.10962349 |
| 6 | 3.66812443 | 1.53975349 | 0.01948814 |
| 6 | 4.76986827 | 1.85778776 | -0.83200320 |
| 6 | 5.50420582 | 3.01427046 | -0.53547730 |
| 6 | 5.19963386 | 3.85109760 | 0.54166751 |
| 6 | 4.12849972 | 3.52942858 | 1.36681108 |
| 6 | 3.36823368 | 2.37924586 | 1.12395215 |
| 6 | 2.26718059 | 2.01691997 | 2.08518770 |
| 1 | 5.79672205 | 4.74352350 | 0.72848628 |
| 6 | 5.14833791 | 0.96597747 | -2.03070737 |
| 6 | -3.48349757 | 1.22032035 | -3.91518787 |
| 6 | -2.24120370 | -0.47586575 | -2.58795045 |
| 6 | -1.00342383 | 1.14482515 | -4.08567117 |
| 6 | 6.37108215 | 1.50795621 | -2.79498480 |
| 6 | 3.97533316 | 0.89834628 | -3.03700432 |
| 6 | 5.51484279 | -0.45202587 | -1.53230265 |
| 1 | -0.11557139 | 2.47346563 | 3.22716608 |
| 1 | -1.08908133 | 1.50729335 | 2.07735393 |
| 1 | 0.49679971 | 3.95996583 | 1.34365838 |
| 1 | 1.37587217 | 3.26146375 | -0.03433676 |
| 1 | -3.91567328 | 2.97008681 | -2.32116838 |

| | | | |
|---|---|---|---|
| 1 | -1.63312439 | 4.67500946 | 0.88247558 |
| 1 | 2.46855989 | 1.04865246 | 2.57131024 |
| 1 | 2.20441235 | 2.78444498 | 2.87535613 |
| 1 | 6.34636232 | 3.28162036 | -1.17108875 |
| 1 | 3.87545168 | 4.16480201 | 2.21859716 |
| 1 | -3.52731116 | 0.49890616 | -4.74362376 |
| 1 | -4.38691267 | 1.07726573 | -3.30456975 |
| 1 | -3.51513627 | 2.22844089 | -4.35279137 |
| 1 | -1.32067472 | -0.73935348 | -2.05629863 |
| 1 | -3.09419292 | -0.61614715 | -1.90722285 |
| 1 | -2.36726652 | -1.17107781 | -3.43229621 |
| 1 | -0.98608162 | 2.16179114 | -4.50371066 |
| 1 | -0.04593520 | 0.95776623 | -3.58687360 |
| 1 | -1.10687191 | 0.43747727 | -4.92288021 |
| 1 | 6.59688474 | 0.83182193 | -3.63204747 |
| 1 | 7.26750392 | 1.55839194 | -2.16026396 |
| 1 | 6.18629215 | 2.50604126 | -3.21759277 |
| 1 | 3.06489837 | 0.50374649 | -2.57450605 |
| 1 | 3.75136975 | 1.89852382 | -3.43525936 |
| 1 | 4.24693069 | 0.25093206 | -3.88489425 |
| 1 | 6.39723950 | -0.41316683 | -0.87700618 |
| 1 | 4.68982164 | -0.90460656 | -0.97133799 |
| 1 | 5.75810226 | -1.10017956 | -2.38828304 |
| 1 | 0.16729971 | 0.70282014 | 3.05611337 |
| 6 | 2.10449102 | -2.95876116 | 1.17915244 |
| 6 | 2.27450487 | -4.40597449 | 0.70181001 |
| 6 | 2.61571535 | -4.56629418 | -0.78399093 |
| 6 | 0.83665190 | -2.22711111 | 0.71693423 |
| 6 | 1.63670120 | -3.89231826 | -1.75587872 |
| 6 | 1.70669706 | -2.36776214 | -1.76058757 |
| 1 | 2.96866150 | -2.33536514 | 0.90113927 |
| 1 | 3.07857269 | -4.86866837 | 1.29567855 |
| 1 | 2.67051990 | -5.64139373 | -1.01826141 |
| 1 | 1.88761983 | -4.21057732 | -2.78109663 |
| 1 | 2.04648527 | -2.94285181 | 2.27734761 |
| 1 | 1.35497566 | -4.96226388 | 0.93384727 |
| 1 | 3.62454796 | -4.15953960 | -0.97301936 |
| 1 | 0.60292612 | -4.22088231 | -1.56918029 |
| 1 | 1.26924943 | -1.96754121 | -2.68615190 |
| 1 | 2.75644711 | -2.03596337 | -1.70810708 |
| 8 | 0.61968812 | -1.04359267 | 1.37758306 |
| 8 | 0.95639862 | -1.69736802 | -0.71249332 |
| 31 | 1.09391058 | 0.28454617 | 0.11392852 |
| 8 | -7.89857346 | -0.92852831 | 4.20305076 |
| 6 | -8.65615094 | -0.70617298 | 5.41643628 |
| 1 | -8.60530475 | -1.58775454 | 6.06775252 |
| 6 | -5.85941201 | -1.36385411 | 3.07424625 |
| 6 | -4.42112512 | -1.85986781 | 3.22169333 |
| 6 | -3.70205626 | -1.98155933 | 1.87413714 |
| 6 | -6.56961807 | -1.17567006 | 4.40074400 |
| 6 | -2.26050513 | -2.48414248 | 2.01260799 |
| 6 | -1.55708165 | -2.57632395 | 0.66270660 |
| 1 | -6.46077466 | -2.04756393 | 2.45489945 |

```
   1    -4.42236685  -2.83524032   3.73305737
   1    -4.26867844  -2.66233124   1.21471444
   1    -2.25316852  -3.47733058   2.49014084
   1    -5.88544660  -0.39338859   2.54989255
   1    -3.86775550  -1.17473607   3.88136338
   1    -3.70128149  -0.99873352   1.37088446
   1    -1.68479986  -1.80894949   2.66363920
   1    -1.51335831  -1.59211826   0.17796445
   1    -2.09759472  -3.26164835  -0.00918930
   8    -6.05409188  -1.22115170   5.50163355
   8    -0.22567233  -3.12860705   0.79479817
   1    -9.68397428  -0.52591974   5.08706579
   1    -8.26427256   0.16249536   5.96054523
Electronic energy = -3908.669991 a.u.
DFT-D3(BJ) dispersion correction = -0.138876 a.u.
Thermal free energy = 0.743039 a.u.
Gibbs free energy = -3908.065828 a.u.
Number of imaginary frequencies = 0.
```

**Int3b CL isomer/conformer 16**

```
Atomic N.      X            Y             Z
   7     0.48574546   1.91899896   2.71727822
   8    -0.02979514   1.43330375  -0.16549664
   8     2.72699766   0.72154663   1.12974593
   6    -0.66219719   1.54591487   3.57921461
   6    -0.97125143   2.39152560   0.00474860
   6    -1.99822318   2.54791830  -0.97503174
   6    -2.96569547   3.53625877  -0.74518041
   6    -2.95154341   4.36460500   0.38024872
   6    -1.92260924   4.23094515   1.30571642
   6    -0.92466739   3.26731047   1.12111600
   6     0.25469776   3.24507706   2.05563577
   1    -3.73047610   5.11427668   0.51920023
   6    -2.03904590   1.68072500  -2.24902439
   6     3.41870933   1.76478250   1.64179343
   6     4.62470167   2.19840176   1.01015694
   6     5.31048882   3.27339993   1.59198251
   6     4.86243996   3.92515959   2.74417501
   6     3.68924204   3.49414272   3.35187029
   6     2.97025740   2.41695093   2.82032355
   6     1.74356620   1.93272409   3.54740926
   1     5.42750853   4.76101726   3.15633574
   6     5.16300470   1.51273951  -0.26086642
   6    -3.21694993   2.05706406  -3.16753327
   6    -2.20307052   0.18952339  -1.87750659
   6    -0.73878149   1.87975105  -3.06424994
   6     6.47132755   2.15588426  -0.75868524
   6     4.13282098   1.63006690  -1.40902238
   6     5.47203547   0.02684830   0.03883484
   1    -0.77970616   2.28649582   4.38794049
   1    -1.57880695   1.52398886   2.97974482
   1     0.12904548   4.01347438   2.83806510
```

| | | | |
|---|---|---|---|
| 1 | 1.18125380 | 3.47905140 | 1.51053055 |
| 1 | -3.76656743 | 3.66874872 | -1.47029118 |
| 1 | -1.87092990 | 4.88976746 | 2.17567168 |
| 1 | 1.87229067 | 0.89687896 | 3.90095706 |
| 1 | 1.56720366 | 2.57091512 | 4.43004867 |
| 1 | 6.23006602 | 3.62521749 | 1.12799318 |
| 1 | 3.32104098 | 3.98548032 | 4.25537749 |
| 1 | -3.19005964 | 1.41751310 | -4.06117245 |
| 1 | -4.18989617 | 1.90141313 | -2.67895564 |
| 1 | -3.16074970 | 3.10182864 | -3.50558766 |
| 1 | -1.35826489 | -0.16498227 | -1.27744669 |
| 1 | -3.12954394 | 0.03585107 | -1.30375413 |
| 1 | -2.26659989 | -0.42120818 | -2.79102884 |
| 1 | -0.63666455 | 2.92888522 | -3.37775725 |
| 1 | 0.14681663 | 1.60394769 | -2.48113975 |
| 1 | -0.76971348 | 1.25737124 | -3.97168818 |
| 1 | 6.80845945 | 1.62568989 | -1.66093682 |
| 1 | 7.27736418 | 2.08589169 | -0.01412441 |
| 1 | 6.33634070 | 3.21323749 | -1.02836630 |
| 1 | 3.17296093 | 1.17563185 | -1.14314469 |
| 1 | 3.95447381 | 2.68584247 | -1.65940000 |
| 1 | 4.51946775 | 1.13067433 | -2.31060986 |
| 1 | 6.26336573 | -0.05593433 | 0.79808997 |
| 1 | 4.58516367 | -0.49973816 | 0.40804492 |
| 1 | 5.82617945 | -0.47548721 | -0.87452510 |
| 1 | -0.48528845 | 0.55148569 | 4.00182846 |
| 6 | 1.91120333 | -2.74950053 | 1.98049290 |
| 6 | 2.24424987 | -4.11492398 | 1.36805450 |
| 6 | 2.75439970 | -4.07517038 | -0.07687478 |
| 6 | 0.65485649 | -2.04332569 | 1.44916835 |
| 6 | 1.83433310 | -3.35836995 | -1.07541984 |
| 6 | 1.78854226 | -1.84202577 | -0.91238570 |
| 1 | 2.75185473 | -2.04493497 | 1.88374417 |
| 1 | 3.01378577 | -4.59136365 | 1.99597914 |
| 1 | 2.91830000 | -5.10906382 | -0.42020568 |
| 1 | 2.21460177 | -3.54035897 | -2.09400586 |
| 1 | 1.72542928 | -2.87006750 | 3.05777959 |
| 1 | 1.35194983 | -4.75443166 | 1.42860135 |
| 1 | 3.74245835 | -3.58328070 | -0.10298658 |
| 1 | 0.81504196 | -3.77259497 | -1.04369552 |
| 1 | 1.41182260 | -1.37037178 | -1.83110213 |
| 1 | 2.79995984 | -1.45109821 | -0.71688246 |
| 8 | 0.28259062 | -0.96411760 | 2.21467661 |
| 8 | 0.89393834 | -1.33959752 | 0.11830310 |
| 31 | 0.86027888 | 0.52800018 | 1.20663078 |
| 8 | -7.18907345 | -2.73049899 | -3.02925435 |
| 6 | -7.48134009 | -2.68790382 | -4.44649220 |
| 1 | -6.88817597 | -1.90620005 | -4.93755365 |
| 6 | -5.67998665 | -3.01811585 | -1.22384319 |
| 6 | -4.24486222 | -3.34457685 | -0.81318801 |
| 6 | -4.06376853 | -3.35445840 | 0.71010773 |
| 6 | -5.88492477 | -3.00135822 | -2.72638441 |
| 6 | -2.63388726 | -3.68261620 | 1.16852837 |

| | | | |
|---|---|---|---|
| 6 | -1.65050174 | -2.54638318 | 0.91517226 |
| 1 | -5.98832206 | -2.03359348 | -0.83488649 |
| 1 | -3.56772286 | -2.61639695 | -1.28157014 |
| 1 | -4.36808398 | -2.37798374 | 1.12638990 |
| 1 | -2.27760061 | -4.58931222 | 0.65278265 |
| 1 | -6.39389495 | -3.73933244 | -0.79336046 |
| 1 | -3.96341738 | -4.32645133 | -1.22472945 |
| 1 | -4.75202726 | -4.09671071 | 1.14687492 |
| 1 | -2.63022474 | -3.90900394 | 2.24577121 |
| 1 | -1.92041204 | -1.65769957 | 1.50578919 |
| 1 | -1.62438587 | -2.26421611 | -0.14848936 |
| 8 | -5.02859098 | -3.19561987 | -3.56819847 |
| 8 | -0.34059419 | -3.01112689 | 1.30853602 |
| 1 | -8.55030007 | -2.46426478 | -4.51497746 |
| 1 | -7.25543798 | -3.65437328 | -4.91444905 |

Electronic energy = -3908.669410 a.u.
DFT-D3(BJ) dispersion correction = -0.139510 a.u.
Thermal free energy = 0.743226 a.u.
Gibbs free energy = -3908.065694 a.u.
Number of imaginary frequencies = 0.

**Int3b CL isomer/conformer 17**

| Atomic N. | X | Y | Z |
|---|---|---|---|
| 6 | -2.79574348 | -0.43538141 | 1.53285615 |
| 6 | -3.67457322 | -1.64124105 | 1.18406180 |
| 6 | -3.69876018 | -2.04680489 | -0.29910213 |
| 6 | -1.31643293 | -0.53145269 | 1.14781054 |
| 6 | -2.38342760 | -2.67954063 | -0.78215211 |
| 6 | -1.28624608 | -1.69115276 | -1.18299369 |
| 1 | -3.17847181 | 0.47246273 | 1.04516566 |
| 1 | -4.70170308 | -1.40398286 | 1.50395302 |
| 1 | -4.50572155 | -2.78330456 | -0.43636109 |
| 1 | -2.57074496 | -3.30087270 | -1.67304634 |
| 1 | -2.82284382 | -0.25688464 | 2.61827463 |
| 1 | -3.35558638 | -2.50947996 | 1.78216105 |
| 1 | -3.96078429 | -1.18004139 | -0.92804576 |
| 1 | -2.00926843 | -3.35641822 | -0.00081477 |
| 1 | -0.30363705 | -2.18763068 | -1.19039264 |
| 1 | -1.47692773 | -1.29683346 | -2.19141375 |
| 8 | -0.61144823 | 0.60035291 | 1.46777809 |
| 8 | -1.21236374 | -0.49425173 | -0.36671373 |
| 7 | 1.08058971 | 2.87502709 | 0.64608385 |
| 8 | -0.91884657 | 2.21311869 | -1.45292910 |
| 8 | 1.54276198 | 0.35722728 | -0.89556167 |
| 6 | 0.47835722 | 3.36256291 | 1.91124151 |
| 6 | -1.20222430 | 3.53515980 | -1.37110780 |
| 6 | -2.42865185 | 4.02816362 | -1.90984004 |
| 6 | -2.68381608 | 5.40104104 | -1.78279149 |
| 6 | -1.79093733 | 6.28541524 | -1.17150169 |
| 6 | -0.58175555 | 5.79945932 | -0.68655789 |
| 6 | -0.27233455 | 4.43911990 | -0.79461871 |
| 6 | 1.09559436 | 3.95915940 | -0.39274193 |

| | | | |
|---|---|---|---|
| 1 | -2.03750852 | 7.34398374 | -1.09000170 |
| 6 | -3.42972161 | 3.09841239 | -2.62390061 |
| 6 | 2.74112427 | 0.93166193 | -1.15131003 |
| 6 | 3.51654512 | 0.49248903 | -2.26669450 |
| 6 | 4.76018537 | 1.10662668 | -2.46965759 |
| 6 | 5.25298548 | 2.11582248 | -1.63760474 |
| 6 | 4.48862213 | 2.53534259 | -0.55513807 |
| 6 | 3.24450522 | 1.94728457 | -0.29820427 |
| 6 | 2.47355932 | 2.37148367 | 0.92370097 |
| 1 | 6.22402101 | 2.56739634 | -1.84018711 |
| 6 | 3.01407070 | -0.61375496 | -3.21534252 |
| 6 | -4.65982349 | 3.86496249 | -3.14510900 |
| 6 | -3.94100948 | 2.01049779 | -1.65198211 |
| 6 | -2.74936530 | 2.43392315 | -3.84481904 |
| 6 | 4.02337307 | -0.91438665 | -4.33962008 |
| 6 | 1.69326564 | -0.17666208 | -3.89200150 |
| 6 | 2.80235835 | -1.92826459 | -2.42778345 |
| 1 | 1.08023275 | 4.19329979 | 2.31610825 |
| 1 | -0.54000358 | 3.71719468 | 1.72036330 |
| 1 | 1.69453034 | 4.80408671 | -0.01110874 |
| 1 | 1.62755209 | 3.54184747 | -1.26051016 |
| 1 | -3.61712752 | 5.80228881 | -2.17349680 |
| 1 | 0.14635672 | 6.47656041 | -0.23386130 |
| 1 | 2.34846362 | 1.53156982 | 1.62635593 |
| 1 | 3.02953853 | 3.16708019 | 1.44845516 |
| 1 | 5.37152946 | 0.79329098 | -3.31390179 |
| 1 | 4.85461887 | 3.32073938 | 0.11003576 |
| 1 | -5.33006735 | 3.15878776 | -3.65577555 |
| 1 | -5.23192790 | 4.33426637 | -2.33154971 |
| 1 | -4.38501469 | 4.64335509 | -3.87137848 |
| 1 | -3.12011126 | 1.37718979 | -1.29915322 |
| 1 | -4.43366693 | 2.46786301 | -0.78101015 |
| 1 | -4.68008646 | 1.37288794 | -2.16162488 |
| 1 | -2.42431480 | 3.19619830 | -4.56773512 |
| 1 | -1.87605945 | 1.84394473 | -3.54537140 |
| 1 | -3.46428846 | 1.76922042 | -4.35385415 |
| 1 | 3.61668578 | -1.70960029 | -4.98060623 |
| 1 | 4.98782458 | -1.26763146 | -3.94710782 |
| 1 | 4.20637788 | -0.03760528 | -4.97740603 |
| 1 | 0.90718461 | 0.02469748 | -3.15688684 |
| 1 | 1.84758310 | 0.73402577 | -4.48861032 |
| 1 | 1.34249322 | -0.96914896 | -4.57067270 |
| 1 | 3.75164453 | -2.27722813 | -1.99590519 |
| 1 | 2.08026035 | -1.79500451 | -1.61508087 |
| 1 | 2.43044912 | -2.71372657 | -3.10337925 |
| 1 | 0.43847244 | 2.53979395 | 2.63221777 |
| 31 | 0.05524595 | 1.25078112 | -0.18193879 |
| 8 | 0.44446601 | -6.95192220 | 6.26324368 |
| 6 | 0.66185846 | -8.38271003 | 6.23099739 |
| 1 | 0.06390301 | -8.84455747 | 5.43517884 |
| 6 | 0.49287819 | -4.81679867 | 5.23344495 |
| 6 | 1.01715896 | -3.99649323 | 4.05499293 |
| 6 | 0.66540595 | -2.51017916 | 4.18600692 |

| | | | |
|---|---|---|---|
| 6 | 0.78119537 | -6.30033965 | 5.11043886 |
| 6 | 1.22572624 | -1.62736893 | 3.06096817 |
| 6 | 0.63380177 | -1.89463820 | 1.67797762 |
| 1 | -0.59988475 | -4.70195628 | 5.33241497 |
| 1 | 0.60556021 | -4.41337913 | 3.12452218 |
| 1 | -0.42980062 | -2.39143483 | 4.22193241 |
| 1 | 2.31811363 | -1.77125616 | 2.98575577 |
| 1 | 0.91226166 | -4.46319693 | 6.18860141 |
| 1 | 2.11001799 | -4.11866705 | 3.98533079 |
| 1 | 1.04837462 | -2.13260467 | 5.14889639 |
| 1 | 1.05649879 | -0.56851331 | 3.30727601 |
| 1 | 0.80331102 | -2.93851701 | 1.37298605 |
| 1 | 1.10434177 | -1.24494539 | 0.92680108 |
| 8 | 1.23892035 | -6.86258300 | 4.13364951 |
| 8 | -0.80739140 | -1.72673293 | 1.66802239 |
| 1 | 0.34585418 | -8.74581533 | 7.21372578 |
| 1 | 1.72181329 | -8.60623008 | 6.05618061 |

Electronic energy = -3908.669435 a.u.
DFT-D3(BJ) dispersion correction = -0.139905 a.u.
Thermal free energy = 0.743676 a.u.
Gibbs free energy = -3908.065664 a.u.
Number of imaginary frequencies = 0.

**TS4 eq CL isomer/conformer 1**

| Atomic N. | X | Y | Z |
|---|---|---|---|
| 7 | 0.49747721 | 2.29965970 | 1.66512296 |
| 8 | -0.34037006 | 1.66994250 | -1.06814194 |
| 8 | 2.71176392 | 1.52754028 | -0.14603100 |
| 6 | -0.52982786 | 1.86093921 | 2.64372705 |
| 6 | -1.37318734 | 2.48449186 | -0.78039072 |
| 6 | -2.58511162 | 2.40141779 | -1.53224152 |
| 6 | -3.61718930 | 3.28662941 | -1.19104249 |
| 6 | -3.50539798 | 4.22290020 | -0.15805423 |
| 6 | -2.32151215 | 4.29238678 | 0.56911298 |
| 6 | -1.25683273 | 3.43993948 | 0.25991544 |
| 6 | 0.05092510 | 3.56985897 | 0.98335843 |
| 1 | -4.33778821 | 4.88860315 | 0.07002693 |
| 6 | -2.75833670 | 1.37040157 | -2.66361640 |
| 6 | 3.29399571 | 2.64631841 | 0.33676070 |
| 6 | 4.35294095 | 3.26644552 | -0.39478229 |
| 6 | 4.94068131 | 4.41020157 | 0.16274799 |
| 6 | 4.52529610 | 4.96021512 | 1.37859197 |
| 6 | 3.48545390 | 4.35514504 | 2.07501074 |
| 6 | 2.87408999 | 3.20016179 | 1.57335419 |
| 6 | 1.80105395 | 2.53238951 | 2.39318941 |
| 1 | 5.00838607 | 5.85585856 | 1.76888833 |
| 6 | 4.83167353 | 2.71021858 | -1.75039972 |
| 6 | -4.13372510 | 1.48556470 | -3.34700822 |
| 6 | -2.64957350 | -0.06096400 | -2.08466672 |
| 6 | -1.68326853 | 1.58592617 | -3.75504349 |
| 6 | 5.98957170 | 3.53781874 | -2.33962560 |
| 6 | 3.67332026 | 2.74810189 | -2.77525728 |

| | | | |
|---|---|---|---|
| 6 | 5.34558999 | 1.26116453 | -1.58145958 |
| 1 | -0.71547827 | 2.66958089 | 3.36951796 |
| 1 | -1.46543982 | 1.63650287 | 2.12023934 |
| 1 | -0.01338440 | 4.36042078 | 1.74976063 |
| 1 | 0.85760443 | 3.84752834 | 0.28986523 |
| 1 | -4.55210187 | 3.24582466 | -1.74754763 |
| 1 | -2.20684544 | 5.02079452 | 1.37542916 |
| 1 | 2.12020603 | 1.54105438 | 2.74638378 |
| 1 | 1.58238821 | 3.15629789 | 3.27601300 |
| 1 | 5.75066483 | 4.90154455 | -0.37321221 |
| 1 | 3.14130947 | 4.76979693 | 3.02536812 |
| 1 | -4.19968544 | 0.73370075 | -4.14656133 |
| 1 | -4.96197018 | 1.29821841 | -2.64819890 |
| 1 | -4.28432802 | 2.47253342 | -3.80776003 |
| 1 | -1.68117442 | -0.22697554 | -1.59898002 |
| 1 | -3.44708670 | -0.23682805 | -1.34697395 |
| 1 | -2.76667442 | -0.80110703 | -2.89124529 |
| 1 | -1.78693538 | 2.58403172 | -4.20528741 |
| 1 | -0.67150440 | 1.49342636 | -3.34620377 |
| 1 | -1.80918370 | 0.84024566 | -4.55511409 |
| 1 | 6.29051717 | 3.09585501 | -3.30024358 |
| 1 | 6.87308271 | 3.53911980 | -1.68489362 |
| 1 | 5.69773754 | 4.58007891 | -2.53326358 |
| 1 | 2.81526099 | 2.15656942 | -2.43841543 |
| 1 | 3.33696527 | 3.78209393 | -2.93957326 |
| 1 | 4.01513481 | 2.34637025 | -3.74129947 |
| 1 | 6.19827456 | 1.23160292 | -0.88721611 |
| 1 | 4.55936650 | 0.60302891 | -1.19682468 |
| 1 | 5.68657417 | 0.87023822 | -2.55230650 |
| 1 | -0.17222713 | 0.96748548 | 3.16205624 |
| 6 | 2.96848403 | -1.90570334 | 1.22323107 |
| 6 | 3.25501734 | -3.05396241 | 0.23871428 |
| 6 | 3.23777615 | -2.72078114 | -1.26657895 |
| 6 | 1.52905745 | -1.61338420 | 1.57111716 |
| 6 | 1.87584782 | -2.51234149 | -1.96015870 |
| 6 | 1.30672851 | -1.09626677 | -1.88279116 |
| 1 | 3.41116665 | -0.95956591 | 0.88401104 |
| 1 | 4.26512036 | -3.42580470 | 0.47273326 |
| 1 | 3.74263679 | -3.56124421 | -1.77005591 |
| 1 | 1.98765679 | -2.75427016 | -3.03078257 |
| 1 | 3.44436104 | -2.14152023 | 2.19348689 |
| 1 | 2.57161692 | -3.89182198 | 0.44233477 |
| 1 | 3.87485152 | -1.83873766 | -1.45096641 |
| 1 | 1.13221292 | -3.22079950 | -1.55792733 |
| 1 | 0.41559796 | -1.02096721 | -2.53348020 |
| 1 | 2.06031127 | -0.38590512 | -2.27445293 |
| 8 | 1.12004525 | -0.49966363 | 1.96009728 |
| 8 | 0.96182145 | -0.79610893 | -0.53478266 |
| 31 | 0.94614223 | 0.94778213 | 0.12197444 |
| 8 | -7.13000808 | -6.10286536 | 2.49016784 |
| 6 | -7.91367794 | -7.32036695 | 2.46000375 |
| 1 | -7.82466993 | -7.80954639 | 1.48192046 |
| 6 | -5.06344740 | -4.95383152 | 2.33182183 |

| | | | |
|---|---|---|---|
| 6 | -3.56232722 | -5.08170186 | 2.07616517 |
| 6 | -2.84159785 | -3.73114024 | 2.14172006 |
| 6 | -5.79352754 | -6.28259961 | 2.27951975 |
| 6 | -1.33678635 | -3.85384006 | 1.87294866 |
| 6 | -0.64414201 | -2.50396434 | 1.94364222 |
| 1 | -5.53648283 | -4.28005569 | 1.59916635 |
| 1 | -3.39991284 | -5.54399030 | 1.09055942 |
| 1 | -3.28924899 | -3.03912908 | 1.40780499 |
| 1 | -1.17077314 | -4.29075433 | 0.87586331 |
| 1 | -5.26202267 | -4.50135717 | 3.31732159 |
| 1 | -3.12595145 | -5.77569965 | 2.81059103 |
| 1 | -3.00377900 | -3.27642982 | 3.13453066 |
| 1 | -0.87966672 | -4.53879002 | 2.60461877 |
| 1 | -0.73420718 | -2.04833992 | 2.94065103 |
| 1 | -1.04046136 | -1.80090565 | 1.19665904 |
| 8 | -5.28489389 | -7.36950622 | 2.08137102 |
| 8 | 0.76948896 | -2.71304009 | 1.64898677 |
| 1 | -8.94515474 | -7.00489493 | 2.64358808 |
| 1 | -7.57436797 | -8.01449575 | 3.23909631 |

Electronic energy = -3908.667779 a.u.
DFT-D3(BJ) dispersion correction = -0.137364 a.u.
Thermal free energy = 0.737684 a.u.
Gibbs free energy = -3908.067459 a.u.
Number of imaginary frequencies = 1.

**TS4 eq CL isomer/conformer 2**

| Atomic N. | X | Y | Z |
|---|---|---|---|
| 6 | -3.34668398 | -1.10362995 | 2.07381797 |
| 6 | -4.17291641 | -2.30265093 | 1.59092987 |
| 6 | -4.45139599 | -2.39635205 | 0.07963928 |
| 6 | -1.86097205 | -1.12704980 | 1.78668511 |
| 6 | -3.25557780 | -2.75333500 | -0.82419425 |
| 6 | -2.40568185 | -1.56219625 | -1.26303923 |
| 1 | -3.73660564 | -0.16051859 | 1.66905808 |
| 1 | -5.14458036 | -2.24894905 | 2.10742378 |
| 1 | -5.22392082 | -3.17075729 | -0.05254879 |
| 1 | -3.62829018 | -3.22972393 | -1.74592412 |
| 1 | -3.42342782 | -1.03335881 | 3.17393494 |
| 1 | -3.69692779 | -3.23391557 | 1.93481696 |
| 1 | -4.90540838 | -1.45391130 | -0.27150717 |
| 1 | -2.61848116 | -3.50043058 | -0.32366574 |
| 1 | -1.58423865 | -1.91342485 | -1.91409886 |
| 1 | -3.03119516 | -0.86654770 | -1.85299444 |
| 8 | -1.14446688 | -0.06180171 | 1.93363428 |
| 8 | -1.87086642 | -0.87353987 | -0.14014797 |
| 7 | 0.26095754 | 2.27954602 | 1.05466366 |
| 8 | -1.86840236 | 1.85902882 | -0.92388672 |
| 8 | 0.75158000 | -0.00654190 | -0.74256355 |
| 6 | -0.35659835 | 2.74915957 | 2.31877851 |
| 6 | -2.03298831 | 3.19262767 | -0.79828233 |
| 6 | -3.25921631 | 3.79336286 | -1.21927476 |
| 6 | -3.38768721 | 5.18039989 | -1.06387019 |

| | | | |
|---|---|---|---|
| 6 | -2.37556052 | 5.97971201 | -0.52472085 |
| 6 | -1.18057692 | 5.38607550 | -0.13314714 |
| 6 | -0.99589014 | 4.00665188 | -0.27594781 |
| 6 | 0.33835527 | 3.40024757 | 0.05795246 |
| 1 | -2.52340913 | 7.05441809 | -0.41889083 |
| 6 | -4.39796638 | 2.95410490 | -1.83115804 |
| 6 | 1.98487246 | 0.52619344 | -0.84386420 |
| 6 | 2.81721210 | 0.17833115 | -1.95217288 |
| 6 | 4.10048151 | 0.73983347 | -1.99836433 |
| 6 | 4.57888317 | 1.61821222 | -1.02147985 |
| 6 | 3.75668645 | 1.95588398 | 0.04771889 |
| 6 | 2.47131348 | 1.41178131 | 0.15141742 |
| 6 | 1.63378811 | 1.73941147 | 1.35907221 |
| 1 | 5.58207273 | 2.03619576 | -1.10427344 |
| 6 | 2.32354188 | -0.77029634 | -3.06261992 |
| 6 | -5.61230516 | 3.81883931 | -2.21791029 |
| 6 | -4.89072084 | 1.89994478 | -0.81212902 |
| 6 | -3.90250897 | 2.25409079 | -3.11907578 |
| 6 | 3.39759374 | -1.00537622 | -4.14150333 |
| 6 | 1.08881211 | -0.16120143 | -3.76857591 |
| 6 | 1.96312869 | -2.15232849 | -2.46744156 |
| 1 | 0.22690241 | 3.59033990 | 2.72898388 |
| 1 | -1.38086200 | 3.08667612 | 2.12891459 |
| 1 | 1.01481211 | 4.17662382 | 0.45519143 |
| 1 | 0.81344938 | 2.98227549 | -0.84191412 |
| 1 | -4.31394243 | 5.66259050 | -1.37132800 |
| 1 | -0.36889037 | 5.99205017 | 0.27620453 |
| 1 | 1.46748996 | 0.84797657 | 1.98483741 |
| 1 | 2.16587043 | 2.48431778 | 1.97527552 |
| 1 | 4.75578022 | 0.49260941 | -2.83171725 |
| 1 | 4.10902739 | 2.64159369 | 0.82170779 |
| 1 | -6.38729239 | 3.17213273 | -2.65393376 |
| 1 | -6.05490732 | 4.32536983 | -1.34789741 |
| 1 | -5.35550594 | 4.57913637 | -2.96948552 |
| 1 | -4.08364201 | 1.21541297 | -0.52897584 |
| 1 | -5.27293777 | 2.38879681 | 0.09639717 |
| 1 | -5.71180248 | 1.31157410 | -1.25022221 |
| 1 | -3.60415792 | 2.99882531 | -3.87125802 |
| 1 | -3.04444098 | 1.60364795 | -2.91688514 |
| 1 | -4.71248150 | 1.64508379 | -3.54958200 |
| 1 | 2.99526167 | -1.68819332 | -4.90347862 |
| 1 | 4.30548859 | -1.46831942 | -3.72819757 |
| 1 | 3.68339419 | -0.07397138 | -4.65109205 |
| 1 | 0.26333454 | 0.00395836 | -3.06795263 |
| 1 | 1.34607935 | 0.80136788 | -4.23388147 |
| 1 | 0.74055952 | -0.83846074 | -4.56342936 |
| 1 | 2.84543514 | -2.61176538 | -1.99710166 |
| 1 | 1.16921842 | -2.06709790 | -1.71740735 |
| 1 | 1.61918664 | -2.82519674 | -3.26787806 |
| 1 | -0.37627998 | 1.92421842 | 3.03697610 |
| 31 | -0.78306818 | 0.70423347 | 0.09218367 |
| 8 | 6.61850357 | -3.51084447 | 3.43332267 |
| 6 | 7.58572578 | -2.87157679 | 4.30058384 |

| | | | |
|---|---|---|---|
| 1 | 7.60419941 | -1.78904164 | 4.12246656 |
| 6 | 4.40531397 | -3.78942394 | 2.63292837 |
| 6 | 2.93236065 | -3.44727492 | 2.85413361 |
| 6 | 2.01919365 | -4.13700819 | 1.83288026 |
| 6 | 5.33420992 | -3.07980967 | 3.59922576 |
| 6 | 0.51842511 | -3.89152241 | 2.05672693 |
| 6 | 0.13516584 | -2.43037534 | 1.88133299 |
| 1 | 4.72117472 | -3.51813626 | 1.61176944 |
| 1 | 2.81619835 | -2.35506463 | 2.80715394 |
| 1 | 2.29295254 | -3.81065679 | 0.81494743 |
| 1 | 0.22826928 | -4.22831774 | 3.06507468 |
| 1 | 4.58452368 | -4.87309933 | 2.71792793 |
| 1 | 2.63906956 | -3.73997068 | 3.87499475 |
| 1 | 2.19916248 | -5.22404575 | 1.86423039 |
| 1 | -0.05963847 | -4.49336815 | 1.33884692 |
| 1 | 0.42804480 | -2.05097461 | 0.89032835 |
| 1 | 0.58304846 | -1.78739142 | 2.65042639 |
| 8 | 5.00930738 | -2.23683095 | 4.41415071 |
| 8 | -1.31687641 | -2.33935380 | 1.99690771 |
| 1 | 8.54837799 | -3.32336330 | 4.04283524 |
| 1 | 7.33839655 | -3.05656934 | 5.35348129 |

Electronic energy = -3908.665834 a.u.
DFT-D3(BJ) dispersion correction = -0.139421 a.u.
Thermal free energy = 0.739193 a.u.
Gibbs free energy = -3908.066062 a.u.
Number of imaginary frequencies = 1.

**TS4 eq CL isomer/conformer 3**

| Atomic N. | X | Y | Z |
|---|---|---|---|
| 6 | -3.81208420 | 0.06888643 | 1.12920725 |
| 6 | -4.62978935 | -1.04973233 | 0.47064978 |
| 6 | -4.43501282 | -1.26047862 | -1.04188156 |
| 6 | -2.33655190 | -0.18930487 | 1.34555209 |
| 6 | -3.08995223 | -1.86346662 | -1.49056768 |
| 6 | -1.96660769 | -0.84807599 | -1.69178081 |
| 1 | -3.89502096 | 1.00881934 | 0.56789583 |
| 1 | -5.69178772 | -0.81126189 | 0.64097339 |
| 1 | -5.24032354 | -1.93535662 | -1.37345254 |
| 1 | -3.22792196 | -2.37434864 | -2.45771050 |
| 1 | -4.21783495 | 0.26474547 | 2.13827419 |
| 1 | -4.44198036 | -1.99657750 | 0.99993002 |
| 1 | -4.60240793 | -0.30749866 | -1.57236791 |
| 1 | -2.76872516 | -2.63646722 | -0.77369094 |
| 1 | -1.05331600 | -1.36961973 | -2.03226852 |
| 1 | -2.26092982 | -0.13112241 | -2.48064017 |
| 8 | -1.53701365 | 0.77502036 | 1.66265190 |
| 8 | -1.69503796 | -0.13890533 | -0.48952898 |
| 7 | 0.38764140 | 2.84053397 | 1.15175879 |
| 8 | -1.04416311 | 2.47961664 | -1.38454640 |
| 8 | 1.08112216 | 0.33613470 | -0.24297975 |
| 6 | -0.52486712 | 3.50992274 | 2.11012459 |
| 6 | -1.03668380 | 3.82928491 | -1.40007627 |

| | | | |
|---|---|---|---|
| 6 | -1.96193576 | 4.52918339 | -2.23447251 |
| 6 | -1.92234933 | 5.93008137 | -2.21272254 |
| 6 | -1.02403021 | 6.65102148 | -1.42079628 |
| 6 | -0.11772078 | 5.95913935 | -0.62493581 |
| 6 | -0.10763910 | 4.56014347 | -0.61670351 |
| 6 | 0.94482881 | 3.83322716 | 0.17242897 |
| 1 | -1.03394473 | 7.74086666 | -1.43376076 |
| 6 | -2.96217585 | 3.77790475 | -3.13471866 |
| 6 | 2.34689760 | 0.70218247 | 0.03746147 |
| 6 | 3.42791915 | 0.14044009 | -0.70929414 |
| 6 | 4.72693729 | 0.53720170 | -0.36411536 |
| 6 | 4.99332809 | 1.45207739 | 0.65905982 |
| 6 | 3.93278861 | 1.99658537 | 1.37453735 |
| 6 | 2.61597705 | 1.62323618 | 1.08181345 |
| 6 | 1.49498701 | 2.17251778 | 1.92362261 |
| 1 | 6.02071095 | 1.73658109 | 0.88597155 |
| 6 | 3.17936492 | -0.85909808 | -1.85642898 |
| 6 | -3.84553933 | 4.74064207 | -3.95007539 |
| 6 | -3.90612698 | 2.90809989 | -2.27186394 |
| 6 | -2.19727564 | 2.88907623 | -4.14423084 |
| 6 | 4.49422646 | -1.33825099 | -2.50012398 |
| 6 | 2.33954978 | -0.18944564 | -2.97002459 |
| 6 | 2.44657016 | -2.11390305 | -1.32455635 |
| 1 | 0.01477585 | 4.31154156 | 2.64181137 |
| 1 | -1.37161660 | 3.94894099 | 1.57214832 |
| 1 | 1.56810808 | 4.55820751 | 0.72391921 |
| 1 | 1.61327219 | 3.26866674 | -0.49437058 |
| 1 | -2.62029409 | 6.48755407 | -2.83484530 |
| 1 | 0.60488302 | 6.50002193 | -0.00937978 |
| 1 | 1.00626564 | 1.37663162 | 2.50826073 |
| 1 | 1.90663588 | 2.90727353 | 2.63691139 |
| 1 | 5.56955767 | 0.12555477 | -0.91684443 |
| 1 | 4.11745119 | 2.71448350 | 2.17711926 |
| 1 | -4.53120995 | 4.15239906 | -4.57677174 |
| 1 | -4.45869160 | 5.38755035 | -3.30592585 |
| 1 | -3.25174952 | 5.37910700 | -4.61986589 |
| 1 | -3.34370589 | 2.16401768 | -1.69732487 |
| 1 | -4.47803783 | 3.53511119 | -1.57168424 |
| 1 | -4.62500143 | 2.38141537 | -2.91841912 |
| 1 | -1.56864834 | 3.50727630 | -4.80150652 |
| 1 | -1.55456054 | 2.16373968 | -3.63329434 |
| 1 | -2.91202402 | 2.34154391 | -4.77789307 |
| 1 | 4.25961208 | -2.04856396 | -3.30577636 |
| 1 | 5.14294863 | -1.85717487 | -1.77952456 |
| 1 | 5.06300116 | -0.50972158 | -2.94613314 |
| 1 | 1.36766362 | 0.14882354 | -2.59506154 |
| 1 | 2.87165451 | 0.67841148 | -3.38588691 |
| 1 | 2.16695237 | -0.90430290 | -3.78910828 |
| 1 | 3.05285692 | -2.61824560 | -0.55727792 |
| 1 | 1.47552669 | -1.85280132 | -0.88983893 |
| 1 | 2.28263760 | -2.82674861 | -2.14717817 |
| 1 | -0.89681929 | 2.77172422 | 2.82677388 |
| 31 | -0.51615286 | 1.30980694 | -0.00643861 |

| | | | |
|---|---|---|---|
| 8 | 3.70736957 | -7.37049294 | 4.17889500 |
| 6 | 3.92362165 | -8.76612568 | 4.49805212 |
| 1 | 3.31098628 | -9.06253242 | 5.35877991 |
| 6 | 2.29668117 | -5.57689667 | 3.53851295 |
| 6 | 0.87882823 | -5.15798950 | 3.15203738 |
| 6 | 0.77718127 | -3.66038418 | 2.84484863 |
| 6 | 2.41940260 | -7.05542231 | 3.85341287 |
| 6 | -0.64227039 | -3.23475146 | 2.45061159 |
| 6 | -0.71607149 | -1.74771559 | 2.14809442 |
| 1 | 2.65095234 | -5.01607895 | 4.41896248 |
| 1 | 0.18673490 | -5.42226839 | 3.96619916 |
| 1 | 1.10365999 | -3.08215141 | 3.72669411 |
| 1 | -1.34659243 | -3.47898221 | 3.26190233 |
| 1 | 3.01340532 | -5.34263897 | 2.73472834 |
| 1 | 0.55341798 | -5.74201584 | 2.27761698 |
| 1 | 1.47611415 | -3.40258384 | 2.03102493 |
| 1 | -0.96757245 | -3.79997945 | 1.56296313 |
| 1 | -0.06378024 | -1.46896362 | 1.30727518 |
| 1 | -0.43915886 | -1.14095080 | 3.02289653 |
| 8 | 1.51550293 | -7.86903763 | 3.82892036 |
| 8 | -2.09443521 | -1.43695831 | 1.78498280 |
| 1 | 4.98904228 | -8.84724808 | 4.73376179 |
| 1 | 3.66558862 | -9.40033245 | 3.64063573 |

Electronic energy = -3908.666678 a.u.
DFT-D3(BJ) dispersion correction = -0.138348 a.u.
Thermal free energy = 0.739022 a.u.
Gibbs free energy = -3908.066004 a.u.
Number of imaginary frequencies = 1.

**TS4 eq CL isomer/conformer 4**

| Atomic N. | X | Y | Z |
|---|---|---|---|
| 7 | 0.67181331 | 2.27956605 | 2.05240250 |
| 8 | 0.01993084 | 1.58538890 | -0.72221905 |
| 8 | 2.93072581 | 1.10610187 | 0.52041984 |
| 6 | -0.48297766 | 1.99053085 | 2.93661642 |
| 6 | -0.90821338 | 2.55373859 | -0.58255982 |
| 6 | -2.01567411 | 2.60410213 | -1.48362613 |
| 6 | -2.95857143 | 3.62357879 | -1.29206395 |
| 6 | -2.84944630 | 4.57565641 | -0.27401409 |
| 6 | -1.75551701 | 4.52868986 | 0.58378887 |
| 6 | -0.78073275 | 3.53710318 | 0.43121490 |
| 6 | 0.44779423 | 3.55770826 | 1.29702640 |
| 1 | -3.61045504 | 5.34799194 | -0.16243929 |
| 6 | -2.16316509 | 1.58802223 | -2.63352537 |
| 6 | 3.60764265 | 2.16571903 | 1.01144552 |
| 6 | 4.81211472 | 2.59744191 | 0.37487784 |
| 6 | 5.48713589 | 3.68878293 | 0.93849832 |
| 6 | 5.02691317 | 4.36123419 | 2.07407808 |
| 6 | 3.85111284 | 3.93624115 | 2.68165970 |
| 6 | 3.14475489 | 2.84179401 | 2.16942096 |
| 6 | 1.91688740 | 2.36238599 | 2.89636660 |
| 1 | 5.58330584 | 5.20965528 | 2.47221637 |

| | | | |
|---|---|---|---|
| 6 | 5.35301256 | 1.89867187 | -0.88794994 |
| 6 | -3.42542815 | 1.84842443 | -3.47671270 |
| 6 | -2.27326155 | 0.15064581 | -2.07199669 |
| 6 | -0.94502819 | 1.68757808 | -3.58277559 |
| 6 | 6.66921043 | 2.52861905 | -1.38152683 |
| 6 | 4.32691193 | 2.02206039 | -2.03918791 |
| 6 | 5.64487267 | 0.40955150 | -0.58754790 |
| 1 | -0.63316536 | 2.82700729 | 3.63977814 |
| 1 | -1.38962972 | 1.86743212 | 2.33461165 |
| 1 | 0.38506284 | 4.38783169 | 2.02177310 |
| 1 | 1.34999406 | 3.71994233 | 0.68906212 |
| 1 | -3.81631041 | 3.68227363 | -1.95989120 |
| 1 | -1.63859558 | 5.27374363 | 1.37431693 |
| 1 | 2.06316400 | 1.34878671 | 3.30261207 |
| 1 | 1.71185040 | 3.03851867 | 3.74414921 |
| 1 | 6.40704536 | 4.03771591 | 0.47285378 |
| 1 | 3.47172713 | 4.44614697 | 3.57021761 |
| 1 | -3.47622633 | 1.10362732 | -4.28389454 |
| 1 | -4.34684038 | 1.75738156 | -2.88313150 |
| 1 | -3.41125584 | 2.84284544 | -3.94541121 |
| 1 | -1.37309277 | -0.12778068 | -1.51242316 |
| 1 | -3.14632869 | 0.06251854 | -1.40750217 |
| 1 | -2.40513325 | -0.56382388 | -2.89929581 |
| 1 | -0.88587403 | 2.69053864 | -4.02980137 |
| 1 | -0.00743264 | 1.48794222 | -3.05233622 |
| 1 | -1.04817927 | 0.95836216 | -4.40118170 |
| 1 | 7.00857735 | 1.98961401 | -2.27770495 |
| 1 | 7.46915627 | 2.45809364 | -0.63031274 |
| 1 | 6.54444838 | 3.58504772 | -1.65957236 |
| 1 | 3.36182594 | 1.57867765 | -1.77188027 |
| 1 | 4.16106844 | 3.07851553 | -2.29544187 |
| 1 | 4.70798635 | 1.51242042 | -2.93740582 |
| 1 | 6.41648245 | 0.31661516 | 0.19084045 |
| 1 | 4.74370384 | -0.11125898 | -0.24681935 |
| 1 | 6.01844692 | -0.08946481 | -1.49490047 |
| 1 | -0.28778347 | 1.06832767 | 3.49179959 |
| 6 | 2.32596135 | -2.30339742 | 1.77176380 |
| 6 | 2.69657850 | -3.57616138 | 1.00040388 |
| 6 | 3.09492970 | -3.39237595 | -0.47275245 |
| 6 | 0.94207507 | -1.73349559 | 1.53864312 |
| 6 | 1.99962425 | -2.91055870 | -1.44067240 |
| 6 | 1.73431647 | -1.40646255 | -1.43008685 |
| 1 | 3.04649472 | -1.49391353 | 1.58884776 |
| 1 | 3.55149293 | -4.03529882 | 1.52160120 |
| 1 | 3.46501184 | -4.36691141 | -0.83022916 |
| 1 | 2.30739737 | -3.16279769 | -2.46908689 |
| 1 | 2.35784292 | -2.51325679 | 2.85607862 |
| 1 | 1.87039006 | -4.29944754 | 1.07370770 |
| 1 | 3.95430088 | -2.70244980 | -0.53496081 |
| 1 | 1.05801284 | -3.45362496 | -1.25676680 |
| 1 | 1.08369124 | -1.14376056 | -2.28342938 |
| 1 | 2.69128013 | -0.86889672 | -1.55784106 |
| 8 | 0.61597508 | -0.57870024 | 2.02955484 |

| | | | |
|---|---|---|---|
| 8 | 1.08519316 | -0.97713727 | -0.23242959 |
| 31 | 1.07906294 | 0.77676082 | 0.61064810 |
| 8 | -8.29758167 | -4.72142887 | 0.19251904 |
| 6 | -9.23856544 | -5.79410839 | -0.05387581 |
| 1 | -8.98169231 | -6.32480431 | -0.97922570 |
| 6 | -6.09827709 | -3.93325400 | 0.59089786 |
| 6 | -4.62212276 | -4.30991936 | 0.71037692 |
| 6 | -3.72953129 | -3.09437609 | 0.98116118 |
| 6 | -7.00084496 | -5.12464428 | 0.33255383 |
| 6 | -2.24540019 | -3.46513510 | 1.08679891 |
| 6 | -1.38008165 | -2.24534750 | 1.35499203 |
| 1 | -6.25955391 | -3.20470357 | -0.22001284 |
| 1 | -4.29941034 | -4.81131411 | -0.21495669 |
| 1 | -3.86488247 | -2.35163331 | 0.17630647 |
| 1 | -1.91397309 | -3.94429302 | 0.15211064 |
| 1 | -6.45642900 | -3.43563890 | 1.50725794 |
| 1 | -4.49982357 | -5.05204773 | 1.51395822 |
| 1 | -4.05406618 | -2.60126638 | 1.91399646 |
| 1 | -2.09899855 | -4.20104933 | 1.89355874 |
| 1 | -1.64763212 | -1.75650227 | 2.30294728 |
| 1 | -1.45890558 | -1.50718892 | 0.54382688 |
| 8 | -6.64738369 | -6.28609133 | 0.25800407 |
| 8 | 0.00426457 | -2.69290733 | 1.43249309 |
| 1 | -10.21457386 | -5.30772352 | -0.14366049 |
| 1 | -9.23452854 | -6.50731945 | 0.77998346 |

Electronic energy = -3908.667367 a.u.
DFT-D3(BJ) dispersion correction = -0.137982 a.u.
Thermal free energy = 0.739369 a.u.
Gibbs free energy = -3908.065980 a.u.
Number of imaginary frequencies = 1.

**TS4_eq_CL isomer/conformer 5**

| Atomic N. | X | Y | Z |
|---|---|---|---|
| 6 | -3.25398207 | -0.55644190 | 2.36037445 |
| 6 | -4.26483965 | -1.54637909 | 1.76723921 |
| 6 | -4.55824327 | -1.42713547 | 0.26083189 |
| 6 | -1.79090726 | -0.79613888 | 2.05556417 |
| 6 | -3.43538213 | -1.85781169 | -0.70211416 |
| 6 | -2.40535331 | -0.77386874 | -1.01389790 |
| 1 | -3.48061037 | 0.47513786 | 2.06059527 |
| 1 | -5.21409988 | -1.39425683 | 2.30528903 |
| 1 | -5.44128799 | -2.05471277 | 0.05972436 |
| 1 | -3.88133478 | -2.15298462 | -1.66624165 |
| 1 | -3.32326794 | -0.58896917 | 3.46282578 |
| 1 | -3.94520307 | -2.57403779 | 1.99902773 |
| 1 | -4.86313009 | -0.39316261 | 0.02501457 |
| 1 | -2.92585063 | -2.75230718 | -0.30814710 |
| 1 | -1.66486895 | -1.16451418 | -1.73560977 |
| 1 | -2.91496062 | 0.08796882 | -1.48360693 |
| 8 | -0.90597850 | 0.11031948 | 2.30920911 |
| 8 | -1.73994815 | -0.34188229 | 0.16621318 |
| 7 | 0.82378167 | 2.31384563 | 1.67989159 |

| | | | |
|---|---|---|---|
| 8 | -1.35022461 | 2.43994784 | -0.28981477 |
| 8 | 0.97420299 | 0.20119067 | -0.36993548 |
| 6 | 0.28014922 | 2.72210574 | 2.99780369 |
| 6 | -1.31054676 | 3.76128888 | -0.01640657 |
| 6 | -2.43224502 | 4.58266020 | -0.34717482 |
| 6 | -2.34980345 | 5.94712067 | -0.03716248 |
| 6 | -1.22834408 | 6.52060366 | 0.56878388 |
| 6 | -0.13616012 | 5.71465015 | 0.87049061 |
| 6 | -0.16190389 | 4.34774065 | 0.57336050 |
| 6 | 1.06698000 | 3.51649952 | 0.81424618 |
| 1 | -1.21225452 | 7.58669329 | 0.79510421 |
| 6 | -3.68471718 | 3.99876189 | -1.02967882 |
| 6 | 2.27314878 | 0.55414379 | -0.42985731 |
| 6 | 3.04613256 | 0.21254152 | -1.58168411 |
| 6 | 4.39795542 | 0.58198524 | -1.58574867 |
| 6 | 4.99913740 | 1.26749825 | -0.52588886 |
| 6 | 4.23489046 | 1.60008943 | 0.58689600 |
| 6 | 2.88349366 | 1.24134135 | 0.64982277 |
| 6 | 2.10169911 | 1.54704928 | 1.89962018 |
| 1 | 6.05339384 | 1.53904366 | -0.57685030 |
| 6 | 2.41848898 | -0.52692503 | -2.77999830 |
| 6 | -4.75316334 | 5.07459593 | -1.29989648 |
| 6 | -4.33309937 | 2.92506433 | -0.12485245 |
| 6 | -3.29975557 | 3.37993383 | -2.39463830 |
| 6 | 3.44884372 | -0.80509549 | -3.89070368 |
| 6 | 1.29256511 | 0.33302805 | -3.40167379 |
| 6 | 1.85068071 | -1.89397693 | -2.32954383 |
| 1 | 0.98071492 | 3.41600299 | 3.49192691 |
| 1 | -0.68245608 | 3.22636914 | 2.86268330 |
| 1 | 1.85154915 | 4.13424063 | 1.28433788 |
| 1 | 1.47486579 | 3.13764191 | -0.13459235 |
| 1 | -3.19290066 | 6.59392071 | -0.27334902 |
| 1 | 0.75835747 | 6.14257193 | 1.32908416 |
| 1 | 1.80376875 | 0.62329149 | 2.42119026 |
| 1 | 2.73666692 | 2.13031387 | 2.58851123 |
| 1 | 5.01138735 | 0.33419240 | -2.45019817 |
| 1 | 4.68413448 | 2.13555169 | 1.42652035 |
| 1 | -5.61662626 | 4.60493231 | -1.79264355 |
| 1 | -5.11519718 | 5.54211330 | -0.37268308 |
| 1 | -4.38277006 | 5.86624002 | -1.96692443 |
| 1 | -3.63874602 | 2.10048366 | 0.06999625 |
| 1 | -4.63891506 | 3.36283922 | 0.83707088 |
| 1 | -5.23260593 | 2.51821637 | -0.61231261 |
| 1 | -2.89284372 | 4.15082788 | -3.06510448 |
| 1 | -2.54886150 | 2.59055591 | -2.27949786 |
| 1 | -4.19180584 | 2.95027304 | -2.87618494 |
| 1 | 2.95125723 | -1.33529842 | -4.71540642 |
| 1 | 4.27549934 | -1.43930268 | -3.53847289 |
| 1 | 3.87384367 | 0.12117502 | -4.30324078 |
| 1 | 0.49995384 | 0.54574329 | -2.67626715 |
| 1 | 1.69419754 | 1.28944063 | -3.76659632 |
| 1 | 0.84749246 | -0.19566651 | -4.25855827 |
| 1 | 2.65039325 | -2.52639103 | -1.91600013 |

| | | | |
|---|---|---|---|
| 1 | 1.07379055 | -1.77088702 | -1.56724811 |
| 1 | 1.41531909 | -2.41980219 | -3.19324303 |
| 1 | 0.13698094 | 1.83239996 | 3.61814404 |
| 31 | -0.43964520 | 1.02846718 | 0.56172764 |
| 8 | 3.40500736 | -6.04834938 | -0.51002204 |
| 6 | 4.20660877 | -5.61957979 | -1.63733482 |
| 1 | 4.48905182 | -4.56532812 | -1.52698469 |
| 6 | 3.12502933 | -6.40990353 | 1.82879400 |
| 6 | 1.67052495 | -5.91672897 | 1.78242755 |
| 6 | 1.53666401 | -4.39377069 | 1.89303982 |
| 6 | 4.01616049 | -5.91405201 | 0.70354533 |
| 6 | 0.07341386 | -3.93827009 | 1.94431806 |
| 6 | -0.04396342 | -2.42432451 | 2.00337434 |
| 1 | 3.13873839 | -7.51150799 | 1.78751838 |
| 1 | 1.12220454 | -6.39192867 | 2.61221457 |
| 1 | 2.06374693 | -4.04457712 | 2.79736233 |
| 1 | -0.42334163 | -4.37483501 | 2.82586908 |
| 1 | 3.61468744 | -6.11359787 | 2.76595426 |
| 1 | 1.19595206 | -6.26269579 | 0.85214615 |
| 1 | 2.04040098 | -3.91691232 | 1.03589404 |
| 1 | -0.46512344 | -4.30726099 | 1.05700910 |
| 1 | 0.34672490 | -1.94788623 | 1.09211910 |
| 1 | 0.48799375 | -2.00970888 | 2.87246203 |
| 8 | 5.14189959 | -5.47448492 | 0.84103227 |
| 8 | -1.46036649 | -2.09798765 | 2.12816334 |
| 1 | 3.57014728 | -5.76136589 | -2.51602507 |
| 1 | 5.11696243 | -6.22765017 | -1.71234822 |

Electronic energy = -3908.665147 a.u.
DFT-D3(BJ) dispersion correction = -0.139832 a.u.
Thermal free energy = 0.739000 a.u.
Gibbs free energy = -3908.065979 a.u.
Number of imaginary frequencies = 1.

**TS4_eq_CL isomer/conformer 6**

| Atomic N. | X | Y | Z |
|---|---|---|---|
| 6 | -2.74036980 | -0.33484378 | 2.77826095 |
| 6 | -3.63943100 | -1.51207304 | 2.37807250 |
| 6 | -3.89258027 | -1.71847057 | 0.87364393 |
| 6 | -1.24887824 | -0.49890667 | 2.58468461 |
| 6 | -2.71020031 | -2.23236609 | 0.03094870 |
| 6 | -1.75100565 | -1.15473354 | -0.46878484 |
| 1 | -3.03278232 | 0.58636445 | 2.25727272 |
| 1 | -4.61564016 | -1.34583402 | 2.86095405 |
| 1 | -4.71487713 | -2.44728875 | 0.78897792 |
| 1 | -3.09878826 | -2.74386287 | -0.86437702 |
| 1 | -2.86402917 | -0.13890445 | 3.85909796 |
| 1 | -3.23909545 | -2.44003057 | 2.81507778 |
| 1 | -4.27476263 | -0.78200108 | 0.43286896 |
| 1 | -2.14876342 | -2.99191546 | 0.59898800 |
| 1 | -0.97798342 | -1.62115538 | -1.10508120 |
| 1 | -2.30994868 | -0.42958713 | -1.08827949 |
| 8 | -0.45831171 | 0.51644993 | 2.64497423 |

| | | | |
|---|---|---|---|
| 8 | -1.12946308 | -0.47510552 | 0.61795038 |
| 7 | 1.01478028 | 2.73535681 | 1.60438693 |
| 8 | -1.06958270 | 2.19752979 | -0.38954362 |
| 8 | 1.52167547 | 0.30424288 | 0.02965253 |
| 6 | 0.35639659 | 3.34026480 | 2.78707027 |
| 6 | -1.17714131 | 3.54294443 | -0.41218343 |
| 6 | -2.35902405 | 4.14612007 | -0.94443685 |
| 6 | -2.43251729 | 5.54577684 | -0.93749297 |
| 6 | -1.40803158 | 6.35681152 | -0.44148520 |
| 6 | -0.25562677 | 5.75995874 | 0.05719467 |
| 6 | -0.12577675 | 4.36688948 | 0.06471844 |
| 6 | 1.17101800 | 3.75231075 | 0.51126504 |
| 1 | -1.51274705 | 7.44169426 | -0.45276061 |
| 6 | -3.51024795 | 3.29648829 | -1.51697183 |
| 6 | 2.77054119 | 0.80089891 | -0.06027053 |
| 6 | 3.63636351 | 0.35834518 | -1.10791230 |
| 6 | 4.93481874 | 0.88469803 | -1.13853538 |
| 6 | 5.39769268 | 1.81756914 | -0.20549558 |
| 6 | 4.54161453 | 2.25136948 | 0.80039966 |
| 6 | 3.23917055 | 1.74631560 | 0.88724405 |
| 6 | 2.35563445 | 2.19049072 | 2.02208281 |
| 1 | 6.41456842 | 2.20382810 | -0.27467048 |
| 6 | 3.16143513 | -0.65057582 | -2.17198515 |
| 6 | -4.66639233 | 4.16554976 | -2.04661465 |
| 6 | -4.09278584 | 2.38053012 | -0.41565907 |
| 6 | -2.99679446 | 2.44388890 | -2.70162058 |
| 6 | 4.26841164 | -0.98065245 | -3.19098520 |
| 6 | 1.96905410 | -0.06256617 | -2.96370077 |
| 6 | 2.74414897 | -1.98029315 | -1.50122619 |
| 1 | 0.94382477 | 4.20449352 | 3.14014912 |
| 1 | -0.64924175 | 3.68103886 | 2.51917243 |
| 1 | 1.85842240 | 4.54165745 | 0.86149096 |
| 1 | 1.66675329 | 3.23625565 | -0.32416442 |
| 1 | -3.32451367 | 6.02907848 | -1.33202982 |
| 1 | 0.56665504 | 6.37231207 | 0.43469647 |
| 1 | 2.13336587 | 1.35835922 | 2.70924282 |
| 1 | 2.87778640 | 2.97207093 | 2.60057378 |
| 1 | 5.61579657 | 0.56429678 | -1.92507827 |
| 1 | 4.87895107 | 2.98525667 | 1.53614771 |
| 1 | -5.45155716 | 3.50896668 | -2.44832015 |
| 1 | -5.12097263 | 4.77913618 | -1.25523889 |
| 1 | -4.34385490 | 4.83065081 | -2.86051393 |
| 1 | -3.32881689 | 1.70060563 | -0.02344911 |
| 1 | -4.49070120 | 2.98078084 | 0.41613057 |
| 1 | -4.91941404 | 1.77994788 | -0.82568419 |
| 1 | -2.63712049 | 3.09225893 | -3.51375699 |
| 1 | -2.17635727 | 1.78506255 | -2.39683819 |
| 1 | -3.81514406 | 1.82503295 | -3.10082889 |
| 1 | 3.87670493 | -1.70467067 | -3.91982508 |
| 1 | 5.14894867 | -1.43447316 | -2.71318388 |
| 1 | 4.59539843 | -0.09256629 | -3.75096393 |
| 1 | 1.12130189 | 0.16344240 | -2.30825710 |
| 1 | 2.26610589 | 0.86225599 | -3.47948503 |

| | | | |
|---|---|---|---|
| 1 | 1.63448703 | -0.78383756 | -3.72456193 |
| 1 | 3.60157943 | -2.43656707 | -0.98393226 |
| 1 | 1.93896282 | -1.82186377 | -0.77654576 |
| 1 | 2.38618898 | -2.68830562 | -2.26394939 |
| 1 | 0.28352827 | 2.59024262 | 3.58012652 |
| 31 | -0.03183691 | 1.10098422 | 0.73947644 |
| 8 | 1.44147646 | -6.52945185 | -1.50572538 |
| 6 | 1.86481094 | -6.48038387 | -2.89035392 |
| 1 | 1.00923145 | -6.63996649 | -3.55823326 |
| 6 | 0.14131387 | -5.68037319 | 0.29967061 |
| 6 | 0.74830478 | -4.49686527 | 1.07735360 |
| 6 | 0.27815861 | -4.46839046 | 2.53530192 |
| 6 | 0.49594837 | -5.60338831 | -1.17030871 |
| 6 | 0.90946352 | -3.35246062 | 3.38191581 |
| 6 | 0.63116747 | -1.93250358 | 2.91187763 |
| 1 | -0.95585382 | -5.63747740 | 0.37002814 |
| 1 | 0.46737751 | -3.56512570 | 0.56595343 |
| 1 | -0.81857479 | -4.36712217 | 2.56191945 |
| 1 | 2.00597429 | -3.47201848 | 3.39599848 |
| 1 | 0.48241121 | -6.63522673 | 0.72166723 |
| 1 | 1.84821510 | -4.56096792 | 1.03951609 |
| 1 | 0.51051724 | -5.43490744 | 3.01189375 |
| 1 | 0.57166606 | -3.45292878 | 4.42554235 |
| 1 | 0.97078139 | -1.75105119 | 1.88196349 |
| 1 | 1.11472178 | -1.19861078 | 3.57085705 |
| 8 | 0.02863940 | -4.80476046 | -1.96148789 |
| 8 | -0.81197226 | -1.71658039 | 2.95222330 |
| 1 | 2.59813166 | -7.28537846 | -2.99749303 |
| 1 | 2.31911349 | -5.50748396 | -3.11639404 |

Electronic energy = -3908.665194 a.u.
DFT-D3(BJ) dispersion correction = -0.142824 a.u.
Thermal free energy = 0.742053 a.u.
Gibbs free energy = -3908.065966 a.u.
Number of imaginary frequencies = 1.

**TS4 eq CL isomer/conformer 7**

| Atomic N. | X | Y | Z |
|---|---|---|---|
| 7 | 0.11304467 | 2.53828001 | 2.45096827 |
| 8 | -0.45160973 | 1.89983356 | -0.35562471 |
| 8 | 2.42212725 | 1.40900457 | 0.95768255 |
| 6 | -1.06683707 | 2.22484279 | 3.29275703 |
| 6 | -1.38629866 | 2.86317587 | -0.22555529 |
| 6 | -2.46523571 | 2.92904377 | -1.15947795 |
| 6 | -3.41704845 | 3.94180202 | -0.97654152 |
| 6 | -3.34302330 | 4.87279558 | 0.06387849 |
| 6 | -2.27651119 | 4.81082678 | 0.95457321 |
| 6 | -1.29396868 | 3.82537889 | 0.81212062 |
| 6 | -0.09363488 | 3.83177209 | 1.71698594 |
| 1 | -4.11005354 | 5.64021492 | 0.16782635 |
| 6 | -2.57338786 | 1.93665946 | -2.33420181 |
| 6 | 3.07923269 | 2.45894408 | 1.49446166 |
| 6 | 4.30045605 | 2.90997314 | 0.90480387 |

| | | | |
|---|---|---|---|
| 6 | 4.95365953 | 3.99017453 | 1.51375926 |
| 6 | 4.45699406 | 4.63396978 | 2.65053678 |
| 6 | 3.26544428 | 4.19050121 | 3.21235967 |
| 6 | 2.57915807 | 3.10601211 | 2.65368104 |
| 6 | 1.33209026 | 2.60666990 | 3.33326983 |
| 1 | 4.99760675 | 5.47474575 | 3.08497715 |
| 6 | 4.88242912 | 2.24298215 | -0.35696492 |
| 6 | -3.80994153 | 2.21114993 | -3.21049285 |
| 6 | -2.69594646 | 0.48739964 | -1.80649424 |
| 6 | -1.32708025 | 2.06030846 | -3.24300838 |
| 6 | 6.20919371 | 2.89063406 | -0.79647970 |
| 6 | 3.89082813 | 2.38703609 | -1.53562188 |
| 6 | 5.17393208 | 0.74888968 | -0.08171761 |
| 1 | -1.24044085 | 3.04308152 | 4.01175594 |
| 1 | -1.95469952 | 2.11512446 | 2.66086125 |
| 1 | -0.18339746 | 4.64549398 | 2.45734310 |
| 1 | 0.82664931 | 4.01118088 | 1.14188671 |
| 1 | -4.25392532 | 4.01175594 | -1.66924536 |
| 1 | -2.18722272 | 5.53926706 | 1.76395071 |
| 1 | 1.47025502 | 1.58496797 | 3.72164583 |
| 1 | 1.09945810 | 3.26351857 | 4.18901348 |
| 1 | 5.88555050 | 4.35327625 | 1.08421910 |
| 1 | 2.85746694 | 4.67791700 | 4.10086346 |
| 1 | -3.83306766 | 1.48353338 | -4.03445435 |
| 1 | -4.74892235 | 2.10467029 | -2.64789820 |
| 1 | -3.78478241 | 3.21529937 | -3.65748811 |
| 1 | -1.81326067 | 0.20024458 | -1.22400582 |
| 1 | -3.58975005 | 0.38133085 | -1.17320120 |
| 1 | -2.79767418 | -0.20963621 | -2.65289426 |
| 1 | -1.25801265 | 3.07276797 | -3.66656399 |
| 1 | -0.40539584 | 1.85252023 | -2.68829870 |
| 1 | -1.40222418 | 1.34852505 | -4.07964993 |
| 1 | 6.57783556 | 2.37443519 | -1.69452024 |
| 1 | 6.98701811 | 2.80644464 | -0.02376793 |
| 1 | 6.08679581 | 3.95265198 | -1.05344105 |
| 1 | 2.92074037 | 1.93273866 | -1.30768406 |
| 1 | 3.72645903 | 3.44814777 | -1.77288389 |
| 1 | 4.30196285 | 1.90009546 | -2.43317890 |
| 1 | 5.92038774 | 0.64221895 | 0.71907705 |
| 1 | 4.26572132 | 0.21439920 | 0.21674366 |
| 1 | 5.58034325 | 0.27367675 | -0.98776811 |
| 1 | -0.88561267 | 1.28946745 | 3.83046484 |
| 6 | 1.78923237 | -2.04963589 | 2.10002184 |
| 6 | 2.17631078 | -3.30292010 | 1.30552018 |
| 6 | 2.62685537 | -3.07884026 | -0.14666811 |
| 6 | 0.41646534 | -1.46245396 | 1.84113550 |
| 6 | 1.56775701 | -2.56081319 | -1.13524270 |
| 6 | 1.29837763 | -1.05874133 | -1.07905483 |
| 1 | 2.52064228 | -1.24095488 | 1.96222436 |
| 1 | 3.00885892 | -3.78488255 | 1.84201288 |
| 1 | 3.00397587 | -4.04428720 | -0.52121186 |
| 1 | 1.91444850 | -2.77308297 | -2.16033340 |
| 1 | 1.78563857 | -2.28974509 | 3.17834187 |

| | | | |
|---|---|---|---|
| 1 | 1.34224045 | -4.02073002 | 1.32922423 |
| 1 | 3.49135780 | -2.39263868 | -0.15828443 |
| 1 | 0.62095517 | -3.11119103 | -1.00865531 |
| 1 | 0.67751807 | -0.76667160 | -1.94468486 |
| 1 | 2.25780749 | -0.51582259 | -1.15408921 |
| 8 | 0.08754376 | -0.31981552 | 2.36262846 |
| 8 | 0.60504603 | -0.66784376 | 0.10803377 |
| 31 | 0.57054734 | 1.07025135 | 0.99217963 |
| 8 | -4.06959915 | -7.36353397 | -3.05051279 |
| 6 | -4.91522455 | -8.49230289 | -3.37776661 |
| 1 | -4.75654650 | -9.31062794 | -2.66416740 |
| 6 | -3.35544348 | -5.66002131 | -1.56572831 |
| 6 | -3.55725527 | -5.00226688 | -0.20124389 |
| 6 | -2.60295129 | -3.82577944 | 0.02540137 |
| 6 | -4.28745747 | -6.83073425 | -1.81220889 |
| 6 | -2.79943657 | -3.16219640 | 1.39269602 |
| 6 | -1.91081190 | -1.95368183 | 1.63571334 |
| 1 | -2.32152462 | -6.02451897 | -1.68142617 |
| 1 | -3.41870880 | -5.75644732 | 0.58879703 |
| 1 | -1.56085479 | -4.17234707 | -0.06358592 |
| 1 | -2.63763094 | -3.89803529 | 2.19731402 |
| 1 | -3.50262141 | -4.93471432 | -2.38246870 |
| 1 | -4.60102129 | -4.66271734 | -0.11345023 |
| 1 | -2.74832630 | -3.07102108 | -0.76666760 |
| 1 | -3.84322047 | -2.81909823 | 1.48972344 |
| 1 | -2.15486574 | -1.45832610 | 2.58528399 |
| 1 | -1.99417841 | -1.21910393 | 0.82104123 |
| 8 | -5.12060690 | -7.25792933 | -1.03571379 |
| 8 | -0.52737969 | -2.41272283 | 1.69469273 |
| 1 | -4.61740971 | -8.79405308 | -4.38649035 |
| 1 | -5.97237968 | -8.19913673 | -3.35567355 |

Electronic energy = -3908.667364 a.u.
DFT-D3(BJ) dispersion correction = -0.138788 a.u.
Thermal free energy = 0.740215 a.u.
Gibbs free energy = -3908.065936 a.u.
Number of imaginary frequencies = 1.

**TS4 eq CL isomer/conformer 8**

| Atomic N. | X | Y | Z |
|---|---|---|---|
| 7 | 0.34322032 | 2.75230193 | 1.51004839 |
| 8 | -0.21384104 | 1.97015309 | -1.26065695 |
| 8 | 2.57872486 | 1.32976365 | 0.16596580 |
| 6 | -0.87776983 | 2.60097861 | 2.33776283 |
| 6 | -1.06647277 | 3.01423335 | -1.22037899 |
| 6 | -2.11823225 | 3.10326910 | -2.18288255 |
| 6 | -2.98341489 | 4.20266724 | -2.09403610 |
| 6 | -2.84999847 | 5.19573212 | -1.11892128 |
| 6 | -1.81004536 | 5.10791540 | -0.19945149 |
| 6 | -0.91267371 | 4.03597832 | -0.24916986 |
| 6 | 0.26540443 | 4.00391912 | 0.68375528 |
| 1 | -3.55053520 | 6.03021383 | -1.08759558 |
| 6 | -2.28967929 | 2.04131532 | -3.28702116 |

| | | | |
|---|---|---|---|
| 6 | 3.31128478 | 2.35715747 | 0.64558464 |
| 6 | 4.57787895 | 2.66276979 | 0.05853628 |
| 6 | 5.30783606 | 3.72472930 | 0.60972536 |
| 6 | 4.84420776 | 4.48578739 | 1.68643510 |
| 6 | 3.60756993 | 4.18414259 | 2.24500489 |
| 6 | 2.84352756 | 3.12397838 | 1.74360263 |
| 6 | 1.54345119 | 2.77968836 | 2.41994333 |
| 1 | 5.44519377 | 5.30699778 | 2.07673526 |
| 6 | 5.12597275 | 1.86397672 | -1.14025199 |
| 6 | -3.48237705 | 2.35461640 | -4.20984411 |
| 6 | -2.54525304 | 0.64950228 | -2.66145802 |
| 6 | -1.02126265 | 1.99349952 | -4.17212534 |
| 6 | 6.51184559 | 2.36615515 | -1.58759224 |
| 6 | 4.17386675 | 2.01000547 | -2.35087085 |
| 6 | 5.28297997 | 0.37311345 | -0.75893933 |
| 1 | -0.99639893 | 3.48081446 | 2.99235034 |
| 1 | -1.75743520 | 2.52094603 | 1.69016731 |
| 1 | 0.23208965 | 4.87244081 | 1.36408460 |
| 1 | 1.20916271 | 4.06270790 | 0.12187114 |
| 1 | -3.79812932 | 4.29288578 | -2.81042433 |
| 1 | -1.67457461 | 5.88168049 | 0.55992907 |
| 1 | 1.58477664 | 1.77964365 | 2.88034868 |
| 1 | 1.34865320 | 3.51202679 | 3.22221804 |
| 1 | 6.27580786 | 3.97804523 | 0.18117382 |
| 1 | 3.22379851 | 4.76560020 | 3.08649230 |
| 1 | -3.55302763 | 1.57358813 | -4.98042393 |
| 1 | -4.43688679 | 2.36810803 | -3.66368651 |
| 1 | -3.36363745 | 3.31814623 | -4.72592211 |
| 1 | -1.69997740 | 0.33145899 | -2.04074168 |
| 1 | -3.45511961 | 0.66516244 | -2.04260707 |
| 1 | -2.69307232 | -0.09574586 | -3.45823908 |
| 1 | -0.85843372 | 2.96286654 | -4.66521120 |
| 1 | -0.13075489 | 1.75016642 | -3.58207989 |
| 1 | -1.14193726 | 1.23110652 | -4.95722055 |
| 1 | 6.85293150 | 1.76034594 | -2.43933558 |
| 1 | 7.26388025 | 2.27021170 | -0.79106122 |
| 1 | 6.48597431 | 3.41453576 | -1.91792965 |
| 1 | 3.16389871 | 1.65626788 | -2.11790442 |
| 1 | 4.10641909 | 3.06232142 | -2.66284657 |
| 1 | 4.55891275 | 1.42916143 | -3.20299053 |
| 1 | 5.99964714 | 0.25915912 | 0.06767523 |
| 1 | 4.32615137 | -0.06214513 | -0.45148784 |
| 1 | 5.66742325 | -0.19497018 | -1.61998725 |
| 1 | -0.78968710 | 1.69505298 | 2.94473648 |
| 6 | 1.64832878 | -1.95069790 | 1.53265083 |
| 6 | 1.95571625 | -3.28491521 | 0.84140623 |
| 6 | 2.43538308 | -3.20656300 | -0.61681157 |
| 6 | 0.32487625 | -1.29070032 | 1.20376551 |
| 6 | 1.42685854 | -2.69422579 | -1.66031325 |
| 6 | 1.27824879 | -1.17595375 | -1.73391736 |
| 1 | 2.43666601 | -1.20752776 | 1.34806311 |
| 1 | 2.74743700 | -3.77961850 | 1.42607725 |
| 1 | 2.74607420 | -4.22280550 | -0.90880424 |

| | | | |
|---|---|---|---|
| 1 | 1.76173627 | -3.02090716 | -2.65884399 |
| 1 | 1.61399949 | -2.10723209 | 2.62588191 |
| 1 | 1.07369578 | -3.94008088 | 0.90625352 |
| 1 | 3.34720278 | -2.58655643 | -0.66776919 |
| 1 | 0.43844447 | -3.15504265 | -1.49875987 |
| 1 | 0.68955171 | -0.90900606 | -2.62974906 |
| 1 | 2.27877998 | -0.71884793 | -1.83938754 |
| 8 | 0.06864626 | -0.09117459 | 1.62255085 |
| 8 | 0.61048013 | -0.63906240 | -0.59109867 |
| 31 | 0.70424497 | 1.15144694 | 0.16594759 |
| 8 | -5.22431755 | -7.71411419 | 3.87324023 |
| 6 | -6.13242722 | -8.83810997 | 3.78041291 |
| 1 | -7.16018343 | -8.48903275 | 3.61954522 |
| 6 | -4.20868397 | -5.77844620 | 2.95742846 |
| 6 | -4.08922720 | -4.86877203 | 1.73535323 |
| 6 | -3.10313368 | -3.71785045 | 1.95793068 |
| 6 | -5.18212986 | -6.92467070 | 2.75981092 |
| 6 | -2.97529626 | -2.80481815 | 0.73344105 |
| 6 | -2.02131677 | -1.63772500 | 0.92417151 |
| 1 | -4.53560829 | -5.21112537 | 3.84439826 |
| 1 | -5.08339977 | -4.46987581 | 1.48098111 |
| 1 | -3.42617321 | -3.11637878 | 2.82583356 |
| 1 | -3.96154022 | -2.38046098 | 0.48159984 |
| 1 | -3.23140454 | -6.20912743 | 3.22966981 |
| 1 | -3.77468729 | -5.46820307 | 0.86711174 |
| 1 | -2.11120129 | -4.12373686 | 2.21415663 |
| 1 | -2.65513754 | -3.39085531 | -0.14307100 |
| 1 | -2.27679133 | -1.04349637 | 1.81358826 |
| 1 | -2.01088953 | -0.97669423 | 0.04626834 |
| 8 | -5.84579849 | -7.13555098 | 1.76262903 |
| 8 | -0.67847228 | -2.18112993 | 1.09638190 |
| 1 | -6.04552507 | -9.36011124 | 4.73813391 |
| 1 | -5.84489059 | -9.49639511 | 2.95099115 |

Electronic energy = -3908.667229 a.u.
DFT-D3(BJ) dispersion correction = -0.137840 a.u.
Thermal free energy = 0.739358 a.u.
Gibbs free energy = -3908.065711 a.u.
Number of imaginary frequencies = 1.

**TS4 eq CL isomer/conformer 9**

| Atomic N. | X | Y | Z |
|---|---|---|---|
| 6 | -2.78841757 | -0.76064113 | 2.64660164 |
| 6 | -3.67056121 | -1.87176119 | 2.06207015 |
| 6 | -3.90402336 | -1.85159306 | 0.54053685 |
| 6 | -1.29321272 | -0.88252850 | 2.45079888 |
| 6 | -2.70703591 | -2.22251780 | -0.35498838 |
| 6 | -1.75237877 | -1.07387768 | -0.67182622 |
| 1 | -3.08353406 | 0.22607961 | 2.26609631 |
| 1 | -4.65409066 | -1.78827898 | 2.55141609 |
| 1 | -4.71767733 | -2.56628856 | 0.33649832 |
| 1 | -3.08094011 | -2.59405594 | -1.32271723 |
| 1 | -2.92703028 | -0.73042265 | 3.74283378 |

| | | | |
|---|---|---|---|
| 1 | -3.26602101 | -2.85152990 | 2.35990149 |
| 1 | -4.29069313 | -0.86265865 | 0.24074880 |
| 1 | -2.14516440 | -3.05589511 | 0.09749097 |
| 1 | -0.96724898 | -1.43141635 | -1.36178043 |
| 1 | -2.31068583 | -0.26683245 | -1.18081105 |
| 8 | -0.51255191 | 0.11802585 | 2.67394101 |
| 8 | -1.14941049 | -0.56385027 | 0.51337283 |
| 7 | 0.97342156 | 2.46727728 | 1.99549907 |
| 8 | -1.09967900 | 2.23444799 | -0.06973319 |
| 8 | 1.49614383 | 0.30726960 | 0.07092545 |
| 6 | 0.30869703 | 2.88099672 | 3.25453112 |
| 6 | -1.21517158 | 3.56647202 | 0.11706104 |
| 6 | -2.39850780 | 4.23908467 | -0.32006391 |
| 6 | -2.47994366 | 5.62023004 | -0.09572131 |
| 6 | -1.46174138 | 6.34932456 | 0.52482216 |
| 6 | -0.30769304 | 5.68798930 | 0.92953839 |
| 6 | -0.17008683 | 4.31137575 | 0.72085042 |
| 6 | 1.12833147 | 3.64091680 | 1.07215384 |
| 1 | -1.57253429 | 7.42220490 | 0.68202203 |
| 6 | -3.54271592 | 3.48314633 | -1.02320956 |
| 6 | 2.74330534 | 0.81593453 | 0.06227690 |
| 6 | 3.61435301 | 0.54019201 | -1.03686635 |
| 6 | 4.91089329 | 1.06923523 | -0.98192700 |
| 6 | 5.36671595 | 1.85141950 | 0.08368226 |
| 6 | 4.50545393 | 2.12501135 | 1.14028157 |
| 6 | 3.20481349 | 1.60801480 | 1.14423697 |
| 6 | 2.31547578 | 1.87042715 | 2.32995918 |
| 1 | 6.38231108 | 2.24708505 | 0.07798863 |
| 6 | 3.14609074 | -0.29557663 | -2.24437434 |
| 6 | -4.70253104 | 4.41793092 | -1.41490850 |
| 6 | -4.12321862 | 2.40278984 | -0.08136938 |
| 6 | -3.02005697 | 2.82944248 | -2.32458030 |
| 6 | 4.25862772 | -0.46630894 | -3.29591603 |
| 6 | 1.95755819 | 0.40677589 | -2.94332321 |
| 6 | 2.72595489 | -1.71201158 | -1.78676727 |
| 1 | 0.89075641 | 3.68380536 | 3.73756306 |
| 1 | -0.69802797 | 3.25380508 | 3.03857523 |
| 1 | 1.81102462 | 4.36981445 | 1.54211910 |
| 1 | 1.62882744 | 3.26137277 | 0.16912727 |
| 1 | -3.37328453 | 6.15453549 | -0.41406628 |
| 1 | 0.50981094 | 6.23830246 | 1.40120258 |
| 1 | 2.09476349 | 0.94203590 | 2.88090344 |
| 1 | 2.83219255 | 2.55625369 | 3.02335849 |
| 1 | 5.59595600 | 0.87406061 | -1.80512259 |
| 1 | 4.83743210 | 2.73986361 | 1.98018771 |
| 1 | -5.48245952 | 3.82799908 | -1.91752320 |
| 1 | -5.16320127 | 4.89753477 | -0.53901646 |
| 1 | -4.38168897 | 5.20402397 | -2.11343037 |
| 1 | -3.35621424 | 1.67440023 | 0.20308455 |
| 1 | -4.52809585 | 2.86330068 | 0.83214304 |
| 1 | -4.94438689 | 1.86884306 | -0.58403961 |
| 1 | -2.66260181 | 3.59897622 | -3.02411505 |
| 1 | -2.19594365 | 2.13634240 | -2.12313700 |

| | | | |
|---|---|---|---|
| 1 | -3.83301917 | 2.27529591 | -2.81864637 |
| 1 | 3.87184986 | -1.07186904 | -4.12811945 |
| 1 | 5.13761177 | -0.98629038 | -2.88773346 |
| 1 | 4.58679610 | 0.49668337 | -3.71317532 |
| 1 | 1.10623173 | 0.53030766 | -2.26548210 |
| 1 | 2.25677848 | 1.39951612 | -3.31001360 |
| 1 | 1.62748393 | -0.18948641 | -3.80739656 |
| 1 | 3.58128368 | -2.24303430 | -1.34244599 |
| 1 | 1.91854693 | -1.66537398 | -1.04882525 |
| 1 | 2.37050338 | -2.29441129 | -2.65033610 |
| 1 | 0.23754625 | 2.01819320 | 3.92333814 |
| 31 | -0.06397011 | 0.98211513 | 0.88502648 |
| 8 | 1.71682774 | -6.21935451 | -2.32028440 |
| 6 | 2.21832441 | -5.98096354 | -3.65819821 |
| 1 | 1.42742866 | -6.15035009 | -4.39938073 |
| 6 | 0.23549392 | -5.69570102 | -0.53383368 |
| 6 | 0.80044074 | -4.62175168 | 0.41651788 |
| 6 | 0.28100555 | -4.79452201 | 1.84772482 |
| 6 | 0.65957272 | -5.43222579 | -1.96303277 |
| 6 | 0.87913086 | -3.80597455 | 2.86041775 |
| 6 | 0.59549547 | -2.33574307 | 2.59138612 |
| 1 | -0.86339633 | -5.66475186 | -0.50990596 |
| 1 | 0.52565746 | -3.63111156 | 0.02649839 |
| 1 | -0.81652176 | -4.69937387 | 1.85182005 |
| 1 | 1.97618943 | -3.91841364 | 2.88466876 |
| 1 | 0.56787229 | -6.69471827 | -0.22039039 |
| 1 | 1.90153357 | -4.67272520 | 0.40977617 |
| 1 | 0.50352155 | -5.81740094 | 2.19340650 |
| 1 | 0.51781404 | -4.05650098 | 3.87042042 |
| 1 | 0.94884512 | -2.00772518 | 1.60312156 |
| 1 | 1.06211699 | -1.69929303 | 3.35548205 |
| 8 | 0.15737433 | -4.60643633 | -2.70307960 |
| 8 | -0.84995527 | -2.13827936 | 2.63905809 |
| 1 | 3.03688282 | -6.69466465 | -3.79193073 |
| 1 | 2.58225453 | -4.94998951 | -3.75151891 |

Electronic energy = -3908.665180 a.u.
DFT-D3(BJ) dispersion correction = -0.142671 a.u.
Thermal free energy = 0.742365 a.u.
Gibbs free energy = -3908.065486 a.u.
Number of imaginary frequencies = 1.

**TS4 eq CL isomer/conformer 10**

| Atomic N. | X | Y | Z |
|---|---|---|---|
| 6 | -3.39380097 | 0.04685285 | 1.35196149 |
| 6 | -4.22780752 | -1.05642581 | 0.68851924 |
| 6 | -4.02619457 | -1.26986945 | -0.82261431 |
| 6 | -1.92179275 | -0.23537879 | 1.56582332 |
| 6 | -2.68707228 | -1.89393365 | -1.25983644 |
| 6 | -1.54642749 | -0.89633012 | -1.45228684 |
| 1 | -3.46278358 | 0.99030840 | 0.79465395 |
| 1 | -5.28641844 | -0.79913765 | 0.85234529 |
| 1 | -4.83978176 | -1.93125236 | -1.16130722 |

| | | | |
|---|---|---|---|
| 1 | -2.82417321 | -2.40238357 | -2.22843122 |
| 1 | -3.79602504 | 0.24383222 | 2.36200666 |
| 1 | -4.05964756 | -2.00642323 | 1.21887398 |
| 1 | -4.17344618 | -0.31398946 | -1.35391557 |
| 1 | -2.38373637 | -2.67138314 | -0.54003733 |
| 1 | -0.63698888 | -1.43273079 | -1.77935255 |
| 1 | -1.82104957 | -0.17877577 | -2.24765444 |
| 8 | -1.11179757 | 0.71801293 | 1.89872694 |
| 8 | -1.27728891 | -0.18224245 | -0.25194061 |
| 7 | 0.86327398 | 2.74301553 | 1.41872990 |
| 8 | -0.56550884 | 2.42721176 | -1.12545002 |
| 8 | 1.50975919 | 0.23728725 | 0.00397947 |
| 6 | -0.04023182 | 3.42693949 | 2.37540579 |
| 6 | -0.52574235 | 3.77629709 | -1.13464320 |
| 6 | -1.43099654 | 4.50098801 | -1.96978617 |
| 6 | -1.35816026 | 5.90047884 | -1.94254673 |
| 6 | -0.44629735 | 6.59691715 | -1.14423871 |
| 6 | 0.43963227 | 5.88078213 | -0.34690860 |
| 6 | 0.41663983 | 4.48190212 | -0.34389669 |
| 6 | 1.44812274 | 3.72818160 | 0.44816080 |
| 1 | -0.43029863 | 7.68673277 | -1.15311444 |
| 6 | -2.44603705 | 3.77685690 | -2.87585950 |
| 6 | 2.78114891 | 0.56989962 | 0.29994422 |
| 6 | 3.85544086 | -0.01025810 | -0.44269434 |
| 6 | 5.16060162 | 0.35107014 | -0.08188876 |
| 6 | 5.43984079 | 1.24910629 | 0.95259285 |
| 6 | 4.38618374 | 1.81267631 | 1.66332984 |
| 6 | 3.06332493 | 1.47458470 | 1.35531533 |
| 6 | 1.94976318 | 2.04477787 | 2.19318652 |
| 1 | 6.47169781 | 1.50594020 | 1.19185817 |
| 6 | 3.59351778 | -0.99080223 | -1.60327840 |
| 6 | -3.30397916 | 4.76393318 | -3.68962646 |
| 6 | -3.41304135 | 2.92617393 | -2.01939893 |
| 6 | -1.70017147 | 2.87397051 | -3.88699579 |
| 6 | 4.90228510 | -1.49339259 | -2.24146581 |
| 6 | 2.77934909 | -0.28887621 | -2.71608639 |
| 6 | 2.82698107 | -2.23395157 | -1.09208441 |
| 1 | 0.51385403 | 4.21264839 | 2.91591120 |
| 1 | -0.87317312 | 3.88838816 | 1.83464825 |
| 1 | 2.08352637 | 4.43679667 | 1.00703740 |
| 1 | 2.10835552 | 3.15248156 | -0.21733259 |
| 1 | -2.04011416 | 6.47676992 | -2.56525421 |
| 1 | 1.17174041 | 6.40213585 | 0.27424568 |
| 1 | 1.43878782 | 1.25712311 | 2.76995039 |
| 1 | 2.37389898 | 2.76511836 | 2.91380692 |
| 1 | 5.99791050 | -0.07571915 | -0.63117754 |
| 1 | 4.58076334 | 2.51825261 | 2.47443938 |
| 1 | -4.00172329 | 4.19479895 | -4.32064867 |
| 1 | -3.90321898 | 5.42271805 | -3.04445434 |
| 1 | -2.69319987 | 5.39069462 | -4.35518694 |
| 1 | -2.87045813 | 2.16427588 | -1.44909799 |
| 1 | -3.96930981 | 3.56318355 | -1.31569660 |
| 1 | -4.14445496 | 2.42227554 | -2.67008328 |

| | | | |
|---|---|---|---|
| 1 | -1.05529058 | 3.47933960 | -4.54047394 |
| 1 | -1.07623887 | 2.13163400 | -3.37715578 |
| 1 | -2.42628717 | 2.34603858 | -4.52438927 |
| 1 | 4.65798950 | -2.18874907 | -3.05726004 |
| 1 | 5.53247690 | -2.03561592 | -1.52158046 |
| 1 | 5.49407387 | -0.67339367 | -2.67306638 |
| 1 | 1.81186378 | 0.06648999 | -2.34555984 |
| 1 | 3.33462548 | 0.57185084 | -3.11618328 |
| 1 | 2.59855175 | -0.98952812 | -3.54562664 |
| 1 | 3.41500235 | -2.76165342 | -0.32635319 |
| 1 | 1.85923553 | -1.95477271 | -0.66131544 |
| 1 | 2.65228391 | -2.93282485 | -1.92448437 |
| 1 | -0.43379194 | 2.69276524 | 3.08467317 |
| 31 | -0.06792771 | 1.24037588 | 0.24865279 |
| 8 | -0.96446002 | -6.85547638 | 1.08271706 |
| 6 | -2.36008096 | -7.21483564 | 0.93575722 |
| 1 | -2.60050035 | -8.08419037 | 1.56043351 |
| 6 | 0.89339709 | -6.24226713 | 2.43447733 |
| 6 | 1.26933324 | -5.21017408 | 3.50839353 |
| 6 | 1.13805664 | -3.74581981 | 3.06198716 |
| 6 | -0.59443682 | -6.53219795 | 2.35636616 |
| 6 | -0.28561947 | -3.30652571 | 2.69442153 |
| 6 | -0.33080497 | -1.82129204 | 2.37638426 |
| 1 | 1.36669385 | -7.20986223 | 2.67243123 |
| 1 | 2.31377840 | -5.38512993 | 3.80673409 |
| 1 | 1.51352227 | -3.10557866 | 3.87780929 |
| 1 | -0.97747475 | -3.53122878 | 3.52067208 |
| 1 | 1.26438677 | -5.95319271 | 1.44078875 |
| 1 | 0.64929420 | -5.39127779 | 4.39966440 |
| 1 | 1.80643892 | -3.56871581 | 2.20205593 |
| 1 | -0.64547646 | -3.86224627 | 1.81446314 |
| 1 | 0.33034706 | -1.56215966 | 1.53588998 |
| 1 | -0.04665984 | -1.21186161 | 3.24692082 |
| 8 | -1.36806262 | -6.52237988 | 3.29564929 |
| 8 | -1.70136547 | -1.48817205 | 2.00376034 |
| 1 | -2.48750401 | -7.45106030 | -0.12496372 |
| 1 | -3.00341892 | -6.37544584 | 1.22812879 |

Electronic energy = -3908.664499 a.u.
DFT-D3(BJ) dispersion correction = -0.140177 a.u.
Thermal free energy = 0.739405 a.u.
Gibbs free energy = -3908.065270 a.u.
Number of imaginary frequencies = 1.

**TS4 eq CL isomer/conformer 11**

| Atomic N. | X | Y | Z |
|---|---|---|---|
| 6 | -3.59205222 | 0.23913541 | 1.33267522 |
| 6 | -4.43966103 | -0.85715610 | 0.67454112 |
| 6 | -4.24870729 | -1.07510781 | -0.83735669 |
| 6 | -2.12464046 | -0.05998553 | 1.55205655 |
| 6 | -2.91888213 | -1.71201456 | -1.28433490 |
| 6 | -1.76923954 | -0.72587448 | -1.48230779 |
| 1 | -3.64771247 | 1.18010807 | 0.76975459 |

| | | | |
|---|---|---|---|
| 1 | -5.49488497 | -0.58902121 | 0.84283853 |
| 1 | -5.07061195 | -1.72964406 | -1.16911030 |
| 1 | -3.06848431 | -2.21746135 | -2.25262713 |
| 1 | -3.99374366 | 0.44796693 | 2.34072351 |
| 1 | -4.27910995 | -1.80795681 | 1.20577407 |
| 1 | -4.39128923 | -0.11881218 | -1.36913204 |
| 1 | -2.61889839 | -2.49430442 | -0.56813288 |
| 1 | -0.87042356 | -1.26959002 | -1.82660043 |
| 1 | -2.04574060 | 0.00195600 | -2.26762199 |
| 8 | -1.29995215 | 0.88200307 | 1.87267649 |
| 8 | -1.47619808 | -0.02941795 | -0.27765018 |
| 7 | 0.68508059 | 2.89210415 | 1.36792278 |
| 8 | -0.75645584 | 2.57279277 | -1.16879046 |
| 8 | 1.30928898 | 0.37056205 | -0.02771678 |
| 6 | -0.20947862 | 3.58725572 | 2.32489443 |
| 6 | -0.70811301 | 3.92174172 | -1.18533635 |
| 6 | -1.61136496 | 4.64835787 | -2.02101135 |
| 6 | -1.52984428 | 6.04745483 | -2.00049639 |
| 6 | -0.61090654 | 6.74182892 | -1.20854414 |
| 6 | 0.27354252 | 6.02379322 | -0.41129395 |
| 6 | 0.24167825 | 4.62510920 | -0.40163764 |
| 6 | 1.27167058 | 3.86840463 | 0.38930324 |
| 1 | -0.58807427 | 7.83146334 | -1.22248745 |
| 6 | -2.63244152 | 3.92658353 | -2.92204332 |
| 6 | 2.58472896 | 0.69968683 | 0.25556156 |
| 6 | 3.65042472 | 0.10707531 | -0.48919138 |
| 6 | 4.95973206 | 0.46566686 | -0.14104803 |
| 6 | 5.25036812 | 1.37190580 | 0.88315594 |
| 6 | 4.20463324 | 1.94672382 | 1.59664524 |
| 6 | 2.87817240 | 1.61192465 | 1.30095899 |
| 6 | 1.77198517 | 2.19272757 | 2.14125562 |
| 1 | 6.28509045 | 1.62581933 | 1.11288631 |
| 6 | 3.37531590 | -0.88418883 | -1.63752937 |
| 6 | -3.48670387 | 4.91457796 | -3.73817992 |
| 6 | -3.60211539 | 3.08430767 | -2.06039929 |
| 6 | -1.89251614 | 3.01604247 | -3.93085790 |
| 6 | 4.67725706 | -1.40073550 | -2.27848911 |
| 6 | 2.55774617 | -0.18968193 | -2.75238442 |
| 6 | 2.60508370 | -2.11749911 | -1.10799658 |
| 1 | 0.35190123 | 4.37324429 | 2.85735345 |
| 1 | -1.04264951 | 4.05002737 | 1.78562140 |
| 1 | 1.91450536 | 4.57542944 | 0.94160056 |
| 1 | 1.92490852 | 3.28497338 | -0.27634823 |
| 1 | -2.21026921 | 6.62502813 | -2.62368488 |
| 1 | 1.01148164 | 6.54334211 | 0.20442718 |
| 1 | 1.26014817 | 1.41098797 | 2.72519732 |
| 1 | 2.20329595 | 2.91532564 | 2.85527945 |
| 1 | 5.79127121 | 0.02968021 | -0.69183618 |
| 1 | 4.40844870 | 2.65822220 | 2.40025449 |
| 1 | -4.18877125 | 4.34654856 | -4.36537313 |
| 1 | -4.08117008 | 5.57918930 | -3.09453249 |
| 1 | -2.87403440 | 5.53536844 | -4.40756035 |
| 1 | -3.06202245 | 2.32383633 | -1.48591435 |

| | | | |
|---|---|---|---|
| 1 | -4.15592051 | 3.72729874 | -1.36015332 |
| 1 | -4.33565426 | 2.57931781 | -2.70780230 |
| 1 | -1.24559104 | 3.61582518 | -4.58742285 |
| 1 | -1.27153921 | 2.27248359 | -3.41920948 |
| 1 | -2.62210822 | 2.48937750 | -4.56531191 |
| 1 | 4.42492342 | -2.10355258 | -3.08544540 |
| 1 | 5.30908251 | -1.93841267 | -1.55665874 |
| 1 | 5.27066708 | -0.58853173 | -2.72239828 |
| 1 | 1.59531260 | 0.17648412 | -2.37921262 |
| 1 | 3.11582088 | 0.66257328 | -3.16644979 |
| 1 | 2.36623025 | -0.89863968 | -3.57241631 |
| 1 | 3.19486380 | -2.63959599 | -0.33974928 |
| 1 | 1.64120901 | -1.82810068 | -0.67513436 |
| 1 | 2.42225051 | -2.82453823 | -1.93170798 |
| 1 | -0.60321456 | 2.85998464 | 3.04111123 |
| 31 | -0.25964165 | 1.38866568 | 0.20826177 |
| 8 | 0.66877651 | -8.29118061 | 1.51535273 |
| 6 | 0.84664333 | -9.70875645 | 1.75031042 |
| 1 | 1.88817465 | -9.92565346 | 2.01899743 |
| 6 | 0.75165373 | -6.03577518 | 2.23766160 |
| 6 | 0.99362195 | -5.09703970 | 3.42070293 |
| 6 | 0.89393872 | -3.60900807 | 3.05550528 |
| 6 | 0.95583808 | -7.49742174 | 2.58875847 |
| 6 | -0.51373136 | -3.15305495 | 2.64770627 |
| 6 | -0.55130160 | -1.66016161 | 2.36709762 |
| 1 | 1.43105674 | -5.79705286 | 1.40213704 |
| 1 | 1.99117672 | -5.30321789 | 3.83451509 |
| 1 | 1.22461212 | -3.01638937 | 3.92425370 |
| 1 | -1.23100626 | -3.39228511 | 3.44982100 |
| 1 | -0.26498574 | -5.93253469 | 1.82848358 |
| 1 | 0.28007823 | -5.33304882 | 4.22563362 |
| 1 | 1.60373294 | -3.38258982 | 2.24164438 |
| 1 | -0.84911644 | -3.68760061 | 1.74575794 |
| 1 | 0.11724378 | -1.38443708 | 1.53816092 |
| 1 | -0.27122837 | -1.07348764 | 3.25448298 |
| 8 | 1.32518315 | -7.93107557 | 3.66344285 |
| 8 | -1.91851056 | -1.31380868 | 1.99376202 |
| 1 | 0.58050179 | -10.19410038 | 0.80644143 |
| 1 | 0.19036146 | -10.04879093 | 2.56127024 |

Electronic energy = -3908.665443 a.u.
DFT-D3(BJ) dispersion correction = -0.138849 a.u.
Thermal free energy = 0.739219 a.u.
Gibbs free energy = -3908.065073 a.u.
Number of imaginary frequencies = 1.

**TS4 eq CL isomer/conformer 12**

| Atomic N. | X | Y | Z |
|---|---|---|---|
| 6 | -3.84549546 | -0.22563955 | 1.26500797 |
| 6 | -4.66044140 | -1.34600520 | 0.60612649 |
| 6 | -4.48141670 | -1.54013848 | -0.91021186 |
| 6 | -2.36808872 | -0.47846356 | 1.47574854 |
| 6 | -3.13088965 | -2.10968137 | -1.38433146 |

| | | | |
|---|---|---|---|
| 6 | -2.02542496 | -1.07311893 | -1.57430136 |
| 1 | -3.93595576 | 0.71670789 | 0.70885944 |
| 1 | -5.72274017 | -1.11970818 | 0.79058468 |
| 1 | -5.27702856 | -2.22872162 | -1.23723495 |
| 1 | -3.27112818 | -2.59940672 | -2.36205816 |
| 1 | -4.24697495 | -0.03582634 | 2.27693725 |
| 1 | -4.45709515 | -2.29558372 | 1.12453568 |
| 1 | -4.67610931 | -0.58629590 | -1.42978883 |
| 1 | -2.78868270 | -2.89433455 | -0.69003439 |
| 1 | -1.11806250 | -1.56946301 | -1.96439624 |
| 1 | -2.35100245 | -0.32650739 | -2.32227635 |
| 8 | -1.57356775 | 0.48798704 | 1.80365872 |
| 8 | -1.71696711 | -0.41545749 | -0.35112405 |
| 7 | 0.30817753 | 2.59390783 | 1.31863284 |
| 8 | -1.20625269 | 2.27215576 | -1.18115389 |
| 8 | 1.03304029 | 0.17585380 | -0.19610037 |
| 6 | -0.58702463 | 3.20136166 | 2.33269382 |
| 6 | -1.23591781 | 3.62159562 | -1.15308380 |
| 6 | -2.20258260 | 4.32114029 | -1.93984938 |
| 6 | -2.20487952 | 5.72102690 | -1.86962962 |
| 6 | -1.30818355 | 6.44206619 | -1.07618701 |
| 6 | -0.35875782 | 5.75171375 | -0.33106598 |
| 6 | -0.30574849 | 4.35418987 | -0.37215412 |
| 6 | 0.79672253 | 3.63735795 | 0.35608131 |
| 1 | -1.35183334 | 7.53084421 | -1.04999042 |
| 6 | -3.20068812 | 3.57178307 | -2.84413385 |
| 6 | 2.29381919 | 0.58299744 | 0.04562524 |
| 6 | 3.36219192 | 0.11116427 | -0.77832484 |
| 6 | 4.65922546 | 0.53656060 | -0.46050817 |
| 6 | 4.93475008 | 1.40049899 | 0.60383850 |
| 6 | 3.88495612 | 1.86763489 | 1.38676667 |
| 6 | 2.57170606 | 1.46106303 | 1.12413490 |
| 6 | 1.46316934 | 1.93447411 | 2.02592182 |
| 1 | 5.95991850 | 1.70959997 | 0.80739850 |
| 6 | 3.10157275 | -0.81677592 | -1.98167741 |
| 6 | -4.12992191 | 4.53510427 | -3.60632253 |
| 6 | -4.09940863 | 2.64506149 | -1.99283624 |
| 6 | -2.43290544 | 2.74160242 | -3.90030026 |
| 6 | 4.40371752 | -1.19387996 | -2.71309876 |
| 6 | 2.18784022 | -0.10461647 | -3.00746441 |
| 6 | 2.44110584 | -2.13468528 | -1.51339841 |
| 1 | -0.05285218 | 4.00359583 | 2.86905384 |
| 1 | -1.46834016 | 3.62801290 | 1.84250867 |
| 1 | 1.41472781 | 4.36489153 | 0.91019523 |
| 1 | 1.45938933 | 3.11953521 | -0.35292619 |
| 1 | -2.93552136 | 6.27746725 | -2.45396066 |
| 1 | 0.36523968 | 6.29344034 | 0.28210476 |
| 1 | 1.02182305 | 1.09878063 | 2.59272981 |
| 1 | 1.87406087 | 2.65337944 | 2.75561404 |
| 1 | 5.49243116 | 0.19140770 | -1.07014978 |
| 1 | 4.07563400 | 2.55070400 | 2.21785259 |
| 1 | -4.81102037 | 3.94839072 | -4.23940420 |
| 1 | -4.74815607 | 5.13984823 | -2.92701578 |

| | | | |
|---|---|---|---|
| 1 | -3.57012892 | 5.21462631 | -4.26487112 |
| 1 | -3.50225353 | 1.89800942 | -1.45879936 |
| 1 | -4.67194843 | 3.22973704 | -1.25746250 |
| 1 | -4.81797504 | 2.12075377 | -2.64163065 |
| 1 | -1.83732569 | 3.40064311 | -4.54861593 |
| 1 | -1.75814176 | 2.01847243 | -3.42913866 |
| 1 | -3.14533615 | 2.19518852 | -4.53753328 |
| 1 | 4.16055202 | -1.85478365 | -3.55730009 |
| 1 | 5.10351133 | -1.73535621 | -2.05994511 |
| 1 | 4.91979218 | -0.31302765 | -3.12141514 |
| 1 | 1.22007036 | 0.16258579 | -2.56968188 |
| 1 | 2.66587424 | 0.81342250 | -3.37893414 |
| 1 | 2.00862551 | -0.76509112 | -3.86958790 |
| 1 | 3.10238481 | -2.67309594 | -0.81819105 |
| 1 | 1.48587275 | -1.94555485 | -1.01185215 |
| 1 | 2.25959873 | -2.78951311 | -2.37934089 |
| 1 | -0.90675926 | 2.42923880 | 3.03862357 |
| 31 | -0.59083074 | 1.07582343 | 0.13644920 |
| 8 | 4.24253750 | -6.23856783 | 3.66446137 |
| 6 | 4.39136314 | -7.66411591 | 3.87080550 |
| 1 | 3.50090003 | -8.07558727 | 4.36238623 |
| 6 | 3.14969039 | -4.38343954 | 2.66787243 |
| 6 | 1.80668998 | -3.86919594 | 2.14366555 |
| 6 | 0.68369985 | -3.93868828 | 3.18718863 |
| 6 | 3.18406034 | -5.88626099 | 2.87807226 |
| 6 | -0.69293743 | -3.50500607 | 2.66033673 |
| 6 | -0.74354774 | -2.03373718 | 2.27873421 |
| 1 | 3.43699718 | -3.88873935 | 3.60794854 |
| 1 | 1.51946914 | -4.45784140 | 1.25885785 |
| 1 | 0.59851587 | -4.97318649 | 3.55216813 |
| 1 | -1.45478582 | -3.69178033 | 3.43301892 |
| 1 | 3.95449352 | -4.15097189 | 1.95058405 |
| 1 | 1.94903445 | -2.83272576 | 1.80398905 |
| 1 | 0.95492309 | -3.31859350 | 4.05992746 |
| 1 | -0.96635240 | -4.11793804 | 1.78696692 |
| 1 | -0.10231157 | -1.79637837 | 1.41818631 |
| 1 | -0.45345533 | -1.39003122 | 3.12276006 |
| 8 | 2.40633440 | -6.69247580 | 2.40222192 |
| 8 | -2.12155223 | -1.72655106 | 1.91082191 |
| 1 | 5.27287006 | -7.77347755 | 4.50977039 |
| 1 | 4.53962898 | -8.17738628 | 2.91236472 |

Electronic energy = -3908.665576 a.u.
DFT-D3(BJ) dispersion correction = -0.140280 a.u.
Thermal free energy = 0.740908 a.u.
Gibbs free energy = -3908.064947 a.u.
Number of imaginary frequencies = 1.

**TS4_eq_CL isomer/conformer 13**

| Atomic N. | X | Y | Z |
|---|---|---|---|
| 6 | -3.82087970 | -0.86543232 | 1.06717694 |
| 6 | -4.56658125 | -2.09313583 | 0.52953517 |
| 6 | -4.13721132 | -2.61158776 | -0.85204810 |

| | | | |
|---|---|---|---|
| 6 | -2.43651724 | -1.09425759 | 1.64598930 |
| 6 | -2.69965959 | -3.14239192 | -0.98306578 |
| 6 | -1.61775088 | -2.07237864 | -1.11589456 |
| 1 | -3.74291992 | -0.07302936 | 0.31288835 |
| 1 | -5.63334274 | -1.82549632 | 0.46769211 |
| 1 | -4.83082962 | -3.42432809 | -1.12235940 |
| 1 | -2.63791156 | -3.75812054 | -1.89578283 |
| 1 | -4.39390755 | -0.43969616 | 1.91040564 |
| 1 | -4.49830723 | -2.90683770 | 1.26721001 |
| 1 | -4.29309225 | -1.82060266 | -1.60510349 |
| 1 | -2.45540977 | -3.81006360 | -0.14073600 |
| 1 | -0.66844553 | -2.54846621 | -1.41677082 |
| 1 | -1.90255404 | -1.35969841 | -1.91162372 |
| 8 | -1.70913327 | -0.07859468 | 2.00803232 |
| 8 | -1.38257444 | -1.37338948 | 0.11006216 |
| 7 | 0.27845421 | 1.95991254 | 1.57788682 |
| 8 | -1.32240522 | 1.43289828 | -0.92143869 |
| 8 | 1.15154493 | -0.29688042 | -0.08821433 |
| 6 | -0.51500964 | 2.36522341 | 2.76335025 |
| 6 | -1.62787902 | 2.74712801 | -0.82661307 |
| 6 | -2.66715956 | 3.30316043 | -1.63543510 |
| 6 | -3.00213456 | 4.64817810 | -1.42141855 |
| 6 | -2.33856750 | 5.46027470 | -0.49824730 |
| 6 | -1.25969708 | 4.93638992 | 0.20509127 |
| 6 | -0.88420469 | 3.59991741 | 0.03177893 |
| 6 | 0.40761206 | 3.11755395 | 0.63046569 |
| 1 | -2.64300203 | 6.49780226 | -0.36111036 |
| 6 | -3.35943627 | 2.49049997 | -2.74849749 |
| 6 | 2.32384253 | 0.37038785 | -0.10633911 |
| 6 | 3.26948190 | 0.14157560 | -1.15331483 |
| 6 | 4.49238729 | 0.82277209 | -1.07520115 |
| 6 | 4.80246162 | 1.71153843 | -0.04122116 |
| 6 | 3.86220098 | 1.94672322 | 0.95617920 |
| 6 | 2.63229775 | 1.28121519 | 0.93455994 |
| 6 | 1.64369154 | 1.51206326 | 2.04333496 |
| 1 | 5.76642418 | 2.22047234 | -0.02749261 |
| 6 | 2.95752883 | -0.79309875 | -2.33954692 |
| 6 | -4.38220882 | 3.33366036 | -3.53308892 |
| 6 | -4.11472082 | 1.27982163 | -2.16190553 |
| 6 | -2.29533148 | 1.99740064 | -3.75858521 |
| 6 | 4.12842178 | -0.87156862 | -3.33756280 |
| 6 | 1.72881758 | -0.26085645 | -3.11421704 |
| 6 | 2.68631005 | -2.23171282 | -1.84193933 |
| 1 | -0.03543856 | 3.23259425 | 3.24722624 |
| 1 | -1.52973223 | 2.63654900 | 2.45662713 |
| 1 | 0.90558195 | 3.94685102 | 1.16239917 |
| 1 | 1.08730304 | 2.78592157 | -0.16765852 |
| 1 | -3.81010151 | 5.08818054 | -2.00300717 |
| 1 | -0.67799801 | 5.56848764 | 0.88013983 |
| 1 | 1.48448455 | 0.59471530 | 2.63190317 |
| 1 | 2.03898954 | 2.27861261 | 2.73087120 |
| 1 | 5.23451519 | 0.66555941 | -1.85587180 |
| 1 | 4.07794714 | 2.64291334 | 1.76961434 |

| | | | |
|---|---|---|---|
| 1 | -4.81950474 | 2.71356511 | -4.32878399 |
| 1 | -5.20750999 | 3.68415141 | -2.89625978 |
| 1 | -3.91745067 | 4.20710993 | -4.01215410 |
| 1 | -3.41877341 | 0.59592688 | -1.66644478 |
| 1 | -4.87222958 | 1.60457051 | -1.43317974 |
| 1 | -4.62938881 | 0.73125601 | -2.96587205 |
| 1 | -1.78485262 | 2.85037446 | -4.22848701 |
| 1 | -1.54205477 | 1.36777496 | -3.27139235 |
| 1 | -2.77846956 | 1.41158378 | -4.55568743 |
| 1 | 3.85124803 | -1.54635692 | -4.16009760 |
| 1 | 5.04116631 | -1.27281845 | -2.87368822 |
| 1 | 4.36303473 | 0.10776936 | -3.77881241 |
| 1 | 0.84168708 | -0.20010290 | -2.47500110 |
| 1 | 1.93114376 | 0.74153882 | -3.51839852 |
| 1 | 1.50266373 | -0.92763549 | -3.96041083 |
| 1 | 3.56936002 | -2.63533807 | -1.32511151 |
| 1 | 1.83344567 | -2.26094174 | -1.15474164 |
| 1 | 2.46808457 | -2.88757277 | -2.69875002 |
| 1 | -0.56899488 | 1.53119528 | 3.46916723 |
| 31 | -0.54357719 | 0.36839232 | 0.42937019 |
| 8 | 5.20396233 | -2.11240888 | 2.09812832 |
| 6 | 6.26700068 | -1.23098993 | 2.53509307 |
| 1 | 6.02065086 | -0.18955097 | 2.29316545 |
| 6 | 2.93129230 | -2.79123664 | 2.10598993 |
| 6 | 1.72534597 | -2.96043253 | 3.02931714 |
| 6 | 0.54464042 | -3.66818810 | 2.35508299 |
| 6 | 3.99847126 | -1.88888359 | 2.69144058 |
| 6 | -0.68732083 | -3.73466897 | 3.26982594 |
| 6 | -1.42028034 | -2.40643382 | 3.43513417 |
| 1 | 2.61053658 | -2.32406664 | 1.15979660 |
| 1 | 1.42269325 | -1.96331847 | 3.38253140 |
| 1 | 0.28198597 | -3.14623547 | 1.42014885 |
| 1 | -0.37718830 | -4.07478714 | 4.27259779 |
| 1 | 3.38388109 | -3.75542688 | 1.83278668 |
| 1 | 2.02429605 | -3.51968527 | 3.93154383 |
| 1 | 0.84215701 | -4.69091940 | 2.07165551 |
| 1 | -1.40981162 | -4.47958708 | 2.90259957 |
| 1 | -0.74184740 | -1.54561675 | 3.44090939 |
| 1 | -2.00637984 | -2.39660859 | 4.36568880 |
| 8 | 3.82313299 | -1.05196583 | 3.56037593 |
| 8 | -2.40210724 | -2.23698926 | 2.36985350 |
| 1 | 7.15528345 | -1.55585694 | 1.98478043 |
| 1 | 6.42233419 | -1.32591581 | 3.61718130 |

Electronic energy = -3908.660115 a.u.
DFT-D3(BJ) dispersion correction = -0.147057 a.u.
Thermal free energy = 0.744070 a.u.
Gibbs free energy = -3908.063103 a.u.
Number of imaginary frequencies = 1.

**TS4 eq CL isomer/conformer 14**

| Atomic N. | X | Y | Z |
|---|---|---|---|
| 7 | 0.79521477 | 1.83184266 | 1.20989561 |

| | | | |
|---|---|---|---|
| 8 | -0.02763038 | 0.99439794 | -1.47759163 |
| 8 | 2.90608239 | 0.40299425 | -0.31178102 |
| 6 | -0.33794382 | 1.68945968 | 2.15547681 |
| 6 | -0.88334501 | 2.03062320 | -1.36772072 |
| 6 | -2.03144383 | 2.08952999 | -2.21574998 |
| 6 | -2.89397049 | 3.18353438 | -2.05950999 |
| 6 | -2.66964793 | 4.19850683 | -1.12435091 |
| 6 | -1.53934491 | 4.13815451 | -0.31590566 |
| 6 | -0.64094323 | 3.07284021 | -0.43725669 |
| 6 | 0.62557775 | 3.06676412 | 0.37212852 |
| 1 | -3.37107944 | 5.02833891 | -1.03781199 |
| 6 | -2.30745959 | 1.00074160 | -3.27151346 |
| 6 | 3.66916013 | 1.44849527 | 0.07251371 |
| 6 | 4.86842585 | 1.75569272 | -0.64127946 |
| 6 | 5.63416243 | 2.83745790 | -0.18517607 |
| 6 | 5.26779318 | 3.61529422 | 0.91675276 |
| 6 | 4.09588814 | 3.31115556 | 1.59968734 |
| 6 | 3.30092263 | 2.23241949 | 1.19560552 |
| 6 | 2.07660794 | 1.88709939 | 1.99975860 |
| 1 | 5.89311457 | 4.45110846 | 1.23027027 |
| 6 | 5.30873823 | 0.93742859 | -1.87089956 |
| 6 | -3.59311819 | 1.28413200 | -4.07087803 |
| 6 | -2.48287320 | -0.37732905 | -2.59024239 |
| 6 | -1.13852584 | 0.94015515 | -4.28369617 |
| 6 | 6.63652229 | 1.44708705 | -2.46255755 |
| 6 | 4.24102783 | 1.04322422 | -2.98528695 |
| 6 | 5.52431870 | -0.54203647 | -1.47399414 |
| 1 | -0.40375945 | 2.58469343 | 2.79644084 |
| 1 | -1.27528322 | 1.58388078 | 1.59888029 |
| 1 | 0.65384835 | 3.94839311 | 1.03565562 |
| 1 | 1.50750160 | 3.12026930 | -0.28296354 |
| 1 | -3.77972722 | 3.25195646 | -2.68874073 |
| 1 | -1.33415174 | 4.92839813 | 0.41012308 |
| 1 | 2.17398643 | 0.89574558 | 2.47070003 |
| 1 | 1.95136094 | 2.63104177 | 2.80522919 |
| 1 | 6.55220842 | 3.09281206 | -0.71126145 |
| 1 | 3.78831625 | 3.90514779 | 2.46341205 |
| 1 | -3.73675203 | 0.48488221 | -4.81207132 |
| 1 | -4.48526859 | 1.30374289 | -3.42797041 |
| 1 | -3.53825974 | 2.23618174 | -4.61809731 |
| 1 | -1.57484996 | -0.67612052 | -2.05463648 |
| 1 | -3.32297850 | -0.35252595 | -1.87958670 |
| 1 | -2.70706773 | -1.14255011 | -3.34938192 |
| 1 | -1.03600776 | 1.89899111 | -4.81236219 |
| 1 | -0.18933201 | 0.71508396 | -3.78509808 |
| 1 | -1.33489633 | 0.15946820 | -5.03478336 |
| 1 | 6.90192509 | 0.82624608 | -3.33028293 |
| 1 | 7.46417427 | 1.37917387 | -1.74175549 |
| 1 | 6.56264830 | 2.48717666 | -2.81140661 |
| 1 | 3.26475978 | 0.67959392 | -2.64762998 |
| 1 | 4.12581825 | 2.08741903 | -3.31034827 |
| 1 | 4.55084658 | 0.44968379 | -3.85902572 |
| 1 | 6.32358694 | -0.62773621 | -0.72319633 |

| | | | |
|---|---|---|---|
| 1 | 4.61014032 | -0.98027653 | -1.05957782 |
| 1 | 5.82603788 | -1.12654114 | -2.35672283 |
| 1 | -0.18011487 | 0.80099744 | 2.77400351 |
| 6 | 2.09495163 | -2.85479259 | 1.21946728 |
| 6 | 2.37004542 | -4.19653368 | 0.52910602 |
| 6 | 2.74705291 | -4.13765001 | -0.96009076 |
| 6 | 0.74325228 | -2.21685624 | 0.97524226 |
| 6 | 1.66131938 | -3.66041493 | -1.94131613 |
| 6 | 1.49014878 | -2.14555383 | -2.03784347 |
| 1 | 2.85876012 | -2.10559130 | 0.96814692 |
| 1 | 3.20626688 | -4.67246723 | 1.06539750 |
| 1 | 3.05302143 | -5.15473366 | -1.25414526 |
| 1 | 1.92849934 | -4.00687885 | -2.95355511 |
| 1 | 2.13933659 | -2.99388361 | 2.31483960 |
| 1 | 1.50279748 | -4.86009121 | 0.66593909 |
| 1 | 3.64338350 | -3.50569487 | -1.08473730 |
| 1 | 0.69234228 | -4.12720585 | -1.69918776 |
| 1 | 0.83235955 | -1.90566230 | -2.89259577 |
| 1 | 2.47472739 | -1.68179679 | -2.22953987 |
| 8 | 0.50373501 | -1.00922537 | 1.37825656 |
| 8 | 0.90515953 | -1.59202647 | -0.85955572 |
| 31 | 1.04088223 | 0.20792726 | -0.14058261 |
| 8 | -7.90108633 | -1.17388082 | 4.56783962 |
| 6 | -8.63141823 | -0.96472609 | 5.80068016 |
| 1 | -8.53731060 | -1.84010494 | 6.45543289 |
| 6 | -5.88547993 | -1.56447816 | 3.38395524 |
| 6 | -4.41664791 | -1.97136366 | 3.49704170 |
| 6 | -3.73963404 | -2.10122919 | 2.12864304 |
| 6 | -6.56184483 | -1.38239229 | 4.72937536 |
| 6 | -2.26880264 | -2.52273607 | 2.23430943 |
| 6 | -1.61702633 | -2.62263298 | 0.86162442 |
| 1 | -6.46430445 | -2.30190730 | 2.80583572 |
| 1 | -4.34374380 | -2.92567444 | 4.04154539 |
| 1 | -4.28923035 | -2.83489013 | 1.51359284 |
| 1 | -2.19465232 | -3.49780178 | 2.74189687 |
| 1 | -5.98811769 | -0.61417222 | 2.83339667 |
| 1 | -3.88186550 | -1.23180473 | 4.11175537 |
| 1 | -3.80817437 | -1.13859010 | 1.59291494 |
| 1 | -1.71244955 | -1.79537559 | 2.84466863 |
| 1 | -1.61246991 | -1.65600657 | 0.34324715 |
| 1 | -2.13432598 | -3.35946274 | 0.23158963 |
| 8 | -6.01211357 | -1.40530181 | 5.81427479 |
| 8 | -0.25025198 | -3.12316036 | 0.95045829 |
| 1 | -9.67245102 | -0.81614619 | 5.49840784 |
| 1 | -8.24958324 | -0.08137182 | 6.32783651 |

Electronic energy = -3908.666249 a.u.
DFT-D3(BJ) dispersion correction = -0.138513 a.u.
Thermal free energy = 0.741789 a.u.
Gibbs free energy = -3908.062973 a.u.
Number of imaginary frequencies = 1.

**TS4_eq_CL isomer/conformer 15**

| Atomic N. | X | Y | Z |
|---|---|---|---|
| 6 | -2.69817138 | -0.45012820 | 1.72952676 |
| 6 | -3.55464745 | -1.65735471 | 1.32542849 |
| 6 | -3.79554510 | -1.87388027 | -0.17982079 |
| 6 | -1.20512176 | -0.55319721 | 1.50037038 |
| 6 | -2.58936763 | -2.34244204 | -1.01668501 |
| 6 | -1.69327605 | -1.21810400 | -1.53332853 |
| 1 | -3.03816938 | 0.46671286 | 1.23039186 |
| 1 | -4.53766346 | -1.52587473 | 1.80534101 |
| 1 | -4.58840752 | -2.63411140 | -0.26604208 |
| 1 | -2.95420098 | -2.88685679 | -1.90314770 |
| 1 | -2.80813742 | -0.27905089 | 2.81603265 |
| 1 | -3.12231350 | -2.57087445 | 1.76203179 |
| 1 | -4.21137142 | -0.95307410 | -0.62324071 |
| 1 | -1.98719406 | -3.06065011 | -0.43688160 |
| 1 | -0.87096560 | -1.64712489 | -2.13475776 |
| 1 | -2.28439188 | -0.55481905 | -2.19183612 |
| 8 | -0.45208788 | 0.49217489 | 1.56996894 |
| 8 | -1.15829599 | -0.46019787 | -0.45647156 |
| 7 | 0.90987813 | 2.79650426 | 0.53577965 |
| 8 | -1.12814236 | 2.18609810 | -1.48053420 |
| 8 | 1.50026572 | 0.35369110 | -1.00474608 |
| 6 | 0.22353347 | 3.37577343 | 1.71562994 |
| 6 | -1.29155862 | 3.52579975 | -1.49691904 |
| 6 | -2.49458933 | 4.08195972 | -2.03188729 |
| 6 | -2.62364936 | 5.47758818 | -2.02136564 |
| 6 | -1.63412869 | 6.32698345 | -1.51818752 |
| 6 | -0.46153042 | 5.77525902 | -1.01424146 |
| 6 | -0.27682504 | 4.38843012 | -1.01034093 |
| 6 | 1.03780091 | 3.82000160 | -0.55547601 |
| 1 | -1.78176010 | 7.40686989 | -1.52723718 |
| 6 | -3.60838151 | 3.18694115 | -2.60932469 |
| 6 | 2.73546314 | 0.88505137 | -1.09544206 |
| 6 | 3.62444901 | 0.43650541 | -2.11978602 |
| 6 | 4.90459776 | 1.00551152 | -2.15829468 |
| 6 | 5.32763672 | 1.98232937 | -1.25173736 |
| 6 | 4.45155287 | 2.41403484 | -0.26222125 |
| 6 | 3.16617894 | 1.86796713 | -0.16850844 |
| 6 | 2.26710939 | 2.29963946 | 0.95942628 |
| 1 | 6.33117342 | 2.40102553 | -1.32622719 |
| 6 | 3.19464564 | -0.62853229 | -3.14818931 |
| 6 | -4.79798079 | 4.00916386 | -3.13919163 |
| 6 | -4.15478086 | 2.24375892 | -1.51204991 |
| 6 | -3.05725312 | 2.35931277 | -3.79489136 |
| 6 | 4.32570696 | -0.96257693 | -4.13903904 |
| 6 | 1.99491596 | -0.11085563 | -3.97677779 |
| 6 | 2.81163859 | -1.94394767 | -2.42886066 |
| 1 | 0.78214735 | 4.25453568 | 2.07926869 |
| 1 | -0.78882504 | 3.68933511 | 1.43956244 |
| 1 | 1.69452453 | 4.63231850 | -0.19898933 |
| 1 | 1.55750108 | 3.32199335 | -1.38742447 |
| 1 | -3.53242779 | 5.92646408 | -2.41834879 |
| 1 | 0.33327761 | 6.41882420 | -0.62988800 |

| | | | |
|---|---|---|---|
| 1 | 2.07152295 | 1.46878552 | 1.65602791 |
| 1 | 2.76358843 | 3.10431433 | 1.52873611 |
| 1 | 5.60304070 | 0.68290079 | -2.92837143 |
| 1 | 4.76064920 | 3.17669177 | 0.45641902 |
| 1 | -5.55490303 | 3.32221508 | -3.54429126 |
| 1 | -5.27878380 | 4.60147905 | -2.34711099 |
| 1 | -4.50135660 | 4.68922949 | -3.95058441 |
| 1 | -3.36495090 | 1.59420705 | -1.11926281 |
| 1 | -4.57985640 | 2.82428312 | -0.67969787 |
| 1 | -4.95504475 | 1.61121321 | -1.92635226 |
| 1 | -2.72251034 | 3.02377915 | -4.60469437 |
| 1 | -2.21123433 | 1.73422050 | -3.48885608 |
| 1 | -3.84873939 | 1.70849895 | -4.19757891 |
| 1 | 3.96956038 | -1.72715473 | -4.84424162 |
| 1 | 5.21324539 | -1.36818969 | -3.63202691 |
| 1 | 4.63185644 | -0.08666375 | -4.72908020 |
| 1 | 1.13127077 | 0.11125007 | -3.34080410 |
| 1 | 2.26766682 | 0.80365312 | -4.52327013 |
| 1 | 1.69623029 | -0.86996609 | -4.71582890 |
| 1 | 3.66999078 | -2.34188437 | -1.86775005 |
| 1 | 1.97905183 | -1.79062164 | -1.73375309 |
| 1 | 2.51422691 | -2.70086288 | -3.17070341 |
| 1 | 0.16499877 | 2.61903071 | 2.50365734 |
| 31 | -0.07800871 | 1.12774062 | -0.32828683 |
| 8 | 0.60733557 | -7.10146379 | 6.24217701 |
| 6 | 0.76302505 | -8.53918362 | 6.16692066 |
| 1 | 0.10041883 | -8.95736313 | 5.39875126 |
| 6 | 0.69287342 | -4.94525051 | 5.26130342 |
| 6 | 1.15353715 | -4.12490845 | 4.05665159 |
| 6 | 0.88624704 | -2.62716961 | 4.24391603 |
| 6 | 0.90621394 | -6.43683147 | 5.08754778 |
| 6 | 1.39770424 | -1.74352312 | 3.09574461 |
| 6 | 0.72095317 | -1.97140431 | 1.74856317 |
| 1 | -0.38123566 | -4.78620577 | 5.45534229 |
| 1 | 0.64452136 | -4.50282955 | 3.15822387 |
| 1 | -0.19534862 | -2.45980883 | 4.37714911 |
| 1 | 2.47655463 | -1.92033446 | 2.94407749 |
| 1 | 1.20727158 | -4.63029909 | 6.18303776 |
| 1 | 2.22918677 | -4.29579926 | 3.89083195 |
| 1 | 1.36481655 | -2.28865027 | 5.17785501 |
| 1 | 1.28707254 | -0.68331194 | 3.36801863 |
| 1 | 0.82068974 | -3.01316929 | 1.41722918 |
| 1 | 1.13579440 | -1.31572104 | 0.97276968 |
| 8 | 1.28256798 | -6.99141979 | 4.07251978 |
| 8 | -0.72581810 | -1.76600063 | 1.82535124 |
| 1 | 0.49058157 | -8.91194916 | 7.15893507 |
| 1 | 1.80068898 | -8.80045795 | 5.92436886 |

Electronic energy = -3908.663908 a.u.
DFT-D3(BJ) dispersion correction = -0.139039 a.u.
Thermal free energy = 0.739991 a.u.
Gibbs free energy = -3908.062956 a.u.
Number of imaginary frequencies = 1.

**Int4_eq CL isomer/conformer 1**

| Atomic N. | X | Y | Z |
|---|---|---|---|
| 7 | 0.07282277 | 2.44047158 | 2.35206369 |
| 8 | -0.43604809 | 1.91359168 | -0.47405512 |
| 8 | 2.42572817 | 1.38129214 | 0.89927987 |
| 6 | -1.14486283 | 2.13814140 | 3.14444725 |
| 6 | -1.37117082 | 2.87091942 | -0.32745843 |
| 6 | -2.45700895 | 2.95338288 | -1.25216232 |
| 6 | -3.39664418 | 3.97459341 | -1.05402924 |
| 6 | -3.30911703 | 4.89309448 | -0.00297805 |
| 6 | -2.24585294 | 4.80372144 | 0.88973223 |
| 6 | -1.27550811 | 3.80933869 | 0.73018444 |
| 6 | -0.08612082 | 3.76038602 | 1.64498300 |
| 1 | -4.06623898 | 5.66896308 | 0.11049928 |
| 6 | -2.59091965 | 1.95915276 | -2.42178541 |
| 6 | 3.06667065 | 2.40515570 | 1.50154710 |
| 6 | 4.30542119 | 2.87986263 | 0.97004595 |
| 6 | 4.94110627 | 3.92877861 | 1.64840886 |
| 6 | 4.41013289 | 4.52128341 | 2.79750207 |
| 6 | 3.19958246 | 4.05790070 | 3.29963295 |
| 6 | 2.52989399 | 3.00186891 | 2.67088918 |
| 6 | 1.25793499 | 2.47959125 | 3.28391664 |
| 1 | 4.93832381 | 5.33952328 | 3.28678634 |
| 6 | 4.92279680 | 2.27287262 | -0.30512080 |
| 6 | -3.83136799 | 2.24851601 | -3.28750692 |
| 6 | -2.73402685 | 0.51657332 | -1.87896888 |
| 6 | -1.35203167 | 2.05537526 | -3.34360655 |
| 6 | 6.26434093 | 2.93529278 | -0.67184776 |
| 6 | 3.96693971 | 2.47956241 | -1.50374883 |
| 6 | 5.19971116 | 0.76531793 | -0.09777186 |
| 1 | -1.34246139 | 2.96285255 | 3.84924581 |
| 1 | -2.00616183 | 2.03197601 | 2.47633123 |
| 1 | -0.16057773 | 4.55365926 | 2.40820758 |
| 1 | 0.84613556 | 3.92725590 | 1.08623360 |
| 1 | -4.23595417 | 4.06074796 | -1.74214641 |
| 1 | -2.15136773 | 5.51542479 | 1.71337911 |
| 1 | 1.38074067 | 1.44753812 | 3.64671776 |
| 1 | 0.98849729 | 3.11177670 | 4.14708919 |
| 1 | 5.88647023 | 4.30851595 | 1.26518577 |
| 1 | 2.76336364 | 4.50745387 | 4.19470980 |
| 1 | -3.87493661 | 1.51621503 | -4.10651633 |
| 1 | -4.76639898 | 2.16129318 | -2.71509361 |
| 1 | -3.79387301 | 3.24945095 | -3.74103660 |
| 1 | -1.85963729 | 0.22149384 | -1.28758277 |
| 1 | -3.63313213 | 0.43011227 | -1.25007774 |
| 1 | -2.84102705 | -0.18812395 | -2.71830137 |
| 1 | -1.27113645 | 3.06339527 | -3.77584405 |
| 1 | -0.42838219 | 1.83689076 | -2.79673365 |
| 1 | -1.44718732 | 1.33843388 | -4.17374054 |
| 1 | 6.65830668 | 2.46216571 | -1.58272683 |
| 1 | 7.01802326 | 2.80933056 | 0.11896209 |
| 1 | 6.15340499 | 4.00928273 | -0.87959286 |

| | | | |
|---|---|---|---|
| 1 | 2.99090122 | 2.01421193 | -1.32941107 |
| 1 | 3.80949895 | 3.55169443 | -1.69096957 |
| 1 | 4.40469480 | 2.03861912 | -2.41235875 |
| 1 | 5.91548249 | 0.61319198 | 0.72349640 |
| 1 | 4.27865682 | 0.22139384 | 0.13695172 |
| 1 | 5.63791554 | 0.33639935 | -1.01196899 |
| 1 | -0.99183502 | 1.20699654 | 3.69672344 |
| 6 | 1.85579142 | -2.09465790 | 2.08796836 |
| 6 | 2.19895797 | -3.26159970 | 1.14559042 |
| 6 | 2.61717999 | -2.90748537 | -0.29521030 |
| 6 | 0.44296761 | -1.56664857 | 2.06819757 |
| 6 | 1.52528941 | -2.45225759 | -1.28368693 |
| 6 | 1.16689018 | -0.96754499 | -1.22452048 |
| 1 | 2.52116184 | -1.23577739 | 1.92784392 |
| 1 | 3.04395655 | -3.79962249 | 1.60352134 |
| 1 | 3.08736821 | -3.81417791 | -0.70963609 |
| 1 | 1.87326723 | -2.65892254 | -2.30997511 |
| 1 | 2.01252645 | -2.42175214 | 3.13328736 |
| 1 | 1.36248475 | -3.97652770 | 1.12666559 |
| 1 | 3.41550342 | -2.14626194 | -0.26440624 |
| 1 | 0.60967290 | -3.05048239 | -1.13986254 |
| 1 | 0.47566162 | -0.72308625 | -2.05338746 |
| 1 | 2.08731869 | -0.37145075 | -1.37534304 |
| 8 | 0.13209596 | -0.40137003 | 2.43585578 |
| 8 | 0.56015137 | -0.66767319 | 0.02336074 |
| 31 | 0.57786332 | 1.02375896 | 0.85459353 |
| 8 | -4.07741153 | -7.20990732 | -3.02841586 |
| 6 | -4.90987863 | -8.33362629 | -3.40368082 |
| 1 | -4.71002239 | -9.19384694 | -2.75245490 |
| 6 | -3.34926146 | -5.59289364 | -1.45649589 |
| 6 | -3.51955159 | -5.03418953 | -0.04438657 |
| 6 | -2.58307722 | -3.85409361 | 0.23190467 |
| 6 | -4.26424008 | -6.76600583 | -1.75107845 |
| 6 | -2.75184502 | -3.28836833 | 1.64628670 |
| 6 | -1.87813077 | -2.08176188 | 1.94118071 |
| 1 | -2.31225310 | -5.92404526 | -1.62985731 |
| 1 | -3.33935860 | -5.83704085 | 0.68715278 |
| 1 | -1.53717181 | -4.16910324 | 0.08835222 |
| 1 | -2.55504007 | -4.07195977 | 2.39597958 |
| 1 | -3.53929991 | -4.81772962 | -2.21653661 |
| 1 | -4.56672265 | -4.72548551 | 0.09929342 |
| 1 | -2.76803201 | -3.05134409 | -0.50231969 |
| 1 | -3.79762598 | -2.97093892 | 1.79527442 |
| 1 | -2.09824824 | -1.64866527 | 2.92659400 |
| 1 | -1.98696411 | -1.30015157 | 1.17557436 |
| 8 | -5.06131355 | -7.26275668 | -0.97793304 |
| 8 | -0.48165725 | -2.51946128 | 1.93399545 |
| 1 | -4.63998177 | -8.56027761 | -4.43953588 |
| 1 | -5.97153127 | -8.06697940 | -3.32776075 |

Electronic energy = -3908.668063 a.u.
DFT-D3(BJ) dispersion correction = -0.138760 a.u.
Thermal free energy = 0.736972 a.u.
Gibbs free energy = -3908.069850 a.u.

Number of imaginary frequencies = 0.

**Int4_eq_CL isomer/conformer 2**

| Atomic N. | X | Y | Z |
|---|---|---|---|
| 7 | 0.62391373 | 2.39005375 | 1.67521983 |
| 8 | -0.38788499 | 1.52826688 | -0.92450853 |
| 8 | 2.75185499 | 1.55616007 | -0.20769855 |
| 6 | -0.32730286 | 1.98641589 | 2.74001734 |
| 6 | -1.41335944 | 2.34797686 | -0.62675688 |
| 6 | -2.67002318 | 2.18431417 | -1.28638100 |
| 6 | -3.70044174 | 3.06934372 | -0.93994617 |
| 6 | -3.54253469 | 4.08353822 | 0.01003404 |
| 6 | -2.31277166 | 4.23544416 | 0.64256065 |
| 6 | -1.24883879 | 3.38506352 | 0.32515287 |
| 6 | 0.10084889 | 3.59919392 | 0.94702295 |
| 1 | -4.37488362 | 4.74627595 | 0.24685083 |
| 6 | -2.88494341 | 1.07971984 | -2.33907516 |
| 6 | 3.32819531 | 2.71627178 | 0.17074519 |
| 6 | 4.32751396 | 3.31643237 | -0.65602728 |
| 6 | 4.91121891 | 4.50797109 | -0.20432974 |
| 6 | 4.54899614 | 5.12202914 | 0.99788108 |
| 6 | 3.56901954 | 4.53408932 | 1.78929752 |
| 6 | 2.96445270 | 3.33531464 | 1.39376653 |
| 6 | 1.95869826 | 2.69015454 | 2.30934292 |
| 1 | 5.02694079 | 6.05240400 | 1.30429273 |
| 6 | 4.74767226 | 2.68886051 | -1.99965740 |
| 6 | -4.31168862 | 1.10295335 | -2.91858183 |
| 6 | -2.66935023 | -0.31366220 | -1.70044659 |
| 6 | -1.90397531 | 1.27501508 | -3.51955703 |
| 6 | 5.85118588 | 3.50376436 | -2.70044192 |
| 6 | 3.53547009 | 2.63873881 | -2.95950979 |
| 6 | 5.30704883 | 1.26497696 | -1.77160645 |
| 1 | -0.50226511 | 2.83527326 | 3.42187631 |
| 1 | -1.28242322 | 1.69108093 | 2.29282489 |
| 1 | 0.06211002 | 4.44136852 | 1.65885906 |
| 1 | 0.85010204 | 3.84898030 | 0.18190075 |
| 1 | -4.66971978 | 2.96725302 | -1.42519470 |
| 1 | -2.16231131 | 5.02693000 | 1.38064206 |
| 1 | 2.32525124 | 1.72551015 | 2.69302876 |
| 1 | 1.78163709 | 3.35269297 | 3.17370302 |
| 1 | 5.67567849 | 4.98534876 | -0.81465188 |
| 1 | 3.26735868 | 4.99705955 | 2.73175800 |
| 1 | -4.40775173 | 0.30150582 | -3.66513810 |
| 1 | -5.07592065 | 0.93027383 | -2.14672664 |
| 1 | -4.53678844 | 2.05340783 | -3.42347421 |
| 1 | -1.65162255 | -0.42158446 | -1.30804962 |
| 1 | -3.38531268 | -0.47519554 | -0.88002322 |
| 1 | -2.83731984 | -1.09878271 | -2.45383749 |
| 1 | -2.08099281 | 2.24261802 | -4.01134826 |
| 1 | -0.86202414 | 1.24210071 | -3.18307404 |
| 1 | -2.05692629 | 0.48221276 | -4.26801239 |
| 1 | 6.11256695 | 3.00952766 | -3.64710698 |

| | | | |
|---|---|---|---|
| 1 | 6.76741795 | 3.56573559 | -2.09548539 |
| 1 | 5.52293667 | 4.52511391 | -2.94136364 |
| 1 | 2.70924951 | 2.05543901 | -2.53910915 |
| 1 | 3.16883208 | 3.65376977 | -3.17072640 |
| 1 | 3.83290846 | 2.18107184 | -3.91536950 |
| 1 | 6.19777707 | 1.29830285 | -1.12705208 |
| 1 | 4.56071610 | 0.61574663 | -1.30180477 |
| 1 | 5.60300371 | 0.82088259 | -2.73436850 |
| 1 | 0.09283038 | 1.14423714 | 3.29654729 |
| 6 | 3.00973443 | -1.84920080 | 1.28593047 |
| 6 | 3.34068602 | -3.03828171 | 0.36769143 |
| 6 | 3.38626997 | -2.76829324 | -1.14912381 |
| 6 | 1.55457782 | -1.53197169 | 1.52454166 |
| 6 | 2.05248119 | -2.56835803 | -1.89609027 |
| 6 | 1.48765877 | -1.14878667 | -1.85577730 |
| 1 | 3.48656117 | -0.92302231 | 0.93792502 |
| 1 | 4.33961511 | -3.39699044 | 0.66220643 |
| 1 | 3.89636519 | -3.63526753 | -1.59963195 |
| 1 | 2.20337363 | -2.82484873 | -2.95832741 |
| 1 | 3.41753477 | -2.05142420 | 2.29437554 |
| 1 | 2.64875784 | -3.86772795 | 0.57778931 |
| 1 | 4.04219372 | -1.90257252 | -1.34464712 |
| 1 | 1.29343988 | -3.27036086 | -1.51156308 |
| 1 | 0.61054652 | -1.08274107 | -2.52722113 |
| 1 | 2.25261152 | -0.44761251 | -2.24076832 |
| 8 | 1.14278888 | -0.39605289 | 1.89119811 |
| 8 | 1.11126586 | -0.81688734 | -0.52783095 |
| 31 | 1.02170860 | 0.92690343 | 0.18722412 |
| 8 | -7.17634522 | -5.88493870 | 2.38386452 |
| 6 | -7.97342765 | -7.09389731 | 2.40378469 |
| 1 | -7.84860092 | -7.65100797 | 1.46683329 |
| 6 | -5.09037236 | -4.76989567 | 2.24165745 |
| 6 | -3.58495176 | -4.92632223 | 2.03098538 |
| 6 | -2.85084410 | -3.58170431 | 2.05496593 |
| 6 | -5.83460176 | -6.09175640 | 2.24353839 |
| 6 | -1.34004336 | -3.73076891 | 1.83749230 |
| 6 | -0.63933141 | -2.38398369 | 1.86862401 |
| 1 | -5.53814274 | -4.12997567 | 1.46438641 |
| 1 | -3.40274329 | -5.43450721 | 1.07173529 |
| 1 | -3.26893787 | -2.91918063 | 1.27778216 |
| 1 | -1.14812685 | -4.21409771 | 0.86687175 |
| 1 | -5.30644010 | -4.26478863 | 3.19764486 |
| 1 | -3.17647883 | -5.59102556 | 2.80731314 |
| 1 | -3.03655330 | -3.08036217 | 3.02086499 |
| 1 | -0.91191222 | -4.38498520 | 2.61352882 |
| 1 | -0.76015453 | -1.88051847 | 2.83862395 |
| 1 | -1.00198479 | -1.71562071 | 1.07463149 |
| 8 | -5.33197091 | -7.19451954 | 2.14012068 |
| 8 | 0.78443563 | -2.61606179 | 1.63698349 |
| 1 | -9.00774827 | -6.75636982 | 2.51966213 |
| 1 | -7.67692392 | -7.73517607 | 3.24332077 |

Electronic energy = -3908.667895 a.u.
DFT-D3(BJ) dispersion correction = -0.137805 a.u.

Thermal free energy = 0.736146 a.u.
Gibbs free energy = -3908.069554 a.u.
Number of imaginary frequencies = 0.

**Int4_eq_CL isomer/conformer 3**

| Atomic N. | X | Y | Z |
|---|---|---|---|
| 7 | 0.59848579 | 2.36176292 | 1.73523956 |
| 8 | -0.37127515 | 1.54590951 | -0.89460446 |
| 8 | 2.75601055 | 1.55596468 | -0.12514053 |
| 6 | -0.37218611 | 1.94207433 | 2.77598718 |
| 6 | -1.39958104 | 2.36341894 | -0.60049192 |
| 6 | -2.64565739 | 2.21347645 | -1.28307485 |
| 6 | -3.67894076 | 3.09677196 | -0.94078764 |
| 6 | -3.53370094 | 4.09626448 | 0.02663940 |
| 6 | -2.31442665 | 4.23420953 | 0.68224964 |
| 6 | -1.24803188 | 3.38494230 | 0.37019440 |
| 6 | 0.09131576 | 3.58457103 | 1.01890347 |
| 1 | -4.36775266 | 4.75833214 | 0.25930960 |
| 6 | -2.84643005 | 1.12498616 | -2.35514209 |
| 6 | 3.33002630 | 2.70667415 | 0.28440289 |
| 6 | 4.34609035 | 3.31787394 | -0.51328130 |
| 6 | 4.92597603 | 4.49862048 | -0.02940850 |
| 6 | 4.54458005 | 5.09200650 | 1.17724836 |
| 6 | 3.54837534 | 4.49366202 | 1.94011360 |
| 6 | 2.94669921 | 3.30470919 | 1.51193002 |
| 6 | 1.92264133 | 2.64693218 | 2.39787333 |
| 1 | 5.02019862 | 6.01483683 | 1.50905166 |
| 6 | 4.78765491 | 2.71359636 | -1.86071570 |
| 6 | -4.26366785 | 1.16005627 | -2.95664366 |
| 6 | -2.64416048 | -0.27807948 | -1.73339059 |
| 6 | -1.84635191 | 1.33470356 | -3.51703992 |
| 6 | 5.90632597 | 3.53695377 | -2.52670966 |
| 6 | 3.59236617 | 2.68622190 | -2.84243247 |
| 6 | 5.33753211 | 1.28347182 | -1.64933801 |
| 1 | -0.55738637 | 2.77932486 | 3.46938548 |
| 1 | -1.31990817 | 1.65674863 | 2.30707942 |
| 1 | 0.04219165 | 4.41465618 | 1.74419661 |
| 1 | 0.85452119 | 3.84538570 | 0.27143032 |
| 1 | -4.64033742 | 3.00513768 | -1.44349443 |
| 1 | -2.17411549 | 5.01370800 | 1.43494244 |
| 1 | 2.27960350 | 1.67466183 | 2.77125207 |
| 1 | 1.73214268 | 3.29491500 | 3.27037765 |
| 1 | 5.70289439 | 4.98413799 | -0.61715076 |
| 1 | 3.23158978 | 4.94053836 | 2.88537465 |
| 1 | -4.34984755 | 0.36943566 | -3.71584487 |
| 1 | -5.04036537 | 0.97843132 | -2.19940871 |
| 1 | -4.47861450 | 2.11810990 | -3.45149122 |
| 1 | -1.63242499 | -0.39483696 | -1.32824212 |
| 1 | -3.37214410 | -0.44878676 | -0.92548591 |
| 1 | -2.80389906 | -1.05196016 | -2.50007996 |
| 1 | -2.01521883 | 2.30855710 | -3.99927657 |
| 1 | -0.80993244 | 1.29675704 | -3.16443587 |

| | | | |
|---|---|---|---|
| 1 | -1.98782042 | 0.55164886 | -4.27791139 |
| 1 | 6.18257764 | 3.05935852 | -3.47768782 |
| 1 | 6.81195684 | 3.58411201 | -1.90469745 |
| 1 | 5.58604078 | 4.56387384 | -2.75431028 |
| 1 | 2.75677667 | 2.09843724 | -2.44754380 |
| 1 | 3.23326118 | 3.70649298 | -3.04096458 |
| 1 | 3.90492577 | 2.24536757 | -3.80136689 |
| 1 | 6.21676415 | 1.30118299 | -0.98857942 |
| 1 | 4.58050136 | 0.62871897 | -1.20499097 |
| 1 | 5.64884719 | 0.85604479 | -2.61479734 |
| 1 | 0.03631775 | 1.08932287 | 3.32505075 |
| 6 | 2.98366300 | -1.87347859 | 1.31272618 |
| 6 | 3.32967664 | -3.04707054 | 0.38023438 |
| 6 | 3.40225157 | -2.75119122 | -1.13067323 |
| 6 | 1.52469767 | -1.55921318 | 1.53112556 |
| 6 | 2.08204779 | -2.53646809 | -1.89744360 |
| 6 | 1.51762912 | -1.11723528 | -1.84192775 |
| 1 | 3.46700325 | -0.94191247 | 0.98895833 |
| 1 | 4.32288595 | -3.41208745 | 0.68611006 |
| 1 | 3.91914936 | -3.61104478 | -1.58703479 |
| 1 | 2.25165190 | -2.77409489 | -2.96124794 |
| 1 | 3.37354077 | -2.09328227 | 2.32457247 |
| 1 | 2.63333277 | -3.87908857 | 0.56383015 |
| 1 | 4.06248292 | -1.88318495 | -1.29961562 |
| 1 | 1.31598503 | -3.24466950 | -1.53910283 |
| 1 | 0.65229037 | -1.03845311 | -2.52715872 |
| 1 | 2.28964985 | -0.41025483 | -2.20139648 |
| 8 | 1.10719537 | -0.42949914 | 1.91072302 |
| 8 | 1.11869824 | -0.80796988 | -0.51510765 |
| 31 | 1.01868495 | 0.92303778 | 0.22951295 |
| 8 | -7.22606583 | -5.91959127 | 2.09642044 |
| 6 | -8.02393580 | -7.12777283 | 2.06422561 |
| 1 | -7.87867267 | -7.65802977 | 1.11471463 |
| 6 | -5.13701207 | -4.80313324 | 2.03033437 |
| 6 | -3.62670393 | -4.95609188 | 1.85527149 |
| 6 | -2.89295386 | -3.61290625 | 1.92647204 |
| 6 | -5.88164193 | -6.12381579 | 1.98001235 |
| 6 | -1.37744990 | -3.75944099 | 1.74256355 |
| 6 | -0.67569533 | -2.41509226 | 1.82057574 |
| 1 | -5.56374750 | -4.14428817 | 1.25699234 |
| 1 | -3.42009810 | -5.44379884 | 0.89034960 |
| 1 | -3.29124110 | -2.93384289 | 1.15300146 |
| 1 | -1.16292470 | -4.22066486 | 0.76591920 |
| 1 | -5.37805310 | -4.32078639 | 2.99203877 |
| 1 | -3.23833443 | -5.63759883 | 2.62734419 |
| 1 | -3.10215724 | -3.13209814 | 2.89822010 |
| 1 | -0.96879778 | -4.43218854 | 2.51323224 |
| 1 | -0.81388484 | -1.93536564 | 2.80027711 |
| 1 | -1.02213108 | -1.72694540 | 1.03638496 |
| 8 | -5.37736074 | -7.22382166 | 1.85714204 |
| 8 | 0.75204094 | -2.64431767 | 1.61086998 |
| 1 | -9.06040867 | -6.79257108 | 2.16686293 |
| 1 | -7.74630029 | -7.79308258 | 2.89141959 |

Electronic energy = -3908.667916 a.u.
DFT-D3(BJ) dispersion correction = -0.137828 a.u.
Thermal free energy = 0.736714 a.u.
Gibbs free energy = -3908.069030 a.u.
Number of imaginary frequencies = 0.

**Int4 eq CL isomer/conformer 4**

| Atomic N. | X | Y | Z |
|---|---|---|---|
| 7 | 0.29642115 | 2.65191535 | 1.43041206 |
| 8 | -0.18281116 | 2.02072870 | -1.38018395 |
| 8 | 2.57761486 | 1.30341676 | 0.11044419 |
| 6 | -0.96443432 | 2.50483434 | 2.19863732 |
| 6 | -1.02719617 | 3.06749748 | -1.31372530 |
| 6 | -2.07224471 | 3.20123913 | -2.27852279 |
| 6 | -2.91741923 | 4.31367275 | -2.16345728 |
| 6 | -2.77533240 | 5.27420823 | -1.15689132 |
| 6 | -1.75149706 | 5.13477878 | -0.22542807 |
| 6 | -0.87433731 | 4.04819675 | -0.30211158 |
| 6 | 0.27954197 | 3.94057830 | 0.65236068 |
| 1 | -3.45959537 | 6.12126644 | -1.10795545 |
| 6 | -2.26339370 | 2.16478257 | -3.40263141 |
| 6 | 3.29404411 | 2.29753129 | 0.67613290 |
| 6 | 4.58303944 | 2.63046202 | 0.15655575 |
| 6 | 5.29468164 | 3.65377642 | 0.79763599 |
| 6 | 4.79185011 | 4.35287433 | 1.89852779 |
| 6 | 3.53222187 | 4.02770337 | 2.38846530 |
| 6 | 2.78509900 | 3.00298955 | 1.79629925 |
| 6 | 1.45494585 | 2.63155644 | 2.39552894 |
| 1 | 5.37997368 | 5.14620570 | 2.35967402 |
| 6 | 5.17283788 | 1.90331594 | -1.06779547 |
| 6 | -3.44474916 | 2.52231226 | -4.32381980 |
| 6 | -2.55671713 | 0.77096321 | -2.79692283 |
| 6 | -0.99370277 | 2.09950016 | -4.28444860 |
| 6 | 6.57666382 | 2.42338829 | -1.43064769 |
| 6 | 4.26735947 | 2.12916433 | -2.30157531 |
| 6 | 5.30801378 | 0.39057601 | -0.77512451 |
| 1 | -1.10345091 | 3.38078710 | 2.85394926 |
| 1 | -1.81391405 | 2.44152535 | 1.51025790 |
| 1 | 0.25773440 | 4.77756217 | 1.37100363 |
| 1 | 1.23822631 | 3.99183209 | 0.11622134 |
| 1 | -3.72414540 | 4.44048266 | -2.88348988 |
| 1 | -1.61442553 | 5.87749725 | 0.56415735 |
| 1 | 1.47224113 | 1.61279595 | 2.81249011 |
| 1 | 1.22294530 | 3.32981333 | 3.21772765 |
| 1 | 6.27947000 | 3.92639772 | 0.42260222 |
| 1 | 3.11673102 | 4.56286339 | 3.24552243 |
| 1 | -3.53191598 | 1.75602792 | -5.10743585 |
| 1 | -4.40060610 | 2.55044914 | -3.78058171 |
| 1 | -3.30036374 | 3.49109418 | -4.82346437 |
| 1 | -1.73038356 | 0.42668745 | -2.16449409 |
| 1 | -3.47707015 | 0.79937202 | -2.19414171 |
| 1 | -2.70610230 | 0.03773823 | -3.60447230 |

| | | | |
|---|---|---|---|
| 1 | -0.80494494 | 3.07271881 | -4.76063041 |
| 1 | -0.11170622 | 1.82379836 | -3.69618946 |
| 1 | -1.13119569 | 1.35368654 | -5.08253615 |
| 1 | 6.94742450 | 1.86869528 | -2.30450555 |
| 1 | 7.29745849 | 2.27407361 | -0.61367558 |
| 1 | 6.56764067 | 3.49017428 | -1.69680581 |
| 1 | 3.24920153 | 1.76393640 | -2.12977387 |
| 1 | 4.21276408 | 3.19928373 | -2.54892251 |
| 1 | 4.68340589 | 1.60159588 | -3.17344180 |
| 1 | 5.98914164 | 0.22002081 | 0.07160838 |
| 1 | 4.33673678 | -0.05572601 | -0.53693659 |
| 1 | 5.72542795 | -0.12453981 | -1.65385509 |
| 1 | -0.91299545 | 1.59464610 | 2.80233918 |
| 6 | 1.65926301 | -2.02307457 | 1.45815675 |
| 6 | 1.93340402 | -3.27177832 | 0.60250150 |
| 6 | 2.41759304 | -3.04609173 | -0.84332515 |
| 6 | 0.29961239 | -1.37972467 | 1.34236331 |
| 6 | 1.39570007 | -2.55564361 | -1.88836941 |
| 6 | 1.17715448 | -1.04349192 | -1.92867631 |
| 1 | 2.40338964 | -1.23583484 | 1.27714995 |
| 1 | 2.71918213 | -3.84415325 | 1.12048274 |
| 1 | 2.81288765 | -4.01518420 | -1.18904569 |
| 1 | 1.74625475 | -2.85711357 | -2.88991127 |
| 1 | 1.74584214 | -2.29580203 | 2.52703319 |
| 1 | 1.04224355 | -3.91748329 | 0.59971012 |
| 1 | 3.28101863 | -2.35893116 | -0.83361956 |
| 1 | 0.42519898 | -3.05704423 | -1.73498310 |
| 1 | 0.53688092 | -0.78722436 | -2.79396839 |
| 1 | 2.15345781 | -0.54452051 | -2.07999374 |
| 8 | 0.07388580 | -0.17489024 | 1.64342520 |
| 8 | 0.56329661 | -0.61225576 | -0.72324751 |
| 31 | 0.70580895 | 1.11473700 | 0.02335277 |
| 8 | -5.20617169 | -7.80958278 | 4.04549993 |
| 6 | -6.11805512 | -8.93126776 | 3.95875934 |
| 1 | -7.14586547 | -8.57922220 | 3.80512252 |
| 6 | -4.18999895 | -5.87789878 | 3.12266577 |
| 6 | -4.10028827 | -4.95049769 | 1.91145891 |
| 6 | -3.10337756 | -3.80715891 | 2.12500817 |
| 6 | -5.17117683 | -7.01906068 | 2.93300060 |
| 6 | -3.00592608 | -2.87446970 | 0.91261035 |
| 6 | -2.04689308 | -1.71198453 | 1.09835809 |
| 1 | -4.49044274 | -5.32379313 | 4.02700838 |
| 1 | -5.09916862 | -4.54332674 | 1.69083163 |
| 1 | -3.39925119 | -3.21910942 | 3.01162759 |
| 1 | -3.99708614 | -2.44360226 | 0.69345949 |
| 1 | -3.20698626 | -6.31597007 | 3.36084585 |
| 1 | -3.81246983 | -5.53767509 | 1.02590714 |
| 1 | -2.10671758 | -4.22215723 | 2.34630092 |
| 1 | -2.70964573 | -3.44455745 | 0.01764338 |
| 1 | -2.26982529 | -1.13693453 | 2.00869629 |
| 1 | -2.05326345 | -1.03225024 | 0.23508086 |
| 8 | -5.84467010 | -7.22515715 | 1.94146896 |
| 8 | -0.69533578 | -2.25981328 | 1.21462478 |

| | | | |
|---|---|---|---|
| 1 | -6.02578724 | -9.45363693 | 4.91575028 |
| 1 | -5.83787939 | -9.59009302 | 3.12727580 |

Electronic energy = -3908.667798 a.u.
DFT-D3(BJ) dispersion correction = -0.137694 a.u.
Thermal free energy = 0.736900 a.u.
Gibbs free energy = -3908.068592 a.u.
Number of imaginary frequencies = 0.

**Int4 eq CL isomer/conformer 5**

| Atomic N. | X | Y | Z |
|---|---|---|---|
| 6 | -3.58468572 | 0.23213278 | 1.56396984 |
| 6 | -4.40926268 | -0.85148638 | 0.84758037 |
| 6 | -4.31637335 | -0.92204163 | -0.69062553 |
| 6 | -2.12989812 | -0.05987921 | 1.84116967 |
| 6 | -3.04810983 | -1.54252712 | -1.31216153 |
| 6 | -1.89196710 | -0.56456870 | -1.52063731 |
| 1 | -3.62245100 | 1.18685530 | 1.02409468 |
| 1 | -5.46417204 | -0.65965545 | 1.10061379 |
| 1 | -5.18016596 | -1.52139892 | -1.02106785 |
| 1 | -3.30245925 | -1.96334457 | -2.29931860 |
| 1 | -4.02938551 | 0.41491354 | 2.56032176 |
| 1 | -4.17066853 | -1.83543402 | 1.27912845 |
| 1 | -4.47334046 | 0.08512243 | -1.11326123 |
| 1 | -2.70332202 | -2.38705161 | -0.69223010 |
| 1 | -1.04324207 | -1.09301384 | -1.99672542 |
| 1 | -2.21275466 | 0.23816570 | -2.21109597 |
| 8 | -1.26166059 | 0.84252246 | 2.00525889 |
| 8 | -1.51051986 | -0.02789913 | -0.26858689 |
| 7 | 0.60592262 | 2.88019391 | 1.29860319 |
| 8 | -0.80283801 | 2.55631365 | -1.23859289 |
| 8 | 1.28113581 | 0.35514502 | -0.03170265 |
| 6 | -0.30742253 | 3.63066074 | 2.19490974 |
| 6 | -0.70681911 | 3.90089668 | -1.29011733 |
| 6 | -1.58304490 | 4.64173731 | -2.14282696 |
| 6 | -1.44331516 | 6.03624636 | -2.16091073 |
| 6 | -0.49629072 | 6.71437287 | -1.38805973 |
| 6 | 0.35416265 | 5.98324064 | -0.56617395 |
| 6 | 0.26414413 | 4.58800574 | -0.51909274 |
| 6 | 1.24843083 | 3.81095451 | 0.30732243 |
| 1 | -0.42781517 | 7.80123019 | -1.43345820 |
| 6 | -2.64187931 | 3.94003835 | -3.01477957 |
| 6 | 2.54357732 | 0.66775163 | 0.31663579 |
| 6 | 3.63712079 | 0.04803249 | -0.36306495 |
| 6 | 4.93193760 | 0.39027179 | 0.04944484 |
| 6 | 5.18276774 | 1.30642831 | 1.07551478 |
| 6 | 4.11029384 | 1.90857495 | 1.72405385 |
| 6 | 2.79588282 | 1.59036076 | 1.36333531 |
| 6 | 1.65723776 | 2.20030195 | 2.13763827 |
| 1 | 6.20791971 | 1.54746643 | 1.35638187 |
| 6 | 3.40496283 | -0.95342612 | -1.51206331 |
| 6 | -3.45794582 | 4.93934907 | -3.85604779 |
| 6 | -3.64157340 | 3.17431961 | -2.11634990 |

| | | | |
|---|---|---|---|
| 6 | -1.95304478 | 2.96607653 | -3.99995902 |
| 6 | 4.72950357 | -1.50144748 | -2.07592529 |
| 6 | 2.66067957 | -0.25918637 | -2.67735384 |
| 6 | 2.58345339 | -2.16532509 | -1.01070711 |
| 1 | 0.24766927 | 4.43890230 | 2.69933686 |
| 1 | -1.12167996 | 4.07247811 | 1.61092393 |
| 1 | 1.90592630 | 4.50450369 | 0.85872481 |
| 1 | 1.89338962 | 3.18860516 | -0.33037699 |
| 1 | -2.09997806 | 6.62370645 | -2.80018948 |
| 1 | 1.10920774 | 6.48880625 | 0.04041028 |
| 1 | 1.11840578 | 1.44132034 | 2.72572192 |
| 1 | 2.06201808 | 2.94651294 | 2.84266699 |
| 1 | 5.78412704 | -0.06670716 | -0.45049276 |
| 1 | 4.28328195 | 2.62912136 | 2.52692972 |
| 1 | -4.19060313 | 4.38389583 | -4.45908788 |
| 1 | -4.01606130 | 5.65056994 | -3.22993619 |
| 1 | -2.82377094 | 5.50973714 | -4.54984515 |
| 1 | -3.13152720 | 2.42070179 | -1.50635463 |
| 1 | -4.16900956 | 3.86992804 | -1.44668078 |
| 1 | -4.39516707 | 2.66849520 | -2.73949666 |
| 1 | -1.28838197 | 3.51659288 | -4.68158154 |
| 1 | -1.35836245 | 2.21337291 | -3.47108801 |
| 1 | -2.71114572 | 2.45115340 | -4.61005583 |
| 1 | 4.50694276 | -2.21080326 | -2.88582702 |
| 1 | 5.31238861 | -2.03996426 | -1.31456565 |
| 1 | 5.36033612 | -0.70605985 | -2.49822412 |
| 1 | 1.68427101 | 0.12339076 | -2.36124113 |
| 1 | 3.25343312 | 0.58036204 | -3.06866071 |
| 1 | 2.50376277 | -0.97432996 | -3.49930091 |
| 1 | 3.12074098 | -2.68720804 | -0.20452811 |
| 1 | 1.60186620 | -1.85348093 | -0.63726002 |
| 1 | 2.43428873 | -2.88006358 | -1.83455760 |
| 1 | -0.72316852 | 2.94450971 | 2.93818479 |
| 31 | -0.30686768 | 1.35820604 | 0.13733914 |
| 8 | 0.76455349 | -8.23189390 | 1.28282793 |
| 6 | 0.96092305 | -9.65817418 | 1.43664654 |
| 1 | 2.00620080 | -9.87668429 | 1.68890969 |
| 6 | 0.82000745 | -6.02027004 | 2.13167014 |
| 6 | 1.07924599 | -5.14541341 | 3.35920699 |
| 6 | 0.95560050 | -3.64084200 | 3.07922642 |
| 6 | 1.04653162 | -7.49647190 | 2.39799772 |
| 6 | -0.46776426 | -3.17969503 | 2.73591939 |
| 6 | -0.52202102 | -1.67603001 | 2.53055955 |
| 1 | 1.47674892 | -5.72796943 | 1.29524966 |
| 1 | 2.08859488 | -5.36153995 | 3.73784616 |
| 1 | 1.30450165 | -3.09265335 | 3.96976962 |
| 1 | -1.15815611 | -3.46546466 | 3.54624388 |
| 1 | -0.20705008 | -5.90676006 | 1.75202081 |
| 1 | 0.38785186 | -5.43411015 | 4.16625017 |
| 1 | 1.63962604 | -3.36256503 | 2.25965082 |
| 1 | -0.82507727 | -3.67058226 | 1.81793467 |
| 1 | 0.10941208 | -1.35260846 | 1.69079913 |
| 1 | -0.21790751 | -1.12825174 | 3.43458991 |

| | | | |
|---|---|---|---|
| 8 | 1.42735239 | -7.98478535 | 3.44493873 |
| 8 | -1.90729120 | -1.31912169 | 2.22059376 |
| 1 | 0.69676394 | -10.09266839 | 0.46779562 |
| 1 | 0.31231494 | -10.05169606 | 2.22938427 |

Electronic energy = -3908.665880 a.u.
DFT-D3(BJ) dispersion correction = -0.138719 a.u.
Thermal free energy = 0.736570 a.u.
Gibbs free energy = -3908.068029 a.u.
Number of imaginary frequencies = 0.

**Int4 eq CL isomer/conformer 6**

| Atomic N. | X | Y | Z |
|---|---|---|---|
| 6 | -3.80793231 | 0.06448268 | 1.33211306 |
| 6 | -4.60119144 | -1.04125194 | 0.61438612 |
| 6 | -4.50008407 | -1.11163050 | -0.92328820 |
| 6 | -2.34626681 | -0.18816875 | 1.61138629 |
| 6 | -3.21188990 | -1.69606114 | -1.53873319 |
| 6 | -2.08350845 | -0.68551158 | -1.74393902 |
| 1 | -3.87071907 | 1.01785775 | 0.79211724 |
| 1 | -5.66174708 | -0.87634861 | 0.86276559 |
| 1 | -5.34469015 | -1.73652660 | -1.25598139 |
| 1 | -3.45000603 | -2.12505008 | -2.52641222 |
| 1 | -4.25915813 | 0.23537331 | 2.32766822 |
| 1 | -4.33893560 | -2.01800555 | 1.04845199 |
| 1 | -4.68471842 | -0.11016529 | -1.34832724 |
| 1 | -2.84541366 | -2.52971600 | -0.91664310 |
| 1 | -1.21875908 | -1.18941449 | -2.21776025 |
| 1 | -2.42507630 | 0.10819611 | -2.43488451 |
| 8 | -1.50394118 | 0.73879023 | 1.78078015 |
| 8 | -1.72005137 | -0.13826209 | -0.49098445 |
| 7 | 0.30473726 | 2.83201116 | 1.08985435 |
| 8 | -1.09409519 | 2.47161284 | -1.44823694 |
| 8 | 1.05766343 | 0.33230216 | -0.24673037 |
| 6 | -0.63050890 | 3.55072294 | 1.98940943 |
| 6 | -1.03947010 | 3.81858057 | -1.49631178 |
| 6 | -1.93714111 | 4.53400274 | -2.34846997 |
| 6 | -1.84164361 | 5.93227872 | -2.36195871 |
| 6 | -0.91758514 | 6.63761784 | -1.58565335 |
| 6 | -0.04502786 | 5.93113145 | -0.76538542 |
| 6 | -0.09114665 | 4.53360971 | -0.72246439 |
| 6 | 0.91730628 | 3.78552515 | 0.10158368 |
| 1 | -0.88364361 | 7.72625315 | -1.62739836 |
| 6 | -2.97115343 | 3.80181569 | -3.22512026 |
| 6 | 2.30970360 | 0.68459583 | 0.10020245 |
| 6 | 3.42188089 | 0.10316991 | -0.58331023 |
| 6 | 4.70552888 | 0.48587090 | -0.17144809 |
| 6 | 4.92819152 | 1.40616946 | 0.85746303 |
| 6 | 3.83772105 | 1.97095229 | 1.50963543 |
| 6 | 2.53381537 | 1.61154425 | 1.14957458 |
| 6 | 1.37672139 | 2.18203815 | 1.92627655 |
| 1 | 5.94543306 | 1.67992182 | 1.13717690 |
| 6 | 3.22064046 | -0.90084448 | -1.73580862 |

| | | | |
|---|---|---|---|
| 6 | -3.81599348 | 4.77739018 | -4.06583792 |
| 6 | -3.94877877 | 3.00239942 | -2.33179600 |
| 6 | -2.24929466 | 2.85301411 | -4.21117872 |
| 6 | 4.56137347 | -1.40290702 | -2.30413233 |
| 6 | 2.45187796 | -0.22668262 | -2.89705313 |
| 6 | 2.43998763 | -2.14089259 | -1.23824927 |
| 1 | -0.10051224 | 4.37453299 | 2.49572507 |
| 1 | -1.45912165 | 3.96844219 | 1.40788531 |
| 1 | 1.55280437 | 4.49793715 | 0.65482435 |
| 1 | 1.58130584 | 3.18583749 | -0.53830277 |
| 1 | -2.51565539 | 6.50065235 | -3.00043869 |
| 1 | 0.69334160 | 6.45836479 | -0.15671183 |
| 1 | 0.86181626 | 1.40378522 | 2.51068362 |
| 1 | 1.75776264 | 2.93743340 | 2.63478842 |
| 1 | 5.57127874 | 0.05889378 | -0.67463685 |
| 1 | 3.98839650 | 2.69427867 | 2.31453068 |
| 1 | -4.52927872 | 4.20089734 | -4.67235032 |
| 1 | -4.39779423 | 5.46903419 | -3.43936613 |
| 1 | -3.19818600 | 5.36927579 | -4.75640756 |
| 1 | -3.41682826 | 2.26452298 | -1.72128644 |
| 1 | -4.50106031 | 3.67943132 | -1.66312678 |
| 1 | -4.68318108 | 2.47353447 | -2.95870970 |
| 1 | -1.60107422 | 3.42645121 | -4.88973909 |
| 1 | -1.63207983 | 2.11815944 | -3.68302271 |
| 1 | -2.98909671 | 2.31578492 | -4.82448678 |
| 1 | 4.36066979 | -2.11568714 | -3.11670853 |
| 1 | 5.16276024 | -1.92543836 | -1.54599071 |
| 1 | 5.16532441 | -0.58560882 | -2.72391724 |
| 1 | 1.46376824 | 0.12166809 | -2.57778725 |
| 1 | 3.01566593 | 0.63387805 | -3.28552786 |
| 1 | 2.31755160 | -0.94313110 | -3.72183821 |
| 1 | 2.99456275 | -2.64796090 | -0.43431277 |
| 1 | 1.44892031 | -1.86312038 | -0.86307958 |
| 1 | 2.31369641 | -2.85740823 | -2.06429926 |
| 1 | -1.02376305 | 2.84965854 | 2.73105578 |
| 31 | -0.56181919 | 1.28460969 | -0.07610332 |
| 8 | 3.81452383 | -7.43791596 | 3.93733560 |
| 6 | 4.04491607 | -8.84102520 | 4.21081878 |
| 1 | 3.48700227 | -9.15508495 | 5.10196526 |
| 6 | 2.37417451 | -5.62679754 | 3.42639196 |
| 6 | 0.93911919 | -5.19820703 | 3.12298997 |
| 6 | 0.82469757 | -3.69249389 | 2.86362788 |
| 6 | 2.51037150 | -7.11265044 | 3.69852981 |
| 6 | -0.61156150 | -3.25706016 | 2.54790844 |
| 6 | -0.69517203 | -1.76225284 | 2.29484287 |
| 1 | 2.77294097 | -5.08839814 | 4.30188146 |
| 1 | 0.28811407 | -5.48582290 | 3.96269562 |
| 1 | 1.19261360 | -3.13961394 | 3.74539573 |
| 1 | -1.27834446 | -3.52707710 | 3.38234883 |
| 1 | 3.04971587 | -5.37227481 | 2.59376529 |
| 1 | 0.57014914 | -5.75705275 | 2.24941349 |
| 1 | 1.48444863 | -3.41133959 | 2.02543567 |
| 1 | -0.97635411 | -3.79333996 | 1.65789795 |

| | | | |
|---|---|---|---|
| 1 | -0.08219655 | -1.45344807 | 1.43596743 |
| 1 | -0.38863486 | -1.17979166 | 3.17599270 |
| 8 | 1.60345941 | -7.92318395 | 3.71086944 |
| 8 | -2.09030856 | -1.44048743 | 1.99127627 |
| 1 | 5.12281486 | -8.93023106 | 4.37640649 |
| 1 | 3.73032516 | -9.45495099 | 3.35755257 |

Electronic energy = -3908.667106 a.u.
DFT-D3(BJ) dispersion correction = -0.138163 a.u.
Thermal free energy = 0.737372 a.u.
Gibbs free energy = -3908.067897 a.u.
Number of imaginary frequencies = 0.

**Int4 eq CL isomer/conformer 7**

| Atomic N. | X | Y | Z |
|---|---|---|---|
| 6 | -2.70463139 | -0.82240994 | 2.78946315 |
| 6 | -3.57959293 | -1.91253060 | 2.14743105 |
| 6 | -3.89678451 | -1.77938208 | 0.64400846 |
| 6 | -1.20751039 | -0.94115989 | 2.63804476 |
| 6 | -2.78225753 | -2.12746137 | -0.36338131 |
| 6 | -1.82443011 | -0.98301748 | -0.69087098 |
| 1 | -2.98515802 | 0.17706968 | 2.43311954 |
| 1 | -4.54072596 | -1.89962593 | 2.68574914 |
| 1 | -4.74749862 | -2.45279456 | 0.45004388 |
| 1 | -3.24257750 | -2.44639585 | -1.31282244 |
| 1 | -2.87695820 | -0.82642762 | 3.88236864 |
| 1 | -3.13638264 | -2.90009793 | 2.34732210 |
| 1 | -4.26997161 | -0.76175035 | 0.43818499 |
| 1 | -2.20394044 | -2.99173574 | 0.00355803 |
| 1 | -1.09531255 | -1.32223713 | -1.45026596 |
| 1 | -2.39615009 | -0.14220467 | -1.12597603 |
| 8 | -0.42400706 | 0.04751865 | 2.73152898 |
| 8 | -1.15726398 | -0.57468473 | 0.49213193 |
| 7 | 0.90998468 | 2.43911577 | 1.95425622 |
| 8 | -1.14406108 | 2.22027714 | -0.11251940 |
| 8 | 1.49243376 | 0.30278977 | 0.03837884 |
| 6 | 0.21535354 | 2.87980338 | 3.18813863 |
| 6 | -1.24135196 | 3.55532897 | 0.05653023 |
| 6 | -2.41758029 | 4.24050901 | -0.38121289 |
| 6 | -2.47403647 | 5.62625266 | -0.17878813 |
| 6 | -1.43993804 | 6.34827157 | 0.42376051 |
| 6 | -0.29642653 | 5.67350810 | 0.83627057 |
| 6 | -0.18425707 | 4.29176276 | 0.64839367 |
| 6 | 1.09652180 | 3.59913821 | 1.01679699 |
| 1 | -1.53145164 | 7.42541737 | 0.56315532 |
| 6 | -3.58187220 | 3.49172565 | -1.05756002 |
| 6 | 2.74253178 | 0.80092459 | 0.07719188 |
| 6 | 3.64908608 | 0.52403550 | -0.99267196 |
| 6 | 4.94712022 | 1.04202628 | -0.88958811 |
| 6 | 5.37141263 | 1.81524874 | 0.19549647 |
| 6 | 4.47558130 | 2.09103246 | 1.22246637 |
| 6 | 3.17170362 | 1.58421120 | 1.17840526 |
| 6 | 2.24370379 | 1.84870837 | 2.33391337 |

| | | | |
|---|---|---|---|
| 1 | 6.38965339 | 2.20278315 | 0.22747561 |
| 6 | 3.21596699 | -0.30212699 | -2.21973386 |
| 6 | -4.73122677 | 4.43772526 | -1.45316788 |
| 6 | -4.16784832 | 2.44033833 | -0.08650635 |
| 6 | -3.08536307 | 2.80649364 | -2.35272822 |
| 6 | 4.36278533 | -0.47622567 | -3.23312574 |
| 6 | 2.05672748 | 0.41181213 | -2.95525444 |
| 6 | 2.76884834 | -1.71732001 | -1.78402850 |
| 1 | 0.78076116 | 3.70016916 | 3.66086219 |
| 1 | -0.78934875 | 3.23924424 | 2.94194326 |
| 1 | 1.79021243 | 4.31785152 | 1.48564902 |
| 1 | 1.59733252 | 3.19917953 | 0.12286749 |
| 1 | -3.36047358 | 6.17020861 | -0.50020514 |
| 1 | 0.53130405 | 6.21637448 | 1.29868417 |
| 1 | 2.01007926 | 0.92411846 | 2.88461535 |
| 1 | 2.73466875 | 2.54329860 | 3.03707208 |
| 1 | 5.65906012 | 0.84541034 | -1.68931459 |
| 1 | 4.78251542 | 2.69998400 | 2.07614299 |
| 1 | -5.52681232 | 3.85102327 | -1.93461303 |
| 1 | -5.17304416 | 4.94183343 | -0.58133739 |
| 1 | -4.40630941 | 5.20465256 | -2.17089539 |
| 1 | -3.40754304 | 1.71041608 | 0.21182601 |
| 1 | -4.56191730 | 2.92779781 | 0.81775162 |
| 1 | -4.99800643 | 1.90318811 | -0.57058073 |
| 1 | -2.72829102 | 3.55853662 | -3.07129947 |
| 1 | -2.26685075 | 2.10739753 | -2.14978515 |
| 1 | -3.91128485 | 2.25301585 | -2.82541298 |
| 1 | 4.00036116 | -1.07413877 | -4.08163996 |
| 1 | 5.22306207 | -1.00543892 | -2.79780804 |
| 1 | 4.71281833 | 0.48633785 | -3.63328647 |
| 1 | 1.18267120 | 0.53453978 | -2.30686185 |
| 1 | 2.37406703 | 1.40518359 | -3.30470635 |
| 1 | 1.75385110 | -0.17701555 | -3.83422953 |
| 1 | 3.60252574 | -2.25572394 | -1.30846471 |
| 1 | 1.93192491 | -1.66821233 | -1.07986039 |
| 1 | 2.44477527 | -2.29364709 | -2.66389154 |
| 1 | 0.13962439 | 2.03475794 | 3.87828557 |
| 31 | -0.09756460 | 0.95029503 | 0.81868193 |
| 8 | 1.65338129 | -6.16774839 | -2.37992603 |
| 6 | 2.14076103 | -5.92156527 | -3.72139292 |
| 1 | 1.34034901 | -6.07976540 | -4.45481372 |
| 6 | 0.19781036 | -5.65239382 | -0.57162771 |
| 6 | 0.79938119 | -4.60491203 | 0.38565878 |
| 6 | 0.32939379 | -4.81828725 | 1.82864654 |
| 6 | 0.60315160 | -5.37877419 | -2.00449282 |
| 6 | 0.97304719 | -3.86927441 | 2.85115877 |
| 6 | 0.68360167 | -2.39046724 | 2.64805544 |
| 1 | -0.89968494 | -5.60247395 | -0.52807314 |
| 1 | 0.51504052 | -3.60296762 | 0.03284753 |
| 1 | -0.76635687 | -4.71517176 | 1.87543199 |
| 1 | 2.06986975 | -3.98340875 | 2.82411584 |
| 1 | 0.51769539 | -6.66176544 | -0.27844845 |
| 1 | 1.89935566 | -4.66010624 | 0.34013236 |

| | | | |
|---|---|---|---|
| 1 | 0.55529069 | -5.85334554 | 2.13328760 |
| 1 | 0.65520079 | -4.15629275 | 3.86601756 |
| 1 | 0.98936121 | -2.02477951 | 1.65765206 |
| 1 | 1.18004250 | -1.77982350 | 3.41417194 |
| 8 | 0.09516825 | -4.54621415 | -2.73243383 |
| 8 | -0.76224407 | -2.19410601 | 2.76178550 |
| 1 | 2.95328221 | -6.63912022 | -3.87059029 |
| 1 | 2.51008534 | -4.89211420 | -3.81073434 |

Electronic energy = -3908.665406 a.u.
DFT-D3(BJ) dispersion correction = -0.142457 a.u.
Thermal free energy = 0.740206 a.u.
Gibbs free energy = -3908.067657 a.u.
Number of imaginary frequencies = 0.

**Int4 eq CL isomer/conformer 8**

| Atomic N. | X | Y | Z |
|---|---|---|---|
| 6 | -3.28930104 | -1.14309302 | 2.22467025 |
| 6 | -4.10574844 | -2.32057258 | 1.66409875 |
| 6 | -4.49799740 | -2.27347028 | 0.17283394 |
| 6 | -1.79540526 | -1.15878012 | 2.01074263 |
| 6 | -3.40978950 | -2.59611598 | -0.87207875 |
| 6 | -2.55803111 | -1.40127540 | -1.30040752 |
| 1 | -3.65701875 | -0.18248615 | 1.84208977 |
| 1 | -5.04102277 | -2.35681555 | 2.24513643 |
| 1 | -5.31148648 | -3.00642044 | 0.04797174 |
| 1 | -3.89209488 | -2.99610552 | -1.77965204 |
| 1 | -3.41579688 | -1.11515688 | 3.32331310 |
| 1 | -3.58079661 | -3.26351719 | 1.88001852 |
| 1 | -4.94405357 | -1.29078455 | -0.05747341 |
| 1 | -2.75151359 | -3.39454129 | -0.49053236 |
| 1 | -1.82810062 | -1.72494603 | -2.06769606 |
| 1 | -3.20944081 | -0.63598200 | -1.76350768 |
| 8 | -1.08601951 | -0.11301701 | 2.01040738 |
| 8 | -1.89579122 | -0.87680100 | -0.16609510 |
| 7 | 0.13608511 | 2.26553723 | 1.02654758 |
| 8 | -1.95398143 | 1.85662255 | -0.97300177 |
| 8 | 0.72347304 | 0.00980850 | -0.75170155 |
| 6 | -0.53917885 | 2.77087106 | 2.24711342 |
| 6 | -2.09062817 | 3.19545839 | -0.88224991 |
| 6 | -3.30156691 | 3.81502234 | -1.32200181 |
| 6 | -3.39493964 | 5.20878340 | -1.20708804 |
| 6 | -2.36532103 | 5.99808047 | -0.68652473 |
| 6 | -1.18988902 | 5.38520722 | -0.26678250 |
| 6 | -1.04069646 | 3.99807121 | -0.36948286 |
| 6 | 0.26768195 | 3.36460419 | 0.00868424 |
| 1 | -2.48594725 | 7.07890907 | -0.61376348 |
| 6 | -4.46325938 | 2.98794644 | -1.90543352 |
| 6 | 1.96622015 | 0.52653018 | -0.77228838 |
| 6 | 2.86122537 | 0.17054202 | -1.82816490 |
| 6 | 4.15168785 | 0.71593388 | -1.79318911 |
| 6 | 4.57898017 | 1.58691026 | -0.78621756 |
| 6 | 3.69583566 | 1.93327018 | 0.23045219 |

| | | | |
|---|---|---|---|
| 6 | 2.40020329 | 1.40431184 | 0.25333809 |
| 6 | 1.49500711 | 1.73851843 | 1.40968278 |
| 1 | 5.59015170 | 1.99324604 | -0.80572428 |
| 6 | 2.42374602 | -0.76953475 | -2.96912551 |
| 6 | -5.65560979 | 3.87099189 | -2.31875910 |
| 6 | -4.98122826 | 1.98491275 | -0.84778466 |
| 6 | -3.99008722 | 2.23378981 | -3.17077625 |
| 6 | 3.56029564 | -1.02000299 | -3.97818230 |
| 6 | 1.24525474 | -0.14055509 | -3.74984883 |
| 6 | 2.00324243 | -2.14603879 | -2.40089256 |
| 1 | 0.01678000 | 3.63392417 | 2.64939490 |
| 1 | -1.55840107 | 3.08901445 | 2.00394427 |
| 1 | 0.95297373 | 4.12997999 | 0.41114796 |
| 1 | 0.75717938 | 2.91837048 | -0.86951640 |
| 1 | -4.30763845 | 5.70483521 | -1.53256382 |
| 1 | -0.36682422 | 5.98085345 | 0.13497140 |
| 1 | 1.30216854 | 0.85480306 | 2.03749600 |
| 1 | 1.98652028 | 2.49733156 | 2.04226490 |
| 1 | 4.85447127 | 0.46201136 | -2.58484007 |
| 1 | 4.00764612 | 2.61418446 | 1.02587620 |
| 1 | -6.44853547 | 3.23045350 | -2.73101872 |
| 1 | -6.08233745 | 4.41810764 | -1.46556753 |
| 1 | -5.38100008 | 4.59829343 | -3.09626626 |
| 1 | -4.18883066 | 1.29921442 | -0.52826021 |
| 1 | -5.36154213 | 2.51862174 | 0.03598686 |
| 1 | -5.80902686 | 1.39211464 | -1.26663337 |
| 1 | -3.67944730 | 2.94666122 | -3.94845270 |
| 1 | -3.14514104 | 1.57202055 | -2.95145015 |
| 1 | -4.81508123 | 1.62857241 | -3.57724554 |
| 1 | 3.19709449 | -1.69652812 | -4.76498783 |
| 1 | 4.43333949 | -1.49634867 | -3.50870782 |
| 1 | 3.89159649 | -0.09272995 | -4.46741061 |
| 1 | 0.37881320 | 0.03140720 | -3.10224574 |
| 1 | 1.54462728 | 0.82029927 | -4.19310174 |
| 1 | 0.93945311 | -0.80995500 | -4.56841134 |
| 1 | 2.84563328 | -2.61870737 | -1.87359069 |
| 1 | 1.16110959 | -2.04982815 | -1.70688549 |
| 1 | 1.70461840 | -2.81323193 | -3.22394694 |
| 1 | -0.57570391 | 1.97195435 | 2.99302197 |
| 31 | -0.85962212 | 0.68172378 | 0.02470204 |
| 8 | 6.73634478 | -3.56585714 | 3.20827887 |
| 6 | 7.73351580 | -2.99000863 | 4.08605610 |
| 1 | 7.69985085 | -1.89437330 | 4.03809924 |
| 6 | 4.48696902 | -3.85549272 | 2.52098284 |
| 6 | 3.02447863 | -3.52101199 | 2.81177653 |
| 6 | 2.07225696 | -4.16270567 | 1.79494944 |
| 6 | 5.45095625 | -3.21188038 | 3.49911119 |
| 6 | 0.58139335 | -3.92487159 | 2.08445885 |
| 6 | 0.19842501 | -2.45756951 | 1.98301802 |
| 1 | 4.77207486 | -3.53039419 | 1.50707238 |
| 1 | 2.91025587 | -2.42735408 | 2.81760413 |
| 1 | 2.30896603 | -3.79329905 | 0.78267452 |
| 1 | 0.32794421 | -4.30309759 | 3.08794330 |

| | | | |
|---|---|---|---|
| 1 | 4.66022689 | -4.94378162 | 2.54022668 |
| 1 | 2.76881675 | -3.85822031 | 3.82880153 |
| 1 | 2.24847905 | -5.25049650 | 1.77242541 |
| 1 | -0.02469145 | -4.49364063 | 1.36300419 |
| 1 | 0.44828337 | -2.03624831 | 0.99793860 |
| 1 | 0.67156942 | -1.84500226 | 2.76156178 |
| 8 | 5.14885717 | -2.47814049 | 4.42142313 |
| 8 | -1.25306914 | -2.36873671 | 2.15345804 |
| 1 | 8.69346149 | -3.36504060 | 3.71855966 |
| 1 | 7.56239382 | -3.30806659 | 5.12219776 |

Electronic energy = -3908.666222 a.u.
DFT-D3(BJ) dispersion correction = -0.139176 a.u.
Thermal free energy = 0.737975 a.u.
Gibbs free energy = -3908.067423 a.u.
Number of imaginary frequencies = 0.

**Int4 eq CL isomer/conformer 9**

| Atomic N. | X | Y | Z |
|---|---|---|---|
| 6 | -3.36602418 | 0.03282935 | 1.60397636 |
| 6 | -4.17896149 | -1.05538866 | 0.88108076 |
| 6 | -4.08620823 | -1.11484964 | -0.65773627 |
| 6 | -1.90797845 | -0.24582202 | 1.87800797 |
| 6 | -2.81287437 | -1.72010640 | -1.28426216 |
| 6 | -1.66747677 | -0.72897903 | -1.49076059 |
| 1 | -3.41465725 | 0.99060507 | 1.07041146 |
| 1 | -5.23576384 | -0.87685005 | 1.13589596 |
| 1 | -4.94524924 | -1.71903708 | -0.99194074 |
| 1 | -3.06455754 | -2.14000572 | -2.27254694 |
| 1 | -3.81230659 | 0.20377605 | 2.60168016 |
| 1 | -3.92883478 | -2.03907567 | 1.30667434 |
| 1 | -4.25216245 | -0.10610406 | -1.07308931 |
| 1 | -2.45727684 | -2.56256928 | -0.66756185 |
| 1 | -0.81305311 | -1.24705700 | -1.96819267 |
| 1 | -1.99716067 | 0.07196825 | -2.17926539 |
| 8 | -1.04833290 | 0.66521935 | 2.04257057 |
| 8 | -1.29291823 | -0.19199693 | -0.23724939 |
| 7 | 0.79418329 | 2.72776889 | 1.35240628 |
| 8 | -0.61743395 | 2.40949103 | -1.18390067 |
| 8 | 1.49579389 | 0.22322412 | -0.00285177 |
| 6 | -0.12470508 | 3.45805863 | 2.25953936 |
| 6 | -0.53897369 | 3.75541938 | -1.22318697 |
| 6 | -1.42637848 | 4.49268813 | -2.06746975 |
| 6 | -1.30532329 | 5.88903780 | -2.07247842 |
| 6 | -0.36594843 | 6.57234573 | -1.29484068 |
| 6 | 0.49601302 | 5.84475325 | -0.48181500 |
| 6 | 0.42455728 | 4.44808157 | -0.44773623 |
| 6 | 1.42138314 | 3.67618873 | 0.36842496 |
| 1 | -0.31210515 | 7.66041737 | -1.32987974 |
| 6 | -2.47702942 | 3.78506991 | -2.94450509 |
| 6 | 2.75537117 | 0.55088515 | 0.34097695 |
| 6 | 3.85415280 | -0.04475574 | -0.35187895 |
| 6 | 5.14612045 | 0.31160228 | 0.05758672 |

| | | | |
|---|---|---|---|
| 6 | 5.38941305 | 1.21965838 | 1.09265484 |
| 6 | 4.31190452 | 1.79917151 | 1.75340883 |
| 6 | 3.00032294 | 1.46597608 | 1.39606483 |
| 6 | 1.85676786 | 2.05247558 | 2.18099387 |
| 1 | 6.41253408 | 1.47244794 | 1.37065862 |
| 6 | 3.63019497 | -1.03554056 | -1.51161697 |
| 6 | -3.30732747 | 4.78120741 | -3.77552784 |
| 6 | -3.46520683 | 2.99776941 | -2.05198816 |
| 6 | -1.77667948 | 2.82942751 | -3.93944775 |
| 6 | 4.95936256 | -1.55672982 | -2.08984755 |
| 6 | 2.86886814 | -0.33807712 | -2.66395501 |
| 6 | 2.82962467 | -2.26553311 | -1.02089042 |
| 1 | 0.42213433 | 4.26861517 | 2.76931763 |
| 1 | -0.94718164 | 3.89485247 | 1.68333116 |
| 1 | 2.07144774 | 4.37310645 | 0.92447632 |
| 1 | 2.07236575 | 3.06859033 | -0.27730453 |
| 1 | -1.97093186 | 6.47375930 | -2.70500015 |
| 1 | 1.24564615 | 6.35449810 | 0.12799606 |
| 1 | 1.32967440 | 1.28012292 | 2.76224878 |
| 1 | 2.25435032 | 2.79612146 | 2.89282233 |
| 1 | 6.00204534 | -0.12702866 | -0.45234684 |
| 1 | 4.47879233 | 2.51361512 | 2.56301454 |
| 1 | -4.03335979 | 4.22172146 | -4.38285171 |
| 1 | -3.87393459 | 5.47923260 | -3.14223642 |
| 1 | -2.68157533 | 5.36628922 | -4.46474784 |
| 1 | -2.94422777 | 2.24559239 | -1.44950796 |
| 1 | -4.00119590 | 3.68012471 | -1.37551696 |
| 1 | -4.21262667 | 2.48748119 | -2.67894419 |
| 1 | -1.12042158 | 3.39502049 | -4.61687105 |
| 1 | -1.17134015 | 2.07988512 | -3.41822027 |
| 1 | -2.52874676 | 2.30999244 | -4.55319715 |
| 1 | 4.74250273 | -2.25902698 | -2.90742373 |
| 1 | 5.55436644 | -2.09626471 | -1.33860594 |
| 1 | 5.57611609 | -0.74688238 | -2.50544080 |
| 1 | 1.88865655 | 0.02560394 | -2.33749422 |
| 1 | 3.44656160 | 0.51525536 | -3.04793378 |
| 1 | 2.71792166 | -1.04519477 | -3.49392911 |
| 1 | 3.37905729 | -2.78970082 | -0.22445737 |
| 1 | 1.84583576 | -1.97316403 | -0.63779261 |
| 1 | 2.68594090 | -2.97212410 | -1.85270568 |
| 1 | -0.52846789 | 2.75978244 | 2.99810206 |
| 31 | -0.10520133 | 1.20394875 | 0.18025702 |
| 8 | -0.91485893 | -6.79554667 | 0.89653452 |
| 6 | -2.31092011 | -7.15784719 | 0.76075689 |
| 1 | -2.52940743 | -8.06632393 | 1.33584318 |
| 6 | 0.97188817 | -6.25091936 | 2.23575227 |
| 6 | 1.37236378 | -5.30117908 | 3.37458947 |
| 6 | 1.21729919 | -3.80800922 | 3.04685086 |
| 6 | -0.51550750 | -6.54843873 | 2.17786129 |
| 6 | -0.22133194 | -3.35544288 | 2.76217743 |
| 6 | -0.28250462 | -1.85326675 | 2.54829454 |
| 1 | 1.45932857 | -7.22934912 | 2.38394160 |
| 1 | 2.42675623 | -5.48790168 | 3.62711817 |

| | | | |
|---|---|---|---|
| 1 | 1.61635433 | -3.22842808 | 3.89611820 |
| 1 | -0.88142470 | -3.64249593 | 3.59497352 |
| 1 | 1.31122585 | -5.88301634 | 1.25688184 |
| 1 | 0.78133860 | -5.55616246 | 4.26746959 |
| 1 | 1.85389401 | -3.56005287 | 2.18055144 |
| 1 | -0.60953267 | -3.84969268 | 1.85823231 |
| 1 | 0.33796802 | -1.53069900 | 1.69982950 |
| 1 | 0.02780995 | -1.29845195 | 3.44587808 |
| 8 | -1.26478150 | -6.60318555 | 3.13533868 |
| 8 | -1.67247127 | -1.50366306 | 2.24908851 |
| 1 | -2.46211550 | -7.32899626 | -0.30925724 |
| 1 | -2.95323426 | -6.34384217 | 1.11954722 |

Electronic energy = -3908.664998 a.u.
DFT-D3(BJ) dispersion correction = -0.140037 a.u.
Thermal free energy = 0.737657 a.u.
Gibbs free energy = -3908.067379 a.u.
Number of imaginary frequencies = 0.

**Int4 eq CL isomer/conformer 10**

| Atomic N. | X | Y | Z |
|---|---|---|---|
| 6 | -2.67547105 | -0.37537652 | 2.91780485 |
| 6 | -3.57042452 | -1.53683231 | 2.45302170 |
| 6 | -3.90685530 | -1.62076643 | 0.95025558 |
| 6 | -1.18184319 | -0.53272890 | 2.76693625 |
| 6 | -2.80968932 | -2.12597891 | -0.00842859 |
| 6 | -1.84458345 | -1.05371310 | -0.51178702 |
| 1 | -2.95092658 | 0.56430776 | 2.42215498 |
| 1 | -4.52378911 | -1.43359000 | 2.99539767 |
| 1 | -4.76713754 | -2.30475302 | 0.86706187 |
| 1 | -3.28597275 | -2.57467730 | -0.89548997 |
| 1 | -2.83245787 | -0.21735632 | 4.00155652 |
| 1 | -3.13481137 | -2.48998008 | 2.78983407 |
| 1 | -4.27208202 | -0.63952849 | 0.60237397 |
| 1 | -2.23549252 | -2.93427939 | 0.47417626 |
| 1 | -1.12875160 | -1.50987133 | -1.22078666 |
| 1 | -2.41336225 | -0.27944692 | -1.05963017 |
| 8 | -0.38750790 | 0.44971435 | 2.70586600 |
| 8 | -1.15831544 | -0.48309790 | 0.59050909 |
| 7 | 0.95851394 | 2.68535787 | 1.57116174 |
| 8 | -1.11942876 | 2.19022125 | -0.42310643 |
| 8 | 1.49716551 | 0.28444212 | -0.01594914 |
| 6 | 0.28127970 | 3.31149409 | 2.73244624 |
| 6 | -1.19785084 | 3.53669322 | -0.45562150 |
| 6 | -2.36801388 | 4.16489763 | -0.98553365 |
| 6 | -2.40494155 | 5.56594966 | -0.99321746 |
| 6 | -1.35734579 | 6.35566521 | -0.51091965 |
| 6 | -0.21997719 | 5.73445479 | -0.00742378 |
| 6 | -0.12692597 | 4.33869978 | 0.01351199 |
| 6 | 1.14910068 | 3.69262247 | 0.47214980 |
| 1 | -1.43374921 | 7.44270672 | -0.53432046 |
| 6 | -3.54725429 | 3.33935501 | -1.53479964 |
| 6 | 2.75357443 | 0.76618393 | -0.06000276 |

| | | | |
|---|---|---|---|
| 6 | 3.64654151 | 0.32381500 | -1.08479724 |
| 6 | 4.95165503 | 0.83395663 | -1.06867827 |
| 6 | 5.39559384 | 1.75199613 | -0.11191541 |
| 6 | 4.51286231 | 2.18731970 | 0.87016127 |
| 6 | 3.20258868 | 1.69692494 | 0.91089921 |
| 6 | 2.28932793 | 2.14048418 | 2.02251465 |
| 1 | 6.41866211 | 2.12651758 | -0.14493424 |
| 6 | 3.19168020 | -0.66613033 | -2.17513308 |
| 6 | -4.68661790 | 4.23125158 | -2.06271655 |
| 6 | -4.14034982 | 2.45573265 | -0.41251407 |
| 6 | -3.06996883 | 2.45903395 | -2.71413096 |
| 6 | 4.32658185 | -1.00159388 | -3.16105510 |
| 6 | 2.03465430 | -0.05072942 | -2.99785602 |
| 6 | 2.73093610 | -1.99666408 | -1.53497626 |
| 1 | 0.86029685 | 4.18565912 | 3.07404429 |
| 1 | -0.72189271 | 3.64301159 | 2.44437240 |
| 1 | 1.85401136 | 4.46432716 | 0.82598694 |
| 1 | 1.63916301 | 3.16042391 | -0.35633252 |
| 1 | -3.28637628 | 6.06782152 | -1.38840102 |
| 1 | 0.61814409 | 6.32895517 | 0.36378182 |
| 1 | 2.05089579 | 1.31018704 | 2.70541893 |
| 1 | 2.79519555 | 2.92490036 | 2.61134145 |
| 1 | 5.65351231 | 0.51287867 | -1.83638563 |
| 1 | 4.83516450 | 2.91066114 | 1.62294501 |
| 1 | -5.49355709 | 3.59006997 | -2.44576709 |
| 1 | -5.11525485 | 4.86736637 | -1.27465167 |
| 1 | -4.35676504 | 4.87633171 | -2.88973455 |
| 1 | -3.38756365 | 1.77005667 | -0.00868722 |
| 1 | -4.52331728 | 3.08067943 | 0.40806331 |
| 1 | -4.97986691 | 1.86193312 | -0.80575291 |
| 1 | -2.70831953 | 3.08851876 | -3.54018312 |
| 1 | -2.25947732 | 1.78689464 | -2.41221224 |
| 1 | -3.90692726 | 1.85247175 | -3.09292476 |
| 1 | 3.94868839 | -1.71182321 | -3.91046621 |
| 1 | 5.18438149 | -1.47335793 | -2.65980934 |
| 1 | 4.68456733 | -0.11249721 | -3.70007834 |
| 1 | 1.16851202 | 0.17681438 | -2.36740452 |
| 1 | 2.36106685 | 0.87688391 | -3.49037767 |
| 1 | 1.71593661 | -0.75678415 | -3.77949396 |
| 1 | 3.56240448 | -2.47209390 | -0.99304058 |
| 1 | 1.90162684 | -1.83437280 | -0.83888976 |
| 1 | 2.39058186 | -2.68983129 | -2.31917424 |
| 1 | 0.20356183 | 2.57835844 | 3.54029613 |
| 31 | -0.07700508 | 1.05886364 | 0.67612318 |
| 8 | 1.51306775 | -6.45846418 | -1.50742307 |
| 6 | 1.96447492 | -6.40147229 | -2.88256841 |
| 1 | 1.13688743 | -6.62587779 | -3.56691078 |
| 6 | 0.12752045 | -5.66879811 | 0.25788034 |
| 6 | 0.75149251 | -4.50170521 | 1.04780143 |
| 6 | 0.30542438 | -4.50339371 | 2.51386122 |
| 6 | 0.49945740 | -5.59301371 | -1.20784179 |
| 6 | 0.97284012 | -3.42377223 | 3.37975006 |
| 6 | 0.69497395 | -1.98677198 | 2.96761725 |

| | | | |
|---|---|---|---|
| 1 | -0.96875683 | -5.60823115 | 0.31905528 |
| 1 | 0.46656034 | -3.55786632 | 0.56082253 |
| 1 | -0.78863466 | -4.38433887 | 2.56279655 |
| 1 | 2.06801551 | -3.55328302 | 3.35528709 |
| 1 | 0.45236376 | -6.63025215 | 0.67847766 |
| 1 | 1.85023554 | -4.57128572 | 0.99204460 |
| 1 | 0.52920473 | -5.48635308 | 2.95982693 |
| 1 | 0.66693063 | -3.55716721 | 4.42947486 |
| 1 | 0.99223921 | -1.77219409 | 1.93142586 |
| 1 | 1.20670390 | -1.27719865 | 3.63165978 |
| 8 | -0.00482329 | -4.84241328 | -2.02231106 |
| 8 | -0.74739097 | -1.75946725 | 3.06710818 |
| 1 | 2.75088324 | -7.15828462 | -2.96081050 |
| 1 | 2.36012156 | -5.40440693 | -3.11369977 |

Electronic energy = -3908.665420 a.u.
DFT-D3(BJ) dispersion correction = -0.142496 a.u.
Thermal free energy = 0.740624 a.u.
Gibbs free energy = -3908.067292 a.u.
Number of imaginary frequencies = 0.

**Int4 eq CL isomer/conformer 11**

| Atomic N. | X | Y | Z |
|---|---|---|---|
| 6 | -3.83436911 | -0.25898153 | 1.43354824 |
| 6 | -4.61459579 | -1.36887839 | 0.70776783 |
| 6 | -4.51866242 | -1.42056258 | -0.83070620 |
| 6 | -2.37199971 | -0.50146235 | 1.71738005 |
| 6 | -3.22102912 | -1.96928516 | -1.45840912 |
| 6 | -2.10923116 | -0.93709652 | -1.64399334 |
| 1 | -3.90501257 | 0.69705127 | 0.89928424 |
| 1 | -5.67652107 | -1.22298668 | 0.96202498 |
| 1 | -5.35092540 | -2.06009061 | -1.16682068 |
| 1 | -3.45349943 | -2.38058050 | -2.45498405 |
| 1 | -4.28959369 | -0.09818809 | 2.42903175 |
| 1 | -4.33544980 | -2.34606303 | 1.13003362 |
| 1 | -4.72723052 | -0.41892347 | -1.24398446 |
| 1 | -2.83970269 | -2.81031104 | -0.85539951 |
| 1 | -1.24435495 | -1.41387287 | -2.14476741 |
| 1 | -2.47069736 | -0.12649668 | -2.30479168 |
| 8 | -1.53700125 | 0.43102999 | 1.89908956 |
| 8 | -1.73287138 | -0.42499033 | -0.37964647 |
| 7 | 0.22538018 | 2.57044131 | 1.25739046 |
| 8 | -1.26362510 | 2.26298843 | -1.24057646 |
| 8 | 1.01951840 | 0.17249760 | -0.22129409 |
| 6 | -0.69661423 | 3.22027028 | 2.22052537 |
| 6 | -1.25160907 | 3.61207225 | -1.23668941 |
| 6 | -2.19785092 | 4.33051865 | -2.03203852 |
| 6 | -2.14928495 | 5.73066339 | -1.99042545 |
| 6 | -1.22486769 | 6.43544963 | -1.21423206 |
| 6 | -0.30218064 | 5.72657566 | -0.45313019 |
| 6 | -0.30029708 | 4.32766266 | -0.46659620 |
| 6 | 0.76506909 | 3.58441670 | 0.28775055 |
| 1 | -1.22864078 | 7.52540519 | -1.21128961 |

| | | | |
|---|---|---|---|
| 6 | -3.23165587 | 3.60013456 | -2.91050555 |
| 6 | 2.26845350 | 0.56748023 | 0.08683342 |
| 6 | 3.37318946 | 0.09051905 | -0.68488064 |
| 6 | 4.65602887 | 0.50251820 | -0.29927451 |
| 6 | 4.88441031 | 1.35938700 | 0.78196388 |
| 6 | 3.79997123 | 1.83378196 | 1.51166192 |
| 6 | 2.49840192 | 1.43955548 | 1.18109096 |
| 6 | 1.34940919 | 1.92303587 | 2.02538832 |
| 1 | 5.90048468 | 1.65863386 | 1.03889259 |
| 6 | 3.16456190 | -0.82825202 | -1.90529571 |
| 6 | -4.13268292 | 4.58037343 | -3.68517953 |
| 6 | -4.15588794 | 2.73015886 | -2.02689151 |
| 6 | -2.50669217 | 2.72020707 | -3.95632567 |
| 6 | 4.49840517 | -1.21037266 | -2.57430496 |
| 6 | 2.30701670 | -0.10380110 | -2.97024083 |
| 6 | 2.47149673 | -2.14358185 | -1.47830786 |
| 1 | -0.17613098 | 4.04541952 | 2.73461505 |
| 1 | -1.56438146 | 3.62584708 | 1.68986684 |
| 1 | 1.39659394 | 4.29732480 | 0.84493933 |
| 1 | 1.42344562 | 3.03658431 | -0.40224290 |
| 1 | -2.86160141 | 6.30080430 | -2.58413030 |
| 1 | 0.43905780 | 6.25378610 | 0.15206720 |
| 1 | 0.88474807 | 1.09720920 | 2.58665679 |
| 1 | 1.72652418 | 2.65663766 | 2.75848911 |
| 1 | 5.51621068 | 0.15285796 | -0.86751549 |
| 1 | 3.95400780 | 2.51342271 | 2.35317485 |
| 1 | -4.84236233 | 4.00537866 | -4.29730804 |
| 1 | -4.72018594 | 5.22341154 | -3.01382744 |
| 1 | -3.55518392 | 5.22290015 | -4.36524243 |
| 1 | -3.58203147 | 1.98126068 | -1.47025205 |
| 1 | -4.70497840 | 3.35761009 | -1.30904208 |
| 1 | -4.89473997 | 2.20921322 | -2.65515727 |
| 1 | -1.89803797 | 3.34436561 | -4.62666807 |
| 1 | -1.85004417 | 1.98557814 | -3.47760688 |
| 1 | -3.24507992 | 2.18372823 | -4.57199152 |
| 1 | 4.29244624 | -1.86393609 | -3.43394716 |
| 1 | 5.16136060 | -1.76127186 | -1.89122579 |
| 1 | 5.04034681 | -0.33073301 | -2.95055888 |
| 1 | 1.31823762 | 0.16047439 | -2.58024186 |
| 1 | 2.80573417 | 0.81628122 | -3.30811356 |
| 1 | 2.17089828 | -0.75600769 | -3.84635542 |
| 1 | 3.09465251 | -2.69045120 | -0.75494259 |
| 1 | 1.49345895 | -1.95039776 | -1.02445160 |
| 1 | 2.32884185 | -2.79203379 | -2.35620946 |
| 1 | -1.03211141 | 2.47946197 | 2.95170560 |
| 31 | -0.63364418 | 1.04054328 | 0.05634483 |
| 8 | 4.34005397 | -6.29234157 | 3.48730671 |
| 6 | 4.49763132 | -7.72208639 | 3.65463147 |
| 1 | 3.64224837 | -8.14120590 | 4.19918980 |
| 6 | 3.18775802 | -4.41490214 | 2.60725980 |
| 6 | 1.82485244 | -3.89059892 | 2.14896675 |
| 6 | 0.74508000 | -3.98140236 | 3.23559814 |
| 6 | 3.23147348 | -5.92194137 | 2.78271648 |

| | | | |
|---|---|---|---|
| 6 | -0.65245160 | -3.54127965 | 2.77195119 |
| 6 | -0.71718779 | -2.06376637 | 2.42173210 |
| 1 | 3.51221349 | -3.94128936 | 3.54626715 |
| 1 | 1.50116502 | -4.46081301 | 1.26475896 |
| 1 | 0.67510983 | -5.02261507 | 3.58375658 |
| 1 | -1.38293239 | -3.74557873 | 3.56997882 |
| 1 | 3.96366367 | -4.16431162 | 1.86487378 |
| 1 | 1.95447764 | -2.84756367 | 1.82462695 |
| 1 | 1.05013453 | -3.37728330 | 4.10836068 |
| 1 | -0.95796830 | -4.13612983 | 1.89695882 |
| 1 | -0.10846634 | -1.80384629 | 1.54504189 |
| 1 | -0.40799081 | -1.43293176 | 3.26832511 |
| 8 | 2.42020172 | -6.71664590 | 2.34497849 |
| 8 | -2.11006170 | -1.75217704 | 2.09688444 |
| 1 | 5.42121289 | -7.84614731 | 4.22810206 |
| 1 | 4.57657041 | -8.21646300 | 2.67822893 |

Electronic energy = -3908.665955 a.u.
DFT-D3(BJ) dispersion correction = -0.140075 a.u.
Thermal free energy = 0.738895 a.u.
Gibbs free energy = -3908.067136 a.u.
Number of imaginary frequencies = 0.

**Int4_eq_CL isomer/conformer 12**

| Atomic N. | X | Y | Z |
|---|---|---|---|
| 7 | 0.18203496 | 1.65728589 | 2.28683436 |
| 8 | -0.11408126 | 1.24537582 | -0.58780589 |
| 8 | 2.64107043 | 0.67518278 | 0.97109017 |
| 6 | -1.09855003 | 1.32602226 | 2.95901345 |
| 6 | -1.03620247 | 2.22074813 | -0.48389687 |
| 6 | -2.03023951 | 2.37146528 | -1.49937371 |
| 6 | -2.96286104 | 3.40588035 | -1.34114484 |
| 6 | -2.95471331 | 4.27504062 | -0.24561242 |
| 6 | -1.97971866 | 4.12106337 | 0.73444176 |
| 6 | -1.01858378 | 3.11163853 | 0.61838733 |
| 6 | 0.08709311 | 3.00786116 | 1.62834641 |
| 1 | -3.70331666 | 5.06329366 | -0.16605111 |
| 6 | -2.07873382 | 1.43266837 | -2.72015111 |
| 6 | 3.22904943 | 1.66605556 | 1.67435746 |
| 6 | 4.50085372 | 2.17166536 | 1.26252937 |
| 6 | 5.07979143 | 3.18174949 | 2.04299823 |
| 6 | 4.46242349 | 3.70924064 | 3.18049744 |
| 6 | 3.21926383 | 3.21943326 | 3.56368915 |
| 6 | 2.60275397 | 2.19927475 | 2.83004015 |
| 6 | 1.28650974 | 1.65072272 | 3.31402040 |
| 1 | 4.94904379 | 4.49932305 | 3.75222834 |
| 6 | 5.21336436 | 1.63830559 | 0.00407203 |
| 6 | -3.22434388 | 1.79568046 | -3.68361255 |
| 6 | -2.31288429 | -0.02504575 | -2.25667550 |
| 6 | -0.75721995 | 1.52833348 | -3.51885471 |
| 6 | 6.57148656 | 2.32682423 | -0.22919700 |
| 6 | 4.34427052 | 1.90472608 | -1.24770741 |
| 6 | 5.48871622 | 0.12353647 | 0.15028811 |

| | | | |
|---|---|---|---|
| 1 | -1.35285241 | 2.11772955 | 3.68315349 |
| 1 | -1.90045104 | 1.25768249 | 2.21600535 |
| 1 | -0.04794020 | 3.76668256 | 2.41783707 |
| 1 | 1.06426243 | 3.19282215 | 1.15921450 |
| 1 | -3.73243131 | 3.54334248 | -2.09890915 |
| 1 | -1.94566775 | 4.79456166 | 1.59414543 |
| 1 | 1.38048990 | 0.60333490 | 3.63857935 |
| 1 | 0.94617821 | 2.24445421 | 4.17941061 |
| 1 | 6.04901727 | 3.58275527 | 1.75228795 |
| 1 | 2.71456461 | 3.62045823 | 4.44575203 |
| 1 | -3.21010639 | 1.09871782 | -4.53395687 |
| 1 | -4.21026644 | 1.71442534 | -3.20253446 |
| 1 | -3.11851663 | 2.81282965 | -4.08762483 |
| 1 | -1.50711177 | -0.37050379 | -1.59883144 |
| 1 | -3.26917373 | -0.10355292 | -1.71729763 |
| 1 | -2.36192094 | -0.69391085 | -3.12955885 |
| 1 | -0.60818724 | 2.55071062 | -3.89581889 |
| 1 | 0.10480817 | 1.25762066 | -2.89968935 |
| 1 | -0.79451558 | 0.85019092 | -4.38525373 |
| 1 | 7.03276860 | 1.90726849 | -1.13470165 |
| 1 | 7.26883812 | 2.16042837 | 0.60465031 |
| 1 | 6.46602967 | 3.41020785 | -0.38442057 |
| 1 | 3.36215276 | 1.42627797 | -1.16951519 |
| 1 | 4.19185756 | 2.98470758 | -1.38829926 |
| 1 | 4.85007314 | 1.51547247 | -2.14446158 |
| 1 | 6.14468288 | -0.06874622 | 1.01207368 |
| 1 | 4.55805380 | -0.43688140 | 0.28805757 |
| 1 | 5.99541537 | -0.25359991 | -0.75123133 |
| 1 | -0.99458668 | 0.36870817 | 3.47710269 |
| 6 | 2.10877630 | -2.78044910 | 2.01541116 |
| 6 | 2.56014987 | -3.90897100 | 1.07153154 |
| 6 | 3.02048908 | -3.50828128 | -0.34414556 |
| 6 | 0.67365076 | -2.32428360 | 1.92448100 |
| 6 | 1.95139135 | -3.08643152 | -1.37224397 |
| 6 | 1.53272482 | -1.61738173 | -1.31676549 |
| 1 | 2.73695600 | -1.88584921 | 1.90862448 |
| 1 | 3.41367487 | -4.40601027 | 1.55912698 |
| 1 | 3.54983446 | -4.38467290 | -0.75227568 |
| 1 | 2.34772227 | -3.26985984 | -2.38526665 |
| 1 | 2.22524930 | -3.12154294 | 3.06144685 |
| 1 | 1.76760724 | -4.66928328 | 1.00388709 |
| 1 | 3.78020626 | -2.71178040 | -0.26708709 |
| 1 | 1.05484989 | -3.72099467 | -1.27042761 |
| 1 | 0.87443261 | -1.38986735 | -2.17639726 |
| 1 | 2.43498717 | -0.98370657 | -1.41468752 |
| 8 | 0.27927440 | -1.18640439 | 2.29329932 |
| 8 | 0.85173785 | -1.35795474 | -0.09737635 |
| 31 | 0.80397325 | 0.30714232 | 0.77793281 |
| 8 | -6.56079158 | -1.37752750 | -1.95341877 |
| 6 | -6.83902469 | -0.85128526 | -3.27497776 |
| 1 | -5.90454293 | -0.70739856 | -3.83139564 |
| 6 | -5.65550105 | -3.06715463 | -0.53750093 |
| 6 | -4.15700886 | -3.33219387 | -0.31983650 |

| | | | |
|---|---|---|---|
| 6 | -3.86638461 | -3.96686206 | 1.04550814 |
| 6 | -5.92913464 | -2.58658410 | -1.94935317 |
| 6 | -2.37309453 | -4.23411364 | 1.29861922 |
| 6 | -1.59190664 | -2.95551083 | 1.55792187 |
| 1 | -6.03881645 | -2.33770166 | 0.18816238 |
| 1 | -3.61029917 | -2.38286656 | -0.42802951 |
| 1 | -4.26995711 | -3.32817009 | 1.85052019 |
| 1 | -1.93671716 | -4.75439148 | 0.43149589 |
| 1 | -6.21683809 | -4.00621685 | -0.40409410 |
| 1 | -3.79661555 | -3.99572077 | -1.12055350 |
| 1 | -4.41002483 | -4.92253400 | 1.11509458 |
| 1 | -2.25587230 | -4.90132473 | 2.16622743 |
| 1 | -1.92896573 | -2.45397134 | 2.47641795 |
| 1 | -1.65148285 | -2.24458350 | 0.72250674 |
| 8 | -5.62243651 | -3.18868084 | -2.96149240 |
| 8 | -0.18598732 | -3.32246179 | 1.70733141 |
| 1 | -7.34126889 | 0.10573344 | -3.10494934 |
| 1 | -7.48675754 | -1.53944285 | -3.83218850 |

Electronic energy = -3908.666296 a.u.
DFT-D3(BJ) dispersion correction = -0.140403 a.u.
Thermal free energy = 0.740269 a.u.
Gibbs free energy = -3908.066430 a.u.
Number of imaginary frequencies = 0.

**Int4 eq CL isomer/conformer 13**

| Atomic N. | X | Y | Z |
|---|---|---|---|
| 7 | 0.67472008 | 1.74742895 | 1.08479570 |
| 8 | -0.01518431 | 1.00500498 | -1.65343089 |
| 8 | 2.87202107 | 0.38369313 | -0.35344752 |
| 6 | -0.52167521 | 1.61425109 | 1.95162618 |
| 6 | -0.86784334 | 2.04226558 | -1.55536733 |
| 6 | -1.99128987 | 2.12385417 | -2.43411966 |
| 6 | -2.84218132 | 3.22878107 | -2.29198075 |
| 6 | -2.63285778 | 4.23020210 | -1.33831880 |
| 6 | -1.53358528 | 4.14082472 | -0.49031257 |
| 6 | -0.64811599 | 3.06361679 | -0.59766541 |
| 6 | 0.57947850 | 3.00614973 | 0.26516015 |
| 1 | -3.32382329 | 5.07010300 | -1.26567765 |
| 6 | -2.25698328 | 1.04100776 | -3.49778289 |
| 6 | 3.61242779 | 1.40727836 | 0.12181772 |
| 6 | 4.85371975 | 1.73942226 | -0.50367931 |
| 6 | 5.59368927 | 2.79476428 | 0.04705730 |
| 6 | 5.16269224 | 3.52523445 | 1.15799008 |
| 6 | 3.94875167 | 3.19972123 | 1.75191066 |
| 6 | 3.17700521 | 2.14446767 | 1.25222906 |
| 6 | 1.90156893 | 1.77513375 | 1.96062685 |
| 1 | 5.77040783 | 4.34232012 | 1.54652001 |
| 6 | 5.36383537 | 0.97739220 | -1.74242230 |
| 6 | -3.51730064 | 1.34457973 | -4.32911860 |
| 6 | -2.47286391 | -0.33248815 | -2.81739361 |
| 6 | -1.06418712 | 0.95988410 | -4.48011132 |
| 6 | 6.72821609 | 1.50402090 | -2.22631766 |

| | | | |
|---|---|---|---|
| 6 | 4.36628832 | 1.14652083 | -2.91261910 |
| 6 | 5.54538771 | -0.52169730 | -1.40808786 |
| 1 | -0.62587571 | 2.51388249 | 2.58084951 |
| 1 | -1.41980576 | 1.51144400 | 1.33328531 |
| 1 | 0.60185214 | 3.86948674 | 0.95191158 |
| 1 | 1.49207268 | 3.04837105 | -0.34683199 |
| 1 | -3.70741077 | 3.31663605 | -2.94700474 |
| 1 | -1.34428971 | 4.91565223 | 0.25645552 |
| 1 | 1.96623299 | 0.77086347 | 2.40773411 |
| 1 | 1.72046814 | 2.49628513 | 2.77586895 |
| 1 | 6.54327124 | 3.06763550 | -0.40968567 |
| 1 | 3.58949250 | 3.75829999 | 2.61935148 |
| 1 | -3.65620331 | 0.54661457 | -5.07262918 |
| 1 | -4.42471987 | 1.38062314 | -3.70867241 |
| 1 | -3.43251010 | 2.29473189 | -4.87598276 |
| 1 | -1.58975079 | -0.64166560 | -2.24674855 |
| 1 | -3.33840542 | -0.29353059 | -2.13856082 |
| 1 | -2.67902719 | -1.09816539 | -3.58115792 |
| 1 | -0.93284808 | 1.91639701 | -5.00676274 |
| 1 | -0.13174336 | 0.71976485 | -3.95777414 |
| 1 | -1.25452427 | 0.18155634 | -5.23515321 |
| 1 | 7.04280330 | 0.92370819 | -3.10554857 |
| 1 | 7.50964600 | 1.39418392 | -1.46044105 |
| 1 | 6.68173939 | 2.56037759 | -2.52781144 |
| 1 | 3.36993956 | 0.77240530 | -2.65397710 |
| 1 | 4.27519107 | 2.20639807 | -3.19134632 |
| 1 | 4.72613569 | 0.59490126 | -3.79456956 |
| 1 | 6.29049319 | -0.65206976 | -0.60947194 |
| 1 | 4.60217832 | -0.97254071 | -1.08203066 |
| 1 | 5.90451085 | -1.06251303 | -2.29707684 |
| 1 | -0.40955790 | 0.73056349 | 2.58586176 |
| 6 | 2.07735596 | -2.89183995 | 1.18028205 |
| 6 | 2.33849228 | -4.16819161 | 0.36232569 |
| 6 | 2.74014207 | -3.99140363 | -1.11512822 |
| 6 | 0.69797650 | -2.28504554 | 1.10937521 |
| 6 | 1.65323165 | -3.56281428 | -2.12102834 |
| 6 | 1.40269236 | -2.05750307 | -2.20801027 |
| 1 | 2.79255986 | -2.09664837 | 0.93050737 |
| 1 | 3.16459515 | -4.69990305 | 0.86050680 |
| 1 | 3.13669865 | -4.96596053 | -1.44356837 |
| 1 | 1.95474580 | -3.89818540 | -3.12779137 |
| 1 | 2.22282857 | -3.11871182 | 2.25353642 |
| 1 | 1.46521124 | -4.83395040 | 0.43351795 |
| 1 | 3.58816995 | -3.28821659 | -1.17937219 |
| 1 | 0.70295045 | -4.07486996 | -1.89413511 |
| 1 | 0.70859201 | -1.84789676 | -3.04404679 |
| 1 | 2.35840452 | -1.54765940 | -2.43525939 |
| 8 | 0.45944592 | -1.07252389 | 1.37436035 |
| 8 | 0.84923060 | -1.58929723 | -0.98798983 |
| 31 | 1.00182495 | 0.16241788 | -0.29946880 |
| 8 | -7.81114971 | -1.16045832 | 4.89091988 |
| 6 | -8.50051099 | -0.92136709 | 6.14207319 |
| 1 | -8.39865390 | -1.78776527 | 6.80747967 |

| | | | |
|---|---|---|---|
| 6 | -5.83967426 | -1.60159651 | 3.65251389 |
| 6 | -4.36576737 | -1.99827467 | 3.72803699 |
| 6 | -3.73488763 | -2.16624167 | 2.34181521 |
| 6 | -6.47097266 | -1.38436335 | 5.01473444 |
| 6 | -2.25762852 | -2.57342882 | 2.41111516 |
| 6 | -1.65457039 | -2.71380420 | 1.02026617 |
| 1 | -6.43322174 | -2.35951841 | 3.11720549 |
| 1 | -4.26925251 | -2.93511968 | 4.29840206 |
| 1 | -4.29830443 | -2.92331669 | 1.76935539 |
| 1 | -2.15858611 | -3.53076993 | 2.94713650 |
| 1 | -5.96733698 | -0.66922503 | 3.07737018 |
| 1 | -3.81454108 | -1.23796217 | 4.30162612 |
| 1 | -3.82945818 | -1.22165571 | 1.77885549 |
| 1 | -1.68872950 | -1.82212705 | 2.97957487 |
| 1 | -1.67315473 | -1.76698036 | 0.46661823 |
| 1 | -2.17896327 | -3.48140611 | 0.43591936 |
| 8 | -5.88695747 | -1.39560288 | 6.08178294 |
| 8 | -0.27624268 | -3.19689495 | 1.07264528 |
| 1 | -9.54804802 | -0.76244765 | 5.86910663 |
| 1 | -8.09020966 | -0.03550457 | 6.64287264 |

Electronic energy = -3908.666695 a.u.
DFT-D3(BJ) dispersion correction = -0.138322 a.u.
Thermal free energy = 0.739005 a.u.
Gibbs free energy = -3908.066013 a.u.
Number of imaginary frequencies = 0.

**Int4 eq CL isomer/conformer 14**

| Atomic N. | X | Y | Z |
|---|---|---|---|
| 6 | -2.68785235 | -0.82726280 | 2.81423210 |
| 6 | -3.56457722 | -1.91935417 | 2.17798658 |
| 6 | -3.89033392 | -1.78808166 | 0.67618657 |
| 6 | -1.19116132 | -0.94475454 | 2.65748928 |
| 6 | -2.78190816 | -2.13799691 | -0.33725759 |
| 6 | -1.82602357 | -0.99428271 | -0.67303614 |
| 1 | -2.97034114 | 0.17114273 | 2.45644671 |
| 1 | -4.52273134 | -1.90747179 | 2.72161326 |
| 1 | -4.74234356 | -2.46155068 | 0.48813305 |
| 1 | -3.24785842 | -2.45870452 | -1.28333375 |
| 1 | -2.85598344 | -0.82890785 | 3.90778633 |
| 1 | -3.11883127 | -2.90607836 | 2.37643546 |
| 1 | -4.26445534 | -0.77058431 | 0.47134616 |
| 1 | -2.20147738 | -3.00161220 | 0.02772121 |
| 1 | -1.10050688 | -1.33606715 | -1.43464560 |
| 1 | -2.40012351 | -0.15516778 | -1.10809052 |
| 8 | -0.40876983 | 0.04477174 | 2.74239372 |
| 8 | -1.15366999 | -0.58206395 | 0.50592720 |
| 7 | 0.90438229 | 2.44176007 | 1.95176778 |
| 8 | -1.14367972 | 2.20861300 | -0.11962093 |
| 8 | 1.49608398 | 0.30108262 | 0.04297514 |
| 6 | 0.20640267 | 2.88506336 | 3.18294868 |
| 6 | -1.24389069 | 3.54438455 | 0.04145360 |
| 6 | -2.41889139 | 4.22530350 | -0.40632135 |

| | | | |
|---|---|---|---|
| 6 | -2.47882827 | 5.61205678 | -0.21236876 |
| 6 | -1.44909709 | 6.33934678 | 0.39128021 |
| 6 | -0.30671549 | 5.66884369 | 0.81361747 |
| 6 | -0.19109849 | 4.28616637 | 0.63440932 |
| 6 | 1.09040360 | 3.59966504 | 1.01185816 |
| 1 | -1.54306378 | 7.41713616 | 0.52390578 |
| 6 | -3.57868764 | 3.47099865 | -1.08429860 |
| 6 | 2.74490762 | 0.80220875 | 0.08347481 |
| 6 | 3.65433423 | 0.52480765 | -0.98382308 |
| 6 | 4.95122356 | 1.04528331 | -0.87906644 |
| 6 | 5.37156703 | 1.82198305 | 0.20505366 |
| 6 | 4.47270196 | 2.09904929 | 1.22902445 |
| 6 | 3.16986868 | 1.58963028 | 1.18343218 |
| 6 | 2.23894478 | 1.85602875 | 2.33626773 |
| 1 | 6.38895697 | 2.21163642 | 0.23835073 |
| 6 | 3.22504844 | -0.30390725 | -2.21047520 |
| 6 | -4.72596662 | 4.41352659 | -1.49390702 |
| 6 | -4.17051910 | 2.42698865 | -0.10875191 |
| 6 | -3.07448640 | 2.77629376 | -2.37146423 |
| 6 | 4.37456483 | -0.47876421 | -3.22072530 |
| 6 | 2.06703505 | 0.40780485 | -2.94997055 |
| 6 | 2.77792031 | -1.71875400 | -1.77372091 |
| 1 | 0.76917747 | 3.70834588 | 3.65369814 |
| 1 | -0.79855218 | 3.24161776 | 2.93346259 |
| 1 | 1.77829820 | 4.32274085 | 1.48260929 |
| 1 | 1.59882429 | 3.19997257 | 0.12223304 |
| 1 | -3.36441138 | 6.15259980 | -0.54174569 |
| 1 | 0.51788055 | 6.21582692 | 1.27678072 |
| 1 | 2.00649233 | 0.93310539 | 2.89008006 |
| 1 | 2.72675408 | 2.55480181 | 3.03744552 |
| 1 | 5.66533915 | 0.84806898 | -1.67670354 |
| 1 | 4.77645221 | 2.71109939 | 2.08162411 |
| 1 | -5.51812942 | 3.82292424 | -1.97624496 |
| 1 | -5.17375337 | 4.92340739 | -0.62850673 |
| 1 | -4.39692472 | 5.17557652 | -2.21494042 |
| 1 | -3.41141584 | 1.70150492 | 0.20327895 |
| 1 | -4.57346685 | 2.92196755 | 0.78745703 |
| 1 | -4.99530434 | 1.88347741 | -0.59492109 |
| 1 | -2.71213385 | 3.52298470 | -3.09297449 |
| 1 | -2.25803709 | 2.07781480 | -2.15845642 |
| 1 | -3.89814202 | 2.22047740 | -2.84542103 |
| 1 | 4.01479567 | -1.07861560 | -4.06902141 |
| 1 | 5.23418381 | -1.00644052 | -2.78226122 |
| 1 | 4.72472271 | 0.48332718 | -3.62189384 |
| 1 | 1.19111511 | 0.53036092 | -2.30405041 |
| 1 | 2.38419683 | 1.40102925 | -3.29996925 |
| 1 | 1.76698282 | -0.18254849 | -3.82891048 |
| 1 | 3.61077753 | -2.25615463 | -1.29562558 |
| 1 | 1.93910614 | -1.66949555 | -1.07187394 |
| 1 | 2.45637657 | -2.29608574 | -2.65386706 |
| 1 | 0.13126150 | 2.04227067 | 3.87584336 |
| 31 | -0.09620911 | 0.94563569 | 0.82031014 |
| 8 | 1.61334635 | -6.12176753 | -2.41948254 |

| | | | |
|---|---|---|---|
| 6 | 2.05836116 | -5.85001423 | -3.77071587 |
| 1 | 1.23645675 | -5.99928775 | -4.48189146 |
| 6 | 0.20654392 | -5.64567674 | -0.56083746 |
| 6 | 0.80294603 | -4.59374299 | 0.39492501 |
| 6 | 0.35086675 | -4.81859263 | 1.84186280 |
| 6 | 0.57225060 | -5.34375247 | -1.99868731 |
| 6 | 0.99695465 | -3.86823381 | 2.86166615 |
| 6 | 0.70231981 | -2.39018590 | 2.66084424 |
| 1 | -0.89073363 | -5.62227247 | -0.49281971 |
| 1 | 0.49985823 | -3.59475355 | 0.04916925 |
| 1 | -0.74521297 | -4.72544790 | 1.90060241 |
| 1 | 2.09399197 | -3.97862313 | 2.82814436 |
| 1 | 0.55635928 | -6.65040454 | -0.28701956 |
| 1 | 1.90321027 | -4.63247327 | 0.33762859 |
| 1 | 0.58915226 | -5.85287008 | 2.13967196 |
| 1 | 0.68599144 | -4.15726093 | 3.87808219 |
| 1 | 1.00257003 | -2.02355030 | 1.66911681 |
| 1 | 1.20094236 | -1.77839468 | 3.42464426 |
| 8 | 0.04132690 | -4.49898473 | -2.69549582 |
| 8 | -0.74357034 | -2.19693878 | 2.78114869 |
| 1 | 2.86886442 | -6.56108920 | -3.95717385 |
| 1 | 2.42047424 | -4.81748944 | -3.85345795 |

Electronic energy = -3908.665414 a.u.
DFT-D3(BJ) dispersion correction = -0.142515 a.u.
Thermal free energy = 0.742119 a.u.
Gibbs free energy = -3908.065810 a.u.
Number of imaginary frequencies = 0.

**Int4 eq CL isomer/conformer 15**

| Atomic N. | X | Y | Z |
|---|---|---|---|
| 6 | -3.37219619 | 0.27767770 | 1.60325676 |
| 6 | -4.21071414 | -0.86145338 | 0.99770698 |
| 6 | -4.11855184 | -1.08653050 | -0.52572690 |
| 6 | -1.92167480 | -0.00523920 | 1.91074242 |
| 6 | -2.85863078 | -1.78352894 | -1.08008165 |
| 6 | -1.69066589 | -0.84625083 | -1.38775620 |
| 1 | -3.39702813 | 1.17393788 | 0.97041395 |
| 1 | -5.26308898 | -0.63189810 | 1.22886792 |
| 1 | -4.99070793 | -1.70370974 | -0.79610485 |
| 1 | -3.11903479 | -2.30071536 | -2.01876620 |
| 1 | -3.81483504 | 0.56497239 | 2.57557348 |
| 1 | -3.98444454 | -1.80000198 | 1.52612786 |
| 1 | -4.26128853 | -0.12461046 | -1.04717307 |
| 1 | -2.52269289 | -2.56312960 | -0.37586809 |
| 1 | -0.84821079 | -1.43179661 | -1.80461261 |
| 1 | -2.00165071 | -0.11612681 | -2.15874602 |
| 8 | -1.04053225 | 0.89697180 | 1.98246293 |
| 8 | -1.30421763 | -0.18747525 | -0.19753262 |
| 7 | 0.83847731 | 2.84135299 | 1.07953125 |
| 8 | -0.57142223 | 2.28169518 | -1.41490795 |
| 8 | 1.49353853 | 0.19276785 | 0.00424541 |
| 6 | -0.06925583 | 3.68384432 | 1.89616500 |

| | | | |
|---|---|---|---|
| 6 | -0.46340806 | 3.61351440 | -1.59867645 |
| 6 | -1.33267056 | 4.27430867 | -2.52153216 |
| 6 | -1.18048380 | 5.65887517 | -2.67726599 |
| 6 | -0.22789102 | 6.40168753 | -1.97379750 |
| 6 | 0.61544595 | 5.74789873 | -1.08232028 |
| 6 | 0.51302410 | 4.36499006 | -0.89774696 |
| 6 | 1.48978500 | 3.66464915 | 0.00285024 |
| 1 | -0.14971040 | 7.47810430 | -2.12624976 |
| 6 | -2.39751222 | 3.49908451 | -3.32085830 |
| 6 | 2.75795398 | 0.52815370 | 0.32191455 |
| 6 | 3.84711148 | -0.16265184 | -0.29349051 |
| 6 | 5.14438512 | 0.20784617 | 0.08548332 |
| 6 | 5.40200190 | 1.21712580 | 1.01828350 |
| 6 | 4.33393456 | 1.88780755 | 1.60382483 |
| 6 | 3.01722572 | 1.54623620 | 1.27427613 |
| 6 | 1.88296960 | 2.23828029 | 1.98322981 |
| 1 | 6.42890800 | 1.47657333 | 1.27540811 |
| 6 | 3.60763615 | -1.26847116 | -1.34072548 |
| 6 | -3.20497090 | 4.41733758 | -4.25746516 |
| 6 | -3.40370119 | 2.83459851 | -2.35191234 |
| 6 | -1.71722629 | 2.42652255 | -4.20424370 |
| 6 | 4.92817379 | -1.87794691 | -1.84810942 |
| 6 | 2.86876394 | -0.68412143 | -2.56813728 |
| 6 | 2.77703896 | -2.42031475 | -0.72586258 |
| 1 | 0.49193585 | 4.53337229 | 2.31954148 |
| 1 | -0.87950313 | 4.07257787 | 1.27032440 |
| 1 | 2.15277936 | 4.40349729 | 0.48447552 |
| 1 | 2.12990558 | 2.97725040 | -0.56956398 |
| 1 | -1.83155839 | 6.18603593 | -3.37241242 |
| 1 | 1.37449799 | 6.30429274 | -0.52741404 |
| 1 | 1.33723347 | 1.54474570 | 2.64150086 |
| 1 | 2.29331869 | 3.04654388 | 2.61260760 |
| 1 | 5.99316076 | -0.30186138 | -0.36707790 |
| 1 | 4.51213805 | 2.68196146 | 2.33273766 |
| 1 | -3.94241780 | 3.81130037 | -4.80324506 |
| 1 | -3.75693174 | 5.19166287 | -3.70508303 |
| 1 | -2.56594686 | 4.91101075 | -5.00367162 |
| 1 | -2.90011773 | 2.14006030 | -1.67059930 |
| 1 | -3.92437404 | 3.59752223 | -1.75411887 |
| 1 | -4.16222242 | 2.27678671 | -2.92245575 |
| 1 | -1.04783062 | 2.90101595 | -4.93652515 |
| 1 | -1.12919891 | 1.72471160 | -3.60289985 |
| 1 | -2.47984961 | 1.86027534 | -4.76091939 |
| 1 | 4.70041431 | -2.66046694 | -2.58598530 |
| 1 | 5.50694776 | -2.34487399 | -1.03787153 |
| 1 | 5.56498864 | -1.13161993 | -2.34452250 |
| 1 | 1.89491203 | -0.26630867 | -2.29117961 |
| 1 | 3.46752876 | 0.10987886 | -3.03759269 |
| 1 | 2.70714945 | -1.47380335 | -3.31777132 |
| 1 | 3.31036539 | -2.86673285 | 0.12691112 |
| 1 | 1.79798260 | -2.06686984 | -0.38434716 |
| 1 | 2.62201801 | -3.20967868 | -1.47732114 |
| 1 | -0.49107400 | 3.07749580 | 2.70271584 |

| | | | |
|---|---|---|---|
| 31 | -0.08749142 | 1.21905786 | 0.07307888 |
| 8 | -0.95303902 | -6.59729793 | 1.40528946 |
| 6 | -2.34755048 | -6.94366471 | 1.22125757 |
| 1 | -2.61396887 | -7.80737431 | 1.84319585 |
| 6 | 0.87381641 | -5.99202144 | 2.79975724 |
| 6 | 1.23376148 | -4.97184540 | 3.89013827 |
| 6 | 1.11340468 | -3.50252102 | 3.45656339 |
| 6 | -0.61378206 | -6.27213807 | 2.68648970 |
| 6 | -0.30734562 | -3.05228838 | 3.09058582 |
| 6 | -0.33704946 | -1.57149796 | 2.75543047 |
| 1 | 1.33656686 | -6.96444493 | 3.03877852 |
| 1 | 2.27311226 | -5.15185844 | 4.20280999 |
| 1 | 1.49054607 | -2.87146764 | 4.27873255 |
| 1 | -0.99716180 | -3.26219874 | 3.92230491 |
| 1 | 1.26771053 | -5.69655606 | 1.81678944 |
| 1 | 0.59949843 | -5.16030363 | 4.76981395 |
| 1 | 1.78443013 | -3.32293394 | 2.59939768 |
| 1 | -0.67694490 | -3.61149439 | 2.21733984 |
| 1 | 0.30027666 | -1.33045198 | 1.89268060 |
| 1 | -0.02801403 | -0.95103108 | 3.60959203 |
| 8 | -1.41035941 | -6.25318605 | 3.60641880 |
| 8 | -1.71680649 | -1.22308396 | 2.41091528 |
| 1 | -2.44768159 | -7.18381039 | 0.15854646 |
| 1 | -2.99013556 | -6.09621167 | 1.49116202 |

Electronic energy = -3908.665008 a.u.
DFT-D3(BJ) dispersion correction = -0.140118 a.u.
Thermal free energy = 0.741327 a.u.
Gibbs free energy = -3908.063799 a.u.
Number of imaginary frequencies = 0.

**Int4 eq CL isomer/conformer 16**

| Atomic N. | X | Y | Z |
|---|---|---|---|
| 6 | -3.18827180 | -0.63707420 | 2.51352683 |
| 6 | -4.18955520 | -1.59607051 | 1.84674355 |
| 6 | -4.57264756 | -1.32219567 | 0.37790696 |
| 6 | -1.71754567 | -0.87586091 | 2.27128734 |
| 6 | -3.55094079 | -1.69149477 | -0.71714585 |
| 6 | -2.51881755 | -0.60646678 | -1.02336310 |
| 1 | -3.39244332 | 0.40691932 | 2.24384767 |
| 1 | -5.11654527 | -1.54211547 | 2.43959235 |
| 1 | -5.49165145 | -1.90049423 | 0.18920276 |
| 1 | -4.09194499 | -1.90176424 | -1.65491462 |
| 1 | -3.30643770 | -0.70850880 | 3.61124530 |
| 1 | -3.82684463 | -2.63016434 | 1.94879264 |
| 1 | -4.85900497 | -0.26243213 | 0.26585871 |
| 1 | -3.02890318 | -2.62327779 | -0.44215454 |
| 1 | -1.85482399 | -0.94948871 | -1.84049445 |
| 1 | -3.03964337 | 0.30294568 | -1.37822315 |
| 8 | -0.84244997 | 0.02913922 | 2.37962028 |
| 8 | -1.77308986 | -0.33335870 | 0.14727001 |
| 7 | 0.71443763 | 2.30686408 | 1.65173276 |
| 8 | -1.42024226 | 2.45301000 | -0.33601975 |

| | | | |
|---|---|---|---|
| 8 | 0.94781185 | 0.20735303 | -0.38101168 |
| 6 | 0.12480300 | 2.76237523 | 2.93440587 |
| 6 | -1.34843429 | 3.77810671 | -0.09366001 |
| 6 | -2.45000370 | 4.62167738 | -0.43812030 |
| 6 | -2.32744429 | 5.99115436 | -0.16621780 |
| 6 | -1.18784527 | 6.54954879 | 0.41984675 |
| 6 | -0.12021073 | 5.72049657 | 0.74566016 |
| 6 | -0.18656993 | 4.34757847 | 0.48540267 |
| 6 | 1.01023654 | 3.48398034 | 0.76410396 |
| 1 | -1.14034564 | 7.62078806 | 0.61531724 |
| 6 | -3.72626925 | 4.05435361 | -1.08856100 |
| 6 | 2.25482028 | 0.53020587 | -0.36831273 |
| 6 | 3.08224750 | 0.16370147 | -1.47378155 |
| 6 | 4.44027944 | 0.50244271 | -1.40480655 |
| 6 | 4.99695452 | 1.18190012 | -0.31696646 |
| 6 | 4.17975830 | 1.53945386 | 0.74961415 |
| 6 | 2.81933123 | 1.21068120 | 0.73992522 |
| 6 | 1.97833596 | 1.53975942 | 1.94495859 |
| 1 | 6.05822010 | 1.42982611 | -0.31077156 |
| 6 | 2.50346514 | -0.56846363 | -2.70088043 |
| 6 | -4.76851687 | 5.15106221 | -1.37778845 |
| 6 | -4.39206687 | 3.02972792 | -0.14012471 |
| 6 | -3.37706183 | 3.38642675 | -2.43967462 |
| 6 | 3.58825968 | -0.88416716 | -3.74789587 |
| 6 | 1.43942612 | 0.31725003 | -3.39157631 |
| 6 | 1.87012773 | -1.91489306 | -2.27600438 |
| 1 | 0.80639784 | 3.47745050 | 3.42426519 |
| 1 | -0.83345880 | 3.25864619 | 2.74808500 |
| 1 | 1.80467955 | 4.08514846 | 1.23828042 |
| 1 | 1.42540534 | 3.07363806 | -0.16823211 |
| 1 | -3.15328520 | 6.65456637 | -0.41689278 |
| 1 | 0.78551264 | 6.13375302 | 1.19566827 |
| 1 | 1.65353313 | 0.62793342 | 2.46974023 |
| 1 | 2.58070581 | 2.13702720 | 2.65073795 |
| 1 | 5.09516258 | 0.23464383 | -2.23208892 |
| 1 | 4.59382514 | 2.07128902 | 1.60946471 |
| 1 | -5.65134874 | 4.69065365 | -1.84429176 |
| 1 | -5.10464190 | 5.65656437 | -0.46081965 |
| 1 | -4.38591522 | 5.91125743 | -2.07389905 |
| 1 | -3.71453590 | 2.19922940 | 0.08661709 |
| 1 | -4.68460699 | 3.51256590 | 0.80421344 |
| 1 | -5.30175131 | 2.62164427 | -0.60700354 |
| 1 | -2.96226479 | 4.12794597 | -3.13789647 |
| 1 | -2.64313247 | 2.58311107 | -2.31298178 |
| 1 | -4.28591309 | 2.96433952 | -2.89569823 |
| 1 | 3.12496687 | -1.40851394 | -4.59596993 |
| 1 | 4.37598282 | -1.53650460 | -3.34321493 |
| 1 | 4.06143259 | 0.02651430 | -4.14236474 |
| 1 | 0.61106203 | 0.55291511 | -2.71469621 |
| 1 | 1.88670514 | 1.26085357 | -3.73627183 |
| 1 | 1.03139841 | -0.20473388 | -4.27069022 |
| 1 | 2.62234702 | -2.56274386 | -1.80167608 |
| 1 | 1.04553984 | -1.76264670 | -1.57129265 |

| | | | |
|---|---|---|---|
| 1 | 1.48141862 | -2.43942304 | -3.16243745 |
| 1 | -0.03412970 | 1.89617688 | 3.58294791 |
| 31 | -0.51121935 | 1.01829850 | 0.49472529 |
| 8 | 3.40380032 | -6.03198517 | -0.65104797 |
| 6 | 4.18197892 | -5.59483507 | -1.79185296 |
| 1 | 4.50232848 | -4.55400544 | -1.66058832 |
| 6 | 3.19137684 | -6.46045693 | 1.68415148 |
| 6 | 1.73740619 | -5.96463933 | 1.68989635 |
| 6 | 1.60798725 | -4.44578640 | 1.85214005 |
| 6 | 4.05536840 | -5.94696486 | 0.54552383 |
| 6 | 0.14619571 | -3.99555461 | 1.96432974 |
| 6 | 0.03018469 | -2.48619755 | 2.08773904 |
| 1 | 3.20113529 | -7.56109522 | 1.61940244 |
| 1 | 1.21003468 | -6.46484353 | 2.51861811 |
| 1 | 2.16270277 | -4.12526770 | 2.75058177 |
| 1 | -0.32313840 | -4.46849777 | 2.84209516 |
| 1 | 3.70803520 | -6.18644858 | 2.61361645 |
| 1 | 1.23813693 | -6.28117213 | 0.76212708 |
| 1 | 2.08432257 | -3.94130970 | 0.99534939 |
| 1 | -0.41800792 | -4.32842542 | 1.07897313 |
| 1 | 0.38440569 | -1.96754774 | 1.18549137 |
| 1 | 0.58449659 | -2.10309841 | 2.95711075 |
| 8 | 5.19457754 | -5.53647963 | 0.66000539 |
| 8 | -1.38683886 | -2.16813042 | 2.26953592 |
| 1 | 3.51290640 | -5.68989668 | -2.65237173 |
| 1 | 5.06879001 | -6.22939907 | -1.91454555 |

Electronic energy = -3908.665535 a.u.
DFT-D3(BJ) dispersion correction = -0.139709 a.u.
Thermal free energy = 0.741627 a.u.
Gibbs free energy = -3908.063616 a.u.
Number of imaginary frequencies = 0.

**Int4 eq CL isomer/conformer 17**

| Atomic N. | X | Y | Z |
|---|---|---|---|
| 6 | -2.65274642 | -0.46048836 | 1.91911548 |
| 6 | -3.52489563 | -1.63381644 | 1.43909331 |
| 6 | -3.90342100 | -1.68077709 | -0.05585420 |
| 6 | -1.15821984 | -0.56810850 | 1.72848955 |
| 6 | -2.82462134 | -2.13516539 | -1.06106923 |
| 6 | -1.92297374 | -1.01514643 | -1.57992969 |
| 1 | -2.96711496 | 0.48563648 | 1.46055245 |
| 1 | -4.46523557 | -1.57853171 | 2.01057030 |
| 1 | -4.75157467 | -2.38017378 | -0.13334244 |
| 1 | -3.31794462 | -2.59054895 | -1.93594814 |
| 1 | -2.78820006 | -0.34254111 | 3.01096933 |
| 1 | -3.05015229 | -2.58301254 | 1.73057955 |
| 1 | -4.29853645 | -0.69771898 | -0.36421892 |
| 1 | -2.20106934 | -2.92404707 | -0.60847219 |
| 1 | -1.19692241 | -1.43121508 | -2.30493229 |
| 1 | -2.53828359 | -0.26891120 | -2.11664657 |
| 8 | -0.39839906 | 0.43470256 | 1.65138000 |
| 8 | -1.25481170 | -0.42210341 | -0.48255658 |

| | | | |
|---|---|---|---|
| 7 | 0.79364470 | 2.76006309 | 0.49456466 |
| 8 | -1.19485615 | 2.20841806 | -1.56870166 |
| 8 | 1.43049295 | 0.33925830 | -1.03635670 |
| 6 | 0.06352277 | 3.37331346 | 1.63076678 |
| 6 | -1.31256835 | 3.55151664 | -1.60567679 |
| 6 | -2.48523894 | 4.14643233 | -2.16704136 |
| 6 | -2.55795744 | 5.54606537 | -2.18165783 |
| 6 | -1.54461044 | 6.36458551 | -1.67442985 |
| 6 | -0.40659666 | 5.77536015 | -1.13496589 |
| 6 | -0.27849235 | 4.38261242 | -1.10665562 |
| 6 | 0.99530261 | 3.76696913 | -0.60434620 |
| 1 | -1.64856565 | 7.44914774 | -1.70502971 |
| 6 | -3.63026925 | 3.28766126 | -2.73687526 |
| 6 | 2.67958132 | 0.84307795 | -1.04925580 |
| 6 | 3.61585337 | 0.38089934 | -2.02440918 |
| 6 | 4.90880303 | 0.91989687 | -1.98423365 |
| 6 | 5.30028653 | 1.88128007 | -1.04735615 |
| 6 | 4.37794373 | 2.32741749 | -0.10737395 |
| 6 | 3.07740326 | 1.81009705 | -0.09163544 |
| 6 | 2.12597937 | 2.25591539 | 0.98755955 |
| 1 | 6.31550097 | 2.27768613 | -1.06064290 |
| 6 | 3.22050221 | -0.66643284 | -3.08430072 |
| 6 | -4.77616992 | 4.14687118 | -3.30340808 |
| 6 | -4.23032478 | 2.40434375 | -1.61771279 |
| 6 | -3.10452537 | 2.40486034 | -3.89351071 |
| 6 | 4.39926677 | -1.02262931 | -4.00969713 |
| 6 | 2.08514497 | -0.11237344 | -3.97764980 |
| 6 | 2.76130280 | -1.97647575 | -2.40038795 |
| 1 | 0.61464742 | 4.25613163 | 1.99509620 |
| 1 | -0.93292197 | 3.68979944 | 1.30434626 |
| 1 | 1.67134588 | 4.55492488 | -0.23081493 |
| 1 | 1.52397233 | 3.24131558 | -1.41314251 |
| 1 | -3.44120737 | 6.02356308 | -2.60224362 |
| 1 | 0.40414969 | 6.39280212 | -0.74109886 |
| 1 | 1.89146656 | 1.43447355 | 1.68151060 |
| 1 | 2.59811081 | 3.06525781 | 1.57050353 |
| 1 | 5.64333178 | 0.58586271 | -2.71488887 |
| 1 | 4.66177417 | 3.07939929 | 0.63271215 |
| 1 | -5.55829256 | 3.48300504 | -3.69906768 |
| 1 | -5.23924998 | 4.78136781 | -2.53380433 |
| 1 | -4.44024335 | 4.79045260 | -4.12918123 |
| 1 | -3.47097734 | 1.74826127 | -1.17816332 |
| 1 | -4.65698320 | 3.03060132 | -0.82002432 |
| 1 | -5.03935420 | 1.77870775 | -2.02529164 |
| 1 | -2.73271707 | 3.03278420 | -4.71629453 |
| 1 | -2.28996789 | 1.75152991 | -3.56280822 |
| 1 | -3.91985941 | 1.77916374 | -4.28818796 |
| 1 | 4.06555340 | -1.77313612 | -4.74041254 |
| 1 | 5.24501673 | -1.45410159 | -3.45477821 |
| 1 | 4.76148324 | -0.15104702 | -4.57386624 |
| 1 | 1.19081771 | 0.12582647 | -3.39184167 |
| 1 | 2.41271408 | 0.79863790 | -4.49939421 |
| 1 | 1.81185267 | -0.85853376 | -4.73931717 |

| | | | |
|---|---|---|---|
| 1 | 3.57281438 | -2.39829287 | -1.78905913 |
| 1 | 1.88899225 | -1.80800972 | -1.75966315 |
| 1 | 2.49466139 | -2.72125623 | -3.16577821 |
| 1 | -0.03205079 | 2.63648910 | 2.43347012 |
| 31 | -0.17303070 | 1.10758972 | -0.41657106 |
| 8 | 0.78890885 | -7.24141940 | 6.19405785 |
| 6 | 0.94620350 | -8.67696104 | 6.08514055 |
| 1 | 0.28852920 | -9.07685036 | 5.30318208 |
| 6 | 0.87485430 | -5.06240551 | 5.26548487 |
| 6 | 1.29026386 | -4.21821786 | 4.06089516 |
| 6 | 1.02723160 | -2.72500751 | 4.28602552 |
| 6 | 1.08680349 | -6.54968311 | 5.05554646 |
| 6 | 1.48853875 | -1.81985701 | 3.13315493 |
| 6 | 0.75108245 | -2.01784274 | 1.81415556 |
| 1 | -0.19116867 | -4.90977537 | 5.50309158 |
| 1 | 0.74984820 | -4.58063340 | 3.17437244 |
| 1 | -0.04811243 | -2.56342826 | 4.46876716 |
| 1 | 2.55804427 | -1.99709222 | 2.92710645 |
| 1 | 1.42330567 | -4.76402817 | 6.17324136 |
| 1 | 2.35932469 | -4.38317891 | 3.85281864 |
| 1 | 1.54417981 | -2.40149624 | 5.20460635 |
| 1 | 1.39853355 | -0.76445841 | 3.43123233 |
| 1 | 0.81737297 | -3.05415026 | 1.46058277 |
| 1 | 1.13072373 | -1.34946891 | 1.03182885 |
| 8 | 1.46243066 | -7.07928620 | 4.02704873 |
| 8 | -0.69185908 | -1.79710743 | 1.95937666 |
| 1 | 0.66833327 | -9.07355879 | 7.06632111 |
| 1 | 1.98554530 | -8.93153318 | 5.84265777 |

Electronic energy = -3908.664285 a.u.
DFT-D3(BJ) dispersion correction = -0.138619 a.u.
Thermal free energy = 0.739536 a.u.
Gibbs free energy = -3908.063368 a.u.
Number of imaginary frequencies = 0.

**Int4 CL isomer/conformer 1**

| Atomic N. | X | Y | Z |
|---|---|---|---|
| 6 | -0.20735816 | 5.52933669 | -0.52093367 |
| 6 | -1.47139760 | 4.67577246 | -0.67114755 |
| 6 | -1.49608893 | 3.85773695 | -1.96810362 |
| 6 | 1.03379040 | 4.72241821 | -0.19130227 |
| 6 | -2.77275984 | 3.02645924 | -2.16089530 |
| 6 | -2.92320807 | 1.85116572 | -1.19393504 |
| 1 | -0.32819175 | 6.24483969 | 0.31070175 |
| 1 | -2.34684367 | 5.34477685 | -0.64499763 |
| 1 | -1.38639668 | 4.54497687 | -2.82407630 |
| 1 | -2.79117983 | 2.62227007 | -3.18546478 |
| 1 | -0.01261903 | 6.13357063 | -1.41888203 |
| 1 | -1.55124474 | 4.01274033 | 0.20257194 |
| 1 | -0.62939850 | 3.17885948 | -1.99801494 |
| 1 | -3.66262124 | 3.67271493 | -2.05751718 |
| 1 | -3.90450356 | 1.37158397 | -1.37109461 |
| 1 | -2.92287863 | 2.21123266 | -0.14816509 |

| | | | |
|---|---|---|---|
| 8 | 1.04771852 | 3.65957932 | 0.40739632 |
| 8 | -1.86540565 | 0.91271812 | -1.41883178 |
| 7 | 0.54493468 | -0.53357879 | 0.02716343 |
| 8 | -1.91927549 | -2.10057894 | -0.56816236 |
| 8 | -1.97123618 | 0.13466648 | 1.56540041 |
| 6 | 1.29318852 | 0.10059678 | -1.09173309 |
| 6 | -1.07064295 | -2.83344955 | -1.33577956 |
| 6 | -1.57942144 | -3.66934623 | -2.37141691 |
| 6 | -0.64696694 | -4.40491653 | -3.11774733 |
| 6 | 0.72985790 | -4.34412661 | -2.88367162 |
| 6 | 1.21218572 | -3.53596818 | -1.85910619 |
| 6 | 0.32437668 | -2.79210267 | -1.07471381 |
| 6 | 0.83931210 | -2.01028529 | 0.10486216 |
| 1 | 1.41456585 | -4.92957337 | -3.49752051 |
| 6 | -3.08978143 | -3.76231485 | -2.66426320 |
| 6 | -1.24543006 | -0.31222203 | 2.62238378 |
| 6 | -1.90647686 | -0.69745675 | 3.82503450 |
| 6 | -1.10449292 | -1.14266193 | 4.88552905 |
| 6 | 0.28843435 | -1.22012743 | 4.80102611 |
| 6 | 0.92119546 | -0.82932163 | 3.62622420 |
| 6 | 0.17142213 | -0.36249748 | 2.54005794 |
| 6 | 0.89288276 | 0.16381804 | 1.32444084 |
| 1 | 0.86868567 | -1.58173601 | 5.64995601 |
| 6 | -3.44085734 | -0.63118433 | 3.95934398 |
| 6 | -3.39595089 | -4.75829467 | -3.79877025 |
| 6 | -3.63102515 | -2.38187867 | -3.10573719 |
| 6 | -3.84647774 | -4.25154141 | -1.40638624 |
| 6 | -3.91516607 | -1.06418783 | 5.35961026 |
| 6 | -4.10720023 | -1.58050634 | 2.93559292 |
| 6 | -3.93257771 | 0.81900386 | 3.73684032 |
| 1 | 2.37609642 | 0.00513429 | -0.91661136 |
| 1 | 1.02948214 | -0.39818540 | -2.03079198 |
| 1 | 1.92987995 | -2.13597887 | 0.20143250 |
| 1 | 0.36897627 | -2.36127417 | 1.03521246 |
| 1 | -1.00347082 | -5.04800954 | -3.92048337 |
| 1 | 2.28260044 | -3.48408358 | -1.64767874 |
| 1 | 0.66312665 | 1.22806948 | 1.15878819 |
| 1 | 1.98131611 | 0.07299743 | 1.47131916 |
| 1 | -1.58091126 | -1.44960267 | 5.81483873 |
| 1 | 2.00969533 | -0.86931955 | 3.54356623 |
| 1 | -4.48322442 | -4.79504291 | -3.95728958 |
| 1 | -2.93387113 | -4.45882050 | -4.75070946 |
| 1 | -3.06243510 | -5.77747075 | -3.55503036 |
| 1 | -3.48478142 | -1.62148728 | -2.33118531 |
| 1 | -3.12569202 | -2.04222149 | -4.02142618 |
| 1 | -4.70823450 | -2.45614824 | -3.32007658 |
| 1 | -3.49777821 | -5.25190927 | -1.11066520 |
| 1 | -3.70492244 | -3.57004824 | -0.56078225 |
| 1 | -4.92331686 | -4.32035757 | -1.62418610 |
| 1 | -5.01082502 | -0.98639876 | 5.40463487 |
| 1 | -3.50487660 | -0.42195248 | 6.15240011 |
| 1 | -3.64943483 | -2.10746958 | 5.58351309 |
| 1 | -3.85757590 | -1.30634544 | 1.90530028 |

| | | | |
|---|---|---|---|
| 1 | -3.78686993 | -2.61881952 | 3.10368237 |
| 1 | -5.20134491 | -1.54054725 | 3.04885099 |
| 1 | -3.50011193 | 1.49355759 | 4.49032592 |
| 1 | -3.65967397 | 1.18777920 | 2.74227469 |
| 1 | -5.02804671 | 0.85904529 | 3.83688149 |
| 1 | 1.00827588 | 1.15671815 | -1.15222675 |
| 31 | -1.46968819 | -0.33719591 | -0.16120978 |
| 8 | 7.19436970 | -0.67765935 | -0.60069657 |
| 6 | 7.02523627 | -1.95189643 | -1.27156280 |
| 1 | 6.47142748 | -1.81929414 | -2.20900393 |
| 6 | 6.31292019 | 1.23557307 | 0.49610147 |
| 6 | 5.17326329 | 2.25204809 | 0.37916767 |
| 6 | 5.13382504 | 2.95577420 | -0.98733708 |
| 6 | 6.03645979 | -0.04839273 | -0.26643582 |
| 6 | 3.80025350 | 3.65565076 | -1.29327845 |
| 6 | 3.42313294 | 4.71987063 | -0.26848708 |
| 1 | 7.27441846 | 1.65238649 | 0.16298971 |
| 1 | 4.22016986 | 1.73083420 | 0.55445046 |
| 1 | 5.30888981 | 2.21401817 | -1.78288509 |
| 1 | 3.84658443 | 4.12201008 | -2.28933349 |
| 1 | 6.45617803 | 0.93936352 | 1.54910261 |
| 1 | 5.28157822 | 2.99288851 | 1.18502694 |
| 1 | 5.96580282 | 3.67773432 | -1.05236772 |
| 1 | 2.99981845 | 2.90204681 | -1.32359618 |
| 1 | 3.32955005 | 4.29377203 | 0.73853633 |
| 1 | 4.15302064 | 5.54091393 | -0.25506017 |
| 8 | 4.93111795 | -0.49389745 | -0.52666462 |
| 8 | 2.16106726 | 5.36565864 | -0.61288818 |
| 1 | 8.03979178 | -2.31118027 | -1.46685306 |
| 1 | 6.48128554 | -2.65381544 | -0.62735895 |

Electronic energy = -3908.684551 a.u.
DFT-D3(BJ) dispersion correction = -0.133507 a.u.
Thermal free energy = 0.734592 a.u.
Gibbs free energy = -3908.083466 a.u.
Number of imaginary frequencies = 0.

**Int4 CL isomer/conformer 2**

| Atomic N. | X | Y | Z |
|---|---|---|---|
| 6 | -4.47515011 | -2.00942045 | 2.49713100 |
| 6 | -4.15192753 | -3.05084941 | 1.40394160 |
| 6 | -3.18213899 | -2.57013257 | 0.31299378 |
| 6 | -3.39954600 | -1.74193872 | 3.53216674 |
| 6 | -1.70566935 | -2.51987299 | 0.72059936 |
| 6 | -0.80799256 | -1.94583762 | -0.37767164 |
| 1 | -4.72314446 | -1.04209396 | 2.04081903 |
| 1 | -5.10796563 | -3.31383312 | 0.92496020 |
| 1 | -3.27668851 | -3.24161414 | -0.55726234 |
| 1 | -1.35436321 | -3.53865058 | 0.96370346 |
| 1 | -5.36042297 | -2.35322980 | 3.05627961 |
| 1 | -3.77967023 | -3.97450553 | 1.87447357 |
| 1 | -3.48830860 | -1.57138186 | -0.03539213 |
| 1 | -1.57263142 | -1.90598475 | 1.62491240 |

| | | | |
|---|---|---|---|
| 1 | 0.25062091 | -2.02537596 | -0.07004951 |
| 1 | -0.92304795 | -2.54714448 | -1.30022557 |
| 8 | -2.90407762 | -0.65318443 | 3.76853819 |
| 8 | -1.18322883 | -0.59388767 | -0.65562704 |
| 7 | 0.44801108 | 1.80000270 | 0.82145117 |
| 8 | -0.57507252 | 2.08921387 | -1.97072432 |
| 8 | 1.74512859 | 0.11455453 | -1.31132222 |
| 6 | -0.51904974 | 1.53851059 | 1.92021354 |
| 6 | -1.29776028 | 3.10405668 | -1.42649338 |
| 6 | -2.45335042 | 3.59997213 | -2.09535869 |
| 6 | -3.14495675 | 4.65455788 | -1.48109164 |
| 6 | -2.74575250 | 5.21863940 | -0.26607033 |
| 6 | -1.60574496 | 4.73533150 | 0.36763932 |
| 6 | -0.87113711 | 3.69334460 | -0.20803136 |
| 6 | 0.42548862 | 3.25225259 | 0.41651193 |
| 1 | -3.32207820 | 6.03228984 | 0.17429039 |
| 6 | -2.93022107 | 3.00344380 | -3.43408701 |
| 6 | 2.83069542 | 0.88154176 | -1.02754587 |
| 6 | 3.90242640 | 0.97207716 | -1.96286812 |
| 6 | 5.00596709 | 1.75837937 | -1.60041799 |
| 6 | 5.08086915 | 2.44352442 | -0.38469194 |
| 6 | 4.02831548 | 2.34432662 | 0.51877211 |
| 6 | 2.90835958 | 1.56114537 | 0.21621677 |
| 6 | 1.83316998 | 1.38018505 | 1.25814459 |
| 1 | 5.95640740 | 3.04942545 | -0.15151621 |
| 6 | 3.85313716 | 0.24804934 | -3.32356639 |
| 6 | -4.16942927 | 3.73729775 | -3.98096596 |
| 6 | -3.32084640 | 1.51864197 | -3.24393337 |
| 6 | -1.81620994 | 3.12772919 | -4.50076399 |
| 6 | 5.13457564 | 0.48160728 | -4.14624607 |
| 6 | 2.66644843 | 0.77609423 | -4.16395023 |
| 6 | 3.71523160 | -1.27873901 | -3.11476837 |
| 1 | -0.23804164 | 2.12363349 | 2.81029150 |
| 1 | -1.52696823 | 1.82414919 | 1.60263734 |
| 1 | 0.64536860 | 3.86074244 | 1.30986408 |
| 1 | 1.25687217 | 3.37886849 | -0.29253527 |
| 1 | -4.03642058 | 5.05185005 | -1.96312875 |
| 1 | -1.26500392 | 5.17435084 | 1.30807774 |
| 1 | 1.75334248 | 0.32257524 | 1.55296905 |
| 1 | 2.09249749 | 1.95920168 | 2.16013058 |
| 1 | 5.83877354 | 1.85164801 | -2.29500611 |
| 1 | 4.07120285 | 2.86486128 | 1.47804084 |
| 1 | -4.45851666 | 3.28289403 | -4.93935303 |
| 1 | -5.03257219 | 3.65637371 | -3.30437096 |
| 1 | -3.97066679 | 4.80291872 | -4.16626201 |
| 1 | -2.47989588 | 0.91403062 | -2.88787075 |
| 1 | -4.14236781 | 1.42538061 | -2.51866082 |
| 1 | -3.66606339 | 1.10034480 | -4.20192828 |
| 1 | -1.56070241 | 4.18384998 | -4.67153720 |
| 1 | -0.90820662 | 2.59436659 | -4.20009303 |
| 1 | -2.16872860 | 2.70746210 | -5.45508879 |
| 1 | 5.05145337 | -0.06247510 | -5.09780806 |
| 1 | 6.03197063 | 0.11038480 | -3.63015881 |

| | | | |
|---|---|---|---|
| 1 | 5.28512643 | 1.54396348 | -4.38619425 |
| 1 | 1.70561193 | 0.59562884 | -3.67088087 |
| 1 | 2.76669872 | 1.85659923 | -4.34027173 |
| 1 | 2.65110164 | 0.27282038 | -5.14262517 |
| 1 | 4.58326943 | -1.67279806 | -2.56517015 |
| 1 | 2.80440710 | -1.52814908 | -2.55947120 |
| 1 | 3.67807868 | -1.78578714 | -4.09090975 |
| 1 | -0.51376664 | 0.47267337 | 2.17077711 |
| 31 | 0.04177317 | 0.72204298 | -0.87516558 |
| 8 | 3.36462172 | -3.57729427 | 1.56834066 |
| 6 | 4.21768061 | -2.62404535 | 0.87998570 |
| 1 | 3.62809060 | -2.03489615 | 0.16743663 |
| 6 | 1.47387403 | -4.09272669 | 2.90433822 |
| 6 | 0.55793142 | -3.55519200 | 4.00442385 |
| 6 | -0.54940961 | -4.54189359 | 4.38860435 |
| 6 | 2.36508120 | -3.02382892 | 2.30056990 |
| 6 | -1.43409514 | -4.06149304 | 5.54966112 |
| 6 | -2.13634037 | -2.73221994 | 5.31424407 |
| 1 | 0.87377883 | -4.50315553 | 2.07328953 |
| 1 | 0.12229209 | -2.60951772 | 3.65379253 |
| 1 | -1.18254737 | -4.73849497 | 3.50831837 |
| 1 | -0.81897127 | -3.93923257 | 6.45753773 |
| 1 | 2.10157670 | -4.92531319 | 3.25476438 |
| 1 | 1.16190791 | -3.30267879 | 4.89164586 |
| 1 | -0.10434540 | -5.51048559 | 4.66993688 |
| 1 | -2.18604862 | -4.83112113 | 5.78374297 |
| 1 | -1.43795263 | -1.92154614 | 5.07266076 |
| 1 | -2.71010756 | -2.42966613 | 6.20337518 |
| 8 | 2.20489428 | -1.82005331 | 2.42074032 |
| 8 | -3.07921177 | -2.88340529 | 4.21267882 |
| 1 | 4.96162982 | -3.23205763 | 0.35732059 |
| 1 | 4.69877426 | -1.95260568 | 1.60117688 |

Electronic energy = -3908.682229 a.u.
DFT-D3(BJ) dispersion correction = -0.136546 a.u.
Thermal free energy = 0.735552 a.u.
Gibbs free energy = -3908.083223 a.u.
Number of imaginary frequencies = 0.

**Int4 CL isomer/conformer 3**

| Atomic N. | X | Y | Z |
|---|---|---|---|
| 6 | -0.63264231 | 4.32707269 | 0.24623412 |
| 6 | -1.84900232 | 3.42923736 | -0.00251134 |
| 6 | -1.95213551 | 2.93910486 | -1.45181496 |
| 6 | 0.69424481 | 3.59529384 | 0.17867926 |
| 6 | -3.23527996 | 2.15471400 | -1.76179931 |
| 6 | -3.40734326 | 0.86574517 | -0.95787787 |
| 1 | -0.68472929 | 4.76605065 | 1.25751564 |
| 1 | -2.75497633 | 3.99976091 | 0.25979958 |
| 1 | -1.89897651 | 3.80779809 | -2.12997846 |
| 1 | -3.24912426 | 1.89757307 | -2.83290301 |
| 1 | -0.60369249 | 5.17495623 | -0.45349215 |
| 1 | -1.80320597 | 2.57339982 | 0.68642176 |

| | | | |
|---|---|---|---|
| 1 | -1.08590908 | 2.30196090 | -1.68636121 |
| 1 | -4.11945072 | 2.78947937 | -1.57477673 |
| 1 | -4.36433777 | 0.39229999 | -1.24814241 |
| 1 | -3.47666275 | 1.09875048 | 0.12098117 |
| 8 | 0.86938791 | 2.41068477 | 0.41706100 |
| 8 | -2.31567479 | -0.02197693 | -1.22758573 |
| 7 | 0.08789463 | -1.56529663 | 0.14982350 |
| 8 | -2.37958403 | -3.08292051 | -0.55833575 |
| 8 | -2.43781690 | -0.96962259 | 1.69926311 |
| 6 | 0.85070063 | -0.87025439 | -0.91991399 |
| 6 | -1.52839289 | -3.77119424 | -1.36340836 |
| 6 | -2.03062926 | -4.53865109 | -2.45346561 |
| 6 | -1.09375097 | -5.23186965 | -3.23405634 |
| 6 | 0.28074246 | -5.19484114 | -2.98196879 |
| 6 | 0.75619806 | -4.45460680 | -1.90436860 |
| 6 | -0.13609185 | -3.75373735 | -1.08623647 |
| 6 | 0.36512409 | -3.04709752 | 0.14487324 |
| 1 | 0.96873618 | -5.74697212 | -3.62224059 |
| 6 | -3.53832067 | -4.60637021 | -2.76581229 |
| 6 | -1.72778097 | -1.48780723 | 2.73477684 |
| 6 | -2.40475763 | -1.94437264 | 3.90288270 |
| 6 | -1.61676907 | -2.45914966 | 4.94220697 |
| 6 | -0.22325769 | -2.53790695 | 4.86895745 |
| 6 | 0.42507060 | -2.07833623 | 3.72814834 |
| 6 | -0.31062256 | -1.54142648 | 2.66479885 |
| 6 | 0.42500988 | -0.94760396 | 1.49007676 |
| 1 | 0.34551645 | -2.95403114 | 5.70043687 |
| 6 | -3.94027417 | -1.87953991 | 4.02392564 |
| 6 | -3.83692282 | -5.52746725 | -3.96379687 |
| 6 | -4.07046361 | -3.19866119 | -3.12447750 |
| 6 | -4.30840162 | -5.17051109 | -1.54804316 |
| 6 | -4.43228610 | -2.39943641 | 5.38803886 |
| 6 | -4.59878019 | -2.75783228 | 2.93389560 |
| 6 | -4.42328863 | -0.41562135 | 3.88984096 |
| 1 | 1.93135255 | -0.99572097 | -0.74379688 |
| 1 | 0.58930849 | -1.30024265 | -1.89281472 |
| 1 | 1.45260150 | -3.19366891 | 0.25524320 |
| 1 | -0.12436573 | -3.44619419 | 1.04540425 |
| 1 | -1.44494315 | -5.82215152 | -4.07855621 |
| 1 | 1.82437505 | -4.42870860 | -1.67672351 |
| 1 | 0.20605667 | 0.12585730 | 1.38112932 |
| 1 | 1.51185969 | -1.05115735 | 1.64375500 |
| 1 | -2.10515207 | -2.82148725 | 5.84493146 |
| 1 | 1.51437113 | -2.11772099 | 3.65649469 |
| 1 | -4.92263844 | -5.54917089 | -4.13487985 |
| 1 | -3.36429949 | -5.17020646 | -4.89024701 |
| 1 | -3.50995648 | -6.56151885 | -3.78181694 |
| 1 | -3.93036410 | -2.48929137 | -2.30186967 |
| 1 | -3.55496626 | -2.80342062 | -4.01171504 |
| 1 | -5.14556911 | -3.25535572 | -3.35382286 |
| 1 | -3.96619863 | -6.18893259 | -1.31259677 |
| 1 | -4.17275163 | -4.54415990 | -0.65986826 |
| 1 | -5.38328265 | -5.22106367 | -1.77968891 |

| | | | |
|---|---|---|---|
| 1 | -5.52805710 | -2.31986456 | 5.42551200 |
| 1 | -4.02903698 | -1.81132138 | 6.22515413 |
| 1 | -4.17316793 | -3.45612424 | 5.54737386 |
| 1 | -4.33856006 | -2.41817432 | 1.92597898 |
| 1 | -4.28334011 | -3.80604089 | 3.03708020 |
| 1 | -5.69381245 | -2.72146180 | 3.03889102 |
| 1 | -3.99651829 | 0.20699255 | 4.68984980 |
| 1 | -4.13825095 | 0.01498327 | 2.92387990 |
| 1 | -5.51954538 | -0.37666481 | 3.98053684 |
| 1 | 0.59900835 | 0.19556273 | -0.90524941 |
| 31 | -1.92889805 | -1.34744027 | -0.04807073 |
| 8 | 10.17361835 | 5.56729974 | -1.42159478 |
| 6 | 11.21058532 | 6.51676182 | -1.76939943 |
| 1 | 11.23673289 | 7.33913456 | -1.04354341 |
| 6 | 7.90266783 | 5.01866826 | -1.02420906 |
| 6 | 6.46701516 | 5.53915506 | -0.96902069 |
| 6 | 5.46167047 | 4.44333198 | -0.59980873 |
| 6 | 8.91241961 | 6.08831032 | -1.39580701 |
| 6 | 4.01979694 | 4.96208646 | -0.54225968 |
| 6 | 3.03741504 | 3.86176205 | -0.17755689 |
| 1 | 8.20847462 | 4.58917337 | -0.05623556 |
| 1 | 6.40650151 | 6.36279836 | -0.24137783 |
| 1 | 5.73533962 | 4.00708019 | 0.37652330 |
| 1 | 3.94430974 | 5.77465125 | 0.19748020 |
| 1 | 8.00057721 | 4.19759082 | -1.75319043 |
| 1 | 6.20159803 | 5.97746964 | -1.94310783 |
| 1 | 5.52817663 | 3.62184987 | -1.33415883 |
| 1 | 3.73773347 | 5.39068309 | -1.51688578 |
| 1 | 3.04896288 | 3.04289424 | -0.91242495 |
| 1 | 3.25249298 | 3.42968969 | 0.81119205 |
| 8 | 8.66112949 | 7.25255957 | -1.64190898 |
| 8 | 1.70283293 | 4.44609007 | -0.15263670 |
| 1 | 12.14440097 | 5.94710622 | -1.74423438 |
| 1 | 11.03299922 | 6.92895505 | -2.77060320 |

Electronic energy = -3908.684779 a.u.
DFT-D3(BJ) dispersion correction = -0.129209 a.u.
Thermal free energy = 0.734106 a.u.
Gibbs free energy = -3908.079882 a.u.
Number of imaginary frequencies = 0.

**Int4 CL isomer/conformer 4**

| Atomic N. | X | Y | Z |
|---|---|---|---|
| 7 | 0.18559531 | 2.21072023 | 1.08429432 |
| 8 | -0.04787396 | 2.85176160 | -1.82686484 |
| 8 | 2.51369026 | 1.56000952 | -0.73352334 |
| 6 | -0.87913844 | 1.42238950 | 1.76093013 |
| 6 | -1.20836828 | 3.44157425 | -1.43553122 |
| 6 | -2.23141346 | 3.72044164 | -2.38680156 |
| 6 | -3.39738432 | 4.33871077 | -1.91138410 |
| 6 | -3.58088063 | 4.68290620 | -0.56911674 |
| 6 | -2.56585815 | 4.42272410 | 0.34595567 |
| 6 | -1.37751474 | 3.81803898 | -0.07672608 |

| | | | |
|---|---|---|---|
| 6 | -0.23534983 | 3.64551249 | 0.88951742 |
| 1 | -4.50858221 | 5.15686720 | -0.24841706 |
| 6 | -2.06752653 | 3.36270122 | -3.87702470 |
| 6 | 3.14072347 | 2.51085339 | 0.00708564 |
| 6 | 4.32048719 | 3.13593933 | -0.49195635 |
| 6 | 4.93284439 | 4.09782415 | 0.32412782 |
| 6 | 4.43283482 | 4.45584499 | 1.57908573 |
| 6 | 3.28651668 | 3.83163102 | 2.05778510 |
| 6 | 2.64157874 | 2.85278316 | 1.29207178 |
| 6 | 1.46591307 | 2.11733461 | 1.88607103 |
| 1 | 4.93981998 | 5.21577052 | 2.17360123 |
| 6 | 4.90363877 | 2.77605527 | -1.87310258 |
| 6 | -3.28848633 | 3.79559642 | -4.71045154 |
| 6 | -1.91589161 | 1.83236561 | -4.04463716 |
| 6 | -0.83030154 | 4.08455820 | -4.46233432 |
| 6 | 6.19380545 | 3.56181186 | -2.17530524 |
| 6 | 3.88662147 | 3.11926326 | -2.98710009 |
| 6 | 5.26062861 | 1.27129743 | -1.92526238 |
| 1 | -1.06950778 | 1.84496555 | 2.76078239 |
| 1 | -1.79768729 | 1.46767376 | 1.16632593 |
| 1 | -0.50138059 | 4.06196017 | 1.87528439 |
| 1 | 0.65858616 | 4.17406095 | 0.52707660 |
| 1 | -4.20094787 | 4.55852640 | -2.61202524 |
| 1 | -2.67922360 | 4.70406766 | 1.39543922 |
| 1 | 1.67156118 | 1.03932274 | 1.98116134 |
| 1 | 1.25947656 | 2.50764215 | 2.89631925 |
| 1 | 5.83245520 | 4.59650055 | -0.03177854 |
| 1 | 2.88644477 | 4.08789917 | 3.04138420 |
| 1 | -3.11441997 | 3.52982657 | -5.76287357 |
| 1 | -4.21047075 | 3.28885005 | -4.39027604 |
| 1 | -3.45461825 | 4.88170025 | -4.66668992 |
| 1 | -1.03467155 | 1.45353979 | -3.51616425 |
| 1 | -2.80258132 | 1.30957251 | -3.65769761 |
| 1 | -1.81334748 | 1.58359829 | -5.11195750 |
| 1 | -0.94860962 | 5.17546617 | -4.38804758 |
| 1 | 0.08819433 | 3.79815202 | -3.93881402 |
| 1 | -0.72035905 | 3.82894895 | -5.52733399 |
| 1 | 6.57147383 | 3.26060001 | -3.16277211 |
| 1 | 6.98596792 | 3.35547581 | -1.44102369 |
| 1 | 6.02082190 | 4.64725470 | -2.20590574 |
| 1 | 2.95159616 | 2.56148393 | -2.87043218 |
| 1 | 3.65039109 | 4.19303171 | -2.97911626 |
| 1 | 4.31572709 | 2.87395395 | -3.97054509 |
| 1 | 6.01582916 | 1.02650881 | -1.16390888 |
| 1 | 4.37945440 | 0.64308759 | -1.75647356 |
| 1 | 5.68314028 | 1.02415320 | -2.91116890 |
| 1 | -0.55037060 | 0.38180304 | 1.85676790 |
| 6 | 2.84711800 | -2.76959536 | 1.84940209 |
| 6 | 2.99705809 | -3.12738514 | 0.35304808 |
| 6 | 2.44313367 | -2.06319604 | -0.61611975 |
| 6 | 1.42145238 | -2.53557407 | 2.30219116 |
| 6 | 1.01593765 | -2.33902478 | -1.11140922 |
| 6 | 0.37380820 | -1.15518521 | -1.83905809 |

| | | | |
|---|---|---|---|
| 1 | 3.40354016 | -1.84899360 | 2.06797287 |
| 1 | 4.07365746 | -3.26947979 | 0.17514563 |
| 1 | 3.10330005 | -1.99535338 | -1.49550064 |
| 1 | 1.02861284 | -3.20953459 | -1.79048252 |
| 1 | 3.26974065 | -3.58393979 | 2.45725329 |
| 1 | 2.52130451 | -4.10161471 | 0.16115344 |
| 1 | 2.48321723 | -1.07316524 | -0.13563368 |
| 1 | 0.35166745 | -2.60754285 | -0.27443301 |
| 1 | -0.51203584 | -1.50459507 | -2.39820537 |
| 1 | 1.08466270 | -0.74865303 | -2.58392926 |
| 8 | 0.94519305 | -1.45305720 | 2.60871756 |
| 8 | -0.06783085 | -0.15985016 | -0.91071278 |
| 31 | 0.65490181 | 1.49330324 | -0.75836763 |
| 8 | -6.86448272 | -7.58075586 | 3.77948081 |
| 6 | -7.57876771 | -8.84052242 | 3.77021640 |
| 1 | -7.58552082 | -9.27025279 | 2.76065881 |
| 6 | -4.90121423 | -6.29929775 | 3.43317668 |
| 6 | -3.42505514 | -6.32819930 | 3.03923805 |
| 6 | -2.78369839 | -4.93694696 | 3.07436802 |
| 6 | -5.55704440 | -7.66671083 | 3.39667504 |
| 6 | -1.30209852 | -4.96170735 | 2.67980178 |
| 6 | -0.68333959 | -3.57466182 | 2.72229166 |
| 1 | -5.47665013 | -5.63442275 | 2.76829027 |
| 1 | -3.32632486 | -6.76021422 | 2.03177897 |
| 1 | -3.33318425 | -4.26323100 | 2.39425455 |
| 1 | -1.19316659 | -5.37545149 | 1.66481469 |
| 1 | -5.03587766 | -5.88784619 | 4.44676098 |
| 1 | -2.88163717 | -7.00787674 | 3.71333087 |
| 1 | -2.88849117 | -4.50935449 | 4.08643524 |
| 1 | -0.74567707 | -5.62801834 | 3.35787933 |
| 1 | -0.72987767 | -3.13461106 | 3.72963406 |
| 1 | -1.18199412 | -2.88135064 | 2.02830470 |
| 8 | -5.01877538 | -8.70871778 | 3.07454159 |
| 8 | 0.71474865 | -3.69783228 | 2.33159627 |
| 1 | -8.59504708 | -8.59912002 | 4.09593536 |
| 1 | -7.10831564 | -9.55337443 | 4.45898059 |

Electronic energy = -3908.679640 a.u.
DFT-D3(BJ) dispersion correction = -0.131411 a.u.
Thermal free energy = 0.732056 a.u.
Gibbs free energy = -3908.078994 a.u.
Number of imaginary frequencies = 0.

**Int4 CL isomer/conformer 5**

| Atomic N. | X | Y | Z |
|---|---|---|---|
| 6 | -0.25049711 | 5.00569698 | 0.54615151 |
| 6 | -1.49057479 | 4.11950859 | 0.39365576 |
| 6 | -1.69047529 | 3.59632095 | -1.03391522 |
| 6 | 1.06375573 | 4.25771823 | 0.42843421 |
| 6 | -3.00686257 | 2.83458920 | -1.24604402 |
| 6 | -3.16106938 | 1.57208747 | -0.39783943 |
| 1 | -0.23969980 | 5.47777653 | 1.54375088 |
| 1 | -2.37117067 | 4.70979914 | 0.69584672 |

| | | | |
|---|---|---|---|
| 1 | -1.66128750 | 4.44708398 | -1.73587815 |
| 1 | -3.09031621 | 2.54965579 | -2.30687902 |
| 1 | -0.25002465 | 5.83168495 | -0.18007927 |
| 1 | -1.41496539 | 3.28005091 | 1.09992603 |
| 1 | -0.85311997 | 2.93540313 | -1.30420213 |
| 1 | -3.86347754 | 3.49572804 | -1.02509683 |
| 1 | -4.14531640 | 1.11629507 | -0.61630355 |
| 1 | -3.15835657 | 1.83508801 | 0.67636322 |
| 8 | 1.23112248 | 3.07105994 | 0.66119613 |
| 8 | -2.11059006 | 0.64894675 | -0.70883152 |
| 7 | 0.35056865 | -0.90059822 | 0.54383754 |
| 8 | -2.19153174 | -2.38710884 | 0.07511311 |
| 8 | -2.04238302 | -0.19581273 | 2.25166502 |
| 6 | 1.04532008 | -0.25646704 | -0.60128080 |
| 6 | -1.41711161 | -3.11877039 | -0.76870443 |
| 6 | -2.01495411 | -3.90898392 | -1.79212945 |
| 6 | -1.15312159 | -4.64640194 | -2.61745234 |
| 6 | 0.23694345 | -4.63112758 | -2.47038511 |
| 6 | 0.80658499 | -3.86761197 | -1.45665454 |
| 6 | -0.00782413 | -3.12226786 | -0.59760858 |
| 6 | 0.59863881 | -2.38743535 | 0.56792727 |
| 1 | 0.86388336 | -5.21740546 | -3.14217546 |
| 6 | -3.54295939 | -3.95385179 | -1.98841824 |
| 6 | -1.26929988 | -0.69543486 | 3.25049434 |
| 6 | -1.86769955 | -1.09468142 | 4.48084685 |
| 6 | -1.01642316 | -1.59310370 | 5.47739590 |
| 6 | 0.36571949 | -1.70883077 | 5.30506585 |
| 6 | 0.93765986 | -1.30424319 | 4.10416289 |
| 6 | 0.13736979 | -0.78545855 | 3.07921428 |
| 6 | 0.79718966 | -0.24794080 | 1.83440441 |
| 1 | 0.98519196 | -2.11062435 | 6.10683352 |
| 6 | -3.38814726 | -0.98717855 | 4.71182854 |
| 6 | -3.94864769 | -4.90616963 | -3.12914581 |
| 6 | -4.07345060 | -2.54717260 | -2.35340360 |
| 6 | -4.22984470 | -4.46195146 | -0.69837332 |
| 6 | -3.78871966 | -1.44482942 | 6.12720495 |
| 6 | -4.14483304 | -1.88792115 | 3.70722068 |
| 6 | -3.84725269 | 0.48248236 | 4.55808785 |
| 1 | 2.13383474 | -0.39523468 | -0.49827436 |
| 1 | 0.70894462 | -0.71408992 | -1.53781282 |
| 1 | 1.68846810 | -2.55325688 | 0.60054350 |
| 1 | 0.17180796 | -2.74687229 | 1.51579542 |
| 1 | -1.57823172 | -5.25504284 | -3.41354706 |
| 1 | 1.88921018 | -3.85687479 | -1.31081472 |
| 1 | 0.59314681 | 0.82627580 | 1.70542487 |
| 1 | 1.89004792 | -0.37168573 | 1.91279719 |
| 1 | -1.44446015 | -1.91207370 | 6.42595525 |
| 1 | 2.01755320 | -1.37270898 | 3.95443177 |
| 1 | -5.04441445 | -4.91030945 | -3.21741972 |
| 1 | -3.54088599 | -4.58884575 | -4.09987667 |
| 1 | -3.62833220 | -5.94058134 | -2.93765067 |
| 1 | -3.85824407 | -1.81496894 | -1.56784085 |
| 1 | -3.61875504 | -2.19182361 | -3.28938713 |

| | | | |
|---|---|---|---|
| 1 | -5.16366244 | -2.58794989 | -2.49984124 |
| 1 | -3.89031217 | -5.47937125 | -0.45489483 |
| 1 | -4.01607616 | -3.81039893 | 0.15565008 |
| 1 | -5.31985549 | -4.49685208 | -0.84761816 |
| 1 | -4.87659455 | -1.33638308 | 6.24222697 |
| 1 | -3.31104496 | -0.83715380 | 6.90939933 |
| 1 | -3.54222791 | -2.50124577 | 6.30700724 |
| 1 | -3.94828484 | -1.59533161 | 2.67050290 |
| 1 | -3.84930228 | -2.93976075 | 3.83057783 |
| 1 | -5.22835629 | -1.81689770 | 3.88707453 |
| 1 | -3.34857102 | 1.12281716 | 5.30044888 |
| 1 | -3.62522567 | 0.87062700 | 3.55822296 |
| 1 | -4.93261759 | 0.55194202 | 4.72735745 |
| 1 | 0.81115506 | 0.81346290 | -0.60690295 |
| 31 | -1.67127167 | -0.64519449 | 0.48659160 |
| 8 | 8.52309547 | 1.48195047 | -4.48043776 |
| 6 | 8.92574147 | 0.91260993 | -5.74998290 |
| 1 | 8.77983589 | 1.63884689 | -6.55934786 |
| 6 | 6.88942468 | 2.43271025 | -3.05083415 |
| 6 | 5.45066568 | 2.93409349 | -2.93295396 |
| 6 | 5.13561066 | 3.48399562 | -1.53778162 |
| 6 | 7.21294084 | 1.85922719 | -4.41775193 |
| 6 | 3.69522253 | 3.99582395 | -1.41553158 |
| 6 | 3.40380559 | 4.53103511 | -0.01897677 |
| 1 | 7.61217855 | 3.23776027 | -2.84147711 |
| 1 | 5.27341602 | 3.71298398 | -3.69035389 |
| 1 | 5.83773859 | 4.30132084 | -1.29729632 |
| 1 | 3.51877202 | 4.79590825 | -2.15173035 |
| 1 | 7.09878466 | 1.64961332 | -2.30334006 |
| 1 | 4.75875810 | 2.11388685 | -3.17721688 |
| 1 | 5.31048277 | 2.69593645 | -0.78484162 |
| 1 | 2.98937461 | 3.18255778 | -1.64439246 |
| 1 | 3.49962036 | 3.74041636 | 0.73757238 |
| 1 | 4.07289653 | 5.36480270 | 0.23301743 |
| 8 | 6.43350527 | 1.73778485 | -5.34340365 |
| 8 | 2.06652431 | 5.10564070 | 0.06606170 |
| 1 | 9.98595802 | 0.66840926 | -5.63403889 |
| 1 | 8.34026488 | 0.01087633 | -5.96891501 |

Electronic energy = -3908.684279 a.u.
DFT-D3(BJ) dispersion correction = -0.130151 a.u.
Thermal free energy = 0.735812 a.u.
Gibbs free energy = -3908.078618 a.u.
Number of imaginary frequencies = 0.

**Int4 CL isomer/conformer 6**

| Atomic N. | X | Y | Z |
|---|---|---|---|
| 6 | -0.00041924 | 4.76442603 | -0.32339222 |
| 6 | -1.29734195 | 3.99956235 | -0.60659128 |
| 6 | -1.45250293 | 3.59470997 | -2.07749422 |
| 6 | 1.25262258 | 3.91755767 | -0.43541948 |
| 6 | -2.81235265 | 2.96796640 | -2.41812925 |
| 6 | -3.12837072 | 1.67392395 | -1.66801627 |

| | | | |
|---|---|---|---|
| 1 | -0.00731104 | 5.15527381 | 0.70884507 |
| 1 | -2.14327663 | 4.64044472 | -0.30862809 |
| 1 | -1.30855044 | 4.48526947 | -2.71272859 |
| 1 | -2.85075277 | 2.75868191 | -3.49901965 |
| 1 | 0.10653455 | 5.64009839 | -0.97992836 |
| 1 | -1.33203026 | 3.10997414 | 0.03884844 |
| 1 | -0.65846948 | 2.88236351 | -2.34800641 |
| 1 | -3.62233086 | 3.68749213 | -2.20381367 |
| 1 | -4.13084136 | 1.32155670 | -1.97638098 |
| 1 | -3.17514389 | 1.86882594 | -0.58044605 |
| 8 | 1.31320731 | 2.71043219 | -0.26249439 |
| 8 | -2.14132897 | 0.68191610 | -1.97544086 |
| 7 | 0.08704197 | -1.15087970 | -0.66258471 |
| 8 | -2.52870553 | -2.37610368 | -1.40363607 |
| 8 | -2.35719404 | -0.33988673 | 0.91910101 |
| 6 | 0.91507771 | -0.51083211 | -1.71790017 |
| 6 | -1.75617695 | -3.12927858 | -2.22963540 |
| 6 | -2.33843312 | -3.80893743 | -3.33805893 |
| 6 | -1.48180928 | -4.57745891 | -4.14003889 |
| 6 | -0.11106679 | -4.69447384 | -3.89206744 |
| 6 | 0.44170711 | -4.03829566 | -2.79708209 |
| 6 | -0.36980937 | -3.26833280 | -1.95719431 |
| 6 | 0.20598007 | -2.65295247 | -0.70979424 |
| 1 | 0.51341700 | -5.29957422 | -4.54933234 |
| 6 | -3.84479852 | -3.70596558 | -3.64724840 |
| 6 | -1.70047904 | -0.95540485 | 1.93629971 |
| 6 | -2.41618247 | -1.36785884 | 3.09771960 |
| 6 | -1.68159652 | -1.98755263 | 4.11876994 |
| 6 | -0.30437263 | -2.20976930 | 4.03373937 |
| 6 | 0.38313396 | -1.79216483 | 2.89978905 |
| 6 | -0.29699203 | -1.15459113 | 1.85522339 |
| 6 | 0.49244148 | -0.60987042 | 0.69164774 |
| 1 | 0.22159434 | -2.70344491 | 4.85081356 |
| 6 | -3.93598929 | -1.14620198 | 3.23110547 |
| 6 | -4.24140225 | -4.55973172 | -4.86647340 |
| 6 | -4.22155448 | -2.24046266 | -3.96948585 |
| 6 | -4.67142970 | -4.21335612 | -2.44163307 |
| 6 | -4.47288188 | -1.64247965 | 4.58704531 |
| 6 | -4.68685884 | -1.92710122 | 2.12673337 |
| 6 | -4.26525949 | 0.36248503 | 3.13209215 |
| 1 | 1.97723153 | -0.75347779 | -1.55179213 |
| 1 | 0.60757453 | -0.88394900 | -2.70073099 |
| 1 | 1.27223291 | -2.91633122 | -0.60956241 |
| 1 | -0.32036216 | -3.02316044 | 0.18228171 |
| 1 | -1.89478345 | -5.10398752 | -4.99861153 |
| 1 | 1.50673018 | -4.13277863 | -2.57282466 |
| 1 | 0.38826658 | 0.48342382 | 0.61353945 |
| 1 | 1.56278277 | -0.83214949 | 0.83549423 |
| 1 | -2.20103088 | -2.31877644 | 5.01609119 |
| 1 | 1.46198300 | -1.94352461 | 2.81949980 |
| 1 | -5.32324455 | -4.46039394 | -5.03458588 |
| 1 | -3.73367066 | -4.23258690 | -5.78544976 |
| 1 | -4.02731232 | -5.62707802 | -4.71088492 |

| | | | |
|---|---|---|---|
| 1 | -4.00476637 | -1.57092167 | -3.13025475 |
| 1 | -3.66688252 | -1.88169374 | -4.84857425 |
| 1 | -5.29661604 | -2.17468784 | -4.19658974 |
| 1 | -4.44113363 | -5.26816346 | -2.23171378 |
| 1 | -4.46935147 | -3.62719885 | -1.53881661 |
| 1 | -5.74540063 | -4.14205639 | -2.67201746 |
| 1 | -5.55421101 | -1.45023675 | 4.63410719 |
| 1 | -4.00642925 | -1.11829912 | 5.43374485 |
| 1 | -4.32443617 | -2.72366596 | 4.72158511 |
| 1 | -4.39505633 | -1.59608309 | 1.12454505 |
| 1 | -4.48333231 | -3.00463455 | 2.20696149 |
| 1 | -5.77164498 | -1.77783656 | 2.23797251 |
| 1 | -3.77206784 | 0.91969215 | 3.94206935 |
| 1 | -3.94229050 | 0.78262139 | 2.17355160 |
| 1 | -5.35107786 | 0.51261019 | 3.23187867 |
| 1 | 0.77643350 | 0.57473134 | -1.67347193 |
| 31 | -1.89599826 | -0.71531027 | -0.84180431 |
| 8 | 9.69288714 | 2.00746905 | 3.02691427 |
| 6 | 10.46157453 | 1.94358152 | 4.25281933 |
| 1 | 9.89745012 | 1.41625729 | 5.03221359 |
| 6 | 7.77762436 | 2.68091699 | 1.80452942 |
| 6 | 6.39387259 | 3.32532685 | 1.87701580 |
| 6 | 5.69057231 | 3.35327145 | 0.51585406 |
| 6 | 8.49812681 | 2.65788359 | 3.13949169 |
| 6 | 4.29646988 | 3.98856312 | 0.58404355 |
| 6 | 3.62298922 | 4.01762367 | -0.78234444 |
| 1 | 7.71804397 | 1.64504620 | 1.43356738 |
| 1 | 5.77530222 | 2.77894331 | 2.60536680 |
| 1 | 5.60542353 | 2.32457533 | 0.12486349 |
| 1 | 3.66422059 | 3.42247307 | 1.28526144 |
| 1 | 8.42471642 | 3.21231526 | 1.08700385 |
| 1 | 6.48965650 | 4.34865092 | 2.27034733 |
| 1 | 6.31350543 | 3.90800565 | -0.20748515 |
| 1 | 4.37253768 | 5.01736024 | 0.97017807 |
| 1 | 4.20786379 | 4.60711672 | -1.50098124 |
| 1 | 3.47482757 | 3.00221098 | -1.17409343 |
| 8 | 8.09365566 | 3.14523498 | 4.17773319 |
| 8 | 2.33256977 | 4.69415907 | -0.72959253 |
| 1 | 11.37257262 | 1.39533789 | 3.99459854 |
| 1 | 10.70265882 | 2.95358178 | 4.60737985 |

Electronic energy = -3908.684333 a.u.
DFT-D3(BJ) dispersion correction = -0.129823 a.u.
Thermal free energy = 0.736020 a.u.
Gibbs free energy = -3908.078136 a.u.
Number of imaginary frequencies = 0.

**Int4 CL isomer/conformer 7**

| Atomic N. | X | Y | Z |
|---|---|---|---|
| 7 | 1.30009596 | 4.47945330 | 0.08543338 |
| 8 | -0.15184318 | 3.22832399 | -2.20293477 |
| 8 | 2.90857221 | 3.61870888 | -2.32407559 |
| 6 | 1.11799390 | 3.94223875 | 1.45823924 |

| | | | |
|---|---|---|---|
| 6 | -1.24961594 | 3.55123472 | -1.46759213 |
| 6 | -2.49483444 | 2.90384882 | -1.71053450 |
| 6 | -3.58594711 | 3.29792965 | -0.92185683 |
| 6 | -3.49211070 | 4.28012720 | 0.06828780 |
| 6 | -2.27389782 | 4.91574837 | 0.28440583 |
| 6 | -1.15616810 | 4.56963898 | -0.48259118 |
| 6 | 0.12959408 | 5.33746360 | -0.32687310 |
| 1 | -4.36887720 | 4.54657156 | 0.65840946 |
| 6 | -2.64290412 | 1.81683115 | -2.79318643 |
| 6 | 3.10484390 | 4.95529293 | -2.46496362 |
| 6 | 3.52830913 | 5.48705551 | -3.71731362 |
| 6 | 3.72335557 | 6.87358196 | -3.79201991 |
| 6 | 3.52075585 | 7.72874815 | -2.70489056 |
| 6 | 3.12178130 | 7.19558501 | -1.48476344 |
| 6 | 2.92406556 | 5.81599747 | -1.35022372 |
| 6 | 2.59970987 | 5.24918173 | 0.00817132 |
| 1 | 3.67887121 | 8.80133232 | -2.81603246 |
| 6 | 3.76261383 | 4.58124497 | -4.94233541 |
| 6 | -4.08884283 | 1.29481847 | -2.89047008 |
| 6 | -1.73891489 | 0.60663164 | -2.45930713 |
| 6 | -2.26676678 | 2.39288473 | -4.17916379 |
| 6 | 4.24804291 | 5.38249639 | -6.16518767 |
| 6 | 2.44424684 | 3.88033696 | -5.34700514 |
| 6 | 4.84877481 | 3.52632504 | -4.62333614 |
| 1 | 1.12387241 | 4.76807942 | 2.18772100 |
| 1 | 0.16111151 | 3.41247533 | 1.51634402 |
| 1 | 0.00929567 | 6.13979890 | 0.41998045 |
| 1 | 0.42148617 | 5.80123864 | -1.28037553 |
| 1 | -4.55022563 | 2.81804116 | -1.07892085 |
| 1 | -2.18381473 | 5.70128490 | 1.03822653 |
| 1 | 3.37843063 | 4.53830034 | 0.32815785 |
| 1 | 2.55811609 | 6.06306741 | 0.75080700 |
| 1 | 4.03997122 | 7.31091237 | -4.73706871 |
| 1 | 2.97305578 | 7.84423018 | -0.61855158 |
| 1 | -4.14065116 | 0.53386334 | -3.68205300 |
| 1 | -4.42369148 | 0.82183987 | -1.95593076 |
| 1 | -4.79937230 | 2.09223697 | -3.15192448 |
| 1 | -0.68175985 | 0.89004981 | -2.41956436 |
| 1 | -2.01633259 | 0.16938021 | -1.48918923 |
| 1 | -1.85966117 | -0.17054017 | -3.22925238 |
| 1 | -2.93223237 | 3.22737144 | -4.44430441 |
| 1 | -1.23259199 | 2.75307373 | -4.19950908 |
| 1 | -2.38071760 | 1.61359465 | -4.94802760 |
| 1 | 4.41073769 | 4.68974901 | -7.00297201 |
| 1 | 5.20057001 | 5.89675265 | -5.97178631 |
| 1 | 3.50919971 | 6.12794545 | -6.49314043 |
| 1 | 2.05823501 | 3.24436081 | -4.54351348 |
| 1 | 1.67263944 | 4.62090270 | -5.60206090 |
| 1 | 2.61570726 | 3.25054933 | -6.23309464 |
| 1 | 5.80327016 | 4.01516666 | -4.37915510 |
| 1 | 4.55805806 | 2.89047681 | -3.78020340 |
| 1 | 5.01342863 | 2.88606749 | -5.50323744 |
| 1 | 1.92752164 | 3.23798523 | 1.68078128 |

| | | | |
|---|---|---|---|
| 6 | 0.35848206 | -3.23168161 | 1.78991591 |
| 6 | 1.65746841 | -2.54431635 | 1.36954561 |
| 6 | 1.42663328 | -1.43011477 | 0.34425204 |
| 6 | 0.55625667 | -4.30179127 | 2.84652798 |
| 6 | 2.72612278 | -0.74893996 | -0.09608207 |
| 6 | 2.51278573 | 0.39518301 | -1.08334691 |
| 1 | -0.15252127 | -3.68901286 | 0.92816913 |
| 1 | 2.15491473 | -2.13589799 | 2.26238672 |
| 1 | 0.74927343 | -0.66968610 | 0.76422157 |
| 1 | 3.25919315 | -0.35483749 | 0.78546819 |
| 1 | -0.35660511 | -2.49703954 | 2.19778701 |
| 1 | 2.34895768 | -3.29696856 | 0.95810927 |
| 1 | 0.91683807 | -1.84258406 | -0.54439237 |
| 1 | 3.39617141 | -1.48861021 | -0.56778133 |
| 1 | 1.96607582 | 0.01488015 | -1.96895291 |
| 1 | 3.49286101 | 0.76762439 | -1.43420887 |
| 8 | 1.58407259 | -4.51628580 | 3.46194355 |
| 8 | 1.76910931 | 1.44314292 | -0.44514586 |
| 31 | 1.47142407 | 3.00702580 | -1.31704953 |
| 8 | -4.42276493 | -11.51950865 | 7.23266675 |
| 6 | -5.63797983 | -12.28859815 | 7.40147478 |
| 1 | -6.44798636 | -11.64958808 | 7.77505864 |
| 6 | -3.20855077 | -9.73644340 | 6.25332114 |
| 6 | -3.21971757 | -8.61232451 | 5.21827288 |
| 6 | -1.87667032 | -7.87811540 | 5.14702182 |
| 6 | -4.52505734 | -10.48334814 | 6.34906131 |
| 6 | -1.86719710 | -6.75283325 | 4.10547052 |
| 6 | -0.51998841 | -6.05173839 | 4.05054589 |
| 1 | -2.97439159 | -9.34653614 | 7.25788981 |
| 1 | -4.02471632 | -7.90185813 | 5.46006048 |
| 1 | -1.63306537 | -7.46195622 | 6.13996883 |
| 1 | -2.65196626 | -6.01679321 | 4.34163240 |
| 1 | -2.41804697 | -10.47231979 | 6.03440049 |
| 1 | -3.47087839 | -9.02898622 | 4.23072520 |
| 1 | -1.07618711 | -8.60103129 | 4.91236891 |
| 1 | -2.10304664 | -7.16261007 | 3.11051196 |
| 1 | 0.29141704 | -6.74545988 | 3.78438912 |
| 1 | -0.25937757 | -5.58931822 | 5.01458368 |
| 8 | -5.54425470 | -10.21850389 | 5.74048383 |
| 8 | -0.59640203 | -5.01000954 | 3.03676042 |
| 1 | -5.38948114 | -13.06594941 | 8.13032625 |
| 1 | -5.94576420 | -12.73414662 | 6.44716551 |

Electronic energy = -3908.682433 a.u.
DFT-D3(BJ) dispersion correction = -0.125645 a.u.
Thermal free energy = 0.732716 a.u.
Gibbs free energy = -3908.075361 a.u.
Number of imaginary frequencies = 0.

**Int4 CL isomer/conformer 8**

| Atomic N. | X | Y | Z |
|---|---|---|---|
| 7 | -1.42364065 | 4.37255287 | 2.90936022 |
| 8 | -1.82920513 | 4.55774852 | -0.04345783 |

| | | | |
|---|---|---|---|
| 8 | 0.98195507 | 4.03918285 | 1.10899389 |
| 6 | -2.25886776 | 3.44365118 | 3.71344283 |
| 6 | -3.09538874 | 4.89846871 | 0.31855573 |
| 6 | -4.16349306 | 4.79713764 | -0.61818722 |
| 6 | -5.44022369 | 5.17544462 | -0.17706926 |
| 6 | -5.69009595 | 5.63836010 | 1.11792864 |
| 6 | -4.63478486 | 5.75014951 | 2.01710078 |
| 6 | -3.33885788 | 5.39709819 | 1.62560956 |
| 6 | -2.18199545 | 5.62928180 | 2.56110111 |
| 1 | -6.70222626 | 5.91401648 | 1.41385609 |
| 6 | -3.93149253 | 4.29647829 | -2.05739165 |
| 6 | 1.36868892 | 5.20608525 | 1.68591679 |
| 6 | 2.36221339 | 6.01595088 | 1.06307461 |
| 6 | 2.72481866 | 7.20240372 | 1.71644755 |
| 6 | 2.15679856 | 7.60615140 | 2.92824016 |
| 6 | 1.19867278 | 6.80044879 | 3.53242191 |
| 6 | 0.80827473 | 5.59814945 | 2.93038833 |
| 6 | -0.14845554 | 4.68897404 | 3.65827112 |
| 1 | 2.46724734 | 8.54185046 | 3.39288264 |
| 6 | 3.01349507 | 5.61037879 | -0.27388787 |
| 6 | -5.22913122 | 4.30967701 | -2.88726006 |
| 6 | -3.41366685 | 2.83896203 | -2.03787345 |
| 6 | -2.91320743 | 5.21094259 | -2.77920754 |
| 6 | 4.07771460 | 6.62780513 | -0.72691974 |
| 6 | 1.94287387 | 5.53926479 | -1.38831685 |
| 6 | 3.71933045 | 4.24124187 | -0.12728225 |
| 1 | -2.48628079 | 3.89642135 | 4.69195090 |
| 1 | -3.19357073 | 3.24201468 | 3.17937883 |
| 1 | -2.53338190 | 6.09204818 | 3.49832294 |
| 1 | -1.44466930 | 6.30503300 | 2.10371020 |
| 1 | -6.27916380 | 5.10266509 | -0.86675975 |
| 1 | -4.80411039 | 6.13092430 | 3.02692953 |
| 1 | 0.32078086 | 3.71228988 | 3.85855382 |
| 1 | -0.42427138 | 5.13672127 | 4.62722421 |
| 1 | 3.47634129 | 7.84553419 | 1.26230213 |
| 1 | 0.75328910 | 7.08977515 | 4.48694950 |
| 1 | -5.00572669 | 3.95652093 | -3.90399307 |
| 1 | -5.99728143 | 3.64380704 | -2.46797726 |
| 1 | -5.65351000 | 5.32032840 | -2.97439498 |
| 1 | -2.45934321 | 2.75681236 | -1.50674388 |
| 1 | -4.14033654 | 2.17419028 | -1.54897033 |
| 1 | -3.26696602 | 2.48298131 | -3.06895004 |
| 1 | -3.29266654 | 6.24156364 | -2.83705029 |
| 1 | -1.94691969 | 5.22471718 | -2.26360069 |
| 1 | -2.75565485 | 4.85156828 | -3.80756470 |
| 1 | 4.51705511 | 6.28550737 | -1.67458960 |
| 1 | 4.89606811 | 6.72293917 | 0.00140857 |
| 1 | 3.64990681 | 7.62524100 | -0.90356903 |
| 1 | 1.17241363 | 4.79378173 | -1.16552913 |
| 1 | 1.45212186 | 6.51447306 | -1.51861771 |
| 1 | 2.41883438 | 5.26904462 | -2.34314984 |
| 1 | 4.51414147 | 4.29497945 | 0.63103201 |
| 1 | 3.01495851 | 3.45393841 | 0.16216807 |

| | | | |
|---|---|---|---|
| 1 | 4.18465231 | 3.95964824 | -1.08409320 |
| 1 | -1.71911376 | 2.50004845 | 3.85113551 |
| 6 | 0.66011056 | -4.10306258 | -0.18664492 |
| 6 | -0.09095362 | -3.05655074 | 0.63553964 |
| 6 | 0.15565893 | -1.62923191 | 0.13486198 |
| 6 | 0.44547077 | -5.52310408 | 0.30258884 |
| 6 | -0.59782605 | -0.57527788 | 0.95361094 |
| 6 | -0.34510298 | 0.84756419 | 0.46415414 |
| 1 | 1.74542268 | -3.90853675 | -0.17841786 |
| 1 | -1.16872913 | -3.27961842 | 0.61137723 |
| 1 | -0.14480667 | -1.55540492 | -0.92487958 |
| 1 | -1.68152120 | -0.77133667 | 0.91374581 |
| 1 | 0.36514264 | -4.06364972 | -1.24785434 |
| 1 | 0.20986381 | -3.13969385 | 1.69108341 |
| 1 | 1.23820734 | -1.41391410 | 0.16335373 |
| 1 | -0.30189214 | -0.64086240 | 2.01347948 |
| 1 | -0.63915889 | 0.92624637 | -0.60076507 |
| 1 | 0.73717750 | 1.07261572 | 0.52355218 |
| 8 | -0.21655124 | -5.85279449 | 1.26894087 |
| 8 | -1.10580324 | 1.76246423 | 1.26244226 |
| 31 | -0.81113452 | 3.54945775 | 1.14701912 |
| 8 | 3.11410447 | -14.67528811 | -2.01125952 |
| 6 | 3.80283349 | -15.59502340 | -2.89237846 |
| 1 | 3.36561674 | -15.56327424 | -3.89828020 |
| 6 | 2.40943900 | -12.49054534 | -1.42321402 |
| 6 | 2.48211421 | -11.00247347 | -1.76307910 |
| 6 | 1.69530769 | -10.13919448 | -0.77137694 |
| 6 | 3.16530689 | -13.36807368 | -2.40263972 |
| 6 | 1.76922730 | -8.64341274 | -1.10077311 |
| 6 | 0.97589837 | -7.80820105 | -0.10968486 |
| 1 | 1.36411482 | -12.84084693 | -1.40142336 |
| 1 | 2.10100346 | -10.84341081 | -2.78323386 |
| 1 | 0.63976245 | -10.46186948 | -0.76139334 |
| 1 | 1.38300027 | -8.46499447 | -2.11687885 |
| 1 | 2.80565995 | -12.69002611 | -0.41447465 |
| 1 | 3.53586218 | -10.68433565 | -1.78165483 |
| 1 | 2.07868011 | -10.30983952 | 0.24953483 |
| 1 | 2.81958495 | -8.31145744 | -1.09609053 |
| 1 | 1.34932112 | -7.92782394 | 0.91842682 |
| 1 | -0.09129082 | -8.07592333 | -0.10977813 |
| 8 | 3.74197380 | -12.98979033 | -3.40446218 |
| 8 | 1.10812189 | -6.41070176 | -0.49591235 |
| 1 | 3.66800244 | -16.58300619 | -2.44183156 |
| 1 | 4.86783384 | -15.33917227 | -2.95758088 |

Electronic energy = -3908.682172 a.u.
DFT-D3(BJ) dispersion correction = -0.124859 a.u.
Thermal free energy = 0.732315 a.u.
Gibbs free energy = -3908.074716 a.u.
Number of imaginary frequencies = 0.

**Int4 CL isomer/conformer 9**

| Atomic N. | X | Y | Z |
|---|---|---|---|

| | | | |
|---|---|---|---|
| 7 | -1.94174679 | 2.90545954 | 0.39531335 |
| 8 | -3.43813926 | 2.65463806 | -2.17697748 |
| 8 | -0.38876322 | 3.11386208 | -2.18816995 |
| 6 | -2.08655213 | 1.86047549 | 1.44085009 |
| 6 | -4.52183039 | 2.64007372 | -1.35482377 |
| 6 | -5.76984306 | 2.13295941 | -1.81739071 |
| 6 | -6.84478258 | 2.15826393 | -0.91646308 |
| 6 | -6.73338081 | 2.65429480 | 0.38557698 |
| 6 | -5.51402299 | 3.16492044 | 0.81805294 |
| 6 | -4.41205917 | 3.17457937 | -0.04391728 |
| 6 | -3.12919961 | 3.83610142 | 0.38423857 |
| 1 | -7.59784650 | 2.64595406 | 1.04933756 |
| 6 | -5.93872843 | 1.58145332 | -3.24665904 |
| 6 | -0.20787007 | 4.39768736 | -1.78426300 |
| 6 | 0.17846090 | 5.39534168 | -2.72563770 |
| 6 | 0.35973640 | 6.69850480 | -2.24094946 |
| 6 | 0.17743073 | 7.04156146 | -0.89808444 |
| 6 | -0.18536729 | 6.05625001 | 0.01280909 |
| 6 | -0.36695886 | 4.73517984 | -0.41418601 |
| 6 | -0.65117691 | 3.66499058 | 0.60830920 |
| 1 | 0.32287910 | 8.07132302 | -0.57215407 |
| 6 | 0.38793226 | 5.06211323 | -4.21598193 |
| 6 | -7.38452007 | 1.12607581 | -3.52064987 |
| 6 | -5.02298938 | 0.35248106 | -3.45569430 |
| 6 | -5.59692991 | 2.67909912 | -4.28227921 |
| 6 | 0.83315999 | 6.29586592 | -5.02382668 |
| 6 | -0.93447846 | 4.55761156 | -4.84040107 |
| 6 | 1.49180001 | 3.98881202 | -4.37051285 |
| 1 | -2.07337803 | 2.32548279 | 2.43976459 |
| 1 | -3.03565834 | 1.33300474 | 1.29738334 |
| 1 | -3.23840479 | 4.26828649 | 1.39287193 |
| 1 | -2.86378296 | 4.64894407 | -0.30735794 |
| 1 | -7.81015528 | 1.77108674 | -1.23725960 |
| 1 | -5.41111240 | 3.57947706 | 1.82352528 |
| 1 | 0.14162089 | 2.89978472 | 0.59983141 |
| 1 | -0.68120048 | 4.11145690 | 1.61597232 |
| 1 | 0.64836151 | 7.48397749 | -2.93692832 |
| 1 | -0.31778506 | 6.29992720 | 1.06935365 |
| 1 | -7.45163806 | 0.75536427 | -4.55331678 |
| 1 | -7.69558288 | 0.30753292 | -2.85547653 |
| 1 | -8.10410574 | 1.95122390 | -3.41852939 |
| 1 | -3.96600880 | 0.61098044 | -3.33103369 |
| 1 | -5.27126288 | -0.44464390 | -2.74020651 |
| 1 | -5.16328768 | -0.04691544 | -4.47159254 |
| 1 | -6.27346124 | 3.53909126 | -4.17158099 |
| 1 | -4.56574636 | 3.03150557 | -4.17146853 |
| 1 | -5.72238690 | 2.28055316 | -5.30053798 |
| 1 | 0.97953924 | 6.00087496 | -6.07255072 |
| 1 | 1.78593724 | 6.70722954 | -4.66025788 |
| 1 | 0.07914873 | 7.09609028 | -5.00915324 |
| 1 | -1.29254517 | 3.64487527 | -4.35285678 |
| 1 | -1.71960327 | 5.32303435 | -4.75944477 |
| 1 | -0.78127093 | 4.34087503 | -5.90845850 |

| | | | |
|---|---|---|---|
| 1 | 2.44873825 | 4.35807022 | -3.97356869 |
| 1 | 1.23081309 | 3.06373050 | -3.84527568 |
| 1 | 1.63631298 | 3.75654639 | -5.43655814 |
| 1 | -1.26403322 | 1.14219448 | 1.34971595 |
| 6 | 2.00204459 | -3.22473936 | 0.77546155 |
| 6 | 0.95225605 | -3.21589219 | -0.33511722 |
| 6 | 0.60212398 | -1.79781075 | -0.79678069 |
| 6 | 2.38150212 | -4.61758176 | 1.24188138 |
| 6 | -0.45323664 | -1.77425341 | -1.90644374 |
| 6 | -0.82037940 | -0.36957948 | -2.37757858 |
| 1 | 1.65793199 | -2.65560559 | 1.65416364 |
| 1 | 1.31994156 | -3.81018013 | -1.18582711 |
| 1 | 1.51535958 | -1.28999336 | -1.15501443 |
| 1 | -0.08881676 | -2.34251477 | -2.77979319 |
| 1 | 2.92739774 | -2.72253324 | 0.44691954 |
| 1 | 0.04424035 | -3.72820176 | 0.01919854 |
| 1 | 0.23428905 | -1.20769613 | 0.05810116 |
| 1 | -1.37155042 | -2.27705631 | -1.56069378 |
| 1 | -1.50400186 | -0.44638891 | -3.24397503 |
| 1 | 0.09268544 | 0.15543762 | -2.72013416 |
| 8 | 1.98154195 | -5.66408905 | 0.76730676 |
| 8 | -1.44934077 | 0.34832507 | -1.30626392 |
| 31 | -1.79363450 | 2.12205949 | -1.48214871 |
| 8 | 7.99758645 | -8.86921972 | 8.07314187 |
| 6 | 8.96903603 | -8.75505516 | 9.14102620 |
| 1 | 9.91957479 | -8.36597440 | 8.75450235 |
| 6 | 6.67486426 | -7.91337566 | 6.35374127 |
| 6 | 6.16415926 | -6.61543704 | 5.72915409 |
| 6 | 5.18425348 | -6.86643628 | 4.57831078 |
| 6 | 7.67644332 | -7.68711727 | 7.47011864 |
| 6 | 4.66223351 | -5.56681578 | 3.95410822 |
| 6 | 3.70233080 | -5.83556986 | 2.80693491 |
| 1 | 7.16419472 | -8.54842921 | 5.59616375 |
| 1 | 7.02015008 | -6.02404607 | 5.37072352 |
| 1 | 5.67924599 | -7.47493506 | 3.80172291 |
| 1 | 5.50726301 | -4.96353179 | 3.58592405 |
| 1 | 5.84724003 | -8.52020287 | 6.75404913 |
| 1 | 5.67889206 | -6.00413900 | 6.50581105 |
| 1 | 4.33288699 | -7.46674391 | 4.94310204 |
| 1 | 4.15037275 | -4.96382141 | 4.72094707 |
| 1 | 2.82213330 | -6.40894598 | 3.13433451 |
| 1 | 4.18313638 | -6.39905216 | 1.99328296 |
| 8 | 8.14982227 | -6.61603011 | 7.79903903 |
| 8 | 3.25745569 | -4.55018255 | 2.28800413 |
| 1 | 9.09460768 | -9.77079087 | 9.52796623 |
| 1 | 8.59948811 | -8.08142831 | 9.92415810 |

Electronic energy = -3908.682143 a.u.
DFT-D3(BJ) dispersion correction = -0.125477 a.u.
Thermal free energy = 0.737111 a.u.
Gibbs free energy = -3908.070509 a.u.
Number of imaginary frequencies = 0.

**Propagation step of LA addition to the product of CL addition on initiation step**

**Int2 CL vdW LA isomer/conformer 1**

| Atomic N. | X | Y | Z |
|---|---|---|---|
| 7 | 1.95652567 | 2.09175851 | 0.44945639 |
| 8 | -0.97025896 | 2.13321306 | 1.18152696 |
| 8 | 0.01875785 | 0.95719715 | -1.53682114 |
| 6 | 2.90693730 | 1.56121463 | 1.46249379 |
| 6 | -0.55864145 | 3.00221418 | 2.14614887 |
| 6 | -1.43271488 | 3.33601715 | 3.22235662 |
| 6 | -0.92783149 | 4.18703523 | 4.21686825 |
| 6 | 0.35864756 | 4.72996441 | 4.17258532 |
| 6 | 1.17057927 | 4.45887596 | 3.07729634 |
| 6 | 0.71717319 | 3.61892368 | 2.05368207 |
| 6 | 1.53131514 | 3.49523147 | 0.79290850 |
| 6 | -2.88864531 | 2.82993853 | 3.28138355 |
| 6 | 0.46871250 | 1.98102357 | -2.31263867 |
| 6 | -0.31211851 | 2.43666143 | -3.41392486 |
| 6 | 0.22837261 | 3.46559398 | -4.19929488 |
| 6 | 1.46913628 | 4.05050732 | -3.93297952 |
| 6 | 2.21356593 | 3.60448036 | -2.84690874 |
| 6 | 1.73091705 | 2.56850362 | -2.03844230 |
| 6 | 2.59862059 | 2.04643661 | -0.92116774 |
| 6 | -1.70189956 | 1.84515571 | -3.72662035 |
| 6 | -3.64489802 | 3.39109907 | 4.50048346 |
| 6 | -2.93210110 | 1.28963200 | 3.38307641 |
| 6 | -3.64844835 | 3.29219179 | 2.01463791 |
| 6 | -2.33593516 | 2.49414166 | -4.97174786 |
| 6 | -2.66168059 | 2.09642154 | -2.53954388 |
| 6 | -1.59539153 | 0.32763280 | -4.00581749 |
| 1 | 3.81260444 | 2.18790027 | 1.48233402 |
| 1 | 2.43524396 | 1.58081342 | 2.45116365 |
| 1 | 2.43788690 | 4.11860525 | 0.86718801 |
| 1 | 0.95063089 | 3.85225833 | -0.07079758 |
| 1 | -1.56329249 | 4.44230510 | 5.06257038 |
| 1 | 2.15853039 | 4.91689047 | 2.99239489 |
| 1 | 2.87756946 | 0.99536649 | -1.08899022 |
| 1 | 3.53196972 | 2.63066894 | -0.87411913 |
| 1 | -0.34183773 | 3.83600964 | -5.04895314 |
| 1 | 3.18622058 | 4.04761288 | -2.62266199 |
| 1 | -4.67950974 | 3.02049834 | 4.47826668 |
| 1 | -3.19915804 | 3.06650453 | 5.45197000 |
| 1 | -3.68709799 | 4.48960960 | 4.49199033 |
| 1 | -2.48205541 | 0.82300954 | 2.50151266 |
| 1 | -2.39832941 | 0.94129420 | 4.27958749 |
| 1 | -3.97609613 | 0.94955564 | 3.45785467 |
| 1 | -3.66705616 | 4.38992266 | 1.95389657 |
| 1 | -3.18645303 | 2.90102430 | 1.10127710 |
| 1 | -4.68986839 | 2.93874419 | 2.05614386 |
| 1 | -3.31767272 | 2.03398010 | -5.15231147 |
| 1 | -1.72762974 | 2.33970095 | -5.87467699 |
| 1 | -2.49734181 | 3.57371480 | -4.84029104 |

| | | | |
|---|---|---|---|
| 1 | -2.30759665 | 1.61633528 | -1.62105335 |
| 1 | -2.76964252 | 3.17357560 | -2.34760356 |
| 1 | -3.65800894 | 1.69330923 | -2.77606586 |
| 1 | -0.94731071 | 0.13826012 | -4.87396227 |
| 1 | -1.19186829 | -0.21139851 | -3.14187534 |
| 1 | -2.59214158 | -0.07855321 | -4.23495334 |
| 1 | 3.18655630 | 0.53443696 | 1.20325082 |
| 8 | 4.37242971 | -1.22725411 | -0.15408443 |
| 31 | 0.23112535 | 1.00891734 | 0.30635674 |
| 1 | 0.70740925 | 5.37857132 | 4.97594322 |
| 1 | 1.84413906 | 4.85107773 | -4.57033461 |
| 8 | -0.13792512 | -7.82581268 | 0.45072395 |
| 6 | 0.71525257 | -8.92780255 | 0.84555701 |
| 1 | 1.74488899 | -8.75268780 | 0.50957161 |
| 6 | -0.66820158 | -5.51226255 | 0.42684455 |
| 6 | -0.75785357 | -4.30907532 | 1.36973653 |
| 6 | -1.44886529 | -3.10209238 | 0.72629928 |
| 6 | 0.22303848 | -6.61599126 | 0.96058392 |
| 6 | -1.57645240 | -1.88663963 | 1.65663556 |
| 6 | -0.25298987 | -1.21359849 | 2.03153943 |
| 1 | -0.23101197 | -5.21007951 | -0.54103295 |
| 1 | 0.25799591 | -4.04682321 | 1.69808024 |
| 1 | -0.90129062 | -2.80123557 | -0.18249099 |
| 1 | -2.08106753 | -2.18568187 | 2.59237278 |
| 1 | -1.65945295 | -5.92677371 | 0.19535298 |
| 1 | -1.30229986 | -4.60199976 | 2.28283916 |
| 1 | -2.45649793 | -3.39611451 | 0.38974194 |
| 1 | -2.22694977 | -1.13366529 | 1.18129386 |
| 1 | 0.41681669 | -1.94018461 | 2.52341596 |
| 1 | -0.45168910 | -0.41684271 | 2.76904870 |
| 8 | 1.16117657 | -6.46438404 | 1.72410078 |
| 8 | 0.45042396 | -0.70546105 | 0.88712033 |
| 1 | 0.29225574 | -9.80995084 | 0.35572514 |
| 1 | 0.70835473 | -9.04610959 | 1.93625760 |
| 8 | 3.11653880 | -1.46233871 | -1.98178649 |
| 6 | 3.50056756 | -1.84216735 | -0.73402423 |
| 6 | 2.73503221 | -3.02065523 | -0.14492865 |
| 8 | 2.44397255 | -4.02621073 | -1.16937411 |
| 6 | 1.92554071 | -3.59258733 | -2.35016051 |
| 6 | 1.90384409 | -2.07556096 | -2.53548052 |
| 8 | 1.50801584 | -4.38455849 | -3.16577826 |
| 6 | 1.81302731 | -1.66514658 | -3.99058264 |
| 6 | 3.47492818 | -3.71606827 | 0.97975575 |
| 1 | 1.77405369 | -2.60531465 | 0.21320453 |
| 1 | 1.04555881 | -1.67225559 | -1.96705855 |
| 1 | 1.77979478 | -0.57230266 | -4.07069703 |
| 1 | 0.90035622 | -2.08326075 | -4.42997397 |
| 1 | 2.67371875 | -2.04606752 | -4.55396851 |
| 1 | 3.67047806 | -2.99834985 | 1.78500813 |
| 1 | 4.43704216 | -4.10702963 | 0.62593034 |
| 1 | 2.86882960 | -4.54318955 | 1.37063358 |

Electronic energy = -4057.855629 a.u.
DFT-D3(BJ) dispersion correction = -0.140685 a.u.

Thermal free energy = 0.717034 a.u.
Gibbs free energy = -4057.279280 a.u.
Number of imaginary frequencies = 0.

**Int2_CL_vdW_LA isomer/conformer 2**

| Atomic N. | X | Y | Z |
|---|---|---|---|
| 7 | 1.71564496 | 1.52895223 | 1.18809162 |
| 8 | -1.24081441 | 1.71762933 | 0.70136371 |
| 8 | 0.77260293 | 1.69700933 | -1.66931635 |
| 6 | 2.23377784 | 0.45006712 | 2.07114132 |
| 6 | -1.29503546 | 1.90759058 | 2.04704550 |
| 6 | -2.53623388 | 1.77735591 | 2.73512592 |
| 6 | -2.51570478 | 1.94386052 | 4.12789748 |
| 6 | -1.35204341 | 2.24899512 | 4.83931666 |
| 6 | -0.16039377 | 2.43478063 | 4.14669325 |
| 6 | -0.12443198 | 2.28580689 | 2.75584490 |
| 6 | 1.12459060 | 2.65310583 | 1.99860367 |
| 6 | -3.85564084 | 1.50375856 | 1.98490169 |
| 6 | 1.41981396 | 2.89429129 | -1.63428139 |
| 6 | 1.10266231 | 3.90328349 | -2.58897007 |
| 6 | 1.83259638 | 5.09918929 | -2.52245722 |
| 6 | 2.82480798 | 5.32988087 | -1.56593599 |
| 6 | 3.11643290 | 4.33989513 | -0.63464059 |
| 6 | 2.43150343 | 3.11904090 | -0.66442808 |
| 6 | 2.83279456 | 2.02935657 | 0.29801037 |
| 6 | -0.00558378 | 3.70714663 | -3.64369386 |
| 6 | -5.06245804 | 1.46202481 | 2.94121161 |
| 6 | -3.80116200 | 0.14234401 | 1.25489100 |
| 6 | -4.11552216 | 2.63576675 | 0.96195671 |
| 6 | -0.14226422 | 4.93197533 | -4.56815574 |
| 6 | -1.37101522 | 3.50435899 | -2.94523866 |
| 6 | 0.31187604 | 2.48917162 | -4.54261984 |
| 1 | 3.03983928 | 0.84979626 | 2.70717952 |
| 1 | 1.42426427 | 0.08392541 | 2.71253865 |
| 1 | 1.89798478 | 3.01662980 | 2.69569448 |
| 1 | 0.91083214 | 3.46017902 | 1.28222033 |
| 1 | -3.44386626 | 1.83340930 | 4.68544868 |
| 1 | 0.75077579 | 2.72018418 | 4.67720730 |
| 1 | 3.20549142 | 1.14317052 | -0.23842058 |
| 1 | 3.64526444 | 2.39182102 | 0.94893459 |
| 1 | 1.61544753 | 5.89019590 | -3.23756617 |
| 1 | 3.89059418 | 4.49930286 | 0.11904181 |
| 1 | -5.97555589 | 1.28515330 | 2.35534994 |
| 1 | -4.98099541 | 0.65000294 | 3.67846892 |
| 1 | -5.19514586 | 2.41044002 | 3.48115132 |
| 1 | -3.00611734 | 0.12338240 | 0.50190648 |
| 1 | -3.63168800 | -0.67578112 | 1.97062105 |
| 1 | -4.75966278 | -0.04641700 | 0.74809403 |
| 1 | -4.20370048 | 3.60553676 | 1.47272110 |
| 1 | -3.31031739 | 2.70373341 | 0.22204404 |
| 1 | -5.06075156 | 2.44693594 | 0.43079978 |
| 1 | -0.93712404 | 4.73717704 | -5.30187040 |

| | | | |
|---|---|---|---|
| 1 | 0.78216465 | 5.13368220 | -5.12846533 |
| 1 | -0.42039185 | 5.84049625 | -4.01496248 |
| 1 | -1.37470919 | 2.61325174 | -2.30868498 |
| 1 | -1.62311307 | 4.37515125 | -2.32321858 |
| 1 | -2.16115324 | 3.38879423 | -3.70246468 |
| 1 | 1.26524288 | 2.63551241 | -5.07101519 |
| 1 | 0.37049932 | 1.56540779 | -3.95684835 |
| 1 | -0.47784077 | 2.37002454 | -5.29952550 |
| 1 | 2.62833762 | -0.36997647 | 1.45939967 |
| 8 | 3.63096075 | -1.87821887 | -0.08121158 |
| 31 | 0.23869868 | 0.90050235 | -0.08352781 |
| 1 | -1.38706395 | 2.36055252 | 5.92289412 |
| 1 | 3.36051104 | 6.27886741 | -1.55194377 |
| 8 | -1.54603031 | -7.82650514 | 3.56507334 |
| 6 | -0.73970863 | -8.89607908 | 4.11540999 |
| 1 | -0.27187102 | -9.47690652 | 3.31062224 |
| 6 | -1.78351778 | -5.85157545 | 2.27545637 |
| 6 | -1.07489565 | -4.82498638 | 1.39263284 |
| 6 | -2.03482024 | -3.75155116 | 0.86387371 |
| 6 | -0.85518389 | -6.92837006 | 2.80375162 |
| 6 | -1.38487072 | -2.73554712 | -0.08863364 |
| 6 | -0.41058344 | -1.77719800 | 0.59643868 |
| 1 | -2.60138348 | -6.34922149 | 1.72913468 |
| 1 | -0.59896927 | -5.34496523 | 0.54639191 |
| 1 | -2.86530846 | -4.24595896 | 0.33340168 |
| 1 | -2.16630861 | -2.13314248 | -0.57724989 |
| 1 | -2.26310918 | -5.36716416 | 3.14183788 |
| 1 | -0.25532210 | -4.36876237 | 1.96662145 |
| 1 | -2.49559223 | -3.21606693 | 1.71247603 |
| 1 | -0.85286426 | -3.26901887 | -0.89414153 |
| 1 | 0.39921329 | -2.33872644 | 1.09620342 |
| 1 | -0.95292384 | -1.22346532 | 1.38595796 |
| 8 | 0.34082735 | -7.00594488 | 2.59493366 |
| 8 | 0.17755583 | -0.89353789 | -0.37021702 |
| 1 | -1.43503712 | -9.51700867 | 4.68829862 |
| 1 | 0.04523676 | -8.48955465 | 4.76542267 |
| 8 | 3.65774352 | -0.71476692 | -1.98496633 |
| 6 | 3.29144483 | -1.79396783 | -1.24349736 |
| 6 | 2.43905324 | -2.82141518 | -1.98110677 |
| 8 | 2.93802204 | -3.02164559 | -3.34239012 |
| 6 | 3.18839717 | -1.90840706 | -4.08639387 |
| 6 | 3.07201640 | -0.58619384 | -3.32536347 |
| 8 | 3.47553887 | -2.00093948 | -5.25799653 |
| 6 | 3.78072467 | 0.55390176 | -4.02559776 |
| 6 | 2.41839182 | -4.17576890 | -1.30294974 |
| 1 | 1.42055578 | -2.39587969 | -2.03926543 |
| 1 | 2.00042662 | -0.35171733 | -3.19306563 |
| 1 | 3.65340312 | 1.48396877 | -3.45914507 |
| 1 | 3.35405213 | 0.68476825 | -5.02653672 |
| 1 | 4.85000884 | 0.33456115 | -4.13410507 |
| 1 | 2.03726229 | -4.07517181 | -0.28056422 |
| 1 | 3.42931421 | -4.59820014 | -1.25259652 |
| 1 | 1.77059979 | -4.86133195 | -1.86250406 |

Electronic energy = -4057.855019 a.u.
DFT-D3(BJ) dispersion correction = -0.138340 a.u.
Thermal free energy = 0.715327 a.u.
Gibbs free energy = -4057.278032 a.u.
Number of imaginary frequencies = 0.

**Int2 CL vdW LA isomer/conformer 3**

| Atomic N. | X | Y | Z |
|---|---|---|---|
| 7 | 1.74826393 | 1.80901098 | -0.63886955 |
| 8 | -0.88910288 | 2.63862974 | 0.56348528 |
| 8 | -0.67361541 | 0.14176240 | -1.24070342 |
| 6 | 2.93309619 | 1.73495360 | 0.25686290 |
| 6 | -0.22881356 | 3.79958134 | 0.82186586 |
| 6 | -0.77508842 | 4.74036537 | 1.74407425 |
| 6 | -0.02317772 | 5.89570119 | 2.00352329 |
| 6 | 1.20243243 | 6.15962481 | 1.38652599 |
| 6 | 1.69231495 | 5.26459559 | 0.44212269 |
| 6 | 0.98025843 | 4.09861979 | 0.13902561 |
| 6 | 1.43500607 | 3.23547432 | -1.00839752 |
| 6 | -2.14969820 | 4.52524332 | 2.40897621 |
| 6 | -0.42484989 | 0.53313800 | -2.51692420 |
| 6 | -1.45476206 | 0.45611490 | -3.49973691 |
| 6 | -1.12969850 | 0.85419078 | -4.80446698 |
| 6 | 0.14003505 | 1.31583006 | -5.16193872 |
| 6 | 1.13655138 | 1.38145049 | -4.19469839 |
| 6 | 0.87183340 | 0.98352116 | -2.87884140 |
| 6 | 1.99795039 | 0.97243306 | -1.87677307 |
| 6 | -2.87063781 | -0.04406121 | -3.15071839 |
| 6 | -2.54599344 | 5.70918922 | 3.31123520 |
| 6 | -2.13436967 | 3.25856217 | 3.29335172 |
| 6 | -3.24030473 | 4.39082265 | 1.31923635 |
| 6 | -3.80060017 | -0.04705884 | -4.37882241 |
| 6 | -3.51805429 | 0.87604785 | -2.08899087 |
| 6 | -2.80278665 | -1.49690261 | -2.62300635 |
| 1 | 3.80887502 | 2.16113860 | -0.25814165 |
| 1 | 2.73665186 | 2.30713972 | 1.16998627 |
| 1 | 2.33315807 | 3.67161761 | -1.47631428 |
| 1 | 0.65011577 | 3.17995898 | -1.77740771 |
| 1 | -0.40537310 | 6.62352278 | 2.71679052 |
| 1 | 2.62442859 | 5.47276624 | -0.08795906 |
| 1 | 2.20408829 | -0.04435006 | -1.50971102 |
| 1 | 2.91971702 | 1.34521574 | -2.35245073 |
| 1 | -1.89652094 | 0.81138051 | -5.57573626 |
| 1 | 2.13855295 | 1.73000960 | -4.45450352 |
| 1 | -3.53984807 | 5.51393709 | 3.73855079 |
| 1 | -1.84741367 | 5.84510680 | 4.14961413 |
| 1 | -2.60656254 | 6.65352310 | 2.75157010 |
| 1 | -1.93250903 | 2.36382757 | 2.69613043 |
| 1 | -1.36794149 | 3.33893114 | 4.07816306 |
| 1 | -3.11119455 | 3.13336545 | 3.78484427 |
| 1 | -3.29514449 | 5.30622618 | 0.71243580 |
| 1 | -3.04241452 | 3.54366177 | 0.65313366 |

| | | | |
|---|---|---|---|
| 1 | -4.22259072 | 4.24035008 | 1.79251212 |
| 1 | -4.78985802 | -0.41869420 | -4.07585647 |
| 1 | -3.43032938 | -0.70607575 | -5.17743950 |
| 1 | -3.94029214 | 0.96075545 | -4.79560483 |
| 1 | -2.93953858 | 0.89343305 | -1.15956047 |
| 1 | -3.59786356 | 1.90623047 | -2.46481790 |
| 1 | -4.53390580 | 0.52035331 | -1.85875598 |
| 1 | -2.39452637 | -2.16638534 | -3.39485108 |
| 1 | -2.17551884 | -1.57062321 | -1.72826822 |
| 1 | -3.81429775 | -1.85027995 | -2.37100068 |
| 1 | 3.13136223 | 0.68685705 | 0.50521508 |
| 8 | 3.70559528 | -1.59795981 | -0.27246847 |
| 31 | 0.05412579 | 1.06678368 | 0.20103220 |
| 1 | 1.75370667 | 7.06776442 | 1.62984888 |
| 1 | 0.34247138 | 1.62103606 | -6.18846588 |
| 8 | -1.20302066 | -4.76543444 | -0.68481995 |
| 6 | -0.36734267 | -4.77679577 | -1.87055655 |
| 1 | 0.24345405 | -3.86693998 | -1.91179414 |
| 6 | -1.47452053 | -4.62696364 | 1.67649316 |
| 6 | -1.33530852 | -3.29222708 | 2.43606245 |
| 6 | -1.77990255 | -2.08327834 | 1.60571271 |
| 6 | -0.53543489 | -4.69414001 | 0.49173565 |
| 6 | -1.74226438 | -0.75079448 | 2.36699250 |
| 6 | -0.33982982 | -0.23860608 | 2.71064166 |
| 1 | -2.50823994 | -4.77282240 | 1.33648664 |
| 1 | -0.28874556 | -3.17953891 | 2.75653697 |
| 1 | -1.14907748 | -1.99390558 | 0.70722791 |
| 1 | -2.31573986 | -0.83455320 | 3.30724480 |
| 1 | -1.20750337 | -5.45930151 | 2.34488571 |
| 1 | -1.94205921 | -3.35840886 | 3.35360741 |
| 1 | -2.80685203 | -2.25676002 | 1.24458889 |
| 1 | -2.25880101 | 0.01995951 | 1.76702347 |
| 1 | 0.17784340 | -0.96736627 | 3.35907147 |
| 1 | -0.43293653 | 0.69421718 | 3.29567692 |
| 8 | 0.68578106 | -4.67408453 | 0.57002064 |
| 8 | 0.50295091 | -0.04621210 | 1.57373059 |
| 1 | -1.06501808 | -4.81225994 | -2.71191251 |
| 1 | 0.28550392 | -5.65859366 | -1.86807910 |
| 8 | 4.09431246 | -3.77293991 | -0.58416249 |
| 6 | 3.75047054 | -2.71790203 | 0.19624986 |
| 6 | 3.44381546 | -3.07344707 | 1.64907191 |
| 8 | 4.46022384 | -4.00580241 | 2.15042842 |
| 6 | 4.71860379 | -5.10157988 | 1.38377260 |
| 6 | 4.01180153 | -5.10511902 | 0.02724113 |
| 8 | 5.45048893 | -5.97937864 | 1.78230633 |
| 6 | 4.61277480 | -6.09306641 | -0.94995896 |
| 6 | 3.41658583 | -1.87347363 | 2.56996480 |
| 1 | 2.46908861 | -3.59302441 | 1.65823326 |
| 1 | 2.94332277 | -5.32349929 | 0.20018219 |
| 1 | 4.06254110 | -6.06239074 | -1.89848086 |
| 1 | 4.54959674 | -7.10389822 | -0.53042295 |
| 1 | 5.66908753 | -5.86407045 | -1.13545787 |
| 1 | 2.61469021 | -1.19169140 | 2.25504154 |

| | | | |
|---|---|---|---|
| 1 | 4.37331563 | -1.33717191 | 2.54376228 |
| 1 | 3.22094982 | -2.20409878 | 3.59774073 |

Electronic energy = -4057.857037 a.u.
DFT-D3(BJ) dispersion correction = -0.138203 a.u.
Thermal free energy = 0.717763 a.u.
Gibbs free energy = -4057.277476 a.u.
Number of imaginary frequencies = 0.

**Int2 CL vdW LA isomer/conformer 4**

| Atomic N. | X | Y | Z |
|---|---|---|---|
| 7 | 2.72180381 | 1.13052570 | 0.50567390 |
| 8 | -0.12212326 | 2.08150109 | 0.37751448 |
| 8 | 1.21267282 | 0.69424182 | -2.06622174 |
| 6 | 3.14809803 | 0.26596544 | 1.63837215 |
| 6 | 0.18347340 | 2.67035144 | 1.56507645 |
| 6 | -0.85792349 | 3.14076272 | 2.41644516 |
| 6 | -0.47531592 | 3.70102080 | 3.64435821 |
| 6 | 0.85971035 | 3.83012750 | 4.03742098 |
| 6 | 1.86706256 | 3.42189363 | 3.16970008 |
| 6 | 1.54229886 | 2.86036124 | 1.92990569 |
| 6 | 2.63227431 | 2.57346508 | 0.93100616 |
| 6 | -2.34079397 | 3.07574722 | 1.99762365 |
| 6 | 2.12288554 | 1.58227927 | -2.55344743 |
| 6 | 1.85026212 | 2.29354854 | -3.75743359 |
| 6 | 2.84626134 | 3.16285138 | -4.22625629 |
| 6 | 4.05880256 | 3.35717287 | -3.55949343 |
| 6 | 4.30643454 | 2.66386346 | -2.38035970 |
| 6 | 3.35507559 | 1.76981460 | -1.87433553 |
| 6 | 3.68913827 | 0.96151769 | -0.64565127 |
| 6 | 0.51472470 | 2.13557593 | -4.51274941 |
| 6 | -3.26627946 | 3.69247175 | 3.06334193 |
| 6 | -2.79121405 | 1.61158370 | 1.79029352 |
| 6 | -2.54536656 | 3.87569271 | 0.68852387 |
| 6 | 0.47227994 | 2.99530523 | -5.79052842 |
| 6 | -0.65914360 | 2.59073987 | -3.61379378 |
| 6 | 0.30778328 | 0.66479082 | -4.94441386 |
| 1 | 4.15435987 | 0.56588711 | 1.97248979 |
| 1 | 2.44699407 | 0.38554153 | 2.47196070 |
| 1 | 3.61167362 | 2.87151685 | 1.34112922 |
| 1 | 2.46379033 | 3.14877421 | 0.00859813 |
| 1 | -1.24714289 | 4.05418430 | 4.32560924 |
| 1 | 2.91763483 | 3.55589877 | 3.43708934 |
| 1 | 3.69834619 | -0.11624465 | -0.87007403 |
| 1 | 4.69388892 | 1.23611300 | -0.28460607 |
| 1 | 2.67008171 | 3.72085714 | -5.14374027 |
| 1 | 5.24916574 | 2.79927316 | -1.84585502 |
| 1 | -4.30496275 | 3.63859474 | 2.70776953 |
| 1 | -3.21650698 | 3.15240100 | 4.02013104 |
| 1 | -3.03537295 | 4.75119243 | 3.24887786 |
| 1 | -2.22298863 | 1.12672402 | 0.98935720 |
| 1 | -2.66485430 | 1.03136552 | 2.71651252 |
| 1 | -3.85747563 | 1.58566990 | 1.51920809 |

| | | | |
|---|---|---|---|
| 1 | -2.27263569 | 4.93097708 | 0.83371685 |
| 1 | -1.94200439 | 3.46937333 | -0.13083016 |
| 1 | -3.60451015 | 3.83849132 | 0.39186771 |
| 1 | -0.49386528 | 2.84109595 | -6.29132015 |
| 1 | 1.26191104 | 2.71744984 | -6.50360837 |
| 1 | 0.56185652 | 4.06894761 | -5.57134657 |
| 1 | -0.73486755 | 1.98526858 | -2.70441052 |
| 1 | -0.53892503 | 3.64329741 | -3.31993401 |
| 1 | -1.60633567 | 2.49949963 | -4.16648245 |
| 1 | 1.12101444 | 0.33761559 | -5.60865153 |
| 1 | 0.26840897 | -0.00344792 | -4.07731952 |
| 1 | -0.63822228 | 0.56923500 | -5.49807407 |
| 1 | 3.17049313 | -0.78155789 | 1.31455382 |
| 8 | 3.31017459 | -2.94657760 | 0.29397334 |
| 31 | 0.87964248 | 0.63863506 | -0.24396143 |
| 1 | 1.10465371 | 4.26589253 | 5.00588950 |
| 1 | 4.79923660 | 4.04826186 | -3.96186161 |
| 8 | -5.67269432 | -4.90454989 | 5.09857334 |
| 6 | -6.88024724 | -5.70330731 | 5.11215874 |
| 1 | -7.69177998 | -5.18116322 | 4.58992064 |
| 6 | -3.92689100 | -3.79221015 | 3.94349264 |
| 6 | -3.36678153 | -3.38792589 | 2.58034357 |
| 6 | -2.07859452 | -2.56620876 | 2.69757619 |
| 6 | -5.19356166 | -4.62128958 | 3.85129715 |
| 6 | -1.51332736 | -2.14927574 | 1.33527129 |
| 6 | -0.21975749 | -1.34673935 | 1.45519779 |
| 1 | -4.14619351 | -2.90761141 | 4.56305131 |
| 1 | -4.12760339 | -2.81281017 | 2.03034352 |
| 1 | -2.27241739 | -1.66531987 | 3.30540475 |
| 1 | -2.25558915 | -1.54781545 | 0.78649917 |
| 1 | -3.18849034 | -4.37567250 | 4.51817421 |
| 1 | -3.18053756 | -4.29204317 | 1.98076901 |
| 1 | -1.32012310 | -3.15041646 | 3.24796598 |
| 1 | -1.32082670 | -3.04521041 | 0.72193564 |
| 1 | 0.53201922 | -1.93544686 | 2.01549348 |
| 1 | -0.41646135 | -0.43297770 | 2.04638349 |
| 8 | -5.72877950 | -4.99820580 | 2.82627344 |
| 8 | 0.30627488 | -1.03953357 | 0.15594859 |
| 1 | -7.12464475 | -5.84039724 | 6.16989031 |
| 1 | -6.70628110 | -6.67075559 | 4.62449604 |
| 8 | 3.26714376 | -2.44581407 | -1.88057528 |
| 6 | 2.77362411 | -3.08395602 | -0.78623945 |
| 6 | 1.53117953 | -3.93037406 | -1.04430861 |
| 8 | 1.66768524 | -4.67101444 | -2.29860636 |
| 6 | 2.06939750 | -3.97048739 | -3.39624260 |
| 6 | 2.48051956 | -2.52307483 | -3.11893884 |
| 8 | 2.08336631 | -4.49010145 | -4.48844190 |
| 6 | 3.32003556 | -1.93159519 | -4.23127778 |
| 6 | 1.26164141 | -4.94169348 | 0.05069448 |
| 1 | 0.68610089 | -3.22451931 | -1.13974222 |
| 1 | 1.56637597 | -1.92231075 | -2.96364544 |
| 1 | 3.57187590 | -0.88906763 | -4.00327737 |
| 1 | 2.75265311 | -1.96417992 | -5.16836498 |

| | | | |
|---|---|---|---|
| 1 | 4.24199849 | -2.50994131 | -4.36811976 |
| 1 | 1.13993236 | -4.42369621 | 1.00875636 |
| 1 | 2.09864730 | -5.64469172 | 0.14188938 |
| 1 | 0.34557354 | -5.49997404 | -0.17724979 |

Electronic energy = -4057.856174 a.u.
DFT-D3(BJ) dispersion correction = -0.137765 a.u.
Thermal free energy = 0.717441 a.u.
Gibbs free energy = -4057.276498 a.u.
Number of imaginary frequencies = 0.

**Int2 CL vdW LA isomer/conformer 5**

| Atomic N. | X | Y | Z |
|---|---|---|---|
| 7 | 0.47680513 | 1.47543024 | 3.00430161 |
| 8 | -1.38209245 | 1.47442615 | 0.63351733 |
| 8 | 1.67813751 | 1.00556149 | 0.29939188 |
| 6 | 0.05170944 | 0.67350402 | 4.18232709 |
| 6 | -2.23990017 | 2.07225520 | 1.50429454 |
| 6 | -3.62705498 | 2.16713735 | 1.18472956 |
| 6 | -4.46816427 | 2.75242969 | 2.14253893 |
| 6 | -4.00129092 | 3.25522491 | 3.35953901 |
| 6 | -2.63838882 | 3.21534374 | 3.62983509 |
| 6 | -1.75180478 | 2.64882584 | 2.70692753 |
| 6 | -0.27038824 | 2.78123498 | 2.94001524 |
| 6 | -4.18959049 | 1.67971251 | -0.16575037 |
| 6 | 2.36870912 | 2.14002365 | 0.58842716 |
| 6 | 2.96471450 | 2.89703604 | -0.46207246 |
| 6 | 3.69268373 | 4.03862264 | -0.09603827 |
| 6 | 3.83888967 | 4.45157841 | 1.23093863 |
| 6 | 3.24659140 | 3.70692797 | 2.24448065 |
| 6 | 2.52215252 | 2.54839187 | 1.93914541 |
| 6 | 1.97008913 | 1.71365711 | 3.06727755 |
| 6 | 2.81152679 | 2.49384819 | -1.94284076 |
| 6 | -5.69724961 | 1.96562346 | -0.30098342 |
| 6 | -3.99655038 | 0.15409769 | -0.31066316 |
| 6 | -3.47723061 | 2.41416431 | -1.32686053 |
| 6 | 3.56213340 | 3.45804227 | -2.88127996 |
| 6 | 1.31919936 | 2.52642371 | -2.34953821 |
| 6 | 3.39252549 | 1.08013285 | -2.18182971 |
| 1 | 0.26336386 | 1.23678420 | 5.10535024 |
| 1 | -1.02422551 | 0.47708046 | 4.12074969 |
| 1 | -0.08446029 | 3.32783350 | 3.87957428 |
| 1 | 0.19508060 | 3.35374325 | 2.12373163 |
| 1 | -5.53396122 | 2.82203603 | 1.93389156 |
| 1 | -2.24312058 | 3.64610668 | 4.55252396 |
| 1 | 2.42891452 | 0.71357694 | 3.08795185 |
| 1 | 2.19163639 | 2.20355671 | 4.02960559 |
| 1 | 4.15931141 | 4.63878120 | -0.87481024 |
| 1 | 3.35130651 | 4.01087372 | 3.28836135 |
| 1 | -6.03717156 | 1.62054430 | -1.28777362 |
| 1 | -6.29139686 | 1.43421134 | 0.45660457 |
| 1 | -5.92239673 | 3.03944103 | -0.23059050 |
| 1 | -2.93585877 | -0.11332761 | -0.28007056 |

| | | | |
|---|---|---|---|
| 1 | -4.51878369 | -0.38374442 | 0.49425263 |
| 1 | -4.41151657 | -0.18608792 | -1.27176547 |
| 1 | -3.64644141 | 3.49856971 | -1.25864579 |
| 1 | -2.39738944 | 2.23014344 | -1.32066133 |
| 1 | -3.88344146 | 2.06930618 | -2.28973279 |
| 1 | 3.42288659 | 3.12458285 | -3.91927617 |
| 1 | 4.64337770 | 3.47344873 | -2.68103208 |
| 1 | 3.17978121 | 4.48653928 | -2.81157865 |
| 1 | 0.71975921 | 1.83077024 | -1.75290686 |
| 1 | 0.90474381 | 3.53696209 | -2.22314924 |
| 1 | 1.21613691 | 2.24779703 | -3.40901635 |
| 1 | 4.45742829 | 1.04643646 | -1.90759757 |
| 1 | 2.85462871 | 0.32163283 | -1.60330462 |
| 1 | 3.31013617 | 0.81917644 | -3.24740383 |
| 1 | 0.60301605 | -0.27312261 | 4.19898923 |
| 8 | 2.35318102 | -1.95720183 | 4.27725336 |
| 31 | 0.12699615 | 0.54807241 | 1.22365170 |
| 1 | -4.69747285 | 3.69248164 | 4.07505813 |
| 1 | 4.40883839 | 5.35082323 | 1.46429728 |
| 8 | -3.26434730 | -4.90008116 | -5.70595211 |
| 6 | -3.35085059 | -4.86734943 | -7.15031394 |
| 1 | -3.88610951 | -3.96908797 | -7.48345843 |
| 6 | -2.59580493 | -3.94510458 | -3.64088663 |
| 6 | -1.65973876 | -2.94351603 | -2.96451403 |
| 6 | -1.73421096 | -3.01818170 | -1.43588242 |
| 6 | -2.60292991 | -3.83886209 | -5.15330701 |
| 6 | -0.76922950 | -2.04695370 | -0.74599412 |
| 6 | -0.88977014 | -2.08057351 | 0.77742932 |
| 1 | -3.63537884 | -3.79624193 | -3.30128134 |
| 1 | -1.91062865 | -1.92738264 | -3.30401441 |
| 1 | -2.76806561 | -2.80876938 | -1.10970050 |
| 1 | -0.96709739 | -1.01970587 | -1.10295160 |
| 1 | -2.34304266 | -4.98138924 | -3.36795070 |
| 1 | -0.62611193 | -3.12886820 | -3.29503651 |
| 1 | -1.50678956 | -4.04657266 | -1.10599629 |
| 1 | 0.26629865 | -2.28950651 | -1.03049755 |
| 1 | -0.72043474 | -3.11265833 | 1.13161705 |
| 1 | -1.91711013 | -1.80230531 | 1.07845918 |
| 8 | -2.11122471 | -2.94214289 | -5.81146000 |
| 8 | 0.07622744 | -1.26072455 | 1.44747867 |
| 1 | -3.90074055 | -5.77178248 | -7.42817715 |
| 1 | -2.34781912 | -4.87006403 | -7.59522922 |
| 8 | 3.23125355 | -1.54296303 | 2.28134137 |
| 6 | 2.58949341 | -2.34706239 | 3.14888082 |
| 6 | 2.22941367 | -3.76327178 | 2.73171308 |
| 8 | 2.13460745 | -4.00453518 | 1.30097732 |
| 6 | 2.74908629 | -3.18992465 | 0.41045824 |
| 6 | 3.46541552 | -1.93763716 | 0.89211430 |
| 8 | 2.72336167 | -3.45945999 | -0.77168165 |
| 6 | 4.96997076 | -2.03148793 | 0.65221649 |
| 6 | 3.19546943 | -4.77133537 | 3.35658440 |
| 1 | 1.21310768 | -3.93753714 | 3.10920922 |
| 1 | 3.03902753 | -1.10931036 | 0.30706569 |

| | | | |
|---|---|---|---|
| 1 | 5.44617380 | -1.08511219 | 0.93587285 |
| 1 | 5.15333042 | -2.21710392 | -0.41282431 |
| 1 | 5.42034909 | -2.84494279 | 1.23745076 |
| 1 | 3.17492737 | -4.67217736 | 4.44891466 |
| 1 | 4.22323675 | -4.60804245 | 3.00489304 |
| 1 | 2.88763703 | -5.78775594 | 3.08207176 |

Electronic energy = -4057.850760 a.u.
DFT-D3(BJ) dispersion correction = -0.138494 a.u.
Thermal free energy = 0.712844 a.u.
Gibbs free energy = -4057.276410 a.u.
Number of imaginary frequencies = 0.

**Int2 CL vdW LA isomer/conformer 6**

| Atomic N. | X | Y | Z |
|---|---|---|---|
| 7 | 1.78005712 | 1.81894541 | -0.77935444 |
| 8 | -0.68868901 | 2.88548533 | 0.57991401 |
| 8 | -0.87554452 | 0.57320063 | -1.43904486 |
| 6 | 2.96801215 | 1.46897400 | 0.04394623 |
| 6 | 0.14935023 | 3.88317880 | 0.97109379 |
| 6 | -0.22848473 | 4.76243696 | 2.02747169 |
| 6 | 0.69807209 | 5.74528065 | 2.40612440 |
| 6 | 1.93843517 | 5.90085156 | 1.78163026 |
| 6 | 2.26858684 | 5.07545613 | 0.71225211 |
| 6 | 1.37933980 | 4.08163154 | 0.28888763 |
| 6 | 1.67404777 | 3.30982362 | -0.97077326 |
| 6 | -1.60946679 | 4.66994643 | 2.70726942 |
| 6 | -0.61835722 | 1.07378854 | -2.67573928 |
| 6 | -1.68135302 | 1.25139374 | -3.60873837 |
| 6 | -1.34779586 | 1.75309539 | -4.87507415 |
| 6 | -0.03906178 | 2.07925140 | -5.24070516 |
| 6 | 0.98896154 | 1.89525671 | -4.32312647 |
| 6 | 0.71563574 | 1.38459615 | -3.04866783 |
| 6 | 1.86245092 | 1.10283035 | -2.11179386 |
| 6 | -3.14096398 | 0.90893368 | -3.24907005 |
| 6 | -1.79615268 | 5.75600576 | 3.78345114 |
| 6 | -1.78771894 | 3.30053233 | 3.40050334 |
| 6 | -2.72211225 | 4.86957718 | 1.65020319 |
| 6 | -4.10121409 | 1.17134687 | -4.42474914 |
| 6 | -3.61880971 | 1.78153276 | -2.06451392 |
| 6 | -3.25948481 | -0.59027443 | -2.88581715 |
| 1 | 3.88063248 | 1.82424856 | -0.46088053 |
| 1 | 2.88645539 | 1.94844581 | 1.02525746 |
| 1 | 2.61606343 | 3.66447041 | -1.42083772 |
| 1 | 0.87084172 | 3.46164111 | -1.70701604 |
| 1 | 0.44625820 | 6.42027868 | 3.22195242 |
| 1 | 3.21286823 | 5.20892266 | 0.17959739 |
| 1 | 1.92919395 | 0.03189535 | -1.86645712 |
| 1 | 2.81166476 | 1.39305254 | -2.59081510 |
| 1 | -2.13830348 | 1.90412816 | -5.60780340 |
| 1 | 2.02051523 | 2.13322297 | -4.59197363 |
| 1 | -2.80022070 | 5.65652432 | 4.21978829 |
| 1 | -1.06883004 | 5.65843862 | 4.60253848 |

| | | | |
|---|---|---|---|
| 1 | -1.71650236 | 6.76988207 | 3.36586609 |
| 1 | -1.75133440 | 2.48291749 | 2.67337800 |
| 1 | -1.00290095 | 3.13800513 | 4.15382365 |
| 1 | -2.76158620 | 3.26477964 | 3.91195646 |
| 1 | -2.63743550 | 5.86126198 | 1.18275298 |
| 1 | -2.67090654 | 4.10860394 | 0.86338953 |
| 1 | -3.70961369 | 4.80663150 | 2.13231689 |
| 1 | -5.12220680 | 0.90362174 | -4.11743567 |
| 1 | -3.85160683 | 0.56420881 | -5.30688835 |
| 1 | -4.11328267 | 2.22988608 | -4.72196959 |
| 1 | -3.01400221 | 1.61422637 | -1.16719807 |
| 1 | -3.56512201 | 2.84906029 | -2.32218553 |
| 1 | -4.66660561 | 1.54308736 | -1.82619640 |
| 1 | -2.96794257 | -1.21912788 | -3.73977336 |
| 1 | -2.62339678 | -0.84693712 | -2.03201916 |
| 1 | -4.30307627 | -0.82989266 | -2.63027602 |
| 1 | 3.01723750 | 0.38117137 | 0.16073603 |
| 8 | 3.38321657 | -1.88331558 | -0.76424666 |
| 31 | 0.01892389 | 1.23993119 | 0.05026642 |
| 1 | 2.62762019 | 6.67487378 | 2.11898422 |
| 1 | 0.16925263 | 2.47266451 | -6.23554293 |
| 8 | -1.40350887 | -6.62035595 | 1.39839690 |
| 6 | -0.70884673 | -7.79230514 | 1.89702729 |
| 1 | -0.10525578 | -8.24452319 | 1.10060291 |
| 6 | -1.38829899 | -4.41779370 | 0.49595166 |
| 6 | -1.29958314 | -3.26389013 | 1.51607919 |
| 6 | -1.92095354 | -1.97689745 | 0.96512852 |
| 6 | -0.59984150 | -5.62072179 | 0.95628077 |
| 6 | -1.89761023 | -0.79530487 | 1.94521501 |
| 6 | -0.49947712 | -0.34397072 | 2.38090346 |
| 1 | -0.95117460 | -4.08727622 | -0.45801408 |
| 1 | -0.24143353 | -3.09320645 | 1.76283822 |
| 1 | -1.38897311 | -1.68948497 | 0.04489403 |
| 1 | -2.47020655 | -1.04390133 | 2.85620360 |
| 1 | -2.43573796 | -4.69667640 | 0.32211730 |
| 1 | -1.80344490 | -3.56205347 | 2.45036985 |
| 1 | -2.96549282 | -2.17262165 | 0.67217171 |
| 1 | -2.42087523 | 0.05984955 | 1.48110641 |
| 1 | -0.01067171 | -1.14969355 | 2.95644822 |
| 1 | -0.59872114 | 0.51597099 | 3.06758738 |
| 8 | 0.62210408 | -5.69524905 | 0.96441839 |
| 8 | 0.38052665 | -0.03738322 | 1.29951187 |
| 1 | -1.49759577 | -8.47674099 | 2.22220268 |
| 1 | -0.05592839 | -7.52127002 | 2.73547252 |
| 8 | 3.77644075 | -4.05014345 | -1.13163607 |
| 6 | 3.45417808 | -3.01156239 | -0.31990401 |
| 6 | 3.19869174 | -3.40019515 | 1.13487582 |
| 8 | 4.24138952 | -4.32853578 | 1.58360369 |
| 6 | 4.48187818 | -5.40870398 | 0.78872347 |
| 6 | 3.72531465 | -5.39443458 | -0.54083034 |
| 8 | 5.23829011 | -6.28409968 | 1.14331879 |
| 6 | 4.29510018 | -6.36042173 | -1.55740339 |
| 6 | 3.18788442 | -2.21928087 | 2.08076952 |

| | | | |
|---|---|---|---|
| 1 | 2.23324672 | -3.93790568 | 1.16873591 |
| 1 | 2.66759260 | -5.62698290 | -0.32498048 |
| 1 | 3.71214633 | -6.31407326 | -2.48539214 |
| 1 | 4.25060719 | -7.37922224 | -1.15521776 |
| 1 | 5.34346392 | -6.12286058 | -1.77470072 |
| 1 | 2.38613595 | -1.52473397 | 1.79450452 |
| 1 | 4.14602985 | -1.68558250 | 2.04903757 |
| 1 | 3.00900213 | -2.56964824 | 3.10491352 |

Electronic energy = -4057.856178 a.u.
DFT-D3(BJ) dispersion correction = -0.135897 a.u.
Thermal free energy = 0.716882 a.u.
Gibbs free energy = -4057.275194 a.u.
Number of imaginary frequencies = 0.

**Int2 CL vdW LA isomer/conformer 7**

| Atomic N. | X | Y | Z |
|---|---|---|---|
| 7 | 1.74255947 | 1.71825854 | 0.77582127 |
| 8 | -1.10255986 | 2.61258760 | 1.05029178 |
| 8 | -0.05457603 | 1.46488714 | -1.63180887 |
| 6 | 2.31741898 | 0.74891393 | 1.74498333 |
| 6 | -0.68327730 | 3.07564103 | 2.25682611 |
| 6 | -1.63357755 | 3.42545132 | 3.25993048 |
| 6 | -1.12939093 | 3.86055751 | 4.49458680 |
| 6 | 0.23833116 | 3.97967214 | 4.75751504 |
| 6 | 1.15263319 | 3.69067867 | 3.74982334 |
| 6 | 0.70434445 | 3.25778596 | 2.49719825 |
| 6 | 1.68235402 | 3.10367959 | 1.36253136 |
| 6 | -3.15140848 | 3.36352457 | 2.99491039 |
| 6 | 0.76682913 | 2.41930149 | -2.14255692 |
| 6 | 0.34345691 | 3.19887821 | -3.25760955 |
| 6 | 1.24133433 | 4.15839311 | -3.74736213 |
| 6 | 2.50483244 | 4.36903918 | -3.18814790 |
| 6 | 2.90949458 | 3.59258835 | -2.10800517 |
| 6 | 2.05932477 | 2.60951593 | -1.58742947 |
| 6 | 2.56421044 | 1.70188629 | -0.49434210 |
| 6 | -1.04316003 | 3.00178702 | -3.90219944 |
| 6 | -3.96703606 | 3.83915374 | 4.21190786 |
| 6 | -3.59623503 | 1.91679514 | 2.68043196 |
| 6 | -3.50905528 | 4.28969535 | 1.80760407 |
| 6 | -1.25223910 | 3.93307468 | -5.11136817 |
| 6 | -2.15787568 | 3.32091597 | -2.87810371 |
| 6 | -1.19293985 | 1.54791565 | -4.41074048 |
| 1 | 3.35730378 | 1.02941414 | 1.97771296 |
| 1 | 1.72819952 | 0.76321053 | 2.66865084 |
| 1 | 2.69722426 | 3.38051105 | 1.69440956 |
| 1 | 1.40607896 | 3.76789423 | 0.53018357 |
| 1 | -1.82864290 | 4.11878904 | 5.28777534 |
| 1 | 2.22413108 | 3.81978829 | 3.91891166 |
| 1 | 2.57417723 | 0.65113857 | -0.82147255 |
| 1 | 3.59785125 | 1.98075527 | -0.23005788 |
| 1 | 0.94523987 | 4.77448138 | -4.59438567 |
| 1 | 3.89854423 | 3.73137958 | -1.66562403 |

| | | | |
|---|---|---|---|
| 1 | -5.03693826 | 3.79413805 | 3.96360063 |
| 1 | -3.80612972 | 3.20290782 | 5.09445776 |
| 1 | -3.73465009 | 4.87855324 | 4.48486648 |
| 1 | -3.10751893 | 1.53341343 | 1.77841257 |
| 1 | -3.36467281 | 1.24548469 | 3.52064188 |
| 1 | -4.68463331 | 1.89030856 | 2.51887227 |
| 1 | -3.24088829 | 5.33160398 | 2.03526088 |
| 1 | -2.98953397 | 3.98807951 | 0.89130309 |
| 1 | -4.59316756 | 4.25300724 | 1.62118073 |
| 1 | -2.24690083 | 3.74376140 | -5.53930738 |
| 1 | -0.51142066 | 3.75473445 | -5.90428756 |
| 1 | -1.21193340 | 4.99503368 | -4.82910474 |
| 1 | -2.11192310 | 2.65813541 | -2.00768286 |
| 1 | -2.07734373 | 4.35973645 | -2.52705098 |
| 1 | -3.14362265 | 3.19862508 | -3.35205829 |
| 1 | -0.42739279 | 1.32278575 | -5.16800913 |
| 1 | -1.10086770 | 0.82509847 | -3.59292103 |
| 1 | -2.17990222 | 1.41916272 | -4.88048254 |
| 1 | 2.30349326 | -0.25497867 | 1.30603648 |
| 8 | 2.97349204 | -1.74162339 | -0.69061888 |
| 31 | -0.16768509 | 1.23529437 | 0.20230613 |
| 1 | 0.57950005 | 4.31462088 | 5.73698490 |
| 1 | 3.16398419 | 5.13373092 | -3.59896356 |
| 8 | -0.22729354 | -7.52343850 | 0.82020819 |
| 6 | 0.87355478 | -8.46189845 | 0.89744707 |
| 1 | 1.52793547 | -8.34229934 | 0.02481626 |
| 6 | -1.06625905 | -5.31059182 | 0.66977254 |
| 6 | -1.00324532 | -4.00686636 | 1.47221518 |
| 6 | -2.11917903 | -3.03495115 | 1.07320603 |
| 6 | 0.13041956 | -6.21223383 | 0.88789601 |
| 6 | -2.20219243 | -1.77347504 | 1.94383742 |
| 6 | -0.96034662 | -0.88227954 | 1.90513596 |
| 1 | -1.07986076 | -5.08341140 | -0.41090755 |
| 1 | -0.01647142 | -3.54657713 | 1.32097774 |
| 1 | -1.98297753 | -2.73546430 | 0.02180987 |
| 1 | -2.37669767 | -2.05795827 | 2.99678346 |
| 1 | -1.98720185 | -5.87195778 | 0.87966764 |
| 1 | -1.07640529 | -4.23896802 | 2.54821113 |
| 1 | -3.09037660 | -3.55531902 | 1.12311636 |
| 1 | -3.07092883 | -1.17408257 | 1.62859169 |
| 1 | -0.08936486 | -1.43505784 | 2.30517969 |
| 1 | -1.12947796 | -0.01997241 | 2.57578984 |
| 8 | 1.27678652 | -5.83739398 | 1.07383380 |
| 8 | -0.67236183 | -0.46985333 | 0.56228214 |
| 1 | 0.40770553 | -9.45202287 | 0.89727829 |
| 1 | 1.45064912 | -8.30256876 | 1.81686366 |
| 8 | 3.61771554 | -3.86153899 | -0.92568706 |
| 6 | 2.84029399 | -2.80493873 | -1.26095208 |
| 6 | 1.84246079 | -3.05810657 | -2.39081591 |
| 8 | 1.23810533 | -4.38462942 | -2.26224918 |
| 6 | 2.07230347 | -5.43901141 | -2.05192468 |
| 6 | 3.52477297 | -5.07158447 | -1.74094336 |
| 8 | 1.66266906 | -6.57477987 | -2.13515362 |

| | | | |
|---|---|---|---|
| 6 | 4.25761090 | -6.16966194 | -0.99542948 |
| 6 | 0.72071685 | -2.04161345 | -2.43619706 |
| 1 | 2.41128951 | -3.04317544 | -3.34002475 |
| 1 | 4.02228643 | -4.86084904 | -2.70662118 |
| 1 | 5.29885223 | -5.87094234 | -0.82407293 |
| 1 | 4.23851092 | -7.09089511 | -1.58892848 |
| 1 | 3.76432072 | -6.35596370 | -0.03369417 |
| 1 | 1.12786686 | -1.04453233 | -2.63993432 |
| 1 | 0.18756574 | -1.99490789 | -1.47815350 |
| 1 | 0.01900387 | -2.30378182 | -3.23745945 |

Electronic energy = -4057.853842 a.u.
DFT-D3(BJ) dispersion correction = -0.137537 a.u.
Thermal free energy = 0.717786 a.u.
Gibbs free energy = -4057.273593 a.u.
Number of imaginary frequencies = 0.

**Int2 CL vdW LA isomer/conformer 8**

| Atomic N. | X | Y | Z |
|---|---|---|---|
| 7 | -2.11412059 | 2.03745909 | 0.87379147 |
| 8 | -2.24920684 | -0.95208084 | 0.73712294 |
| 8 | -1.44584617 | 0.77320564 | -1.76214332 |
| 6 | -1.35748837 | 2.61895058 | 2.01069914 |
| 6 | -2.86279124 | -0.81265994 | 1.94471821 |
| 6 | -2.96016976 | -1.92819256 | 2.82560360 |
| 6 | -3.60816183 | -1.72095476 | 4.05221961 |
| 6 | -4.14879415 | -0.48823800 | 4.42776579 |
| 6 | -4.06558098 | 0.58618826 | 3.54946085 |
| 6 | -3.44130359 | 0.43158780 | 2.30627260 |
| 6 | -3.47692924 | 1.56293447 | 1.31495529 |
| 6 | -2.37818798 | -3.30661094 | 2.45733268 |
| 6 | -2.53051810 | 1.46877853 | -2.19241485 |
| 6 | -3.18230384 | 1.09588238 | -3.40301964 |
| 6 | -4.28728175 | 1.86386256 | -3.79738502 |
| 6 | -4.75695778 | 2.95401560 | -3.05898158 |
| 6 | -4.10436554 | 3.31377645 | -1.88564681 |
| 6 | -2.98778361 | 2.58890507 | -1.45169093 |
| 6 | -2.23329058 | 3.05854373 | -0.23538881 |
| 6 | -2.69777458 | -0.09910354 | -4.24779655 |
| 6 | -2.64824876 | -4.35880916 | 3.54955352 |
| 6 | -0.84392282 | -3.20315101 | 2.29230698 |
| 6 | -3.01925499 | -3.82932994 | 1.14955343 |
| 6 | -3.54439305 | -0.28323090 | -5.52143725 |
| 6 | -2.80592332 | -1.40885343 | -3.43178287 |
| 6 | -1.23438907 | 0.12740286 | -4.69699802 |
| 1 | -1.88352628 | 3.50657832 | 2.39726341 |
| 1 | -1.26768157 | 1.87578101 | 2.81015097 |
| 1 | -4.01882047 | 2.42430477 | 1.74006026 |
| 1 | -3.99852649 | 1.25915861 | 0.39532242 |
| 1 | -3.69233545 | -2.55255469 | 4.74921099 |
| 1 | -4.50367148 | 1.55202371 | 3.81108135 |
| 1 | -1.19838210 | 3.33025314 | -0.49891953 |
| 1 | -2.72083906 | 3.95595881 | 0.18019849 |

| | | | |
|---|---|---|---|
| 1 | -4.81099755 | 1.60129782 | -4.71460185 |
| 1 | -4.44741736 | 4.17006125 | -1.30076950 |
| 1 | -2.22263840 | -5.32004311 | 3.22920564 |
| 1 | -2.17901215 | -4.09404879 | 4.50818336 |
| 1 | -3.72390505 | -4.51226765 | 3.71800025 |
| 1 | -0.57661273 | -2.49028522 | 1.50364470 |
| 1 | -0.37635348 | -2.87185224 | 3.23144124 |
| 1 | -0.42954798 | -4.18844075 | 2.03359651 |
| 1 | -4.10369237 | -3.95995846 | 1.27745119 |
| 1 | -2.84964623 | -3.14428075 | 0.31207073 |
| 1 | -2.58811938 | -4.80805564 | 0.89171696 |
| 1 | -3.15128123 | -1.13760735 | -6.09028648 |
| 1 | -3.50169204 | 0.59798336 | -6.17769192 |
| 1 | -4.59824958 | -0.49730781 | -5.29223651 |
| 1 | -2.18629249 | -1.37892222 | -2.52924589 |
| 1 | -3.84646237 | -1.59493870 | -3.12949897 |
| 1 | -2.47658946 | -2.25865956 | -4.04858787 |
| 1 | -1.15530058 | 1.03182715 | -5.31762240 |
| 1 | -0.56134391 | 0.23528405 | -3.83943372 |
| 1 | -0.89611099 | -0.72673145 | -5.30310620 |
| 1 | -0.35476398 | 2.90613589 | 1.67340598 |
| 8 | 1.12236895 | -5.24465382 | -0.55908819 |
| 31 | -1.19328242 | 0.40141802 | 0.04071718 |
| 1 | -4.63723517 | -0.37710046 | 5.39567763 |
| 1 | -5.62495244 | 3.51506178 | -3.40458595 |
| 8 | 8.18784706 | 4.29033369 | 0.37515295 |
| 6 | 9.19995691 | 4.95476346 | 1.16855164 |
| 1 | 8.82343303 | 5.91310653 | 1.54823702 |
| 6 | 6.03095961 | 3.33596580 | 0.13040236 |
| 6 | 4.83226834 | 2.74453429 | 0.87184387 |
| 6 | 3.78818018 | 2.15520636 | -0.08188982 |
| 6 | 7.03661933 | 4.00013905 | 1.05038584 |
| 6 | 2.61120879 | 1.50935259 | 0.65792238 |
| 6 | 1.55177257 | 0.95879516 | -0.29317926 |
| 1 | 5.70266879 | 4.09952556 | -0.59584170 |
| 1 | 4.37588867 | 3.52313534 | 1.50185650 |
| 1 | 3.41634222 | 2.94965177 | -0.75336484 |
| 1 | 2.14752937 | 2.23845015 | 1.34431423 |
| 1 | 6.55385057 | 2.57035057 | -0.46269981 |
| 1 | 5.18414905 | 1.96143506 | 1.56128363 |
| 1 | 4.26439912 | 1.39723217 | -0.72549413 |
| 1 | 2.98261243 | 0.68438807 | 1.28654863 |
| 1 | 2.03418456 | 0.30879512 | -1.04484039 |
| 1 | 1.08729562 | 1.78874881 | -0.85821108 |
| 8 | 6.86628513 | 4.26346014 | 2.22606528 |
| 8 | 0.57085449 | 0.19281996 | 0.43456719 |
| 1 | 10.04353781 | 5.11126863 | 0.48937498 |
| 1 | 9.49584248 | 4.32594674 | 2.01764120 |
| 8 | 2.93936547 | -2.33012937 | -1.26356245 |
| 6 | 3.59647253 | -2.11872683 | -0.08967323 |
| 6 | 2.90528748 | -2.71287440 | 1.13767577 |
| 8 | 2.40890858 | -4.05593068 | 0.83370926 |
| 6 | 1.67000738 | -4.19652786 | -0.30342193 |

| | | | |
|---|---|---|---|
| 6 | 1.60902528 | -2.94085332 | -1.17376584 |
| 8 | 4.63542315 | -1.49593686 | -0.06734490 |
| 6 | 1.13080764 | -3.21586857 | -2.58327150 |
| 6 | 3.81938494 | -2.82443471 | 2.33978155 |
| 1 | 2.03687318 | -2.06896789 | 1.37046724 |
| 1 | 0.94316381 | -2.21363445 | -0.67273073 |
| 1 | 1.09693845 | -2.28043059 | -3.15506563 |
| 1 | 0.12472621 | -3.65005590 | -2.54753152 |
| 1 | 1.79588970 | -3.92780971 | -3.08676070 |
| 1 | 4.19131700 | -1.82975073 | 2.61173220 |
| 1 | 4.68026280 | -3.46509237 | 2.11388045 |
| 1 | 3.26636209 | -3.24607779 | 3.18786055 |

Electronic energy = -4057.851672 a.u.
DFT-D3(BJ) dispersion correction = -0.135434 a.u.
Thermal free energy = 0.714476 a.u.
Gibbs free energy = -4057.272630 a.u.
Number of imaginary frequencies = 0.

**Int2 CL vdW LA isomer/conformer 9**

| Atomic N. | X | Y | Z |
|---|---|---|---|
| 7 | 2.87975684 | 1.84108369 | 0.42117557 |
| 8 | -0.04937473 | 1.96842388 | 1.12050260 |
| 8 | 0.88293145 | 0.97312070 | -1.64724914 |
| 6 | 3.82127896 | 1.15308309 | 1.34361050 |
| 6 | 0.39558334 | 2.66278885 | 2.20188347 |
| 6 | -0.46783602 | 2.88530671 | 3.31457685 |
| 6 | 0.06999361 | 3.56503483 | 4.41755938 |
| 6 | 1.38245906 | 4.04382212 | 4.44926899 |
| 6 | 2.19052851 | 3.88294340 | 3.32907401 |
| 6 | 1.70404035 | 3.21610444 | 2.19924917 |
| 6 | 2.52090826 | 3.21070939 | 0.93365447 |
| 6 | -1.94444020 | 2.44070924 | 3.30060546 |
| 6 | 1.35139608 | 2.05540285 | -2.32199964 |
| 6 | 0.58220595 | 2.62727694 | -3.37679318 |
| 6 | 1.13262167 | 3.72934279 | -4.04727585 |
| 6 | 2.37743902 | 4.27283178 | -3.71814378 |
| 6 | 3.11908864 | 3.70150311 | -2.69019817 |
| 6 | 2.62545190 | 2.59002099 | -1.99673723 |
| 6 | 3.49409929 | 1.92437133 | -0.95959576 |
| 6 | -0.80091619 | 2.06812072 | -3.76637483 |
| 6 | -2.68202949 | 2.85099374 | 4.58914143 |
| 6 | -2.05451041 | 0.90469015 | 3.17714692 |
| 6 | -2.67659120 | 3.11375190 | 2.11464696 |
| 6 | -1.41839459 | 2.83517685 | -4.95114136 |
| 6 | -1.78054648 | 2.19633538 | -2.57596981 |
| 6 | -0.67939539 | 0.58598333 | -4.19344838 |
| 1 | 4.75814580 | 1.72952861 | 1.40963123 |
| 1 | 3.36865190 | 1.08170537 | 2.33843259 |
| 1 | 3.45565187 | 3.77624348 | 1.08447718 |
| 1 | 1.96138703 | 3.69650867 | 0.12035668 |
| 1 | -0.55810302 | 3.73180197 | 5.29065330 |
| 1 | 3.20204478 | 4.29505719 | 3.31094125 |

| | | | |
|---|---|---|---|
| 1 | 3.72673136 | 0.88597589 | -1.24074697 |
| 1 | 4.44787121 | 2.47066595 | -0.87113489 |
| 1 | 0.56798765 | 4.19133979 | -4.85496700 |
| 1 | 4.09874594 | 4.10460049 | -2.42369608 |
| 1 | -3.73129508 | 2.53149634 | 4.51581786 |
| 1 | -2.25347639 | 2.37461047 | 5.48295234 |
| 1 | -2.67797288 | 3.94014304 | 4.73900645 |
| 1 | -1.61767320 | 0.55179991 | 2.23755125 |
| 1 | -1.54227383 | 0.40719851 | 4.01377602 |
| 1 | -3.11299763 | 0.60368340 | 3.20016904 |
| 1 | -2.64766008 | 4.20852141 | 2.21379214 |
| 1 | -2.22571948 | 2.83888335 | 1.15452293 |
| 1 | -3.73247821 | 2.80330445 | 2.10470503 |
| 1 | -2.39489487 | 2.39179200 | -5.19213429 |
| 1 | -0.79471142 | 2.77305043 | -5.85477611 |
| 1 | -1.58716535 | 3.89599625 | -4.71613647 |
| 1 | -1.43288046 | 1.63549279 | -1.70213857 |
| 1 | -1.90173920 | 3.24958142 | -2.28453176 |
| 1 | -2.76894779 | 1.80778838 | -2.86428303 |
| 1 | -0.00931862 | 0.48687662 | -5.06026608 |
| 1 | -0.29305693 | -0.03559904 | -3.37884075 |
| 1 | -1.66832318 | 0.20130375 | -4.48508334 |
| 1 | 4.03205476 | 0.14759514 | 0.96358187 |
| 8 | 4.43367329 | -1.42496919 | -1.02091743 |
| 31 | 1.11835789 | 0.84073065 | 0.19428012 |
| 1 | 1.75630776 | 4.55793938 | 5.33461770 |
| 1 | 2.75908111 | 5.13564721 | -4.26396082 |
| 8 | -6.16720285 | -5.19652277 | 0.60391850 |
| 6 | -7.36639886 | -5.51191345 | -0.14178973 |
| 1 | -7.94809406 | -4.60223773 | -0.33788731 |
| 6 | -4.09772353 | -4.06334104 | 0.83118263 |
| 6 | -3.09643573 | -3.10324404 | 0.19008801 |
| 6 | -1.88025624 | -2.85065811 | 1.08726201 |
| 6 | -5.31801212 | -4.33004006 | -0.02740100 |
| 6 | -0.85283609 | -1.90944647 | 0.44857402 |
| 6 | 0.34758782 | -1.64607943 | 1.35691860 |
| 1 | -4.45005216 | -3.67908404 | 1.80267472 |
| 1 | -3.60007720 | -2.15156313 | -0.03923624 |
| 1 | -2.21689938 | -2.43142586 | 2.05172182 |
| 1 | -1.33806584 | -0.94778514 | 0.19834815 |
| 1 | -3.62661202 | -5.03435377 | 1.05527204 |
| 1 | -2.76730227 | -3.51337735 | -0.77665331 |
| 1 | -1.38972393 | -3.81177923 | 1.31720002 |
| 1 | -0.49163604 | -2.33896582 | -0.49901998 |
| 1 | 0.80358114 | -2.60964556 | 1.64375152 |
| 1 | 0.00940968 | -1.16590684 | 2.29402351 |
| 8 | -5.54225534 | -3.85743474 | -1.12539795 |
| 8 | 1.38048259 | -0.87653931 | 0.73365143 |
| 1 | -7.92955566 | -6.20261335 | 0.49337523 |
| 1 | -7.11117240 | -5.98453228 | -1.09876704 |
| 8 | 4.11286333 | -3.27231129 | 0.18810887 |
| 6 | 3.89697520 | -2.50687062 | -0.91303078 |
| 6 | 2.98977536 | -3.13460473 | -1.97235658 |

| | | | |
|---|---|---|---|
| 8 | 1.85726356 | -3.82022729 | -1.35158393 |
| 6 | 2.11216488 | -4.62606150 | -0.28787053 |
| 6 | 3.56944396 | -4.62984322 | 0.18040189 |
| 8 | 1.23015920 | -5.28413052 | 0.21679602 |
| 6 | 3.74205586 | -5.19809257 | 1.57357675 |
| 6 | 2.43369059 | -2.12466378 | -2.95466989 |
| 1 | 3.59167237 | -3.89641183 | -2.50422776 |
| 1 | 4.15264842 | -5.22196207 | -0.54974117 |
| 1 | 4.80294786 | -5.18948984 | 1.85147299 |
| 1 | 3.36981078 | -6.22868287 | 1.59873437 |
| 1 | 3.17158498 | -4.60677932 | 2.29982188 |
| 1 | 3.26017198 | -1.63107086 | -3.47944712 |
| 1 | 1.84547097 | -1.36065245 | -2.43141614 |
| 1 | 1.79879827 | -2.63488200 | -3.68919857 |

Electronic energy = -4057.852566 a.u.
DFT-D3(BJ) dispersion correction = -0.136234 a.u.
Thermal free energy = 0.716948 a.u.
Gibbs free energy = -4057.271852 a.u.
Number of imaginary frequencies = 0.

**Int2 CL vdW LA isomer/conformer 10**

| Atomic N. | X | Y | Z |
|---|---|---|---|
| 7 | 0.16059510 | 1.92060687 | 1.96170649 |
| 8 | -0.96895031 | 2.10963752 | -0.81936194 |
| 8 | 2.03299920 | 1.52542967 | -0.36335127 |
| 6 | -0.48962834 | 1.02935343 | 2.95844546 |
| 6 | -2.07597336 | 2.56562883 | -0.17380034 |
| 6 | -3.32619788 | 2.62940962 | -0.85544180 |
| 6 | -4.43679513 | 3.06040412 | -0.11441385 |
| 6 | -4.35289579 | 3.44707251 | 1.22601575 |
| 6 | -3.11327231 | 3.44892300 | 1.85649372 |
| 6 | -1.97138579 | 3.03122059 | 1.16402106 |
| 6 | -0.61377301 | 3.20128432 | 1.79441605 |
| 6 | -3.45422822 | 2.28893213 | -2.35432942 |
| 6 | 2.58270869 | 2.68174850 | 0.09902360 |
| 6 | 3.39494497 | 3.47837227 | -0.75917261 |
| 6 | 3.95663474 | 4.64135123 | -0.21178643 |
| 6 | 3.74126967 | 5.03771609 | 1.11065376 |
| 6 | 2.94184367 | 4.25358933 | 1.93452282 |
| 6 | 2.36862276 | 3.07315483 | 1.44662275 |
| 6 | 1.58566110 | 2.19464264 | 2.38970380 |
| 6 | 3.64151516 | 3.09689796 | -2.23302911 |
| 6 | -4.89263592 | 2.48959589 | -2.86779980 |
| 6 | -3.07078606 | 0.81686363 | -2.62547646 |
| 6 | -2.53496295 | 3.22605786 | -3.17447262 |
| 6 | 4.56473650 | 4.10581898 | -2.94268795 |
| 6 | 2.30266000 | 3.07869812 | -3.00803279 |
| 6 | 4.32294014 | 1.71150444 | -2.32705231 |
| 1 | -0.51209261 | 1.52934131 | 3.94014262 |
| 1 | -1.51700824 | 0.81698639 | 2.64226957 |
| 1 | -0.71241468 | 3.67524985 | 2.78537500 |
| 1 | 0.01537498 | 3.85285915 | 1.16999246 |

| | | | |
|---|---|---|---|
| 1 | -5.41022366 | 3.10015448 | -0.59956380 |
| 1 | -3.01533725 | 3.79731154 | 2.88716104 |
| 1 | 2.05573113 | 1.20496930 | 2.49397753 |
| 1 | 1.55482792 | 2.65981590 | 3.38871859 |
| 1 | 4.58072058 | 5.27231192 | -0.84166840 |
| 1 | 2.76447519 | 4.54335146 | 2.97257463 |
| 1 | -4.92300662 | 2.25700547 | -3.94167312 |
| 1 | -5.60937536 | 1.82351163 | -2.36559029 |
| 1 | -5.23559656 | 3.52685424 | -2.74464924 |
| 1 | -2.02487989 | 0.62271243 | -2.36761171 |
| 1 | -3.70900047 | 0.13274561 | -2.04700999 |
| 1 | -3.21022778 | 0.58784169 | -3.69283824 |
| 1 | -2.82663577 | 4.27666399 | -3.03180790 |
| 1 | -1.48410627 | 3.11355666 | -2.88483813 |
| 1 | -2.62648807 | 2.99239045 | -4.24606779 |
| 1 | 4.70881557 | 3.78450339 | -3.98382633 |
| 1 | 5.55768269 | 4.16089109 | -2.47315309 |
| 1 | 4.13387348 | 5.11707982 | -2.96652664 |
| 1 | 1.60385933 | 2.34251958 | -2.59724178 |
| 1 | 1.82215061 | 4.06709307 | -2.97637516 |
| 1 | 2.48803550 | 2.82523094 | -4.06274498 |
| 1 | 5.28178326 | 1.71255423 | -1.78778179 |
| 1 | 3.68576444 | 0.92270785 | -1.91386075 |
| 1 | 4.52831235 | 1.46848656 | -3.38028273 |
| 1 | 0.07721804 | 0.09492967 | 3.03968299 |
| 8 | 1.79224671 | -1.64633883 | 3.51379879 |
| 31 | 0.28935621 | 1.08309447 | 0.09920368 |
| 1 | -5.24774998 | 3.76631933 | 1.76007918 |
| 1 | 4.19449813 | 5.95451009 | 1.48748363 |
| 8 | -4.89267759 | -7.77705318 | 0.25053161 |
| 6 | -5.07570477 | -9.10271827 | 0.80262695 |
| 1 | -4.19185646 | -9.72391049 | 0.61050955 |
| 6 | -3.73408329 | -5.71170963 | 0.13863269 |
| 6 | -2.48046421 | -4.94356938 | 0.55538807 |
| 6 | -2.42356133 | -3.53646819 | -0.04880340 |
| 6 | -3.82901141 | -7.09037736 | 0.76311093 |
| 6 | -1.15657273 | -2.77356628 | 0.35467698 |
| 6 | -1.09660410 | -1.37081005 | -0.24256310 |
| 1 | -3.78909398 | -5.82747148 | -0.95540859 |
| 1 | -1.58809901 | -5.51324877 | 0.25282682 |
| 1 | -2.47393142 | -3.60630573 | -1.14910374 |
| 1 | -0.26720146 | -3.33386960 | 0.02408342 |
| 1 | -4.64610052 | -5.15913770 | 0.42100604 |
| 1 | -2.44251581 | -4.88156883 | 1.65367981 |
| 1 | -3.31524998 | -2.96625238 | 0.26539564 |
| 1 | -1.10408480 | -2.69361082 | 1.45356241 |
| 1 | -1.98629045 | -0.79687376 | 0.08032616 |
| 1 | -1.14123768 | -1.44034752 | -1.34498399 |
| 8 | -3.08301914 | -7.54608964 | 1.60915554 |
| 8 | 0.12006315 | -0.72649037 | 0.15982283 |
| 1 | -5.95412737 | -9.50987928 | 0.29276596 |
| 1 | -5.24511994 | -9.04804538 | 1.88544379 |
| 8 | 2.98187205 | -1.06327192 | 1.73190889 |

| | | | |
|---|---|---|---|
| 6 | 2.32550155 | -1.97702336 | 2.47098383 |
| 6 | 2.31417858 | -3.42749600 | 2.01417919 |
| 8 | 2.48026202 | -3.63357250 | 0.58446013 |
| 6 | 3.14477792 | -2.72595469 | -0.17499115 |
| 6 | 3.58843396 | -1.40928447 | 0.44516319 |
| 8 | 3.38112119 | -2.96574976 | -1.33843697 |
| 6 | 5.10828465 | -1.32915352 | 0.56436665 |
| 6 | 3.34449187 | -4.25267657 | 2.78727274 |
| 1 | 1.30593358 | -3.80457516 | 2.22893312 |
| 1 | 3.22542415 | -0.62216395 | -0.23058760 |
| 1 | 5.39496530 | -0.33878465 | 0.93867503 |
| 1 | 5.55352846 | -1.47573817 | -0.42716896 |
| 1 | 5.50312237 | -2.09435610 | 1.24647306 |
| 1 | 3.13291432 | -4.19038307 | 3.86214618 |
| 1 | 4.36613809 | -3.89265314 | 2.60647025 |
| 1 | 3.27936475 | -5.30091801 | 2.47070561 |

Electronic energy = -4057.850699 a.u.
DFT-D3(BJ) dispersion correction = -0.136517 a.u.
Thermal free energy = 0.716196 a.u.
Gibbs free energy = -4057.271020 a.u.
Number of imaginary frequencies = 0.

**Int2_CL_vdW_LA isomer/conformer 11**

| Atomic N. | X | Y | Z |
|---|---|---|---|
| 7 | 1.96011182 | 1.56908189 | 0.16123267 |
| 8 | -0.61444921 | 2.62490991 | 1.30835909 |
| 8 | -0.58925935 | 1.13214681 | -1.37698953 |
| 6 | 2.85287545 | 0.73414936 | 1.00786700 |
| 6 | 0.21586200 | 3.24140438 | 2.19182519 |
| 6 | -0.30129242 | 3.77799236 | 3.40677618 |
| 6 | 0.61497540 | 4.37286357 | 4.28677739 |
| 6 | 1.98087012 | 4.47146928 | 4.00842537 |
| 6 | 2.46325982 | 3.99313927 | 2.79487828 |
| 6 | 1.59241993 | 3.39610468 | 1.87690260 |
| 6 | 2.09561070 | 3.02986376 | 0.50487727 |
| 6 | -1.80769114 | 3.74113928 | 3.73381829 |
| 6 | 0.01057034 | 1.94744301 | -2.28316478 |
| 6 | -0.77176339 | 2.61179806 | -3.27234520 |
| 6 | -0.09154180 | 3.42481140 | -4.19038801 |
| 6 | 1.29455453 | 3.60112109 | -4.16486459 |
| 6 | 2.04655806 | 2.94049680 | -3.19992441 |
| 6 | 1.42144483 | 2.10523257 | -2.26647374 |
| 6 | 2.27247029 | 1.32206134 | -1.29938296 |
| 6 | -2.30351597 | 2.44857204 | -3.33699328 |
| 6 | -2.12148247 | 4.41663134 | 5.08206298 |
| 6 | -2.31236493 | 2.28311311 | 3.82180949 |
| 6 | -2.59512416 | 4.50178992 | 2.64001767 |
| 6 | -2.91935350 | 3.24607873 | -4.50225031 |
| 6 | -2.95222521 | 2.96870809 | -2.03261449 |
| 6 | -2.66988056 | 0.96110032 | -3.55453846 |
| 1 | 3.90260311 | 1.00831956 | 0.81717854 |
| 1 | 2.61574750 | 0.90492817 | 2.06337551 |

| | | | |
|---|---|---|---|
| 1 | 3.15755065 | 3.30879711 | 0.40318617 |
| 1 | 1.52880653 | 3.57258211 | -0.26610532 |
| 1 | 0.25321683 | 4.77697981 | 5.23044515 |
| 1 | 3.51976013 | 4.09805317 | 2.53784339 |
| 1 | 2.15636912 | 0.23772578 | -1.44681638 |
| 1 | 3.33582881 | 1.56157221 | -1.46169158 |
| 1 | -0.66196119 | 3.94967692 | -4.95447379 |
| 1 | 3.13247337 | 3.05434748 | -3.17000967 |
| 1 | -3.20623204 | 4.37843228 | 5.25614250 |
| 1 | -1.63322621 | 3.90684873 | 5.92539270 |
| 1 | -1.82211356 | 5.47443980 | 5.09411299 |
| 1 | -2.19808087 | 1.76702334 | 2.86300600 |
| 1 | -1.76044868 | 1.72336554 | 4.59115196 |
| 1 | -3.37870371 | 2.27299319 | 4.09411744 |
| 1 | -2.28084419 | 5.55475824 | 2.59940777 |
| 1 | -2.44324977 | 4.05491484 | 1.65107228 |
| 1 | -3.67100594 | 4.47899848 | 2.87148033 |
| 1 | -4.00711483 | 3.08701546 | -4.50732429 |
| 1 | -2.53270483 | 2.91859153 | -5.47816790 |
| 1 | -2.74474794 | 4.32713685 | -4.40294524 |
| 1 | -2.60125650 | 2.41447394 | -1.15590989 |
| 1 | -2.72200564 | 4.03325529 | -1.88206858 |
| 1 | -4.04623040 | 2.86420814 | -2.09417985 |
| 1 | -2.25164281 | 0.59461909 | -4.50350550 |
| 1 | -2.29117531 | 0.33207796 | -2.74185977 |
| 1 | -3.76408190 | 0.85077806 | -3.60262197 |
| 1 | 2.70259305 | -0.32210056 | 0.76319313 |
| 8 | 3.70487044 | -1.92996406 | -1.39666857 |
| 31 | -0.00622195 | 1.11540464 | 0.39056323 |
| 1 | 2.65392526 | 4.93557341 | 4.72924068 |
| 1 | 1.77709568 | 4.24950896 | -4.89610492 |
| 8 | -2.18579512 | -6.58106352 | -1.34286578 |
| 6 | -1.76141272 | -7.94173991 | -1.07782147 |
| 1 | -0.94257938 | -8.22408334 | -1.75093691 |
| 6 | -1.74674648 | -4.24223264 | -1.41833930 |
| 6 | -1.87614750 | -3.44763620 | -0.10338517 |
| 6 | -2.24830959 | -1.98367868 | -0.35808067 |
| 6 | -1.22288542 | -5.63705982 | -1.16613662 |
| 6 | -2.45870305 | -1.16001763 | 0.92048352 |
| 6 | -1.22776375 | -1.04590703 | 1.82568262 |
| 1 | -1.02348555 | -3.73487709 | -2.07455810 |
| 1 | -0.91866500 | -3.50534045 | 0.43418790 |
| 1 | -1.45879284 | -1.51172534 | -0.96355680 |
| 1 | -3.27904132 | -1.59305682 | 1.51998183 |
| 1 | -2.71325647 | -4.29062638 | -1.93667198 |
| 1 | -2.63595999 | -3.92566179 | 0.53736799 |
| 1 | -3.17024553 | -1.94088466 | -0.96108746 |
| 1 | -2.78999811 | -0.14434123 | 0.63896963 |
| 1 | -0.94616750 | -2.04633481 | 2.19854982 |
| 1 | -1.49144034 | -0.44227719 | 2.71337233 |
| 8 | -0.08254798 | -5.90608701 | -0.81844219 |
| 8 | -0.07031020 | -0.52442853 | 1.17635297 |
| 1 | -2.64538632 | -8.55983711 | -1.26015316 |

| | | | |
|---|---|---|---|
| 1 | -1.42291973 | -8.04100242 | -0.03916392 |
| 8 | 3.70296238 | -2.97173073 | 0.56982105 |
| 6 | 3.82953659 | -2.96737570 | -0.77559556 |
| 6 | 4.15748107 | -4.27255246 | -1.48799140 |
| 8 | 4.75081896 | -5.30266485 | -0.64336689 |
| 6 | 4.43623416 | -5.37844866 | 0.67382846 |
| 6 | 3.64805286 | -4.23681482 | 1.30411690 |
| 8 | 4.79569033 | -6.32529534 | 1.33805038 |
| 6 | 2.19961021 | -4.62356142 | 1.59263286 |
| 6 | 2.95611317 | -4.82741192 | -2.25214867 |
| 1 | 4.95675390 | -4.02317281 | -2.19900210 |
| 1 | 4.16217914 | -4.01082873 | 2.24757074 |
| 1 | 1.69557943 | -3.78441418 | 2.08889944 |
| 1 | 2.19072063 | -5.49228533 | 2.26330962 |
| 1 | 1.64774516 | -4.88320548 | 0.68003715 |
| 1 | 2.61057253 | -4.07392610 | -2.97179954 |
| 1 | 2.12332661 | -5.08965577 | -1.58685631 |
| 1 | 3.26575132 | -5.72531397 | -2.80141147 |

Electronic energy = -4057.849604 a.u.
DFT-D3(BJ) dispersion correction = -0.134605 a.u.
Thermal free energy = 0.713513 a.u.
Gibbs free energy = -4057.270696 a.u.
Number of imaginary frequencies = 0.

**Int2 CL vdW LA isomer/conformer 12**

| Atomic N. | X | Y | Z |
|---|---|---|---|
| 7 | -1.63583798 | 1.12123631 | 1.22515202 |
| 8 | -1.98088861 | -1.68179561 | 0.25636667 |
| 8 | -1.15930006 | 0.56420472 | -1.70607406 |
| 6 | -0.74656774 | 1.44082121 | 2.37287490 |
| 6 | -2.40295938 | -1.90889267 | 1.52670213 |
| 6 | -2.45735934 | -3.23674624 | 2.04058565 |
| 6 | -2.88677457 | -3.39091047 | 3.36737739 |
| 6 | -3.26083247 | -2.31225165 | 4.17498576 |
| 6 | -3.24013266 | -1.02480042 | 3.64674005 |
| 6 | -2.83199071 | -0.81589973 | 2.32505281 |
| 6 | -2.94762748 | 0.55101275 | 1.70318600 |
| 6 | -2.07885652 | -4.45404189 | 1.17383088 |
| 6 | -2.29177283 | 1.30079660 | -1.86440558 |
| 6 | -3.03470021 | 1.21765191 | -3.07670261 |
| 6 | -4.18600708 | 2.01207735 | -3.17777381 |
| 6 | -4.61544456 | 2.85976242 | -2.15279641 |
| 6 | -3.87266588 | 2.94151447 | -0.98053125 |
| 6 | -2.70762052 | 2.17972719 | -0.83072275 |
| 6 | -1.85737340 | 2.36866255 | 0.39944596 |
| 6 | -2.59753141 | 0.29592828 | -4.23248473 |
| 6 | -2.27859709 | -5.78144709 | 1.92922229 |
| 6 | -0.59091882 | -4.37998211 | 0.75767715 |
| 6 | -2.97722907 | -4.50152398 | -0.08548573 |
| 6 | -3.54128130 | 0.40763292 | -5.44486779 |
| 6 | -2.61329409 | -1.18121887 | -3.77322813 |
| 6 | -1.18034546 | 0.68589386 | -4.71635915 |

| | | | |
|---|---|---|---|
| 1 | -1.19854763 | 2.23871984 | 2.98331576 |
| 1 | -0.61094989 | 0.54828509 | 2.99130040 |
| 1 | -3.38900032 | 1.26234840 | 2.42091865 |
| 1 | -3.59849617 | 0.51196707 | 0.81746531 |
| 1 | -2.92990148 | -4.39121045 | 3.79457476 |
| 1 | -3.56151545 | -0.17254272 | 4.24958883 |
| 1 | -0.85000820 | 2.72069795 | 0.12605126 |
| 1 | -2.31172064 | 3.13106151 | 1.05332456 |
| 1 | -4.77942110 | 1.96690241 | -4.08903003 |
| 1 | -4.18200385 | 3.60991444 | -0.17397265 |
| 1 | -2.01013989 | -6.61430752 | 1.26400175 |
| 1 | -1.63870832 | -5.85326029 | 2.82078462 |
| 1 | -3.32369076 | -5.92822825 | 2.23792261 |
| 1 | -0.36641694 | -3.46346203 | 0.20163385 |
| 1 | 0.06151308 | -4.41909187 | 1.64223347 |
| 1 | -0.34154945 | -5.24164752 | 0.11997188 |
| 1 | -4.03587349 | -4.59298863 | 0.19801501 |
| 1 | -2.85784978 | -3.60353273 | -0.70118411 |
| 1 | -2.71418502 | -5.37961172 | -0.69458138 |
| 1 | -3.17819969 | -0.25708735 | -6.24147335 |
| 1 | -3.57114865 | 1.42797627 | -5.85365315 |
| 1 | -4.56813671 | 0.09906647 | -5.20118999 |
| 1 | -1.91869592 | -1.35780893 | -2.94528308 |
| 1 | -3.62096444 | -1.47637184 | -3.44729036 |
| 1 | -2.32355262 | -1.83329126 | -4.61113025 |
| 1 | -1.16909408 | 1.72137349 | -5.08680834 |
| 1 | -0.44181886 | 0.59556142 | -3.91242675 |
| 1 | -0.87738767 | 0.02792237 | -5.54497643 |
| 1 | 0.23314781 | 1.76854085 | 2.01021652 |
| 8 | 2.46704675 | 1.63951939 | 3.51446658 |
| 31 | -0.83359021 | -0.25088778 | -0.06804661 |
| 1 | -3.57955386 | -2.48420906 | 5.20300696 |
| 1 | -5.52187248 | 3.45200751 | -2.27691476 |
| 8 | 6.18869962 | 5.01702848 | -3.77843073 |
| 6 | 6.58235691 | 6.40237637 | -3.92645907 |
| 1 | 5.73788449 | 7.00549577 | -4.28303763 |
| 6 | 4.83240544 | 3.33175217 | -2.80960140 |
| 6 | 3.70864322 | 3.02148940 | -1.82154519 |
| 6 | 3.37739137 | 1.52650193 | -1.76164719 |
| 6 | 5.17246073 | 4.80756970 | -2.88999254 |
| 6 | 2.24934691 | 1.20461632 | -0.77447919 |
| 6 | 1.90572280 | -0.28503097 | -0.73480576 |
| 1 | 4.57190246 | 2.99243187 | -3.82560320 |
| 1 | 2.81018513 | 3.59093706 | -2.10449539 |
| 1 | 3.09376538 | 1.17379733 | -2.76847376 |
| 1 | 1.34543996 | 1.76901932 | -1.06137662 |
| 1 | 5.75505246 | 2.78826230 | -2.54790634 |
| 1 | 3.99232368 | 3.38310998 | -0.82126069 |
| 1 | 4.28347604 | 0.96023746 | -1.48220548 |
| 1 | 2.52885624 | 1.53570902 | 0.23953040 |
| 1 | 2.81055487 | -0.86580085 | -0.48100192 |
| 1 | 1.59216713 | -0.61417536 | -1.74341890 |
| 8 | 4.63408975 | 5.70142917 | -2.26510452 |

| | | | |
|---|---|---|---|
| 8 | 0.92043934 | -0.61806571 | 0.25118521 |
| 1 | 7.39232411 | 6.39603752 | -4.66216772 |
| 1 | 6.93065051 | 6.80722633 | -2.96789591 |
| 8 | 1.59543076 | -0.09157307 | 4.61460768 |
| 6 | 2.61723101 | 0.52832111 | 3.97979468 |
| 6 | 3.96160555 | -0.18276201 | 3.88797398 |
| 8 | 4.16418506 | -1.25104748 | 4.85779367 |
| 6 | 3.11029432 | -1.98470797 | 5.29677477 |
| 6 | 1.70053639 | -1.50712155 | 4.97007289 |
| 8 | 3.29100635 | -2.98463890 | 5.95544263 |
| 6 | 1.02298281 | -2.37354580 | 3.91101681 |
| 6 | 4.25098373 | -0.68217927 | 2.47293669 |
| 1 | 4.71062038 | 0.57127390 | 4.16500375 |
| 1 | 1.14279698 | -1.58193589 | 5.91293580 |
| 1 | -0.03274136 | -2.09011833 | 3.81927933 |
| 1 | 1.07654419 | -3.42506628 | 4.22042271 |
| 1 | 1.49857251 | -2.26430428 | 2.92776987 |
| 1 | 4.24317436 | 0.16956742 | 1.78142679 |
| 1 | 3.50383075 | -1.41171800 | 2.13604312 |
| 1 | 5.24300279 | -1.15013457 | 2.45229919 |

Electronic energy = -4057.847956 a.u.
DFT-D3(BJ) dispersion correction = -0.135580 a.u.
Thermal free energy = 0.714892 a.u.
Gibbs free energy = -4057.268643 a.u.
Number of imaginary frequencies = 0.

**Int2 CL vdW LA isomer/conformer 13**

| Atomic N. | X | Y | Z |
|---|---|---|---|
| 7 | -2.00557031 | -1.14603636 | 2.06303871 |
| 8 | -1.90039149 | -0.84665999 | -0.91808621 |
| 8 | -1.92130418 | 1.64599691 | 0.93528080 |
| 6 | -1.01463500 | -2.00286084 | 2.76558602 |
| 6 | -2.10925570 | -2.18961816 | -0.91220876 |
| 6 | -1.87835169 | -2.95522311 | -2.09204460 |
| 6 | -2.07161251 | -4.34186533 | -2.00263713 |
| 6 | -2.49590490 | -4.97584576 | -0.83141608 |
| 6 | -2.77922707 | -4.20813743 | 0.29343744 |
| 6 | -2.60869786 | -2.81991393 | 0.25823170 |
| 6 | -3.08019989 | -1.97775966 | 1.41425079 |
| 6 | -1.47051691 | -2.29106321 | -3.42273121 |
| 6 | -3.19107135 | 1.70473909 | 1.42352664 |
| 6 | -4.09825635 | 2.68827871 | 0.93540597 |
| 6 | -5.38398020 | 2.70770890 | 1.49605906 |
| 6 | -5.79180881 | 1.81220300 | 2.48829334 |
| 6 | -4.89364560 | 0.86059620 | 2.95828824 |
| 6 | -3.59241934 | 0.80500316 | 2.44486467 |
| 6 | -2.60294609 | -0.16129915 | 3.04515642 |
| 6 | -3.69613095 | 3.68909157 | -0.16675223 |
| 6 | -1.33807571 | -3.31964551 | -4.56160756 |
| 6 | -0.10564345 | -1.57858074 | -3.28574139 |
| 6 | -2.55292823 | -1.26897965 | -3.84593830 |
| 6 | -4.83897953 | 4.66732658 | -0.49891881 |

| | | | |
|---|---|---|---|
| 6 | -3.34497485 | 2.93339801 | -1.47007841 |
| 6 | -2.48643818 | 4.53646723 | 0.29391327 |
| 1 | -1.51381924 | -2.55771846 | 3.57632268 |
| 1 | -0.58343925 | -2.71847238 | 2.05648487 |
| 1 | -3.53443436 | -2.61693218 | 2.18995226 |
| 1 | -3.84501397 | -1.26358233 | 1.07464538 |
| 1 | -1.88664390 | -4.95737696 | -2.88105689 |
| 1 | -3.15938243 | -4.67708382 | 1.20387066 |
| 1 | -1.74608009 | 0.36874578 | 3.48759701 |
| 1 | -3.09026737 | -0.74044608 | 3.84700835 |
| 1 | -6.10274258 | 3.44440455 | 1.14240875 |
| 1 | -5.18914534 | 0.15882955 | 3.74136637 |
| 1 | -1.06711050 | -2.79326113 | -5.48777805 |
| 1 | -0.55179767 | -4.06224714 | -4.36174720 |
| 1 | -2.28103920 | -3.85308702 | -4.74858090 |
| 1 | -0.14398631 | -0.78042917 | -2.53678474 |
| 1 | 0.67932994 | -2.29492044 | -3.00094497 |
| 1 | 0.17911222 | -1.13174034 | -4.25038176 |
| 1 | -3.52115570 | -1.76937805 | -3.99188881 |
| 1 | -2.67754173 | -0.48041479 | -3.09555387 |
| 1 | -2.26822870 | -0.79968544 | -4.79987634 |
| 1 | -4.49755197 | 5.36198483 | -1.27935277 |
| 1 | -5.13567478 | 5.26886452 | 0.37247118 |
| 1 | -5.72908640 | 4.15005563 | -0.88503890 |
| 1 | -2.49507861 | 2.25684159 | -1.33173222 |
| 1 | -4.20377148 | 2.34323727 | -1.82081091 |
| 1 | -3.08458013 | 3.65518931 | -2.25902485 |
| 1 | -2.73302425 | 5.10094491 | 1.20524647 |
| 1 | -1.61165170 | 3.90905561 | 0.49511674 |
| 1 | -2.22121410 | 5.26147343 | -0.49044425 |
| 1 | -0.22555974 | -1.37169082 | 3.19065026 |
| 8 | 0.41133382 | 0.72384323 | 4.43123907 |
| 31 | -1.16428228 | -0.00731955 | 0.57904447 |
| 1 | -2.62364598 | -6.05805355 | -0.81114747 |
| 1 | -6.80472610 | 1.86305746 | 2.88750624 |
| 8 | 7.58708732 | -2.65213529 | -3.70117564 |
| 6 | 8.75577448 | -2.27181286 | -4.46674227 |
| 1 | 8.46730177 | -1.65153666 | -5.32458320 |
| 6 | 5.69694758 | -2.10330781 | -2.37961036 |
| 6 | 4.73406542 | -0.99284429 | -1.95987958 |
| 6 | 3.59211529 | -1.51100864 | -1.07932582 |
| 6 | 6.87881597 | -1.60300437 | -3.18775281 |
| 6 | 2.60730717 | -0.41100529 | -0.66839864 |
| 6 | 1.49234065 | -0.92827877 | 0.23841443 |
| 1 | 5.18375005 | -2.88257677 | -2.96430031 |
| 1 | 4.32098182 | -0.50944294 | -2.85886239 |
| 1 | 3.04818962 | -2.30865502 | -1.61461223 |
| 1 | 2.15551242 | 0.04513162 | -1.56369128 |
| 1 | 6.10482328 | -2.62122006 | -1.49459167 |
| 1 | 5.29452818 | -0.20972884 | -1.42734118 |
| 1 | 4.01503534 | -1.98359737 | -0.17495350 |
| 1 | 3.14399646 | 0.39596189 | -0.14335748 |
| 1 | 1.93828024 | -1.40558520 | 1.13292376 |

| | | | |
|---|---|---|---|
| 1 | 0.93130231 | -1.71702526 | -0.29779545 |
| 8 | 7.18510613 | -0.43886332 | -3.36190875 |
| 8 | 0.64359502 | 0.14505374 | 0.66071596 |
| 1 | 9.20161455 | -3.21342839 | -4.80125861 |
| 1 | 9.45928291 | -1.70927565 | -3.84003722 |
| 8 | 2.39413574 | 3.72801256 | 4.50502421 |
| 6 | 3.64345251 | 3.21657497 | 4.39360549 |
| 6 | 3.79957073 | 1.73094643 | 4.08718935 |
| 8 | 2.62564113 | 0.91037164 | 4.37920701 |
| 6 | 1.38601039 | 1.44033801 | 4.30515971 |
| 6 | 1.23813454 | 2.93968989 | 4.08632775 |
| 8 | 4.61640576 | 3.91874744 | 4.55999180 |
| 6 | 0.83860966 | 3.28163604 | 2.65275356 |
| 6 | 4.28137030 | 1.48287119 | 2.65984264 |
| 1 | 4.55586646 | 1.36541095 | 4.79447012 |
| 1 | 0.44213166 | 3.26069211 | 4.77104306 |
| 1 | 0.69525230 | 4.36633506 | 2.57107444 |
| 1 | -0.10215076 | 2.77702532 | 2.39649788 |
| 1 | 1.59780327 | 2.96541918 | 1.92698014 |
| 1 | 5.21923934 | 2.02836560 | 2.49601471 |
| 1 | 3.54293995 | 1.81542096 | 1.91989164 |
| 1 | 4.46269905 | 0.41031964 | 2.51709252 |

Electronic energy = -4057.849086 a.u.
DFT-D3(BJ) dispersion correction = -0.134914 a.u.
Thermal free energy = 0.715661 a.u.
Gibbs free energy = -4057.268339 a.u.
Number of imaginary frequencies = 0.

**Int2 CL vdW LA isomer/conformer 14**

| Atomic N. | X | Y | Z |
|---|---|---|---|
| 8 | 3.10201918 | -2.98505075 | -2.60970617 |
| 6 | 3.27357860 | -2.17964816 | -3.69709668 |
| 6 | 3.29081988 | -0.68490103 | -3.37610493 |
| 8 | 4.11088118 | -0.44454582 | -2.18498079 |
| 6 | 2.81764533 | -2.32192137 | -1.33677992 |
| 8 | 3.39925538 | -2.65372462 | -4.80323244 |
| 6 | 2.82772838 | -3.37956105 | -0.25333986 |
| 6 | 3.85713009 | 0.15425804 | -4.50151155 |
| 1 | 2.26053648 | -0.36604742 | -3.13416768 |
| 1 | 1.82059935 | -1.85288903 | -1.41066758 |
| 1 | 2.07601090 | -4.14672684 | -0.47563376 |
| 1 | 2.59429459 | -2.91596055 | 0.71231133 |
| 1 | 3.81572071 | -3.85019054 | -0.18102282 |
| 1 | 3.25046884 | 0.01047125 | -5.40326587 |
| 1 | 4.88750850 | -0.14438149 | -4.72951208 |
| 1 | 3.83923062 | 1.21426752 | -4.21998781 |
| 7 | -1.84052892 | 2.12714945 | -1.43020219 |
| 8 | -1.50434660 | -0.27615289 | 0.33685425 |
| 8 | -0.90851407 | -0.46964473 | -2.65427774 |
| 6 | -1.20192986 | 3.43158384 | -1.12904325 |
| 6 | -2.08922574 | 0.50878066 | 1.28210117 |
| 6 | -2.06292120 | 0.12437697 | 2.65317867 |

| | | | |
|---|---|---|---|
| 6 | -2.61641327 | 1.02501407 | 3.57626426 |
| 6 | -3.21375426 | 2.23115132 | 3.19918156 |
| 6 | -3.31690343 | 2.54651442 | 1.84830366 |
| 6 | -2.77831350 | 1.68561048 | 0.88548045 |
| 6 | -3.06426522 | 1.91861517 | -0.57443028 |
| 6 | -1.51533840 | -1.24507298 | 3.10588864 |
| 6 | -2.13329913 | -0.45008472 | -3.24045913 |
| 6 | -2.69217021 | -1.64419662 | -3.78030827 |
| 6 | -3.95894659 | -1.54592076 | -4.37332427 |
| 6 | -4.66980897 | -0.34461310 | -4.45404361 |
| 6 | -4.10383560 | 0.81745486 | -3.94301893 |
| 6 | -2.83652620 | 0.77818314 | -3.34843160 |
| 6 | -2.18933833 | 2.06336323 | -2.90352936 |
| 6 | -1.93690275 | -2.98658892 | -3.72661890 |
| 6 | -1.67504675 | -1.45220580 | 4.62391588 |
| 6 | -0.01213420 | -1.38817860 | 2.78059529 |
| 6 | -2.31117674 | -2.36765042 | 2.39666384 |
| 6 | -2.73097511 | -4.12209309 | -4.39934358 |
| 6 | -1.69162739 | -3.40710185 | -2.25819434 |
| 6 | -0.58978694 | -2.86039123 | -4.47674326 |
| 1 | -1.89135989 | 4.25390560 | -1.37988454 |
| 1 | -0.95851375 | 3.48338095 | -0.06165804 |
| 1 | -3.72406954 | 2.79333541 | -0.70033477 |
| 1 | -3.57621845 | 1.04413566 | -1.00201314 |
| 1 | -2.58431259 | 0.77771778 | 4.63556267 |
| 1 | -3.84050879 | 3.44990802 | 1.52700295 |
| 1 | -1.23754994 | 2.22314065 | -3.43507382 |
| 1 | -2.85324801 | 2.91383922 | -3.13052227 |
| 1 | -4.41721395 | -2.44232019 | -4.78696877 |
| 1 | -4.63384542 | 1.76998445 | -4.01330941 |
| 1 | -1.29706342 | -2.44991445 | 4.88814199 |
| 1 | -1.09944877 | -0.71802050 | 5.20569114 |
| 1 | -2.72693954 | -1.40337977 | 4.94000867 |
| 1 | 0.17716190 | -1.30024301 | 1.70554428 |
| 1 | 0.57554130 | -0.62194273 | 3.30526381 |
| 1 | 0.34468449 | -2.37367228 | 3.11637031 |
| 1 | -3.37888182 | -2.30823334 | 2.65300279 |
| 1 | -2.21010495 | -2.30774738 | 1.30693107 |
| 1 | -1.93952000 | -3.34975623 | 2.72587408 |
| 1 | -2.14137470 | -5.04813241 | -4.34642160 |
| 1 | -2.92663263 | -3.91672196 | -5.46163223 |
| 1 | -3.69087459 | -4.31231916 | -3.89755715 |
| 1 | -1.09059985 | -2.66829180 | -1.71759666 |
| 1 | -2.64427520 | -3.53162845 | -1.72341015 |
| 1 | -1.16065857 | -4.37069280 | -2.23393883 |
| 1 | -0.75702724 | -2.62486893 | -5.53763521 |
| 1 | 0.03634313 | -2.07295763 | -4.04395944 |
| 1 | -0.03822929 | -3.81049782 | -4.42572332 |
| 1 | -0.28485308 | 3.53472341 | -1.72077660 |
| 8 | 4.34208488 | -0.95555756 | -0.01998050 |
| 31 | -0.61844888 | 0.50827754 | -1.10343597 |
| 1 | -3.61989286 | 2.89984900 | 3.95784983 |
| 1 | -5.65503706 | -0.32286961 | -4.91940635 |

| | | | |
|---|---|---|---|
| 8 | 4.27490975 | 3.54559592 | 6.52427116 |
| 6 | 4.19980322 | 3.42738226 | 7.96511105 |
| 1 | 3.23461949 | 3.79927119 | 8.33167591 |
| 6 | 3.46607861 | 3.05017095 | 4.35116427 |
| 6 | 2.56604964 | 2.13287224 | 3.52404614 |
| 6 | 2.68580008 | 2.39373060 | 2.01885113 |
| 6 | 3.31895777 | 2.84383512 | 5.84637098 |
| 6 | 1.79925108 | 1.45987526 | 1.18619723 |
| 6 | 1.90796618 | 1.73782276 | -0.30936277 |
| 1 | 3.24140389 | 4.11056060 | 4.14438300 |
| 1 | 1.52195879 | 2.26178202 | 3.84683896 |
| 1 | 2.42088713 | 3.44531756 | 1.80813725 |
| 1 | 0.74852805 | 1.56377433 | 1.51091492 |
| 1 | 4.52699250 | 2.91781806 | 4.08759933 |
| 1 | 2.82374887 | 1.08405064 | 3.73765182 |
| 1 | 3.73550876 | 2.26765319 | 1.70432387 |
| 1 | 2.09156706 | 0.41540515 | 1.37202610 |
| 1 | 2.96205191 | 1.64483400 | -0.61898472 |
| 1 | 1.60837743 | 2.78443062 | -0.51368359 |
| 8 | 2.47271543 | 2.16377661 | 6.39535976 |
| 8 | 1.17140272 | 0.83323408 | -1.14242678 |
| 1 | 5.02216794 | 4.03874204 | 8.34898449 |
| 1 | 4.31820787 | 2.38027334 | 8.27081276 |
| 6 | 3.82253426 | -1.19598825 | -1.08802745 |

Electronic energy = -4057.849182 a.u.
DFT-D3(BJ) dispersion correction = -0.137827 a.u.
Thermal free energy = 0.718788 a.u.
Gibbs free energy = -4057.268221 a.u.
Number of imaginary frequencies = 0.

**Int2 CL vdW LA isomer/conformer 15**

| Atomic N. | X | Y | Z |
|---|---|---|---|
| 7 | -1.18400492 | 1.26379745 | 1.47667209 |
| 8 | -2.84892261 | 2.29065588 | -0.77087884 |
| 8 | 0.21469074 | 1.85320566 | -1.12929846 |
| 6 | -1.59809533 | 0.02168669 | 2.18015939 |
| 6 | -3.82251435 | 2.32122941 | 0.17655478 |
| 6 | -5.19489633 | 2.36356140 | -0.20288686 |
| 6 | -6.14308133 | 2.40370025 | 0.83013888 |
| 6 | -5.79279332 | 2.40466718 | 2.18351190 |
| 6 | -4.44825176 | 2.38027499 | 2.53952425 |
| 6 | -3.46130330 | 2.35194907 | 1.54862012 |
| 6 | -2.00793027 | 2.44347512 | 1.92760358 |
| 6 | -5.62387573 | 2.36135178 | -1.68310547 |
| 6 | 0.82249107 | 2.82247191 | -0.39648717 |
| 6 | 1.46092771 | 3.91544730 | -1.05123496 |
| 6 | 2.08145304 | 4.87622006 | -0.23981729 |
| 6 | 2.09092722 | 4.79766656 | 1.15559221 |
| 6 | 1.47335072 | 3.72016418 | 1.78069187 |
| 6 | 0.85046255 | 2.72401880 | 1.01936317 |
| 6 | 0.28820407 | 1.51095986 | 1.71588685 |
| 6 | 1.46936133 | 4.04407712 | -2.58751288 |

| | | | |
|---|---|---|---|
| 6 | -7.15324323 | 2.45897521 | -1.83857076 |
| 6 | -5.17418532 | 1.04642205 | -2.36306888 |
| 6 | -5.00918137 | 3.57703180 | -2.41712733 |
| 6 | 2.24877799 | 5.28739441 | -3.05640287 |
| 6 | 0.02302036 | 4.18205205 | -3.11878384 |
| 6 | 2.15208840 | 2.80884899 | -3.22192764 |
| 1 | -1.43983972 | 0.13928916 | 3.26408005 |
| 1 | -2.65823646 | -0.17245557 | 1.98537153 |
| 1 | -1.90201773 | 2.53621257 | 3.02153651 |
| 1 | -1.54372667 | 3.32702245 | 1.46529911 |
| 1 | -7.20069221 | 2.42962497 | 0.57372969 |
| 1 | -4.15145811 | 2.40149947 | 3.59068432 |
| 1 | 0.79986265 | 0.59314054 | 1.39042538 |
| 1 | 0.44252341 | 1.60154406 | 2.80359155 |
| 1 | 2.57234625 | 5.72638142 | -0.70988636 |
| 1 | 1.48061439 | 3.63281324 | 2.86949595 |
| 1 | -7.40255794 | 2.46872240 | -2.90923237 |
| 1 | -7.67226814 | 1.60087027 | -1.38733090 |
| 1 | -7.55588942 | 3.38201138 | -1.39665144 |
| 1 | -4.08716130 | 0.91698429 | -2.32856535 |
| 1 | -5.63885074 | 0.17872377 | -1.87263972 |
| 1 | -5.48929494 | 1.04799817 | -3.41786677 |
| 1 | -5.36372685 | 4.51765690 | -1.97055515 |
| 1 | -3.91497002 | 3.56230713 | -2.37386074 |
| 1 | -5.31825611 | 3.56745579 | -3.47348514 |
| 1 | 2.23425330 | 5.32365381 | -4.15499779 |
| 1 | 3.30160726 | 5.25971628 | -2.74004396 |
| 1 | 1.79897936 | 6.22149719 | -2.69012909 |
| 1 | -0.58636940 | 3.30629537 | -2.87237240 |
| 1 | -0.46337551 | 5.07169915 | -2.69364279 |
| 1 | 0.03829842 | 4.29447004 | -4.21354443 |
| 1 | 3.19648318 | 2.72937414 | -2.88600952 |
| 1 | 1.62981568 | 1.88252978 | -2.95931741 |
| 1 | 2.15676639 | 2.90936175 | -4.31799343 |
| 1 | -0.99628575 | -0.81906201 | 1.81899995 |
| 8 | 1.63090142 | -1.57435276 | 2.69096127 |
| 31 | -1.40418277 | 1.13818570 | -0.55768740 |
| 1 | -6.56867653 | 2.43049054 | 2.94867801 |
| 1 | 2.57928202 | 5.57457512 | 1.74376452 |
| 8 | 4.94809596 | -6.05988511 | -1.81398857 |
| 6 | 5.92222668 | -6.86449825 | -1.10523884 |
| 1 | 6.57373820 | -6.22735515 | -0.49436538 |
| 6 | 3.13974549 | -4.53156516 | -1.85910036 |
| 6 | 2.15722940 | -3.70121211 | -1.03407873 |
| 6 | 1.19391022 | -2.88970588 | -1.90668054 |
| 6 | 4.11234650 | -5.33650343 | -1.02171609 |
| 6 | 0.20723174 | -2.05547101 | -1.08230931 |
| 6 | -0.74672089 | -1.23551064 | -1.95182041 |
| 1 | 3.73254117 | -3.89042236 | -2.53208734 |
| 1 | 2.71841522 | -3.02139769 | -0.37483226 |
| 1 | 1.77387460 | -2.22396448 | -2.56920484 |
| 1 | 0.76808188 | -1.37412486 | -0.42026989 |
| 1 | 2.60881720 | -5.23432345 | -2.52190442 |

| | | | |
|---|---|---|---|
| 1 | 1.58371297 | -4.36704285 | -0.37069373 |
| 1 | 0.63409696 | -3.57284884 | -2.56898904 |
| 1 | -0.38977833 | -2.71393227 | -0.43030646 |
| 1 | -1.29937345 | -1.91234836 | -2.62776728 |
| 1 | -0.15995838 | -0.54970899 | -2.59145792 |
| 8 | 4.16710928 | -5.35538421 | 0.19768367 |
| 8 | -1.72739244 | -0.53353204 | -1.18425932 |
| 1 | 6.49757078 | -7.37278339 | -1.88452255 |
| 1 | 5.41916536 | -7.59204591 | -0.45629549 |
| 8 | 1.12932512 | -3.60432548 | 3.45185661 |
| 6 | 1.98794006 | -2.57709982 | 3.27889219 |
| 6 | 3.39470884 | -2.68596279 | 3.85228385 |
| 8 | 3.55269697 | -3.67088541 | 4.91567254 |
| 6 | 2.78661394 | -4.78949758 | 4.93701269 |
| 6 | 1.60066915 | -4.88344825 | 3.98487574 |
| 8 | 3.02809336 | -5.68515316 | 5.71569664 |
| 6 | 1.85045585 | -5.87306437 | 2.84941585 |
| 6 | 4.44394001 | -2.89893457 | 2.76186303 |
| 1 | 3.58165336 | -1.72650062 | 4.35332497 |
| 1 | 0.76042659 | -5.23430254 | 4.59863768 |
| 1 | 0.93708159 | -5.97204617 | 2.24925270 |
| 1 | 2.09912725 | -6.85177792 | 3.27924519 |
| 1 | 2.67089144 | -5.55460329 | 2.19360936 |
| 1 | 4.40150324 | -2.06060520 | 2.05468331 |
| 1 | 4.28384138 | -3.83167078 | 2.20640678 |
| 1 | 5.43897224 | -2.92209728 | 3.22345445 |

Electronic energy = -4057.850379 a.u.
DFT-D3(BJ) dispersion correction = -0.131956 a.u.
Thermal free energy = 0.714264 a.u.
Gibbs free energy = -4057.268072 a.u.
Number of imaginary frequencies = 0.

**Int2 CL vdW LA isomer/conformer 16**

| Atomic N. | X | Y | Z |
|---|---|---|---|
| 7 | -2.95177872 | 1.45163135 | 0.75064488 |
| 8 | -2.08589428 | -1.38282145 | 1.07244623 |
| 8 | -1.55253047 | 0.23062366 | -1.59818055 |
| 6 | -2.61454523 | 2.30094564 | 1.91909578 |
| 6 | -2.97070848 | -1.35454048 | 2.10180508 |
| 6 | -2.89788903 | -2.35269359 | 3.11843055 |
| 6 | -3.83972380 | -2.28819163 | 4.15517188 |
| 6 | -4.82572086 | -1.30057410 | 4.22435969 |
| 6 | -4.89634372 | -0.34262079 | 3.21956813 |
| 6 | -3.99000633 | -0.36790888 | 2.15335234 |
| 6 | -4.16617555 | 0.60530625 | 1.01933894 |
| 6 | -1.82592657 | -3.45980430 | 3.09048173 |
| 6 | -2.69232238 | 0.57254815 | -2.24717975 |
| 6 | -3.01765566 | -0.05585384 | -3.48537653 |
| 6 | -4.19720842 | 0.35551228 | -4.12193035 |
| 6 | -5.04552631 | 1.33346201 | -3.59390375 |
| 6 | -4.71388329 | 1.94211503 | -2.38937040 |
| 6 | -3.53968015 | 1.58002954 | -1.71779833 |

| | | | |
|---|---|---|---|
| 6 | -3.15473276 | 2.32211265 | -0.46533550 |
| 6 | -2.11917888 | -1.14696652 | -4.10011460 |
| 6 | -1.97600870 | -4.43922365 | 4.26990476 |
| 6 | -0.41965869 | -2.82525228 | 3.19421522 |
| 6 | -1.93957548 | -4.28712116 | 1.78785453 |
| 6 | -2.66489362 | -1.64338765 | -5.45244570 |
| 6 | -2.04674394 | -2.36856571 | -3.15368409 |
| 6 | -0.69702993 | -0.59180254 | -4.35254480 |
| 1 | -3.42719867 | 3.02090273 | 2.10918852 |
| 1 | -2.47767856 | 1.66984049 | 2.80369369 |
| 1 | -5.01952237 | 1.27368203 | 1.22471399 |
| 1 | -4.37848968 | 0.07631341 | 0.07816019 |
| 1 | -3.80381536 | -3.03416697 | 4.94680990 |
| 1 | -5.67074333 | 0.42754967 | 3.24169164 |
| 1 | -2.20273364 | 2.86045803 | -0.60389033 |
| 1 | -3.92971696 | 3.06960941 | -0.22598326 |
| 1 | -4.47473513 | -0.10836003 | -5.06658586 |
| 1 | -5.35729215 | 2.71497811 | -1.96274483 |
| 1 | -1.19591893 | -5.21014578 | 4.19457617 |
| 1 | -1.85482741 | -3.94161583 | 5.24296848 |
| 1 | -2.94853067 | -4.95203313 | 4.25935060 |
| 1 | -0.22345906 | -2.15305693 | 2.35181431 |
| 1 | -0.31823921 | -2.25312354 | 4.12794135 |
| 1 | 0.34428941 | -3.61891918 | 3.20258519 |
| 1 | -2.92702137 | -4.76639721 | 1.72028712 |
| 1 | -1.79290430 | -3.66490675 | 0.89865284 |
| 1 | -1.17991157 | -5.08409402 | 1.78796839 |
| 1 | -1.98361451 | -2.40882919 | -5.85002426 |
| 1 | -2.72634816 | -0.83609485 | -6.19690057 |
| 1 | -3.65828862 | -2.10524861 | -5.35585895 |
| 1 | -1.59278123 | -2.11154095 | -2.19163073 |
| 1 | -3.05039769 | -2.78102571 | -2.97407359 |
| 1 | -1.43442415 | -3.15724722 | -3.61624243 |
| 1 | -0.73139298 | 0.26248232 | -5.04501555 |
| 1 | -0.21913643 | -0.27381151 | -3.42041059 |
| 1 | -0.07296257 | -1.37382676 | -4.81058217 |
| 1 | -1.68020549 | 2.83703616 | 1.71773101 |
| 8 | 0.90273714 | -2.03043519 | -0.88455327 |
| 31 | -1.43123688 | 0.14721724 | 0.25176604 |
| 1 | -5.53203022 | -1.28846770 | 5.05430807 |
| 1 | -5.95574080 | 1.61268728 | -4.12442272 |
| 8 | 6.80588465 | 6.20314552 | -0.11945287 |
| 6 | 7.67695374 | 7.16517943 | 0.52278857 |
| 1 | 7.10566225 | 8.04420717 | 0.84682129 |
| 6 | 4.93498403 | 4.74760013 | -0.09262023 |
| 6 | 3.88211989 | 4.06039809 | 0.77632043 |
| 6 | 2.99575041 | 3.10067264 | -0.02459208 |
| 6 | 5.80074912 | 5.72888786 | 0.67423236 |
| 6 | 1.94469767 | 2.39563282 | 0.84004602 |
| 6 | 1.05301794 | 1.45328234 | 0.03481657 |
| 1 | 4.46051565 | 5.30325688 | -0.91902168 |
| 1 | 3.26027081 | 4.82557647 | 1.26508583 |
| 1 | 2.49346010 | 3.65749251 | -0.83497863 |

| | | | |
|---|---|---|---|
| 1 | 1.31178537 | 3.14407863 | 1.34520343 |
| 1 | 5.59810086 | 4.01344168 | -0.57721549 |
| 1 | 4.38247312 | 3.51471396 | 1.59143905 |
| 1 | 3.62848140 | 2.34448829 | -0.52212407 |
| 1 | 2.43944563 | 1.81862871 | 1.63864539 |
| 1 | 1.67857861 | 0.71247958 | -0.49860044 |
| 1 | 0.52035356 | 2.02535116 | -0.74886258 |
| 8 | 5.64245566 | 6.07006533 | 1.83094095 |
| 8 | 0.14264639 | 0.78289488 | 0.91449611 |
| 1 | 8.41433921 | 7.44048860 | -0.23730991 |
| 1 | 8.16703296 | 6.71616014 | 1.39577312 |
| 8 | 3.70096418 | -3.74134200 | 0.62896813 |
| 6 | 4.59777069 | -3.70712008 | -0.38481656 |
| 6 | 4.25849950 | -2.89981347 | -1.63336701 |
| 8 | 2.84317426 | -2.60027025 | -1.81721605 |
| 6 | 2.03229382 | -2.44773793 | -0.74171669 |
| 6 | 2.56052653 | -2.82863902 | 0.63488513 |
| 8 | 5.63852018 | -4.32348625 | -0.31102567 |
| 6 | 2.85595937 | -1.60236464 | 1.49601112 |
| 6 | 5.08991885 | -1.62143688 | -1.73306156 |
| 1 | 4.50269537 | -3.55534856 | -2.47987546 |
| 1 | 1.75787543 | -3.41274535 | 1.10369769 |
| 1 | 3.14778463 | -1.92867040 | 2.50187248 |
| 1 | 1.95283054 | -0.98010680 | 1.56831112 |
| 1 | 3.67076948 | -0.99833431 | 1.07426447 |
| 1 | 6.15592401 | -1.87972595 | -1.70555190 |
| 1 | 4.87253949 | -0.93194315 | -0.90663660 |
| 1 | 4.86780741 | -1.11540561 | -2.68066755 |

Electronic energy = -4057.843848 a.u.
DFT-D3(BJ) dispersion correction = -0.133885 a.u.
Thermal free energy = 0.712055 a.u.
Gibbs free energy = -4057.265678 a.u.
Number of imaginary frequencies = 0.

**Int2 CL vdW LA isomer/conformer 17**

| Atomic N. | X | Y | Z |
|---|---|---|---|
| 7 | 1.22732003 | 2.37968188 | 1.14051952 |
| 8 | -1.70711366 | 2.32586705 | 0.62858812 |
| 8 | 0.32038084 | 1.84459967 | -1.68767150 |
| 6 | 1.80170430 | 1.53385538 | 2.21648918 |
| 6 | -1.77604250 | 2.69742279 | 1.93517054 |
| 6 | -2.98043352 | 2.51637766 | 2.67352777 |
| 6 | -2.97463043 | 2.93038149 | 4.01401724 |
| 6 | -1.85712654 | 3.50284032 | 4.62902776 |
| 6 | -0.69427651 | 3.69283704 | 3.88966870 |
| 6 | -0.64886343 | 3.30678005 | 2.54563035 |
| 6 | 0.56735321 | 3.60997235 | 1.71283949 |
| 6 | -4.23809131 | 1.89718102 | 2.03287261 |
| 6 | 0.88586417 | 3.05753935 | -1.92530128 |
| 6 | 0.54205021 | 3.78815320 | -3.09895811 |
| 6 | 1.17113379 | 5.02694572 | -3.28894946 |
| 6 | 2.10005093 | 5.55332674 | -2.38667537 |

| | | | |
|---|---|---|---|
| 6 | 2.43676482 | 4.82336939 | -1.25263493 |
| 6 | 1.84815196 | 3.57466791 | -1.01924028 |
| 6 | 2.31037035 | 2.74996844 | 0.15413011 |
| 6 | -0.47745485 | 3.24611219 | -4.12011619 |
| 6 | -5.42136481 | 1.85385860 | 3.01840694 |
| 6 | -3.95390555 | 0.44202145 | 1.59151707 |
| 6 | -4.68329164 | 2.74437036 | 0.81675958 |
| 6 | -0.65539813 | 4.20046242 | -5.31626292 |
| 6 | -1.86435642 | 3.08108158 | -3.45505989 |
| 6 | 0.00582169 | 1.88849186 | -4.68382265 |
| 1 | 2.61422333 | 2.07601098 | 2.72691100 |
| 1 | 1.01885507 | 1.28809493 | 2.94222486 |
| 1 | 1.31598565 | 4.15663902 | 2.31051605 |
| 1 | 0.29949673 | 4.23460103 | 0.84832335 |
| 1 | -3.87663982 | 2.79840448 | 4.60878269 |
| 1 | 0.18268497 | 4.16054211 | 4.34329400 |
| 1 | 2.73456912 | 1.79268860 | -0.18929928 |
| 1 | 3.10027162 | 3.29073613 | 0.70146880 |
| 1 | 0.92540917 | 5.61250422 | -4.17288621 |
| 1 | 3.17204040 | 5.20930463 | -0.54305356 |
| 1 | -6.29225772 | 1.41829934 | 2.50855060 |
| 1 | -5.20759810 | 1.22610574 | 3.89542314 |
| 1 | -5.70805354 | 2.85701940 | 3.36635381 |
| 1 | -3.14265304 | 0.38793852 | 0.85749426 |
| 1 | -3.68515054 | -0.18522490 | 2.45334233 |
| 1 | -4.85832975 | 0.01104362 | 1.13623482 |
| 1 | -4.92735343 | 3.77094353 | 1.12746506 |
| 1 | -3.90391841 | 2.78914295 | 0.04842967 |
| 1 | -5.58742602 | 2.30281420 | 0.37087548 |
| 1 | -1.38041687 | 3.76202138 | -6.01649528 |
| 1 | 0.28409409 | 4.35445941 | -5.86658481 |
| 1 | -1.04553927 | 5.18179544 | -5.00988556 |
| 1 | -1.83619833 | 2.36851997 | -2.62411733 |
| 1 | -2.22940087 | 4.04471480 | -3.07174356 |
| 1 | -2.58990589 | 2.71493978 | -4.19708923 |
| 1 | 0.97082954 | 2.00429942 | -5.19854574 |
| 1 | 0.12131580 | 1.14159647 | -3.89098164 |
| 1 | -0.72372098 | 1.51030446 | -5.41591267 |
| 1 | 2.19102039 | 0.60606711 | 1.78191054 |
| 8 | -3.30315429 | -3.16510679 | 3.07672727 |
| 31 | -0.20854781 | 1.40161109 | 0.03690232 |
| 1 | -1.90395494 | 3.80355184 | 5.67570684 |
| 1 | 2.55630468 | 6.52477504 | -2.57621682 |
| 8 | 5.65064472 | -5.89670852 | -2.81317281 |
| 6 | 7.02020613 | -6.35929349 | -2.75472375 |
| 1 | 7.70895644 | -5.56354541 | -3.06586867 |
| 6 | 3.92417171 | -4.41741516 | -2.14225189 |
| 6 | 3.53575122 | -3.24595172 | -1.24133822 |
| 6 | 2.06570583 | -2.84791056 | -1.40750626 |
| 6 | 5.37671417 | -4.82730283 | -2.00684068 |
| 6 | 1.64902407 | -1.71006359 | -0.46887295 |
| 6 | 0.17791436 | -1.32753885 | -0.62245786 |
| 1 | 3.74171901 | -4.17983429 | -3.20342231 |

| | | | |
|---|---|---|---|
| 1 | 4.18925567 | -2.38664603 | -1.45829992 |
| 1 | 1.88110806 | -2.55043785 | -2.45462164 |
| 1 | 2.27713768 | -0.82143680 | -0.66009027 |
| 1 | 3.30141353 | -5.30098002 | -1.92877970 |
| 1 | 3.72885591 | -3.51838811 | -0.19244757 |
| 1 | 1.42891818 | -3.72639646 | -1.21086481 |
| 1 | 1.82312309 | -2.01392711 | 0.57610996 |
| 1 | -0.44625941 | -2.22864225 | -0.49358469 |
| 1 | -0.00375623 | -0.95341481 | -1.64637283 |
| 8 | 6.20878472 | -4.30031884 | -1.29224595 |
| 8 | -0.25405126 | -0.38171839 | 0.36730197 |
| 1 | 7.06980395 | -7.20640201 | -3.44576099 |
| 1 | 7.27691927 | -6.67404894 | -1.73524728 |
| 8 | -0.06586731 | -4.03387311 | 1.97901246 |
| 6 | -0.29022698 | -5.25360838 | 1.42159722 |
| 6 | -1.50187197 | -5.99896075 | 1.98482930 |
| 8 | -2.62991695 | -5.08471746 | 2.14483848 |
| 6 | -2.39123768 | -3.90251210 | 2.78664302 |
| 6 | -0.91748259 | -3.63409285 | 3.10244004 |
| 8 | 0.43665970 | -5.67812310 | 0.55058614 |
| 6 | -0.62898553 | -2.17680460 | 3.39786269 |
| 6 | -1.95763386 | -7.14151752 | 1.10103147 |
| 1 | -1.22557430 | -6.37823681 | 2.98678341 |
| 1 | -0.64676894 | -4.26623589 | 3.96921609 |
| 1 | 0.41999232 | -2.06198751 | 3.69879172 |
| 1 | -1.27732212 | -1.83625567 | 4.21370564 |
| 1 | -0.81837367 | -1.55882618 | 2.50985788 |
| 1 | -1.13981860 | -7.86088435 | 0.97773916 |
| 1 | -2.24154220 | -6.76868792 | 0.10968265 |
| 1 | -2.81758806 | -7.64567762 | 1.55814416 |

Electronic energy = -4057.848712 a.u.
DFT-D3(BJ) dispersion correction = -0.131626 a.u.
Thermal free energy = 0.715493 a.u.
Gibbs free energy = -4057.264845 a.u.
Number of imaginary frequencies = 0.

**Int2 CL vdW LA isomer/conformer 18**

| Atomic N. | X | Y | Z |
|---|---|---|---|
| 7 | -2.10936458 | 1.09257265 | -2.61881265 |
| 8 | -1.67969104 | -0.25327709 | 0.03063419 |
| 8 | -0.99890411 | -1.70833334 | -2.57215946 |
| 6 | -1.57024258 | 2.44417324 | -2.90243000 |
| 6 | -2.35737104 | 0.81508363 | 0.52494326 |
| 6 | -2.36066747 | 1.07758946 | 1.92555337 |
| 6 | -3.01790981 | 2.24153140 | 2.35236057 |
| 6 | -3.68511155 | 3.10445530 | 1.47791941 |
| 6 | -3.75288814 | 2.78380213 | 0.12597256 |
| 6 | -3.11312529 | 1.63567349 | -0.35453111 |
| 6 | -3.35139595 | 1.17985469 | -1.76988979 |
| 6 | -1.72993618 | 0.09994452 | 2.93847758 |
| 6 | -2.19156824 | -2.02106137 | -3.13439665 |
| 6 | -2.66532177 | -3.36519809 | -3.10992154 |

| | | | |
|---|---|---|---|
| 6 | -3.90050341 | -3.61872957 | -3.72300201 |
| 6 | -4.66272564 | -2.62222168 | -4.34096932 |
| 6 | -4.18274207 | -1.31818784 | -4.37077212 |
| 6 | -2.94830529 | -1.01137925 | -3.78490774 |
| 6 | -2.38988129 | 0.37971443 | -3.92580437 |
| 6 | -1.86038644 | -4.49337661 | -2.43582755 |
| 6 | -1.93654642 | 0.56855424 | 4.39126523 |
| 6 | -0.20774116 | -0.04079291 | 2.71457946 |
| 6 | -2.40550374 | -1.28565218 | 2.79607187 |
| 6 | -2.55841392 | -5.85928772 | -2.57762813 |
| 6 | -1.70610375 | -4.20921236 | -0.92320032 |
| 6 | -0.46573727 | -4.61917889 | -3.09485240 |
| 1 | -2.30346159 | 3.02660585 | -3.48399508 |
| 1 | -1.36924983 | 2.96526915 | -1.95944216 |
| 1 | -4.06544765 | 1.85331594 | -2.27328804 |
| 1 | -3.78185634 | 0.16767580 | -1.77278555 |
| 1 | -3.01377739 | 2.48797107 | 3.41248830 |
| 1 | -4.32625408 | 3.40656724 | -0.56474015 |
| 1 | -1.42506283 | 0.35887073 | -4.45771610 |
| 1 | -3.08734985 | 1.00432496 | -4.50853194 |
| 1 | -4.29331942 | -4.63381324 | -3.71493143 |
| 1 | -4.75437853 | -0.52746404 | -4.86207054 |
| 1 | -1.49417791 | -0.17453132 | 5.06965211 |
| 1 | -1.44365865 | 1.53092345 | 4.59155252 |
| 1 | -3.00140181 | 0.65964085 | 4.64968921 |
| 1 | 0.01658613 | -0.42623726 | 1.71463956 |
| 1 | 0.29546842 | 0.92794154 | 2.84532819 |
| 1 | 0.20923977 | -0.74194637 | 3.45293916 |
| 1 | -3.48455681 | -1.21381002 | 2.99612910 |
| 1 | -2.26153973 | -1.70388288 | 1.79352804 |
| 1 | -1.97124631 | -1.98509059 | 3.52606424 |
| 1 | -1.93750309 | -6.62664739 | -2.09417198 |
| 1 | -2.68760653 | -6.15218589 | -3.62991559 |
| 1 | -3.54256291 | -5.87575206 | -2.08715127 |
| 1 | -1.15491235 | -3.28312167 | -0.73375295 |
| 1 | -2.69108816 | -4.13688820 | -0.43942598 |
| 1 | -1.15055481 | -5.02988328 | -0.44630817 |
| 1 | -0.56215983 | -4.86981307 | -4.16176588 |
| 1 | 0.10649403 | -3.69003185 | -3.00282818 |
| 1 | 0.10089573 | -5.42559932 | -2.60574907 |
| 1 | -0.64179629 | 2.35364710 | -3.47821403 |
| 8 | 1.81006829 | -3.32424041 | 0.32600643 |
| 31 | -0.80063503 | -0.13889993 | -1.60958989 |
| 1 | -4.17191685 | 4.00121522 | 1.86093712 |
| 1 | -5.62117246 | -2.87069162 | -4.79654007 |
| 8 | 3.70462829 | 6.36638122 | 3.83214638 |
| 6 | 3.69908340 | 6.83093179 | 5.20286743 |
| 1 | 2.67669191 | 7.06529158 | 5.52529598 |
| 6 | 3.05571975 | 4.81778188 | 2.15816199 |
| 6 | 2.19788737 | 3.59696526 | 1.82867908 |
| 6 | 2.31001227 | 3.18234748 | 0.35793783 |
| 6 | 2.97411157 | 5.23234252 | 3.61415765 |
| 6 | 1.46862298 | 1.94436359 | 0.02536934 |

| | | | |
|---|---|---|---|
| 6 | 1.59669526 | 1.53025852 | -1.43787128 |
| 1 | 2.77135818 | 5.68615215 | 1.54202990 |
| 1 | 1.14742751 | 3.80887950 | 2.08124035 |
| 1 | 2.00083527 | 4.02503252 | -0.28570464 |
| 1 | 0.40927366 | 2.13619317 | 0.27129437 |
| 1 | 4.11628524 | 4.62704981 | 1.92502987 |
| 1 | 2.50144465 | 2.75924393 | 2.47461831 |
| 1 | 3.36629468 | 2.97622114 | 0.11542604 |
| 1 | 1.78573371 | 1.10182421 | 0.65936233 |
| 1 | 2.66409925 | 1.39406368 | -1.67984491 |
| 1 | 1.22967285 | 2.35084949 | -2.08498653 |
| 8 | 2.35756659 | 4.65558347 | 4.49000236 |
| 8 | 0.95849628 | 0.29500877 | -1.77228696 |
| 1 | 4.32257836 | 7.73022757 | 5.20640386 |
| 1 | 4.11667496 | 6.06588094 | 5.86954892 |
| 8 | 3.18759362 | -2.22404058 | 1.68948735 |
| 6 | 2.71507515 | -2.52899292 | 0.45207047 |
| 6 | 3.31024182 | -1.82767063 | -0.76039703 |
| 8 | 4.10580589 | -0.63188814 | -0.47355586 |
| 6 | 4.68714024 | -0.44776198 | 0.73142050 |
| 6 | 4.42055351 | -1.46415728 | 1.83462387 |
| 8 | 5.40598049 | 0.51098955 | 0.92722372 |
| 6 | 5.60877679 | -2.40603929 | 2.03179652 |
| 6 | 4.11027781 | -2.78579822 | -1.64002754 |
| 1 | 2.44960914 | -1.43338813 | -1.32349697 |
| 1 | 4.26627799 | -0.87665697 | 2.74914168 |
| 1 | 5.40184727 | -3.08136414 | 2.87118858 |
| 1 | 6.50701543 | -1.81789259 | 2.25855502 |
| 1 | 5.80018532 | -3.00944768 | 1.13454418 |
| 1 | 3.46292475 | -3.61769694 | -1.94425951 |
| 1 | 4.98522642 | -3.19175464 | -1.11450134 |
| 1 | 4.45225719 | -2.25581232 | -2.53740364 |

Electronic energy = -4057.841971 a.u.
DFT-D3(BJ) dispersion correction = -0.136567 a.u.
Thermal free energy = 0.716537 a.u.
Gibbs free energy = -4057.262001 a.u.
Number of imaginary frequencies = 0.

## Propagation step of axial LA addition to the product of CL addition on initiation step

**TS3 ax LA isomer/conformer 1**

| Atomic N. | X | Y | Z |
|---|---|---|---|
| 7 | 1.03671002 | 1.76562405 | 0.98329365 |
| 8 | -1.87438345 | 1.83115923 | 1.27730286 |
| 8 | -0.55432391 | 1.09864640 | -1.47886825 |
| 6 | 1.74936807 | 1.11620545 | 2.10945439 |
| 6 | -1.62459111 | 2.60515571 | 2.35357332 |
| 6 | -2.65437818 | 2.85960627 | 3.31055236 |
| 6 | -2.30921364 | 3.62570119 | 4.43368292 |
| 6 | -1.03272820 | 4.16222906 | 4.62823582 |

| | | | |
|---|---|---|---|
| 6 | -0.06174520 | 3.97098923 | 3.65088725 |
| 6 | -0.35630640 | 3.21669459 | 2.51066351 |
| 6 | 0.61729401 | 3.15519834 | 1.37266016 |
| 6 | -4.09905100 | 2.36316371 | 3.10258961 |
| 6 | 0.15001331 | 2.12350488 | -2.00622916 |
| 6 | -0.32586029 | 2.75871873 | -3.19314075 |
| 6 | 0.44524524 | 3.80281162 | -3.72111177 |
| 6 | 1.63608718 | 4.23788452 | -3.13211918 |
| 6 | 2.08958769 | 3.61157203 | -1.97748220 |
| 6 | 1.36807108 | 2.55117774 | -1.41414642 |
| 6 | 1.94881546 | 1.84618044 | -0.21265611 |
| 6 | -1.63876963 | 2.31687188 | -3.87052584 |
| 6 | -5.03703690 | 2.82844853 | 4.23221684 |
| 6 | -4.14947271 | 0.81939507 | 3.06340170 |
| 6 | -4.65488338 | 2.93271613 | 1.77507365 |
| 6 | -1.93268847 | 3.13668132 | -5.14123487 |
| 6 | -2.83168745 | 2.51996589 | -2.90615773 |
| 6 | -1.54880047 | 0.83081424 | -4.29337740 |
| 1 | 2.66970444 | 1.67538393 | 2.34778190 |
| 1 | 1.10164940 | 1.11087549 | 2.99360919 |
| 1 | 1.52743745 | 3.73069239 | 1.61685359 |
| 1 | 0.17529035 | 3.60156417 | 0.46986961 |
| 1 | -3.06671095 | 3.81966615 | 5.19096088 |
| 1 | 0.92659527 | 4.42569160 | 3.75162721 |
| 1 | 2.23067141 | 0.81119704 | -0.45312399 |
| 1 | 2.86906505 | 2.36994958 | 0.09951436 |
| 1 | 0.10481117 | 4.30642653 | -4.62397146 |
| 1 | 3.01933908 | 3.93404341 | -1.50304806 |
| 1 | -6.05393600 | 2.46738553 | 4.02197647 |
| 1 | -4.73989105 | 2.42615747 | 5.21186972 |
| 1 | -5.08381748 | 3.92441750 | 4.30618143 |
| 1 | -3.56395531 | 0.42745253 | 2.22478557 |
| 1 | -3.76752257 | 0.39693126 | 4.00556993 |
| 1 | -5.19165134 | 0.48457935 | 2.94446659 |
| 1 | -4.66273928 | 4.03213120 | 1.79930878 |
| 1 | -4.05642080 | 2.60529971 | 0.91769034 |
| 1 | -5.69045210 | 2.58990860 | 1.62775445 |
| 1 | -2.87201762 | 2.77905440 | -5.58638716 |
| 1 | -1.14366174 | 3.02400160 | -5.89901686 |
| 1 | -2.05733347 | 4.20751858 | -4.92441463 |
| 1 | -2.72666240 | 1.91813600 | -1.99760795 |
| 1 | -2.92035890 | 3.57716131 | -2.61663485 |
| 1 | -3.76693845 | 2.22726631 | -3.40725684 |
| 1 | -0.72572708 | 0.68105990 | -5.00776005 |
| 1 | -1.38816953 | 0.17694698 | -3.42966461 |
| 1 | -2.48453999 | 0.53047204 | -4.78926277 |
| 1 | 2.00146103 | 0.08562037 | 1.84002507 |
| 8 | -2.24851489 | -0.64121068 | 0.39087665 |
| 31 | -0.68890762 | 0.70098239 | 0.33553016 |
| 1 | -0.81427449 | 4.74446487 | 5.52337265 |
| 1 | 2.19768977 | 5.06072092 | -3.57418108 |
| 8 | 7.08793068 | -5.87935925 | -1.57136691 |
| 6 | 8.33834839 | -6.44599581 | -1.11133945 |

| | | | |
|---|---|---|---|
| 1 | 9.07391071 | -5.65326786 | -0.92500132 |
| 6 | 5.21763468 | -4.45233345 | -1.27669525 |
| 6 | 4.37275696 | -3.69007540 | -0.25608778 |
| 6 | 3.13216019 | -3.04858112 | -0.88595515 |
| 6 | 6.49758911 | -5.01769829 | -0.69175607 |
| 6 | 2.25683427 | -2.31902194 | 0.14029127 |
| 6 | 1.05808210 | -1.63187385 | -0.51326293 |
| 1 | 5.50746059 | -3.79306984 | -2.11300707 |
| 1 | 4.99469900 | -2.91908765 | 0.22334827 |
| 1 | 3.44599414 | -2.34388518 | -1.67638898 |
| 1 | 2.86846590 | -1.57573748 | 0.67900836 |
| 1 | 4.65039968 | -5.27740717 | -1.73409581 |
| 1 | 4.06561899 | -4.37683678 | 0.54777217 |
| 1 | 2.52950907 | -3.82627678 | -1.38530695 |
| 1 | 1.89328718 | -3.03497338 | 0.89363205 |
| 1 | 0.52109247 | -2.35326099 | -1.15634549 |
| 1 | 1.38489139 | -0.83576179 | -1.20269048 |
| 8 | 6.96509552 | -4.74879980 | 0.39867783 |
| 8 | 0.12643269 | -1.11166704 | 0.44292006 |
| 1 | 8.67286205 | -7.10330963 | -1.91964853 |
| 1 | 8.18416023 | -7.01488638 | -0.18591349 |
| 8 | -0.81356961 | -3.82218695 | 1.41882467 |
| 6 | -0.84075350 | -4.48374319 | 0.23148409 |
| 6 | -1.84864867 | -3.93246436 | -0.77856493 |
| 8 | -1.79364729 | -2.47004795 | -0.82696187 |
| 6 | -1.74930704 | -1.80466557 | 0.35686997 |
| 6 | -1.74663424 | -2.71271849 | 1.58730423 |
| 8 | -0.11574322 | -5.43295336 | 0.03317052 |
| 6 | -1.40735912 | -2.03168511 | 2.89614201 |
| 6 | -1.63544142 | -4.45169640 | -2.18438792 |
| 1 | -2.85897279 | -4.20829105 | -0.42267933 |
| 1 | -2.76994491 | -3.13411021 | 1.64296079 |
| 1 | -1.52852869 | -2.74329829 | 3.72202611 |
| 1 | -2.08085871 | -1.18240774 | 3.05877662 |
| 1 | -0.37050506 | -1.67852521 | 2.87319565 |
| 1 | -1.73250639 | -5.54362488 | -2.18917036 |
| 1 | -0.63258231 | -4.19420862 | -2.54560590 |
| 1 | -2.38499689 | -4.01927042 | -2.85800433 |

Electronic energy = -4057.828238 a.u.
DFT-D3(BJ) dispersion correction = -0.140439 a.u.
Thermal free energy = 0.718852 a.u.
Gibbs free energy = -4057.249825 a.u.
Number of imaginary frequencies = 1.

**TS3 ax LA isomer/conformer 2**

| Atomic N. | X | Y | Z |
|---|---|---|---|
| 8 | 3.74561977 | -2.00161076 | -3.08915949 |
| 6 | 4.64332533 | -2.09460330 | -2.06888461 |
| 6 | 4.35198355 | -1.15899134 | -0.89020914 |
| 8 | 2.92509270 | -1.18371749 | -0.58649343 |
| 6 | 2.76538253 | -0.91410661 | -2.99333858 |
| 8 | 5.57796574 | -2.86030364 | -2.12685132 |

| | | | |
|---|---|---|---|
| 6 | 1.84899306 | -1.04171836 | -4.18878126 |
| 6 | 5.09149933 | -1.55735731 | 0.37096885 |
| 1 | 4.64610243 | -0.13769315 | -1.19554555 |
| 1 | 3.31838155 | 0.03842675 | -3.02436352 |
| 1 | 2.44276190 | -1.01592338 | -5.11082554 |
| 1 | 1.13251615 | -0.21168768 | -4.20422363 |
| 1 | 1.28685331 | -1.98052144 | -4.13862514 |
| 1 | 6.17050028 | -1.56042993 | 0.17781992 |
| 1 | 4.79422283 | -2.56518507 | 0.68399030 |
| 1 | 4.86826515 | -0.84895658 | 1.17835009 |
| 7 | -1.65687323 | 1.55770302 | -1.51630628 |
| 8 | -1.10292935 | -0.73253715 | 0.35549071 |
| 8 | -1.35955441 | -1.07943153 | -2.78645492 |
| 6 | -0.96071374 | 2.84456396 | -1.30130756 |
| 6 | -1.53705716 | 0.13507783 | 1.29756272 |
| 6 | -1.28923595 | -0.12720481 | 2.67793798 |
| 6 | -1.70096493 | 0.85000759 | 3.59629655 |
| 6 | -2.36393046 | 2.02120733 | 3.21612954 |
| 6 | -2.67387462 | 2.22188354 | 1.87486756 |
| 6 | -2.28274703 | 1.28011906 | 0.91662693 |
| 6 | -2.75920248 | 1.40310645 | -0.50356215 |
| 6 | -0.64560151 | -1.44614041 | 3.15217447 |
| 6 | -2.63722277 | -0.87663984 | -3.17437744 |
| 6 | -3.46452975 | -1.97789145 | -3.54696918 |
| 6 | -4.76856375 | -1.68075621 | -3.96848345 |
| 6 | -5.27220917 | -0.37717029 | -4.03015900 |
| 6 | -4.45479155 | 0.68591851 | -3.66333771 |
| 6 | -3.14153075 | 0.44252130 | -3.24635100 |
| 6 | -2.22960091 | 1.58510780 | -2.91516423 |
| 6 | -2.95685554 | -3.43153334 | -3.48735166 |
| 6 | -0.56395233 | -1.52303898 | 4.68851709 |
| 6 | 0.79231632 | -1.59487009 | 2.60597682 |
| 6 | -1.50819921 | -2.63879657 | 2.67266870 |
| 6 | -4.02934265 | -4.43488789 | -3.95144343 |
| 6 | -2.57299685 | -3.80295777 | -2.03543663 |
| 6 | -1.73025227 | -3.60293651 | -4.41380596 |
| 1 | -1.67861879 | 3.67695856 | -1.39658666 |
| 1 | -0.52340150 | 2.87524915 | -0.29867956 |
| 1 | -3.44255853 | 2.26460052 | -0.60213155 |
| 1 | -3.31651855 | 0.50179338 | -0.79614311 |
| 1 | -1.49970984 | 0.69538069 | 4.65466070 |
| 1 | -3.24455595 | 3.09926510 | 1.56125760 |
| 1 | -1.35875618 | 1.60250533 | -3.59061003 |
| 1 | -2.77217031 | 2.53823233 | -3.03816509 |
| 1 | -5.42736435 | -2.49809146 | -4.25616598 |
| 1 | -4.82429218 | 1.71314716 | -3.70545363 |
| 1 | -0.12223243 | -2.48867273 | 4.97276735 |
| 1 | 0.07249159 | -0.73216593 | 5.11130667 |
| 1 | -1.55576575 | -1.46191227 | 5.15929985 |
| 1 | 0.81369239 | -1.60246205 | 1.51137662 |
| 1 | 1.43033707 | -0.77722371 | 2.97277737 |
| 1 | 1.22470248 | -2.54202986 | 2.96298337 |
| 1 | -2.52611732 | -2.57113338 | 3.08356214 |

| | | | |
|---|---|---|---|
| 1 | -1.57228971 | -2.67522979 | 1.57949638 |
| 1 | -1.06341231 | -3.58177209 | 3.02490616 |
| 1 | -3.61351895 | -5.45090437 | -3.89653587 |
| 1 | -4.33812523 | -4.25983763 | -4.99236536 |
| 1 | -4.92456055 | -4.41001415 | -3.31322885 |
| 1 | -1.75551212 | -3.17981863 | -1.65869045 |
| 1 | -3.43661928 | -3.69189715 | -1.36381280 |
| 1 | -2.24665380 | -4.85320807 | -1.99623287 |
| 1 | -1.99498308 | -3.37433195 | -5.45671082 |
| 1 | -0.90990376 | -2.94620752 | -4.10597038 |
| 1 | -1.37660134 | -4.64428949 | -4.37437630 |
| 1 | -0.17675164 | 2.96378732 | -2.05724978 |
| 8 | 0.93491507 | -1.65924096 | -1.51081634 |
| 31 | -0.48483506 | -0.26065871 | -1.34140480 |
| 1 | -2.65624285 | 2.75251842 | 3.96965289 |
| 1 | -6.29614067 | -0.20223057 | -4.36021996 |
| 8 | 3.53891659 | 5.00502729 | 5.69315434 |
| 6 | 3.49816012 | 5.04322290 | 7.14081621 |
| 1 | 2.46564102 | 4.94298935 | 7.49790049 |
| 6 | 3.17293549 | 3.89576149 | 3.63033962 |
| 6 | 2.44993591 | 2.74052858 | 2.93866324 |
| 6 | 2.62847710 | 2.76319480 | 1.41651654 |
| 6 | 3.05772567 | 3.85205340 | 5.14261532 |
| 6 | 1.87635756 | 1.62229633 | 0.72116899 |
| 6 | 2.06168365 | 1.65316820 | -0.79364353 |
| 1 | 2.79838252 | 4.87189817 | 3.28453946 |
| 1 | 1.37800884 | 2.77844048 | 3.18644166 |
| 1 | 2.27991486 | 3.73319721 | 1.01925194 |
| 1 | 0.80407310 | 1.69130170 | 0.96581829 |
| 1 | 4.24892759 | 3.88915443 | 3.38562346 |
| 1 | 2.81871009 | 1.78688347 | 3.34455490 |
| 1 | 3.70421910 | 2.70270300 | 1.17273605 |
| 1 | 2.21700168 | 0.65178019 | 1.10707498 |
| 1 | 3.12988758 | 1.52330685 | -1.04054022 |
| 1 | 1.77754748 | 2.63996768 | -1.19617057 |
| 8 | 2.61435604 | 2.92900252 | 5.79857302 |
| 8 | 1.34019709 | 0.64344436 | -1.52069676 |
| 1 | 3.91149330 | 6.01822996 | 7.41595888 |
| 1 | 4.10293007 | 4.23092508 | 7.56314564 |
| 6 | 2.04753923 | -1.03462839 | -1.63390529 |

Electronic energy = -4057.825299 a.u.
DFT-D3(BJ) dispersion correction = -0.143094 a.u.
Thermal free energy = 0.720028 a.u.
Gibbs free energy = -4057.248364 a.u.
Number of imaginary frequencies = 1.

**TS3 ax LA isomer/conformer 3**

| Atomic N. | X | Y | Z |
|---|---|---|---|
| 7 | -2.95332575 | -0.55388999 | 2.04437733 |
| 8 | -0.86108375 | -1.58958566 | 0.29108432 |
| 8 | -1.42076409 | 1.59740543 | 0.78452623 |
| 6 | -3.06550217 | -1.59054875 | 3.09918070 |

| | | | |
|---|---|---|---|
| 6 | -1.78405190 | -2.54713678 | 0.03678259 |
| 6 | -1.36092424 | -3.81032991 | -0.48414889 |
| 6 | -2.34882998 | -4.76714993 | -0.75447762 |
| 6 | -3.70777726 | -4.53972101 | -0.52537531 |
| 6 | -4.10670376 | -3.31573153 | -0.00206357 |
| 6 | -3.16417170 | -2.31865549 | 0.27265635 |
| 6 | -3.64740705 | -0.99042314 | 0.78740609 |
| 6 | 0.12608339 | -4.12753296 | -0.73685187 |
| 6 | -2.61280656 | 2.22839022 | 0.72521210 |
| 6 | -2.78055215 | 3.33076525 | -0.17080075 |
| 6 | -4.02941179 | 3.96572185 | -0.19183260 |
| 6 | -5.10059643 | 3.56057906 | 0.60900116 |
| 6 | -4.92816734 | 2.48564959 | 1.47241080 |
| 6 | -3.69545889 | 1.82614410 | 1.54802442 |
| 6 | -3.53314734 | 0.73076063 | 2.56787252 |
| 6 | -1.64039779 | 3.80516458 | -1.09281516 |
| 6 | 0.33379456 | -5.54589939 | -1.30120409 |
| 6 | 0.90970176 | -4.04986620 | 0.59509552 |
| 6 | 0.71135163 | -3.13779235 | -1.77058804 |
| 6 | -2.04186463 | 5.03379297 | -1.93096936 |
| 6 | -1.27253592 | 2.67617249 | -2.08377576 |
| 6 | -0.40556312 | 4.21295404 | -0.25409725 |
| 1 | -4.12797451 | -1.80914259 | 3.29704952 |
| 1 | -2.56729674 | -2.50991035 | 2.77506804 |
| 1 | -4.73238659 | -1.04004145 | 0.98094404 |
| 1 | -3.48696685 | -0.19205493 | 0.04742144 |
| 1 | -2.05053854 | -5.73407173 | -1.15514219 |
| 1 | -5.16268873 | -3.11481953 | 0.19223931 |
| 1 | -2.86565924 | 1.04395735 | 3.38687873 |
| 1 | -4.51850128 | 0.50605243 | 3.01111913 |
| 1 | -4.18045092 | 4.80807972 | -0.86426717 |
| 1 | -5.74981213 | 2.15073538 | 2.10981369 |
| 1 | 1.40906107 | -5.71256542 | -1.45991635 |
| 1 | -0.02262610 | -6.32389688 | -0.61094540 |
| 1 | -0.16778952 | -5.68315029 | -2.26984358 |
| 1 | 0.80863923 | -3.06604910 | 1.06610763 |
| 1 | 0.54087865 | -4.80907679 | 1.30034530 |
| 1 | 1.97786975 | -4.24800920 | 0.41389152 |
| 1 | 0.19519912 | -3.24184084 | -2.73603487 |
| 1 | 0.60652161 | -2.10110807 | -1.43491948 |
| 1 | 1.77882373 | -3.35320997 | -1.93394518 |
| 1 | -1.19044697 | 5.32817745 | -2.56097746 |
| 1 | -2.30325198 | 5.89669514 | -1.30136025 |
| 1 | -2.88771749 | 4.82171488 | -2.60056186 |
| 1 | -0.97220641 | 1.76436973 | -1.55755150 |
| 1 | -2.13079619 | 2.43804765 | -2.72886825 |
| 1 | -0.44216588 | 2.99603105 | -2.73157310 |
| 1 | -0.65351903 | 5.05614710 | 0.40758240 |
| 1 | -0.04670966 | 3.38115120 | 0.36143848 |
| 1 | 0.40722418 | 4.53657341 | -0.92223090 |
| 1 | -2.58651137 | -1.22358167 | 4.01194477 |
| 8 | -0.50363553 | -0.12128476 | 3.41325378 |
| 31 | -0.97945833 | -0.10462069 | 1.44587195 |

| | | | |
|---|---|---|---|
| 1 | -4.44007874 | -5.31558418 | -0.74809623 |
| 1 | -6.05689573 | 4.08019876 | 0.55138171 |
| 8 | 4.62217808 | 0.99544281 | -6.23509359 |
| 6 | 4.58951092 | 1.66727579 | -7.51726246 |
| 1 | 3.59833217 | 1.56785750 | -7.97717237 |
| 6 | 3.86508012 | 0.73661435 | -4.00277710 |
| 6 | 2.72515154 | 1.02708888 | -3.02657938 |
| 6 | 2.91871858 | 0.33027002 | -1.67553699 |
| 6 | 3.73001313 | 1.47699332 | -5.31954241 |
| 6 | 1.76816821 | 0.60851175 | -0.70136017 |
| 6 | 1.96593928 | -0.08774115 | 0.64485025 |
| 1 | 3.94950104 | -0.33999237 | -4.21673441 |
| 1 | 1.77162814 | 0.70775580 | -3.47502398 |
| 1 | 3.01522756 | -0.75777417 | -1.83257318 |
| 1 | 0.81842095 | 0.27061692 | -1.14493680 |
| 1 | 4.83569098 | 1.02857769 | -3.56589508 |
| 1 | 2.64182568 | 2.11436152 | -2.88044643 |
| 1 | 3.87102890 | 0.66378295 | -1.22617376 |
| 1 | 1.67190337 | 1.69168615 | -0.53048486 |
| 1 | 2.93403697 | 0.23802061 | 1.06453598 |
| 1 | 2.01592612 | -1.18133843 | 0.49999952 |
| 8 | 2.95552635 | 2.38527989 | -5.55310011 |
| 8 | 0.94676620 | 0.25515920 | 1.58266270 |
| 1 | 5.35103607 | 1.16878760 | -8.12468815 |
| 1 | 4.82274628 | 2.73298550 | -7.39870739 |
| 8 | 2.14463592 | 0.64009792 | 5.44360447 |
| 6 | 2.90039063 | -0.48565641 | 5.42123318 |
| 6 | 2.64551806 | -1.49310374 | 4.30558443 |
| 8 | 1.33455682 | -1.34978223 | 3.68211961 |
| 6 | 0.77104479 | -0.13432436 | 3.52701402 |
| 6 | 1.43452716 | 1.06646526 | 4.23307371 |
| 8 | 3.71405196 | -0.70574278 | 6.29111624 |
| 6 | 2.32485914 | 1.99038196 | 3.41769600 |
| 6 | 3.79389501 | -1.56736493 | 3.30261731 |
| 1 | 2.56503725 | -2.46553111 | 4.81033659 |
| 1 | 0.59052306 | 1.64276481 | 4.63151741 |
| 1 | 2.68758225 | 2.79121280 | 4.07514334 |
| 1 | 1.74784982 | 2.42546988 | 2.59451723 |
| 1 | 3.19143653 | 1.46603858 | 2.99751353 |
| 1 | 4.70534039 | -1.85549319 | 3.84197998 |
| 1 | 3.97365069 | -0.60967267 | 2.80122757 |
| 1 | 3.57272291 | -2.32913160 | 2.54499841 |

Electronic energy = -4057.824100 a.u.
DFT-D3(BJ) dispersion correction = -0.142129 a.u.
Thermal free energy = 0.721608 a.u.
Gibbs free energy = -4057.244621 a.u.
Number of imaginary frequencies = 1.

**TS3 ax LA isomer/conformer 4**

| Atomic N. | X | Y | Z |
|---|---|---|---|
| 7 | -1.21138930 | 0.81549138 | -2.22836089 |
| 8 | -1.34317625 | -0.41708368 | 0.48722181 |

| | | | |
|---|---|---|---|
| 8 | -0.71303105 | -2.09436607 | -2.17671037 |
| 6 | -0.54969352 | 2.13437009 | -2.34054732 |
| 6 | -1.99555171 | 0.72520953 | 0.80183589 |
| 6 | -2.14727879 | 1.10475445 | 2.16826677 |
| 6 | -2.78903747 | 2.32615209 | 2.42206097 |
| 6 | -3.29900289 | 3.14338756 | 1.40828371 |
| 6 | -3.20149398 | 2.72686362 | 0.08446964 |
| 6 | -2.56830740 | 1.51805174 | -0.22378638 |
| 6 | -2.58192301 | 0.98919070 | -1.62968266 |
| 6 | -1.67223835 | 0.19707730 | 3.32086158 |
| 6 | -1.79062533 | -2.14355230 | -2.99329543 |
| 6 | -2.54094672 | -3.34882593 | -3.13401365 |
| 6 | -3.63140893 | -3.32297921 | -4.01547909 |
| 6 | -3.99816155 | -2.18519306 | -4.74100828 |
| 6 | -3.25645518 | -1.01835561 | -4.59587955 |
| 6 | -2.15371537 | -0.99535501 | -3.73507738 |
| 6 | -1.31181514 | 0.24051653 | -3.62327290 |
| 6 | -2.17376924 | -4.62951136 | -2.36012983 |
| 6 | -2.00045872 | 0.80298686 | 4.69852924 |
| 6 | -0.14258187 | -0.01930438 | 3.26543689 |
| 6 | -2.39424586 | -1.16909015 | 3.22912598 |
| 6 | -3.13829660 | -5.78920126 | -2.67158079 |
| 6 | -2.23611903 | -4.37538290 | -0.83545852 |
| 6 | -0.75279278 | -5.08886099 | -2.76184678 |
| 1 | -1.18305480 | 2.81804657 | -2.93048573 |
| 1 | -0.39743847 | 2.56999636 | -1.34796834 |
| 1 | -3.16226172 | 1.66099894 | -2.28549004 |
| 1 | -3.05903316 | -0.00074593 | -1.66227531 |
| 1 | -2.90027380 | 2.65745926 | 3.45278978 |
| 1 | -3.63531089 | 3.32505417 | -0.72031355 |
| 1 | -0.27401814 | 0.04220875 | -3.93612981 |
| 1 | -1.72059321 | 1.02576363 | -4.28182697 |
| 1 | -4.22780228 | -4.22476721 | -4.14122534 |
| 1 | -3.52065063 | -0.11773428 | -5.15473366 |
| 1 | -1.66188538 | 0.10951162 | 5.48130131 |
| 1 | -1.48705101 | 1.76137578 | 4.86317205 |
| 1 | -3.08053422 | 0.95575964 | 4.83737993 |
| 1 | 0.16613358 | -0.51245952 | 2.33747792 |
| 1 | 0.38626435 | 0.94070768 | 3.35648251 |
| 1 | 0.16909517 | -0.65302908 | 4.10964870 |
| 1 | -3.48353457 | -1.03843856 | 3.30700421 |
| 1 | -2.16816926 | -1.68038321 | 2.28677440 |
| 1 | -2.07078981 | -1.81465220 | 4.05970764 |
| 1 | -2.82616186 | -6.67525816 | -2.10072160 |
| 1 | -3.12891650 | -6.06263876 | -3.73661780 |
| 1 | -4.17279911 | -5.55641365 | -2.38031244 |
| 1 | -1.51314402 | -3.61687517 | -0.51856196 |
| 1 | -3.24176550 | -4.04554462 | -0.53706688 |
| 1 | -2.01324725 | -5.30815411 | -0.29553157 |
| 1 | -0.71224558 | -5.33458757 | -3.83310080 |
| 1 | -0.01402104 | -4.30745554 | -2.55450821 |
| 1 | -0.47676122 | -5.99181080 | -2.19600224 |
| 1 | 0.41075054 | 2.01334524 | -2.85191488 |

| | | | |
|---|---|---|---|
| 8 | 1.08187592 | -1.79008079 | -0.10238135 |
| 31 | -0.26169178 | -0.69187587 | -1.00696385 |
| 1 | -3.78575253 | 4.08575869 | 1.65946519 |
| 1 | -4.85799503 | -2.21722054 | -5.40999269 |
| 8 | 2.51548481 | 6.86634016 | 4.06945944 |
| 6 | 2.18129373 | 7.43323565 | 5.35953474 |
| 1 | 1.09388387 | 7.43996811 | 5.50540543 |
| 6 | 2.51979494 | 5.06852579 | 2.52481484 |
| 6 | 1.95036697 | 3.68868637 | 2.19754243 |
| 6 | 2.41542053 | 3.17142653 | 0.83200407 |
| 6 | 2.10220385 | 5.57734060 | 3.89175034 |
| 6 | 1.81865942 | 1.80109119 | 0.48601827 |
| 6 | 2.30340910 | 1.30230761 | -0.87228084 |
| 1 | 2.21844172 | 5.81688261 | 1.77481735 |
| 1 | 0.85069466 | 3.73197436 | 2.22356653 |
| 1 | 2.14190793 | 3.90112996 | 0.04924996 |
| 1 | 0.71832544 | 1.87570906 | 0.48244947 |
| 1 | 3.62261057 | 5.05642033 | 2.50145817 |
| 1 | 2.24265790 | 2.97886872 | 2.98561621 |
| 1 | 3.51779890 | 3.10887575 | 0.81926346 |
| 1 | 2.07861328 | 1.06717694 | 1.26130080 |
| 1 | 3.39297152 | 1.15401995 | -0.85760260 |
| 1 | 2.11222982 | 2.05818915 | -1.64919627 |
| 8 | 1.49242783 | 4.94573593 | 4.73333693 |
| 8 | 1.69564962 | 0.07373357 | -1.33324170 |
| 1 | 2.57657838 | 8.45329952 | 5.33834696 |
| 1 | 2.64640045 | 6.85247803 | 6.16598940 |
| 8 | 3.16511726 | -1.09420586 | 0.34728208 |
| 6 | 2.21553040 | -1.37412477 | -0.60924375 |
| 6 | 2.73236489 | -2.16826487 | -1.81576025 |
| 8 | 3.96976233 | -1.61066484 | -2.36018205 |
| 6 | 4.91491032 | -1.16567719 | -1.50177956 |
| 6 | 4.57464838 | -1.03175020 | -0.01608035 |
| 8 | 6.01191568 | -0.83923513 | -1.90554917 |
| 6 | 5.36191940 | -2.02431846 | 0.83981723 |
| 6 | 2.89473009 | -3.65309978 | -1.51341307 |
| 1 | 2.00404739 | -2.03946805 | -2.62534189 |
| 1 | 4.90700436 | -0.01663340 | 0.25107971 |
| 1 | 5.14833975 | -1.83467638 | 1.89893162 |
| 1 | 6.43496895 | -1.88563251 | 0.65898061 |
| 1 | 5.09368563 | -3.06249595 | 0.60974652 |
| 1 | 1.92745161 | -4.06371784 | -1.20184207 |
| 1 | 3.62257099 | -3.83895493 | -0.71358377 |
| 1 | 3.22950768 | -4.17334700 | -2.41970468 |

Electronic energy = -4057.824569 a.u.
DFT-D3(BJ) dispersion correction = -0.143258 a.u.
Thermal free energy = 0.724726 a.u.
Gibbs free energy = -4057.243102 a.u.
Number of imaginary frequencies = 1.

**TS3 ax LA isomer/conformer 5**

Atomic N. X Y Z

| | | | |
|---|---|---|---|
| 7 | -1.48447083 | 1.52607589 | 0.72387270 |
| 8 | -2.28038941 | -1.29193380 | 0.90504236 |
| 8 | -1.46452997 | 0.17290550 | -1.90715562 |
| 6 | -0.58188961 | 1.91527604 | 1.83328654 |
| 6 | -2.81117517 | -0.91839845 | 2.08904192 |
| 6 | -3.12109939 | -1.90133744 | 3.07635176 |
| 6 | -3.66103055 | -1.44194235 | 4.28646045 |
| 6 | -3.90794517 | -0.09033190 | 4.54765775 |
| 6 | -3.62789887 | 0.85324725 | 3.56515987 |
| 6 | -3.09582678 | 0.44518829 | 2.33761771 |
| 6 | -2.90265809 | 1.43379683 | 1.22882678 |
| 6 | -2.88670940 | -3.40368447 | 2.82726188 |
| 6 | -2.31928442 | 1.16000193 | -2.25852238 |
| 6 | -3.14755343 | 1.00302199 | -3.40942758 |
| 6 | -4.00125646 | 2.06606155 | -3.73568999 |
| 6 | -4.06740949 | 3.24424106 | -2.98572101 |
| 6 | -3.25085129 | 3.38624235 | -1.86979385 |
| 6 | -2.36963810 | 2.36050985 | -1.50766939 |
| 6 | -1.42492555 | 2.57468078 | -0.35665004 |
| 6 | -3.11257247 | -0.28216652 | -4.26023132 |
| 6 | -3.34281624 | -4.26272390 | 4.02155002 |
| 6 | -1.38191606 | -3.67940654 | 2.60245973 |
| 6 | -3.69382541 | -3.86593486 | 1.59048511 |
| 6 | -4.07638131 | -0.20344033 | -5.45926967 |
| 6 | -3.53832771 | -1.49981743 | -3.40591150 |
| 6 | -1.69060926 | -0.50786962 | -4.82744966 |
| 1 | -0.91986358 | 2.86586883 | 2.27877243 |
| 1 | -0.58811428 | 1.14010268 | 2.60673566 |
| 1 | -3.20874972 | 2.44151859 | 1.55927309 |
| 1 | -3.51927549 | 1.16840827 | 0.35760068 |
| 1 | -3.90033335 | -2.16574142 | 5.06346616 |
| 1 | -3.83711611 | 1.91219699 | 3.73405113 |
| 1 | -0.38316373 | 2.59805028 | -0.70982836 |
| 1 | -1.63470324 | 3.55240712 | 0.11093153 |
| 1 | -4.64936156 | 1.97431166 | -4.60529215 |
| 1 | -3.28153464 | 4.30090814 | -1.27324062 |
| 1 | -3.16736298 | -5.32186835 | 3.78504629 |
| 1 | -2.78034236 | -4.03226116 | 4.93817754 |
| 1 | -4.41567260 | -4.14430096 | 4.23215951 |
| 1 | -1.00174695 | -3.15843110 | 1.71816550 |
| 1 | -0.79529961 | -3.36519016 | 3.47896375 |
| 1 | -1.22170020 | -4.75923649 | 2.45911537 |
| 1 | -4.77074481 | -3.70805310 | 1.74927765 |
| 1 | -3.38842655 | -3.32513448 | 0.68833133 |
| 1 | -3.53091190 | -4.94152902 | 1.42308286 |
| 1 | -4.00498221 | -1.13667709 | -6.03571613 |
| 1 | -3.82653486 | 0.62464178 | -6.13838016 |
| 1 | -5.12345782 | -0.09113143 | -5.14288969 |
| 1 | -2.85413945 | -1.66833671 | -2.56774489 |
| 1 | -4.55289271 | -1.35806311 | -3.00638869 |
| 1 | -3.54515772 | -2.40616677 | -4.03034175 |
| 1 | -1.39079210 | 0.33323930 | -5.46972427 |
| 1 | -0.95458465 | -0.61601670 | -4.02333614 |

| | | | |
|---|---|---|---|
| 1 | -1.67678829 | -1.42204797 | -5.44030885 |
| 1 | 0.43661422 | 2.03967549 | 1.45264441 |
| 8 | -0.21615518 | -2.06822082 | -0.69820259 |
| 31 | -1.04886415 | -0.37412473 | -0.17123134 |
| 1 | -4.32531357 | 0.21380653 | 5.50752022 |
| 1 | -4.75427595 | 4.03932114 | -3.27534535 |
| 8 | 7.76702366 | 5.23894072 | 0.62174909 |
| 6 | 8.77800857 | 5.80998253 | 1.48778246 |
| 1 | 8.31229969 | 6.42815256 | 2.26559809 |
| 6 | 5.83512808 | 3.91036037 | 0.27276153 |
| 6 | 4.97008969 | 2.79021349 | 0.85034465 |
| 6 | 3.86762555 | 2.34442340 | -0.11600684 |
| 6 | 6.86895156 | 4.42978614 | 1.25441306 |
| 6 | 3.01898254 | 1.19742214 | 0.44835738 |
| 6 | 1.91755289 | 0.78255862 | -0.52430095 |
| 1 | 5.21186024 | 4.77065909 | -0.02632984 |
| 1 | 4.52655124 | 3.12890925 | 1.79868863 |
| 1 | 3.21818400 | 3.20555248 | -0.35368161 |
| 1 | 2.57114002 | 1.49918727 | 1.40918332 |
| 1 | 6.35476427 | 3.58883388 | -0.64271344 |
| 1 | 5.60941690 | 1.93048880 | 1.10347116 |
| 1 | 4.32004752 | 2.02538397 | -1.07031965 |
| 1 | 3.67633298 | 0.34230230 | 0.66377119 |
| 1 | 2.36368524 | 0.44300010 | -1.47105655 |
| 1 | 1.29533136 | 1.65485511 | -0.77747879 |
| 8 | 6.90581698 | 4.18593358 | 2.44549958 |
| 8 | 1.04083291 | -0.24436102 | -0.00065376 |
| 1 | 9.40846803 | 6.41932730 | 0.83332384 |
| 1 | 9.36554298 | 5.01622175 | 1.96561024 |
| 8 | 3.36058360 | -1.85086532 | -1.46593343 |
| 6 | 3.87442317 | -2.15629454 | -0.24596440 |
| 6 | 2.93149634 | -2.50711773 | 0.90639094 |
| 8 | 1.52732945 | -2.61034548 | 0.58323636 |
| 6 | 1.05761603 | -1.90461809 | -0.50138300 |
| 6 | 1.93784514 | -2.04110307 | -1.74195548 |
| 8 | 5.07150666 | -2.07651976 | -0.06407022 |
| 6 | 1.72097063 | -3.39677207 | -2.40756454 |
| 6 | 3.34346106 | -3.81013687 | 1.58409785 |
| 1 | 3.04071366 | -1.67345191 | 1.62271359 |
| 1 | 1.68026591 | -1.23955849 | -2.44671170 |
| 1 | 2.37917371 | -3.47928747 | -3.28139089 |
| 1 | 0.67707229 | -3.48468219 | -2.73049126 |
| 1 | 1.94360595 | -4.21819435 | -1.71381865 |
| 1 | 4.38674787 | -3.74088350 | 1.91166826 |
| 1 | 3.23996860 | -4.65657679 | 0.89277383 |
| 1 | 2.69832159 | -3.98591314 | 2.45346333 |

Electronic energy = -4057.824887 a.u.
DFT-D3(BJ) dispersion correction = -0.138978 a.u.
Thermal free energy = 0.722093 a.u.
Gibbs free energy = -4057.241772 a.u.
Number of imaginary frequencies = 1.

**TS3 ax LA isomer/conformer 6**

| Atomic N. | X | Y | Z |
|---|---|---|---|
| 7 | -2.07110238 | 1.34607089 | 1.83900420 |
| 8 | -1.81866024 | -1.47514156 | 0.76071984 |
| 8 | -0.89940915 | 0.79759617 | -0.85639640 |
| 6 | -1.70119300 | 1.74532727 | 3.21974366 |
| 6 | -2.66087946 | -1.78968542 | 1.77911016 |
| 6 | -2.86203464 | -3.14753267 | 2.15679331 |
| 6 | -3.71367627 | -3.38965792 | 3.24536942 |
| 6 | -4.37301748 | -2.36947046 | 3.93601221 |
| 6 | -4.22457775 | -1.05369783 | 3.50933900 |
| 6 | -3.39236218 | -0.75803156 | 2.42493522 |
| 6 | -3.40214687 | 0.62764679 | 1.83618682 |
| 6 | -2.20521234 | -4.31086876 | 1.38701993 |
| 6 | -1.97702725 | 1.47730425 | -1.29947884 |
| 6 | -2.42555869 | 1.33746499 | -2.64805049 |
| 6 | -3.53787357 | 2.09625147 | -3.03831398 |
| 6 | -4.21144950 | 2.96281918 | -2.17209913 |
| 6 | -3.76514938 | 3.09403012 | -0.86251049 |
| 6 | -2.65077202 | 2.36912133 | -0.42401758 |
| 6 | -2.13526687 | 2.58971086 | 0.97154872 |
| 6 | -1.72337447 | 0.39145791 | -3.64225951 |
| 6 | -2.65026374 | -5.68327780 | 1.92666808 |
| 6 | -0.66678161 | -4.24372245 | 1.51185457 |
| 6 | -2.61543181 | -4.24907146 | -0.10399436 |
| 6 | -2.38272471 | 0.42533510 | -5.03409099 |
| 6 | -1.79767932 | -1.06666708 | -3.13245188 |
| 6 | -0.24669412 | 0.81479021 | -3.82237586 |
| 1 | -2.45242771 | 2.45163609 | 3.60807980 |
| 1 | -1.65129519 | 0.85916967 | 3.85735463 |
| 1 | -4.12812603 | 1.26312028 | 2.36968886 |
| 1 | -3.70746369 | 0.58184153 | 0.78105342 |
| 1 | -3.87231931 | -4.41567678 | 3.57230543 |
| 1 | -4.77584227 | -0.24663527 | 3.99755614 |
| 1 | -1.10799503 | 2.98878841 | 0.95736973 |
| 1 | -2.77592614 | 3.32308842 | 1.48776124 |
| 1 | -3.90455335 | 2.00674918 | -4.05917497 |
| 1 | -4.27177472 | 3.76897455 | -0.16885672 |
| 1 | -2.17280011 | -6.47324770 | 1.32945403 |
| 1 | -2.35057293 | -5.83561670 | 2.97371539 |
| 1 | -3.73821046 | -5.82368535 | 1.85219046 |
| 1 | -0.26941267 | -3.29634444 | 1.13361839 |
| 1 | -0.36790374 | -4.35267467 | 2.56499487 |
| 1 | -0.21093710 | -5.06879016 | 0.94270149 |
| 1 | -3.70703201 | -4.33259472 | -0.20856863 |
| 1 | -2.29187306 | -3.31362690 | -0.57293015 |
| 1 | -2.15913444 | -5.08908759 | -0.64961721 |
| 1 | -1.84129904 | -0.26016142 | -5.70166347 |
| 1 | -2.34513422 | 1.42664988 | -5.48696223 |
| 1 | -3.43098499 | 0.09521483 | -5.00342930 |
| 1 | -1.31770199 | -1.17993995 | -2.15488754 |
| 1 | -2.84404858 | -1.39126613 | -3.03986368 |
| 1 | -1.29748033 | -1.73733143 | -3.84774325 |

```
   1    -0.18157649   1.83495399  -4.22838407
   1     0.29255722   0.78436713  -2.86962244
   1     0.25241390   0.13579728  -4.53055442
   1    -0.71937601   2.23390354   3.20551792
   8    -0.00622667  -0.69422598   2.95544143
  31    -0.77801078   0.06483070   0.88439992
   1    -5.01549818  -2.60926680   4.78315414
   1    -5.07592332   3.52569998  -2.52354421
   8     5.94487220   4.16921948  -5.04570836
   6     6.42827272   5.42318114  -5.58452383
   1     5.58740927   6.07268694  -5.85831954
   6     4.74233472   2.92724442  -3.42389314
   6     3.75537879   2.99668445  -2.25906303
   6     3.36725536   1.60957048  -1.73651174
   6     5.17965813   4.29164153  -3.92138633
   6     2.36360698   1.67393011  -0.57907514
   6     1.98596986   0.28658040  -0.06706016
   1     4.31986715   2.37082108  -4.27537996
   1     2.85213186   3.53831978  -2.57970413
   1     2.93834729   1.01367454  -2.56037921
   1     1.44681260   2.18825640  -0.90597839
   1     5.65304509   2.37553318  -3.13549702
   1     4.19518752   3.59545790  -1.44704474
   1     4.27548920   1.07368808  -1.40863329
   1     2.79018062   2.26580230   0.24719464
   1     2.88028151  -0.26764232   0.26382197
   1     1.52612246  -0.29963984  -0.87875605
   8     4.90982110   5.36057327  -3.40734446
   8     1.09652468   0.34116804   1.06878670
   1     7.01297237   5.15190490  -6.46873179
   1     7.05494368   5.94161611  -4.84788935
   8     1.92581571  -1.75208291   2.27578078
   6     1.22695493  -0.66781248   2.79866001
   6     2.13314484   0.18939200   3.68727347
   8     2.56581821  -0.66902349   4.80612813
   6     2.80487083  -1.99907162   4.62012789
   6     2.33802852  -2.67333503   3.32302632
   8     3.30481330  -2.64382886   5.51596565
   6     3.40239531  -3.59166456   2.74428067
   6     3.36338587   0.80985571   3.05204192
   1     1.51320661   0.95232118   4.17489541
   1     1.45367844  -3.26846061   3.61639443
   1     2.99209596  -4.12690406   1.87924194
   1     3.71164847  -4.31573733   3.50646971
   1     4.28002004  -3.01490760   2.42388547
   1     3.07096952   1.61009254   2.36258702
   1     3.94743560   0.06025234   2.50284550
   1     3.99344087   1.23792743   3.84265136
Electronic energy = -4057.815537 a.u.
DFT-D3(BJ) dispersion correction = -0.140469 a.u.
Thermal free energy = 0.720317 a.u.
Gibbs free energy = -4057.235689 a.u.
Number of imaginary frequencies = 1.
```

**TS3 ax LA isomer/conformer 7**

| Atomic N. | X | Y | Z |
|---|---|---|---|
| 7 | -1.88891861 | 1.27363257 | 0.30435436 |
| 8 | -1.75630627 | -1.54450555 | 1.14361104 |
| 8 | -1.20270425 | -0.49682901 | -1.94536902 |
| 6 | -1.29870416 | 2.13452474 | 1.35677142 |
| 6 | -2.52892871 | -1.10758930 | 2.16312092 |
| 6 | -2.61473680 | -1.87607615 | 3.36280727 |
| 6 | -3.44245484 | -1.38344179 | 4.38171742 |
| 6 | -4.16905450 | -0.19474515 | 4.26343380 |
| 6 | -4.08484458 | 0.53766078 | 3.08442471 |
| 6 | -3.28101322 | 0.08408259 | 2.03314465 |
| 6 | -3.26358243 | 0.82174919 | 0.72758568 |
| 6 | -1.83526885 | -3.19382076 | 3.53721464 |
| 6 | -2.24654445 | 0.10954095 | -2.55365099 |
| 6 | -2.85639984 | -0.50514627 | -3.68763833 |
| 6 | -3.92724683 | 0.17313278 | -4.28634432 |
| 6 | -4.40398637 | 1.40204303 | -3.81973839 |
| 6 | -3.79193963 | 1.99570492 | -2.72180846 |
| 6 | -2.71078129 | 1.36593518 | -2.09435606 |
| 6 | -1.99672923 | 2.06602704 | -0.97212726 |
| 6 | -2.35963605 | -1.85661163 | -4.23774683 |
| 6 | -2.11210436 | -3.85262186 | 4.90141015 |
| 6 | -0.31558533 | -2.91809961 | 3.45553672 |
| 6 | -2.24565505 | -4.20662454 | 2.44176495 |
| 6 | -3.15807369 | -2.30122347 | -5.47754499 |
| 6 | -2.51589572 | -2.96201068 | -3.16670067 |
| 6 | -0.87570404 | -1.73941609 | -4.66193664 |
| 1 | -1.98924937 | 2.95927967 | 1.59989021 |
| 1 | -1.11435874 | 1.54887767 | 2.26321663 |
| 1 | -3.91198464 | 1.71277989 | 0.78768066 |
| 1 | -3.64546239 | 0.19035086 | -0.08839739 |
| 1 | -3.52567474 | -1.94536137 | 5.31021642 |
| 1 | -4.65563939 | 1.46101593 | 2.96157026 |
| 1 | -0.96499176 | 2.31585736 | -1.26676471 |
| 1 | -2.51540214 | 3.01290643 | -0.74197860 |
| 1 | -4.41791616 | -0.27362431 | -5.14911671 |
| 1 | -4.14060872 | 2.96068139 | -2.34677672 |
| 1 | -1.53760612 | -4.78739365 | 4.96913627 |
| 1 | -1.80463047 | -3.21407534 | 5.74235165 |
| 1 | -3.17404198 | -4.10806017 | 5.02824804 |
| 1 | -0.04869175 | -2.48415463 | 2.48596232 |
| 1 | -0.00188370 | -2.22721125 | 4.25201961 |
| 1 | 0.24138180 | -3.85909876 | 3.58315184 |
| 1 | -3.32098271 | -4.42830358 | 2.50515240 |
| 1 | -2.02449810 | -3.82616036 | 1.43899569 |
| 1 | -1.69806426 | -5.15046545 | 2.58578030 |
| 1 | -2.75677214 | -3.26008108 | -5.83511280 |
| 1 | -3.07717526 | -1.57991402 | -6.30365871 |
| 1 | -4.22326449 | -2.45395316 | -5.25125867 |
| 1 | -1.91044826 | -2.75613836 | -2.27795581 |

| | | | |
|---|---|---|---|
| 1 | -3.56752379 | -3.05947687 | -2.86037895 |
| 1 | -2.19591287 | -3.92920501 | -3.58335016 |
| 1 | -0.75875938 | -0.99223269 | -5.46071482 |
| 1 | -0.24410894 | -1.44897689 | -3.81496536 |
| 1 | -0.52421035 | -2.70740682 | -5.05116650 |
| 1 | -0.35519588 | 2.55713518 | 0.99656223 |
| 8 | 0.31713475 | -2.30413266 | -0.40126568 |
| 31 | -0.82077677 | -0.53866559 | -0.11983294 |
| 1 | -4.79668605 | 0.14716374 | 5.08629644 |
| 1 | -5.24558616 | 1.88608891 | -4.31495139 |
| 8 | 5.69235346 | 7.18222717 | 1.36317310 |
| 6 | 6.41534646 | 8.02477330 | 2.29356912 |
| 1 | 5.74639897 | 8.38299006 | 3.08605331 |
| 6 | 4.40372221 | 5.25608394 | 0.86849451 |
| 6 | 3.79053751 | 3.96927752 | 1.41944060 |
| 6 | 3.03637283 | 3.17444713 | 0.34817978 |
| 6 | 5.13869984 | 6.06625324 | 1.91991517 |
| 6 | 2.42117675 | 1.87808253 | 0.88994562 |
| 6 | 1.69412275 | 1.09458024 | -0.20341223 |
| 1 | 3.63146744 | 5.90641227 | 0.42547802 |
| 1 | 3.11240089 | 4.21687246 | 2.25036504 |
| 1 | 2.24214007 | 3.80763071 | -0.08595162 |
| 1 | 1.72304040 | 2.10757656 | 1.71087481 |
| 1 | 5.11103108 | 5.04298028 | 0.05071694 |
| 1 | 4.58574644 | 3.34616376 | 1.85653697 |
| 1 | 3.72219296 | 2.93471721 | -0.48294622 |
| 1 | 3.21346839 | 1.25145469 | 1.32528038 |
| 1 | 2.40481163 | 0.78060845 | -0.98649884 |
| 1 | 0.96403958 | 1.75066111 | -0.70938612 |
| 8 | 5.23422960 | 5.78510038 | 3.09939951 |
| 8 | 1.03404146 | -0.08312402 | 0.29072937 |
| 1 | 6.79065694 | 8.86034256 | 1.69519373 |
| 1 | 7.24439264 | 7.46910244 | 2.74927713 |
| 8 | 3.25538800 | -3.57415742 | -0.23667927 |
| 6 | 3.34193217 | -3.58343752 | -1.59681490 |
| 6 | 2.54421443 | -2.54795039 | -2.39848956 |
| 8 | 2.09992364 | -1.42975313 | -1.58060888 |
| 6 | 1.50413801 | -1.90245805 | -0.41697702 |
| 6 | 2.54041727 | -2.52510572 | 0.51950834 |
| 8 | 3.99722948 | -4.44374640 | -2.14398768 |
| 6 | 3.57157059 | -1.59944857 | 1.13363544 |
| 6 | 3.34160364 | -1.98718525 | -3.56297151 |
| 1 | 1.64833984 | -3.07557104 | -2.77444653 |
| 1 | 2.00017589 | -3.08214846 | 1.29491238 |
| 1 | 4.33709618 | -2.20595577 | 1.63470288 |
| 1 | 3.09242999 | -0.95106364 | 1.87606824 |
| 1 | 4.05620843 | -0.98073072 | 0.36705643 |
| 1 | 3.69353433 | -2.81350018 | -4.19069871 |
| 1 | 4.21139278 | -1.42391396 | -3.20028357 |
| 1 | 2.70440513 | -1.32240465 | -4.15887496 |

Electronic energy = -4057.814068 a.u.
DFT-D3(BJ) dispersion correction = -0.139027 a.u.
Thermal free energy = 0.718393 a.u.

Gibbs free energy = -4057.234702 a.u.
Number of imaginary frequencies = 1.

**TS3 ax LA isomer/conformer 8**

| Atomic N. | X | Y | Z |
|---|---|---|---|
| 7 | -1.66920697 | 0.93625286 | 1.08036961 |
| 8 | -1.99108509 | -1.96591099 | 1.01306766 |
| 8 | -1.11662987 | -0.33028325 | -1.57648190 |
| 6 | -0.98982385 | 1.41013043 | 2.30956379 |
| 6 | -2.74626920 | -1.77393653 | 2.11431331 |
| 6 | -3.04836206 | -2.87154860 | 2.97667697 |
| 6 | -3.81411621 | -2.59610066 | 4.11888988 |
| 6 | -4.29474415 | -1.31899882 | 4.42510623 |
| 6 | -4.03156395 | -0.26812394 | 3.55288199 |
| 6 | -3.27692432 | -0.49160792 | 2.39663113 |
| 6 | -3.10428837 | 0.59811785 | 1.38223326 |
| 6 | -2.58344626 | -4.30571534 | 2.65738542 |
| 6 | -2.05536056 | 0.54385382 | -2.00238042 |
| 6 | -2.71366212 | 0.31062987 | -3.24720366 |
| 6 | -3.66331854 | 1.25463970 | -3.66090650 |
| 6 | -3.98405100 | 2.38769870 | -2.90727382 |
| 6 | -3.33568996 | 2.60360626 | -1.69712044 |
| 6 | -2.36573300 | 1.70134148 | -1.24218871 |
| 6 | -1.62542592 | 2.01773317 | 0.03333779 |
| 6 | -2.39749532 | -0.93086448 | -4.10515967 |
| 6 | -3.06236980 | -5.31644646 | 3.71618168 |
| 6 | -1.03973221 | -4.37854215 | 2.60989257 |
| 6 | -3.16496688 | -4.74976311 | 1.29373695 |
| 6 | -3.21884128 | -0.95163805 | -5.40820164 |
| 6 | -2.74163815 | -2.22127989 | -3.32368192 |
| 6 | -0.90151358 | -0.93879219 | -4.50413167 |
| 1 | -1.47561358 | 2.32804317 | 2.68109341 |
| 1 | -1.05895394 | 0.63804671 | 3.08427134 |
| 1 | -3.60770789 | 1.52005529 | 1.72195487 |
| 1 | -3.55629501 | 0.30786872 | 0.42261223 |
| 1 | -4.05007261 | -3.40957445 | 4.80261607 |
| 1 | -4.42644518 | 0.73130230 | 3.75025740 |
| 1 | -0.56073686 | 2.21201083 | -0.16117401 |
| 1 | -2.04979239 | 2.93529018 | 0.47670512 |
| 1 | -4.18309808 | 1.10143925 | -4.60482785 |
| 1 | -3.56979217 | 3.48368900 | -1.09366169 |
| 1 | -2.71637881 | -6.32041115 | 3.43211020 |
| 1 | -2.65549181 | -5.09689989 | 4.71420821 |
| 1 | -4.15898399 | -5.35273350 | 3.78772232 |
| 1 | -0.64288504 | -3.75179780 | 1.80382093 |
| 1 | -0.60671767 | -4.05804939 | 3.57003825 |
| 1 | -0.72097885 | -5.41605525 | 2.42769880 |
| 1 | -4.26434452 | -4.74193293 | 1.32122226 |
| 1 | -2.82964391 | -4.09314961 | 0.48333558 |
| 1 | -2.83889279 | -5.77618996 | 1.06678010 |
| 1 | -2.95130860 | -1.84936626 | -5.98330151 |
| 1 | -3.01112613 | -0.07811278 | -6.04328270 |

| | | | |
|---|---|---|---|
| 1 | -4.30078564 | -0.99370603 | -5.21611583 |
| 1 | -2.14640515 | -2.31424022 | -2.40950151 |
| 1 | -3.80681924 | -2.23704298 | -3.05077462 |
| 1 | -2.54118074 | -3.10059487 | -3.95433028 |
| 1 | -0.65377219 | -0.04353705 | -5.09342313 |
| 1 | -0.25154430 | -0.97154618 | -3.62327211 |
| 1 | -0.69047279 | -1.82187055 | -5.12605309 |
| 1 | 0.06362432 | 1.61244708 | 2.09132077 |
| 8 | 0.47111281 | -2.32029787 | -0.01155057 |
| 31 | -0.77007729 | -0.78350684 | 0.19500052 |
| 1 | -4.88178211 | -1.15759465 | 5.32918401 |
| 1 | -4.73694123 | 3.08929239 | -3.26615831 |
| 8 | 5.64304757 | 7.11600917 | -1.38801158 |
| 6 | 6.28087211 | 8.30670039 | -0.86471688 |
| 1 | 5.54244318 | 8.95290868 | -0.37386759 |
| 6 | 4.38614507 | 5.12846217 | -1.09648703 |
| 6 | 3.73228169 | 4.18437105 | -0.08819520 |
| 6 | 3.06963722 | 2.97711437 | -0.75983249 |
| 6 | 5.02954760 | 6.34018305 | -0.44863747 |
| 6 | 2.40880822 | 2.02365541 | 0.24371241 |
| 6 | 1.74051444 | 0.83919914 | -0.45498056 |
| 1 | 3.65022861 | 5.49443887 | -1.83133403 |
| 1 | 2.98729484 | 4.74277652 | 0.49925650 |
| 1 | 2.31335030 | 3.32791914 | -1.48326456 |
| 1 | 1.66479276 | 2.58109209 | 0.83686638 |
| 1 | 5.15780952 | 4.61034521 | -1.68833826 |
| 1 | 4.48998261 | 3.84278841 | 0.63402167 |
| 1 | 3.82132722 | 2.42456359 | -1.34998782 |
| 1 | 3.16133933 | 1.65523800 | 0.95944222 |
| 1 | 2.48273581 | 0.29389628 | -1.06153207 |
| 1 | 0.97883825 | 1.18810515 | -1.17159773 |
| 8 | 5.01239050 | 6.60751201 | 0.73800943 |
| 8 | 1.14627634 | -0.09577028 | 0.46022806 |
| 1 | 6.71705041 | 8.80678437 | -1.73458826 |
| 1 | 7.05903621 | 8.03747471 | -0.13965557 |
| 8 | 3.19666033 | -3.35185379 | 1.51869865 |
| 6 | 4.16438260 | -3.25483066 | 0.57440499 |
| 6 | 3.92267213 | -2.31443762 | -0.60440802 |
| 8 | 2.52835267 | -1.92938751 | -0.78728183 |
| 6 | 1.65839191 | -1.90165130 | 0.25852277 |
| 6 | 2.24468243 | -2.24360117 | 1.65210658 |
| 8 | 5.16092905 | -3.94152445 | 0.62752339 |
| 6 | 2.86845255 | -1.14529911 | 2.50025356 |
| 6 | 4.88662222 | -1.12973728 | -0.63470579 |
| 1 | 4.12169394 | -2.93443472 | -1.49051487 |
| 1 | 1.41345359 | -2.69924548 | 2.20294088 |
| 1 | 3.27476790 | -1.60361230 | 3.41183947 |
| 1 | 2.10921080 | -0.40703948 | 2.77788813 |
| 1 | 3.68158799 | -0.62167578 | 1.98367774 |
| 1 | 5.91357310 | -1.51468607 | -0.58935761 |
| 1 | 4.73401087 | -0.44203312 | 0.20563179 |
| 1 | 4.76136814 | -0.57644411 | -1.57379247 |

Electronic energy = -4057.813732 a.u.

DFT-D3(BJ) dispersion correction = -0.140034 a.u.
Thermal free energy = 0.720330 a.u.
Gibbs free energy = -4057.233436 a.u.
Number of imaginary frequencies = 1.

**Int3 LA isomer/conformer 1**

| Atomic N. | X | Y | Z |
|---|---|---|---|
| 7 | 1.88296044 | 2.95001254 | -0.81337131 |
| 8 | 0.74582522 | 1.76645641 | 1.68698466 |
| 8 | -0.56678116 | 1.19586023 | -1.03568076 |
| 6 | 3.33943817 | 2.82797617 | -1.08174012 |
| 6 | 1.64721871 | 2.58538210 | 2.29105490 |
| 6 | 2.04361821 | 2.34927374 | 3.63844784 |
| 6 | 2.97540235 | 3.23800866 | 4.19515627 |
| 6 | 3.50886061 | 4.32082245 | 3.49013213 |
| 6 | 3.09482979 | 4.55331349 | 2.18282718 |
| 6 | 2.15915813 | 3.70366261 | 1.58294940 |
| 6 | 1.61781256 | 4.02440586 | 0.21628696 |
| 1 | 4.23539578 | 4.97866206 | 3.96684717 |
| 6 | 1.47544763 | 1.17191999 | 4.45490158 |
| 6 | -1.14625599 | 2.35362450 | -1.45323768 |
| 6 | -2.56424524 | 2.49134199 | -1.41388525 |
| 6 | -3.10429560 | 3.70094782 | -1.87383417 |
| 6 | -2.31621049 | 4.75053331 | -2.35358976 |
| 6 | -0.93505695 | 4.60035953 | -2.39247771 |
| 6 | -0.34213113 | 3.40841738 | -1.95858705 |
| 6 | 1.14843046 | 3.23893915 | -2.10449998 |
| 1 | -2.78337723 | 5.67467870 | -2.69327450 |
| 6 | -3.47504220 | 1.36343679 | -0.88954621 |
| 6 | 2.01605827 | 1.15888184 | 5.89726221 |
| 6 | 1.87479421 | -0.17187938 | 3.80091088 |
| 6 | -0.06558428 | 1.28191040 | 4.54307918 |
| 6 | -4.96537123 | 1.74740029 | -0.95856495 |
| 6 | -3.15050995 | 1.05627555 | 0.59152174 |
| 6 | -3.28896653 | 0.09181732 | -1.75053918 |
| 1 | 3.72359457 | 3.78021593 | -1.48013368 |
| 1 | 3.86473697 | 2.58757899 | -0.15157420 |
| 1 | 2.05180685 | 4.96637613 | -0.15744855 |
| 1 | 0.52518161 | 4.14371118 | 0.25085514 |
| 1 | 3.30600425 | 3.08035167 | 5.22015478 |
| 1 | 3.47997649 | 5.40791499 | 1.62198199 |
| 1 | 1.38390563 | 2.39150276 | -2.76653466 |
| 1 | 1.58443518 | 4.14930564 | -2.54680122 |
| 1 | -4.18368441 | 3.83813392 | -1.85179000 |
| 1 | -0.30104508 | 5.40395229 | -2.77350699 |
| 1 | 1.56911786 | 0.31223284 | 6.43746018 |
| 1 | 3.10779478 | 1.03145435 | 5.93103890 |
| 1 | 1.75498812 | 2.07582800 | 6.44520022 |
| 1 | 1.49289483 | -0.25904853 | 2.77827308 |
| 1 | 2.96915980 | -0.27740308 | 3.77009290 |
| 1 | 1.47181571 | -1.00691401 | 4.39422454 |
| 1 | -0.35846097 | 2.21586401 | 5.04463249 |

| | | | |
|---|---|---|---|
| 1 | -0.52998583 | 1.25948996 | 3.55136856 |
| 1 | -0.46333211 | 0.44305325 | 5.13431121 |
| 1 | -5.56691042 | 0.90801196 | -0.58190051 |
| 1 | -5.29359328 | 1.95327181 | -1.98758457 |
| 1 | -5.19698646 | 2.62375749 | -0.33625501 |
| 1 | -2.11705446 | 0.71923626 | 0.72222486 |
| 1 | -3.30323008 | 1.94861381 | 1.21532996 |
| 1 | -3.82160176 | 0.26633724 | 0.96158918 |
| 1 | -3.57340473 | 0.28605517 | -2.79501558 |
| 1 | -2.25057564 | -0.25621833 | -1.73249854 |
| 1 | -3.93556937 | -0.71316231 | -1.36873051 |
| 1 | 3.51457297 | 2.03266561 | -1.81252532 |
| 8 | 2.06986548 | -0.24744341 | -0.00486438 |
| 8 | 1.95144793 | -0.38068311 | -2.26952564 |
| 31 | 1.01634434 | 1.25624991 | -0.07817472 |
| 8 | 4.60878789 | -0.49101004 | -1.98591825 |
| 6 | 4.11353311 | -0.28144966 | -3.24162738 |
| 6 | 2.68958689 | -0.81143945 | -3.43641339 |
| 6 | 2.35960503 | -1.06879092 | -1.06305476 |
| 6 | 3.87562554 | -1.44144107 | -1.15069625 |
| 8 | 4.75002630 | 0.35472094 | -4.05444632 |
| 6 | 4.56732094 | -1.48532929 | 0.19526264 |
| 6 | 2.02260048 | -0.26453519 | -4.68247245 |
| 1 | 2.70215652 | -1.91654729 | -3.47376188 |
| 1 | 3.93174442 | -2.42615761 | -1.63772508 |
| 1 | 5.61525485 | -1.78390874 | 0.06566018 |
| 1 | 4.06674167 | -2.21734228 | 0.84128461 |
| 1 | 4.52202685 | -0.50540326 | 0.68408310 |
| 1 | 2.57747849 | -0.58658349 | -5.57192399 |
| 1 | 2.01298370 | 0.83249160 | -4.67311878 |
| 1 | 0.99247673 | -0.63606424 | -4.74600122 |
| 8 | -5.58986161 | -6.80278309 | -0.01733281 |
| 6 | -6.21026074 | -8.10781013 | -0.10684039 |
| 1 | -6.16525531 | -8.48472178 | -1.13639819 |
| 6 | -3.71741113 | -5.36860243 | -0.25375182 |
| 6 | -2.22849960 | -5.27359737 | -0.58443330 |
| 6 | -1.69593360 | -3.84131435 | -0.47169762 |
| 6 | -4.27141752 | -6.77530028 | -0.37312658 |
| 6 | -0.20210938 | -3.74001168 | -0.80031630 |
| 6 | 0.31870023 | -2.31511274 | -0.67840743 |
| 1 | -4.31196243 | -4.71879434 | -0.91708276 |
| 1 | -2.05734034 | -5.65853214 | -1.60134330 |
| 1 | -2.26754723 | -3.18303502 | -1.14825906 |
| 1 | -0.01894067 | -4.10453411 | -1.82385174 |
| 1 | -3.92283139 | -5.00983849 | 0.76780972 |
| 1 | -1.66338865 | -5.93707842 | 0.08814201 |
| 1 | -1.87427438 | -3.46233781 | 0.54950119 |
| 1 | 0.37492317 | -4.38988432 | -0.12312806 |
| 1 | 0.16528267 | -1.93237314 | 0.34364466 |
| 1 | -0.19974994 | -1.64557343 | -1.38435492 |
| 8 | -3.65436330 | -7.75924376 | -0.73435963 |
| 8 | 1.73218671 | -2.33634001 | -0.97550099 |
| 1 | -7.24790662 | -7.95705262 | 0.20605076 |

```
   1    -5.70361973  -8.82007244   0.55649840
Electronic energy = -4057.849002 a.u.
DFT-D3(BJ) dispersion correction = -0.137689 a.u.
Thermal free energy = 0.719345 a.u.
Gibbs free energy = -4057.267346 a.u.
Number of imaginary frequencies = 0.
```

**Int3 LA isomer/conformer 2**

```
Atomic N.     X           Y            Z
   7     0.45606356   2.42200740   0.31918658
   8     0.38565770   1.06989776  -2.34081784
   8     2.86825309   0.79780610  -0.49981915
   6    -0.65366056   2.23095326   1.28828135
   6    -0.78686886   1.74595579  -2.47661166
   6    -1.74712681   1.33781353  -3.44568835
   6    -2.93110101   2.08637047  -3.52460697
   6    -3.18835070   3.19231918  -2.70906866
   6    -2.23019352   3.59533281  -1.78494125
   6    -1.02709144   2.89005385  -1.67232006
   6     0.06303351   3.39551143  -0.76769700
   1    -4.12735197   3.73683314  -2.80748040
   6    -1.50126549   0.13090396  -4.37211167
   6     3.47237459   2.01908420  -0.53514285
   6     4.68456476   2.19468942  -1.26168327
   6     5.26029192   3.47365278  -1.24500948
   6     4.69529472   4.55200264  -0.55901130
   6     3.51690827   4.36322235   0.15348027
   6     2.90572187   3.10384671   0.18294314
   6     1.69058697   2.89890697   1.05116796
   1     5.17795608   5.52878806  -0.58324812
   6     5.34412439   1.03605062  -2.03614115
   6    -2.65902602  -0.07408241  -5.36736626
   6    -1.37630267  -1.16512225  -3.53707648
   6    -0.21501204   0.35355619  -5.20310172
   6     6.65451057   1.47324345  -2.71820679
   6     4.39681241   0.52686415  -3.14804210
   6     5.69612033  -0.11747896  -1.06713759
   1    -0.87567417   3.18590974   1.79096862
   1    -1.54735223   1.88863180   0.75505833
   1    -0.24323956   4.33820093  -0.28485115
   1     0.98158614   3.58874760  -1.34070199
   1    -3.68981439   1.79585271  -4.24895888
   1    -2.39810251   4.47359818  -1.15737910
   1     1.88142129   2.13376077   1.81841030
   1     1.43737147   3.84129161   1.56394976
   1     6.18432108   3.64240080  -1.79450301
   1     3.06572218   5.18954191   0.70714725
   1    -2.42649713  -0.93358253  -6.01186297
   1    -3.61010453  -0.29141916  -4.85995101
   1    -2.80325291   0.79919059  -6.01990961
   1    -0.54335759  -1.11699214  -2.82783918
   1    -2.29999548  -1.35210063  -2.97037954
```

| | | | |
|---|---|---|---|
| 1 | -1.21179136 | -2.02280394 | -4.20696698 |
| 1 | -0.31097028 | 1.25104746 | -5.83152412 |
| 1 | 0.66565876 | 0.47016235 | -4.56241302 |
| 1 | -0.05087465 | -0.50762501 | -5.86839080 |
| 1 | 7.08655750 | 0.61057974 | -3.24464684 |
| 1 | 7.40208086 | 1.82536816 | -1.99285318 |
| 1 | 6.49026167 | 2.26504668 | -3.46317036 |
| 1 | 3.45777000 | 0.13911350 | -2.73957376 |
| 1 | 4.15771648 | 1.33426937 | -3.85483508 |
| 1 | 4.88724512 | -0.28160587 | -3.71080991 |
| 1 | 6.40318674 | 0.22420311 | -0.29732794 |
| 1 | 4.80297054 | -0.51012767 | -0.56897404 |
| 1 | 6.17327026 | -0.93835172 | -1.62313686 |
| 1 | -0.35758553 | 1.47976590 | 2.02892031 |
| 8 | 0.25598081 | -0.87467969 | -0.09165548 |
| 8 | 1.24401252 | -0.54473585 | 1.94332048 |
| 31 | 1.02039216 | 0.70428403 | -0.63432297 |
| 8 | 2.41148956 | -3.16579972 | 1.82460326 |
| 6 | 2.65885330 | -2.37562453 | 2.90012141 |
| 6 | 2.45018297 | -0.87030358 | 2.68019820 |
| 6 | 0.75087593 | -1.54870375 | 1.00994193 |
| 6 | 1.90389454 | -2.50303988 | 0.62832034 |
| 8 | 3.07901537 | -2.85301366 | 3.93202148 |
| 6 | 1.50338541 | -3.59263160 | -0.34639751 |
| 6 | 2.42819923 | -0.11243893 | 3.99631715 |
| 1 | 3.31469066 | -0.53629175 | 2.07133958 |
| 1 | 2.70924422 | -1.87949620 | 0.20420430 |
| 1 | 2.37736093 | -4.20909490 | -0.59184050 |
| 1 | 1.11615016 | -3.14163885 | -1.26809064 |
| 1 | 0.72697822 | -4.22820130 | 0.09446894 |
| 1 | 3.35583537 | -0.30029712 | 4.54887441 |
| 1 | 1.58564232 | -0.44419941 | 4.61578402 |
| 1 | 2.33765897 | 0.96635468 | 3.81250892 |
| 8 | -8.12075666 | -2.86024869 | 5.07799386 |
| 6 | -9.14700685 | -3.74929770 | 5.58090580 |
| 1 | -9.51552289 | -4.40253241 | 4.77994288 |
| 6 | -6.01454069 | -2.48074531 | 4.05848375 |
| 6 | -4.71004642 | -3.11408040 | 3.57610927 |
| 6 | -3.72251205 | -2.07513492 | 3.03437524 |
| 6 | -7.02110175 | -3.49291597 | 4.57130322 |
| 6 | -2.40613403 | -2.69977656 | 2.55888412 |
| 6 | -1.44366679 | -1.65707764 | 2.00850760 |
| 1 | -6.50070515 | -1.91339268 | 3.24718627 |
| 1 | -4.93406366 | -3.85932893 | 2.79810281 |
| 1 | -4.19256665 | -1.52544391 | 2.20008013 |
| 1 | -2.60680400 | -3.44770004 | 1.77573060 |
| 1 | -5.83165793 | -1.74737965 | 4.86023528 |
| 1 | -4.24771572 | -3.67238783 | 4.40462899 |
| 1 | -3.51237115 | -1.32606180 | 3.81800140 |
| 1 | -1.92055320 | -3.23252503 | 3.39154617 |
| 1 | -1.20473282 | -0.90615609 | 2.78171443 |
| 1 | -1.87939027 | -1.14228762 | 1.13751151 |
| 8 | -6.89723809 | -4.70229181 | 4.54359199 |

| | | | |
|---|---|---|---|
| 8 | -0.23810950 | -2.34002209 | 1.60973325 |
| 1 | -9.94439908 | -3.09433901 | 5.94499192 |
| 1 | -8.75169873 | -4.37037873 | 6.39449780 |

Electronic energy = -4057.847634 a.u.
DFT-D3(BJ) dispersion correction = -0.136844 a.u.
Thermal free energy = 0.718673 a.u.
Gibbs free energy = -4057.265805 a.u.
Number of imaginary frequencies = 0.

**Int3 LA isomer/conformer 3**

| Atomic N. | X | Y | Z |
|---|---|---|---|
| 7 | 2.17284985 | 2.49341431 | -0.01785263 |
| 8 | -0.55116770 | 2.55395129 | 1.21624194 |
| 8 | -0.12846522 | 1.34247208 | -1.59772589 |
| 6 | 3.30859979 | 1.94739903 | 0.76859552 |
| 6 | 0.07467883 | 3.30812183 | 2.15954020 |
| 6 | -0.51102873 | 3.47791251 | 3.44659205 |
| 6 | 0.18865531 | 4.27215140 | 4.36723702 |
| 6 | 1.40658096 | 4.88928522 | 4.06767016 |
| 6 | 1.95505891 | 4.73427980 | 2.79904872 |
| 6 | 1.29349381 | 3.96092947 | 1.83878392 |
| 6 | 1.82554319 | 3.89245628 | 0.43253629 |
| 6 | -1.85696298 | 2.82411093 | 3.81651369 |
| 6 | 0.15652927 | 2.37151839 | -2.44051481 |
| 6 | -0.81786406 | 2.80115997 | -3.38680003 |
| 6 | -0.46142521 | 3.85666214 | -4.23839599 |
| 6 | 0.78638466 | 4.48443108 | -4.18828555 |
| 6 | 1.73185925 | 4.04622924 | -3.26850331 |
| 6 | 1.43451487 | 2.98689159 | -2.40250890 |
| 6 | 2.51256345 | 2.45844453 | -1.49177768 |
| 6 | -2.20563488 | 2.13654933 | -3.47813876 |
| 6 | -2.30188517 | 3.19342218 | 5.24426750 |
| 6 | -1.73649885 | 1.28304907 | 3.75614233 |
| 6 | -2.96468241 | 3.30779892 | 2.85026992 |
| 6 | -3.06609654 | 2.75489410 | -4.59627223 |
| 6 | -2.97694239 | 2.32407224 | -2.15019520 |
| 6 | -2.05142636 | 0.62992594 | -3.79566389 |
| 1 | 4.20783607 | 2.55996254 | 0.59495961 |
| 1 | 3.05750454 | 1.96989818 | 1.83452452 |
| 1 | 2.72821432 | 4.51767831 | 0.33295212 |
| 1 | 1.07707617 | 4.26073740 | -0.28421982 |
| 1 | -0.22768379 | 4.41496201 | 5.36270582 |
| 1 | 2.89279523 | 5.22869289 | 2.53538893 |
| 1 | 2.72603640 | 1.40067319 | -1.71177458 |
| 1 | 3.44046831 | 3.03473354 | -1.64019492 |
| 1 | -1.18589250 | 4.21178061 | -4.96881675 |
| 1 | 2.71821658 | 4.51319160 | -3.22261973 |
| 1 | -3.26971434 | 2.71496826 | 5.45070684 |
| 1 | -1.59127377 | 2.84071453 | 6.00580375 |
| 1 | -2.43599858 | 4.27776172 | 5.36814039 |
| 1 | -1.46550185 | 0.93406923 | 2.75415313 |
| 1 | -0.97447258 | 0.92595682 | 4.46421275 |

| | | | |
|---|---|---|---|
| 1 | -2.69778997 | 0.82509340 | 4.03462341 |
| 1 | -3.08643586 | 4.39877906 | 2.91699811 |
| 1 | -2.73780278 | 3.04626361 | 1.81114309 |
| 1 | -3.92424379 | 2.84430937 | 3.12539812 |
| 1 | -4.03580757 | 2.23822529 | -4.62603631 |
| 1 | -2.60147673 | 2.64250974 | -5.58650686 |
| 1 | -3.26689252 | 3.82218216 | -4.42379673 |
| 1 | -2.45711022 | 1.85741017 | -1.30692442 |
| 1 | -3.11035334 | 3.39206513 | -1.92547323 |
| 1 | -3.97529307 | 1.86881796 | -2.23452863 |
| 1 | -1.55020512 | 0.48802626 | -4.76429960 |
| 1 | -1.46996319 | 0.11225861 | -3.02508297 |
| 1 | -3.04544753 | 0.16155695 | -3.85945479 |
| 1 | 3.49461188 | 0.91305460 | 0.45916319 |
| 8 | 0.65675999 | -0.17142554 | 1.08748931 |
| 31 | 0.45099473 | 1.40870431 | 0.15440801 |
| 1 | 1.91328727 | 5.49246823 | 4.82087414 |
| 1 | 1.01277478 | 5.30637575 | -4.86716531 |
| 8 | -0.10107336 | -8.76393733 | -0.39689103 |
| 6 | 0.04278857 | -9.86720001 | 0.52727183 |
| 1 | 0.70426277 | -9.58887061 | 1.35746483 |
| 6 | -0.72513069 | -6.53102270 | -0.88822052 |
| 6 | -1.38912061 | -5.26023347 | -0.35847218 |
| 6 | -1.48835120 | -4.17177896 | -1.43283304 |
| 6 | -0.63808949 | -7.63234838 | 0.14851276 |
| 6 | -2.08876934 | -2.84488797 | -0.94147111 |
| 6 | -1.26441858 | -2.12820700 | 0.12290694 |
| 1 | 0.30026381 | -6.31928321 | -1.23382196 |
| 1 | -0.80797870 | -4.90449035 | 0.50436205 |
| 1 | -0.49035455 | -3.97903904 | -1.85614711 |
| 1 | -3.09158091 | -3.01898752 | -0.51604721 |
| 1 | -1.25999778 | -6.93047816 | -1.76521572 |
| 1 | -2.39228369 | -5.50132541 | 0.02791976 |
| 1 | -2.10629469 | -4.54292270 | -2.26736680 |
| 1 | -2.22067249 | -2.16365515 | -1.79628320 |
| 1 | -1.15647615 | -2.76450886 | 1.01627774 |
| 1 | -1.75993777 | -1.19335995 | 0.43148774 |
| 8 | -0.98621570 | -7.55201533 | 1.31199581 |
| 8 | 0.02813401 | -1.82853106 | -0.44448430 |
| 1 | 0.47965783 | -10.68135328 | -0.05922133 |
| 1 | -0.93407842 | -10.16071428 | 0.93243966 |
| 8 | 2.14846775 | -1.04318809 | -0.40333757 |
| 6 | 1.02228660 | -1.31878158 | 0.43924208 |
| 6 | 1.47166158 | -2.37140096 | 1.47869437 |
| 8 | 1.91309300 | -3.58653876 | 0.79280815 |
| 6 | 2.41098100 | -3.53536581 | -0.46946351 |
| 6 | 2.55854696 | -2.18659868 | -1.18494290 |
| 8 | 2.71968894 | -4.56754198 | -1.03141658 |
| 6 | 4.00243585 | -1.96292690 | -1.62354743 |
| 6 | 2.55869760 | -1.88684054 | 2.42507275 |
| 1 | 0.60162977 | -2.70007054 | 2.06287626 |
| 1 | 1.90874554 | -2.26223176 | -2.07187162 |
| 1 | 4.07022540 | -1.04296781 | -2.21896349 |

| | | | |
|---|---|---|---|
| 1 | 4.33260231 | -2.80845961 | -2.23763397 |
| 1 | 4.66746812 | -1.87379469 | -0.75381421 |
| 1 | 2.17635293 | -1.05797364 | 3.03314948 |
| 1 | 3.44174451 | -1.54290540 | 1.87104682 |
| 1 | 2.85664044 | -2.70725128 | 3.09038274 |

Electronic energy = -4057.847115 a.u.
DFT-D3(BJ) dispersion correction = -0.139350 a.u.
Thermal free energy = 0.720956 a.u.
Gibbs free energy = -4057.265509 a.u.
Number of imaginary frequencies = 0.

**Int3 LA isomer/conformer 4**

| Atomic N. | X | Y | Z |
|---|---|---|---|
| 7 | 1.07052854 | 1.84341208 | 2.97464197 |
| 8 | -1.27147075 | 1.63531802 | 1.12599486 |
| 8 | 1.57929916 | 0.98501049 | 0.12392883 |
| 6 | 1.02719515 | 1.15859050 | 4.29179876 |
| 6 | -1.93999456 | 2.18453231 | 2.17531508 |
| 6 | -3.35896679 | 2.08892955 | 2.25297411 |
| 6 | -3.98010025 | 2.68140207 | 3.36254348 |
| 6 | -3.27003894 | 3.34934135 | 4.36425568 |
| 6 | -1.88706157 | 3.45702342 | 4.26272653 |
| 6 | -1.21788938 | 2.89323963 | 3.17099895 |
| 6 | 0.26052980 | 3.11709836 | 2.99815723 |
| 6 | -4.18280561 | 1.37150986 | 1.16556092 |
| 6 | 2.24697059 | 2.16942136 | 0.06448462 |
| 6 | 2.53712144 | 2.75561916 | -1.20121889 |
| 6 | 3.23471550 | 3.97201374 | -1.19823566 |
| 6 | 3.64585338 | 4.60902358 | -0.02395722 |
| 6 | 3.37351759 | 4.01553011 | 1.20298736 |
| 6 | 2.68982649 | 2.79475022 | 1.25870350 |
| 6 | 2.50825884 | 2.11454518 | 2.59087706 |
| 6 | 2.11583238 | 2.08164977 | -2.52182464 |
| 6 | -5.69546192 | 1.44677027 | 1.44659278 |
| 6 | -3.79988930 | -0.12612489 | 1.10767962 |
| 6 | -3.93723950 | 2.03504326 | -0.21067301 |
| 6 | 2.56651918 | 2.89281958 | -3.75140453 |
| 6 | 0.57533419 | 1.95957926 | -2.59522911 |
| 6 | 2.76859724 | 0.68270392 | -2.62912467 |
| 1 | 1.48715246 | 1.80132487 | 5.05954130 |
| 1 | -0.01457070 | 0.95856755 | 4.56451480 |
| 1 | 0.64918250 | 3.75514367 | 3.80909569 |
| 1 | 0.46663519 | 3.62007925 | 2.04207405 |
| 1 | -5.06272287 | 2.61866365 | 3.45598463 |
| 1 | -1.31533044 | 3.99805402 | 5.02017595 |
| 1 | 2.99750006 | 1.12788021 | 2.59451924 |
| 1 | 2.96324553 | 2.72656778 | 3.38691452 |
| 1 | 3.46494357 | 4.44894889 | -2.14903150 |
| 1 | 3.70216682 | 4.48686239 | 2.13191822 |
| 1 | -6.23437322 | 0.93557527 | 0.63627548 |
| 1 | -5.96879749 | 0.94980167 | 2.38871219 |
| 1 | -6.05817304 | 2.48418351 | 1.48170848 |

| | | | |
|---|---|---|---|
| 1 | -2.73996882 | -0.26595304 | 0.87006137 |
| 1 | -4.00526239 | -0.61604742 | 2.07065831 |
| 1 | -4.39946538 | -0.63242944 | 0.33571438 |
| 1 | -4.24667041 | 3.09018158 | -0.19148858 |
| 1 | -2.88197785 | 1.98795912 | -0.50019055 |
| 1 | -4.53434043 | 1.52309814 | -0.98062092 |
| 1 | 2.25312288 | 2.36245599 | -4.66160012 |
| 1 | 3.65944137 | 3.00511656 | -3.79515546 |
| 1 | 2.11113716 | 3.89342452 | -3.77919071 |
| 1 | 0.17534212 | 1.34845812 | -1.77934008 |
| 1 | 0.10378855 | 2.95168305 | -2.54615493 |
| 1 | 0.28677754 | 1.49436038 | -3.54997295 |
| 1 | 3.86499959 | 0.76864118 | -2.63958136 |
| 1 | 2.47996853 | 0.03519064 | -1.79412780 |
| 1 | 2.46083956 | 0.20015340 | -3.56921477 |
| 1 | 1.57328170 | 0.21124928 | 4.22137062 |
| 8 | -0.13161439 | -0.98869080 | 1.98376192 |
| 31 | 0.31431070 | 0.72105421 | 1.44305607 |
| 1 | -3.79983528 | 3.78836004 | 5.20952419 |
| 1 | 4.17918482 | 5.55811120 | -0.07453992 |
| 8 | -2.20469729 | -4.07445365 | -7.30181291 |
| 6 | -2.02501906 | -3.92362813 | -8.73046823 |
| 1 | -2.04189519 | -2.86261195 | -9.00979738 |
| 6 | -1.52315898 | -3.67909650 | -5.06618343 |
| 6 | -0.44861550 | -3.08376308 | -4.15734891 |
| 6 | -0.76283500 | -3.28240415 | -2.67074981 |
| 6 | -1.24296652 | -3.47142885 | -6.54217470 |
| 6 | 0.31532047 | -2.68681263 | -1.75878236 |
| 6 | -0.01745293 | -2.86114447 | -0.28341025 |
| 1 | -2.51144810 | -3.24015316 | -4.85049753 |
| 1 | -0.34163883 | -2.01046279 | -4.37620680 |
| 1 | -1.73836832 | -2.82035080 | -2.43864877 |
| 1 | 0.43648121 | -1.61345075 | -1.97032688 |
| 1 | -1.63939696 | -4.76112978 | -4.89271591 |
| 1 | 0.52445966 | -3.53905543 | -4.39753475 |
| 1 | -0.87305212 | -4.36002598 | -2.45837610 |
| 1 | 1.28665723 | -3.16384382 | -1.96470272 |
| 1 | -0.14670194 | -3.93242792 | -0.04637276 |
| 1 | -0.95188750 | -2.32995782 | -0.03486073 |
| 8 | -0.30520176 | -2.85645684 | -7.01301211 |
| 8 | 1.07350410 | -2.31918473 | 0.48999867 |
| 1 | -2.86439879 | -4.45798901 | -9.18570238 |
| 1 | -1.06901677 | -4.36054597 | -9.04535814 |
| 8 | 2.02786865 | -1.57457819 | 2.40463857 |
| 6 | 0.78191470 | -2.00073483 | 1.84954504 |
| 6 | 0.29988961 | -3.22941495 | 2.65066160 |
| 8 | 1.29756886 | -4.29551613 | 2.57798120 |
| 6 | 2.62188018 | -4.01046753 | 2.44328983 |
| 6 | 3.07781196 | -2.55694511 | 2.25945374 |
| 8 | 3.42702254 | -4.91813427 | 2.43003162 |
| 6 | 4.18549873 | -2.20373035 | 3.24535840 |
| 6 | -0.02975884 | -2.93225146 | 4.10548205 |
| 1 | -0.58255463 | -3.65572586 | 2.15398141 |

| | | | |
|---|---|---|---|
| 1 | 3.46708288 | -2.50959789 | 1.22912173 |
| 1 | 4.57029590 | -1.19925038 | 3.02590644 |
| 1 | 5.00269686 | -2.92735328 | 3.14992762 |
| 1 | 3.81174598 | -2.22512511 | 4.27813826 |
| 1 | -0.87272079 | -2.23275578 | 4.15981143 |
| 1 | 0.83211203 | -2.49060953 | 4.62272311 |
| 1 | -0.30612457 | -3.86421277 | 4.61506748 |

Electronic energy = -4057.847469 a.u.
DFT-D3(BJ) dispersion correction = -0.137361 a.u.
Thermal free energy = 0.720229 a.u.
Gibbs free energy = -4057.264601 a.u.
Number of imaginary frequencies = 0.

**Int3 LA isomer/conformer 5**

| Atomic N. | X | Y | Z |
|---|---|---|---|
| 7 | -1.23394700 | 0.69125380 | 2.11517390 |
| 8 | -2.55223219 | -1.41047493 | 0.43305126 |
| 8 | -0.48821472 | 0.52400615 | -0.79557227 |
| 6 | -0.65727997 | 0.34035312 | 3.43878291 |
| 6 | -3.32481618 | -1.57832532 | 1.53905903 |
| 6 | -4.07143429 | -2.77703418 | 1.72151135 |
| 6 | -4.84650507 | -2.87435834 | 2.88679752 |
| 6 | -4.91157817 | -1.85814175 | 3.84465702 |
| 6 | -4.19668642 | -0.68266141 | 3.64097785 |
| 6 | -3.41564951 | -0.52970424 | 2.49033670 |
| 6 | -2.74192959 | 0.78339548 | 2.20255087 |
| 6 | -4.03674331 | -3.91840558 | 0.68654238 |
| 6 | -1.05160991 | 1.75991891 | -0.84590460 |
| 6 | -1.47829486 | 2.29987859 | -2.09359128 |
| 6 | -2.03139928 | 3.58844436 | -2.07754505 |
| 6 | -2.17793706 | 4.33769118 | -0.90679500 |
| 6 | -1.75366900 | 3.80006865 | 0.30256293 |
| 6 | -1.18090180 | 2.52299701 | 0.34283858 |
| 6 | -0.63783032 | 2.00383042 | 1.64842750 |
| 6 | -1.34713553 | 1.50672937 | -3.40907574 |
| 6 | -4.96255072 | -5.08329280 | 1.08491729 |
| 6 | -2.60339910 | -4.48830916 | 0.56853253 |
| 6 | -4.51599003 | -3.39956320 | -0.69026306 |
| 6 | -1.85299051 | 2.31358097 | -4.61992324 |
| 6 | -2.18877035 | 0.21080984 | -3.33625715 |
| 6 | 0.13675614 | 1.16032555 | -3.67649255 |
| 1 | -0.92084640 | 1.12156449 | 4.16964866 |
| 1 | -1.05798002 | -0.62227773 | 3.77171518 |
| 1 | -2.98898646 | 1.52258806 | 2.98207148 |
| 1 | -3.07522382 | 1.18613900 | 1.23518583 |
| 1 | -5.42235530 | -3.78133685 | 3.06155488 |
| 1 | -4.25439633 | 0.13498415 | 4.36306864 |
| 1 | 0.44632973 | 1.82131820 | 1.58212096 |
| 1 | -0.80949691 | 2.74917108 | 2.44194109 |
| 1 | -2.37044147 | 4.02802379 | -3.01361507 |
| 1 | -1.84976293 | 4.37264374 | 1.22762225 |
| 1 | -4.91239426 | -5.85974452 | 0.30846871 |

| | | | |
|---|---|---|---|
| 1 | -4.66068384 | -5.54769455 | 2.03492960 |
| 1 | -6.01243013 | -4.76733924 | 1.16928926 |
| 1 | -1.88723797 | -3.72776013 | 0.24008407 |
| 1 | -2.26477322 | -4.88789166 | 1.53562577 |
| 1 | -2.59253360 | -5.31287793 | -0.16067627 |
| 1 | -5.54954116 | -3.02913874 | -0.62564870 |
| 1 | -3.87872562 | -2.58959019 | -1.06109766 |
| 1 | -4.49751588 | -4.22100392 | -1.42252066 |
| 1 | -1.72816678 | 1.70800431 | -5.52868506 |
| 1 | -1.28708568 | 3.24549321 | -4.76294129 |
| 1 | -2.92037547 | 2.56322451 | -4.53511331 |
| 1 | -1.85492945 | -0.44803382 | -2.52781611 |
| 1 | -3.25020610 | 0.44658640 | -3.17365880 |
| 1 | -2.10584934 | -0.33943636 | -4.28567970 |
| 1 | 0.73774486 | 2.07752936 | -3.76500782 |
| 1 | 0.55413052 | 0.54372467 | -2.87320670 |
| 1 | 0.22476826 | 0.60580532 | -4.62297996 |
| 1 | 0.43051852 | 0.26203948 | 3.34893518 |
| 8 | 0.35141152 | -2.01249135 | 0.65007079 |
| 31 | -0.87262980 | -0.63752648 | 0.59912663 |
| 1 | -5.52520088 | -1.98644678 | 4.73622961 |
| 1 | -2.61974551 | 5.33309451 | -0.94626974 |
| 8 | 5.89902751 | 5.13432194 | -4.15232339 |
| 6 | 6.25760790 | 6.52487371 | -4.33618300 |
| 1 | 5.36067084 | 7.15690699 | -4.32887555 |
| 6 | 4.92586394 | 3.38726239 | -2.88029779 |
| 6 | 4.17810544 | 3.01877709 | -1.59970933 |
| 6 | 3.86180535 | 1.52182990 | -1.51793497 |
| 6 | 5.25406149 | 4.86465199 | -2.97907366 |
| 6 | 3.10827598 | 1.15184369 | -0.23502675 |
| 6 | 2.77345744 | -0.33123725 | -0.17596654 |
| 1 | 4.34377742 | 3.11073483 | -3.77441233 |
| 1 | 3.24500209 | 3.59993297 | -1.54408901 |
| 1 | 3.26207928 | 1.22582574 | -2.39554722 |
| 1 | 2.17636756 | 1.73518815 | -0.17417168 |
| 1 | 5.87242371 | 2.82816098 | -2.96293753 |
| 1 | 4.77718245 | 3.32378755 | -0.72805824 |
| 1 | 4.80005668 | 0.94327367 | -1.57305058 |
| 1 | 3.71808800 | 1.41516189 | 0.64422917 |
| 1 | 3.69590839 | -0.92687535 | -0.28255235 |
| 1 | 2.07472154 | -0.60016891 | -0.98531106 |
| 8 | 4.99430357 | 5.71274663 | -2.14680615 |
| 8 | 2.16819924 | -0.60978880 | 1.10929008 |
| 1 | 6.75542498 | 6.56904585 | -5.30960032 |
| 1 | 6.93406976 | 6.85569660 | -3.53805973 |
| 8 | 3.75433362 | -3.01371382 | 1.47731472 |
| 6 | 3.84031118 | -2.54610711 | 2.75143128 |
| 6 | 2.59643791 | -1.96808693 | 3.43838539 |
| 8 | 1.37201405 | -2.03798554 | 2.67812091 |
| 6 | 1.56311306 | -1.90455701 | 1.27133124 |
| 6 | 2.46199431 | -3.06602952 | 0.79569935 |
| 8 | 4.91270403 | -2.55278980 | 3.31947903 |
| 6 | 1.82829966 | -4.43684961 | 0.97942105 |

| | | | |
|---|---|---|---|
| 6 | 2.34725233 | -2.65610483 | 4.77760600 |
| 1 | 2.85203795 | -0.90764135 | 3.61051926 |
| 1 | 2.71359224 | -2.91710911 | -0.26272856 |
| 1 | 2.54771746 | -5.21139311 | 0.68406040 |
| 1 | 0.93404653 | -4.52008408 | 0.35047931 |
| 1 | 1.53415501 | -4.60077677 | 2.02373516 |
| 1 | 3.24782403 | -2.58893167 | 5.39842897 |
| 1 | 2.09137716 | -3.71341547 | 4.62935426 |
| 1 | 1.51365859 | -2.16473303 | 5.29575874 |

Electronic energy = -4057.846078 a.u.
DFT-D3(BJ) dispersion correction = -0.138724 a.u.
Thermal free energy = 0.720962 a.u.
Gibbs free energy = -4057.263840 a.u.
Number of imaginary frequencies = 0.

**Int3 LA isomer/conformer 6**

| Atomic N. | X | Y | Z |
|---|---|---|---|
| 8 | 3.65283664 | -0.80253584 | -3.55597312 |
| 6 | 3.88943042 | -1.98094028 | -2.91095694 |
| 6 | 3.66733033 | -1.92980989 | -1.39525616 |
| 8 | 2.44175545 | -1.24532892 | -1.05725300 |
| 6 | 3.29508794 | 0.33308035 | -2.71764941 |
| 8 | 4.26784605 | -2.95247310 | -3.52586741 |
| 6 | 2.98582571 | 1.48990702 | -3.64702726 |
| 6 | 3.61053637 | -3.31357940 | -0.77486701 |
| 1 | 4.52770757 | -1.37182104 | -0.96940110 |
| 1 | 4.16672771 | 0.57749496 | -2.08760442 |
| 1 | 3.86376333 | 1.71424255 | -4.26581432 |
| 1 | 2.72984391 | 2.38225523 | -3.06151605 |
| 1 | 2.14325792 | 1.23061864 | -4.29831172 |
| 1 | 4.54070022 | -3.85401646 | -0.98546621 |
| 1 | 2.77456239 | -3.88403034 | -1.19723298 |
| 1 | 3.47702348 | -3.22894256 | 0.31087517 |
| 7 | -1.72392190 | 1.30497662 | -1.91609628 |
| 8 | -1.02909588 | -0.90069468 | 0.02044714 |
| 8 | -1.59489552 | -1.52128646 | -2.92536533 |
| 6 | -0.87501900 | 2.52097986 | -1.96178909 |
| 6 | -1.30066158 | 0.04022157 | 0.96290392 |
| 6 | -0.94226783 | -0.19429861 | 2.32286263 |
| 6 | -1.17655439 | 0.84650355 | 3.23444194 |
| 6 | -1.76492235 | 2.06050348 | 2.86935904 |
| 6 | -2.19588721 | 2.23605884 | 1.55871452 |
| 6 | -1.99503129 | 1.22795206 | 0.60911390 |
| 6 | -2.66017034 | 1.34294384 | -0.73797155 |
| 6 | -0.36420900 | -1.54486607 | 2.79250566 |
| 6 | -2.91241206 | -1.32669060 | -3.17329792 |
| 6 | -3.77763139 | -2.44432975 | -3.35905854 |
| 6 | -5.12678268 | -2.17249421 | -3.62608980 |
| 6 | -5.63813487 | -0.87393443 | -3.71404811 |
| 6 | -4.78150815 | 0.20743681 | -3.54498393 |
| 6 | -3.42166388 | -0.00679613 | -3.28832684 |
| 6 | -2.49106968 | 1.17472986 | -3.21575588 |

| | | | |
|---|---|---|---|
| 6 | -3.25572118 | -3.89191793 | -3.27355706 |
| 6 | -0.17586316 | -1.58850514 | 4.32098796 |
| 6 | 1.01565158 | -1.80321936 | 2.14890323 |
| 6 | -1.33959185 | -2.68764097 | 2.41985421 |
| 6 | -4.37048101 | -4.92067798 | -3.54158949 |
| 6 | -2.69984939 | -4.17558425 | -1.85785732 |
| 6 | -2.15145445 | -4.12200211 | -4.33292554 |
| 1 | -1.50919418 | 3.41057279 | -2.10556821 |
| 1 | -0.31605947 | 2.61121520 | -1.02530543 |
| 1 | -3.24619697 | 2.27563306 | -0.79207598 |
| 1 | -3.35594530 | 0.50352231 | -0.88639251 |
| 1 | -0.89059338 | 0.70848018 | 4.27554060 |
| 1 | -2.71831117 | 3.14935294 | 1.26525210 |
| 1 | -1.72183375 | 1.11585553 | -4.00248975 |
| 1 | -3.06071613 | 2.10660473 | -3.36635553 |
| 1 | -5.81379759 | -3.00491645 | -3.76669501 |
| 1 | -5.15679363 | 1.23022553 | -3.62462743 |
| 1 | 0.20918971 | -2.57868661 | 4.60323314 |
| 1 | 0.55034142 | -0.84114694 | 4.67293467 |
| 1 | -1.12185139 | -1.43485089 | 4.85978869 |
| 1 | 0.96390194 | -1.80508614 | 1.05581122 |
| 1 | 1.73618644 | -1.03321228 | 2.46376637 |
| 1 | 1.40200189 | -2.77939733 | 2.48097298 |
| 1 | -2.31636786 | -2.53532566 | 2.90156288 |
| 1 | -1.49320683 | -2.75199273 | 1.33723519 |
| 1 | -0.93425986 | -3.64793325 | 2.77250888 |
| 1 | -3.94366727 | -5.93176313 | -3.48062724 |
| 1 | -4.80658074 | -4.80632127 | -4.54457467 |
| 1 | -5.18004461 | -4.85935966 | -2.79976139 |
| 1 | -1.85975145 | -3.51773023 | -1.61182320 |
| 1 | -3.48281517 | -4.03839782 | -1.09791149 |
| 1 | -2.34990988 | -5.21734891 | -1.79965691 |
| 1 | -2.54951923 | -3.96549608 | -5.34618177 |
| 1 | -1.30096031 | -3.44813737 | -4.18512505 |
| 1 | -1.78888142 | -5.15917112 | -4.27022497 |
| 1 | -0.16376663 | 2.43414756 | -2.79086086 |
| 8 | 0.98502155 | -0.24146668 | -2.52948804 |
| 31 | -0.66775645 | -0.43869099 | -1.74110048 |
| 1 | -1.90519637 | 2.84646790 | 3.61101924 |
| 1 | -6.69722166 | -0.71587402 | -3.91701509 |
| 8 | 3.63964947 | 5.05788004 | 6.54506046 |
| 6 | 3.15143640 | 5.99276767 | 7.53725288 |
| 1 | 2.95661665 | 6.97092356 | 7.07986878 |
| 6 | 3.37481654 | 3.82765268 | 4.53654660 |
| 6 | 2.47720507 | 3.55198269 | 3.33111047 |
| 6 | 3.08825580 | 2.53121508 | 2.36525577 |
| 6 | 2.77738029 | 4.82303680 | 5.51283886 |
| 6 | 2.17696563 | 2.24309981 | 1.16686139 |
| 6 | 2.78195610 | 1.22211730 | 0.21293020 |
| 1 | 4.35990847 | 4.21074940 | 4.22416563 |
| 1 | 2.28016031 | 4.49658629 | 2.80102171 |
| 1 | 4.06743967 | 2.89868041 | 2.01096831 |
| 1 | 1.97951906 | 3.17841407 | 0.61795906 |

| | | | |
|---|---|---|---|
| 1 | 3.58533282 | 2.89995258 | 5.09402637 |
| 1 | 1.49768951 | 3.19230257 | 3.68018965 |
| 1 | 3.28933121 | 1.59011170 | 2.90604242 |
| 1 | 1.20583374 | 1.86512917 | 1.52112774 |
| 1 | 2.98660878 | 0.27404700 | 0.73385688 |
| 1 | 3.73270168 | 1.60837418 | -0.19886478 |
| 8 | 1.68591751 | 5.35151471 | 5.41730149 |
| 8 | 1.83277002 | 0.99234219 | -0.84985270 |
| 1 | 3.94855890 | 6.06523850 | 8.28328819 |
| 1 | 2.22521740 | 5.62035152 | 7.99263245 |
| 6 | 2.11819777 | -0.05573077 | -1.78941227 |

Electronic energy = -4057.843025 a.u.
DFT-D3(BJ) dispersion correction = -0.139640 a.u.
Thermal free energy = 0.720184 a.u.
Gibbs free energy = -4057.262481 a.u.
Number of imaginary frequencies = 0.

**Int3 LA isomer/conformer 7**

| Atomic N. | X | Y | Z |
|---|---|---|---|
| 7 | 0.78719635 | 2.13742696 | 1.21352841 |
| 8 | -2.19211721 | 2.28509981 | 1.08340425 |
| 8 | -0.57913606 | 1.19131273 | -1.30816646 |
| 6 | 1.43576565 | 1.48750304 | 2.38088700 |
| 6 | -2.05014860 | 2.95008094 | 2.25950486 |
| 6 | -3.15609235 | 3.08754178 | 3.14682822 |
| 6 | -2.93380920 | 3.78813432 | 4.34162733 |
| 6 | -1.69669286 | 4.34613780 | 4.67716989 |
| 6 | -0.63253523 | 4.22743628 | 3.78941453 |
| 6 | -0.80256222 | 3.54711314 | 2.57872424 |
| 6 | 0.30795193 | 3.52571134 | 1.56374734 |
| 6 | -4.54035849 | 2.49897956 | 2.81091207 |
| 6 | 0.07833991 | 2.24394805 | -1.86030953 |
| 6 | -0.35178532 | 2.76482460 | -3.11483027 |
| 6 | 0.37361886 | 3.84152085 | -3.64449031 |
| 6 | 1.47509177 | 4.40698783 | -2.99564791 |
| 6 | 1.89100606 | 3.88119090 | -1.77796577 |
| 6 | 1.21280125 | 2.79592352 | -1.20950631 |
| 6 | 1.75455385 | 2.17294535 | 0.05194733 |
| 6 | -1.56443579 | 2.17448877 | -3.86173368 |
| 6 | -5.58176178 | 2.82268163 | 3.89868371 |
| 6 | -4.45317006 | 0.95851254 | 2.69951526 |
| 6 | -5.06193167 | 3.09765165 | 1.48176302 |
| 6 | -1.80590151 | 2.87943696 | -5.21001059 |
| 6 | -2.84751123 | 2.35100940 | -3.01540704 |
| 6 | -1.32944281 | 0.67461507 | -4.16218254 |
| 1 | 2.33316492 | 2.05675495 | 2.67226508 |
| 1 | 0.73362745 | 1.46713445 | 3.22148722 |
| 1 | 1.17307065 | 4.10401310 | 1.92853704 |
| 1 | -0.02537797 | 3.97416763 | 0.61642479 |
| 1 | -3.75614263 | 3.90310372 | 5.04546476 |
| 1 | 0.33510408 | 4.67966859 | 4.01876297 |
| 1 | 2.03553061 | 1.12248579 | -0.11902267 |

| | | | |
|---|---|---|---|
| 1 | 2.65533165 | 2.71905625 | 0.37747405 |
| 1 | 0.06672616 | 4.26509364 | -4.59894107 |
| 1 | 2.75798539 | 4.29924074 | -1.26143392 |
| 1 | -6.55061605 | 2.39592357 | 3.60273036 |
| 1 | -5.31437103 | 2.38840682 | 4.87301354 |
| 1 | -5.72287129 | 3.90566210 | 4.02673199 |
| 1 | -3.76287657 | 0.64477926 | 1.90931670 |
| 1 | -4.11337233 | 0.52052404 | 3.64948014 |
| 1 | -5.44888126 | 0.54687408 | 2.47395458 |
| 1 | -5.16832000 | 4.18902152 | 1.56538670 |
| 1 | -4.38731705 | 2.87708844 | 0.64749236 |
| 1 | -6.05287404 | 2.67805857 | 1.25108180 |
| 1 | -2.67018146 | 2.41443195 | -5.70496853 |
| 1 | -0.94529077 | 2.78281777 | -5.88772838 |
| 1 | -2.03378735 | 3.94793870 | -5.08532902 |
| 1 | -2.77746957 | 1.82892517 | -2.05539974 |
| 1 | -3.03903194 | 3.41550430 | -2.81761687 |
| 1 | -3.71171606 | 1.94779366 | -3.56481640 |
| 1 | -0.44092768 | 0.54182909 | -4.79687172 |
| 1 | -1.19253005 | 0.09424225 | -3.24345484 |
| 1 | -2.19506576 | 0.26763970 | -4.70679039 |
| 1 | 1.71411220 | 0.46267899 | 2.11107616 |
| 8 | -1.15830230 | -0.50229122 | 1.32984593 |
| 31 | -0.85107145 | 1.12700468 | 0.51894385 |
| 1 | -1.57464802 | 4.87671992 | 5.62138626 |
| 1 | 2.00102527 | 5.24956310 | -3.44425368 |
| 8 | 7.31020585 | -6.41474231 | -2.00817796 |
| 6 | 8.49367437 | -7.13688563 | -1.59065170 |
| 1 | 9.26988651 | -6.43915767 | -1.25174505 |
| 6 | 5.52342995 | -4.91928965 | -1.57197210 |
| 6 | 4.68787022 | -4.24713310 | -0.48277190 |
| 6 | 3.51867389 | -3.44014290 | -1.05685303 |
| 6 | 6.73247763 | -5.65743630 | -1.02999378 |
| 6 | 2.65773623 | -2.78928907 | 0.03321517 |
| 6 | 1.52790332 | -1.95939527 | -0.56851564 |
| 1 | 5.89562734 | -4.17308117 | -2.29476558 |
| 1 | 5.33689788 | -3.59341923 | 0.11925562 |
| 1 | 3.90968411 | -2.66074089 | -1.73436147 |
| 1 | 3.28796887 | -2.14245383 | 0.66613274 |
| 1 | 4.92183776 | -5.62696652 | -2.16300854 |
| 1 | 4.30553200 | -5.01448820 | 0.20807909 |
| 1 | 2.88523422 | -4.09808237 | -1.67599512 |
| 1 | 2.23813641 | -3.57265727 | 0.68237924 |
| 1 | 0.84271735 | -2.58031307 | -1.15858397 |
| 1 | 1.93834516 | -1.19584001 | -1.24884248 |
| 8 | 7.15864208 | -5.59304079 | 0.10759822 |
| 8 | 0.78085686 | -1.21936280 | 0.43329646 |
| 1 | 8.82553560 | -7.68559064 | -2.47725776 |
| 1 | 8.25275090 | -7.82779604 | -0.77308688 |
| 8 | -0.39641470 | -4.04016207 | 1.24489410 |
| 6 | -1.19983493 | -4.36283167 | 0.18442648 |
| 6 | -2.05197560 | -3.19279651 | -0.31905558 |
| 8 | -1.20528994 | -2.02686189 | -0.42531271 |

| | | | |
|---|---|---|---|
| 6 | -0.55855276 | -1.61079135 | 0.77785912 |
| 6 | -0.64653716 | -2.73436447 | 1.84627545 |
| 8 | -1.16469891 | -5.47324190 | -0.29664639 |
| 6 | 0.30367943 | -2.57051327 | 3.01516413 |
| 6 | -2.65697067 | -3.45548967 | -1.68456739 |
| 1 | -2.86072239 | -3.00359255 | 0.41343553 |
| 1 | -1.68352271 | -2.71984380 | 2.21725704 |
| 1 | 0.15090568 | -3.38771401 | 3.73133722 |
| 1 | 0.09444914 | -1.61891056 | 3.51918345 |
| 1 | 1.34777363 | -2.57833010 | 2.68272694 |
| 1 | -3.30464992 | -4.33901439 | -1.64041874 |
| 1 | -1.86997090 | -3.64209785 | -2.42528743 |
| 1 | -3.24886076 | -2.58620934 | -1.99628093 |

Electronic energy = -4057.845570 a.u.
DFT-D3(BJ) dispersion correction = -0.136635 a.u.
Thermal free energy = 0.719794 a.u.
Gibbs free energy = -4057.262411 a.u.
Number of imaginary frequencies = 0.

**Int3 LA isomer/conformer 8**

| Atomic N. | X | Y | Z |
|---|---|---|---|
| 7 | -1.61964403 | 0.73049854 | 1.80355809 |
| 8 | -2.29561268 | -2.00739489 | 0.82717326 |
| 8 | -0.71070629 | -0.05251133 | -0.96807904 |
| 6 | -0.93576510 | 0.90115721 | 3.11139280 |
| 6 | -2.99444617 | -2.07698780 | 1.99324334 |
| 6 | -3.37698174 | -3.33878170 | 2.53100077 |
| 6 | -4.10011727 | -3.32646794 | 3.73314005 |
| 6 | -4.45005052 | -2.14824980 | 4.39909364 |
| 6 | -4.08857111 | -0.92228667 | 3.85087007 |
| 6 | -3.37626779 | -0.87694042 | 2.64744325 |
| 6 | -3.09128791 | 0.44606309 | 1.99012099 |
| 6 | -3.01632176 | -4.66291167 | 1.82946860 |
| 6 | -1.60074032 | 0.90318594 | -1.34967412 |
| 6 | -2.08780578 | 0.92243402 | -2.68818613 |
| 6 | -2.98854417 | 1.94246943 | -3.02709163 |
| 6 | -3.41501818 | 2.91200254 | -2.11514097 |
| 6 | -2.92444625 | 2.88629741 | -0.81477076 |
| 6 | -2.01071862 | 1.89925407 | -0.42530350 |
| 6 | -1.40724455 | 1.96266668 | 0.95506468 |
| 6 | -1.65240289 | -0.13353062 | -3.72393354 |
| 6 | -3.58145650 | -5.88341957 | 2.58031815 |
| 6 | -1.48013813 | -4.83109484 | 1.76811936 |
| 6 | -3.61140350 | -4.68556172 | 0.40118099 |
| 6 | -2.28309958 | 0.12423764 | -5.10561842 |
| 6 | -2.10358777 | -1.54160304 | -3.26889035 |
| 6 | -0.11618804 | -0.10308650 | -3.90804897 |
| 1 | -1.34959105 | 1.77673493 | 3.63677280 |
| 1 | -1.09034851 | 0.00535723 | 3.72241438 |
| 1 | -3.52593411 | 1.27013868 | 2.57986041 |
| 1 | -3.53045416 | 0.47944559 | 0.98230468 |
| 1 | -4.40035774 | -4.27441434 | 4.17576052 |

| | | | |
|---|---|---|---|
| 1 | -4.37315626 | 0.01148134 | 4.34126264 |
| 1 | -0.31476614 | 2.08526441 | 0.89859113 |
| 1 | -1.81908050 | 2.82735368 | 1.50111272 |
| 1 | -3.38310429 | 1.98219135 | -4.04061362 |
| 1 | -3.23532840 | 3.64239716 | -0.09042501 |
| 1 | -3.30788472 | -6.79596677 | 2.03212993 |
| 1 | -3.16851642 | -5.97402831 | 3.59539109 |
| 1 | -4.67862238 | -5.85512744 | 2.65028756 |
| 1 | -1.00529416 | -4.01718242 | 1.21048656 |
| 1 | -1.04865428 | -4.85096576 | 2.77887133 |
| 1 | -1.22751055 | -5.78171638 | 1.27528911 |
| 1 | -4.70808762 | -4.60926317 | 0.43706258 |
| 1 | -3.22562277 | -3.86298620 | -0.21070234 |
| 1 | -3.35533966 | -5.63616834 | -0.09068122 |
| 1 | -1.93253721 | -0.64653082 | -5.80641944 |
| 1 | -1.99347706 | 1.10199596 | -5.51718988 |
| 1 | -3.38084901 | 0.06857592 | -5.07793585 |
| 1 | -1.65370861 | -1.82435537 | -2.31138647 |
| 1 | -3.19693523 | -1.58372563 | -3.16199024 |
| 1 | -1.80796197 | -2.28737654 | -4.02222781 |
| 1 | 0.21164177 | 0.88484336 | -4.26392132 |
| 1 | 0.40719212 | -0.32973882 | -2.97283820 |
| 1 | 0.18038491 | -0.84730984 | -4.66268271 |
| 1 | 0.13722054 | 1.04380488 | 2.93920827 |
| 8 | 0.60814214 | -1.61166664 | 1.46723294 |
| 31 | -0.87198125 | -0.82217931 | 0.70726041 |
| 1 | -5.00737949 | -2.19455681 | 5.33461836 |
| 1 | -4.12417531 | 3.67947379 | -2.42472815 |
| 8 | 5.20118773 | 7.49744366 | -2.26551072 |
| 6 | 5.37785725 | 8.92176358 | -2.07251951 |
| 1 | 4.40437225 | 9.42407502 | -2.00988157 |
| 6 | 4.38184186 | 5.38782041 | -1.56258682 |
| 6 | 3.64040689 | 4.61959645 | -0.46942563 |
| 6 | 3.50013906 | 3.12870526 | -0.79338359 |
| 6 | 4.51671149 | 6.86926765 | -1.26576505 |
| 6 | 2.75558426 | 2.35454374 | 0.30088542 |
| 6 | 2.60917143 | 0.88029430 | -0.04754797 |
| 1 | 3.87201380 | 5.28595694 | -2.53460325 |
| 1 | 2.64471315 | 5.06618541 | -0.32520953 |
| 1 | 2.96847823 | 3.00890339 | -1.75277744 |
| 1 | 1.75804781 | 2.80012772 | 0.44703145 |
| 1 | 5.39442517 | 4.98182305 | -1.71934256 |
| 1 | 4.16960436 | 4.74743071 | 0.48746685 |
| 1 | 4.50129864 | 2.68722501 | -0.93851311 |
| 1 | 3.28964828 | 2.45270541 | 1.26005056 |
| 1 | 3.59801308 | 0.41627946 | -0.16833368 |
| 1 | 2.05315203 | 0.74977100 | -0.98773884 |
| 8 | 4.08439574 | 7.44337166 | -0.28424894 |
| 8 | 1.87576842 | 0.23609527 | 1.02931961 |
| 1 | 5.93603316 | 9.26521352 | -2.94867855 |
| 1 | 5.94097096 | 9.11716277 | -1.15130418 |
| 8 | 2.94056726 | -3.15237694 | 2.15230503 |
| 6 | 2.69697265 | -3.86031904 | 1.01774763 |

| | | | |
|---|---|---|---|
| 6 | 2.20744896 | -3.13143429 | -0.24032919 |
| 8 | 2.27133085 | -1.69211074 | -0.17467600 |
| 6 | 1.86834023 | -1.19201458 | 1.09609130 |
| 6 | 2.87172254 | -1.69074426 | 2.16236129 |
| 8 | 2.82121499 | -5.06746622 | 1.03024020 |
| 6 | 4.28578189 | -1.14277815 | 2.03891669 |
| 6 | 2.98847644 | -3.56625433 | -1.47415668 |
| 1 | 1.15151971 | -3.43781537 | -0.34622347 |
| 1 | 2.45231321 | -1.44939949 | 3.14905132 |
| 1 | 4.92058483 | -1.61911956 | 2.79691722 |
| 1 | 4.29677301 | -0.05891595 | 2.21114429 |
| 1 | 4.70991149 | -1.35587148 | 1.04866040 |
| 1 | 2.93875136 | -4.65609528 | -1.57410368 |
| 1 | 4.04114005 | -3.26393544 | -1.39333785 |
| 1 | 2.55395950 | -3.09834771 | -2.36661725 |

Electronic energy = -4057.843488 a.u.
DFT-D3(BJ) dispersion correction = -0.137587 a.u.
Thermal free energy = 0.719896 a.u.
Gibbs free energy = -4057.261180 a.u.
Number of imaginary frequencies = 0.

**Int3 LA isomer/conformer 9**

| Atomic N. | X | Y | Z |
|---|---|---|---|
| 7 | -0.97312542 | 0.63403754 | -2.43039937 |
| 8 | -1.50293714 | -0.45597271 | 0.32098037 |
| 8 | -0.75456625 | -2.33320157 | -2.01471816 |
| 6 | -0.06623894 | 1.77949759 | -2.70226498 |
| 6 | -1.84318126 | 0.83339924 | 0.59547013 |
| 6 | -1.91176884 | 1.29294212 | 1.94056801 |
| 6 | -2.23465697 | 2.64502226 | 2.13374154 |
| 6 | -2.50855432 | 3.51994964 | 1.07769311 |
| 6 | -2.49076662 | 3.04246769 | -0.22889607 |
| 6 | -2.17421655 | 1.70204149 | -0.47649011 |
| 6 | -2.28598253 | 1.13485823 | -1.86455177 |
| 6 | -1.66719546 | 0.35052316 | 3.13580265 |
| 6 | -1.87810741 | -2.36653557 | -2.78384981 |
| 6 | -2.74001265 | -3.50028044 | -2.76119175 |
| 6 | -3.86622755 | -3.46048764 | -3.59668302 |
| 6 | -4.16199245 | -2.37479100 | -4.42569689 |
| 6 | -3.30824241 | -1.27830993 | -4.44180099 |
| 6 | -2.16406490 | -1.27061613 | -3.63523289 |
| 6 | -1.20312612 | -0.11719413 | -3.72756688 |
| 6 | -2.45891515 | -4.72352556 | -1.86648113 |
| 6 | -1.86546375 | 1.07324627 | 4.48186056 |
| 6 | -0.22122135 | -0.19680519 | 3.11417613 |
| 6 | -2.67418275 | -0.82364433 | 3.08477236 |
| 6 | -3.54202206 | -5.80851672 | -2.01840538 |
| 6 | -2.43437067 | -4.30822342 | -0.37666454 |
| 6 | -1.10799411 | -5.36318255 | -2.26333984 |
| 1 | -0.50382691 | 2.40846448 | -3.49395937 |
| 1 | 0.04449014 | 2.37726525 | -1.79169857 |
| 1 | -2.68539462 | 1.89042908 | -2.56090707 |

| | | | |
|---|---|---|---|
| 1 | -2.96505909 | 0.27050865 | -1.87205204 |
| 1 | -2.26071742 | 3.04032900 | 3.14726370 |
| 1 | -2.74294087 | 3.69865017 | -1.06520715 |
| 1 | -0.20695015 | -0.45761522 | -4.05177882 |
| 1 | -1.56761898 | 0.61031260 | -4.47088796 |
| 1 | -4.54921873 | -4.30767811 | -3.59905091 |
| 1 | -3.51466944 | -0.42266114 | -5.08840062 |
| 1 | -1.69408806 | 0.35631946 | 5.29751569 |
| 1 | -1.16047005 | 1.90567329 | 4.61759752 |
| 1 | -2.88844802 | 1.46126814 | 4.59378587 |
| 1 | -0.00142925 | -0.75255944 | 2.19670206 |
| 1 | 0.50309062 | 0.62483835 | 3.20560131 |
| 1 | -0.07119672 | -0.87231111 | 3.96997360 |
| 1 | -3.70760081 | -0.45082247 | 3.13693103 |
| 1 | -2.56208840 | -1.41480484 | 2.16906502 |
| 1 | -2.51122384 | -1.48648244 | 3.94802533 |
| 1 | -3.28906477 | -6.65662490 | -1.36668165 |
| 1 | -3.60513954 | -6.19114385 | -3.04729023 |
| 1 | -4.53608388 | -5.44685190 | -1.71818515 |
| 1 | -1.63572846 | -3.59026451 | -0.16428325 |
| 1 | -3.39218086 | -3.85586654 | -0.08225168 |
| 1 | -2.27200663 | -5.19680368 | 0.25165601 |
| 1 | -1.13669105 | -5.71665123 | -3.30428953 |
| 1 | -0.28156477 | -4.65204795 | -2.16204643 |
| 1 | -0.90132042 | -6.22975007 | -1.61737261 |
| 1 | 0.91718462 | 1.40937477 | -3.01031976 |
| 8 | 1.36691986 | -0.89615329 | -0.38213602 |
| 31 | -0.32872924 | -0.78722598 | -1.07271400 |
| 1 | -2.74542162 | 4.56380698 | 1.28340993 |
| 1 | -5.05401316 | -2.39231632 | -5.05154875 |
| 8 | 1.77619835 | 6.34134629 | 4.82192784 |
| 6 | 1.01850809 | 6.74630603 | 5.98750366 |
| 1 | -0.02429462 | 6.95235063 | 5.71518826 |
| 6 | 2.17086360 | 4.87473813 | 3.00133162 |
| 6 | 1.77567134 | 3.55730483 | 2.33621754 |
| 6 | 2.61373750 | 3.24523344 | 1.09209568 |
| 6 | 1.32145986 | 5.20741118 | 4.21329952 |
| 6 | 2.18486520 | 1.93861532 | 0.41025239 |
| 6 | 3.06124612 | 1.65653099 | -0.81024912 |
| 1 | 2.08132328 | 5.71704174 | 2.29491366 |
| 1 | 0.70934683 | 3.59207097 | 2.06803622 |
| 1 | 2.53167440 | 4.08172427 | 0.37536258 |
| 1 | 1.12637698 | 2.01383742 | 0.11717450 |
| 1 | 3.22643238 | 4.86847342 | 3.31729322 |
| 1 | 1.87805087 | 2.73896687 | 3.06516983 |
| 1 | 3.68024014 | 3.18338086 | 1.37142873 |
| 1 | 2.25459387 | 1.10332847 | 1.11955368 |
| 1 | 4.10771895 | 1.54391620 | -0.48344237 |
| 1 | 3.03323191 | 2.51383580 | -1.50039260 |
| 8 | 0.35978432 | 4.57497162 | 4.60915364 |
| 8 | 2.69739608 | 0.54107802 | -1.66103048 |
| 1 | 1.51059657 | 7.65327733 | 6.35151927 |
| 1 | 1.03957589 | 5.95898347 | 6.75136108 |

| | | | |
|---|---|---|---|
| 8 | 3.56295211 | -0.98466710 | -0.07042661 |
| 6 | 2.56463455 | -0.75266684 | -1.05116720 |
| 6 | 2.73027128 | -1.77761284 | -2.19762191 |
| 8 | 4.04137329 | -1.64516496 | -2.82837275 |
| 6 | 5.11297979 | -1.34365406 | -2.05683047 |
| 6 | 4.91829928 | -1.02634431 | -0.56955852 |
| 8 | 6.22167248 | -1.29785178 | -2.55003380 |
| 6 | 5.70183983 | -2.00819571 | 0.30056045 |
| 6 | 2.51844624 | -3.21982329 | -1.76337687 |
| 1 | 2.02928582 | -1.51443132 | -3.00359389 |
| 1 | 5.36785491 | -0.02400094 | -0.45346247 |
| 1 | 5.61712982 | -1.70807149 | 1.35253051 |
| 1 | 6.75725434 | -1.99999326 | 0.00432055 |
| 1 | 5.30682842 | -3.02691045 | 0.19496452 |
| 1 | 1.48374735 | -3.37302512 | -1.43940379 |
| 1 | 3.18752619 | -3.48487362 | -0.93505633 |
| 1 | 2.72164059 | -3.88473980 | -2.61243659 |

Electronic energy = -4057.833283 a.u.
DFT-D3(BJ) dispersion correction = -0.143265 a.u.
Thermal free energy = 0.723219 a.u.
Gibbs free energy = -4057.253329 a.u.
Number of imaginary frequencies = 0.

**TS4 ax LA isomer/conformer 1**

| Atomic N. | X | Y | Z |
|---|---|---|---|
| 7 | 0.94548017 | 2.82947016 | 0.38423148 |
| 8 | -0.01020453 | 0.98372418 | -1.64738703 |
| 8 | 2.94942331 | 0.78093559 | -0.46786451 |
| 6 | -0.12312236 | 3.11308551 | 1.37315512 |
| 6 | -0.87419343 | 1.97219694 | -1.95475841 |
| 6 | -2.03846478 | 1.68935978 | -2.73285365 |
| 6 | -2.90653110 | 2.75748682 | -3.00064301 |
| 6 | -2.67165518 | 4.06140232 | -2.55321622 |
| 6 | -1.51638091 | 4.33089304 | -1.82767689 |
| 6 | -0.61367774 | 3.30141687 | -1.54162848 |
| 6 | 0.69487888 | 3.60938430 | -0.88007641 |
| 1 | -3.38141704 | 4.85568094 | -2.78399158 |
| 6 | -2.32493210 | 0.27683488 | -3.27765679 |
| 6 | 3.71574974 | 1.87512898 | -0.69470811 |
| 6 | 4.82702637 | 1.78695261 | -1.58637512 |
| 6 | 5.60751486 | 2.93866158 | -1.75888801 |
| 6 | 5.33235741 | 4.14617538 | -1.11172748 |
| 6 | 4.23820639 | 4.22365284 | -0.25869340 |
| 6 | 3.43298459 | 3.09996390 | -0.03653278 |
| 6 | 2.29218936 | 3.21065640 | 0.94338018 |
| 1 | 5.96564579 | 5.01641703 | -1.28291667 |
| 6 | 5.16201210 | 0.48461893 | -2.34156609 |
| 6 | -3.61573792 | 0.23168734 | -4.11636734 |
| 6 | -2.49319744 | -0.72186971 | -2.10968566 |
| 6 | -1.16214252 | -0.17940542 | -4.19116831 |
| 6 | 6.39281797 | 0.64780134 | -3.25387645 |
| 6 | 3.97555137 | 0.06973134 | -3.24392629 |

| | | | |
|---|---|---|---|
| 6 | 5.48618746 | -0.64598161 | -1.33736479 |
| 1 | -0.08814455 | 4.17315102 | 1.67478645 |
| 1 | -1.09734213 | 2.90925026 | 0.91484702 |
| 1 | 0.75533152 | 4.68351078 | -0.63364542 |
| 1 | 1.53284323 | 3.37760568 | -1.55291426 |
| 1 | -3.80699420 | 2.57039809 | -3.58292007 |
| 1 | -1.29361081 | 5.34680462 | -1.49303174 |
| 1 | 2.45819187 | 2.56830549 | 1.81912374 |
| 1 | 2.22720003 | 4.25078297 | 1.30577469 |
| 1 | 6.46215391 | 2.90033603 | -2.43147683 |
| 1 | 4.00050545 | 5.15930176 | 0.25238702 |
| 1 | -3.76323700 | -0.79219431 | -4.48853254 |
| 1 | -4.50415850 | 0.50006300 | -3.52615929 |
| 1 | -3.56504798 | 0.89663172 | -4.99057102 |
| 1 | -1.57588255 | -0.79031467 | -1.51564193 |
| 1 | -3.32377648 | -0.41606697 | -1.45530033 |
| 1 | -2.72674918 | -1.72180974 | -2.50751019 |
| 1 | -1.05263829 | 0.50280553 | -5.04676247 |
| 1 | -0.21313135 | -0.21132408 | -3.64492702 |
| 1 | -1.37165999 | -1.18540573 | -4.58574820 |
| 1 | 6.58462763 | -0.30546981 | -3.76646090 |
| 1 | 7.29895401 | 0.90555102 | -2.68680978 |
| 1 | 6.23589897 | 1.41282547 | -4.02780676 |
| 1 | 3.06790185 | -0.12327355 | -2.66266799 |
| 1 | 3.75561333 | 0.85677826 | -3.97964358 |
| 1 | 4.23302984 | -0.84621501 | -3.79705238 |
| 1 | 6.35393047 | -0.37894887 | -0.71690714 |
| 1 | 4.63540697 | -0.84723687 | -0.67805129 |
| 1 | 5.73076963 | -1.57059371 | -1.88177645 |
| 1 | 0.00357603 | 2.47404075 | 2.25131059 |
| 8 | 0.63542604 | -1.13658738 | -0.20626225 |
| 8 | 1.07175493 | 0.04770263 | 1.81130302 |
| 31 | 1.10626781 | 0.78785825 | -0.13553718 |
| 8 | 2.59372067 | -2.27354360 | 2.63303137 |
| 6 | 2.42593050 | -1.21553659 | 3.47150588 |
| 6 | 2.03940034 | 0.16129474 | 2.86543798 |
| 6 | 0.93972212 | -1.63443279 | 0.94544250 |
| 6 | 2.36753154 | -2.09860516 | 1.21136594 |
| 8 | 2.64284730 | -1.36790502 | 4.65418100 |
| 6 | 2.65929103 | -3.43671656 | 0.54730636 |
| 6 | 1.55631709 | 1.06771600 | 3.99674749 |
| 1 | 2.99228311 | 0.55453330 | 2.45248294 |
| 1 | 3.03847575 | -1.32112694 | 0.81777602 |
| 1 | 3.70524597 | -3.71431899 | 0.72759813 |
| 1 | 2.49763489 | -3.35718322 | -0.53520066 |
| 1 | 2.00766492 | -4.21893978 | 0.95558870 |
| 1 | 2.26142716 | 1.02500248 | 4.83438540 |
| 1 | 0.57663178 | 0.72760415 | 4.35687685 |
| 1 | 1.47287619 | 2.10969210 | 3.66630626 |
| 8 | -8.23407078 | -3.93987727 | 3.51378727 |
| 6 | -9.14450073 | -4.68152046 | 4.36131907 |
| 1 | -8.99497604 | -5.76090956 | 4.23289585 |
| 6 | -6.04390526 | -3.35292006 | 2.82502747 |

| | | | |
|---|---|---|---|
| 6 | -4.54721451 | -3.55100942 | 3.06135178 |
| 6 | -3.69140124 | -2.72327995 | 2.09685421 |
| 6 | -6.91176128 | -4.17000580 | 3.76331687 |
| 6 | -2.18776274 | -2.91500974 | 2.32972693 |
| 6 | -1.36370909 | -2.09622288 | 1.35141695 |
| 1 | -6.32194710 | -3.62130356 | 1.79262090 |
| 1 | -4.30111027 | -4.61876440 | 2.95796919 |
| 1 | -3.94043231 | -2.99667239 | 1.05702353 |
| 1 | -1.92480695 | -3.97893882 | 2.22032118 |
| 1 | -6.32897663 | -2.29435205 | 2.93909574 |
| 1 | -4.30394793 | -3.28487110 | 4.10129642 |
| 1 | -3.94715095 | -1.65474427 | 2.20312142 |
| 1 | -1.92557383 | -2.62498212 | 3.35911083 |
| 1 | -1.55039942 | -1.01789904 | 1.46584702 |
| 1 | -1.56591773 | -2.37652874 | 0.30892354 |
| 8 | -6.51337051 | -4.93096876 | 4.62426329 |
| 8 | 0.04476769 | -2.35221553 | 1.63773739 |
| 1 | -10.14693546 | -4.38723946 | 4.03614140 |
| 1 | -8.98527527 | -4.42162371 | 5.41536808 |

Electronic energy = -4057.832284 a.u.
DFT-D3(BJ) dispersion correction = -0.139350 a.u.
Thermal free energy = 0.721262 a.u.
Gibbs free energy = -4057.250372 a.u.
Number of imaginary frequencies = 1.

**TS4 ax LA isomer/conformer 2**

| Atomic N. | X | Y | Z |
|---|---|---|---|
| 7 | 1.49640775 | 2.77003217 | -1.07575953 |
| 8 | 1.80342722 | 1.70464575 | 1.58994091 |
| 8 | -0.70525217 | 0.98785007 | -0.38187146 |
| 6 | 2.90633535 | 3.05982304 | -1.42959130 |
| 6 | 2.13715601 | 2.97494578 | 1.91134810 |
| 6 | 2.83514881 | 3.21732807 | 3.13545990 |
| 6 | 3.16665816 | 4.54447126 | 3.43943930 |
| 6 | 2.85303783 | 5.61711550 | 2.60035896 |
| 6 | 2.17487001 | 5.36919117 | 1.41322470 |
| 6 | 1.80333090 | 4.06434917 | 1.06905043 |
| 6 | 0.97686046 | 3.85397601 | -0.16899997 |
| 1 | 3.13933682 | 6.63197994 | 2.87607431 |
| 6 | 3.22330618 | 2.06953835 | 4.08820152 |
| 6 | -1.43459535 | 1.86489296 | -1.10331655 |
| 6 | -2.84320021 | 1.96026719 | -0.87212068 |
| 6 | -3.57445097 | 2.84178233 | -1.67963266 |
| 6 | -2.98231721 | 3.64060092 | -2.66139174 |
| 6 | -1.60560369 | 3.58024669 | -2.83966136 |
| 6 | -0.82848901 | 2.70016265 | -2.07746625 |
| 6 | 0.65851945 | 2.66411662 | -2.32277179 |
| 1 | -3.59177828 | 4.31693316 | -3.26046753 |
| 6 | -3.54057145 | 1.15087891 | 0.24028490 |
| 6 | 3.91078091 | 2.58536410 | 5.36676311 |
| 6 | 4.22086477 | 1.12699068 | 3.37661934 |
| 6 | 1.96693718 | 1.28289449 | 4.53273678 |

| | | | |
|---|---|---|---|
| 6 | -5.05095053 | 1.44584441 | 0.31036311 |
| 6 | -2.93649220 | 1.51840866 | 1.61686051 |
| 6 | -3.37981892 | -0.36492744 | -0.01329560 |
| 1 | 2.97297096 | 4.04717970 | -1.91533768 |
| 1 | 3.52168012 | 3.06786919 | -0.52433312 |
| 1 | 0.92739600 | 4.79195023 | -0.74738663 |
| 1 | -0.05588972 | 3.58183074 | 0.09051564 |
| 1 | 3.69686937 | 4.75547028 | 4.36615419 |
| 1 | 1.91227555 | 6.19074106 | 0.74273491 |
| 1 | 0.96256667 | 1.72612059 | -2.81336951 |
| 1 | 0.93040031 | 3.49696875 | -2.99300551 |
| 1 | -4.64962006 | 2.92309403 | -1.53212011 |
| 1 | -1.11550629 | 4.21831179 | -3.57865405 |
| 1 | 4.15735626 | 1.72728455 | 6.00843573 |
| 1 | 4.85011959 | 3.11411238 | 5.14992142 |
| 1 | 3.25865054 | 3.25540614 | 5.94572878 |
| 1 | 3.79875016 | 0.71918517 | 2.45267916 |
| 1 | 5.14743042 | 1.66287875 | 3.12426662 |
| 1 | 4.48629999 | 0.29012892 | 4.04153109 |
| 1 | 1.26466990 | 1.94430673 | 5.06119251 |
| 1 | 1.44774997 | 0.83396083 | 3.67968583 |
| 1 | 2.26047826 | 0.48115695 | 5.22796965 |
| 1 | -5.49226475 | 0.84982449 | 1.12186944 |
| 1 | -5.57064390 | 1.17355621 | -0.61979079 |
| 1 | -5.25760174 | 2.50354028 | 0.52793008 |
| 1 | -1.86772263 | 1.28350461 | 1.66521525 |
| 1 | -3.07030153 | 2.59013748 | 1.82398772 |
| 1 | -3.44936204 | 0.95494741 | 2.41110921 |
| 1 | -3.82503223 | -0.64612943 | -0.97934669 |
| 1 | -2.32298756 | -0.64769739 | -0.01440840 |
| 1 | -3.89553285 | -0.93225318 | 0.77684915 |
| 1 | 3.29232335 | 2.29921627 | -2.11174846 |
| 8 | 1.05051410 | -0.80683893 | 0.87993193 |
| 8 | 1.85595751 | -0.39287642 | -1.34882271 |
| 31 | 1.13651848 | 0.99005836 | -0.01012618 |
| 8 | 4.08893061 | -0.83778989 | 0.16274677 |
| 6 | 4.21715593 | -0.33303770 | -1.11009347 |
| 6 | 3.06423044 | -0.70740473 | -2.04304600 |
| 6 | 1.69610012 | -1.62218761 | 0.13624886 |
| 6 | 3.18840885 | -1.96014023 | 0.36952436 |
| 8 | 5.13414478 | 0.41386068 | -1.37128782 |
| 6 | 3.38434792 | -2.42537069 | 1.80596244 |
| 6 | 3.17636585 | -0.12914573 | -3.44607902 |
| 1 | 3.06791902 | -1.80925310 | -2.16032982 |
| 1 | 3.46426058 | -2.76497436 | -0.32681125 |
| 1 | 4.44189072 | -2.66340923 | 1.97334862 |
| 1 | 2.78593469 | -3.32712507 | 1.99080038 |
| 1 | 3.07245231 | -1.64029789 | 2.50416064 |
| 1 | 4.06731892 | -0.53992593 | -3.93990612 |
| 1 | 3.27172852 | 0.96128887 | -3.45482159 |
| 1 | 2.29045677 | -0.41729653 | -4.02662897 |
| 8 | -6.15050220 | -6.83092260 | -2.61167860 |
| 6 | -6.70876789 | -8.04235840 | -3.17535019 |

| | | | |
|---|---|---|---|
| 1 | -6.34912157 | -8.18932247 | -4.20151854 |
| 6 | -4.30336475 | -5.50681162 | -1.94065583 |
| 6 | -2.78302526 | -5.43222952 | -1.80579329 |
| 6 | -2.31617475 | -4.10143852 | -1.20722091 |
| 6 | -4.78762245 | -6.81375360 | -2.53908920 |
| 6 | -0.79092807 | -4.02307463 | -1.06707954 |
| 6 | -0.34929240 | -2.69805956 | -0.47065565 |
| 1 | -4.68675518 | -4.68732262 | -2.57043409 |
| 1 | -2.32321072 | -5.58072233 | -2.79481578 |
| 1 | -2.67048383 | -3.26862097 | -1.83788228 |
| 1 | -0.31870276 | -4.15317726 | -2.05398798 |
| 1 | -4.79698896 | -5.37989759 | -0.96349269 |
| 1 | -2.43206120 | -6.26806402 | -1.18120468 |
| 1 | -2.78409457 | -3.95731401 | -0.21842919 |
| 1 | -0.43235871 | -4.84611988 | -0.42822465 |
| 1 | -0.75901395 | -2.53936434 | 0.53607094 |
| 1 | -0.63398594 | -1.84364927 | -1.10101759 |
| 8 | -4.08379269 | -7.73339033 | -2.91136265 |
| 8 | 1.11026108 | -2.72128415 | -0.37839064 |
| 1 | -7.79284859 | -7.89481354 | -3.16156602 |
| 1 | -6.42678070 | -8.91272831 | -2.56964707 |

Electronic energy = -4057.826850 a.u.
DFT-D3(BJ) dispersion correction = -0.140866 a.u.
Thermal free energy = 0.721873 a.u.
Gibbs free energy = -4057.245843 a.u.
Number of imaginary frequencies = 1.

## Propagation step of equatorial LA addition to the product of CL addition on initiation step

**TS3 eq LA isomer/conformer 1**

| Atomic N. | X | Y | Z |
|---|---|---|---|
| 7 | 3.02664351 | 2.22881198 | -0.01470287 |
| 8 | 0.63170606 | 1.43666458 | 1.46747124 |
| 8 | 0.96267438 | 1.05437446 | -1.74486136 |
| 6 | 4.19161510 | 2.02296305 | 0.88110405 |
| 6 | 0.98806190 | 2.45861316 | 2.27591801 |
| 6 | 0.49842060 | 2.50925064 | 3.61782384 |
| 6 | 0.89508718 | 3.59536386 | 4.41043520 |
| 6 | 1.73741651 | 4.60864735 | 3.94447780 |
| 6 | 2.20657277 | 4.54939651 | 2.63702321 |
| 6 | 1.83264947 | 3.49259686 | 1.80036843 |
| 6 | 2.28888440 | 3.48295903 | 0.36998338 |
| 6 | -0.43304360 | 1.41853023 | 4.18019724 |
| 6 | 1.25092828 | 2.09481406 | -2.55332685 |
| 6 | 0.31081077 | 2.49780464 | -3.55086613 |
| 6 | 0.66591489 | 3.57488847 | -4.37422752 |
| 6 | 1.88026428 | 4.25659275 | -4.25091171 |
| 6 | 2.78591132 | 3.85604548 | -3.27530718 |
| 6 | 2.48783708 | 2.77694130 | -2.43519378 |
| 6 | 3.51821303 | 2.32091236 | -1.43756270 |

| | | | |
|---|---|---|---|
| 6 | -1.04143250 | 1.77821815 | -3.72248697 |
| 6 | -0.83370209 | 1.69116807 | 5.64237452 |
| 6 | 0.28301612 | 0.04705421 | 4.15229321 |
| 6 | -1.73898900 | 1.36603475 | 3.35271096 |
| 6 | -1.87911236 | 2.38574767 | -4.86349297 |
| 6 | -1.87797236 | 1.89851391 | -2.42649484 |
| 6 | -0.80707073 | 0.28851217 | -4.06791639 |
| 1 | 4.84022093 | 2.91407776 | 0.85434413 |
| 1 | 3.84897900 | 1.86410868 | 1.90876210 |
| 1 | 2.94948602 | 4.34548521 | 0.17915145 |
| 1 | 1.43547559 | 3.56658912 | -0.31867912 |
| 1 | 0.53741276 | 3.65669131 | 5.43667507 |
| 1 | 2.86074734 | 5.33224106 | 2.24625587 |
| 1 | 3.90017629 | 1.31789970 | -1.68393075 |
| 1 | 4.37190247 | 3.01950622 | -1.45561147 |
| 1 | -0.03207090 | 3.90417123 | -5.14180946 |
| 1 | 3.74139524 | 4.37329054 | -3.16072893 |
| 1 | -1.49929094 | 0.88623017 | 5.98591900 |
| 1 | 0.03654991 | 1.71008158 | 6.31435442 |
| 1 | -1.37908733 | 2.63950658 | 5.75198698 |
| 1 | 0.59476095 | -0.22470696 | 3.13858461 |
| 1 | 1.17427778 | 0.06885349 | 4.79695988 |
| 1 | -0.39182168 | -0.73507488 | 4.53330946 |
| 1 | -2.28597260 | 2.31598306 | 3.44105673 |
| 1 | -1.53701937 | 1.18471122 | 2.29156971 |
| 1 | -2.39152408 | 0.56354886 | 3.72955465 |
| 1 | -2.82625055 | 1.83350337 | -4.94352341 |
| 1 | -1.37042069 | 2.31112838 | -5.83548355 |
| 1 | -2.12649441 | 3.44132972 | -4.67966747 |
| 1 | -1.36326182 | 1.44961429 | -1.57029676 |
| 1 | -2.08247900 | 2.95397496 | -2.19464636 |
| 1 | -2.84424353 | 1.38812625 | -2.55817223 |
| 1 | -0.25816870 | 0.19427671 | -5.01638079 |
| 1 | -0.23604742 | -0.21732678 | -3.28204536 |
| 1 | -1.77327716 | -0.22552077 | -4.18483496 |
| 1 | 4.75153971 | 1.14553046 | 0.54626393 |
| 8 | 3.29023886 | -0.61403888 | -0.05985315 |
| 31 | 1.56257427 | 0.70137954 | -0.00137880 |
| 1 | 2.01983023 | 5.43207979 | 4.60035372 |
| 1 | 2.10834575 | 5.09354782 | -4.91074133 |
| 8 | -6.87083769 | -4.82384825 | -0.85770810 |
| 6 | -7.81643391 | -5.41370964 | -1.78071284 |
| 1 | -8.17757034 | -4.66228104 | -2.49436927 |
| 6 | -4.84061861 | -3.68597269 | -0.40426841 |
| 6 | -3.49980140 | -3.21720028 | -0.96850514 |
| 6 | -2.61233735 | -2.56716847 | 0.09821136 |
| 6 | -5.75557232 | -4.29664660 | -1.44736719 |
| 6 | -1.25570941 | -2.11457491 | -0.45353439 |
| 6 | -0.38700035 | -1.45969355 | 0.61986721 |
| 1 | -5.38604879 | -2.84655905 | 0.05944401 |
| 1 | -3.67938638 | -2.50785780 | -1.79052877 |
| 1 | -3.14097238 | -1.70205271 | 0.53590876 |
| 1 | -1.40463090 | -1.39794123 | -1.27675295 |

| | | | |
|---|---|---|---|
| 1 | -4.70048094 | -4.42400074 | 0.40140405 |
| 1 | -2.97389388 | -4.07406235 | -1.41719031 |
| 1 | -2.45021296 | -3.28083253 | 0.92475492 |
| 1 | -0.72104782 | -2.98103213 | -0.87356317 |
| 1 | -0.24989685 | -2.15968060 | 1.46722794 |
| 1 | -0.90146482 | -0.57696694 | 1.03525674 |
| 8 | -5.55934858 | -4.32329941 | -2.64745307 |
| 8 | 0.89720744 | -1.07707071 | 0.12651722 |
| 1 | -8.63727188 | -5.78287220 | -1.15817487 |
| 1 | -7.34915304 | -6.23681164 | -2.33599734 |
| 8 | 2.99516773 | -2.15878773 | 1.52388775 |
| 6 | 2.91924548 | -1.77370954 | 0.23692410 |
| 6 | 2.84741759 | -2.90159130 | -0.78415072 |
| 8 | 1.86173832 | -3.89925838 | -0.39169210 |
| 6 | 1.83612597 | -4.27433729 | 0.91392136 |
| 6 | 2.87138557 | -3.59000945 | 1.81393838 |
| 8 | 1.06275845 | -5.12083626 | 1.30039144 |
| 6 | 2.55728316 | -3.72968674 | 3.28907537 |
| 6 | 2.53468919 | -2.45187187 | -2.19574094 |
| 1 | 3.84321666 | -3.38979244 | -0.75902021 |
| 1 | 3.85109711 | -4.04883432 | 1.58199298 |
| 1 | 3.34100556 | -3.24659872 | 3.88505292 |
| 1 | 2.50431132 | -4.79197502 | 3.55297613 |
| 1 | 1.59052539 | -3.26599264 | 3.51893997 |
| 1 | 3.33048916 | -1.78485370 | -2.54785943 |
| 1 | 1.58058023 | -1.91482341 | -2.21814799 |
| 1 | 2.48765898 | -3.32712340 | -2.85510302 |

Electronic energy = -4057.835882 a.u.
DFT-D3(BJ) dispersion correction = -0.140478 a.u.
Thermal free energy = 0.717466 a.u.
Gibbs free energy = -4057.258894 a.u.
Number of imaginary frequencies = 1.

**TS3_eq_LA isomer/conformer 2**

| Atomic N. | X | Y | Z |
|---|---|---|---|
| 7 | 2.27911615 | 2.65618157 | 0.19134621 |
| 8 | -0.04615581 | 1.63374352 | 1.61700952 |
| 8 | 0.38469619 | 1.35467815 | -1.60964966 |
| 6 | 3.37691593 | 2.57529497 | 1.18607318 |
| 6 | 0.11670110 | 2.74859738 | 2.36553907 |
| 6 | -0.45919055 | 2.81243563 | 3.67304635 |
| 6 | -0.27223670 | 3.99137807 | 4.40771246 |
| 6 | 0.44978940 | 5.08368731 | 3.92021537 |
| 6 | 1.01072025 | 5.00851154 | 2.65084100 |
| 6 | 0.84328288 | 3.86009192 | 1.86923313 |
| 6 | 1.40010118 | 3.84254479 | 0.47373581 |
| 6 | -1.25417578 | 1.63386893 | 4.26689625 |
| 6 | 0.70417798 | 2.36002755 | -2.45042443 |
| 6 | -0.17600285 | 2.67982388 | -3.53109193 |
| 6 | 0.20913774 | 3.71276832 | -4.39611673 |
| 6 | 1.39223289 | 4.43859291 | -4.23200417 |
| 6 | 2.23018503 | 4.13223934 | -3.16653943 |

| | | | |
|---|---|---|---|
| 6 | 1.90454376 | 3.09617066 | -2.28317380 |
| 6 | 2.87837720 | 2.75497389 | -1.18623865 |
| 6 | -1.50903010 | 1.93325222 | -3.73577905 |
| 6 | -1.79598379 | 1.94473207 | 5.67523956 |
| 6 | -0.33106261 | 0.39894348 | 4.40030718 |
| 6 | -2.47625613 | 1.31472278 | 3.37359214 |
| 6 | -2.26971626 | 2.43552041 | -4.97767067 |
| 6 | -2.42706728 | 2.16381001 | -2.51200151 |
| 6 | -1.25647318 | 0.42053562 | -3.93617535 |
| 1 | 3.94690967 | 3.51897931 | 1.18745673 |
| 1 | 2.96311402 | 2.40914416 | 2.18603158 |
| 1 | 1.98537445 | 4.76012850 | 0.29417530 |
| 1 | 0.59449053 | 3.82064342 | -0.27483994 |
| 1 | -0.70279777 | 4.06393766 | 5.40468454 |
| 1 | 1.57821751 | 5.84910631 | 2.24496126 |
| 1 | 3.36653066 | 1.78472733 | -1.36694014 |
| 1 | 3.66811156 | 3.52506113 | -1.15795207 |
| 1 | -0.44093037 | 3.97228265 | -5.22957134 |
| 1 | 3.15587449 | 4.69163465 | -3.01223207 |
| 1 | -2.35553622 | 1.07217205 | 6.04196978 |
| 1 | -0.99037802 | 2.14671850 | 6.39586163 |
| 1 | -2.48502111 | 2.80150104 | 5.67322206 |
| 1 | 0.11457971 | 0.11894806 | 3.43972754 |
| 1 | 0.48293957 | 0.60759270 | 5.11019325 |
| 1 | -0.90318614 | -0.45958090 | 4.78437853 |
| 1 | -3.16832829 | 2.16924143 | 3.35562181 |
| 1 | -2.17990732 | 1.09437799 | 2.34281278 |
| 1 | -3.02126527 | 0.44782731 | 3.77738523 |
| 1 | -3.20725179 | 1.86951458 | -5.07403660 |
| 1 | -1.69567358 | 2.28474402 | -5.90345526 |
| 1 | -2.53409600 | 3.50000000 | -4.90091467 |
| 1 | -1.95566165 | 1.82158899 | -1.58425736 |
| 1 | -2.66645193 | 3.23194551 | -2.40582061 |
| 1 | -3.37298679 | 1.61660016 | -2.64313602 |
| 1 | -0.63096499 | 0.24778704 | -4.82444763 |
| 1 | -0.75943398 | -0.01982737 | -3.06604147 |
| 1 | -2.21402097 | -0.09941191 | -4.09211683 |
| 1 | 4.03668833 | 1.74336255 | 0.92417026 |
| 8 | 2.78570747 | -0.12849680 | 0.16369106 |
| 31 | 0.95589793 | 1.00782418 | 0.14710392 |
| 1 | 0.57097048 | 5.97854757 | 4.53050947 |
| 1 | 1.64559901 | 5.23960781 | -4.92628002 |
| 8 | -1.68109906 | -7.29729748 | -2.07643819 |
| 6 | -2.02250028 | -8.66293144 | -1.74296165 |
| 1 | -1.73137534 | -8.88925171 | -0.70951092 |
| 6 | -1.91426623 | -4.95811939 | -1.74481285 |
| 6 | -2.11804962 | -3.89878821 | -0.66060632 |
| 6 | -1.87121785 | -2.47821903 | -1.18184543 |
| 6 | -2.29138112 | -6.35517550 | -1.29490650 |
| 6 | -1.95513487 | -1.40235507 | -0.08974528 |
| 6 | -0.74479830 | -1.33156800 | 0.84672594 |
| 1 | -0.87568992 | -4.96978092 | -2.10505104 |
| 1 | -1.44500303 | -4.12626791 | 0.17997435 |

| | | | |
|---|---|---|---|
| 1 | -0.88474798 | -2.42245984 | -1.66927266 |
| 1 | -2.85115790 | -1.57840514 | 0.53148031 |
| 1 | -2.53996110 | -4.72932673 | -2.62606525 |
| 1 | -3.14197326 | -3.97908306 | -0.26507083 |
| 1 | -2.61117530 | -2.25382280 | -1.96821702 |
| 1 | -2.08491063 | -0.40938681 | -0.54745239 |
| 1 | -0.51121664 | -2.33755779 | 1.23912311 |
| 1 | -0.99067444 | -0.70146030 | 1.71392691 |
| 8 | -3.04927230 | -6.63489676 | -0.38540477 |
| 8 | 0.43369198 | -0.83306682 | 0.20190550 |
| 1 | -1.46113968 | -9.28306961 | -2.44880676 |
| 1 | -3.10189605 | -8.82875443 | -1.85337579 |
| 8 | 2.57926273 | -1.83525276 | 1.59128463 |
| 6 | 2.50328231 | -1.33865094 | 0.34284097 |
| 6 | 2.59073806 | -2.36648774 | -0.77754331 |
| 8 | 1.71911061 | -3.50292277 | -0.51517862 |
| 6 | 1.73060536 | -4.01742363 | 0.74228269 |
| 6 | 2.63683057 | -3.29230070 | 1.74302566 |
| 8 | 1.08103955 | -5.00153589 | 1.01449490 |
| 6 | 2.29896569 | -3.61363626 | 3.18418860 |
| 6 | 2.27168393 | -1.82949495 | -2.15654325 |
| 1 | 3.63626122 | -2.73691964 | -0.75510812 |
| 1 | 3.67626429 | -3.59600401 | 1.51646698 |
| 1 | 2.99098396 | -3.09017968 | 3.85484409 |
| 1 | 2.38330293 | -4.69397545 | 3.34735370 |
| 1 | 1.27117026 | -3.30798435 | 3.41385937 |
| 1 | 2.99299479 | -1.04810381 | -2.42340970 |
| 1 | 1.26401353 | -1.40108621 | -2.17284536 |
| 1 | 2.34469843 | -2.64235306 | -2.88944364 |

Electronic energy = -4057.833212 a.u.
DFT-D3(BJ) dispersion correction = -0.143477 a.u.
Thermal free energy = 0.720854 a.u.
Gibbs free energy = -4057.255835 a.u.
Number of imaginary frequencies = 1.

**TS3 eq LA isomer/conformer 3**

| Atomic N. | X | Y | Z |
|---|---|---|---|
| 7 | 2.15426302 | 2.37092614 | -1.14041007 |
| 8 | 0.08627423 | 2.17683673 | 0.92774034 |
| 8 | 0.08442253 | 0.39797735 | -1.77410924 |
| 6 | 3.37606287 | 2.74571872 | -0.38921753 |
| 6 | 0.22879909 | 3.52398443 | 0.97720206 |
| 6 | -0.26545456 | 4.23857498 | 2.11465669 |
| 6 | -0.08857254 | 5.62863207 | 2.14041352 |
| 6 | 0.54329503 | 6.33189726 | 1.11286318 |
| 6 | 1.00802493 | 5.63144779 | 0.00686054 |
| 6 | 0.84637851 | 4.24371719 | -0.07930930 |
| 6 | 1.26814663 | 3.55937934 | -1.35200238 |
| 6 | -0.97390020 | 3.52273226 | 3.28178525 |
| 6 | 0.25734216 | 0.83061087 | -3.03953815 |
| 6 | -0.75982183 | 0.57297373 | -4.01137590 |
| 6 | -0.54245055 | 1.03155839 | -5.31716394 |

| | | | |
|---|---|---|---|
| 6 | 0.60930192 | 1.72777390 | -5.69408178 |
| 6 | 1.58916056 | 1.97713518 | -4.74077654 |
| 6 | 1.43173909 | 1.52704227 | -3.42443419 |
| 6 | 2.55897331 | 1.75158131 | -2.45125842 |
| 6 | -2.05243254 | -0.18104833 | -3.64297962 |
| 6 | -1.45865548 | 4.50491762 | 4.36534405 |
| 6 | 0.00936797 | 2.54539919 | 3.96551847 |
| 6 | -2.22190571 | 2.76933002 | 2.76412797 |
| 6 | -2.98252821 | -0.36699042 | -4.85677385 |
| 6 | -2.84258842 | 0.61967349 | -2.58114815 |
| 6 | -1.71089554 | -1.59199393 | -3.10801339 |
| 1 | 3.92473793 | 3.53140235 | -0.93478096 |
| 1 | 3.10247588 | 3.12588954 | 0.60082871 |
| 1 | 1.79137743 | 4.28081703 | -2.00273299 |
| 1 | 0.39209503 | 3.19865537 | -1.91153371 |
| 1 | -0.45483220 | 6.19028378 | 2.99752188 |
| 1 | 1.49347091 | 6.15885592 | -0.81738681 |
| 1 | 3.05616188 | 0.80414993 | -2.18768263 |
| 1 | 3.31299710 | 2.40572262 | -2.92145634 |
| 1 | -1.30165219 | 0.84593260 | -6.07472944 |
| 1 | 2.49832034 | 2.51917291 | -5.01123190 |
| 1 | -1.96191347 | 3.93688941 | 5.16107941 |
| 1 | -0.62833339 | 5.05657673 | 4.82900286 |
| 1 | -2.18239594 | 5.23173714 | 3.96970868 |
| 1 | 0.40597901 | 1.81990349 | 3.24716949 |
| 1 | 0.85485393 | 3.09502745 | 4.40446758 |
| 1 | -0.49766785 | 1.99888039 | 4.77555561 |
| 1 | -2.95103455 | 3.47979283 | 2.34839702 |
| 1 | -1.96303904 | 2.04826045 | 1.98200679 |
| 1 | -2.70853257 | 2.23374271 | 3.59386468 |
| 1 | -3.88185334 | -0.91261429 | -4.53652811 |
| 1 | -2.50687766 | -0.95329100 | -5.65622139 |
| 1 | -3.31255841 | 0.59338254 | -5.27840519 |
| 1 | -2.24482727 | 0.78821266 | -1.67890775 |
| 1 | -3.14523411 | 1.59766090 | -2.98302007 |
| 1 | -3.75578070 | 0.07253011 | -2.30029273 |
| 1 | -1.19600594 | -2.18068266 | -3.88157248 |
| 1 | -1.06721818 | -1.54099619 | -2.22368121 |
| 1 | -2.63787746 | -2.12333679 | -2.84174275 |
| 1 | 4.01046896 | 1.86119926 | -0.27597588 |
| 8 | 2.61931944 | -0.03091332 | 0.29729337 |
| 31 | 0.94612670 | 0.93290848 | -0.19521639 |
| 1 | 0.66455865 | 7.41294527 | 1.17932522 |
| 1 | 0.73164326 | 2.07221437 | -6.72082806 |
| 8 | -1.78710663 | -6.75073242 | 0.25864968 |
| 6 | -0.63845736 | -7.55393887 | -0.10954672 |
| 1 | 0.01881504 | -6.99200344 | -0.78494561 |
| 6 | -2.77494431 | -4.90943003 | 1.37534189 |
| 6 | -2.54048395 | -3.47457314 | 1.85314023 |
| 6 | -2.22012091 | -2.48840976 | 0.72174805 |
| 6 | -1.50334036 | -5.63681746 | 0.98450458 |
| 6 | -1.97292876 | -1.06097353 | 1.22940600 |
| 6 | -0.58380800 | -0.82773000 | 1.83200729 |

| | | | |
|---|---|---|---|
| 1 | -3.47628951 | -4.94746923 | 0.52823007 |
| 1 | -1.72958827 | -3.48225117 | 2.59774065 |
| 1 | -1.34057558 | -2.82879066 | 0.15264453 |
| 1 | -2.72309947 | -0.80671066 | 1.99863362 |
| 1 | -3.23807001 | -5.51213503 | 2.17485976 |
| 1 | -3.44657016 | -3.13153148 | 2.37784910 |
| 1 | -3.06231070 | -2.48094893 | 0.01085863 |
| 1 | -2.11047602 | -0.33575487 | 0.41389295 |
| 1 | -0.31384304 | -1.67677760 | 2.48510599 |
| 1 | -0.59589660 | 0.08025601 | 2.45472717 |
| 8 | -0.36971006 | -5.31007957 | 1.29570508 |
| 8 | 0.41815624 | -0.70977443 | 0.81302476 |
| 1 | -1.05109715 | -8.43078613 | -0.61699837 |
| 1 | -0.07294666 | -7.84874916 | 0.78286803 |
| 8 | 2.31278825 | -2.19947505 | -0.08362934 |
| 6 | 2.24491000 | -1.16710019 | 0.78647786 |
| 6 | 2.53126502 | -1.55673862 | 2.24377251 |
| 8 | 3.62389708 | -2.53117371 | 2.27085662 |
| 6 | 3.46508336 | -3.64008880 | 1.48566842 |
| 6 | 2.30319524 | -3.54213715 | 0.49453384 |
| 8 | 4.21979284 | -4.57944155 | 1.58605063 |
| 6 | 2.42229700 | -4.53042316 | -0.64603269 |
| 6 | 2.94693637 | -0.39016747 | 3.11266255 |
| 1 | 1.64305866 | -2.05472827 | 2.66131139 |
| 1 | 1.35214257 | -3.70086956 | 1.03173339 |
| 1 | 1.56943429 | -4.41385841 | -1.32560146 |
| 1 | 2.42545843 | -5.54786110 | -0.23960538 |
| 1 | 3.35577679 | -4.37295675 | -1.20013487 |
| 1 | 2.14282727 | 0.35494459 | 3.15020776 |
| 1 | 3.84449053 | 0.08916301 | 2.70496035 |
| 1 | 3.15204024 | -0.74167544 | 4.13110638 |

Electronic energy = -4057.835412 a.u.
DFT-D3(BJ) dispersion correction = -0.143417 a.u.
Thermal free energy = 0.723326 a.u.
Gibbs free energy = -4057.255502 a.u.
Number of imaginary frequencies = 1.

**TS3 eq LA isomer/conformer 4**

| Atomic N. | X | Y | Z |
|---|---|---|---|
| 7 | 0.82229400 | 3.14778638 | 1.92099082 |
| 8 | -1.25080419 | 1.60187709 | 0.55332524 |
| 8 | 1.85548222 | 1.47739339 | -0.28405878 |
| 6 | 0.44562787 | 3.24014568 | 3.35261345 |
| 6 | -2.03661036 | 2.64217424 | 0.90301389 |
| 6 | -3.45407581 | 2.46978211 | 0.93925005 |
| 6 | -4.23009443 | 3.57922816 | 1.30215347 |
| 6 | -3.67417979 | 4.82023525 | 1.62688339 |
| 6 | -2.29286432 | 4.97534990 | 1.58732533 |
| 6 | -1.47247398 | 3.90350580 | 1.21905684 |
| 6 | 0.01105624 | 4.11153650 | 1.09935665 |
| 6 | -4.10919952 | 1.11702919 | 0.59822553 |
| 6 | 2.56975245 | 2.57918286 | -0.59201121 |

| | | | |
|---|---|---|---|
| 6 | 3.12573504 | 2.71581697 | -1.90160334 |
| 6 | 3.86596751 | 3.87413955 | -2.17348409 |
| 6 | 4.06949997 | 4.88327456 | -1.22794580 |
| 6 | 3.52503133 | 4.73905563 | 0.04283174 |
| 6 | 2.79034853 | 3.59429479 | 0.37364167 |
| 6 | 2.29554629 | 3.43581390 | 1.78699696 |
| 6 | 2.92062998 | 1.63417888 | -2.98027110 |
| 6 | -5.64534140 | 1.17827618 | 0.69337618 |
| 6 | -3.62955189 | 0.03204230 | 1.59180248 |
| 6 | -3.75565457 | 0.70323104 | -0.85029095 |
| 6 | 3.63226962 | 1.98801792 | -4.29977417 |
| 6 | 1.41420627 | 1.48575330 | -3.29875946 |
| 6 | 3.50188971 | 0.28424430 | -2.49615860 |
| 1 | 0.58519244 | 4.27445221 | 3.70805550 |
| 1 | -0.60568988 | 2.96090317 | 3.47965074 |
| 1 | 0.27354580 | 5.13788223 | 1.40739322 |
| 1 | 0.34456247 | 3.98738623 | 0.05841427 |
| 1 | -5.31310463 | 3.47606134 | 1.33953834 |
| 1 | -1.83575130 | 5.93768215 | 1.82936203 |
| 1 | 2.80454540 | 2.60264635 | 2.29684782 |
| 1 | 2.51357269 | 4.35925388 | 2.35003066 |
| 1 | 4.29888725 | 4.00321865 | -3.16381955 |
| 1 | 3.67422938 | 5.51280642 | 0.79952306 |
| 1 | -6.05866861 | 0.19279321 | 0.43534794 |
| 1 | -5.98846674 | 1.42474377 | 1.70862448 |
| 1 | -6.07412720 | 1.91025424 | -0.00620949 |
| 1 | -2.54329681 | -0.10362317 | 1.54391956 |
| 1 | -3.90649843 | 0.30316207 | 2.62151146 |
| 1 | -4.11129856 | -0.92932338 | 1.35582840 |
| 1 | -4.13814163 | 1.44426155 | -1.56726825 |
| 1 | -2.67274189 | 0.61404926 | -0.98856115 |
| 1 | -4.22187853 | -0.26596367 | -1.08541763 |
| 1 | 3.45868826 | 1.18134856 | -5.02636242 |
| 1 | 4.71956921 | 2.08817172 | -4.16978168 |
| 1 | 3.24714828 | 2.91816235 | -4.74184895 |
| 1 | 0.83815527 | 1.21723735 | -2.40696502 |
| 1 | 1.01110697 | 2.42699170 | -3.69985199 |
| 1 | 1.26652229 | 0.70367593 | -4.05913115 |
| 1 | 4.58625841 | 0.36985216 | -2.33227324 |
| 1 | 3.03228474 | -0.03882361 | -1.56082189 |
| 1 | 3.33608770 | -0.48996073 | -3.26093888 |
| 1 | 1.07701492 | 2.56164694 | 3.93388748 |
| 8 | 1.33511567 | 0.47875097 | 2.80864334 |
| 31 | 0.53344136 | 1.25726688 | 1.03186011 |
| 1 | -4.31910658 | 5.65313339 | 1.90679979 |
| 1 | 4.64575338 | 5.77093029 | -1.48836339 |
| 8 | -2.86758137 | -7.09039164 | -3.93256235 |
| 6 | -3.82506800 | -8.17291641 | -4.01175499 |
| 1 | -3.69688249 | -8.86149216 | -3.16708565 |
| 6 | -1.94904864 | -5.20329571 | -2.82877636 |
| 6 | -1.83672774 | -4.45550108 | -1.50041509 |
| 6 | -0.85364825 | -3.28219843 | -1.57099581 |
| 6 | -2.96764731 | -6.32617092 | -2.80381918 |

| | | | |
|---|---|---|---|
| 6 | -0.71970254 | -2.54172134 | -0.23523769 |
| 6 | 0.24092115 | -1.35770822 | -0.32625815 |
| 1 | -0.97952920 | -5.62362385 | -3.13826370 |
| 1 | -1.52107835 | -5.15696716 | -0.71261442 |
| 1 | 0.13744074 | -3.65189385 | -1.88718414 |
| 1 | -0.37427187 | -3.24362206 | 0.54004014 |
| 1 | -2.24403214 | -4.51529503 | -3.63964891 |
| 1 | -2.83212233 | -4.09305620 | -1.20293462 |
| 1 | -1.18339229 | -2.57559752 | -2.35282159 |
| 1 | -1.70500875 | -2.17007351 | 0.08734095 |
| 1 | -0.12558138 | -0.64888096 | -1.08981657 |
| 1 | 1.23687708 | -1.69374013 | -0.67346221 |
| 8 | -3.78113008 | -6.53664017 | -1.92441058 |
| 8 | 0.36539876 | -0.66658401 | 0.92173600 |
| 1 | -3.61366129 | -8.67784405 | -4.95931864 |
| 1 | -4.85018301 | -7.78133631 | -4.00017452 |
| 8 | 2.84113288 | -0.96144032 | 2.03116846 |
| 6 | 1.62396610 | -0.72684538 | 2.54207921 |
| 6 | 0.94081807 | -1.82985687 | 3.32611513 |
| 8 | 1.14555752 | -3.15881896 | 2.77012706 |
| 6 | 2.23818731 | -3.43317342 | 2.02007246 |
| 6 | 3.23736072 | -2.32690692 | 1.69275296 |
| 8 | 2.42280746 | -4.55436993 | 1.59808028 |
| 6 | 4.59548998 | -2.60120463 | 2.33258319 |
| 6 | 1.37984014 | -1.81119561 | 4.79104519 |
| 1 | -0.13819498 | -1.65829384 | 3.24501753 |
| 1 | 3.33904910 | -2.33372784 | 0.59791136 |
| 1 | 5.30959034 | -1.82988381 | 2.01941085 |
| 1 | 4.95786428 | -3.58199239 | 2.00304794 |
| 1 | 4.52673340 | -2.59747338 | 3.42841983 |
| 1 | 1.11103940 | -0.84892058 | 5.24423027 |
| 1 | 2.46350026 | -1.95881927 | 4.89184952 |
| 1 | 0.86351883 | -2.61492229 | 5.33041620 |

Electronic energy = -4057.834245 a.u.
DFT-D3(BJ) dispersion correction = -0.138976 a.u.
Thermal free energy = 0.718055 a.u.
Gibbs free energy = -4057.255165 a.u.
Number of imaginary frequencies = 1.

**<u>TS3 eq LA isomer/conformer 5</u>**

| Atomic N. | X | Y | Z |
|---|---|---|---|
| 7 | 2.08667445 | 2.94993615 | 0.96625066 |
| 8 | -0.27983677 | 1.32970548 | 1.59989774 |
| 8 | 0.76949579 | 1.73354614 | -1.39602387 |
| 6 | 3.08837724 | 2.78905845 | 2.04840684 |
| 6 | -0.39442331 | 2.18255448 | 2.64222288 |
| 6 | -1.19919240 | 1.82031333 | 3.76628184 |
| 6 | -1.27352130 | 2.73071265 | 4.82963371 |
| 6 | -0.60559219 | 3.95823312 | 4.82544565 |
| 6 | 0.15663585 | 4.30937386 | 3.71690702 |
| 6 | 0.25708601 | 3.44200754 | 2.62374711 |
| 6 | 0.99505979 | 3.88918376 | 1.39319491 |

| | | | |
|---|---|---|---|
| 6 | -1.97052491 | 0.48663515 | 3.81168175 |
| 6 | 0.90932649 | 2.97173691 | -1.91685879 |
| 6 | 0.10426376 | 3.36543608 | -3.03004670 |
| 6 | 0.28718808 | 4.65886021 | -3.53731036 |
| 6 | 1.21198511 | 5.55977821 | -3.00214338 |
| 6 | 1.99498761 | 5.16380548 | -1.92430902 |
| 6 | 1.86156452 | 3.87878442 | -1.38532150 |
| 6 | 2.78260827 | 3.46141648 | -0.27033114 |
| 6 | -0.91890121 | 2.40387726 | -3.66465878 |
| 6 | -2.78436375 | 0.32817584 | 5.10977602 |
| 6 | -0.98336357 | -0.70177370 | 3.74340343 |
| 6 | -2.97083449 | 0.41873750 | 2.63323665 |
| 6 | -1.66445911 | 3.04697061 | -4.84942150 |
| 6 | -1.98817658 | 1.99495399 | -2.62391782 |
| 6 | -0.18667288 | 1.15422702 | -4.20923805 |
| 1 | 3.50884581 | 3.77297735 | 2.31442547 |
| 1 | 2.61181736 | 2.35663819 | 2.93474340 |
| 1 | 1.43743622 | 4.88589191 | 1.56122386 |
| 1 | 0.30995923 | 3.97618341 | 0.53698212 |
| 1 | -1.87412155 | 2.47595048 | 5.70094919 |
| 1 | 0.67190015 | 5.27199984 | 3.68113828 |
| 1 | 3.45395851 | 2.64825058 | -0.58834225 |
| 1 | 3.40836763 | 4.32141924 | 0.02247104 |
| 1 | -0.31672686 | 4.98399496 | -4.38248682 |
| 1 | 2.72931075 | 5.84797955 | -1.49296379 |
| 1 | -3.31861067 | -0.63238370 | 5.08147430 |
| 1 | -2.14341736 | 0.32344049 | 6.00324583 |
| 1 | -3.53666234 | 1.12145257 | 5.22533321 |
| 1 | -0.39213291 | -0.67173684 | 2.82189012 |
| 1 | -0.29765278 | -0.68207341 | 4.60351992 |
| 1 | -1.53564584 | -1.65361238 | 3.77505183 |
| 1 | -3.71961570 | 1.21947813 | 2.71858740 |
| 1 | -2.46308088 | 0.52595764 | 1.66816986 |
| 1 | -3.50364780 | -0.54462850 | 2.64813375 |
| 1 | -2.37295079 | 2.31560254 | -5.26425695 |
| 1 | -0.98085123 | 3.34006691 | -5.65910816 |
| 1 | -2.24302363 | 3.93092442 | -4.54444504 |
| 1 | -1.54057503 | 1.50428379 | -1.75320446 |
| 1 | -2.54207802 | 2.87830830 | -2.27410436 |
| 1 | -2.71175098 | 1.30387604 | -3.08317947 |
| 1 | 0.50907457 | 1.43832064 | -5.01223421 |
| 1 | 0.38349751 | 0.64819115 | -3.42251515 |
| 1 | -0.91613889 | 0.44506875 | -4.63061285 |
| 1 | 3.88690114 | 2.12589264 | 1.70300341 |
| 8 | 2.96724653 | 0.28626874 | 0.49417800 |
| 31 | 1.13018501 | 1.17697251 | 0.35757554 |
| 1 | -0.68960780 | 4.63125229 | 5.67859221 |
| 1 | 1.31446087 | 6.55820942 | -3.42689276 |
| 8 | -4.13236666 | -7.02009535 | -1.32718456 |
| 6 | -4.16979122 | -8.39896679 | -0.88595879 |
| 1 | -3.34415126 | -8.96710777 | -1.33249950 |
| 6 | -3.05289793 | -4.91621685 | -1.49227118 |
| 6 | -1.77784514 | -4.12481403 | -1.20363879 |

| | | | |
|---|---|---|---|
| 6 | -1.86469603 | -2.67665601 | -1.70224571 |
| 6 | -2.99199796 | -6.34805727 | -0.99557620 |
| 6 | -0.57326156 | -1.86485934 | -1.51678050 |
| 6 | -0.23768710 | -1.58397460 | -0.05131140 |
| 1 | -3.27268863 | -4.93969631 | -2.57189846 |
| 1 | -0.92349243 | -4.63079882 | -1.68125689 |
| 1 | -2.12344575 | -2.68127751 | -2.77400160 |
| 1 | -0.66611761 | -0.89926451 | -2.03324580 |
| 1 | -3.92951012 | -4.43739033 | -1.02557170 |
| 1 | -1.58132350 | -4.15545845 | -0.12206914 |
| 1 | -2.69613099 | -2.15921926 | -1.19250476 |
| 1 | 0.26675588 | -2.39618707 | -1.99601364 |
| 1 | -0.06036467 | -2.52624655 | 0.50048107 |
| 1 | -1.09520161 | -1.09427226 | 0.44285932 |
| 8 | -2.06983232 | -6.85242510 | -0.38262534 |
| 8 | 0.90544599 | -0.74861759 | 0.12143901 |
| 1 | -5.13528919 | -8.78521442 | -1.22623062 |
| 1 | -4.09185457 | -8.45427608 | 0.20709182 |
| 8 | 3.21280885 | -1.27583826 | -1.07559383 |
| 6 | 2.81975174 | -0.94171858 | 0.17478859 |
| 6 | 2.92113471 | -2.08376479 | 1.19716418 |
| 8 | 4.16674471 | -2.82193494 | 0.95898014 |
| 6 | 4.36589622 | -3.27077651 | -0.31448472 |
| 6 | 3.37473011 | -2.70270085 | -1.33666408 |
| 8 | 5.26993465 | -4.03026247 | -0.57397550 |
| 6 | 3.84109735 | -2.86256409 | -2.76788354 |
| 6 | 2.94374371 | -1.61089110 | 2.63362932 |
| 1 | 2.08824110 | -2.78498340 | 1.03533661 |
| 1 | 2.39786482 | -3.20005226 | -1.19366932 |
| 1 | 3.09712768 | -2.43709493 | -3.45232487 |
| 1 | 3.97327232 | -3.92746735 | -2.99173021 |
| 1 | 4.80314589 | -2.35828447 | -2.91756225 |
| 1 | 2.00626397 | -1.09127080 | 2.86554337 |
| 1 | 3.78095102 | -0.92274749 | 2.79893327 |
| 1 | 3.04497480 | -2.47321796 | 3.30367875 |

Electronic energy = -4057.834380 a.u.
DFT-D3(BJ) dispersion correction = -0.139823 a.u.
Thermal free energy = 0.719104 a.u.
Gibbs free energy = -4057.255099 a.u.
Number of imaginary frequencies = 1.

**TS3 eq LA isomer/conformer 6**

| Atomic N. | X | Y | Z |
|---|---|---|---|
| 7 | 2.19912148 | 2.26176143 | -1.35564518 |
| 8 | 0.27249065 | 2.16020131 | 0.86735898 |
| 8 | -0.16931640 | 0.59624445 | -1.89046800 |
| 6 | 3.53782654 | 2.40241075 | -0.73454314 |
| 6 | 0.59948391 | 3.46608138 | 1.01742411 |
| 6 | 0.24628004 | 4.14303780 | 2.22768116 |
| 6 | 0.65014440 | 5.47788620 | 2.36876392 |
| 6 | 1.35962868 | 6.16474676 | 1.38125408 |
| 6 | 1.65635514 | 5.51265574 | 0.19087733 |

| | | | |
|---|---|---|---|
| 6 | 1.26849329 | 4.18280935 | -0.00952861 |
| 6 | 1.48023307 | 3.57593107 | -1.37013721 |
| 6 | -0.56827152 | 3.45348549 | 3.34089255 |
| 6 | -0.09944257 | 1.16153681 | -3.11450410 |
| 6 | -1.25789547 | 1.16887784 | -3.95304465 |
| 6 | -1.13686180 | 1.74579287 | -5.22412872 |
| 6 | 0.05134816 | 2.31649232 | -5.68798351 |
| 6 | 1.16667783 | 2.31878543 | -4.85925817 |
| 6 | 1.10736716 | 1.74052942 | -3.58563256 |
| 6 | 2.36058784 | 1.71495390 | -2.75095558 |
| 6 | -2.59765983 | 0.56963718 | -3.48334837 |
| 6 | -0.89203948 | 4.40876961 | 4.50553370 |
| 6 | 0.23390457 | 2.27290249 | 3.93200111 |
| 6 | -1.91945803 | 2.95594549 | 2.77428532 |
| 6 | -3.69492054 | 0.68288153 | -4.55861902 |
| 6 | -3.10418010 | 1.32977653 | -2.23469925 |
| 6 | -2.42187095 | -0.93375784 | -3.16623688 |
| 1 | 4.12926435 | 3.15037727 | -1.28829825 |
| 1 | 3.43197179 | 2.73370719 | 0.30429736 |
| 1 | 2.04418564 | 4.27973413 | -2.00608897 |
| 1 | 0.51533580 | 3.39590478 | -1.86700714 |
| 1 | 0.40271214 | 6.00866461 | 3.28599930 |
| 1 | 2.18001199 | 6.03731441 | -0.61149961 |
| 1 | 2.74111629 | 0.68907249 | -2.62386513 |
| 1 | 3.14286685 | 2.30283642 | -3.26038933 |
| 1 | -2.00420666 | 1.75960982 | -5.88151455 |
| 1 | 2.10513759 | 2.76490068 | -5.19644356 |
| 1 | -1.49010277 | 3.86833215 | 5.25335121 |
| 1 | 0.01458658 | 4.77286959 | 5.00986528 |
| 1 | -1.48028660 | 5.27701998 | 4.17603970 |
| 1 | 0.48107710 | 1.54104292 | 3.15613365 |
| 1 | 1.16996777 | 2.62974620 | 4.38602304 |
| 1 | -0.35266697 | 1.76870763 | 4.71532536 |
| 1 | -2.52159405 | 3.80470467 | 2.41915679 |
| 1 | -1.77674603 | 2.26394415 | 1.93724215 |
| 1 | -2.49138379 | 2.44389844 | 3.56336594 |
| 1 | -4.62251139 | 0.23914666 | -4.16915655 |
| 1 | -3.43049598 | 0.14134747 | -5.47832823 |
| 1 | -3.91382170 | 1.72814727 | -4.82027578 |
| 1 | -2.37774205 | 1.29116201 | -1.41573870 |
| 1 | -3.29332805 | 2.38553023 | -2.47793412 |
| 1 | -4.05068588 | 0.88949615 | -1.88504589 |
| 1 | -2.12345695 | -1.48398185 | -4.07070971 |
| 1 | -1.65989840 | -1.09328985 | -2.39657044 |
| 1 | -3.37620544 | -1.35556817 | -2.81427455 |
| 1 | 4.04765272 | 1.43451858 | -0.75900096 |
| 8 | 2.48618531 | -0.31661627 | -0.23524611 |
| 31 | 0.91302669 | 0.90950710 | -0.39122266 |
| 1 | 1.66052580 | 7.20019197 | 1.53972459 |
| 1 | 0.09629607 | 2.75954676 | -6.68270206 |
| 8 | -1.95921052 | -7.45008230 | 1.25443149 |
| 6 | -1.35708618 | -8.56379700 | 1.96223223 |
| 1 | -0.36449099 | -8.78550911 | 1.55112600 |

| | | | |
|---|---|---|---|
| 6 | -1.91459262 | -5.18519592 | 0.51601273 |
| 6 | -1.87129927 | -3.82831454 | 1.23459506 |
| 6 | -2.02079368 | -2.64172196 | 0.27771080 |
| 6 | -1.25762951 | -6.28727198 | 1.32331598 |
| 6 | -2.00556612 | -1.27819669 | 0.97865629 |
| 6 | -0.66945523 | -0.88808024 | 1.61534309 |
| 1 | -1.34393525 | -5.11775160 | -0.42653948 |
| 1 | -0.91835445 | -3.76385474 | 1.78000331 |
| 1 | -1.21207595 | -2.66905355 | -0.47106427 |
| 1 | -2.76856923 | -1.25828922 | 1.77694166 |
| 1 | -2.93779874 | -5.47509956 | 0.24456342 |
| 1 | -2.66527128 | -3.79649043 | 1.99895597 |
| 1 | -2.96261954 | -2.74111152 | -0.28573376 |
| 1 | -2.28421974 | -0.49159798 | 0.26151216 |
| 1 | -0.33818704 | -1.67202306 | 2.31947327 |
| 1 | -0.79671288 | 0.03646810 | 2.20006776 |
| 8 | -0.21362846 | -6.16878176 | 1.94323194 |
| 8 | 0.36751708 | -0.69270003 | 0.64593387 |
| 1 | -2.03916049 | -9.40511322 | 1.80859518 |
| 1 | -1.25951576 | -8.32949066 | 3.02926850 |
| 8 | 2.00103188 | -2.49016666 | -0.28478813 |
| 6 | 2.10391068 | -1.34844816 | 0.43828323 |
| 6 | 2.54355979 | -1.57042027 | 1.89298892 |
| 8 | 3.63867307 | -2.54367542 | 1.90575933 |
| 6 | 3.40686107 | -3.72755218 | 1.26361895 |
| 6 | 2.11095572 | -3.75142407 | 0.44847295 |
| 8 | 4.19530773 | -4.63995504 | 1.34453380 |
| 6 | 2.08173013 | -4.87548971 | -0.56484020 |
| 6 | 3.05588031 | -0.32009816 | 2.57231164 |
| 1 | 1.70695889 | -2.00754404 | 2.46017313 |
| 1 | 1.26024568 | -3.84722543 | 1.14610636 |
| 1 | 1.16140175 | -4.81680298 | -1.15881073 |
| 1 | 2.11103082 | -5.83541298 | -0.03731075 |
| 1 | 2.94802356 | -4.81582165 | -1.23445153 |
| 1 | 2.26813722 | 0.44219998 | 2.60109782 |
| 1 | 3.91608191 | 0.08320499 | 2.02538562 |
| 1 | 3.35805202 | -0.55420977 | 3.60028553 |

Electronic energy = -4057.834300 a.u.
DFT-D3(BJ) dispersion correction = -0.143722 a.u.
Thermal free energy = 0.723908 a.u.
Gibbs free energy = -4057.254115 a.u.
Number of imaginary frequencies = 1.

**TS3 eq LA isomer/conformer 7**

| Atomic N. | X | Y | Z |
|---|---|---|---|
| 7 | 1.89682126 | 2.94065475 | 0.19643165 |
| 8 | -0.30327630 | 1.75755847 | 1.68164778 |
| 8 | 0.35519749 | 1.15569043 | -1.51423919 |
| 6 | 2.91924071 | 3.15295672 | 1.24977291 |
| 6 | -0.37033266 | 2.98379898 | 2.25391555 |
| 6 | -1.02260959 | 3.13716531 | 3.51776338 |
| 6 | -1.08420074 | 4.42609262 | 4.06508493 |

| | | | |
|---|---|---|---|
| 6 | -0.53083646 | 5.54470825 | 3.43784571 |
| 6 | 0.11346675 | 5.38213396 | 2.21751523 |
| 6 | 0.19070768 | 4.11999321 | 1.61818564 |
| 6 | 0.83730930 | 4.00287199 | 0.26617065 |
| 6 | -1.63132823 | 1.93811619 | 4.26996946 |
| 6 | 0.59429711 | 2.07808113 | -2.46873760 |
| 6 | -0.22156836 | 2.10206389 | -3.64218712 |
| 6 | 0.06467199 | 3.07462502 | -4.60957146 |
| 6 | 1.09689987 | 4.00617838 | -4.46598387 |
| 6 | 1.88461196 | 3.97162223 | -3.32127428 |
| 6 | 1.65175998 | 3.01106405 | -2.33014941 |
| 6 | 2.57837367 | 2.95238304 | -1.14586210 |
| 6 | -1.37145126 | 1.09619153 | -3.84551454 |
| 6 | -2.29851580 | 2.35443878 | 5.59483290 |
| 6 | -0.51192057 | 0.93117666 | 4.62644672 |
| 6 | -2.72432208 | 1.26599360 | 3.40637541 |
| 6 | -2.08780265 | 1.29926395 | -5.19392633 |
| 6 | -2.43101716 | 1.26929986 | -2.73216844 |
| 6 | -0.81770718 | -0.34847793 | -3.83291841 |
| 1 | 3.33826590 | 4.16879797 | 1.16151512 |
| 1 | 2.46662116 | 3.04071665 | 2.24034333 |
| 1 | 1.29087710 | 4.96887589 | -0.01274850 |
| 1 | 0.09692812 | 3.75906801 | -0.51041937 |
| 1 | -1.57976842 | 4.56730223 | 5.02365160 |
| 1 | 0.55759209 | 6.23972750 | 1.70709348 |
| 1 | 3.20057583 | 2.04370093 | -1.16799772 |
| 1 | 3.25489807 | 3.82349348 | -1.17604351 |
| 1 | -0.54234350 | 3.11551714 | -5.51211309 |
| 1 | 2.69909978 | 4.68747711 | -3.18809581 |
| 1 | -2.71409726 | 1.45968163 | 6.08023310 |
| 1 | -1.58415174 | 2.80845189 | 6.29660559 |
| 1 | -3.12755823 | 3.05890894 | 5.43624592 |
| 1 | 0.03452588 | 0.60350037 | 3.73505831 |
| 1 | 0.20631436 | 1.39069259 | 5.32140207 |
| 1 | -0.94113880 | 0.04575356 | 5.11964989 |
| 1 | -3.55350804 | 1.96630359 | 3.22910070 |
| 1 | -2.33401728 | 0.94595838 | 2.43525553 |
| 1 | -3.13108325 | 0.38788339 | 3.93083000 |
| 1 | -2.88978863 | 0.55311728 | -5.28761244 |
| 1 | -1.40866435 | 1.16406739 | -6.04810762 |
| 1 | -2.55159283 | 2.29318738 | -5.27164698 |
| 1 | -1.99832642 | 1.10792196 | -1.73918879 |
| 1 | -2.86321092 | 2.28009272 | -2.76415277 |
| 1 | -3.24891162 | 0.54714280 | -2.87759638 |
| 1 | -0.10157783 | -0.49314263 | -4.65538692 |
| 1 | -0.31557938 | -0.57009584 | -2.88497210 |
| 1 | -1.64122653 | -1.06567633 | -3.97123694 |
| 1 | 3.71568179 | 2.41337585 | 1.12741137 |
| 8 | 2.76921678 | 0.27930287 | 0.72213107 |
| 31 | 0.81754333 | 1.12373221 | 0.30644315 |
| 1 | -0.60084623 | 6.52721882 | 3.90433168 |
| 1 | 1.27653086 | 4.74989557 | -5.24215746 |
| 8 | -1.57581127 | -7.31842041 | -1.66755402 |

| | | | |
|---|---|---|---|
| 6 | -1.68129063 | -8.71476269 | -1.30339241 |
| 1 | -1.01677155 | -8.94143772 | -0.46024230 |
| 6 | -1.79670656 | -5.01383686 | -1.15412974 |
| 6 | -1.87392282 | -3.98942947 | -0.02235914 |
| 6 | -1.88342249 | -2.54452062 | -0.53568363 |
| 6 | -1.91668963 | -6.44656849 | -0.67242980 |
| 6 | -1.87498868 | -1.49451315 | 0.58389473 |
| 6 | -0.53915864 | -1.33511972 | 1.31776881 |
| 1 | -0.85699874 | -4.92041826 | -1.71918237 |
| 1 | -1.02657962 | -4.15647507 | 0.65988386 |
| 1 | -1.02304661 | -2.36589479 | -1.20120585 |
| 1 | -2.64068890 | -1.75414419 | 1.33614659 |
| 1 | -2.60635996 | -4.84814310 | -1.88604796 |
| 1 | -2.78069019 | -4.17608213 | 0.57295781 |
| 1 | -2.77843857 | -2.39435530 | -1.16214705 |
| 1 | -2.16009307 | -0.51035494 | 0.17990315 |
| 1 | -0.17868209 | -2.32254863 | 1.65869427 |
| 1 | -0.69667631 | -0.71959507 | 2.21633720 |
| 8 | -2.27974725 | -6.80268860 | 0.43256542 |
| 8 | 0.48651144 | -0.76429760 | 0.49489281 |
| 1 | -1.37829089 | -9.27071953 | -2.19601536 |
| 1 | -2.71271729 | -8.96310425 | -1.02214277 |
| 8 | 2.80108643 | -1.36438179 | -0.76900434 |
| 6 | 2.62983322 | -0.94892424 | 0.49224731 |
| 6 | 2.80754685 | -1.95347977 | 1.60953426 |
| 8 | 2.43898416 | -3.31664419 | 1.26289165 |
| 6 | 2.19176197 | -3.69683743 | -0.01633298 |
| 6 | 2.27170277 | -2.67117977 | -1.14124227 |
| 8 | 1.87122250 | -4.84232378 | -0.24922298 |
| 6 | 3.09063268 | -3.17854881 | -2.31868553 |
| 6 | 4.25840950 | -1.94878817 | 2.09655333 |
| 1 | 2.13191295 | -1.66598630 | 2.42519474 |
| 1 | 1.23019028 | -2.48008943 | -1.44636977 |
| 1 | 3.05902457 | -2.44185567 | -3.13049912 |
| 1 | 2.66599250 | -4.12425947 | -2.67271852 |
| 1 | 4.13580227 | -3.34644485 | -2.02741814 |
| 1 | 4.51977015 | -0.95416874 | 2.47812104 |
| 1 | 4.94793177 | -2.21412516 | 1.28405488 |
| 1 | 4.36570740 | -2.68212891 | 2.90513539 |

Electronic energy = -4057.830590 a.u.
DFT-D3(BJ) dispersion correction = -0.142823 a.u.
Thermal free energy = 0.719569 a.u.
Gibbs free energy = -4057.253844 a.u.
Number of imaginary frequencies = 1.

**TS3 eq LA isomer/conformer 8**

| Atomic N. | X | Y | Z |
|---|---|---|---|
| 7 | 0.91210055 | 2.14587688 | 2.92347765 |
| 8 | -1.38126183 | 1.11856902 | 1.41521740 |
| 8 | 1.62751234 | 1.34047222 | 0.18821993 |
| 6 | 0.73543262 | 1.76139987 | 4.34517479 |
| 6 | -2.07633495 | 1.95567179 | 2.21608353 |

| | | | |
|---|---|---|---|
| 6 | -3.47768688 | 1.75234127 | 2.40982366 |
| 6 | -4.15133286 | 2.65044713 | 3.24935603 |
| 6 | -3.51265931 | 3.71637201 | 3.88919902 |
| 6 | -2.15021825 | 3.90793014 | 3.68772459 |
| 6 | -1.43195093 | 3.04591084 | 2.85245609 |
| 6 | 0.02238961 | 3.31064796 | 2.58617043 |
| 6 | -4.22983313 | 0.59929061 | 1.71667993 |
| 6 | 2.29244590 | 2.51344943 | 0.16787259 |
| 6 | 2.65643597 | 3.10039854 | -1.08263576 |
| 6 | 3.34945607 | 4.31793165 | -1.04618025 |
| 6 | 3.69020343 | 4.96201992 | 0.14710920 |
| 6 | 3.33965802 | 4.37525558 | 1.35768151 |
| 6 | 2.65636587 | 3.15398693 | 1.37815320 |
| 6 | 2.36129379 | 2.49943113 | 2.69942236 |
| 6 | 2.31659436 | 2.41779780 | -2.42171764 |
| 6 | -5.72602081 | 0.57849234 | 2.08196354 |
| 6 | -3.63536668 | -0.76032203 | 2.15377593 |
| 6 | -4.13440037 | 0.76313925 | 0.18171012 |
| 6 | 2.81949496 | 3.23015070 | -3.62984776 |
| 6 | 0.78424150 | 2.27006626 | -2.57548833 |
| 6 | 2.99552345 | 1.02872920 | -2.48553967 |
| 1 | 0.91680455 | 2.63715720 | 4.99006224 |
| 1 | -0.28556505 | 1.40349579 | 4.51295042 |
| 1 | 0.35879242 | 4.18571997 | 3.16755629 |
| 1 | 0.19517033 | 3.53820300 | 1.52402103 |
| 1 | -5.21857738 | 2.51644206 | 3.41645408 |
| 1 | -1.62999403 | 4.73952341 | 4.16866112 |
| 1 | 2.91552496 | 1.55361354 | 2.80980611 |
| 1 | 2.67611790 | 3.17251134 | 3.51494718 |
| 1 | 3.63523865 | 4.79011631 | -1.98440909 |
| 1 | 3.60234761 | 4.85569954 | 2.30306411 |
| 1 | -6.20969820 | -0.25890326 | 1.55884492 |
| 1 | -5.88780594 | 0.43332058 | 3.15991855 |
| 1 | -6.23966885 | 1.50036943 | 1.77393472 |
| 1 | -2.57432604 | -0.83091521 | 1.89199388 |
| 1 | -3.74240160 | -0.89390326 | 3.24067688 |
| 1 | -4.17288256 | -1.58275199 | 1.65755939 |
| 1 | -4.62477541 | 1.69515336 | -0.13459498 |
| 1 | -3.09262919 | 0.79152042 | -0.15560590 |
| 1 | -4.64379883 | -0.07423925 | -0.31936061 |
| 1 | 2.55745173 | 2.69505596 | -4.55375719 |
| 1 | 3.91192579 | 3.35442901 | -3.61913419 |
| 1 | 2.35598254 | 4.22604609 | -3.68168426 |
| 1 | 0.35445568 | 1.66597295 | -1.76942539 |
| 1 | 0.29764640 | 3.25633621 | -2.56734920 |
| 1 | 0.55111408 | 1.78648901 | -3.53654981 |
| 1 | 4.08938169 | 1.13256514 | -2.43761516 |
| 1 | 2.67015958 | 0.38798285 | -1.65853179 |
| 1 | 2.74526834 | 0.53256470 | -3.43590713 |
| 1 | 1.44563413 | 0.96770239 | 4.59333992 |
| 8 | 1.32602644 | -0.66757083 | 2.89431453 |
| 31 | 0.44780704 | 0.65908229 | 1.47960401 |
| 1 | -4.07930279 | 4.38725233 | 4.53480291 |

| | | | |
|---|---|---|---|
| 1 | 4.22430658 | 5.91179466 | 0.12396276 |
| 8 | -1.51997769 | -3.95242381 | -7.28959703 |
| 6 | -0.98761570 | -4.40915537 | -8.55556107 |
| 1 | -0.38967082 | -3.61989903 | -9.02857590 |
| 6 | -1.22794032 | -3.12377906 | -5.08821106 |
| 6 | -0.22854121 | -2.77570009 | -3.98576403 |
| 6 | -0.91720659 | -2.28536057 | -2.70773745 |
| 6 | -0.57253426 | -3.59425974 | -6.37186146 |
| 6 | 0.07493273 | -1.93717492 | -1.59198451 |
| 6 | -0.63028455 | -1.46625531 | -0.32022601 |
| 1 | -1.85980511 | -2.25496674 | -5.33722925 |
| 1 | 0.46716180 | -2.00667524 | -4.35464382 |
| 1 | -1.53168607 | -1.39802921 | -2.93976235 |
| 1 | 0.76244688 | -1.14703786 | -1.93066561 |
| 1 | -1.92776334 | -3.90875268 | -4.75887918 |
| 1 | 0.38823235 | -3.65960789 | -3.76170444 |
| 1 | -1.61717629 | -3.05972004 | -2.34810233 |
| 1 | 0.68992513 | -2.82476497 | -1.36821508 |
| 1 | -1.29115558 | -2.26963210 | 0.05812966 |
| 1 | -1.28675342 | -0.61114675 | -0.55659020 |
| 8 | 0.62297875 | -3.65339875 | -6.58780718 |
| 8 | 0.28016749 | -1.08768916 | 0.71320248 |
| 1 | -1.86215425 | -4.65199280 | -9.16687965 |
| 1 | -0.35700935 | -5.29532385 | -8.40991879 |
| 8 | 2.47430038 | -2.27056527 | 1.88529360 |
| 6 | 1.32780433 | -1.82798994 | 2.40405726 |
| 6 | 0.29829401 | -2.84151769 | 2.85946918 |
| 8 | 0.18603805 | -4.00046539 | 1.98994505 |
| 6 | 1.22408295 | -4.38083696 | 1.20699966 |
| 6 | 2.49080181 | -3.53062129 | 1.14671969 |
| 8 | 1.14123034 | -5.38508511 | 0.53407747 |
| 6 | 3.71665645 | -4.32096243 | 1.59025419 |
| 6 | 0.59287393 | -3.29965687 | 4.28986120 |
| 1 | -0.68224758 | -2.35420632 | 2.80904174 |
| 1 | 2.59487581 | -3.23500848 | 0.09136842 |
| 1 | 4.61541510 | -3.70554304 | 1.46256852 |
| 1 | 3.80648279 | -5.22232151 | 0.97332126 |
| 1 | 3.63579392 | -4.61841297 | 2.64419460 |
| 1 | 0.55953789 | -2.43909287 | 4.96986055 |
| 1 | 1.58131492 | -3.77268219 | 4.36505461 |
| 1 | -0.16963899 | -4.02557755 | 4.59808588 |

Electronic energy = -4057.833477 a.u.
DFT-D3(BJ) dispersion correction = -0.139255 a.u.
Thermal free energy = 0.719361 a.u.
Gibbs free energy = -4057.253370 a.u.
Number of imaginary frequencies = 1.

**TS3_eq LA isomer/conformer 9**

| Atomic N. | X | Y | Z |
|---|---|---|---|
| 7 | 3.21455383 | 1.92882550 | -0.28970677 |
| 8 | 0.86057270 | 1.60908902 | 1.44304800 |
| 8 | 0.85984629 | 0.71867049 | -1.63130295 |

| | | | |
|---|---|---|---|
| 6 | 4.44787216 | 1.68453383 | 0.49733877 |
| 6 | 1.37671280 | 2.68181300 | 2.08344007 |
| 6 | 0.94804132 | 2.98563623 | 3.41239905 |
| 6 | 1.52901840 | 4.09527731 | 4.04203415 |
| 6 | 2.48711514 | 4.90311050 | 3.42369294 |
| 6 | 2.87595153 | 4.61391020 | 2.12047982 |
| 6 | 2.32129836 | 3.52314162 | 1.44232547 |
| 6 | 2.66893792 | 3.29835272 | -0.00237443 |
| 6 | -0.12480449 | 2.14200664 | 4.12889433 |
| 6 | 1.14027739 | 1.62347507 | -2.59406376 |
| 6 | 0.13421535 | 1.98222661 | -3.54302096 |
| 6 | 0.47487733 | 2.92434311 | -4.52311993 |
| 6 | 1.74055481 | 3.51174641 | -4.60350227 |
| 6 | 2.71473646 | 3.14730048 | -3.68162227 |
| 6 | 2.43139458 | 2.20306706 | -2.68783307 |
| 6 | 3.53313136 | 1.77478898 | -1.75631082 |
| 6 | -1.26930451 | 1.34657931 | -3.50893831 |
| 6 | -0.44091362 | 2.67718244 | 5.53818989 |
| 6 | 0.36349311 | 0.68395776 | 4.29246855 |
| 6 | -1.44328189 | 2.17538977 | 3.31989026 |
| 6 | -2.17968011 | 1.88964701 | -4.62671757 |
| 6 | -1.96874523 | 1.65469193 | -2.16376901 |
| 6 | -1.14817655 | -0.18166254 | -3.71748018 |
| 1 | 5.19830322 | 2.45683455 | 0.26055592 |
| 1 | 4.22048616 | 1.72846520 | 1.56788766 |
| 1 | 3.41035295 | 4.04608679 | -0.33193135 |
| 1 | 1.78032160 | 3.41662049 | -0.63987041 |
| 1 | 1.22635877 | 4.34367371 | 5.05765724 |
| 1 | 3.60575914 | 5.24419498 | 1.60711694 |
| 1 | 3.78258824 | 0.71074355 | -1.89348125 |
| 1 | 4.43964291 | 2.36534548 | -1.97159982 |
| 1 | -0.27525580 | 3.21841502 | -5.25493002 |
| 1 | 3.71381855 | 3.58656335 | -3.72922969 |
| 1 | -1.21700859 | 2.04585934 | 5.99430895 |
| 1 | 0.43760836 | 2.65020490 | 6.19897699 |
| 1 | -0.82558745 | 3.70669770 | 5.51252651 |
| 1 | 0.57346714 | 0.22681823 | 3.31959009 |
| 1 | 1.27767479 | 0.65040869 | 4.90371180 |
| 1 | -0.40535635 | 0.08391583 | 4.80332136 |
| 1 | -1.83273947 | 3.20210361 | 3.26281095 |
| 1 | -1.29749990 | 1.80677462 | 2.29837036 |
| 1 | -2.20402455 | 1.55279076 | 3.81559873 |
| 1 | -3.16090727 | 1.39849889 | -4.55777502 |
| 1 | -1.77631366 | 1.68042958 | -5.62780523 |
| 1 | -2.34501839 | 2.97304416 | -4.53667641 |
| 1 | -1.39661002 | 1.27090359 | -1.31257725 |
| 1 | -2.09428430 | 2.73953009 | -2.03367710 |
| 1 | -2.96946669 | 1.19575477 | -2.15047240 |
| 1 | -0.72729188 | -0.40089154 | -4.70951796 |
| 1 | -0.50193602 | -0.63946027 | -2.96036148 |
| 1 | -2.14355326 | -0.64936793 | -3.66381359 |
| 1 | 4.84322166 | 0.69536054 | 0.24850237 |
| 8 | 3.06040597 | -0.88441372 | 0.12828737 |

| | | | |
|---|---|---|---|
| 31 | 1.63833785 | 0.58033270 | 0.06761807 |
| 1 | 2.91436505 | 5.75268364 | 3.95618892 |
| 1 | 1.95682323 | 4.24459982 | -5.38063622 |
| 8 | -7.29881430 | -3.91007614 | -0.82392967 |
| 6 | -8.18432522 | -4.62795925 | -1.71698678 |
| 1 | -8.14527702 | -4.19592953 | -2.72476196 |
| 6 | -5.13746214 | -3.17760825 | -0.18215206 |
| 6 | -3.69359684 | -2.97122002 | -0.63933641 |
| 6 | -2.83232546 | -2.28703475 | 0.42748812 |
| 6 | -5.98419809 | -3.92858458 | -1.19208896 |
| 6 | -1.39308453 | -2.04033160 | -0.03947971 |
| 6 | -0.53364301 | -1.40913165 | 1.05494845 |
| 1 | -5.63155746 | -2.21829748 | 0.03635136 |
| 1 | -3.68628144 | -2.37172604 | -1.56292081 |
| 1 | -3.29122615 | -1.32419193 | 0.71111119 |
| 1 | -1.38921511 | -1.37840033 | -0.91763765 |
| 1 | -5.17174387 | -3.74876475 | 0.76130825 |
| 1 | -3.25669646 | -3.94472265 | -0.90852726 |
| 1 | -2.82650542 | -2.90534925 | 1.34290946 |
| 1 | -0.94338816 | -2.99437642 | -0.36333650 |
| 1 | -0.47527781 | -2.08727098 | 1.92973638 |
| 1 | -1.00674498 | -0.48075700 | 1.41894400 |
| 8 | -5.57566023 | -4.48778296 | -2.19210672 |
| 8 | 0.79136384 | -1.08667266 | 0.63943672 |
| 1 | -9.18344402 | -4.51615715 | -1.28512990 |
| 1 | -7.89995527 | -5.68640804 | -1.77072501 |
| 8 | 2.29323578 | -2.79625440 | -0.72036546 |
| 6 | 2.46021581 | -1.99025345 | 0.35313758 |
| 6 | 2.56943440 | -2.73412442 | 1.69300056 |
| 8 | 3.39076471 | -3.93510509 | 1.50317979 |
| 6 | 3.00969195 | -4.77246141 | 0.49476337 |
| 6 | 1.94284546 | -4.18343019 | -0.43493024 |
| 8 | 3.50936842 | -5.86633968 | 0.37090537 |
| 6 | 1.83451438 | -4.91575098 | -1.75542331 |
| 6 | 3.21474409 | -1.91521609 | 2.78872848 |
| 1 | 1.56637371 | -3.06762147 | 2.00017262 |
| 1 | 0.97104359 | -4.19978094 | 0.09239289 |
| 1 | 1.05674314 | -4.45393467 | -2.37558103 |
| 1 | 1.57578540 | -5.96486187 | -1.57117665 |
| 1 | 2.79111409 | -4.88675976 | -2.29051304 |
| 1 | 2.60503387 | -1.02701533 | 2.99336553 |
| 1 | 4.21878147 | -1.59432948 | 2.48757148 |
| 1 | 3.28499246 | -2.51462984 | 3.70454431 |

Electronic energy = -4057.835487 a.u.
DFT-D3(BJ) dispersion correction = -0.139524 a.u.
Thermal free energy = 0.722128 a.u.
Gibbs free energy = -4057.252883 a.u.
Number of imaginary frequencies = 1.

**TS3 eq LA isomer/conformer 10**

| Atomic N. | X | Y | Z |
|---|---|---|---|
| 7 | -1.32533777 | 2.67824745 | 2.47474360 |

| | | | |
|---|---|---|---|
| 8 | -1.95073068 | 0.83468461 | 0.26921776 |
| 8 | 1.05566823 | 1.88364100 | 0.95270824 |
| 6 | -2.24480391 | 2.36397338 | 3.59552217 |
| 6 | -3.14049625 | 1.48006403 | 0.29170543 |
| 6 | -4.29191637 | 0.86554772 | -0.29130715 |
| 6 | -5.49885368 | 1.57742655 | -0.24812268 |
| 6 | -5.61376715 | 2.84224916 | 0.33397245 |
| 6 | -4.48643541 | 3.43243742 | 0.89347076 |
| 6 | -3.25434542 | 2.77011871 | 0.86889237 |
| 6 | -2.03775954 | 3.46504068 | 1.41188645 |
| 6 | -4.22250605 | -0.52519435 | -0.95119518 |
| 6 | 1.34130347 | 3.20114279 | 0.98757052 |
| 6 | 2.26020217 | 3.75148225 | 0.04256688 |
| 6 | 2.53542113 | 5.12266254 | 0.13398615 |
| 6 | 1.94963336 | 5.95151186 | 1.09559751 |
| 6 | 1.05708778 | 5.40367317 | 2.01000786 |
| 6 | 0.75744367 | 4.03719378 | 1.97130001 |
| 6 | -0.15299967 | 3.45359731 | 3.01668191 |
| 6 | 2.92758417 | 2.87552738 | -1.03590584 |
| 6 | -5.58394289 | -0.97248429 | -1.51667476 |
| 6 | -3.79564404 | -1.58388567 | 0.09301134 |
| 6 | -3.22889137 | -0.49305442 | -2.13637638 |
| 6 | 3.88953853 | 3.68573594 | -1.92508280 |
| 6 | 1.85160112 | 2.26266122 | -1.96345782 |
| 6 | 3.75520468 | 1.75146592 | -0.36835676 |
| 1 | -2.66969347 | 3.29713249 | 4.00137663 |
| 1 | -3.06010437 | 1.72428286 | 3.24302578 |
| 1 | -2.32215810 | 4.44521999 | 1.83127737 |
| 1 | -1.30152297 | 3.65221691 | 0.61623913 |
| 1 | -6.39048624 | 1.12918103 | -0.68249649 |
| 1 | -4.54761028 | 4.42404938 | 1.34770441 |
| 1 | 0.38907367 | 2.75142479 | 3.67090034 |
| 1 | -0.54506826 | 4.26675177 | 3.65123653 |
| 1 | 3.23069715 | 5.56981277 | -0.57419550 |
| 1 | 0.58921891 | 6.03141832 | 2.77192664 |
| 1 | -5.46981478 | -1.96410239 | -1.97799146 |
| 1 | -6.35051823 | -1.05898714 | -0.73316830 |
| 1 | -5.95480347 | -0.28836963 | -2.29328346 |
| 1 | -2.83067584 | -1.33885992 | 0.54771900 |
| 1 | -4.54672050 | -1.65424287 | 0.89347112 |
| 1 | -3.71839809 | -2.57243109 | -0.38618410 |
| 1 | -3.58766484 | 0.19384284 | -2.91662836 |
| 1 | -2.23438287 | -0.16049466 | -1.82049108 |
| 1 | -3.14153433 | -1.49558651 | -2.58296275 |
| 1 | 4.33968401 | 3.01353216 | -2.66950154 |
| 1 | 4.70972872 | 4.13373470 | -1.34578311 |
| 1 | 3.37235785 | 4.48652887 | -2.47311926 |
| 1 | 1.14716220 | 1.63719046 | -1.40456045 |
| 1 | 1.28377903 | 3.05435514 | -2.47351980 |
| 1 | 2.33349204 | 1.64228129 | -2.73478603 |
| 1 | 4.55262899 | 2.17874599 | 0.25713670 |
| 1 | 3.12371564 | 1.11237705 | 0.25850487 |
| 1 | 4.23084974 | 1.12830949 | -1.14128888 |

| | | | |
|---|---|---|---|
| 1 | -1.69044185 | 1.83896363 | 4.37913465 |
| 8 | -0.48027241 | 0.06587870 | 3.26420856 |
| 31 | -0.51391846 | 0.99173641 | 1.47489190 |
| 1 | -6.57518435 | 3.35551214 | 0.34647152 |
| 1 | 2.19046235 | 7.01411104 | 1.12276435 |
| 8 | 3.07100511 | -4.77366304 | -6.01698160 |
| 6 | 4.20582819 | -5.29728127 | -6.74804306 |
| 1 | 4.97437429 | -4.52359581 | -6.86902094 |
| 6 | 2.10947108 | -3.82517838 | -4.06866503 |
| 6 | 2.38822341 | -3.15058422 | -2.72579479 |
| 6 | 1.10638034 | -2.69031525 | -2.02377343 |
| 6 | 3.36207366 | -4.33056831 | -4.75839138 |
| 6 | 1.37706220 | -1.98452413 | -0.68960404 |
| 6 | 0.08053562 | -1.56116199 | 0.00310684 |
| 1 | 1.58755076 | -3.14467192 | -4.75963688 |
| 1 | 3.05506063 | -2.28859782 | -2.88316727 |
| 1 | 0.54678518 | -2.00872660 | -2.68727469 |
| 1 | 1.99515939 | -1.08894539 | -0.85902357 |
| 1 | 1.43437159 | -4.68848228 | -3.94161081 |
| 1 | 2.94365549 | -3.84672666 | -2.07914519 |
| 1 | 0.44841400 | -3.56157112 | -1.85579562 |
| 1 | 1.96167707 | -2.65131497 | -0.03515012 |
| 1 | -0.52235800 | -2.45001650 | 0.26577550 |
| 1 | -0.53649300 | -0.98168921 | -0.70239288 |
| 8 | 4.48012018 | -4.35852432 | -4.28005600 |
| 8 | 0.29228559 | -0.76992559 | 1.17254651 |
| 1 | 3.80743599 | -5.60374451 | -7.71998453 |
| 1 | 4.64176893 | -6.15395212 | -6.21870089 |
| 8 | -1.00597930 | -2.09622073 | 3.09625888 |
| 6 | -0.06259775 | -1.13591492 | 3.11679339 |
| 6 | 1.31137657 | -1.47853661 | 3.73136163 |
| 8 | 1.17315567 | -2.49732661 | 4.77839613 |
| 6 | 0.37335539 | -3.57029271 | 4.56600094 |
| 6 | -0.67377245 | -3.47104669 | 3.46125531 |
| 8 | 0.45282513 | -4.55577326 | 5.26572418 |
| 6 | -0.35191676 | -4.36097288 | 2.26368022 |
| 6 | 2.46355557 | -1.84347749 | 2.81203032 |
| 1 | 1.57911825 | -0.57899332 | 4.29875898 |
| 1 | -1.60556078 | -3.82987857 | 3.91886473 |
| 1 | -1.18433893 | -4.33482599 | 1.55015743 |
| 1 | -0.22709008 | -5.39057446 | 2.62355947 |
| 1 | 0.56766140 | -4.05510139 | 1.75114715 |
| 1 | 2.64996576 | -1.02327311 | 2.11076403 |
| 1 | 2.25845981 | -2.75240064 | 2.23467970 |
| 1 | 3.35726118 | -2.01261115 | 3.42696047 |

Electronic energy = -4057.823589 a.u.
DFT-D3(BJ) dispersion correction = -0.140558 a.u.
Thermal free energy = 0.720870 a.u.
Gibbs free energy = -4057.243277 a.u.
Number of imaginary frequencies = 1.

**TS3_eq_LA isomer/conformer 11**

| Atomic N. | X | Y | Z |
|---|---|---|---|
| 7 | 2.50525618 | 2.51298118 | -0.12226492 |
| 8 | 0.26779148 | 1.68761384 | 1.54267526 |
| 8 | 0.50874382 | 1.07009780 | -1.70221102 |
| 6 | 3.64600325 | 2.47598028 | 0.82473814 |
| 6 | 0.49806336 | 2.87398458 | 2.15544534 |
| 6 | 0.02669362 | 3.08306098 | 3.49055362 |
| 6 | 0.26377162 | 4.33392668 | 4.07684851 |
| 6 | 0.94606149 | 5.36227083 | 3.42306447 |
| 6 | 1.41972697 | 5.14186525 | 2.13569283 |
| 6 | 1.19668841 | 3.91740274 | 1.49611652 |
| 6 | 1.67774534 | 3.74760818 | 0.08219946 |
| 6 | -0.70281607 | 1.97841895 | 4.27917385 |
| 6 | 0.80862105 | 1.98310232 | -2.65154982 |
| 6 | -0.09996736 | 2.20240045 | -3.73439932 |
| 6 | 0.25322801 | 3.15861320 | -4.69602585 |
| 6 | 1.43984938 | 3.89380574 | -4.63559818 |
| 6 | 2.31883764 | 3.66852903 | -3.58323574 |
| 6 | 2.02181911 | 2.71561337 | -2.60195756 |
| 6 | 3.03967381 | 2.45171642 | -1.52566254 |
| 6 | -1.41593528 | 1.41096568 | -3.85918093 |
| 6 | -1.12797296 | 2.44343638 | 5.68517494 |
| 6 | 0.24748714 | 0.77244091 | 4.47139168 |
| 6 | -1.99081683 | 1.55475545 | 3.53611565 |
| 6 | -2.22215152 | 1.81191254 | -5.10902596 |
| 6 | -2.31655693 | 1.67723262 | -2.63080215 |
| 6 | -1.10165322 | -0.09859292 | -3.98269725 |
| 1 | 4.25821829 | 3.38562202 | 0.70847154 |
| 1 | 3.27701497 | 2.42467642 | 1.85418856 |
| 1 | 2.27505732 | 4.62670946 | -0.21385194 |
| 1 | 0.83398896 | 3.67990804 | -0.62081504 |
| 1 | -0.09144017 | 4.51606131 | 5.08928251 |
| 1 | 1.96392870 | 5.92570353 | 1.60402191 |
| 1 | 3.48458695 | 1.44948196 | -1.63158393 |
| 1 | 3.85558128 | 3.18941832 | -1.61451876 |
| 1 | -0.42421123 | 3.34466958 | -5.52730370 |
| 1 | 3.25535870 | 4.22688150 | -3.51523328 |
| 1 | -1.64191651 | 1.61477911 | 6.19349575 |
| 1 | -0.26767451 | 2.72904634 | 6.30732107 |
| 1 | -1.82725096 | 3.29094720 | 5.64632177 |
| 1 | 0.62720132 | 0.39798969 | 3.51447272 |
| 1 | 1.10853624 | 1.06194043 | 5.09153223 |
| 1 | -0.27962953 | -0.04606926 | 4.98589754 |
| 1 | -2.69292068 | 2.39930463 | 3.47885275 |
| 1 | -1.77620161 | 1.21851170 | 2.51726174 |
| 1 | -2.48937273 | 0.73860180 | 4.08158970 |
| 1 | -3.14264488 | 1.21172488 | -5.14656544 |
| 1 | -1.66681814 | 1.62310040 | -6.03899097 |
| 1 | -2.51900005 | 2.87046289 | -5.08908319 |
| 1 | -1.81955397 | 1.38663435 | -1.69970715 |
| 1 | -2.57631373 | 2.74405932 | -2.56819129 |
| 1 | -3.25340867 | 1.10644758 | -2.72166753 |
| 1 | -0.52106220 | -0.29546380 | -4.89590693 |

| | | | |
|---|---|---|---|
| 1 | -0.52756864 | -0.45477337 | -3.12034774 |
| 1 | -2.03816175 | -0.67392105 | -4.04625750 |
| 1 | 4.25422287 | 1.59089375 | 0.61520022 |
| 8 | 2.78390002 | -0.28746158 | 0.18075800 |
| 31 | 1.14293206 | 0.90477782 | 0.06417483 |
| 1 | 1.10963464 | 6.31761742 | 3.92145753 |
| 1 | 1.66799378 | 4.63297701 | -5.40329313 |
| 8 | -3.81050634 | -6.22339439 | -2.25260258 |
| 6 | -3.83322048 | -7.66680670 | -2.13065958 |
| 1 | -2.83538628 | -8.08317375 | -2.31747961 |
| 6 | -2.95683074 | -4.09645557 | -1.62533736 |
| 6 | -2.61103392 | -3.29285502 | -0.36866072 |
| 6 | -2.35490155 | -1.81236434 | -0.66751212 |
| 6 | -2.96839118 | -5.59356070 | -1.38395536 |
| 6 | -1.98173225 | -0.98683602 | 0.57033759 |
| 6 | -0.60585356 | -1.29790556 | 1.16971195 |
| 1 | -2.19770169 | -3.91298151 | -2.40624690 |
| 1 | -1.73113549 | -3.75492573 | 0.10142174 |
| 1 | -1.55370104 | -1.71713543 | -1.41689622 |
| 1 | -2.73051429 | -1.14991891 | 1.36529160 |
| 1 | -3.91917706 | -3.78952050 | -2.05830240 |
| 1 | -3.43141365 | -3.38835454 | 0.36182195 |
| 1 | -3.25473952 | -1.37427092 | -1.12959409 |
| 1 | -2.02011395 | 0.08697767 | 0.32966146 |
| 1 | -0.52374405 | -2.38315272 | 1.34670842 |
| 1 | -0.51695871 | -0.79845113 | 2.14681697 |
| 8 | -2.30363274 | -6.18252563 | -0.55071980 |
| 8 | 0.47237477 | -0.92028779 | 0.31142569 |
| 1 | -4.54508877 | -8.00950623 | -2.88757539 |
| 1 | -4.16094494 | -7.96085739 | -1.12584615 |
| 8 | 2.52781105 | -1.75228524 | 1.84124458 |
| 6 | 2.42647839 | -1.46215022 | 0.53044444 |
| 6 | 2.61701179 | -2.60916567 | -0.47963917 |
| 8 | 3.61778474 | -3.55896926 | 0.02130478 |
| 6 | 3.55859566 | -3.96851230 | 1.31336689 |
| 6 | 2.75266027 | -3.12418127 | 2.29555082 |
| 8 | 4.17289400 | -4.94334650 | 1.68560660 |
| 6 | 1.48549533 | -3.82187390 | 2.78162241 |
| 6 | 1.39350104 | -3.36101818 | -0.97665012 |
| 1 | 3.12403941 | -2.14293528 | -1.33281684 |
| 1 | 3.41717148 | -2.98100805 | 3.15881228 |
| 1 | 0.97071010 | -3.18373895 | 3.51022911 |
| 1 | 1.77619255 | -4.75925255 | 3.27307248 |
| 1 | 0.79514569 | -4.06119442 | 1.96488225 |
| 1 | 0.72986102 | -2.66653132 | -1.50223410 |
| 1 | 0.83023059 | -3.83283877 | -0.16348305 |
| 1 | 1.72756684 | -4.14513636 | -1.66868520 |

Electronic energy = -4057.822115 a.u.
DFT-D3(BJ) dispersion correction = -0.143990 a.u.
Thermal free energy = 0.724337 a.u.
Gibbs free energy = -4057.241769 a.u.
Number of imaginary frequencies = 1.

**TS3_eq LA isomer/conformer 12**

| Atomic N. | X | Y | Z |
|---|---|---|---|
| 7 | -2.07504916 | 1.34612274 | 1.85450017 |
| 8 | -1.81388795 | -1.46744502 | 0.75851935 |
| 8 | -0.89449036 | 0.81923133 | -0.84023714 |
| 6 | -1.70747650 | 1.74017978 | 3.23744845 |
| 6 | -2.65617394 | -1.79175329 | 1.77378201 |
| 6 | -2.85553193 | -3.15289044 | 2.14040613 |
| 6 | -3.70589995 | -3.40478778 | 3.22779346 |
| 6 | -4.36618376 | -2.39099789 | 3.92691660 |
| 6 | -4.22068644 | -1.07178092 | 3.50992417 |
| 6 | -3.38970304 | -0.76641119 | 2.42728353 |
| 6 | -3.40365815 | 0.62305707 | 1.84757447 |
| 6 | -2.19949889 | -4.30912399 | 1.35940659 |
| 6 | -1.97440171 | 1.49525619 | -1.28309178 |
| 6 | -2.41750479 | 1.36069679 | -2.63404489 |
| 6 | -3.53284049 | 2.11505747 | -3.02392149 |
| 6 | -4.21418858 | 2.97270727 | -2.15481782 |
| 6 | -3.77287722 | 3.09912181 | -0.84298903 |
| 6 | -2.65583801 | 2.37819242 | -0.40476355 |
| 6 | -2.14443684 | 2.59391260 | 0.99324715 |
| 6 | -1.70570672 | 0.42458850 | -3.63016224 |
| 6 | -2.64190030 | -5.68649912 | 1.88875985 |
| 6 | -0.66078472 | -4.24211359 | 1.48014784 |
| 6 | -2.61422229 | -4.23467445 | -0.12978584 |
| 6 | -2.35580492 | 0.46191344 | -5.02604723 |
| 6 | -1.77519238 | -1.03687632 | -3.12899518 |
| 6 | -0.23018336 | 0.85645086 | -3.79978132 |
| 1 | -2.45982599 | 2.44434738 | 3.62747717 |
| 1 | -1.65791082 | 0.85138977 | 3.87138891 |
| 1 | -4.13150883 | 1.25281954 | 2.38528609 |
| 1 | -3.70901990 | 0.58295310 | 0.79221123 |
| 1 | -3.86286640 | -4.43360662 | 3.54668498 |
| 1 | -4.77344370 | -0.26954773 | 4.00438881 |
| 1 | -1.11936760 | 2.99869132 | 0.98307860 |
| 1 | -2.79012489 | 3.32091665 | 1.51220262 |
| 1 | -3.89535141 | 2.02913761 | -4.04665470 |
| 1 | -4.28561258 | 3.76740813 | -0.14736401 |
| 1 | -2.16530037 | -6.47095585 | 1.28363812 |
| 1 | -2.33924770 | -5.84725809 | 2.93369365 |
| 1 | -3.72989106 | -5.82733297 | 1.81589520 |
| 1 | -0.26502550 | -3.29138923 | 1.10879350 |
| 1 | -0.35863474 | -4.35988235 | 2.53140712 |
| 1 | -0.20631430 | -5.06210661 | 0.90264630 |
| 1 | -3.70611477 | -4.31751919 | -0.23175305 |
| 1 | -2.29223609 | -3.29514146 | -0.59161365 |
| 1 | -2.15938139 | -5.06982470 | -0.68402731 |
| 1 | -1.80606210 | -0.21693270 | -5.69353485 |
| 1 | -2.32099605 | 1.46591485 | -5.47326994 |
| 1 | -3.40248489 | 0.12600024 | -5.00424385 |
| 1 | -1.30122113 | -1.15305281 | -2.14885569 |
| 1 | -2.82031465 | -1.36786783 | -3.04525924 |

| | | | |
|---|---|---|---|
| 1 | -1.26645088 | -1.70078039 | -3.84457493 |
| 1 | -0.16801244 | 1.87964261 | -4.19864464 |
| 1 | 0.30423862 | 0.82244372 | -2.84454417 |
| 1 | 0.27593356 | 0.18477292 | -4.50996208 |
| 1 | -0.72571599 | 2.22908640 | 3.22688103 |
| 8 | -0.00861265 | -0.69340456 | 2.96641231 |
| 31 | -0.77625692 | 0.07356983 | 0.89502698 |
| 1 | -5.00749254 | -2.63830543 | 4.77278519 |
| 1 | -5.08074903 | 3.53269219 | -2.50575328 |
| 8 | 5.87448215 | 4.15377378 | -5.09726715 |
| 6 | 6.36600208 | 5.40491295 | -5.63543320 |
| 1 | 5.53152084 | 6.08395433 | -5.85081482 |
| 6 | 4.73145199 | 2.91108966 | -3.43300891 |
| 6 | 3.75525188 | 2.98543453 | -2.25920987 |
| 6 | 3.36915374 | 1.60047400 | -1.72951567 |
| 6 | 5.17514467 | 4.27341890 | -3.93055201 |
| 6 | 2.37211823 | 1.66937089 | -0.56663668 |
| 6 | 1.98710370 | 0.28363276 | -0.05558393 |
| 1 | 4.30028534 | 2.35684061 | -4.28106785 |
| 1 | 2.84955406 | 3.52698922 | -2.57350874 |
| 1 | 2.93481231 | 1.00232387 | -2.54887009 |
| 1 | 1.45701683 | 2.19003010 | -0.88825464 |
| 1 | 5.64181757 | 2.35535192 | -3.15009832 |
| 1 | 4.20343399 | 3.58619857 | -1.45342398 |
| 1 | 4.27891397 | 1.06486249 | -1.40548694 |
| 1 | 2.80642748 | 2.25713348 | 0.25851166 |
| 1 | 2.87852740 | -0.27627462 | 0.27360603 |
| 1 | 1.52328813 | -0.29884228 | -0.86776096 |
| 8 | 4.96293211 | 5.33852673 | -3.38257098 |
| 8 | 1.09898126 | 0.34176859 | 1.08106136 |
| 1 | 6.89505720 | 5.13641453 | -6.55483580 |
| 1 | 7.04563713 | 5.88900805 | -4.92276812 |
| 8 | 1.91705561 | -1.76030111 | 2.28378701 |
| 6 | 1.22463977 | -0.67402345 | 2.81025457 |
| 6 | 2.13593268 | 0.17666721 | 3.69976974 |
| 8 | 2.56568551 | -0.68648219 | 4.81618023 |
| 6 | 2.79878902 | -2.01698947 | 4.62636614 |
| 6 | 2.32830620 | -2.68576765 | 3.32772779 |
| 8 | 3.29619145 | -2.66653085 | 5.52014971 |
| 6 | 3.38879490 | -3.60579562 | 2.74459672 |
| 6 | 3.36864614 | 0.79216564 | 3.06448364 |
| 1 | 1.52053678 | 0.94172239 | 4.18976831 |
| 1 | 1.44253206 | -3.27899218 | 3.62066913 |
| 1 | 2.97482133 | -4.13849497 | 1.87972450 |
| 1 | 3.69812298 | -4.33205080 | 3.50468349 |
| 1 | 4.26703024 | -3.03070807 | 2.42288542 |
| 1 | 3.07959294 | 1.59518206 | 2.37685490 |
| 1 | 3.94830990 | 0.04062398 | 2.51327276 |
| 1 | 4.00159597 | 1.21536982 | 3.85540056 |

Electronic energy = -4057.815540 a.u.
DFT-D3(BJ) dispersion correction = -0.140521 a.u.
Thermal free energy = 0.717156 a.u.
Gibbs free energy = -4057.238904 a.u.

Number of imaginary frequencies = 1.

**Int3a_LA isomer/conformer 1**

| Atomic N. | X | Y | Z |
|---|---|---|---|
| 7 | 3.31233113 | 1.99189784 | -0.25524222 |
| 8 | 0.68908464 | 1.57270057 | 1.11052796 |
| 8 | 1.22069253 | 0.38885729 | -1.70575664 |
| 6 | 4.49727317 | 1.80758530 | 0.62027913 |
| 6 | 1.08918810 | 2.64480962 | 1.84249773 |
| 6 | 0.44034311 | 2.93124158 | 3.08035524 |
| 6 | 0.90485118 | 4.03423528 | 3.81109242 |
| 6 | 1.94782419 | 4.85237457 | 3.36924405 |
| 6 | 2.54119397 | 4.58914588 | 2.13988430 |
| 6 | 2.11157020 | 3.50606858 | 1.36451060 |
| 6 | 2.66984653 | 3.33022749 | -0.02283862 |
| 6 | -0.74331597 | 2.08472146 | 3.58985016 |
| 6 | 1.43599477 | 1.30717713 | -2.67840805 |
| 6 | 0.46101404 | 1.49020764 | -3.70537400 |
| 6 | 0.73486810 | 2.44393911 | -4.69511529 |
| 6 | 1.90507925 | 3.20771670 | -4.70954019 |
| 6 | 2.85121305 | 3.01485830 | -3.71022414 |
| 6 | 2.63555089 | 2.06565795 | -2.70365154 |
| 6 | 3.72729532 | 1.81879507 | -1.69577165 |
| 6 | -0.84447929 | 0.67175947 | -3.73406750 |
| 6 | -1.29690332 | 2.61120017 | 4.92733314 |
| 6 | -0.30116001 | 0.62132124 | 3.81857788 |
| 6 | -1.90013952 | 2.13254210 | 2.56291580 |
| 6 | -1.72918285 | 1.03090121 | -4.94279647 |
| 6 | -1.67441836 | 0.95519838 | -2.46023200 |
| 6 | -0.51632611 | -0.83646051 | -3.83939218 |
| 1 | 5.26078380 | 2.56438913 | 0.37693624 |
| 1 | 4.19977043 | 1.92147963 | 1.66816494 |
| 1 | 3.41294921 | 4.11804116 | -0.23303287 |
| 1 | 1.87153032 | 3.41808556 | -0.77498034 |
| 1 | 0.43752680 | 4.26824478 | 4.76575067 |
| 1 | 3.33434555 | 5.23582192 | 1.75775558 |
| 1 | 4.10616932 | 0.78635663 | -1.76837772 |
| 1 | 4.57021842 | 2.50166255 | -1.89422044 |
| 1 | 0.00586796 | 2.60606396 | -5.48689875 |
| 1 | 3.77821656 | 3.59256938 | -3.70705658 |
| 1 | -2.14478229 | 1.98234023 | 5.23401544 |
| 1 | -0.54776865 | 2.57144348 | 5.73145248 |
| 1 | -1.66522044 | 3.64380918 | 4.84484882 |
| 1 | 0.04980353 | 0.16247358 | 2.88835603 |
| 1 | 0.50692589 | 0.57289384 | 4.56356045 |
| 1 | -1.14901225 | 0.03203709 | 4.20074462 |
| 1 | -2.25884997 | 3.16405452 | 2.43437311 |
| 1 | -1.58636414 | 1.75034431 | 1.58534164 |
| 1 | -2.74428606 | 1.52457808 | 2.92244583 |
| 1 | -2.63891916 | 0.41409216 | -4.91470399 |
| 1 | -1.22457020 | 0.83176287 | -5.89913167 |
| 1 | -2.04469846 | 2.08402298 | -4.92745002 |

| | | | |
|---|---|---|---|
| 1 | -1.11512437 | 0.70906807 | -1.55180568 |
| 1 | -1.95828629 | 2.01632451 | -2.41165138 |
| 1 | -2.60025395 | 0.35935996 | -2.47631770 |
| 1 | 0.00829024 | -1.05124625 | -4.78175608 |
| 1 | 0.11764748 | -1.16814212 | -3.00997258 |
| 1 | -1.44813205 | -1.42315251 | -3.83203061 |
| 1 | 4.90357595 | 0.80204716 | 0.46991760 |
| 8 | 2.95234625 | -0.78556889 | 0.69788712 |
| 31 | 1.84168897 | 0.57836176 | 0.04127403 |
| 1 | 2.27920710 | 5.69405719 | 3.97721812 |
| 1 | 2.07048080 | 3.94282370 | -5.49689989 |
| 8 | -7.53802801 | -3.12618945 | -0.56194404 |
| 6 | -8.89073700 | -2.80797075 | -0.15418556 |
| 1 | -9.07335775 | -3.15177738 | 0.87173024 |
| 6 | -5.18929994 | -2.97184555 | -0.27834436 |
| 6 | -4.05505882 | -2.18031628 | 0.37224743 |
| 6 | -2.67212829 | -2.66528037 | -0.07514359 |
| 6 | -6.56482354 | -2.55343573 | 0.20545823 |
| 6 | -1.53390941 | -1.85583878 | 0.55691858 |
| 6 | -0.16755334 | -2.36592971 | 0.12299030 |
| 1 | -5.08469230 | -4.04986750 | -0.06692514 |
| 1 | -4.14378330 | -2.25299667 | 1.46647845 |
| 1 | -2.55837953 | -3.73283074 | 0.18403257 |
| 1 | -1.60565712 | -1.89580695 | 1.65518601 |
| 1 | -5.16624400 | -2.88332097 | -1.37556518 |
| 1 | -4.16972985 | -1.11240712 | 0.12973435 |
| 1 | -2.59685254 | -2.60500375 | -1.17442548 |
| 1 | -1.62828550 | -0.79695197 | 0.27194214 |
| 1 | -0.06918850 | -2.37430603 | -0.97206818 |
| 1 | -0.01127584 | -3.39284118 | 0.49376944 |
| 8 | -6.79788773 | -1.82246750 | 1.14916091 |
| 8 | 0.84729471 | -1.49525367 | 0.68513299 |
| 1 | -9.53767904 | -3.33595372 | -0.86124509 |
| 1 | -9.06140795 | -1.72532157 | -0.20390922 |
| 8 | 2.48607636 | -2.64123314 | -0.56310817 |
| 6 | 2.24541905 | -1.94674798 | 0.65739761 |
| 6 | 2.51549112 | -2.86696788 | 1.87045204 |
| 8 | 3.81982000 | -3.50297057 | 1.71454566 |
| 6 | 4.03236105 | -4.17184918 | 0.54459234 |
| 6 | 2.89891295 | -4.02311415 | -0.47460462 |
| 8 | 5.04583972 | -4.81242434 | 0.37651527 |
| 6 | 3.30752606 | -4.47063840 | -1.86510517 |
| 6 | 2.53156144 | -2.14304830 | 3.20031087 |
| 1 | 1.74938424 | -3.66152878 | 1.88143274 |
| 1 | 2.05397919 | -4.64885079 | -0.12077662 |
| 1 | 2.46485698 | -4.35123761 | -2.55753968 |
| 1 | 3.61189680 | -5.52359755 | -1.84209591 |
| 1 | 4.15494095 | -3.87373292 | -2.22277295 |
| 1 | 1.55579405 | -1.67908711 | 3.38826647 |
| 1 | 3.29907271 | -1.36103866 | 3.19365695 |
| 1 | 2.74536688 | -2.85719829 | 4.00539303 |

Electronic energy = -4057.848426 a.u.
DFT-D3(BJ) dispersion correction = -0.139363 a.u.

Thermal free energy = 0.718976 a.u.
Gibbs free energy = -4057.268813 a.u.
Number of imaginary frequencies = 0.

**Int3a LA isomer/conformer 2**

| Atomic N. | X | Y | Z |
|---|---|---|---|
| 7 | 2.33466004 | 1.97117954 | -1.53750514 |
| 8 | 0.06821388 | 2.38059390 | 0.38977240 |
| 8 | -0.04663813 | 0.12106681 | -1.69711420 |
| 6 | 3.72396929 | 1.91375748 | -1.01363660 |
| 6 | 0.70199906 | 3.55822718 | 0.63949625 |
| 6 | 0.35941441 | 4.31767150 | 1.79621583 |
| 6 | 1.07254760 | 5.50500751 | 2.01861440 |
| 6 | 2.06734629 | 5.96728702 | 1.15315973 |
| 6 | 2.34734198 | 5.24884669 | -0.00396740 |
| 6 | 1.66108483 | 4.06086241 | -0.27943791 |
| 6 | 1.84737375 | 3.39328698 | -1.61651678 |
| 6 | -0.76689735 | 3.87807469 | 2.75302485 |
| 6 | -0.19193228 | 0.70728951 | -2.91629798 |
| 6 | -1.45008673 | 0.66525072 | -3.58644622 |
| 6 | -1.52870969 | 1.27405835 | -4.84711746 |
| 6 | -0.43999367 | 1.90312695 | -5.45662852 |
| 6 | 0.78429911 | 1.92625404 | -4.79969511 |
| 6 | 0.92163470 | 1.32589548 | -3.54234245 |
| 6 | 2.27868963 | 1.29846720 | -2.89003818 |
| 6 | -2.67557281 | -0.03461243 | -2.96858644 |
| 6 | -1.00549324 | 4.90719083 | 3.87418149 |
| 6 | -0.40833565 | 2.53428527 | 3.42622360 |
| 6 | -2.09517722 | 3.73876889 | 1.97050089 |
| 6 | -3.91569776 | 0.05834927 | -3.87768606 |
| 6 | -3.05075628 | 0.61974956 | -1.61812897 |
| 6 | -2.36148900 | -1.53616907 | -2.77134119 |
| 1 | 4.40357649 | 2.42624685 | -1.71342859 |
| 1 | 3.76630948 | 2.41062921 | -0.03870482 |
| 1 | 2.55747127 | 3.97008445 | -2.23246399 |
| 1 | 0.89016905 | 3.35277141 | -2.15694149 |
| 1 | 0.84559038 | 6.09760570 | 2.90285739 |
| 1 | 3.08662843 | 5.61597576 | -0.71944663 |
| 1 | 2.61337533 | 0.26320377 | -2.71722291 |
| 1 | 3.01631276 | 1.78820045 | -3.54695255 |
| 1 | -2.47866034 | 1.26385608 | -5.37806600 |
| 1 | 1.65225629 | 2.40229299 | -5.26120611 |
| 1 | -1.83011447 | 4.55526045 | 4.51036837 |
| 1 | -0.12339706 | 5.03279112 | 4.51874204 |
| 1 | -1.29054776 | 5.89225297 | 3.47764155 |
| 1 | -0.25364527 | 1.74153224 | 2.68663451 |
| 1 | 0.50703527 | 2.63893121 | 4.02760915 |
| 1 | -1.22203614 | 2.22877614 | 4.10241677 |
| 1 | -2.37963566 | 4.70009739 | 1.51846997 |
| 1 | -2.01947831 | 2.98906291 | 1.17550101 |
| 1 | -2.90031835 | 3.43869178 | 2.65846138 |
| 1 | -4.75244695 | -0.46361545 | -3.39196747 |

| | | | |
|---|---|---|---|
| 1 | -3.75184481 | -0.41969708 | -4.85412468 |
| 1 | -4.22887093 | 1.09888165 | -4.04626576 |
| 1 | -2.23831059 | 0.54528620 | -0.88844879 |
| 1 | -3.29344523 | 1.68313698 | -1.75730684 |
| 1 | -3.93919087 | 0.12248354 | -1.19990385 |
| 1 | -2.16906270 | -2.01913853 | -3.74053151 |
| 1 | -1.48376554 | -1.67988951 | -2.13170489 |
| 1 | -3.22262141 | -2.04075621 | -2.30717092 |
| 1 | 4.02609898 | 0.86844479 | -0.89292082 |
| 8 | 2.14119809 | -0.09324842 | 0.57331983 |
| 31 | 0.97384456 | 0.95657188 | -0.38922281 |
| 1 | 2.59993160 | 6.89186616 | 1.37531965 |
| 1 | -0.55353206 | 2.36723371 | -6.43614680 |
| 8 | -2.48653318 | -6.85693882 | -0.76425834 |
| 6 | -2.05124926 | -8.23565416 | -0.84952601 |
| 1 | -1.08447077 | -8.30123168 | -1.36431597 |
| 6 | -2.13197143 | -4.60219483 | -0.09647474 |
| 6 | -1.81739492 | -3.85516049 | 1.20458586 |
| 6 | -2.10312581 | -2.35340600 | 1.10060492 |
| 6 | -1.63865252 | -6.03474677 | -0.08164061 |
| 6 | -1.80819500 | -1.57637988 | 2.39122280 |
| 6 | -0.33055197 | -1.51796366 | 2.76229014 |
| 1 | -1.61466679 | -4.10695207 | -0.93636770 |
| 1 | -0.75977646 | -4.02939779 | 1.44590148 |
| 1 | -1.50560953 | -1.92479311 | 0.28183802 |
| 1 | -2.34471291 | -2.03534433 | 3.23930958 |
| 1 | -3.20451393 | -4.58460191 | -0.33318926 |
| 1 | -2.40651761 | -4.29253725 | 2.02840579 |
| 1 | -3.15993404 | -2.19728259 | 0.82913660 |
| 1 | -2.18363310 | -0.54490106 | 2.30303279 |
| 1 | 0.10893936 | -2.52572159 | 2.74455685 |
| 1 | -0.20750040 | -1.09599866 | 3.77429488 |
| 8 | -0.60891139 | -6.42311271 | 0.44129675 |
| 8 | 0.37195055 | -0.66785647 | 1.82248958 |
| 1 | -2.83050790 | -8.75006242 | -1.41996349 |
| 1 | -1.95218339 | -8.66902932 | 0.15346003 |
| 8 | 1.59892425 | -2.33166337 | 0.81193501 |
| 6 | 1.69277951 | -1.04861800 | 1.44854820 |
| 6 | 2.66283909 | -1.14367409 | 2.65299696 |
| 8 | 3.97346021 | -1.54183515 | 2.15172941 |
| 6 | 4.00259731 | -2.62579303 | 1.32253546 |
| 6 | 2.64131980 | -3.29263027 | 1.09855036 |
| 8 | 5.05400994 | -3.01258555 | 0.86212097 |
| 6 | 2.66421086 | -4.31235463 | -0.02414277 |
| 6 | 2.85322478 | 0.15817665 | 3.40365843 |
| 1 | 2.30363285 | -1.93067793 | 3.33781666 |
| 1 | 2.39902880 | -3.80939171 | 2.05154359 |
| 1 | 1.68950981 | -4.81195673 | -0.09228798 |
| 1 | 3.43647865 | -5.06487002 | 0.17482236 |
| 1 | 2.89456633 | -3.82126797 | -0.97749815 |
| 1 | 1.90487601 | 0.48303023 | 3.84897845 |
| 1 | 3.20724331 | 0.93972349 | 2.72164803 |
| 1 | 3.59035260 | 0.01806675 | 4.20445882 |

Electronic energy = -4057.844842 a.u.
DFT-D3(BJ) dispersion correction = -0.141640 a.u.
Thermal free energy = 0.719033 a.u.
Gibbs free energy = -4057.267449 a.u.
Number of imaginary frequencies = 0.

**Int3a LA isomer/conformer 3**

| Atomic N. | X | Y | Z |
|---|---|---|---|
| 7 | 3.27928851 | 2.33806456 | -0.11711870 |
| 8 | 0.76794479 | 1.62537823 | 1.32632523 |
| 8 | 1.11157464 | 1.15382559 | -1.77846678 |
| 6 | 4.48652100 | 1.96401508 | 0.65992581 |
| 6 | 1.21685609 | 2.54651609 | 2.21670583 |
| 6 | 0.67158084 | 2.57486631 | 3.53628781 |
| 6 | 1.17340323 | 3.53823279 | 4.42272840 |
| 6 | 2.16483942 | 4.45560924 | 4.06606824 |
| 6 | 2.66856939 | 4.43604054 | 2.77095861 |
| 6 | 2.19454692 | 3.50433488 | 1.84004452 |
| 6 | 2.66266310 | 3.60162742 | 0.41331474 |
| 6 | -0.42733717 | 1.59067194 | 3.98335279 |
| 6 | 1.31076857 | 2.25540536 | -2.53685378 |
| 6 | 0.29436767 | 2.67903744 | -3.44495151 |
| 6 | 0.55806992 | 3.81509197 | -4.22232964 |
| 6 | 1.75503046 | 4.53243018 | -4.13617200 |
| 6 | 2.73857841 | 4.10770016 | -3.25067226 |
| 6 | 2.53300936 | 2.97105720 | -2.45950486 |
| 6 | 3.64808027 | 2.48515225 | -1.57210699 |
| 6 | -1.04121659 | 1.91961307 | -3.57165902 |
| 6 | -0.90350059 | 1.86617869 | 5.42249956 |
| 6 | 0.12111519 | 0.14580441 | 3.95076627 |
| 6 | -1.66634892 | 1.72115704 | 3.06562423 |
| 6 | -1.96768988 | 2.55435288 | -4.62588015 |
| 6 | -1.79771717 | 1.94374360 | -2.22214987 |
| 6 | -0.78153124 | 0.45804587 | -4.00806558 |
| 1 | 5.24899197 | 2.75541038 | 0.57126161 |
| 1 | 4.22014708 | 1.83812411 | 1.71466159 |
| 1 | 3.39772341 | 4.41857111 | 0.31467486 |
| 1 | 1.82167986 | 3.83334227 | -0.25732384 |
| 1 | 0.77976054 | 3.57594721 | 5.43665973 |
| 1 | 3.42522711 | 5.15991382 | 2.45988059 |
| 1 | 4.00087982 | 1.48981992 | -1.88818663 |
| 1 | 4.50135575 | 3.18095253 | -1.64100021 |
| 1 | -0.19983208 | 4.16316822 | -4.92169871 |
| 1 | 3.68385744 | 4.64941692 | -3.17132600 |
| 1 | -1.69522499 | 1.14763266 | 5.67870322 |
| 1 | -0.09657501 | 1.74203829 | 6.15897197 |
| 1 | -1.32451254 | 2.87590737 | 5.53313685 |
| 1 | 0.48615238 | -0.13079539 | 2.95723297 |
| 1 | 0.95258572 | 0.03531659 | 4.66230107 |
| 1 | -0.66952520 | -0.56279304 | 4.24296811 |
| 1 | -2.09370187 | 2.73196431 | 3.13790815 |
| 1 | -1.41618486 | 1.52945332 | 2.01677348 |

| | | | |
|---|---|---|---|
| 1 | -2.44070156 | 1.00542134 | 3.38176470 |
| 1 | -2.89858278 | 1.97217237 | -4.67955066 |
| 1 | -1.51724900 | 2.54981065 | -5.62891691 |
| 1 | -2.23953986 | 3.58874697 | -4.37039904 |
| 1 | -1.21685438 | 1.46794758 | -1.42478657 |
| 1 | -2.01873793 | 2.97762080 | -1.91972209 |
| 1 | -2.75482607 | 1.40943499 | -2.32314954 |
| 1 | -0.28515138 | 0.42957283 | -4.98904649 |
| 1 | -0.15166685 | -0.06939626 | -3.28363492 |
| 1 | -1.73814487 | -0.07883493 | -4.09975916 |
| 1 | 4.88245638 | 1.01777646 | 0.27647689 |
| 8 | 2.90656643 | -0.61187466 | 0.16058978 |
| 31 | 1.78566363 | 0.89888253 | -0.05831429 |
| 1 | 2.52845180 | 5.18122712 | 4.79350571 |
| 1 | 1.91079361 | 5.41426912 | -4.75737043 |
| 8 | -6.82806598 | -5.24692328 | -0.89882558 |
| 6 | -7.73026519 | -5.92376338 | -1.80655366 |
| 1 | -8.09941158 | -5.22844474 | -2.57104099 |
| 6 | -4.85214614 | -4.01027812 | -0.46764185 |
| 6 | -3.49768581 | -3.56887410 | -1.02126162 |
| 6 | -2.66597322 | -2.80415737 | 0.01388536 |
| 6 | -5.71775200 | -4.71759772 | -1.49255286 |
| 6 | -1.29624919 | -2.37707716 | -0.53025851 |
| 6 | -0.50943694 | -1.60007330 | 0.51726993 |
| 1 | -5.42572941 | -3.14314559 | -0.09828386 |
| 1 | -3.65605294 | -2.94266478 | -1.91207339 |
| 1 | -3.22535505 | -1.91244132 | 0.34686327 |
| 1 | -1.43054622 | -1.74603594 | -1.42303815 |
| 1 | -4.73417019 | -4.67639731 | 0.40129670 |
| 1 | -2.93964654 | -4.45263093 | -1.36697286 |
| 1 | -2.52431339 | -3.43348814 | 0.90945795 |
| 1 | -0.73075823 | -3.26933676 | -0.83871789 |
| 1 | -0.29152092 | -2.20883319 | 1.40452768 |
| 1 | -1.07058781 | -0.71507282 | 0.84874338 |
| 8 | -5.48713316 | -4.80833111 | -2.68331514 |
| 8 | 0.74783578 | -1.07257801 | -0.00232635 |
| 1 | -8.55247890 | -6.28510881 | -1.18145897 |
| 1 | -7.22076040 | -6.76030065 | -2.30101932 |
| 8 | 1.98716570 | -2.10409628 | 1.68029563 |
| 6 | 2.05473921 | -1.66411085 | 0.33291681 |
| 6 | 2.46024917 | -2.82577495 | -0.60297919 |
| 8 | 1.76171543 | -4.05774318 | -0.24491995 |
| 6 | 1.86986909 | -4.44657090 | 1.06100969 |
| 6 | 2.54732714 | -3.40814011 | 1.96010529 |
| 8 | 1.42291400 | -5.51130420 | 1.42509731 |
| 6 | 2.34638537 | -3.69391184 | 3.43547113 |
| 6 | 2.21132815 | -2.56675598 | -2.07536884 |
| 1 | 3.53865908 | -2.98107363 | -0.43333222 |
| 1 | 3.63039799 | -3.40261142 | 1.72863688 |
| 1 | 2.84859842 | -2.92338632 | 4.03310453 |
| 1 | 2.76588747 | -4.67624123 | 3.68277070 |
| 1 | 1.27816533 | -3.70401853 | 3.68402915 |
| 1 | 2.72721208 | -1.64755813 | -2.37766840 |

| | | | |
|---|---|---|---|
| 1 | 1.14180352 | -2.44837003 | -2.28011185 |
| 1 | 2.59903838 | -3.40489820 | -2.66771665 |

Electronic energy = -4057.849525 a.u.
DFT-D3(BJ) dispersion correction = -0.139665 a.u.
Thermal free energy = 0.724032 a.u.
Gibbs free energy = -4057.265157 a.u.
Number of imaginary frequencies = 0.

**Int3a LA isomer/conformer 4**

| Atomic N. | X | Y | Z |
|---|---|---|---|
| 7 | 2.72024713 | 2.46337330 | -0.19384294 |
| 8 | 0.26278358 | 1.77506368 | 1.34868996 |
| 8 | 0.57897676 | 1.07219655 | -1.72727891 |
| 6 | 3.91349771 | 2.23907037 | 0.65991999 |
| 6 | 0.64839878 | 2.84590175 | 2.09317180 |
| 6 | 0.12494139 | 3.00250994 | 3.41326377 |
| 6 | 0.52867072 | 4.12987321 | 4.14202294 |
| 6 | 1.41798202 | 5.08135222 | 3.63782231 |
| 6 | 1.92410039 | 4.91724397 | 2.35462054 |
| 6 | 1.54005573 | 3.82101464 | 1.57276265 |
| 6 | 2.02991631 | 3.75298784 | 0.15060379 |
| 6 | -0.82716653 | 1.96514035 | 4.04053452 |
| 6 | 0.81601042 | 2.05487620 | -2.63045518 |
| 6 | -0.16613057 | 2.34760650 | -3.62524407 |
| 6 | 0.12672876 | 3.36372954 | -4.54505817 |
| 6 | 1.32132882 | 4.08815803 | -4.52141136 |
| 6 | 2.27246672 | 3.79081056 | -3.55342098 |
| 6 | 2.03830117 | 2.77540315 | -2.61805406 |
| 6 | 3.13271554 | 2.42993680 | -1.64360387 |
| 6 | -1.49800500 | 1.57647889 | -3.69952340 |
| 6 | -1.31221785 | 2.39064497 | 5.43979692 |
| 6 | -0.07464897 | 0.62411189 | 4.20628631 |
| 6 | -2.08951627 | 1.77997445 | 3.16577262 |
| 6 | -2.38255468 | 2.05562556 | -4.86610651 |
| 6 | -2.30507337 | 1.78769265 | -2.39742338 |
| 6 | -1.21967636 | 0.07159390 | -3.92432375 |
| 1 | 4.64788410 | 3.04413759 | 0.49499236 |
| 1 | 3.61672376 | 2.23521529 | 1.71403839 |
| 1 | 2.72421832 | 4.58736511 | -0.04504497 |
| 1 | 1.19463580 | 3.85013508 | -0.55940672 |
| 1 | 0.14137069 | 4.27154908 | 5.14903506 |
| 1 | 2.61446590 | 5.65181834 | 1.93409758 |
| 1 | 3.51512632 | 1.41075862 | -1.81755316 |
| 1 | 3.97424312 | 3.13006112 | -1.77702749 |
| 1 | -0.60665876 | 3.60760470 | -5.31124326 |
| 1 | 3.21734694 | 4.33769578 | -3.51934626 |
| 1 | -1.99228648 | 1.61985808 | 5.82974905 |
| 1 | -0.48457983 | 2.49047795 | 6.15645998 |
| 1 | -1.86744245 | 3.33935120 | 5.41341259 |
| 1 | 0.33316186 | 0.26422274 | 3.25549186 |
| 1 | 0.76003272 | 0.73984630 | 4.91334705 |
| 1 | -0.75292193 | -0.14398195 | 4.60911132 |

| | | | |
|---|---|---|---|
| 1 | -2.64607400 | 2.72558445 | 3.09040978 |
| 1 | -1.83779529 | 1.44895020 | 2.15346704 |
| 1 | -2.75525445 | 1.03487320 | 3.62763763 |
| 1 | -3.31237080 | 1.46903855 | -4.87170645 |
| 1 | -1.89575473 | 1.91176612 | -5.84142896 |
| 1 | -2.66172711 | 3.11461270 | -4.76822374 |
| 1 | -1.74522170 | 1.45206355 | -1.51834991 |
| 1 | -2.55247279 | 2.85089781 | -2.26444755 |
| 1 | -3.25009184 | 1.22535414 | -2.44721261 |
| 1 | -0.68827727 | -0.08271153 | -4.87480194 |
| 1 | -0.61402391 | -0.34898620 | -3.11459672 |
| 1 | -2.17055711 | -0.48040894 | -3.97654280 |
| 1 | 4.35814706 | 1.27057860 | 0.40683453 |
| 8 | 2.41060097 | -0.44122609 | 0.35785296 |
| 31 | 1.28409326 | 1.00398467 | -0.00327707 |
| 1 | 1.70856435 | 5.93827107 | 4.24523420 |
| 1 | 1.50084183 | 4.87462486 | -5.25413486 |
| 8 | -4.00395106 | -6.40044741 | -2.78106438 |
| 6 | -4.28954559 | -7.81495680 | -2.65736547 |
| 1 | -3.36812370 | -8.37657970 | -2.45848908 |
| 6 | -3.13815549 | -4.36985131 | -1.91499588 |
| 6 | -2.83743051 | -3.59437706 | -0.63186996 |
| 6 | -2.42952128 | -2.14365158 | -0.91260675 |
| 6 | -3.44598404 | -5.83484807 | -1.67132132 |
| 6 | -2.11884733 | -1.33189537 | 0.35322985 |
| 6 | -0.81246624 | -1.68249028 | 1.05055220 |
| 1 | -2.27333121 | -4.33250375 | -2.59993894 |
| 1 | -2.05150445 | -4.12589451 | -0.07527940 |
| 1 | -1.55655817 | -2.12008885 | -1.58461983 |
| 1 | -2.92591768 | -1.47666551 | 1.09136056 |
| 1 | -3.97471277 | -3.92244263 | -2.47278013 |
| 1 | -3.72681055 | -3.60932526 | 0.01861064 |
| 1 | -3.24352350 | -1.64161718 | -1.46040271 |
| 1 | -2.10815295 | -0.25658854 | 0.11988055 |
| 1 | -0.70070770 | -2.76592169 | 1.18711663 |
| 1 | -0.75725277 | -1.20352293 | 2.03524380 |
| 8 | -3.22062673 | -6.45033647 | -0.64627044 |
| 8 | 0.29562241 | -1.18945621 | 0.22829004 |
| 1 | -4.72265309 | -8.10754459 | -3.61860626 |
| 1 | -4.99974101 | -7.99244437 | -1.84015352 |
| 8 | 1.65083154 | -1.98943551 | 1.93011975 |
| 6 | 1.64842146 | -1.56324726 | 0.57491961 |
| 6 | 2.15581570 | -2.72697438 | -0.30346284 |
| 8 | 3.51121497 | -3.09265415 | 0.11918144 |
| 6 | 3.92349053 | -2.90667357 | 1.40157242 |
| 6 | 2.99094065 | -2.21965609 | 2.40857982 |
| 8 | 5.04434509 | -3.24484622 | 1.72206279 |
| 6 | 2.88689951 | -3.01783206 | 3.70216678 |
| 6 | 1.29873439 | -3.98234266 | -0.32105339 |
| 1 | 2.28857407 | -2.33769741 | -1.32241705 |
| 1 | 3.46556544 | -1.24330963 | 2.61040119 |
| 1 | 2.28861585 | -2.45693641 | 4.43126009 |
| 1 | 3.88957483 | -3.18906039 | 4.10932037 |

| | | | |
|---|---|---|---|
| 1 | 2.40524195 | -3.98796595 | 3.52167648 |
| 1 | 0.33638869 | -3.78313778 | -0.80931327 |
| 1 | 1.11708557 | -4.35924141 | 0.69397936 |
| 1 | 1.81807624 | -4.76129212 | -0.89386219 |

Electronic energy = -4057.844181 a.u.
DFT-D3(BJ) dispersion correction = -0.141583 a.u.
Thermal free energy = 0.721173 a.u.
Gibbs free energy = -4057.264590 a.u.
Number of imaginary frequencies = 0.

**Int3a LA isomer/conformer 5**

| Atomic N. | X | Y | Z |
|---|---|---|---|
| 7 | 2.20080337 | 2.96384167 | 0.61712412 |
| 8 | -0.25283213 | 1.60441269 | 1.64528515 |
| 8 | 0.85392430 | 0.96489028 | -1.18625651 |
| 6 | 3.30484717 | 3.05481732 | 1.60559579 |
| 6 | -0.27533753 | 2.66130638 | 2.49763459 |
| 6 | -1.07532683 | 2.59370122 | 3.67711618 |
| 6 | -1.05228421 | 3.70127949 | 4.53674174 |
| 6 | -0.29865924 | 4.84847710 | 4.27564537 |
| 6 | 0.44478772 | 4.91800188 | 3.10299252 |
| 6 | 0.45105331 | 3.84544804 | 2.20393373 |
| 6 | 1.14935336 | 4.00474249 | 0.87936464 |
| 6 | -1.95095460 | 1.36447579 | 3.99243758 |
| 6 | 0.84911404 | 2.00748016 | -2.05341947 |
| 6 | -0.03997306 | 1.99229967 | -3.17019478 |
| 6 | -0.00119069 | 3.08975564 | -4.04116974 |
| 6 | 0.85944903 | 4.17550430 | -3.85775052 |
| 6 | 1.72918472 | 4.17363058 | -2.77425725 |
| 6 | 1.74258541 | 3.09671624 | -1.87901198 |
| 6 | 2.76816109 | 3.08787744 | -0.77629041 |
| 6 | -1.00526335 | 0.81822476 | -3.42101456 |
| 6 | -2.74799504 | 1.54268809 | 5.29839791 |
| 6 | -1.06950513 | 0.10550332 | 4.15830233 |
| 6 | -2.97626652 | 1.14434095 | 2.85435533 |
| 6 | -1.84864339 | 1.01995797 | -4.69457817 |
| 6 | -1.99389155 | 0.68275830 | -2.23897276 |
| 6 | -0.20683051 | -0.49401691 | -3.60907324 |
| 1 | 3.80811932 | 4.03096430 | 1.51224483 |
| 1 | 2.89958988 | 2.95478214 | 2.61817617 |
| 1 | 1.61829242 | 5.00134572 | 0.81771620 |
| 1 | 0.43060936 | 3.92499657 | 0.04995307 |
| 1 | -1.64333154 | 3.67205453 | 5.45016614 |
| 1 | 1.01574641 | 5.81749823 | 2.86176966 |
| 1 | 3.46005966 | 2.23695925 | -0.88597937 |
| 1 | 3.36386297 | 4.01479366 | -0.82213896 |
| 1 | -0.67302566 | 3.10495380 | -4.89726299 |
| 1 | 2.42011016 | 5.00498578 | -2.61720933 |
| 1 | -3.36289655 | 0.64680764 | 5.46561043 |
| 1 | -2.09162355 | 1.66065203 | 6.17278707 |
| 1 | -3.42751574 | 2.40586862 | 5.25396640 |
| 1 | -0.51811788 | -0.11733797 | 3.23886125 |

| | | | |
|---|---|---|---|
| 1 | -0.34863868 | 0.24249742 | 4.97795327 |
| 1 | -1.70167421 | -0.76149716 | 4.40576422 |
| 1 | -3.64100107 | 2.01525842 | 2.76013560 |
| 1 | -2.47986406 | 0.98103492 | 1.89152544 |
| 1 | -3.60099530 | 0.26662183 | 3.08098424 |
| 1 | -2.50232515 | 0.14667164 | -4.82752335 |
| 1 | -1.22341704 | 1.10429703 | -5.59516214 |
| 1 | -2.49072608 | 1.91058927 | -4.63066041 |
| 1 | -1.47312919 | 0.48670526 | -1.29553230 |
| 1 | -2.58386730 | 1.60341689 | -2.12130453 |
| 1 | -2.69248500 | -0.14394831 | -2.43612927 |
| 1 | 0.45018298 | -0.41984039 | -4.48809833 |
| 1 | 0.41529934 | -0.71376046 | -2.73434355 |
| 1 | -0.90126135 | -1.33069814 | -3.77681917 |
| 1 | 4.01915685 | 2.24517532 | 1.42344829 |
| 8 | 2.64272681 | 0.11318109 | 1.29793045 |
| 31 | 1.23420298 | 1.14765887 | 0.62483962 |
| 1 | -0.30667282 | 5.68128468 | 4.97866223 |
| 1 | 0.84740398 | 5.00958671 | -4.55918028 |
| 8 | -3.92626721 | -5.55219289 | -3.92717660 |
| 6 | -4.28957524 | -5.46407608 | -5.32676149 |
| 1 | -4.99230963 | -4.63724711 | -5.48906089 |
| 6 | -2.99779634 | -4.61977741 | -1.95437105 |
| 6 | -2.18363861 | -3.46263362 | -1.37675160 |
| 6 | -1.92876734 | -3.61664437 | 0.12721030 |
| 6 | -3.31647026 | -4.43986235 | -3.42685415 |
| 6 | -1.17308794 | -2.43816389 | 0.76294215 |
| 6 | 0.28345959 | -2.35527649 | 0.32297106 |
| 1 | -3.95701445 | -4.73085501 | -1.42096167 |
| 1 | -2.71770052 | -2.51927531 | -1.56391264 |
| 1 | -2.89677371 | -3.72264965 | 0.64370082 |
| 1 | -1.19686671 | -2.53434715 | 1.85913355 |
| 1 | -2.47877802 | -5.58343742 | -1.83007391 |
| 1 | -1.23482923 | -3.38499599 | -1.92766262 |
| 1 | -1.37907323 | -4.55503063 | 0.32029761 |
| 1 | -1.68022241 | -1.49218397 | 0.51715397 |
| 1 | 0.38684340 | -2.19882386 | -0.75919345 |
| 1 | 0.80425197 | -3.29068317 | 0.58921663 |
| 8 | -3.08011490 | -3.44740200 | -4.09051915 |
| 8 | 0.90319357 | -1.24373689 | 1.01959222 |
| 1 | -4.75794787 | -6.42387357 | -5.56450473 |
| 1 | -3.39757371 | -5.30228993 | -5.94468439 |
| 8 | 2.89783773 | -1.64180435 | -0.15937464 |
| 6 | 2.36311211 | -1.20253767 | 1.08869369 |
| 6 | 2.86023151 | -2.10781142 | 2.23971736 |
| 8 | 4.31259676 | -2.22998275 | 2.16467985 |
| 6 | 4.82586036 | -2.63791854 | 0.96823631 |
| 6 | 3.78255160 | -2.78301351 | -0.14364871 |
| 8 | 6.01073142 | -2.85699670 | 0.84956193 |
| 6 | 4.41320458 | -2.89446766 | -1.51884764 |
| 6 | 2.53062709 | -1.58398434 | 3.62187351 |
| 1 | 2.42744736 | -3.11399487 | 2.10337306 |
| 1 | 3.20463602 | -3.70587272 | 0.07066396 |

| | | | |
|---|---|---|---|
| 1 | 3.63005233 | -3.00128454 | -2.27978542 |
| 1 | 5.07612160 | -3.76693325 | -1.55466406 |
| 1 | 5.00741775 | -1.99931714 | -1.73779333 |
| 1 | 1.44355438 | -1.51267917 | 3.74795765 |
| 1 | 2.96840033 | -0.58923340 | 3.76149875 |
| 1 | 2.93157120 | -2.26838328 | 4.38003959 |

Electronic energy = -4057.847240 a.u.
DFT-D3(BJ) dispersion correction = -0.140359 a.u.
Thermal free energy = 0.724021 a.u.
Gibbs free energy = -4057.263579 a.u.
Number of imaginary frequencies = 0.

**Int3a LA isomer/conformer 6**

| Atomic N. | X | Y | Z |
|---|---|---|---|
| 7 | 2.34867097 | 2.01953994 | -1.32483525 |
| 8 | -0.08451588 | 2.26695404 | 0.40996165 |
| 8 | 0.17190939 | -0.05042530 | -1.60817246 |
| 6 | 3.67932290 | 2.13490857 | -0.67438351 |
| 6 | 0.40866841 | 3.51248516 | 0.64361770 |
| 6 | -0.07744194 | 4.27196619 | 1.74807675 |
| 6 | 0.48253023 | 5.54183624 | 1.95100332 |
| 6 | 1.46676885 | 6.07988305 | 1.11786094 |
| 6 | 1.89741831 | 5.34840308 | 0.01662414 |
| 6 | 1.36558213 | 4.07957523 | -0.23925813 |
| 6 | 1.72334798 | 3.37467171 | -1.52094433 |
| 6 | -1.18553258 | 3.73656051 | 2.67665463 |
| 6 | 0.10537754 | 0.45075926 | -2.86888737 |
| 6 | -1.06534761 | 0.24271598 | -3.65639452 |
| 6 | -1.07003407 | 0.77033015 | -4.95529906 |
| 6 | 0.01054316 | 1.47500245 | -5.49296895 |
| 6 | 1.15101212 | 1.65987837 | -4.72074346 |
| 6 | 1.21371650 | 1.14547668 | -3.42000756 |
| 6 | 2.49359606 | 1.28785919 | -2.63919500 |
| 6 | -2.27472252 | -0.54313483 | -3.11508542 |
| 6 | -1.58863988 | 4.76721990 | 3.74814017 |
| 6 | -0.69605503 | 2.46979563 | 3.41471665 |
| 6 | -2.45500960 | 3.41200509 | 1.85308612 |
| 6 | -3.41264834 | -0.63668587 | -4.14912091 |
| 6 | -2.85396657 | 0.15232398 | -1.86060757 |
| 6 | -1.84124937 | -1.98875735 | -2.77521208 |
| 1 | 4.36774728 | 2.68497614 | -1.33609999 |
| 1 | 3.57713644 | 2.67765186 | 0.27126501 |
| 1 | 2.41598176 | 3.99504823 | -2.11406838 |
| 1 | 0.82210060 | 3.20449456 | -2.12831782 |
| 1 | 0.14113300 | 6.13926464 | 2.79427279 |
| 1 | 2.63702618 | 5.76502403 | -0.67088764 |
| 1 | 2.91062790 | 0.30124115 | -2.38088388 |
| 1 | 3.24151522 | 1.82258020 | -3.24776352 |
| 1 | -1.95333590 | 0.63322302 | -5.57620247 |
| 1 | 2.01236736 | 2.19701102 | -5.12396592 |
| 1 | -2.39112740 | 4.34138007 | 4.36723924 |
| 1 | -0.75408378 | 5.01882980 | 4.41858179 |

| | | | |
|---|---|---|---|
| 1 | -1.97146681 | 5.69742721 | 3.30412704 |
| 1 | -0.42312522 | 1.67447605 | 2.71304835 |
| 1 | 0.17911735 | 2.70303075 | 4.03932365 |
| 1 | -1.49210764 | 2.09441984 | 4.07668952 |
| 1 | -2.83404837 | 4.31652882 | 1.35538561 |
| 1 | -2.25782376 | 2.65198032 | 1.08940446 |
| 1 | -3.24563717 | 3.03975189 | 2.52246380 |
| 1 | -4.24121985 | -1.21394426 | -3.71467199 |
| 1 | -3.09739829 | -1.15326910 | -5.06717669 |
| 1 | -3.80684278 | 0.35264041 | -4.42311836 |
| 1 | -2.12126846 | 0.20363859 | -1.04887582 |
| 1 | -3.17879734 | 1.17532950 | -2.09947534 |
| 1 | -3.73224450 | -0.40535835 | -1.50110396 |
| 1 | -1.48171464 | -2.50237068 | -3.67921966 |
| 1 | -1.04204210 | -2.00269818 | -2.02576176 |
| 1 | -2.69984909 | -2.55660661 | -2.38628522 |
| 1 | 4.07399295 | 1.13425440 | -0.46968806 |
| 8 | 2.16258608 | -0.00575721 | 0.84498318 |
| 31 | 0.99987818 | 0.91006151 | -0.25285418 |
| 1 | 1.87736197 | 7.06842607 | 1.32298471 |
| 1 | -0.04391924 | 1.87149629 | -6.50667808 |
| 8 | -1.92764160 | -5.51018574 | -1.57812565 |
| 6 | -0.81882888 | -5.89260041 | -2.42881171 |
| 1 | -0.07612525 | -5.08630060 | -2.46929260 |
| 6 | -2.81436484 | -4.90833318 | 0.54364853 |
| 6 | -2.50236036 | -3.94137959 | 1.69132042 |
| 6 | -2.29065452 | -2.49233113 | 1.23240310 |
| 6 | -1.59081019 | -5.25884533 | -0.28300934 |
| 6 | -1.89690566 | -1.55951205 | 2.38823262 |
| 6 | -0.41572795 | -1.62755516 | 2.75405895 |
| 1 | -3.60068675 | -4.51725619 | -0.11734433 |
| 1 | -1.61020305 | -4.31116207 | 2.22003518 |
| 1 | -1.51212645 | -2.44758899 | 0.45492865 |
| 1 | -2.48905726 | -1.81034903 | 3.28429230 |
| 1 | -3.19053134 | -5.86338144 | 0.94791928 |
| 1 | -3.33467101 | -3.97087717 | 2.41293760 |
| 1 | -3.21931072 | -2.12785878 | 0.76479503 |
| 1 | -2.13327660 | -0.51302630 | 2.14342838 |
| 1 | -0.03172718 | -2.65264271 | 2.63279616 |
| 1 | -0.25992057 | -1.30676470 | 3.79831543 |
| 8 | -0.45324817 | -5.34463884 | 0.14422161 |
| 8 | 0.31708737 | -0.73272093 | 1.88171128 |
| 1 | -1.25809639 | -6.06378912 | -3.41609528 |
| 1 | -0.34165143 | -6.80546464 | -2.05046011 |
| 8 | 1.72611820 | -2.27711509 | 0.92552216 |
| 6 | 1.68989862 | -1.03041884 | 1.62698037 |
| 6 | 2.53954153 | -1.13570125 | 2.91695216 |
| 8 | 3.90984342 | -1.45863924 | 2.53659787 |
| 6 | 4.07128699 | -2.50881071 | 1.67910612 |
| 6 | 2.77066842 | -3.21173102 | 1.27733919 |
| 8 | 5.18180866 | -2.84018455 | 1.32628484 |
| 6 | 2.95644015 | -4.16564054 | 0.11243559 |
| 6 | 2.59660957 | 0.14063102 | 3.73115434 |

| | | | |
|---|---|---|---|
| 1 | 2.15010263 | -1.96603078 | 3.53018119 |
| 1 | 2.44820814 | -3.78859298 | 2.16994524 |
| 1 | 2.00414546 | -4.66554828 | -0.10303442 |
| 1 | 3.71462590 | -4.91658186 | 0.36502683 |
| 1 | 3.29478125 | -3.61638299 | -0.77489612 |
| 1 | 1.59570824 | 0.40974696 | 4.09084093 |
| 1 | 2.98060371 | 0.96251019 | 3.11618296 |
| 1 | 3.25662690 | -0.00227499 | 4.59617833 |

Electronic energy = -4057.844797 a.u.
DFT-D3(BJ) dispersion correction = -0.142466 a.u.
Thermal free energy = 0.723799 a.u.
Gibbs free energy = -4057.263464 a.u.
Number of imaginary frequencies = 0.

**Int3a LA isomer/conformer 7**

| Atomic N. | X | Y | Z |
|---|---|---|---|
| 7 | -1.27986501 | 2.64500684 | 2.39716407 |
| 8 | -1.64098408 | 0.65851900 | 0.20145268 |
| 8 | 1.20565647 | 1.75572773 | 1.02885264 |
| 6 | -2.21669807 | 2.32801271 | 3.50382115 |
| 6 | -2.86408233 | 1.24461987 | 0.11813370 |
| 6 | -3.92474148 | 0.55256611 | -0.54250859 |
| 6 | -5.16669240 | 1.19699827 | -0.62686492 |
| 6 | -5.40190123 | 2.46382772 | -0.08775729 |
| 6 | -4.36398112 | 3.12656388 | 0.55560727 |
| 6 | -3.09669556 | 2.53942148 | 0.65320856 |
| 6 | -1.97986461 | 3.34362360 | 1.26540945 |
| 6 | -3.73318497 | -0.86198654 | -1.12480464 |
| 6 | 1.48095392 | 3.08122802 | 1.00865589 |
| 6 | 2.46095389 | 3.57777038 | 0.09603676 |
| 6 | 2.73062926 | 4.95270618 | 0.12296501 |
| 6 | 2.07922891 | 5.83698292 | 0.98738955 |
| 6 | 1.12553797 | 5.34288125 | 1.86869361 |
| 6 | 0.82944658 | 3.97440271 | 1.89755435 |
| 6 | -0.13830529 | 3.47047258 | 2.93552262 |
| 6 | 3.18937711 | 2.64683257 | -0.89311957 |
| 6 | -4.99845221 | -1.37308333 | -1.84020064 |
| 6 | -3.42803765 | -1.84982562 | 0.02565335 |
| 6 | -2.59120763 | -0.87191105 | -2.16842103 |
| 6 | 4.23001416 | 3.40248782 | -1.74101472 |
| 6 | 2.16411501 | 2.02682935 | -1.87151638 |
| 6 | 3.94605427 | 1.53356635 | -0.12975203 |
| 1 | -2.63392310 | 3.25964252 | 3.91947706 |
| 1 | -3.03515105 | 1.70566936 | 3.12699428 |
| 1 | -2.37219312 | 4.30621251 | 1.63419147 |
| 1 | -1.20222747 | 3.57146858 | 0.52065640 |
| 1 | -5.98997632 | 0.69045908 | -1.12676819 |
| 1 | -4.52093734 | 4.12156507 | 0.97797744 |
| 1 | 0.36828837 | 2.82445440 | 3.67147028 |
| 1 | -0.56448823 | 4.32808955 | 3.48245275 |
| 1 | 3.47392980 | 5.35739032 | -0.56142109 |
| 1 | 0.60722359 | 6.01430787 | 2.55694751 |

| | | | |
|---|---|---|---|
| 1 | -4.80000815 | -2.37805436 | -2.23948208 |
| 1 | -5.85797872 | -1.45437740 | -1.15965919 |
| 1 | -5.28278722 | -0.73082773 | -2.68615868 |
| 1 | -2.54894169 | -1.54479850 | 0.60337011 |
| 1 | -4.28460763 | -1.91142288 | 0.71298057 |
| 1 | -3.24757581 | -2.85812211 | -0.37817615 |
| 1 | -2.83092895 | -0.20240157 | -3.00730255 |
| 1 | -1.64112856 | -0.54776989 | -1.73184484 |
| 1 | -2.46855823 | -1.88806251 | -2.57419308 |
| 1 | 4.71834541 | 2.69115516 | -2.42200179 |
| 1 | 5.01464101 | 3.86016303 | -1.12121749 |
| 1 | 3.77189145 | 4.18751160 | -2.35962142 |
| 1 | 1.38640644 | 1.46949895 | -1.33859066 |
| 1 | 1.67876796 | 2.81207810 | -2.46912315 |
| 1 | 2.67329589 | 1.34069817 | -2.56538496 |
| 1 | 4.70369610 | 1.97090868 | 0.53702947 |
| 1 | 3.26531642 | 0.92190710 | 0.47153526 |
| 1 | 4.46617721 | 0.88113808 | -0.84770175 |
| 1 | -1.67723240 | 1.78036683 | 4.28408412 |
| 8 | -0.35700134 | -0.10495142 | 3.08048789 |
| 31 | -0.40420436 | 0.98410817 | 1.55482657 |
| 1 | -6.38692899 | 2.92243283 | -0.17220589 |
| 1 | 2.31780556 | 6.90010830 | 0.96619458 |
| 8 | 2.55145212 | -4.14570638 | -6.50079974 |
| 6 | 3.27409590 | -3.90321430 | -7.73193772 |
| 1 | 2.98631377 | -2.93542501 | -8.16133552 |
| 6 | 1.98794065 | -3.57281395 | -4.27110171 |
| 6 | 2.05446200 | -2.49239192 | -3.19247258 |
| 6 | 1.29592264 | -2.88805732 | -1.92115143 |
| 6 | 2.76775499 | -3.21787933 | -5.52284096 |
| 6 | 1.34715010 | -1.79502991 | -0.84689617 |
| 6 | 0.58707537 | -2.20128643 | 0.40424788 |
| 1 | 0.94747877 | -3.78448114 | -4.56403808 |
| 1 | 1.64327041 | -1.55279829 | -3.59246633 |
| 1 | 0.24401162 | -3.10636360 | -2.17321956 |
| 1 | 0.91520544 | -0.86503494 | -1.24641690 |
| 1 | 2.38804579 | -4.52963973 | -3.89518671 |
| 1 | 3.10775963 | -2.28338566 | -2.95206499 |
| 1 | 1.71863162 | -3.82504806 | -1.51818825 |
| 1 | 2.39326769 | -1.57241116 | -0.58399151 |
| 1 | 1.02243678 | -3.10296476 | 0.85542495 |
| 1 | -0.47024857 | -2.39934278 | 0.18424864 |
| 8 | 3.49688066 | -2.25431309 | -5.66148787 |
| 8 | 0.67564748 | -1.10087033 | 1.36161673 |
| 1 | 2.99207051 | -4.72321271 | -8.39923161 |
| 1 | 4.35598179 | -3.90523861 | -7.54868013 |
| 8 | -0.68011090 | -2.39171674 | 2.72768615 |
| 6 | 0.22312191 | -1.29582400 | 2.72229100 |
| 6 | 1.40264464 | -1.62729539 | 3.66110450 |
| 8 | 0.88992187 | -1.88047319 | 5.01092717 |
| 6 | -0.36737740 | -2.35883713 | 5.21244344 |
| 6 | -1.30962532 | -2.54533834 | 4.01535919 |
| 8 | -0.74387342 | -2.58478828 | 6.34400582 |

| | | | |
|---|---|---|---|
| 6 | -1.97783916 | -3.91388815 | 4.04131977 |
| 6 | 2.28345240 | -2.79711235 | 3.25063587 |
| 1 | 2.00477063 | -0.71450958 | 3.76829786 |
| 1 | -2.07249242 | -1.75523312 | 4.13079858 |
| 1 | -2.71055200 | -3.97916639 | 3.22708843 |
| 1 | -2.48693613 | -4.05552303 | 5.00106383 |
| 1 | -1.23381936 | -4.71118046 | 3.91246192 |
| 1 | 2.82962545 | -2.56180800 | 2.32773251 |
| 1 | 1.69162919 | -3.70988677 | 3.10112542 |
| 1 | 3.01857241 | -2.98529636 | 4.04364447 |

Electronic energy = -4057.846167 a.u.
DFT-D3(BJ) dispersion correction = -0.139890 a.u.
Thermal free energy = 0.723295 a.u.
Gibbs free energy = -4057.262763 a.u.
Number of imaginary frequencies = 0.

**Int3a LA isomer/conformer 8**

| Atomic N. | X | Y | Z |
|---|---|---|---|
| 7 | 2.44820005 | 2.72098086 | -0.01124261 |
| 8 | 0.00834457 | 1.76624331 | 1.41795993 |
| 8 | 0.52020840 | 1.09161963 | -1.61546383 |
| 6 | 3.65965335 | 2.59614637 | 0.83628515 |
| 6 | 0.27563341 | 2.82837258 | 2.22434204 |
| 6 | -0.33030093 | 2.89707524 | 3.51573256 |
| 6 | -0.02494255 | 4.00772402 | 4.31502774 |
| 6 | 0.83240596 | 5.02973600 | 3.90078235 |
| 6 | 1.39715024 | 4.96402637 | 2.63307311 |
| 6 | 1.11539318 | 3.88566427 | 1.78579587 |
| 6 | 1.63569004 | 3.92463932 | 0.37420887 |
| 6 | -1.28369847 | 1.79973725 | 4.02821770 |
| 6 | 0.60456016 | 2.14482927 | -2.46562710 |
| 6 | -0.41836027 | 2.35376543 | -3.43988475 |
| 6 | -0.27641946 | 3.44967861 | -4.30219914 |
| 6 | 0.80941386 | 4.32723719 | -4.24383582 |
| 6 | 1.80417453 | 4.10916920 | -3.29881044 |
| 6 | 1.71887199 | 3.02275593 | -2.41957426 |
| 6 | 2.85442725 | 2.77686511 | -1.46241875 |
| 6 | -1.62853436 | 1.40797316 | -3.55927548 |
| 6 | -1.86179054 | 2.13342014 | 5.41678414 |
| 6 | -0.51433879 | 0.46594528 | 4.15929132 |
| 6 | -2.48407296 | 1.63901745 | 3.06542023 |
| 6 | -2.59424761 | 1.83908423 | -4.67936199 |
| 6 | -2.43535475 | 1.39948038 | -2.23978780 |
| 6 | -1.13623524 | -0.01790495 | -3.90192957 |
| 1 | 4.30831364 | 3.47569446 | 0.69241291 |
| 1 | 3.36602555 | 2.53493438 | 1.88965688 |
| 1 | 2.25272441 | 4.82712471 | 0.22667814 |
| 1 | 0.80527321 | 3.97336349 | -0.34604571 |
| 1 | -0.46935556 | 4.08012175 | 5.30573235 |
| 1 | 2.05095496 | 5.76285103 | 2.27585733 |
| 1 | 3.34765248 | 1.81285074 | -1.66809465 |
| 1 | 3.60939037 | 3.57259841 | -1.57763853 |

| | | | |
|---|---|---|---|
| 1 | -1.04558589 | 3.63389194 | -5.04974534 |
| 1 | 2.66693246 | 4.77636608 | -3.23872848 |
| 1 | -2.54197065 | 1.32525843 | 5.72151058 |
| 1 | -1.07996197 | 2.21179517 | 6.18586366 |
| 1 | -2.44088938 | 3.06813082 | 5.41069218 |
| 1 | -0.06399052 | 0.16353654 | 3.20897936 |
| 1 | 0.28948283 | 0.55615836 | 4.90456884 |
| 1 | -1.19725302 | -0.32987550 | 4.49489936 |
| 1 | -3.06275686 | 2.57275950 | 3.01184312 |
| 1 | -2.15924450 | 1.37622793 | 2.05345806 |
| 1 | -3.15443642 | 0.84885669 | 3.43658906 |
| 1 | -3.43105198 | 1.12735425 | -4.72087140 |
| 1 | -2.11137987 | 1.83763554 | -5.66714056 |
| 1 | -3.01880138 | 2.83738652 | -4.49971123 |
| 1 | -1.82762606 | 1.05715552 | -1.39608977 |
| 1 | -2.81059268 | 2.40727669 | -2.01004402 |
| 1 | -3.30398986 | 0.73091623 | -2.33897438 |
| 1 | -0.62018891 | -0.02278721 | -4.87312290 |
| 1 | -0.44681399 | -0.39637900 | -3.13942466 |
| 1 | -1.99389240 | -0.70426663 | -3.97176282 |
| 1 | 4.19823436 | 1.68342449 | 0.56063460 |
| 8 | 2.46791006 | -0.21545737 | 0.42605511 |
| 31 | 1.17579856 | 1.10430443 | 0.13057486 |
| 1 | 1.04559545 | 5.86938317 | 4.56212826 |
| 1 | 0.87156201 | 5.16967590 | -4.93239584 |
| 8 | -2.17013332 | -6.37743432 | -3.69013584 |
| 6 | -2.44696126 | -7.78639603 | -3.87831397 |
| 1 | -1.95648059 | -8.38061256 | -3.09734244 |
| 6 | -2.32809375 | -4.38112873 | -2.41343715 |
| 6 | -2.17389423 | -3.88158183 | -0.97421323 |
| 6 | -2.06041944 | -2.35482168 | -0.90028807 |
| 6 | -2.60388621 | -5.86991411 | -2.49866463 |
| 6 | -1.87043254 | -1.81521074 | 0.52448859 |
| 6 | -0.50320853 | -2.07406196 | 1.14053544 |
| 1 | -1.44696211 | -4.14159496 | -3.02554334 |
| 1 | -1.28822811 | -4.35918371 | -0.52569861 |
| 1 | -1.23265034 | -2.00182743 | -1.53468529 |
| 1 | -2.61866970 | -2.27181141 | 1.19399784 |
| 1 | -3.18258508 | -3.87559933 | -2.89716459 |
| 1 | -3.03655754 | -4.22430906 | -0.38320980 |
| 1 | -2.97568106 | -1.91121472 | -1.32521188 |
| 1 | -2.05755299 | -0.73063624 | 0.54788640 |
| 1 | -0.19111483 | -3.12004704 | 1.00375414 |
| 1 | -0.51113453 | -1.85158861 | 2.21458125 |
| 8 | -3.15656976 | -6.54020427 | -1.64717069 |
| 8 | 0.47612333 | -1.20464024 | 0.49008339 |
| 1 | -2.04223177 | -8.03157335 | -4.86492021 |
| 1 | -3.52772517 | -7.97325678 | -3.84335150 |
| 8 | 1.93431584 | -1.57658273 | 2.23623957 |
| 6 | 1.86789347 | -1.38314556 | 0.82518878 |
| 6 | 2.55883095 | -2.55986545 | 0.08334567 |
| 8 | 2.34856031 | -3.83385550 | 0.76484634 |
| 6 | 2.68940486 | -3.86555733 | 2.08981560 |

| | | | |
|---|---|---|---|
| 6 | 2.96270979 | -2.48239792 | 2.68946665 |
| 8 | 2.71455325 | -4.91493642 | 2.69232626 |
| 6 | 2.94167565 | -2.49503483 | 4.20593532 |
| 6 | 2.11010600 | -2.73218425 | -1.35403246 |
| 1 | 3.63714455 | -2.33490257 | 0.11003311 |
| 1 | 3.95294526 | -2.13465313 | 2.33454421 |
| 1 | 3.14879521 | -1.48793268 | 4.58815907 |
| 1 | 3.70074256 | -3.19076118 | 4.58241521 |
| 1 | 1.96175096 | -2.82509812 | 4.57181259 |
| 1 | 2.22196420 | -1.78227859 | -1.89133833 |
| 1 | 1.05830664 | -3.03704545 | -1.39966227 |
| 1 | 2.72345050 | -3.49666826 | -1.84692496 |

Electronic energy = -4057.847391 a.u.
DFT-D3(BJ) dispersion correction = -0.141624 a.u.
Thermal free energy = 0.726996 a.u.
Gibbs free energy = -4057.262019 a.u.
Number of imaginary frequencies = 0.

**Int3a LA isomer/conformer 9**

| Atomic N. | X | Y | Z |
|---|---|---|---|
| 7 | 1.16373753 | 3.33254606 | 1.86950354 |
| 8 | -1.06777380 | 1.82743192 | 0.59601506 |
| 8 | 1.89276689 | 1.46816745 | -0.35622170 |
| 6 | 1.08458836 | 3.39234293 | 3.34987744 |
| 6 | -1.82191070 | 2.79974537 | 1.16545938 |
| 6 | -3.22360372 | 2.59511172 | 1.33648326 |
| 6 | -3.95759300 | 3.62977637 | 1.93402212 |
| 6 | -3.37557357 | 4.82989149 | 2.35130847 |
| 6 | -2.01328200 | 5.02737506 | 2.15479189 |
| 6 | -1.23465417 | 4.03188763 | 1.55410200 |
| 6 | 0.21071645 | 4.31109720 | 1.24150951 |
| 6 | -3.91374086 | 1.29626898 | 0.87429849 |
| 6 | 2.45663621 | 2.58205855 | -0.88129625 |
| 6 | 2.74408174 | 2.64275151 | -2.27864048 |
| 6 | 3.34304242 | 3.81284073 | -2.76517199 |
| 6 | 3.65897285 | 4.90066819 | -1.94666719 |
| 6 | 3.37933541 | 4.82838417 | -0.58727932 |
| 6 | 2.79345938 | 3.67792472 | -0.04589206 |
| 6 | 2.58521153 | 3.59885128 | 1.44269607 |
| 6 | 2.41222496 | 1.47168647 | -3.22358700 |
| 6 | -5.43033948 | 1.32099491 | 1.14188862 |
| 6 | -3.33136392 | 0.08071801 | 1.63272885 |
| 6 | -3.71793857 | 1.10810755 | -0.64924045 |
| 6 | 2.83244027 | 1.76024953 | -4.67729457 |
| 6 | 0.88586002 | 1.21906303 | -3.23436141 |
| 6 | 3.17202150 | 0.20096298 | -2.77416842 |
| 1 | 1.36962840 | 4.39896762 | 3.69805576 |
| 1 | 0.06037270 | 3.17785965 | 3.67250877 |
| 1 | 0.47995715 | 5.32766653 | 1.57564008 |
| 1 | 0.39126516 | 4.25929672 | 0.15758141 |
| 1 | -5.02739588 | 3.49802333 | 2.08519544 |
| 1 | -1.54112641 | 5.96712846 | 2.45039938 |

| | | | |
|---|---|---|---|
| 1 | 3.18137935 | 2.78247442 | 1.88211896 |
| 1 | 2.91263686 | 4.54360933 | 1.90866592 |
| 1 | 3.56950100 | 3.88637793 | -3.82722918 |
| 1 | 3.62406180 | 5.66296873 | 0.07359436 |
| 1 | -5.87089251 | 0.37958950 | 0.78407325 |
| 1 | -5.66170322 | 1.40771086 | 2.21348220 |
| 1 | -5.93058974 | 2.14304801 | 0.61004525 |
| 1 | -2.26105360 | -0.03567255 | 1.43115919 |
| 1 | -3.47848547 | 0.19344458 | 2.71725130 |
| 1 | -3.84634933 | -0.83901926 | 1.31578361 |
| 1 | -4.17045314 | 1.94383600 | -1.20248779 |
| 1 | -2.65625423 | 1.05082547 | -0.91335454 |
| 1 | -4.21208790 | 0.18034128 | -0.97608264 |
| 1 | 2.57915128 | 0.89091213 | -5.30090537 |
| 1 | 3.91509587 | 1.92840399 | -4.76935507 |
| 1 | 2.30723970 | 2.63058073 | -5.09630930 |
| 1 | 0.49990617 | 1.00458849 | -2.23198813 |
| 1 | 0.35317438 | 2.09948249 | -3.62179833 |
| 1 | 0.65133399 | 0.36711495 | -3.89096986 |
| 1 | 4.25800346 | 0.35726563 | -2.84985350 |
| 1 | 2.93595362 | -0.06656513 | -1.73876365 |
| 1 | 2.90787173 | -0.64438916 | -3.42821516 |
| 1 | 1.75952126 | 2.64329750 | 3.77659272 |
| 8 | 1.12098586 | 0.48860005 | 2.65296126 |
| 31 | 0.69584864 | 1.46425879 | 1.07869788 |
| 1 | -3.98747646 | 5.60264318 | 2.81648943 |
| 1 | 4.11843246 | 5.79226827 | -2.37291464 |
| 8 | -2.01503960 | -7.19664222 | -4.32719064 |
| 6 | -3.07593387 | -8.10672435 | -4.70422882 |
| 1 | -3.36915585 | -8.72945375 | -3.84972541 |
| 6 | -1.17396543 | -5.41287471 | -3.01243080 |
| 6 | -1.43489742 | -4.48009773 | -1.83038940 |
| 6 | -0.26169533 | -3.52962778 | -1.56924218 |
| 6 | -2.34136120 | -6.32974520 | -3.32321685 |
| 6 | -0.50827694 | -2.60297896 | -0.37175483 |
| 6 | 0.66173862 | -1.64838965 | -0.16153685 |
| 1 | -0.28542504 | -6.04059892 | -2.83847991 |
| 1 | -1.63504412 | -5.07997171 | -0.92959916 |
| 1 | 0.65478619 | -4.11946234 | -1.39388935 |
| 1 | -0.67609779 | -3.20495320 | 0.53366264 |
| 1 | -0.95278574 | -4.83818227 | -3.92743993 |
| 1 | -2.35196872 | -3.90193111 | -2.01962097 |
| 1 | -0.07225347 | -2.92356284 | -2.47259343 |
| 1 | -1.42110884 | -2.00985470 | -0.53954034 |
| 1 | 0.81573794 | -1.02547977 | -1.05306864 |
| 1 | 1.60526242 | -2.18132776 | 0.01934776 |
| 8 | -3.42826586 | -6.31494948 | -2.77778453 |
| 8 | 0.42497412 | -0.71181999 | 0.92493796 |
| 1 | -2.65797019 | -8.72174388 | -5.50699765 |
| 1 | -3.95278372 | -7.54946048 | -5.05740157 |
| 8 | 2.42334976 | -1.28747497 | 2.13558356 |
| 6 | 1.10935447 | -0.81112089 | 2.27159045 |
| 6 | 0.25200281 | -1.66106963 | 3.22558432 |

| | | | |
|---|---|---|---|
| 8 | 0.33557594 | -3.08748130 | 2.91035006 |
| 6 | 1.53530853 | -3.62504294 | 2.57875396 |
| 6 | 2.71666708 | -2.69758900 | 2.29949795 |
| 8 | 1.65246877 | -4.82731511 | 2.46296055 |
| 6 | 3.81783822 | -2.86792642 | 3.34842356 |
| 6 | 0.55791247 | -1.42613005 | 4.69878810 |
| 1 | -0.79611784 | -1.41098011 | 3.01938466 |
| 1 | 3.11491675 | -3.04928117 | 1.33361923 |
| 1 | 4.67864320 | -2.24877110 | 3.06727690 |
| 1 | 4.12805504 | -3.91957844 | 3.38453141 |
| 1 | 3.47712747 | -2.56213701 | 4.34548885 |
| 1 | 0.32269783 | -0.38742551 | 4.95734829 |
| 1 | 1.61341765 | -1.60832747 | 4.93651418 |
| 1 | -0.06246297 | -2.09635843 | 5.30755396 |

Electronic energy = -4057.842297 a.u.
DFT-D3(BJ) dispersion correction = -0.139582 a.u.
Thermal free energy = 0.723674 a.u.
Gibbs free energy = -4057.258204 a.u.
Number of imaginary frequencies = 0.

**Int3b LA isomer/conformer 1**

| Atomic N. | X | Y | Z |
|---|---|---|---|
| 7 | 0.70263742 | 1.96119427 | 2.37171966 |
| 8 | -0.53283997 | 1.37939348 | -0.29910315 |
| 8 | 2.44254438 | 2.23720735 | -0.07275019 |
| 6 | 0.46243315 | 0.97365172 | 3.45315719 |
| 6 | -1.69928169 | 1.51440574 | 0.38455016 |
| 6 | -2.92072340 | 1.08221344 | -0.21284614 |
| 6 | -4.08648155 | 1.18881419 | 0.55988633 |
| 6 | -4.09598037 | 1.72079199 | 1.85191726 |
| 6 | -2.91203140 | 2.21247621 | 2.39001875 |
| 6 | -1.71956024 | 2.13554323 | 1.66140883 |
| 6 | -0.49681586 | 2.84601189 | 2.17794830 |
| 1 | -5.02720457 | 1.77426307 | 2.41565423 |
| 6 | -2.97862352 | 0.56424553 | -1.66439468 |
| 6 | 2.52032539 | 3.52893334 | 0.34339002 |
| 6 | 2.91580805 | 4.55122933 | -0.56968279 |
| 6 | 2.98692713 | 5.86113849 | -0.07490712 |
| 6 | 2.69510775 | 6.19142373 | 1.25153628 |
| 6 | 2.32712645 | 5.18465404 | 2.13539093 |
| 6 | 2.24889790 | 3.85672325 | 1.69726139 |
| 6 | 1.93280247 | 2.77685515 | 2.69732539 |
| 1 | 2.76025055 | 7.22693347 | 1.58522528 |
| 6 | 3.27260825 | 4.23717180 | -2.03486386 |
| 6 | -4.41970485 | 0.24583436 | -2.10589481 |
| 6 | -2.15268586 | -0.73199697 | -1.81641879 |
| 6 | -2.43336413 | 1.64909410 | -2.62464101 |
| 6 | 3.66388890 | 5.50380890 | -2.81950715 |
| 6 | 2.05910509 | 3.61066804 | -2.76101242 |
| 6 | 4.48324224 | 3.27576682 | -2.07726839 |
| 1 | 0.31051808 | 1.49977407 | 4.40978036 |
| 1 | -0.43264501 | 0.38702888 | 3.21922249 |

| | | | |
|---|---|---|---|
| 1 | -0.72443802 | 3.34020050 | 3.13762286 |
| 1 | -0.18264366 | 3.62608461 | 1.46855044 |
| 1 | -5.03020692 | 0.84804128 | 0.13825300 |
| 1 | -2.90517658 | 2.68690287 | 3.37399569 |
| 1 | 2.75727554 | 2.04908967 | 2.76683327 |
| 1 | 1.79170104 | 3.22544974 | 3.69481105 |
| 1 | 3.27872901 | 6.66237123 | -0.75118137 |
| 1 | 2.10767125 | 5.41616418 | 3.18010347 |
| 1 | -4.40458885 | -0.09416566 | -3.15116997 |
| 1 | -4.87142021 | -0.55680988 | -1.50483058 |
| 1 | -5.07268833 | 1.12882163 | -2.05494781 |
| 1 | -1.10065201 | -0.56782307 | -1.56014396 |
| 1 | -2.55657402 | -1.52553714 | -1.16992908 |
| 1 | -2.20651191 | -1.08824471 | -2.85678047 |
| 1 | -3.03891363 | 2.56440832 | -2.55636797 |
| 1 | -1.39206165 | 1.90296584 | -2.39811314 |
| 1 | -2.48605771 | 1.28673241 | -3.66255167 |
| 1 | 3.91315173 | 5.21791483 | -3.85115266 |
| 1 | 4.54850261 | 5.99848008 | -2.39316381 |
| 1 | 2.84320572 | 6.23462568 | -2.86724483 |
| 1 | 1.73626114 | 2.68157334 | -2.28071090 |
| 1 | 1.20881724 | 4.30838184 | -2.77067085 |
| 1 | 2.32781266 | 3.38723735 | -3.80433142 |
| 1 | 5.36735008 | 3.75426962 | -1.63095349 |
| 1 | 4.28581712 | 2.34382011 | -1.53914206 |
| 1 | 4.72529102 | 3.01923780 | -3.11878648 |
| 1 | 1.32252258 | 0.30015910 | 3.52719767 |
| 8 | 1.72483637 | -0.53467867 | 1.16242858 |
| 8 | 1.59126829 | -0.79456046 | -1.05619807 |
| 31 | 1.10563239 | 1.10822479 | 0.54096120 |
| 6 | 2.07175853 | -1.41701833 | 0.17358419 |
| 6 | 3.59699702 | -1.72159124 | 0.04731136 |
| 8 | 4.27117095 | -0.78918570 | -0.84786292 |
| 6 | 3.75478068 | -0.67650396 | -2.10761960 |
| 6 | 2.31949903 | -1.19646155 | -2.24234280 |
| 8 | 4.39791618 | -0.16439849 | -2.99597359 |
| 6 | 1.61438706 | -0.65541719 | -3.47061697 |
| 6 | 4.32381019 | -1.66841119 | 1.37539256 |
| 1 | 3.68184767 | -2.72811514 | -0.39013254 |
| 1 | 2.34238387 | -2.30201250 | -2.27927166 |
| 1 | 0.60921814 | -1.08697031 | -3.54742090 |
| 1 | 2.18998597 | -0.91595714 | -4.36652494 |
| 1 | 1.53601846 | 0.43753908 | -3.42140515 |
| 1 | 3.86809807 | -2.37993049 | 2.07568409 |
| 1 | 4.25979398 | -0.66063551 | 1.80220457 |
| 1 | 5.37831155 | -1.93580021 | 1.23353049 |
| 8 | -5.62794179 | -7.33709114 | 1.79300650 |
| 6 | -6.22564807 | -8.65459917 | 1.73486040 |
| 1 | -6.19005629 | -9.04731627 | 0.71092454 |
| 6 | -3.78053546 | -5.87685905 | 1.52264892 |
| 6 | -2.34160008 | -5.73543360 | 1.02775395 |
| 6 | -1.81592549 | -4.30257471 | 1.16262651 |
| 6 | -4.31597764 | -7.29186433 | 1.41626047 |

| | | | |
|---|---|---|---|
| 6 | -0.37620720 | -4.15004142 | 0.65880530 |
| 6 | 0.13034209 | -2.72136169 | 0.79526585 |
| 1 | -4.46180728 | -5.21333208 | 0.96621055 |
| 1 | -2.28592600 | -6.05620188 | -0.02394126 |
| 1 | -2.47598519 | -3.61548077 | 0.60533847 |
| 1 | -0.31510161 | -4.45485818 | -0.39806581 |
| 1 | -3.86769604 | -5.56763417 | 2.57760131 |
| 1 | -1.69464168 | -6.42704856 | 1.58849710 |
| 1 | -1.87065541 | -3.99033027 | 2.22010092 |
| 1 | 0.29033785 | -4.82219141 | 1.22284675 |
| 1 | 0.07899990 | -2.38553107 | 1.84312964 |
| 1 | -0.47197975 | -2.03356308 | 0.18187080 |
| 8 | -3.68849602 | -8.26882569 | 1.05416277 |
| 8 | 1.50661396 | -2.69751322 | 0.35370007 |
| 1 | -7.26076416 | -8.51747230 | 2.06203991 |
| 1 | -5.69551906 | -9.34659621 | 2.40133116 |

Electronic energy = -4057.849163 a.u.
DFT-D3(BJ) dispersion correction = -0.139314 a.u.
Thermal free energy = 0.723522 a.u.
Gibbs free energy = -4057.264955 a.u.
Number of imaginary frequencies = 0.

**Int3b_LA isomer/conformer 2**

| Atomic N. | X | Y | Z |
|---|---|---|---|
| 7 | 0.88920097 | 1.57311153 | 2.63442557 |
| 8 | -0.40112421 | 1.30383265 | -0.06174800 |
| 8 | 2.55702661 | 2.09770176 | 0.18068368 |
| 6 | 0.65777428 | 0.48566504 | 3.61807683 |
| 6 | -1.55618927 | 1.38746871 | 0.64813944 |
| 6 | -2.79552303 | 1.06785838 | 0.01827456 |
| 6 | -3.94676225 | 1.09449819 | 0.81928138 |
| 6 | -3.92452822 | 1.45450970 | 2.16942181 |
| 6 | -2.72386507 | 1.85780716 | 2.74344090 |
| 6 | -1.54427362 | 1.85119245 | 1.99022870 |
| 6 | -0.30256809 | 2.48591303 | 2.55952759 |
| 1 | -4.84532709 | 1.45053227 | 2.75240955 |
| 6 | -2.88811035 | 0.77484879 | -1.49386197 |
| 6 | 2.66762814 | 3.33176344 | 0.73457541 |
| 6 | 3.03781048 | 4.44497338 | -0.07736046 |
| 6 | 3.16699968 | 5.68863590 | 0.55676564 |
| 6 | 2.94285279 | 5.87138648 | 1.92422381 |
| 6 | 2.58027577 | 4.78002669 | 2.70415518 |
| 6 | 2.45132289 | 3.51117653 | 2.12582059 |
| 6 | 2.13893304 | 2.33463918 | 3.01288745 |
| 1 | 3.05025495 | 6.86033668 | 2.36935032 |
| 6 | 3.28157503 | 4.29918446 | -1.59255705 |
| 6 | -4.34136520 | 0.54020557 | -1.94738349 |
| 6 | -2.08042792 | -0.48609302 | -1.87145146 |
| 6 | -2.35000795 | 1.99367053 | -2.28248799 |
| 6 | 3.67896600 | 5.63691668 | -2.24492094 |
| 6 | 1.98976076 | 3.81395124 | -2.29185170 |
| 6 | 4.43522363 | 3.30242153 | -1.85331610 |

| | | | |
|---|---|---|---|
| 1 | 0.53628446 | 0.91622283 | 4.62542735 |
| 1 | -0.25257769 | -0.06117492 | 3.34918638 |
| 1 | -0.50796849 | 2.87548458 | 3.57099877 |
| 1 | 0.00844453 | 3.33491976 | 1.93268441 |
| 1 | -4.90427051 | 0.83103277 | 0.37391458 |
| 1 | -2.69407317 | 2.20895324 | 3.77742405 |
| 1 | 2.95251082 | 1.59188400 | 2.98768222 |
| 1 | 2.02810063 | 2.67792620 | 4.05497420 |
| 1 | 3.44690280 | 6.55621477 | -0.03765993 |
| 1 | 2.40448447 | 4.89872777 | 3.77564196 |
| 1 | -4.35149465 | 0.35956257 | -3.03162560 |
| 1 | -4.79078149 | -0.33863741 | -1.46239669 |
| 1 | -4.98190781 | 1.41183739 | -1.75150398 |
| 1 | -1.02375954 | -0.37279797 | -1.60655262 |
| 1 | -2.48189090 | -1.37239473 | -1.35821669 |
| 1 | -2.15305074 | -0.66586729 | -2.95487961 |
| 1 | -2.94415165 | 2.89131537 | -2.05851511 |
| 1 | -1.30185993 | 2.20123645 | -2.03904806 |
| 1 | -2.42509668 | 1.80019104 | -3.36334400 |
| 1 | 3.84385719 | 5.47352638 | -3.31933190 |
| 1 | 4.61151378 | 6.04160152 | -1.82579679 |
| 1 | 2.89169235 | 6.39789938 | -2.14437985 |
| 1 | 1.66140245 | 2.84236135 | -1.90860773 |
| 1 | 1.17463208 | 4.53728650 | -2.14541935 |
| 1 | 2.16622011 | 3.71849845 | -3.37402387 |
| 1 | 5.36908088 | 3.66191827 | -1.39719782 |
| 1 | 4.20832840 | 2.31233828 | -1.44428020 |
| 1 | 4.60515613 | 3.20385052 | -2.93619120 |
| 1 | 1.50718410 | -0.20475288 | 3.60387766 |
| 8 | 1.90096900 | -0.78966564 | 1.19007233 |
| 8 | 1.74683402 | -0.71007725 | -1.02619970 |
| 31 | 1.24191748 | 0.89915632 | 0.71705747 |
| 6 | 2.14379314 | -1.56521988 | 0.08750489 |
| 6 | 3.63163980 | -1.92447151 | -0.10430127 |
| 8 | 3.85193862 | -2.53705064 | -1.41763100 |
| 6 | 3.06573382 | -2.25377705 | -2.48530772 |
| 6 | 1.85884131 | -1.31818264 | -2.33240945 |
| 8 | 3.30107235 | -2.77136821 | -3.55772699 |
| 6 | 1.89125695 | -0.20495558 | -3.37231557 |
| 6 | 4.58694557 | -0.75710339 | 0.06955730 |
| 1 | 3.87203693 | -2.73121805 | 0.60161109 |
| 1 | 0.97599715 | -1.95948275 | -2.49887646 |
| 1 | 0.97813625 | 0.39895840 | -3.29790607 |
| 1 | 1.95365418 | -0.64715747 | -4.37285186 |
| 1 | 2.75955857 | 0.44770041 | -3.21406104 |
| 1 | 4.56413789 | -0.40326394 | 1.10747427 |
| 1 | 4.31993888 | 0.08056776 | -0.58706868 |
| 1 | 5.60593753 | -1.08723784 | -0.16836940 |
| 8 | -6.01638395 | -6.99533184 | 1.04841954 |
| 6 | -6.70255384 | -8.25956286 | 0.88265481 |
| 1 | -6.64540938 | -8.59313803 | -0.16106421 |
| 6 | -4.05517009 | -5.66633474 | 0.96222053 |
| 6 | -2.61338854 | -5.57779115 | 0.46281338 |

| | | | |
|---|---|---|---|
| 6 | -1.98274953 | -4.20926307 | 0.74074419 |
| 6 | -4.68744156 | -7.02591809 | 0.73387353 |
| 6 | -0.53987321 | -4.10933068 | 0.23289962 |
| 6 | 0.07717553 | -2.74738749 | 0.51766055 |
| 1 | -4.69349240 | -4.90641901 | 0.48456735 |
| 1 | -2.58899770 | -5.78694963 | -0.61790698 |
| 1 | -2.59635460 | -3.42050909 | 0.27206653 |
| 1 | -0.50867017 | -4.30258953 | -0.85109364 |
| 1 | -4.10889847 | -5.45724242 | 2.04408986 |
| 1 | -2.01518639 | -6.37041136 | 0.93684991 |
| 1 | -2.00483930 | -4.01018679 | 1.82642665 |
| 1 | 0.07889667 | -4.88551531 | 0.71050519 |
| 1 | 0.04609080 | -2.52208315 | 1.59556345 |
| 1 | -0.46503074 | -1.95063237 | -0.01584616 |
| 8 | -4.11573989 | -8.02292053 | 0.33630760 |
| 8 | 1.45476825 | -2.79131435 | 0.08295035 |
| 1 | -7.73994347 | -8.06593443 | 1.17192385 |
| 1 | -6.25559213 | -9.02668424 | 1.52753211 |

Electronic energy = -4057.847770 a.u.
DFT-D3(BJ) dispersion correction = -0.140827 a.u.
Thermal free energy = 0.723727 a.u.
Gibbs free energy = -4057.264870 a.u.
Number of imaginary frequencies = 0.

**TS4 eq LA isomer/conformer 1**

| Atomic N. | X | Y | Z |
|---|---|---|---|
| 7 | 0.72881037 | 1.61458802 | 1.93882227 |
| 8 | -0.56455934 | 0.96878934 | -0.60990524 |
| 8 | 2.34316182 | 2.30298281 | -0.43123662 |
| 6 | 0.25074929 | 0.65740287 | 2.96676373 |
| 6 | -1.69304860 | 1.29048455 | 0.05374647 |
| 6 | -2.95265102 | 0.85377312 | -0.45913869 |
| 6 | -4.09784555 | 1.18457031 | 0.27868989 |
| 6 | -4.04822445 | 1.92192686 | 1.46576905 |
| 6 | -2.81789970 | 2.36839509 | 1.93663585 |
| 6 | -1.64429152 | 2.07166195 | 1.23547006 |
| 6 | -0.33461317 | 2.65221071 | 1.69177175 |
| 1 | -4.96643162 | 2.15250230 | 2.00591421 |
| 6 | -3.05499220 | 0.07721424 | -1.78742802 |
| 6 | 2.54893112 | 3.45414758 | 0.25894877 |
| 6 | 2.88722157 | 4.66030407 | -0.43203217 |
| 6 | 3.14645982 | 5.79808283 | 0.34574863 |
| 6 | 3.05957842 | 5.80780458 | 1.73937893 |
| 6 | 2.67576075 | 4.64580870 | 2.39559245 |
| 6 | 2.42448735 | 3.47492170 | 1.67110014 |
| 6 | 2.01295018 | 2.24363494 | 2.42833567 |
| 1 | 3.26936483 | 6.72040129 | 2.29705095 |
| 6 | 2.95460725 | 4.74295378 | -1.96937954 |
| 6 | -4.51392746 | -0.26544696 | -2.14292026 |
| 6 | -2.27527308 | -1.25633144 | -1.70385587 |
| 6 | -2.48790765 | 0.93926775 | -2.94099212 |
| 6 | 3.25449157 | 6.17164373 | -2.46420598 |

| | | | |
|---|---|---|---|
| 6 | 1.58890975 | 4.34162474 | -2.57604909 |
| 6 | 4.08428240 | 3.83283234 | -2.49800205 |
| 1 | -0.05905835 | 1.21608984 | 3.86535263 |
| 1 | -0.61047530 | 0.09845331 | 2.58637691 |
| 1 | -0.47914717 | 3.22866201 | 2.62069821 |
| 1 | 0.06773839 | 3.34429431 | 0.93851590 |
| 1 | -5.07164764 | 0.85937148 | -0.08330785 |
| 1 | -2.75760961 | 2.96837664 | 2.84766936 |
| 1 | 2.77211928 | 1.44794285 | 2.37181473 |
| 1 | 1.87821853 | 2.50612164 | 3.49072886 |
| 1 | 3.41822314 | 6.72412586 | -0.15650655 |
| 1 | 2.56899881 | 4.63159180 | 3.48262382 |
| 1 | -4.53116226 | -0.80715209 | -3.09937525 |
| 1 | -4.98432016 | -0.91195279 | -1.38741708 |
| 1 | -5.13279581 | 0.63530385 | -2.26259851 |
| 1 | -1.20762503 | -1.08489859 | -1.52602911 |
| 1 | -2.67977309 | -1.88833964 | -0.89812553 |
| 1 | -2.38371539 | -1.81028879 | -2.64891791 |
| 1 | -3.06191874 | 1.87139988 | -3.04443169 |
| 1 | -1.43629932 | 1.19390130 | -2.76951742 |
| 1 | -2.56397080 | 0.38793480 | -3.89078736 |
| 1 | 3.26860619 | 6.16741657 | -3.56351662 |
| 1 | 4.23705578 | 6.53094244 | -2.12611771 |
| 1 | 2.48841262 | 6.89343691 | -2.14532328 |
| 1 | 1.28312039 | 3.33521628 | -2.27234840 |
| 1 | 0.80603415 | 5.04689789 | -2.25982404 |
| 1 | 1.64513910 | 4.37108803 | -3.67465949 |
| 1 | 5.06049967 | 4.18376875 | -2.13291860 |
| 1 | 3.96536732 | 2.79205251 | -2.18624282 |
| 1 | 4.10750103 | 3.86283326 | -3.59826279 |
| 1 | 1.05628264 | -0.03542948 | 3.21984267 |
| 8 | 2.04745507 | -0.79649490 | 1.30506349 |
| 8 | 1.67989087 | -0.53498101 | -1.18607748 |
| 31 | 1.20595479 | 0.87876618 | 0.01142624 |
| 6 | 2.33601809 | -1.75329459 | 0.55530089 |
| 6 | 3.71315885 | -2.00785017 | -0.05405027 |
| 8 | 4.38082504 | -0.80023223 | -0.45927238 |
| 6 | 4.05059338 | -0.20364198 | -1.66783786 |
| 6 | 2.64671969 | -0.51391876 | -2.21979403 |
| 8 | 4.86310482 | 0.52542442 | -2.18441629 |
| 6 | 2.28128314 | 0.39813092 | -3.38594413 |
| 6 | 4.61820412 | -2.67411351 | 0.98410380 |
| 1 | 3.57875848 | -2.67719245 | -0.91691065 |
| 1 | 2.69767237 | -1.55274796 | -2.61076188 |
| 1 | 1.29388630 | 0.10893190 | -3.76731491 |
| 1 | 3.02148485 | 0.30974674 | -4.19132376 |
| 1 | 2.25160956 | 1.44438946 | -3.06223249 |
| 1 | 4.18288851 | -3.62387943 | 1.32012749 |
| 1 | 4.75367928 | -2.01080275 | 1.84791720 |
| 1 | 5.59911060 | -2.87100053 | 0.53504610 |
| 8 | -5.80526924 | -6.94854879 | 2.24528217 |
| 6 | -6.55532646 | -8.17772102 | 2.08845592 |
| 1 | -6.67179346 | -8.42257023 | 1.02536273 |

| | | | |
|---|---|---|---|
| 6 | -3.83977246 | -5.65799093 | 1.95123434 |
| 6 | -2.47224569 | -5.58198833 | 1.27341330 |
| 6 | -1.75969899 | -4.25310707 | 1.54516745 |
| 6 | -4.54573631 | -6.98122168 | 1.72187638 |
| 6 | -0.39309433 | -4.16912842 | 0.85392892 |
| 6 | 0.28575984 | -2.83750510 | 1.11959267 |
| 1 | -4.50443840 | -4.85079479 | 1.60494363 |
| 1 | -2.59593821 | -5.72251463 | 0.18860920 |
| 1 | -2.39575458 | -3.41788054 | 1.20587707 |
| 1 | -0.51403862 | -4.30060530 | -0.23241800 |
| 1 | -3.74856400 | -5.51681042 | 3.04125905 |
| 1 | -1.84864235 | -6.42081356 | 1.61775982 |
| 1 | -1.63121247 | -4.12136221 | 2.63364983 |
| 1 | 0.25593740 | -4.98554373 | 1.20844221 |
| 1 | 0.44121736 | -2.66152477 | 2.19395304 |
| 1 | -0.29078227 | -1.99914873 | 0.70257056 |
| 8 | -4.07592058 | -7.95222616 | 1.15982652 |
| 8 | 1.59156895 | -2.86289620 | 0.46254587 |
| 1 | -7.52767038 | -7.98395872 | 2.55114675 |
| 1 | -6.04278135 | -9.00669003 | 2.59248328 |

Electronic energy = -4057.833662 a.u.
DFT-D3(BJ) dispersion correction = -0.141402 a.u.
Thermal free energy = 0.724402 a.u.
Gibbs free energy = -4057.250662 a.u.
Number of imaginary frequencies = 1.

**TS4 eq LA isomer/conformer 2**

| Atomic N. | X | Y | Z |
|---|---|---|---|
| 7 | 0.57425237 | 1.35181999 | 2.14091277 |
| 8 | -0.64652061 | 1.33203030 | -0.53427446 |
| 8 | 2.39117336 | 2.13480306 | -0.06668935 |
| 6 | -0.01494482 | 0.27699900 | 2.97969866 |
| 6 | -1.78089631 | 1.59259701 | 0.15329099 |
| 6 | -3.05137563 | 1.32288122 | -0.44049445 |
| 6 | -4.19254541 | 1.61891174 | 0.31830603 |
| 6 | -4.13000107 | 2.15316939 | 1.60867286 |
| 6 | -2.88689375 | 2.41290689 | 2.17501163 |
| 6 | -1.71675301 | 2.14758444 | 1.45568180 |
| 6 | -0.38285026 | 2.51466990 | 2.03897643 |
| 1 | -5.04664326 | 2.36426830 | 2.15914416 |
| 6 | -3.17122960 | 0.72652966 | -1.85623050 |
| 6 | 2.71624637 | 3.15068126 | 0.77065712 |
| 6 | 3.32163119 | 4.33412552 | 0.24666589 |
| 6 | 3.65920877 | 5.34422970 | 1.15826201 |
| 6 | 3.41741896 | 5.24202728 | 2.53029537 |
| 6 | 2.81425858 | 4.09267998 | 3.02552438 |
| 6 | 2.47172832 | 3.04406095 | 2.16335821 |
| 6 | 1.88266587 | 1.79224920 | 2.75805521 |
| 1 | 3.69241905 | 6.05795479 | 3.19836807 |
| 6 | 3.59365392 | 4.51375723 | -1.26004255 |
| 6 | -4.63864422 | 0.54184073 | -2.28641391 |
| 6 | -2.50276923 | -0.66817522 | -1.89609873 |

| | | | |
|---|---|---|---|
| 6 | -2.50664115 | 1.66771019 | -2.88843894 |
| 6 | 4.24329615 | 5.87474108 | -1.57548559 |
| 6 | 2.26337719 | 4.45194149 | -2.04700017 |
| 6 | 4.56867552 | 3.42101693 | -1.75613093 |
| 1 | -0.26331639 | 0.68423980 | 3.97306037 |
| 1 | -0.93327045 | -0.09496803 | 2.51277161 |
| 1 | -0.51215225 | 2.93697906 | 3.04910350 |
| 1 | 0.11981123 | 3.27707100 | 1.42637908 |
| 1 | -5.17435694 | 1.42322731 | -0.10907441 |
| 1 | -2.81200290 | 2.84008121 | 3.17768645 |
| 1 | 2.56037617 | 0.93261051 | 2.65255046 |
| 1 | 1.70390487 | 1.95586503 | 3.83350229 |
| 1 | 4.12356663 | 6.25500631 | 0.78545141 |
| 1 | 2.60953474 | 3.99126077 | 4.09368801 |
| 1 | -4.66278219 | 0.11970595 | -3.30116868 |
| 1 | -5.18080330 | -0.15148143 | -1.62714827 |
| 1 | -5.18441868 | 1.49588609 | -2.31259203 |
| 1 | -1.44587672 | -0.61274278 | -1.61278212 |
| 1 | -3.01451778 | -1.35913348 | -1.20916188 |
| 1 | -2.57114077 | -1.08964443 | -2.91036940 |
| 1 | -3.01625252 | 2.64200425 | -2.90392518 |
| 1 | -1.44939482 | 1.83585203 | -2.65778518 |
| 1 | -2.58165455 | 1.23100483 | -3.89585090 |
| 1 | 4.41172981 | 5.94486427 | -2.65954113 |
| 1 | 5.21868563 | 5.99355745 | -1.08205044 |
| 1 | 3.60030937 | 6.71848631 | -1.28647172 |
| 1 | 1.73950040 | 3.50455451 | -1.88595831 |
| 1 | 1.59724092 | 5.27145863 | -1.74033892 |
| 1 | 2.45989776 | 4.56188059 | -3.12416101 |
| 1 | 5.53812599 | 3.50816107 | -1.24426556 |
| 1 | 4.17260456 | 2.41708231 | -1.57329416 |
| 1 | 4.74462271 | 3.53632903 | -2.83630514 |
| 1 | 0.70988101 | -0.53553921 | 3.07519460 |
| 8 | 2.09473920 | -0.98433107 | 1.28211510 |
| 8 | 1.43708801 | -0.54235274 | -1.01147878 |
| 31 | 1.03771663 | 0.86909789 | 0.17068399 |
| 6 | 2.29856968 | -1.79804993 | 0.35027653 |
| 6 | 3.60307336 | -1.88517916 | -0.44886166 |
| 8 | 3.40818286 | -2.33332109 | -1.82322490 |
| 6 | 2.57651782 | -1.78049886 | -2.76228499 |
| 6 | 1.52943039 | -0.70499754 | -2.41151547 |
| 8 | 2.65891004 | -2.19427085 | -3.89982247 |
| 6 | 1.83276665 | 0.58515775 | -3.17735291 |
| 6 | 4.47309208 | -0.64433920 | -0.36228722 |
| 1 | 4.15578985 | -2.73731732 | -0.01722391 |
| 1 | 0.58275670 | -1.12367940 | -2.80081677 |
| 1 | 1.02005339 | 1.30597806 | -3.01772594 |
| 1 | 1.90523493 | 0.36687750 | -4.24972963 |
| 1 | 2.77474618 | 1.03449368 | -2.83818030 |
| 1 | 4.75452471 | -0.46234614 | 0.68313658 |
| 1 | 3.95317817 | 0.24613801 | -0.73189622 |
| 1 | 5.38771868 | -0.80544865 | -0.94645745 |
| 8 | -5.72443676 | -7.27260876 | 1.65905154 |

| | | | |
|---|---|---|---|
| 6 | -6.43741179 | -8.51607895 | 1.45073533 |
| 1 | -6.48708916 | -8.75464916 | 0.38100287 |
| 6 | -3.76940870 | -5.94279861 | 1.49553418 |
| 6 | -2.38584924 | -5.81294727 | 0.85939664 |
| 6 | -1.71281970 | -4.47823715 | 1.19622159 |
| 6 | -4.43479633 | -7.27643824 | 1.21197057 |
| 6 | -0.33517388 | -4.33098555 | 0.53910017 |
| 6 | 0.31899562 | -3.00810170 | 0.89995718 |
| 1 | -4.44527960 | -5.14244366 | 1.15571904 |
| 1 | -2.47568631 | -5.91905928 | -0.23270544 |
| 1 | -2.36311269 | -3.64661646 | 0.87384576 |
| 1 | -0.43342769 | -4.39591694 | -0.55545282 |
| 1 | -3.70968413 | -5.83267307 | 2.59159040 |
| 1 | -1.75239730 | -6.64845228 | 1.19296813 |
| 1 | -1.61266673 | -4.38596010 | 2.29195023 |
| 1 | 0.32173595 | -5.15787172 | 0.85144234 |
| 1 | 0.49701047 | -2.92223763 | 1.98208821 |
| 1 | -0.29734096 | -2.15534401 | 0.57435441 |
| 8 | -3.91355538 | -8.23354912 | 0.67235208 |
| 8 | 1.59493077 | -2.93720865 | 0.20549700 |
| 1 | -7.43883419 | -8.34683800 | 1.85777092 |
| 1 | -5.93693972 | -9.33826542 | 1.97753084 |

Electronic energy = -4057.830169 a.u.
DFT-D3(BJ) dispersion correction = -0.142162 a.u.
Thermal free energy = 0.723760 a.u.
Gibbs free energy = -4057.248572 a.u.
Number of imaginary frequencies = 1.

**Int4 eq LA isomer/conformer 1**

| Atomic N. | X | Y | Z |
|---|---|---|---|
| 7 | 1.88783548 | 3.76905857 | -0.50545891 |
| 8 | -0.32269219 | 1.85586638 | -0.63448591 |
| 8 | 2.80545232 | 0.94442696 | -0.25936867 |
| 6 | 1.45043913 | 4.92308441 | 0.31704241 |
| 6 | -0.93810321 | 2.87607097 | -1.27462457 |
| 6 | -2.36387716 | 2.88874808 | -1.35280447 |
| 6 | -2.96335061 | 3.94875818 | -2.04721085 |
| 6 | -2.22719669 | 4.97196278 | -2.65085840 |
| 6 | -0.83942275 | 4.94603750 | -2.57087343 |
| 6 | -0.18971984 | 3.90458755 | -1.89959131 |
| 6 | 1.31252438 | 3.85441880 | -1.88857379 |
| 1 | -2.73865052 | 5.77862450 | -3.17594129 |
| 6 | -3.21819601 | 1.78192966 | -0.70332463 |
| 6 | 3.64735997 | 1.31527291 | -1.25637628 |
| 6 | 4.23932616 | 0.32380491 | -2.09685018 |
| 6 | 5.12233760 | 0.76458370 | -3.09293699 |
| 6 | 5.43101062 | 2.11251992 | -3.29144854 |
| 6 | 4.84349734 | 3.06866122 | -2.47153637 |
| 6 | 3.96393594 | 2.68293268 | -1.45378364 |
| 6 | 3.38849744 | 3.73582417 | -0.54603225 |
| 1 | 6.11979162 | 2.40632230 | -4.08330630 |
| 6 | 3.93026825 | -1.17685606 | -1.92795578 |

| | | | |
|---|---|---|---|
| 6 | -4.72438493 | 2.00247487 | -0.93913089 |
| 6 | -2.99046473 | 1.76388357 | 0.82663269 |
| 6 | -2.85458029 | 0.40651260 | -1.31223807 |
| 6 | 4.67796761 | -2.04519410 | -2.95753009 |
| 6 | 2.41807163 | -1.43620661 | -2.12439601 |
| 6 | 4.37851576 | -1.64624723 | -0.52475412 |
| 1 | 1.81045982 | 5.86379541 | -0.13258742 |
| 1 | 0.35712805 | 4.94664989 | 0.36794650 |
| 1 | 1.72199789 | 4.74853823 | -2.39020308 |
| 1 | 1.68203647 | 2.97357037 | -2.43463333 |
| 1 | -4.04873008 | 3.98583623 | -2.11951440 |
| 1 | -0.24221525 | 5.73126436 | -3.04005092 |
| 1 | 3.71439362 | 3.58005850 | 0.49537685 |
| 1 | 3.75154692 | 4.72907403 | -0.86197732 |
| 1 | 5.58625728 | 0.03012578 | -3.74851338 |
| 1 | 5.06770856 | 4.12890403 | -2.60811421 |
| 1 | -5.28363798 | 1.18447323 | -0.46260447 |
| 1 | -5.07977451 | 2.94507917 | -0.49852109 |
| 1 | -4.98087346 | 1.99922330 | -2.00838405 |
| 1 | -1.94401180 | 1.56272043 | 1.07894385 |
| 1 | -3.27569492 | 2.72771742 | 1.27250513 |
| 1 | -3.61739406 | 0.98420544 | 1.28729220 |
| 1 | -3.05024177 | 0.39966152 | -2.39454231 |
| 1 | -1.79978650 | 0.16248918 | -1.14601032 |
| 1 | -3.47817854 | -0.37808683 | -0.85461928 |
| 1 | 4.42226827 | -3.10071624 | -2.78626826 |
| 1 | 5.76953977 | -1.95122000 | -2.86580718 |
| 1 | 4.39242112 | -1.79987816 | -3.99047463 |
| 1 | 1.81130468 | -0.88160111 | -1.40145377 |
| 1 | 2.10510467 | -1.14029342 | -3.13615713 |
| 1 | 2.20696611 | -2.51077618 | -2.00886340 |
| 1 | 5.46688763 | -1.53860016 | -0.40982400 |
| 1 | 3.89054303 | -1.06026270 | 0.26118263 |
| 1 | 4.12807014 | -2.70928001 | -0.38484529 |
| 1 | 1.84716991 | 4.81470673 | 1.33092647 |
| 8 | 0.45840103 | -0.19922801 | 0.86053203 |
| 8 | 1.26513508 | 2.35206587 | 2.09746028 |
| 31 | 1.28559926 | 1.90128144 | 0.30530043 |
| 8 | 1.49566974 | -0.20323637 | 3.86580467 |
| 6 | 1.66160259 | 1.15023270 | 4.11778837 |
| 6 | 2.29115018 | 2.05656810 | 3.03593851 |
| 6 | 0.76041121 | -1.00551471 | 1.73955896 |
| 6 | 1.97428048 | -0.85713060 | 2.66129525 |
| 8 | 1.25732724 | 1.55653419 | 5.18246304 |
| 6 | 2.61295624 | -2.17325741 | 3.07422755 |
| 6 | 2.81612983 | 3.32802468 | 3.69837488 |
| 1 | 3.14824506 | 1.53812724 | 2.56474238 |
| 1 | 2.69277577 | -0.22702565 | 2.12357298 |
| 1 | 3.49491180 | -1.96928628 | 3.69469348 |
| 1 | 2.93244972 | -2.73950913 | 2.18911191 |
| 1 | 1.90641932 | -2.78178824 | 3.64952336 |
| 1 | 3.54711078 | 3.08624484 | 4.48258939 |
| 1 | 1.98837846 | 3.88076231 | 4.15781327 |

| | | | |
|---|---|---|---|
| 1 | 3.31032781 | 3.96157300 | 2.94894736 |
| 8 | -7.12180647 | -6.66714526 | 0.41324439 |
| 6 | -7.85399626 | -7.87909019 | 0.71799092 |
| 1 | -7.28004683 | -8.76179856 | 0.40925529 |
| 6 | -5.22094102 | -5.27099224 | 0.64219913 |
| 6 | -3.89683119 | -5.05171106 | 1.37289870 |
| 6 | -3.20088928 | -3.75653209 | 0.94201542 |
| 6 | -5.91124101 | -6.56387531 | 1.03423037 |
| 6 | -1.87892589 | -3.52528375 | 1.68542736 |
| 6 | -1.20465365 | -2.24518437 | 1.22844013 |
| 1 | -5.07167540 | -5.29172807 | -0.45010424 |
| 1 | -3.23498310 | -5.91192679 | 1.19092841 |
| 1 | -3.01316235 | -3.78370897 | -0.14514698 |
| 1 | -1.20064905 | -4.37644955 | 1.51630534 |
| 1 | -5.92244113 | -4.44168698 | 0.82661268 |
| 1 | -4.07893136 | -5.03486914 | 2.45844077 |
| 1 | -3.87407295 | -2.89981775 | 1.11687537 |
| 1 | -2.06426383 | -3.47071526 | 2.76946585 |
| 1 | -1.82784015 | -1.35783039 | 1.40521215 |
| 1 | -0.93750640 | -2.26946151 | 0.16273108 |
| 8 | -5.46886307 | -7.40821927 | 1.78974809 |
| 8 | 0.03102805 | -2.08914915 | 2.00690150 |
| 1 | -8.78612182 | -7.80413386 | 0.14998187 |
| 1 | -8.05792483 | -7.94337008 | 1.79412056 |

Electronic energy = -4057.847832 a.u.
DFT-D3(BJ) dispersion correction = -0.138465 a.u.
Thermal free energy = 0.721930 a.u.
Gibbs free energy = -4057.264367 a.u.
Number of imaginary frequencies = 0.

**Int4 LA isomer/conformer 1**

| Atomic N. | X | Y | Z |
|---|---|---|---|
| 8 | 1.60209775 | 2.25692691 | 2.64664033 |
| 8 | -2.62636350 | -0.51831698 | 3.40458642 |
| 7 | -3.42813899 | -2.79692069 | 1.76568362 |
| 8 | -0.71670941 | -1.83892282 | 1.05982424 |
| 8 | -3.35319640 | -0.20508294 | 0.31324344 |
| 6 | -3.45948807 | -3.37306935 | 3.13231126 |
| 6 | -0.40484129 | -3.14690005 | 1.20149798 |
| 6 | 0.95680218 | -3.52764832 | 1.40398377 |
| 6 | 1.23415427 | -4.89585995 | 1.53216018 |
| 6 | 0.24310558 | -5.87941695 | 1.47060214 |
| 6 | -1.07754845 | -5.49756851 | 1.26171192 |
| 6 | -1.40866071 | -4.14623103 | 1.11437991 |
| 6 | -2.82675867 | -3.76465129 | 0.78865060 |
| 1 | 0.50682628 | -6.93116345 | 1.58206042 |
| 6 | 2.08425303 | -2.47932248 | 1.48110779 |
| 6 | -4.23428546 | -0.81564017 | -0.50398458 |
| 6 | -4.44182887 | -0.30998624 | -1.82343595 |
| 6 | -5.38034487 | -0.97050474 | -2.62783902 |
| 6 | -6.10081280 | -2.08659289 | -2.19144916 |
| 6 | -5.89194156 | -2.56802463 | -0.90383454 |

| | | | |
|---|---|---|---|
| 6 | -4.97604644 | -1.93761730 | -0.05378903 |
| 6 | -4.83036398 | -2.42330822 | 1.36341187 |
| 1 | -6.81564866 | -2.57122805 | -2.85640278 |
| 6 | -3.66210887 | 0.91252388 | -2.34701403 |
| 6 | 3.46731663 | -3.13023738 | 1.67176428 |
| 6 | 1.84945131 | -1.53525658 | 2.68303225 |
| 6 | 2.13949079 | -1.66134140 | 0.16855353 |
| 6 | -4.06780220 | 1.28209961 | -3.78623274 |
| 6 | -2.14454815 | 0.61076294 | -2.35892017 |
| 6 | -3.94708653 | 2.14670106 | -1.45799194 |
| 1 | -4.07977990 | -4.28531600 | 3.14036204 |
| 1 | -2.44268160 | -3.62830418 | 3.44816907 |
| 1 | -3.45991177 | -4.66813236 | 0.75097011 |
| 1 | -2.88430808 | -3.28071262 | -0.19777455 |
| 1 | 2.26305167 | -5.21329112 | 1.69193334 |
| 1 | -1.86780715 | -6.24903640 | 1.19551872 |
| 1 | -5.14328480 | -1.64664935 | 2.08020134 |
| 1 | -5.47825981 | -3.30369208 | 1.51616544 |
| 1 | -5.55692237 | -0.60897880 | -3.63936538 |
| 1 | -6.44784186 | -3.43524683 | -0.53984360 |
| 1 | 4.23029865 | -2.33967291 | 1.71535457 |
| 1 | 3.53149459 | -3.70110349 | 2.60936431 |
| 1 | 3.72862459 | -3.79899139 | 0.83874030 |
| 1 | 0.89490796 | -1.00745933 | 2.59288850 |
| 1 | 1.85085372 | -2.10277844 | 3.62536290 |
| 1 | 2.64988088 | -0.78240407 | 2.73751496 |
| 1 | 2.33222235 | -2.32099875 | -0.69025512 |
| 1 | 1.20210929 | -1.12287879 | -0.00811213 |
| 1 | 2.96294899 | -0.93168634 | 0.22411410 |
| 1 | -3.48923698 | 2.16051493 | -4.10636439 |
| 1 | -5.13366950 | 1.54186092 | -3.86223779 |
| 1 | -3.85595001 | 0.47060313 | -4.49732840 |
| 1 | -1.76858651 | 0.38906355 | -1.35468749 |
| 1 | -1.92612247 | -0.24779187 | -3.01045018 |
| 1 | -1.59582909 | 1.48086977 | -2.75168174 |
| 1 | -5.01716477 | 2.40033851 | -1.47905636 |
| 1 | -3.65203080 | 1.96331138 | -0.41896481 |
| 1 | -3.38652599 | 3.01524210 | -1.83749984 |
| 1 | -3.87013860 | -2.62958198 | 3.82281390 |
| 31 | -2.30245441 | -1.02664234 | 1.63505049 |
| 6 | 1.01407905 | 2.87828542 | 1.78696204 |
| 6 | -0.41220989 | 3.43399963 | 1.91589707 |
| 8 | -1.11693736 | 2.72055784 | 2.97492330 |
| 6 | -1.45666606 | 1.45628176 | 2.71331278 |
| 6 | -2.19675938 | 0.73959085 | 3.83687405 |
| 8 | -1.21685579 | 0.90023203 | 1.63695987 |
| 6 | -1.30045784 | 0.63651165 | 5.08358486 |
| 6 | -0.40655542 | 4.90551440 | 2.30833182 |
| 1 | -0.94193110 | 3.26982950 | 0.96806113 |
| 1 | -3.06765007 | 1.38289219 | 4.08325448 |
| 1 | -1.86091451 | 0.11433183 | 5.86887711 |
| 1 | -1.01011119 | 1.62987422 | 5.44974797 |
| 1 | -0.39561907 | 0.05895997 | 4.85403427 |

| | | | |
|---|---|---|---|
| 1 | 0.10224024 | 5.49019532 | 1.53198999 |
| 1 | 0.11503460 | 5.04846956 | 3.26339739 |
| 1 | -1.43598221 | 5.27220725 | 2.40561682 |
| 8 | 8.06815812 | 2.48388599 | -4.99956681 |
| 6 | 8.56482380 | 2.84712765 | -6.31058184 |
| 1 | 7.85250655 | 2.54363374 | -7.08791466 |
| 6 | 6.45302640 | 2.63306604 | -3.27160692 |
| 6 | 5.04991409 | 3.10900568 | -2.89718632 |
| 6 | 4.66126823 | 2.71282240 | -1.46900025 |
| 6 | 6.87551419 | 3.05721705 | -4.66550731 |
| 6 | 3.24520842 | 3.17017324 | -1.09732965 |
| 6 | 2.88904851 | 2.78346993 | 0.32679862 |
| 1 | 6.53662702 | 1.53681672 | -3.20651041 |
| 1 | 4.32193756 | 2.69230490 | -3.61028301 |
| 1 | 4.73284098 | 1.61735826 | -1.35742511 |
| 1 | 2.51470500 | 2.71804156 | -1.78623654 |
| 1 | 7.20366342 | 3.02769622 | -2.56620425 |
| 1 | 4.99444868 | 4.20196192 | -3.01243716 |
| 1 | 5.38776052 | 3.14413507 | -0.75854397 |
| 1 | 3.16246481 | 4.26267938 | -1.21228248 |
| 1 | 3.54999664 | 3.26215303 | 1.06448196 |
| 1 | 2.93270433 | 1.69659119 | 0.48366366 |
| 8 | 6.26185446 | 3.80769566 | -5.40011287 |
| 8 | 1.51728879 | 3.22517212 | 0.58114836 |
| 1 | 9.51148320 | 2.30954995 | -6.42020440 |
| 1 | 8.72319218 | 3.93105207 | -6.37419794 |

Electronic energy = -4057.876632 a.u.
DFT-D3(BJ) dispersion correction = -0.133123 a.u.
Thermal free energy = 0.719350 a.u.
Gibbs free energy = -4057.290405 a.u.
Number of imaginary frequencies = 0.

**Int4 LA isomer/conformer 2**

| Atomic N. | X | Y | Z |
|---|---|---|---|
| 8 | -0.79318909 | 5.41899493 | 2.88573240 |
| 8 | 0.51210091 | -0.30055694 | 1.92758162 |
| 7 | 0.08220709 | -2.66772337 | 0.27026167 |
| 8 | 2.44475453 | -1.13725421 | -0.64448896 |
| 8 | -0.54238980 | -0.08497924 | -1.06031469 |
| 6 | 0.31466855 | -3.27154747 | 1.60569446 |
| 6 | 3.03915795 | -2.35004605 | -0.61414926 |
| 6 | 4.46517276 | -2.43325567 | -0.60176098 |
| 6 | 5.03772888 | -3.71225445 | -0.56020803 |
| 6 | 4.27561409 | -4.88383718 | -0.54058983 |
| 6 | 2.88872510 | -4.79039401 | -0.58307102 |
| 6 | 2.26513254 | -3.53909150 | -0.63425905 |
| 6 | 0.77213110 | -3.46151310 | -0.80002763 |
| 1 | 4.76643957 | -5.85636254 | -0.50243574 |
| 6 | 5.34743884 | -1.16974152 | -0.65185436 |
| 6 | -1.37086733 | -0.84309153 | -1.80460333 |
| 6 | -1.84420456 | -0.34400982 | -3.05729934 |
| 6 | -2.72332385 | -1.15618799 | -3.78660626 |

| | | | |
|---|---|---|---|
| 6 | -3.13837774 | -2.41451483 | -3.33948650 |
| 6 | -2.67200982 | -2.88802696 | -2.11759146 |
| 6 | -1.80303528 | -2.11260064 | -1.34113415 |
| 6 | -1.40168921 | -2.60724419 | 0.02326479 |
| 1 | -3.81751452 | -3.01467236 | -3.94526313 |
| 6 | -1.40202607 | 1.03273245 | -3.59149329 |
| 6 | 6.84894486 | -1.51294745 | -0.65591831 |
| 6 | 5.08228751 | -0.27919229 | 0.58437042 |
| 6 | 5.05183586 | -0.37048370 | -1.94354162 |
| 6 | -2.05349513 | 1.36155037 | -4.94788571 |
| 6 | 0.13114925 | 1.05778771 | -3.79719023 |
| 6 | -1.81627723 | 2.14651140 | -2.59980840 |
| 1 | -0.08263771 | -4.30028343 | 1.62437806 |
| 1 | 1.38872659 | -3.29789634 | 1.81734509 |
| 1 | 0.34214974 | -4.47807277 | -0.81600256 |
| 1 | 0.51423996 | -2.97940952 | -1.75505957 |
| 1 | 6.12216845 | -3.80496472 | -0.54101061 |
| 1 | 2.27255132 | -5.69249967 | -0.59369367 |
| 1 | -1.81468598 | -1.96053063 | 0.81224594 |
| 1 | -1.81882958 | -3.61576805 | 0.18298609 |
| 1 | -3.09648172 | -0.80296620 | -4.74639189 |
| 1 | -2.99128428 | -3.86318589 | -1.74313775 |
| 1 | 7.42821155 | -0.57962049 | -0.70257151 |
| 1 | 7.15551694 | -2.04794740 | 0.25484282 |
| 1 | 7.13103773 | -2.12082499 | -1.52756802 |
| 1 | 4.03981316 | 0.05503426 | 0.60610688 |
| 1 | 5.30224726 | -0.82760389 | 1.51262397 |
| 1 | 5.73181071 | 0.60899285 | 0.55161444 |
| 1 | 5.27317142 | -0.97931288 | -2.83222352 |
| 1 | 4.00405556 | -0.05438000 | -1.98809268 |
| 1 | 5.68938307 | 0.52624766 | -1.98069490 |
| 1 | -1.71071940 | 2.35272838 | -5.27767786 |
| 1 | -3.15112278 | 1.39428434 | -4.88433552 |
| 1 | -1.77231601 | 0.63909717 | -5.72764880 |
| 1 | 0.66805640 | 0.88875508 | -2.85819053 |
| 1 | 0.43635728 | 0.28617073 | -4.51881740 |
| 1 | 0.43664863 | 2.03616719 | -4.19858191 |
| 1 | -2.91000121 | 2.16319235 | -2.47572480 |
| 1 | -1.34918041 | 1.99485467 | -1.62051295 |
| 1 | -1.50380908 | 3.12719454 | -2.99061892 |
| 1 | -0.18704779 | -2.66230767 | 2.36373870 |
| 31 | 0.78689105 | -0.69662857 | 0.11260330 |
| 6 | -0.34946961 | 4.45477684 | 2.29202732 |
| 6 | 1.08026161 | 3.92926004 | 2.53159782 |
| 8 | 1.55710735 | 3.16813279 | 1.38574795 |
| 6 | 1.30937441 | 1.85846843 | 1.24093616 |
| 6 | 0.90876083 | 0.94553356 | 2.40804602 |
| 8 | 1.49134048 | 1.37272558 | 0.12307892 |
| 6 | 2.10368337 | 0.79764144 | 3.37776164 |
| 6 | 2.06625135 | 5.06493167 | 2.75548152 |
| 1 | 1.03424939 | 3.29033796 | 3.42559771 |
| 1 | 0.06325354 | 1.42132833 | 2.94331572 |
| 1 | 1.79916648 | 0.12295268 | 4.18725985 |

| | | | |
|---|---|---|---|
| 1 | 2.42990888 | 1.74979498 | 3.82054956 |
| 1 | 2.95379410 | 0.34568885 | 2.85070994 |
| 1 | 1.70039331 | 5.71261308 | 3.55982946 |
| 1 | 2.17088175 | 5.66390862 | 1.84224641 |
| 1 | 3.04854914 | 4.66120311 | 3.02952969 |
| 8 | -5.64323824 | -0.29845623 | 1.00908672 |
| 6 | -5.83743651 | -1.05707426 | -0.21371685 |
| 1 | -4.89909766 | -1.11750375 | -0.77857141 |
| 6 | -4.62977014 | -0.01500871 | 3.15153954 |
| 6 | -4.62674827 | 1.50740568 | 2.95623581 |
| 6 | -3.46361261 | 2.01743372 | 2.09751013 |
| 6 | -4.77005059 | -0.85244275 | 1.89208335 |
| 6 | -3.41399142 | 3.54850580 | 2.04765937 |
| 6 | -2.38631241 | 4.12055350 | 1.08173926 |
| 1 | -5.46616513 | -0.30011806 | 3.81160381 |
| 1 | -4.57805610 | 1.97661591 | 3.95280141 |
| 1 | -2.51344096 | 1.62589051 | 2.49553864 |
| 1 | -3.23954283 | 3.96283148 | 3.05286885 |
| 1 | -3.70835727 | -0.35393563 | 3.64442391 |
| 1 | -5.58127282 | 1.82264502 | 2.50864122 |
| 1 | -3.55490341 | 1.61900970 | 1.07386543 |
| 1 | -4.39354475 | 3.93787520 | 1.71945071 |
| 1 | -2.41173584 | 5.21763045 | 1.08316798 |
| 1 | -2.53743589 | 3.74072196 | 0.06350553 |
| 8 | -4.19809698 | -1.91108458 | 1.69845209 |
| 8 | -1.01697885 | 3.69761874 | 1.40145702 |
| 1 | -6.59359789 | -0.50230321 | -0.77709016 |
| 1 | -6.18613043 | -2.07067241 | 0.01817791 |

Electronic energy = -4057.864232 a.u.
DFT-D3(BJ) dispersion correction = -0.139786 a.u.
Thermal free energy = 0.720578 a.u.
Gibbs free energy = -4057.283440 a.u.
Number of imaginary frequencies = 0.

**Int4 LA isomer/conformer 3**

| Atomic N. | X | Y | Z |
|---|---|---|---|
| 8 | -1.00946043 | 5.41637852 | 2.60330602 |
| 8 | 0.23259824 | -0.28251853 | 1.66416076 |
| 7 | -0.26785238 | -2.65842080 | -0.01643257 |
| 8 | 2.16034087 | -1.18596899 | -0.87076394 |
| 8 | -0.80083887 | -0.05259052 | -1.33766114 |
| 6 | -0.12234207 | -3.26624566 | 1.32999132 |
| 6 | 2.73387990 | -2.40692548 | -0.79206644 |
| 6 | 4.15670505 | -2.51644066 | -0.73252016 |
| 6 | 4.70142467 | -3.80464684 | -0.63385048 |
| 6 | 3.91577958 | -4.96042866 | -0.60666812 |
| 6 | 2.53342740 | -4.84239455 | -0.70462630 |
| 6 | 1.93820309 | -3.58114964 | -0.81241822 |
| 6 | 0.45679883 | -3.47483179 | -1.04652899 |
| 1 | 4.38562641 | -5.94045502 | -0.52307057 |
| 6 | 5.06591287 | -1.27312219 | -0.80048762 |
| 6 | -1.57802273 | -0.79741526 | -2.15123594 |

| | | | |
|---|---|---|---|
| 6 | -1.95767127 | -0.28561985 | -3.42910496 |
| 6 | -2.78014643 | -1.09148189 | -4.22855013 |
| 6 | -3.22695026 | -2.35326619 | -3.82478240 |
| 6 | -2.85406098 | -2.83795410 | -2.57573869 |
| 6 | -2.04576766 | -2.06848226 | -1.73103065 |
| 6 | -1.73630847 | -2.57096952 | -0.34658573 |
| 1 | -3.85873064 | -2.94750761 | -4.48505245 |
| 6 | -1.48006533 | 1.09681509 | -3.91589245 |
| 6 | 6.55953578 | -1.64520676 | -0.74940657 |
| 6 | 4.78423687 | -0.33178758 | 0.39328700 |
| 6 | 4.82154139 | -0.51636446 | -2.12807071 |
| 6 | -2.04021870 | 1.44319273 | -5.30821834 |
| 6 | 0.06331937 | 1.12177535 | -4.01900108 |
| 6 | -1.95855778 | 2.19916886 | -2.94034743 |
| 1 | -0.49556402 | -4.30389951 | 1.31039990 |
| 1 | 0.93252545 | -3.26958472 | 1.62355901 |
| 1 | 0.00643898 | -4.48235524 | -1.07225262 |
| 1 | 0.25268192 | -2.99717387 | -2.01662647 |
| 1 | 5.78256385 | -3.91759819 | -0.57559732 |
| 1 | 1.90021621 | -5.73261266 | -0.71620488 |
| 1 | -2.17564384 | -1.91998846 | 0.42440876 |
| 1 | -2.17494293 | -3.57462223 | -0.21387011 |
| 1 | -3.08203011 | -0.72942578 | -5.20983909 |
| 1 | -3.19806824 | -3.81772151 | -2.23630107 |
| 1 | 7.15886237 | -0.72562058 | -0.81279651 |
| 1 | 6.82962050 | -2.15282344 | 0.18821215 |
| 1 | 6.85333343 | -2.28959174 | -1.59038939 |
| 1 | 3.74924326 | 0.02459199 | 0.36947225 |
| 1 | 4.96452668 | -0.84954011 | 1.34743615 |
| 1 | 5.45382274 | 0.54081622 | 0.34770088 |
| 1 | 5.05558892 | -1.16105706 | -2.98769070 |
| 1 | 3.78139187 | -0.18347510 | -2.21325262 |
| 1 | 5.47645100 | 0.36704729 | -2.17958233 |
| 1 | -1.67762090 | 2.43887073 | -5.60137386 |
| 1 | -3.13961888 | 1.47421274 | -5.31705084 |
| 1 | -1.70665562 | 0.73142018 | -6.07708730 |
| 1 | 0.53684489 | 0.94125805 | -3.04861908 |
| 1 | 0.41531417 | 0.35810180 | -4.72765772 |
| 1 | 0.39592638 | 2.10426998 | -4.38758034 |
| 1 | -3.05794367 | 2.21647358 | -2.88917634 |
| 1 | -1.55790849 | 2.03477195 | -1.93403960 |
| 1 | -1.61963073 | 3.18383764 | -3.29773466 |
| 1 | -0.70566650 | -2.68261358 | 2.04857663 |
| 31 | 0.49522913 | -0.70517347 | -0.15140566 |
| 6 | -0.56708730 | 4.46196566 | 1.99282434 |
| 6 | 0.86753001 | 3.94388675 | 2.21681200 |
| 8 | 1.33337852 | 3.16384907 | 1.07994049 |
| 6 | 1.07104016 | 1.85503909 | 0.95028445 |
| 6 | 0.65867930 | 0.96212019 | 2.12826194 |
| 8 | 1.24947000 | 1.35297580 | -0.15980343 |
| 6 | 1.85813416 | 0.80304429 | 3.09054467 |
| 6 | 1.85256776 | 5.08747249 | 2.40485332 |
| 1 | 0.83720708 | 3.32234911 | 3.12371506 |

| | | | |
|---|---|---|---|
| 1 | -0.17345850 | 1.46094817 | 2.66346579 |
| 1 | 1.54882190 | 0.15114742 | 3.91702925 |
| 1 | 2.21063875 | 1.75571294 | 3.51156314 |
| 1 | 2.69225541 | 0.32170887 | 2.56440253 |
| 1 | 1.49618728 | 5.74859938 | 3.20244056 |
| 1 | 1.94142211 | 5.66901437 | 1.47874887 |
| 1 | 2.84043463 | 4.69239436 | 2.67135640 |
| 8 | -2.97421062 | -0.19764222 | 4.22766606 |
| 6 | -1.79689277 | -0.88292927 | 4.72468570 |
| 1 | -2.06331721 | -1.88078419 | 5.09477382 |
| 6 | -4.84241283 | -0.07484182 | 2.74503464 |
| 6 | -4.73501524 | 1.45623268 | 2.74515103 |
| 6 | -3.70121197 | 1.99948810 | 1.75227906 |
| 6 | -3.60689543 | -0.82980105 | 3.20082600 |
| 6 | -3.61715470 | 3.52908756 | 1.78742860 |
| 6 | -2.61795365 | 4.13131208 | 0.80965029 |
| 1 | -5.08953333 | -0.45882233 | 1.74593796 |
| 1 | -4.49338491 | 1.80536874 | 3.76026092 |
| 1 | -2.71247334 | 1.56930953 | 1.97610814 |
| 1 | -3.38155767 | 3.87978475 | 2.80439900 |
| 1 | -5.66121344 | -0.38767059 | 3.41471686 |
| 1 | -5.72751849 | 1.86857187 | 2.50028437 |
| 1 | -3.95531936 | 1.66726421 | 0.73160189 |
| 1 | -4.60142195 | 3.96040499 | 1.53492839 |
| 1 | -2.65229975 | 5.22767879 | 0.83542491 |
| 1 | -2.78464342 | 3.77168036 | -0.21356353 |
| 8 | -3.23093604 | -1.89292876 | 2.73827974 |
| 8 | -1.23936128 | 3.71388859 | 1.09785886 |
| 1 | -1.42354459 | -0.25642407 | 5.54088547 |
| 1 | -1.05057340 | -0.97638655 | 3.92467216 |

Electronic energy = -4057.864718 a.u.
DFT-D3(BJ) dispersion correction = -0.140754 a.u.
Thermal free energy = 0.722625 a.u.
Gibbs free energy = -4057.282847 a.u.
Number of imaginary frequencies = 0.

**Int4 LA isomer/conformer 4**

| Atomic N. | X | Y | Z |
|---|---|---|---|
| 8 | -1.70344386 | 1.61239116 | 1.21601895 |
| 8 | 1.03259332 | -1.58346465 | 0.84967891 |
| 7 | 1.49494831 | -3.35209377 | -1.40896515 |
| 8 | 3.39439297 | -1.07551616 | -1.42437524 |
| 8 | 0.30994806 | -0.72608228 | -2.13871698 |
| 6 | 1.72323836 | -4.25443361 | -0.25488532 |
| 6 | 4.31111840 | -2.05550760 | -1.57557077 |
| 6 | 5.68624147 | -1.78001106 | -1.30285258 |
| 6 | 6.60013777 | -2.83139425 | -1.45995835 |
| 6 | 6.22040489 | -4.11016306 | -1.87758408 |
| 6 | 4.88411628 | -4.35670138 | -2.17348269 |
| 6 | 3.93088162 | -3.34113111 | -2.04160674 |
| 6 | 2.51487722 | -3.59309870 | -2.48248938 |
| 1 | 6.96711510 | -4.89765148 | -1.98001623 |

| | | | |
|---|---|---|---|
| 6 | 6.15788535 | -0.37572250 | -0.87508319 |
| 6 | -0.13992549 | -1.40199714 | -3.21346652 |
| 6 | -0.55058667 | -0.68374401 | -4.37769554 |
| 6 | -1.02302058 | -1.43304194 | -5.46371102 |
| 6 | -1.10169644 | -2.82932688 | -5.44626286 |
| 6 | -0.70518066 | -3.51670984 | -4.30468581 |
| 6 | -0.23759226 | -2.81681544 | -3.18622626 |
| 6 | 0.09982181 | -3.57459617 | -1.93067967 |
| 1 | -1.46918512 | -3.36818921 | -6.31964978 |
| 6 | -0.47849482 | 0.85462765 | -4.43941445 |
| 6 | 7.68248546 | -0.31732205 | -0.66430821 |
| 6 | 5.49272740 | 0.03458209 | 0.45929080 |
| 6 | 5.80213863 | 0.65544729 | -1.97281500 |
| 6 | -0.98791648 | 1.39977267 | -5.78711000 |
| 6 | 0.98551159 | 1.32777608 | -4.27568606 |
| 6 | -1.36049935 | 1.47380598 | -3.32884917 |
| 1 | 1.63858288 | -5.30574073 | -0.57910387 |
| 1 | 2.72536656 | -4.08557624 | 0.15288444 |
| 1 | 2.41194668 | -4.63241034 | -2.84044570 |
| 1 | 2.24451552 | -2.92905256 | -3.31708001 |
| 1 | 7.65288942 | -2.65188865 | -1.24902733 |
| 1 | 4.56754518 | -5.34029761 | -2.52849631 |
| 1 | -0.57052118 | -3.28512196 | -1.10496346 |
| 1 | -0.03194709 | -4.65584095 | -2.10994064 |
| 1 | -1.33869653 | -0.91258429 | -6.36625398 |
| 1 | -0.76476016 | -4.60699373 | -4.26530932 |
| 1 | 7.96335044 | 0.70477072 | -0.37227041 |
| 1 | 8.01789044 | -0.99440392 | 0.13497155 |
| 1 | 8.23582677 | -0.56234975 | -1.58225450 |
| 1 | 4.40415971 | 0.08278887 | 0.35216741 |
| 1 | 5.74383885 | -0.68240375 | 1.25541642 |
| 1 | 5.85582657 | 1.02689534 | 0.76804818 |
| 1 | 6.30136046 | 0.39888840 | -2.91850567 |
| 1 | 4.72164195 | 0.69751657 | -2.14776395 |
| 1 | 6.14608573 | 1.65624844 | -1.66937254 |
| 1 | -0.92140325 | 2.49709299 | -5.77508221 |
| 1 | -2.03925812 | 1.13394794 | -5.97059098 |
| 1 | -0.38513549 | 1.04154359 | -6.63444988 |
| 1 | 1.40143147 | 1.02997746 | -3.30786848 |
| 1 | 1.61903531 | 0.91118602 | -5.07254771 |
| 1 | 1.03020060 | 2.42532320 | -4.34684603 |
| 1 | -2.41206893 | 1.17925684 | -3.46172640 |
| 1 | -1.03386841 | 1.16145167 | -2.33151010 |
| 1 | -1.30814665 | 2.57202233 | -3.38418896 |
| 1 | 0.98185658 | -4.03696879 | 0.52032978 |
| 31 | 1.60334584 | -1.30431741 | -0.91725623 |
| 6 | -1.29085673 | 1.95057727 | 2.30457769 |
| 6 | 0.18729092 | 2.21562099 | 2.64647356 |
| 8 | 0.99398374 | 2.02817883 | 1.45909422 |
| 6 | 1.18606476 | 0.81060515 | 0.92469727 |
| 6 | 0.98254881 | -0.49046193 | 1.71245073 |
| 8 | 1.63571885 | 0.76457231 | -0.22101827 |
| 6 | 2.08250738 | -0.60916613 | 2.79334377 |

| | | | |
|---|---|---|---|
| 6 | 0.43394811 | 3.64849885 | 3.10672512 |
| 1 | 0.48484430 | 1.51160404 | 3.43680544 |
| 1 | -0.00935913 | -0.45115935 | 2.20258460 |
| 1 | 1.92904236 | -1.55575793 | 3.32598223 |
| 1 | 2.07029583 | 0.20945828 | 3.52724157 |
| 1 | 3.06964887 | -0.63641515 | 2.31436427 |
| 1 | -0.15785587 | 3.85837143 | 4.00485014 |
| 1 | 0.15252286 | 4.35618813 | 2.31675074 |
| 1 | 1.49738108 | 3.78526656 | 3.33849804 |
| 8 | -9.86981214 | 2.73547830 | 4.63860571 |
| 6 | -10.62435212 | 3.78586753 | 3.98476817 |
| 1 | -10.72182135 | 4.65598099 | 4.64592773 |
| 6 | -7.91311210 | 1.93976665 | 5.71799275 |
| 6 | -6.39407530 | 2.10514175 | 5.80463390 |
| 6 | -5.68475442 | 1.92154658 | 4.45797758 |
| 6 | -8.60057990 | 3.08325125 | 4.99200133 |
| 6 | -4.16656567 | 2.11103884 | 4.56547562 |
| 6 | -3.48620828 | 1.91479729 | 3.22292006 |
| 1 | -8.35386384 | 1.90171819 | 6.72807946 |
| 1 | -6.00162450 | 1.37307387 | 6.52830951 |
| 1 | -5.90522318 | 0.91450035 | 4.06360223 |
| 1 | -3.74709637 | 1.39794921 | 5.29331924 |
| 1 | -8.19430620 | 0.99289330 | 5.23287487 |
| 1 | -6.16742373 | 3.10548658 | 6.20344280 |
| 1 | -6.09183104 | 2.64448862 | 3.73348110 |
| 1 | -3.94563349 | 3.12288600 | 4.94003236 |
| 1 | -3.84795511 | 2.62814538 | 2.46886144 |
| 1 | -3.63069798 | 0.89927759 | 2.82742736 |
| 8 | -8.11556779 | 4.17683263 | 4.76749015 |
| 8 | -2.04911518 | 2.13512321 | 3.40993451 |
| 1 | -11.60405147 | 3.34786058 | 3.77217782 |
| 1 | -10.12598841 | 4.09426564 | 3.05743850 |

Electronic energy = -4057.862392 a.u.
DFT-D3(BJ) dispersion correction = -0.132447 a.u.
Thermal free energy = 0.719537 a.u.
Gibbs free energy = -4057.275303 a.u.
Number of imaginary frequencies = 0.

**Int4 LA isomer/conformer 5**

| Atomic N. | X | Y | Z |
|---|---|---|---|
| 7 | 1.36598342 | 2.81997441 | 2.12694300 |
| 8 | -0.66197384 | 3.15128904 | -0.01065429 |
| 8 | 2.29398886 | 3.00370279 | -0.75428346 |
| 6 | 1.21642066 | 1.77384333 | 3.17218571 |
| 6 | -1.52251716 | 3.30764508 | 1.02016796 |
| 6 | -2.92872199 | 3.16412533 | 0.83155947 |
| 6 | -3.74596224 | 3.33525466 | 1.95874428 |
| 6 | -3.24189439 | 3.64389148 | 3.22629714 |
| 6 | -1.87082993 | 3.81565534 | 3.39013487 |
| 6 | -1.01341357 | 3.66501194 | 2.29589433 |
| 6 | 0.44874978 | 3.98465629 | 2.41810944 |
| 1 | -3.92107439 | 3.76082924 | 4.07084661 |

| | | | |
|---|---|---|---|
| 6 | -3.52853990 | 2.83962647 | -0.55003664 |
| 6 | 2.85496885 | 4.16011817 | -0.32121577 |
| 6 | 3.23987848 | 5.15507145 | -1.26866015 |
| 6 | 3.82256245 | 6.32916082 | -0.77164499 |
| 6 | 4.02992489 | 6.55339551 | 0.59191380 |
| 6 | 3.65791441 | 5.57300121 | 1.50428734 |
| 6 | 3.08588847 | 4.37292580 | 1.06435512 |
| 6 | 2.80344825 | 3.29229640 | 2.07972306 |
| 1 | 4.47853247 | 7.48674126 | 0.93190080 |
| 6 | 3.02606952 | 4.95512827 | -2.78263354 |
| 6 | -5.06803262 | 2.80206833 | -0.51519100 |
| 6 | -3.04151915 | 1.45171410 | -1.02836859 |
| 6 | -3.11800243 | 3.92515023 | -1.57366617 |
| 6 | 3.53794002 | 6.15603219 | -3.60083669 |
| 6 | 1.51895333 | 4.79889375 | -3.09493577 |
| 6 | 3.80336950 | 3.70845927 | -3.26762210 |
| 1 | 1.47740890 | 2.19669187 | 4.15588597 |
| 1 | 0.17681188 | 1.42896752 | 3.19455870 |
| 1 | 0.68180606 | 4.34864324 | 3.43282956 |
| 1 | 0.72797541 | 4.77034933 | 1.70145310 |
| 1 | -4.82325118 | 3.22101522 | 1.85012772 |
| 1 | -1.45686417 | 4.08941697 | 4.36357875 |
| 1 | 3.40309235 | 2.39550318 | 1.87702543 |
| 1 | 3.06366084 | 3.66156243 | 3.08567135 |
| 1 | 4.12147122 | 7.10733284 | -1.47146192 |
| 1 | 3.82091085 | 5.72337915 | 2.57397855 |
| 1 | -5.44235417 | 2.58640666 | -1.52626243 |
| 1 | -5.44906186 | 2.01529636 | 0.15217801 |
| 1 | -5.49830972 | 3.76399070 | -0.20042445 |
| 1 | -1.95010460 | 1.40536985 | -1.10615360 |
| 1 | -3.36907272 | 0.66721891 | -0.33018421 |
| 1 | -3.47366922 | 1.22810977 | -2.01636441 |
| 1 | -3.49582308 | 4.91077360 | -1.26439351 |
| 1 | -2.02920870 | 3.98798062 | -1.67661623 |
| 1 | -3.55170863 | 3.68907676 | -2.55750039 |
| 1 | 3.37050104 | 5.95633488 | -4.66890413 |
| 1 | 4.61561017 | 6.32421691 | -3.46073352 |
| 1 | 3.00456788 | 7.08471557 | -3.35162344 |
| 1 | 1.08507987 | 3.93191808 | -2.58641598 |
| 1 | 0.96410813 | 5.69475684 | -2.78110238 |
| 1 | 1.37565028 | 4.67419943 | -4.17922475 |
| 1 | 4.88315826 | 3.83770691 | -3.10319829 |
| 1 | 3.47900305 | 2.80484465 | -2.74066258 |
| 1 | 3.64025023 | 3.56381176 | -4.34663469 |
| 1 | 1.87886990 | 0.93351714 | 2.94393581 |
| 8 | 1.28221646 | -2.96031256 | 1.82177977 |
| 8 | 0.37519054 | 0.50367287 | -0.27246406 |
| 31 | 0.93517398 | 2.18565561 | 0.21736886 |
| 6 | 1.60649865 | -3.46962863 | 0.76974934 |
| 6 | 2.74460002 | -2.98007147 | -0.14294370 |
| 8 | 3.23483015 | -1.70417298 | 0.32391306 |
| 6 | 2.47117014 | -0.58331461 | 0.24345306 |
| 6 | 1.24252805 | -0.51752382 | -0.67247155 |

| | | | |
|---|---|---|---|
| 8 | 2.84257971 | 0.40397766 | 0.85746080 |
| 6 | 1.72571405 | -0.32463044 | -2.12687305 |
| 6 | 3.93557417 | -3.93485303 | -0.10942535 |
| 1 | 2.35928557 | -2.90268946 | -1.16989335 |
| 1 | 0.68012000 | -1.46498270 | -0.60175515 |
| 1 | 0.84353327 | -0.25091224 | -2.77479869 |
| 1 | 2.35563943 | -1.15385251 | -2.48123613 |
| 1 | 2.30006767 | 0.60828029 | -2.20638244 |
| 1 | 3.62898062 | -4.92878745 | -0.45615047 |
| 1 | 4.33110945 | -4.01361056 | 0.91117755 |
| 1 | 4.72939378 | -3.55720789 | -0.76559423 |
| 8 | -5.13730748 | -10.58643663 | 0.81589830 |
| 6 | -5.72890133 | -11.75113712 | 0.18990386 |
| 1 | -6.06495586 | -11.51067341 | -0.82643516 |
| 6 | -3.49662789 | -8.87910562 | 0.90491749 |
| 6 | -2.33097687 | -8.21118389 | 0.17672452 |
| 6 | -1.77207283 | -7.01021486 | 0.94684170 |
| 6 | -4.04767760 | -10.08462028 | 0.16648049 |
| 6 | -0.60885553 | -6.33212116 | 0.21192089 |
| 6 | -0.06615093 | -5.14950169 | 0.99387567 |
| 1 | -4.32544426 | -8.17193398 | 1.06836617 |
| 1 | -2.66222938 | -7.89187412 | -0.82338469 |
| 1 | -2.57700170 | -6.27503340 | 1.11806333 |
| 1 | -0.94397191 | -5.98836985 | -0.77934284 |
| 1 | -3.19459160 | -9.21520694 | 1.91078096 |
| 1 | -1.53524664 | -8.95259776 | 0.00863533 |
| 1 | -1.43598033 | -7.33815679 | 1.94563409 |
| 1 | 0.20071628 | -7.05961279 | 0.04264176 |
| 1 | 0.32872535 | -5.44791651 | 1.97578032 |
| 1 | -0.82866979 | -4.37414202 | 1.15572216 |
| 8 | -3.59767889 | -10.55943153 | -0.85898625 |
| 8 | 1.02717003 | -4.56185172 | 0.21682308 |
| 1 | -6.57729618 | -12.02126200 | 0.82575287 |
| 1 | -5.00180036 | -12.57142126 | 0.14179592 |

Electronic energy = -4057.857380 a.u.
DFT-D3(BJ) dispersion correction = -0.132279 a.u.
Thermal free energy = 0.719160 a.u.
Gibbs free energy = -4057.270499 a.u.
Number of imaginary frequencies = 0.

**Int4 LA isomer/conformer 6**

| Atomic N. | X | Y | Z |
|---|---|---|---|
| 7 | 1.54533121 | 4.25500178 | -0.96436505 |
| 8 | 1.24473336 | 2.32394675 | 1.30406533 |
| 8 | -0.02623746 | 1.70883073 | -1.42161846 |
| 6 | 2.90582685 | 4.76008572 | -1.28307221 |
| 6 | 1.75631465 | 3.35516762 | 2.03348957 |
| 6 | 2.28728218 | 3.11435194 | 3.33312670 |
| 6 | 2.81427257 | 4.21749096 | 4.02147479 |
| 6 | 2.81922815 | 5.51231103 | 3.49587223 |
| 6 | 2.25337925 | 5.74053482 | 2.24671789 |
| 6 | 1.70672939 | 4.67754359 | 1.51895924 |

| | | | |
|---|---|---|---|
| 6 | 0.96999373 | 4.95989752 | 0.23672190 |
| 1 | 3.25074754 | 6.33341086 | 4.06830513 |
| 6 | 2.26656948 | 1.71328908 | 3.97578001 |
| 6 | -1.04348423 | 2.57908906 | -1.64006320 |
| 6 | -2.39448781 | 2.12826188 | -1.56628784 |
| 6 | -3.40376491 | 3.06868675 | -1.81587127 |
| 6 | -3.13562725 | 4.40484566 | -2.12550788 |
| 6 | -1.81506683 | 4.83095702 | -2.20458923 |
| 6 | -0.76664896 | 3.93096961 | -1.97834069 |
| 6 | 0.65189623 | 4.40291478 | -2.17351438 |
| 1 | -3.95509695 | 5.10093898 | -2.30344086 |
| 6 | -2.73993569 | 0.66599183 | -1.22260033 |
| 6 | 2.86272052 | 1.72646105 | 5.39626082 |
| 6 | 3.09286889 | 0.71369883 | 3.13605134 |
| 6 | 0.80489875 | 1.21750933 | 4.09265963 |
| 6 | -4.26010525 | 0.41608525 | -1.23221191 |
| 6 | -2.22553007 | 0.31625268 | 0.19373201 |
| 6 | -2.11304369 | -0.28521517 | -2.26968479 |
| 1 | 2.84896384 | 5.82081666 | -1.57631758 |
| 1 | 3.54652850 | 4.65971503 | -0.40056750 |
| 1 | 0.96206698 | 6.04351418 | 0.03205758 |
| 1 | -0.07446799 | 4.62252133 | 0.30989769 |
| 1 | 3.24431289 | 4.06342599 | 5.00922667 |
| 1 | 2.21193861 | 6.75009348 | 1.83136332 |
| 1 | 1.14826177 | 3.83028286 | -2.97346036 |
| 1 | 0.65004660 | 5.46518029 | -2.46905203 |
| 1 | -4.44414784 | 2.75388828 | -1.76048521 |
| 1 | -1.58241027 | 5.86805320 | -2.45653302 |
| 1 | 2.80389870 | 0.71128844 | 5.81362292 |
| 1 | 3.92213631 | 2.02149156 | 5.39746426 |
| 1 | 2.31205579 | 2.39538037 | 6.07386192 |
| 1 | 2.65581141 | 0.55721414 | 2.14616685 |
| 1 | 4.12699247 | 1.06079956 | 3.00542005 |
| 1 | 3.12254407 | -0.25963495 | 3.64887876 |
| 1 | 0.21237808 | 1.89456574 | 4.72574815 |
| 1 | 0.32593052 | 1.14613820 | 3.10984471 |
| 1 | 0.79037247 | 0.21975180 | 4.55701956 |
| 1 | -4.44878273 | -0.64070917 | -0.99437840 |
| 1 | -4.70773110 | 0.61871053 | -2.21590572 |
| 1 | -4.78451816 | 1.02112720 | -0.47879501 |
| 1 | -1.13604882 | 0.39718246 | 0.26722110 |
| 1 | -2.67268335 | 0.98341654 | 0.94442842 |
| 1 | -2.51383590 | -0.71529630 | 0.44954326 |
| 1 | -2.50331249 | -0.06714789 | -3.27449910 |
| 1 | -1.02164217 | -0.19248244 | -2.29278437 |
| 1 | -2.37492474 | -1.32776490 | -2.02964390 |
| 1 | 3.32963295 | 4.16306875 | -2.09748296 |
| 8 | 1.71013633 | -1.51739477 | 0.46532584 |
| 8 | 3.02251208 | 1.68011966 | -1.36807936 |
| 31 | 1.51392988 | 2.26508338 | -0.52886772 |
| 8 | 4.34764836 | -1.60173312 | -0.36528282 |
| 6 | 4.25751412 | -0.22922986 | -0.52170043 |
| 6 | 3.35500882 | 0.33708036 | -1.64029866 |

| | | | |
|---|---|---|---|
| 6 | 2.04539179 | -2.41362485 | -0.28351118 |
| 6 | 3.42335883 | -2.52485128 | -0.96279785 |
| 8 | 4.97508249 | 0.46045720 | 0.16045193 |
| 6 | 4.04835006 | -3.91119839 | -0.81852117 |
| 6 | 4.15098589 | 0.29543864 | -2.95528132 |
| 1 | 2.45011157 | -0.28295552 | -1.74653503 |
| 1 | 3.27402972 | -2.30574723 | -2.03382107 |
| 1 | 5.02152582 | -3.92540035 | -1.32450094 |
| 1 | 3.39915151 | -4.67225439 | -1.26553042 |
| 1 | 4.20440497 | -4.14904689 | 0.24139073 |
| 1 | 4.48020819 | -0.72290341 | -3.20865044 |
| 1 | 5.03832430 | 0.93462933 | -2.85984488 |
| 1 | 3.52232851 | 0.67639620 | -3.77047217 |
| 8 | -6.62825792 | -6.97987008 | -0.34345777 |
| 6 | -7.47400452 | -8.05135028 | -0.82730790 |
| 1 | -7.55697369 | -8.01048360 | -1.92060802 |
| 6 | -4.54591178 | -5.85268279 | -0.25062511 |
| 6 | -3.11320412 | -5.79892645 | -0.77928223 |
| 6 | -2.31689385 | -4.63119413 | -0.18777594 |
| 6 | -5.34946479 | -7.00702212 | -0.81913043 |
| 6 | -0.87874399 | -4.57133524 | -0.71789503 |
| 6 | -0.10922942 | -3.40806451 | -0.11802680 |
| 1 | -5.08931606 | -4.92034564 | -0.47411588 |
| 1 | -3.13351734 | -5.71943295 | -1.87711975 |
| 1 | -2.83187427 | -3.68199357 | -0.41568192 |
| 1 | -0.88969335 | -4.47219386 | -1.81493600 |
| 1 | -4.56058421 | -5.94424253 | 0.84791238 |
| 1 | -2.60680715 | -6.75016156 | -0.55442313 |
| 1 | -2.30052044 | -4.71627832 | 0.91244025 |
| 1 | -0.35635314 | -5.51308676 | -0.48615811 |
| 1 | -0.03524786 | -3.48159691 | 0.97665870 |
| 1 | -0.56405533 | -2.43833153 | -0.36245300 |
| 8 | -4.93701071 | -7.85269384 | -1.58997450 |
| 8 | 1.24533886 | -3.43422703 | -0.67176624 |
| 1 | -8.44812953 | -7.88526972 | -0.35758906 |
| 1 | -7.06267865 | -9.02598216 | -0.53606609 |

Electronic energy = -4057.847019 a.u.
DFT-D3(BJ) dispersion correction = -0.134302 a.u.
Thermal free energy = 0.717861 a.u.
Gibbs free energy = -4057.263460 a.u.
Number of imaginary frequencies = 0.

**Int4 LA isomer/conformer 7**

| Atomic N. | X | Y | Z |
|---|---|---|---|
| 7 | 0.43043328 | 1.37333408 | 1.84774321 |
| 8 | -0.69846024 | 2.09558490 | -0.84035273 |
| 8 | 2.35656109 | 2.27880143 | -0.25681402 |
| 6 | -0.13096792 | 0.14103723 | 2.46092009 |
| 6 | -1.86266700 | 2.17905263 | -0.14129572 |
| 6 | -3.10952738 | 2.15635993 | -0.83297962 |
| 6 | -4.27527691 | 2.23599758 | -0.05720891 |
| 6 | -4.25586351 | 2.34210135 | 1.33594655 |

| | | | |
|---|---|---|---|
| 6 | -3.03303635 | 2.39509964 | 1.99494689 |
| 6 | -1.83775672 | 2.33082177 | 1.26968908 |
| 6 | -0.53018405 | 2.52896894 | 1.98773134 |
| 1 | -5.19039925 | 2.39527042 | 1.89416478 |
| 6 | -3.18650307 | 2.05700928 | -2.36946526 |
| 6 | 2.69129578 | 3.20875976 | 0.67365582 |
| 6 | 3.37250577 | 4.39613700 | 0.27547151 |
| 6 | 3.70761271 | 5.31208975 | 1.28273491 |
| 6 | 3.39910266 | 5.10487102 | 2.63003680 |
| 6 | 2.73895184 | 3.94058833 | 3.00464716 |
| 6 | 2.39297755 | 2.98398713 | 2.04232771 |
| 6 | 1.76393943 | 1.69149546 | 2.49482602 |
| 1 | 3.67497500 | 5.85032321 | 3.37564136 |
| 6 | 3.72970259 | 4.66947372 | -1.19916225 |
| 6 | -4.63890354 | 2.10884371 | -2.88030334 |
| 6 | -2.58135129 | 0.71636125 | -2.84449424 |
| 6 | -2.43193701 | 3.24270714 | -3.01702496 |
| 6 | 4.47411133 | 6.00696182 | -1.37259351 |
| 6 | 2.44279696 | 4.74742505 | -2.05417988 |
| 6 | 4.65950675 | 3.55515073 | -1.73561717 |
| 1 | -0.33819676 | 0.32597862 | 3.52726463 |
| 1 | -1.06774052 | -0.12292071 | 1.95764553 |
| 1 | -0.70942337 | 2.69306484 | 3.06289399 |
| 1 | -0.00136882 | 3.41228749 | 1.59978442 |
| 1 | -5.24183325 | 2.21219005 | -0.55684872 |
| 1 | -2.99249702 | 2.50875822 | 3.08058330 |
| 1 | 2.40854591 | 0.82547327 | 2.27524371 |
| 1 | 1.60558446 | 1.72342388 | 3.58506738 |
| 1 | 4.22499471 | 6.22977121 | 1.00961400 |
| 1 | 2.49615888 | 3.75468657 | 4.05321730 |
| 1 | -4.63209152 | 2.04903758 | -3.97783620 |
| 1 | -5.24126648 | 1.26727185 | -2.50880611 |
| 1 | -5.14115604 | 3.04684105 | -2.60322086 |
| 1 | -1.53339179 | 0.62276490 | -2.54236122 |
| 1 | -3.14111609 | -0.13318485 | -2.42696248 |
| 1 | -2.63147344 | 0.64775041 | -3.94145776 |
| 1 | -2.88926009 | 4.19790072 | -2.72068829 |
| 1 | -1.37620355 | 3.26069125 | -2.72612373 |
| 1 | -2.49139483 | 3.16626070 | -4.11319770 |
| 1 | 4.70965669 | 6.14802380 | -2.43695539 |
| 1 | 5.42388375 | 6.02736149 | -0.81889259 |
| 1 | 3.86545675 | 6.86520798 | -1.05317108 |
| 1 | 1.87471509 | 3.81206476 | -2.02005779 |
| 1 | 1.79171457 | 5.56051886 | -1.70185066 |
| 1 | 2.70509713 | 4.95474536 | -3.10259380 |
| 1 | 5.59929729 | 3.52791031 | -1.16508887 |
| 1 | 4.18641124 | 2.56958022 | -1.67117714 |
| 1 | 4.90960725 | 3.75433713 | -2.78863971 |
| 1 | 0.59630088 | -0.67308420 | 2.36144375 |
| 8 | 2.37966791 | -1.88608455 | 1.72337150 |
| 8 | 1.15499106 | -0.46353856 | -0.69835982 |
| 31 | 0.81039591 | 1.25233511 | -0.14402886 |
| 6 | 2.33900307 | -2.40492103 | 0.61864744 |

| | | | |
|---|---|---|---|
| 6 | 3.43021986 | -2.22811091 | -0.44010967 |
| 8 | 3.00258624 | -2.54822925 | -1.78927962 |
| 6 | 1.91027228 | -2.07399871 | -2.46621091 |
| 6 | 1.09802712 | -0.81680406 | -2.07446865 |
| 8 | 1.62474877 | -2.63290052 | -3.50540029 |
| 6 | 1.54948351 | 0.31463971 | -3.01387547 |
| 6 | 4.22016053 | -0.93288073 | -0.32886813 |
| 1 | 4.12695772 | -3.06140540 | -0.23542296 |
| 1 | 0.06491806 | -1.09857875 | -2.34282175 |
| 1 | 0.88504517 | 1.18509186 | -2.89607494 |
| 1 | 1.49040683 | -0.01166944 | -4.05953439 |
| 1 | 2.57989703 | 0.62274105 | -2.79067281 |
| 1 | 4.65692893 | -0.86253493 | 0.67566167 |
| 1 | 3.59124021 | -0.05301692 | -0.49626446 |
| 1 | 5.03697783 | -0.94692540 | -1.06173572 |
| 8 | -5.18652713 | -8.41862893 | 2.41878088 |
| 6 | -5.88769728 | -9.65370353 | 2.13480251 |
| 1 | -6.20135069 | -9.68354519 | 1.08385645 |
| 6 | -3.38256518 | -6.92234719 | 2.06590084 |
| 6 | -2.19661442 | -6.56673895 | 1.16998500 |
[truncated: 682,484 more chars]
